# Pericardial fluid proteomic label-free quantification of differentially expressed proteins in ischemic heart disease patients with systolic dysfunction by nano-LC-ESI-MS/MS analysis†

**DOI:** 10.1039/d0ra08389e

**Published:** 2020-12-23

**Authors:** Junaid Ullah, Satwat Hashmi, Arslan Ali, Faisal Khan, Shahid Ahmed Sami, Nageeb Basir, Syeda Saira Bokhari, Hasanat Sharif, Hesham R. El-Seedi, Syed Ghulam Musharraf

**Affiliations:** H.E.J. Research Institute of Chemistry, International Center for Chemical and Biological Sciences, University of Karachi Karachi-75270 Pakistan musharraf1977@yahoo.com +92 213 4819018 +92 213 4819019 +92 213 4824924 +92 213 4824925 +92 213 4819010; Department of Biological and Biomedical Sciences, Agha Khan University Karachi-74800 Pakistan; Dr Panjwani Center for Molecular Medicine and Drug Research, International Center for Chemical and Biological Sciences, University of Karachi Karachi-75270 Pakistan arslanali1986@gmail.com; Department of Surgery, The Aga Khan University Hospital Karachi-74800 Pakistan; Department of Medicine, The Aga Khan University Hospital Karachi-74800 Pakistan; Pharmacognosy Group, Department of Pharmaceutical Biosciences, BMC, Uppsala University SE-751 23 Uppsala Sweden; International Research Center for Food Nutrition and Safety, Jiangsu University Zhenjiang 212013 China

## Abstract

Left ventricular systolic dysfunction (LVSD) is common in patients with pre-existing ischemic heart disease (IHD) and myocardial infarction. An untargeted proteomic approach is used to improve the understanding of the molecular mechanisms associated with LVSD and to find out potential proteomic signatures in pericardial fluid. The pericardial fluid of IHD (*n* = 45) patients was grouped into two categories according to the left ventricular ejection fraction, LVEF ≥45 (*n* = 33) and LVEF <45 (*n* = 12), and analyzed by using nano-liquid chromatography–mass spectrometry (nano-LC-MS/MS) technique. The nano-LC-MS/MS analysis resulted in the identification of 709 pericardial fluid (PF) proteins in both normal and impaired systolic functional groups (LVEF ≥45 *vs.* LVEF <45). Sixteen proteins were found to be differentially expressed (*p* < 0.05, fold change >2) including 12 down-regulated and 4 up-regulated in the impaired systolic functional group (LVEF <45) compared to the normal group (LVEF ≥45). Among the differentially expressed proteins the inflammatory marker albumin, atherosclerosis marker apolipoprotein A-IV and hedgehog-interacting protein marker of angiogenesis were predominantly associated with the impaired LVEF <45 group. KEGG pathway analysis revealed that the hedgehog (Hh) signalling pathway is up-regulated in LVSD reflecting the underlying molecular and pathophysiological processes.

## Abstract 2

Proteomics of pericardial fluid from patients with ischemic heart disease having impaired systolic function.

## Graphical abstract

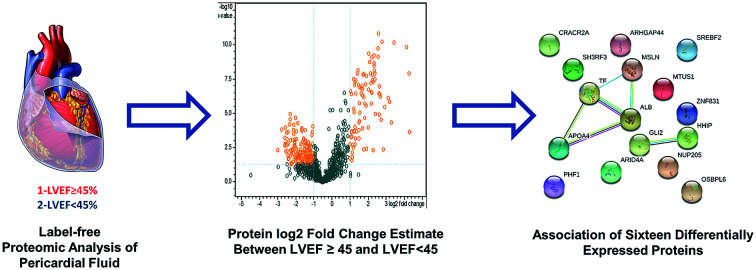

## Introduction

Cardiovascular disease (CVD) is one of the main causes of mortality and disability worldwide. According to the World Health Organization (WHO), in 2008 approximately 17.3 million people died from cardiovascular disease and this will, expectedly, rise to 23.3 million annually by 2030.^1^ Ischemic heart disease (IHD) is the most prevalent condition among the CVDs. Left ventricular systolic dysfunction (LVSD) is common in patients with IHD.^2^ LVSD refers to an impairment of the left ventricular contractility that results in reduced left ventricular ejection fraction (LVEF) and cardiac output. Patients with LVSD were at increased risk of death compared to subjects with a normal LVEF.^3^ A 10% reduction in LVEF predicts a 39% increase in all-cause mortality, hospitalization and an increased heart failure risk.^4^ Prevalence of LVSD increases substantially with age and is more prevalent in men (7.6%) as compared to women (2.6%)^5^

The measurement of proteins in biological samples plays a key role in the diagnosis and prognosis of patients that are at high risk of diseases. Pericardial fluid has been used to understand the molecular mechanism of disease in a number of studies.^6^ Pericardial fluid (PF) is an ultrafiltrate of the plasma and a biochemical window into the heart by which information related to the pathophysiological status of the heart can be obtained.^7^ Pericardial fluid is present between the two layers; parietal and visceral of the pericardium and is produced by the visceral pericardium of the heart. Under normal conditions, it measures about 15–50 mL in volume. Many diseases including ischemic heart disease, infections, and idiopathic causes can increase the volume and change its composition.^8^ The proteomic analysis of pericardial fluid may be an important tool to find out the pathophysiological mechanisms underlying cardiac diseases and to find out potential therapeutic targets.^9^ Several proteomic signatures have been discovered and used in the management of LVSD such as Brain Natriuretic Peptide (BNP), which is released in the pericardial fluid and serum by the ventricles in response to increased wall tension or stretch of cardiac chambers.^10^ The proteomic analysis of pericardial fluid in patients with lung bullae disease resulted in the identification of 1007 proteins which provided valuable insight into disease mechanism.^11^ To the best of our knowledge, this is the first study constituting the proteomic profiling of the pericardial fluid in patients with LVSD with established IHD.

The present study aimed to uncover the differentially expressed proteins in the pericardial fluid by a label-free quantitative proteomic approach in IHD patients with impaired (LVEF <45) and normal (LVEF ≥45) systolic function. The study will help to improve the understanding of the molecular mechanisms associated with LVSD and to find out potential proteomic signatures.

## Material and methods

### Experimental design and statistical analysis

For the present study fourty five patients (43 men and 2 women) were recruited with confirmed ischemic heart disease (IHD) at South city and Aga Khan University Hospital Karachi, Pakistan. A written informed consent was taken from each patient before coronary artery bypass graft (CABG). In this study, patients with renal insufficiency, metabolic bone diseases, valvular disease moderate to severe, constrictive pericarditis and infiltrative, hypertrophic or restrictive cardiomyopathies, established pulmonary disease and malignancy were excluded. Two-dimensional and Doppler echocardiography was used for the assessment of left ventricular systolic function following ASE guideline 2009. Patients were further categorized into two groups according to the LV ejection fraction: group A was 33 patients with normal left ventricular systolic function (LVEF ≥45), and group B was 12 patients with impaired left ventricular systolic function (LVEF <45) as depicted in Table 1. The study was approved by the Ethical Review Board (ERB) of the Aga Khan University Hospital and Institutional Review Board (IRB) of International Centre for Chemical and Biological Sciences (ICCBS), University of Karachi, Pakistan. All procedures followed in this study were in accordance with the ethical principles of the 1964 declaration of Helsinki.

**Table 1:** Baseline characteristics^a^

	(LVEF ≥45%)	(LVEF <45%)
*N*	33	12
Age [years; mean (SD)]	59.7 ± 7.1	58.0 ± 9.6
Body weight [kg; mean (SD)]	72.7 ± 11.3	76.6 ± 12.7

**Medical history**		
Diabetes/non-diabetes	18/15	9/3
Hypertension/non-hypertension	23/10	7/5

**Renal function**		
Creatinine [mg dL^−1^; mean (SD)]	1.0 ± 0.2	1.0 ± 0.3
BUN [mg dL^−1^; mean (SD)]	16.0 ± 3.4	19.2 ± 11.3

**Echocardiographic parameters**		
LVEF [%; mean (SD)]	58.2 ± 4.9	36.6 ± 8

a SD standard deviation, BUN blood urea nitrogen, LVEF left ventricular ejection fraction.

### Sample collection and processing

Pericardial fluid samples were collected from each subject in pre-labeled clean and sterile falcon tubes. Samples were centrifuged at 3500 rpm, for 15 min at 4 °C to remove cellular components. For further analysis aliquoted samples were stored at −80 °C in microcentrifuge tubes. Bicinchoninic acid (BCA) procedure was followed for measurement of total protein concentration according to the Kit protocol.^12^

### FASP digestion

Each pericardial fluid sample equivalent to 200 μg protein, were digested using filter-aided sample preparation (FASP) method.^13^ In short, protein concentrates in 0.5 mL 10 kDa cutoff centrifuge filters were mixed with 500 μL of 6 M urea in 0.1 M tris/HCl and the samples were centrifuged at 14 000*g* for 25 min at 20 °C. After centrifugation, 10 μL of 0.1 M Dithiotreitol (DTT) was added to the filters, and incubated at 56 °C for 30 min. Then, 10 μL of 0.3 M iodoacetamide (IAA) was added to the filters, after which the samples were incubated for 30 min in darkness. Finally, 2 μg of trypsin (MS Grade, Thermo Scientific) in 50 μL of 100 mM NH_4_HCO_3_ was added to each filter. The protein to enzyme ratio was 50 : 1. Samples were incubated overnight at 37 °C and peptides were released and collected by centrifugation at 14 000*g* at 20 °C for 25 min.

### LC-MS/MS analysis

The protein digest were dissolved in 0.1% TFA and 1 μL (equivalent to 2 μg) was injected on a C18-reverse-phase trapping column (C18, Acclaim™ PepMap™ 100, 75 μm × 2 cm, particle size 3 μm, nanoViper, Thermo Fisher Scientific) and was separated by C18-reverse-phase nano-LC column (75 μm × 15 cm Acclaim™ PepMap™ RSLC C18 column with 2 μm particle size and 100 Å pore size, NanoViper, Thermo Fisher Scientific), using Nano-UHPLC, (Dionex, UltiMate 3000 RSLCnano System, Thermo Scientific, USA) coupled to MaXis II ESI-QTOF Mass spectrometer (Bruker Daltonics, Bremen, Germany). Buffers A and B contained 0.1% formic acid in ultra-pure water and 0.1% formic acid in acetonitrile (ACN), respectively were used for peptide elusion. ACN gradient at 300 nL min^−1^ was used to separate peptides after 6 min sample load at a flow rate of 5 μL min^−1^ with 2% B. Elution of the peptides were achieved by applying a linear gradient of 1–35% B over 29 min, with subsequent gradient increase to 95% B over 2 min, hold B for 7 min at 95%, and then decreased to 2% B for 4 min followed by 12 min equilibration at 2% B. Data dependent acquisition (DDA) was carried out in positive ion mode. The parameters were set as follows: end plate offset voltage was 500 V, capillary voltage was 4500 V, dry gas flow rate was 4.0 L min^−1^ and dry temperature was 180 °C. Scan mode for MS and MS/MS were between 150 to 2200 *m*/*z*. The threshold for MS/MS was 2500 and cycle time of 3 s.

### Label-free quantification

Bruker Daltonics Compass Data Analysis Software (version 4.4) was used to generate the molecular feature list for each LC-MS/MS run. The resulting molecular features were transferred to ProfileAnalysis Software to calculate buckets. A bucket is characterized by its MH^+^ mass value, retention time, and intensity values. The parameters for bucket calculation were set as, retention time range 6–45 min, mass range 150–2200 *m*/*z*, normalization with a quantile, value count of group attribute within bucket were set as 2 and value count of bucket 30. After bucket generation, peptide regulation ratios were calculated using *T*-test and transferred to ProteinScape software.

To link peptide regulation output with the identification results, the LC-MS/MS raw data files of each run were converted to (.xml) using Bruker Daltonics Compass Data Analysis Software (version 4.4). The resulting xml files were imported to ProteinScape software for the Mascot Database search. After the protein database search, peptide identification was linked to their peptide regulation ratios by selecting the retention time correction option and *p*-value <0.05 as assignment criteria. Finally, protein regulation ratios were calculated from peptide regulation ratios and a list of proteins includes quantification information such as the number of peptides ratios, coefficient of variation (CV) values, and the regulation ratios were generated.^14^

### MS/MS data analysis using ProteinScape

Nano-LC-MS/MS raw data files of each run were converted to (.xml) and were searched using the Mascot search engine (Version 2.6, Matrix Science, London, UK) triggered by ProteinScape against Swiss-Prot database with the following parameters: taxonomy filter was set to *homo sapiens*, for proteolytic cleavages, trypsin was used with maximum missed cleavages up to 1, oxidation of methionine as variable modifications and carbamidomethyl of cysteine as fixed modifications were allowed. Peptide mass tolerance was set at 0.08 Da and fragment mass tolerance was set at 0.2 Da. Peptide charges were set at +2, +3, and +4. The accepted FDR was set to <1%.

### Bioinformatic analysis

Label-free quantified proteins were further subjected to bioinformatic analysis, including gene ontology (GO) and KEGG pathway analysis. The 16 differentially expressed proteins sequence information was extracted from the online UniProt knowledgebase database (https://www.uniprot.org/) and retrieved in FASTA format. Functional classification of proteins was done by gene ontology on the basis of biological process, molecular process and cellular component using OmicsBox (version 1.2, https://www.biobam.com/omicsbox/).^15^ HSP Length cutoff 33 and expect value 1 × 10^−3^ were set as parameter for blastp-fast. Parameters for the gene ontology (GO) annotations were set as; Hit filter 500, GO weight 5, HSP-Hit coverage cutoff 0, annotation cutoff 55 and *E*-value-hit-filter 1 × 10^−6^. The level 2 pie/bar chart configuration was used for showing annotated sequences in GO graphs. The association between differentially expressed quantified proteins with each other and their interaction with other proteins were assessed by the STRING EMBL software (version 11.0, https://string-db.org/). The medium confidence score of 0.400 was used.^16^

## Results

**Fig. 1:**
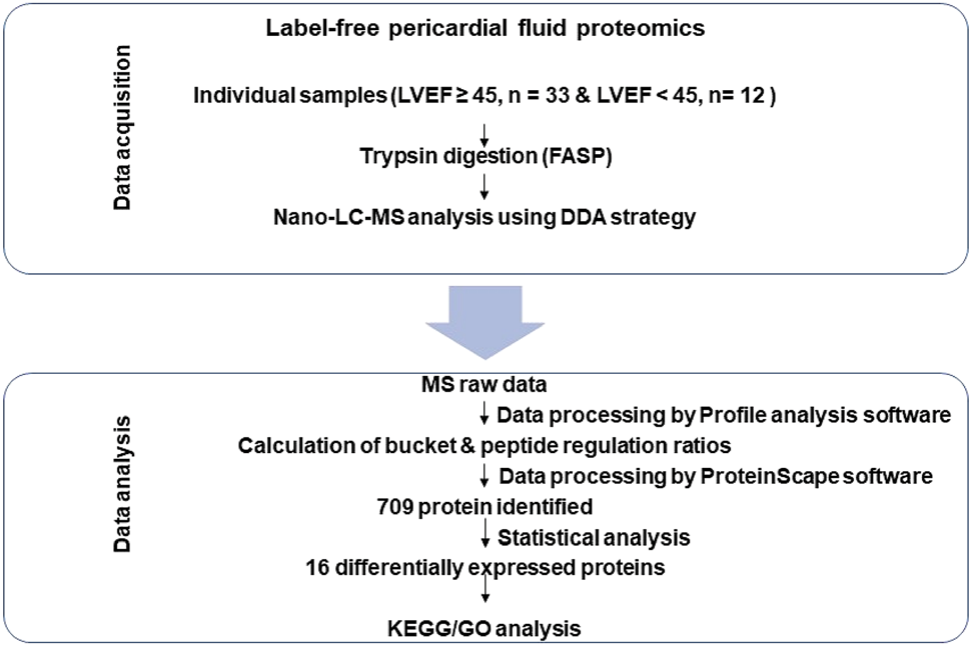
Workflow of the present study. First stage is the data acquisition stage; it consisted of pericardial fluid (PF) sample preparation and its nano-LC MS/MS analysis using DDA strategy. The second stage is the data analysis stage; it consisted of statistical analysis and bioinformatic analysis (GO and KEGG pathway analysis).

For the present study, workflow is shown in Fig. 1. The data acquisition stage consisted of pericardial fluid sample analysis through nano-LC-ESI-MS/MS by using the data dependent acquisition (DDA) strategy. The statistical analysis stage was used to find out significantly differentially expressed proteins in both groups (LVEF ≥45 *vs.* LVEF <45) followed by the bioinformatics analysis (GO and KEGG) to understand the biological role of these proteins.

### Label-free proteomics and identification of differentially expressed proteins

**Fig. 2:**
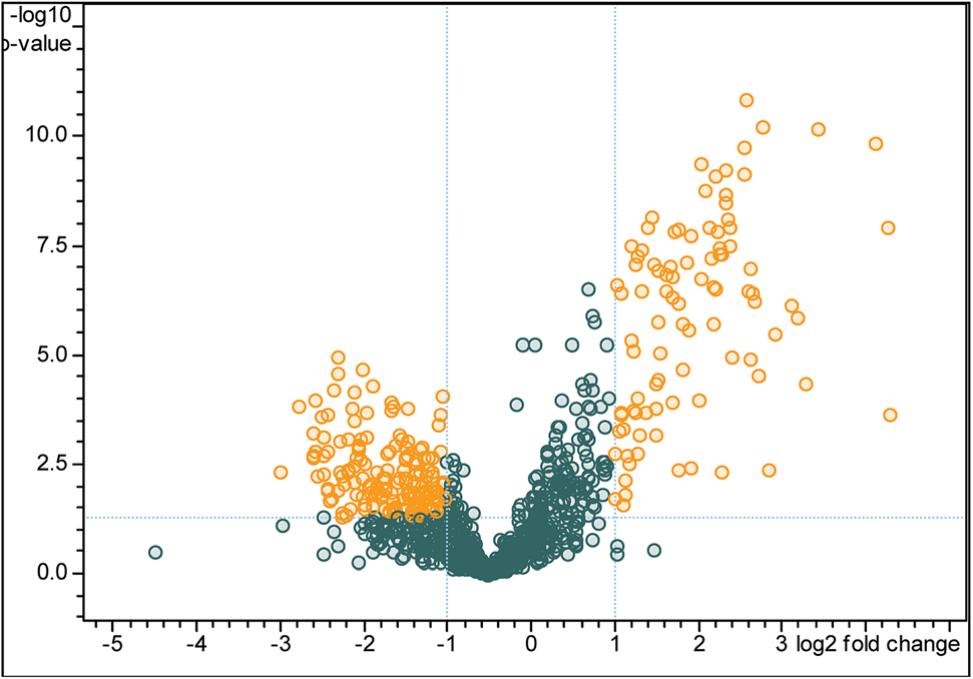
Volcano plot of peptide areas, derived from the quotient (LVEF <45/LVEF ≥45) with log 2 fold-change (*x*-axis) *versus* −log 10 *p*-value (*y*-axis). The orange dots, located in the upper-left and upper-right sections, corresponding to high fold changes and low *p*-values, indicate significantly regulated signals while the gray dots indicate the non-significant differentially expressed signals. For the differentially expressed signals, a *p*-value of <0.05 and fold change of >2 are set as the significant thresholds.

A total of 709 pericardial fluid proteins were identified using the DDA strategy in both groups (LVEF ≥45 *vs.* LVEF <45) (Table S1†). The annotated spectra of all the identified proteins along with the differentially expressed proteins are given in the ESI file.† For a better look, the double fold change for the log 2-based values derived from the quotient (LVEF <45/LVEF ≥45) is presented in Fig. 2 as a volcano plot, statistically determined by a *P*-value <0.05, at 2.0-fold change. The identified proteins in the two groups of the experiment (LVEF ≥45/LVEF <45) were analyzed and compared, 16 proteins were found differentially expressed, including 12 down-regulated and 4 up-regulated proteins in impaired (LVEF <45) systolic function group as compared to normal (LVEF ≥45) systolic function group. Serum albumin, mesothelin, E3 ubiquitin-protein ligase SH3RF3, Rho GTPase-activating protein 44 and serotransferrin were the most down-regulated while AT-rich interactive domain-containing protein 4A, zinc finger protein GLI2, zinc finger protein 831, and hedgehog-interacting protein were the most up-regulated proteins. The results with protein name, accession number, isoelectric point, sequence coverage, molecular weight, fold-change and *t*-test *p*-values are presented in Table 2.

**Table 2:** Differentially expressed proteins identified in ischemic heart disease patients with normal LV systolic function (LVEF ≥45) and impaired LV systolic function (LVEF <45)

Accession	Protein	MW [kDa]	pI	Mascot score	#Peptides	Sequence coverage [%]	RMS90 [ppm]	Fold change log 2 (LVEF ≥45): (LVEF<45)	*p*-Value	Regulation
ALBU_HUMAN	Serum albumin	69.3	5.9	3565.8	62	73.9	42.24	5.02	0.0029	Down
TRFE_HUMAN	Serotransferrin	77.0	6.8	2660.8	43	50.9	44.56	2.09	0.0006	Down
APOA4_HUMAN	Apolipoprotein A-IV	45.4	5.3	461.2	12	32.6	44.87	1.86	0.0029	Down
MSLN_HUMAN	Mesothelin	68.9	6.0	76.2	3	5.4	572.57	4.60	0.0001	Down
RHG44_HUMAN	Rho GTPase-activating protein 44	89.2	6.1	51.1	2	1.1	12.74	2.26	0.0007	Down
OSBL6_HUMAN	Oxysterol-binding protein-related protein 6	106.2	6.5	38.4	2	2.1	36.23	1.89	0.0001	Down
GLI2_HUMAN	Zinc finger protein GLI2	167.7	6.9	37.7	2	1.5	664.68	−4.47	0.005	Up
SRBP2_HUMAN	Sterol regulatory element-binding protein 2	123.6	8.7	34.2	1	1.0	1.51	1.50	0.0147	Down
PHF1_HUMAN	PHD finger protein 1	62.1	9.3	31.2	1	1.4	6.49	1.42	0.0001	Down
ZN831_HUMAN	Zinc finger protein 831	177.8	8.7	25.7	1	1.0	5.91	−4.03	0.0009	Up
EFC4B_HUMAN	EF-hand calcium-binding domain-containing protein 4B	45.6	4.9	20.2	1	2.5	16.97	1.50	0.0147	Down
NU205_HUMAN	Nuclear pore complex protein Nup205	227.8	5.8	20.0	1	0.4	922.75	1.08	0.0116	Down
MTUS1_HUMAN	Microtubule-associated tumor suppressor 1	141.3	7.3	19.6	1	0.8	8.45	1.50	0.0147	Down
HHIP_HUMAN	Hedgehog-interacting protein	78.8	8.2	17.6	1	2.0	6.71	−2.34	0.0184	Up
SH3R3_HUMAN	E3 ubiquitin-protein ligase SH3RF3	92.7	9.1	17.0	1	1.0	10.22	3.17	0.0001	Down
ARI4A_HUMAN	AT-rich interactive domain-containing protein 4A	142.7	5.0	15.6	1	1.0	3.54	−4.49	0.0042	Up

### Functional classification of differentially expressed proteins

**Fig. 3:**
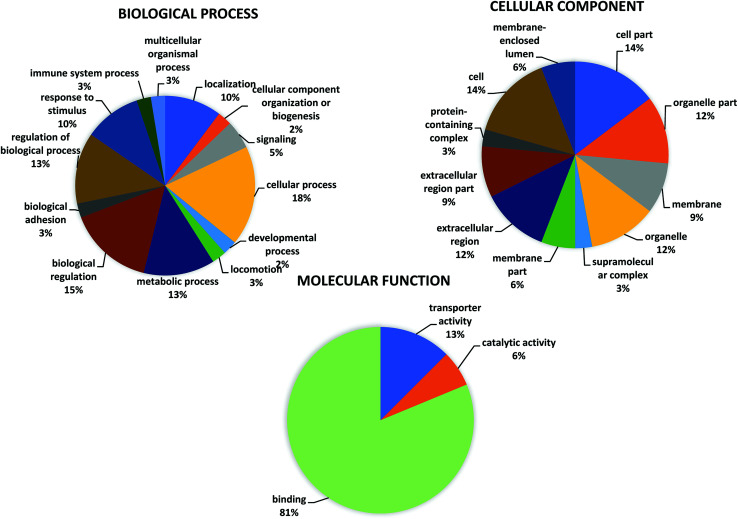
Go annotation of “biological process” “molecular function” and “cellular component” analyze by OmicxBox (version 1.2, https://www.biobam.com/omicsbox/).

A GO classification was performed for the 16 significant differentially expressed proteins in patients with normal (LVEF ≥45) and impaired (LVEF <45) systolic function group as shown in the pie chart at graph level 2 (Fig. 3 and Table S2†). The GO analysis of differentially expressed protein revealed involvement in multiple biological processes in both groups. The highest proportion of (18%, 7 proteins: MTUS1, TRFE, PHF1, MSLN, SH3R3, OSBL6, RHG44) sequences were found to be involved in cellular processes followed by biological regulation (15%, 6 proteins: MTUS1, TRFE, PHF1, SH3R3, OSBL6, RHG44), and metabolic processes (13%, 5 proteins: PHF1, MSLN, SH3R3, OSBL6, APOA4), while the remaining functions such as response to a stimulus; localization, signaling, developmental process, cellular component organization, locomotion, immune system process biological adhesion and multicellular organismal process, were observed in less proportions in both groups (LVEF ≥45/LVEF <45). Differentially expressed, proteins were also classified based on their molecular functions, such as protein involved with binding (81%, 13 proteins: MTUS1, PHF1, MSLN, EFC4B, GLI2, RHG44, TRFE, SRBP2, ARI4A, SH3R3, OSBL6, APOA4, ZN831), transporter activity (12%, 2 proteins: TRFE, OSBL6) and catalytic activity (6%, 1 protein: HHIP).

OmicsBox analysis, also classified the differentially expressed proteins according to their cellular component. Most of the proteins were assigned to the organelle (12%, 4 proteins MTUS1, MSLN, NU205, OSBL6), followed by extracellular region (12%, 4 proteins: TRFE, MSLN, APOA4, ALBU) and membrane (9%, 3 proteins: MTUS1, MSLN, OSBL6), while the remaining component such as extracellular region part, membrane part, membrane-enclosed lumen, supramolecular complex and protein-containing complex were observed in fewer proportions.

### KEGG pathway analysis

**Fig. 4:**
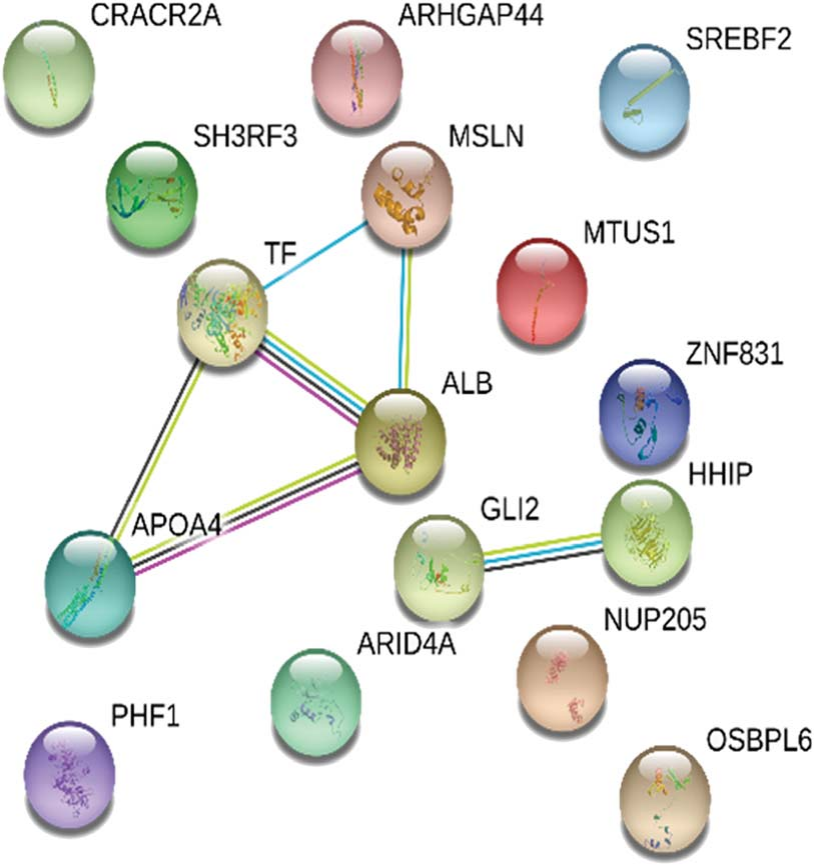
Interactive network analysis and association of 16 differentially expressed pericardial fluid proteins obtained from STRING (version 11.0, https://string-db.org/).

STRING was used to gain an insight into the interactive links known to be associated with the 16 proteins expressed differentially in the dataset. Interactive links between the differentially regulated proteins could be traced as shown in Fig. 4. A total of 8 RCTM/KEGG pathways were observed in the present study. The suggested pathways of these differentially regulated proteins are listed in Table 3, along with term ID, term definition, observed gene count, background gene count, FDR and matching proteins within a network.

**Table 3:** Pathways of differentially expressed protein identified in ischemic heart disease patients with normal LV systolic function (LVEF ≥45) and impaired LV systolic function (LVEF <45)

KEGG/RCTM pathways					
#Term ID	Term description	Observed gene count	Background gene count	False discovery rate	Matching proteins in your network (labels)
HSA04340	Hedgehog signaling pathway	2	46	0.009	GLI2, HHIP
HSA05217	Basal cell carcinoma	2	63	0.009	GLI2, HHIP
HSA-381426	Regulation of insulin-like growth factor (IGF) transport and uptake by insulin-like growth factor binding proteins (IGFBPs)	3	123	0.012	ALB, MSLN, TF
HSA-8957275	Post-translational protein phosphorylation	3	106	0.012	ALB, MSLN, TF
HSA-8957322	Metabolism of steroids	3	146	0.012	ALB, OSBPL6, SREBF2
HSA-8963899	Plasma lipoprotein remodeling	2	30	0.012	ALB, APOA4
HSA-194068	Bile acid and bile salt metabolism	2	42	0.0157	ALB, OSBPL6
HSA-5632684	Hedgehog ‘on’ state	2	82	0.0405	GLI2, HHIP

## Discussion

Proteomics is used to perform quantitative as well as qualitative analyses of a large array of proteins in biological samples.^17^ Recently, label-free quantitative proteomics (LFQP) has gained more popularity as mass spectrometer's efficiency has significantly improved. The LFQP becomes a widely applied approach for identifying as well as quantifying differentially expressed proteins. It increases the dynamic range of proteins 3 to 4 time as compared to 2DE and is more sensitive and rapid than many other proteomic methods.^18^

In the present study, we have found 16 significantly differentially expressed proteins in LVEF ≥45 *vs.* LVEF <45. Among the differentially expressed proteins 12 proteins down-regulated in impaired (LVEF <45) systolic function group, including serum albumin, serotransferrin, apolipoprotein A-IV, mesothelin, Rho GTPase-activating protein 44, sterol regulatory element-binding protein 2, oxysterol-binding protein-related protein 6, PHD finger protein 1, nuclear pore complex protein Nup205, EF-hand calcium-binding domain-containing protein 4B, Microtubule-associated tumor suppressor 1, and E3 ubiquitin-protein ligase SH3RF3. While, the remaining 4 proteins were found up-regulated in impaired (LVEF <45) systolic function group including, zinc finger protein 831, zinc finger protein GLI2, AT-rich interactive domain-containing protein 4A and hedgehog-interacting protein. One of the down-regulated protein in patients with impaired (LVEF <45) group is albumin. Albumin is a 66.5 kDa protein which is the most abundant protein in plasma. Albumin plays a significant role in regulating plasma oncotic pressure and fluid distribution throughout the body compartment.^19^ Serum albumin's physiological properties include anti-inflammatory, antioxidant, anticoagulant activity and regulating the transport of cholesterol.^20^ Normal pericardial fluid contains between one quarter and one third of the protein of plasma and demonstrates a far higher proportion of albumin to other proteins in the pericardial fluid.^21,22^ Hypoalbuminemia, with serum albumin levels less than 3.5 g dL^−1^ (ref. 23) has been linked to several cardiovascular diseases such as coronary artery disease, congenital heart disease, stroke and heart failure.^24^ Hypoalbuminemia has been reported in one-third of heart failure patients with reduced ejection fraction (LVEF <45).^25^ In concordance with the findings in the serum, the present findings suggest that the down-regulation of albumin in the pericardial fluid is associated with impaired systolic function of the left ventricle. Hence, it could be used as a potential risk marker in identifying the patients with left ventricular systolic dysfunction.

Another down-regulated protein in impaired (LVEF <45) group is serotransferrin. It is an iron binding protein, responsible for transporting iron from sites of heme degradation and absorption to those of utilization and storage.^26^ The serum level of serotransferrin decreases in inflammatory conditions such as diabetes mellitus which in turn increases the risk of CVD.^27^ It has been demonstrated that decrease level of plasma serotransferrin is associated with rheumatic valvular heart disease (RVD),^28^ and coronary artery dilation (CAD) caused by Kawasaki disease.^29^ The down-regulation of serotransferrin in impaired (LVEF <45) group may be due to the inflammatory response of the body as its level also decreases in patients with CKD and ESRD.^30^

Apo A-IV is another protein that is down-regulated in impaired (LVEF <45) group. Apo A-IV, is an oligosaccharide containing protein with a molecular weight of 46 kDa.^31^ Its concentration in plasma is 15–37 mg dL^−1^.^32^ Apo A-IV is synthesized in the intestine and is incorporated into the chylomicrons and secreted into the intestinal lymph during fat absorption.^33^ Apo A-IV plays a significant role in the lipid and glucose metabolism.^34^ It also acts as anti-inflammatory agent and potentially exert its anti-atherogenic effect^35^ by reverse cholesterol pathway, which removes cholesterol from peripheral cells and transports it to the liver.^36^ Clinical studies have demonstrated an inverse relationship between apo A-IV and coronary artery diseases.^37^ Apo A-IV deficiency is associated with atherosclerosis, cardiovascular diseases, chronic pancreatitis, malabsorption disorders^38^ and diabetes mellitus.^39^ Low Apo A-IV levels in these patients with systolic dysfunction as compared to patients with preserved EF may point towards a disease mechanism underlying the progression from preserved EF to reduced EF.

Among the up-regulated differentially expressed proteins in patients with impaired (LVEF <45) group is AT-rich interactive domain containing protein 4A. It is also known as *ARID4A* or retinoblastoma – binding protein 1 is encoded by the gene *ARID4A*.^40^ Studies shows that *ARID4A* play a significant role in various types of cancer such as breast cancer and leukemia. *ARID4A* binds with other proteins and play a key role in cell proliferation and differentiation.^41^ Zinc finger protein 831 belongs to a large class of zinc finger proteins (ZNFs) is up-regulated in impaired (LVEF <45) group. Zinc finger proteins (ZNFs) have a key role in tissue development and differentiation. Alterations in ZNFs are involved in the development of several diseases such as congenital heart disease, defects of the cardiac outflow tract and diabetes.^42^

KEGG network analysis with pathway enrichment identified that the hedgehog signaling pathway is up-regulated in impaired (LVEF <45) group as compared to the normal (LVEF ≥45) group. Network analysis revealed that two up-regulated proteins zinc finger protein GLI2 and hedgehog-interacting protein are involved in hedgehog signaling (Hh) pathway. Hh signaling pathway was first identified as a key mediator of organogenesis in invertebrates by Nusslein–Volhard and Wieschaus in 1980.^43^ Three Hh homologues have been discovered in mammals in the early 1990s, including Indian hedgehog (Ihh), Sonic (Shh) and Desert (Dhh). Sonic is one of the best studied and most widely expressed during embryonic development as compared to Dhh and Ihh.^44^ Hh signaling pathway is known to regulate cell differentiation and migration during embryonic development but in post-natal life, the Hh signaling can be recapitulated under several pathological conditions, including cardiac ischemia.^45^ The absence of tissue specific Hh signalling components reduces proangiogenic gene expression and thus loss of coronary vasculature that leads to cardiomyocyte cell death and ventricular failure. The presence of the Hh signalling pathway may protect and limit the extent of myocardial ischemic damage.^46^ Many theories are postulated to elucidate the regulation mechanism of Hh signaling pathway in ischemic heart tissues. It has been shown that the expression of hypoxia-induced factor-1 (HIF-1) and inflammation in cardiac ischemia, activate the Hh signaling pathway, resulting in elevated expression of angiogenic and proangiogenic factors to facilitate angiogenesis and neovascularization in ischemic tissues.^47^ In concordance with the previous findings, our results also validate the recapitulation of Hh signaling pathway in ischemic heart disease patients but more prominently in impaired (LVEF <45) group.^48^ Considering that the Hh signaling pathway is essential for the formation of new coronary vessels, this finding signals an important adaptive mechanism in humans with LVSD.^49^

KEGG pathway analysis also showed that pathways involved in the Metabolism of steroids (pathway proteins: albumin, oxysterol-binding protein-related protein 6, sterol regulatory element-binding protein 2), plasma lipoprotein remodelling (pathway proteins: albumin, apolipoprotein A-IV) and bile acid and bile salt metabolic pathways (pathway proteins: albumin, oxysterol-binding protein-related protein 6) were down-regulated in impaired (LVEF <45) group as compared to the normal (LVEF ≥45) group.

## Conclusion

In conclusion, sixteen proteins were found to be differentially expressed between IHD patients with LVEF <45 and LVEF ≥45. Among the differentially expressed proteins the inflammatory marker albumin, atherosclerosis marker apolipoprotein A-IV and hedgehog-interacting protein marker of angiogenesis were predominantly associated with impaired LVEF <45 group. The KEGG pathway analysis revealed that the hedgehog signaling pathway is up regulated in impaired LVEF <45 group. These findings suggest important associations of heart systolic function with specific proteins reflecting its underlying pathophysiological mechanisms, however, they need to be further validated in a larger number of samples.

## Conflicts of interest

The authors declare that they have no conflict of interest.

## Supplementary Material

RA-011-D0RA08389E-s001

RA-011-D0RA08389E-s002

## References

[cit1] Vilahur G., Badimon J. J., Bugiardini R., Badimon L. (2014). Eur. Heart J. Suppl..

[cit2] Chavey W. E., Blaum C. S., Bleske B. E., Van Harrison R., Kesterson S., Nicklas J. M. (2001). Am. Fam. Physician.

[cit3] Sara J. D., Toya T., Taher R., Lerman A., Gersh B., Anavekar N. S. (2020). European Cardiology Review.

[cit4] Japp A. G., Moir S., Mottram P. M. (2015). Heart, Lung Circ..

[cit5] Raymond I., Pedersen F., Steensgaard-Hansen F., Green A., Busch-Sorensen M., Tuxen C., Appel J., Jacobsen J., Atar D., Hildebrandt P. (2003). Heart.

[cit6] Israr M. Z., Heaney L. M., Suzuki T. (2018). Heart failure clinics.

[cit7] Yavuz S., Kasap M., Akpinar G., Ozbudak E., Ural D., Berki T. (2014). BioMed Res. Int..

[cit8] Ege T., Us M. H., Cikirikcioglu M., Arar C., Duran E. (2006). Yonsei Med. J..

[cit9] Trindade F., Bastos P., Leite-Moreira A., Manadas B., Ferreira R., Soares S. F., Daniel-da-Silva A. L., Falcão-Pires I., Vitorino R. (2017). Arch. Biochem. Biophys..

[cit10] Amjad S., Sami S. A., Basir M. N., Hameed K., Fujita M., Ahmad H. R. (2013). Asian Cardiovasc. Thorac. Ann..

[cit11] Xiang F., Guo X., Chen W., Wang J., Zhou T., Huang F., Cao C., Chen X. (2013). Proteomics.

[cit12] Bainor A., Chang L., McQuade T. J., Webb B., Gestwicki J. E. (2011). Anal. Biochem..

[cit13] Wiśniewski J. R., Zougman A., Nagaraj N., Mann M. (2009). Nat. Methods.

[cit14] da Silva Frozza C. O., da Silva Brum E., Alving A., Moura S., Henriques J. A., Roesch-Ely M. (2016). J. Pharm. Pharmacol..

[cit15] Götz S., García-Gómez J. M., Terol J., Williams T. D., Nagaraj S. H., Nueda M. J., Robles M., Talón M., Dopazo J., Conesa A. (2008). Nucleic Acids Res..

[cit16] Szklarczyk D., Franceschini A., Wyder S., Forslund K., Heller D., Huerta-Cepas J., Simonovic M., Roth A., Santos A., Tsafou K. P. (2015). Nucleic Acids Res..

[cit17] Favicchio R., Thepaut C., Zhang H., Arends R., Stebbing J., Giamas G. (2016). Cancer Lett..

[cit18] Wang M., You J., Bemis K. G., Tegeler T. J., Brown D. P. (2008). Briefings Funct. Genomics Proteomics.

[cit19] Suzuki S., Hashizume N., Kanzaki Y., Maruyama T., Kozuka A., Yahikozawa K. (2019). PLoS One.

[cit20] Chien S.-C., Chen C.-Y., Lin C.-F., Yeh H.-I. (2017). Biomarker research.

[cit21] Vogiatzidis K., Zarogiannis S. G., Aidonidis I., Solenov E. I., Molyvdas P.-A., Gourgoulianis K. I., Hatzoglou C. (2015). Front. physiol..

[cit22] Gibson A., Segal M. (1978). J. Physiol..

[cit23] Levitt D. G., Levitt M. D. (2016). Int. J. Gen. Med..

[cit24] Arques S. (2018). Eur. J. Intern. Med..

[cit25] Hussein M. F. (2012). Iraqi Academic Scientific Journal.

[cit26] Aisen P., Listowsky I. (1980). Annu. Rev. Biochem..

[cit27] Gomme P. T., McCann K. B., Bertolini J. (2005). Drug discovery today.

[cit28] Gao G., Xuan C., Yang Q., Liu X.-C., Liu Z.-G., He G.-W. (2013). PLoS One.

[cit29] Zhang L., Wang W., Bai J., Xu Y.-F., Li L.-Q., Hua L., Deng L., Jia H.-L. (2016). Revista Portuguesa de Cardiologia.

[cit30] Pupim L. , Martin C. J. and Ikizler T. A., in Nutritional Management of Renal Disease, Lippincott Williams & Wilkins, Philadelphia, 2004, pp. 223–240

[cit31] Utermann G., Beisiegel U. (1979). Eur. J. Biochem..

[cit32] Weinstock P. H., Bisgaier C. L., Hayek T., Aalto-Setala K., Sehayek E., Wu L., Sheiffele P., Merkel M., Essenburg A., Breslow J. (1997). J. Lipid Res..

[cit33] Green P. H., Glickman R. M., Riley J. W., Quinet E. (1980). J. Clin. Invest..

[cit34] Wang Z., Wang L., Zhang Z., Feng L., Song X., Wu J. (2019). Nutr. Metab..

[cit35] Duverger N., Tremp G., Caillaud J.-M., Emmanuel F., Castro G., Fruchart J.-C., Steinmetz A., Denefle P. (1996). Science.

[cit36] Steinmetz A., Barbaras R., Ghalim N., Clavey V., Fruchart J., Ailhaud G. (1990). J. Biol. Chem..

[cit37] Kronenberg F., Stühlinger M., Trenkwalder E., Geethanjali F., Pachinger O., von Eckardstein A., Dieplinger H. (2000). J. Am. Coll. Cardiol..

[cit38] Koga S., Miyata Y., Funakoshi A., Ibayashi H. (1985). Digestion.

[cit39] Qu J., Ko C.-W., Tso P., Bhargava A. (2019). Cells.

[cit40] Liang Y. K., Han Z. D., Lu J. M., Liu Z. Z., Zhuo Y. J., Zhu X. J., Chen J. X., Ye J. H., Liang Y. X., He H. C., Zhong W. D. (2018). J. Cell. Biochem..

[cit41] Lin C., Song W., Bi X., Zhao J., Huang Z., Li Z., Zhou J., Cai J., Zhao H. (2014). OncoTargets Ther..

[cit42] Cassandri M., Smirnov A., Novelli F., Pitolli C., Agostini M., Malewicz M., Melino G., Raschella G. (2017). Cell Death Discovery.

[cit43] Nüsslein-Volhard C., Wieschaus E. (1980). Nature.

[cit44] Yao E., Chuang P.-T. (2015). J. Formosan Med. Assoc..

[cit45] Giarretta I., Gaetani E., Bigossi M., Tondi P., Asahara T., Pola R. (2019). Int. J. Mol. Sci..

[cit46] Lavine K. J., Kovacs A., Ornitz D. M. (2008). J. Clin. Invest..

[cit47] Pan J., Zhou S. (2012). Die Pharmazie-An International Journal of Pharmaceutical Sciences.

[cit48] Lima E. G., Carvalho F. P. C. d., Linhares Filho J. P. P., Pitta F. G., Serrano Jr C. V. (2017). Rev. Assoc. Med. Bras..

[cit49] Lavine K. J., White A. C., Park C., Smith C. S., Choi K., Long F., Hui C.-c., Ornitz D. M. (2006). Genes Dev..

