# Supplementary material for: Pericardial fluid proteomic label-free quantification of differentially expressed proteins in ischemic heart disease patients with systolic dysfunction by nano-LC-ESI-MS/MS analysis
Source: RSC Adv. 2020 Dec 23;11(1):320–7. doi: 10.1039/d0ra08389e (PMC8691035; doi:10.1039/d0ra08389e)
Supplement: RA-011-D0RA08389E-s002 [file RA-011-D0RA08389E-s002.pdf]

Electronic Supplementary Material (ESI) for RSC Advances.
This journal is © The Royal Society of Chemistry 2020

Supplementary Information

Pericardial fluid proteomic label-free quantification of differentially expressed proteins in ischemic heart disease patients with systolic dysfunction by nano-LC-ESI-MS/MS analysis

Junaid Ullah[a], Satwat Hashmi[b], Arslan Ali[c*], Faisal Khan[c], Shahid Ahmed Sami[d], Nageeb Basir[e], Syeda Saira Bokhari[e], Hasanat Sharif[d], Hesham R. El-Seedi[f,g] and Syed Ghulam Musharraf[a,c*]

[a]*H.E.J. Research Institute of Chemistry, International Center for Chemical and Biological Sciences, University of Karachi, Karachi-75270, Pakistan*

[b]*Department of Biological and Biomedical Sciences, Agha Khan University, Karachi-74800, Pakistan*

[c]*Dr. Panjwani Center for Molecular Medicine and Drug Research, International Center for Chemical and Biological Sciences, University of Karachi, Karachi-75270, Pakistan*

[d]*Department of Surgery, Agha Khan University, Karachi-74800, Pakistan*

[e]*Department of Medicine, Agha Khan University, Karachi-74800, Pakistan*

[f]*Pharmacognosy Group, Department of Pharmaceutical Biosciences, BMC, Uppsala University, SE-751 23 Uppsala, Sweden.*

[g]*International Research Center for Food Nutrition and Safety, Jiangsu University, Zhenjiang 212013, China.*

*Corresponding author. Tel.: +92 213 4824924-5; 4819010; fax: + 92 213 4819018-9.

E-mail address: musharraf1977@yahoo.com, arslanali1986@gmail.com

Table S1. List of 709 pericardial fluid proteins identified in IHD patients

Row	Accession	Protein	MW [kDa]	pI	Mascot Scores	#Peptides	SC [%]	RMS90 [ppm]	Rank	Median (LVEF>45):(LVEF<45)	#(LVEF>45):(LVEF <45)	CV [%] (LVEF>45):(LVEF<45)
1	ALBU_HUMAN	Serum albumin OS=Homo sapiens OX=9606 GN=ALB PE=1 SV=2	69.3	5.9	3565.8	62	73.9	42.24	1	5.02	1	0.00
2	TRFE_HUMAN	Serotransferrin OS=Homo sapiens OX=9606 GN=TF PE=1 SV=3	77.0	6.8	2660.8	43	50.9	44.56	2	2.09	1	0.00
3	CO3_HUMAN	Complement C3 OS=Homo sapiens OX=9606 GN=C3 PE=1 SV=2	187.0	6.0	1298.8	37	20.4	42.63	3			
4	APOA1_HUMAN	Apolipoprotein A-I OS=Homo sapiens OX=9606 GN=APOA1 PE=1 SV=1	30.8	5.6	1014.4	18	49.4	43.82	4			
5	A2MG_HUMAN	Alpha-2-macroglobulin OS=Homo sapiens OX=9606 GN=A2M PE=1 SV=3	163.2	6.0	679.9	17	13.6	43.51	5			
6	HPT_HUMAN	Haptoglobin OS=Homo sapiens OX=9606 GN=HP PE=1 SV=1	45.2	6.1	659.3	13	33.5	42.29	6			
7	IGG1_HUMAN	Immunoglobulin gamma-1 heavy chain OS=Homo sapiens OX=9606 PE=1 SV=2	49.3	8.9	654.7	12	29.2	41.66	7			
8	A1AT_HUMAN	Alpha-1-antitrypsin OS=Homo sapiens OX=9606 GN=SERPINA1 PE=1 SV=3	46.7	5.4	621.5	14	35.9	42.22	8			
9	IGHA1_HUMAN	Immunoglobulin heavy constant alpha 1 OS=Homo sapiens OX=9606 GN=IGHA1 PE=1 SV=2	37.6	6.1	578.8	10	29.2	42.19	9			
10	HBB_HUMAN	Hemoglobin subunit beta OS=Homo sapiens OX=9606 GN=HBB PE=1 SV=2	16.0	6.7	567.2	9	61.9	43.14	10			
11	IGHG3_HUMAN	Immunoglobulin heavy constant gamma 3 OS=Homo sapiens OX=9606 GN=IGHG3 PE=1 SV=2	41.3	8.2	561.9	5	17.5	43.65	11			
12	IGHG2_HUMAN	Immunoglobulin heavy constant gamma 2 OS=Homo sapiens OX=9606 GN=IGHG2 PE=1 SV=2	35.9	7.7	515.0	5	13.8	42.93	12			
13	APOA4_HUMAN	Apolipoprotein A-IV OS=Homo sapiens OX=9606 GN=APOA4 PE=1 SV=3	45.4	5.3	461.2	12	32.6	44.87	13	1.86	1	0.00
14	CO4A_HUMAN	Complement C4-A OS=Homo sapiens OX=9606 GN=C4A PE=1 SV=2	192.7	6.7	435.1	13	8.3	44.47	14			
15	HBD_HUMAN	Hemoglobin subunit delta OS=Homo sapiens OX=9606 GN=HBD PE=1 SV=2	16.0	7.8	433.2	2	17.0	40.62	15			
16	HEMO_HUMAN	Hemopexin OS=Homo sapiens OX=9606 GN=HPX PE=1 SV=2	51.6	6.5	384.5	11	20.6	44.28	16			
17	IGKC_HUMAN	Immunoglobulin kappa constant OS=Homo sapiens OX=9606 GN=IGKC PE=1 SV=2	11.8	6.1	335.0	5	80.4	129.51	17			
18	HBA_HUMAN	Hemoglobin subunit alpha OS=Homo sapiens OX=9606 GN=HBA1 PE=1 SV=2	15.2	8.7	332.1	8	50.7	45.30	18			
19	A1AG1_HUMAN	Alpha-1-acid glycoprotein 1 OS=Homo sapiens OX=9606 GN=ORM1 PE=1 SV=1	23.5	4.9	302.9	6	27.9	43.47	19			

20	VTDB_HUMAN	Vitamin D-binding protein OS=Homo sapiens OX=9606 GN=GC PE=1 SV=2	52.9	5.3	299.5	10	22.2	43.67	20			
21	CERU_HUMAN	Ceruloplasmin OS=Homo sapiens OX=9606 GN=CP PE=1 SV=1	122.1	5.4	277.1	7	7.6	45.57	21			
22	HPTR_HUMAN	Haptoglobin-related protein OS=Homo sapiens OX=9606 GN=HPR PE=2 SV=2	39.0	6.6	271.5	1	4.0	47.39	22			
23	A1BG_HUMAN	Alpha-1B-glycoprotein OS=Homo sapiens OX=9606 GN=A1BG PE=1 SV=4	54.2	5.6	270.3	8	17.8	41.51	23			
24	CFAB_HUMAN	Complement factor B OS=Homo sapiens OX=9606 GN=CFB PE=1 SV=2	85.5	6.7	259.8	5	7.6	45.71	24			
25	IGLC3_HUMAN	Immunoglobulin lambda constant 3 OS=Homo sapiens OX=9606 GN=IGLC3 PE=1 SV=1	11.3	6.9	244.6	5	62.3	38.32	25			
26	FETUA_HUMAN	Alpha-2-HS-glycoprotein OS=Homo sapiens OX=9606 GN=AHSG PE=1 SV=2	39.3	5.4	236.3	6	21.5	40.30	26			
27	KNG1_HUMAN	Kininogen-1 OS=Homo sapiens OX=9606 GN=KNG1 PE=1 SV=2	71.9	6.3	222.4	5	6.8	45.60	27			
28	AACT_HUMAN	Alpha-1-antichymotrypsin OS=Homo sapiens OX=9606 GN=SERPINA3 PE=1 SV=2	47.6	5.3	221.4	5	11.1	45.72	28			
29	IGHM_HUMAN	Immunoglobulin heavy constant mu OS=Homo sapiens OX=9606 GN=IGHM PE=1 SV=4	49.4	6.3	211.2	7	17.9	43.92	29			
30	ZA2G_HUMAN	Zinc-alpha-2-glycoprotein OS=Homo sapiens OX=9606 GN=AZGP1 PE=1 SV=2	34.2	5.7	206.5	6	23.5	44.38	30			
31	IGM_HUMAN	Immunoglobulin mu heavy chain OS=Homo sapiens OX=9606 PE=1 SV=2	63.4	8.1	189.7	1	1.2	46.04	31			
32	CFAH_HUMAN	Complement factor H OS=Homo sapiens OX=9606 GN=CFH PE=1 SV=4	139.0	6.2	186.9	6	5.5	43.69	32			
33	ANT3_HUMAN	Antithrombin-III OS=Homo sapiens OX=9606 GN=SERPINC1 PE=1 SV=1	52.6	6.3	185.9	6	14.7	43.80	33			
34	ITLN1_HUMAN	Intelectin-1 OS=Homo sapiens OX=9606 GN=ITLN1 PE=1 SV=1	34.9	5.7	185.4	3	10.9	44.30	34			
35	FIBG_HUMAN	Fibrinogen gamma chain OS=Homo sapiens OX=9606 GN=FGG PE=1 SV=3	51.5	5.4	183.9	5	13.2	44.25	35			
36	AFAM_HUMAN	Afamin OS=Homo sapiens OX=9606 GN=AFM PE=1 SV=1	69.0	5.6	173.7	5	9.2	46.04	36			
37	HRG_HUMAN	Histidine-rich glycoprotein OS=Homo sapiens OX=9606 GN=HRG PE=1 SV=1	59.5	7.1	167.7	3	7.0	45.85	37			
38	VTNC_HUMAN	Vitronectin OS=Homo sapiens OX=9606 GN=VTN PE=1 SV=1	54.3	5.6	154.5	3	8.4	47.28	38			
39	FIBB_HUMAN	Fibrinogen beta chain OS=Homo sapiens OX=9606 GN=FGB PE=1 SV=2	55.9	8.5	148.2	3	7.3	275.80	39			
40	APOH_HUMAN	Beta-2-glycoprotein 1 OS=Homo sapiens OX=9606 GN=APOH PE=1 SV=3	38.3	8.3	143.8	4	11.9	45.01	40			

41	A1AG2_HUMAN	Alpha-1-acid glycoprotein 2 OS=Homo sapiens OX=9606 GN=ORM2 PE=1 SV=2	23.6	5.0	141.6	3	11.9	42.12	41			
42	ITIH2_HUMAN	Inter-alpha-trypsin inhibitor heavy chain H2 OS=Homo sapiens OX=9606 GN=ITIH2 PE=1 SV=2	106.4	6.4	139.8	3	4.1	45.23	42			
43	THRB_HUMAN	Prothrombin OS=Homo sapiens OX=9606 GN=F2 PE=1 SV=2	70.0	5.6	133.8	3	5.1	47.00	43			
44	POTEE_HUMAN	POTE ankyrin domain family member E OS=Homo sapiens OX=9606 GN=POTEE PE=2 SV=3	121.3	5.8	132.5	4	4.0	42.18	44			
45	AMBP_HUMAN	Protein AMBP OS=Homo sapiens OX=9606 GN=AMBP PE=1 SV=1	39.0	5.9	132.0	3	7.7	319.68	45			
46	A2GL_HUMAN	Leucine-rich alpha-2-glycoprotein OS=Homo sapiens OX=9606 GN=LRG1 PE=1 SV=2	38.2	6.5	130.6	4	15.6	40.89	46			
47	LUM_HUMAN	Lumican OS=Homo sapiens OX=9606 GN=LUM PE=1 SV=2	38.4	6.2	128.0	4	12.4	41.41	47			
48	PGRP2_HUMAN	N-acetylmuramoyl-L-alanine amidase OS=Homo sapiens OX=9606 GN=PGLYRP2 PE=1 SV=1	62.2	7.3	115.0	2	4.7	46.67	48			
49	FINC_HUMAN	Fibronectin OS=Homo sapiens OX=9606 GN=FN1 PE=1 SV=5	272.1	5.3	114.1	4	1.9	46.55	49			
50	PLMN_HUMAN	Plasminogen OS=Homo sapiens OX=9606 GN=PLG PE=1 SV=2	90.5	7.0	107.3	4	4.6	45.57	50			
51	GELS_HUMAN	Gelsolin OS=Homo sapiens OX=9606 GN=GSN PE=1 SV=1	85.6	5.9	96.2	3	5.0	45.20	51			
52	APOA2_HUMAN	Apolipoprotein A-II OS=Homo sapiens OX=9606 GN=APOA2 PE=1 SV=1	11.2	6.3	86.3	3	21.0	48.51	52			
53	LV39_HUMAN	Immunoglobulin lambda variable 3-9 OS=Homo sapiens OX=9606 GN=IGLV3-9 PE=3 SV=1	12.3	6.8	82.7	1	13.9	45.02	53			
54	KV108_HUMAN	Immunoglobulin kappa variable 1-8 OS=Homo sapiens OX=9606 GN=IGKV1-8 PE=3 SV=1	12.5	9.2	78.6	1	13.9	43.51	54			
55	ITIH4_HUMAN	Inter-alpha-trypsin inhibitor heavy chain H4 OS=Homo sapiens OX=9606 GN=ITIH4 PE=1 SV=4	103.3	6.5	78.2	3	3.3	45.08	55			
56	MSLN_HUMAN	Mesothelin OS=Homo sapiens OX=9606 GN=MSLN PE=1 SV=2	68.9	6.0	76.2	3	5.4	572.57	56	4.60	1	0.00
57	FIBA_HUMAN	Fibrinogen alpha chain OS=Homo sapiens OX=9606 GN=FGA PE=1 SV=2	94.9	5.7	75.8	3	4.0	42.76	57			
58	FA12_HUMAN	Coagulation factor XII OS=Homo sapiens OX=9606 GN=F12 PE=1 SV=3	67.7	8.0	74.5	3	5.7	43.21	58			
59	KVD20_HUMAN	Immunoglobulin kappa variable 3D-20 OS=Homo sapiens OX=9606 GN=IGKV3D-20 PE=3 SV=1	12.5	4.5	74.0	2	27.6	40.27	59			

60	TTHY_HUMAN	Transthyretin OS=Homo sapiens OX=9606 GN=TTR PE=1 SV=1	15.9	5.5	73.2	2	18.4	47.26	60			
61	MACF1_HUMAN	Microtubule-actin cross-linking factor 1, isoforms 1/2/3/5 OS=Homo sapiens OX=9606 GN=MACF1 PE=1 SV=4	837.8	5.3	71.8	3	0.5	31.58	61			
62	KV320_HUMAN	Immunoglobulin kappa variable 3-20 OS=Homo sapiens OX=9606 GN=IGKV3-20 PE=1 SV=2	12.5	4.9	71.8	1	7.8	44.90	62			
63	TITIN_HUMAN	Titin OS=Homo sapiens OX=9606 GN=TTN PE=1 SV=4	3813.7	6.0	71.3	3	0.1	1086.00	63			
64	KDM5C_HUMAN	Lysine-specific demethylase 5C OS=Homo sapiens OX=9606 GN=KDM5C PE=1 SV=2	175.6	5.4	68.0	3	1.7	44.46	64			
65	WDR87_HUMAN	WD repeat-containing protein 87 OS=Homo sapiens OX=9606 GN=WDR87 PE=1 SV=3	333.0	6.9	67.1	3	0.9	532.39	65			
66	VIME_HUMAN	Vimentin OS=Homo sapiens OX=9606 GN=VIM PE=1 SV=4	53.6	5.1	64.5	2	5.2	47.23	66			
67	PRG4_HUMAN	Proteoglycan 4 OS=Homo sapiens OX=9606 GN=PRG4 PE=1 SV=3	151.0	9.5	64.5	2	2.4	43.49	67			
68	KVD28_HUMAN	Immunoglobulin kappa variable 2D-28 OS=Homo sapiens OX=9606 GN=IGKV2D-28 PE=1 SV=2	12.9	5.6	64.4	1	10.8	42.83	68			
69	IC1_HUMAN	Plasma protease C1 inhibitor OS=Homo sapiens OX=9606 GN=SERPING1 PE=1 SV=2	55.1	6.1	60.1	2	4.2	529.97	69			
70	CO5_HUMAN	Complement C5 OS=Homo sapiens OX=9606 GN=C5 PE=1 SV=4	188.2	6.1	59.7	2	1.1	710.78	70			
71	C1QC_HUMAN	Complement C1q subcomponent subunit C OS=Homo sapiens OX=9606 GN=C1QC PE=1 SV=3	25.8	8.6	59.5	2	9.0	48.02	71			
72	SYNE2_HUMAN	Nesprin-2 OS=Homo sapiens OX=9606 GN=SYNE2 PE=1 SV=3	795.9	5.3	58.6	1	0.1	54.74	72			
73	CFAI_HUMAN	Complement factor I OS=Homo sapiens OX=9606 GN=CFI PE=1 SV=2	65.7	7.7	57.8	2	3.9	48.51	73			
74	NEBU_HUMAN	Nebulin OS=Homo sapiens OX=9606 GN=NEB PE=1 SV=5	772.4	9.1	56.3	3	0.4	868.77	74			
75	ANGT_HUMAN	Angiotensinogen OS=Homo sapiens OX=9606 GN=AGT PE=1 SV=1	53.1	5.9	56.1	2	6.0	44.68	75			
76	KV311_HUMAN	Immunoglobulin kappa variable 3-11 OS=Homo sapiens OX=9606 GN=IGKV3-11 PE=1 SV=1	12.6	4.9	53.8	1	7.8	44.17	76			
77	P3C2B_HUMAN	Phosphatidylinositol 4-phosphate 3-kinase C2 domain-containing subunit beta OS=Homo sapiens OX=9606 GN=PIK3C2B PE=1 SV=2	184.7	6.9	53.0	2	0.9	27.27	77			

78	IDH3A_HUMAN	Isocitrate dehydrogenase [NAD] subunit alpha, mitochondrial OS=Homo sapiens OX=9606 GN=IDH3A PE=1 SV=1	39.6	6.5	51.5	1	2.7	14.85	78			
79	EVPL_HUMAN	Envoplakin OS=Homo sapiens OX=9606 GN=EVPL PE=1 SV=3	231.5	6.6	51.1	2	0.8	14.41	79			
80	RHG44_HUMAN	Rho GTPase-activating protein 44 OS=Homo sapiens OX=9606 GN=ARHGAP44 PE=1 SV=1	89.2	6.1	51.1	2	1.1	12.74	80	2.26	1	0.00
81	ZNF41_HUMAN	Zinc finger protein 41 OS=Homo sapiens OX=9606 GN=ZNF41 PE=1 SV=2	93.7	9.1	51.0	1	1.0	1052.52	81			
82	UN45A_HUMAN	Protein unc-45 homolog A OS=Homo sapiens OX=9606 GN=UNC45A PE=1 SV=1	103.0	5.8	49.7	1	1.1	55.10	82			
83	EPG5_HUMAN	Ectopic P granules protein 5 homolog OS=Homo sapiens OX=9606 GN=EPG5 PE=1 SV=2	292.3	6.0	49.0	2	0.6	868.44	83			
84	AKAP9_HUMAN	A-kinase anchor protein 9 OS=Homo sapiens OX=9606 GN=AKAP9 PE=1 SV=4	452.7	4.9	48.0	1	0.2	31.26	84			
85	CSCL2_HUMAN	CSC1-like protein 2 OS=Homo sapiens OX=9606 GN=TMEM63B PE=1 SV=1	94.9	7.2	47.2	2	1.9	366.04	85			
86	KVD33_HUMAN	Immunoglobulin kappa variable 1D-33 OS=Homo sapiens OX=9606 GN=IGKV1D-33 PE=1 SV=2	12.8	4.5	46.5	1	13.7	44.57	86			
87	ZFHX4_HUMAN	Zinc finger homeobox protein 4 OS=Homo sapiens OX=9606 GN=ZFHX4 PE=1 SV=1	393.5	5.9	46.4	1	0.2	24.68	87			
88	CO7_HUMAN	Complement component C7 OS=Homo sapiens OX=9606 GN=C7 PE=1 SV=2	93.5	6.1	46.3	2	2.0	45.19	88			
89	FHR1_HUMAN	Complement factor H-related protein 1 OS=Homo sapiens OX=9606 GN=CFHR1 PE=1 SV=2	37.6	7.4	46.2	1	3.9	48.09	89			
90	CHD9_HUMAN	Chromodomain-helicase-DNA-binding protein 9 OS=Homo sapiens OX=9606 GN=CHD9 PE=1 SV=2	325.8	6.6	45.2	1	0.3	20.13	90			
91	HV323_HUMAN	Immunoglobulin heavy variable 3-23 OS=Homo sapiens OX=9606 GN=IGHV3-23 PE=1 SV=2	12.6	8.5	42.5	2	18.8	43.58	91			
92	EZRI_HUMAN	Ezrin OS=Homo sapiens OX=9606 GN=EZR PE=1 SV=4	69.4	5.9	42.4	2	2.7	32.83	92			
93	CAPS1_HUMAN	Calcium-dependent secretion activator 1 OS=Homo sapiens OX=9606 GN=CADPS PE=1 SV=3	152.7	5.5	42.2	2	2.2	660.18	93			
94	ABCA3_HUMAN	ATP-binding cassette sub-family A member 3 OS=Homo sapiens OX=9606 GN=ABCA3 PE=1 SV=2	191.2	7.5	42.2	1	0.5	810.51	94			

95	WDR55_HUMAN	WD repeat-containing protein 55 OS=Homo sapiens OX=9606 GN=WDR55 PE=1 SV=2	42.0	4.8	42.1	2	4.4	679.61	95			
96	DOCK9_HUMAN	Dedicator of cytokinesis protein 9 OS=Homo sapiens OX=9606 GN=DOCK9 PE=1 SV=2	236.3	7.2	41.8	1	0.6	37.07	96			
97	KLC4_HUMAN	Kinesin light chain 4 OS=Homo sapiens OX=9606 GN=KLC4 PE=1 SV=3	68.6	5.8	41.4	1	1.9	15.11	97			
98	BUP1_HUMAN	Beta-ureidopropionase OS=Homo sapiens OX=9606 GN=UPB1 PE=1 SV=1	43.1	6.1	41.2	2	5.5	12.70	98			
99	MABP1_HUMAN	Mitogen-activated protein kinase-binding protein 1 OS=Homo sapiens OX=9606 GN=MAPKBP1 PE=1 SV=4	163.7	6.3	41.0	1	0.7	766.28	99			
100	BRCA2_HUMAN	Breast cancer type 2 susceptibility protein OS=Homo sapiens OX=9606 GN=BRCA2 PE=1 SV=3	384.0	6.3	40.9	1	0.4	697.25	100			
101	CO6_HUMAN	Complement component C6 OS=Homo sapiens OX=9606 GN=C6 PE=1 SV=3	104.7	6.4	40.8	1	0.9	46.98	101			
102	ARHGC_HUMAN	Rho guanine nucleotide exchange factor 12 OS=Homo sapiens OX=9606 GN=ARHGEF12 PE=1 SV=1	173.1	5.5	40.6	1	0.7	57.15	102			
103	UBP16_HUMAN	Ubiquitin carboxyl-terminal hydrolase 16 OS=Homo sapiens OX=9606 GN=USP16 PE=1 SV=1	93.5	6.5	40.3	1	1.0	1020.20	103			
104	RGS9_HUMAN	Regulator of G-protein signaling 9 OS=Homo sapiens OX=9606 GN=RGS9 PE=1 SV=1	76.9	9.4	39.8	2	3.1	14.50	104			
105	SPTN4_HUMAN	Spectrin beta chain, non-erythrocytic 4 OS=Homo sapiens OX=9606 GN=SPTBN4 PE=1 SV=2	288.8	5.7	39.5	1	0.3	24.89	105			
106	PEDF_HUMAN	Pigment epithelium-derived factor OS=Homo sapiens OX=9606 GN=SERPINF1 PE=1 SV=4	46.3	6.0	39.2	2	6.2	48.05	106			
107	PKHG1_HUMAN	Pleckstrin homology domain-containing family G member 1 OS=Homo sapiens OX=9606 GN=PLEKHG1 PE=1 SV=2	155.3	5.8	39.0	2	1.2	790.35	107			
108	ASTN2_HUMAN	Astrotactin-2 OS=Homo sapiens OX=9606 GN=ASTN2 PE=1 SV=2	148.1	5.7	38.8	2	1.3	1028.02	108			
109	S26A6_HUMAN	Solute carrier family 26 member 6 OS=Homo sapiens OX=9606 GN=SLC26A6 PE=1 SV=1	82.9	8.6	38.6	2	1.3	1118.68	109			
110	CP19A_HUMAN	Aromatase OS=Homo sapiens OX=9606 GN=CYP19A1 PE=1 SV=3	57.8	7.2	38.5	2	2.6	623.97	110			
111	OSBL6_HUMAN	Oxysterol-binding protein-related protein 6 OS=Homo sapiens OX=9606 GN=OSBPL6 PE=1 SV=1	106.2	6.5	38.4	2	2.1	36.23	111	1.89	1	0.00

112	SKT_HUMAN	Sickle tail protein homolog OS=Homo sapiens OX=9606 GN=KIAA1217 PE=1 SV=2	214.0	6.6	38.3	2	1.0	940.44	112			
113	PRR11_HUMAN	Proline-rich protein 11 OS=Homo sapiens OX=9606 GN=PRR11 PE=1 SV=1	40.1	10.1	38.2	1	2.5	6.03	113			
114	AR6P4_HUMAN	ADP-ribosylation factor-like protein 6-interacting protein 4 OS=Homo sapiens OX=9606 GN=ARL6IP4 PE=1 SV=2	44.9	10.9	38.2	2	6.4	718.94	114			
115	PEPL_HUMAN	Periplakin OS=Homo sapiens OX=9606 GN=PPL PE=1 SV=4	204.6	5.5	38.0	2	1.3	862.46	115			
116	FHAD1_HUMAN	Forkhead-associated domain-containing protein 1 OS=Homo sapiens OX=9606 GN=FHAD1 PE=2 SV=2	161.8	6.5	37.8	1	0.6	53.68	116			
117	GLI2_HUMAN	Zinc finger protein GLI2 OS=Homo sapiens OX=9606 GN=GLI2 PE=1 SV=4	167.7	6.9	37.7	2	1.5	664.68	117	0.22	1	0.00
118	RRBP1_HUMAN	Ribosome-binding protein 1 OS=Homo sapiens OX=9606 GN=RRBP1 PE=1 SV=5	152.4	8.7	37.3	2	1.4	715.23	118			
119	CK5P2_HUMAN	CDK5 regulatory subunit-associated protein 2 OS=Homo sapiens OX=9606 GN=CDK5RAP2 PE=1 SV=5	214.9	5.4	37.2	1	0.4	43.86	119			
120	TETN_HUMAN	Tetranectin OS=Homo sapiens OX=9606 GN=CLEC3B PE=1 SV=3	22.5	5.5	36.9	1	4.5	47.24	120			
121	FBLN3_HUMAN	EGF-containing fibulin-like extracellular matrix protein 1 OS=Homo sapiens OX=9606 GN=EFEMP1 PE=1 SV=2	54.6	5.0	36.7	1	1.8	44.61	121			
122	CBG_HUMAN	Corticosteroid-binding globulin OS=Homo sapiens OX=9606 GN=SERPINA6 PE=1 SV=1	45.1	5.6	36.5	1	2.5	41.67	122			
123	FMNL1_HUMAN	Formin-like protein 1 OS=Homo sapiens OX=9606 GN=FMNL1 PE=1 SV=3	121.8	5.6	36.1	2	1.5	48.85	123			
124	AHNK2_HUMAN	Protein AHNAK2 OS=Homo sapiens OX=9606 GN=AHNAK2 PE=1 SV=2	616.2	5.2	35.9	2	0.3	708.57	124			
125	CC178_HUMAN	Coiled-coil domain-containing protein 178 OS=Homo sapiens OX=9606 GN=CCDC178 PE=2 SV=3	101.9	6.3	35.9	2	2.1	720.62	125			
126	ZN512_HUMAN	Zinc finger protein 512 OS=Homo sapiens OX=9606 GN=ZNF512 PE=1 SV=2	64.6	9.8	35.9	2	3.5	774.09	126			
127	RET4_HUMAN	Retinol-binding protein 4 OS=Homo sapiens OX=9606 GN=RBP4 PE=1 SV=3	23.0	5.8	35.6	1	5.0	45.26	127			
128	CD44_HUMAN	CD44 antigen OS=Homo sapiens OX=9606 GN=CD44 PE=1 SV=3	81.5	5.1	35.5	1	1.6	45.83	128			
129	PRP16_HUMAN	Pre-mRNA-splicing factor ATP-dependent RNA helicase PRP16 OS=Homo sapiens OX=9606 GN=DHX38 PE=1 SV=2	140.4	6.1	35.2	2	1.4	700.92	129			

130	S22A1_HUMAN	Solute carrier family 22 member 1 OS=Homo sapiens OX=9606 GN=SLC22A1 PE=1 SV=2	61.1	6.4	35.1	2	4.5	868.39	130			
131	CE162_HUMAN	Centrosomal protein of 162 kDa OS=Homo sapiens OX=9606 GN=CEP162 PE=1 SV=2	161.8	5.4	34.9	2	0.7	851.47	131			
132	TET3_HUMAN	Methylcytosine dioxygenase TET3 OS=Homo sapiens OX=9606 GN=TET3 PE=1 SV=4	193.6	8.0	34.9	1	0.5	14.60	132			
133	AMOT_HUMAN	Angiomotin OS=Homo sapiens OX=9606 GN=AMOT PE=1 SV=1	118.0	7.3	34.7	1	0.9	65.19	133			
134	NAA25_HUMAN	N-alpha-acetyltransferase 25, NatB auxiliary subunit OS=Homo sapiens OX=9606 GN=NAA25 PE=1 SV=1	112.2	6.2	34.6	1	0.7	1137.69	134			
135	TBC15_HUMAN	TBC1 domain family member 15 OS=Homo sapiens OX=9606 GN=TBC1D15 PE=1 SV=2	79.4	5.4	34.3	2	2.6	31.44	135			
136	SAA2_HUMAN	Serum amyloid A-2 protein OS=Homo sapiens OX=9606 GN=SAA2 PE=1 SV=1	13.5	9.2	34.3	1	11.5	46.31	136			
137	SRBP2_HUMAN	Sterol regulatory element-binding protein 2 OS=Homo sapiens OX=9606 GN=SREBF2 PE=1 SV=2	123.6	8.7	34.2	1	1.0	1.51	137	1.50	1	0.00
138	HSPB1_HUMAN	Heat shock protein beta-1 OS=Homo sapiens OX=9606 GN=HSPB1 PE=1 SV=2	22.8	6.0	34.2	1	4.9	48.95	138			
139	UN13B_HUMAN	Protein unc-13 homolog B OS=Homo sapiens OX=9606 GN=UNC13B PE=1 SV=2	180.6	5.7	34.0	2	1.3	600.80	139			
140	ZN749_HUMAN	Zinc finger protein 749 OS=Homo sapiens OX=9606 GN=ZNF749 PE=1 SV=2	90.3	9.0	34.0	2	1.9	1069.42	140			
141	CO2_HUMAN	Complement C2 OS=Homo sapiens OX=9606 GN=C2 PE=1 SV=2	83.2	7.2	33.9	1	1.6	48.39	141			
142	NAV1_HUMAN	Neuron navigator 1 OS=Homo sapiens OX=9606 GN=NAV1 PE=1 SV=2	202.3	8.2	33.9	2	1.1	599.16	142			
143	SZT2_HUMAN	KICSTOR complex protein SZT2 OS=Homo sapiens OX=9606 GN=SZT2 PE=1 SV=3	377.8	5.9	33.8	2	0.4	1008.32	143			
144	PHLB2_HUMAN	Pleckstrin homology-like domain family B member 2 OS=Homo sapiens OX=9606 GN=PHLDB2 PE=1 SV=2	142.1	7.1	33.8	1	0.9	58.22	144			
145	FBN1_HUMAN	Fibrillin-1 OS=Homo sapiens OX=9606 GN=FBN1 PE=1 SV=4	312.1	4.8	33.7	1	0.4	11.52	145			
146	LAMB2_HUMAN	Laminin subunit beta-2 OS=Homo sapiens OX=9606 GN=LAMB2 PE=1 SV=2	195.9	6.1	33.5	1	0.8	659.99	146			

147	DCAF1_HUMAN	DDB1- and CUL4-associated factor 1 OS=Homo sapiens OX=9606 GN=DCAF1 PE=1 SV=3	168.9	4.9	33.4	1	0.7	4.75	147				
148	ASGR2_HUMAN	Asialoglycoprotein receptor 2 OS=Homo sapiens OX=9606 GN=ASGR2 PE=1 SV=2	35.1	5.8	33.3	1	2.3	1207.34	148				
149	LAMA3_HUMAN	Laminin subunit alpha-3 OS=Homo sapiens OX=9606 GN=LAMA3 PE=1 SV=2	366.4	7.0	33.3	1	0.2	72.76	149				
150	LV147_HUMAN	Immunoglobulin lambda variable 1-47 OS=Homo sapiens OX=9606 GN=IGLV1-47 PE=1 SV=2	12.3	5.6	33.3	1	11.1	42.56	150				
151	FOG2_HUMAN	Zinc finger protein ZFPM2 OS=Homo sapiens OX=9606 GN=ZFPM2 PE=1 SV=3	128.1	6.0	33.1	1	1.0	35.49	151				
152	SCAFB_HUMAN	Protein SCAF11 OS=Homo sapiens OX=9606 GN=SCAF11 PE=1 SV=2	164.6	8.7	32.7	2	1.3	979.89	152				
153	CE170_HUMAN	Centrosomal protein of 170 kDa OS=Homo sapiens OX=9606 GN=CEP170 PE=1 SV=1	175.2	6.6	32.7	1	0.6	916.27	153				
154	UN13A_HUMAN	Protein unc-13 homolog A OS=Homo sapiens OX=9606 GN=UNC13A PE=2 SV=4	192.9	5.2	32.4	1	0.5	7.08	154				
155	PPR21_HUMAN	Protein phosphatase 1 regulatory subunit 21 OS=Homo sapiens OX=9606 GN=PPP1R21 PE=1 SV=1	88.3	6.4	32.4	2	2.1	920.71	155				
156	BICD1_HUMAN	Protein bicaudal D homolog 1 OS=Homo sapiens OX=9606 GN=BICD1 PE=1 SV=3	110.7	5.6	32.3	1	1.1	46.34	156				
157	HEAT3_HUMAN	HEAT repeat-containing protein 3 OS=Homo sapiens OX=9606 GN=HEATR3 PE=1 SV=2	74.5	5.0	32.2	1	2.4	28.46	157				
158	CC186_HUMAN	Coiled-coil domain-containing protein 186 OS=Homo sapiens OX=9606 GN=CCDC186 PE=1 SV=2	103.6	5.9	32.1	1	1.4	17.30	158				
159	KV315_HUMAN	Immunoglobulin kappa variable 3-15 OS=Homo sapiens OX=9606 GN=IGKV3-15 PE=1 SV=2	12.5	5.1	31.9	1	7.8	40.58	159				
160	NID1_HUMAN	Nidogen-1 OS=Homo sapiens OX=9606 GN=NID1 PE=1 SV=3	136.3	5.1	31.9	1	0.6	1116.07	160				
161	CO6A5_HUMAN	Collagen alpha-5(VI) chain OS=Homo sapiens OX=9606 GN=COL6A5 PE=1 SV=1	289.7	6.5	31.8	1	0.3	31.66	161				
162	MBD5_HUMAN	Methyl-CpG-binding domain protein 5 OS=Homo sapiens OX=9606 GN=MBD5 PE=1 SV=3	159.8	9.2	31.7	2	0.7	772.53	162				

163	DYH11_HUMAN	Dynein heavy chain 11, axonemal OS=Homo sapiens OX=9606 GN=DNAH11 PE=1 SV=4	520.0	6.0	31.5	1	0.3	27.16	163			
164	ERAP1_HUMAN	Endoplasmic reticulum aminopeptidase 1 OS=Homo sapiens OX=9606 GN=ERAP1 PE=1 SV=3	107.2	6.0	31.3	1	1.1	55.25	164			
165	XPO2_HUMAN	Exportin-2 OS=Homo sapiens OX=9606 GN=CSE1L PE=1 SV=3	110.3	5.5	31.3	1	0.8	46.32	165			
166	PHF1_HUMAN	PHD finger protein 1 OS=Homo sapiens OX=9606 GN=PHF1 PE=1 SV=3	62.1	9.3	31.2	1	1.4	6.49	166	1.42	1	0.00
167	CC033_HUMAN	Protein C3orf33 OS=Homo sapiens OX=9606 GN=C3orf33 PE=1 SV=2	33.7	9.8	30.1	1	3.1	48.99	167			
168	HV349_HUMAN	Immunoglobulin heavy variable 3-49 OS=Homo sapiens OX=9606 GN=IGHV3-49 PE=3 SV=1	13.0	8.8	29.9	1	7.6	44.90	168			
169	CDK5_HUMAN	Cyclin-dependent-like kinase 5 OS=Homo sapiens OX=9606 GN=CDK5 PE=1 SV=3	33.3	7.6	29.4	1	3.1	8.14	169			
170	SH24B_HUMAN	SH2 domain-containing protein 4B OS=Homo sapiens OX=9606 GN=SH2D4B PE=2 SV=1	51.2	5.9	28.9	1	2.1	24.85	170			
171	ATLA1_HUMAN	Atlastin-1 OS=Homo sapiens OX=9606 GN=ATL1 PE=1 SV=1	63.5	5.8	28.5	1	1.6	934.45	171			
172	KV401_HUMAN	Immunoglobulin kappa variable 4-1 OS=Homo sapiens OX=9606 GN=IGKV4-1 PE=1 SV=1	13.4	5.1	28.3	1	7.4	41.48	172			
173	TIM_HUMAN	Protein timeless homolog OS=Homo sapiens OX=9606 GN=TIMELESS PE=1 SV=2	138.6	5.3	27.9	1	0.7	1154.36	173			
174	RNPC3_HUMAN	RNA-binding region-containing protein 3 OS=Homo sapiens OX=9606 GN=RNPC3 PE=1 SV=1	58.5	7.6	27.7	1	1.9	11.57	174			
175	CAC1C_HUMAN	Voltage-dependent L-type calcium channel subunit alpha-1C OS=Homo sapiens OX=9606 GN=CACNA1C PE=1 SV=4	248.8	6.3	27.6	1	0.8	47.58	175			
176	PCGF2_HUMAN	Polycomb group RING finger protein 2 OS=Homo sapiens OX=9606 GN=PCGF2 PE=1 SV=1	37.8	8.2	27.5	1	3.5	820.74	176			
177	KI16B_HUMAN	Kinesin-like protein KIF16B OS=Homo sapiens OX=9606 GN=KIF16B PE=1 SV=2	151.9	5.9	27.4	1	0.7	24.39	177			
178	KIF15_HUMAN	Kinesin-like protein KIF15 OS=Homo sapiens OX=9606 GN=KIF15 PE=1 SV=1	160.1	5.7	26.6	1	0.6	3.42	178			
179	NDUF2_HUMAN	NADH dehydrogenase [ubiquinone] 1 alpha subcomplex assembly factor 2	19.8	8.9	26.5	1	4.1	58.85	179			

		OS=Homo sapiens OX=9606 GN=NDUFAF2 PE=1 SV=1										
180	LIPR3_HUMAN	Pancreatic lipase-related protein 3 OS=Homo sapiens OX=9606 GN=PNLIPRP3 PE=2 SV=2	52.2	8.6	26.1	1	1.9	27.85	180			
181	VWA3A_HUMAN	von Willebrand factor A domain-containing protein 3A OS=Homo sapiens OX=9606 GN=VWA3A PE=2 SV=3	133.9	8.7	26.0	1	0.8	35.24	181			
182	EZHIP_HUMAN	EZH inhibitory protein OS=Homo sapiens OX=9606 GN=EZHIP PE=1 SV=1	51.9	10.1	26.0	1	2.2	946.03	182			
183	HOIL1_HUMAN	RanBP-type and C3HC4-type zinc finger-containing protein 1 OS=Homo sapiens OX=9606 GN=RBCK1 PE=1 SV=2	57.5	5.5	26.0	1	2.2	38.57	183			
184	CO1A1_HUMAN	Collagen alpha-1(I) chain OS=Homo sapiens OX=9606 GN=COL1A1 PE=1 SV=5	138.9	5.6	26.0	1	0.8	8.39	184			
185	KCC1A_HUMAN	Calcium/calmodulin-dependent protein kinase type 1 OS=Homo sapiens OX=9606 GN=CAMK1 PE=1 SV=1	41.3	5.1	26.0	1	2.7	859.11	185			
186	F120A_HUMAN	Constitutive coactivator of PPAR-gamma-like protein 1 OS=Homo sapiens OX=9606 GN=FAM120A PE=1 SV=2	121.8	9.1	25.8	1	1.7	20.37	186			
187	IGJ_HUMAN	Immunoglobulin J chain OS=Homo sapiens OX=9606 GN=JCHAIN PE=1 SV=4	18.1	5.1	25.7	1	6.9	47.22	187			
188	CRAD_HUMAN	Cancer-related regulator of actin dynamics OS=Homo sapiens OX=9606 GN=CRAD PE=1 SV=3	136.7	5.5	25.7	1	0.7	71.79	188			
189	ZN831_HUMAN	Zinc finger protein 831 OS=Homo sapiens OX=9606 GN=ZNF831 PE=2 SV=4	177.8	8.7	25.7	1	1.0	5.91	189	0.25	1	0.00
190	SPKAP_HUMAN	A-kinase anchor protein SPHKAP OS=Homo sapiens OX=9606 GN=SPHKAP PE=1 SV=1	186.3	5.0	25.6	1	0.9	5.01	190			
191	HV313_HUMAN	Immunoglobulin heavy variable 3-13 OS=Homo sapiens OX=9606 GN=IGHV3-13 PE=1 SV=2	12.5	6.5	25.5	1	9.5	31.85	191			
192	FETUB_HUMAN	Fetuin-B OS=Homo sapiens OX=9606 GN=FETUB PE=1 SV=2	42.0	6.5	25.4	1	2.1	45.18	192			
193	CO9_HUMAN	Complement component C9 OS=Homo sapiens OX=9606 GN=C9 PE=1 SV=2	63.1	5.4	25.4	1	2.0	47.18	193			
194	SPT6H_HUMAN	Transcription elongation factor SPT6 OS=Homo sapiens OX=9606 GN=SUPT6H PE=1 SV=2	198.9	4.8	25.3	1	0.5	40.39	194			

195	TM100_HUMAN	Transmembrane protein 100 OS=Homo sapiens OX=9606 GN=TMEM100 PE=1 SV=2	14.4	9.6	25.3	1	9.0	19.21	195				
196	USP9Y_HUMAN	Probable ubiquitin carboxyl-terminal hydrolase FAF-Y OS=Homo sapiens OX=9606 GN=USP9Y PE=2 SV=2	290.9	5.6	25.2	1	0.5	15.69	196				
197	PCH2_HUMAN	Pachytene checkpoint protein 2 homolog OS=Homo sapiens OX=9606 GN=TRIP13 PE=1 SV=2	48.5	5.7	25.1	1	3.9	33.93	197				
198	RBM19_HUMAN	Probable RNA-binding protein 19 OS=Homo sapiens OX=9606 GN=RBM19 PE=1 SV=3	107.3	6.1	25.1	1	1.0	959.63	198				
199	MYO1H_HUMAN	Unconventional myosin-Ih OS=Homo sapiens OX=9606 GN=MYO1H PE=1 SV=2	119.0	9.2	25.0	1	1.0	23.05	199				
200	ZRAB3_HUMAN	DNA annealing helicase and endonuclease ZRANB3 OS=Homo sapiens OX=9606 GN=ZRANB3 PE=1 SV=2	123.2	8.7	24.9	1	1.0	57.28	200				
201	ACADV_HUMAN	Very long-chain specific acyl-CoA dehydrogenase, mitochondrial OS=Homo sapiens OX=9606 GN=ACADVL PE=1 SV=1	70.3	8.9	24.9	1	1.8	789.41	201				
202	NOS2_HUMAN	Nitric oxide synthase, inducible OS=Homo sapiens OX=9606 GN=NOS2 PE=1 SV=2	131.0	8.2	24.7	1	1.1	48.91	202				
203	GLYL1_HUMAN	Glycine N-acyltransferase-like protein 1 OS=Homo sapiens OX=9606 GN=GLYATL1 PE=1 SV=1	35.1	6.4	24.7	1	3.3	12.20	203				
204	HV551_HUMAN	Immunoglobulin heavy variable 5-51 OS=Homo sapiens OX=9606 GN=IGHV5-51 PE=3 SV=1	12.7	8.4	24.6	1	11.1	46.88	204				
205	CSC2A_HUMAN	Protein CASC2, isoform 3 OS=Homo sapiens OX=9606 GN=CASC2 PE=2 SV=1	11.9	8.5	24.5	1	8.8	51.21	205				
206	PTPRM_HUMAN	Receptor-type tyrosine-protein phosphatase mu OS=Homo sapiens OX=9606 GN=PTPRM PE=1 SV=2	163.6	6.2	24.5	1	0.8	26.11	206				
207	CD22_HUMAN	B-cell receptor CD22 OS=Homo sapiens OX=9606 GN=CD22 PE=1 SV=2	95.3	6.2	24.4	1	1.4	50.87	207				
208	WDR7_HUMAN	WD repeat-containing protein 7 OS=Homo sapiens OX=9606 GN=WDR7 PE=1 SV=2	163.7	6.5	24.4	1	0.5	23.73	208				
209	ZN554_HUMAN	Zinc finger protein 554 OS=Homo sapiens OX=9606 GN=ZNF554 PE=1 SV=1	60.5	7.6	24.4	1	1.5	952.43	209				
210	TSN14_HUMAN	Tetraspanin-14 OS=Homo sapiens OX=9606 GN=TSPAN14 PE=1 SV=1	30.7	6.4	24.3	1	3.0	44.90	210				

211	KDM1A_HUMAN	Lysine-specific histone demethylase 1A OS=Homo sapiens OX=9606 GN=KDM1A PE=1 SV=2	92.8	6.1	24.3	1	1.3	771.41	211				
212	GTD2A_HUMAN	General transcription factor II-I repeat domain-containing protein 2A OS=Homo sapiens OX=9606 GN=GTF2IRD2 PE=1 SV=3	107.1	5.5	24.3	1	1.1	42.71	212				
213	RGS13_HUMAN	Regulator of G-protein signaling 13 OS=Homo sapiens OX=9606 GN=RGS13 PE=2 SV=1	19.1	9.1	24.1	1	4.4	17.92	213				
214	GEMI5_HUMAN	Gem-associated protein 5 OS=Homo sapiens OX=9606 GN=GEMIN5 PE=1 SV=3	168.5	6.2	24.1	1	0.7	910.49	214				
215	TAPT1_HUMAN	Transmembrane anterior posterior transformation protein 1 homolog OS=Homo sapiens OX=9606 GN=TAPT1 PE=1 SV=1	64.2	8.6	23.9	1	1.2	10.46	215				
216	LRC39_HUMAN	Leucine-rich repeat-containing protein 39 OS=Homo sapiens OX=9606 GN=LRRC39 PE=1 SV=1	38.8	6.0	23.9	1	2.1	1138.93	216				
217	C1S_HUMAN	Complement C1s subcomponent OS=Homo sapiens OX=9606 GN=C1S PE=1 SV=1	76.6	4.8	23.9	1	1.2	44.45	217				
218	AT10D_HUMAN	Probable phospholipid-transporting ATPase VD OS=Homo sapiens OX=9606 GN=ATP10D PE=2 SV=3	160.2	6.8	23.9	1	0.8	9.45	218				
219	ZN622_HUMAN	Zinc finger protein 622 OS=Homo sapiens OX=9606 GN=ZNF622 PE=1 SV=1	54.2	5.8	23.8	1	2.5	811.70	219				
220	THOC6_HUMAN	THO complex subunit 6 homolog OS=Homo sapiens OX=9606 GN=THOC6 PE=1 SV=1	37.5	7.1	23.7	1	4.4	10.27	220				
221	GGYF2_HUMAN	GRB10-interacting GYF protein 2 OS=Homo sapiens OX=9606 GN=GIGYF2 PE=1 SV=1	150.0	5.4	23.5	1	1.0	26.49	221				
222	RBG10_HUMAN	Rab GTPase-activating protein 1-like, isoform 10 OS=Homo sapiens OX=9606 GN=RABGAP1L PE=1 SV=1	29.0	5.3	23.5	1	2.8	89.22	222				
223	KPYM_HUMAN	Pyruvate kinase PKM OS=Homo sapiens OX=9606 GN=PKM PE=1 SV=4	57.9	8.0	23.4	1	2.4	47.07	223				
224	CK052_HUMAN	Uncharacterized protein C11orf52 OS=Homo sapiens OX=9606 GN=C11orf52 PE=1 SV=2	13.9	9.5	23.4	1	8.9	30.08	224				
225	RAB21_HUMAN	Ras-related protein Rab-21 OS=Homo sapiens OX=9606 GN=RAB21 PE=1 SV=3	24.3	8.1	23.3	1	6.7	43.00	225				

226	RNF17_HUMAN	RING finger protein 17 OS=Homo sapiens OX=9606 GN=RNF17 PE=1 SV=3	184.5	5.3	23.3	1	0.6	723.70	226			
227	NDUA9_HUMAN	NADH dehydrogenase [ubiquinone] 1 alpha subcomplex subunit 9, mitochondrial OS=Homo sapiens OX=9606 GN=NDUFA9 PE=1 SV=2	42.5	9.8	23.2	1	2.9	19.36	227			
228	RGAP1_HUMAN	Rac GTPase-activating protein 1 OS=Homo sapiens OX=9606 GN=RACGAP1 PE=1 SV=1	71.0	9.1	23.1	1	1.4	22.37	228			
229	AOFA_HUMAN	Amine oxidase [flavin-containing] A OS=Homo sapiens OX=9606 GN=MAOA PE=1 SV=1	59.6	7.9	23.1	1	1.9	34.65	229			
230	GCYA1_HUMAN	Guanylate cyclase soluble subunit alpha-1 OS=Homo sapiens OX=9606 GN=GUCY1A1 PE=1 SV=2	77.4	6.7	23.1	1	1.3	950.37	230			
231	GALT_HUMAN	Galactose-1-phosphate uridylyltransferase OS=Homo sapiens OX=9606 GN=GALT PE=1 SV=3	43.3	6.5	23.1	1	2.6	40.73	231			
232	ZN696_HUMAN	Zinc finger protein 696 OS=Homo sapiens OX=9606 GN=ZNF696 PE=2 SV=2	40.5	9.3	22.9	1	2.7	1107.95	232			
233	CNTN2_HUMAN	Contactin-2 OS=Homo sapiens OX=9606 GN=CNTN2 PE=1 SV=1	113.3	8.1	22.9	1	1.0	9.65	233			
234	ABRX2_HUMAN	BRISC complex subunit Abraxas 2 OS=Homo sapiens OX=9606 GN=ABRAXAS2 PE=1 SV=2	46.9	5.8	22.9	1	2.2	865.73	234			
235	NKRF_HUMAN	NF-kappa-B-repressing factor OS=Homo sapiens OX=9606 GN=NKRF PE=1 SV=2	77.6	8.9	22.8	1	2.0	585.37	235			
236	EHBP1_HUMAN	EH domain-binding protein 1 OS=Homo sapiens OX=9606 GN=EHBP1 PE=1 SV=3	139.9	5.2	22.8	1	0.6	46.61	236			
237	KLK11_HUMAN	Kallikrein-11 OS=Homo sapiens OX=9606 GN=KLK11 PE=1 SV=2	31.0	9.2	22.8	1	6.7	448.92	237			
238	ZC12C_HUMAN	Probable ribonuclease ZC3H12C OS=Homo sapiens OX=9606 GN=ZC3H12C PE=1 SV=2	99.3	6.5	22.8	1	1.0	25.45	238			
239	PITM2_HUMAN	Membrane-associated phosphatidylinositol transfer protein 2 OS=Homo sapiens OX=9606 GN=PITPNM2 PE=1 SV=1	148.8	6.7	22.6	1	0.7	37.59	239			
240	ATRX_HUMAN	Transcriptional regulator ATRX OS=Homo sapiens OX=9606 GN=ATRX PE=1 SV=5	282.4	6.2	22.6	1	0.4	813.76	240			
241	PLXB3_HUMAN	Plexin-B3 OS=Homo sapiens OX=9606 GN=PLXNB3 PE=1 SV=2	206.7	6.0	22.4	1	0.5	4.58	241			

242	HV118_HUMAN	Immunoglobulin heavy variable 1-18 OS=Homo sapiens OX=9606 GN=IGHV1-18 PE=3 SV=1	12.8	9.0	22.4	1	9.4	726.41	242			
243	SAA4_HUMAN	Serum amyloid A-4 protein OS=Homo sapiens OX=9606 GN=SAA4 PE=1 SV=2	14.7	9.2	22.4	1	11.5	569.86	243			
244	LMAN2_HUMAN	Vesicular integral-membrane protein VIP36 OS=Homo sapiens OX=9606 GN=LMAN2 PE=1 SV=1	40.2	6.5	22.4	1	2.5	32.22	244			
245	TRIPB_HUMAN	Thyroid receptor-interacting protein 11 OS=Homo sapiens OX=9606 GN=TRIP11 PE=1 SV=3	227.4	5.2	22.2	1	0.5	32.49	245			
246	SYFB_HUMAN	Phenylalanine--tRNA ligase beta subunit OS=Homo sapiens OX=9606 GN=FARSB PE=1 SV=3	66.1	6.4	22.1	1	1.2	39.92	246			
247	ANR29_HUMAN	Ankyrin repeat domain-containing protein 29 OS=Homo sapiens OX=9606 GN=ANKRD29 PE=2 SV=2	32.4	9.5	22.1	1	5.3	29.78	247			
248	SIR1_HUMAN	NAD-dependent protein deacetylase sirtuin-1 OS=Homo sapiens OX=9606 GN=SIRT1 PE=1 SV=2	81.6	4.6	22.0	1	1.1	23.75	248			
249	IPO11_HUMAN	Importin-11 OS=Homo sapiens OX=9606 GN=IPO11 PE=1 SV=1	112.5	5.1	21.9	1	1.4	552.19	249			
250	CE295_HUMAN	Centrosomal protein of 295 kDa OS=Homo sapiens OX=9606 GN=CEP295 PE=1 SV=4	295.0	5.7	21.9	1	0.4	692.19	250			
251	APOM_HUMAN	Apolipoprotein M OS=Homo sapiens OX=9606 GN=APOM PE=1 SV=2	21.2	5.7	21.9	1	3.7	46.05	251			
252	CAPR2_HUMAN	Caprin-2 OS=Homo sapiens OX=9606 GN=CAPRIN2 PE=1 SV=1	125.8	6.3	21.9	1	0.6	33.85	252			
253	ENKUR_HUMAN	Enkurin OS=Homo sapiens OX=9606 GN=ENKUR PE=2 SV=1	29.4	9.3	21.8	1	5.1	6.01	253			
254	TTL11_HUMAN	Tubulin polyglutamylase TTLL11 OS=Homo sapiens OX=9606 GN=TTLL11 PE=1 SV=2	87.6	9.1	21.8	1	1.3	44.86	254			
255	KI26A_HUMAN	Kinesin-like protein KIF26A OS=Homo sapiens OX=9606 GN=KIF26A PE=1 SV=3	194.5	9.1	21.7	1	0.6	692.36	255			
256	GP155_HUMAN	Integral membrane protein GPR155 OS=Homo sapiens OX=9606 GN=GPR155 PE=2 SV=2	96.9	6.4	21.6	1	1.6	560.77	256			
257	DSE_HUMAN	Dermatan-sulfate epimerase OS=Homo sapiens OX=9606 GN=DSE PE=1 SV=1	109.7	8.2	21.6	1	0.8	38.00	257			
258	PDS5A_HUMAN	Sister chromatid cohesion protein PDS5 homolog A OS=Homo sapiens OX=9606 GN=PDS5A PE=1 SV=1	150.7	8.2	21.6	1	0.5	42.95	258			

259	YT001_HUMAN	Putative uncharacterized protein PRO0628 OS=Homo sapiens OX=9606 GN=PRO0628 PE=5 SV=1	6.4	10.3	21.5	1	18.2	75.91	259				
260	HNRPU_HUMAN	Heterogeneous nuclear ribonucleoprotein U OS=Homo sapiens OX=9606 GN=HNRNPU PE=1 SV=6	90.5	5.8	21.5	1	1.2	26.70	260				
261	WNT16_HUMAN	Protein Wnt-16 OS=Homo sapiens OX=9606 GN=WNT16 PE=2 SV=1	40.7	9.0	21.5	1	3.8	553.87	261				
262	TEN4_HUMAN	Teneurin-4 OS=Homo sapiens OX=9606 GN=TENM4 PE=1 SV=2	307.8	6.1	21.4	1	0.3	26.50	262				
263	CA137_HUMAN	Putative uncharacterized protein C1orf137 OS=Homo sapiens OX=9606 GN=C1orf137 PE=4 SV=1	11.0	7.6	21.4	1	9.2	931.56	263				
264	MPP5_HUMAN	MAGUK p55 subfamily member 5 OS=Homo sapiens OX=9606 GN=MPP5 PE=1 SV=3	77.2	5.8	21.4	1	1.5	876.81	264				
265	DCTN5_HUMAN	Dynactin subunit 5 OS=Homo sapiens OX=9606 GN=DCTN5 PE=1 SV=1	20.1	8.3	21.4	1	5.5	761.46	265				
266	LRC41_HUMAN	Leucine-rich repeat-containing protein 41 OS=Homo sapiens OX=9606 GN=LRRC41 PE=1 SV=3	88.6	8.8	21.3	1	1.2	17.59	266				
267	BMP15_HUMAN	Bone morphogenetic protein 15 OS=Homo sapiens OX=9606 GN=BMP15 PE=1 SV=2	45.0	9.3	21.3	1	2.6	26.96	267				
268	SSU72_HUMAN	RNA polymerase II subunit A C-terminal domain phosphatase SSU72 OS=Homo sapiens OX=9606 GN=SSU72 PE=1 SV=1	22.6	5.2	21.1	1	3.6	69.21	268				
269	IMA1_HUMAN	Importin subunit alpha-1 OS=Homo sapiens OX=9606 GN=KPNA2 PE=1 SV=1	57.8	5.3	21.1	1	1.7	848.72	269				
270	AHDC1_HUMAN	AT-hook DNA-binding motif-containing protein 1 OS=Homo sapiens OX=9606 GN=AHDC1 PE=1 SV=1	168.2	9.2	21.1	1	0.7	770.09	270				
271	HEP2_HUMAN	Heparin cofactor 2 OS=Homo sapiens OX=9606 GN=SERPIND1 PE=1 SV=3	57.0	6.4	21.1	1	1.8	47.44	271				
272	TEKT4_HUMAN	Tektin-4 OS=Homo sapiens OX=9606 GN=TEKT4 PE=1 SV=1	50.6	6.0	21.0	1	2.3	57.55	272				
273	TTC7B_HUMAN	Tetratricopeptide repeat protein 7B OS=Homo sapiens OX=9606 GN=TTC7B PE=1 SV=3	94.1	6.4	21.0	1	2.5	29.28	273				
274	KCNA4_HUMAN	Potassium voltage-gated channel subfamily A member 4 OS=Homo sapiens OX=9606 GN=KCNA4 PE=1 SV=2	73.2	5.1	21.0	1	2.3	628.34	274				
275	CBPC5_HUMAN	Cytosolic carboxypeptidase-like protein 5 OS=Homo sapiens OX=9606 GN=AGBL5 PE=1 SV=1	97.5	9.3	21.0	1	1.2	811.87	275				

276	ZN624_HUMAN	Zinc finger protein 624 OS=Homo sapiens OX=9606 GN=ZNF624 PE=1 SV=3	99.9	9.1	20.9	1	1.2	63.70	276			
277	FURIN_HUMAN	Furin OS=Homo sapiens OX=9606 GN=FURIN PE=1 SV=2	86.6	6.0	20.9	1	1.0	58.63	277			
278	ZN197_HUMAN	Zinc finger protein 197 OS=Homo sapiens OX=9606 GN=ZNF197 PE=2 SV=1	118.8	8.9	20.7	1	0.7	1110.09	278			
279	RS4X_HUMAN	40S ribosomal protein S4, X isoform OS=Homo sapiens OX=9606 GN=RPS4X PE=1 SV=2	29.6	10.2	20.7	1	3.8	57.58	279			
280	MRM2_HUMAN	rRNA methyltransferase 2, mitochondrial OS=Homo sapiens OX=9606 GN=MRM2 PE=1 SV=1	27.4	9.6	20.6	1	2.8	1068.81	280			
281	XPO1_HUMAN	Exportin-1 OS=Homo sapiens OX=9606 GN=XPO1 PE=1 SV=1	123.3	5.7	20.6	1	0.8	799.59	281			
282	UT14C_HUMAN	U3 small nucleolar RNA-associated protein 14 homolog C OS=Homo sapiens OX=9606 GN=UTP14C PE=1 SV=1	87.1	6.7	20.6	1	1.0	6.91	282			
283	TM246_HUMAN	Transmembrane protein 246 OS=Homo sapiens OX=9606 GN=TMEM246 PE=1 SV=1	46.6	7.3	20.5	1	2.2	892.24	283			
284	DCR1A_HUMAN	DNA cross-link repair 1A protein OS=Homo sapiens OX=9606 GN=DCLRE1A PE=1 SV=3	116.3	8.2	20.5	1	1.3	15.53	284			
285	ZN184_HUMAN	Zinc finger protein 184 OS=Homo sapiens OX=9606 GN=ZNF184 PE=1 SV=4	86.1	8.4	20.5	1	1.2	981.00	285			
286	SNP29_HUMAN	Synaptosomal-associated protein 29 OS=Homo sapiens OX=9606 GN=SNAP29 PE=1 SV=1	29.0	5.6	20.3	1	4.7	718.11	286			
287	AP1S3_HUMAN	AP-1 complex subunit sigma-3 OS=Homo sapiens OX=9606 GN=AP1S3 PE=1 SV=1	18.3	6.3	20.3	1	5.8	28.09	287			
288	IMPG1_HUMAN	Interphotoreceptor matrix proteoglycan 1 OS=Homo sapiens OX=9606 GN=IMPG1 PE=1 SV=2	89.3	4.8	20.3	1	1.0	65.60	288			
289	MEF2B_HUMAN	Myocyte-specific enhancer factor 2B OS=Homo sapiens OX=9606 GN=MEF2B PE=1 SV=2	38.6	9.6	20.3	1	2.5	1149.64	289			
290	LCORL_HUMAN	Ligand-dependent nuclear receptor corepressor-like protein OS=Homo sapiens OX=9606 GN=LCORL PE=1 SV=4	66.9	8.2	20.3	1	1.5	916.84	290			
291	LIMK1_HUMAN	LIM domain kinase 1 OS=Homo sapiens OX=9606 GN=LIMK1 PE=1 SV=3	72.5	6.5	20.3	1	3.1	27.06	291			

292	RLGPB_HUMAN	Ral GTPase-activating protein subunit beta OS=Homo sapiens OX=9606 GN=RALGAPB PE=1 SV=1	166.7	6.3	20.2	1	0.7	54.22	292			
293	KR87P_HUMAN	Putative keratin-87 protein OS=Homo sapiens OX=9606 GN=KRT87P PE=5 SV=4	29.1	5.6	20.2	1	4.3	26.63	293			
294	DNMT1_HUMAN	DNA (cytosine-5)-methyltransferase 1 OS=Homo sapiens OX=9606 GN=DNMT1 PE=1 SV=2	183.0	8.0	20.2	1	1.2	4.35	294			
295	PHX2A_HUMAN	Paired mesoderm homeobox protein 2A OS=Homo sapiens OX=9606 GN=PHOX2A PE=1 SV=2	29.6	9.0	20.2	1	3.5	27.02	295			
296	EFC4B_HUMAN	EF-hand calcium-binding domain-containing protein 4B OS=Homo sapiens OX=9606 GN=CRACR2A PE=1 SV=1	45.6	4.9	20.2	1	2.5	16.97	296	1.50	1	0.00
297	TLK1_HUMAN	Serine/threonine-protein kinase tousled-like 1 OS=Homo sapiens OX=9606 GN=TLK1 PE=1 SV=2	86.6	8.9	20.1	1	1.0	1084.07	297			
298	CLMP_HUMAN	CXADR-like membrane protein OS=Homo sapiens OX=9606 GN=CLMP PE=1 SV=1	41.3	8.1	20.0	1	3.5	678.74	298			
299	NU205_HUMAN	Nuclear pore complex protein Nup205 OS=Homo sapiens OX=9606 GN=NUP205 PE=1 SV=3	227.8	5.8	20.0	1	0.4	922.75	299	1.08	1	0.00
300	MFN2_HUMAN	Mitofusin-2 OS=Homo sapiens OX=9606 GN=MFN2 PE=1 SV=3	86.3	6.5	20.0	1	2.8	0.22	300			
301	NUD15_HUMAN	Nucleotide triphosphate diphosphatase NUDT15 OS=Homo sapiens OX=9606 GN=NUDT15 PE=1 SV=1	18.6	5.7	20.0	1	5.5	34.92	301			
302	TGT_HUMAN	Queuine tRNA-ribosyltransferase catalytic subunit 1 OS=Homo sapiens OX=9606 GN=QTRT1 PE=1 SV=3	44.0	6.8	20.0	1	2.2	912.71	302			
303	DPOD2_HUMAN	DNA polymerase delta subunit 2 OS=Homo sapiens OX=9606 GN=POLD2 PE=1 SV=1	51.3	5.3	19.9	1	1.9	995.80	303			
304	FBLN1_HUMAN	Fibulin-1 OS=Homo sapiens OX=9606 GN=FBLN1 PE=1 SV=4	77.2	5.1	19.9	1	1.4	878.42	304			
305	CCHCR_HUMAN	Coiled-coil alpha-helical rod protein 1 OS=Homo sapiens OX=9606 GN=CCHCR1 PE=1 SV=2	88.6	5.8	19.9	1	1.2	28.10	305			
306	ATP4A_HUMAN	Potassium-transporting ATPase alpha chain 1 OS=Homo sapiens OX=9606 GN=ATP4A PE=2 SV=5	114.0	5.6	19.9	1	2.0	23.00	306			
307	KANL1_HUMAN	KAT8 regulatory NSL complex subunit 1 OS=Homo sapiens OX=9606 GN=KANSL1 PE=1 SV=2	121.0	9.0	19.9	1	1.5	12.11	307			

308	U520_HUMAN	U5 small nuclear ribonucleoprotein 200 kDa helicase OS=Homo sapiens OX=9606 GN=SNRNP200 PE=1 SV=2	244.4	5.7	19.8	1	0.5	717.45	308			
309	GFAP_HUMAN	Glial fibrillary acidic protein OS=Homo sapiens OX=9606 GN=GFAP PE=1 SV=1	49.8	5.4	19.8	1	2.5	48.31	309			
310	VIR_HUMAN	Protein virilizer homolog OS=Homo sapiens OX=9606 GN=VIRMA PE=1 SV=2	201.9	4.9	19.7	1	0.4	52.40	310			
311	KVD13_HUMAN	Immunoglobulin kappa variable 1D-13 OS=Homo sapiens OX=9606 GN=IGKV1D-13 PE=3 SV=1	12.6	7.7	19.7	1	13.7	44.55	311			
312	IPRI_HUMAN	Inositol 1,4,5-trisphosphate receptor-interacting protein OS=Homo sapiens OX=9606 GN=ITPRIP PE=1 SV=1	62.0	5.6	19.7	1	2.2	58.15	312			
313	MECP2_HUMAN	Methyl-CpG-binding protein 2 OS=Homo sapiens OX=9606 GN=MECP2 PE=1 SV=1	52.4	10.0	19.7	1	2.1	844.05	313			
314	NUMBL_HUMAN	Numb-like protein OS=Homo sapiens OX=9606 GN=NUMBL PE=1 SV=1	64.9	9.1	19.7	1	2.0	3.60	314			
315	SC61G_HUMAN	Protein transport protein Sec61 subunit gamma OS=Homo sapiens OX=9606 GN=SEC61G PE=1 SV=1	7.7	10.0	19.6	1	17.6	635.74	315			
316	RL21_HUMAN	60S ribosomal protein L21 OS=Homo sapiens OX=9606 GN=RPL21 PE=1 SV=2	18.6	10.5	19.6	1	4.4	1225.37	316			
317	P5F1B_HUMAN	Putative POU domain, class 5, transcription factor 1B OS=Homo sapiens OX=9606 GN=POU5F1B PE=5 SV=2	38.6	6.3	19.6	1	2.5	15.49	317			
318	STX8_HUMAN	Syntaxin-8 OS=Homo sapiens OX=9606 GN=STX8 PE=1 SV=2	26.9	4.9	19.6	1	4.2	21.88	318			
319	MTUS1_HUMAN	Microtubule-associated tumor suppressor 1 OS=Homo sapiens OX=9606 GN=MTUS1 PE=1 SV=2	141.3	7.3	19.6	1	0.8	8.45	319	1.50	1	0.00
320	DMD_HUMAN	Dystrophin OS=Homo sapiens OX=9606 GN=DMD PE=1 SV=3	426.5	5.6	19.5	1	0.2	33.90	320			
321	SLN11_HUMAN	Schlafen family member 11 OS=Homo sapiens OX=9606 GN=SLFN11 PE=1 SV=2	102.8	8.0	19.5	1	1.2	651.15	321			
322	PKN2_HUMAN	Serine/threonine-protein kinase N2 OS=Homo sapiens OX=9606 GN=PKN2 PE=1 SV=1	112.0	5.9	19.5	1	0.9	50.96	322			
323	CHSTF_HUMAN	Carbohydrate sulfotransferase 15 OS=Homo sapiens OX=9606 GN=CHST15 PE=1 SV=1	64.9	8.6	19.4	1	1.4	59.66	323			
324	CTIP_HUMAN	DNA endonuclease RBBP8 OS=Homo sapiens OX=9606 GN=RBBP8 PE=1 SV=2	101.9	5.9	19.4	1	1.0	42.00	324			

325	MS18B_HUMAN	Protein Mis18-beta OS=Homo sapiens OX=9606 GN=OIP5 PE=1 SV=2	24.7	7.0	19.4		1	3.1	1222.57	325			
326	I20RB_HUMAN	Interleukin-20 receptor subunit beta OS=Homo sapiens OX=9606 GN=IL20RB PE=1 SV=1	35.1	5.0	19.4		1	2.3	42.82	326			
327	TLRN1_HUMAN	Talin rod domain-containing protein 1 OS=Homo sapiens OX=9606 GN=TLNRD1 PE=1 SV=1	37.7	8.5	19.4		1	2.2	1.94	327			
328	AFG32_HUMAN	AFG3-like protein 2 OS=Homo sapiens OX=9606 GN=AFG3L2 PE=1 SV=2	88.5	8.8	19.4		1	1.0	101.83	328			
329	GRM4_HUMAN	Metabotropic glutamate receptor 4 OS=Homo sapiens OX=9606 GN=GRM4 PE=2 SV=1	101.8	9.1	19.4		1	1.2	20.62	329			
330	FCGBP_HUMAN	IgGFc-binding protein OS=Homo sapiens OX=9606 GN=FCGBP PE=1 SV=3	571.6	5.1	19.4		1	0.1	59.25	330			
331	C19L2_HUMAN	CWF19-like protein 2 OS=Homo sapiens OX=9606 GN=CWF19L2 PE=1 SV=4	103.7	8.8	19.4		1	1.6	0.86	331			
332	PPIH_HUMAN	Peptidyl-prolyl cis-trans isomerase H OS=Homo sapiens OX=9606 GN=PPIH PE=1 SV=1	19.2	8.3	19.3		1	6.2	54.19	332			
333	ACTBL_HUMAN	Beta-actin-like protein 2 OS=Homo sapiens OX=9606 GN=ACTBL2 PE=1 SV=2	42.0	5.4	19.2		1	2.9	763.63	333			
334	RCC1_HUMAN	Regulator of chromosome condensation OS=Homo sapiens OX=9606 GN=RCC1 PE=1 SV=1	44.9	7.2	19.2		1	2.1	3.45	334			
335	HACD2_HUMAN	Very-long-chain (3R)-3-hydroxyacyl-CoA dehydratase 2 OS=Homo sapiens OX=9606 GN=HACD2 PE=1 SV=1	28.4	9.6	19.2		1	4.3	23.75	335			
336	UAP1_HUMAN	UDP-N-acetylhexosamine pyrophosphorylase OS=Homo sapiens OX=9606 GN=UAP1 PE=1 SV=3	58.7	5.9	19.2		1	2.1	15.47	336			
337	AUP1_HUMAN	Ancient ubiquitous protein 1 OS=Homo sapiens OX=9606 GN=AUP1 PE=1 SV=2	45.8	9.0	19.1		1	2.0	45.09	337			
338	ANTR1_HUMAN	Anthrax toxin receptor 1 OS=Homo sapiens OX=9606 GN=ANTXR1 PE=1 SV=2	62.7	7.5	19.1		1	2.0	49.22	338			
339	WDR26_HUMAN	WD repeat-containing protein 26 OS=Homo sapiens OX=9606 GN=WDR26 PE=1 SV=3	72.1	5.7	19.1		1	1.7	3.88	339			
340	ASHWN_HUMAN	Ashwin OS=Homo sapiens OX=9606 GN=C2orf49 PE=1 SV=1	25.8	9.8	19.1		1	3.9	35.20	340			
341	FA83G_HUMAN	Protein FAM83G OS=Homo sapiens OX=9606 GN=FAM83G PE=1 SV=2	90.8	6.0	19.1		1	1.1	40.16	341			

342	NEDD4_HUMAN	E3 ubiquitin-protein ligase NEDD4 OS=Homo sapiens OX=9606 GN=NEDD4 PE=1 SV=4	149.0	6.1	19.1	1	0.8	7.85	342			
343	ESR2_HUMAN	Estrogen receptor beta OS=Homo sapiens OX=9606 GN=ESR2 PE=1 SV=2	59.2	8.8	19.1	1	1.7	1100.41	343			
344	AP5Z1_HUMAN	AP-5 complex subunit zeta-1 OS=Homo sapiens OX=9606 GN=AP5Z1 PE=1 SV=2	88.5	6.6	19.1	1	0.9	1057.38	344			
345	NOE1_HUMAN	Noelin OS=Homo sapiens OX=9606 GN=OLFM1 PE=1 SV=4	55.3	6.5	19.1	1	2.3	8.81	345			
346	MMP2_HUMAN	72 kDa type IV collagenase OS=Homo sapiens OX=9606 GN=MMP2 PE=1 SV=2	73.8	5.3	19.0	1	1.4	998.94	346			
347	ANKZ1_HUMAN	Ankyrin repeat and zinc finger domain-containing protein 1 OS=Homo sapiens OX=9606 GN=ANKZF1 PE=1 SV=1	80.9	8.7	19.0	1	1.4	946.44	347			
348	MROH5_HUMAN	Maestro heat-like repeat family member 5 OS=Homo sapiens OX=9606 GN=MROH5 PE=2 SV=2	149.0	7.6	19.0	1	0.5	74.76	348			
349	FXDC2_HUMAN	Fatty acid hydroxylase domain-containing protein 2 OS=Homo sapiens OX=9606 GN=FAXDC2 PE=2 SV=1	39.0	9.0	19.0	1	3.0	777.63	349			
350	M3K6_HUMAN	Mitogen-activated protein kinase kinase kinase 6 OS=Homo sapiens OX=9606 GN=MAP3K6 PE=1 SV=3	142.5	6.7	19.0	1	0.7	19.14	350			
351	MYO5C_HUMAN	Unconventional myosin-Vc OS=Homo sapiens OX=9606 GN=MYO5C PE=1 SV=2	202.7	7.7	19.0	1	0.5	1058.56	351			
352	PAR15_HUMAN	Protein mono-ADP-ribosyltransferase PARP15 OS=Homo sapiens OX=9606 GN=PARP15 PE=1 SV=2	74.5	9.0	19.0	1	2.1	552.74	352			
353	CP072_HUMAN	UPF0472 protein C16orf72 OS=Homo sapiens OX=9606 GN=C16orf72 PE=1 SV=1	30.9	7.8	18.9	1	2.9	15.77	353			
354	SMC1A_HUMAN	Structural maintenance of chromosomes protein 1A OS=Homo sapiens OX=9606 GN=SMC1A PE=1 SV=2	143.1	7.5	18.9	1	0.8	57.15	354			
355	CRK_HUMAN	Adapter molecule crk OS=Homo sapiens OX=9606 GN=CRK PE=1 SV=2	33.8	5.4	18.9	1	3.9	33.61	355			
356	RIC3_HUMAN	Protein RIC-3 OS=Homo sapiens OX=9606 GN=RIC3 PE=1 SV=1	41.1	5.0	18.9	1	2.2	1150.70	356			
357	SUCB1_HUMAN	Succinate--CoA ligase [ADP-forming] subunit beta, mitochondrial OS=Homo sapiens OX=9606 GN=SUCLA2 PE=1 SV=3	50.3	7.0	18.9	1	1.7	88.17	357			

358	GHDC_HUMAN	GH3 domain-containing protein OS=Homo sapiens OX=9606 GN=GHDC PE=1 SV=2	57.5	8.1	18.9	1	1.5	29.23	358			
359	SOGA3_HUMAN	Protein SOGA3 OS=Homo sapiens OX=9606 GN=SOGA3 PE=3 SV=1	103.1	5.8	18.8	1	1.5	25.67	359			
360	CJ067_HUMAN	Uncharacterized protein C10orf67, mitochondrial OS=Homo sapiens OX=9606 GN=C10orf67 PE=1 SV=3	63.6	8.9	18.8	1	1.6	24.37	360			
361	PAI1_HUMAN	Plasminogen activator inhibitor 1 OS=Homo sapiens OX=9606 GN=SERPINE1 PE=1 SV=1	45.0	6.7	18.7	1	2.5	55.01	361			
362	FRITZ_HUMAN	WD repeat-containing and planar cell polarity effector protein fritz homolog OS=Homo sapiens OX=9606 GN=WDPCP PE=1 SV=2	85.0	6.0	18.7	1	1.1	70.59	362			
363	TSN12_HUMAN	Tetraspanin-12 OS=Homo sapiens OX=9606 GN=TSPAN12 PE=1 SV=1	35.4	5.7	18.7	1	3.6	42.01	363			
364	SUMO1_HUMAN	Small ubiquitin-related modifier 1 OS=Homo sapiens OX=9606 GN=SUMO1 PE=1 SV=1	11.5	5.3	18.7	1	15.8	30.82	364			
365	SGCG_HUMAN	Gamma-sarcoglycan OS=Homo sapiens OX=9606 GN=SGCG PE=1 SV=4	32.4	5.6	18.7	1	3.8	810.34	365			
366	DOCK4_HUMAN	Dedicator of cytokinesis protein 4 OS=Homo sapiens OX=9606 GN=DOCK4 PE=1 SV=3	225.1	7.6	18.7	1	0.6	21.80	366			
367	WAPL_HUMAN	Wings apart-like protein homolog OS=Homo sapiens OX=9606 GN=WAPL PE=1 SV=1	132.9	5.3	18.7	1	0.8	940.22	367			
368	ARIP4_HUMAN	Helicase ARIP4 OS=Homo sapiens OX=9606 GN=RAD54L2 PE=1 SV=4	162.7	5.7	18.7	1	0.6	65.94	368			
369	SLTM_HUMAN	SAFB-like transcription modulator OS=Homo sapiens OX=9606 GN=SLTM PE=1 SV=2	117.1	7.7	18.7	1	1.0	48.14	369			
370	BIG3_HUMAN	Brefeldin A-inhibited guanine nucleotide-exchange protein 3 OS=Homo sapiens OX=9606 GN=ARFGEF3 PE=1 SV=3	240.5	5.5	18.7	1	0.4	26.52	370			
371	PAR14_HUMAN	Protein mono-ADP-ribosyltransferase PARP14 OS=Homo sapiens OX=9606 GN=PARP14 PE=1 SV=3	202.7	6.8	18.6	1	0.7	20.05	371			
372	PAPP1_HUMAN	Pappalysin-1 OS=Homo sapiens OX=9606 GN=PAPPA PE=1 SV=3	180.9	5.8	18.6	1	0.7	888.17	372			
373	MGAP_HUMAN	MAX gene-associated protein OS=Homo sapiens OX=9606 GN=MGA PE=1 SV=4	336.0	6.5	18.6	1	0.3	993.30	373			
374	DCD_HUMAN	Dermcidin OS=Homo sapiens OX=9606 GN=DCD PE=1 SV=2	11.3	6.1	18.6	1	7.3	31.42	374			

375	CQ082_HUMAN	Putative uncharacterized protein C17orf82 OS=Homo sapiens OX=9606 GN=C17orf82 PE=1 SV=2	25.4	10.4	18.6	1	3.6	8.86	375			
376	ULA1_HUMAN	NEDD8-activating enzyme E1 regulatory subunit OS=Homo sapiens OX=9606 GN=NAE1 PE=1 SV=1	60.2	5.2	18.5	1	1.5	1048.04	376			
377	CYLC1_HUMAN	Cylicin-1 OS=Homo sapiens OX=9606 GN=CYLC1 PE=2 SV=2	74.2	9.7	18.5	1	1.1	1232.42	377			
378	HACE1_HUMAN	E3 ubiquitin-protein ligase HACE1 OS=Homo sapiens OX=9606 GN=HACE1 PE=1 SV=2	102.3	5.6	18.5	1	1.1	20.29	378			
379	ICE2_HUMAN	Little elongation complex subunit 2 OS=Homo sapiens OX=9606 GN=ICE2 PE=1 SV=2	109.9	6.7	18.5	1	1.0	6.62	379			
380	FRPD3_HUMAN	FERM and PDZ domain-containing protein 3 OS=Homo sapiens OX=9606 GN=FRMPD3 PE=2 SV=2	199.1	8.4	18.4	1	0.6	21.50	380			
381	RUFY2_HUMAN	RUN and FYVE domain-containing protein 2 OS=Homo sapiens OX=9606 GN=RUFY2 PE=1 SV=3	70.0	5.6	18.4	1	1.7	42.74	381			
382	LIPS_HUMAN	Hormone-sensitive lipase OS=Homo sapiens OX=9606 GN=LIPE PE=1 SV=4	116.5	6.2	18.4	1	1.5	542.36	382			
383	KIF7_HUMAN	Kinesin-like protein KIF7 OS=Homo sapiens OX=9606 GN=KIF7 PE=1 SV=2	150.5	6.4	18.3	1	0.5	43.60	383			
384	SSX9_HUMAN	Putative protein SSX9 OS=Homo sapiens OX=9606 GN=SSX9P PE=5 SV=1	21.5	9.2	18.3	1	5.9	812.62	384			
385	GPTC4_HUMAN	G patch domain-containing protein 4 OS=Homo sapiens OX=9606 GN=GPATCH4 PE=1 SV=2	50.4	9.6	18.3	1	2.5	51.58	385			
386	TM198_HUMAN	Transmembrane protein 198 OS=Homo sapiens OX=9606 GN=TMEM198 PE=1 SV=1	39.4	10.0	18.3	1	2.5	41.15	386			
387	ZNT6_HUMAN	Zinc transporter 6 OS=Homo sapiens OX=9606 GN=SLC30A6 PE=1 SV=2	51.1	9.3	18.2	1	2.4	793.50	387			
388	CBPQ_HUMAN	Carboxypeptidase Q OS=Homo sapiens OX=9606 GN=CPQ PE=1 SV=1	51.9	5.8	18.2	1	1.9	80.95	388			
389	ZN623_HUMAN	Zinc finger protein 623 OS=Homo sapiens OX=9606 GN=ZNF623 PE=1 SV=2	61.4	8.5	18.2	1	2.6	5.10	389			
390	TF3C5_HUMAN	General transcription factor 3C polypeptide 5 OS=Homo sapiens OX=9606 GN=GTF3C5 PE=1 SV=2	59.5	6.5	18.2	1	2.7	35.70	390			
391	UCP3_HUMAN	Mitochondrial uncoupling protein 3 OS=Homo sapiens OX=9606 GN=UCP3 PE=1 SV=1	34.2	9.3	18.2	1	2.6	1059.68	391			

392	SPT13_HUMAN	Spermatogenesis-associated protein 13 OS=Homo sapiens OX=9606 GN=SPATA13 PE=1 SV=1	74.8	7.0	18.2	1	1.4	926.49	392			
393	LIPN_HUMAN	Lipase member N OS=Homo sapiens OX=9606 GN=LIPN PE=2 SV=2	45.5	6.4	18.2	1	4.5	31.09	393			
394	OTU7A_HUMAN	OTU domain-containing protein 7A OS=Homo sapiens OX=9606 GN=OTUD7A PE=1 SV=1	100.6	8.7	18.2	1	0.8	41.03	394			
395	ARH40_HUMAN	Rho guanine nucleotide exchange factor 40 OS=Homo sapiens OX=9606 GN=ARHGEF40 PE=1 SV=3	164.6	5.8	18.2	1	0.5	30.47	395			
396	F161B_HUMAN	Protein FAM161B OS=Homo sapiens OX=9606 GN=FAM161B PE=1 SV=2	73.6	9.4	18.2	1	1.2	20.80	396			
397	ARHG8_HUMAN	Neuroepithelial cell-transforming gene 1 protein OS=Homo sapiens OX=9606 GN=NET1 PE=1 SV=1	67.7	9.3	18.1	1	2.3	613.12	397			
398	CLD10_HUMAN	Claudin-10 OS=Homo sapiens OX=9606 GN=CLDN10 PE=1 SV=2	24.5	8.3	18.1	1	4.4	12.28	398			
399	RFC1_HUMAN	Replication factor C subunit 1 OS=Homo sapiens OX=9606 GN=RFC1 PE=1 SV=4	128.2	9.4	18.1	1	0.7	56.20	399			
400	RMC1_HUMAN	Regulator of MON1-CCZ1 complex OS=Homo sapiens OX=9606 GN=RMC1 PE=1 SV=2	74.9	7.9	18.0	1	1.2	12.03	400			
401	ENOL_HUMAN	Putative protein T-ENOL OS=Homo sapiens OX=9606 GN=CDIPTOSP PE=4 SV=1	9.0	8.5	18.0	1	14.5	38.79	401			
402	CHSTB_HUMAN	Carbohydrate sulfotransferase 11 OS=Homo sapiens OX=9606 GN=CHST11 PE=1 SV=1	41.5	9.0	18.0	1	2.3	79.83	402			
403	TTF2_HUMAN	Transcription termination factor 2 OS=Homo sapiens OX=9606 GN=TTF2 PE=1 SV=2	129.5	8.6	18.0	1	0.8	41.78	403			
404	F184A_HUMAN	Protein FAM184A OS=Homo sapiens OX=9606 GN=FAM184A PE=2 SV=3	132.9	5.6	18.0	1	1.0	15.20	404			
405	BORC8_HUMAN	BLOC-1-related complex subunit 8 OS=Homo sapiens OX=9606 GN=BORCS8 PE=1 SV=1	13.4	5.5	18.0	1	9.2	798.70	405			
406	TR49D_HUMAN	Tripartite motif-containing protein 49D OS=Homo sapiens OX=9606 GN=TRIM49D1 PE=2 SV=1	52.5	8.7	18.0	1	1.5	55.69	406			
407	UBE3C_HUMAN	Ubiquitin-protein ligase E3C OS=Homo sapiens OX=9606 GN=UBE3C PE=1 SV=3	123.8	6.3	18.0	1	1.3	630.64	407			
408	CK2N1_HUMAN	Calcium/calmodulin-dependent protein kinase II inhibitor 1 OS=Homo sapiens OX=9606 GN=CAMK2N1 PE=1 SV=1	8.5	5.2	18.0	1	14.1	7.42	408			

409	ES8L2_HUMAN	Epidermal growth factor receptor kinase substrate 8-like protein 2 OS=Homo sapiens OX=9606 GN=EPS8L2 PE=1 SV=2	80.6	6.4	18.0	1	1.4	17.75	409			
410	BRWD1_HUMAN	Bromodomain and WD repeat-containing protein 1 OS=Homo sapiens OX=9606 GN=BRWD1 PE=1 SV=4	262.8	8.7	17.9	1	0.4	46.86	410			
411	STAT6_HUMAN	Signal transducer and activator of transcription 6 OS=Homo sapiens OX=9606 GN=STAT6 PE=1 SV=1	94.1	5.8	17.9	1	0.9	1041.43	411			
412	ZN416_HUMAN	Zinc finger protein 416 OS=Homo sapiens OX=9606 GN=ZNF416 PE=2 SV=1	67.1	8.3	17.9	1	1.7	63.70	412			
413	PPR36_HUMAN	Protein phosphatase 1 regulatory subunit 36 OS=Homo sapiens OX=9606 GN=PPP1R36 PE=1 SV=1	49.4	9.4	17.8	1	1.7	1007.03	413			
414	KS6A6_HUMAN	Ribosomal protein S6 kinase alpha-6 OS=Homo sapiens OX=9606 GN=RPS6KA6 PE=1 SV=1	83.8	5.9	17.8	1	1.2	27.74	414			
415	RS6_HUMAN	40S ribosomal protein S6 OS=Homo sapiens OX=9606 GN=RPS6 PE=1 SV=1	28.7	10.9	17.8	1	5.2	693.21	415			
416	CP2CI_HUMAN	Cytochrome P450 2C18 OS=Homo sapiens OX=9606 GN=CYP2C18 PE=1 SV=3	55.7	6.8	17.8	1	1.8	1010.83	416			
417	ENY2_HUMAN	Transcription and mRNA export factor ENY2 OS=Homo sapiens OX=9606 GN=ENY2 PE=1 SV=1	11.5	9.4	17.8	1	7.9	965.79	417			
418	LAMA2_HUMAN	Laminin subunit alpha-2 OS=Homo sapiens OX=9606 GN=LAMA2 PE=1 SV=4	343.7	6.0	17.7	1	0.5	8.10	418			
419	MYOZ3_HUMAN	Myozenin-3 OS=Homo sapiens OX=9606 GN=MYOZ3 PE=1 SV=2	27.1	9.4	17.7	1	5.6	16.38	419			
420	MON2_HUMAN	Protein MON2 homolog OS=Homo sapiens OX=9606 GN=MON2 PE=1 SV=3	190.2	5.7	17.7	1	0.6	691.36	420			
421	FZD2_HUMAN	Frizzled-2 OS=Homo sapiens OX=9606 GN=FZD2 PE=1 SV=1	63.5	8.5	17.7	1	1.9	752.32	421			
422	DC8L1_HUMAN	DDB1- and CUL4-associated factor 8-like protein 1 OS=Homo sapiens OX=9606 GN=DCAF8L1 PE=2 SV=1	67.3	4.7	17.7	1	1.5	19.86	422			
423	RSF1_HUMAN	Remodeling and spacing factor 1 OS=Homo sapiens OX=9606 GN=RSF1 PE=1 SV=2	163.7	4.9	17.7	1	0.7	788.29	423			
424	FLNC_HUMAN	Filamin-C OS=Homo sapiens OX=9606 GN=FLNC PE=1 SV=3	290.8	5.6	17.7	1	0.4	785.97	424			
425	SDCB2_HUMAN	Syntenin-2 OS=Homo sapiens OX=9606 GN=SDCBP2 PE=1 SV=2	31.6	9.2	17.6	1	3.8	24.82	425			

426	OTC_HUMAN	Ornithine carbamoyltransferase, mitochondrial OS=Homo sapiens OX=9606 GN=OTC PE=1 SV=3	39.9	8.7	17.6	1	2.5	16.96	426			
427	COE2_HUMAN	Transcription factor COE2 OS=Homo sapiens OX=9606 GN=EBF2 PE=2 SV=4	62.6	9.2	17.6	1	3.0	24.99	427			
428	ARF5_HUMAN	ADP-ribosylation factor 5 OS=Homo sapiens OX=9606 GN=ARF5 PE=1 SV=2	20.5	6.3	17.6	1	8.3	46.75	428			
429	HHIP_HUMAN	Hedgehog-interacting protein OS=Homo sapiens OX=9606 GN=HHIP PE=1 SV=3	78.8	8.2	17.6	1	2.0	6.71	429	0.43	1	0.00
430	EEA1_HUMAN	Early endosome antigen 1 OS=Homo sapiens OX=9606 GN=EEA1 PE=1 SV=2	162.4	5.5	17.6	1	0.6	935.06	430			
431	EIF3G_HUMAN	Eukaryotic translation initiation factor 3 subunit G OS=Homo sapiens OX=9606 GN=EIF3G PE=1 SV=2	35.6	5.9	17.6	1	2.5	11.94	431			
432	TXND2_HUMAN	Thioredoxin domain-containing protein 2 OS=Homo sapiens OX=9606 GN=TXNDC2 PE=2 SV=4	60.4	4.8	17.6	1	2.4	21.36	432			
433	PUS10_HUMAN	Putative tRNA pseudouridine synthase Pus10 OS=Homo sapiens OX=9606 GN=PUS10 PE=1 SV=1	60.2	6.1	17.6	1	2.6	575.73	433			
434	DDX6L_HUMAN	Probable ATP-dependent RNA helicase DDX60-like OS=Homo sapiens OX=9606 GN=DDX60L PE=2 SV=2	197.5	8.6	17.6	1	0.6	707.81	434			
435	ANR39_HUMAN	Ankyrin repeat domain-containing protein 39 OS=Homo sapiens OX=9606 GN=ANKRD39 PE=1 SV=1	19.6	6.4	17.5	1	7.1	8.63	435			
436	RL3_HUMAN	60S ribosomal protein L3 OS=Homo sapiens OX=9606 GN=RPL3 PE=1 SV=2	46.1	10.2	17.5	1	2.7	840.43	436			
437	MYBPP_HUMAN	MYCBP-associated protein OS=Homo sapiens OX=9606 GN=MYCBPAP PE=1 SV=2	108.1	7.1	17.5	1	1.1	908.12	437			
438	SPT22_HUMAN	Spermatogenesis-associated protein 22 OS=Homo sapiens OX=9606 GN=SPATA22 PE=1 SV=2	41.3	9.4	17.5	1	1.9	1294.82	438			
439	HXA10_HUMAN	Homeobox protein Hox-A10 OS=Homo sapiens OX=9606 GN=HOXA10 PE=1 SV=3	42.4	8.6	17.5	1	2.2	79.55	439			
440	OSBL1_HUMAN	Oxysterol-binding protein-related protein 1 OS=Homo sapiens OX=9606 GN=OSBPL1A PE=1 SV=2	108.4	6.0	17.5	1	1.1	26.54	440			
441	FGF14_HUMAN	Fibroblast growth factor 14 OS=Homo sapiens OX=9606 GN=FGF14 PE=1 SV=1	27.7	10.1	17.4	1	5.7	17.44	441			
442	PTPRE_HUMAN	Receptor-type tyrosine-protein phosphatase epsilon OS=Homo sapiens OX=9606 GN=PTPRE PE=1 SV=1	80.6	6.6	17.4	1	1.1	1163.34	442			

443	AINX_HUMAN	Alpha-internexin OS=Homo sapiens OX=9606 GN=INA PE=1 SV=2	55.4	5.3	17.4	1	1.8	976.64	443			
444	NAT8_HUMAN	N-acetyltransferase 8 OS=Homo sapiens OX=9606 GN=NAT8 PE=1 SV=2	25.6	9.1	17.4	1	4.0	31.41	444			
445	NUDC3_HUMAN	NudC domain-containing protein 3 OS=Homo sapiens OX=9606 GN=NUDCD3 PE=1 SV=3	40.8	5.2	17.4	1	4.4	536.57	445			
446	GP1BA_HUMAN	Platelet glycoprotein Ib alpha chain OS=Homo sapiens OX=9606 GN=GP1BA PE=1 SV=2	71.5	5.9	17.4	1	1.2	22.08	446			
447	ERVV2_HUMAN	Endogenous retrovirus group V member 2 Env polyprotein OS=Homo sapiens OX=9606 GN=ERVV-2 PE=2 SV=1	59.3	8.9	17.4	1	1.3	26.96	447			
448	C4BPA_HUMAN	C4b-binding protein alpha chain OS=Homo sapiens OX=9606 GN=C4BPA PE=1 SV=2	67.0	7.2	17.4	1	2.3	43.35	448			
449	MEIOB_HUMAN	Meiosis-specific with OB domain-containing protein OS=Homo sapiens OX=9606 GN=MEIOB PE=2 SV=3	49.3	6.0	17.3	1	1.6	1275.64	449			
450	KAAG1_HUMAN	Kidney-associated antigen 1 OS=Homo sapiens OX=9606 GN=KAAG1 PE=2 SV=1	9.0	9.5	17.3	1	16.7	44.83	450			
451	S22A3_HUMAN	Solute carrier family 22 member 3 OS=Homo sapiens OX=9606 GN=SLC22A3 PE=1 SV=1	61.2	8.9	17.3	1	1.4	45.48	451			
452	ZN730_HUMAN	Putative zinc finger protein 730 OS=Homo sapiens OX=9606 GN=ZNF730 PE=2 SV=1	59.0	9.5	17.3	1	2.2	38.40	452			
453	SVIL_HUMAN	Supervillin OS=Homo sapiens OX=9606 GN=SVIL PE=1 SV=2	247.6	6.5	17.3	1	0.9	2.10	453			
454	KCNJ1_HUMAN	ATP-sensitive inward rectifier potassium channel 1 OS=Homo sapiens OX=9606 GN=KCNJ1 PE=1 SV=1	44.8	9.0	17.3	1	2.0	39.17	454			
455	M3K11_HUMAN	Mitogen-activated protein kinase kinase kinase 11 OS=Homo sapiens OX=9606 GN=MAP3K11 PE=1 SV=1	92.6	8.4	17.3	1	1.8	598.19	455			
456	OR3A3_HUMAN	Olfactory receptor 3A3 OS=Homo sapiens OX=9606 GN=OR3A3 PE=2 SV=3	34.9	7.0	17.2	1	4.7	9.76	456			
457	CNBD2_HUMAN	Cyclic nucleotide-binding domain-containing protein 2 OS=Homo sapiens OX=9606 GN=CNBD2 PE=2 SV=2	67.5	9.3	17.2	1	2.4	594.00	457			
458	JADE2_HUMAN	E3 ubiquitin-protein ligase Jade-2 OS=Homo sapiens OX=9606 GN=JADE2 PE=1 SV=2	87.4	5.1	17.2	1	1.3	67.88	458			

459	SKOR2_HUMAN	SKI family transcriptional corepressor 2 OS=Homo sapiens OX=9606 GN=SKOR2 PE=1 SV=2	104.2	6.0	17.2		1	1.0	866.32	459			
460	MYL6_HUMAN	Myosin light polypeptide 6 OS=Homo sapiens OX=9606 GN=MYL6 PE=1 SV=2	16.9	4.6	17.2		1	10.6	590.19	460			
461	TNFL8_HUMAN	Tumor necrosis factor ligand superfamily member 8 OS=Homo sapiens OX=9606 GN=TNFSF8 PE=2 SV=1	26.0	7.6	17.2		1	6.0	677.63	461			
462	PALB2_HUMAN	Partner and localizer of BRCA2 OS=Homo sapiens OX=9606 GN=PALB2 PE=1 SV=1	131.2	6.0	17.2		1	0.8	1007.91	462			
463	HS74L_HUMAN	Heat shock 70 kDa protein 4L OS=Homo sapiens OX=9606 GN=HSPA4L PE=1 SV=3	94.5	5.6	17.1		1	1.2	839.21	463			
464	HIPK2_HUMAN	Homeodomain-interacting protein kinase 2 OS=Homo sapiens OX=9606 GN=HIPK2 PE=1 SV=2	130.9	8.7	17.1		1	0.9	27.86	464			
465	R3GEF_HUMAN	Guanine nucleotide exchange factor for Rab-3A OS=Homo sapiens OX=9606 GN=RAB3IL1 PE=1 SV=1	42.6	6.1	17.1		1	2.4	57.86	465			
466	RNF26_HUMAN	E3 ubiquitin-protein ligase RNF26 OS=Homo sapiens OX=9606 GN=RNF26 PE=1 SV=1	47.7	8.9	17.1		1	1.8	64.80	466			
467	ABCC8_HUMAN	ATP-binding cassette sub-family C member 8 OS=Homo sapiens OX=9606 GN=ABCC8 PE=1 SV=6	176.9	8.0	17.1		1	0.5	85.76	467			
468	TM50A_HUMAN	Transmembrane protein 50A OS=Homo sapiens OX=9606 GN=TMEM50A PE=1 SV=1	17.4	5.6	17.1		1	6.4	50.46	468			
469	CCD40_HUMAN	Coiled-coil domain-containing protein 40 OS=Homo sapiens OX=9606 GN=CCDC40 PE=2 SV=2	130.0	5.2	17.1		1	1.0	54.77	469			
470	IGSF3_HUMAN	Immunoglobulin superfamily member 3 OS=Homo sapiens OX=9606 GN=IGSF3 PE=2 SV=3	135.1	5.7	17.1		1	0.8	751.35	470			
471	CBPC1_HUMAN	Cytosolic carboxypeptidase 1 OS=Homo sapiens OX=9606 GN=AGTPBP1 PE=1 SV=3	138.4	5.8	17.1		1	1.3	26.55	471			
472	SH3R3_HUMAN	E3 ubiquitin-protein ligase SH3RF3 OS=Homo sapiens OX=9606 GN=SH3RF3 PE=1 SV=2	92.7	9.1	17.0		1	1.0	10.22	472	3.17	1	0.00
473	ZGRF1_HUMAN	Protein ZGRF1 OS=Homo sapiens OX=9606 GN=ZGRF1 PE=1 SV=3	236.5	5.8	17.0		1	0.4	54.14	473			
474	CDK18_HUMAN	Cyclin-dependent kinase 18 OS=Homo sapiens OX=9606 GN=CDK18 PE=1 SV=4	54.4	8.8	17.0		1	1.7	77.96	474			

475	SRSF1_HUMAN	Serine/arginine-rich splicing factor 1 OS=Homo sapiens OX=9606 GN=SRSF1 PE=1 SV=2	27.7	10.4	17.0		1	3.6	831.90	475			
476	MYH4_HUMAN	Myosin-4 OS=Homo sapiens OX=9606 GN=MYH4 PE=2 SV=2	222.9	5.7	17.0		1	0.6	54.99	476			
477	PPIP2_HUMAN	Proline-serine-threonine phosphatase-interacting protein 2 OS=Homo sapiens OX=9606 GN=PSTPIP2 PE=1 SV=4	38.8	8.7	17.0		1	5.1	7.69	477			
478	NEC1_HUMAN	Neuroendocrine convertase 1 OS=Homo sapiens OX=9606 GN=PCSK1 PE=1 SV=2	84.1	5.7	17.0		1	1.2	51.94	478			
479	DPOLN_HUMAN	DNA polymerase nu OS=Homo sapiens OX=9606 GN=POLN PE=1 SV=2	100.2	8.6	17.0		1	2.8	3.28	479			
480	CANT1_HUMAN	Soluble calcium-activated nucleotidase 1 OS=Homo sapiens OX=9606 GN=CANT1 PE=1 SV=1	44.8	5.7	16.9		1	1.7	25.38	480			
481	ARGAL_HUMAN	Rho guanine nucleotide exchange factor 10-like protein OS=Homo sapiens OX=9606 GN=ARHGEF10L PE=1 SV=4	140.3	5.6	16.9		1	0.7	848.99	481			
482	UBQL1_HUMAN	Ubiquilin-1 OS=Homo sapiens OX=9606 GN=UBQLN1 PE=1 SV=2	62.5	5.0	16.9		1	2.5	42.67	482			
483	PKHG5_HUMAN	Pleckstrin homology domain-containing family G member 5 OS=Homo sapiens OX=9606 GN=PLEKHG5 PE=1 SV=3	117.4	5.9	16.9		1	1.1	2.51	483			
484	T200B_HUMAN	Transmembrane protein 200B OS=Homo sapiens OX=9606 GN=TMEM200B PE=2 SV=1	32.7	11.4	16.9		1	3.3	51.92	484			
485	COE4_HUMAN	Transcription factor COE4 OS=Homo sapiens OX=9606 GN=EBF4 PE=2 SV=2	64.4	8.9	16.9		1	1.3	1055.31	485			
486	DOC11_HUMAN	Dedicator of cytokinesis protein 11 OS=Homo sapiens OX=9606 GN=DOCK11 PE=1 SV=2	237.5	7.9	16.9		1	0.4	29.99	486			
487	FA47E_HUMAN	Protein FAM47E OS=Homo sapiens OX=9606 GN=FAM47E PE=2 SV=3	45.6	9.5	16.9		1	2.0	1159.10	487			
488	PCDGH_HUMAN	Protocadherin gamma-B5 OS=Homo sapiens OX=9606 GN=PCDHGB5 PE=2 SV=1	99.8	4.9	16.9		1	0.9	10.75	488			
489	LRN4L_HUMAN	LRRN4 C-terminal-like protein OS=Homo sapiens OX=9606 GN=LRRN4CL PE=2 SV=1	25.2	6.1	16.9		1	4.2	72.27	489			
490	F102A_HUMAN	Protein FAM102A OS=Homo sapiens OX=9606 GN=FAM102A PE=1 SV=2	41.8	8.8	16.8		1	2.3	12.67	490			
491	OFUT2_HUMAN	GDP-fucose protein O-fucosyltransferase 2 OS=Homo sapiens OX=9606 GN=POFUT2 PE=1 SV=3	49.9	6.1	16.8		1	2.8	26.85	491			

492	SYNP2_HUMAN	Synaptopodin-2 OS=Homo sapiens OX=9606 GN=SYNPO2 PE=1 SV=2	117.4	8.8	16.8	1	1.6	24.20	492			
493	PLXA1_HUMAN	Plexin-A1 OS=Homo sapiens OX=9606 GN=PLXNA1 PE=1 SV=3	210.9	6.5	16.8	1	0.8	40.71	493			
494	PRDX2_HUMAN	Peroxiredoxin-2 OS=Homo sapiens OX=9606 GN=PRDX2 PE=1 SV=5	21.9	5.7	16.8	1	5.6	47.35	494			
495	TICN2_HUMAN	Testican-2 OS=Homo sapiens OX=9606 GN=SPOCK2 PE=1 SV=1	46.7	4.7	16.8	1	4.0	628.35	495			
496	CG026_HUMAN	Uncharacterized protein C7orf26 OS=Homo sapiens OX=9606 GN=C7orf26 PE=2 SV=1	50.0	7.6	16.8	1	2.4	804.82	496			
497	CEBOS_HUMAN	Protein CEBPZOS OS=Homo sapiens OX=9606 GN=CEBPZOS PE=1 SV=2	9.3	9.7	16.7	1	15.0	21.06	497			
498	MED16_HUMAN	Mediator of RNA polymerase II transcription subunit 16 OS=Homo sapiens OX=9606 GN=MED16 PE=1 SV=2	96.7	7.1	16.7	1	1.4	29.92	498			
499	PYRD1_HUMAN	Pyridine nucleotide-disulfide oxidoreductase domain-containing protein 1 OS=Homo sapiens OX=9606 GN=PYROXD1 PE=1 SV=1	55.8	5.6	16.7	1	1.4	25.82	499			
500	SARG_HUMAN	Specifically androgen-regulated gene protein OS=Homo sapiens OX=9606 GN=SARG PE=1 SV=2	63.9	8.8	16.7	1	1.3	54.14	500			
501	CD36_HUMAN	Platelet glycoprotein 4 OS=Homo sapiens OX=9606 GN=CD36 PE=1 SV=2	53.0	8.2	16.7	1	1.7	80.14	501			
502	GALE_HUMAN	UDP-glucose 4-epimerase OS=Homo sapiens OX=9606 GN=GALE PE=1 SV=2	38.3	6.3	16.7	1	2.3	35.26	502			
503	MUS81_HUMAN	Crossover junction endonuclease MUS81 OS=Homo sapiens OX=9606 GN=MUS81 PE=1 SV=3	61.1	9.8	16.7	1	1.5	5.10	503			
504	MMP16_HUMAN	Matrix metalloproteinase-16 OS=Homo sapiens OX=9606 GN=MMP16 PE=1 SV=2	69.5	8.7	16.6	1	1.5	48.01	504			
505	TGO1_HUMAN	Transport and Golgi organization protein 1 homolog OS=Homo sapiens OX=9606 GN=MIA3 PE=1 SV=1	213.6	4.8	16.6	1	0.4	1043.14	505			
506	CARD8_HUMAN	Caspase recruitment domain-containing protein 8 OS=Homo sapiens OX=9606 GN=CARD8 PE=1 SV=1	48.9	5.1	16.6	1	2.1	31.02	506			
507	FHOD3_HUMAN	FH1/FH2 domain-containing protein 3 OS=Homo sapiens OX=9606 GN=FHOD3 PE=1 SV=2	158.5	5.7	16.6	1	0.8	44.68	507			
508	MRCKA_HUMAN	Serine/threonine-protein kinase MRCK alpha OS=Homo sapiens OX=9606 GN=CDC42BPA PE=1 SV=1	197.2	6.2	16.6	1	0.6	3.21	508			

509	PARI_HUMAN	PCNA-interacting partner OS=Homo sapiens OX=9606 GN=PARPBP PE=1 SV=3	65.0	9.0	16.6	1	1.9	52.48	509			
510	SNX13_HUMAN	Sorting nexin-13 OS=Homo sapiens OX=9606 GN=SNX13 PE=1 SV=4	112.1	6.2	16.6	1	1.1	814.56	510			
511	LYSM2_HUMAN	LysM and putative peptidoglycan-binding domain-containing protein 2 OS=Homo sapiens OX=9606 GN=LYSMD2 PE=1 SV=1	23.4	5.3	16.5	1	7.4	571.18	511			
512	TTC29_HUMAN	Tetratricopeptide repeat protein 29 OS=Homo sapiens OX=9606 GN=TTC29 PE=2 SV=2	55.0	5.5	16.5	1	1.9	827.76	512			
513	MCM2_HUMAN	DNA replication licensing factor MCM2 OS=Homo sapiens OX=9606 GN=MCM2 PE=1 SV=4	101.8	5.3	16.5	1	1.2	57.35	513			
514	IQEC3_HUMAN	IQ motif and SEC7 domain-containing protein 3 OS=Homo sapiens OX=9606 GN=IQSEC3 PE=2 SV=3	127.5	6.1	16.5	1	1.0	57.17	514			
515	GMDS_HUMAN	GDP-mannose 4,6 dehydratase OS=Homo sapiens OX=9606 GN=GMDS PE=1 SV=1	41.9	6.9	16.5	1	4.3	586.73	515			
516	K1C14_HUMAN	Keratin, type I cytoskeletal 14 OS=Homo sapiens OX=9606 GN=KRT14 PE=1 SV=4	51.5	5.1	16.5	1	2.3	768.61	516			
517	RETST_HUMAN	All-trans-retinol 13,14-reductase OS=Homo sapiens OX=9606 GN=RETSAT PE=1 SV=2	66.8	8.5	16.5	1	2.0	51.03	517			
518	K1C12_HUMAN	Keratin, type I cytoskeletal 12 OS=Homo sapiens OX=9606 GN=KRT12 PE=1 SV=1	53.5	4.7	16.4	1	2.0	70.81	518			
519	SCLT1_HUMAN	Sodium channel and clathrin linker 1 OS=Homo sapiens OX=9606 GN=SCLT1 PE=1 SV=2	80.9	5.8	16.4	1	1.2	43.47	519			
520	KS6C1_HUMAN	Ribosomal protein S6 kinase delta-1 OS=Homo sapiens OX=9606 GN=RPS6KC1 PE=1 SV=2	118.6	4.8	16.4	1	0.8	45.22	520			
521	Z705D_HUMAN	Zinc finger protein 705D OS=Homo sapiens OX=9606 GN=ZNF705D PE=2 SV=1	34.7	9.1	16.4	1	6.0	462.90	521			
522	STRN4_HUMAN	Striatin-4 OS=Homo sapiens OX=9606 GN=STRN4 PE=1 SV=2	80.5	5.2	16.4	1	1.9	19.58	522			
523	ARMC2_HUMAN	Armadillo repeat-containing protein 2 OS=Homo sapiens OX=9606 GN=ARMC2 PE=1 SV=4	96.8	8.5	16.4	1	1.7	21.17	523			
524	WWTR1_HUMAN	WW domain-containing transcription regulator protein 1 OS=Homo sapiens OX=9606 GN=WWTR1 PE=1 SV=1	44.1	5.5	16.4	1	1.8	982.85	524			

525	ABCB6_HUMAN	ATP-binding cassette sub-family B member 6, mitochondrial OS=Homo sapiens OX=9606 GN=ABCB6 PE=1 SV=1	93.8	8.7	16.4	1	0.8	61.35	525			
526	MSH2_HUMAN	DNA mismatch repair protein Msh2 OS=Homo sapiens OX=9606 GN=MSH2 PE=1 SV=1	104.7	5.6	16.4	1	1.5	648.97	526			
527	CDN1B_HUMAN	Cyclin-dependent kinase inhibitor 1B OS=Homo sapiens OX=9606 GN=CDKN1B PE=1 SV=1	22.1	6.5	16.4	1	7.1	21.07	527			
528	HD_HUMAN	Huntingtin OS=Homo sapiens OX=9606 GN=HTT PE=1 SV=2	347.4	5.8	16.4	1	0.3	88.68	528			
529	MOB3C_HUMAN	MOB kinase activator 3C OS=Homo sapiens OX=9606 GN=MOB3C PE=1 SV=1	25.6	9.0	16.4	1	5.1	8.64	529			
530	CRFR2_HUMAN	Corticotropin-releasing factor receptor 2 OS=Homo sapiens OX=9606 GN=CRHR2 PE=1 SV=2	47.7	7.9	16.4	1	1.7	1221.23	530			
531	ZNT1_HUMAN	Zinc transporter 1 OS=Homo sapiens OX=9606 GN=SLC30A1 PE=1 SV=3	55.3	6.0	16.4	1	2.2	19.64	531			
532	CLIC6_HUMAN	Chloride intracellular channel protein 6 OS=Homo sapiens OX=9606 GN=CLIC6 PE=2 SV=3	73.0	4.3	16.4	1	1.1	22.60	532			
533	RET3_HUMAN	Retinol-binding protein 3 OS=Homo sapiens OX=9606 GN=RBP3 PE=1 SV=2	135.3	5.0	16.3	1	0.6	57.02	533			
534	ELOA1_HUMAN	Elongin-A OS=Homo sapiens OX=9606 GN=ELOA PE=1 SV=2	89.9	9.6	16.3	1	1.3	857.78	534			
535	IFT1B_HUMAN	Interferon-induced protein with tetratricopeptide repeats 1B OS=Homo sapiens OX=9606 GN=IFIT1B PE=1 SV=1	55.0	7.6	16.3	1	3.2	563.71	535			
536	XRN2_HUMAN	5'-3' exoribonuclease 2 OS=Homo sapiens OX=9606 GN=XRN2 PE=1 SV=1	108.5	7.3	16.3	1	0.8	53.67	536			
537	DYR_HUMAN	Dihydrofolate reductase OS=Homo sapiens OX=9606 GN=DHFR PE=1 SV=2	21.4	6.8	16.3	1	5.3	933.56	537			
538	TUT4_HUMAN	Terminal uridylyltransferase 4 OS=Homo sapiens OX=9606 GN=TUT4 PE=1 SV=3	185.0	8.3	16.3	1	0.5	1027.84	538			
539	TR61B_HUMAN	tRNA (adenine(58)-N(1))-methyltransferase, mitochondrial OS=Homo sapiens OX=9606 GN=TRMT61B PE=1 SV=2	52.9	6.2	16.3	1	3.1	492.81	539			
540	INHBA_HUMAN	Inhibin beta A chain OS=Homo sapiens OX=9606 GN=INHBA PE=1 SV=2	47.4	8.3	16.3	1	3.3	606.74	540			
541	RGPS1_HUMAN	Ras-specific guanine nucleotide-releasing factor RalGPS1 OS=Homo sapiens OX=9606 GN=RALGPS1 PE=1 SV=1	62.1	9.2	16.2	1	2.2	7.23	541			

542	UH1BL_HUMAN	UHRF1-binding protein 1-like OS=Homo sapiens OX=9606 GN=UHRF1BP1L PE=1 SV=2	164.1	5.9	16.2	1	0.8	23.16	542			
543	LMO7_HUMAN	LIM domain only protein 7 OS=Homo sapiens OX=9606 GN=LMO7 PE=1 SV=3	192.6	8.3	16.2	1	0.5	43.12	543			
544	ZMYM4_HUMAN	Zinc finger MYM-type protein 4 OS=Homo sapiens OX=9606 GN=ZMYM4 PE=1 SV=1	172.7	6.5	16.2	1	0.8	20.28	544			
545	MGDP1_HUMAN	Magnesium-dependent phosphatase 1 OS=Homo sapiens OX=9606 GN=MDP1 PE=1 SV=1	20.1	6.0	16.2	1	5.7	45.82	545			
546	MTCL1_HUMAN	Microtubule cross-linking factor 1 OS=Homo sapiens OX=9606 GN=MTCL1 PE=1 SV=5	209.4	6.0	16.2	1	0.5	882.44	546			
547	ASM3B_HUMAN	Acid sphingomyelinase-like phosphodiesterase 3b OS=Homo sapiens OX=9606 GN=SMPDL3B PE=1 SV=2	50.8	5.4	16.2	1	2.2	40.41	547			
548	VACHT_HUMAN	Vesicular acetylcholine transporter OS=Homo sapiens OX=9606 GN=SLC18A3 PE=1 SV=2	56.9	5.8	16.2	1	2.4	766.89	548			
549	E41L1_HUMAN	Band 4.1-like protein 1 OS=Homo sapiens OX=9606 GN=EPB41L1 PE=1 SV=2	98.4	5.4	16.2	1	1.5	33.55	549			
550	CYTC_HUMAN	Cystatin-C OS=Homo sapiens OX=9606 GN=CST3 PE=1 SV=1	15.8	9.0	16.2	1	7.5	47.78	550			
551	MRM3_HUMAN	rRNA methyltransferase 3, mitochondrial OS=Homo sapiens OX=9606 GN=MRM3 PE=1 SV=2	47.0	8.9	16.2	1	2.6	25.06	551			
552	NHRF4_HUMAN	Na(+)/H(+) exchange regulatory cofactor NHE-RF4 OS=Homo sapiens OX=9606 GN=PDZD3 PE=1 SV=2	61.0	6.2	16.1	1	3.3	523.76	552			
553	NEB1_HUMAN	Neurabin-1 OS=Homo sapiens OX=9606 GN=PPP1R9A PE=1 SV=2	123.3	5.0	16.1	1	0.9	13.09	553			
554	PLXA4_HUMAN	Plexin-A4 OS=Homo sapiens OX=9606 GN=PLXNA4 PE=1 SV=4	212.3	6.4	16.1	1	0.8	630.32	554			
555	DPYL4_HUMAN	Dihydropyrimidinase-related protein 4 OS=Homo sapiens OX=9606 GN=DPYSL4 PE=1 SV=2	61.8	6.6	16.1	1	1.9	917.05	555			
556	CQ047_HUMAN	Uncharacterized protein C17orf47 OS=Homo sapiens OX=9606 GN=C17orf47 PE=2 SV=3	63.1	9.3	16.1	1	1.6	979.00	556			
557	FA49B_HUMAN	Protein FAM49B OS=Homo sapiens OX=9606 GN=FAM49B PE=1 SV=1	36.7	5.8	16.1	1	2.2	1104.36	557			
558	TRIM9_HUMAN	E3 ubiquitin-protein ligase TRIM9 OS=Homo sapiens OX=9606 GN=TRIM9 PE=1 SV=1	79.1	6.4	16.1	1	1.7	40.31	558			

559	CD1D_HUMAN	Antigen-presenting glycoprotein CD1d OS=Homo sapiens OX=9606 GN=CD1D PE=1 SV=1	37.7	8.4	16.1		1	4.8	39.95	559				
560	GAB1_HUMAN	GRB2-associated-binding protein 1 OS=Homo sapiens OX=9606 GN=GAB1 PE=1 SV=2	76.6	5.6	16.1		1	2.0	3.65	560				
561	ANR35_HUMAN	Ankyrin repeat domain-containing protein 35 OS=Homo sapiens OX=9606 GN=ANKRD35 PE=2 SV=2	109.9	5.8	16.1		1	0.9	9.83	561				
562	PRKDC_HUMAN	DNA-dependent protein kinase catalytic subunit OS=Homo sapiens OX=9606 GN=PRKDC PE=1 SV=3	468.8	6.7	16.1		1	0.3	698.50	562				
563	DYH1_HUMAN	Dynein heavy chain 1, axonemal OS=Homo sapiens OX=9606 GN=DNAH1 PE=1 SV=5	487.2	5.6	16.1		1	0.3	37.58	563				
564	NAR3_HUMAN	Ecto-ADP-ribosyltransferase 3 OS=Homo sapiens OX=9606 GN=ART3 PE=1 SV=2	43.9	5.7	16.0		1	3.6	14.42	564				
565	AP3M1_HUMAN	AP-3 complex subunit mu-1 OS=Homo sapiens OX=9606 GN=AP3M1 PE=1 SV=1	46.9	6.5	16.0		1	2.9	42.81	565				
566	NRDE2_HUMAN	Nuclear exosome regulator NRDE2 OS=Homo sapiens OX=9606 GN=NRDE2 PE=1 SV=3	132.6	7.7	16.0		1	0.8	14.66	566				
567	HPPD_HUMAN	4-hydroxyphenylpyruvate dioxygenase OS=Homo sapiens OX=9606 GN=HPD PE=1 SV=2	44.9	6.5	16.0		1	2.5	966.51	567				
568	MKS1_HUMAN	Meckel syndrome type 1 protein OS=Homo sapiens OX=9606 GN=MKS1 PE=1 SV=2	64.5	6.0	16.0		1	1.8	919.40	568				
569	NCBP3_HUMAN	Nuclear cap-binding protein subunit 3 OS=Homo sapiens OX=9606 GN=NCBP3 PE=1 SV=2	70.5	5.6	16.0		1	1.1	22.98	569				
570	ADM2_HUMAN	Protein ADM2 OS=Homo sapiens OX=9606 GN=ADM2 PE=1 SV=1	15.9	11.8	16.0		1	10.8	1.70	570				
571	GTPB1_HUMAN	GTP-binding protein 1 OS=Homo sapiens OX=9606 GN=GTPBP1 PE=1 SV=3	72.4	8.6	16.0		1	2.1	580.45	571				
572	FGD5_HUMAN	FYVE, RhoGEF and PH domain-containing protein 5 OS=Homo sapiens OX=9606 GN=FGD5 PE=1 SV=3	159.8	4.9	16.0		1	0.5	1122.23	572				
573	PTBP2_HUMAN	Polypyrimidine tract-binding protein 2 OS=Homo sapiens OX=9606 GN=PTBP2 PE=1 SV=1	57.5	8.7	16.0		1	1.3	1097.05	573				
574	ABCG5_HUMAN	ATP-binding cassette sub-family G member 5 OS=Homo sapiens OX=9606 GN=ABCG5 PE=1 SV=1	72.5	9.2	16.0		1	1.7	48.03	574				

575	ZHX2_HUMAN	Zinc fingers and homeoboxes protein 2 OS=Homo sapiens OX=9606 GN=ZHX2 PE=1 SV=1	92.2	6.4	16.0	1	0.8	54.53	575			
576	SO1B1_HUMAN	Solute carrier organic anion transporter family member 1B1 OS=Homo sapiens OX=9606 GN=SLCO1B1 PE=1 SV=2	76.4	8.9	16.0	1	1.2	30.70	576			
577	SMAG1_HUMAN	Protein Smaug homolog 1 OS=Homo sapiens OX=9606 GN=SAMD4A PE=1 SV=3	79.4	8.6	16.0	1	1.3	942.16	577			
578	ICAL_HUMAN	Calpastatin OS=Homo sapiens OX=9606 GN=CAST PE=1 SV=4	76.5	5.0	15.9	1	1.1	12.30	578			
579	CBLB_HUMAN	E3 ubiquitin-protein ligase CBL-B OS=Homo sapiens OX=9606 GN=CBLB PE=1 SV=2	109.4	8.1	15.9	1	1.2	784.33	579			
580	CP2C8_HUMAN	Cytochrome P450 2C8 OS=Homo sapiens OX=9606 GN=CYP2C8 PE=1 SV=2	55.8	8.8	15.9	1	2.0	52.77	580			
581	PLXC1_HUMAN	Plexin-C1 OS=Homo sapiens OX=9606 GN=PLXNC1 PE=1 SV=1	175.6	7.8	15.9	1	0.5	930.14	581			
582	PKHN1_HUMAN	Pleckstrin homology domain-containing family N member 1 OS=Homo sapiens OX=9606 GN=PLEKHN1 PE=1 SV=3	66.4	9.1	15.9	1	1.6	919.83	582			
583	ARI5A_HUMAN	AT-rich interactive domain-containing protein 5A OS=Homo sapiens OX=9606 GN=ARID5A PE=1 SV=2	64.0	9.3	15.9	1	1.7	47.99	583			
584	C1GLC_HUMAN	C1GALT1-specific chaperone 1 OS=Homo sapiens OX=9606 GN=C1GALT1C1 PE=1 SV=1	36.4	6.4	15.9	1	2.2	1215.89	584			
585	RBMX2_HUMAN	RNA-binding motif protein, X-linked 2 OS=Homo sapiens OX=9606 GN=RBMX2 PE=1 SV=2	37.3	9.8	15.8	1	3.1	1.27	585			
586	MCM8_HUMAN	DNA helicase MCM8 OS=Homo sapiens OX=9606 GN=MCM8 PE=1 SV=2	93.6	7.8	15.8	1	1.2	833.89	586			
587	IDD_HUMAN	Integral membrane protein DGCR2/IDD OS=Homo sapiens OX=9606 GN=DGCR2 PE=1 SV=1	60.8	5.1	15.8	1	1.3	1092.04	587			
588	TTLL8_HUMAN	Protein monoglycylase TTLL8 OS=Homo sapiens OX=9606 GN=TTLL8 PE=2 SV=4	94.6	8.5	15.8	1	1.6	20.24	588			
589	ZN473_HUMAN	Zinc finger protein 473 OS=Homo sapiens OX=9606 GN=ZNF473 PE=1 SV=1	100.1	8.6	15.8	1	0.9	1122.34	589			
590	MAST3_HUMAN	Microtubule-associated serine/threonine-protein kinase 3 OS=Homo sapiens OX=9606 GN=MAST3 PE=1 SV=2	143.0	8.2	15.8	1	0.9	690.95	590			

591	CP2CJ_HUMAN	Cytochrome P450 2C19 OS=Homo sapiens OX=9606 GN=CYP2C19 PE=1 SV=3	55.9	7.1	15.8	1	4.3	16.45	591			
592	PDILT_HUMAN	Protein disulfide-isomerase-like protein of the testis OS=Homo sapiens OX=9606 GN=PDILT PE=1 SV=2	66.6	6.4	15.8	1	1.9	3.50	592			
593	COBA2_HUMAN	Collagen alpha-2(XI) chain OS=Homo sapiens OX=9606 GN=COL11A2 PE=1 SV=5	171.7	5.9	15.8	1	1.0	32.08	593			
594	COCA1_HUMAN	Collagen alpha-1(XII) chain OS=Homo sapiens OX=9606 GN=COL12A1 PE=1 SV=2	332.9	5.4	15.8	1	0.5	22.16	594			
595	FA11_HUMAN	Coagulation factor XI OS=Homo sapiens OX=9606 GN=F11 PE=1 SV=1	70.1	8.5	15.7	1	1.1	55.06	595			
596	ZN816_HUMAN	Zinc finger protein 816 OS=Homo sapiens OX=9606 GN=ZNF816 PE=2 SV=2	75.7	9.4	15.7	1	1.2	78.40	596			
597	DYH10_HUMAN	Dynein heavy chain 10, axonemal OS=Homo sapiens OX=9606 GN=DNAH10 PE=1 SV=4	514.5	5.6	15.7	1	0.2	48.59	597			
598	MUC12_HUMAN	Mucin-12 OS=Homo sapiens OX=9606 GN=MUC12 PE=1 SV=2	557.8	5.3	15.7	1	0.2	21.65	598			
599	F86C2_HUMAN	Putative protein FAM86C2P OS=Homo sapiens OX=9606 GN=FAM86C2P PE=5 SV=1	18.5	8.2	15.7	1	11.5	508.33	599			
600	SIA4C_HUMAN	CMP-N-acetylneuraminate-beta-galactosamide-alpha-2,3-sialyltransferase 4 OS=Homo sapiens OX=9606 GN=ST3GAL4 PE=2 SV=1	38.0	9.4	15.7	1	3.0	786.59	600			
601	LAR4B_HUMAN	La-related protein 4B OS=Homo sapiens OX=9606 GN=LARP4B PE=1 SV=3	80.5	6.5	15.7	1	1.9	646.99	601			
602	PZRN3_HUMAN	E3 ubiquitin-protein ligase PDZRN3 OS=Homo sapiens OX=9606 GN=PDZRN3 PE=1 SV=2	119.5	5.7	15.7	1	0.8	47.82	602			
603	REV1_HUMAN	DNA repair protein REV1 OS=Homo sapiens OX=9606 GN=REV1 PE=1 SV=1	138.2	8.8	15.7	1	0.8	41.91	603			
604	MAGD2_HUMAN	Melanoma-associated antigen D2 OS=Homo sapiens OX=9606 GN=MAGED2 PE=1 SV=2	64.9	9.3	15.7	1	1.8	38.13	604			
605	MYO5B_HUMAN	Unconventional myosin-Vb OS=Homo sapiens OX=9606 GN=MYO5B PE=1 SV=3	213.5	6.8	15.7	1	0.5	883.83	605			
606	IDLC_HUMAN	Axonemal dynein light intermediate polypeptide 1 OS=Homo sapiens OX=9606 GN=DNALI1 PE=1 SV=2	29.6	8.7	15.7	1	6.6	639.33	606			

607	CRBB2_HUMAN	Beta-crystallin B2 OS=Homo sapiens OX=9606 GN=CRYBB2 PE=1 SV=2	23.4	6.5	15.6	1	3.4	89.31	607			
608	GBB3_HUMAN	Guanine nucleotide-binding protein G(I)/G(S)/G(T) subunit beta-3 OS=Homo sapiens OX=9606 GN=GNB3 PE=1 SV=1	37.2	5.4	15.6	1	2.9	945.60	608			
609	FA98B_HUMAN	Protein FAM98B OS=Homo sapiens OX=9606 GN=FAM98B PE=1 SV=2	45.5	8.9	15.6	1	2.1	1017.90	609			
610	PIMRE_HUMAN	Protein PIMREG OS=Homo sapiens OX=9606 GN=PIMREG PE=1 SV=1	27.5	10.2	15.6	1	5.6	15.85	610			
611	PLEC_HUMAN	Plectin OS=Homo sapiens OX=9606 GN=PLEC PE=1 SV=3	531.5	5.7	15.6	1	0.2	1014.34	611			
612	ANR40_HUMAN	Ankyrin repeat domain-containing protein 40 OS=Homo sapiens OX=9606 GN=ANKRD40 PE=1 SV=2	41.1	4.9	15.6	1	2.7	14.08	612			
613	TENA_HUMAN	Tenascin OS=Homo sapiens OX=9606 GN=TNC PE=1 SV=3	240.7	4.8	15.6	1	0.4	49.85	613			
614	MYZAP_HUMAN	Myocardial zonula adherens protein OS=Homo sapiens OX=9606 GN=MYZAP PE=1 SV=1	54.2	5.9	15.6	1	1.9	40.79	614			
615	ZN703_HUMAN	Zinc finger protein 703 OS=Homo sapiens OX=9606 GN=ZNF703 PE=1 SV=1	58.2	9.0	15.6	1	2.0	40.88	615			
616	ARI4A_HUMAN	AT-rich interactive domain-containing protein 4A OS=Homo sapiens OX=9606 GN=ARID4A PE=1 SV=3	142.7	5.0	15.6	1	1.0	3.54	616	0.22	1	0.00
617	CHD4_HUMAN	Chromodomain-helicase-DNA-binding protein 4 OS=Homo sapiens OX=9606 GN=CHD4 PE=1 SV=2	217.9	5.6	15.6	1	0.5	17.50	617			
618	DGKK_HUMAN	Diacylglycerol kinase kappa OS=Homo sapiens OX=9606 GN=DGKK PE=1 SV=1	141.7	5.4	15.5	1	0.6	11.57	618			
619	CHERP_HUMAN	Calcium homeostasis endoplasmic reticulum protein OS=Homo sapiens OX=9606 GN=CHERP PE=1 SV=3	103.6	9.1	15.5	1	1.7	36.24	619			
620	SFI1_HUMAN	Protein SFI1 homolog OS=Homo sapiens OX=9606 GN=SFI1 PE=1 SV=2	147.6	10.8	15.5	1	0.9	5.38	620			
621	MET25_HUMAN	Methyltransferase-like protein 25 OS=Homo sapiens OX=9606 GN=METTL25 PE=2 SV=2	68.2	6.7	15.5	1	1.2	30.78	621			
622	OAT_HUMAN	Ornithine aminotransferase, mitochondrial OS=Homo sapiens OX=9606 GN=OAT PE=1 SV=1	48.5	6.6	15.5	1	2.3	917.11	622			
623	IWS1_HUMAN	Protein IWS1 homolog OS=Homo sapiens OX=9606 GN=IWS1 PE=1 SV=2	91.9	4.6	15.5	1	1.1	893.22	623			
624	CHK2_HUMAN	Serine/threonine-protein kinase Chk2 OS=Homo sapiens OX=9606 GN=CHEK2 PE=1 SV=1	60.9	5.7	15.5	1	1.7	18.74	624			

625	GRPE1_HUMAN	GrpE protein homolog 1, mitochondrial OS=Homo sapiens OX=9606 GN=GRPEL1 PE=1 SV=2	24.3	8.2	15.5	1	4.1	928.84	625			
626	PDE7A_HUMAN	High affinity cAMP-specific 3',5'-cyclic phosphodiesterase 7A OS=Homo sapiens OX=9606 GN=PDE7A PE=1 SV=2	55.5	7.1	15.5	1	3.3	536.41	626			
627	KVD12_HUMAN	Immunoglobulin kappa variable 1D-12 OS=Homo sapiens OX=9606 GN=IGKV1D-12 PE=1 SV=2	12.6	7.7	15.5	1	13.7	44.50	627			
628	AL9A1_HUMAN	4-trimethylaminobutyraldehyde dehydrogenase OS=Homo sapiens OX=9606 GN=ALDH9A1 PE=1 SV=3	53.8	5.7	15.5	1	2.4	13.98	628			
629	NAL13_HUMAN	NACHT, LRR and PYD domains-containing protein 13 OS=Homo sapiens OX=9606 GN=NLRP13 PE=2 SV=2	118.8	5.4	15.5	1	0.9	831.86	629			
630	SC5A9_HUMAN	Sodium/glucose cotransporter 4 OS=Homo sapiens OX=9606 GN=SLC5A9 PE=1 SV=2	74.0	6.7	15.4	1	2.5	597.73	630			
631	NHLC2_HUMAN	NHL repeat-containing protein 2 OS=Homo sapiens OX=9606 GN=NHLRC2 PE=1 SV=1	79.4	5.3	15.4	1	1.9	564.11	631			
632	HIP1R_HUMAN	Huntingtin-interacting protein 1-related protein OS=Homo sapiens OX=9606 GN=HIP1R PE=1 SV=2	119.3	6.2	15.4	1	0.8	52.87	632			
633	RHDF1_HUMAN	Inactive rhomboid protein 1 OS=Homo sapiens OX=9606 GN=RHBDF1 PE=1 SV=2	97.3	8.8	15.4	1	1.1	42.18	633			
634	FA8_HUMAN	Coagulation factor VIII OS=Homo sapiens OX=9606 GN=F8 PE=1 SV=1	266.8	6.9	15.4	1	0.5	29.20	634			
635	MPRI_HUMAN	Cation-independent mannose-6-phosphate receptor OS=Homo sapiens OX=9606 GN=IGF2R PE=1 SV=3	274.2	5.6	15.4	1	0.3	61.49	635			
636	NAT14_HUMAN	N-acetyltransferase 14 OS=Homo sapiens OX=9606 GN=NAT14 PE=1 SV=1	21.6	10.7	15.4	1	5.8	2.12	636			
637	EMAL5_HUMAN	Echinoderm microtubule-associated protein-like 5 OS=Homo sapiens OX=9606 GN=EML5 PE=2 SV=3	219.3	7.9	15.4	1	0.5	982.18	637			
638	IF3M_HUMAN	Translation initiation factor IF-3, mitochondrial OS=Homo sapiens OX=9606 GN=MTIF3 PE=1 SV=4	31.7	9.7	15.4	1	3.2	65.13	638			
639	FBX33_HUMAN	F-box only protein 33 OS=Homo sapiens OX=9606 GN=FBXO33 PE=1 SV=1	62.6	7.0	15.4	1	2.5	22.08	639			

640	NIP7_HUMAN	60S ribosome subunit biogenesis protein NIP7 homolog OS=Homo sapiens OX=9606 GN=NIP7 PE=1 SV=1	20.4	8.7	15.4	1	4.4	1020.58	640			
641	CDC5L_HUMAN	Cell division cycle 5-like protein OS=Homo sapiens OX=9606 GN=CDC5L PE=1 SV=2	92.2	8.2	15.4	1	1.1	42.96	641			
642	PDPR_HUMAN	Pyruvate dehydrogenase phosphatase regulatory subunit, mitochondrial OS=Homo sapiens OX=9606 GN=PDPR PE=1 SV=2	99.3	5.9	15.4	1	1.4	33.71	642			
643	TSAP1_HUMAN	tRNA selenocysteine 1-associated protein 1 OS=Homo sapiens OX=9606 GN=TRNAU1AP PE=1 SV=1	32.5	4.6	15.4	1	4.5	35.65	643			
644	TNS2_HUMAN	Tensin-2 OS=Homo sapiens OX=9606 GN=TNS2 PE=1 SV=2	152.5	8.7	15.4	1	0.5	1244.99	644			
645	RREB1_HUMAN	Ras-responsive element-binding protein 1 OS=Homo sapiens OX=9606 GN=RREB1 PE=1 SV=3	181.3	6.5	15.4	1	0.7	840.34	645			
646	KAD9_HUMAN	Adenylate kinase 9 OS=Homo sapiens OX=9606 GN=AK9 PE=1 SV=2	221.3	5.0	15.4	1	0.4	69.34	646			
647	PKHD1_HUMAN	Fibrocystin OS=Homo sapiens OX=9606 GN=PKHD1 PE=1 SV=1	446.4	6.1	15.4	1	0.3	746.36	647			
648	RM52_HUMAN	39S ribosomal protein L52, mitochondrial OS=Homo sapiens OX=9606 GN=MRPL52 PE=1 SV=2	13.7	10.3	15.3	1	7.3	883.71	648			
649	ZN100_HUMAN	Zinc finger protein 100 OS=Homo sapiens OX=9606 GN=ZNF100 PE=2 SV=2	62.7	9.2	15.3	1	3.1	589.26	649			
650	IL36G_HUMAN	Interleukin-36 gamma OS=Homo sapiens OX=9606 GN=IL36G PE=1 SV=1	18.7	5.0	15.3	1	5.3	21.81	650			
651	CN37_HUMAN	2',3'-cyclic-nucleotide 3'-phosphodiesterase OS=Homo sapiens OX=9606 GN=CNP PE=1 SV=2	47.5	9.2	15.3	1	3.3	584.60	651			
652	CSCL1_HUMAN	CSC1-like protein 1 OS=Homo sapiens OX=9606 GN=TMEM63A PE=1 SV=3	92.1	7.0	15.3	1	2.2	437.16	652			
653	RAB17_HUMAN	Ras-related protein Rab-17 OS=Homo sapiens OX=9606 GN=RAB17 PE=1 SV=2	23.5	7.7	15.3	1	4.7	47.57	653			
654	CHPF2_HUMAN	Chondroitin sulfate glucuronyltransferase OS=Homo sapiens OX=9606 GN=CHPF2 PE=2 SV=2	85.9	7.9	15.3	1	1.3	949.80	654			
655	PATL1_HUMAN	Protein PAT1 homolog 1 OS=Homo sapiens OX=9606 GN=PATL1 PE=1 SV=2	86.8	6.2	15.3	1	1.4	25.19	655			
656	LV151_HUMAN	Immunoglobulin lambda variable 1-51 OS=Homo sapiens OX=9606 GN=IGLV1-51 PE=1 SV=2	12.2	6.5	15.3	1	13.7	41.76	656			

657	F1711_HUMAN	Protein FAM171A1 OS=Homo sapiens OX=9606 GN=FAM171A1 PE=1 SV=1	97.8	6.1	15.3	1	1.0	22.90	657			
658	PRCC_HUMAN	Proline-rich protein PRCC OS=Homo sapiens OX=9606 GN=PRCC PE=1 SV=1	52.4	5.0	15.3	1	4.1	449.39	658			
659	RAD9B_HUMAN	Cell cycle checkpoint control protein RAD9B OS=Homo sapiens OX=9606 GN=RAD9B PE=1 SV=2	47.8	6.5	15.2	1	3.1	630.28	659			
660	SAXO1_HUMAN	Stabilizer of axonemal microtubules 1 OS=Homo sapiens OX=9606 GN=SAXO1 PE=1 SV=2	54.6	8.7	15.2	1	2.7	46.64	660			
661	YDJC_HUMAN	Carbohydrate deacetylase OS=Homo sapiens OX=9606 GN=YDJC PE=1 SV=1	34.4	6.0	15.2	1	4.0	12.96	661			
662	T3HPD_HUMAN	Trans-3-hydroxy-L-proline dehydratase OS=Homo sapiens OX=9606 GN=L3HYPDH PE=1 SV=2	38.1	6.2	15.2	1	2.3	1.97	662			
663	DIAP1_HUMAN	Protein diaphanous homolog 1 OS=Homo sapiens OX=9606 GN=DIAPH1 PE=1 SV=2	141.3	5.3	15.2	1	0.6	1068.41	663			
664	UCKL1_HUMAN	Uridine-cytidine kinase-like 1 OS=Homo sapiens OX=9606 GN=UCKL1 PE=1 SV=2	61.1	6.9	15.2	1	1.5	90.47	664			
665	MRP6_HUMAN	Multidrug resistance-associated protein 6 OS=Homo sapiens OX=9606 GN=ABCC6 PE=1 SV=2	164.8	8.8	15.2	1	0.7	791.11	665			
666	LEG2_HUMAN	Galectin-2 OS=Homo sapiens OX=9606 GN=LGALS2 PE=1 SV=3	14.6	5.9	15.2	1	8.3	764.38	666			
667	SI1L2_HUMAN	Signal-induced proliferation-associated 1-like protein 2 OS=Homo sapiens OX=9606 GN=SIPA1L2 PE=1 SV=2	190.3	6.3	15.2	1	1.0	474.09	667			
668	B3GT2_HUMAN	Beta-1,3-galactosyltransferase 2 OS=Homo sapiens OX=9606 GN=B3GALT2 PE=1 SV=1	49.2	9.5	15.2	1	2.8	607.33	668			
669	FBH1_HUMAN	F-box DNA helicase 1 OS=Homo sapiens OX=9606 GN=FBH1 PE=1 SV=2	117.6	8.6	15.2	1	0.8	1102.33	669			
670	ROCK2_HUMAN	Rho-associated protein kinase 2 OS=Homo sapiens OX=9606 GN=ROCK2 PE=1 SV=4	160.8	5.8	15.2	1	0.7	68.90	670			
671	MEGF6_HUMAN	Multiple epidermal growth factor-like domains protein 6 OS=Homo sapiens OX=9606 GN=MEGF6 PE=1 SV=4	161.1	5.9	15.2	1	0.9	2.29	671			
672	ZN608_HUMAN	Zinc finger protein 608 OS=Homo sapiens OX=9606 GN=ZNF608 PE=1 SV=4	162.1	8.9	15.2	1	1.0	17.35	672			
673	ZDH15_HUMAN	Palmitoyltransferase ZDHHC15 OS=Homo sapiens OX=9606 GN=ZDHHC15 PE=1 SV=1	39.3	8.4	15.2	1	3.3	804.18	673			

674	NR2E3_HUMAN	Photoreceptor-specific nuclear receptor OS=Homo sapiens OX=9606 GN=NR2E3 PE=1 SV=1	44.7	8.2	15.2	1	2.2	977.89	674			
675	LRC34_HUMAN	Leucine-rich repeat-containing protein 34 OS=Homo sapiens OX=9606 GN=LRRC34 PE=2 SV=3	51.2	6.3	15.2	1	2.6	717.57	675			
676	HUNK_HUMAN	Hormonally up-regulated neu tumor-associated kinase OS=Homo sapiens OX=9606 GN=HUNK PE=1 SV=1	79.6	9.2	15.2	1	1.3	877.61	676			
677	ZN806_HUMAN	Zinc finger protein 806 OS=Homo sapiens OX=9606 GN=ZNF806 PE=3 SV=1	67.8	8.8	15.2	1	1.5	55.64	677			
678	EXOC1_HUMAN	Exocyst complex component 1 OS=Homo sapiens OX=9606 GN=EXOC1 PE=1 SV=4	101.9	6.2	15.2	1	1.2	727.65	678			
679	SYVM_HUMAN	Valine--tRNA ligase, mitochondrial OS=Homo sapiens OX=9606 GN=VARS2 PE=1 SV=2	118.4	6.6	15.2	1	1.6	614.02	679			
680	MND1_HUMAN	Meiotic nuclear division protein 1 homolog OS=Homo sapiens OX=9606 GN=MND1 PE=1 SV=1	23.7	8.3	15.2	1	3.9	1055.14	680			
681	LYPA2_HUMAN	Acyl-protein thioesterase 2 OS=Homo sapiens OX=9606 GN=LYPLA2 PE=1 SV=1	24.7	6.7	15.2	1	3.5	41.23	681			
682	CLUS_HUMAN	Clusterin OS=Homo sapiens OX=9606 GN=CLU PE=1 SV=1	52.5	5.9	15.2	1	2.4	39.84	682			
683	TOP1M_HUMAN	DNA topoisomerase I, mitochondrial OS=Homo sapiens OX=9606 GN=TOP1MT PE=1 SV=1	69.8	9.5	15.2	1	2.3	22.65	683			
684	MOBP_HUMAN	Myelin-associated oligodendrocyte basic protein OS=Homo sapiens OX=9606 GN=MOBP PE=1 SV=2	20.9	11.3	15.1	1	6.0	959.59	684			
685	PAAT_HUMAN	ATPase PAAT OS=Homo sapiens OX=9606 GN=PAAT PE=1 SV=2	49.2	5.9	15.1	1	3.6	9.38	685			
686	ASXL1_HUMAN	Polycomb group protein ASXL1 OS=Homo sapiens OX=9606 GN=ASXL1 PE=1 SV=4	165.3	5.8	15.1	1	0.5	13.23	686			
687	CSN7A_HUMAN	COP9 signalosome complex subunit 7a OS=Homo sapiens OX=9606 GN=COPS7A PE=1 SV=1	30.3	8.3	15.1	1	8.0	27.49	687			
688	AT7L2_HUMAN	Ataxin-7-like protein 2 OS=Homo sapiens OX=9606 GN=ATXN7L2 PE=1 SV=1	77.1	9.4	15.1	1	2.5	515.49	688			
689	ARVC_HUMAN	Armadillo repeat protein deleted in velo-cardio-facial syndrome OS=Homo sapiens OX=9606 GN=ARVCF PE=1 SV=1	104.6	6.4	15.1	1	0.9	15.17	689			

690	SAMD7_HUMAN	Sterile alpha motif domain-containing protein 7 OS=Homo sapiens OX=9606 GN=SAMD7 PE=1 SV=1	49.1	6.3	15.1	1	2.7	9.15	690				
691	FAD1_HUMAN	FAD synthase OS=Homo sapiens OX=9606 GN=FLAD1 PE=1 SV=1	65.2	6.5	15.1	1	1.5	11.62	691				
692	NEBL_HUMAN	Nebulette OS=Homo sapiens OX=9606 GN=NEBL PE=1 SV=1	116.4	7.9	15.1	1	1.3	13.30	692				
693	TAF2_HUMAN	Transcription initiation factor TFIID subunit 2 OS=Homo sapiens OX=9606 GN=TAF2 PE=1 SV=3	136.9	8.5	15.1	1	1.0	36.51	693				
694	AT5L2_HUMAN	Putative ATP synthase subunit g 2, mitochondrial OS=Homo sapiens OX=9606 GN=ATP5MGL PE=5 SV=1	11.0	9.9	15.1	1	10.0	63.34	694				
695	ZHANG_HUMAN	CREB/ATF bZIP transcription factor OS=Homo sapiens OX=9606 GN=CREBZF PE=1 SV=2	37.1	5.1	15.1	1	2.8	60.42	695				
696	AGAP2_HUMAN	Arf-GAP with GTPase, ANK repeat and PH domain-containing protein 2 OS=Homo sapiens OX=9606 GN=AGAP2 PE=1 SV=2	124.6	9.9	15.1	1	1.1	52.26	696				
697	CHIN_HUMAN	N-chimaerin OS=Homo sapiens OX=9606 GN=CHN1 PE=1 SV=3	53.1	6.5	15.1	1	3.5	498.33	697				
698	SRARP_HUMAN	Steroid receptor-associated and regulated protein OS=Homo sapiens OX=9606 GN=SRARP PE=1 SV=1	17.6	9.2	15.1	1	4.7	12.35	698				
699	ENAM_HUMAN	Enamelin OS=Homo sapiens OX=9606 GN=ENAM PE=1 SV=3	128.7	6.4	15.1	1	0.8	50.94	699				
700	RFX5_HUMAN	DNA-binding protein RFX5 OS=Homo sapiens OX=9606 GN=RFX5 PE=1 SV=1	65.3	9.4	15.1	1	1.5	855.10	700				
701	APOC2_HUMAN	Apolipoprotein C-II OS=Homo sapiens OX=9606 GN=APOC2 PE=1 SV=1	11.3	4.6	15.0	1	10.9	51.56	701				
702	PTHD3_HUMAN	Patched domain-containing protein 3 OS=Homo sapiens OX=9606 GN=PTCHD3 PE=1 SV=3	86.8	5.8	15.0	1	1.2	38.64	702				
703	VP13B_HUMAN	Vacuolar protein sorting-associated protein 13B OS=Homo sapiens OX=9606 GN=VPS13B PE=1 SV=2	448.4	6.0	15.0	1	0.3	990.61	703				
704	RUXF_HUMAN	Small nuclear ribonucleoprotein F OS=Homo sapiens OX=9606 GN=SNRPF PE=1 SV=1	9.7	4.6	15.0	1	9.3	944.18	704				
705	CLPT1_HUMAN	Cleft lip and palate transmembrane protein 1 OS=Homo sapiens OX=9606 GN=CLPTM1 PE=1 SV=1	76.0	5.9	15.0	1	1.2	1184.03	705				
706	ACO11_HUMAN	Acyl-coenzyme A thioesterase 11 OS=Homo sapiens OX=9606 GN=ACOT11 PE=1 SV=1	68.4	8.7	15.0	1	1.3	81.57	706				

707	SYLM_HUMAN	Probable leucine--tRNA ligase, mitochondrial OS=Homo sapiens OX=9606 GN=LARS2 PE=1 SV=2	101.9	8.5	15.0	1	1.1	853.49	707			
708	RIN2_HUMAN	Ras and Rab interactor 2 OS=Homo sapiens OX=9606 GN=RIN2 PE=1 SV=1	100.1	6.2	15.0	1	1.5	14.01	708			
709	NOD2_HUMAN	Nucleotide-binding oligomerization domain-containing protein 2 OS=Homo sapiens OX=9606 GN=NOD2 PE=1 SV=1	115.2	6.3	15.0	1	1.1	50.52	709			

MW=Molecular Weight, PI= Isoelectric Point, SC=Sequence Coverage, LVEF=Left Ventricular Ejection Fraction, CV= coefficients of variation.

Table S2. GO classification for the 16 significant differentially expressed proteins in both groups (LVEF≥45 vs LVEF<45)

Biological process		
GO-term	#Seqs	Sequence Names
localization	4	sp|Q9ULD2|MTUS1_HUMAN, sp|P02787|TRFE_HUMAN, sp|Q9BZF3|OSBL6_HUMAN, sp|P06727|APOA4_HUMAN
cellular component organization or biogenesis	1	sp|O43189|PHF1_HUMAN
signaling	2	sp|Q8TEJ3|SH3R3_HUMAN, sp|Q17R89|RHG44_HUMAN
cellular process	7	sp|Q9ULD2|MTUS1_HUMAN, sp|P02787|TRFE_HUMAN, sp|O43189|PHF1_HUMAN, sp|Q13421|MSLN_HUMAN, sp|Q8TEJ3|SH3R3_HUMAN, sp|Q9BZF3|OSBL6_HUMAN, sp|Q17R89|RHG44_HUMAN
developmental process	1	sp|Q13421|MSLN_HUMAN
locomotion	1	sp|Q9ULD2|MTUS1_HUMAN
metabolic process	5	sp|O43189|PHF1_HUMAN, sp|Q13421|MSLN_HUMAN, sp|Q8TEJ3|SH3R3_HUMAN, sp|Q9BZF3|OSBL6_HUMAN, sp|P06727|APOA4_HUMAN
biological regulation	6	sp|Q9ULD2|MTUS1_HUMAN, sp|P02787|TRFE_HUMAN, sp|O43189|PHF1_HUMAN, sp|Q8TEJ3|SH3R3_HUMAN, sp|Q9BZF3|OSBL6_HUMAN, sp|Q17R89|RHG44_HUMAN
biological adhesion	1	sp|Q13421|MSLN_HUMAN
regulation of biological process	5	sp|Q9ULD2|MTUS1_HUMAN, sp|O43189|PHF1_HUMAN, sp|Q8TEJ3|SH3R3_HUMAN, sp|Q9BZF3|OSBL6_HUMAN, sp|Q17R89|RHG44_HUMAN

response to stimulus	4	sp|Q9ULD2|MTUS1_HUMAN, sp|O43189|PHF1_HUMAN, sp|Q8TEJ3|SH3R3_HUMAN, sp|Q17R89|RHG44_HUMAN
immune system process	1	sp|Q9ULD2|MTUS1_HUMAN
multicellular organismal process	1	sp|Q13421|MSLN_HUMAN
Molecular function		
transporter activity	2	sp|P02787|TRFE_HUMAN, sp|Q9BZF3|OSBL6_HUMAN
catalytic activity	1	sp|Q96QV1|HHIP_HUMAN
binding	13	sp|Q9ULD2|MTUS1_HUMAN, sp|O43189|PHF1_HUMAN, sp|Q13421|MSLN_HUMAN, sp|Q9BSW2|EFC4B_HUMAN, sp|P10070|GLI2_HUMAN, sp|Q17R89|RHG44_HUMAN, sp|P02787|TRFE_HUMAN, sp|Q12772|SRBP2_HUMAN, sp|P29374|ARI4A_HUMAN, sp|Q8TEJ3|SH3R3_HUMAN, sp|Q9BZF3|OSBL6_HUMAN, sp|P06727|APOA4_HUMAN, sp|Q5JPB2|ZN831_HUMAN
cellular component		
cell part	5	sp|Q9ULD2|MTUS1_HUMAN, sp|Q13421|MSLN_HUMAN, sp|Q92621|NU205_HUMAN, sp|Q9BZF3|OSBL6_HUMAN, sp|Q17R89|RHG44_HUMAN
organelle part	4	sp|Q9ULD2|MTUS1_HUMAN, sp|Q13421|MSLN_HUMAN, sp|Q92621|NU205_HUMAN, sp|Q9BZF3|OSBL6_HUMAN
membrane	3	sp|Q9ULD2|MTUS1_HUMAN, sp|Q13421|MSLN_HUMAN, sp|Q9BZF3|OSBL6_HUMAN
organelle	4	sp|Q9ULD2|MTUS1_HUMAN, sp|Q13421|MSLN_HUMAN, sp|Q92621|NU205_HUMAN, sp|Q9BZF3|OSBL6_HUMAN
supramolecular complex	1	sp|Q9ULD2|MTUS1_HUMAN
membrane part	2	sp|Q13421|MSLN_HUMAN, sp|Q9BZF3|OSBL6_HUMAN
extracellular region	4	sp|P02787|TRFE_HUMAN, sp|Q13421|MSLN_HUMAN, sp|P06727|APOA4_HUMAN, sp|P02768|ALBU_HUMAN
extracellular region part	3	sp|P02787|TRFE_HUMAN, sp|Q13421|MSLN_HUMAN, sp|P02768|ALBU_HUMAN
protein-containing complex	1	sp|Q92621|NU205_HUMAN
cell	5	sp|Q9ULD2|MTUS1_HUMAN, sp|Q13421|MSLN_HUMAN, sp|Q92621|NU205_HUMAN, sp|Q9BZF3|OSBL6_HUMAN, sp|Q17R89|RHG44_HUMAN
membrane-enclosed lumen	2	sp|Q9ULD2|MTUS1_HUMAN, sp|Q13421|MSLN_HUMAN
