# Supplementary material for: Pericardial fluid proteomic label-free quantification of differentially expressed proteins in ischemic heart disease patients with systolic dysfunction by nano-LC-ESI-MS/MS analysis
Source: RSC Adv. 2020 Dec 23;11(1):320–7. doi: 10.1039/d0ra08389e (PMC8691035; doi:10.1039/d0ra08389e)

Electronic Supplementary Material (ESI) for RSC Advances.
This journal is © The Royal Society of Chemistry 2020

Supplementary Information

Annotated spectra of all the identified proteins including the differentially expressed proteins.

Project Info

**Name:**	Label-Free Quantitative Analysis 4-3-2020	**Date:**	Mar 6, 2020

Sample Info & Protocols

**Name:**	MS/MS SPECTRA
**Date:**	Mar 6, 2020

Search Result Info

**Search Result:**	QTOF_allOrg_2020-03-06 15:27:13		
**Location:**	/Label-Free Quantitative Analysis 4-3-2020/"MS/MS SPECTRA"/		
**Search Method:**	QTOF_allOrg	**Version:**	13
**Search Engine(s):**	Mascot, 2.6.0	**Ident. Compound(s):**	122/14340 (FDR:0.56%)
**Database(s):**	SwissProt, SwissProt_2019_11.fasta		
**Note:**			

YT001_HUMAN

Accession: YT001_HUMAN
Database: SwissProt
MW [kDa] / pI: 6.4 / 10.3
Score: 21.5
Seq. Coverage [%]: 18.2
No. of Peptides: 1

Peptide Match

Sequence: R.ITVNTAFGTK.F
Score: 21.5
Modifications:

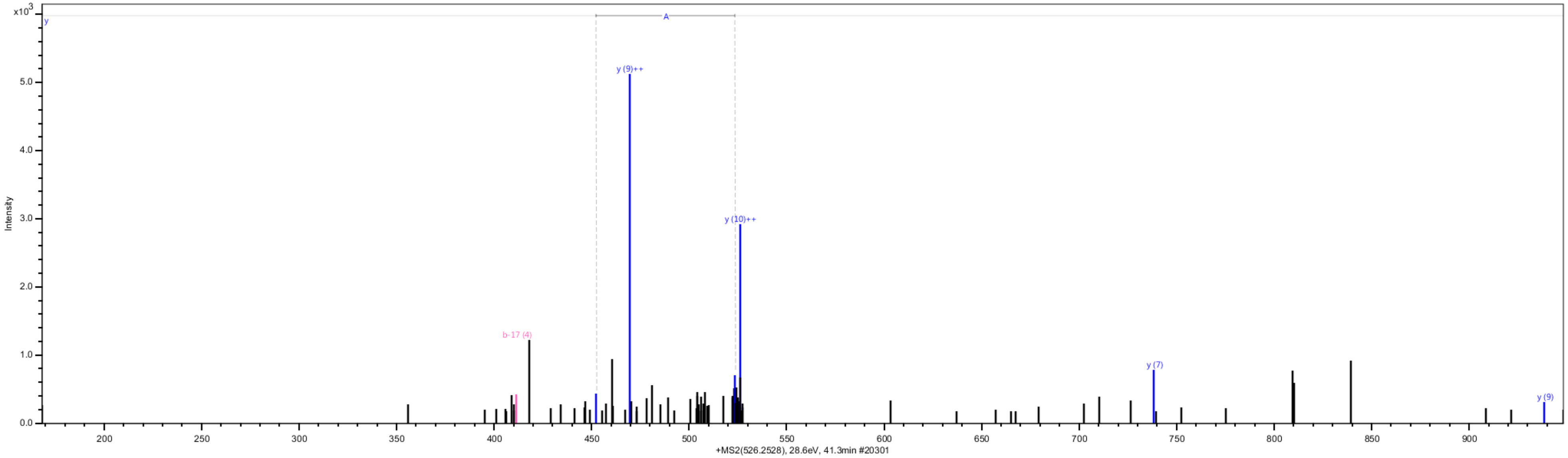

LRC39_HUMAN

Accession: LRC39_HUMAN
Database: SwissProt
MW [kDa] / pI: 38.8 / 6.0
Score: 23.9
Seq. Coverage [%]: 2.1
No. of Peptides: 1

Peptide Match

Sequence: K.LELAVNR.D
Score: 23.9
Modifications:

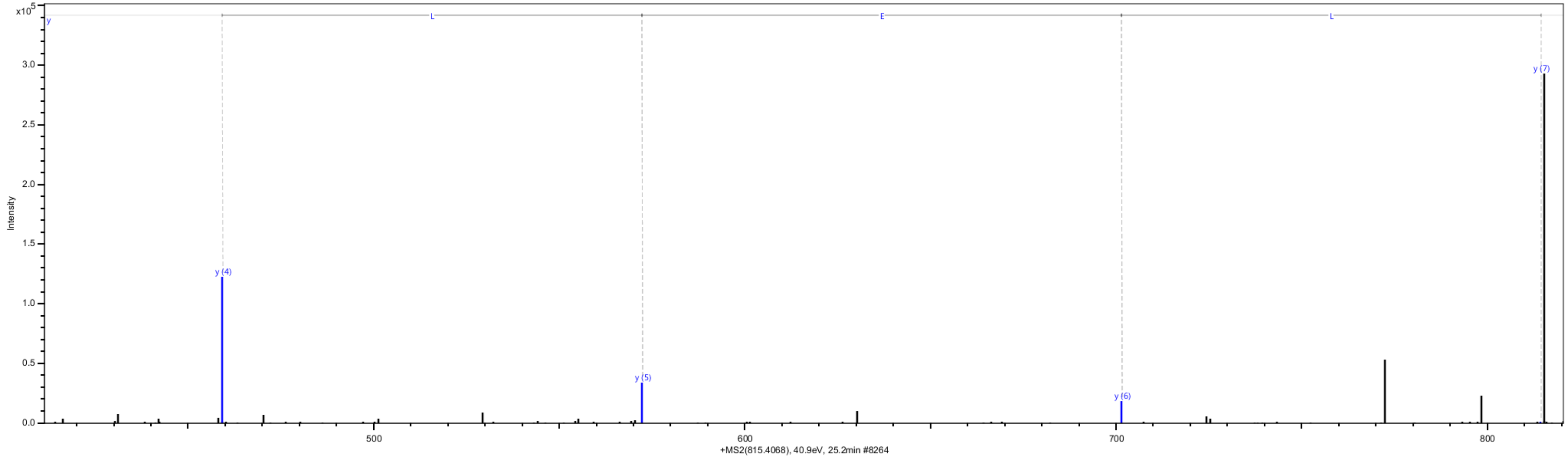

C1S_HUMAN

Accession: C1S_HUMAN
Database: SwissProt
MW [kDa] / pI: 76.6 / 4.8
Score: 23.9
Seq. Coverage [%]: 1.2
No. of Peptides: 1

Peptide Match

Sequence: K.LLEVPEGR.T
Score: 23.8
Modifications:

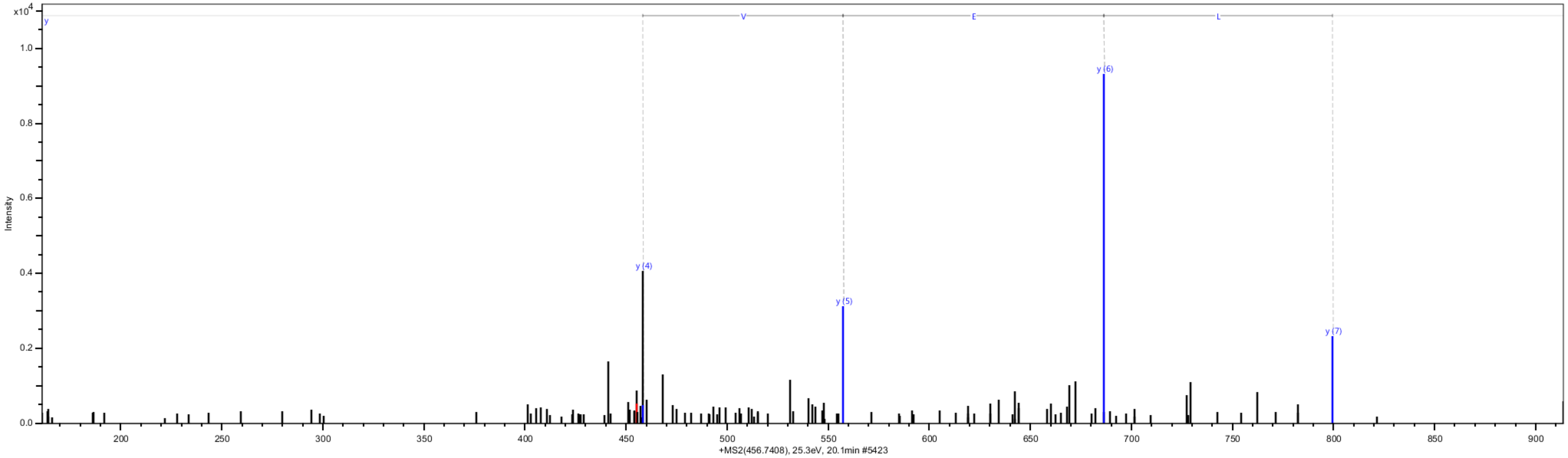

EFC4B_HUMAN

**Accession:**	EFC4B_HUMAN	**Score:**	20.2
**Database:**	SwissProt	**Seq. Coverage [%]:**	2.5
**MW [kDa] / pI:**	45.6 / 4.9	**No. of Peptides:**	1

Peptide Match

**Sequence:**	K.LKLTNQELAR.E	**Score:**	20.2
**Modifications:**			

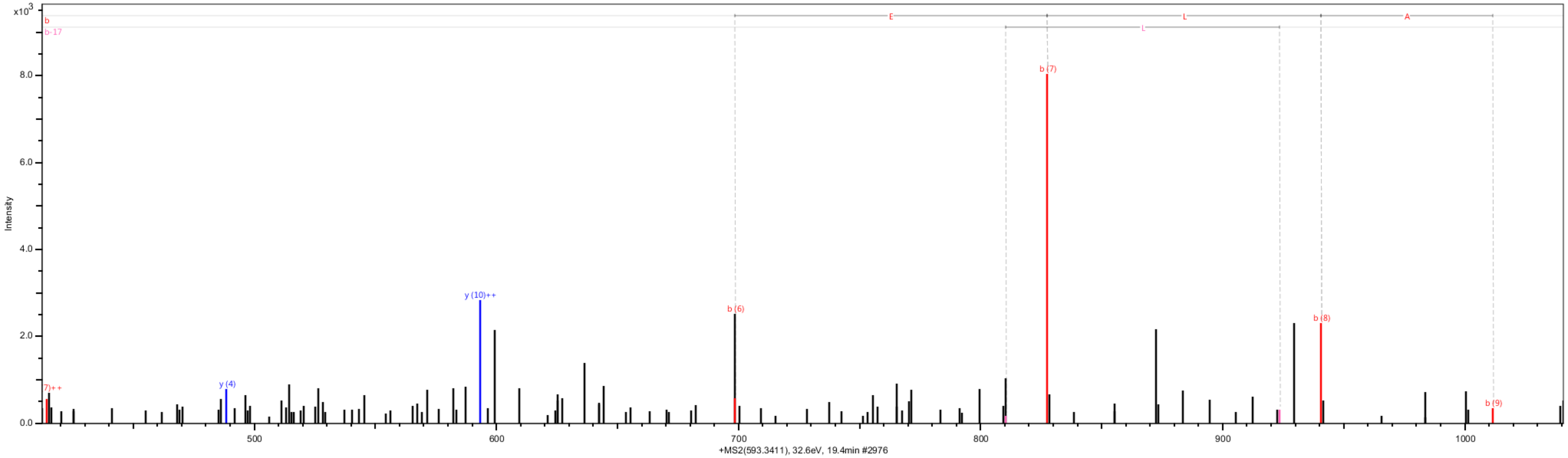

DNMT1_HUMAN

**Accession:**	DNMT1_HUMAN	**Score:**	20.2
**Database:**	SwissProt	**Seq. Coverage [%]:**	1.2
**MW [kDa] / pI:**	183.0 / 8.0	**No. of Peptides:**	1

Peptide Match

**Sequence:**	K.EADDDEEVDDNIPEMPSPKK.M	**Score:**	20.2
**Modifications:**			

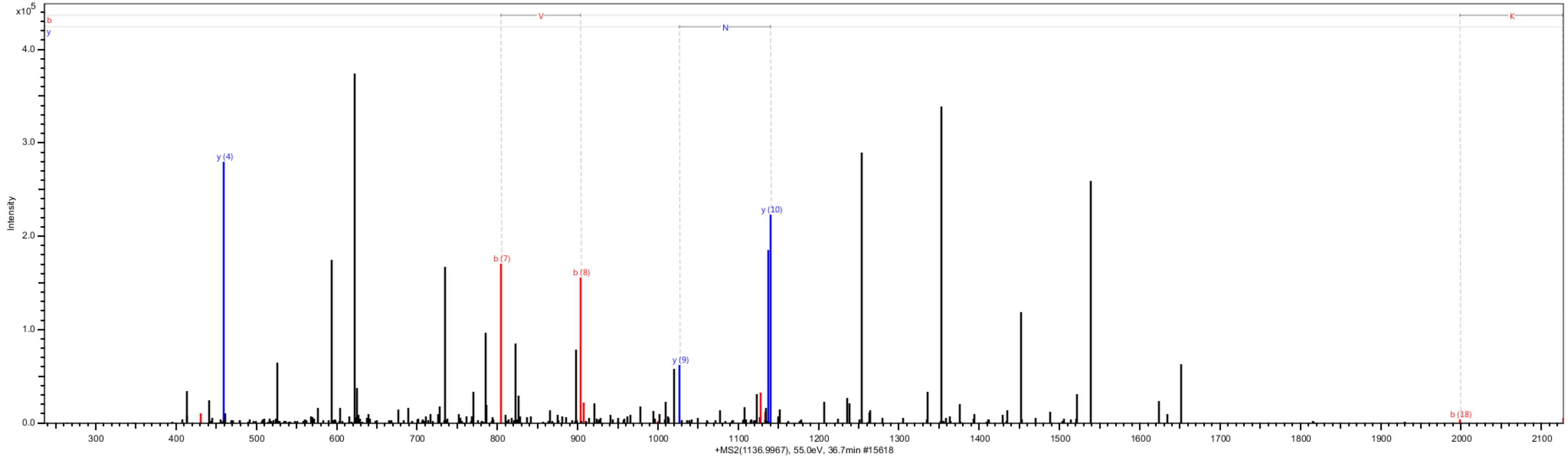

PHX2A_HUMAN

Accession: PHX2A_HUMAN
Database: SwissProt
MW [kDa] / pI: 29.6 / 9.0
Score: 20.2
Seq. Coverage [%]: 3.5
No. of Peptides: 1

Peptide Match

Sequence: K.GAAGAAGAKK.G
Score: 20.2
Modifications:

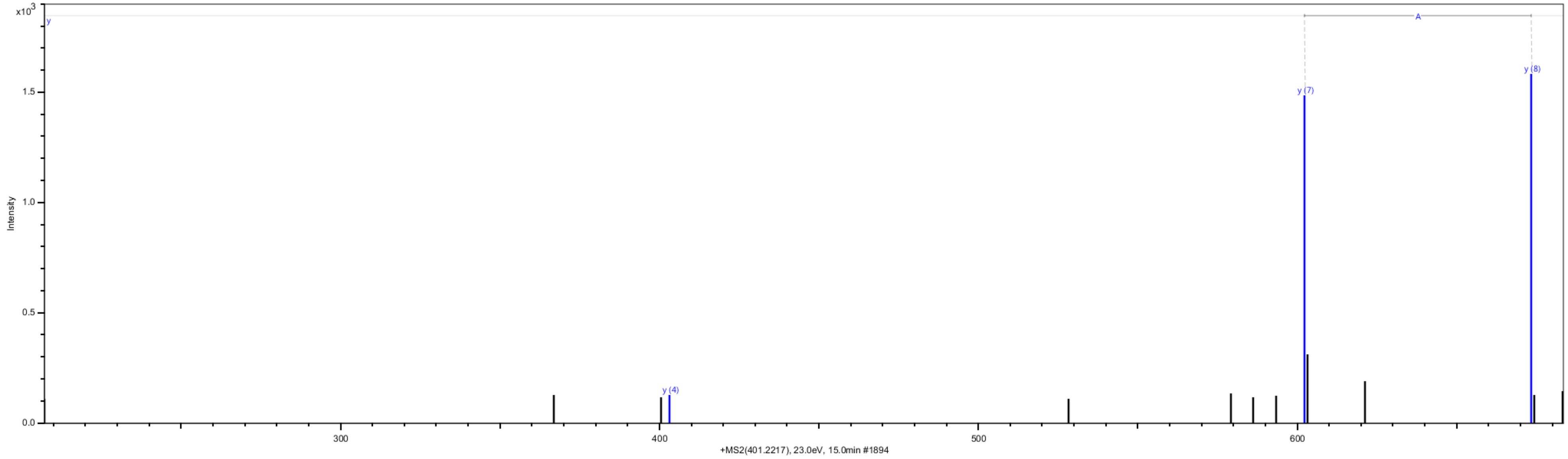

NU205_HUMAN

Accession: NU205_HUMAN **Score:** 20.0
Database: SwissProt **Seq. Coverage [%]:** 0.4
MW [kDa] / pI: 227.8 / 5.8 **No. of Peptides:** 1

Peptide Match

Sequence: R.YSFIQALVR.R **Score:** 20
Modifications:

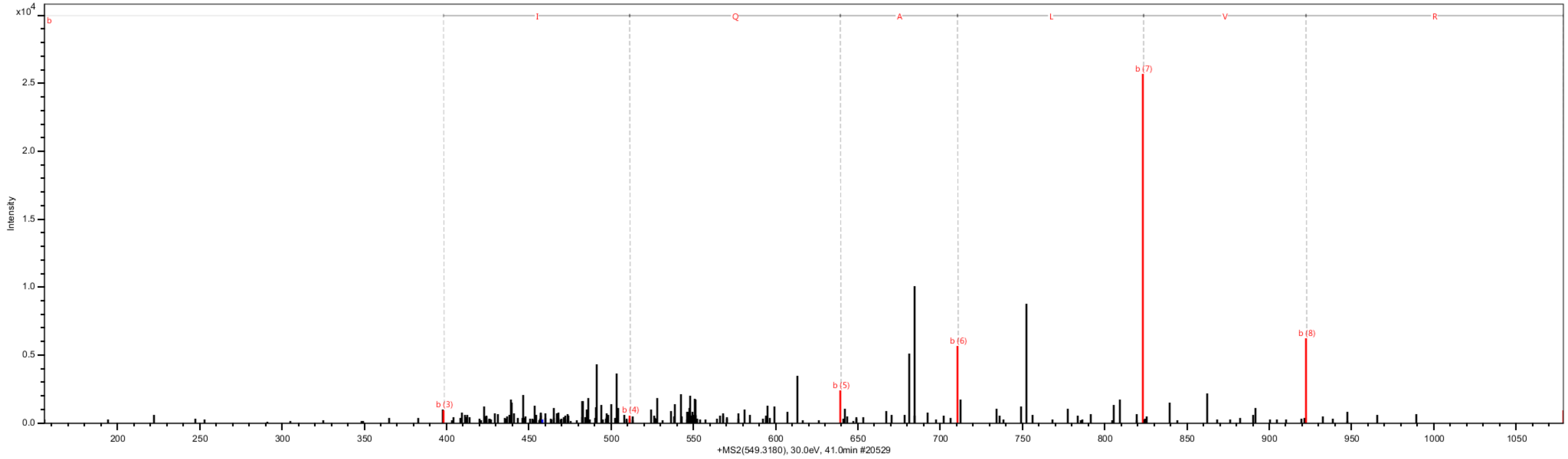

MFN2_HUMAN

Accession: MFN2_HUMAN
Database: SwissProt
MW [kDa] / pI: 86.3 / 6.5
Score: 20.0
Seq. Coverage [%]: 2.8
No. of Peptides: 1

Peptide Match

Sequence: R.LIMDSLHMAAREQQVYCEEMR.E
Score: 20
Modifications: Carbamidomethyl: 17; Oxidation: 3

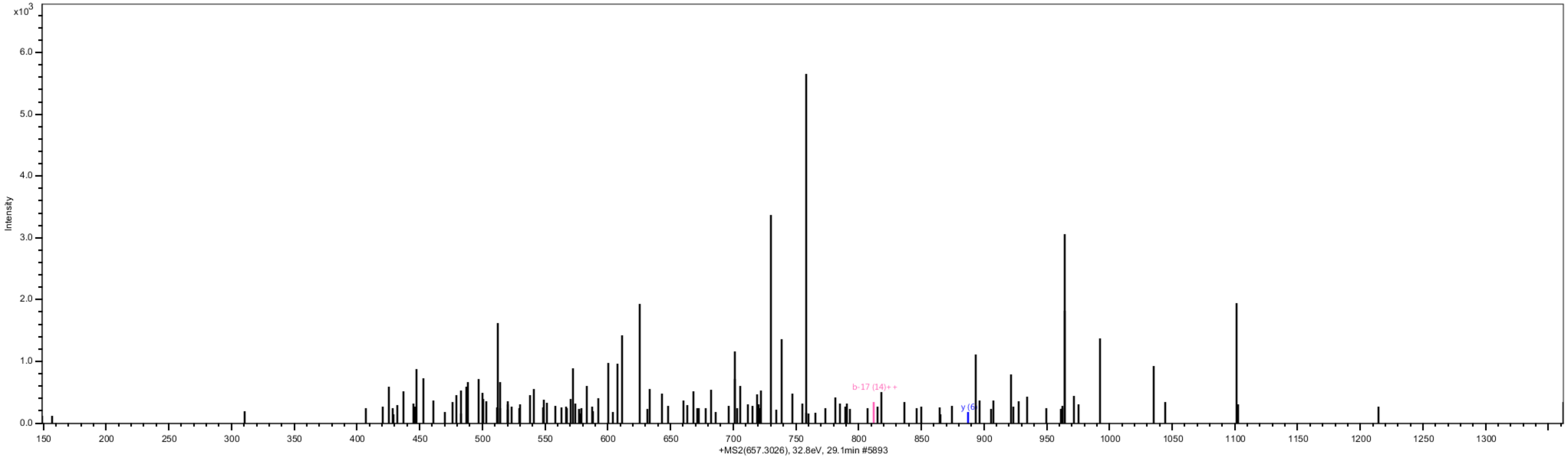

HEAT3_HUMAN

Accession: HEAT3_HUMAN
Database: SwissProt
MW [kDa] / pI: 74.5 / 5.0
Score: 32.2
Seq. Coverage [%]: 2.4
No. of Peptides: 1

Peptide Match

Sequence: K.NISQCMTPDQLMTLCK.A
Score: 15.3
Modifications: Carbamidomethyl: 5, 15; Oxidation: 12

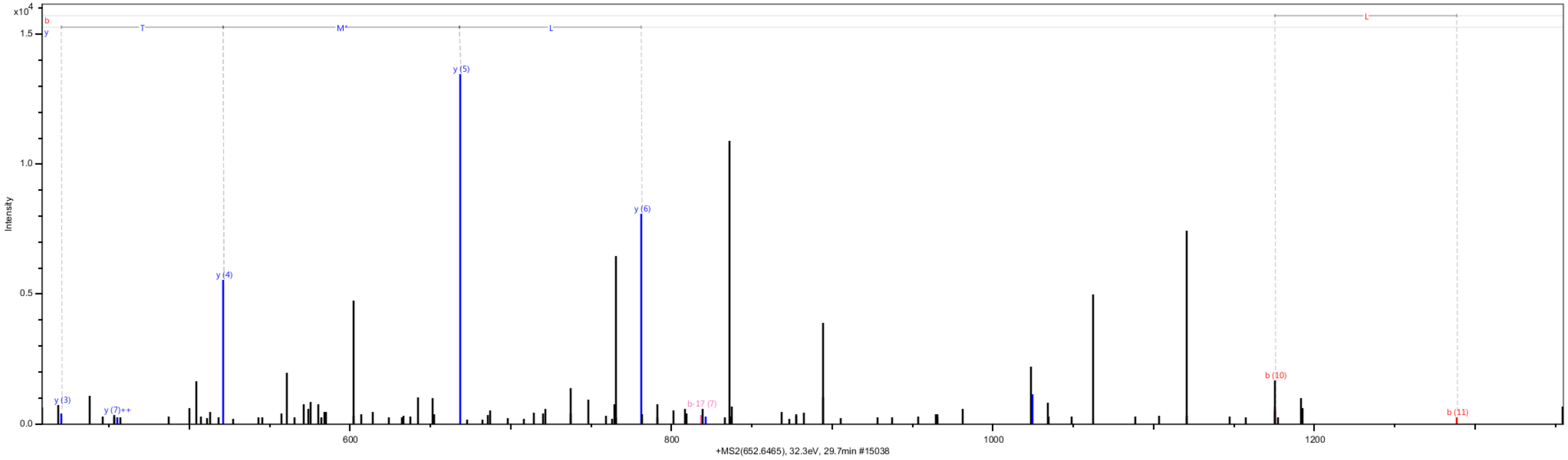

SCAFB_HUMAN

**Accession:**	SCAFB_HUMAN	**Score:**	32.7
**Database:**	SwissProt	**Seq. Coverage [%]:**	1.3
**MW [kDa] / pI:**	164.6 / 8.7	**No. of Peptides:**	2

Peptide Match

**Sequence:**	K.TVCTLNMGDKK.Y	**Score:**	16.6
**Modifications:**	Carbamidomethyl: 3; Oxidation: 7		

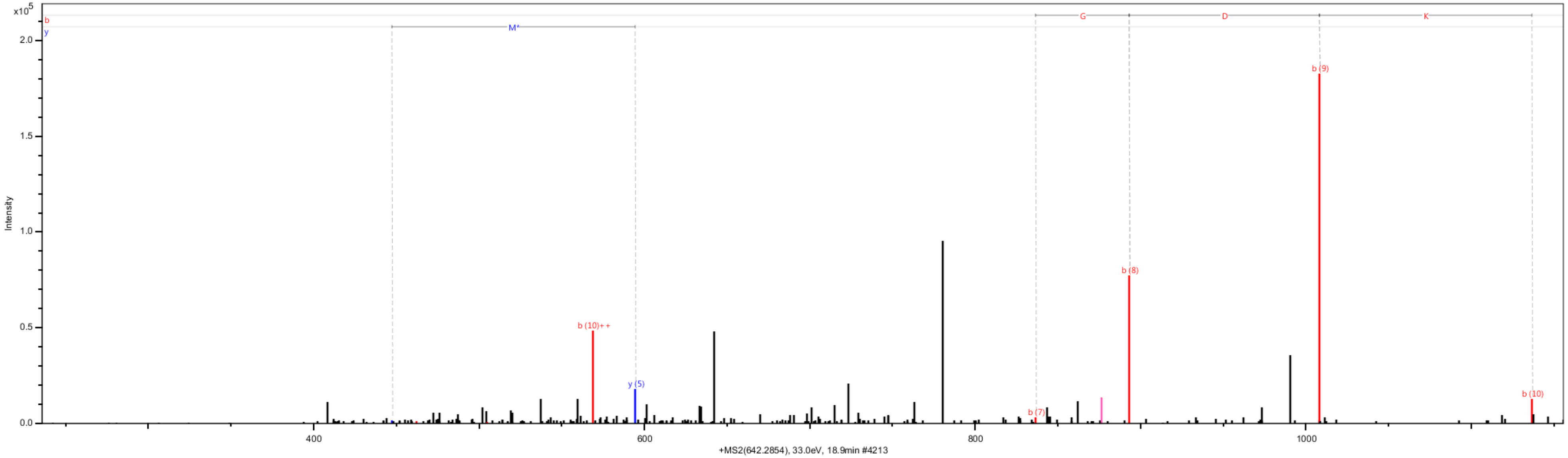

Peptide Match

**Sequence:**	R.SSVAAPEK.S	**Score:**	16.1
**Modifications:**			

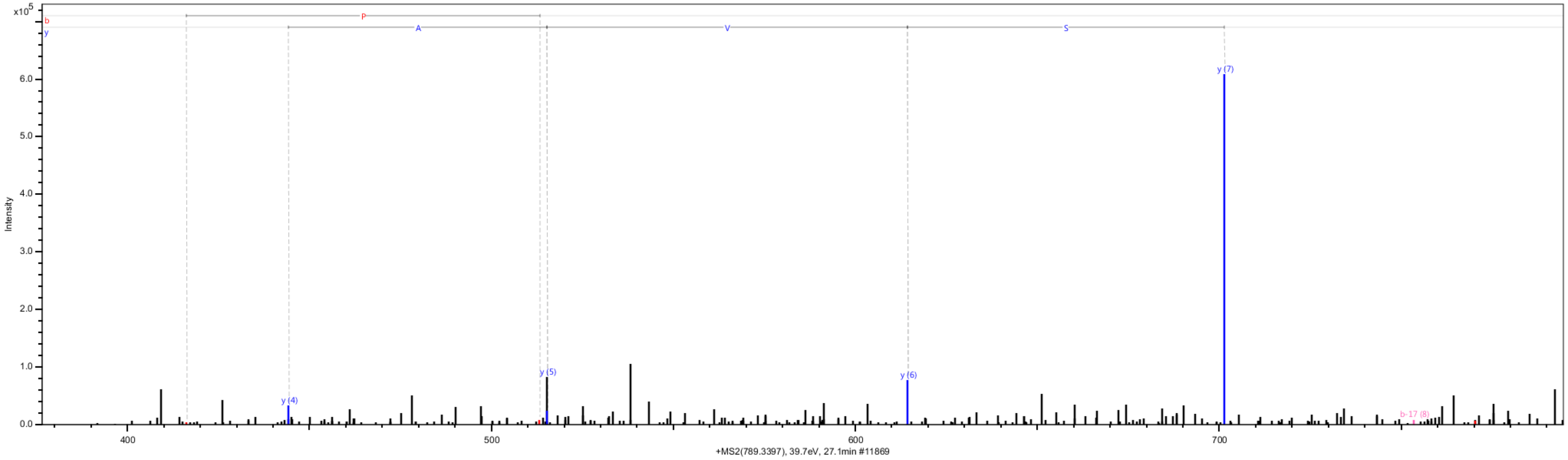
x10^5
b
y
P
A
V
S
y (7)
y (5)
y (6)
y (4)
b-17 (8)
Intensity
6.0
5.0
4.0
3.0
2.0
1.0
0.0
400
500
600
700
+MS2(789.3397), 39.7eV, 27.1min #11869

CE170_HUMAN

Accession: CE170_HUMAN
Database: SwissProt
MW [kDa] / pI: 175.2 / 6.6
Score: 32.7
Seq. Coverage [%]: 0.6
No. of Peptides: 1

Peptide Match

Sequence: K.FVQSSGRIR.Q
Score: 17.4
Modifications:

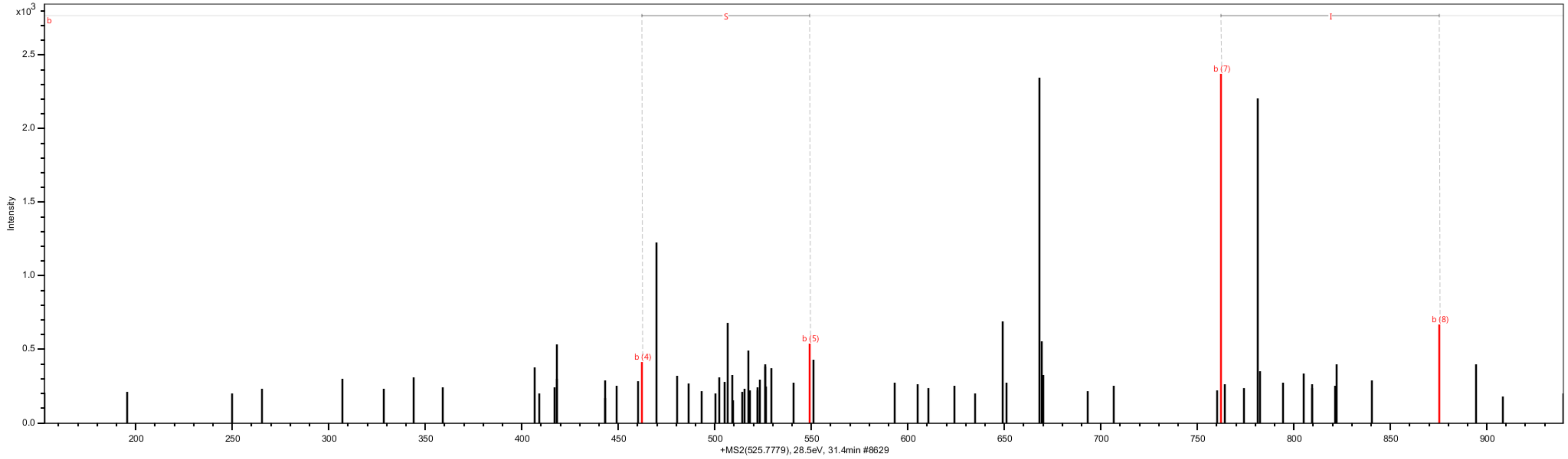

UN13A_HUMAN

**Accession:**	UN13A_HUMAN	**Score:**	32.4
**Database:**	SwissProt	**Seq. Coverage [%]:**	0.5
**MW [kDa] / pI:**	192.9 / 5.2	**No. of Peptides:**	1

Peptide Match

**Sequence:**	R.EDVAVAEPK.D	**Score:**	16.7
**Modifications:**			

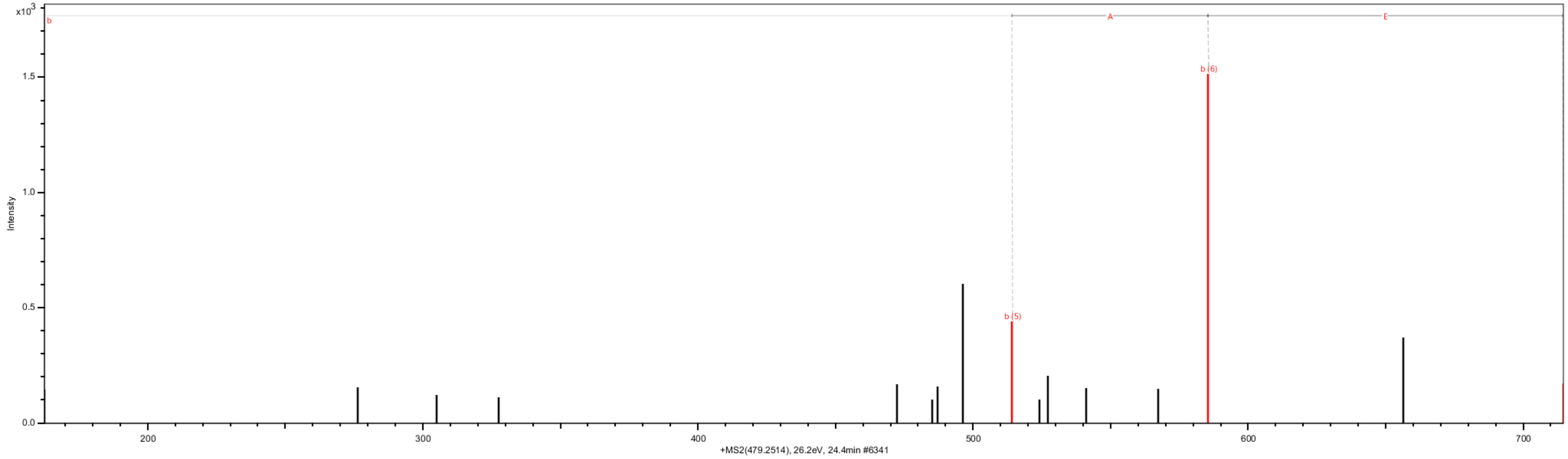

PPR21_HUMAN

Accession: PPR21_HUMAN
Database: SwissProt
MW [kDa] / pI: 88.3 / 6.4
Score: 32.4
Seq. Coverage [%]: 2.1
No. of Peptides: 2

Peptide Match

Sequence: K.VPFNDTK.Y
Score: 15.4
Modifications:

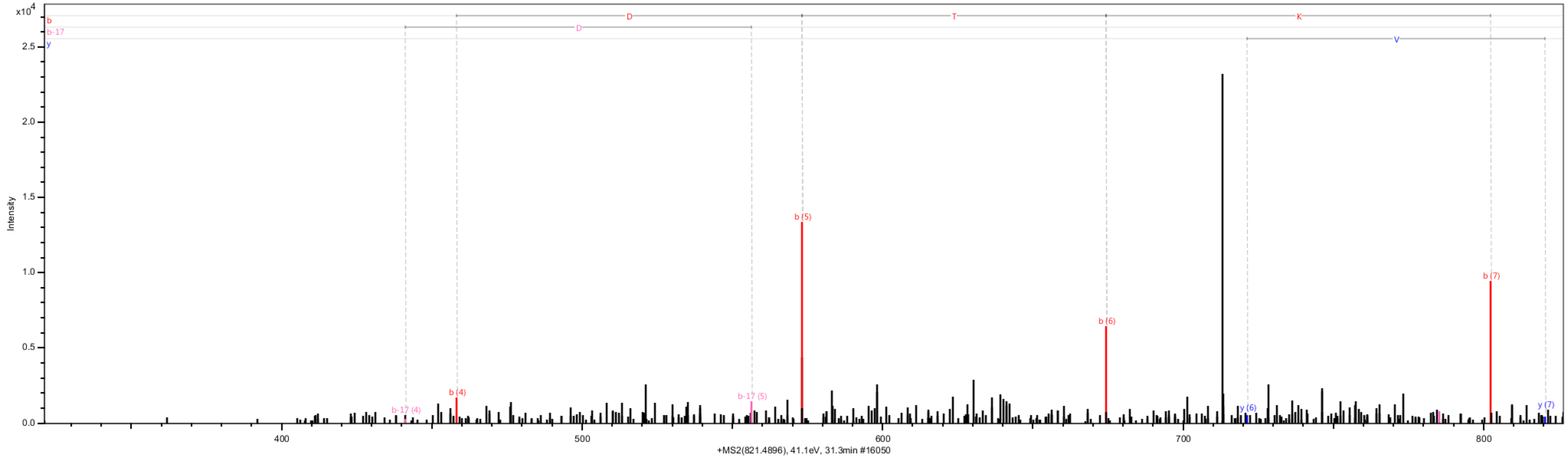

Peptide Match

Sequence: R.ILLSSTESR.E
Score: 17
Modifications:

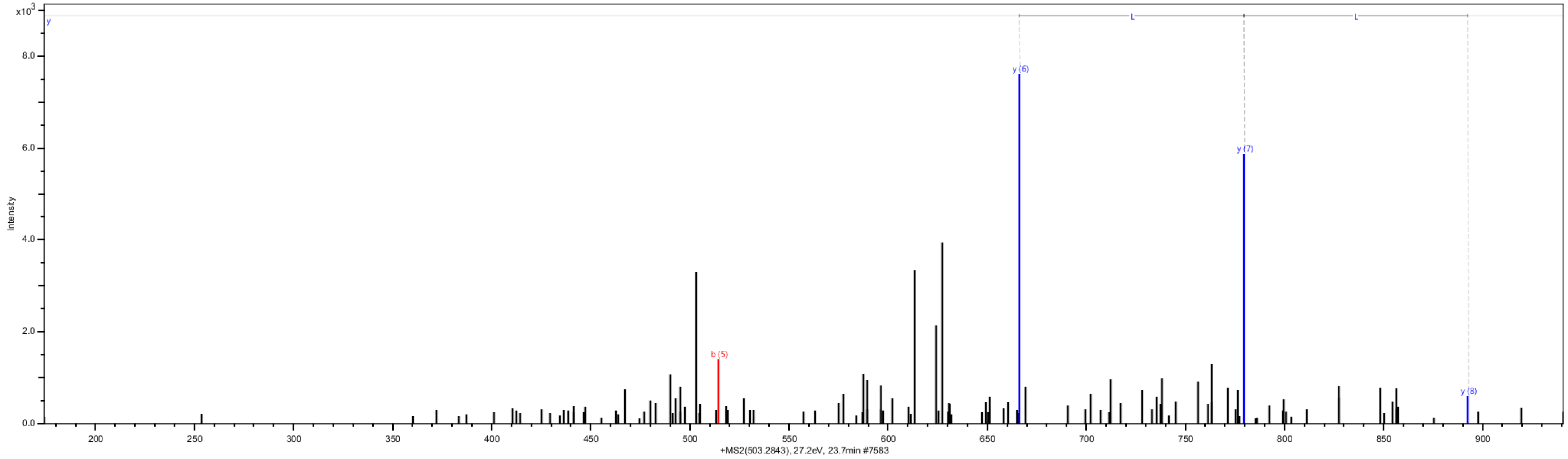
x10^3
y
L
L
y (6)
y (7)
b (5)
y (8)
Intensity
8.0
6.0
4.0
2.0
0.0
200
250
300
350
400
450
500
550
600
650
700
750
800
850
900
+MS2(503.2843), 27.2eV, 23.7min #7583

BICD1_HUMAN

**Accession:**	BICD1_HUMAN	**Score:**	32.3
**Database:**	SwissProt	**Seq. Coverage [%]:**	1.1
**MW [kDa] / pI:**	110.7 / 5.6	**No. of Peptides:**	1

Peptide Match

**Sequence:**	K.DKEALMEEILK.L	**Score:**	17.2
**Modifications:**			

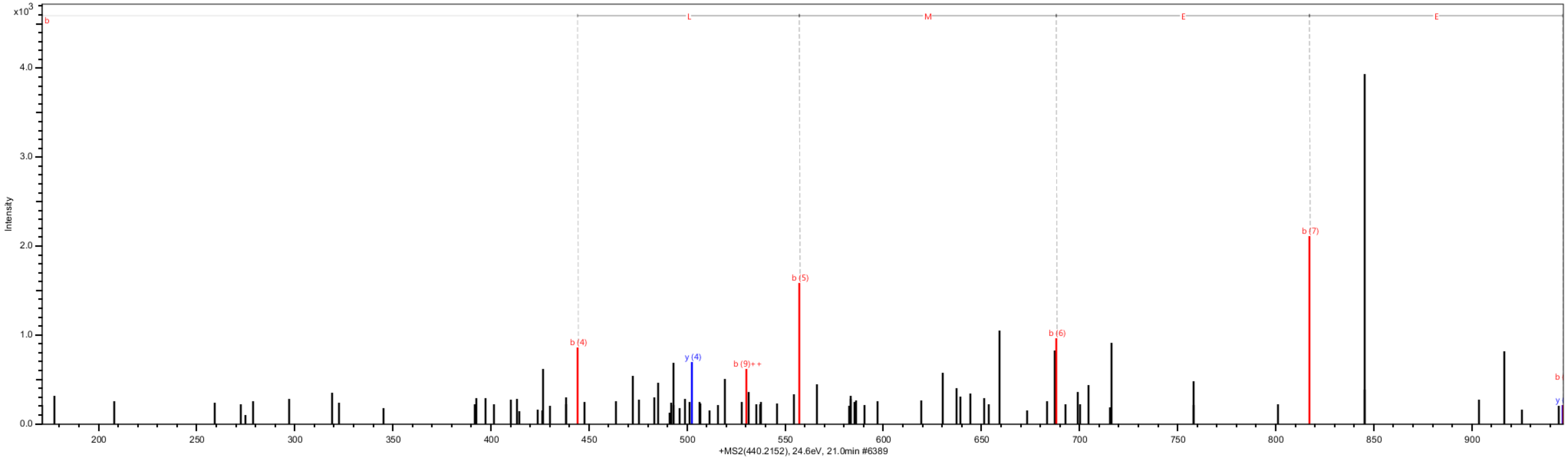

CC186_HUMAN

**Accession:**	CC186_HUMAN	**Score:**	32.1
**Database:**	SwissProt	**Seq. Coverage [%]:**	1.4
**MW [kDa] / pI:**	103.6 / 5.9	**No. of Peptides:**	1

Peptide Match

**Sequence:**	K.EEVQTLQAELACR.Q	**Score:**	16
**Modifications:**	Carbamidomethyl: 12		

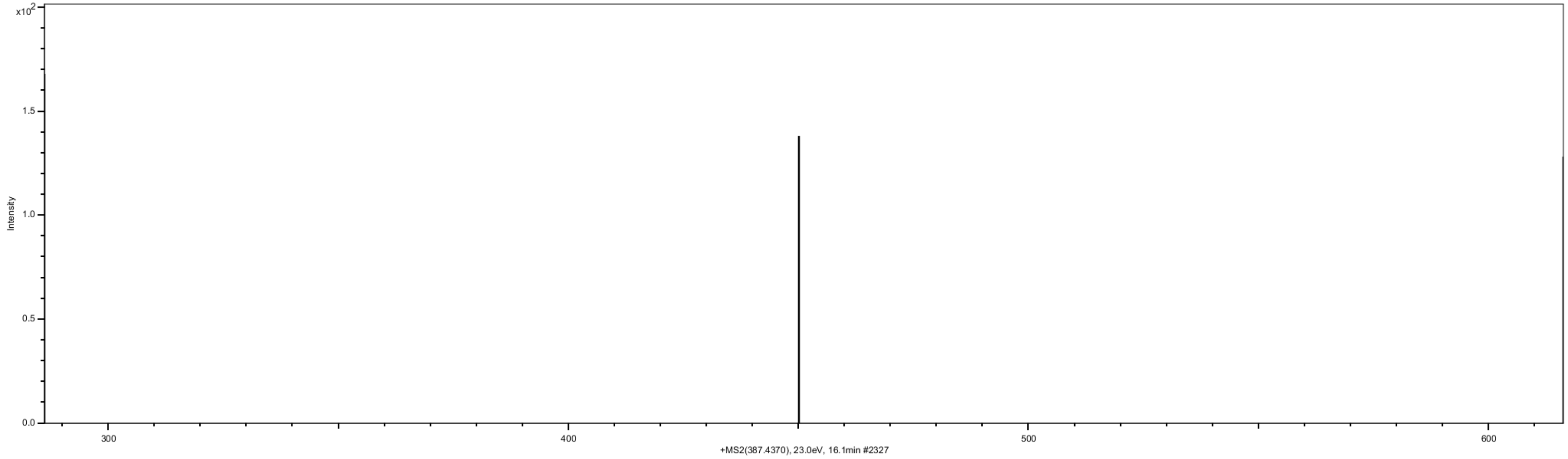

KCNA4_HUMAN

**Accession:**	KCNA4_HUMAN	**Score:**	21.0
**Database:**	SwissProt	**Seq. Coverage [%]:**	2.3
**MW [kDa] / pI:**	73.2 / 5.1	**No. of Peptides:**	1

Peptide Match

**Sequence:**	K.TLAQFPETLLGDPEK.R	**Score:**	21
**Modifications:**			

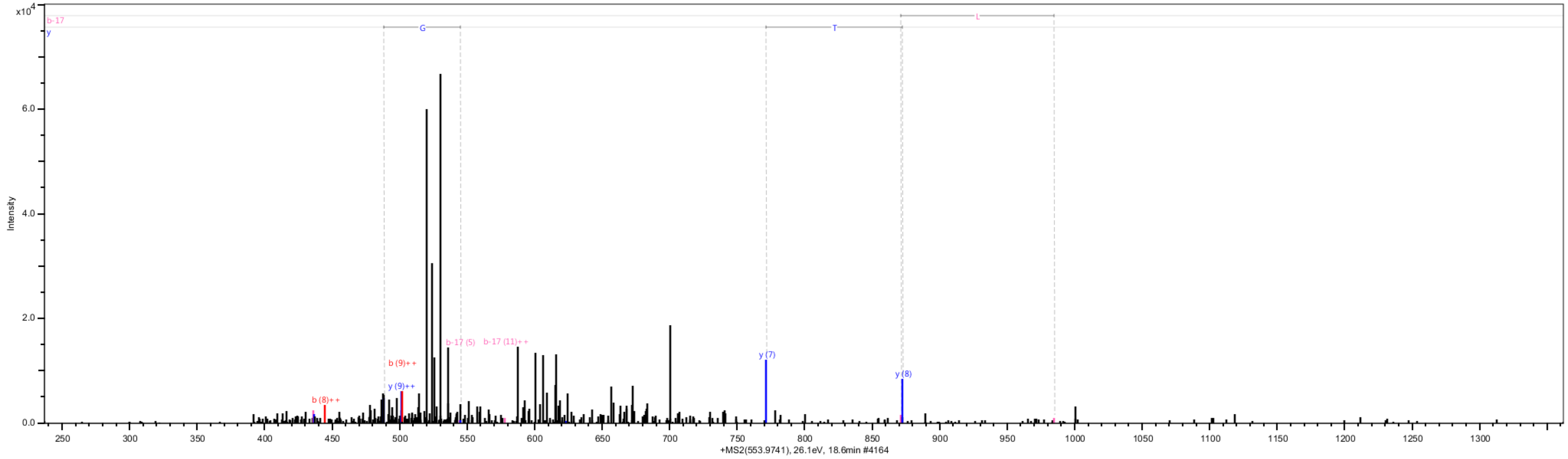

CBPC5_HUMAN

**Accession:**	CBPC5_HUMAN	**Score:**	21.0
**Database:**	SwissProt	**Seq. Coverage [%]:**	1.2
**MW [kDa] / pI:**	97.5 / 9.3	**No. of Peptides:**	1

Peptide Match

**Sequence:**	K.LYSQGMAPFVR.T	**Score:**	21
**Modifications:**	Oxidation: 6		

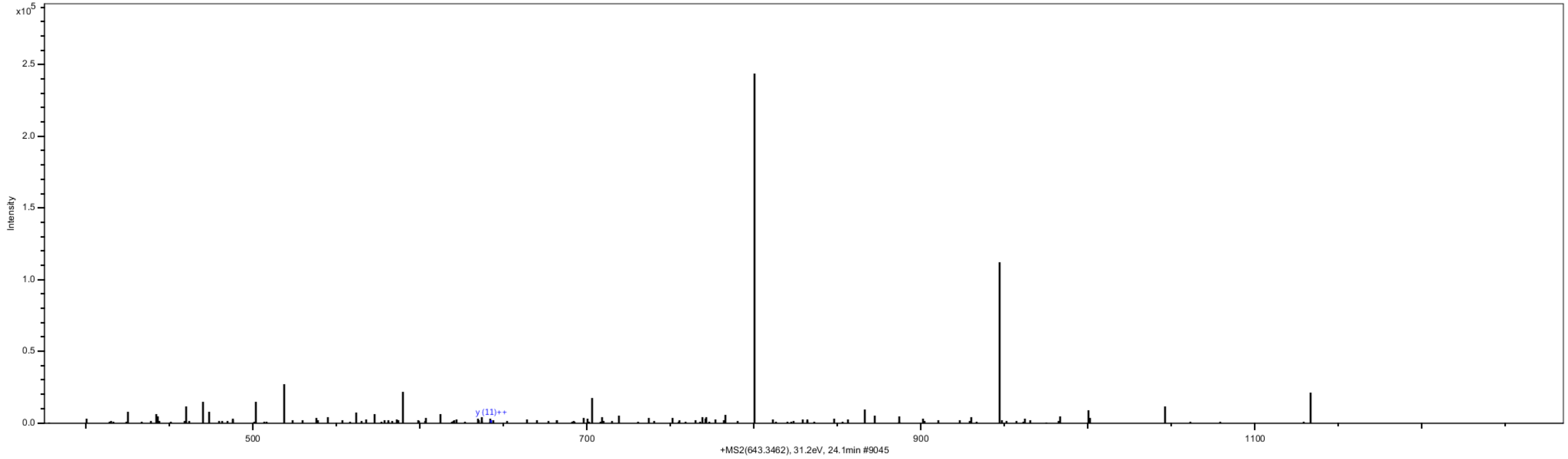

ZN624_HUMAN

**Accession:**	ZN624_HUMAN	**Score:**	20.9
**Database:**	SwissProt	**Seq. Coverage [%]:**	1.2
**MW [kDa] / pI:**	99.9 / 9.1	**No. of Peptides:**	1

Peptide Match

**Sequence:**	R.SNSSLIVHIR.T	**Score:**	20.9
**Modifications:**			

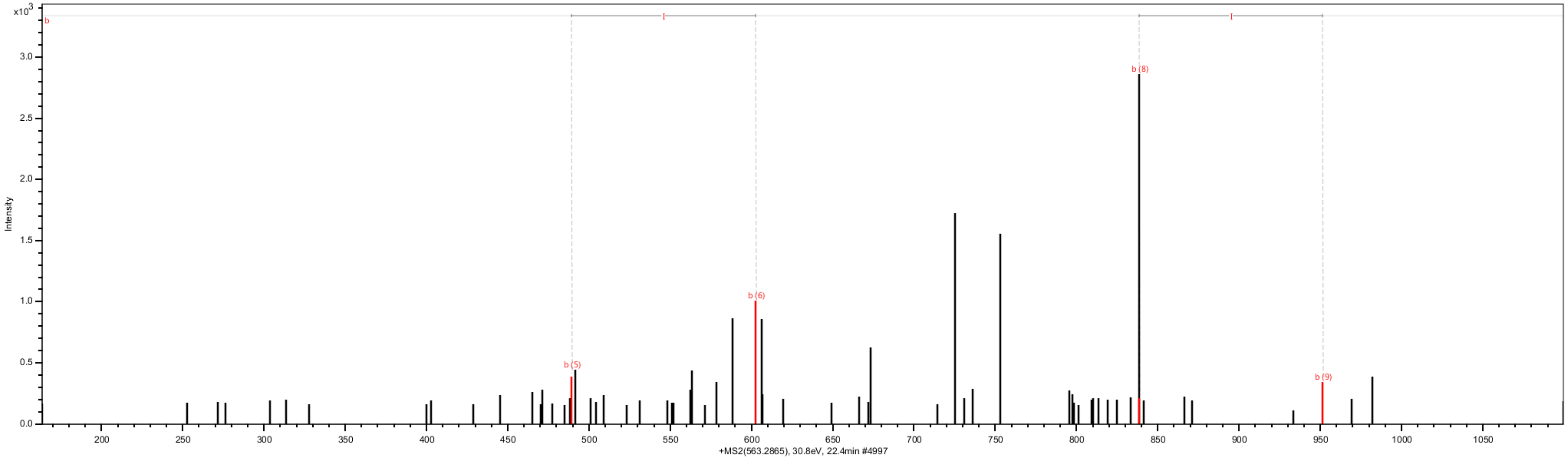

FURIN_HUMAN

Accession: FURIN_HUMAN
Database: SwissProt
MW [kDa] / pI: 86.6 / 6.0
Score: 20.9
Seq. Coverage [%]: 1.0
No. of Peptides: 1

Peptide Match

Sequence: K.QIVTTDLR.Q
Score: 20.9
Modifications:

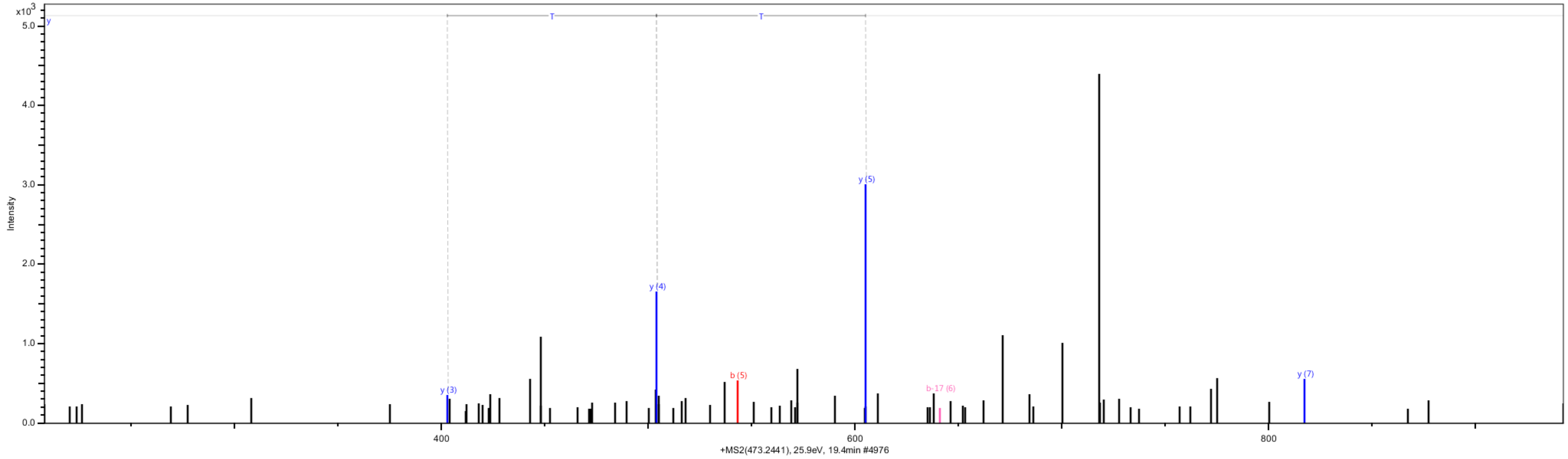

FETUB_HUMAN

**Accession:**	FETUB_HUMAN	**Score:**	25.4
**Database:**	SwissProt	**Seq. Coverage [%]:**	2.1
**MW [kDa] / pI:**	42.0 / 6.5	**No. of Peptides:**	1

Peptide Match

**Sequence:**	K.LVVLPFPK.E	**Score:**	25.4
**Modifications:**			

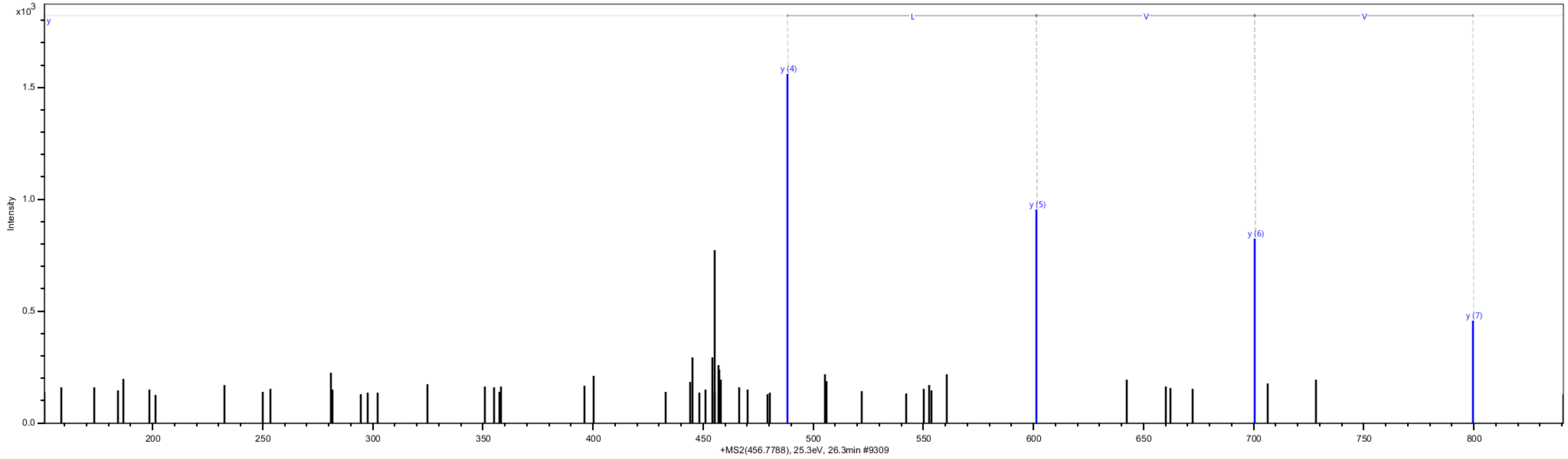

CO9_HUMAN

**Accession:**	CO9_HUMAN	**Score:**	25.4
**Database:**	SwissProt	**Seq. Coverage [%]:**	2.0
**MW [kDa] / pI:**	63.1 / 5.4	**No. of Peptides:**	1

Peptide Match

**Sequence:**	K.LSPIYNLVPVK.M	**Score:**	25.4
**Modifications:**			

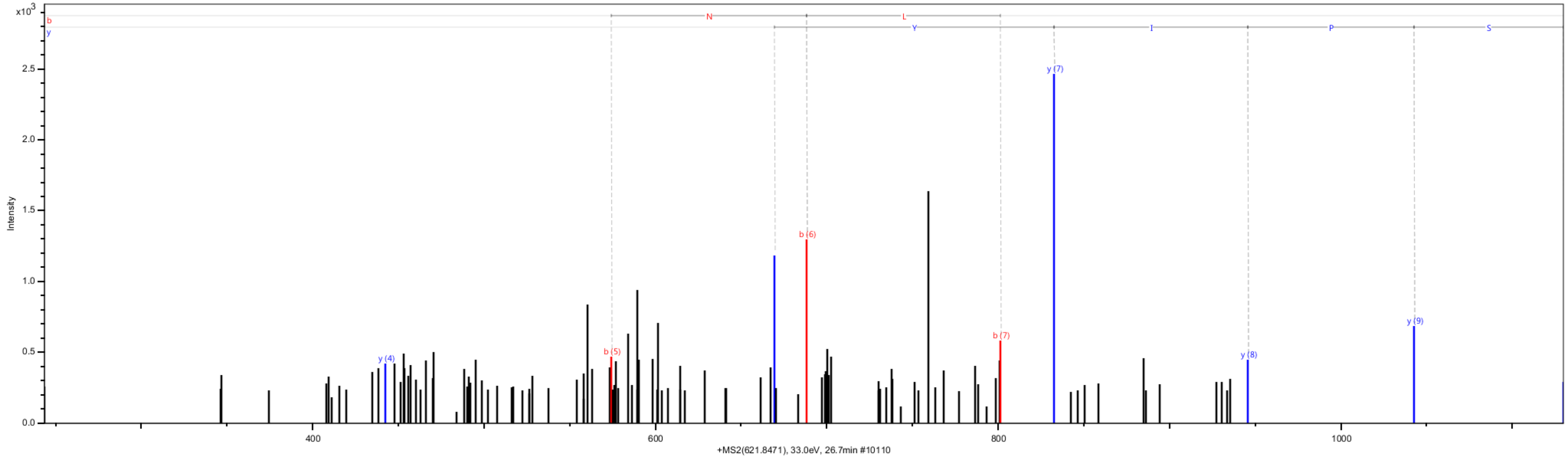

PCH2_HUMAN

Accession: PCH2_HUMAN
Database: SwissProt
MW [kDa] / pI: 48.5 / 5.7

Score: 25.1
Seq. Coverage [%]: 3.9
No. of Peptides: 1

Peptide Match

Sequence: R.YRYGQLIEINSHSLFSK.W
Score: 25.1
Modifications:

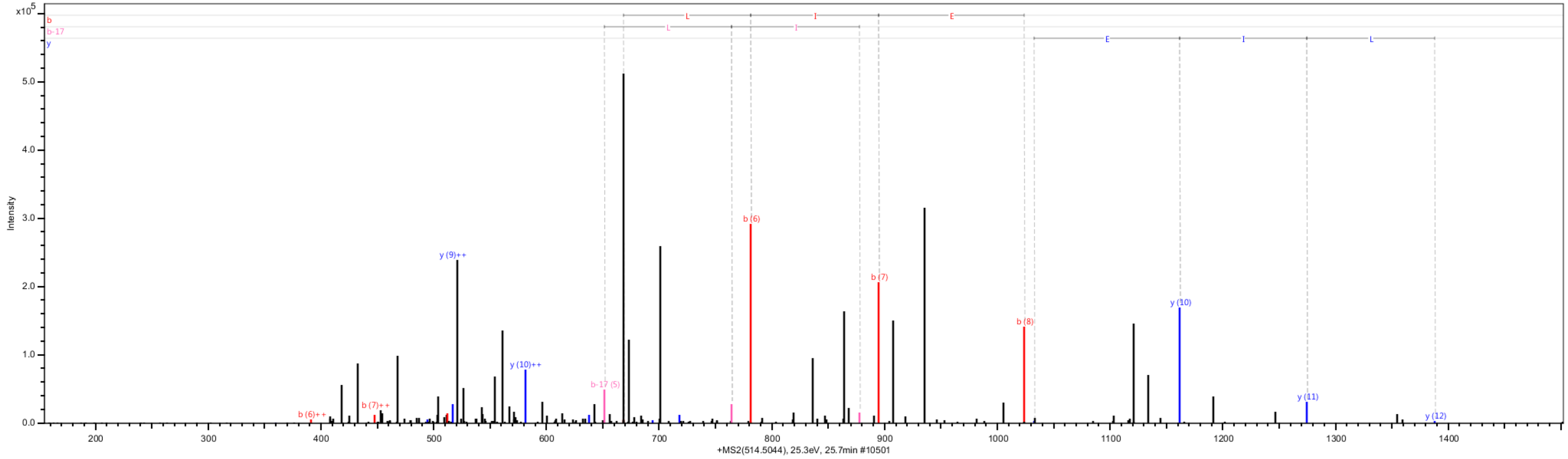

RBM19_HUMAN

Accession: RBM19_HUMAN
Database: SwissProt
MW [kDa] / pI: 107.3 / 6.1
Score: 25.1
Seq. Coverage [%]: 1.0
No. of Peptides: 1

Peptide Match

Sequence: K.NVPTTKGAPK.N
Score: 25.1
Modifications:

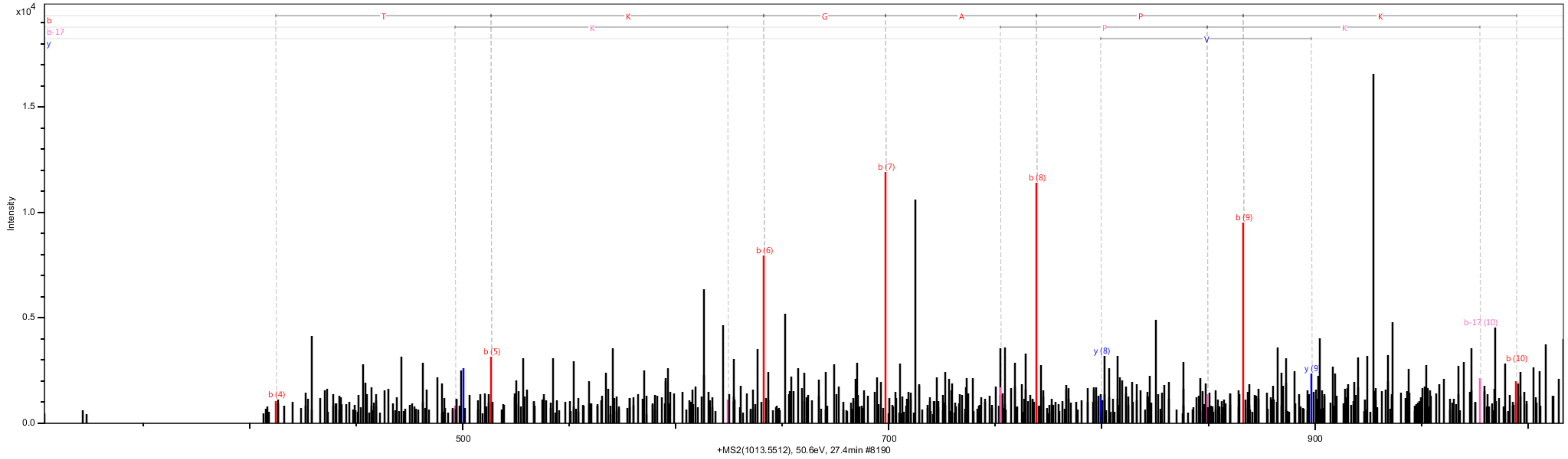

MYO1H_HUMAN

**Accession:**	MYO1H_HUMAN	**Score:**	25.0
**Database:**	SwissProt	**Seq. Coverage [%]:**	1.0
**MW [kDa] / pI:**	119.0 / 9.2	**No. of Peptides:**	1

Peptide Match

**Sequence:**	K.NGQLTVVSVR.R	**Score:**	25
**Modifications:**			

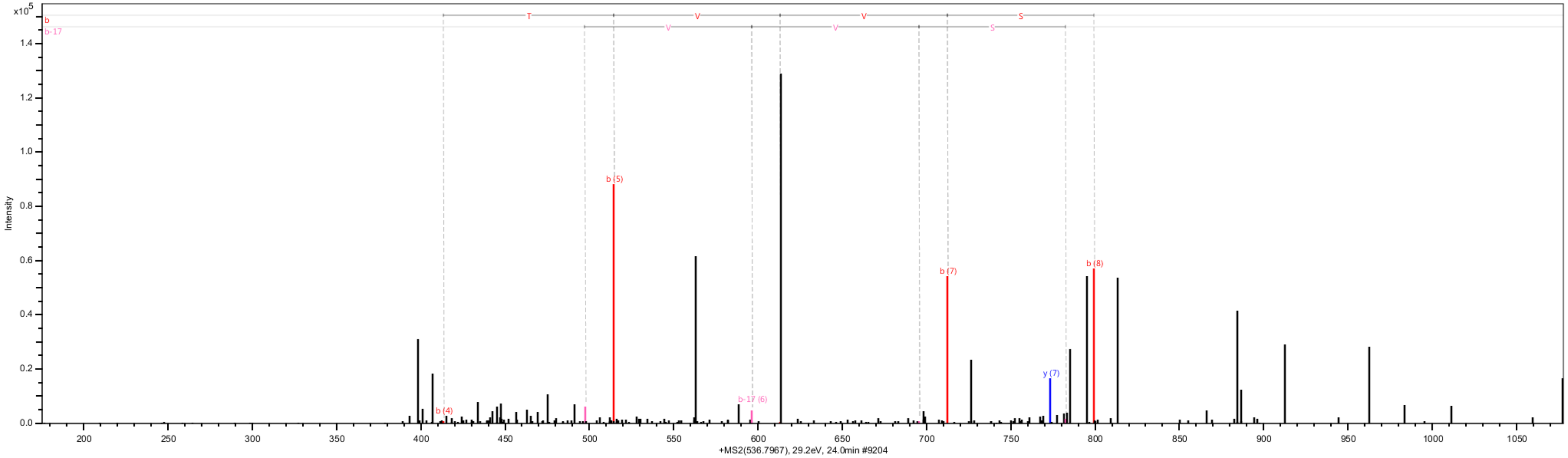

ZRAB3_HUMAN

Accession: ZRAB3_HUMAN
Database: SwissProt
MW [kDa] / pI: 123.2 / 8.7
Score: 24.9
Seq. Coverage [%]: 1.0
No. of Peptides: 1

Peptide Match

Sequence: K.QTAIAKAGAVK.D
Score: 24.9
Modifications:

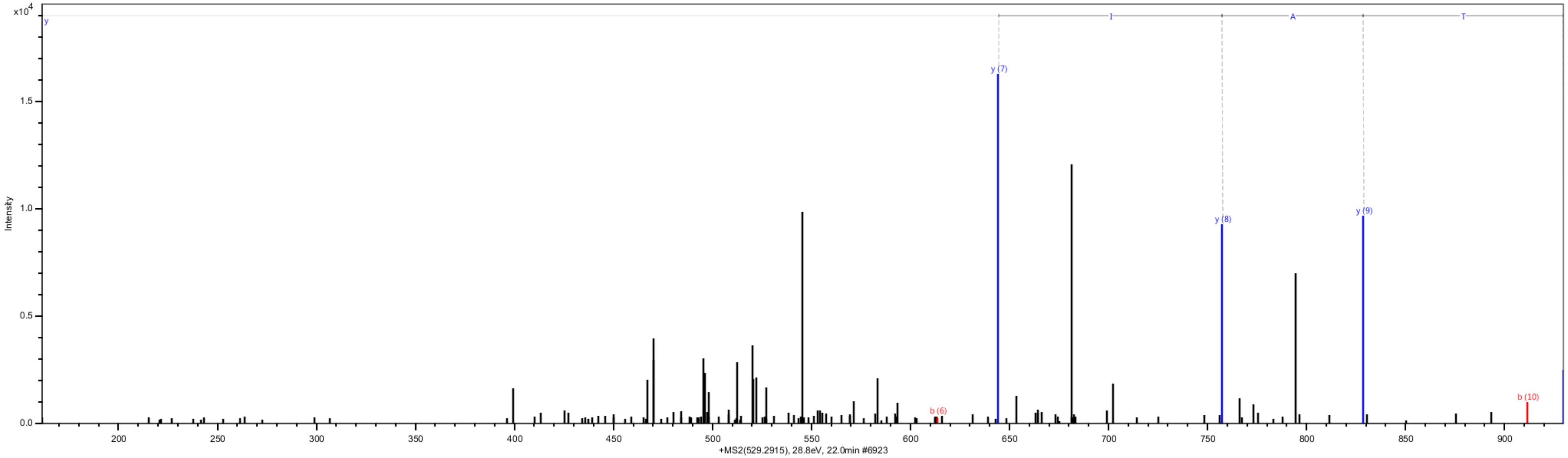

ACADV_HUMAN

**Accession:**	ACADV_HUMAN	**Score:**	24.9
**Database:**	SwissProt	**Seq. Coverage [%]:**	1.8
**MW [kDa] / pI:**	70.3 / 8.9	**No. of Peptides:**	1

Peptide Match

**Sequence:**	R.FGMAAALAGTMR.G	**Score:**	24.9
**Modifications:**	Oxidation: 3, 11		

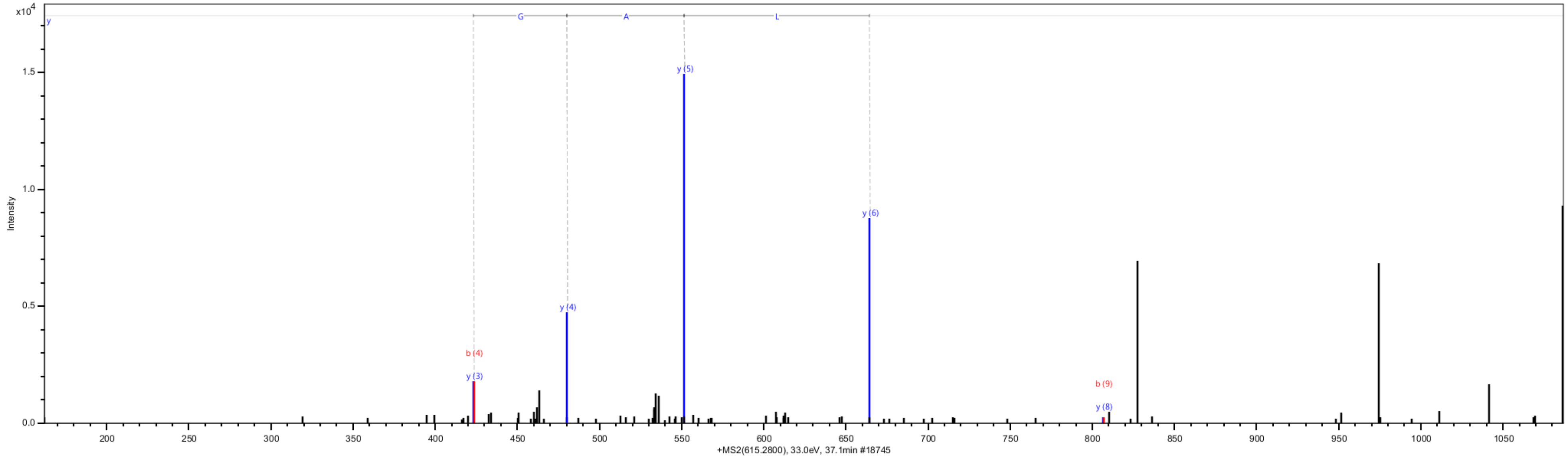

NOS2_HUMAN

Accession: NOS2_HUMAN
Database: SwissProt
MW [kDa] / pI: 131.0 / 8.2
Score: 24.7
Seq. Coverage [%]: 1.1
No. of Peptides: 1

Peptide Match

Sequence: R.MGLETHKLASLWK.D
Score: 24.7
Modifications:

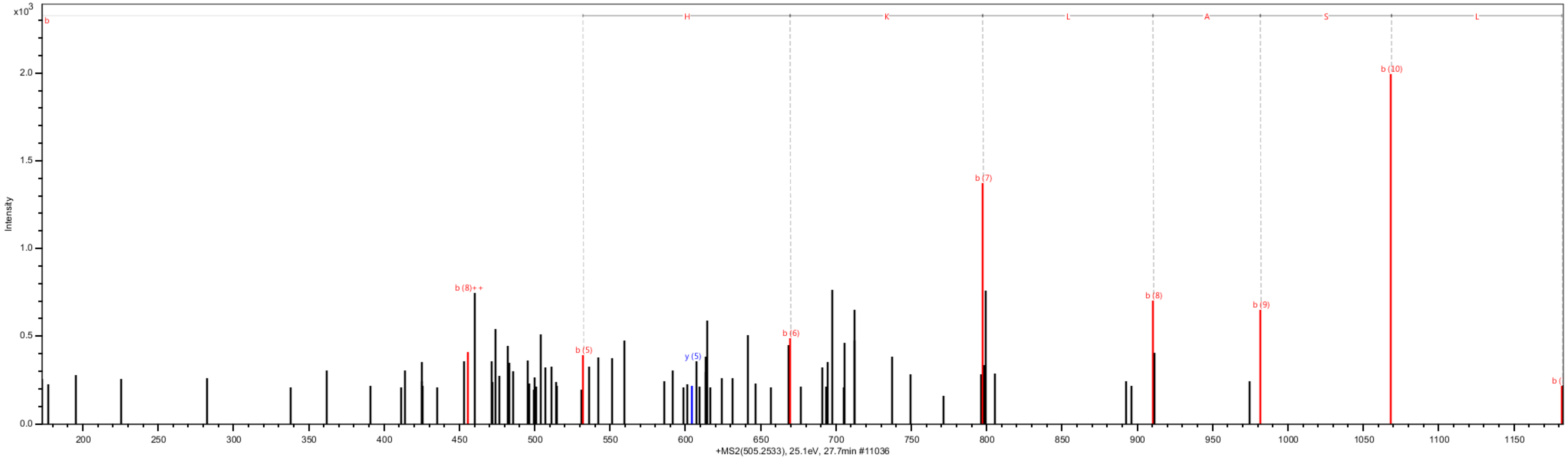

GLYL1_HUMAN

Accession: GLYL1_HUMAN
Database: SwissProt
MW [kDa] / pI: 35.1 / 6.4

Score: 24.7
Seq. Coverage [%]: 3.3
No. of Peptides: 1

Peptide Match

Sequence: K.EPQKSEEVLK.N
Score: 24.7
Modifications:

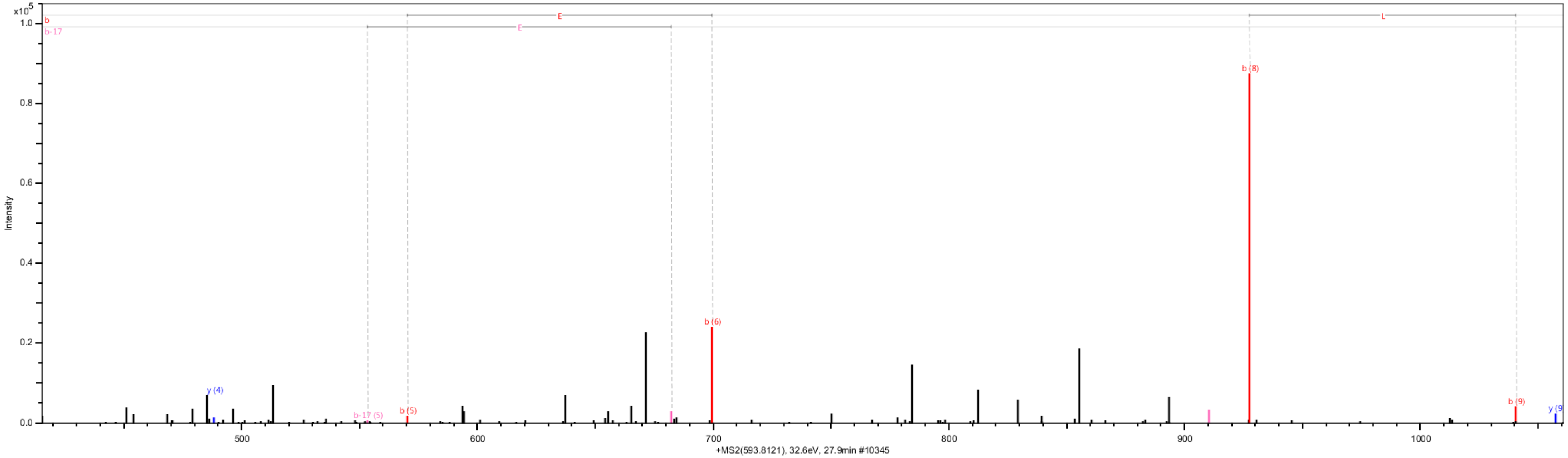

MGDP1_HUMAN

**Accession:**	MGDP1_HUMAN	**Score:**	16.2
**Database:**	SwissProt	**Seq. Coverage [%]:**	5.7
**MW [kDa] / pI:**	20.1 / 6.0	**No. of Peptides:**	1

Peptide Match

**Sequence:**	R.LYPEVPEVLK.R	**Score:**	16.2
**Modifications:**			

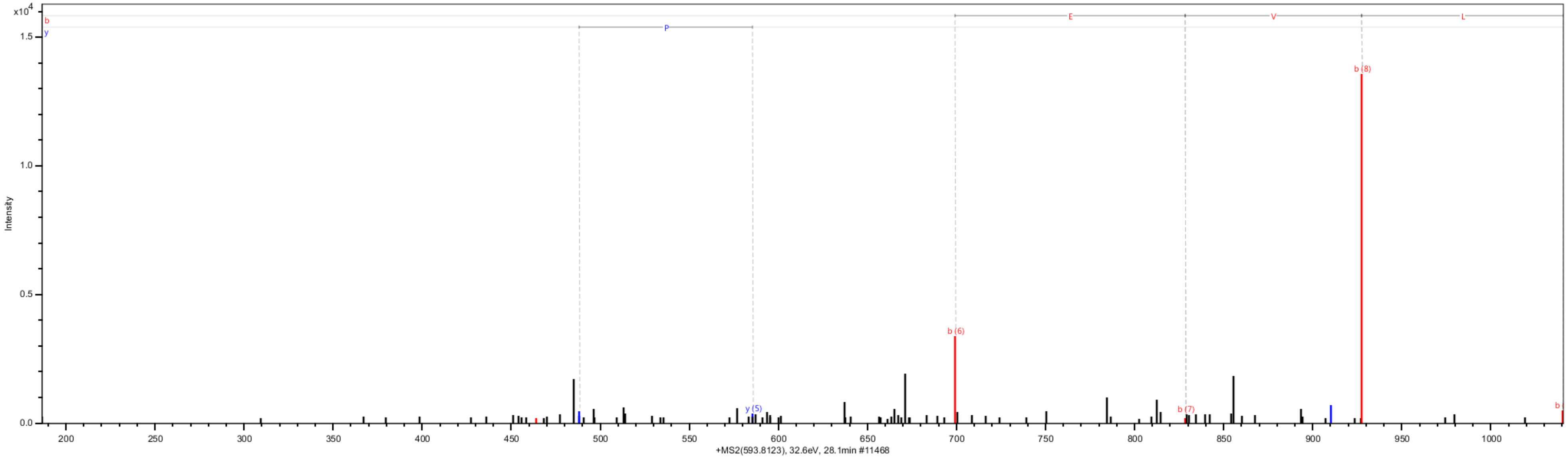

HS74L_HUMAN

Accession: HS74L_HUMAN
Database: SwissProt
MW [kDa] / pI: 94.5 / 5.6
Score: 17.2
Seq. Coverage [%]: 1.2
No. of Peptides: 1

Peptide Match

Sequence: K.AVMEQANLQR.E
Modifications: Oxidation: 3
Score: 17.2

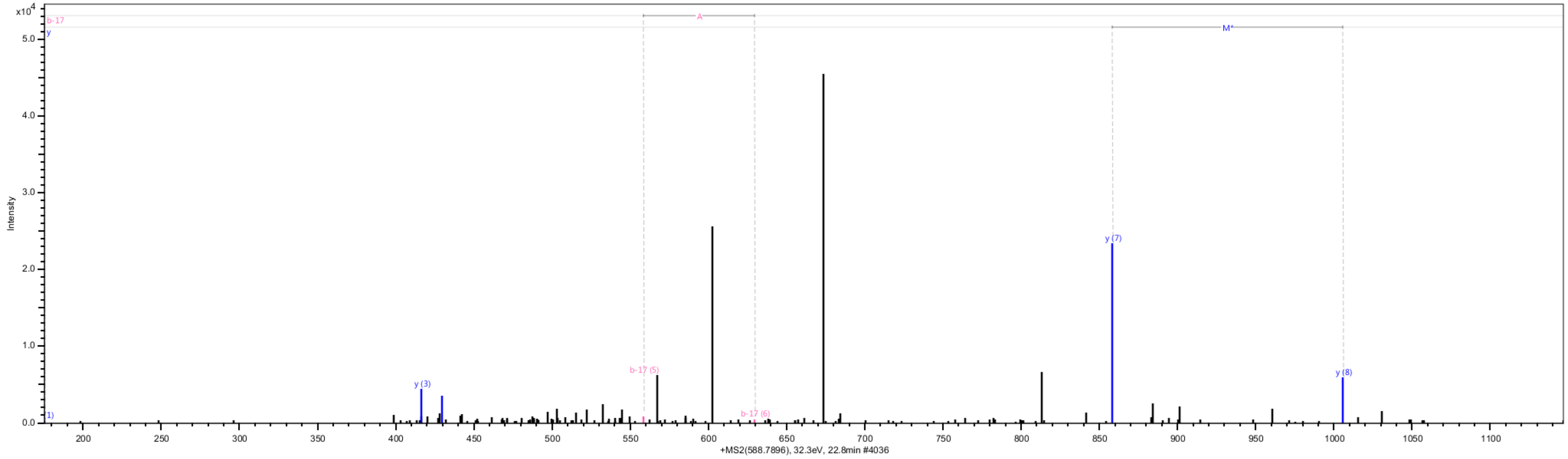

HIPK2_HUMAN

Accession: HIPK2_HUMAN
Database: SwissProt
MW [kDa] / pI: 130.9 / 8.7
Score: 17.1
Seq. Coverage [%]: 0.9
No. of Peptides: 1

Peptide Match

Sequence: K.HAPTSTVSKQR.K
Score: 17.1
Modifications:

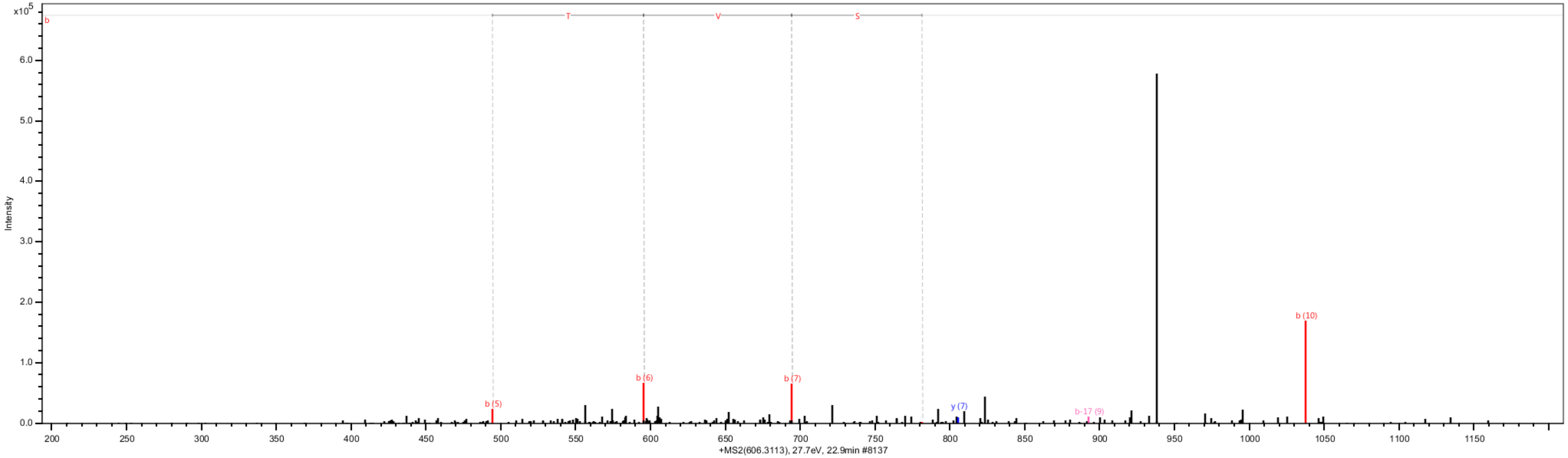

R3GEF_HUMAN

**Accession:**	R3GEF_HUMAN	**Score:**	17.1
**Database:**	SwissProt	**Seq. Coverage [%]:**	2.4
**MW [kDa] / pI:**	42.6 / 6.1	**No. of Peptides:**	1

Peptide Match

**Sequence:**	R.LRSSSMEIR.E	**Score:**	17.1
**Modifications:**	Oxidation: 6		

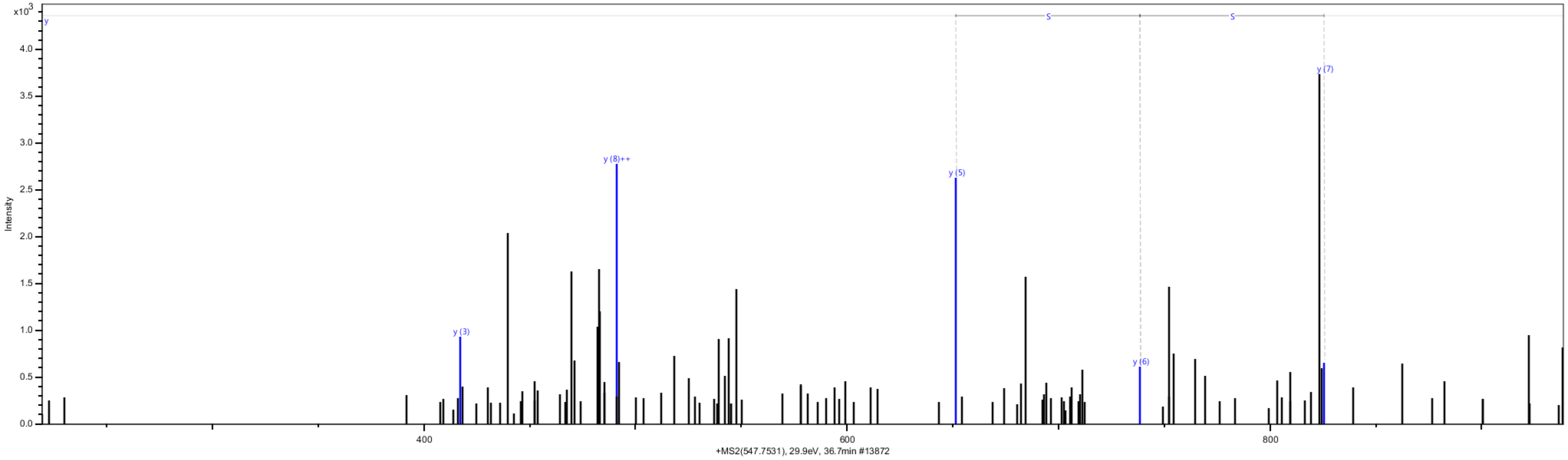

RNF26_HUMAN

**Accession:**	RNF26_HUMAN	**Score:**	17.1
**Database:**	SwissProt	**Seq. Coverage [%]:**	1.8
**MW [kDa] / pI:**	47.7 / 8.9	**No. of Peptides:**	1

Peptide Match

**Sequence:**	R.TIRVTPVR.G	**Score:**	17.1
**Modifications:**			

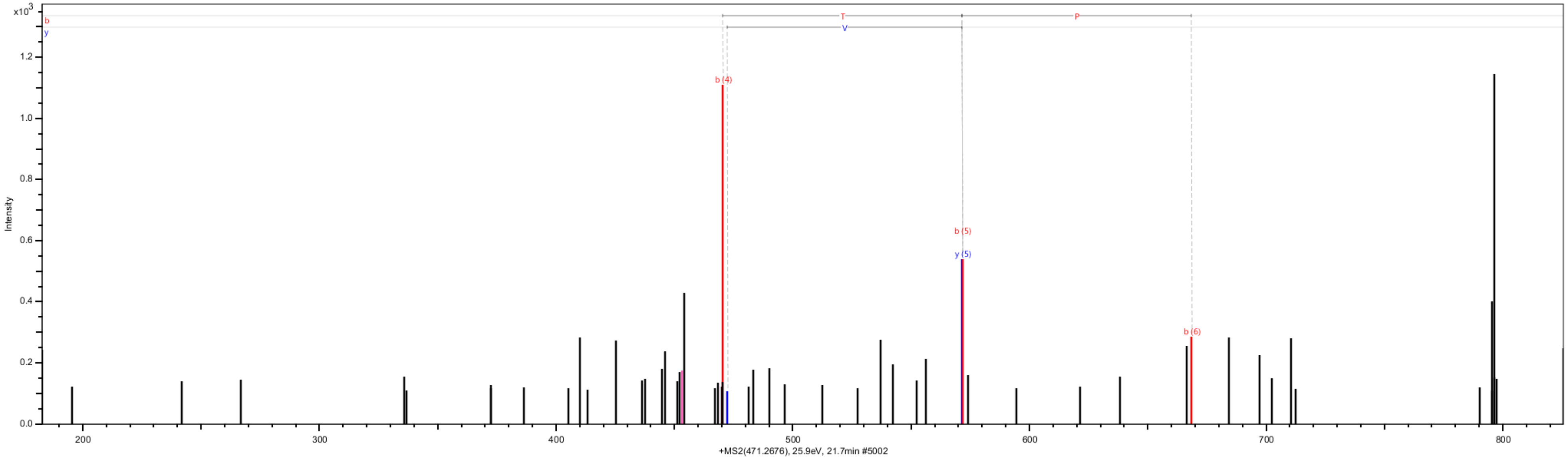

CBPC1_HUMAN

Accession: CBPC1_HUMAN
Database: SwissProt
MW [kDa] / pI: 138.4 / 5.8
Score: 17.1
Seq. Coverage [%]: 1.3
No. of Peptides: 1

Peptide Match

Sequence: K.GSTGMEILLSTLENTK.D
Score: 17
Modifications:

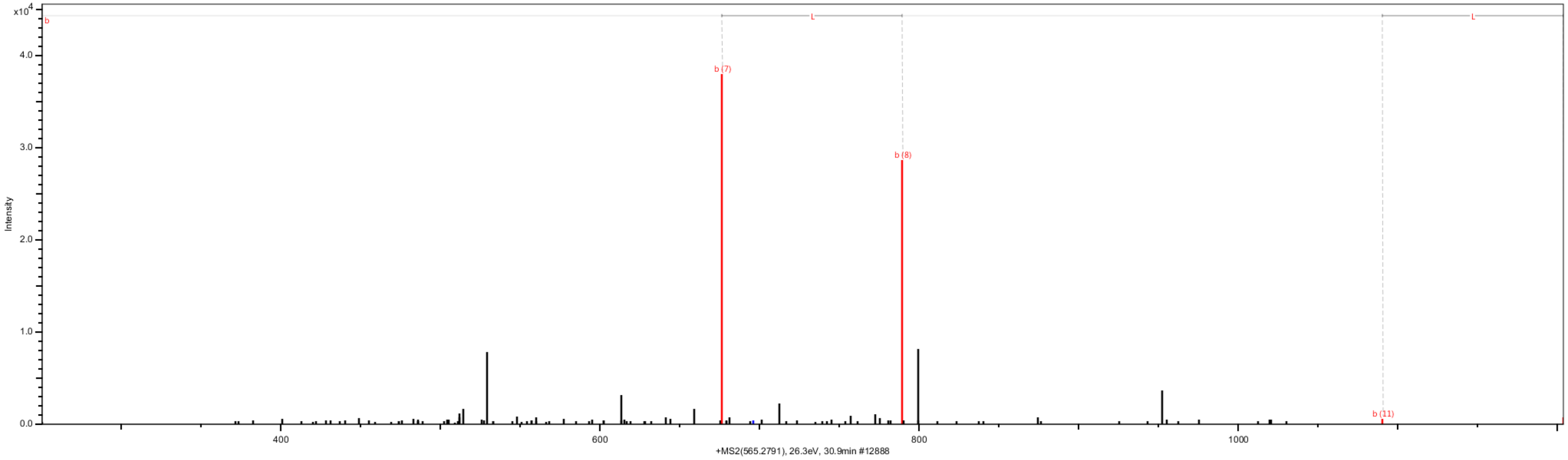

SH3R3_HUMAN

Accession: SH3R3_HUMAN
Database: SwissProt
MW [kDa] / pI: 92.7 / 9.1
Score: 17.0
Seq. Coverage [%]: 1.0
No. of Peptides: 1

Peptide Match

Sequence: K.GEMYRVLEK.C
Score: 17
Modifications: Oxidation: 3

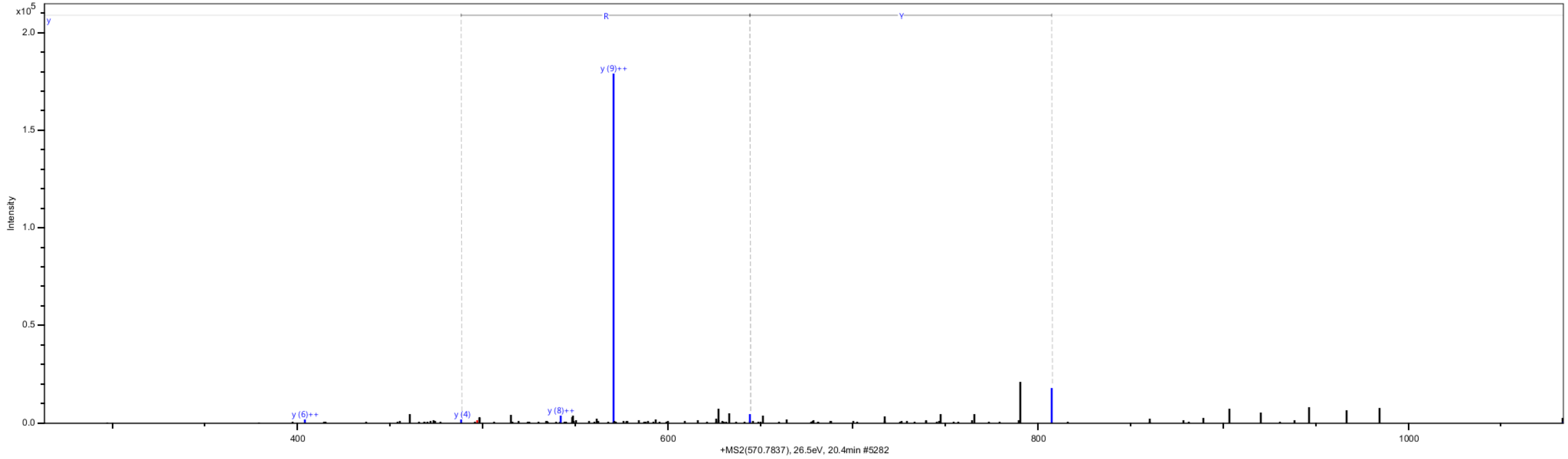

ZGRF1_HUMAN

**Accession:**	ZGRF1_HUMAN	**Score:**	17.0
**Database:**	SwissProt	**Seq. Coverage [%]:**	0.4
**MW [kDa] / pI:**	236.5 / 5.8	**No. of Peptides:**	1

Peptide Match

**Sequence:**	K.RMNVALTR.G	**Score:**	17
**Modifications:**	Oxidation: 2		

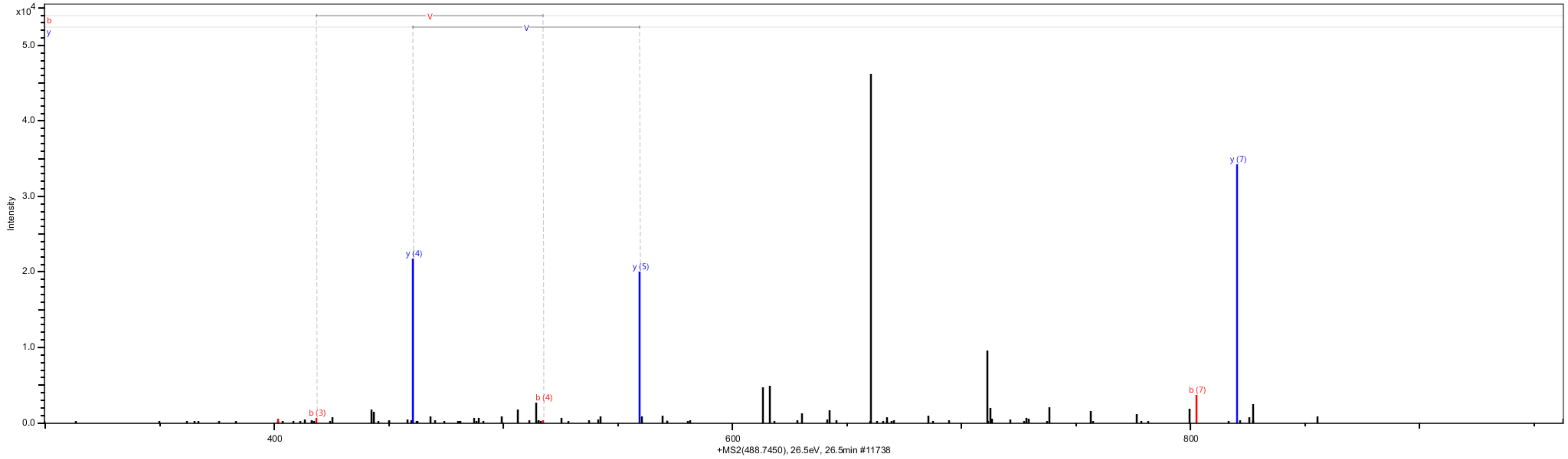

CDK18_HUMAN

Accession: CDK18_HUMAN
Database: SwissProt
MW [kDa] / pI: 54.4 / 8.8
Score: 17.0
Seq. Coverage [%]: 1.7
No. of Peptides: 1

Peptide Match

Sequence: R.RFSLSVPR.T
Score: 17
Modifications:

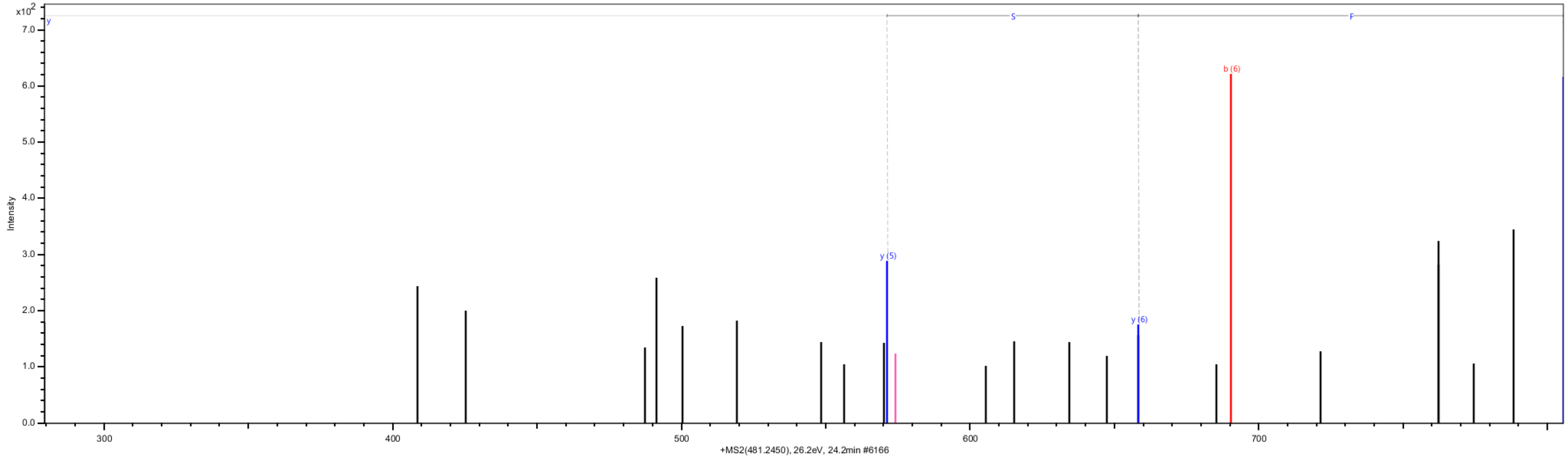

SRSF1_HUMAN

**Accession:**	SRSF1_HUMAN	**Score:**	17.0
**Database:**	SwissProt	**Seq. Coverage [%]:**	3.6
**MW [kDa] / pI:**	27.7 / 10.4	**No. of Peptides:**	1

Peptide Match

**Sequence:**	R.KEDMTYAVR.K	**Score:**	17
**Modifications:**			

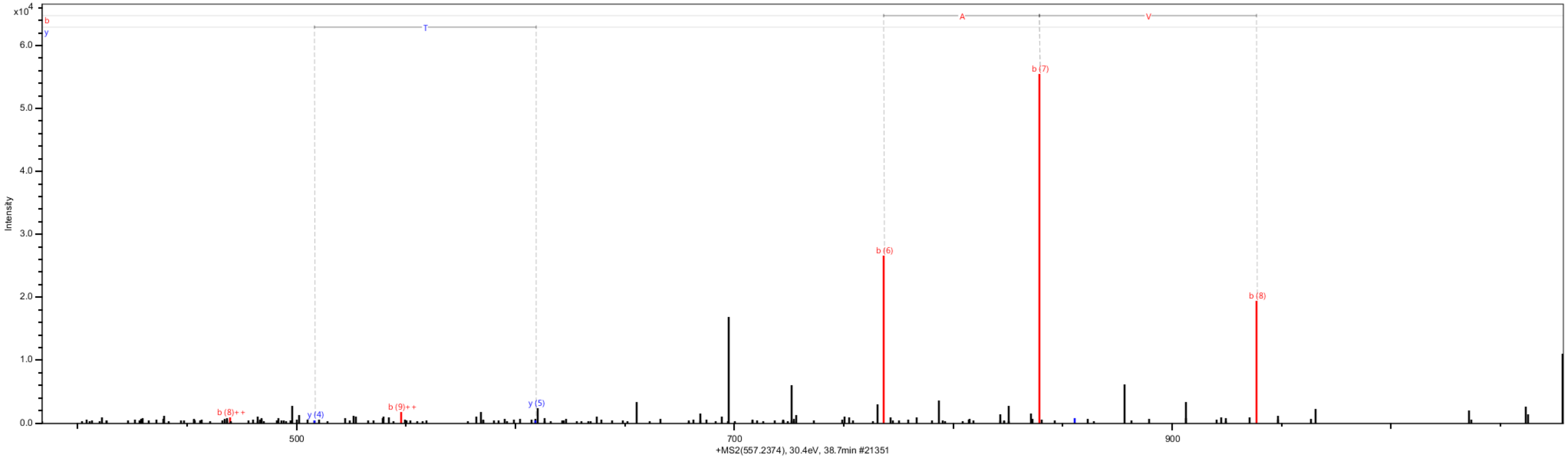

MYH4_HUMAN

Accession: MYH4_HUMAN
Database: SwissProt
MW [kDa] / pI: 222.9 / 5.7
Score: 17.0
Seq. Coverage [%]: 0.6
No. of Peptides: 1

Peptide Match

Sequence: K.MAEQELLDASER.V
Score: 17
Modifications:

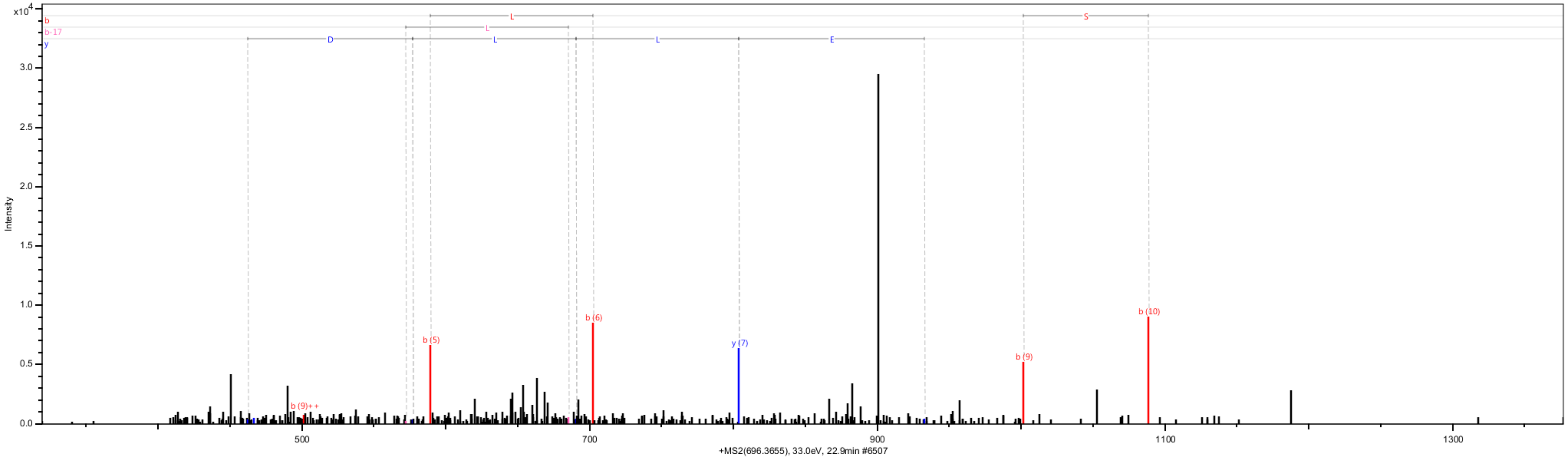

PPIP2_HUMAN

**Accession:**	PPIP2_HUMAN	**Score:**	17.0
**Database:**	SwissProt	**Seq. Coverage [%]:**	5.1
**MW [kDa] / pI:**	38.8 / 8.7	**No. of Peptides:**	1

Peptide Match

Sequence: K.DEAEQAVSRSANLVNPK.Q **Score:** 17

Modifications:

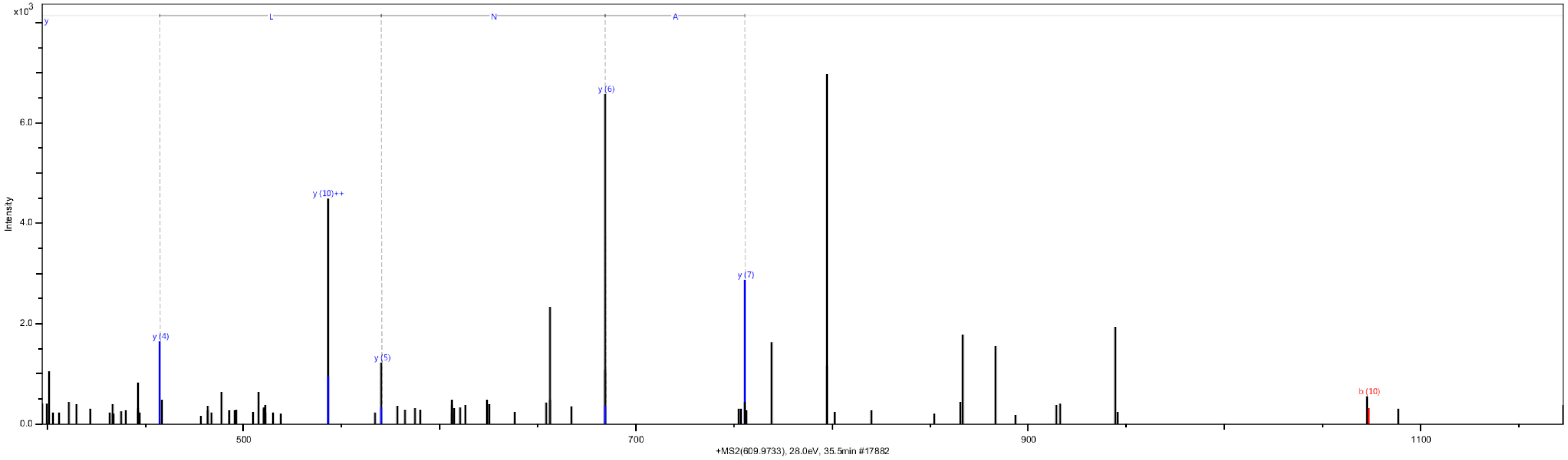

NEC1_HUMAN

Accession: NEC1_HUMAN
Database: SwissProt
MW [kDa] / pI: 84.1 / 5.7
Score: 17.0
Seq. Coverage [%]: 1.2
No. of Peptides: 1

Peptide Match

Sequence: K.ALVDLADPR.T
Score: 17
Modifications:

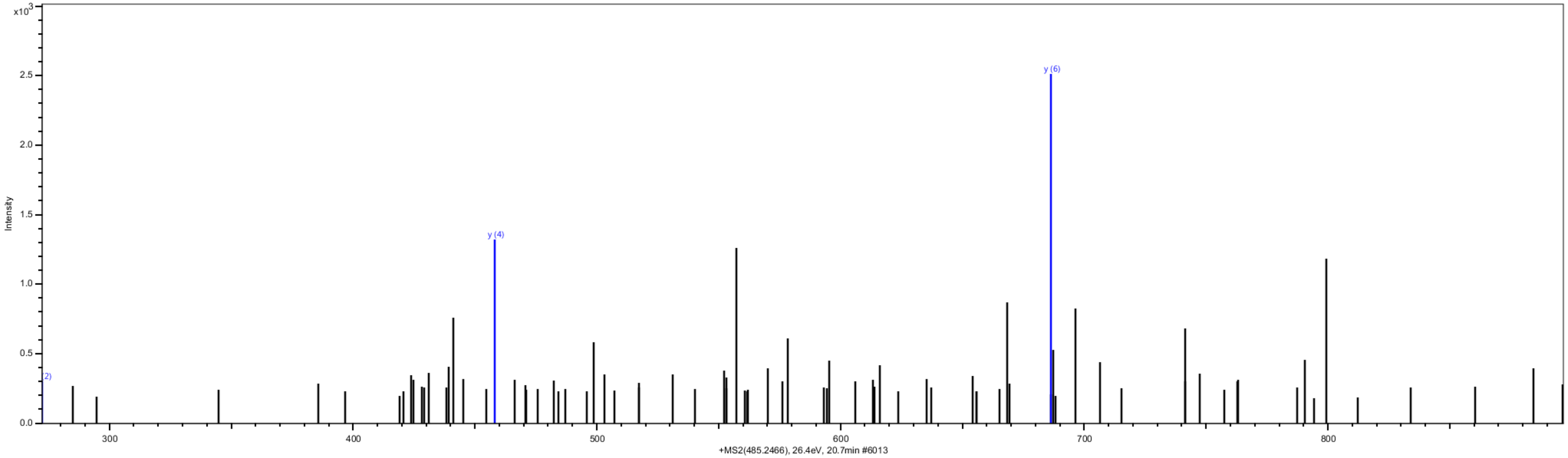

DPOLN_HUMAN

Accession: DPOLN_HUMAN
Database: SwissProt
MW [kDa] / pI: 100.2 / 8.6
Score: 17.0
Seq. Coverage [%]: 2.8
No. of Peptides: 1

Peptide Match

Sequence: K.QMALEEDTDDAEGYLNSGNSGALKK.H
Score: 17

Modifications:

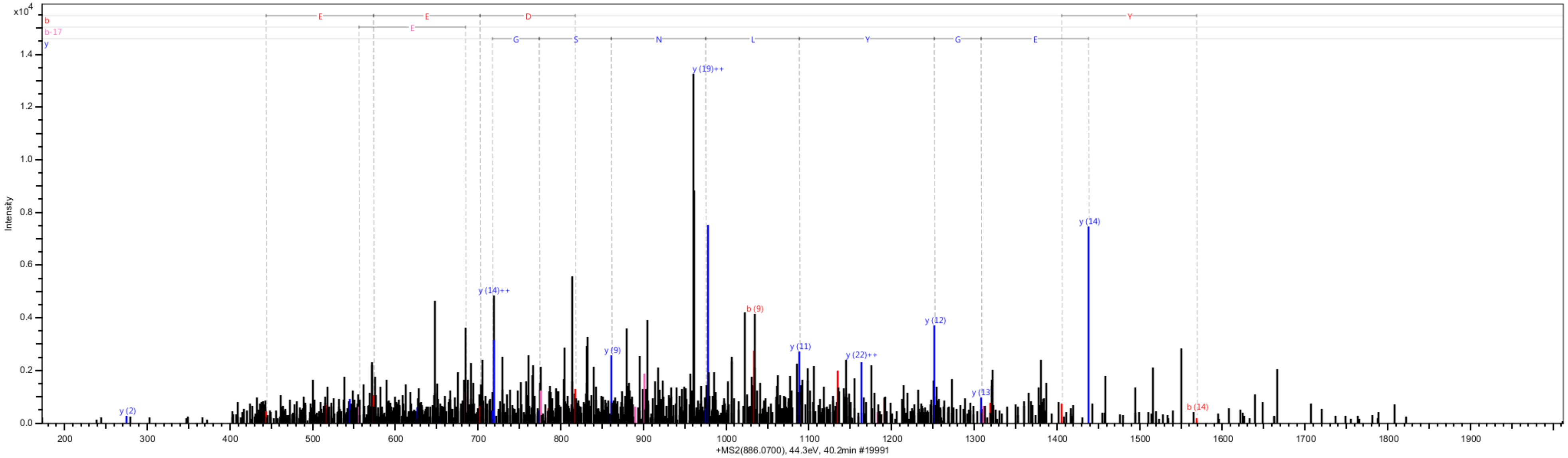

CANT1_HUMAN

**Accession:**	CANT1_HUMAN	**Score:**	16.9
**Database:**	SwissProt	**Seq. Coverage [%]:**	1.7
**MW [kDa] / pI:**	44.8 / 5.7	**No. of Peptides:**	1

Peptide Match

**Sequence:**	K.VAVEWDK.D	**Score:**	16.9
**Modifications:**			

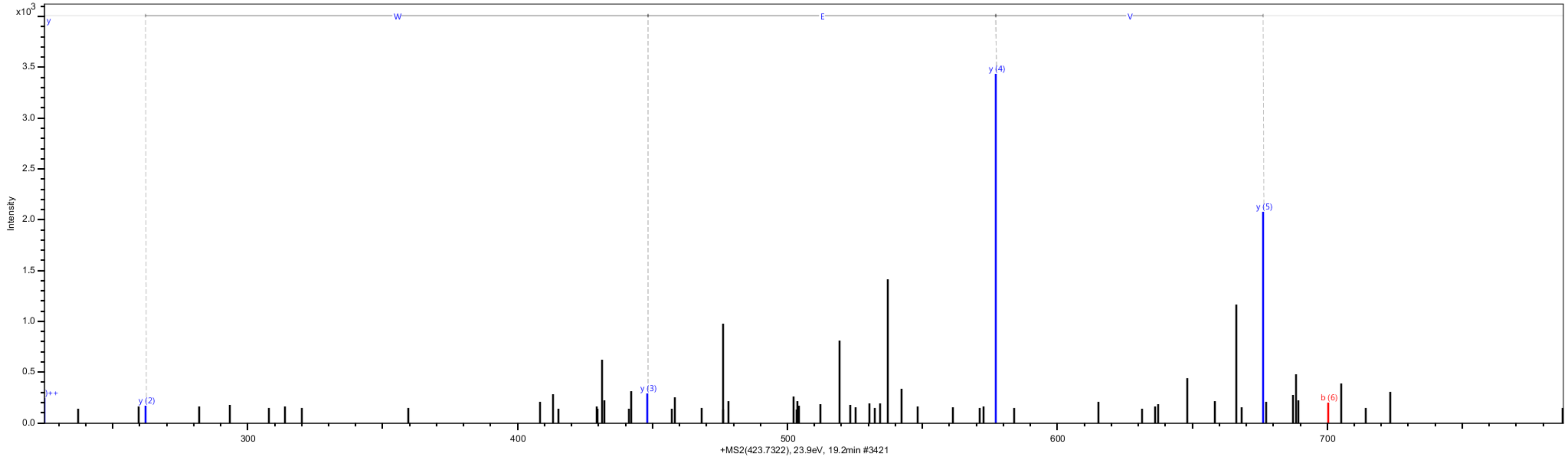

ARGAL_HUMAN

**Accession:**	ARGAL_HUMAN	**Score:**	16.9
**Database:**	SwissProt	**Seq. Coverage [%]:**	0.7
**MW [kDa] / pI:**	140.3 / 5.6	**No. of Peptides:**	1

Peptide Match

**Sequence:**	R.NLLYEDAHR.A	**Score:**	16.9
**Modifications:**			

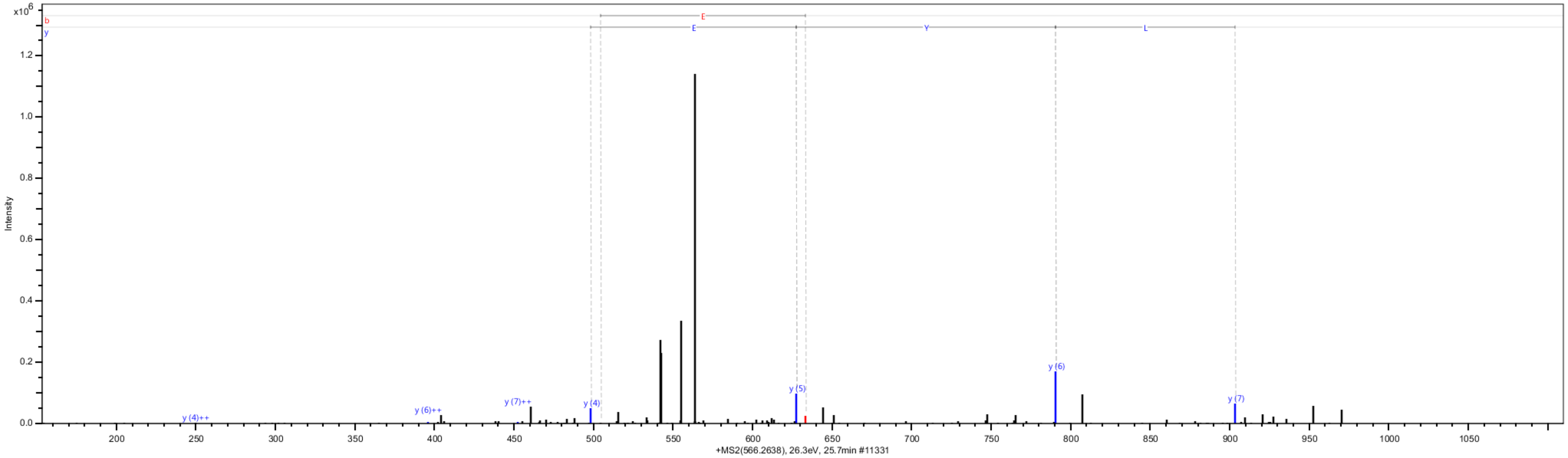

UBQL1_HUMAN

**Accession:**	UBQL1_HUMAN	**Score:**	16.9
**Database:**	SwissProt	**Seq. Coverage [%]:**	2.5
**MW [kDa] / pI:**	62.5 / 5.0	**No. of Peptides:**	1

Peptide Match

**Sequence:**	R.QLIMANPQMQQLIQR.N	**Score:**	16.9
**Modifications:**	Oxidation: 4		

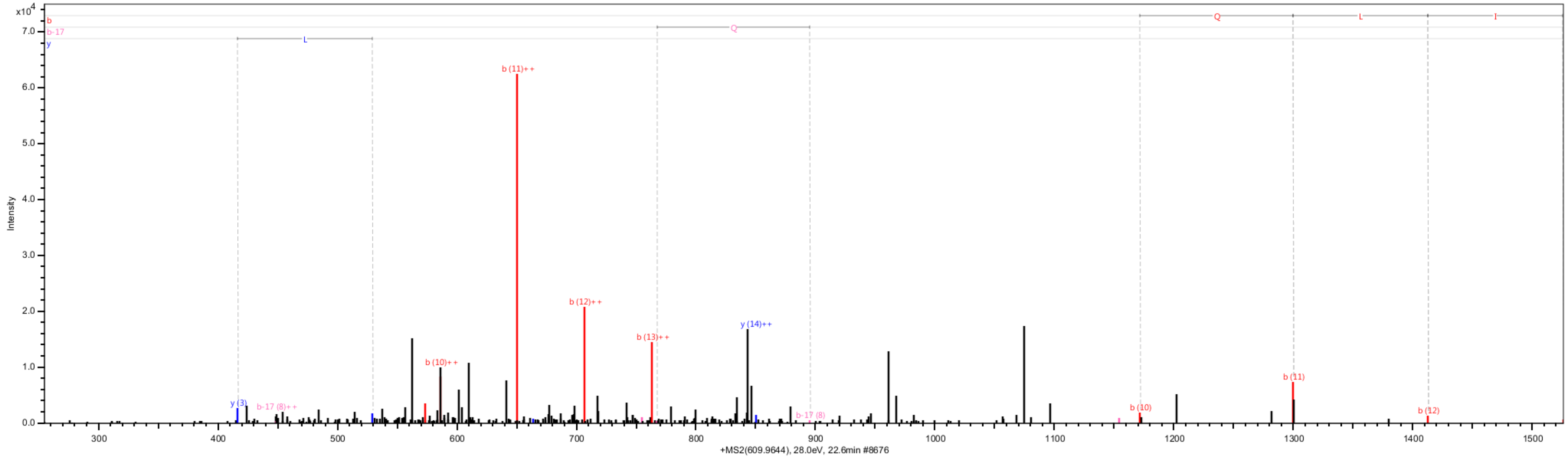

PKHG5_HUMAN

Accession: PKHG5_HUMAN
Database: SwissProt
MW [kDa] / pI: 117.4 / 5.9
Score: 16.9
Seq. Coverage [%]: 1.1
No. of Peptides: 1

Peptide Match

Sequence: R.FDLPPQGSVLAR.N
Score: 16.9
Modifications:

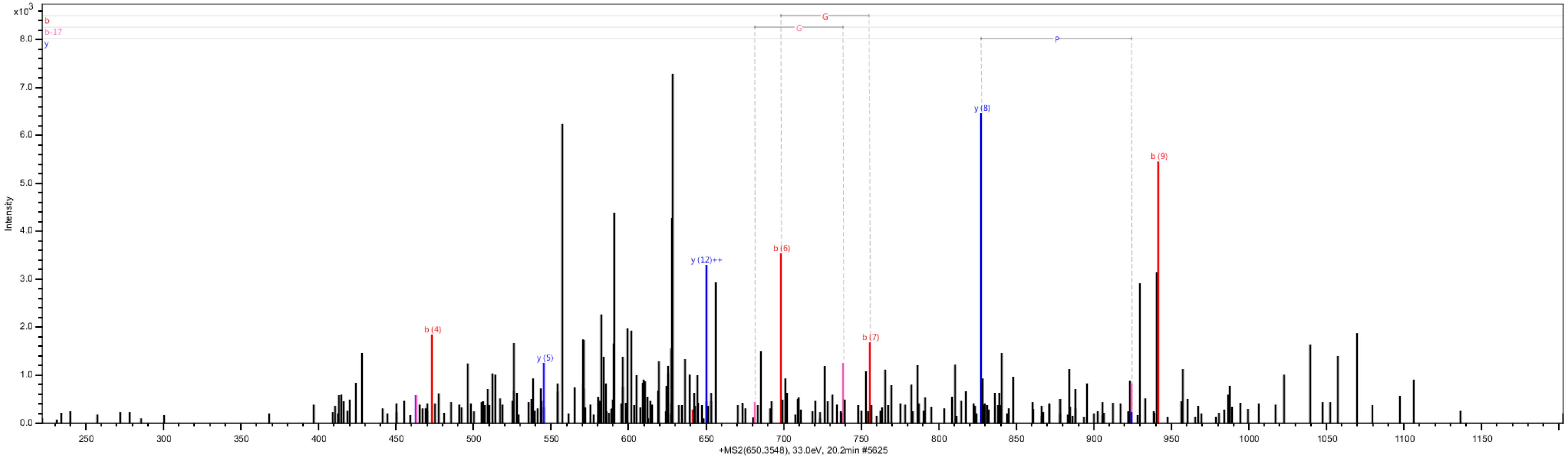

T200B_HUMAN

Accession: T200B_HUMAN
Database: SwissProt
MW [kDa] / pI: 32.7 / 11.4
Score: 16.9
Seq. Coverage [%]: 3.3
No. of Peptides: 1

Peptide Match

Sequence: K.LGGGGDLGAR.V
Score: 16.9
Modifications:

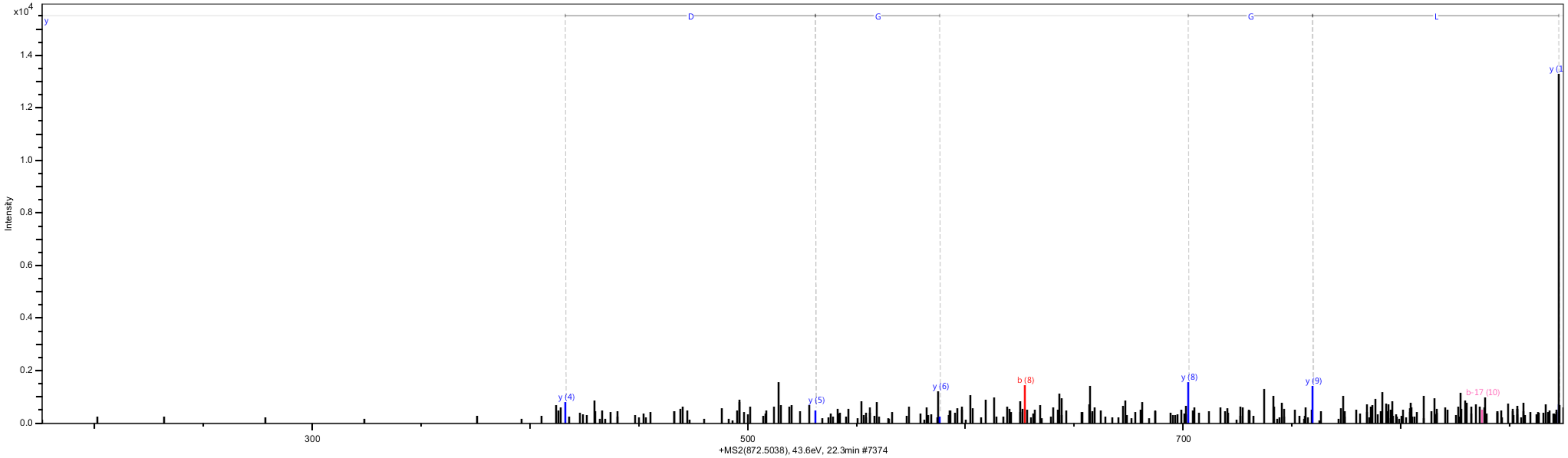

COE4_HUMAN

Accession: COE4_HUMAN
Database: SwissProt
MW [kDa] / pI: 64.4 / 8.9
Score: 16.9
Seq. Coverage [%]: 1.3
No. of Peptides: 1

Peptide Match

Sequence: K.DREPGAEK.T
Score: 16.9
Modifications:

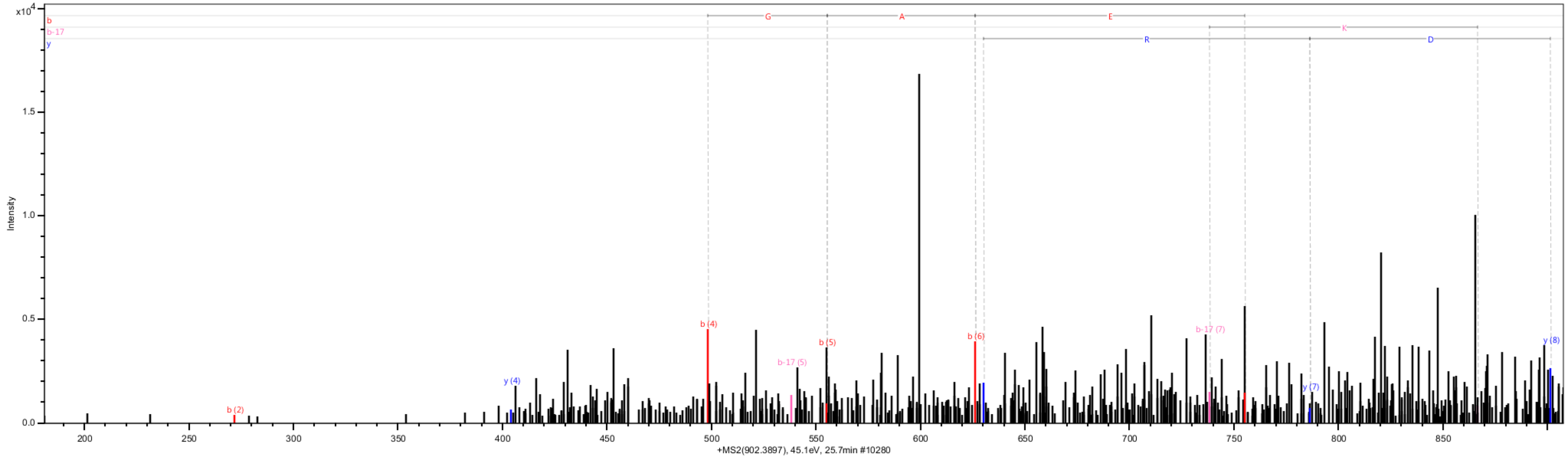

MTCL1_HUMAN

**Accession:**	MTCL1_HUMAN	**Score:**	16.2
**Database:**	SwissProt	**Seq. Coverage [%]:**	0.5
**MW [kDa] / pI:**	209.4 / 6.0	**No. of Peptides:**	1

Peptide Match

**Sequence:**	R.SRQVAPAIEK.V	**Score:**	16.2
**Modifications:**			

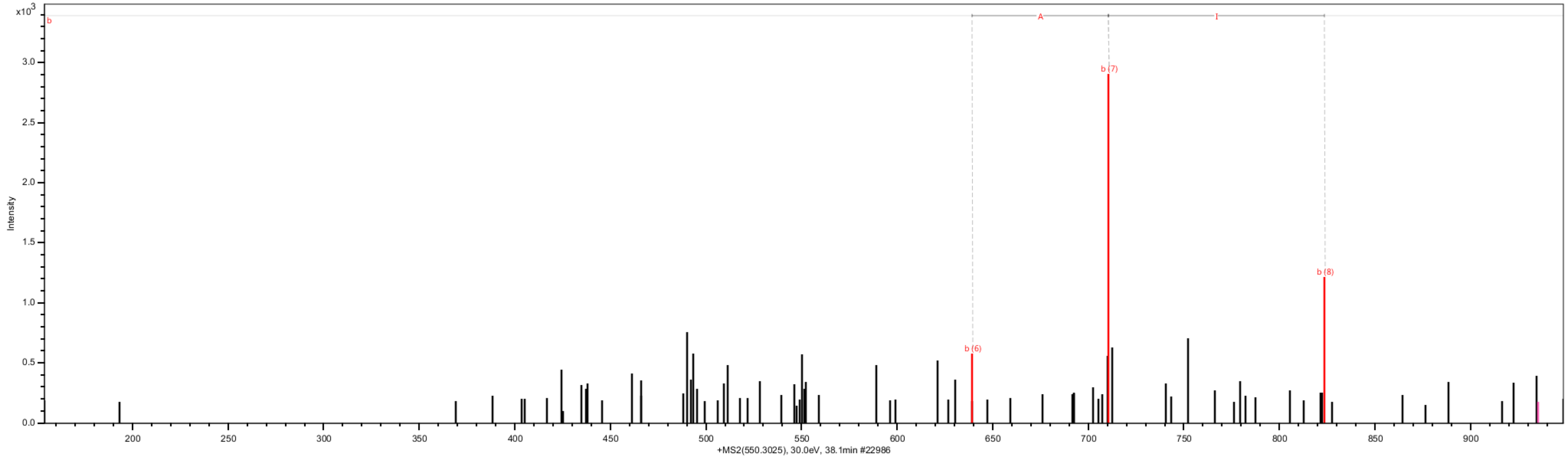

ASM3B_HUMAN

Accession: ASM3B_HUMAN
Database: SwissProt
MW [kDa] / pI: 50.8 / 5.4
Score: 16.2
Seq. Coverage [%]: 2.2
No. of Peptides: 1

Peptide Match

Sequence: K.AWFREGFNEK.Y
Score: 16.2
Modifications:

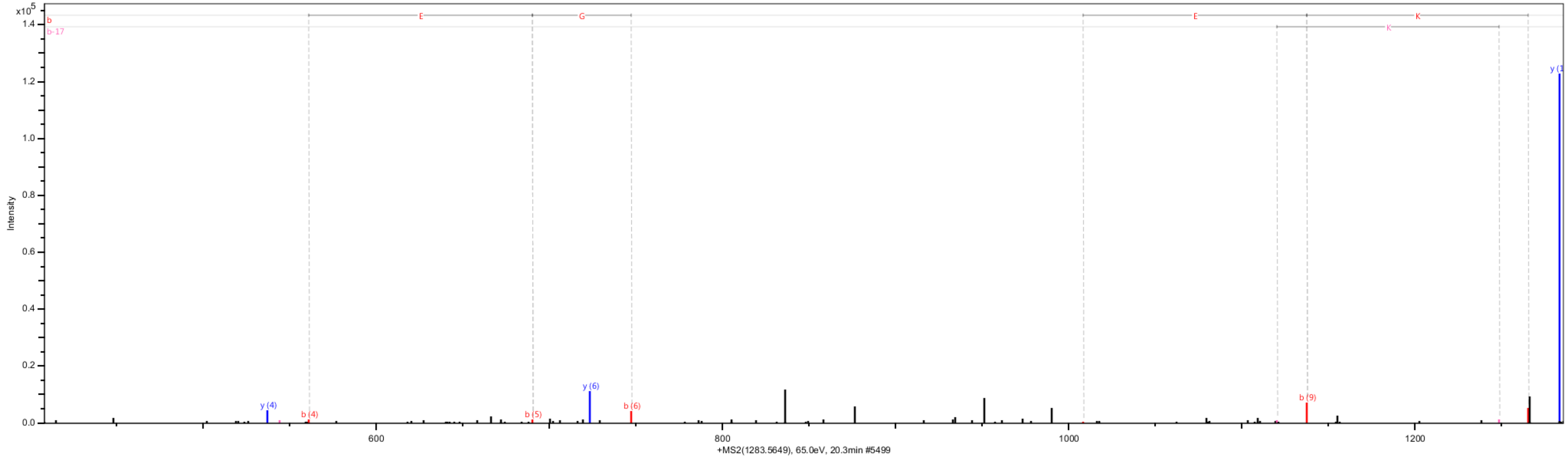

SPT13_HUMAN

Accession: SPT13_HUMAN
Database: SwissProt
MW [kDa] / pI: 74.8 / 7.0
Score: 18.2
Seq. Coverage [%]: 1.4
No. of Peptides: 1

Peptide Match

Sequence: K.KLAMLNAQK.A
Score: 18.2
Modifications:

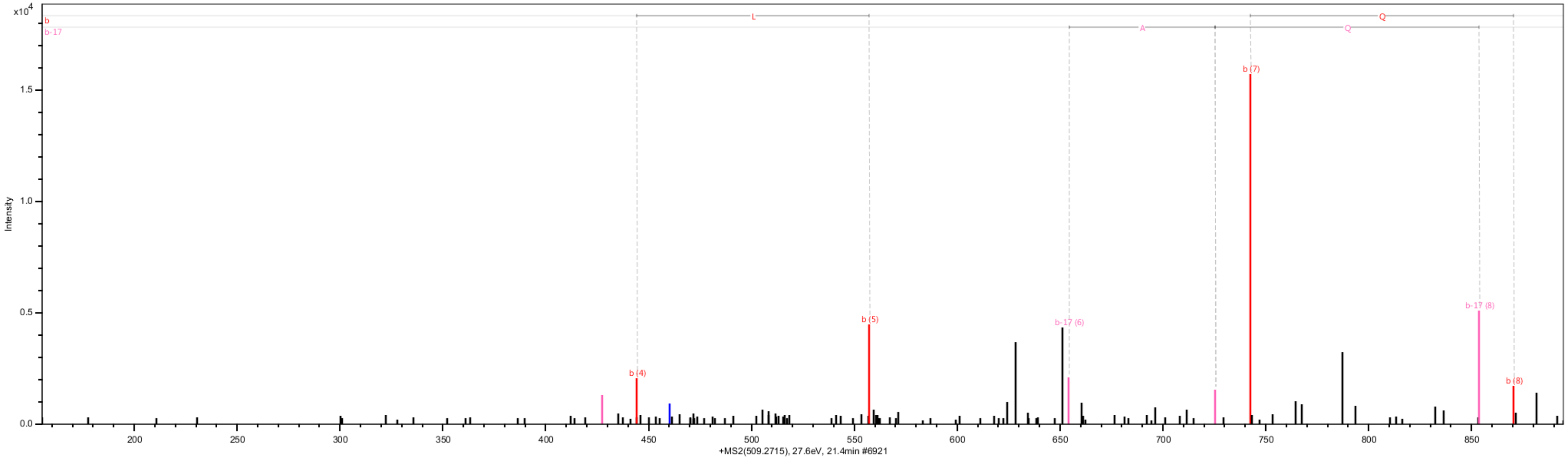

ACTBL_HUMAN

Accession: ACTBL_HUMAN
Database: SwissProt
MW [kDa] / pI: 42.0 / 5.4
Score: 19.2
Seq. Coverage [%]: 2.9
No. of Peptides: 1

Peptide Match

Sequence: R.AVFPSMIGRPR.H
Score: 19.2
Modifications:

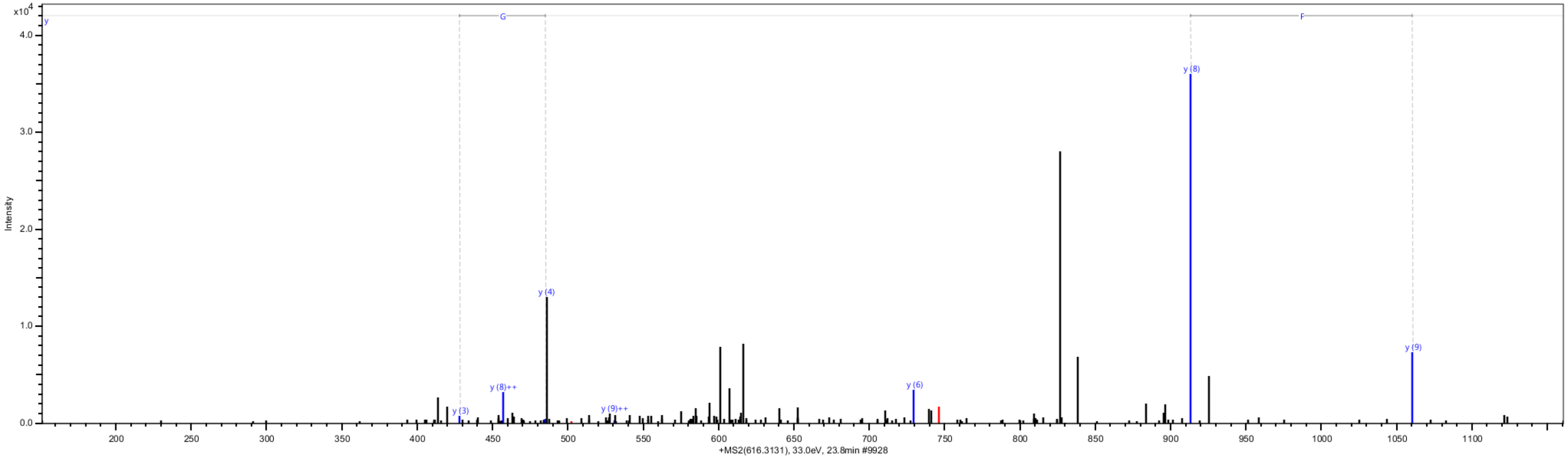

RCC1_HUMAN

**Accession:**	RCC1_HUMAN	**Score:**	19.2
**Database:**	SwissProt	**Seq. Coverage [%]:**	2.1
**MW [kDa] / pI:**	44.9 / 7.2	**No. of Peptides:**	1

Peptide Match

**Sequence:**	R.SPPADAIPK.S	**Score:**	19.2
**Modifications:**			

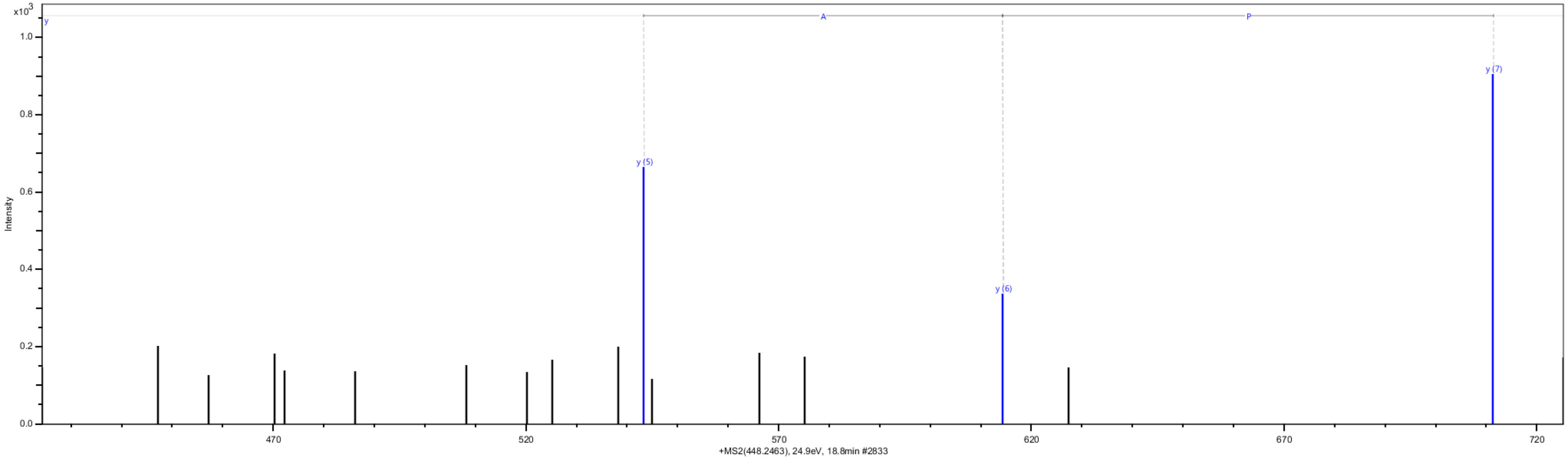

HACD2_HUMAN

**Accession:**	HACD2_HUMAN	**Score:**	19.2
**Database:**	SwissProt	**Seq. Coverage [%]:**	4.3
**MW [kDa] / pI:**	28.4 / 9.6	**No. of Peptides:**	1

Peptide Match

**Sequence:**	-.MAAVAATAAAK.G	**Score:**	19.2
**Modifications:**	Oxidation: 1		

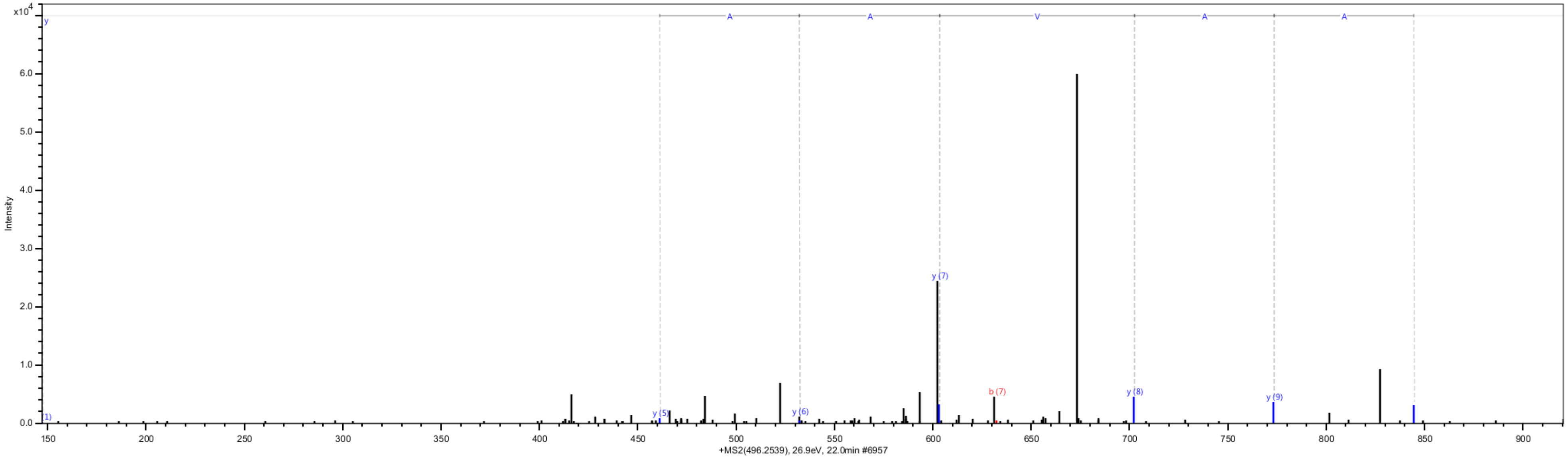

UAP1_HUMAN

**Accession:**	UAP1_HUMAN	**Score:**	19.2
**Database:**	SwissProt	**Seq. Coverage [%]:**	2.1
**MW [kDa] / pI:**	58.7 / 5.9	**No. of Peptides:**	1

Peptide Match

**Sequence:**	K.GADCGAKVVEK.T	**Score:**	19.2
**Modifications:**	Carbamidomethyl: 4		

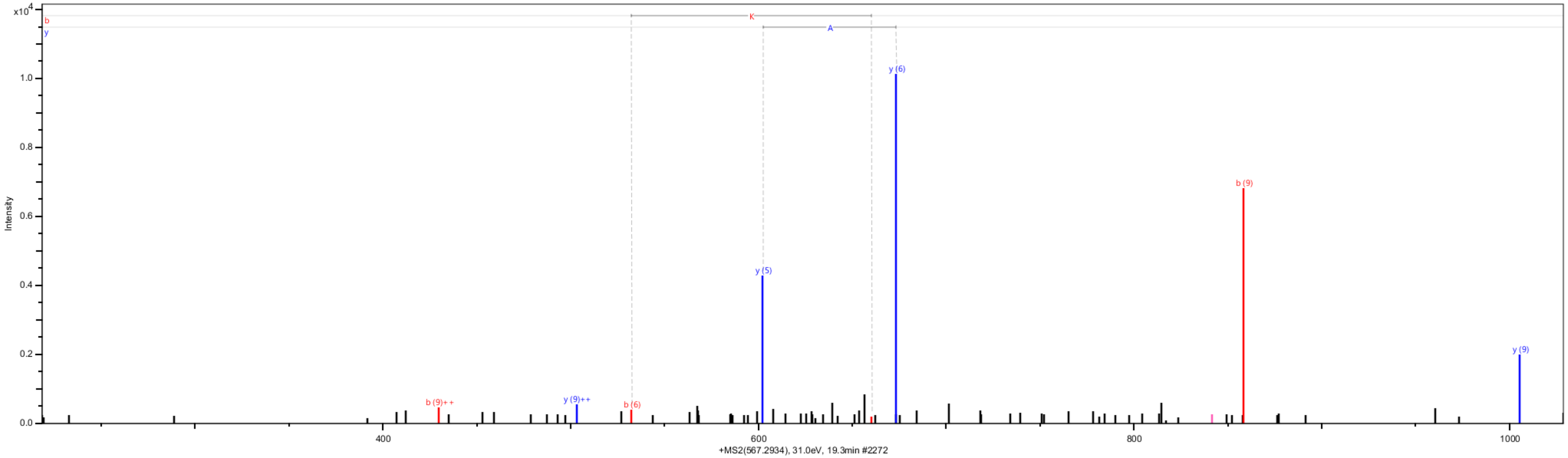

AUP1_HUMAN

Accession: AUP1_HUMAN
Database: SwissProt
MW [kDa] / pI: 45.8 / 9.0
Score: 19.2
Seq. Coverage [%]: 2.0
No. of Peptides: 1

Peptide Match

Sequence: K.QALYEYAR.R
Score: 19.2
Modifications:

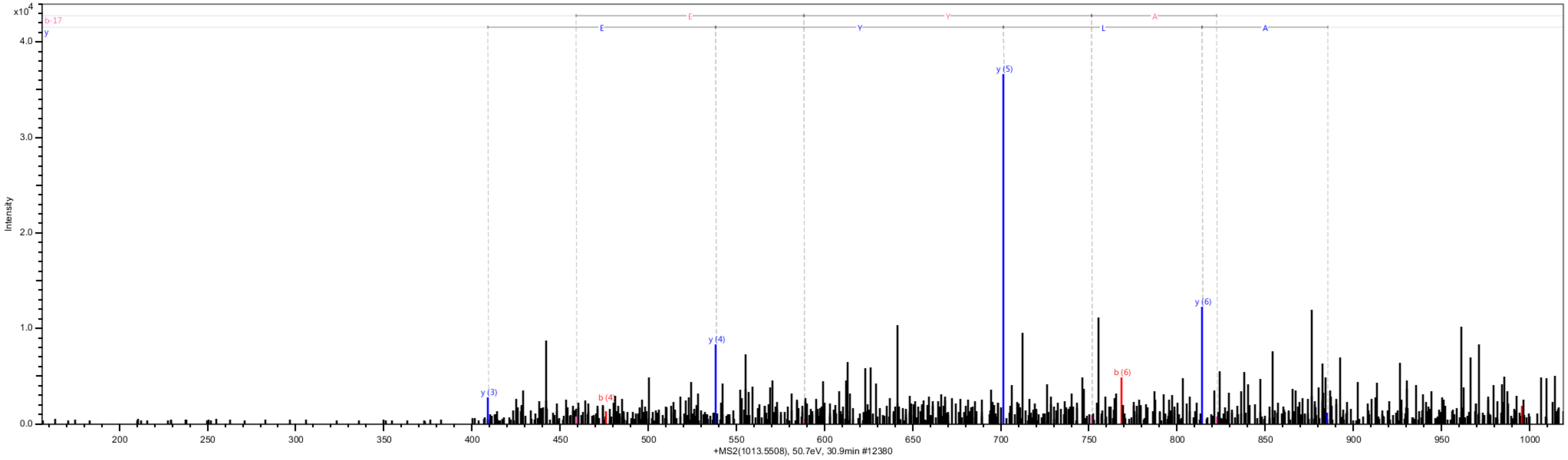

ANTR1_HUMAN

**Accession:**	ANTR1_HUMAN	**Score:**	19.1
**Database:**	SwissProt	**Seq. Coverage [%]:**	2.0
**MW [kDa] / pI:**	62.7 / 7.5	**No. of Peptides:**	1

Peptide Match

**Sequence:**	R.GTTLMKLTEDR.E	**Score:**	19.1
**Modifications:**	Oxidation: 5		

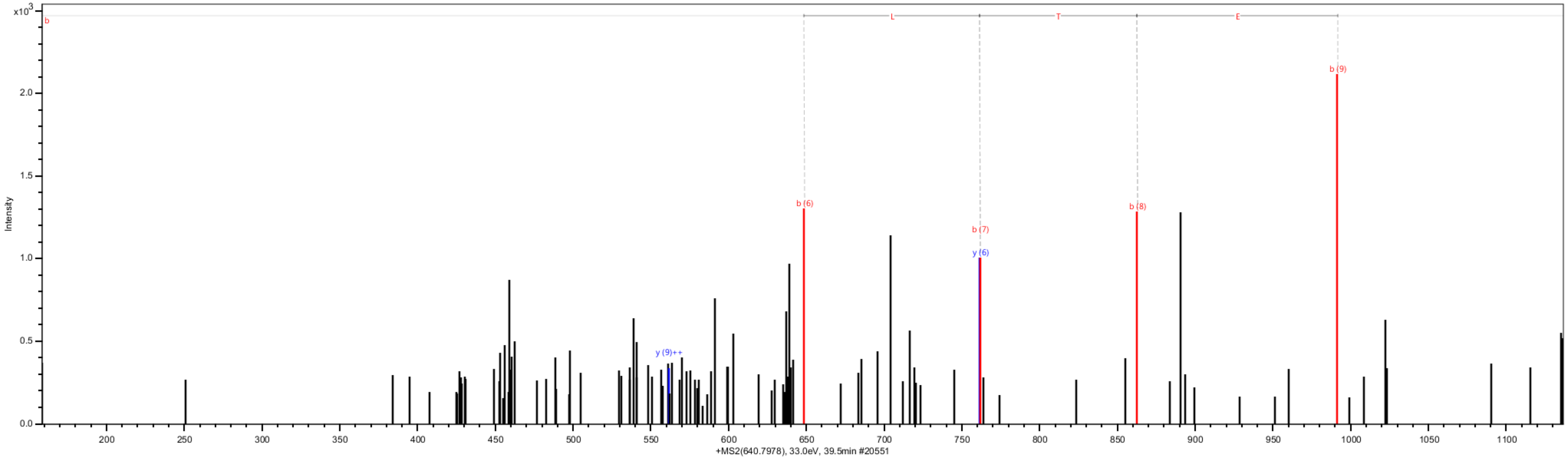

WDR26_HUMAN

**Accession:**	WDR26_HUMAN	**Score:**	19.1
**Database:**	SwissProt	**Seq. Coverage [%]:**	1.7
**MW [kDa] / pI:**	72.1 / 5.7	**No. of Peptides:**	1

Peptide Match

**Sequence:**	K.RLSQSDEDVIR.L	**Score:**	19.1
**Modifications:**			

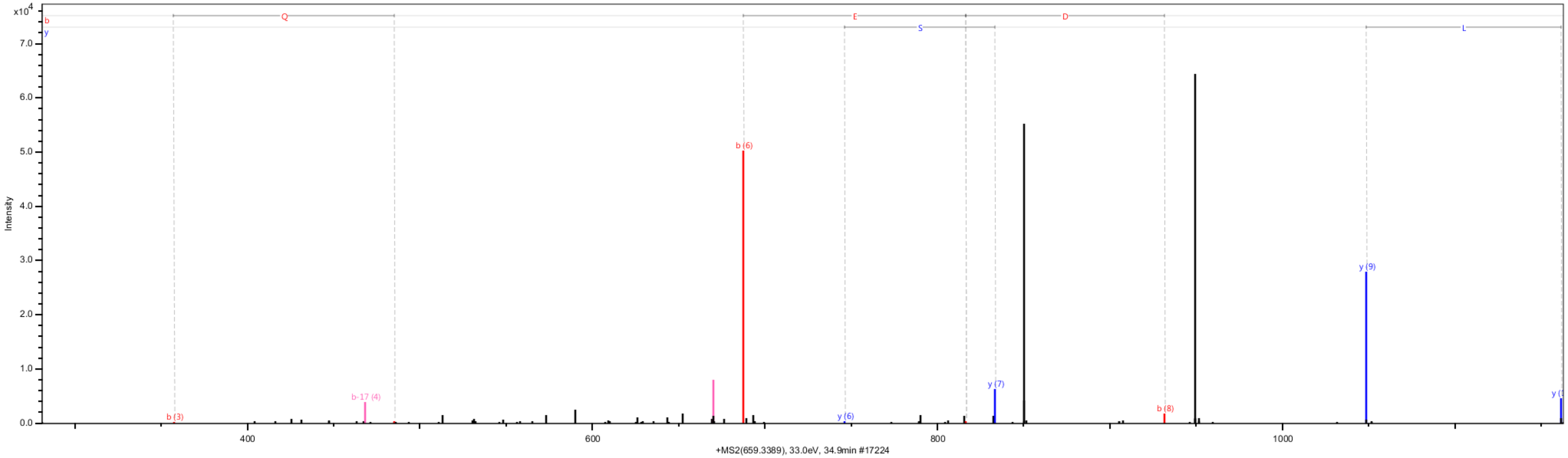

ASHWN_HUMAN

Accession: ASHWN_HUMAN
Database: SwissProt
MW [kDa] / pI: 25.8 / 9.8
Score: 19.1
Seq. Coverage [%]: 3.9
No. of Peptides: 1

Peptide Match

Sequence: K.RSSTVDGLR.K
Score: 19.1
Modifications:

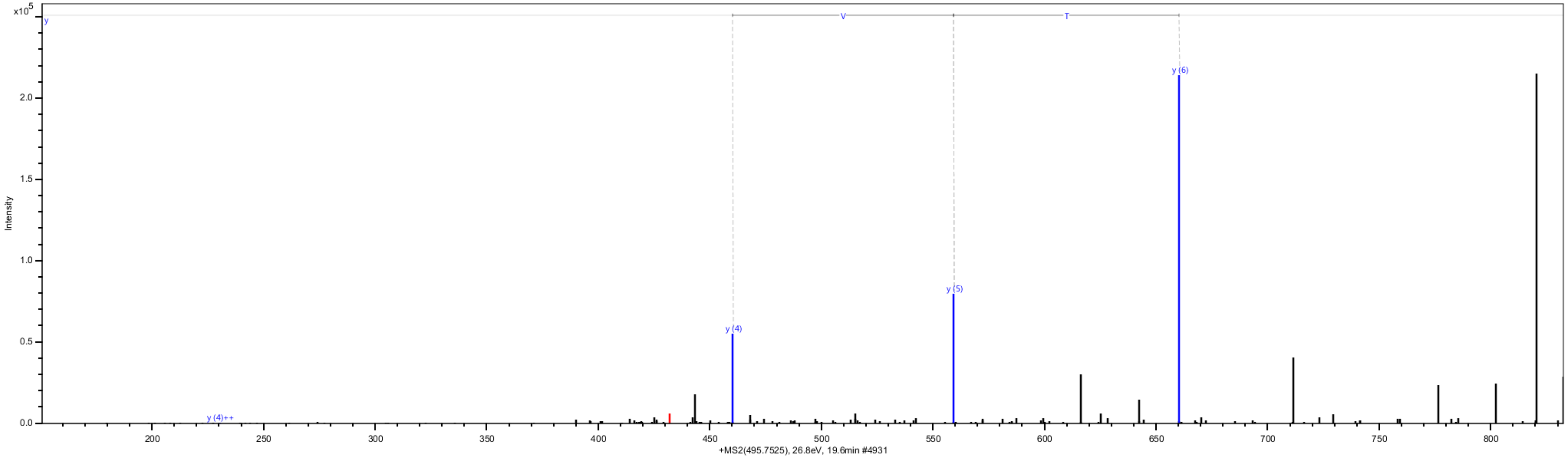

FA83G_HUMAN

Accession: FA83G_HUMAN
Database: SwissProt
MW [kDa] / pI: 90.8 / 6.0
Score: 19.1
Seq. Coverage [%]: 1.1
No. of Peptides: 1

Peptide Match

Sequence: R.DAFYEVLKR.E
Score: 19.1
Modifications:

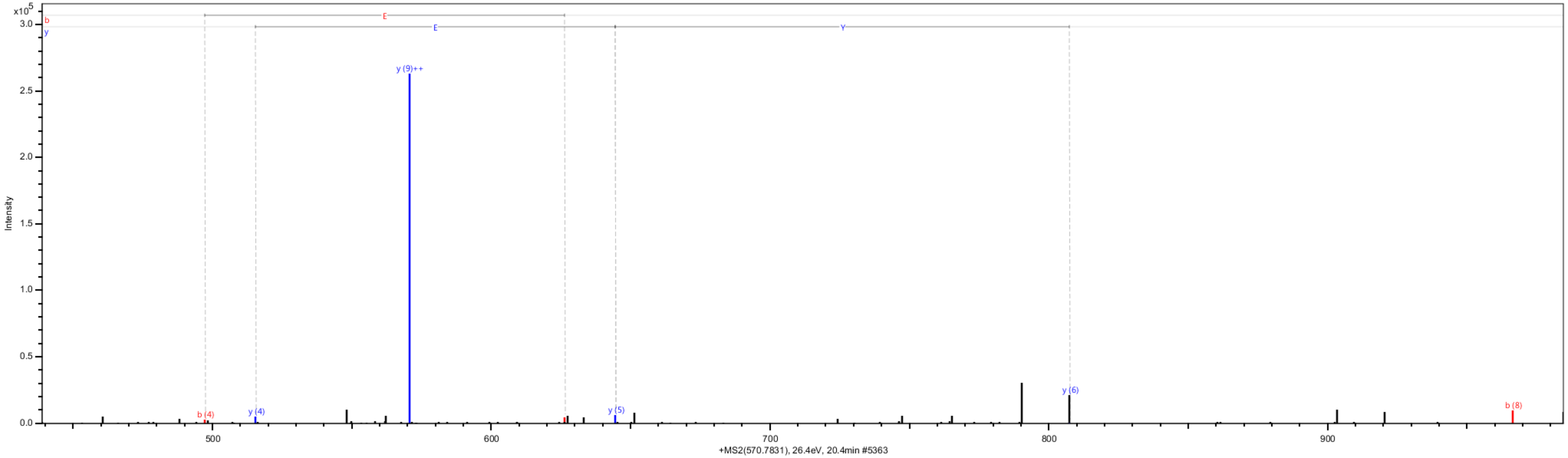

NEDD4_HUMAN

**Accession:**	NEDD4_HUMAN	**Score:**	19.1
**Database:**	SwissProt	**Seq. Coverage [%]:**	0.8
**MW [kDa] / pI:**	149.0 / 6.1	**No. of Peptides:**	1

Peptide Match

**Sequence:**	K.AVLMMDSEKR.I	**Score:**	19.1
**Modifications:**			

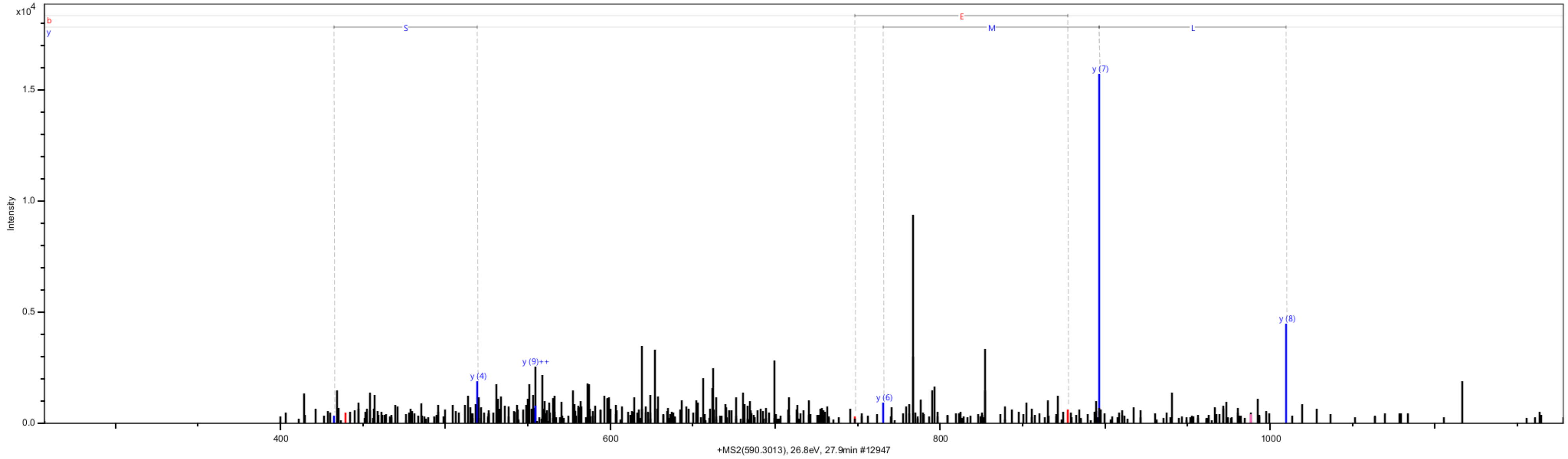

ESR2_HUMAN

**Accession:**	ESR2_HUMAN	**Score:**	19.1
**Database:**	SwissProt	**Seq. Coverage [%]:**	1.7
**MW [kDa] / pI:**	59.2 / 8.8	**No. of Peptides:**	1

Peptide Match

**Sequence:**	K.EGSQNPQSQ.-	**Score:**	19.1
**Modifications:**			

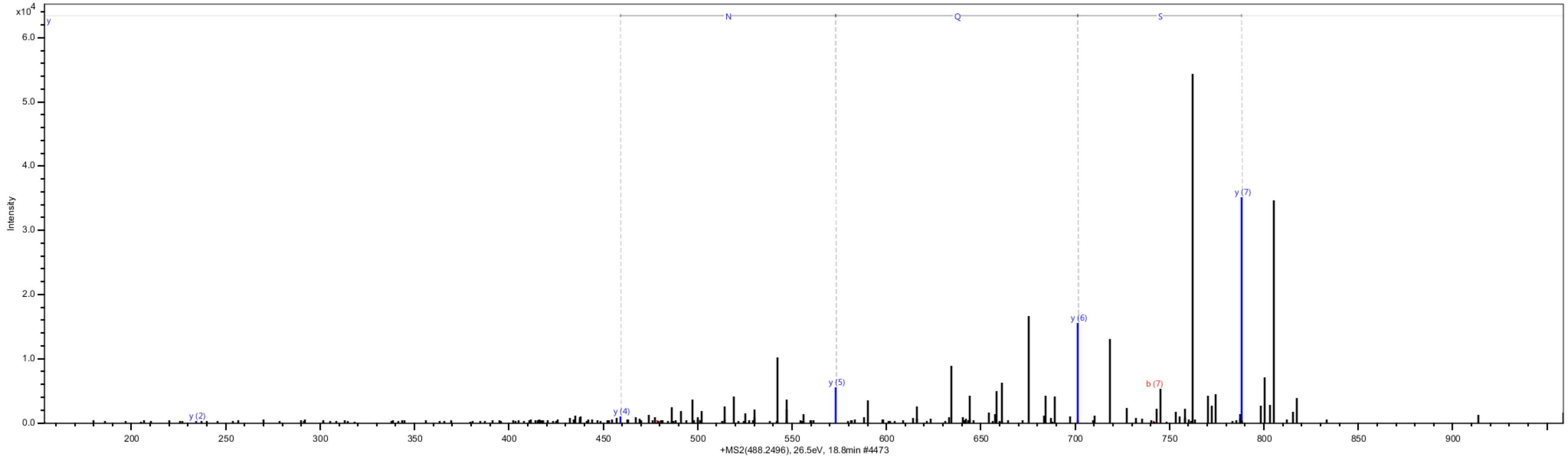

AP5Z1_HUMAN

Accession: AP5Z1_HUMAN
Database: SwissProt
MW [kDa] / pI: 88.5 / 6.6
Score: 19.1
Seq. Coverage [%]: 0.9
No. of Peptides: 1

Peptide Match

Sequence: R.LLPPRER.L
Score: 19.1
Modifications:

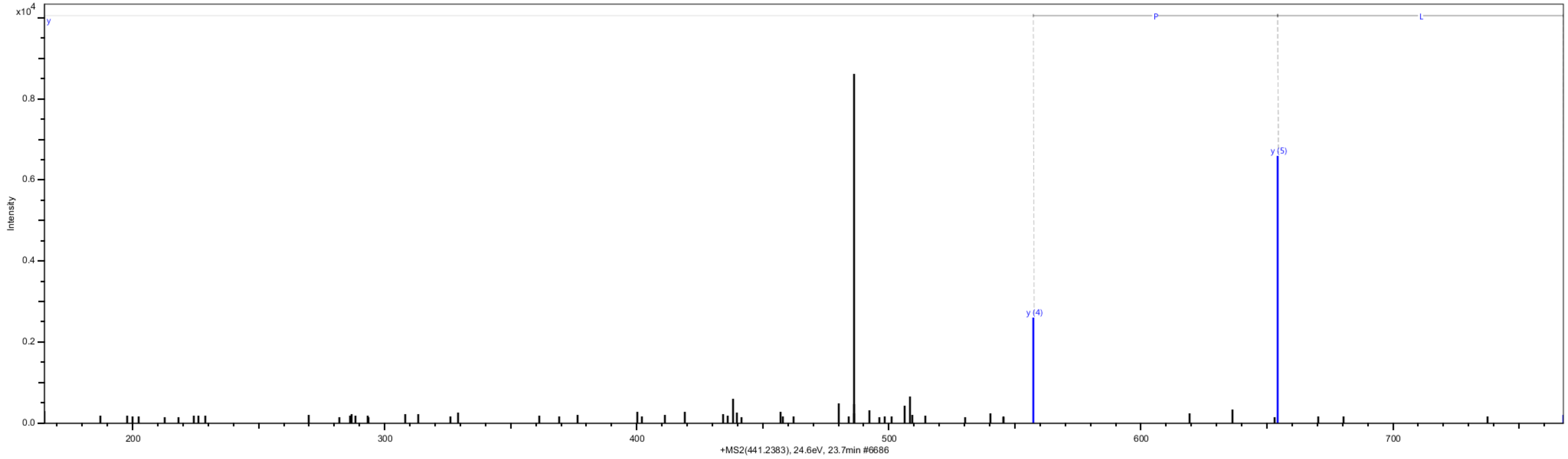

NOE1_HUMAN

Accession: NOE1_HUMAN
Database: SwissProt
MW [kDa] / pI: 55.3 / 6.5
Score: 19.1
Seq. Coverage [%]: 2.3
No. of Peptides: 1

Peptide Match

Sequence: K.MENQMKGLESK.F
Score: 19.1
Modifications: Oxidation: 1, 5

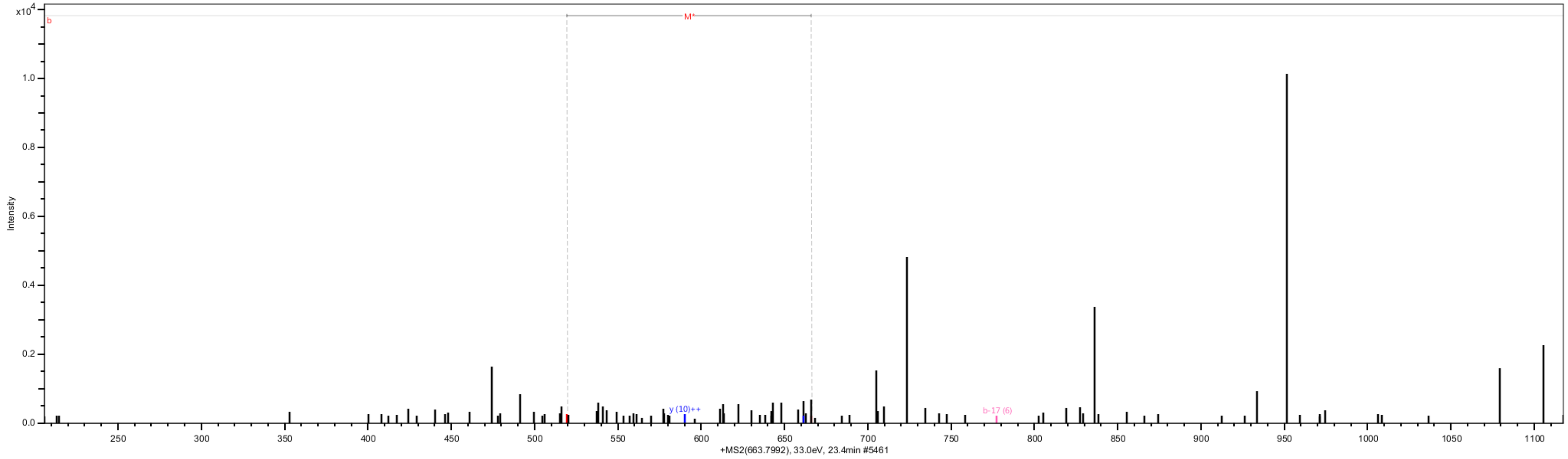

MMP2_HUMAN

Accession: MMP2_HUMAN
Database: SwissProt
MW [kDa] / pI: 73.8 / 5.3
Score: 19.0
Seq. Coverage [%]: 1.4
No. of Peptides: 1

Peptide Match

Sequence: R.VDAAFNWSK.N
Score: 19
Modifications:

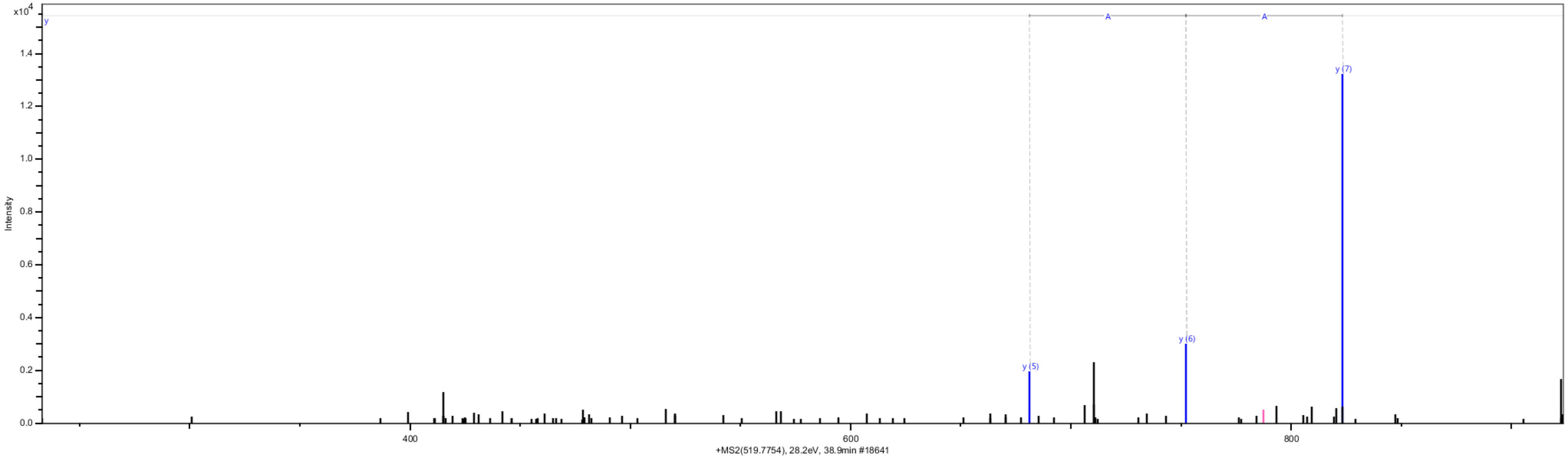

ANKZ1_HUMAN

Accession: ANKZ1_HUMAN
Database: SwissProt
MW [kDa] / pI: 80.9 / 8.7
Score: 19.0
Seq. Coverage [%]: 1.4
No. of Peptides: 1

Peptide Match

Sequence: R.DLLAGPSWAK.A
Score: 19
Modifications:

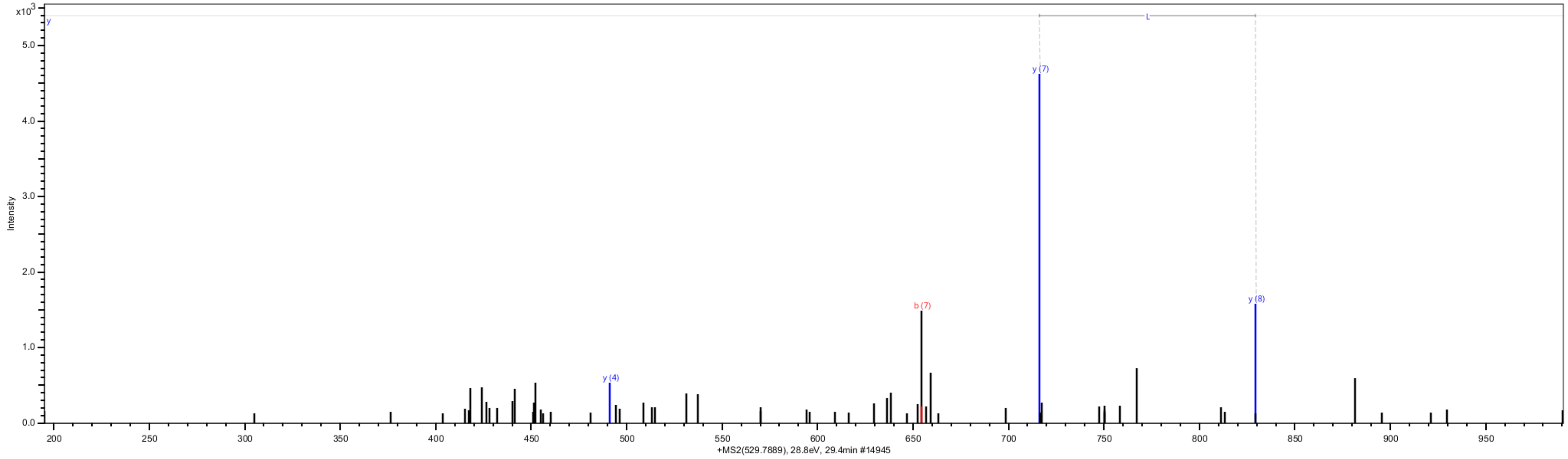

MROH5_HUMAN

**Accession:**	MROH5_HUMAN	**Score:**	19.0
**Database:**	SwissProt	**Seq. Coverage [%]:**	0.5
**MW [kDa] / pI:**	149.0 / 7.6	**No. of Peptides:**	1

Peptide Match

**Sequence:**	K.NGATVRR.H	**Score:**	19
**Modifications:**			

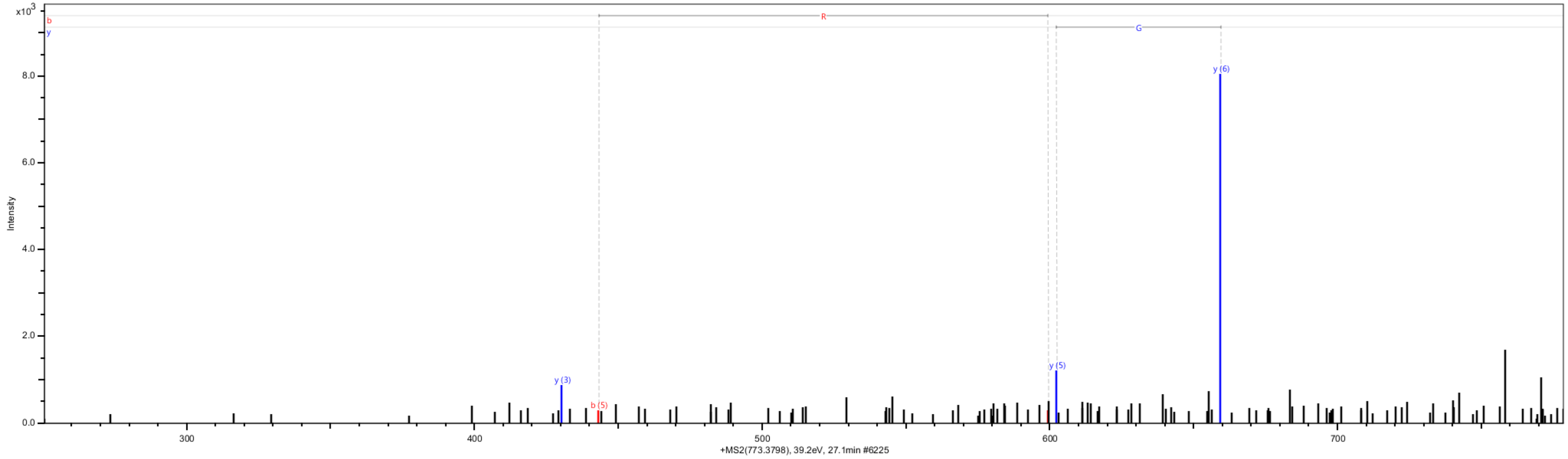

PAI1_HUMAN

Accession: PAI1_HUMAN
Database: SwissProt
MW [kDa] / pI: 45.0 / 6.7
Score: 18.7
Seq. Coverage [%]: 2.5
No. of Peptides: 1

Peptide Match

Sequence: R.VFQQVAQASK.D
Score: 18.7
Modifications:

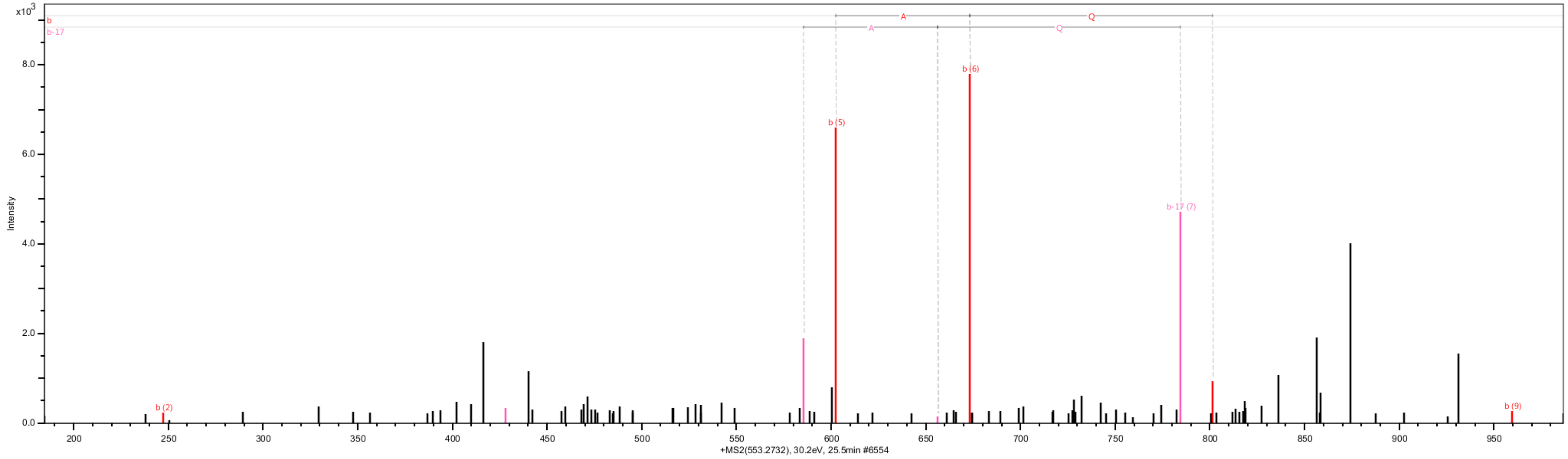

FRITZ_HUMAN

Accession: FRITZ_HUMAN
Database: SwissProt
MW [kDa] / pI: 85.0 / 6.0
Score: 18.7
Seq. Coverage [%]: 1.1
No. of Peptides: 1

Peptide Match

Sequence: R.RFFHHLLR.Y
Score: 18.7
Modifications:

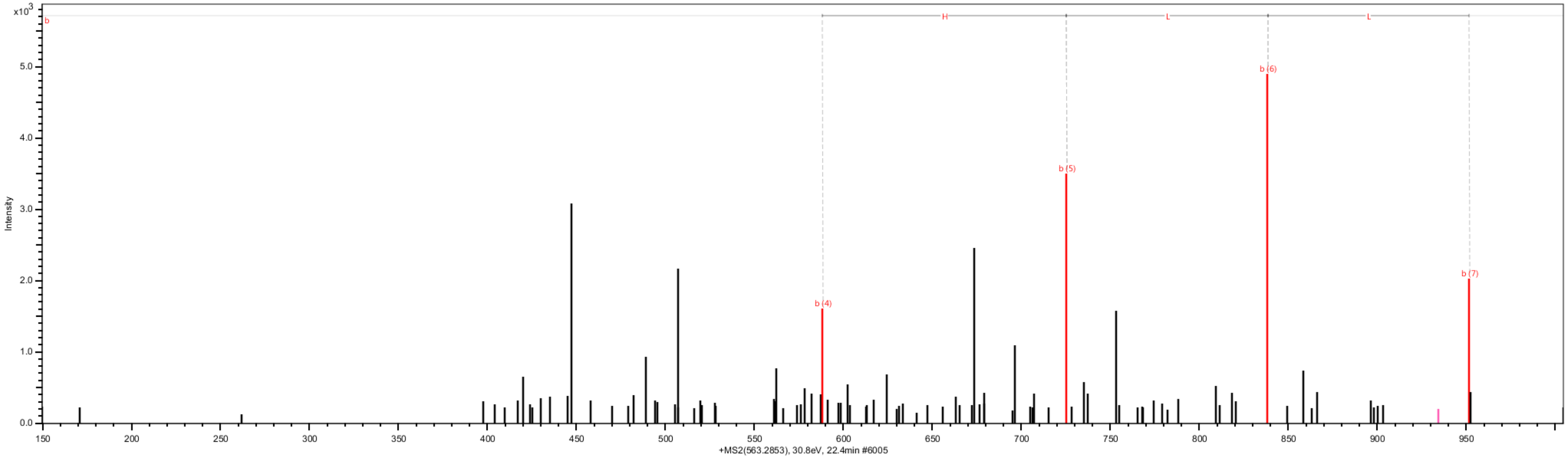

TSN12_HUMAN

**Accession:**	TSN12_HUMAN	**Score:**	18.7
**Database:**	SwissProt	**Seq. Coverage [%]:**	3.6
**MW [kDa] / pI:**	35.4 / 5.7	**No. of Peptides:**	1

Peptide Match

**Sequence:**	R.EPGTDQMMSLK.N	**Score:**	18.7
**Modifications:**	Oxidation: 7		

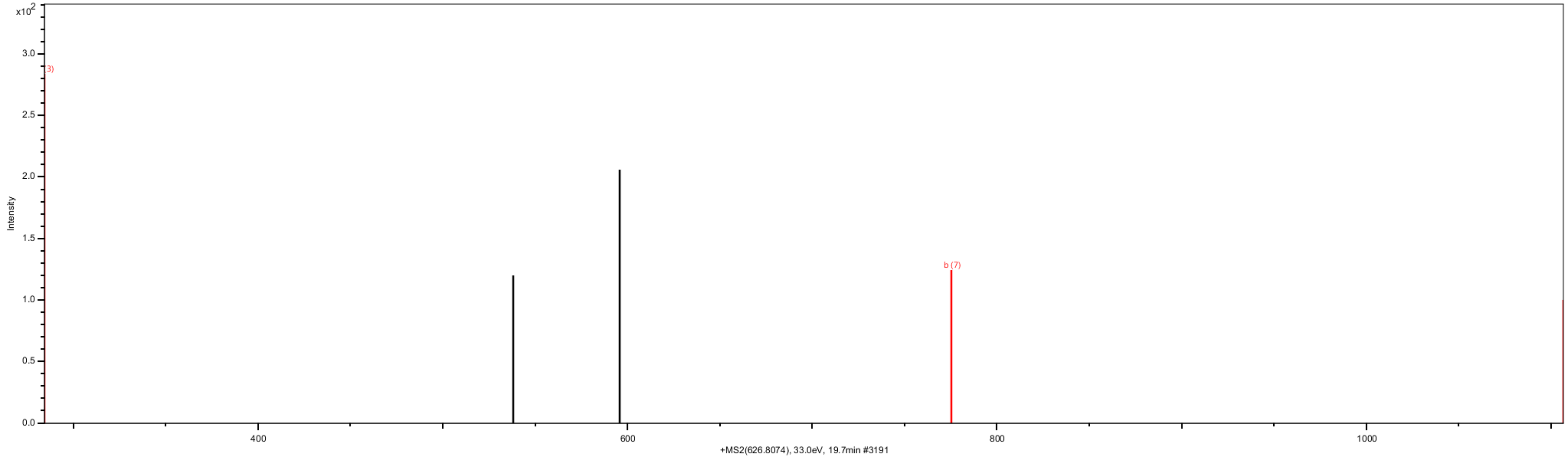

SUMO1_HUMAN

**Accession:**	SUMO1_HUMAN	**Score:**	18.7
**Database:**	SwissProt	**Seq. Coverage [%]:**	15.8
**MW [kDa] / pI:**	11.5 / 5.3	**No. of Peptides:**	1

Peptide Match

Sequence: -.MSDQEAKPSTEDLGDK.K **Score:** 18.7

Modifications:

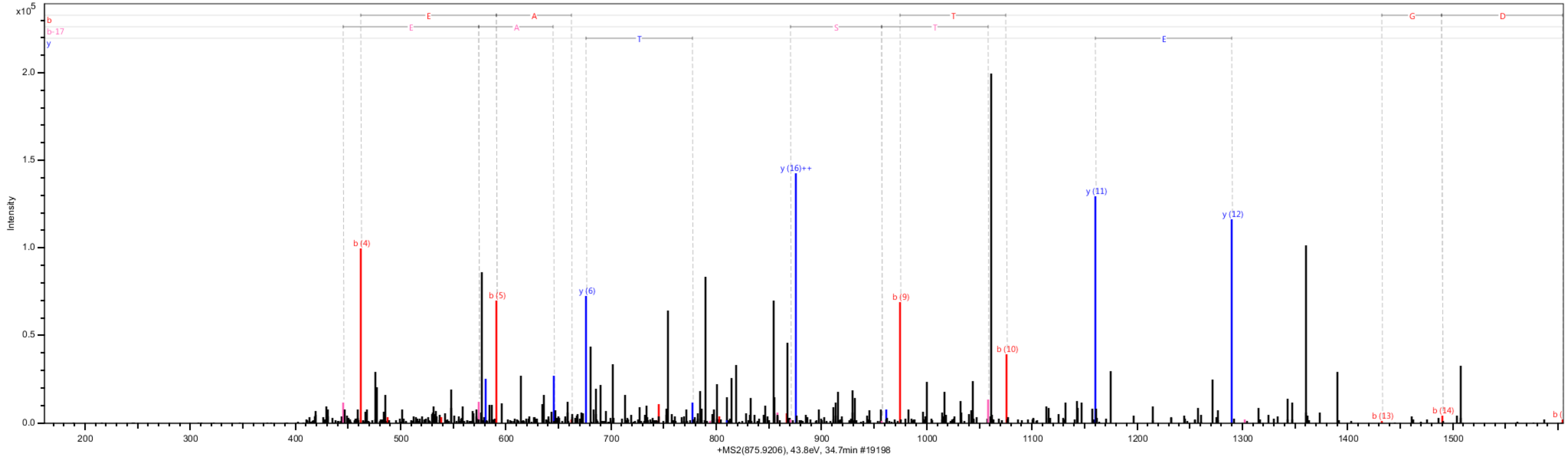

ZN623_HUMAN

Accession: ZN623_HUMAN
Database: SwissProt
MW [kDa] / pI: 61.4 / 8.5
Score: 18.2
Seq. Coverage [%]: 2.6
No. of Peptides: 1

Peptide Match

Sequence: K.ECGKGFSQSSLLIR.H
Score: 18.2
Modifications: Carbamidomethyl: 2

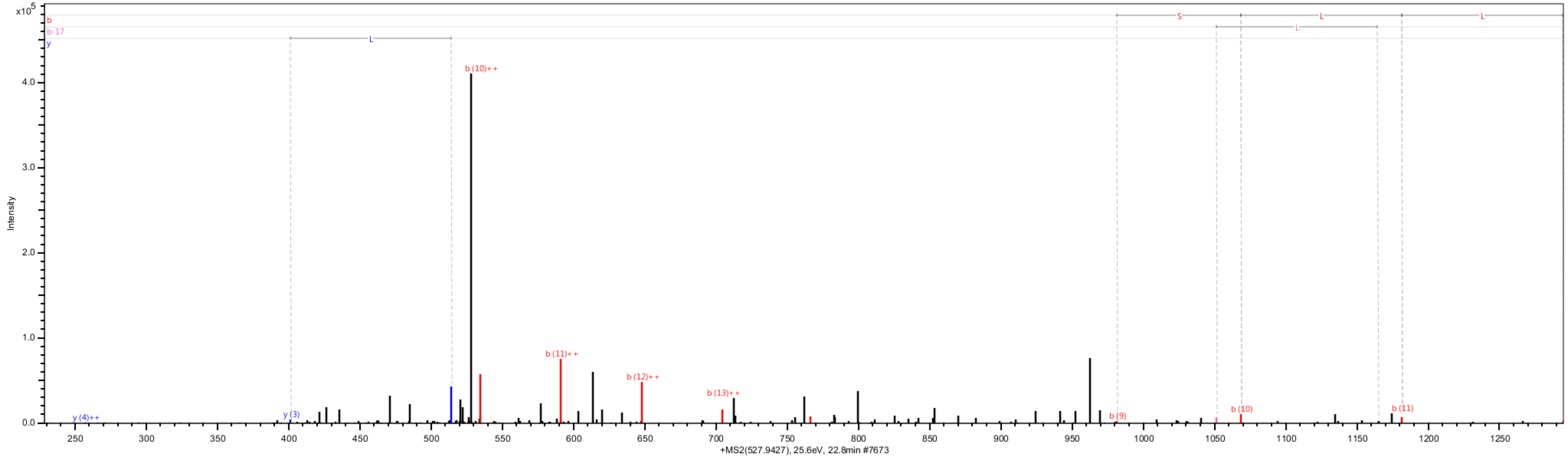

TF3C5_HUMAN

Accession: TF3C5_HUMAN
Database: SwissProt
MW [kDa] / pI: 59.5 / 6.5
Score: 18.2
Seq. Coverage [%]: 2.7
No. of Peptides: 1

Peptide Match

Sequence: K.TSDELRDTMSLMIR.Q
Score: 18.2
Modifications: Oxidation: 9

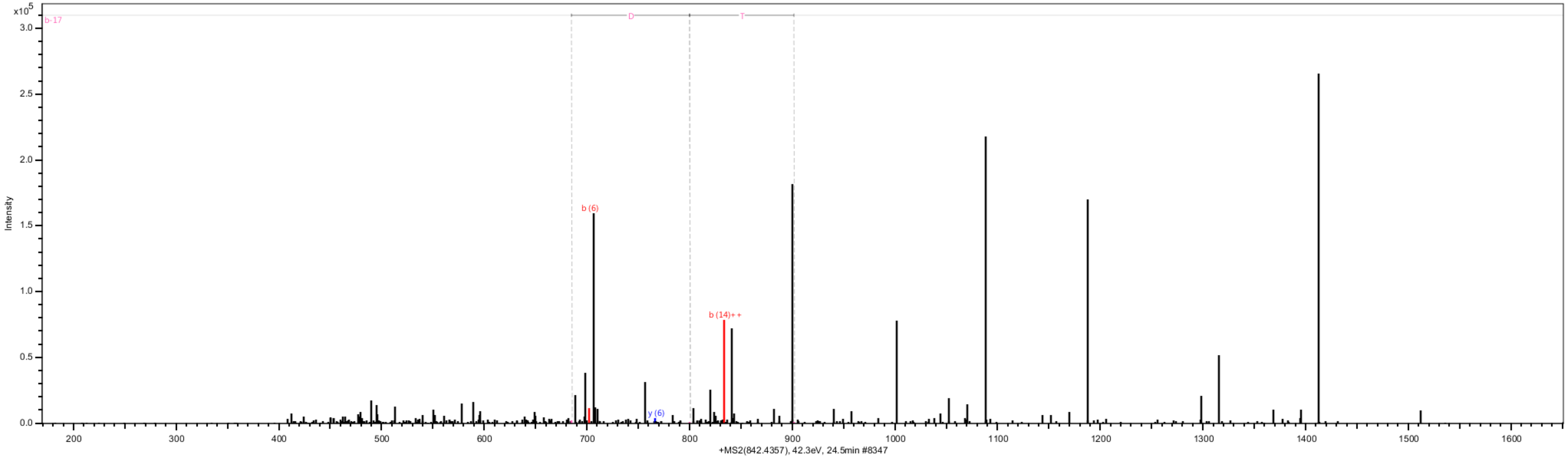

UCP3_HUMAN

Accession: UCP3_HUMAN
Database: SwissProt
MW [kDa] / pI: 34.2 / 9.3
Score: 18.2
Seq. Coverage [%]: 2.6
No. of Peptides: 1

Peptide Match

Sequence: R.QMSFASIR.I
Score: 18.2
Modifications:

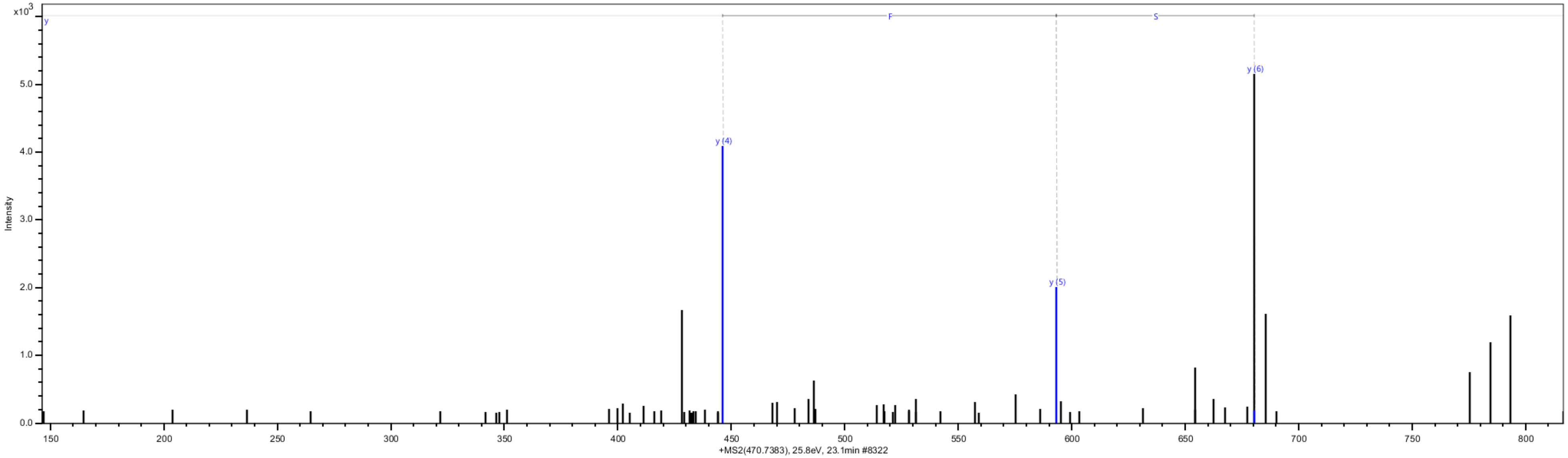

LIPN_HUMAN

**Accession:**	LIPN_HUMAN	**Score:**	18.2
**Database:**	SwissProt	**Seq. Coverage [%]:**	4.5
**MW [kDa] / pI:**	45.5 / 6.4	**No. of Peptides:**	1

Peptide Match

**Sequence:**	K.ILWLICSEFMSLWAGSNK.K	**Score:**	18.2
**Modifications:**	Carbamidomethyl: 6; Oxidation: 10		

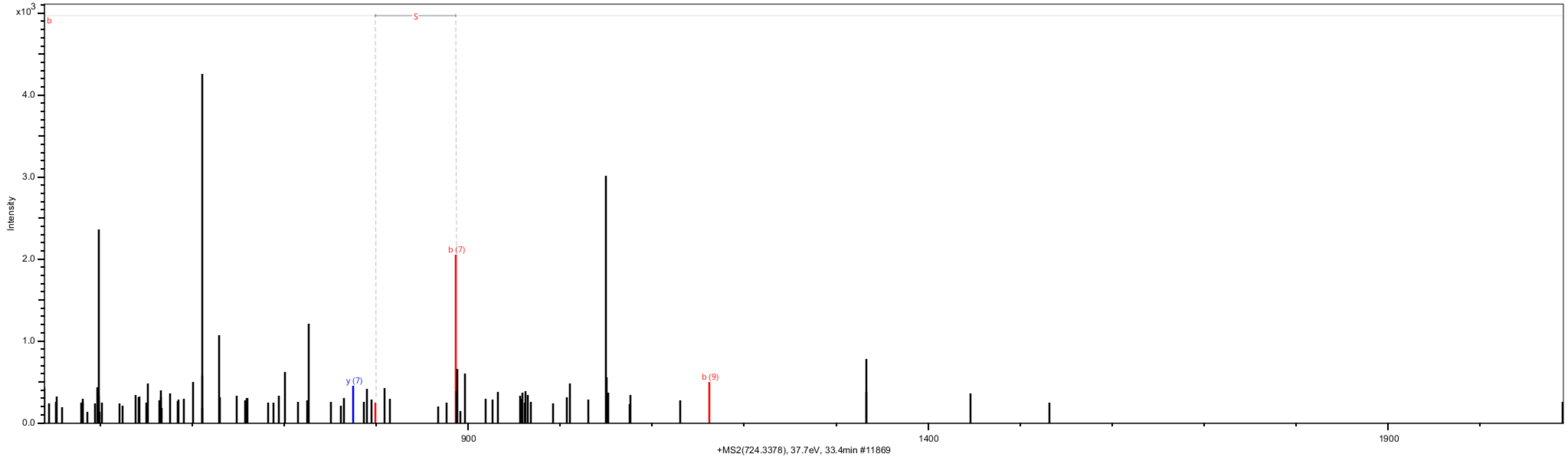

OTU7A_HUMAN

Accession: OTU7A_HUMAN
Database: SwissProt
MW [kDa] / pI: 100.6 / 8.7
Score: 18.2
Seq. Coverage [%]: 0.8
No. of Peptides: 1

Peptide Match

Sequence: K.ALYTMMR.T
Score: 18.2
Modifications:

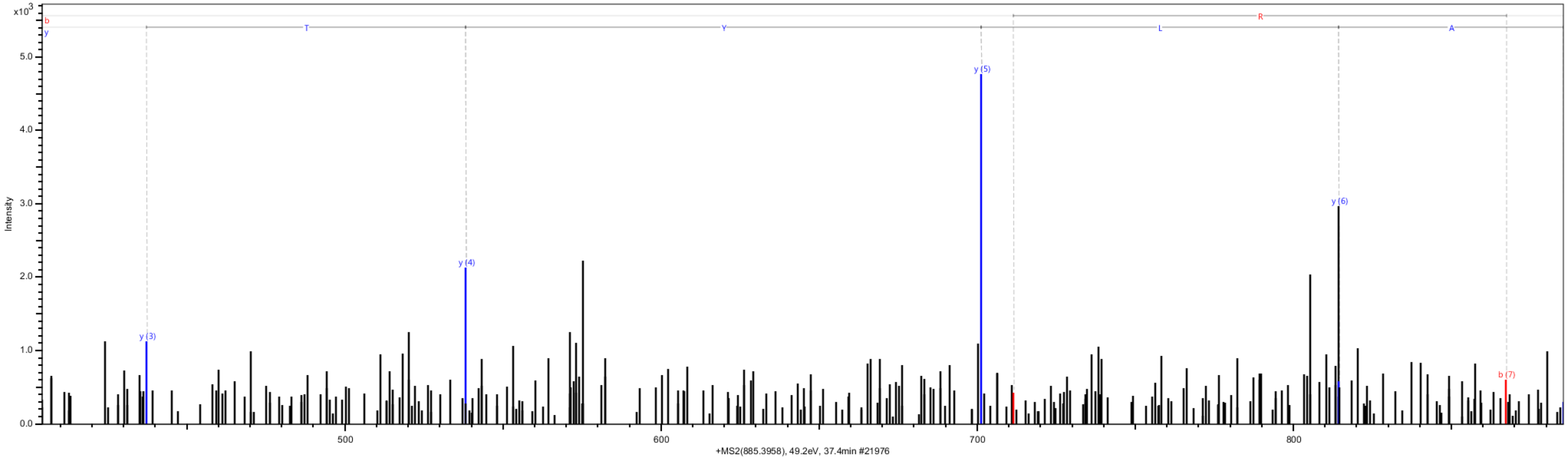

ARH40_HUMAN

Accession: ARH40_HUMAN
Database: SwissProt
MW [kDa] / pI: 164.6 / 5.8
Score: 18.2
Seq. Coverage [%]: 0.5
No. of Peptides: 1

Peptide Match

Sequence: R.LRSTPSSK.L
Score: 18.2
Modifications:

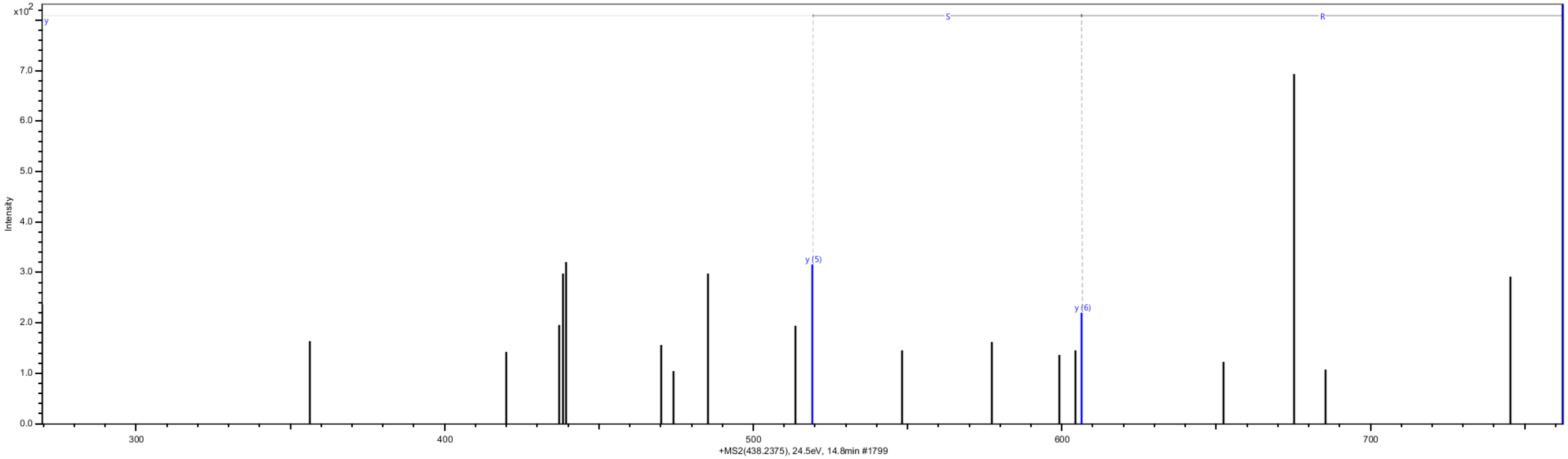

F161B_HUMAN

Accession: F161B_HUMAN
Database: SwissProt
MW [kDa] / pI: 73.6 / 9.4
Score: 18.2
Seq. Coverage [%]: 1.2
No. of Peptides: 1

Peptide Match

Sequence: R.AVQEKETK.I
Score: 18.2
Modifications:

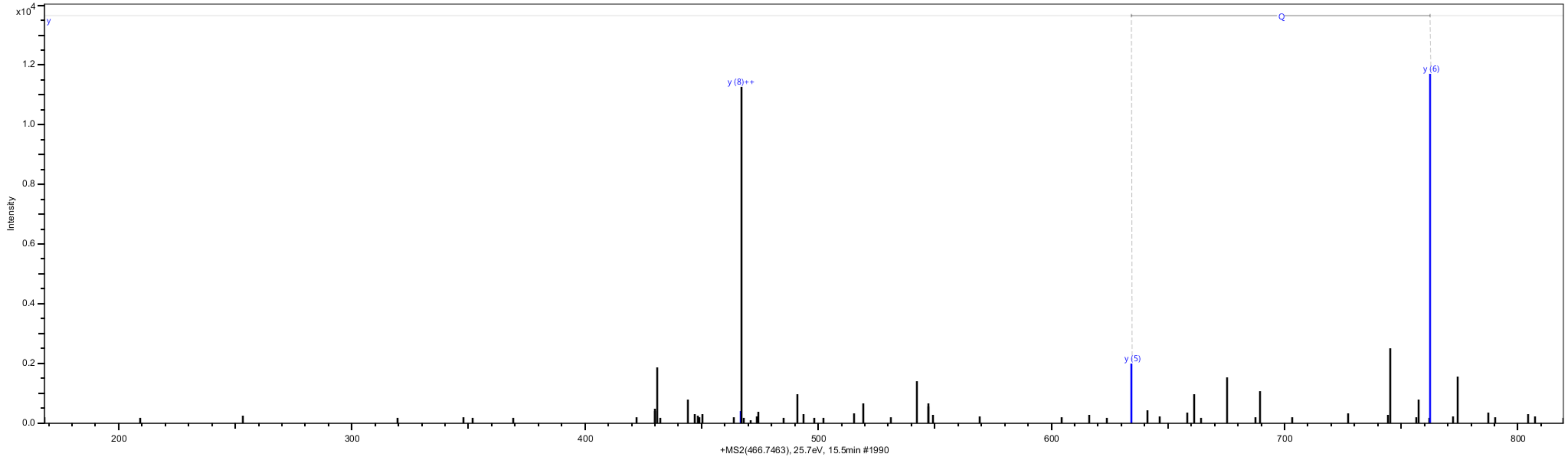

ZHANG_HUMAN

**Accession:**	ZHANG_HUMAN	**Score:**	15.1
**Database:**	SwissProt	**Seq. Coverage [%]:**	2.8
**MW [kDa] / pI:**	37.1 / 5.1	**No. of Peptides:**	1

Peptide Match

**Sequence:**	K.KEYVMGLESR.V	**Score:**	15.1
**Modifications:**	Oxidation: 5		

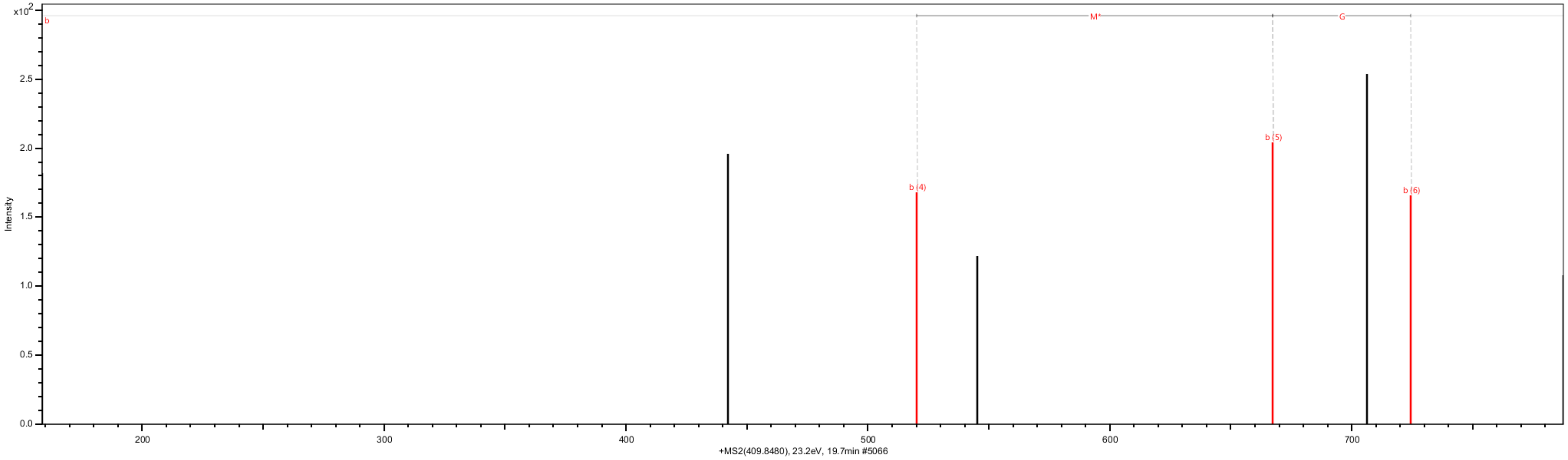

AGAP2_HUMAN

Accession: AGAP2_HUMAN
Database: SwissProt
MW [kDa] / pI: 124.6 / 9.9
Score: 15.1
Seq. Coverage [%]: 1.1
No. of Peptides: 1

Peptide Match

Sequence: R.GETTGSGRAIPIK.Q
Score: 15.1
Modifications:

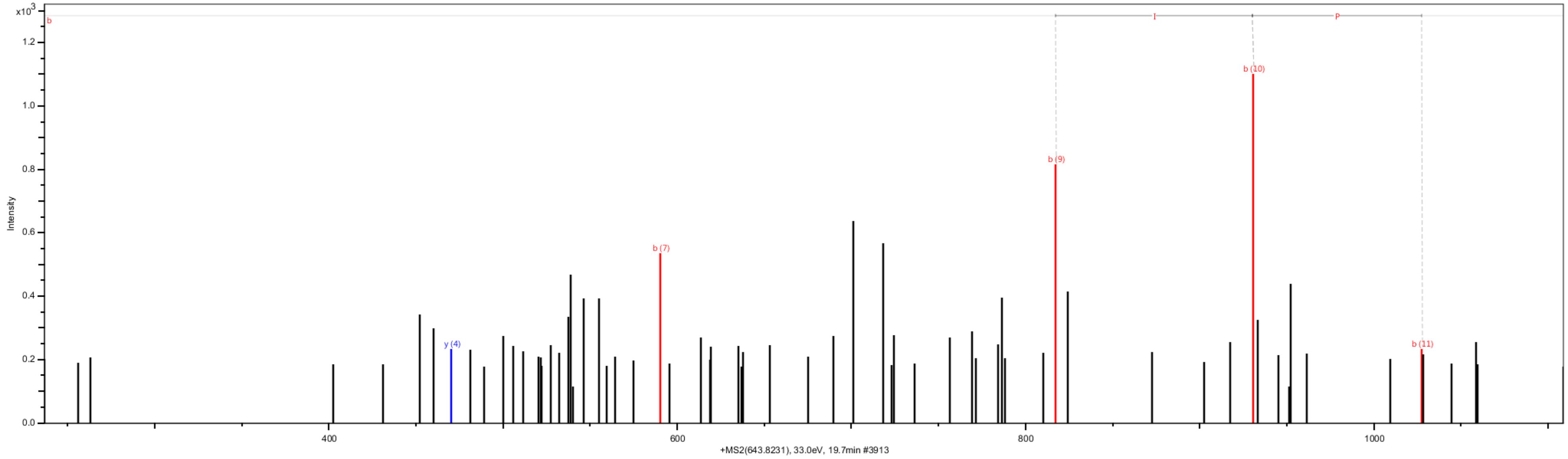

CHIN_HUMAN

Accession: CHIN_HUMAN
Database: SwissProt
MW [kDa] / pI: 53.1 / 6.5
Score: 15.1
Seq. Coverage [%]: 3.5
No. of Peptides: 1

Peptide Match

Sequence: K.IMDPDEQLETLHEALK.L
Score: 15.1
Modifications: Oxidation: 2

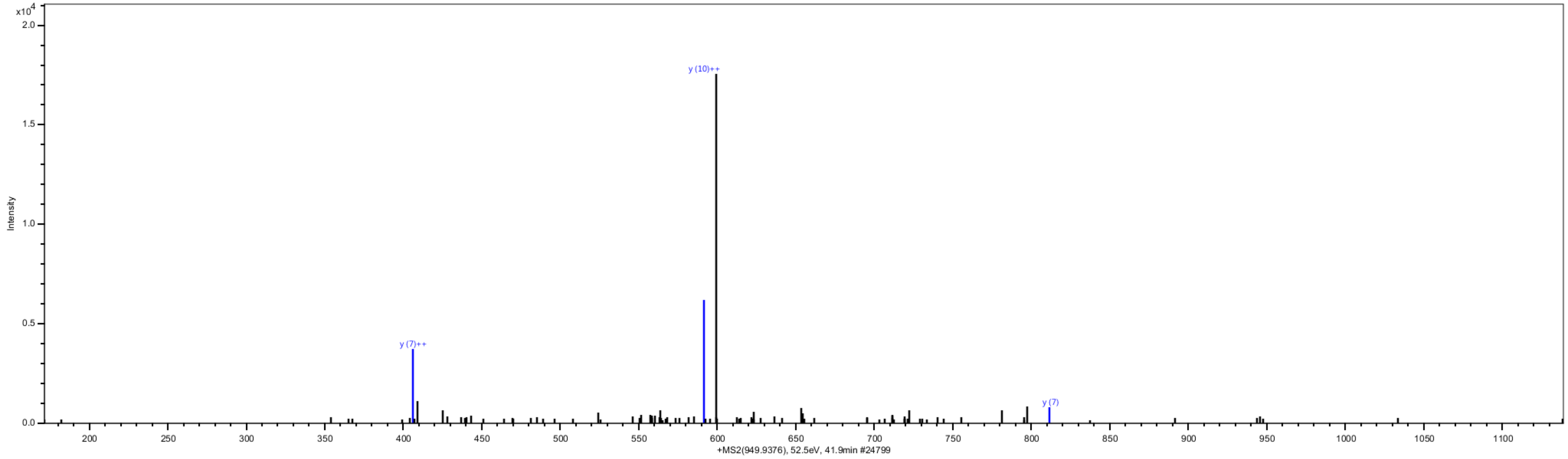

SRARP_HUMAN

**Accession:**	SRARP_HUMAN	**Score:**	15.1
**Database:**	SwissProt	**Seq. Coverage [%]:**	4.7
**MW [kDa] / pI:**	17.6 / 9.2	**No. of Peptides:**	1

Peptide Match

**Sequence:**	R.GLVTPPMK.T	**Score:**	15.1
**Modifications:**			

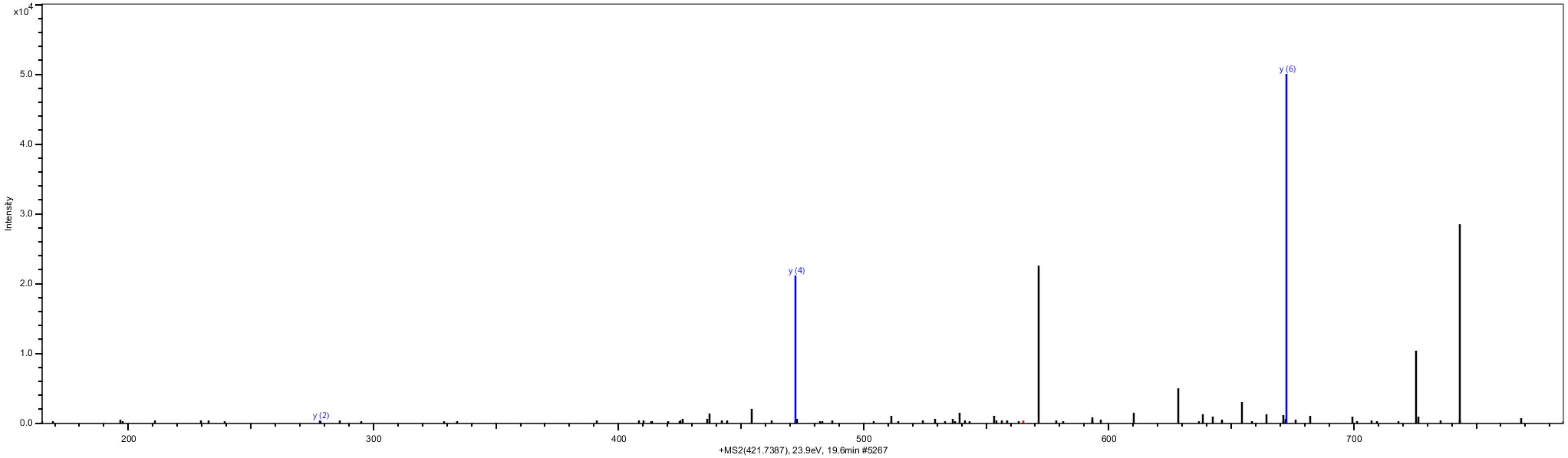

ENAM_HUMAN

Accession: ENAM_HUMAN
Database: SwissProt
MW [kDa] / pI: 128.7 / 6.4
Score: 15.1
Seq. Coverage [%]: 0.8
No. of Peptides: 1

Peptide Match

Sequence: R.WNFFAWERK.Q
Score: 15.1
Modifications:

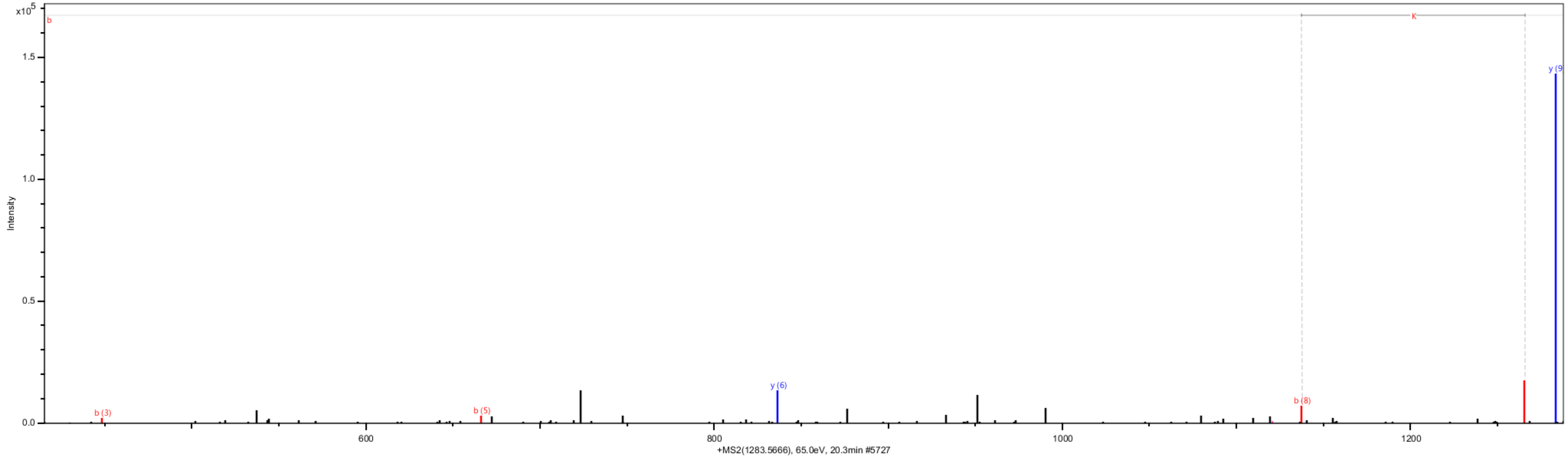

RFX5_HUMAN

Accession: RFX5_HUMAN
Database: SwissProt
MW [kDa] / pI: 65.3 / 9.4
Score: 15.1
Seq. Coverage [%]: 1.5
No. of Peptides: 1

Peptide Match

Sequence: R.EIFPDIKAR.R
Score: 15
Modifications:

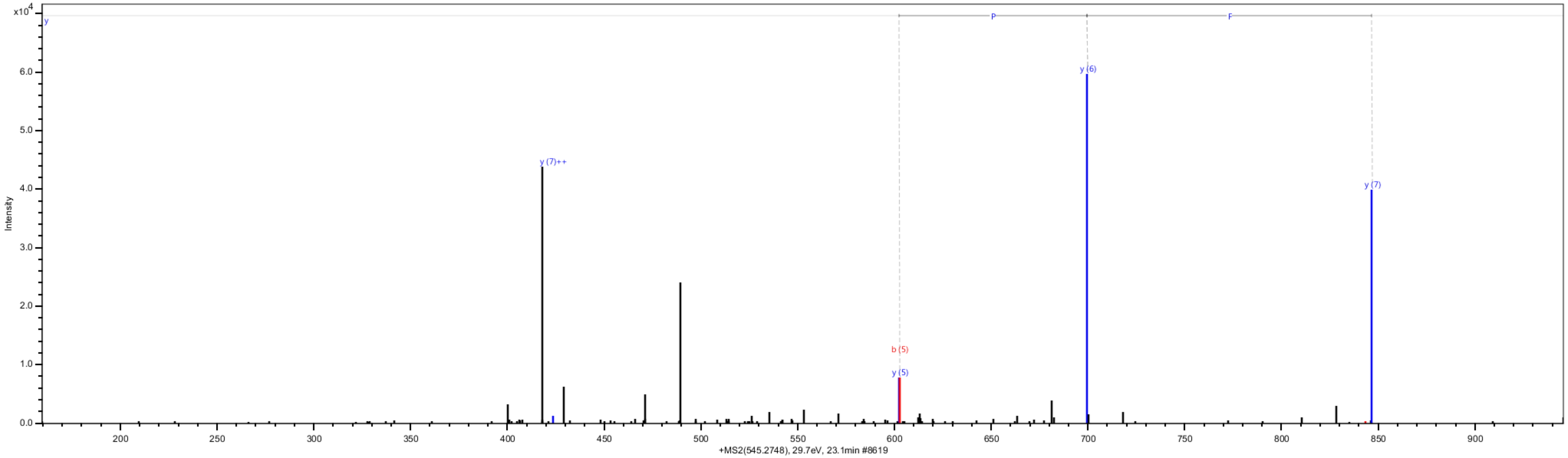

APOC2_HUMAN

Accession: APOC2_HUMAN
Database: SwissProt
MW [kDa] / pI: 11.3 / 4.6
Score: 15.0
Seq. Coverage [%]: 10.9
No. of Peptides: 1

Peptide Match

Sequence: K.ESLSSYWESAK.T
Score: 15
Modifications:

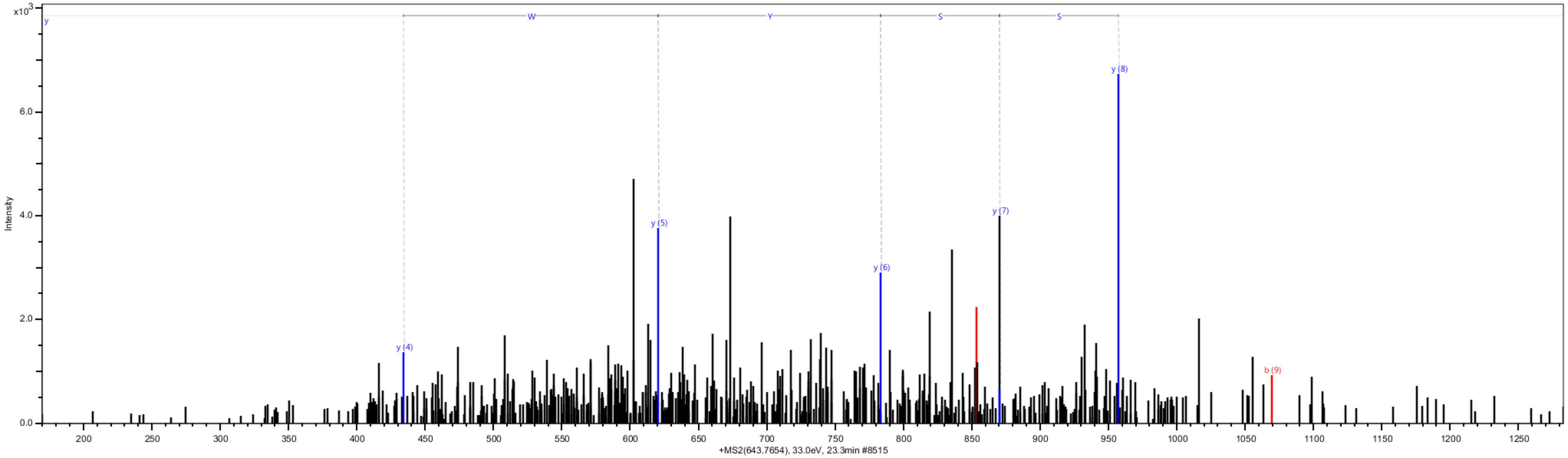

PTHD3_HUMAN

**Accession:**	PTHD3_HUMAN	**Score:**	15.0
**Database:**	SwissProt	**Seq. Coverage [%]:**	1.2
**MW [kDa] / pI:**	86.8 / 5.8	**No. of Peptides:**	1

Peptide Match

**Sequence:**	K.AMRLLYYLK.T	**Score:**	15
**Modifications:**	Oxidation: 2		

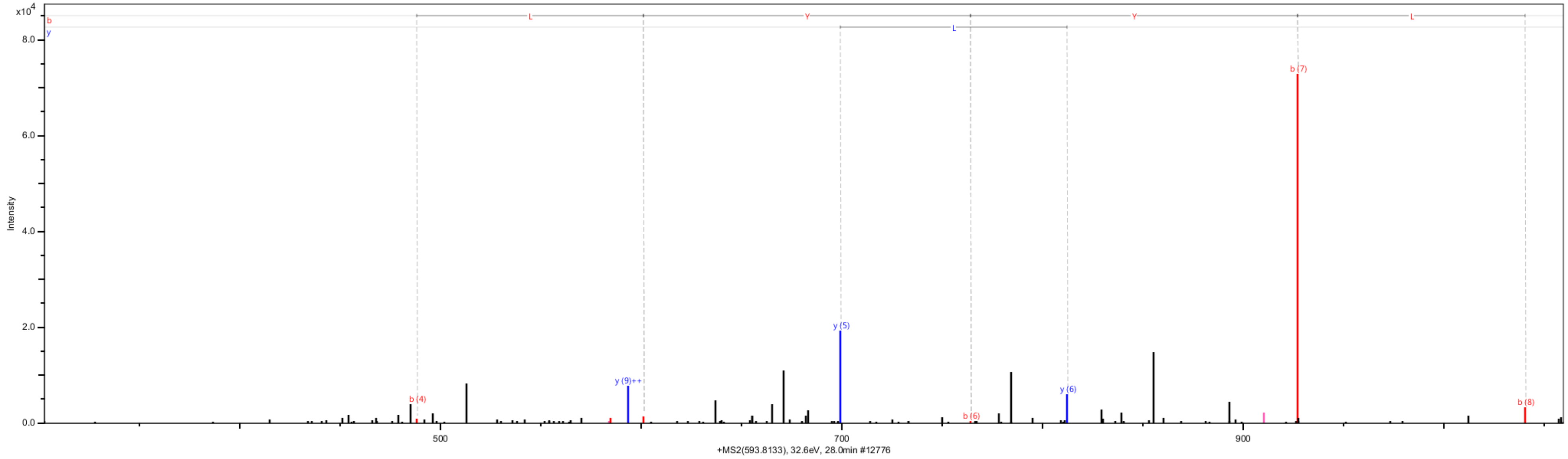

VP13B_HUMAN

Accession: VP13B_HUMAN
Database: SwissProt
MW [kDa] / pI: 448.4 / 6.0
Score: 15.0
Seq. Coverage [%]: 0.3
No. of Peptides: 1

Peptide Match

Sequence: K.TSEAQASAGHK.A
Score: 15
Modifications:

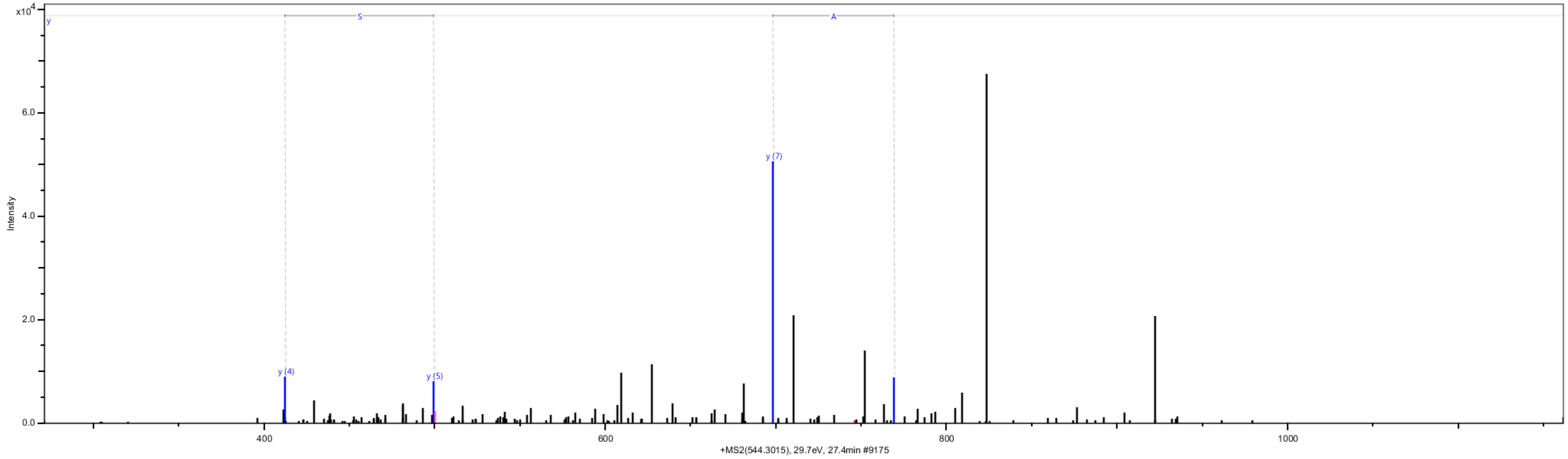

RUXF_HUMAN

**Accession:**	RUXF_HUMAN	**Score:**	15.0
**Database:**	SwissProt	**Seq. Coverage [%]:**	9.3
**MW [kDa] / pI:**	9.7 / 4.6	**No. of Peptides:**	1

Peptide Match

**Sequence:**	K.LKWGMEYK.G	**Score:**	15
**Modifications:**			

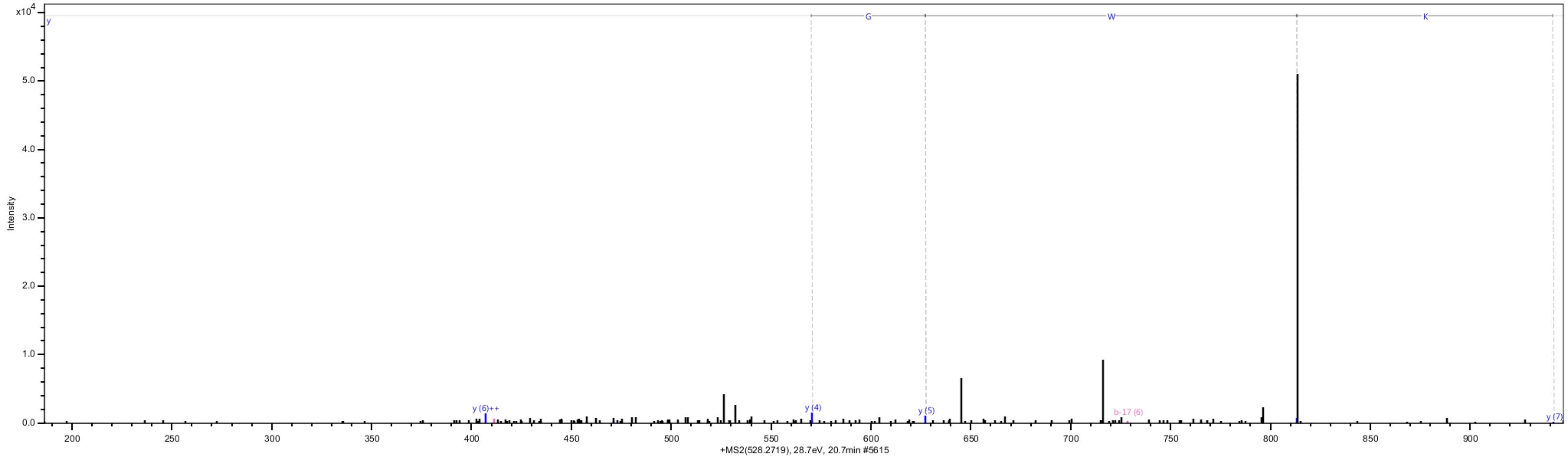

CLPT1_HUMAN

**Accession:**	CLPT1_HUMAN	**Score:**	15.0
**Database:**	SwissProt	**Seq. Coverage [%]:**	1.2
**MW [kDa] / pI:**	76.0 / 5.9	**No. of Peptides:**	1

Peptide Match

**Sequence:**	K.SGFHPDPR.Q	**Score:**	15
**Modifications:**			

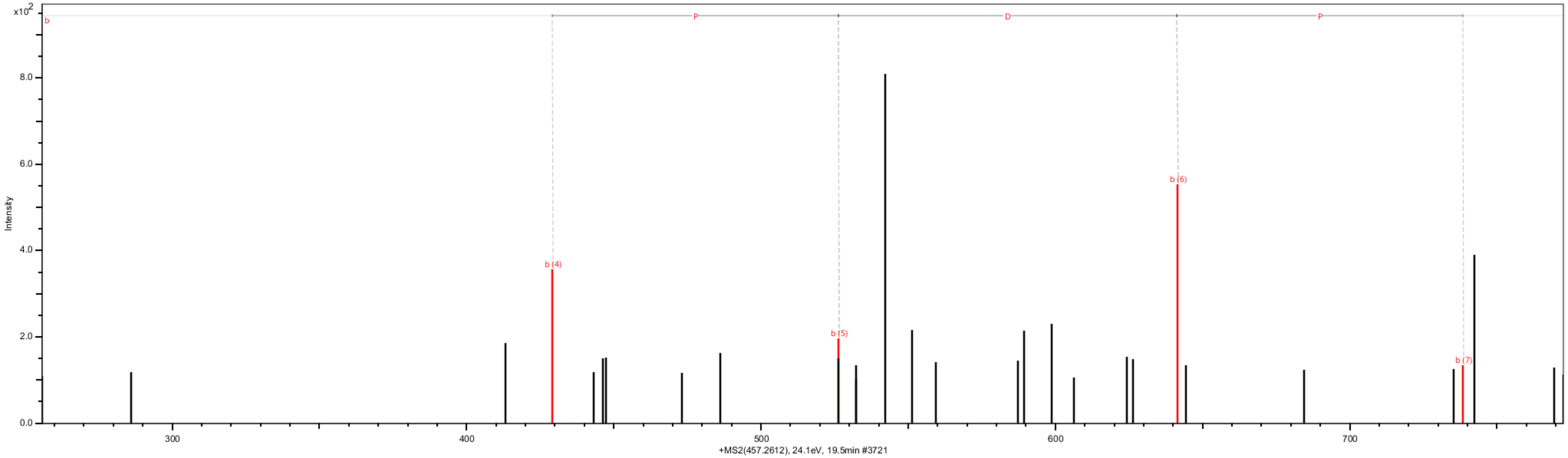

ZN416_HUMAN

Accession: ZN416_HUMAN
Database: SwissProt
MW [kDa] / pI: 67.1 / 8.3
Score: 17.9
Seq. Coverage [%]: 1.7
No. of Peptides: 1

Peptide Match

Sequence: K.AFGSKSTLVR.H
Score: 17.9
Modifications:

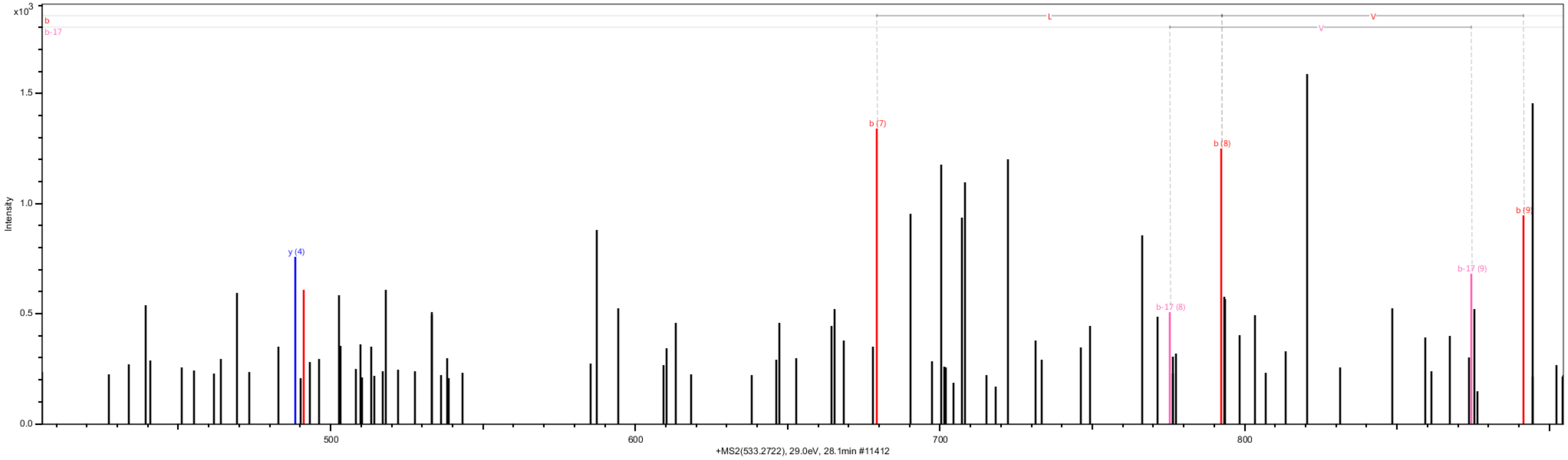

PPR36_HUMAN

Accession: PPR36_HUMAN
Database: SwissProt
MW [kDa] / pI: 49.4 / 9.4
Score: 17.8
Seq. Coverage [%]: 1.7
No. of Peptides: 1

Peptide Match

Sequence: R.LGMWYWK.D
Score: 17.8
Modifications:

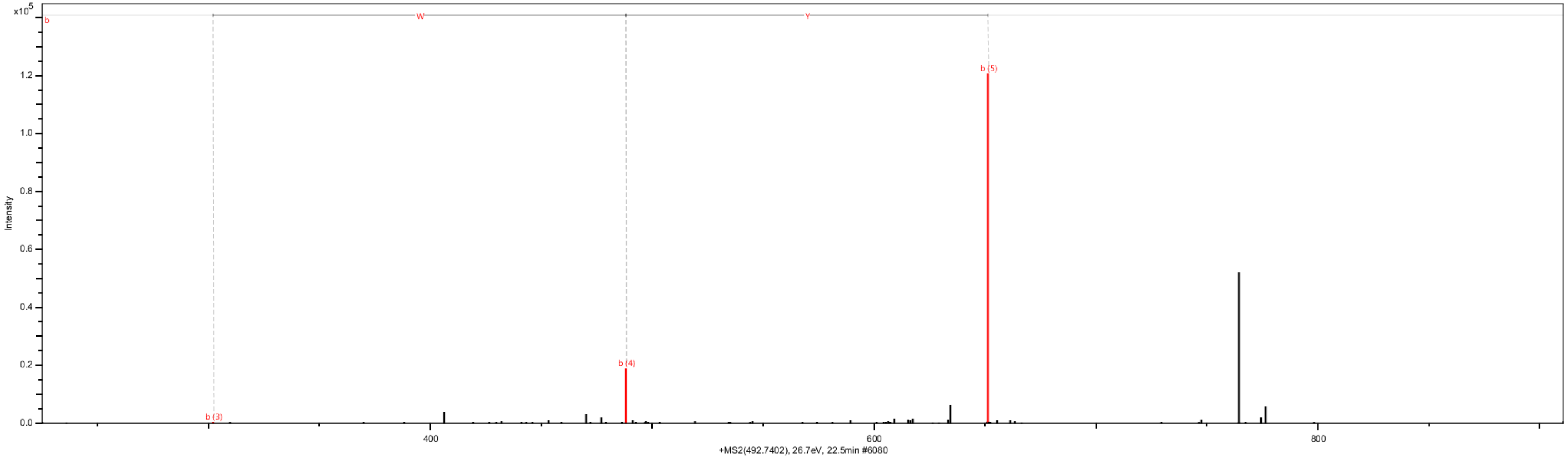

KS6A6_HUMAN

Accession: KS6A6_HUMAN
Database: SwissProt
MW [kDa] / pI: 83.8 / 5.9
Score: 17.8
Seq. Coverage [%]: 1.2
No. of Peptides: 1

Peptide Match

Sequence: R.NETMNMILK.A
Score: 17.8
Modifications: Oxidation: 4, 6

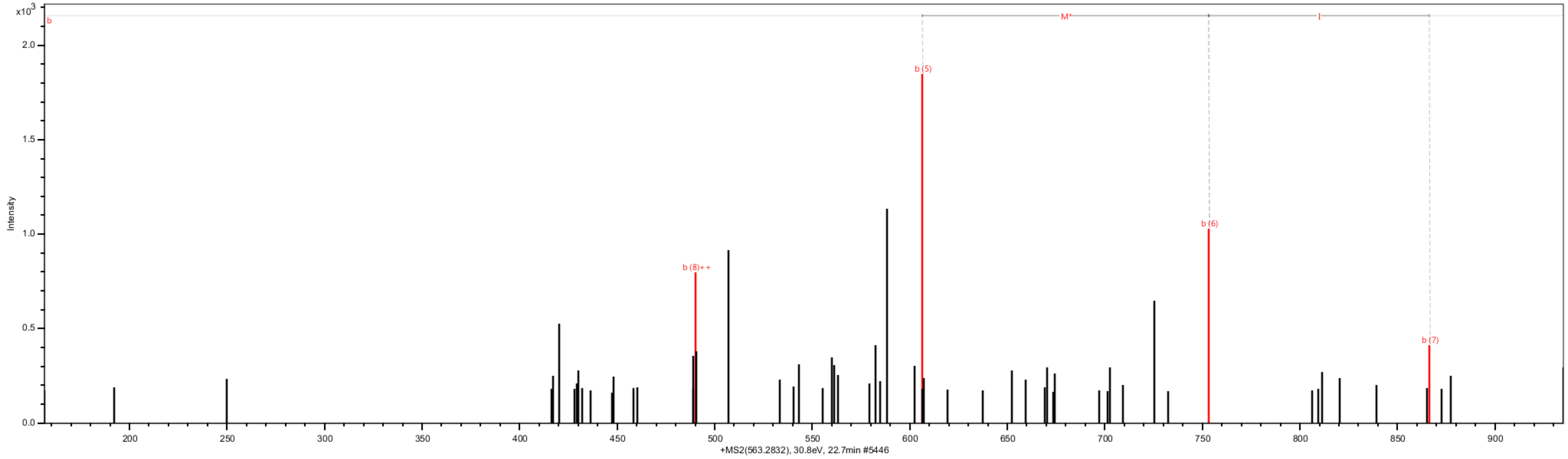

RS6_HUMAN

**Accession:**	RS6_HUMAN	**Score:**	17.8
**Database:**	SwissProt	**Seq. Coverage [%]:**	5.2
**MW [kDa] / pI:**	28.7 / 10.9	**No. of Peptides:**	1

Peptide Match

**Sequence:**	R.ISGGNDKQGFPMK.Q	**Score:**	17.8
**Modifications:**			

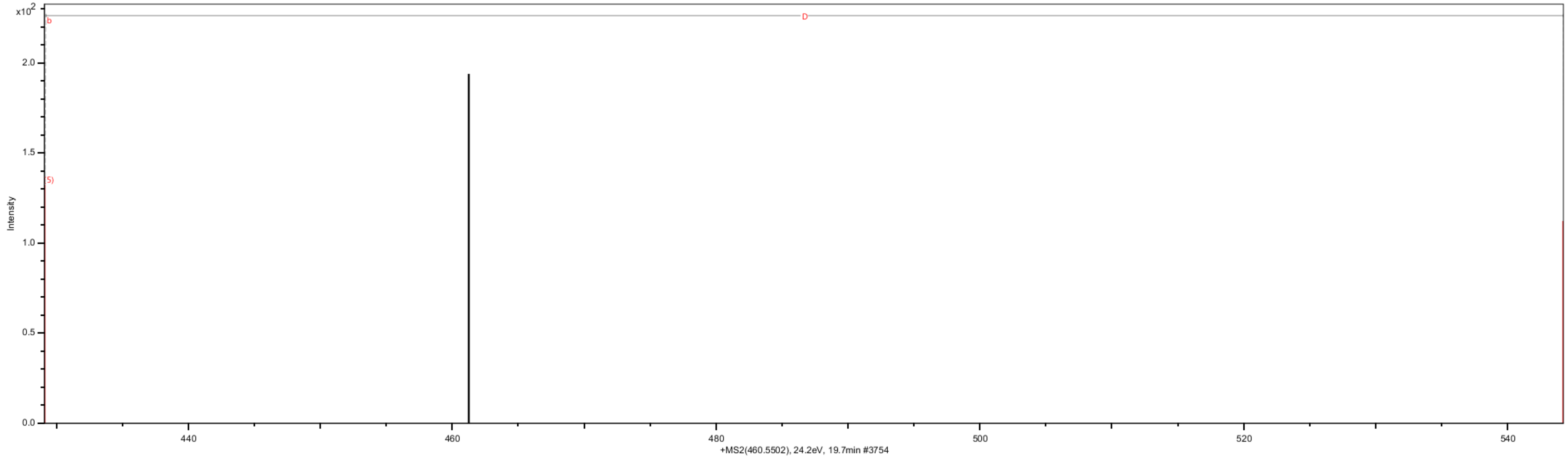

DC8L1_HUMAN

Accession: DC8L1_HUMAN
Database: SwissProt
MW [kDa] / pI: 67.3 / 4.7
Score: 17.7
Seq. Coverage [%]: 1.5
No. of Peptides: 1

Peptide Match

Sequence: R.LASSGDDLR.V
Score: 17.7
Modifications:

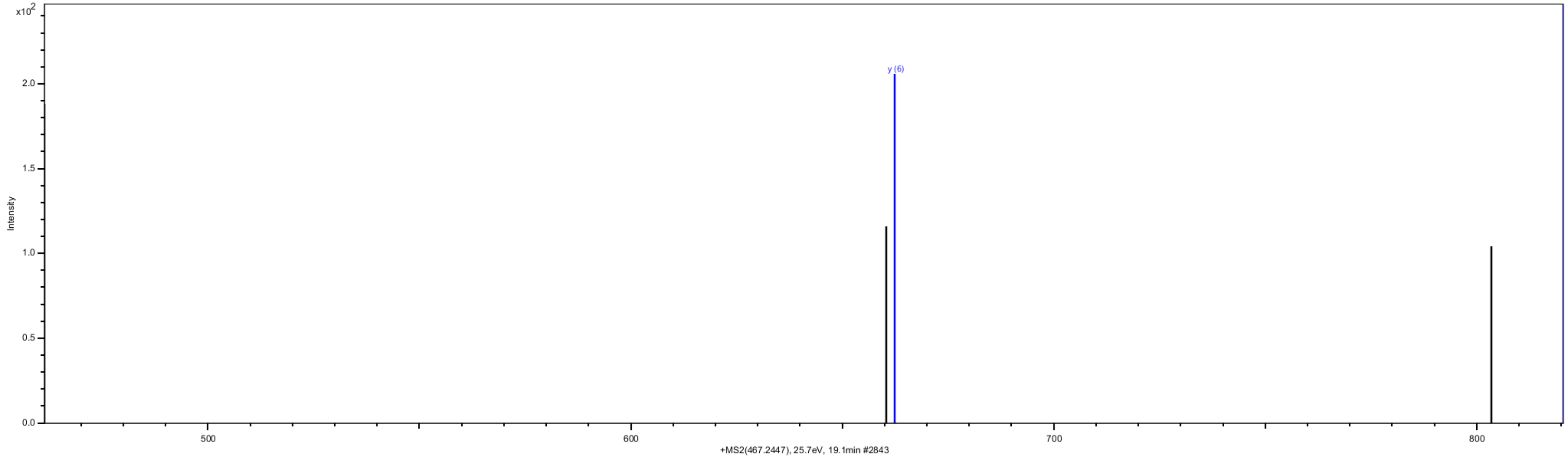

RSF1_HUMAN

**Accession:**	RSF1_HUMAN	**Score:**	17.7
**Database:**	SwissProt	**Seq. Coverage [%]:**	0.7
**MW [kDa] / pI:**	163.7 / 4.9	**No. of Peptides:**	1

Peptide Match

**Sequence:**	K.RPPILECLEK.L	**Score:**	17.7
**Modifications:**	Carbamidomethyl: 7		

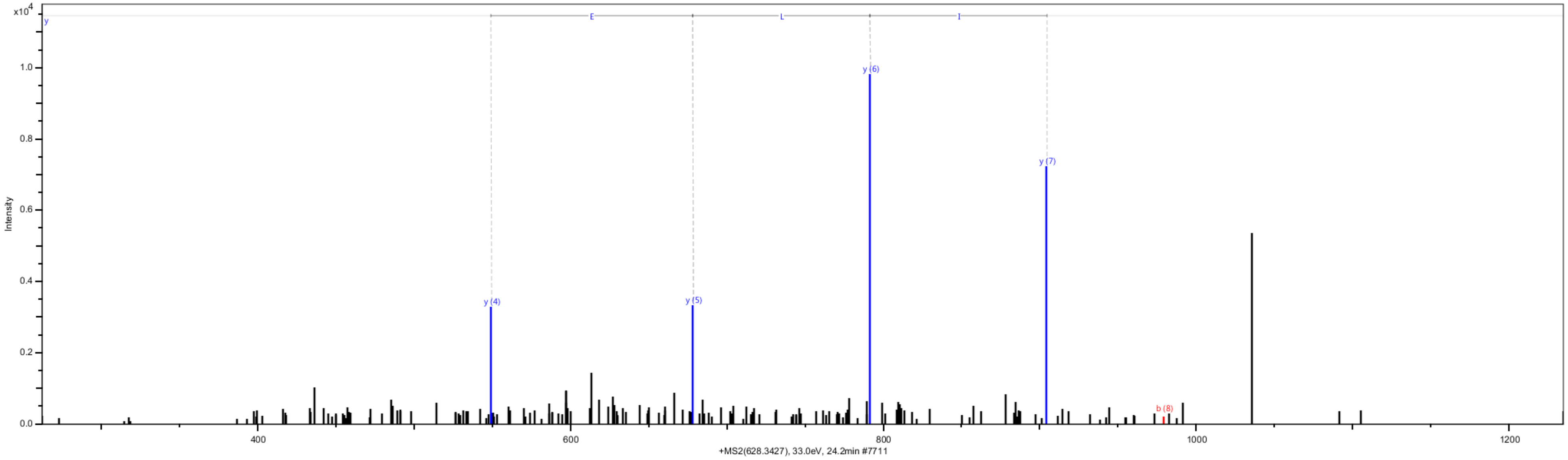

FLNC_HUMAN

**Accession:**	FLNC_HUMAN	**Score:**	17.7
**Database:**	SwissProt	**Seq. Coverage [%]:**	0.4
**MW [kDa] / pI:**	290.8 / 5.6	**No. of Peptides:**	1

Peptide Match

**Sequence:**	K.IKVLPAHDASK.V	**Score:**	17.7
**Modifications:**			

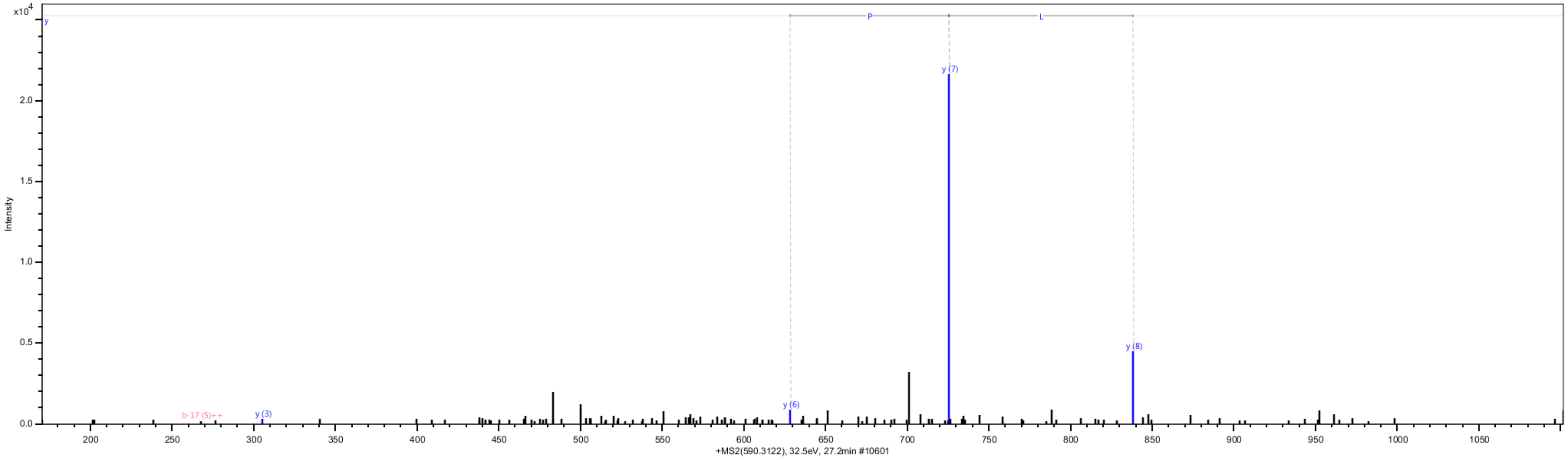

SDCB2_HUMAN

**Accession:**	SDCB2_HUMAN	**Score:**	17.6
**Database:**	SwissProt	**Seq. Coverage [%]:**	3.8
**MW [kDa] / pI:**	31.6 / 9.2	**No. of Peptides:**	1

Peptide Match

**Sequence:**	R.FGDQLLQIDGR.D	**Score:**	17.6
**Modifications:**			

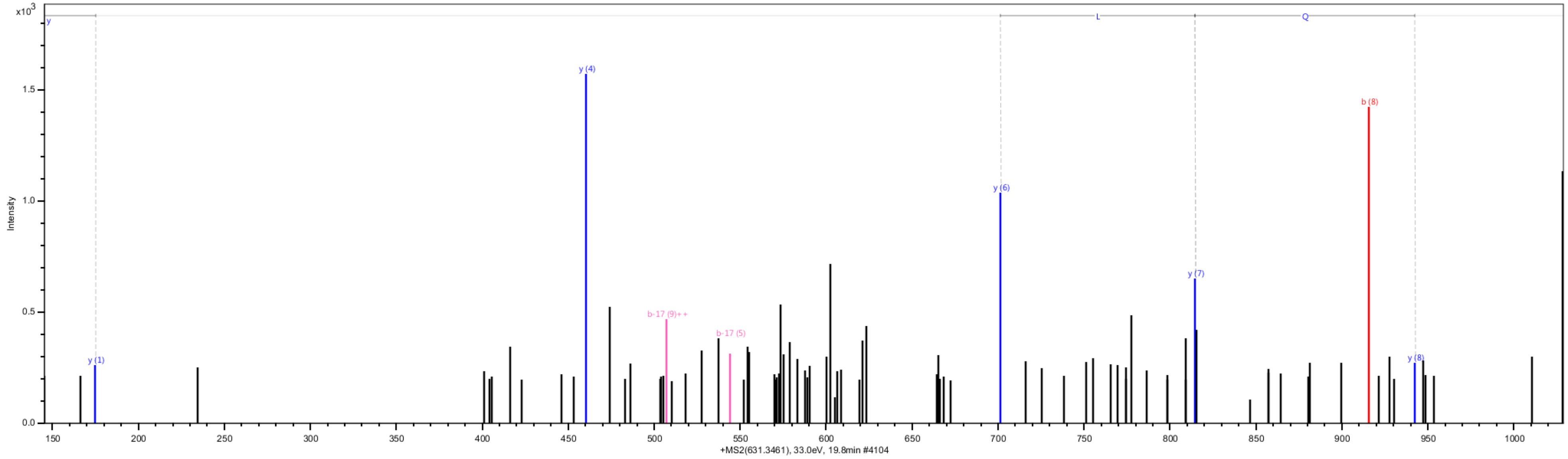

ZN100_HUMAN

Accession: ZN100_HUMAN
Database: SwissProt
MW [kDa] / pI: 62.7 / 9.2
Score: 15.4
Seq. Coverage [%]: 3.1
No. of Peptides: 1

Peptide Match

Sequence: R.YGMCPLKGASGCPGAER.S
Score: 15.4
Modifications: Carbamidomethyl: 4, 12; Oxidation: 3

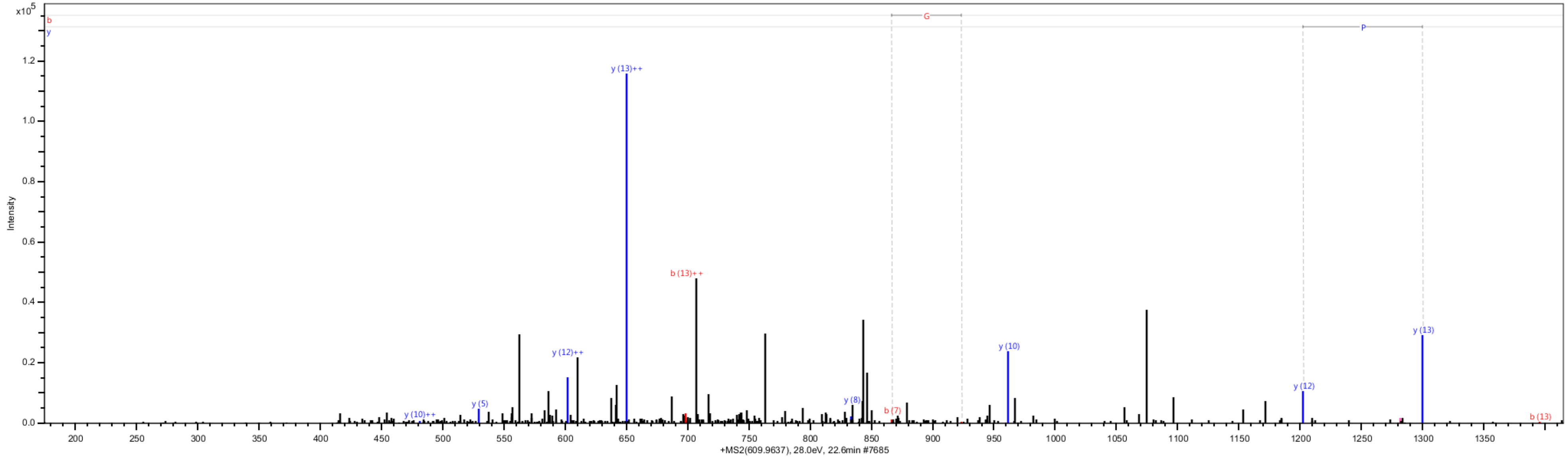

IL36G_HUMAN

Accession: IL36G_HUMAN
Database: SwissProt
MW [kDa] / pI: 18.7 / 5.0
Score: 15.3
Seq. Coverage [%]: 5.3
No. of Peptides: 1

Peptide Match

Sequence: K.YPEALEQGR.G
Score: 15.3
Modifications:

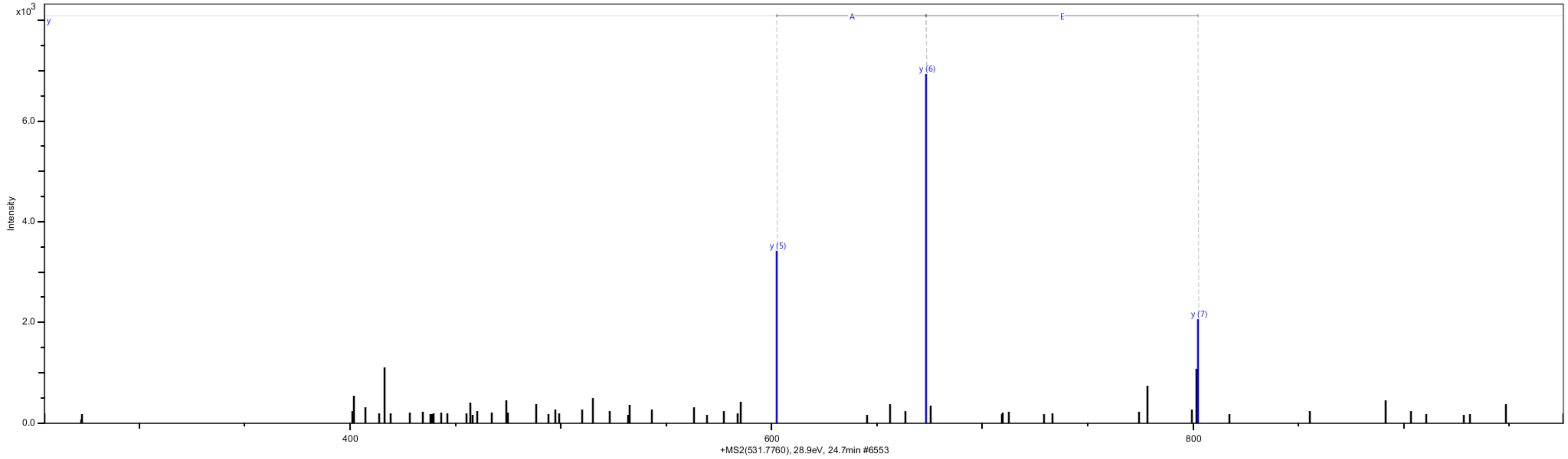

CN37_HUMAN

Accession: CN37_HUMAN
Database: SwissProt
MW [kDa] / pI: 47.5 / 9.2
Score: 15.3
Seq. Coverage [%]: 3.3
No. of Peptides: 1

Peptide Match

Sequence: K.NMEVRAIFTGYYGK.G
Score: 15.3
Modifications:

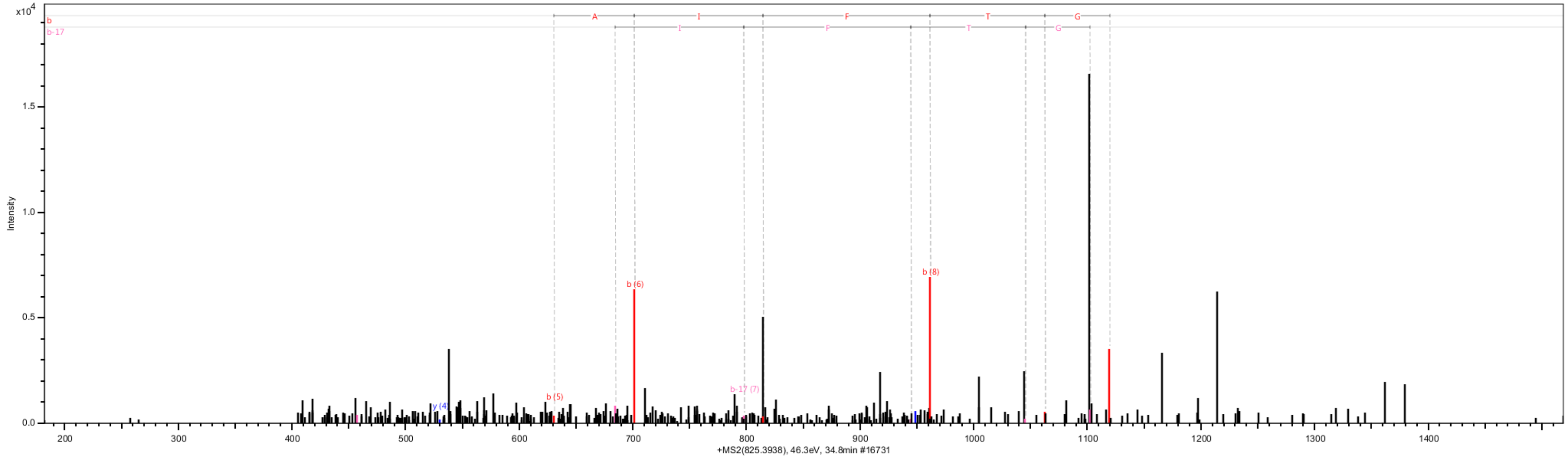

CSCL1_HUMAN

Accession: CSCL1_HUMAN
Database: SwissProt
MW [kDa] / pI: 92.1 / 7.0
Score: 15.3
Seq. Coverage [%]: 2.2
No. of Peptides: 1

Peptide Match

Sequence: -.MMDSPFLELWQSKAVSIR.E
Score: 15.3
Modifications: Oxidation: 1

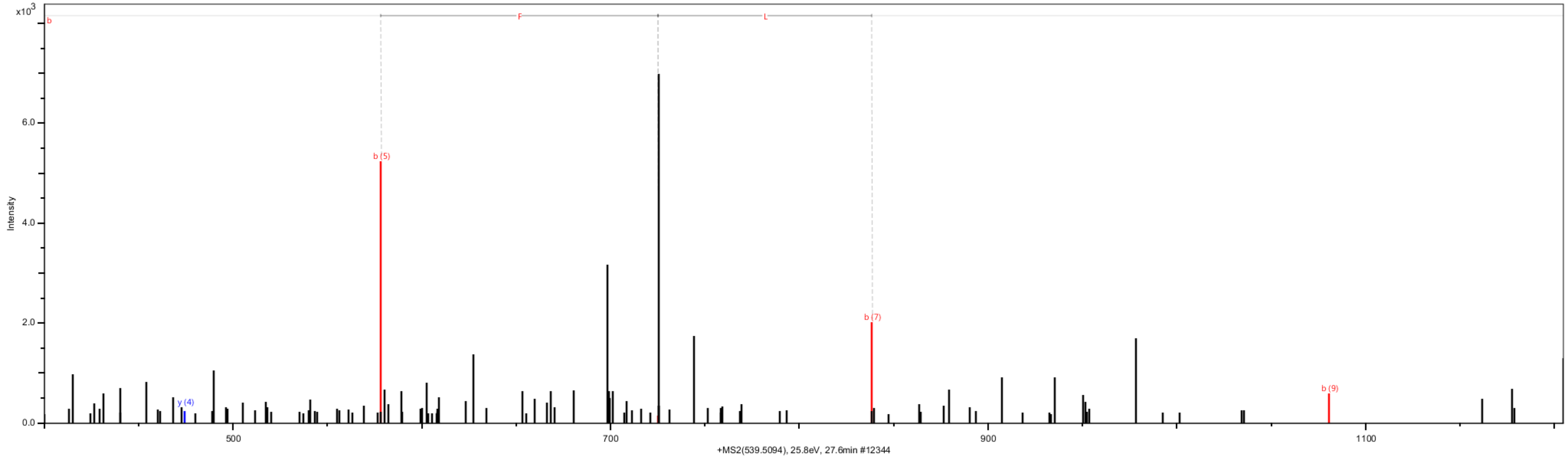

RAB17_HUMAN

Accession: RAB17_HUMAN
Database: SwissProt
MW [kDa] / pI: 23.5 / 7.7
Score: 15.3
Seq. Coverage [%]: 4.7
No. of Peptides: 1

Peptide Match

Sequence: K.VVDVGATSLK.L
Score: 15.3
Modifications:

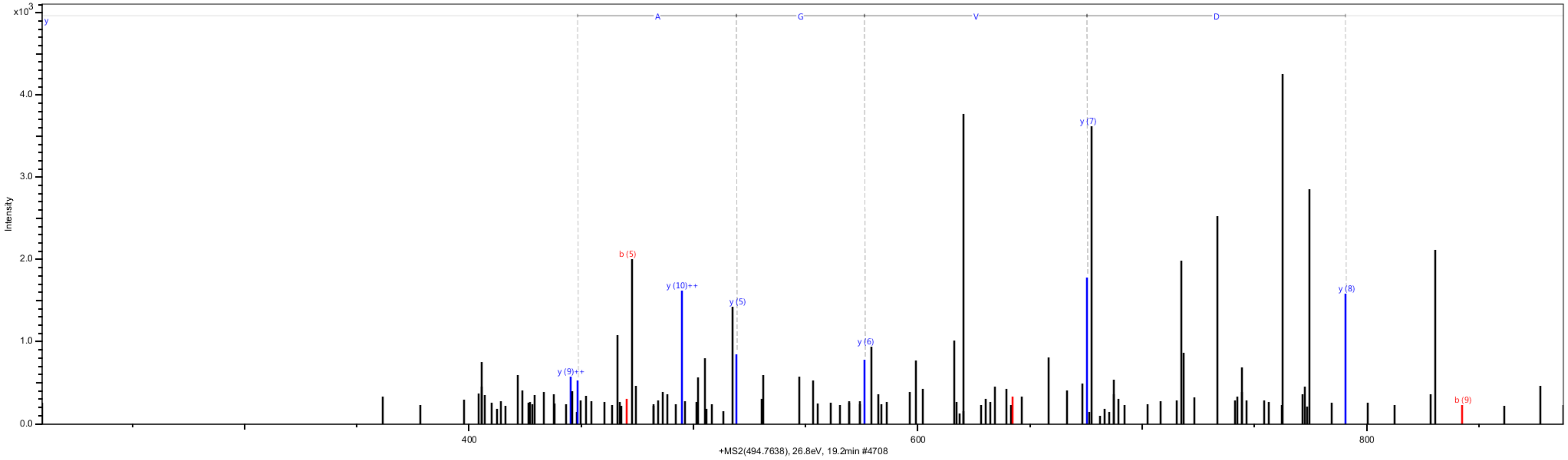

CHPF2_HUMAN

Accession: CHPF2_HUMAN
Database: SwissProt
MW [kDa] / pI: 85.9 / 7.9
Score: 15.3
Seq. Coverage [%]: 1.3
No. of Peptides: 1

Peptide Match

Sequence: R.GAPIGGRFDR.Q
Score: 15.3
Modifications:

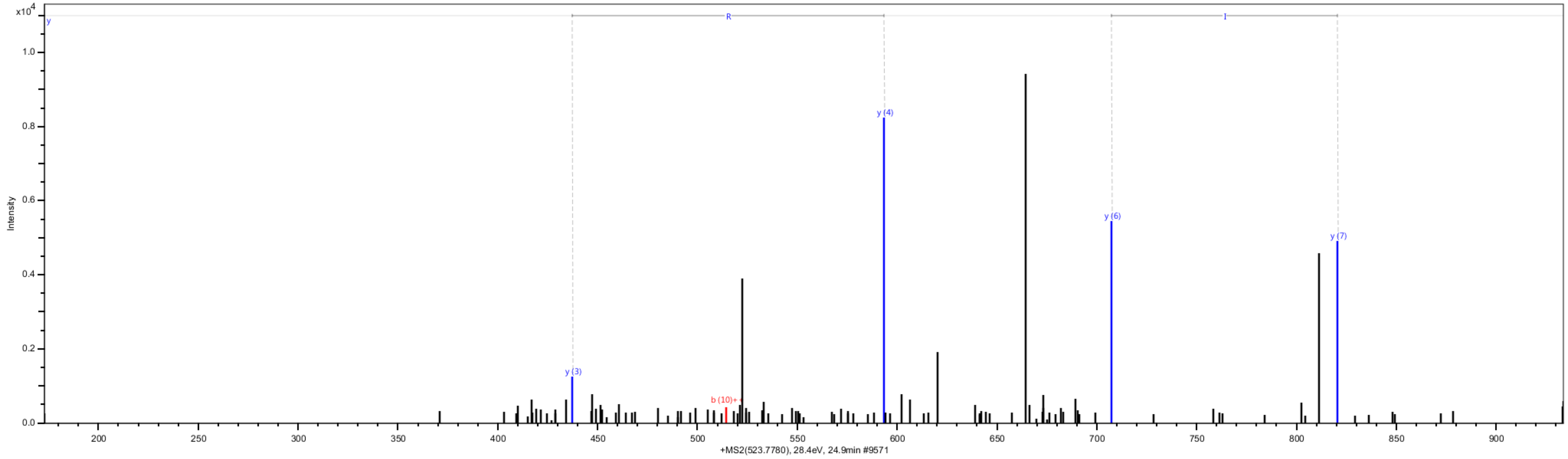

ZNF41_HUMAN

Accession: ZNF41_HUMAN
Database: SwissProt
MW [kDa] / pI: 93.7 / 9.1
Score: 51.1
Seq. Coverage [%]: 1.0
No. of Peptides: 1

Peptide Match

Sequence: R.TNLTTHQK.T
Score: 19.9
Modifications:

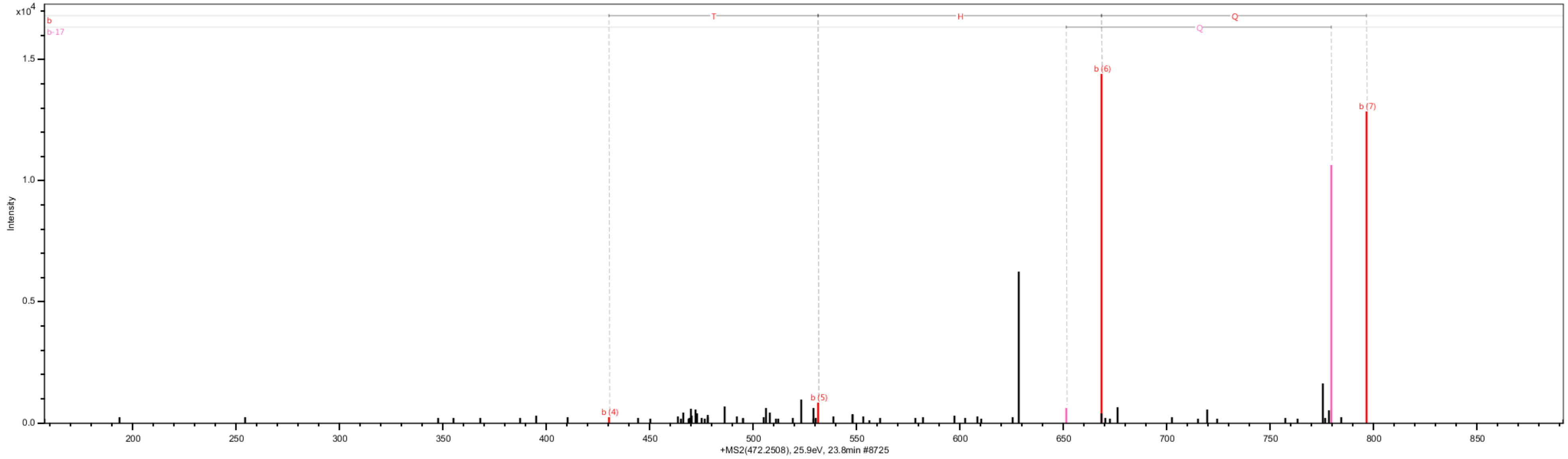

UN45A_HUMAN

Accession: UN45A_HUMAN
Database: SwissProt
MW [kDa] / pI: 103.0 / 5.8
Score: 49.7
Seq. Coverage [%]: 1.1
No. of Peptides: 1

Peptide Match

Sequence: K.ASQNLVVLAR.E
Score: 24.1
Modifications:

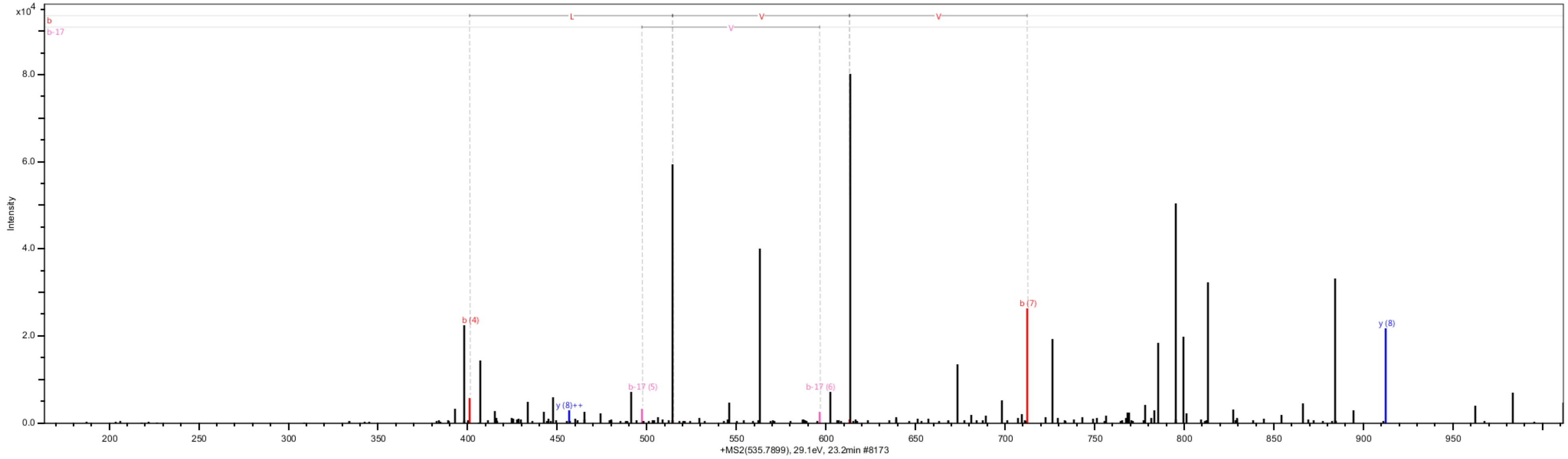

EPG5_HUMAN

**Accession:**	EPG5_HUMAN	**Score:**	49.0
**Database:**	SwissProt	**Seq. Coverage [%]:**	0.6
**MW [kDa] / pI:**	292.3 / 6.0	**No. of Peptides:**	2

Peptide Match

**Sequence:**	R.SSAIHQQGR.A	**Score:**	31.1
**Modifications:**			

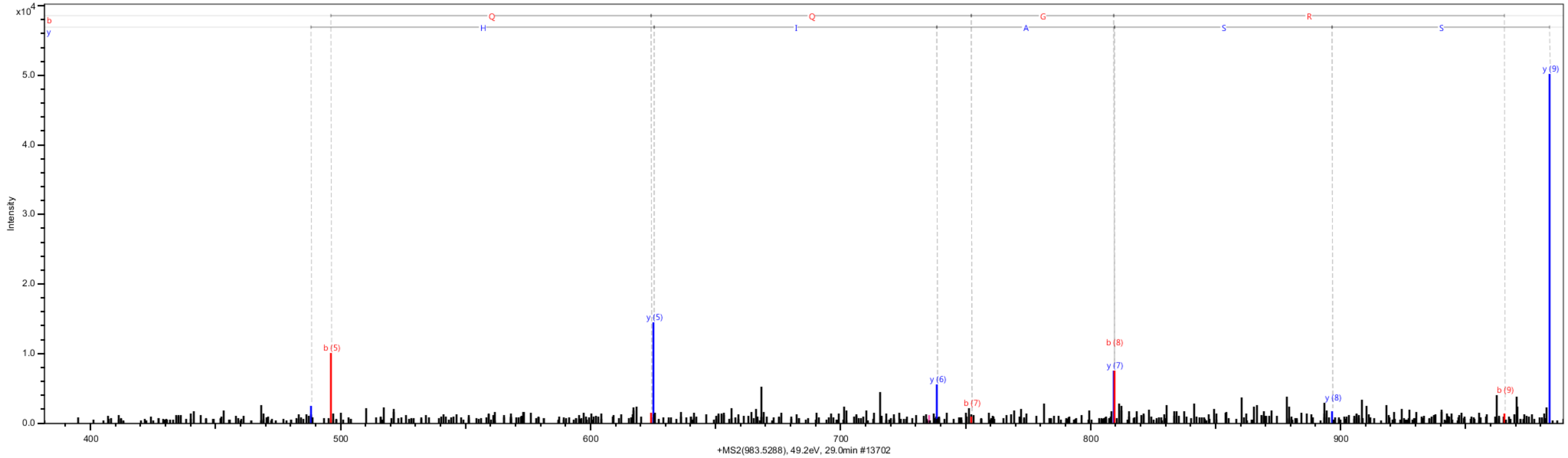

Peptide Match

**Sequence:**	K.LVSVLQR.V	**Score:**	17.9
**Modifications:**			

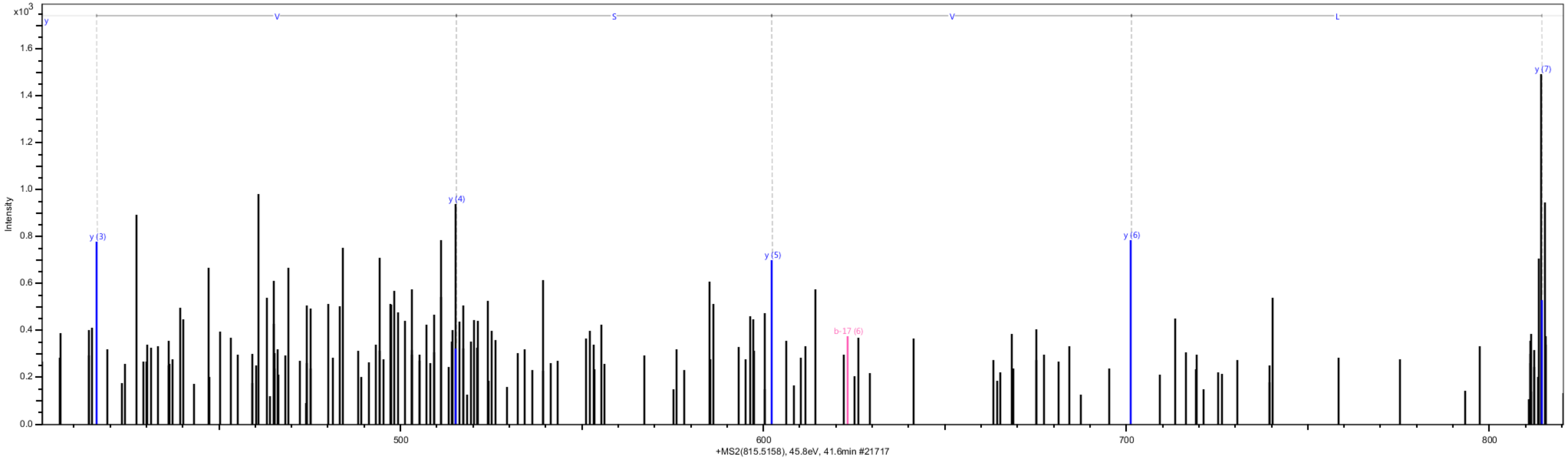
x10^3
Intensity
y
V
S
V
L
y (3)
y (4)
y (5)
b-17 (6)
y (6)
y (7)
1.6
1.4
1.2
1.0
0.8
0.6
0.4
0.2
0.0
500
600
700
800
+MS2(815.5158), 45.8eV, 41.6min #21717

AKAP9_HUMAN

Accession: AKAP9_HUMAN
Database: SwissProt
MW [kDa] / pI: 452.7 / 4.9
Score: 48.0
Seq. Coverage [%]: 0.2
No. of Peptides: 1

Peptide Match

Sequence: R.LQMEAQR.I
Score: 17.4
Modifications:

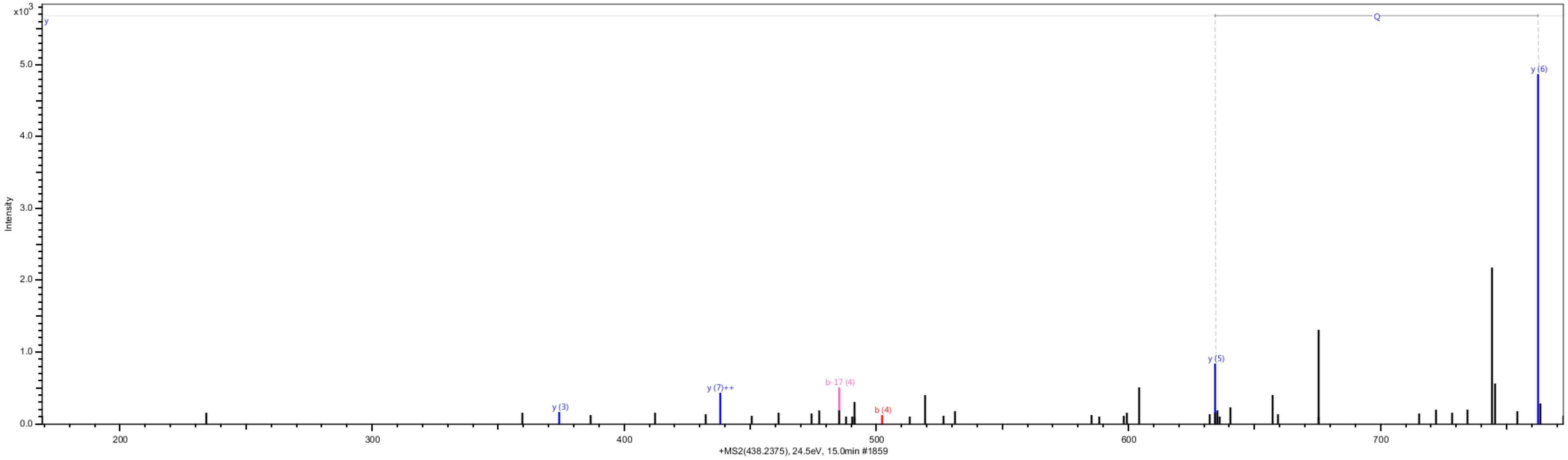

CSCL2_HUMAN

**Accession:**	CSCL2_HUMAN	**Score:**	47.2
**Database:**	SwissProt	**Seq. Coverage [%]:**	1.9
**MW [kDa] / pI:**	94.9 / 7.2	**No. of Peptides:**	2

Peptide Match

**Sequence:**	K.VAWDYGRLALVTDADR.L	**Score:**	19
**Modifications:**			

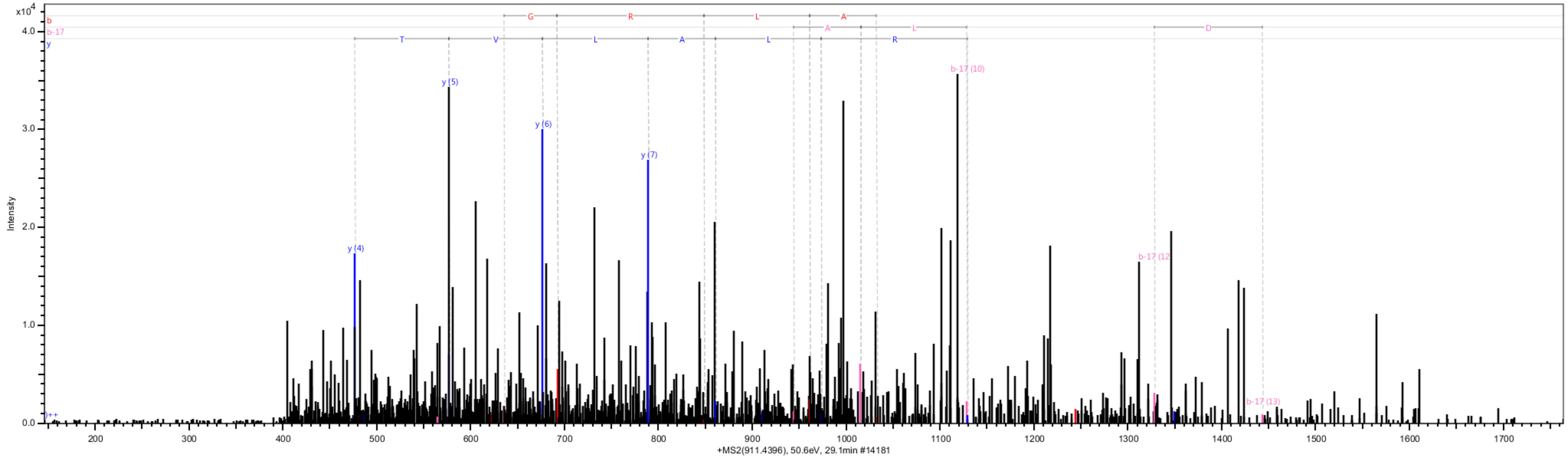

Peptide Match

**Sequence:**	R.LALVTDADR.L	**Score:**	28.2
**Modifications:**			

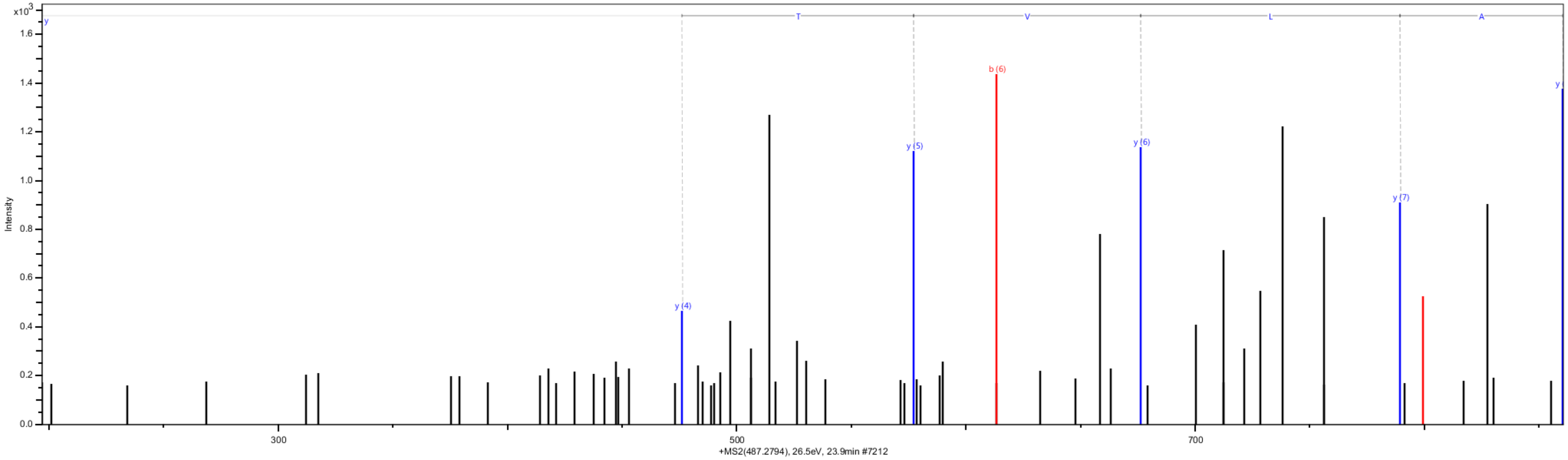
x10^3
Intensity
T
V
L
A
y
y (4)
y (5)
b (6)
y (6)
y (7)
1.6
1.4
1.2
1.0
0.8
0.6
0.4
0.2
0.0
300
500
700
+MS2(487.2794), 26.5eV, 23.9min #7212

ABCA3_HUMAN

**Accession:**	ABCA3_HUMAN	**Score:**	42.2
**Database:**	SwissProt	**Seq. Coverage [%]:**	0.5
**MW [kDa] / pI:**	191.2 / 7.5	**No. of Peptides:**	1

Peptide Match

**Sequence:**	R.YNWMTLSQK.L	**Score:**	20.5
**Modifications:**			

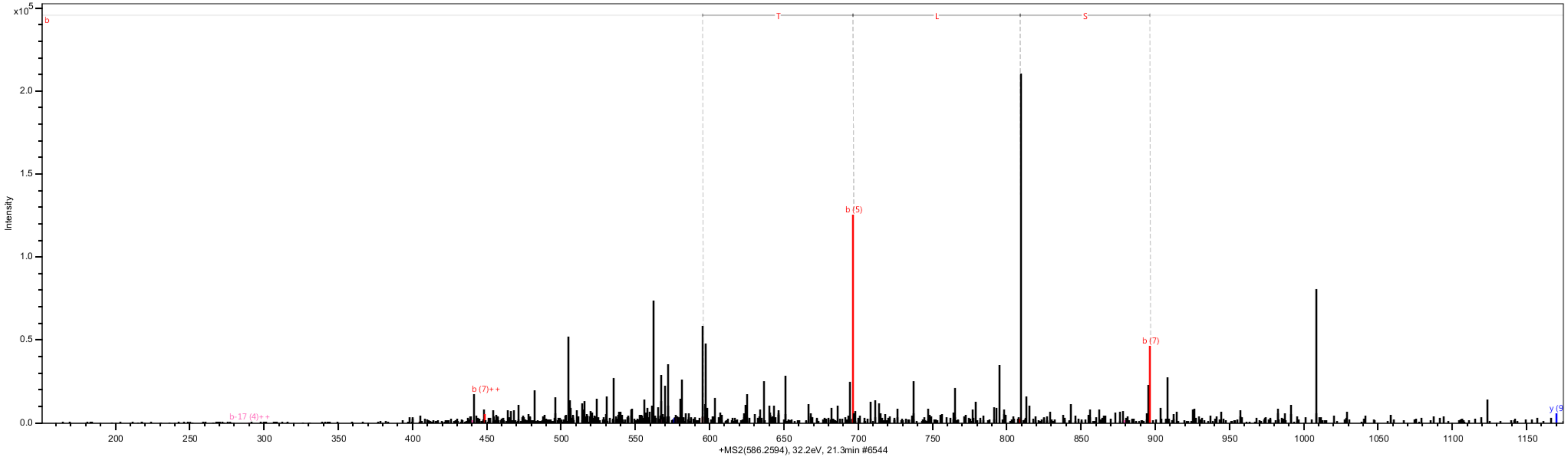

WDR55_HUMAN

Accession: WDR55_HUMAN
Database: SwissProt
MW [kDa] / pI: 42.0 / 4.8
Score: 42.1
Seq. Coverage [%]: 4.4
No. of Peptides: 2

Peptide Match

Sequence: K.FWDMAQLR.A
Score: 18.6
Modifications: Oxidation: 4

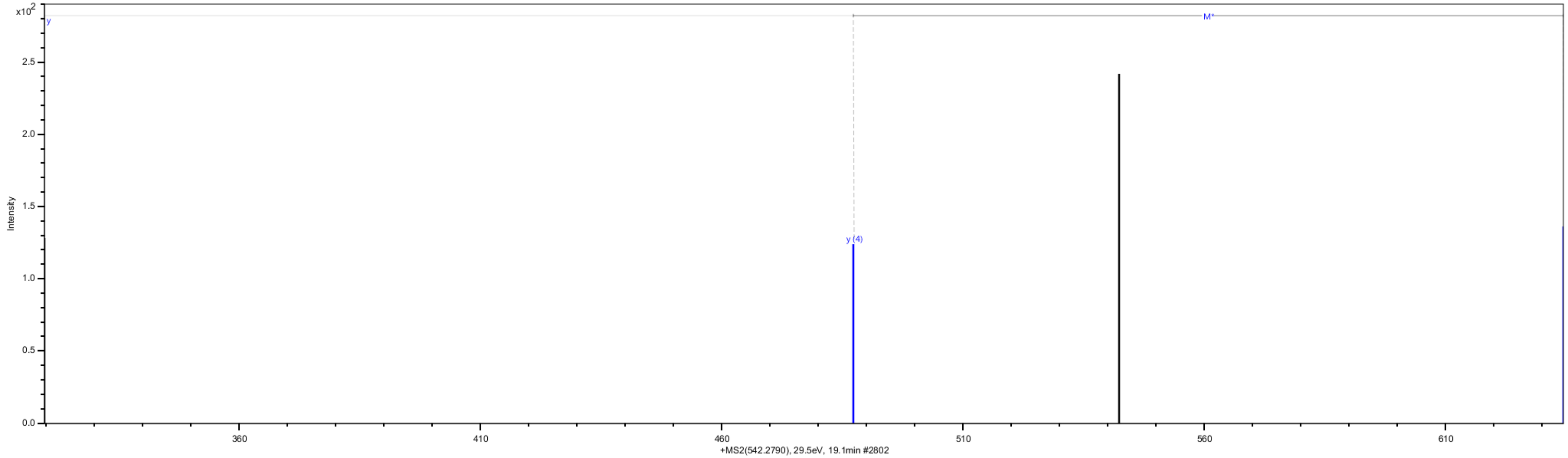

Peptide Match

Sequence: R.AVVVDDYRR.R
Score: 23.5
Modifications:

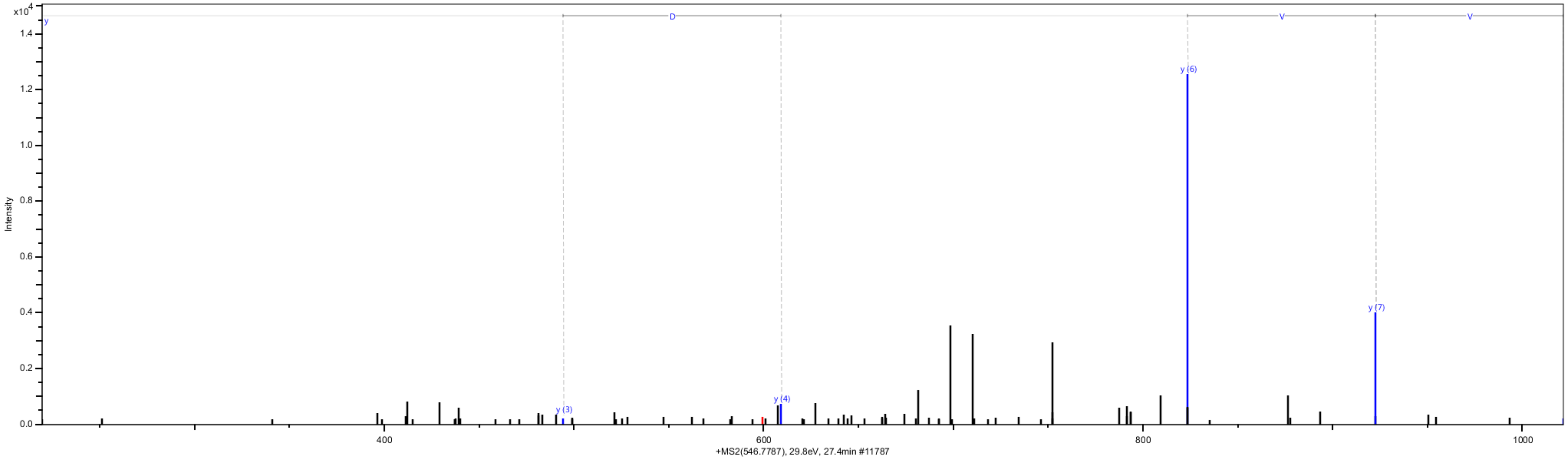
x10^4
1.4
1.2
1.0
0.8
0.6
0.4
0.2
0.0
Intensity
y
D
V
V
y (3)
y (4)
y (6)
y (7)
400
600
800
1000
+MS2(546.7787), 29.8eV, 27.4min #11787

DOCK9_HUMAN

Accession: DOCK9_HUMAN
Database: SwissProt
MW [kDa] / pI: 236.3 / 7.2
Score: 41.8
Seq. Coverage [%]: 0.6
No. of Peptides: 1

Peptide Match

Sequence: K.GNMNSAISVTMR.S
Score: 17.5
Modifications:

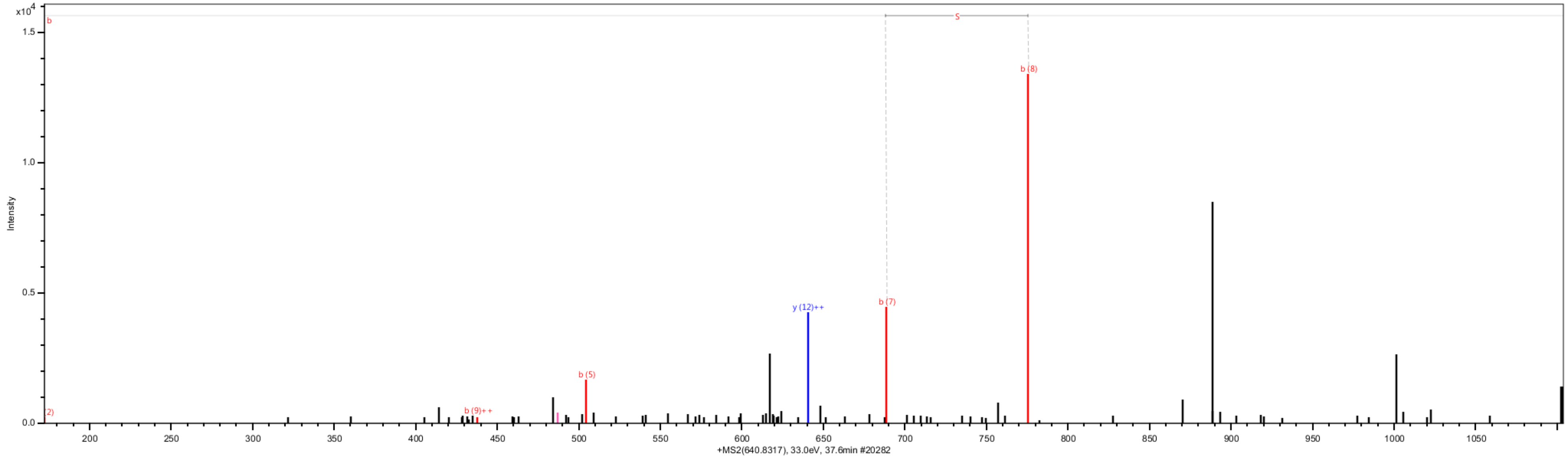

KLC4_HUMAN

Accession: KLC4_HUMAN
Database: SwissProt
MW [kDa] / pI: 68.6 / 5.8
Score: 41.4
Seq. Coverage [%]: 1.9
No. of Peptides: 1

Peptide Match

Sequence: R.RTSQEGPGDSVK.F
Score: 16.5
Modifications:

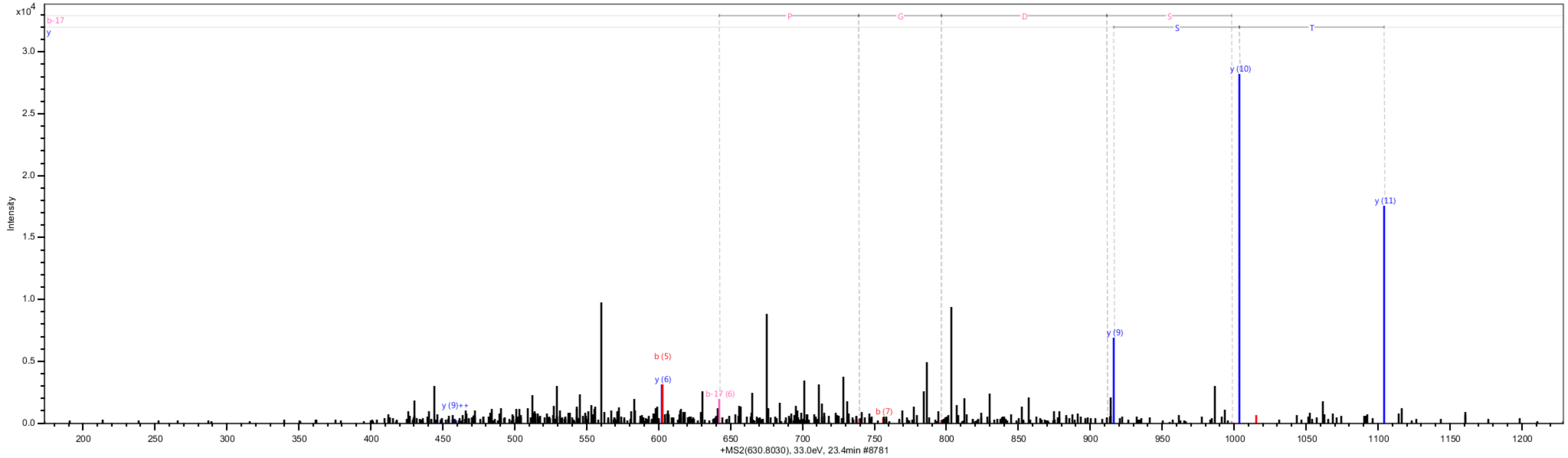

BUP1_HUMAN

Accession: BUP1_HUMAN **Score:** 41.2
Database: SwissProt **Seq. Coverage [%]:** 5.5
MW [kDa] / pI: 43.1 / 6.1 **No. of Peptides:** 2

Peptide Match

Sequence: R.EAFEAASR.E **Score:** 16.8
Modifications:

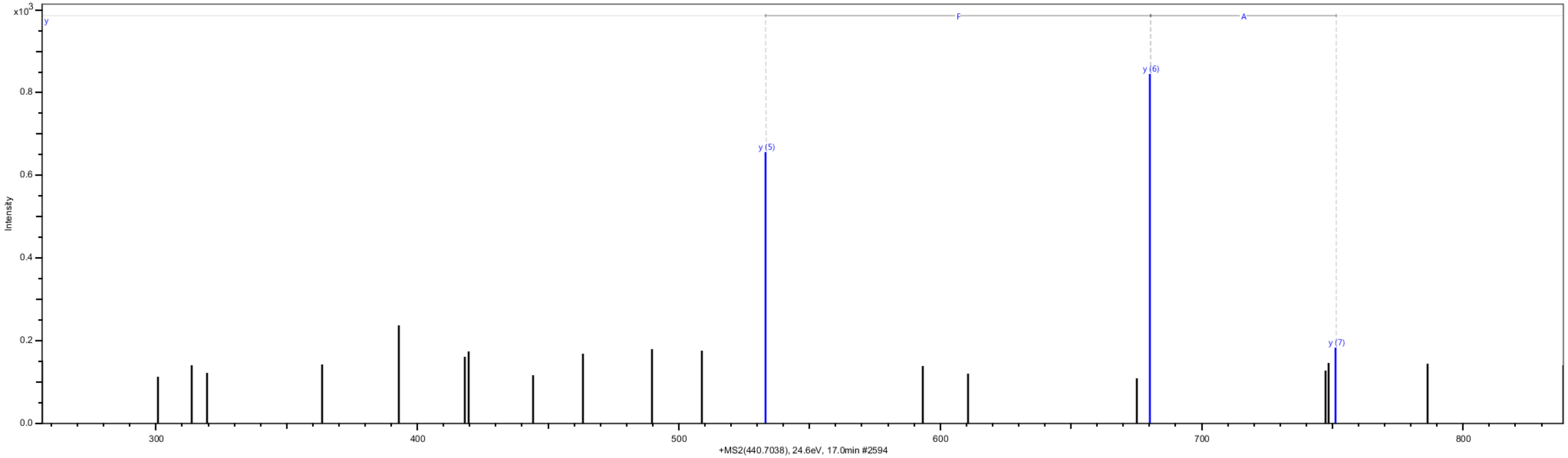

Peptide Match

Sequence: K.NHDMVVVSPILER.D **Score:** 24.4
Modifications: Oxidation: 4

Spectrum Report
Combine Search_2020-10-19 07:53:31

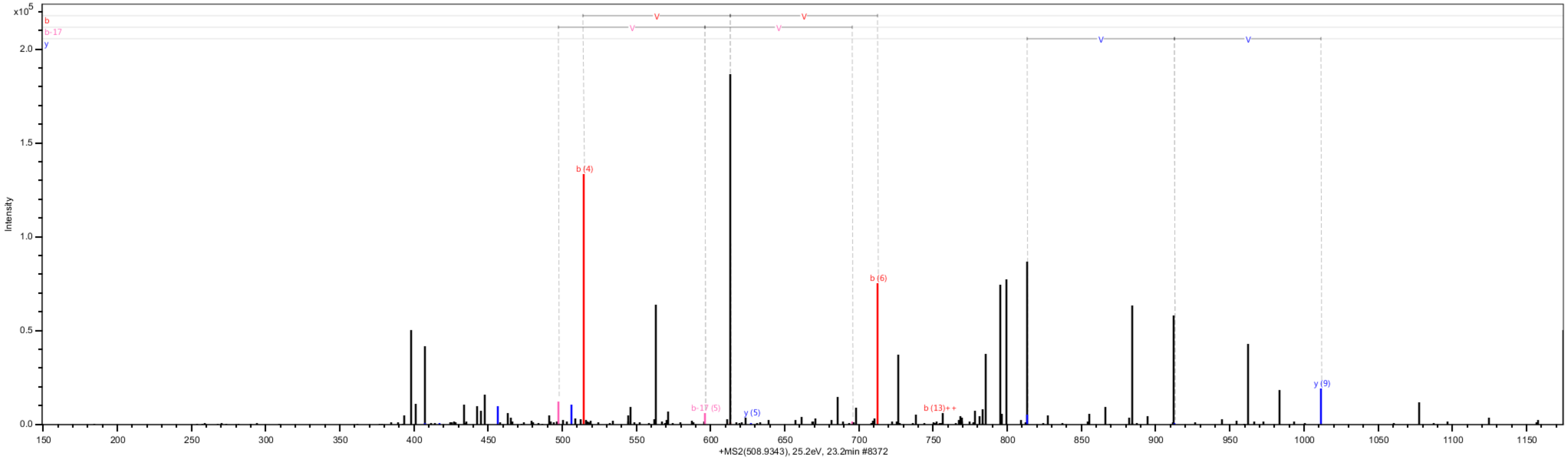

MABP1_HUMAN

**Accession:**	MABP1_HUMAN	**Score:**	41.0
**Database:**	SwissProt	**Seq. Coverage [%]:**	0.7
**MW [kDa] / pI:**	163.7 / 6.3	**No. of Peptides:**	1

Peptide Match

**Sequence:**	K.FWYLDDSKTSK.V	**Score:**	25.8
**Modifications:**			

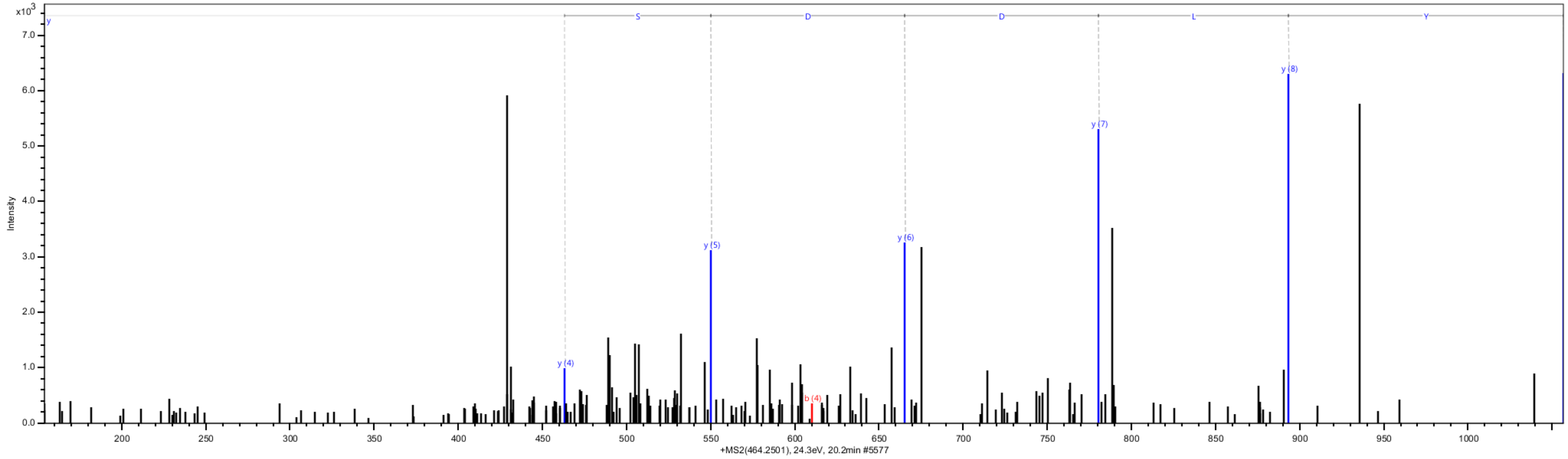

BRCA2_HUMAN

**Accession:**	BRCA2_HUMAN	**Score:**	40.9
**Database:**	SwissProt	**Seq. Coverage [%]:**	0.4
**MW [kDa] / pI:**	384.0 / 6.3	**No. of Peptides:**	1

Peptide Match

**Sequence:**	R.KSVSTPVSAQMTSK.S	**Score:**	22.4
**Modifications:**	Oxidation: 11		

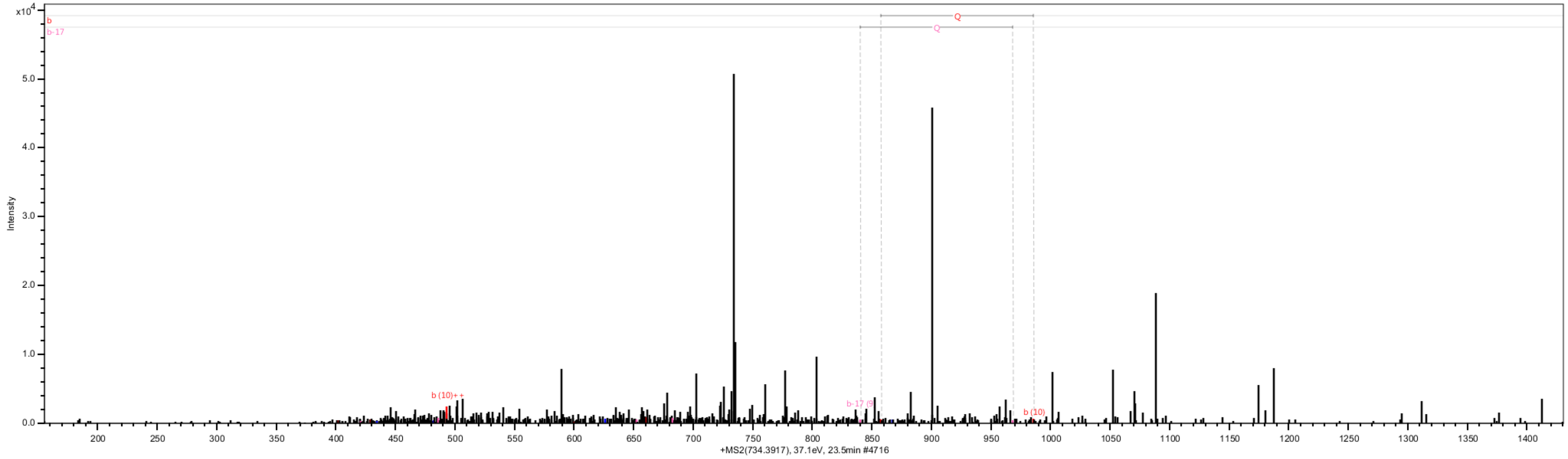

CO6_HUMAN

Accession: CO6_HUMAN
Database: SwissProt
MW [kDa] / pI: 104.7 / 6.4
Score: 40.8
Seq. Coverage [%]: 0.9
No. of Peptides: 1

Peptide Match

Sequence: R.QLEWGLER.T
Score: 40.8
Modifications:

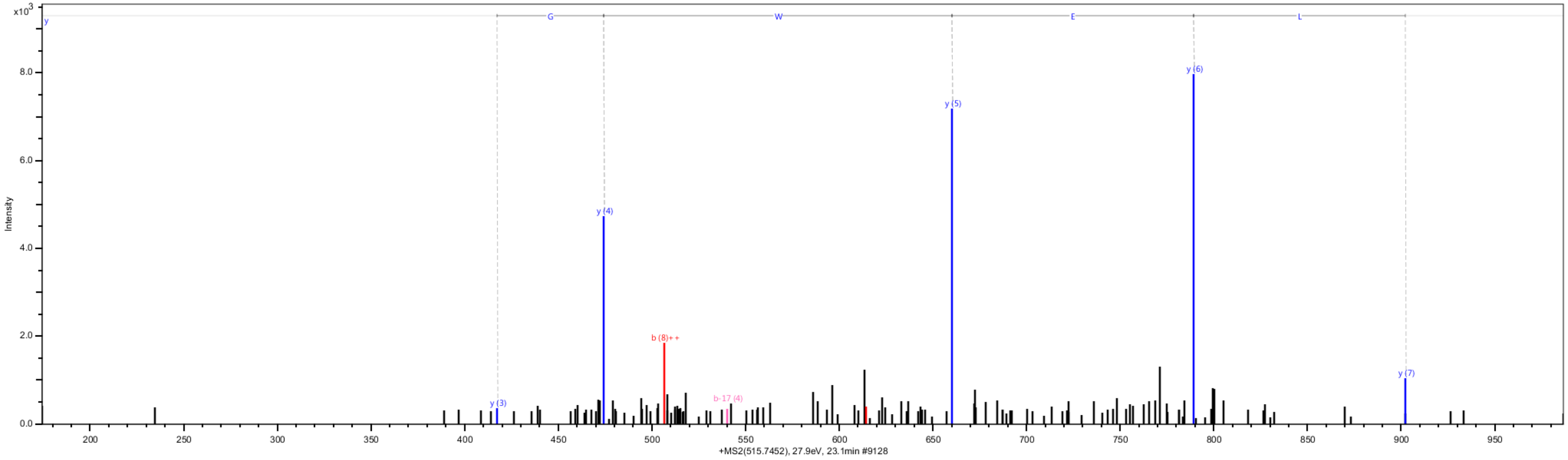

ARHGC_HUMAN

Accession: ARHGC_HUMAN
Database: SwissProt
MW [kDa] / pI: 173.1 / 5.5
Score: 40.6
Seq. Coverage [%]: 0.7
No. of Peptides: 1

Peptide Match

Sequence: R.AGVQTGDRIIK.V
Score: 21
Modifications:

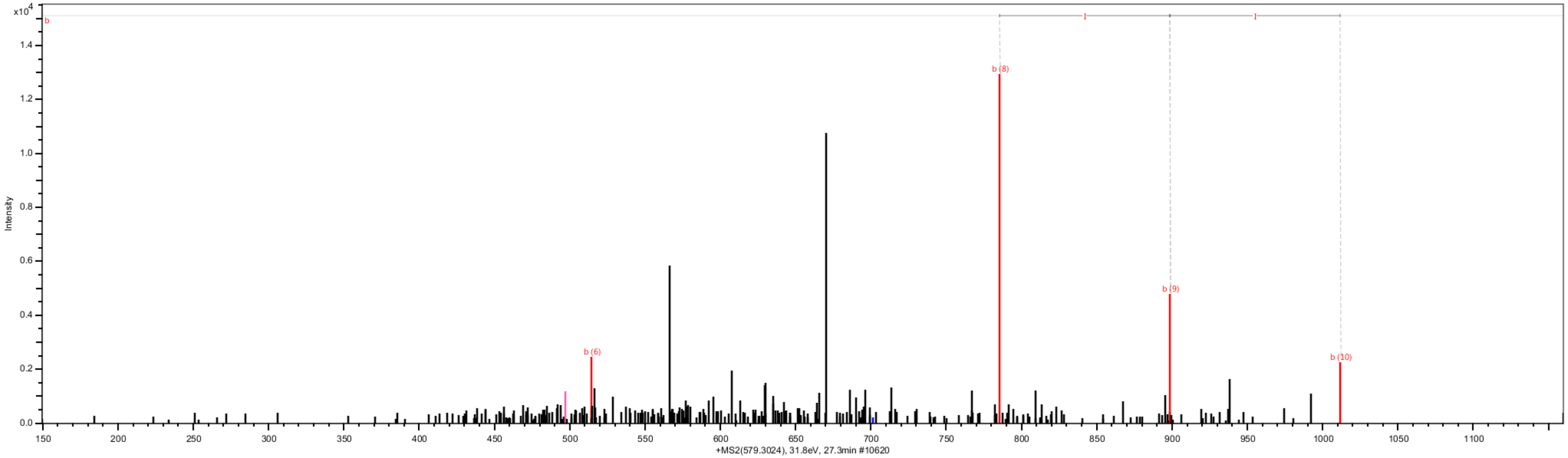

UBP16_HUMAN

**Accession:**	UBP16_HUMAN	**Score:**	40.3
**Database:**	SwissProt	**Seq. Coverage [%]:**	1.0
**MW [kDa] / pI:**	93.5 / 6.5	**No. of Peptides:**	1

Peptide Match

**Sequence:**	K.HVYTNAKK.Q	**Score:**	23.7
**Modifications:**			

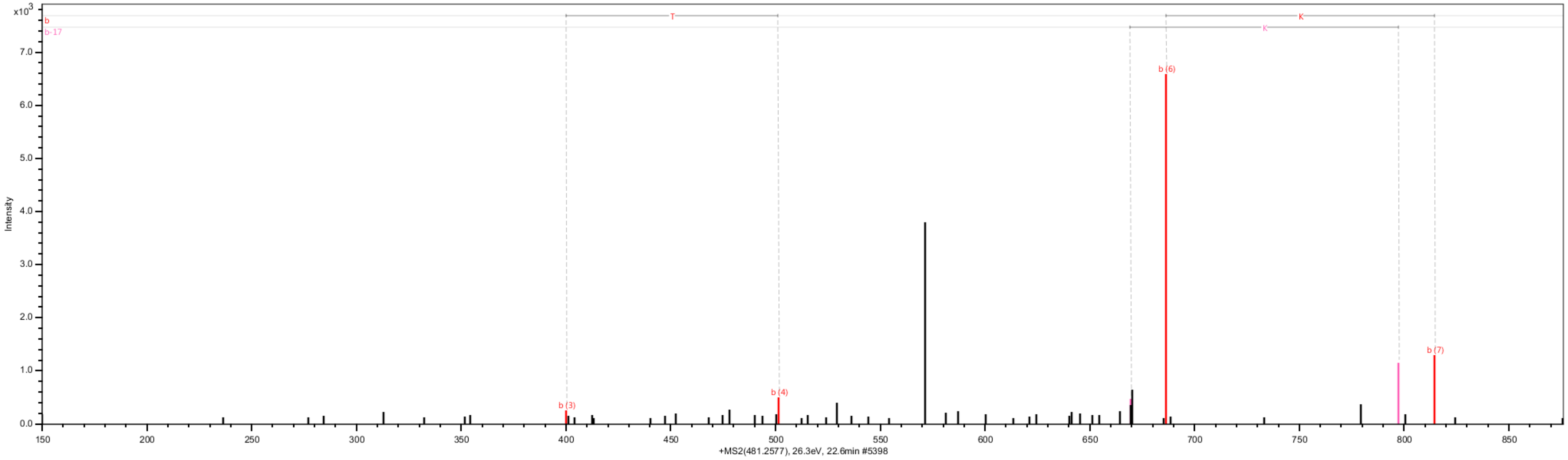

HBB_HUMAN

Accession: HBB_HUMAN
Database: SwissProt
MW [kDa] / pI: 16.0 / 6.7
Score: 567.2
Seq. Coverage [%]: 61.9
No. of Peptides: 9

Peptide Match

Sequence: K.SAVTALWGK.V
Score: 46
Modifications:

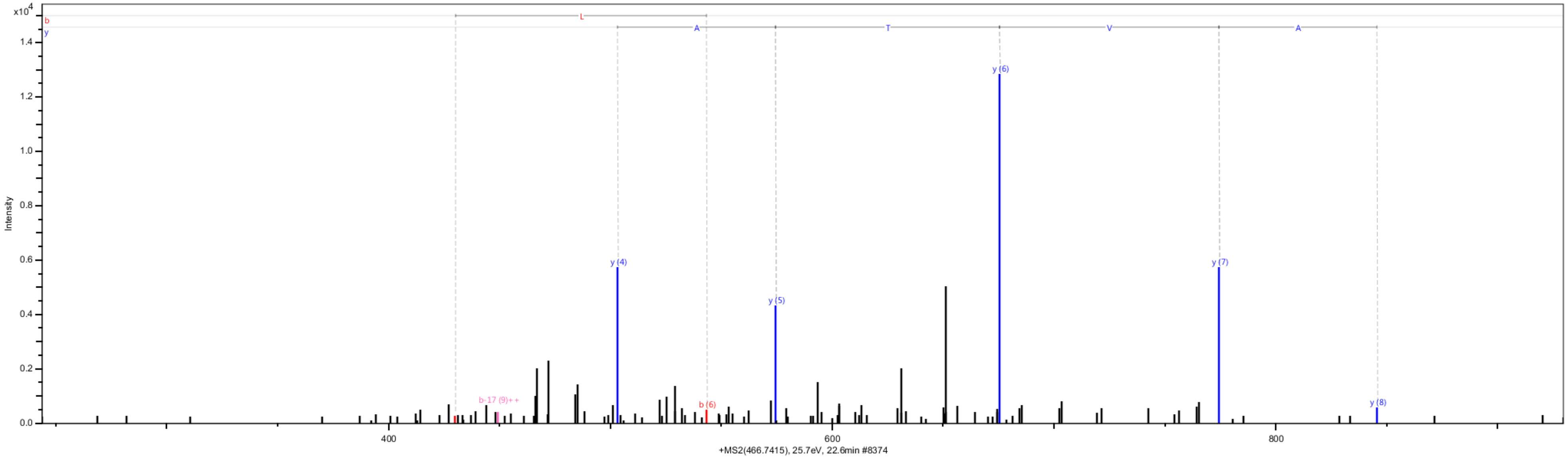

Peptide Match

Sequence: K.VNVDEVGGEALGR.L
Score: 58.2
Modifications:

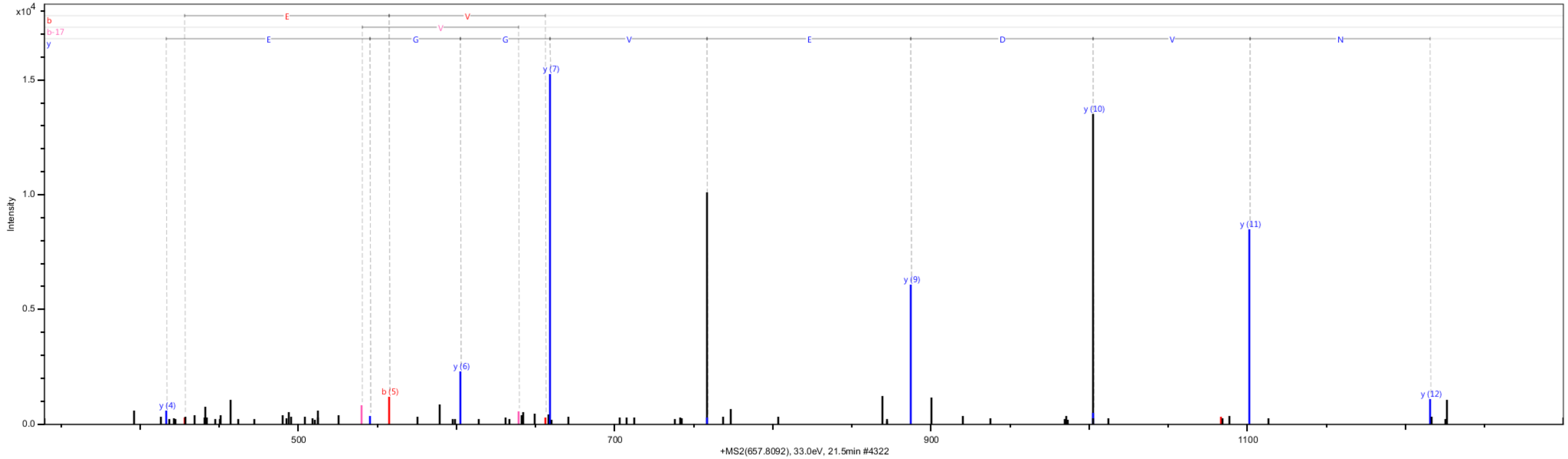

Peptide Match

Sequence: R.LLVVYPWTQR.F **Score:** 57.4

Modifications:

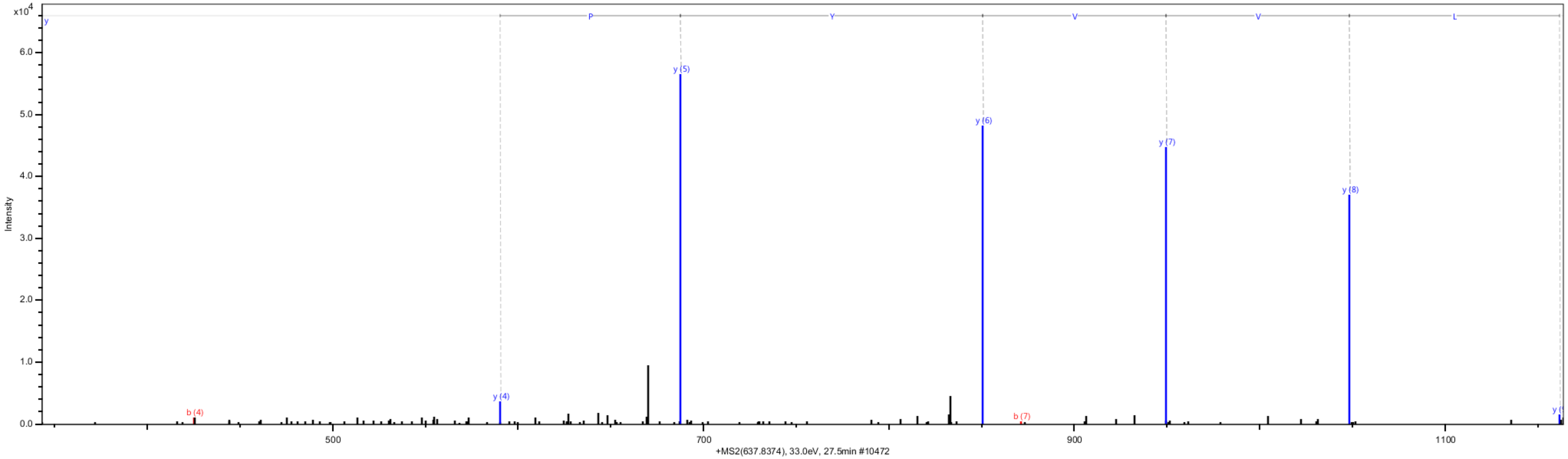

Peptide Match

Sequence: K.KVLGAFSDGLAHLDNLK.G **Score:** 77.4

Modifications:

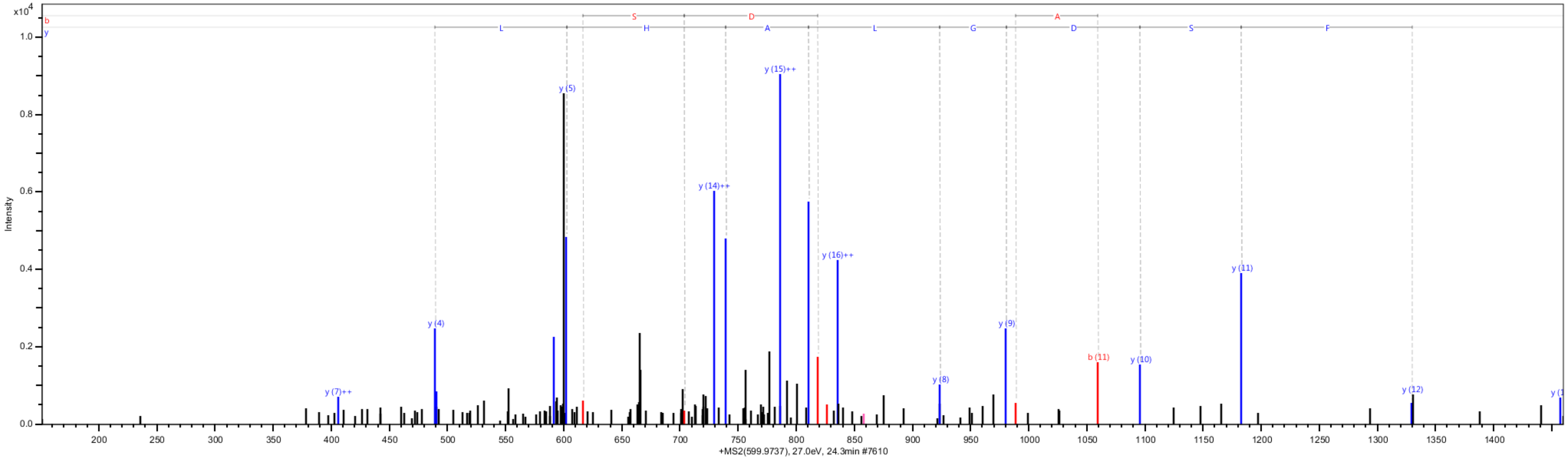

Peptide Match

Sequence: K.VLGAFSDGLAHLDNLK.G **Score:** 59.4

Modifications:

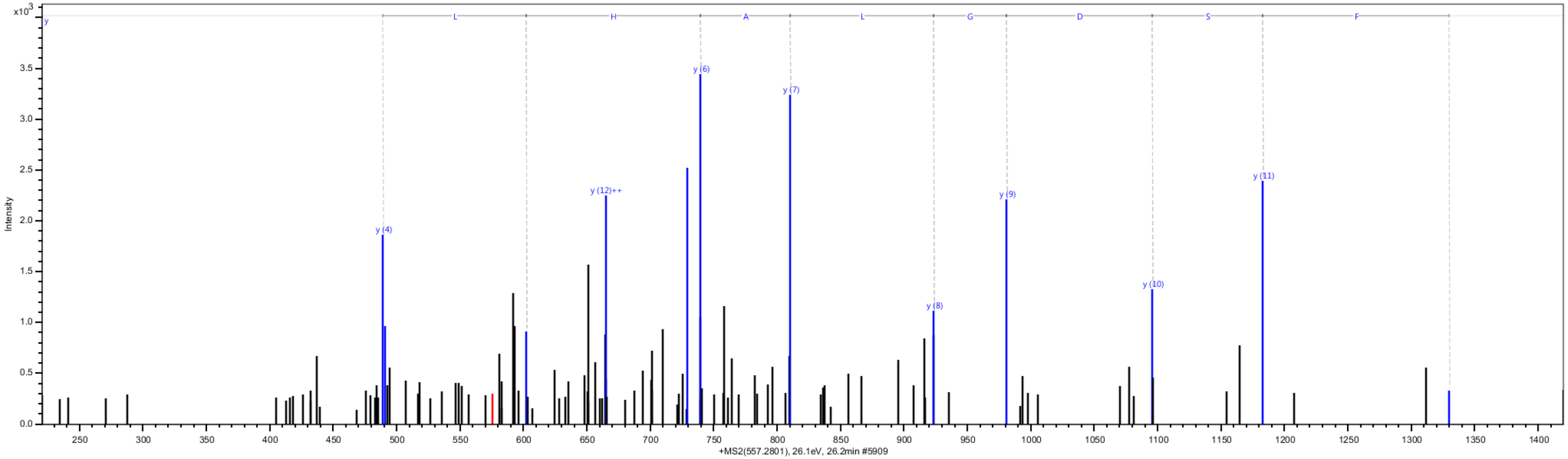

Peptide Match

**Sequence:**	R.LLGNVLVCVLAHHFGK.E	**Score:**	85.3
**Modifications:**	Carbamidomethyl: 8		

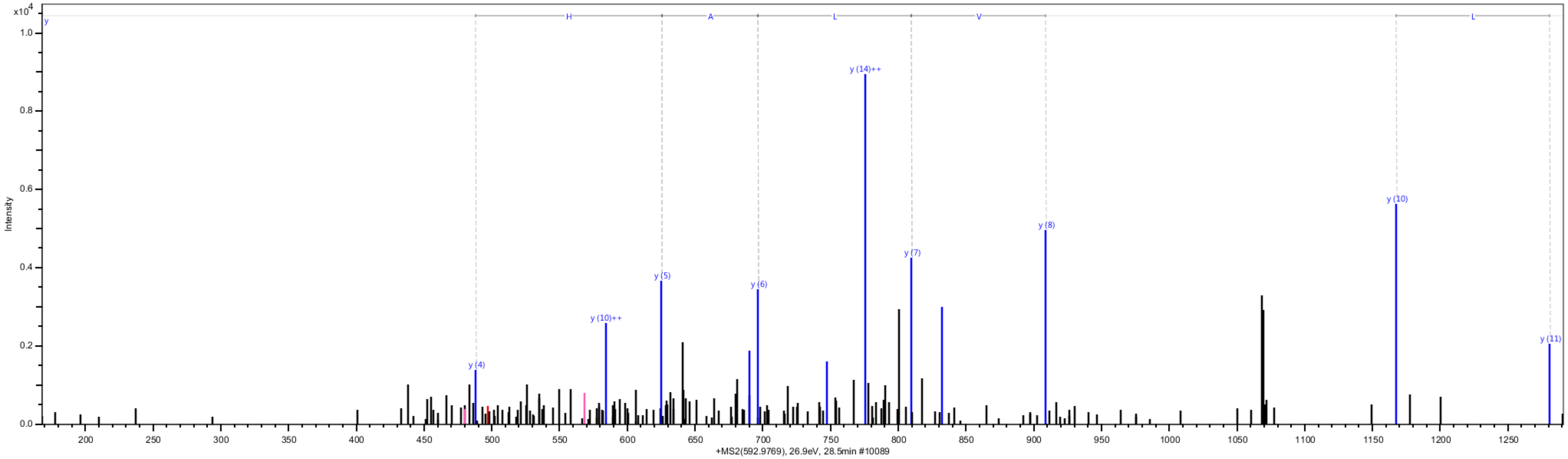

Peptide Match

Sequence: K.EFTPPVQAAYQK.V **Score:** 32.9

Modifications:

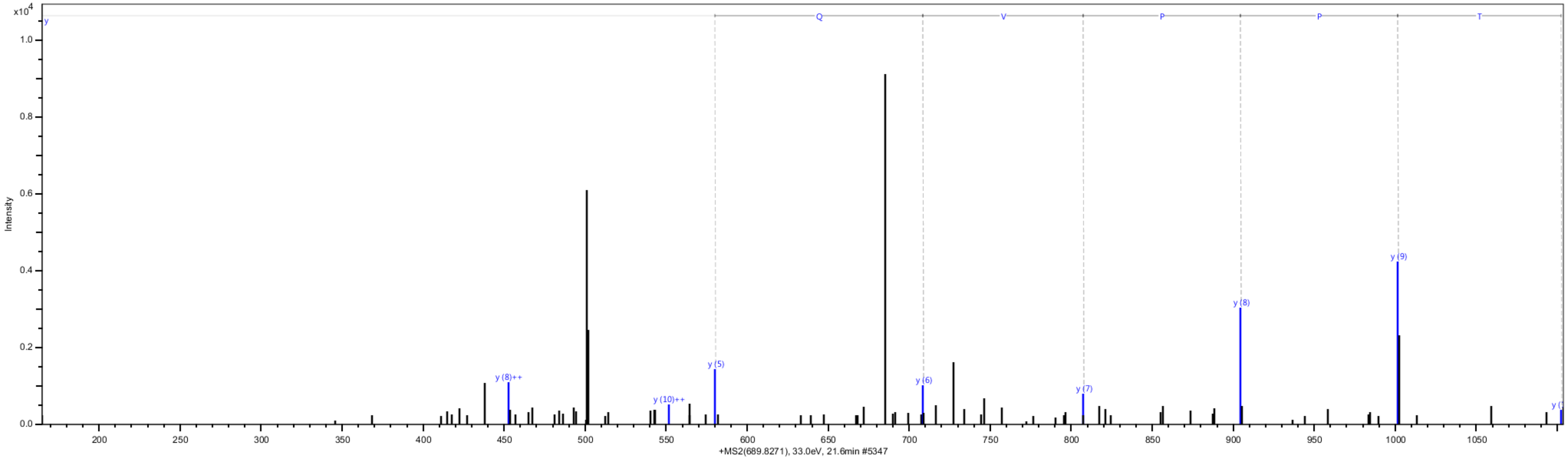

Peptide Match

Sequence: K.VVAGVANALAHK.Y **Score:** 68.2

Modifications:

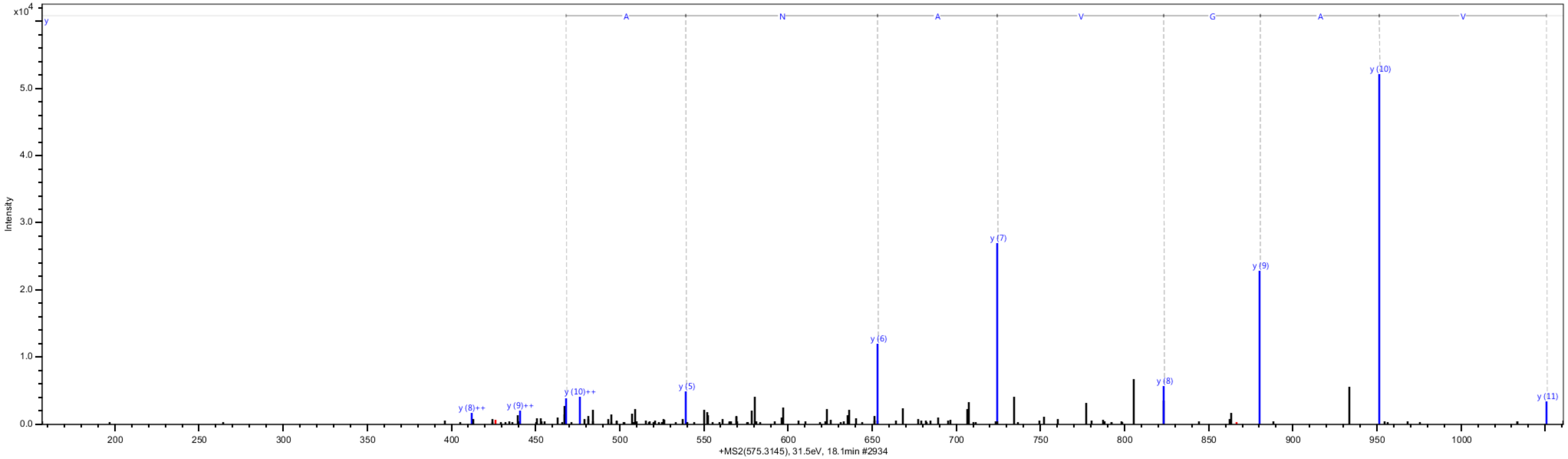

Peptide Match

Sequence: K.VVAGVANALAHKYH.- **Score:** 82.4

Modifications:

Spectrum Report
Combine Search_2020-10-19 07:53:31

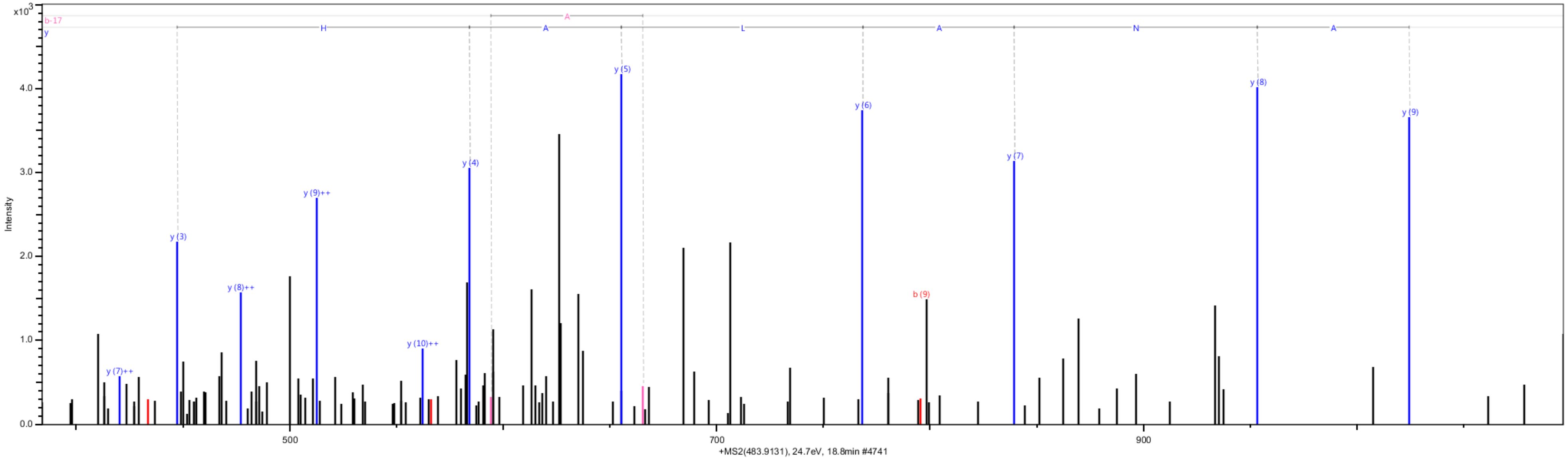

APOA4_HUMAN

**Accession:**	APOA4_HUMAN	**Score:**	461.2
**Database:**	SwissProt	**Seq. Coverage [%]:**	32.6
**MW [kDa] / pI:**	45.4 / 5.3	**No. of Peptides:**	12

Peptide Match

Sequence: K.SELTQQLNALFQDK.L **Score:** 43.6

Modifications:

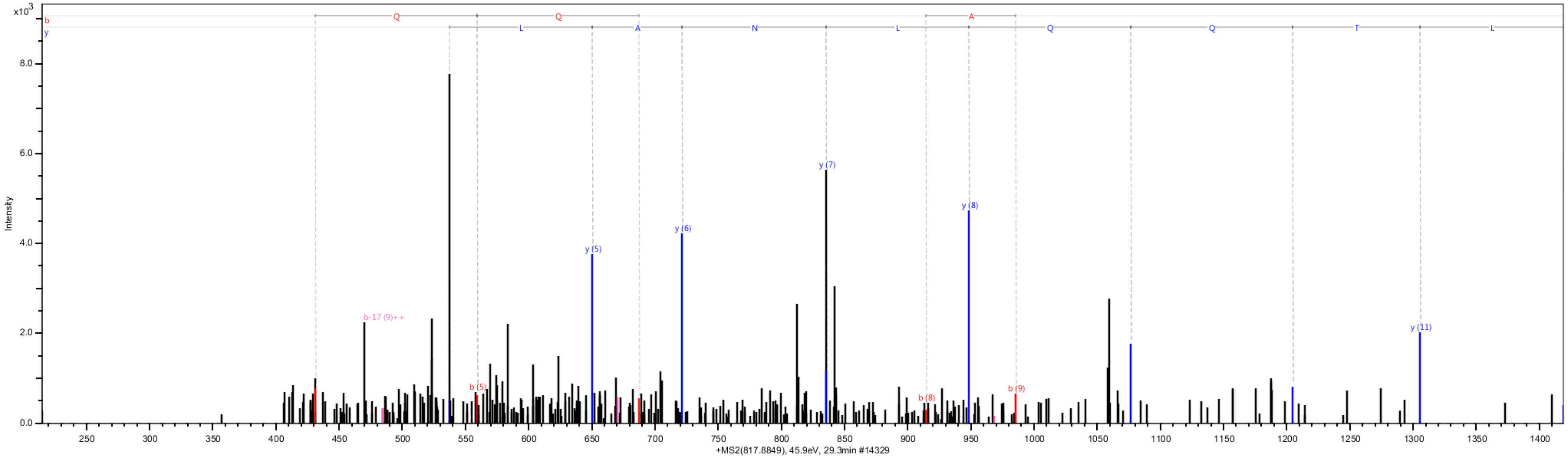

Peptide Match

Sequence: K.KLVPFATELHER.L **Score:** 75

Modifications:

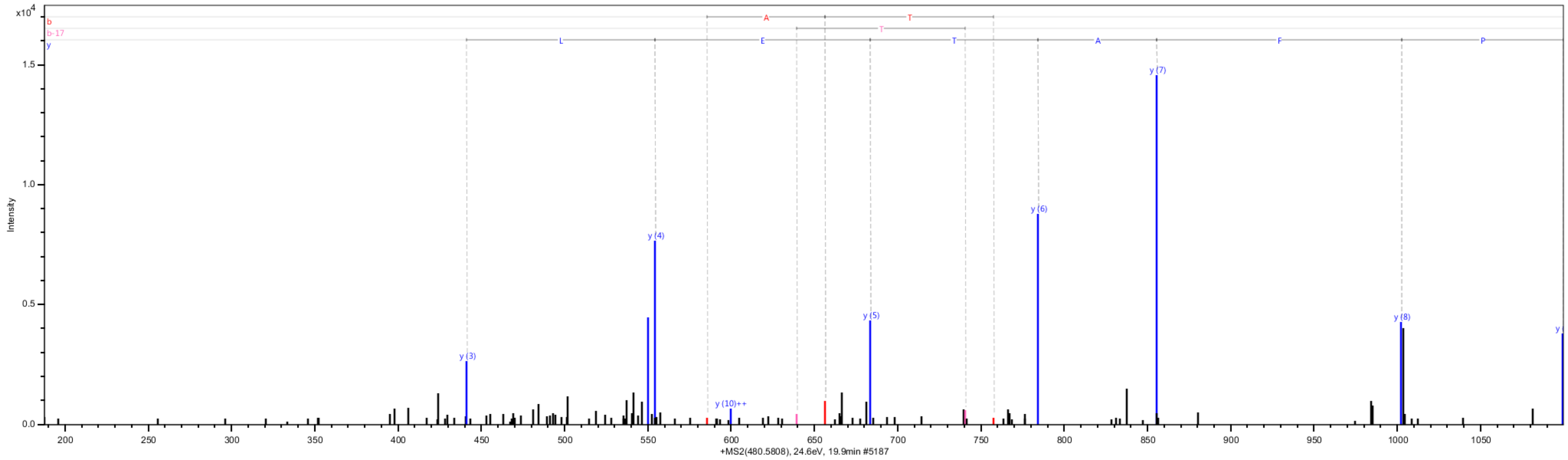

Peptide Match

**Sequence:**	K.LVPFATELHER.L	**Score:**	51.8
**Modifications:**			

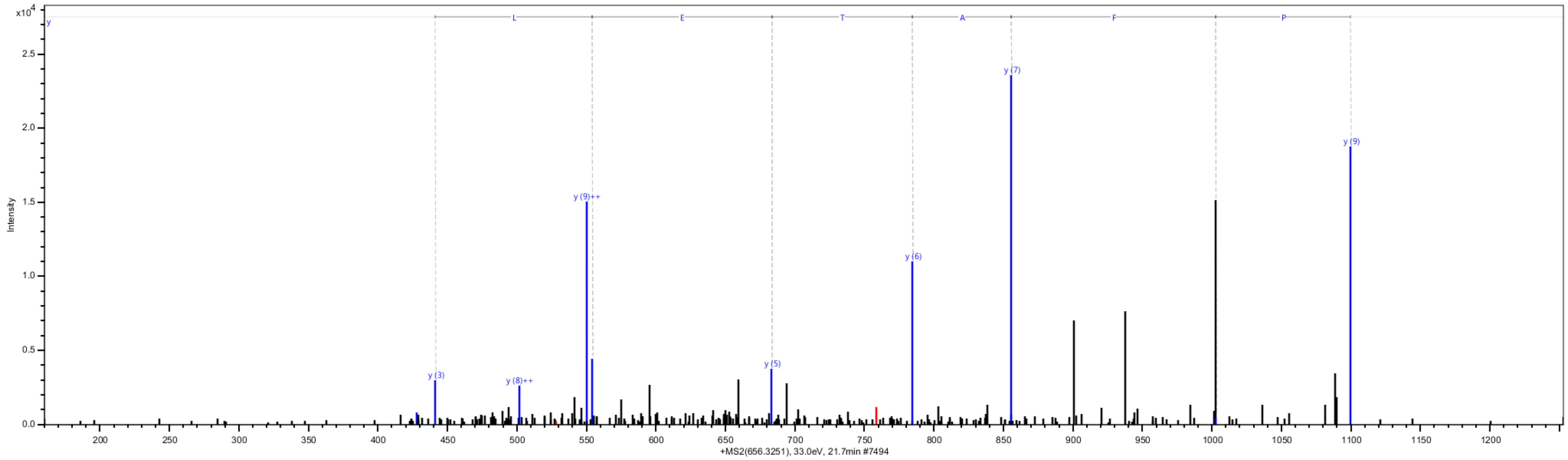

Peptide Match

Sequence: K.IGDNLRELQQR.L **Score:** 23.7

Modifications:

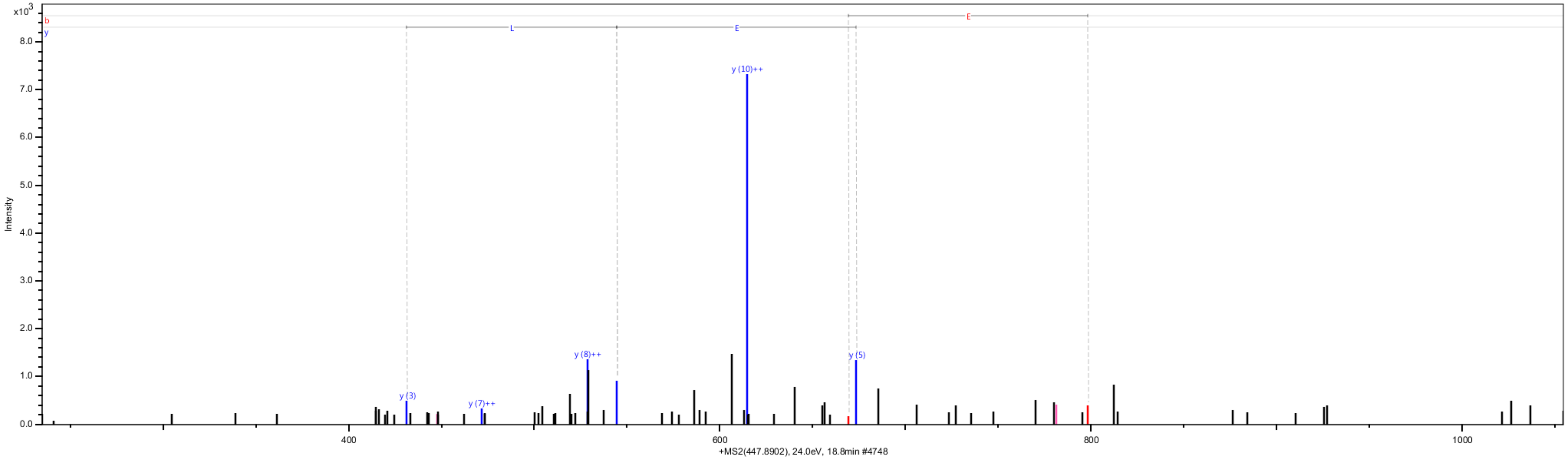

Peptide Match

Sequence: R.LEPYADQLR.T **Score:** 39.5

Modifications:

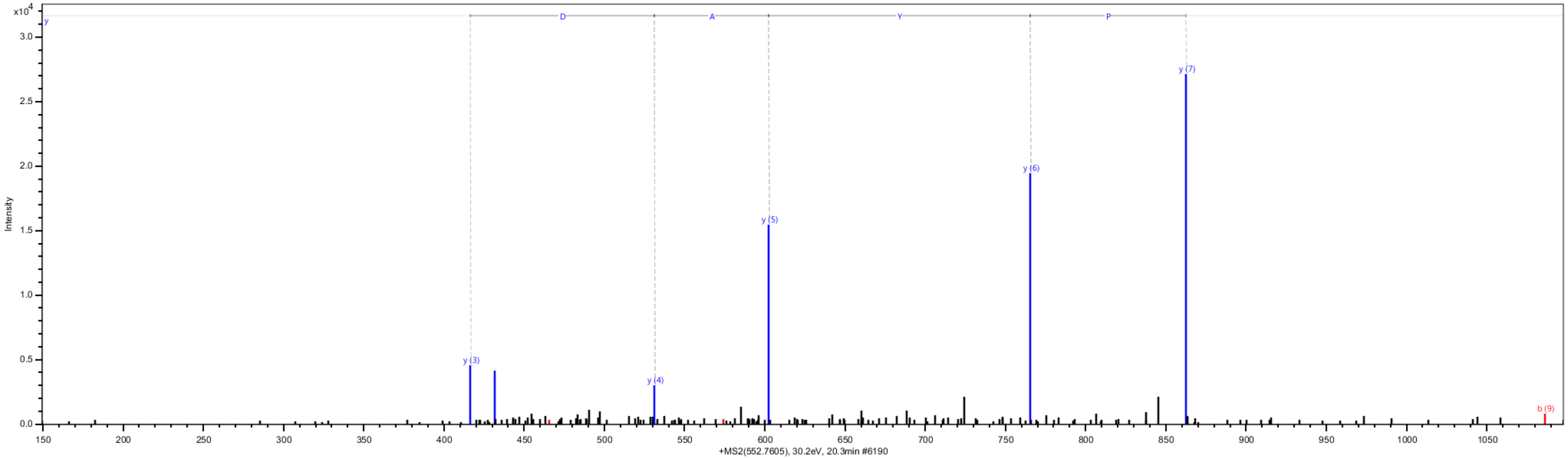

Peptide Match

Sequence: K.IDQNVEELKGR.L **Score:** 32

Modifications:

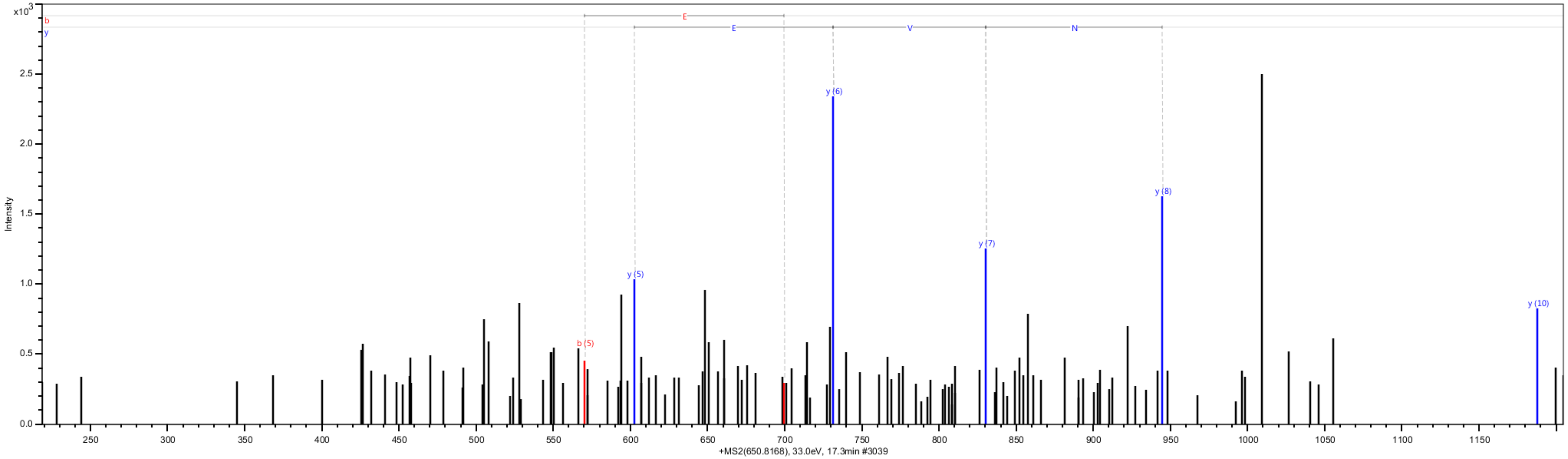

Peptide Match

Sequence: R.LTPYADEFK.V **Score:** 21.7

Modifications:

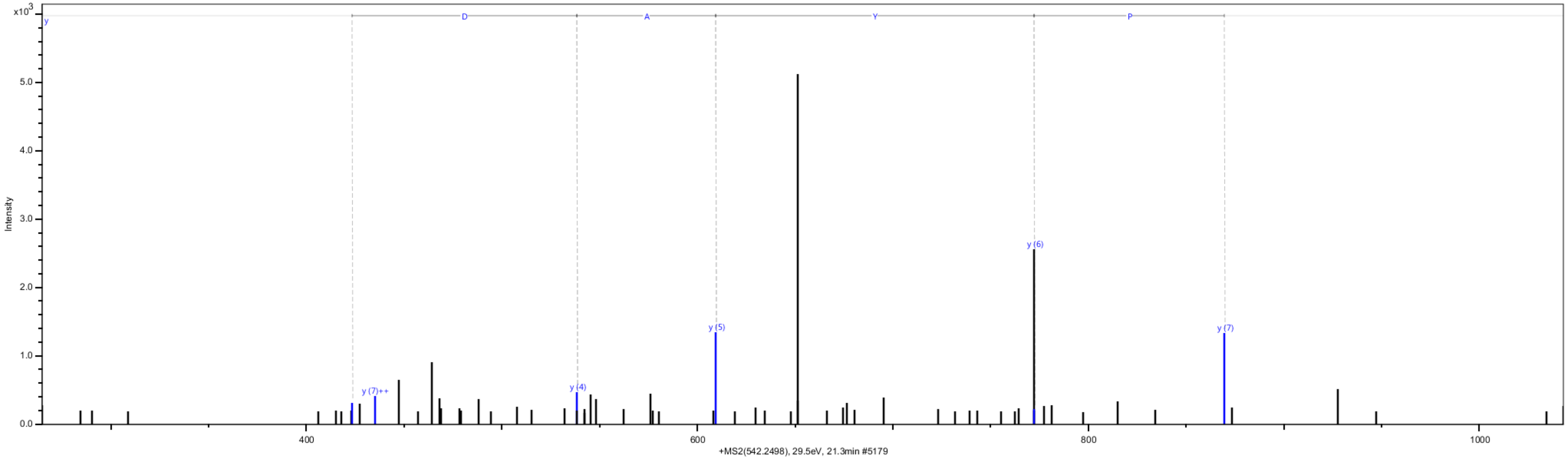

Peptide Match

Sequence: R.ISASAEELR.Q **Score:** 16.2

Modifications:

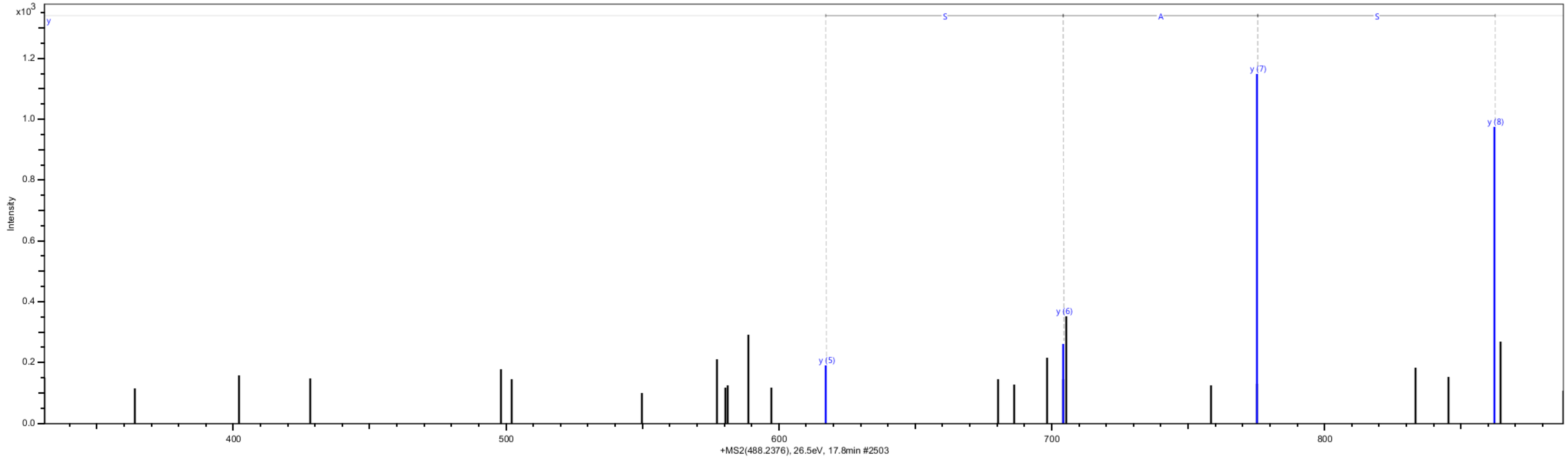

Peptide Match

Sequence: R.LAPLAEDVR.G **Score:** 42.9

Modifications:

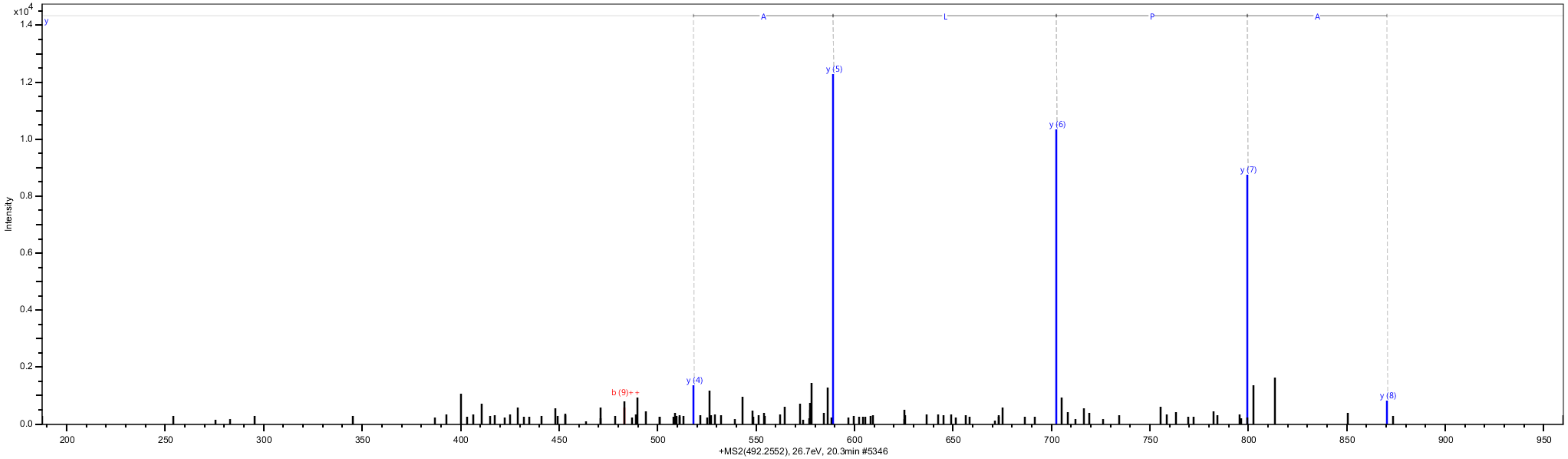

Peptide Match

Sequence: K.SLAELGGHLDQQVEEFRR.R **Score:** 49.8

Modifications:

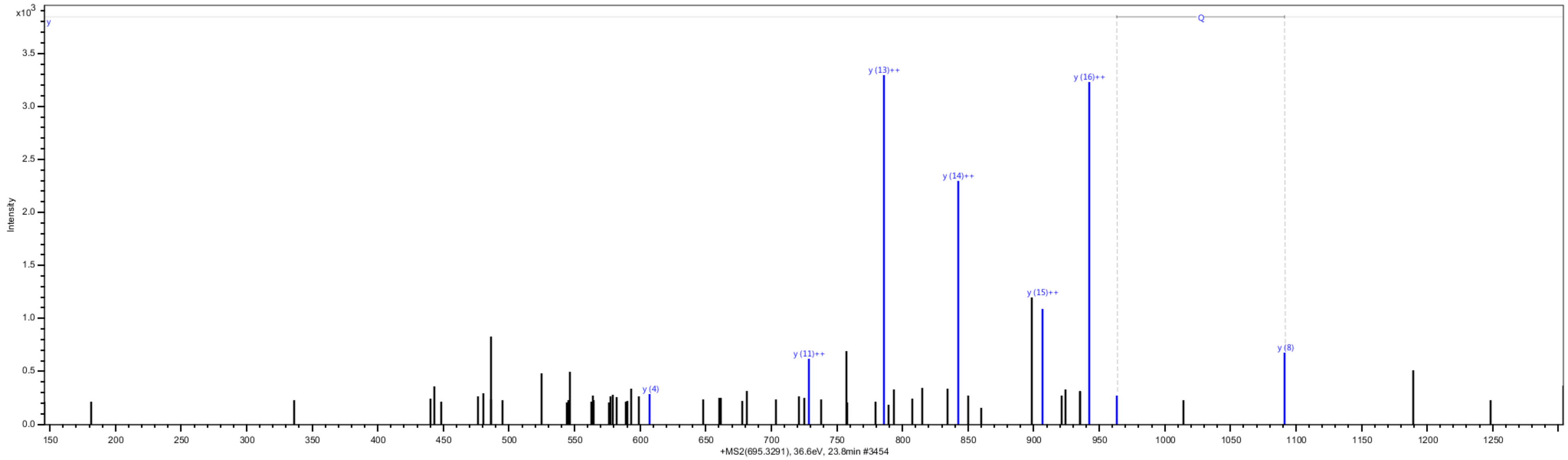

Peptide Match

Sequence: K.ALVQQMEQLR.Q **Score:** 31.8

Modifications:

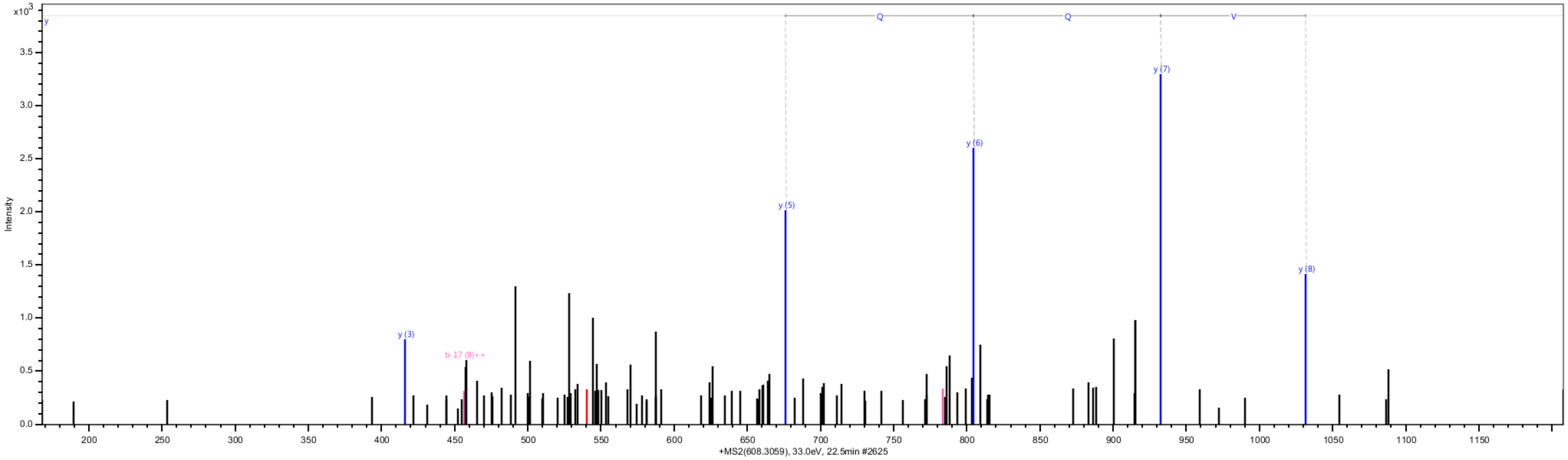

Peptide Match

**Sequence:**	K.LGPHAGDVEGHLSFLEK.D	**Score:**	33.2
**Modifications:**			

Spectrum Report
Combine Search_2020-10-19 07:53:31

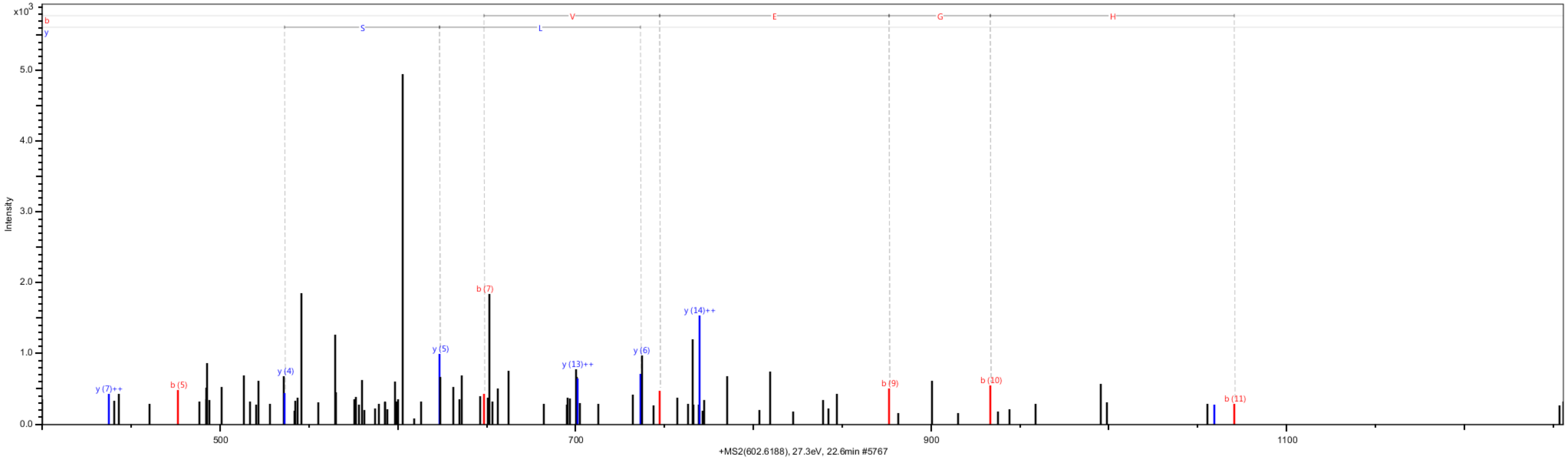

HBD_HUMAN

**Accession:**	HBD_HUMAN	**Score:**	433.2
**Database:**	SwissProt	**Seq. Coverage [%]:**	17.0
**MW [kDa] / pI:**	16.0 / 7.8	**No. of Peptides:**	2

Peptide Match

**Sequence:**	K.VNVDAVGGEALGR.L	**Score:**	45.9
**Modifications:**			

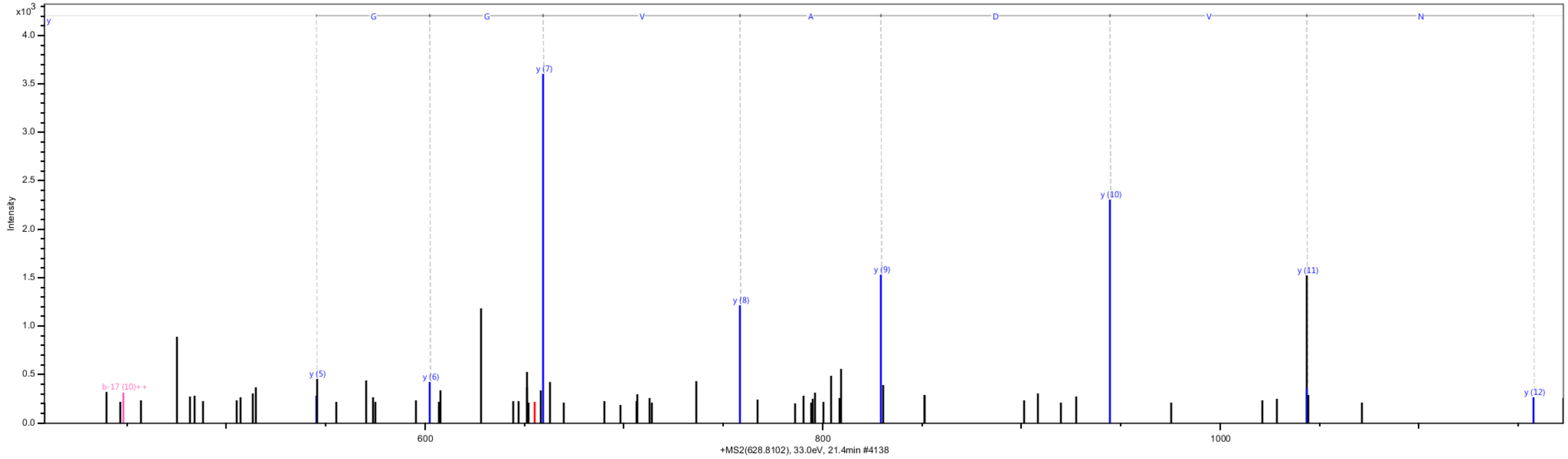

Peptide Match

**Sequence:**	R.LLGNVLVCVLAR.N	**Score:**	42.4
**Modifications:**	Carbamidomethyl: 8		

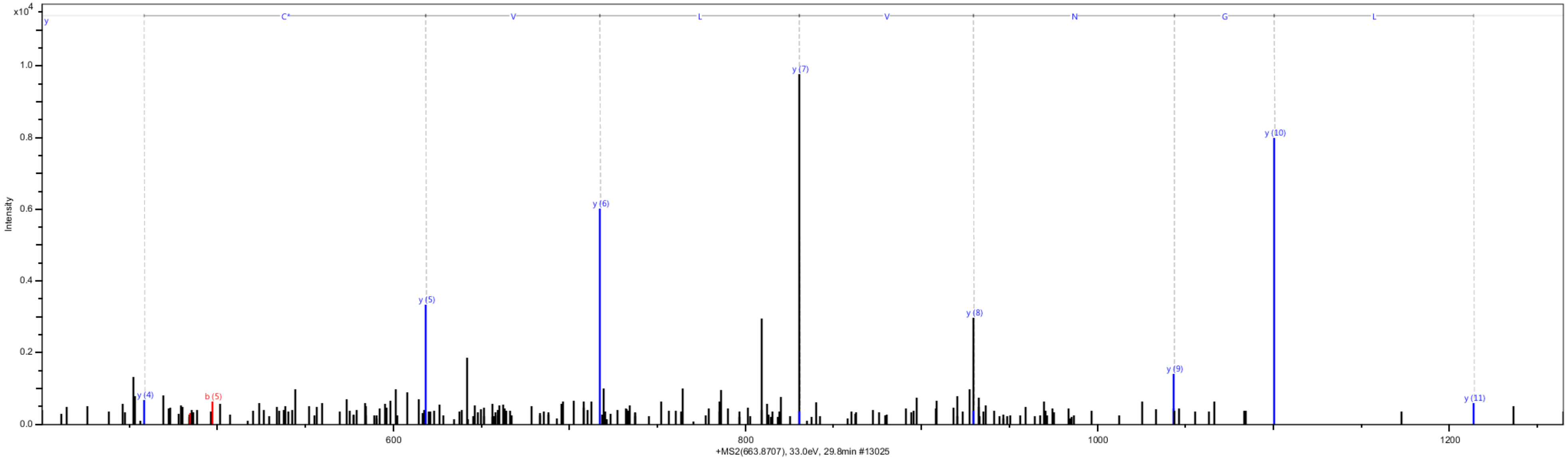
x10^4
Intensity
y
C*
V
L
V
N
G
L
1.0
0.8
0.6
0.4
0.2
0.0
y (7)
y (10)
y (6)
y (5)
y (8)
y (9)
y (4)
b (5)
y (11)
600
800
1000
1200
+MS2(663.8707), 33.0eV, 29.8min #13025

HEMO_HUMAN

Accession: HEMO_HUMAN
Database: SwissProt
MW [kDa] / pI: 51.6 / 6.5

Score: 384.5
Seq. Coverage [%]: 20.6
No. of Peptides: 11

Peptide Match

Sequence: K.WDRELISER.W
Score: 21.2
Modifications:

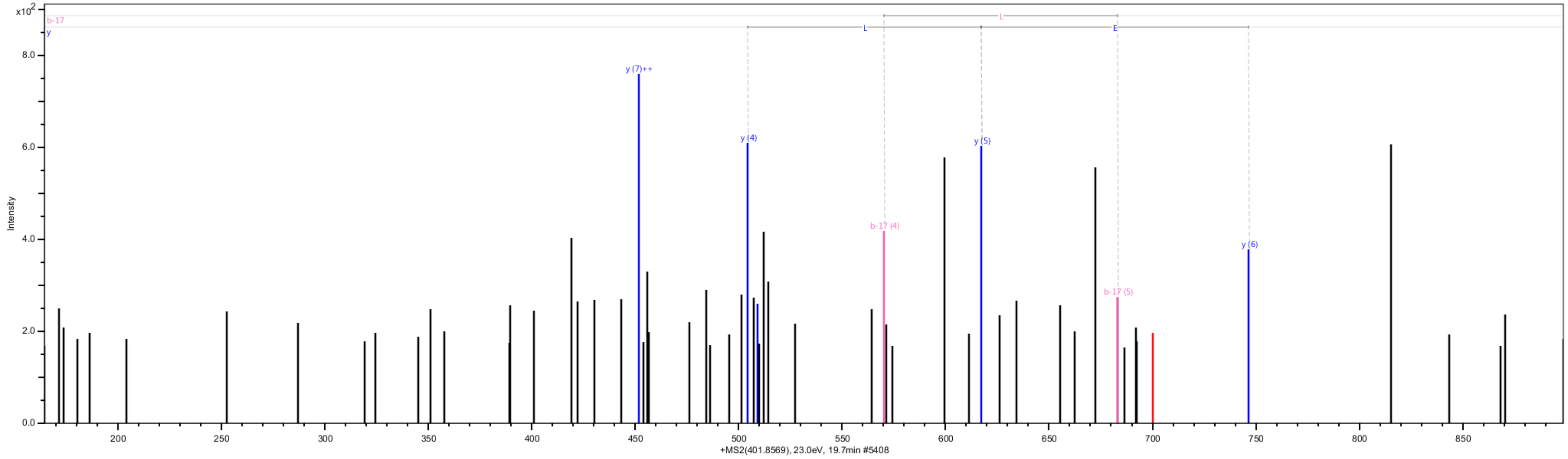

Peptide Match

Sequence: R.WKNFPSPVDAAFR.Q
Score: 64.9
Modifications:

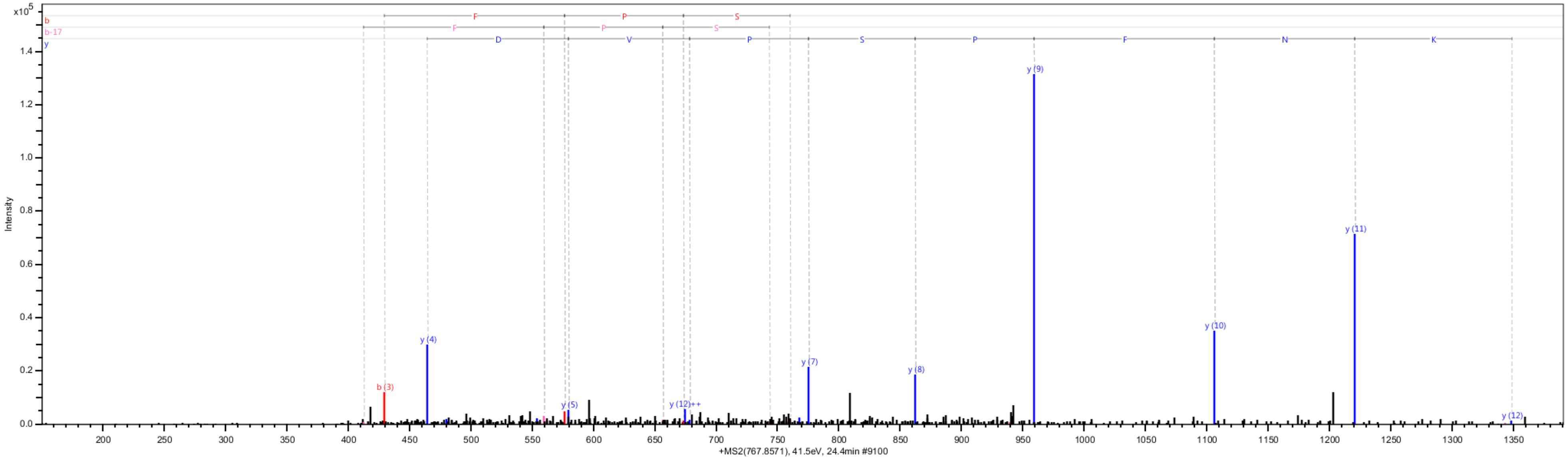

Peptide Match

Sequence: K.NFPSPVDAAFR.Q **Score:** 66.3

Modifications:

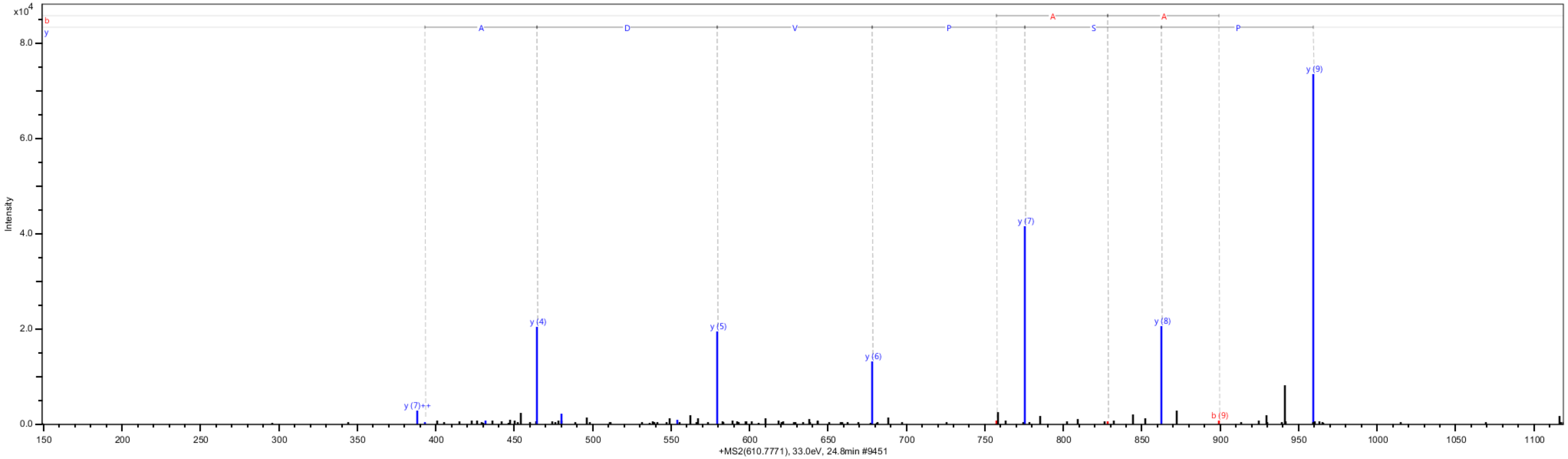

Peptide Match

Sequence: R.QGHNSVFLIK.G **Score:** 26

Modifications:

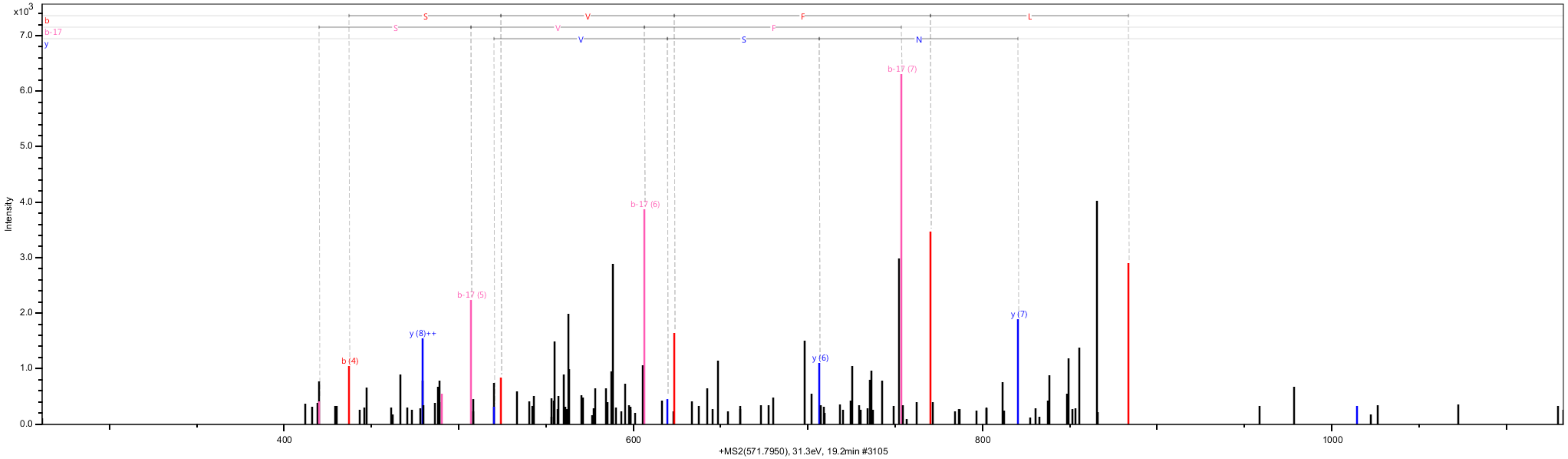

Peptide Match

Sequence: K.VWVYPPEKK.E **Score:** 20.3

Modifications:

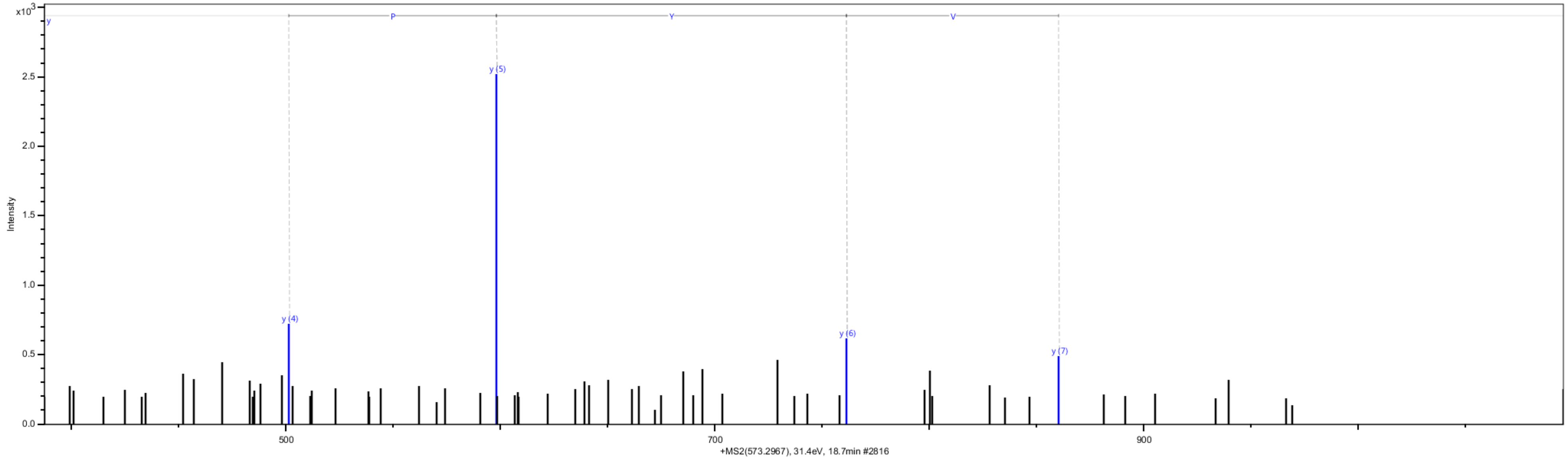

Peptide Match

Sequence: R.EWFWDLATGTMK.E **Score:** 25.7

Modifications:

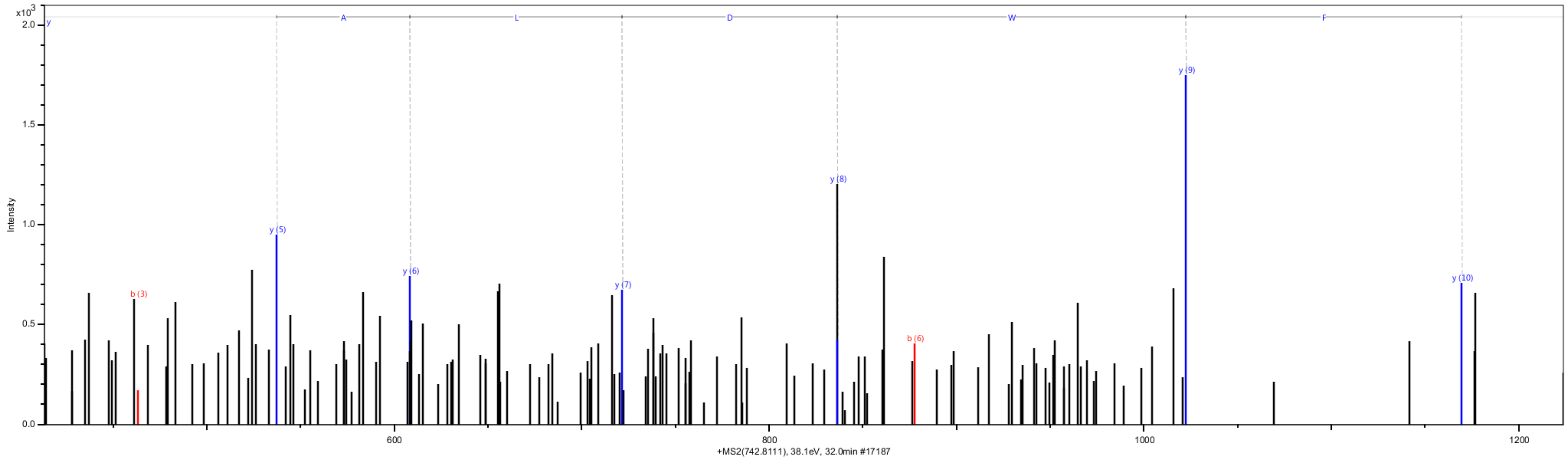

Peptide Match

**Sequence:**	R.YYCFQGNQFLR.F	**Score:**	66.7
**Modifications:**	Carbamidomethyl: 3		

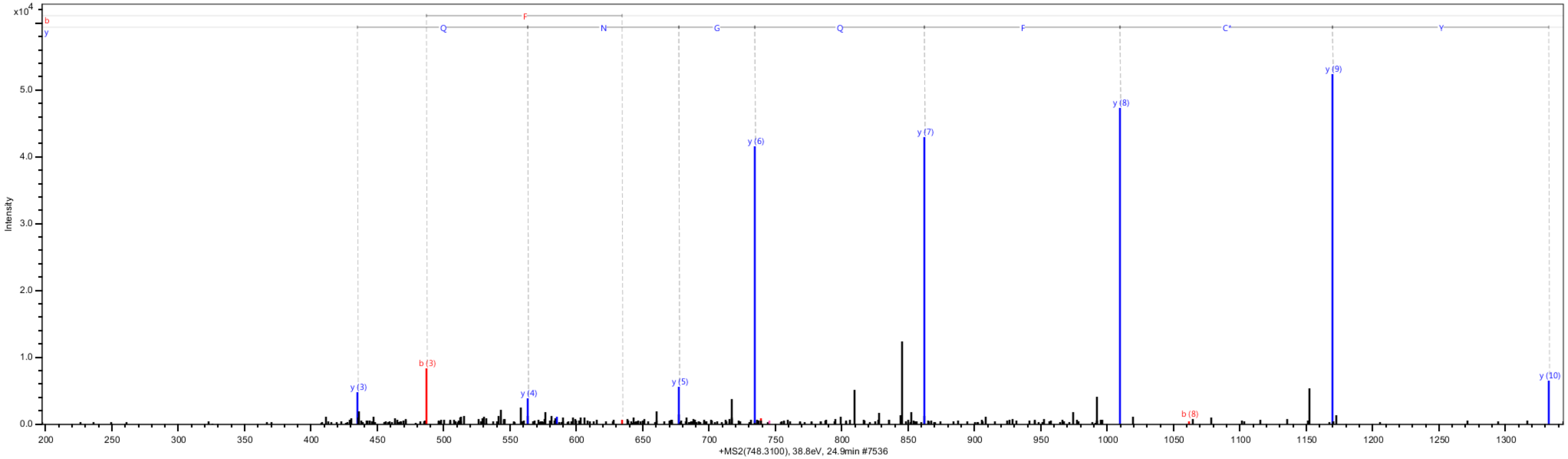

Peptide Match

**Sequence:**	R.DVRDYFMPCPGR.G	**Score:**	16.2
**Modifications:**	Carbamidomethyl: 9		

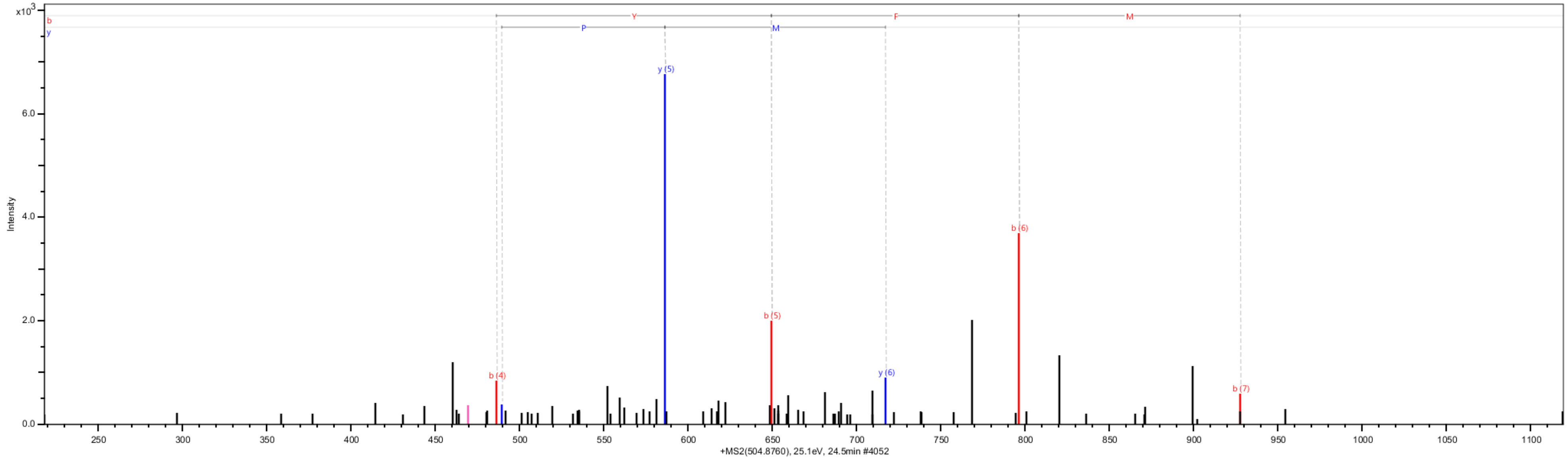

Peptide Match

**Sequence:**	R.DYFMPCPGR.G	**Score:**	15.2
**Modifications:**	Carbamidomethyl: 6		

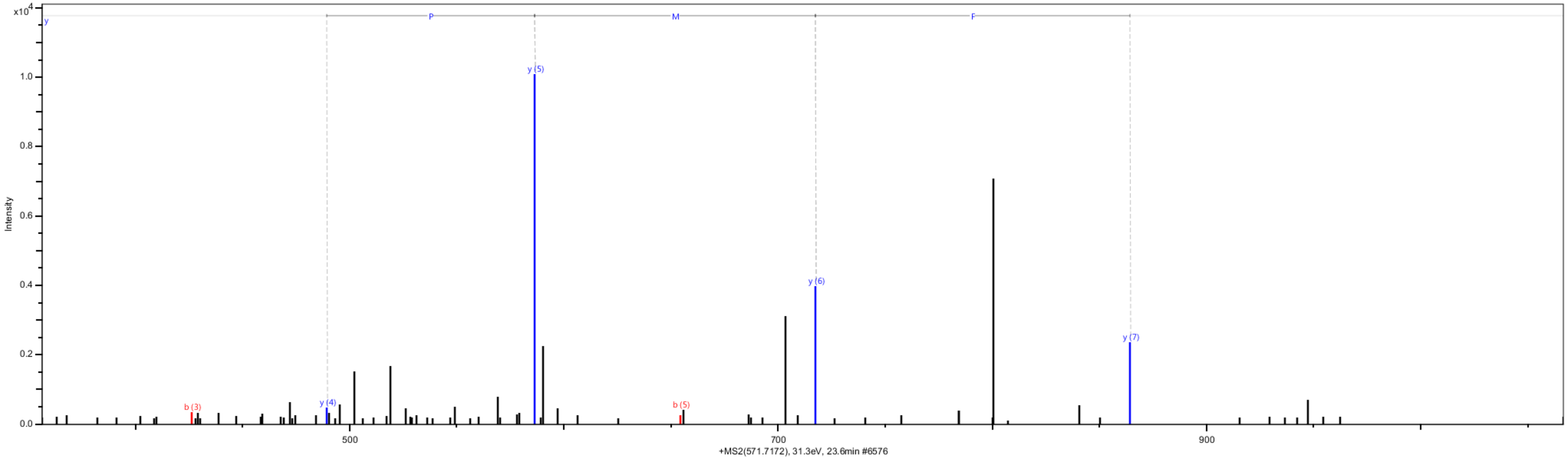

Peptide Match

Sequence: K.GGYTLVSGYPK.R **Score:** 17.2

Modifications:

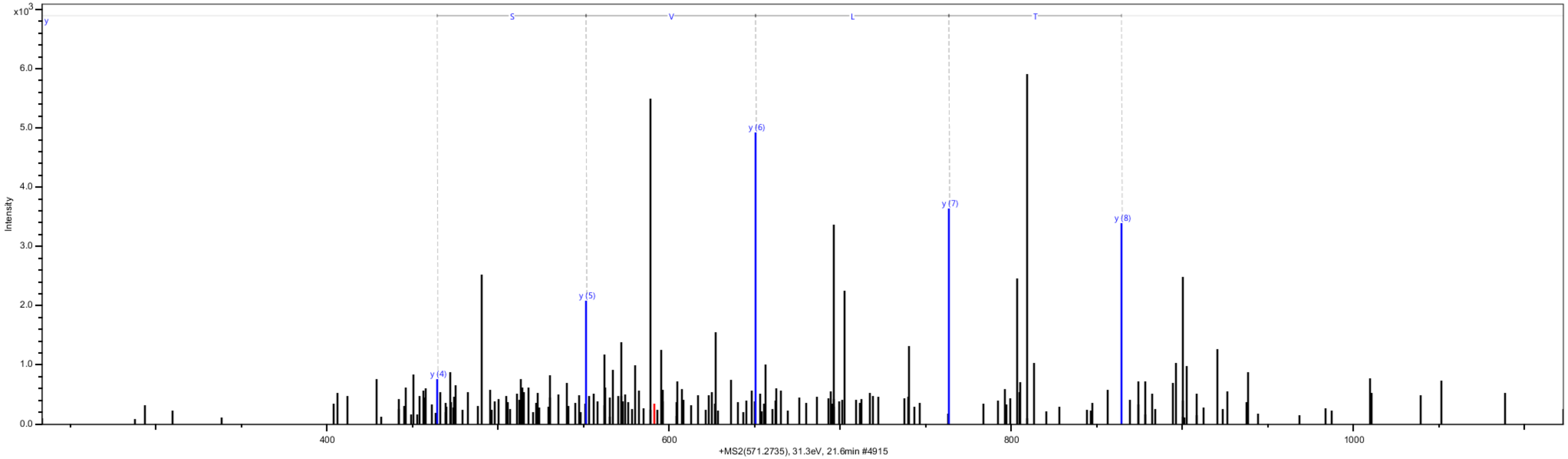

Peptide Match

Sequence: R.RLWWLDLK.S **Score:** 44.8

Modifications:

Spectrum Report
Combine Search_2020-10-19 07:53:31

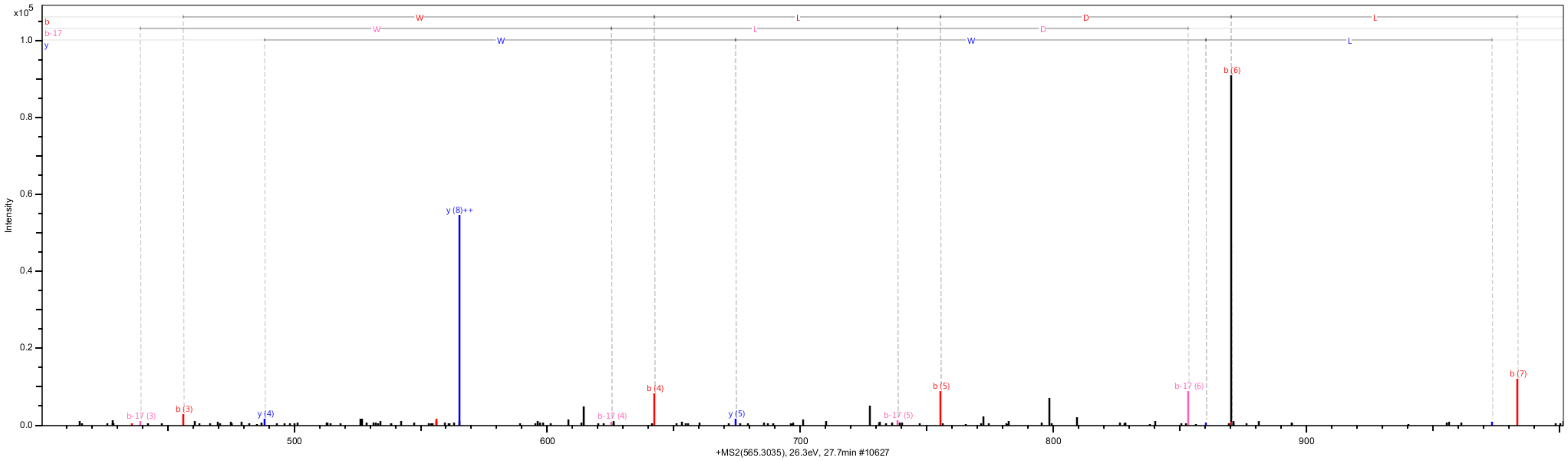

CO4A_HUMAN

Accession: CO4A_HUMAN | **Score:** 435.1
Database: SwissProt | **Seq. Coverage [%]:** 8.3
MW [kDa] / pI: 192.7 / 6.7 | **No. of Peptides:** 13

Peptide Match

Sequence: K.SCGLHQLLR.G | **Score:** 16.2
Modifications: Carbamidomethyl: 2

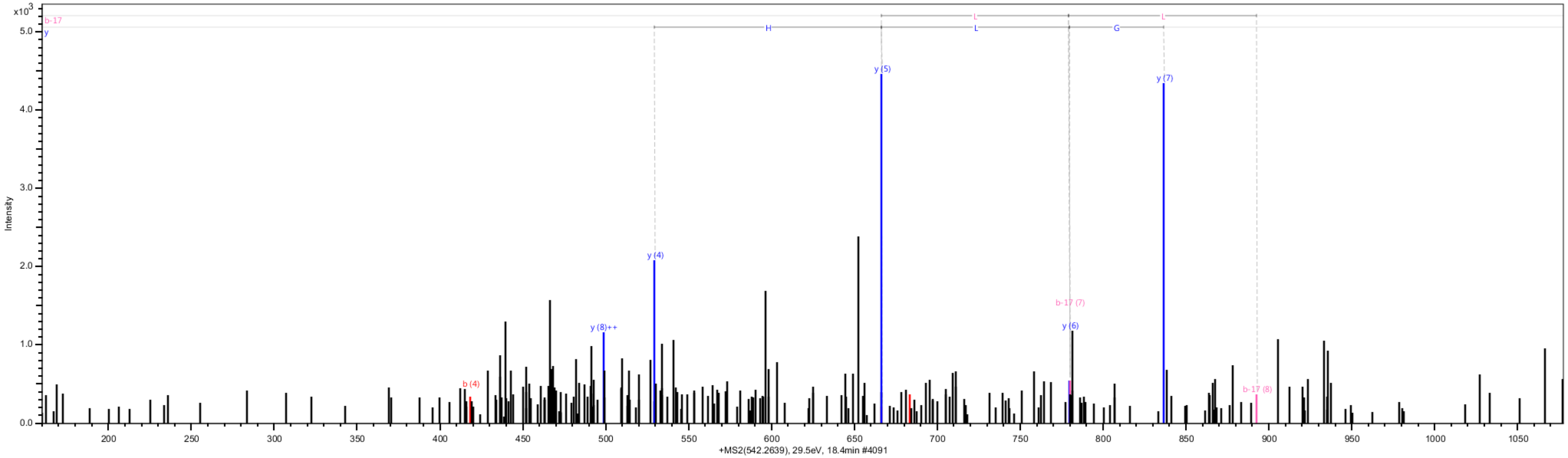

Peptide Match

Sequence: R.TTNIQGINLLFSSR.R | **Score:** 34.6
Modifications:

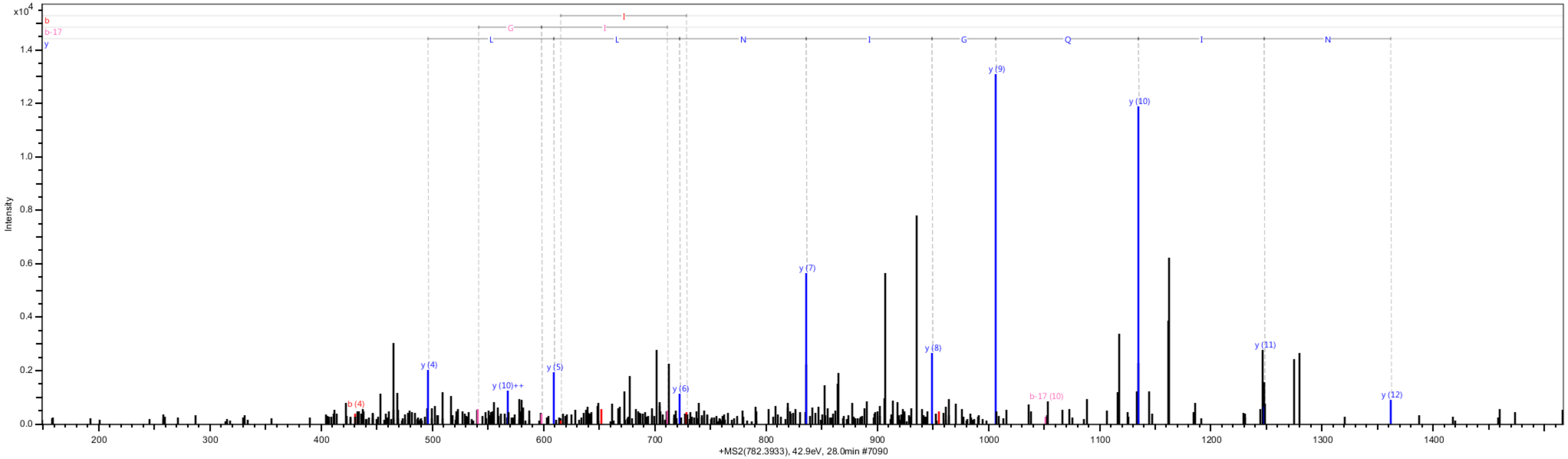

Peptide Match

Sequence: K.YVLPNFEVK.I **Score:** 21.8

Modifications:

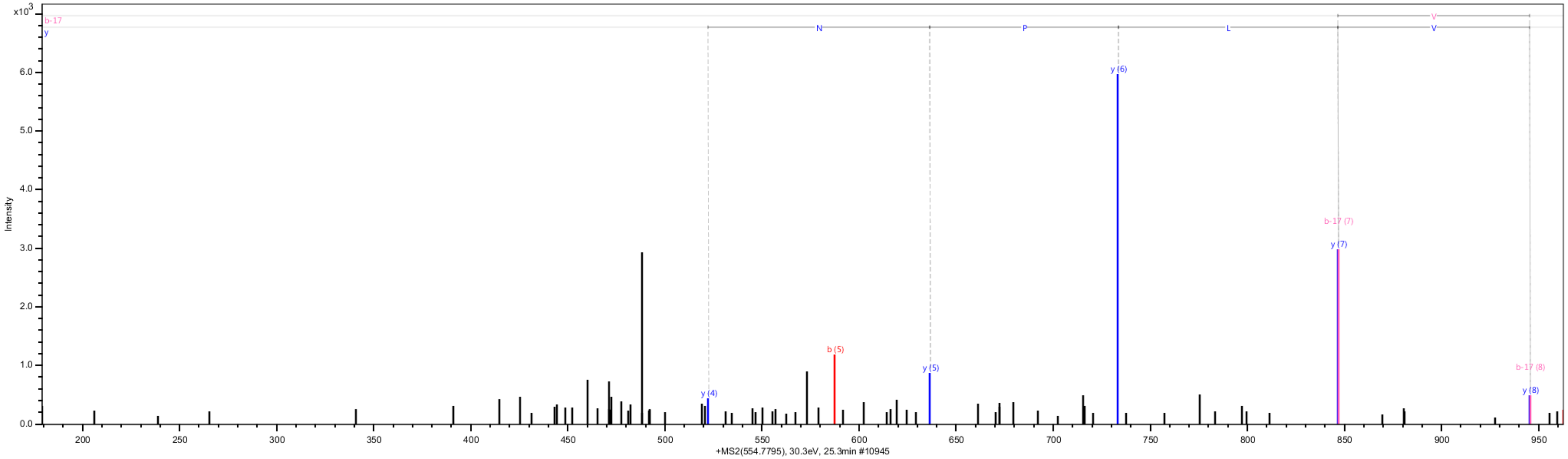

Peptide Match

**Sequence:**	R.YIYGKPVQGVAYVR.F	**Score:**	52.8
**Modifications:**			

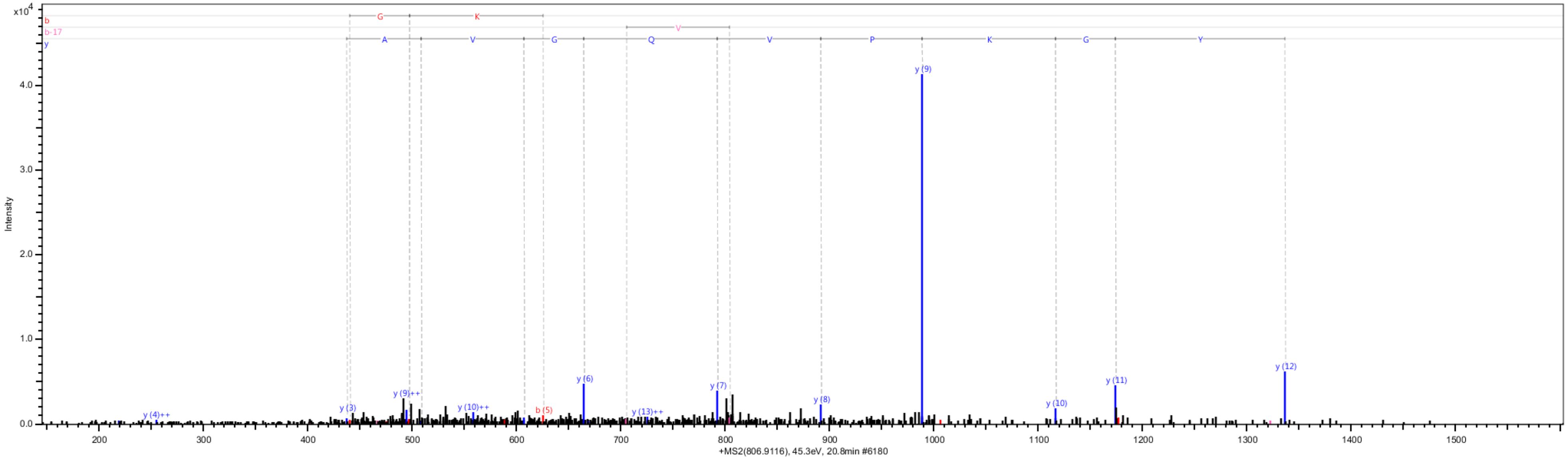

Peptide Match

Sequence: R.HLVPGAPFLLQALVR.E **Score:** 47.7

Modifications:

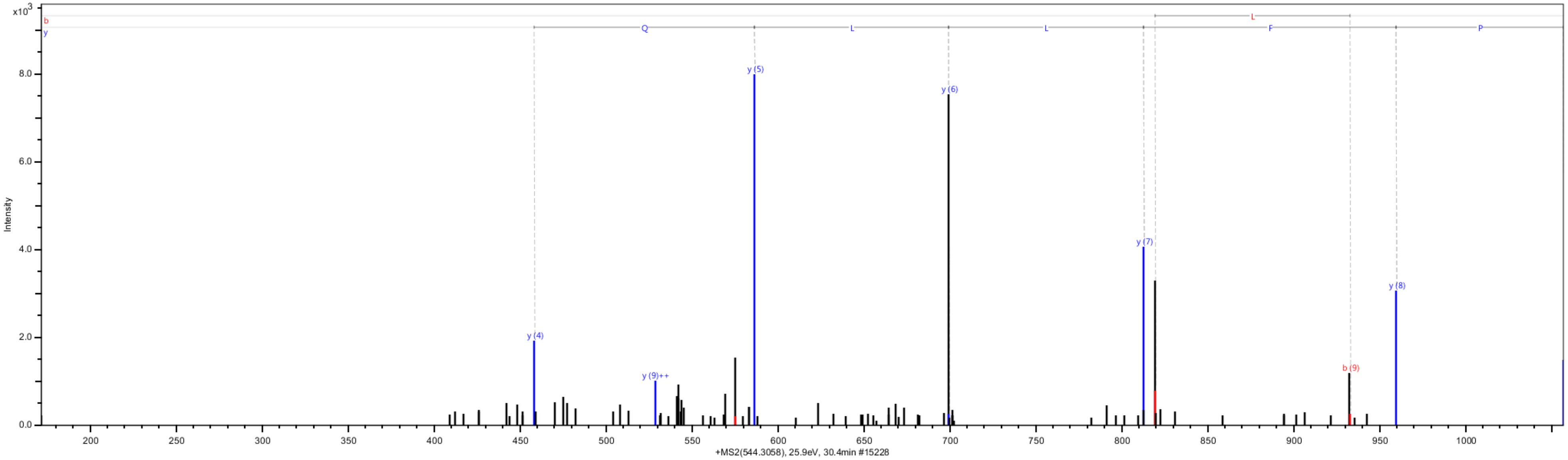

Peptide Match

**Sequence:**	R.VGDTLNLNLR.A	**Score:**	20.3
**Modifications:**			

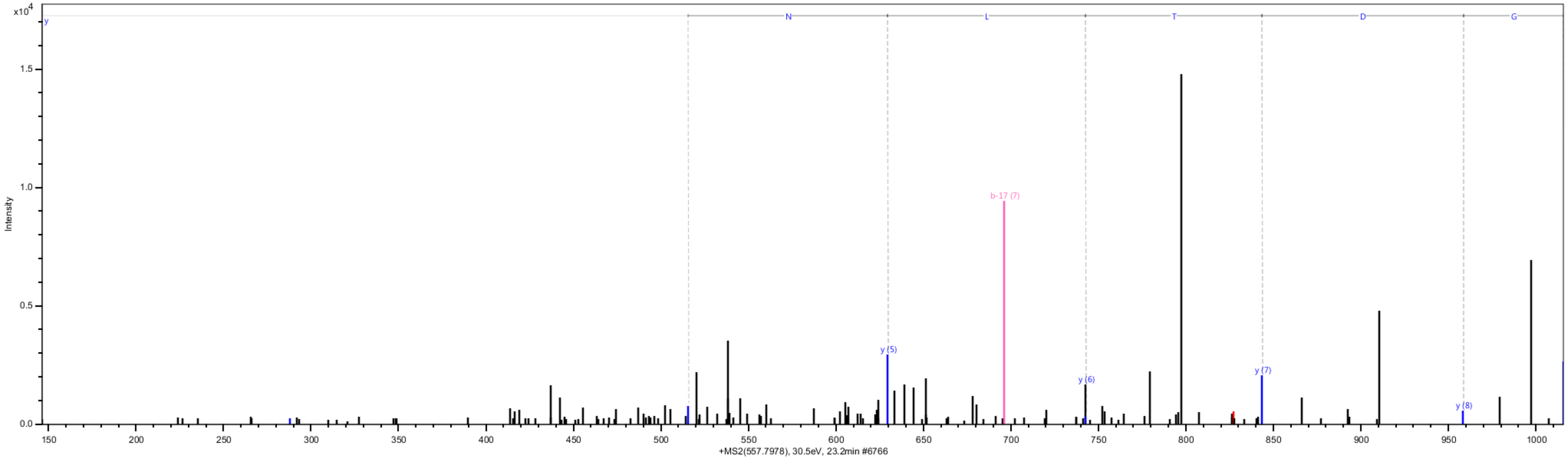

Peptide Match

Sequence: R.SFFPENWLWR.V **Score:** 42.4

Modifications:

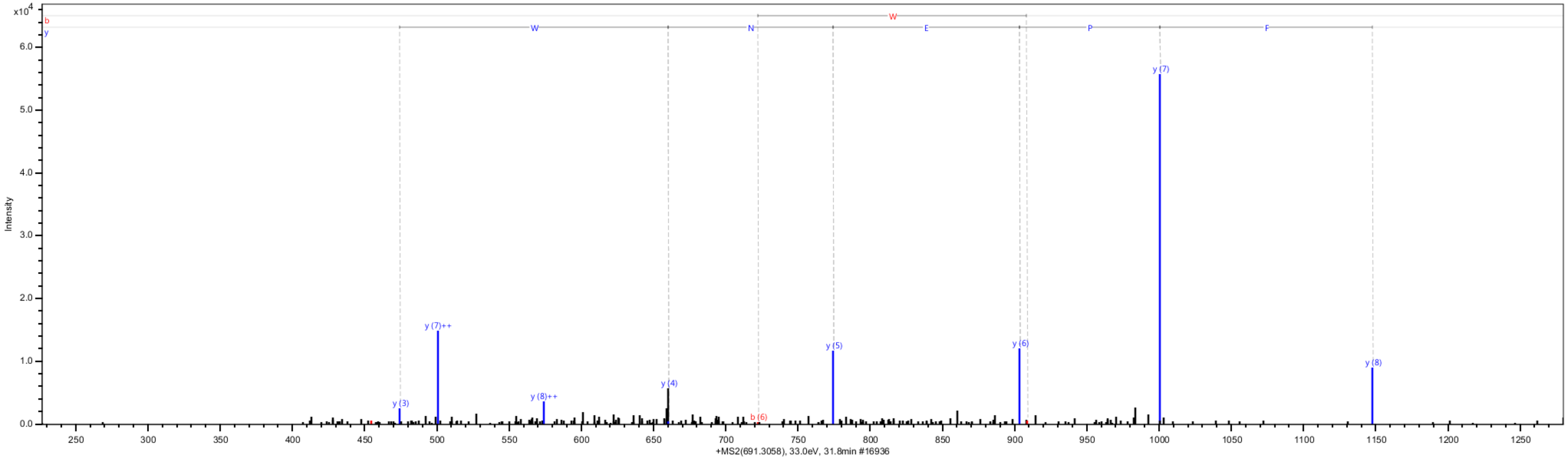

Peptide Match

Sequence: R.DSSTWLTAFVLK.V **Score:** 56.7
Modifications:

Spectrum Report
Combine Search_2020-10-19 07:53:31

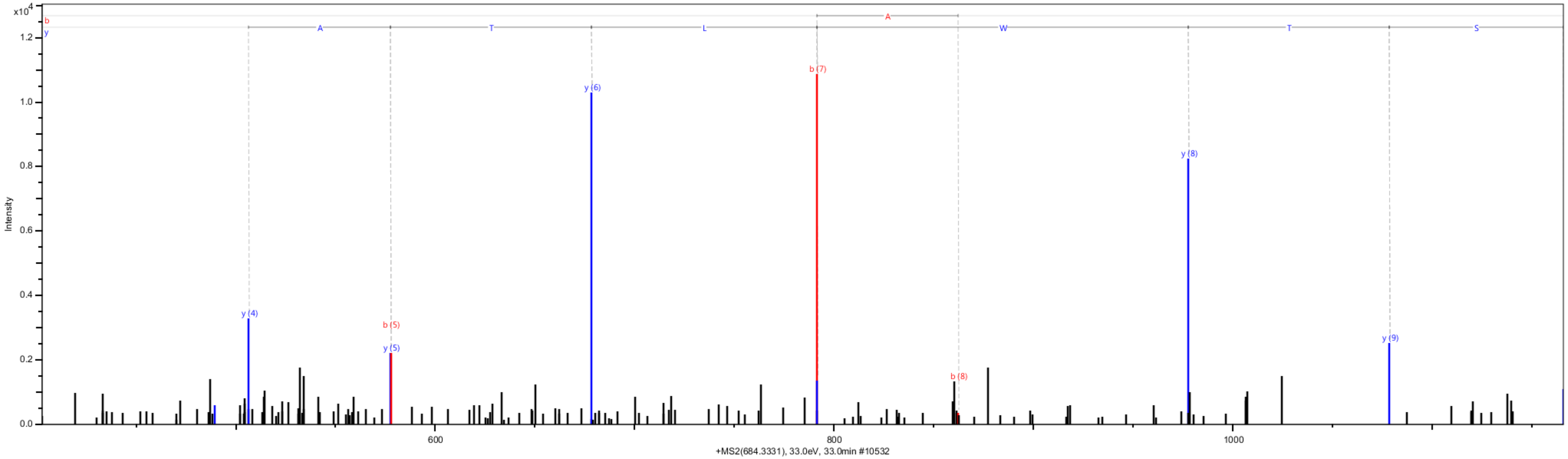

Peptide Match

Sequence: K.VLSLAQEQVGGSPEK.L **Score:** 31.8

Modifications:

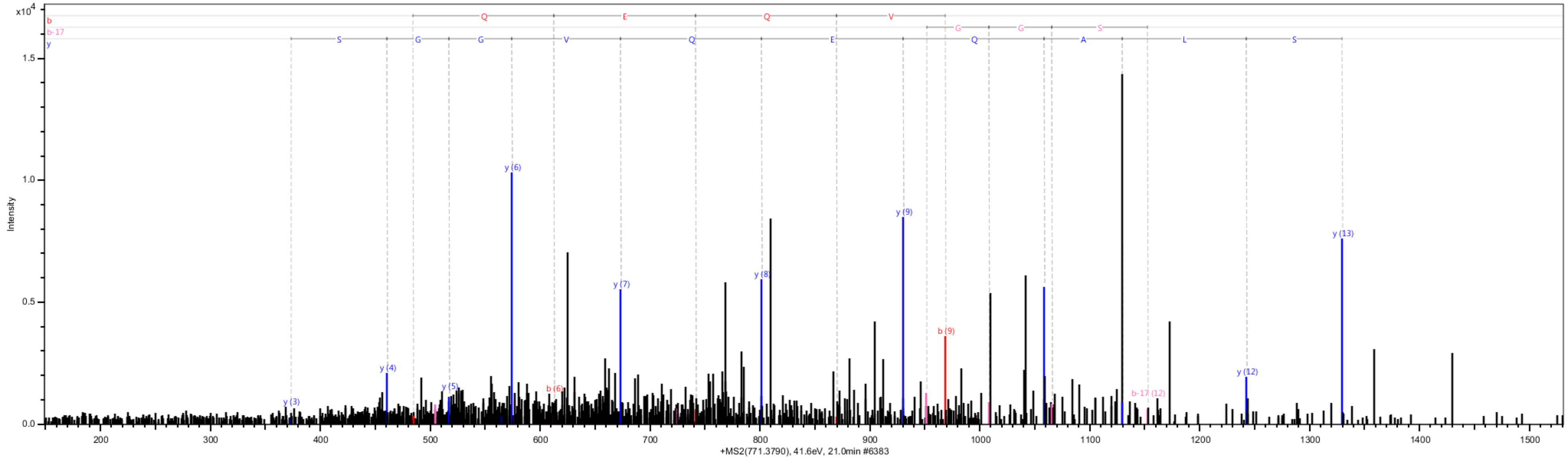

Peptide Match

Sequence: R.GLEEELQFSLGSK.I **Score:** 50.8

Modifications:

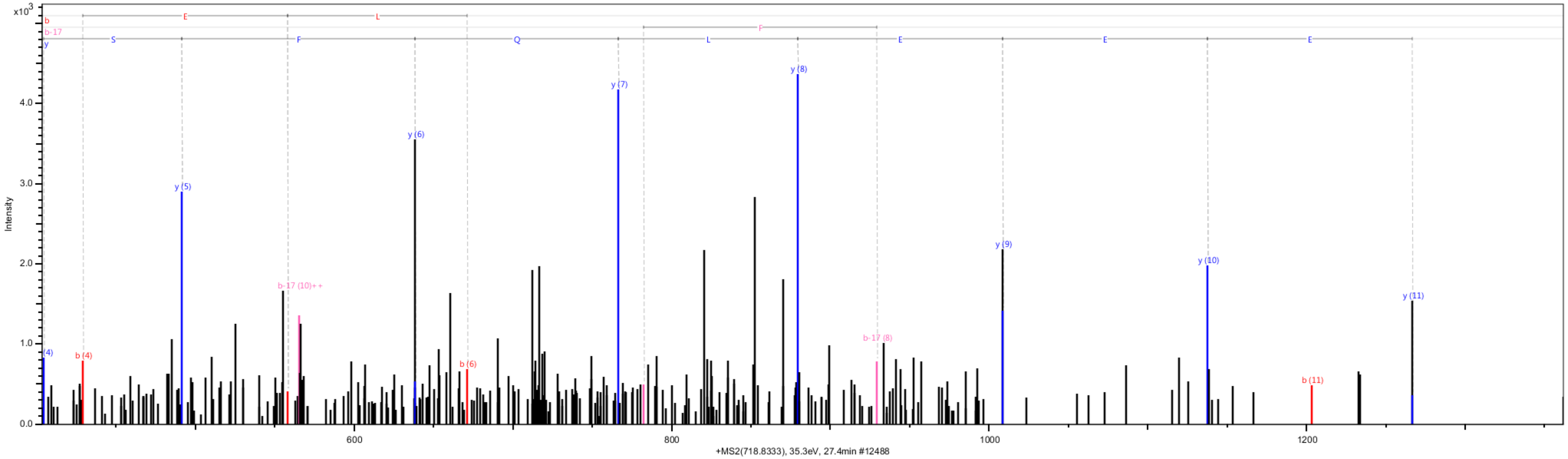

Peptide Match

**Sequence:**	K.FACYYPR.V	**Score:**	23.6
**Modifications:**	Carbamidomethyl: 3		

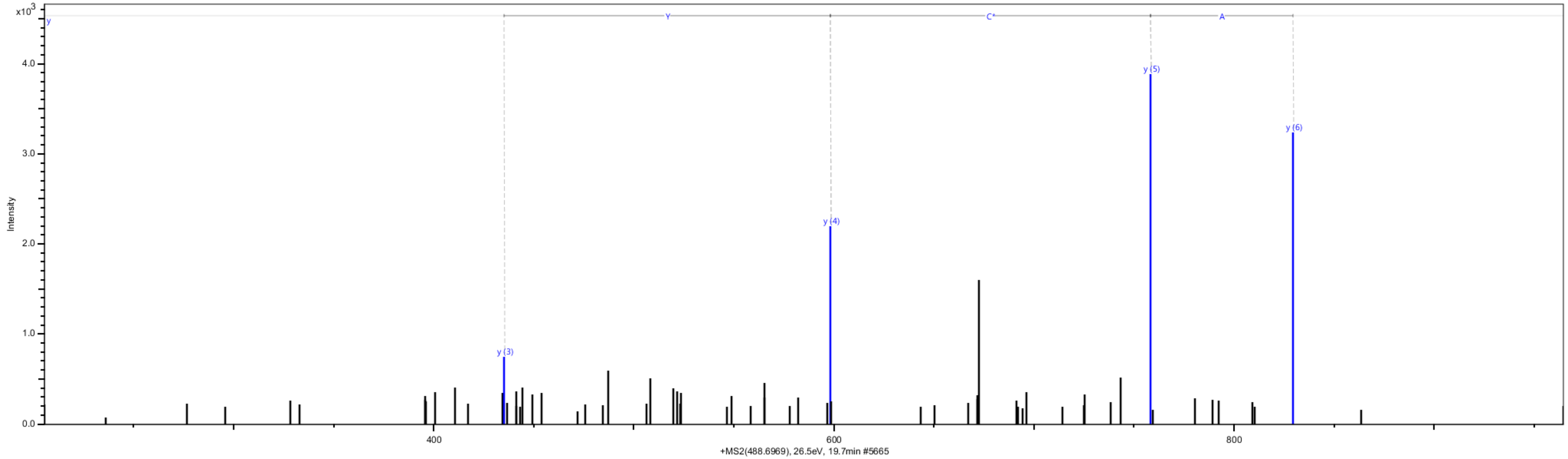

Peptide Match

Sequence: R.VEYGFQVK.V **Score:** 17.1

Modifications:

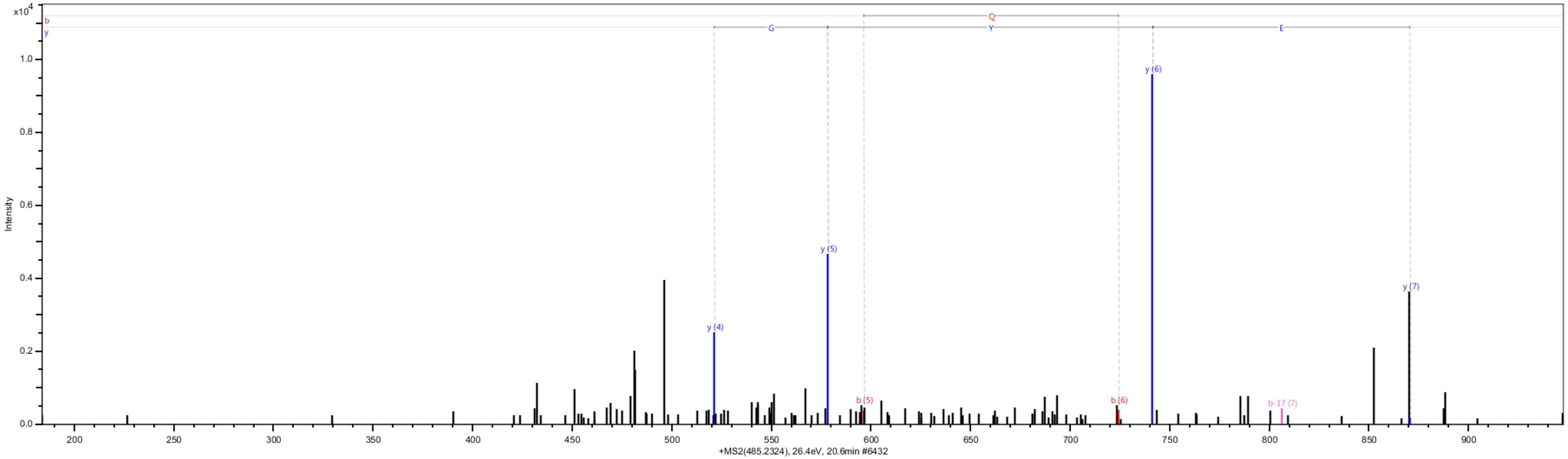

Peptide Match

Sequence: K.ITQVLHFTK.D **Score:** 19.5

Modifications:

Spectrum Report
Combine Search_2020-10-19 07:53:31

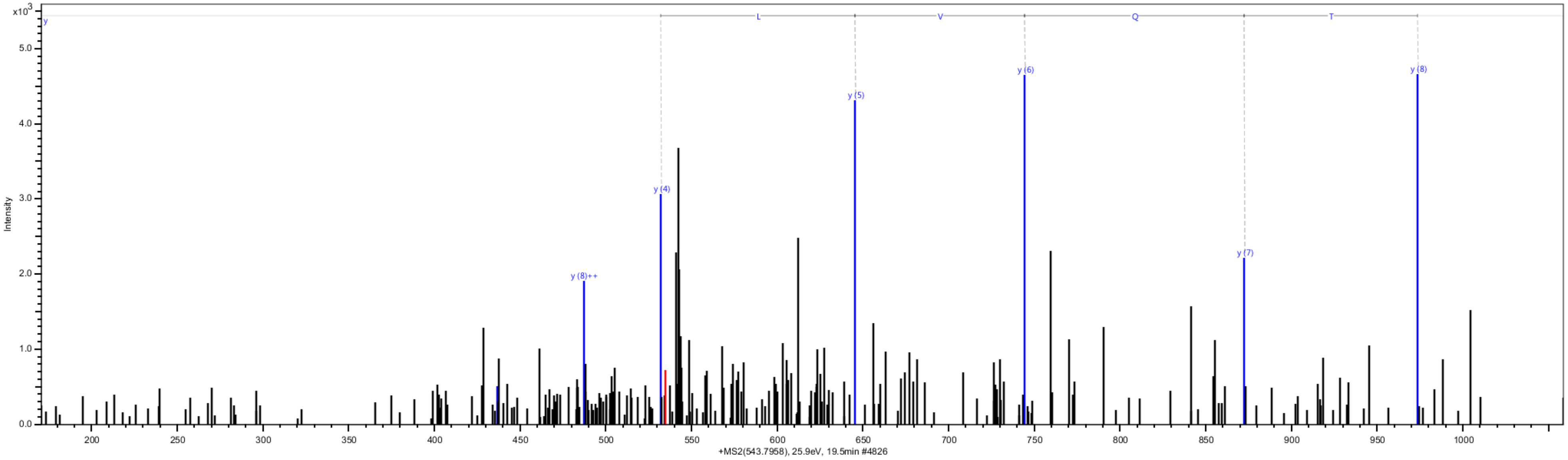

IGKC_HUMAN

Accession: IGKC_HUMAN
Database: SwissProt
MW [kDa] / pI: 11.8 / 6.1
Score: 335.0
Seq. Coverage [%]: 80.4
No. of Peptides: 5

Peptide Match

Sequence: -.RTVAAPSVFIFPPSDEQLK.S **Score:** 25.2
Modifications:

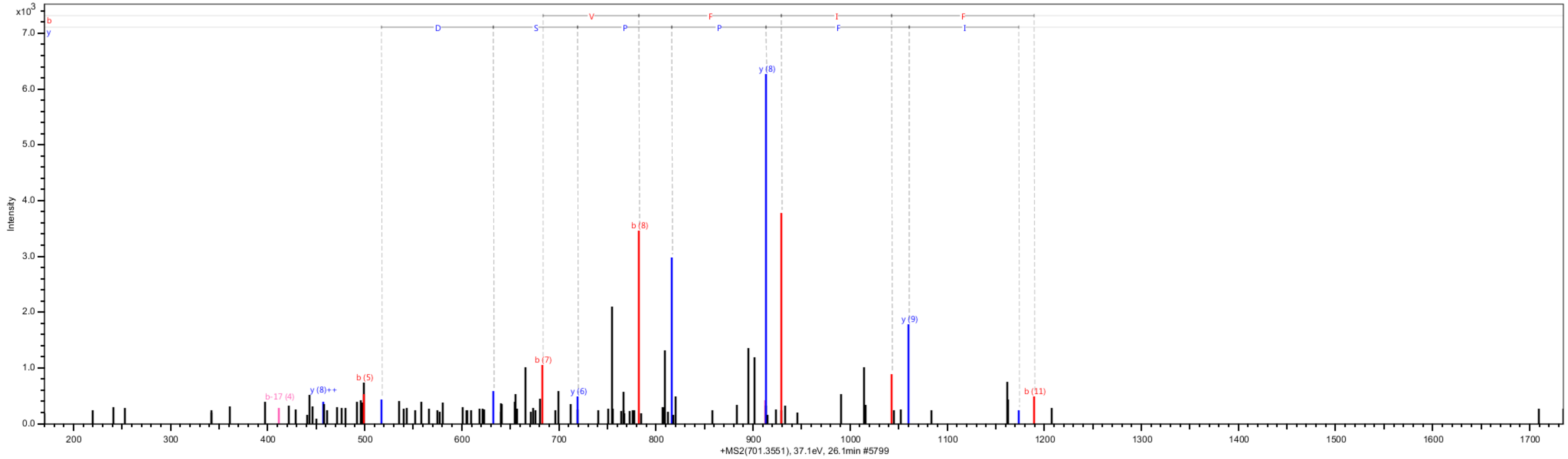

Peptide Match

Sequence: K.SGTASVVCLLNNFYPR.E **Score:** 94.1
Modifications: Carbamidomethyl: 8

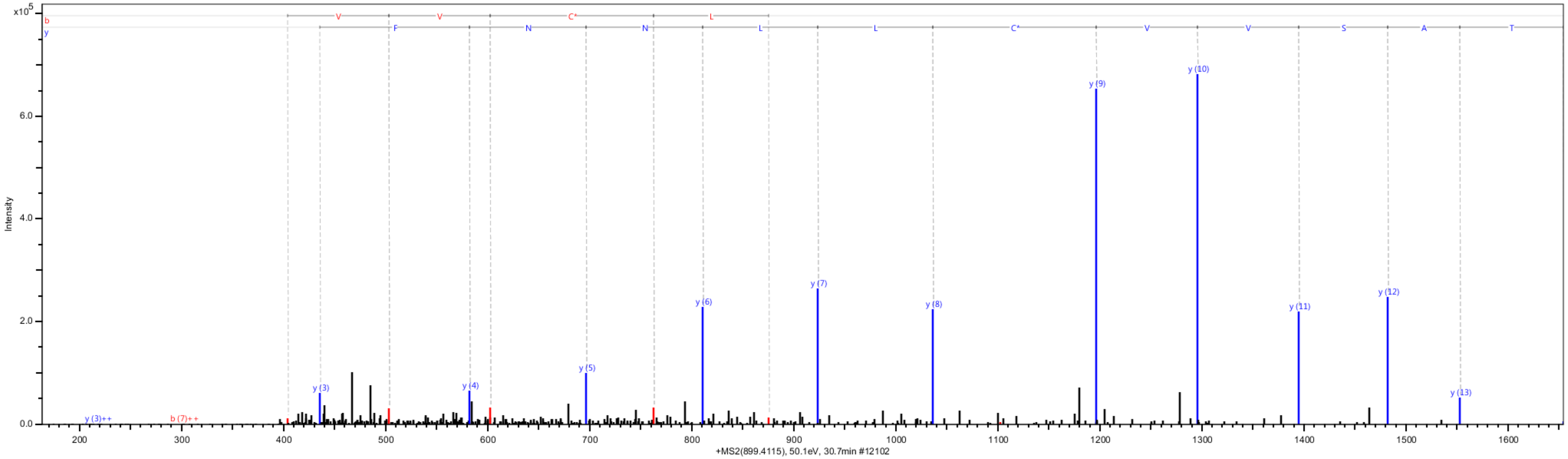

Peptide Match

Sequence: K.VDNALQSGNSQESVTEQDSKDSTYSL SSTLTLSK.A **Score:** 21.9

Modifications:

Spectrum Report
Combine Search_2020-10-19 07:53:31

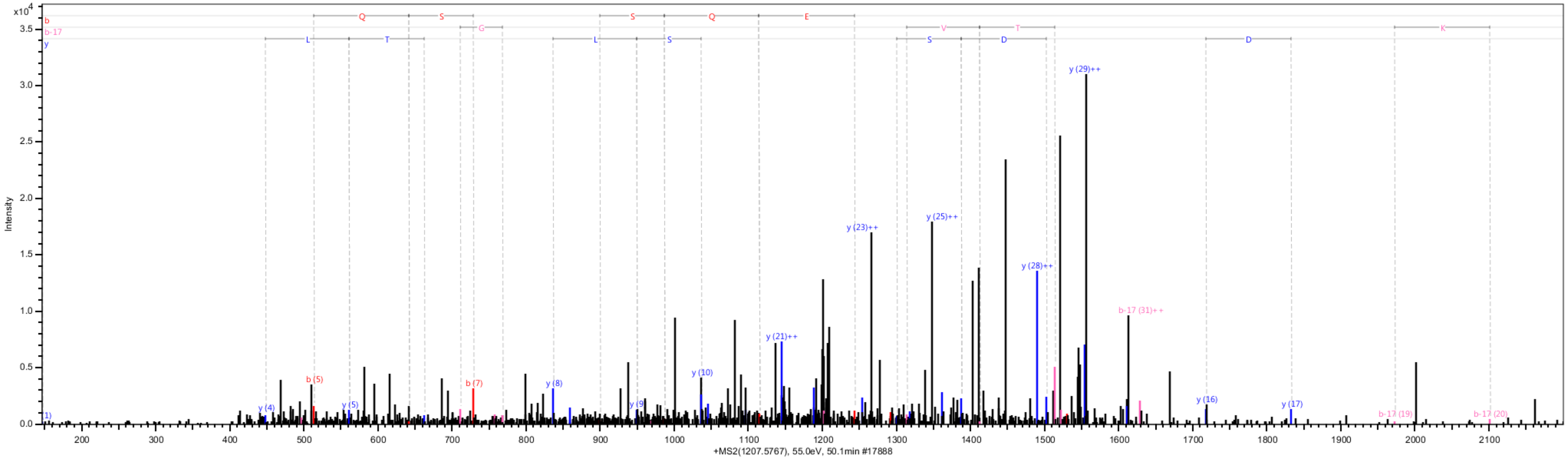

Peptide Match

**Sequence:**	K.DSTYSLSSTLTLSK.A	**Score:**	72.5
**Modifications:**			

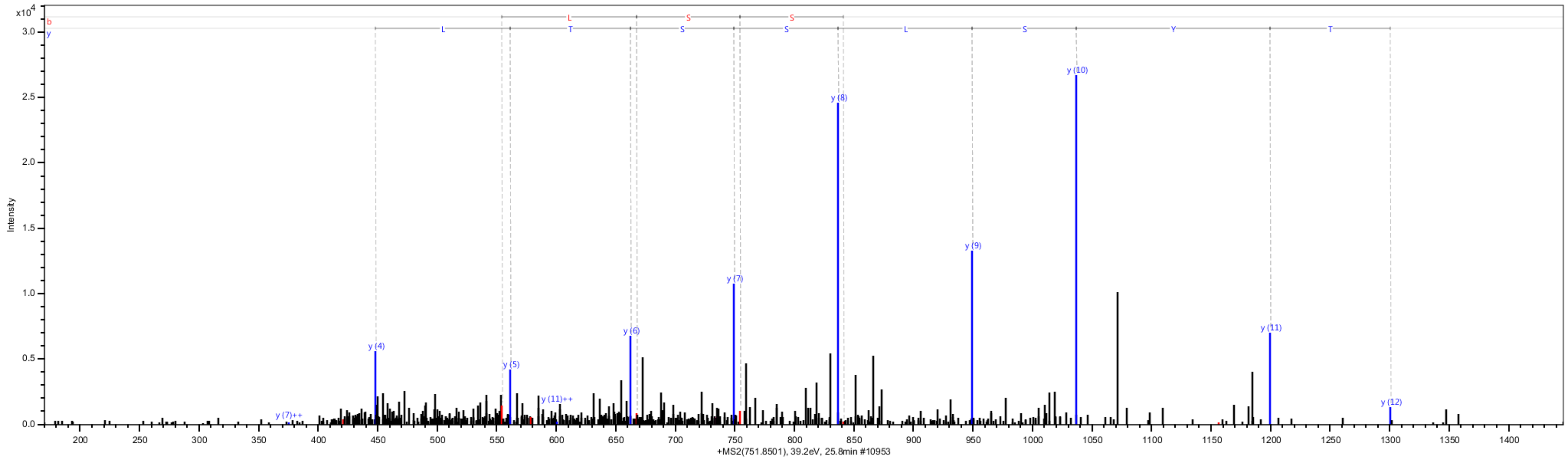

Peptide Match

**Sequence:**	K.VYACEVTHQGLSSPVTK.S	**Score:**	121.3
**Modifications:**	Carbamidomethyl: 4		

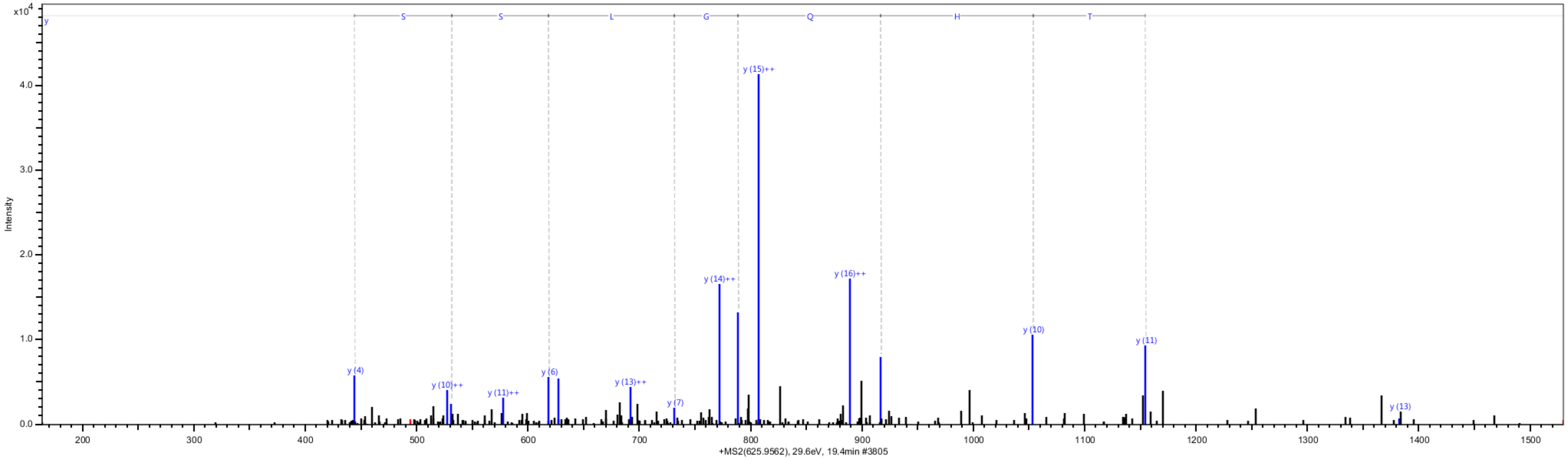
x10^4
Intensity
S
S
L
G
Q
H
T
y (4)
y (10)++
y (11)++
y (6)
y (13)++
y (7)
y (14)++
y (15)++
y (16)++
y (10)
y (11)
y (13)
+MS2(625.9562), 29.6eV, 19.4min #3805

TEN4_HUMAN

Accession: TEN4_HUMAN
Database: SwissProt
MW [kDa] / pI: 307.8 / 6.1
Score: 21.4
Seq. Coverage [%]: 0.3
No. of Peptides: 1

Peptide Match

Sequence: K.MRLSYLSSR.T
Score: 21.4
Modifications:

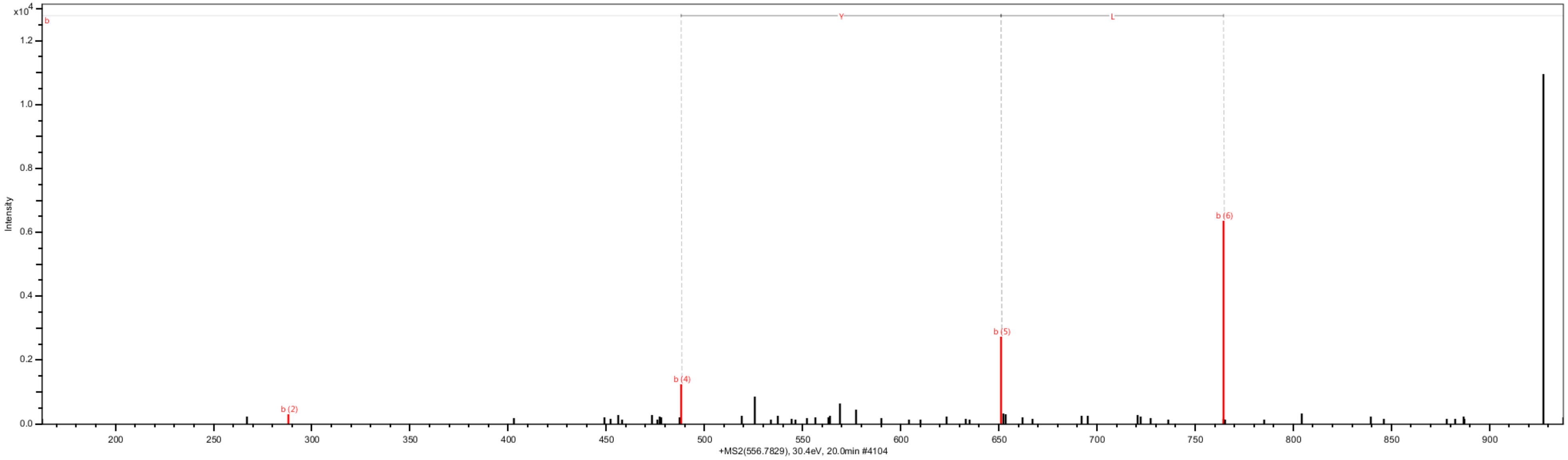

CA137_HUMAN

Accession: CA137_HUMAN
Database: SwissProt
MW [kDa] / pI: 11.0 / 7.6
Score: 21.4
Seq. Coverage [%]: 9.2
No. of Peptides: 1

Peptide Match

Sequence: K.VCKPQAPQK.L
Modifications: Carbamidomethyl: 2
Score: 21.4

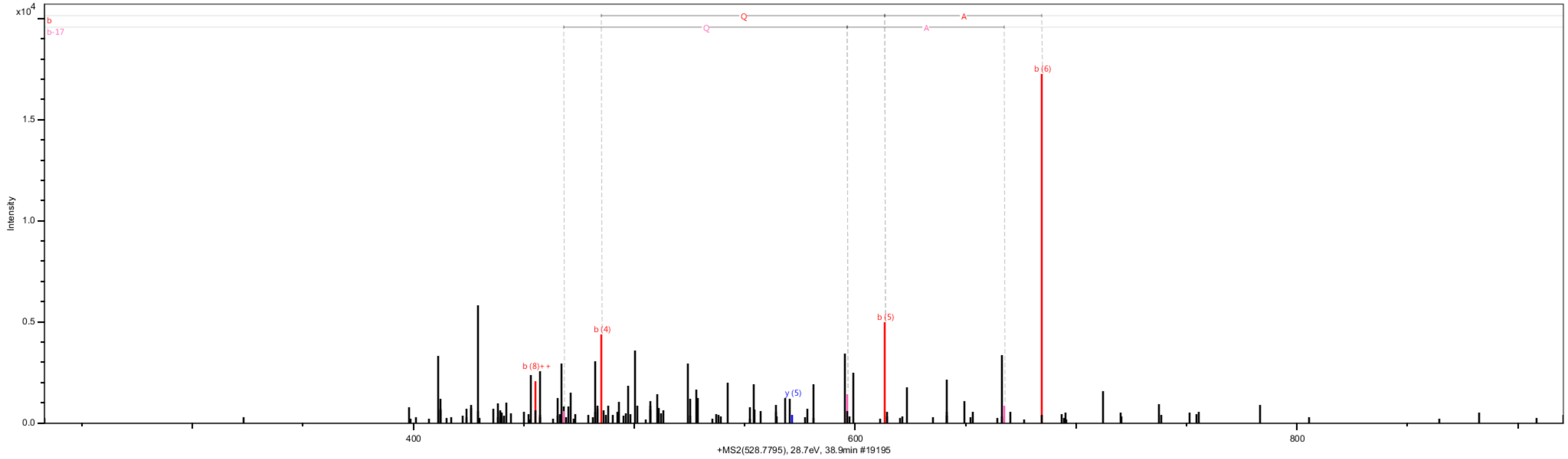

MPP5_HUMAN

Accession: MPP5_HUMAN
Database: SwissProt
MW [kDa] / pI: 77.2 / 5.8
Score: 21.4
Seq. Coverage [%]: 1.5
No. of Peptides: 1

Peptide Match

Sequence: R.NEMDSVIISR.I
Score: 21.4
Modifications: Oxidation: 3

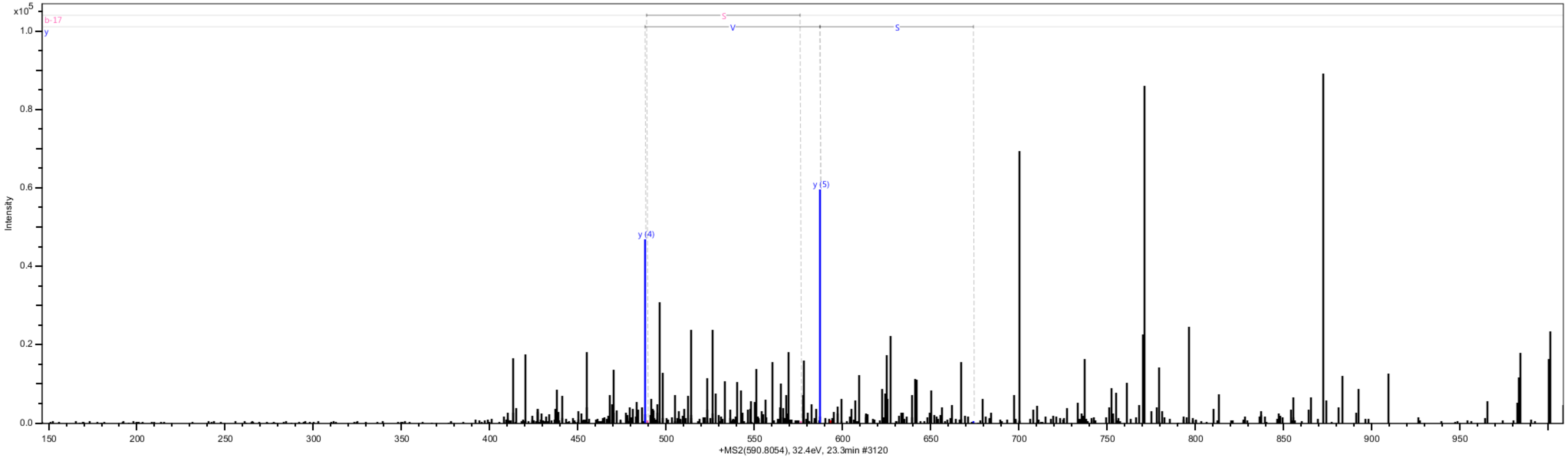

DCTN5_HUMAN

**Accession:**	DCTN5_HUMAN	**Score:**	21.4
**Database:**	SwissProt	**Seq. Coverage [%]:**	5.5
**MW [kDa] / pI:**	20.1 / 8.3	**No. of Peptides:**	1

Peptide Match

**Sequence:**	-.MELGELLYNK.S	**Score:**	21.4
**Modifications:**	Oxidation: 1		

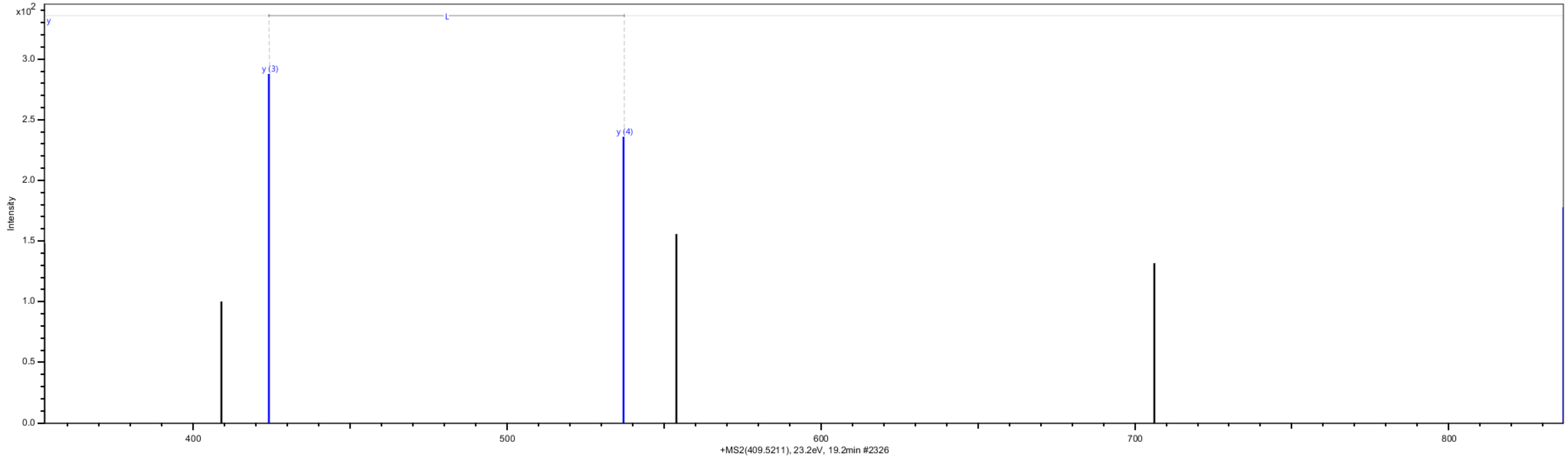

PDS5A_HUMAN

**Accession:**	PDS5A_HUMAN	**Score:**	21.6
**Database:**	SwissProt	**Seq. Coverage [%]:**	0.5
**MW [kDa] / pI:**	150.7 / 8.2	**No. of Peptides:**	1

Peptide Match

**Sequence:**	R.ELLDLHK.Q	**Score:**	21.6
**Modifications:**			

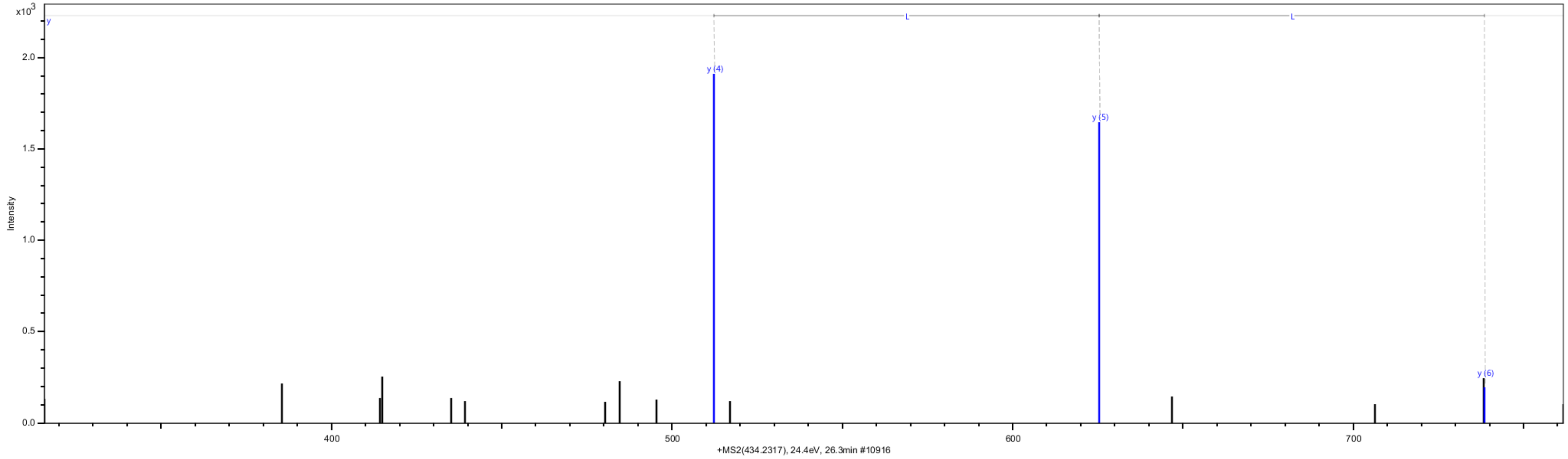

HNRPU_HUMAN

**Accession:**	HNRPU_HUMAN	**Score:**	21.5
**Database:**	SwissProt	**Seq. Coverage [%]:**	1.2
**MW [kDa] / pI:**	90.5 / 5.8	**No. of Peptides:**	1

Peptide Match

**Sequence:**	-.MSSSPVNVKK.L	**Score:**	21.5
**Modifications:**			

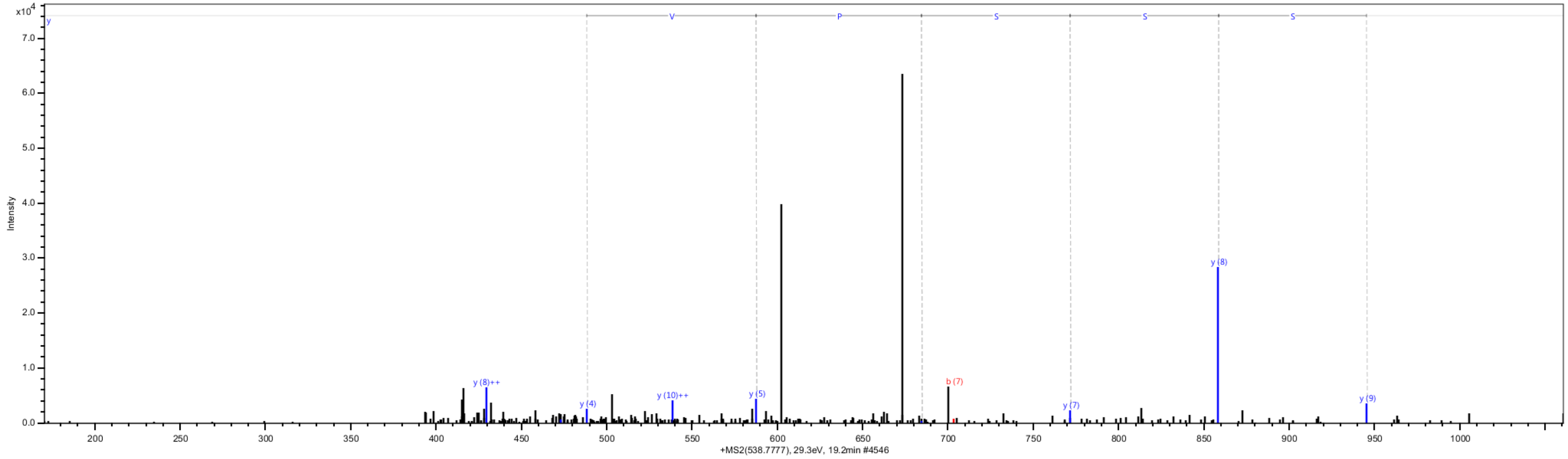

WNT16_HUMAN

Accession: WNT16_HUMAN
Database: SwissProt
MW [kDa] / pI: 40.7 / 9.0
Score: 21.5
Seq. Coverage [%]: 3.8
No. of Peptides: 1

Peptide Match

Sequence: R.LGIQECGSQFRHER.W
Score: 21.5
Modifications: Carbamidomethyl: 6

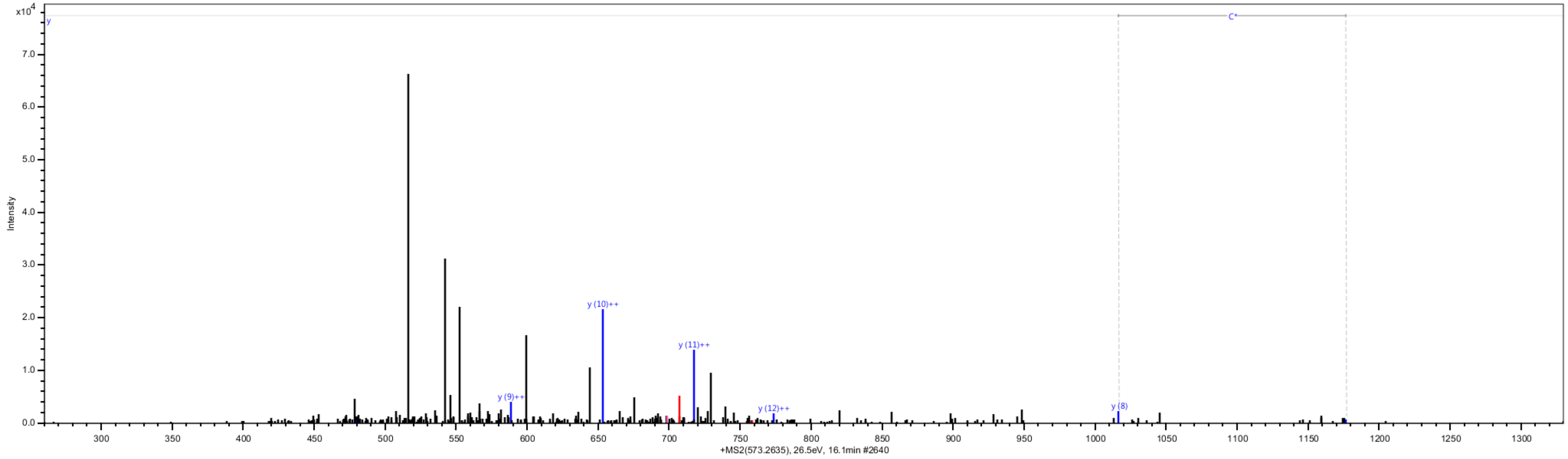

AHDC1_HUMAN

Accession: AHDC1_HUMAN
Database: SwissProt
MW [kDa] / pI: 168.2 / 9.2
Score: 21.1
Seq. Coverage [%]: 0.7
No. of Peptides: 1

Peptide Match

Sequence: R.CSFSDFFEGIGK.K
Score: 21.1
Modifications: Carbamidomethyl: 1

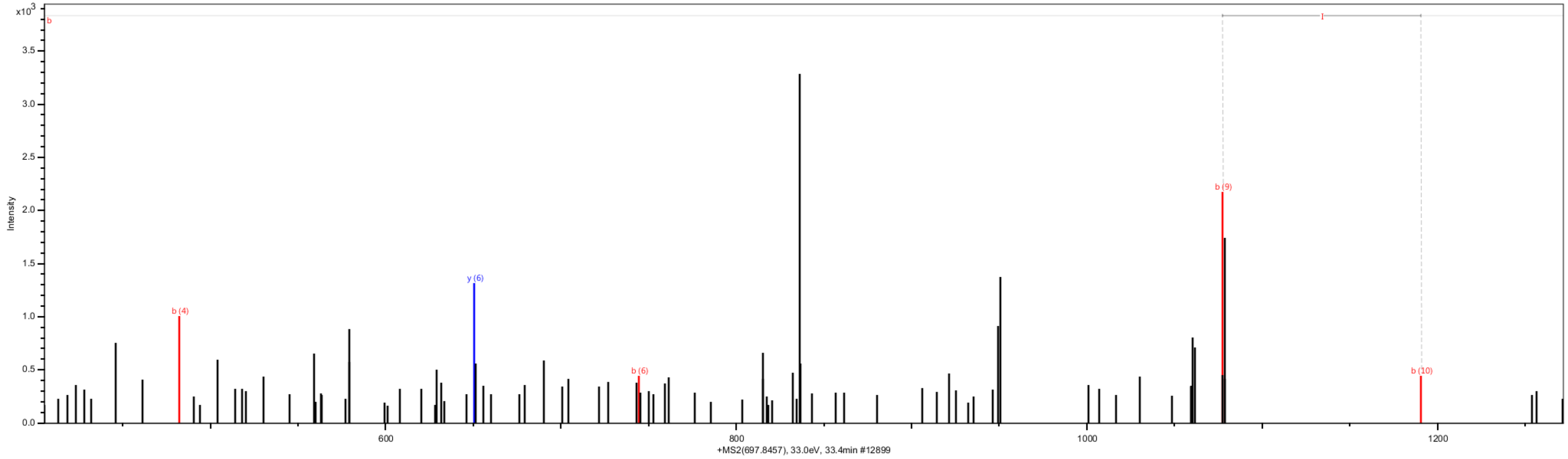

HEP2_HUMAN

**Accession:**	HEP2_HUMAN	**Score:**	21.1
**Database:**	SwissProt	**Seq. Coverage [%]:**	1.8
**MW [kDa] / pI:**	57.0 / 6.4	**No. of Peptides:**	1

Peptide Match

**Sequence:**	K.QFPILLDFK.T	**Score:**	21.1
**Modifications:**			

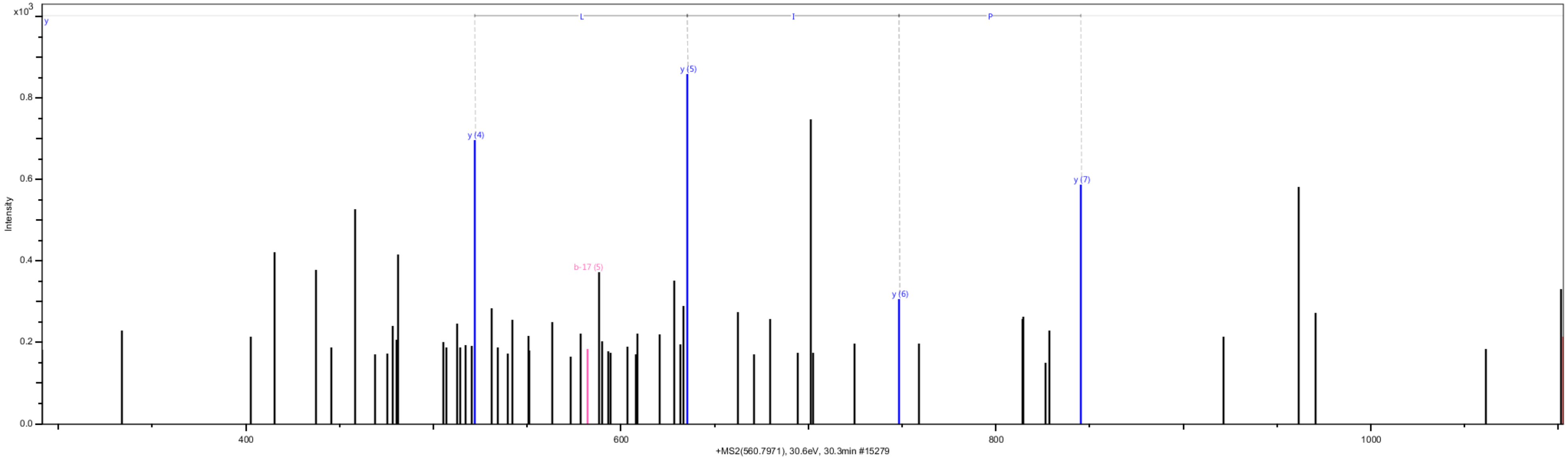

TEKT4_HUMAN

Accession: TEKT4_HUMAN
Database: SwissProt
MW [kDa] / pI: 50.6 / 6.0
Score: 21.0
Seq. Coverage [%]: 2.3
No. of Peptides: 1

Peptide Match

Sequence: R.TIMQAVSQIR.L
Score: 21
Modifications: Oxidation: 3

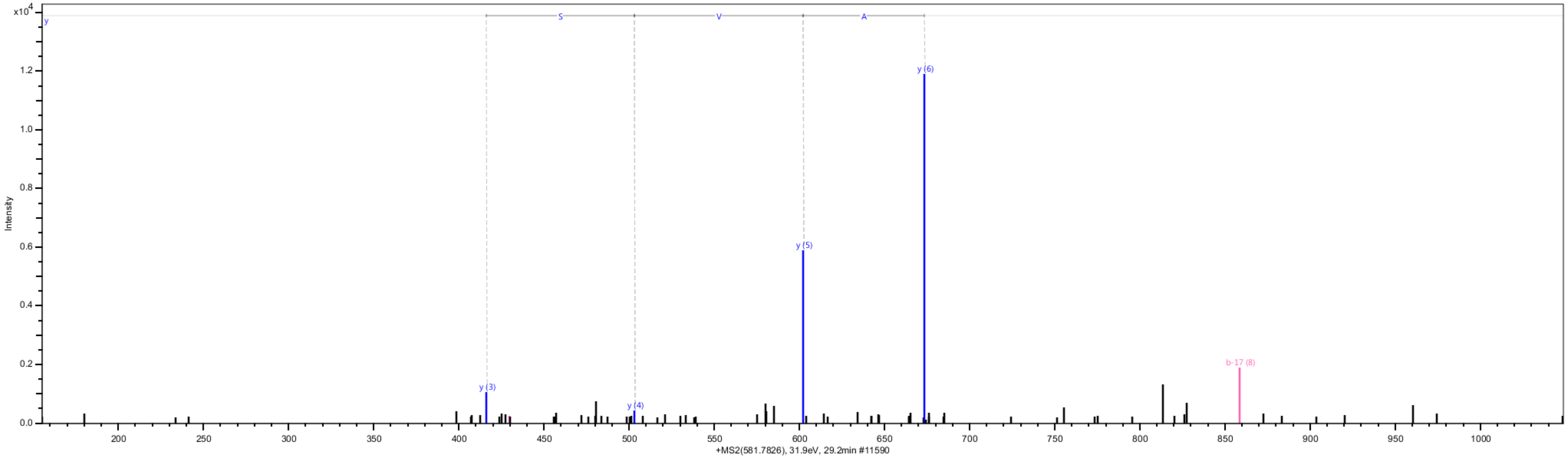

TTC7B_HUMAN

**Accession:**	TTC7B_HUMAN	**Score:**	21.0
**Database:**	SwissProt	**Seq. Coverage [%]:**	2.5
**MW [kDa] / pI:**	94.1 / 6.4	**No. of Peptides:**	1

Peptide Match

**Sequence:**	K.FAFEEFHLWYQFALSLMAAGK.S	**Score:**	21
**Modifications:**	Oxidation: 17		

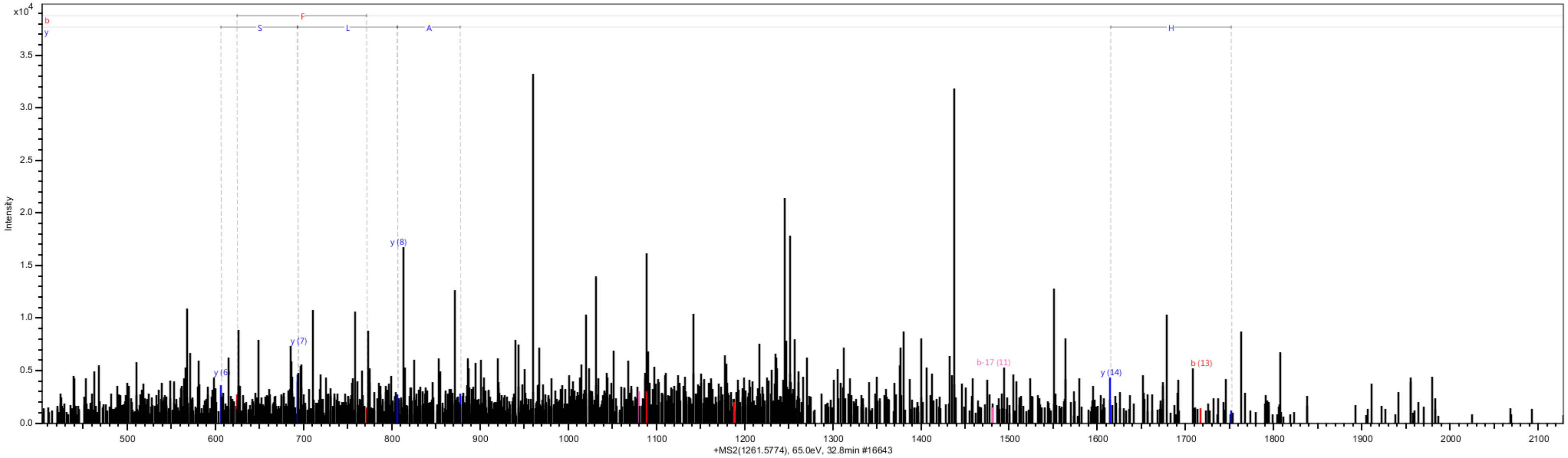

LRC41_HUMAN

Accession: LRC41_HUMAN
Database: SwissProt
MW [kDa] / pI: 88.6 / 8.8
Score: 21.3
Seq. Coverage [%]: 1.2
No. of Peptides: 1

Peptide Match

Sequence: K.QMPRAAPATR.V
Score: 21.3
Modifications: Oxidation: 2

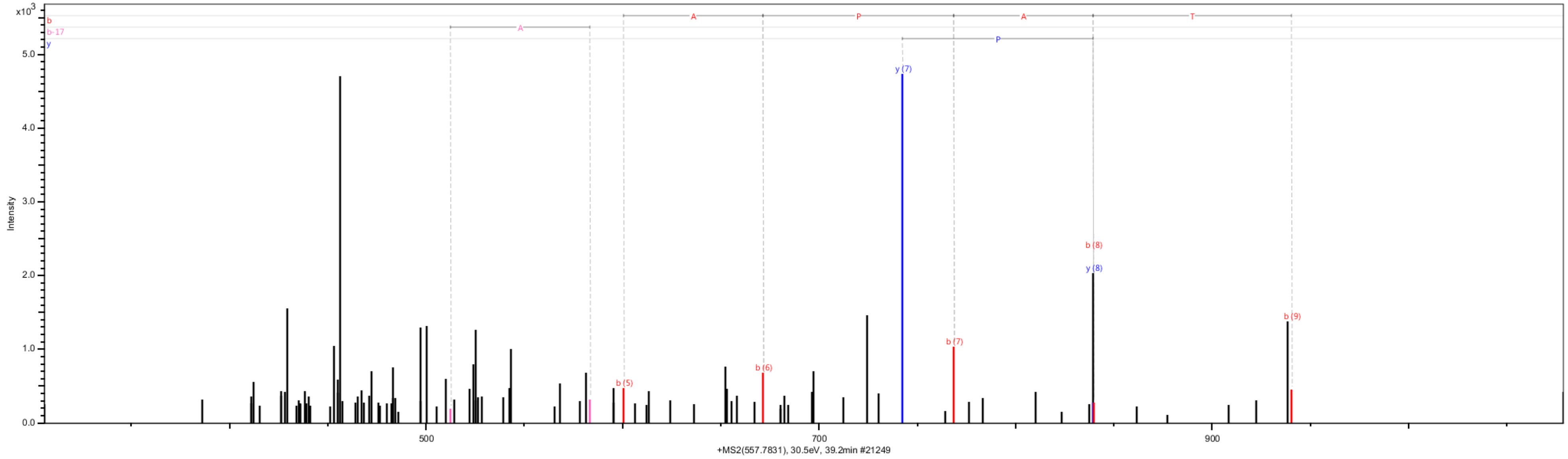

BMP15_HUMAN

Accession: BMP15_HUMAN
Database: SwissProt
MW [kDa] / pI: 45.0 / 9.3
Score: 21.3
Seq. Coverage [%]: 2.6
No. of Peptides: 1

Peptide Match

Sequence: R.RSADSHGHPR.E
Score: 21.3
Modifications:

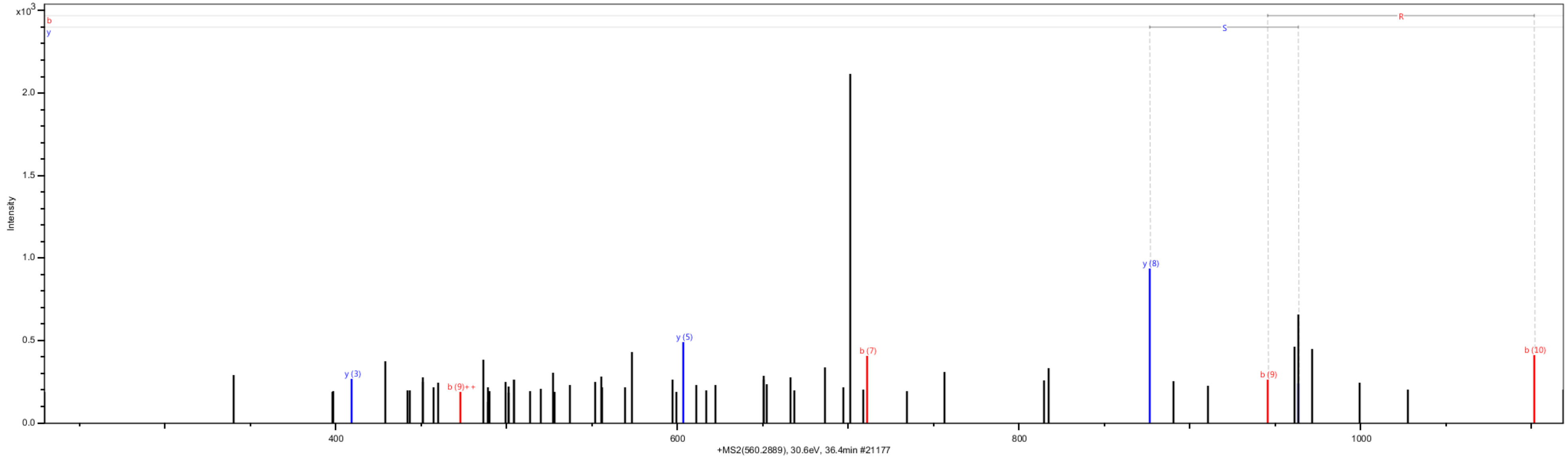

SSU72_HUMAN

**Accession:**	SSU72_HUMAN	**Score:**	21.2
**Database:**	SwissProt	**Seq. Coverage [%]:**	3.6
**MW [kDa] / pI:**	22.6 / 5.2	**No. of Peptides:**	1

Peptide Match

**Sequence:**	R.IKPRPER.F	**Score:**	21.2
**Modifications:**			

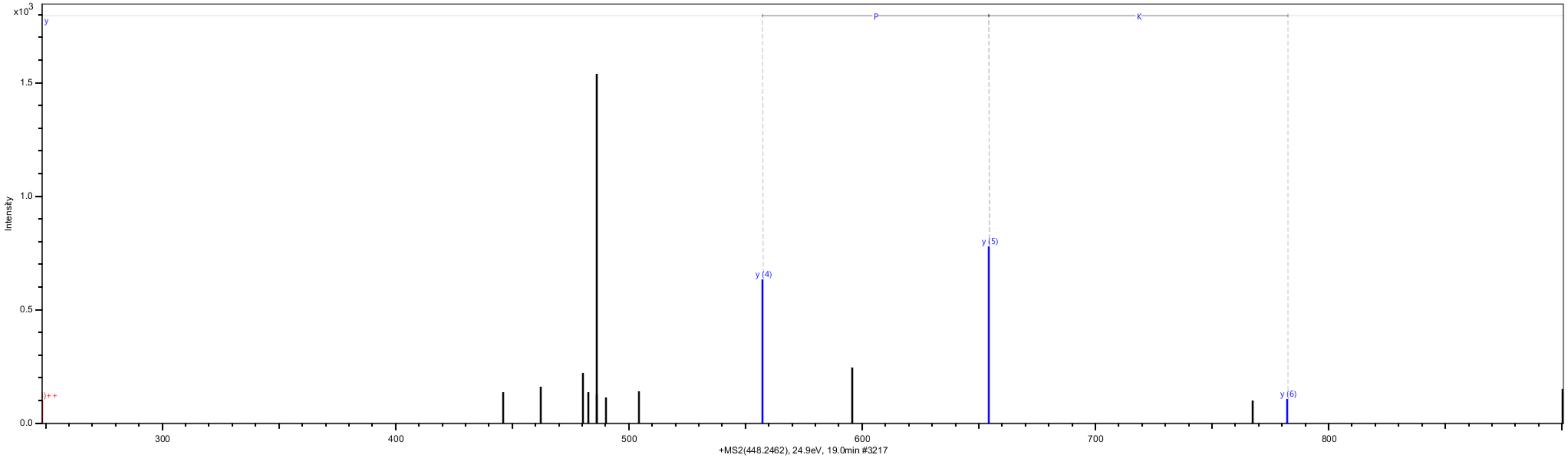

IMA1_HUMAN

Accession: IMA1_HUMAN
Database: SwissProt
MW [kDa] / pI: 57.8 / 5.3
Score: 21.2
Seq. Coverage [%]: 1.7
No. of Peptides: 1

Peptide Match

Sequence: R.IEVNVELRK.A
Score: 21.2
Modifications:

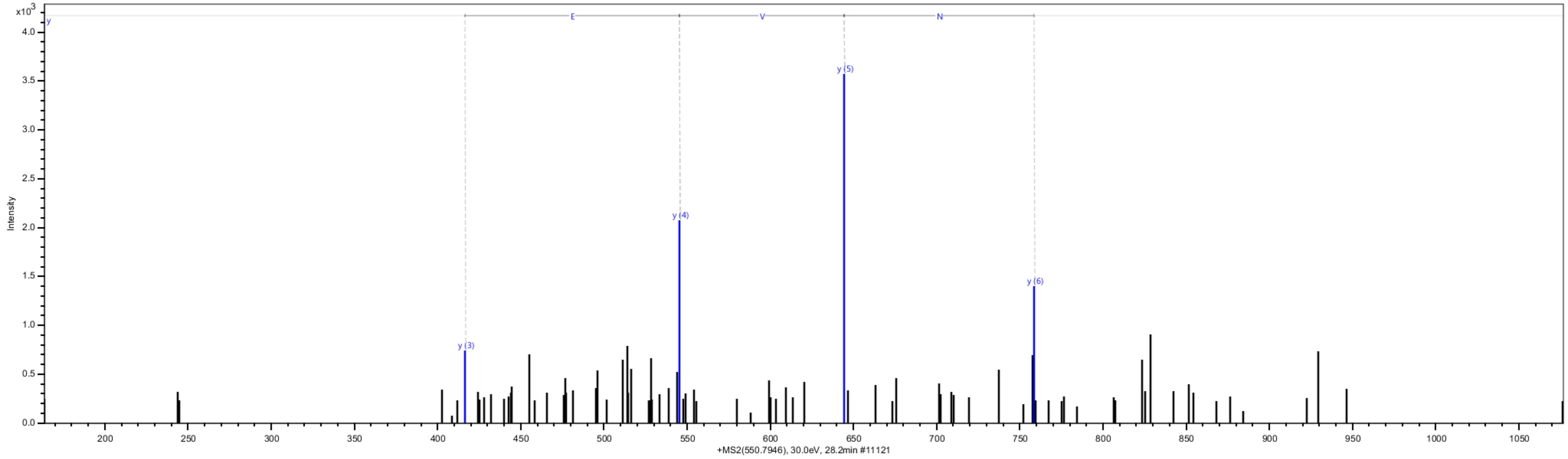

ANR29_HUMAN

Accession: ANR29_HUMAN
Database: SwissProt
MW [kDa] / pI: 32.4 / 9.5
Score: 22.1
Seq. Coverage [%]: 5.3
No. of Peptides: 1

Peptide Match

Sequence: K.NGTSALHAAVLSGNIK.T
Score: 22.1
Modifications:

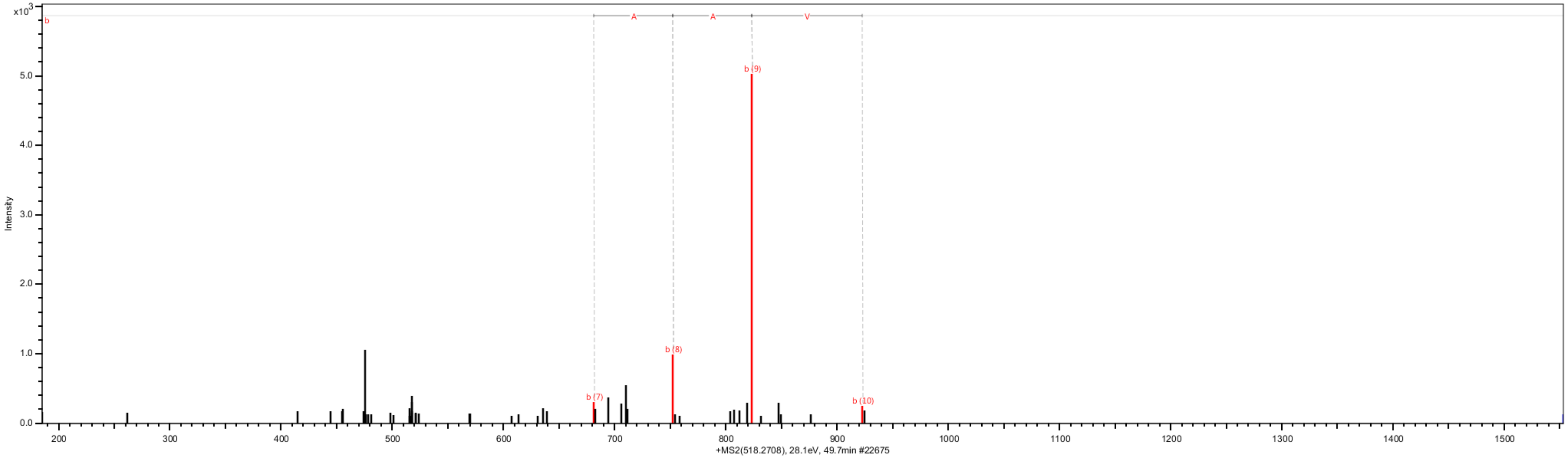

SYFB_HUMAN

**Accession:**	SYFB_HUMAN	**Score:**	22.1
**Database:**	SwissProt	**Seq. Coverage [%]:**	1.2
**MW [kDa] / pI:**	66.1 / 6.4	**No. of Peptides:**	1

Peptide Match

**Sequence:**	R.ADLINKK.V	**Score:**	22.1
**Modifications:**			

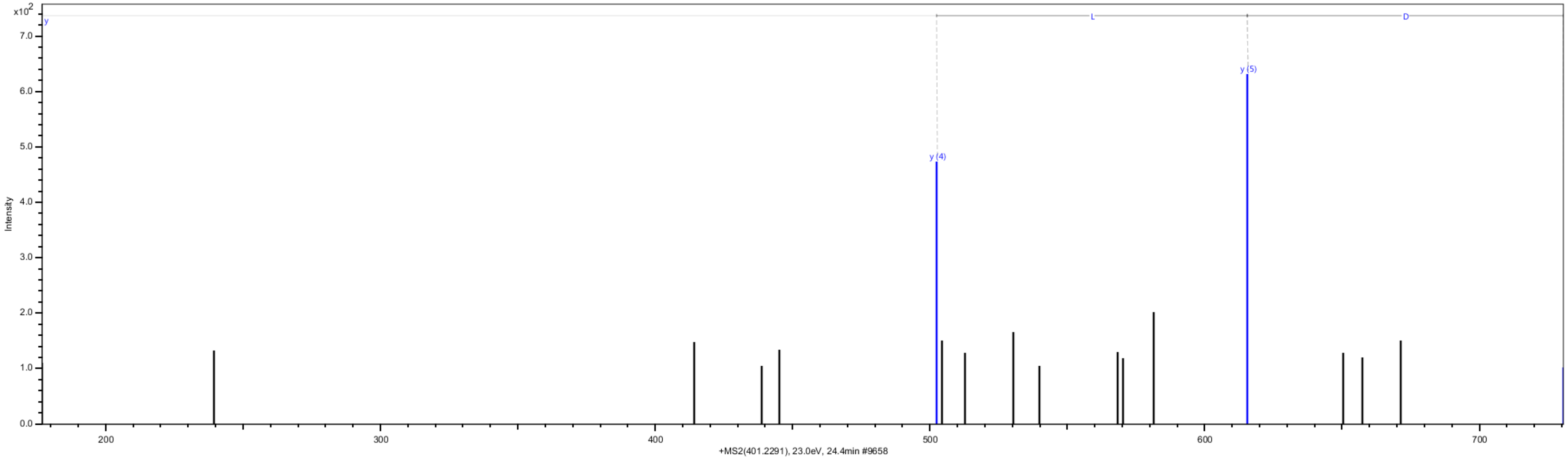

IPO11_HUMAN

**Accession:**	IPO11_HUMAN	**Score:**	21.9
**Database:**	SwissProt	**Seq. Coverage [%]:**	1.4
**MW [kDa] / pI:**	112.5 / 5.1	**No. of Peptides:**	1

Peptide Match

**Sequence:**	K.NMEVMGFLHGIFER.L	**Score:**	21.9
**Modifications:**			

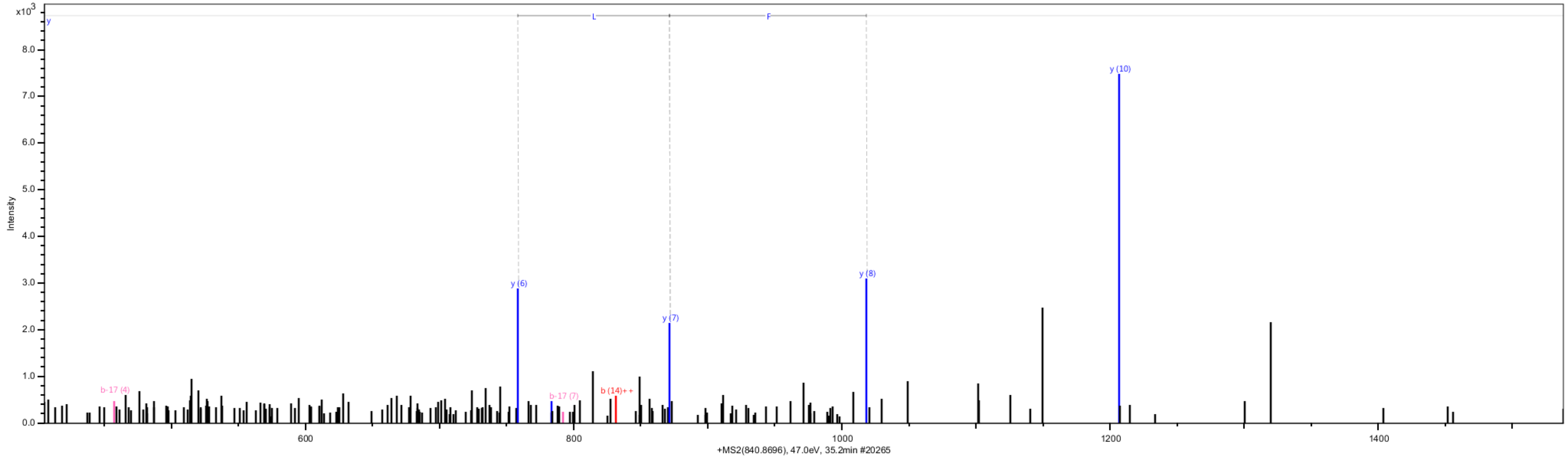

SIR1_HUMAN

**Accession:**	SIR1_HUMAN	**Score:**	22.0
**Database:**	SwissProt	**Seq. Coverage [%]:**	1.1
**MW [kDa] / pI:**	81.6 / 4.6	**No. of Peptides:**	1

Peptide Match

**Sequence:**	R.RDGPGLER.S	**Score:**	22
**Modifications:**			

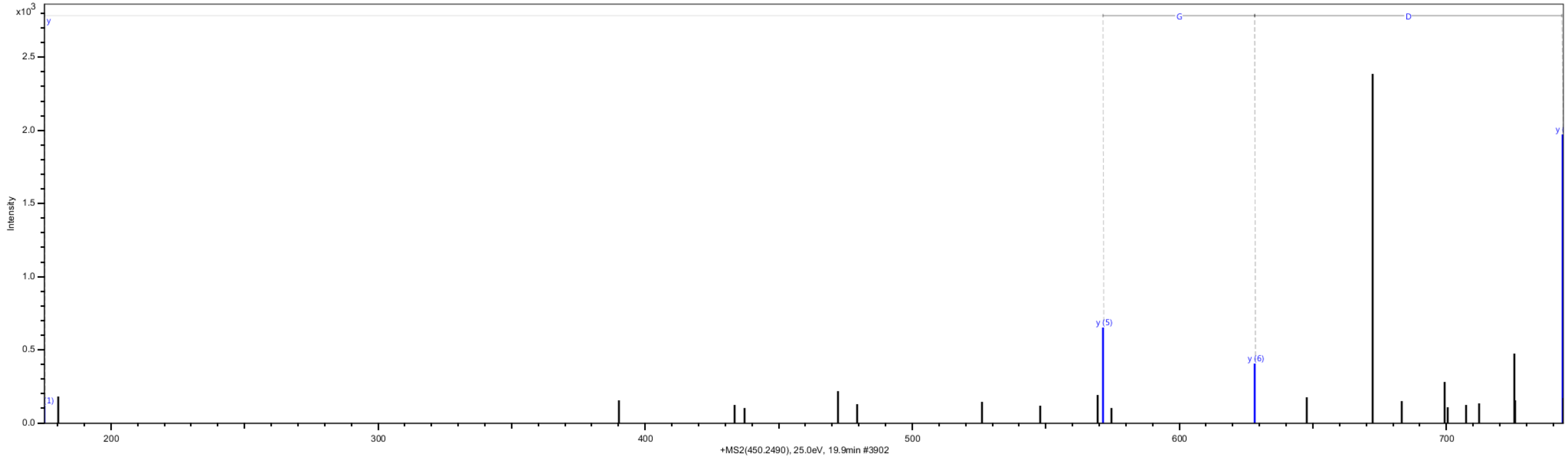

SAA4_HUMAN

**Accession:**	SAA4_HUMAN	**Score:**	22.4
**Database:**	SwissProt	**Seq. Coverage [%]:**	11.5
**MW [kDa] / pI:**	14.7 / 9.2	**No. of Peptides:**	1

Peptide Match

**Sequence:**	R.SFFKEALQGVGDMGR.A	**Score:**	22.4
**Modifications:**			

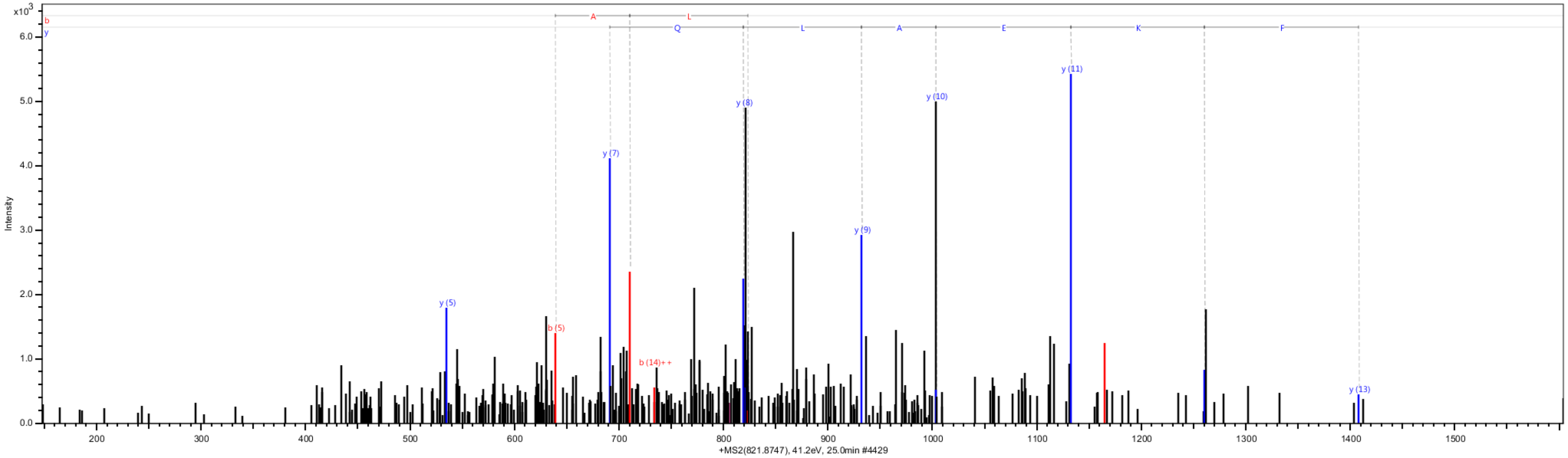

TRIPB_HUMAN

Accession: TRIPB_HUMAN
Database: SwissProt
MW [kDa] / pI: 227.4 / 5.2
Score: 22.2
Seq. Coverage [%]: 0.5
No. of Peptides: 1

Peptide Match

Sequence: R.SSKLQEELDK.Y
Score: 22.2
Modifications:

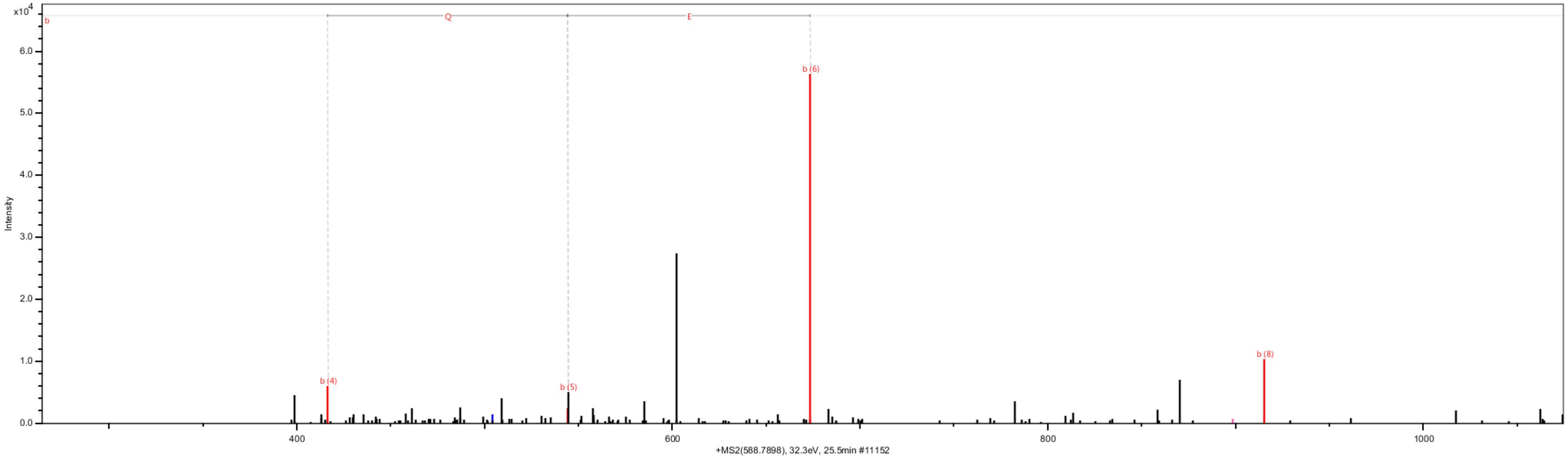

LMAN2_HUMAN

Accession: LMAN2_HUMAN
Database: SwissProt
MW [kDa] / pI: 40.2 / 6.5
Score: 22.4
Seq. Coverage [%]: 2.5
No. of Peptides: 1

Peptide Match

Sequence: K.IEPSVNFLK.S
Score: 22.4
Modifications:

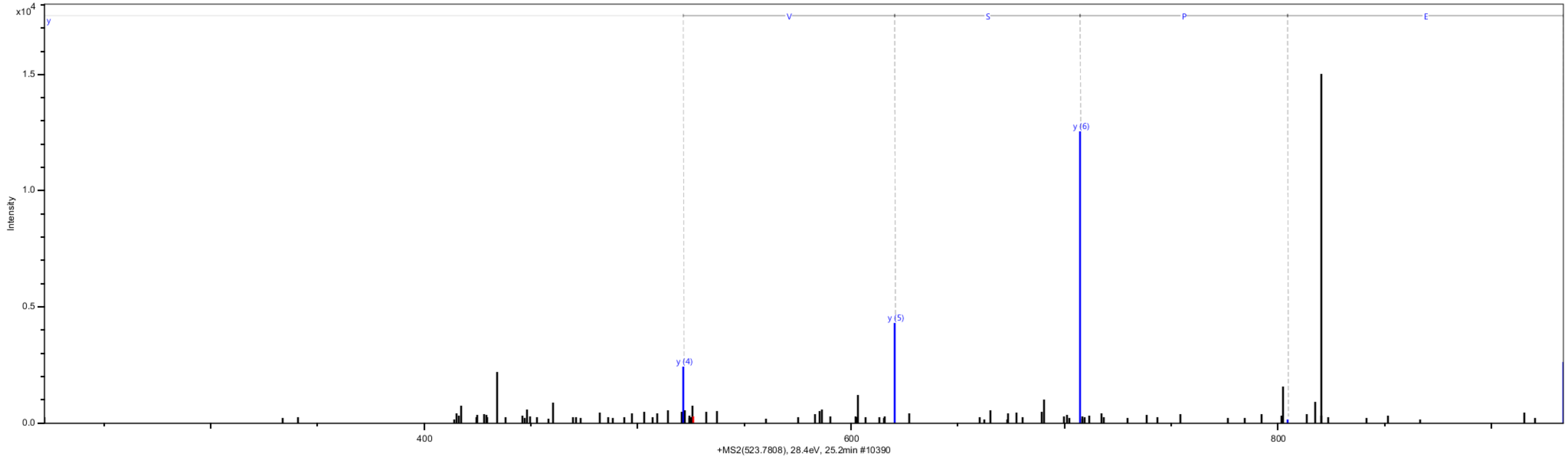

KI26A_HUMAN

**Accession:**	KI26A_HUMAN	**Score:**	21.7
**Database:**	SwissProt	**Seq. Coverage [%]:**	0.6
**MW [kDa] / pI:**	194.5 / 9.1	**No. of Peptides:**	1

Peptide Match

**Sequence:**	K.HLCEELAETQGR.L	**Score:**	21.7
**Modifications:**	Carbamidomethyl: 3		

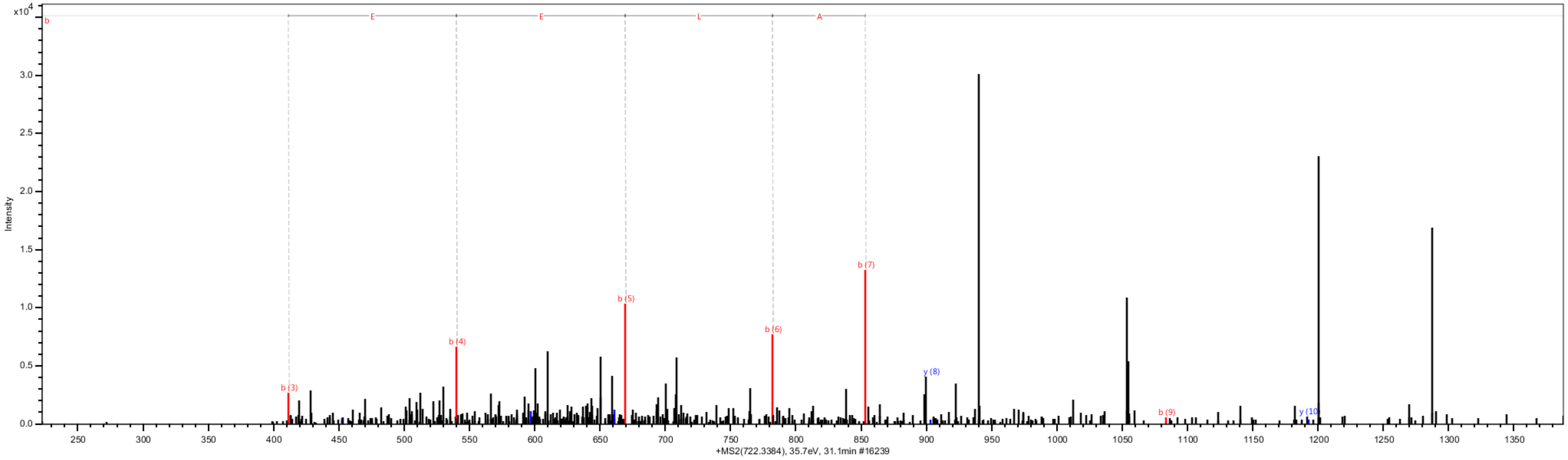

TTL11_HUMAN

**Accession:**	TTL11_HUMAN	**Score:**	21.8
**Database:**	SwissProt	**Seq. Coverage [%]:**	1.3
**MW [kDa] / pI:**	87.6 / 9.1	**No. of Peptides:**	1

Peptide Match

**Sequence:**	R.RWNSMTLDQR.D	**Score:**	21.8
**Modifications:**			

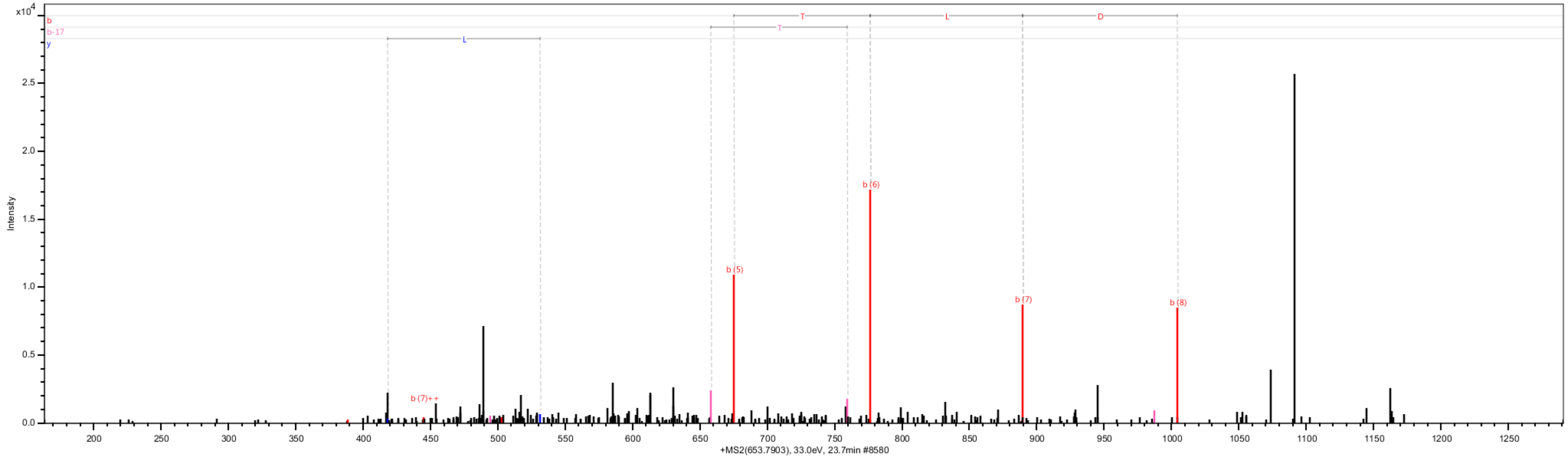

DSE_HUMAN

Accession: DSE_HUMAN
Database: SwissProt
MW [kDa] / pI: 109.7 / 8.2
Score: 21.7
Seq. Coverage [%]: 0.8
No. of Peptides: 1

Peptide Match

Sequence: K.QVLTIMEK.S
Score: 21.6
Modifications:

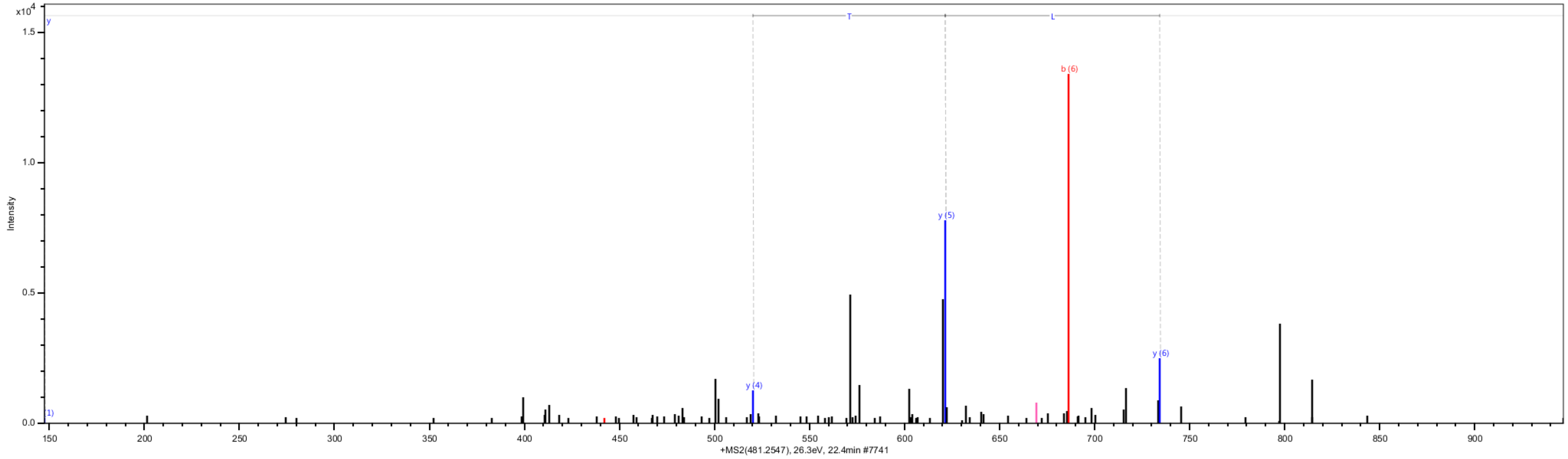

GP155_HUMAN

Accession: GP155_HUMAN
Database: SwissProt
MW [kDa] / pI: 96.9 / 6.4
Score: 21.7
Seq. Coverage [%]: 1.6
No. of Peptides: 1

Peptide Match

Sequence: R.LEFLWNNKDTAENR.D
Score: 21.6
Modifications:

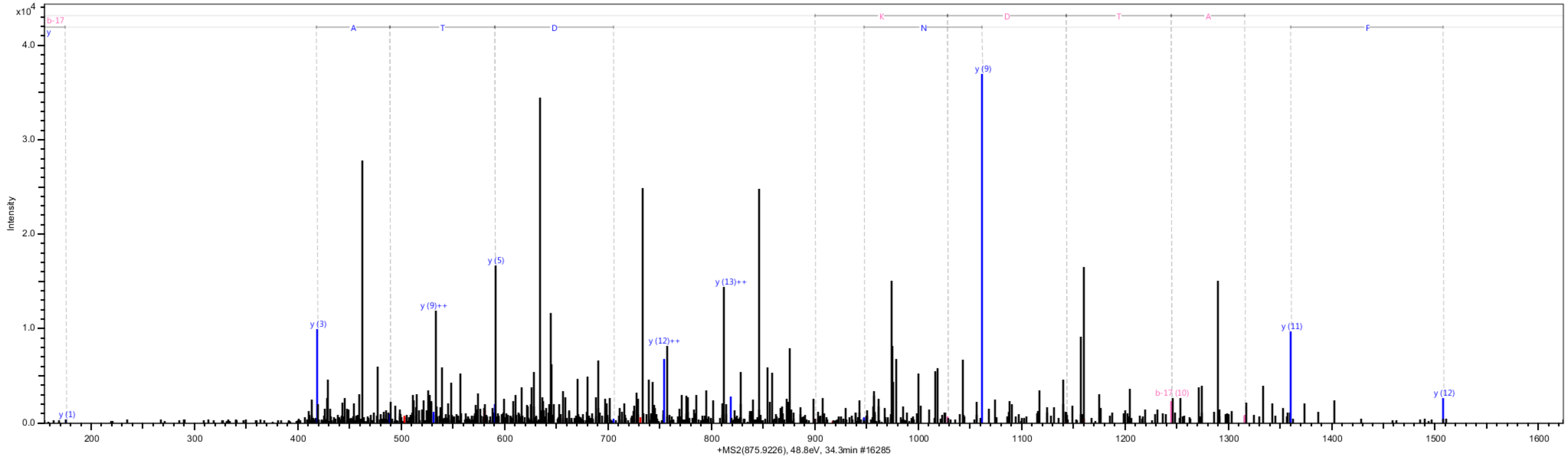

APOM_HUMAN

Accession: APOM_HUMAN
Database: SwissProt
MW [kDa] / pI: 21.2 / 5.7
Score: 21.9
Seq. Coverage [%]: 3.7
No. of Peptides: 1

Peptide Match

Sequence: K.AFLLTPR.N
Score: 21.9
Modifications:

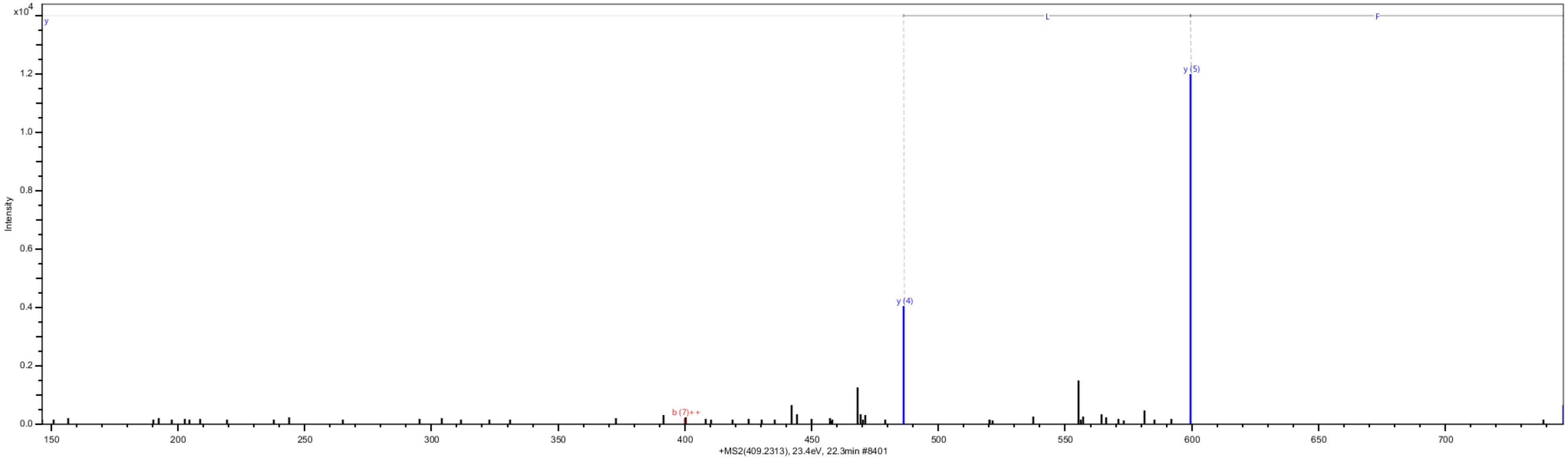

CE295_HUMAN

Accession: CE295_HUMAN
Database: SwissProt
MW [kDa] / pI: 295.0 / 5.7
Score: 21.9
Seq. Coverage [%]: 0.4
No. of Peptides: 1

Peptide Match

Sequence: R.REAQEVLYVHK.Q
Score: 21.9
Modifications:

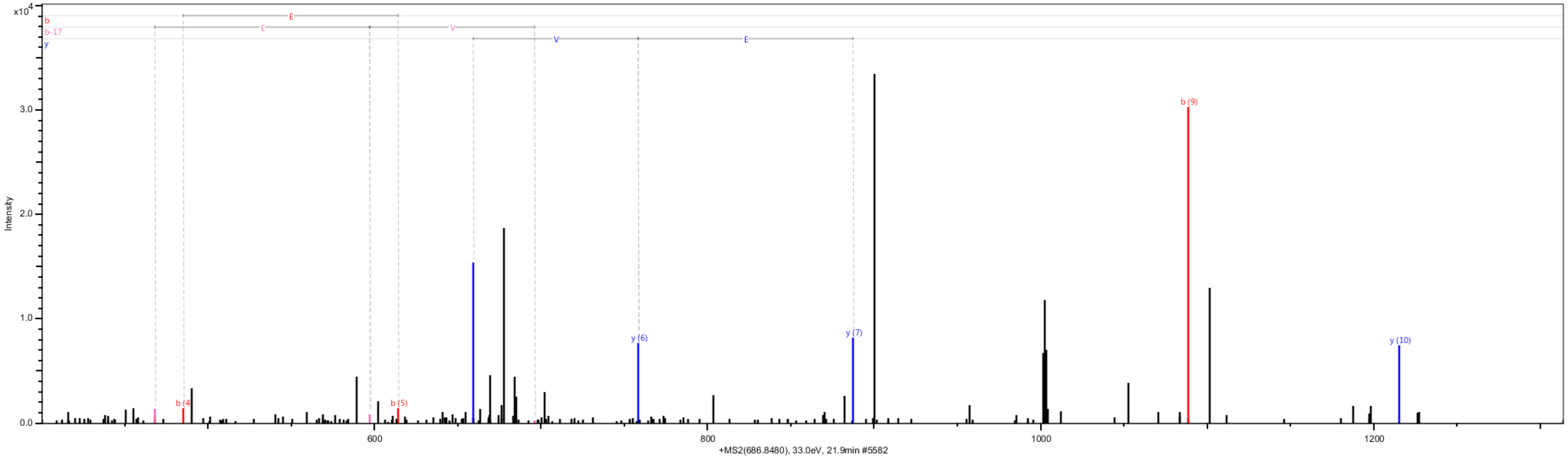

ENKUR_HUMAN

Accession: ENKUR_HUMAN
Database: SwissProt
MW [kDa] / pI: 29.4 / 9.3

Score: 21.8
Seq. Coverage [%]: 5.1
No. of Peptides: 1

Peptide Match

Sequence: K.TMGPAKVEVPSPK.D
Modifications: Oxidation: 2
Score: 21.8

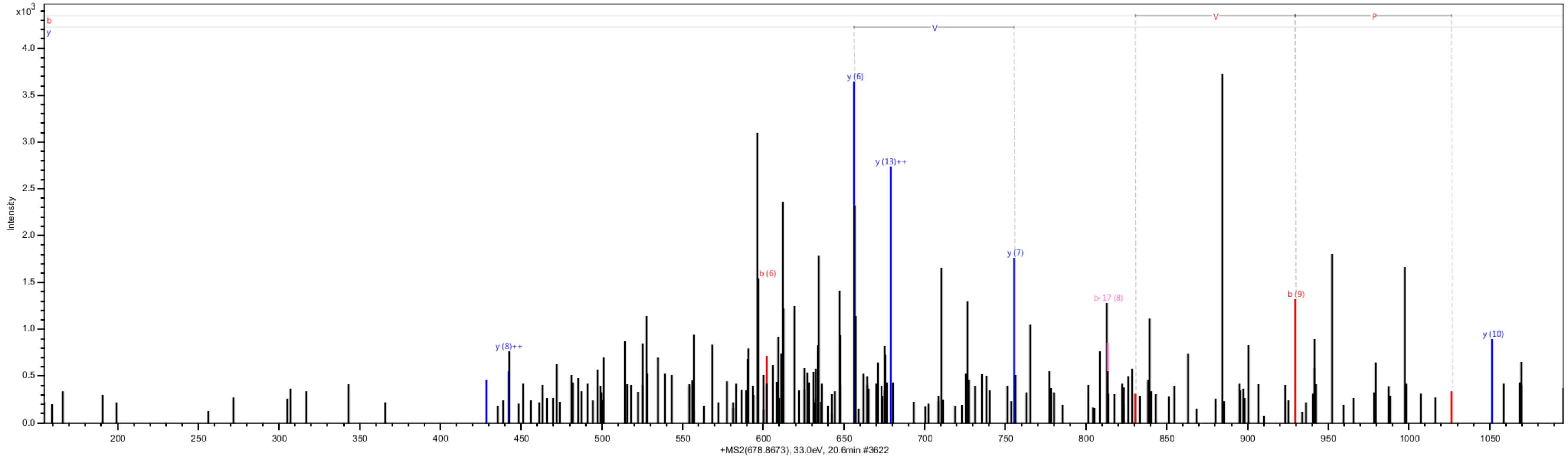

CAPR2_HUMAN

Accession: CAPR2_HUMAN
Database: SwissProt
MW [kDa] / pI: 125.8 / 6.3
Score: 21.9
Seq. Coverage [%]: 0.6
No. of Peptides: 1

Peptide Match

Sequence: K.LEDYKDR.L
Score: 21.9
Modifications:

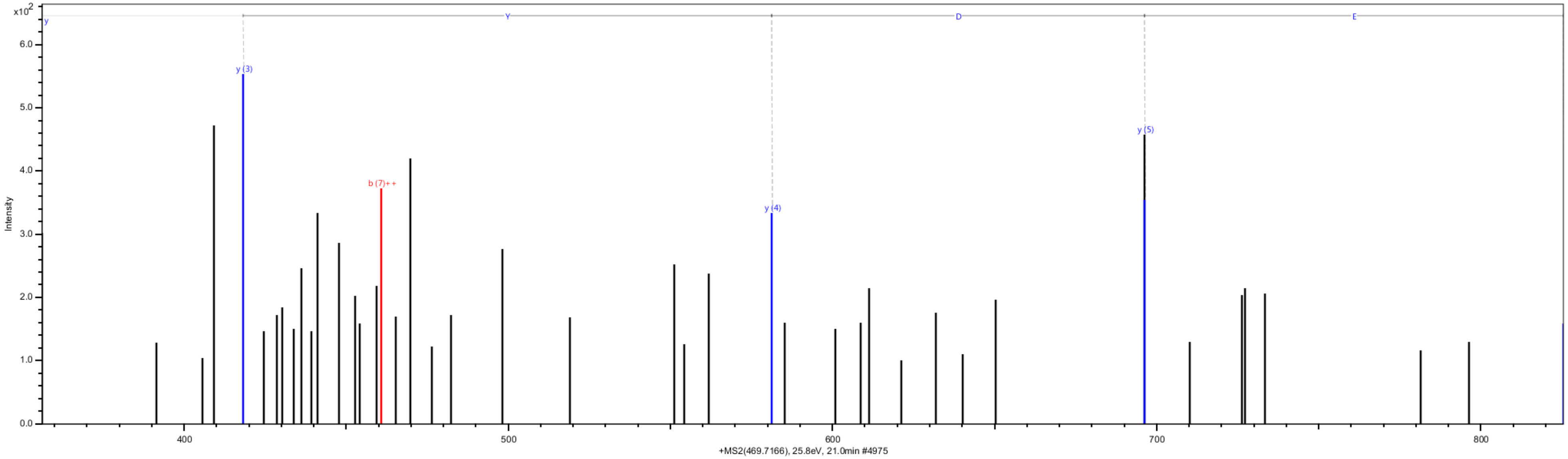

CNTN2_HUMAN

**Accession:**	CNTN2_HUMAN	**Score:**	22.9
**Database:**	SwissProt	**Seq. Coverage [%]:**	1.0
**MW [kDa] / pI:**	113.3 / 8.1	**No. of Peptides:**	1

Peptide Match

**Sequence:**	K.GTEILVNSSR.V	**Score:**	22.9
**Modifications:**			

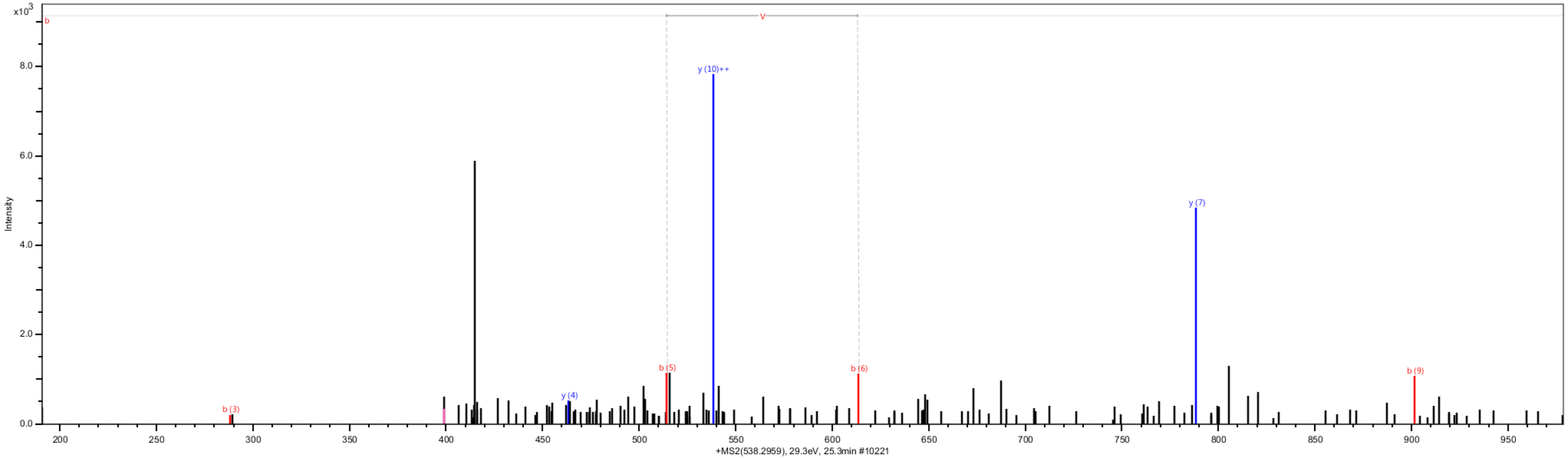

ABRX2_HUMAN

Accession: ABRX2_HUMAN
Database: SwissProt
MW [kDa] / pI: 46.9 / 5.8
Score: 22.9
Seq. Coverage [%]: 2.2
No. of Peptides: 1

Peptide Match

Sequence: R.RLQQAVLSR.Q
Score: 22.9
Modifications:

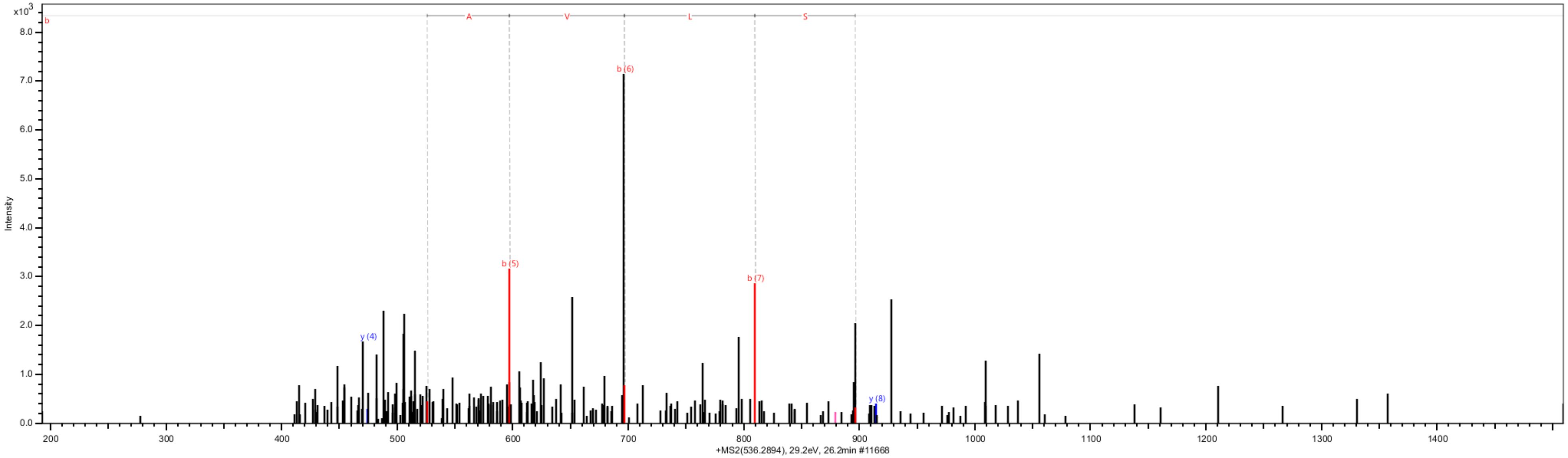

GALT_HUMAN

Accession: GALT_HUMAN
Database: SwissProt
MW [kDa] / pI: 43.3 / 6.5
Score: 23.1
Seq. Coverage [%]: 2.6
No. of Peptides: 1

Peptide Match

Sequence: R.DLTPEQAAER.L
Score: 23
Modifications:

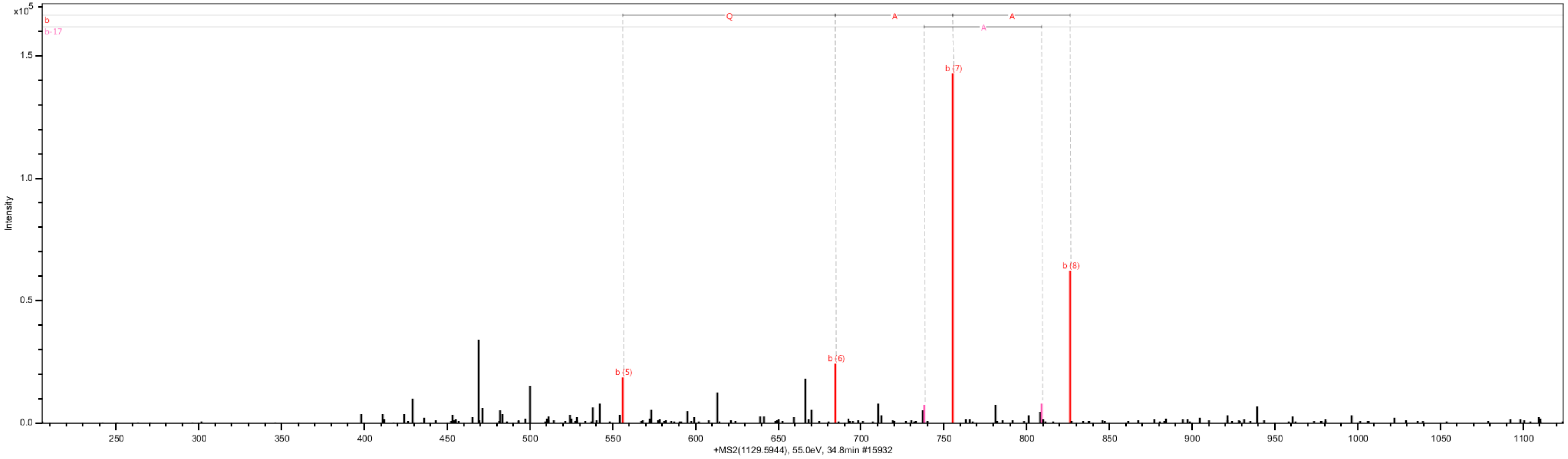

ZN696_HUMAN

**Accession:**	ZN696_HUMAN	**Score:**	22.9
**Database:**	SwissProt	**Seq. Coverage [%]:**	2.7
**MW [kDa] / pI:**	40.5 / 9.3	**No. of Peptides:**	1

Peptide Match

**Sequence:**	M.EPGGEPTGAK.E	**Score:**	22.9
**Modifications:**			

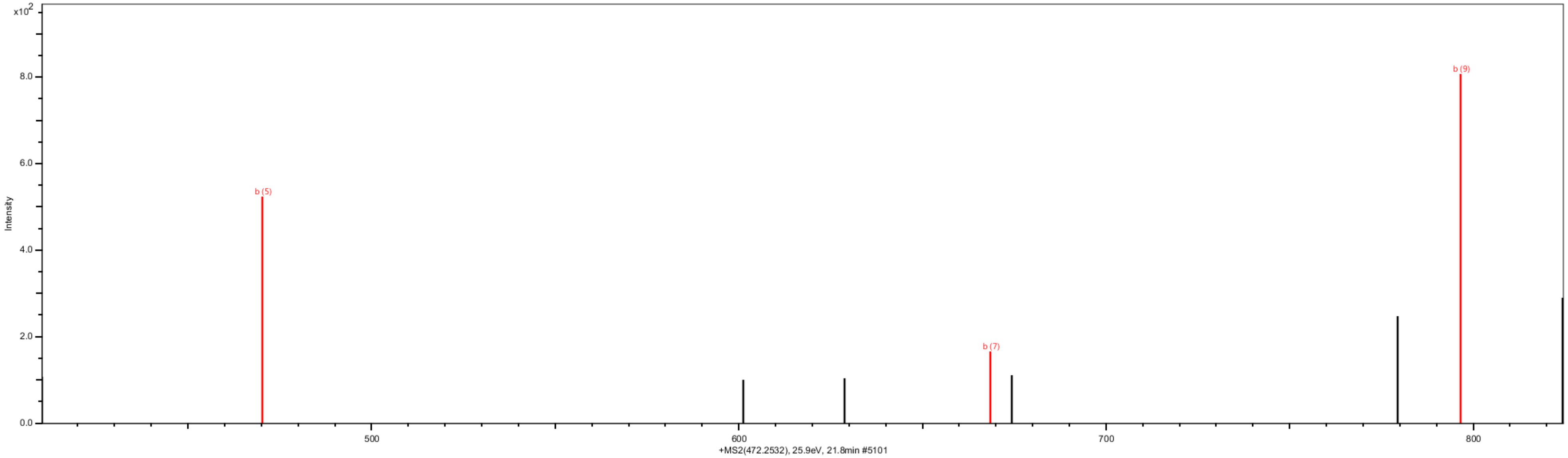

AOFA_HUMAN

**Accession:**	AOFA_HUMAN	**Score:**	23.1
**Database:**	SwissProt	**Seq. Coverage [%]:**	1.9
**MW [kDa] / pI:**	59.6 / 7.9	**No. of Peptides:**	1

Peptide Match

**Sequence:**	R.NLPSVSGLLK.I	**Score:**	23.1
**Modifications:**			

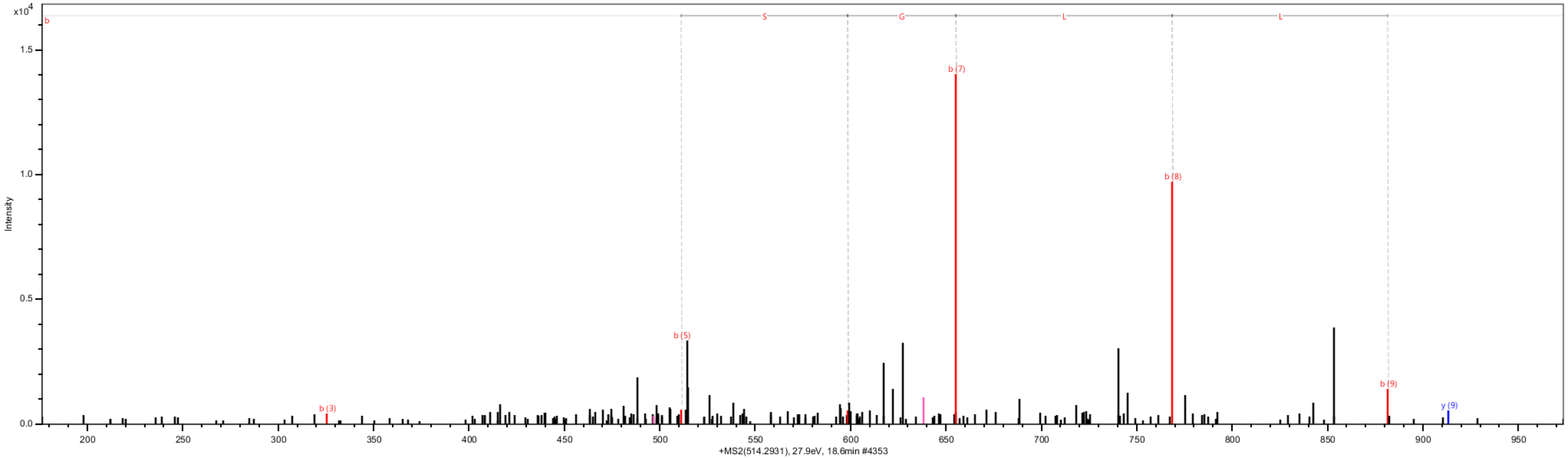

GCYA1_HUMAN

Accession: GCYA1_HUMAN
Database: SwissProt
MW [kDa] / pI: 77.4 / 6.7
Score: 23.1
Seq. Coverage [%]: 1.3
No. of Peptides: 1

Peptide Match

Sequence: K.ESLGEEVFK.I
Score: 23.1
Modifications:

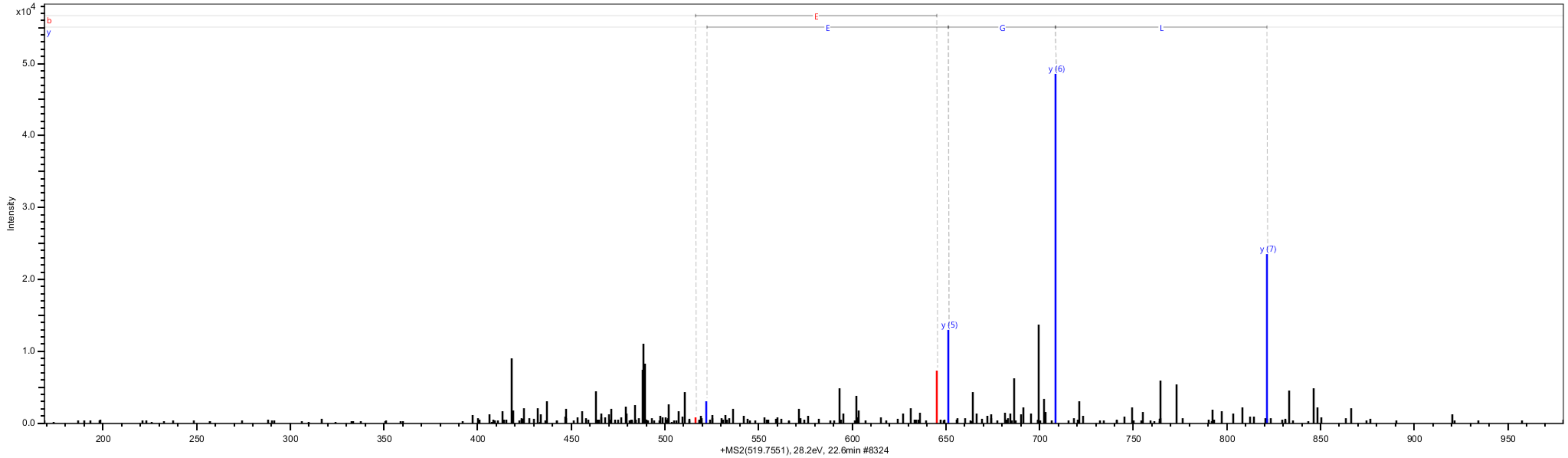

NDUA9_HUMAN

**Accession:**	NDUA9_HUMAN	**Score:**	23.2
**Database:**	SwissProt	**Seq. Coverage [%]:**	2.9
**MW [kDa] / pI:**	42.5 / 9.8	**No. of Peptides:**	1

Peptide Match

**Sequence:**	-.MAAAAQSRVVR.V	**Score:**	23.2
**Modifications:**			

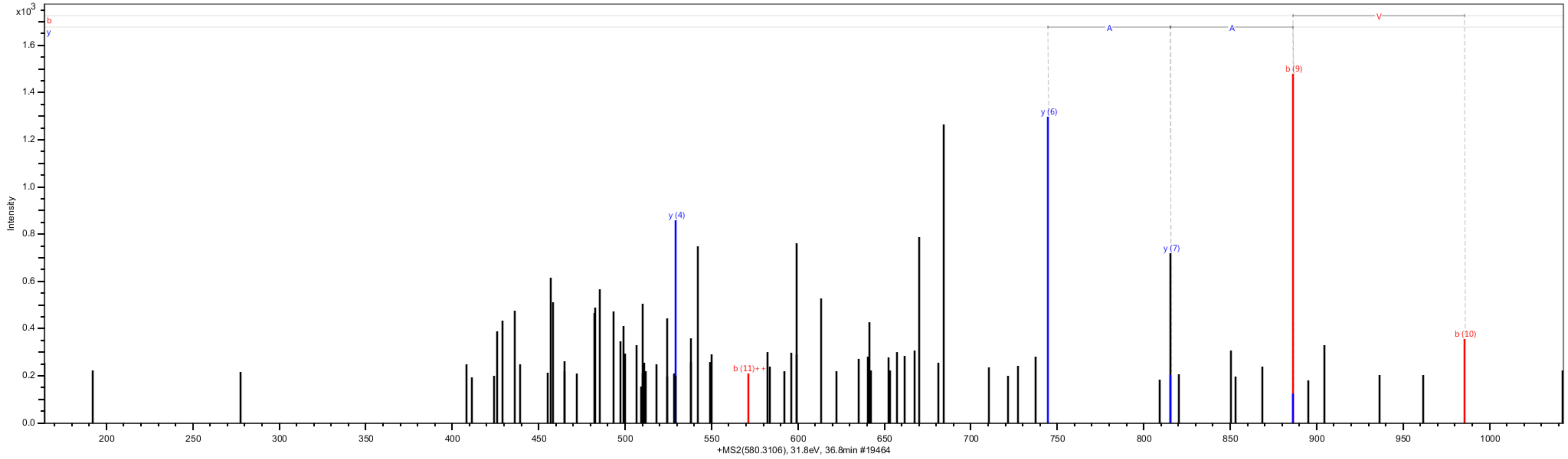

RGAP1_HUMAN

Accession: RGAP1_HUMAN
Database: SwissProt
MW [kDa] / pI: 71.0 / 9.1
Score: 23.1
Seq. Coverage [%]: 1.4
No. of Peptides: 1

Peptide Match

Sequence: R.NQVDVEIKR.R
Score: 23.1
Modifications:

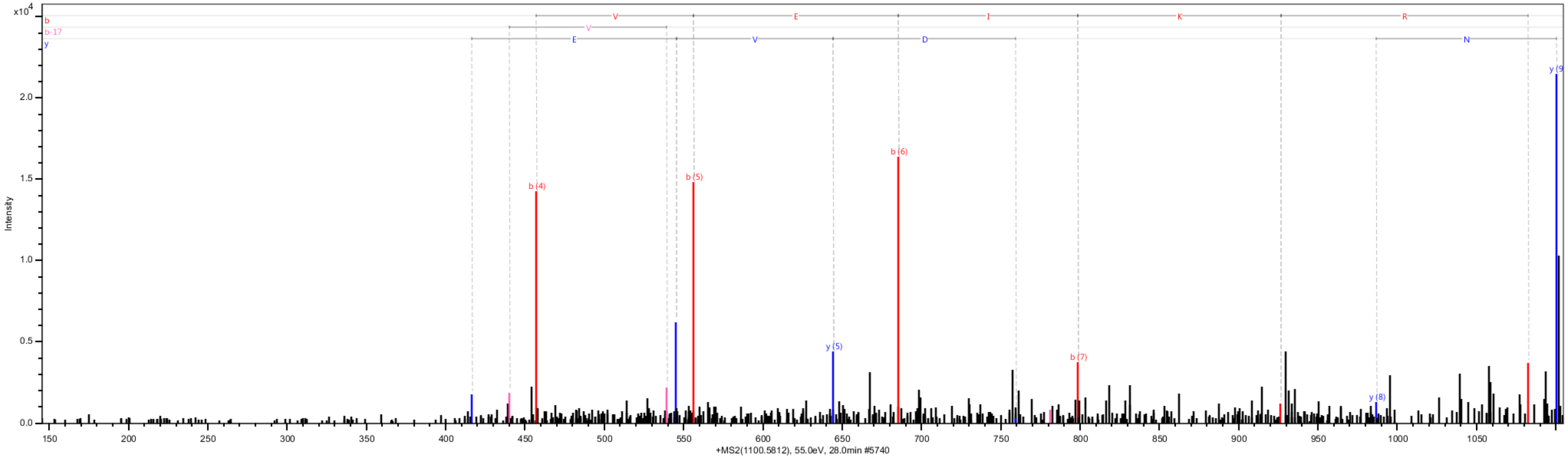

PLXB3_HUMAN

**Accession:**	PLXB3_HUMAN	**Score:**	22.4
**Database:**	SwissProt	**Seq. Coverage [%]:**	0.5
**MW [kDa] / pI:**	206.7 / 6.0	**No. of Peptides:**	1

Peptide Match

**Sequence:**	K.LEYLTDIMR.T	**Score:**	22.4
**Modifications:**	Oxidation: 8		

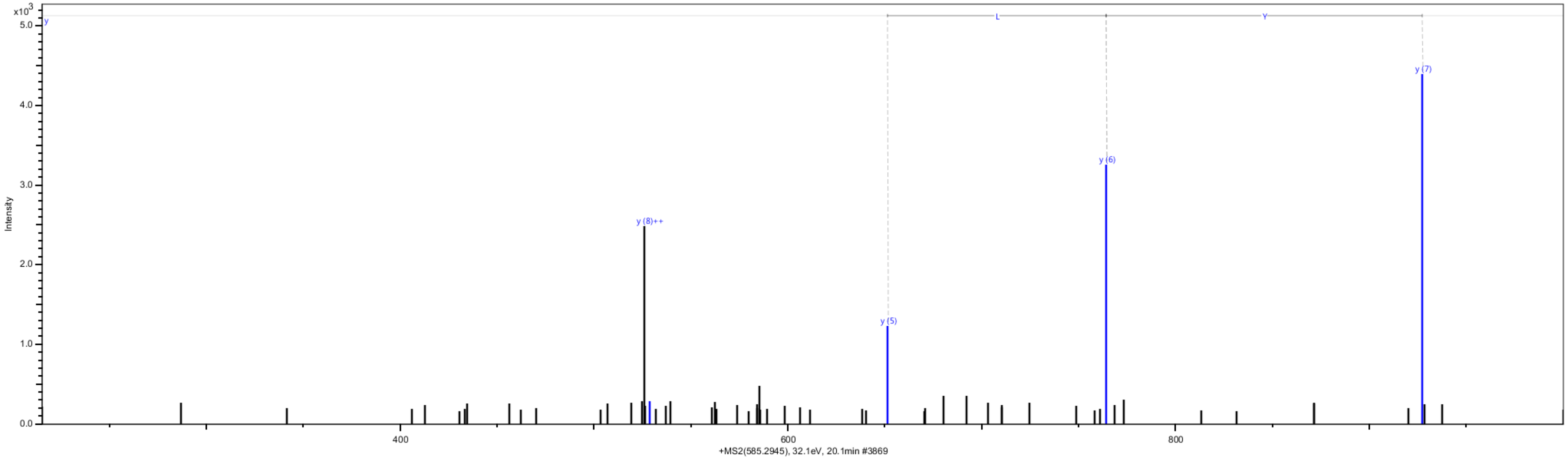

HV118_HUMAN

Accession: HV118_HUMAN
Database: SwissProt
MW [kDa] / pI: 12.8 / 9.0
Score: 22.4
Seq. Coverage [%]: 9.4
No. of Peptides: 1

Peptide Match

Sequence: R.SDDTAVYYCAR.-
Modifications: Carbamidomethyl: 9
Score: 22.4

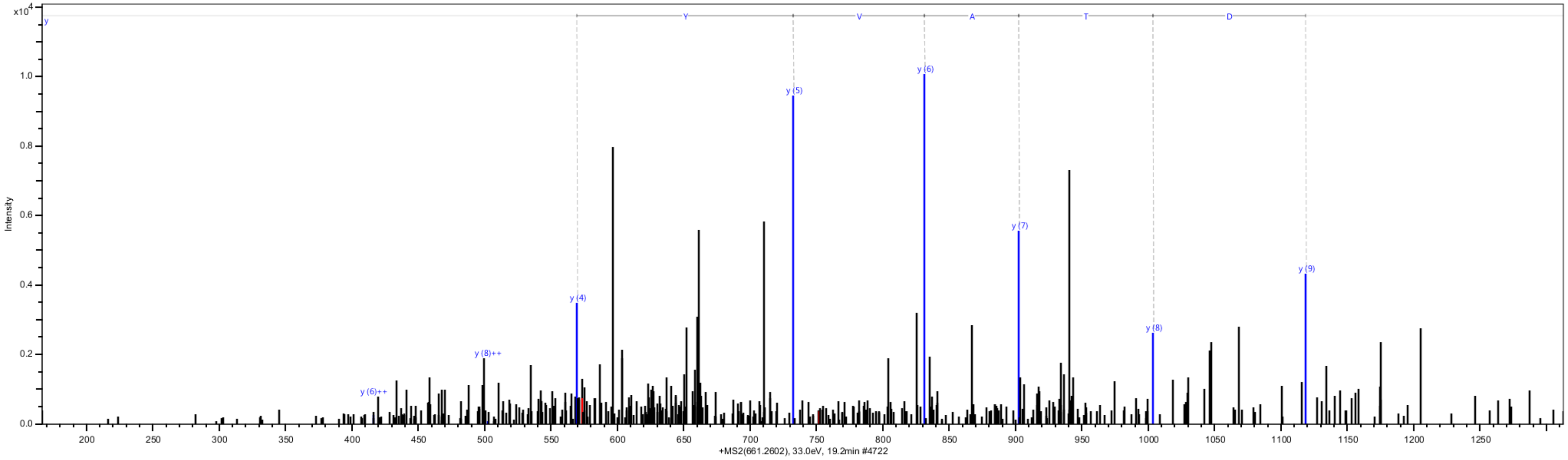

PITM2_HUMAN

**Accession:**	PITM2_HUMAN	**Score:**	22.6
**Database:**	SwissProt	**Seq. Coverage [%]:**	0.7
**MW [kDa] / pI:**	148.8 / 6.7	**No. of Peptides:**	1

Peptide Match

**Sequence:**	K.DPVPHNEYK.T	**Score:**	22.6
**Modifications:**			

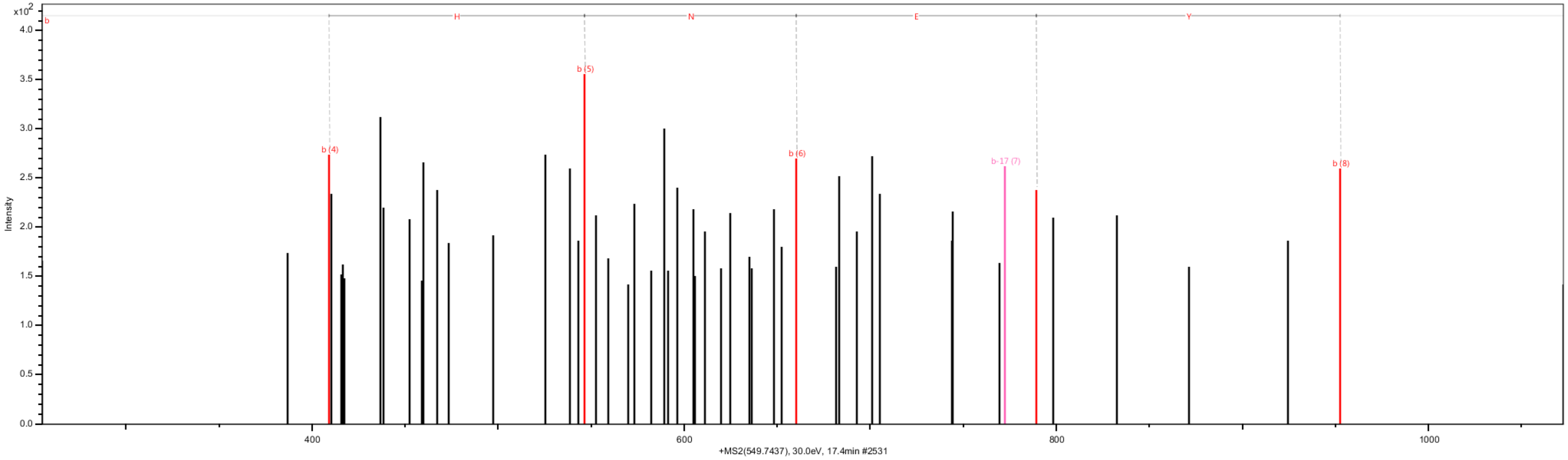

ATRX_HUMAN

**Accession:**	ATRX_HUMAN	**Score:**	22.6
**Database:**	SwissProt	**Seq. Coverage [%]:**	0.4
**MW [kDa] / pI:**	282.4 / 6.2	**No. of Peptides:**	1

Peptide Match

**Sequence:**	K.MVLLFEILR.M	**Score:**	22.6
**Modifications:**			

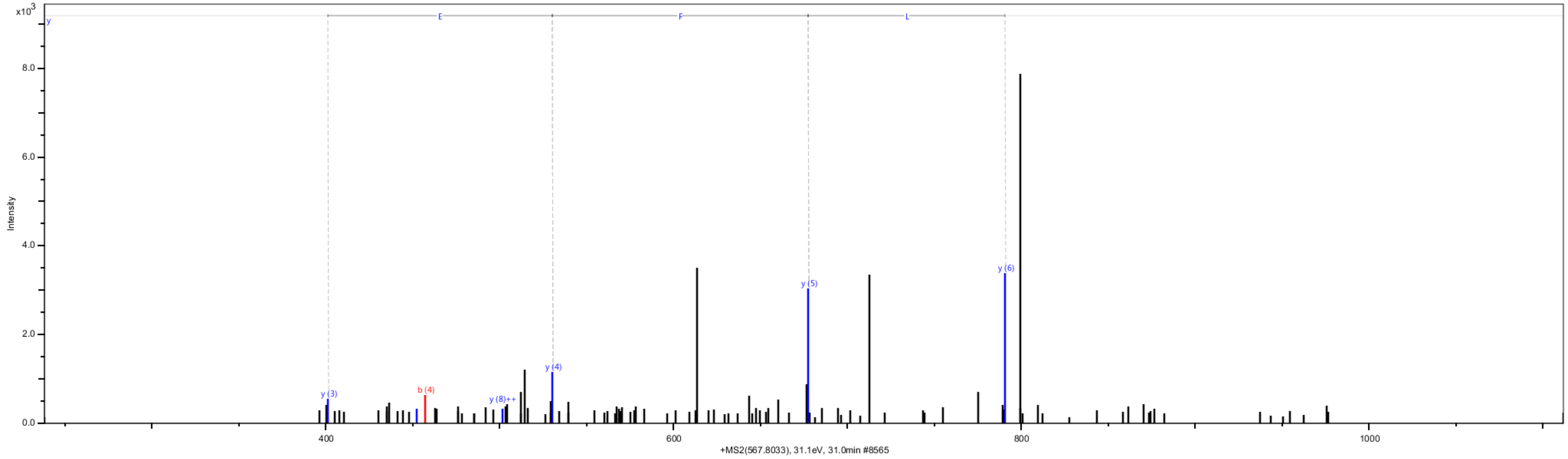

KLK11_HUMAN

Accession: KLK11_HUMAN
Database: SwissProt
MW [kDa] / pI: 31.0 / 9.2
Score: 22.8
Seq. Coverage [%]: 6.7
No. of Peptides: 1

Peptide Match

Sequence: K.MASPVSITWAVRPLTLSSR.C
Score: 22.8
Modifications: Oxidation: 1

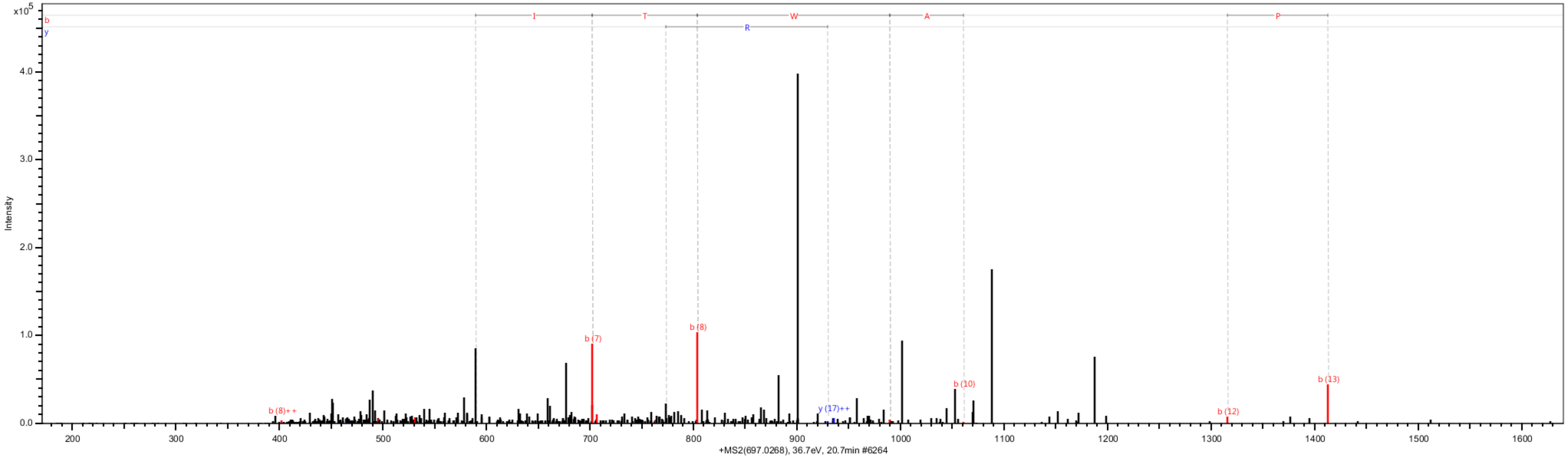

ZC12C_HUMAN

**Accession:**	ZC12C_HUMAN	**Score:**	22.8
**Database:**	SwissProt	**Seq. Coverage [%]:**	1.0
**MW [kDa] / pI:**	99.3 / 6.5	**No. of Peptides:**	1

Peptide Match

**Sequence:**	R.AMSRNTAAK.T	**Score:**	22.8
**Modifications:**	Oxidation: 2		

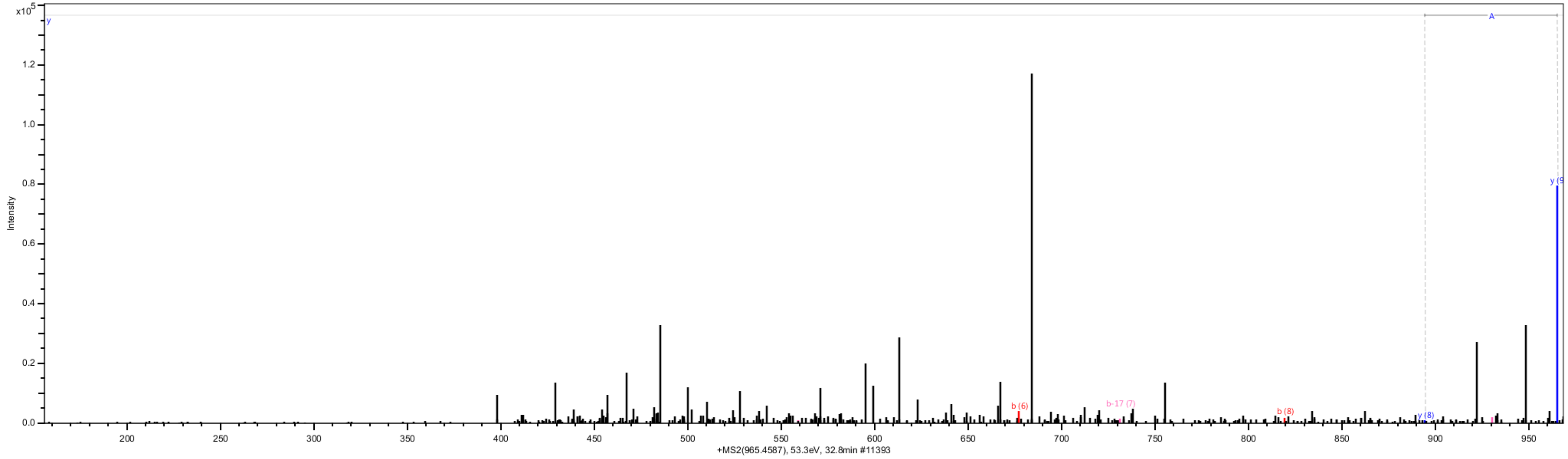

NKRF_HUMAN

Accession: NKRF_HUMAN
Database: SwissProt
MW [kDa] / pI: 77.6 / 8.9
Score: 22.8
Seq. Coverage [%]: 2.0
No. of Peptides: 1

Peptide Match

Sequence: R.MTVEYVYERMTGLR.W
Score: 22.8
Modifications: Oxidation: 1, 10

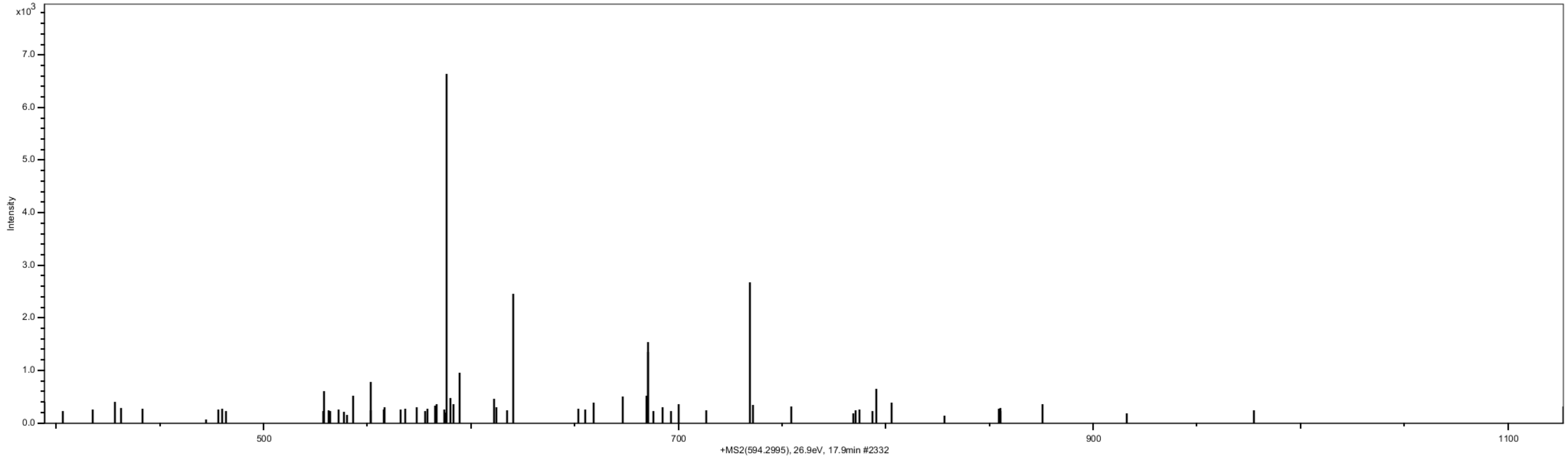

EHBP1_HUMAN

Accession: EHBP1_HUMAN
Database: SwissProt
MW [kDa] / pI: 139.9 / 5.2
Score: 22.8
Seq. Coverage [%]: 0.6
No. of Peptides: 1

Peptide Match

Sequence: R.RYELLNR.E
Score: 22.8
Modifications:

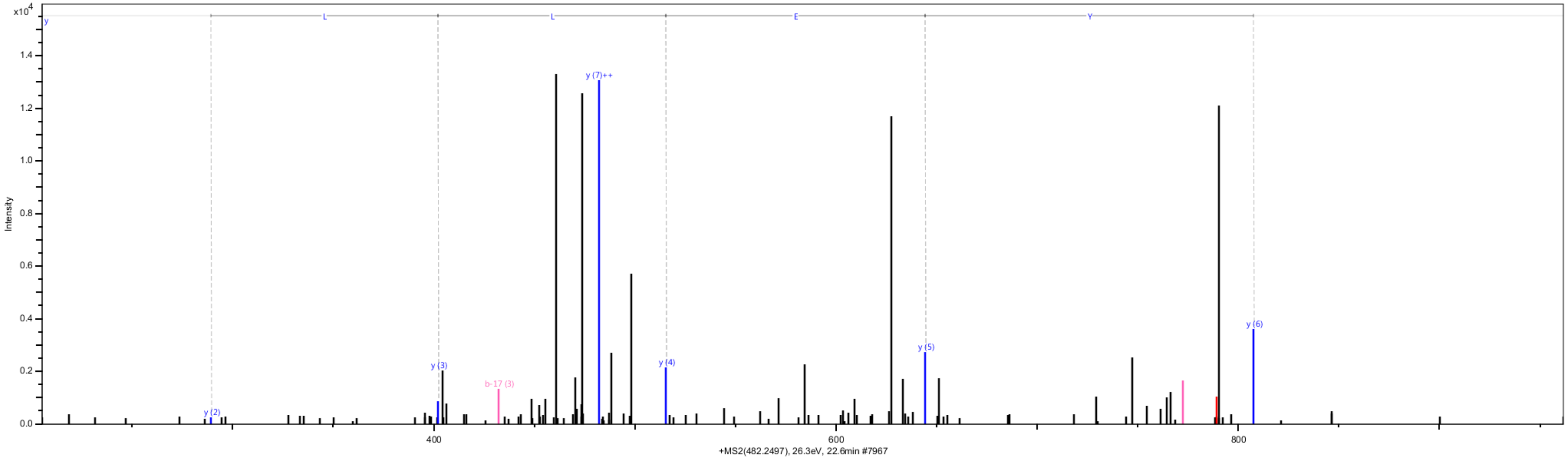

AT10D_HUMAN

Accession: AT10D_HUMAN
Database: SwissProt
MW [kDa] / pI: 160.2 / 6.8
Score: 23.9
Seq. Coverage [%]: 0.8
No. of Peptides: 1

Peptide Match

Sequence: R.RSSSPSLNSGK.E
Score: 23.8
Modifications:

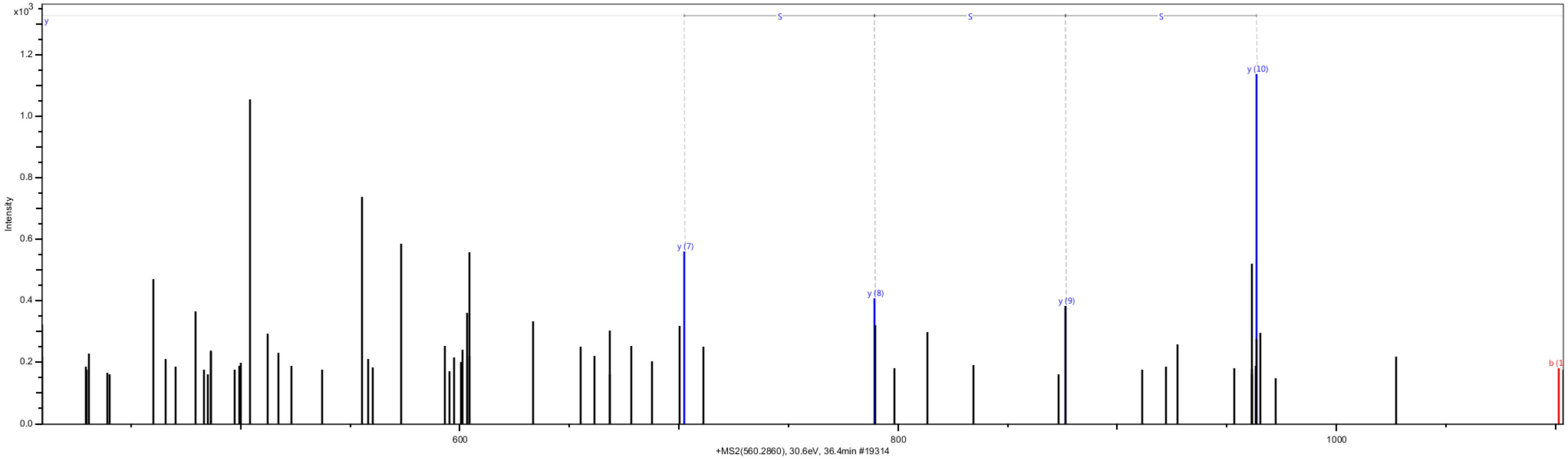

TAPT1_HUMAN

Accession: TAPT1_HUMAN
Database: SwissProt
MW [kDa] / pI: 64.2 / 8.6
Score: 23.9
Seq. Coverage [%]: 1.2
No. of Peptides: 1

Peptide Match

Sequence: K.EAKMEEK.L
Score: 23.9
Modifications: Oxidation: 4

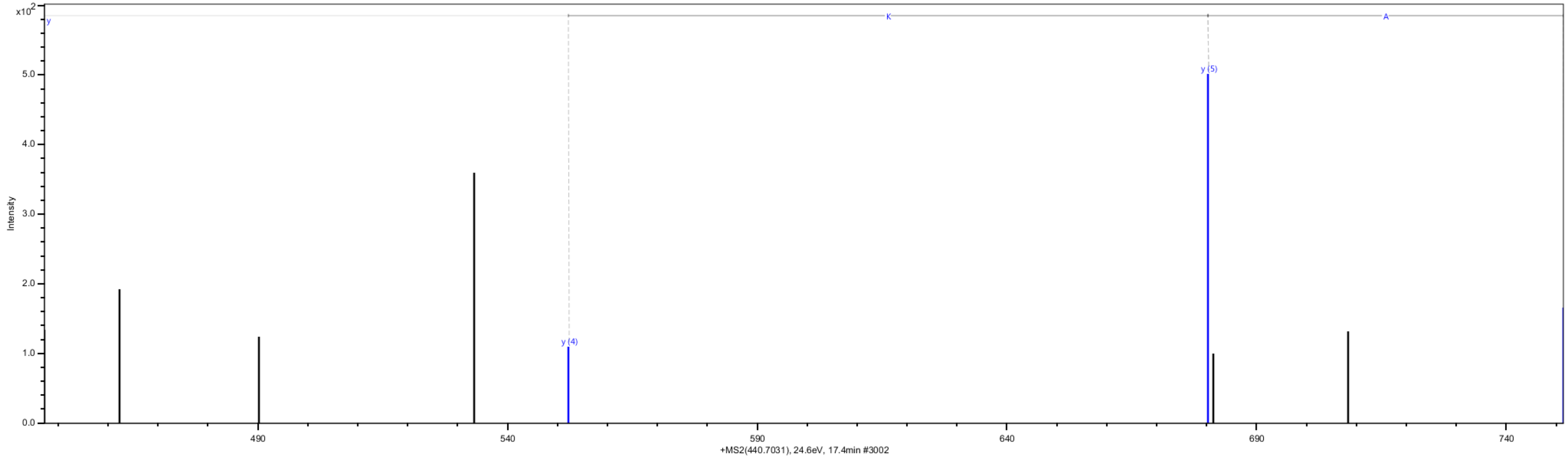

GEMI5_HUMAN

Accession: GEMI5_HUMAN
Database: SwissProt
MW [kDa] / pI: 168.5 / 6.2
Score: 24.1
Seq. Coverage [%]: 0.7
No. of Peptides: 1

Peptide Match

Sequence: K.QLLLSTSMDR.D
Score: 24
Modifications:

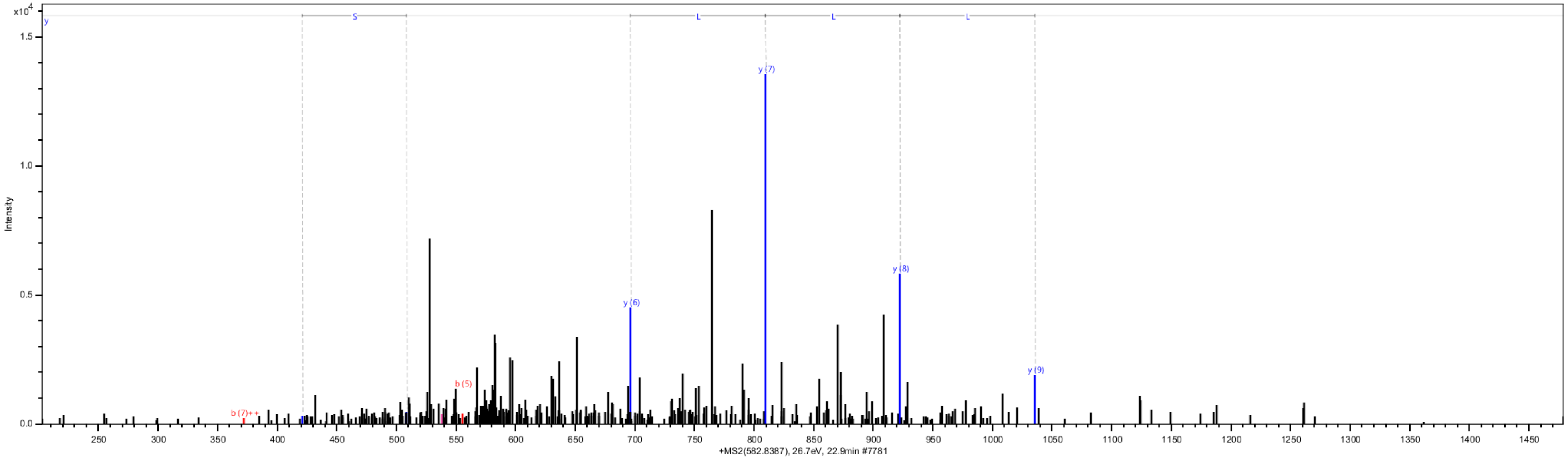

RGS13_HUMAN

**Accession:**	RGS13_HUMAN	**Score:**	24.1
**Database:**	SwissProt	**Seq. Coverage [%]:**	4.4
**MW [kDa] / pI:**	19.1 / 9.1	**No. of Peptides:**	1

Peptide Match

**Sequence:**	K.IASRWSR.I	**Score:**	24.1
**Modifications:**			

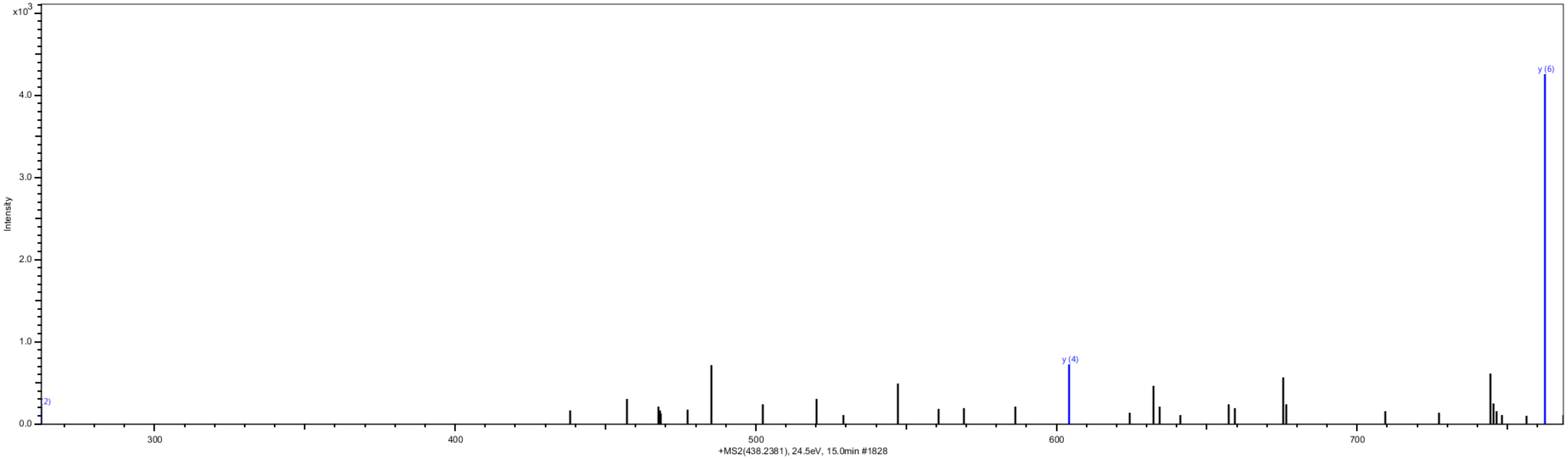

GTD2A_HUMAN

Accession: GTD2A_HUMAN
Database: SwissProt
MW [kDa] / pI: 107.1 / 5.5

Score: 24.3
Seq. Coverage [%]: 1.1
No. of Peptides: 1

Peptide Match

Sequence: K.IAELQTEFQK.R
Score: 24.3
Modifications:

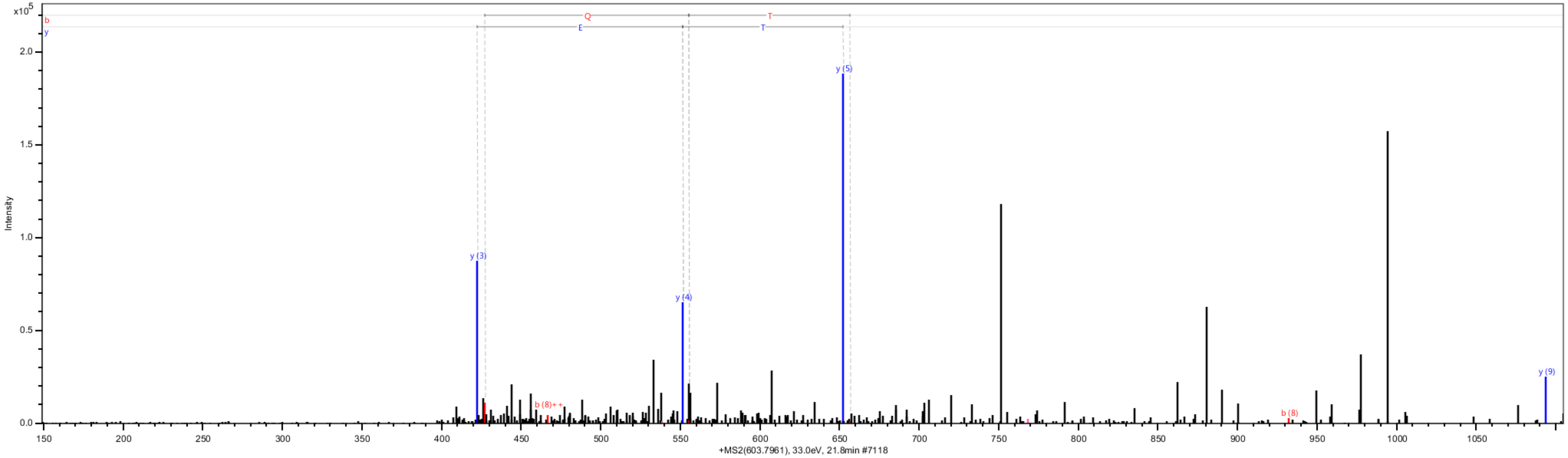

KDM1A_HUMAN

**Accession:**	KDM1A_HUMAN	**Score:**	24.3
**Database:**	SwissProt	**Seq. Coverage [%]:**	1.3
**MW [kDa] / pI:**	92.8 / 6.1	**No. of Peptides:**	1

Peptide Match

**Sequence:**	K.EYDELAETQGK.L	**Score:**	24.3
**Modifications:**			

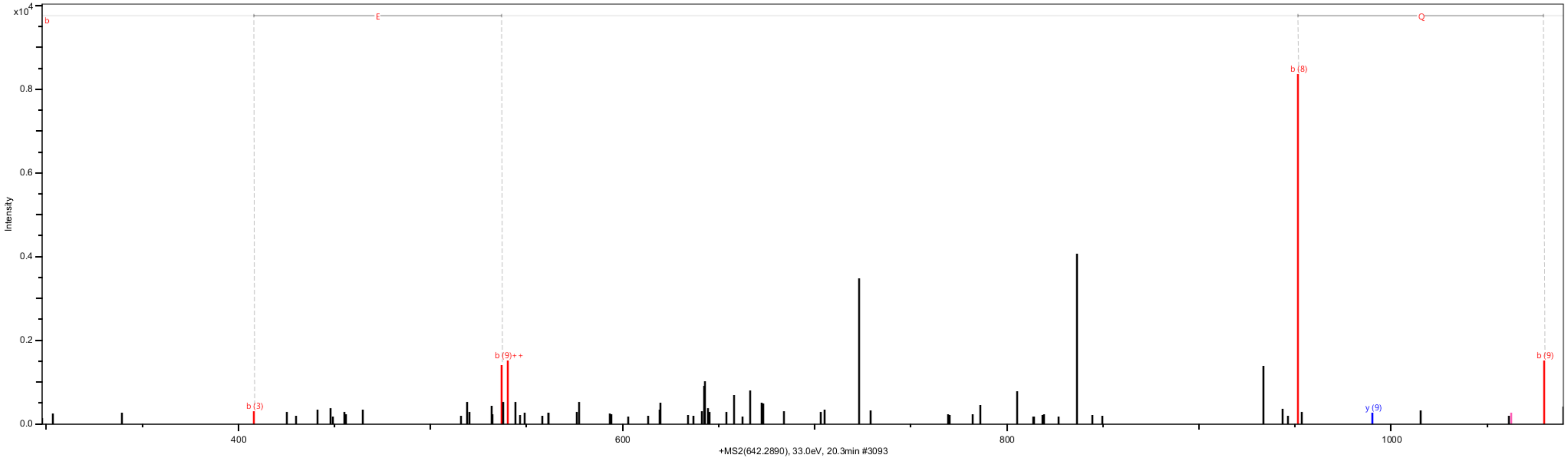

RNF17_HUMAN

Accession: RNF17_HUMAN
Database: SwissProt
MW [kDa] / pI: 184.5 / 5.3
Score: 23.3
Seq. Coverage [%]: 0.6
No. of Peptides: 1

Peptide Match

Sequence: K.FEDGIWYRAK.V
Score: 23.3
Modifications:

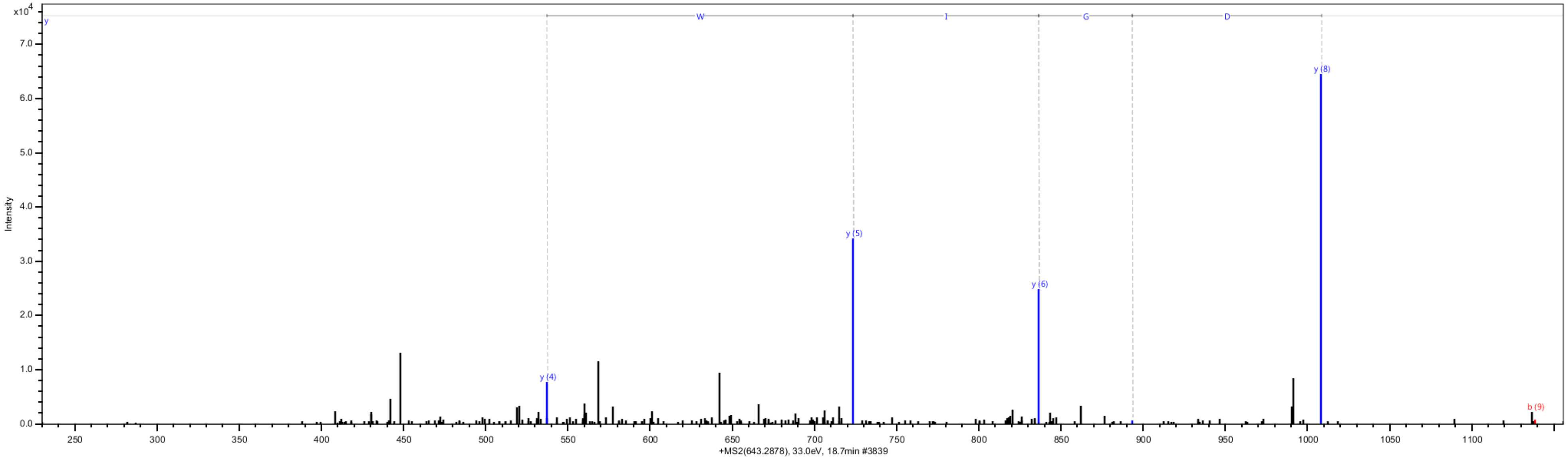

RAB21_HUMAN

Accession: RAB21_HUMAN
Database: SwissProt
MW [kDa] / pI: 24.3 / 8.1
Score: 23.3
Seq. Coverage [%]: 6.7
No. of Peptides: 1

Peptide Match

Sequence: M.AAAGGGGGGAAAAGR.A
Score: 23.3
Modifications:

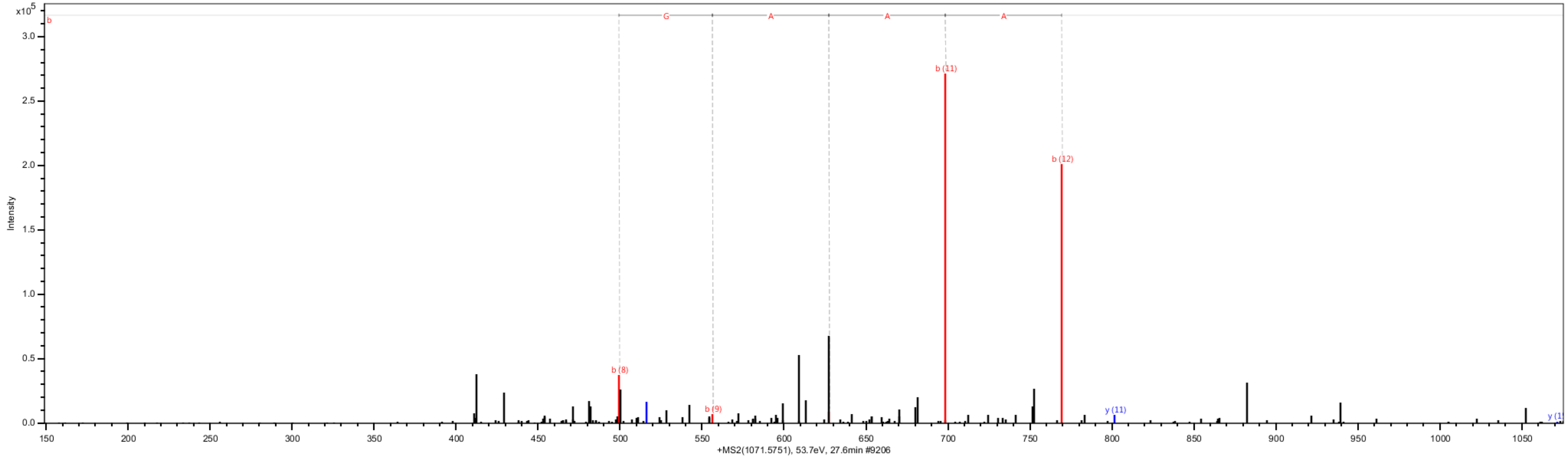

CK052_HUMAN

Accession: CK052_HUMAN
Database: SwissProt
MW [kDa] / pI: 13.9 / 9.5
Score: 23.4
Seq. Coverage [%]: 8.9
No. of Peptides: 1

Peptide Match

Sequence: R.FPQATPRYDSK.N
Score: 23.4
Modifications:

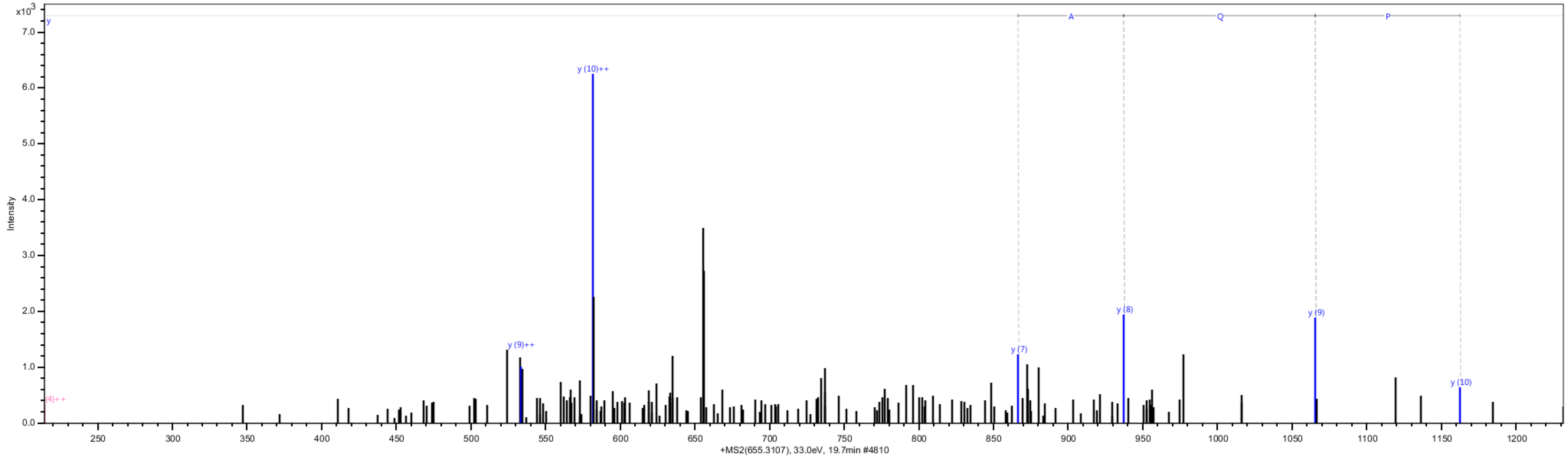

KPYM_HUMAN

Accession: KPYM_HUMAN
Database: SwissProt
MW [kDa] / pI: 57.9 / 8.0
Score: 23.5
Seq. Coverage [%]: 2.4
No. of Peptides: 1

Peptide Match

Sequence: R.NTGIICTIGPASR.S
Score: 23.4
Modifications: Carbamidomethyl: 6

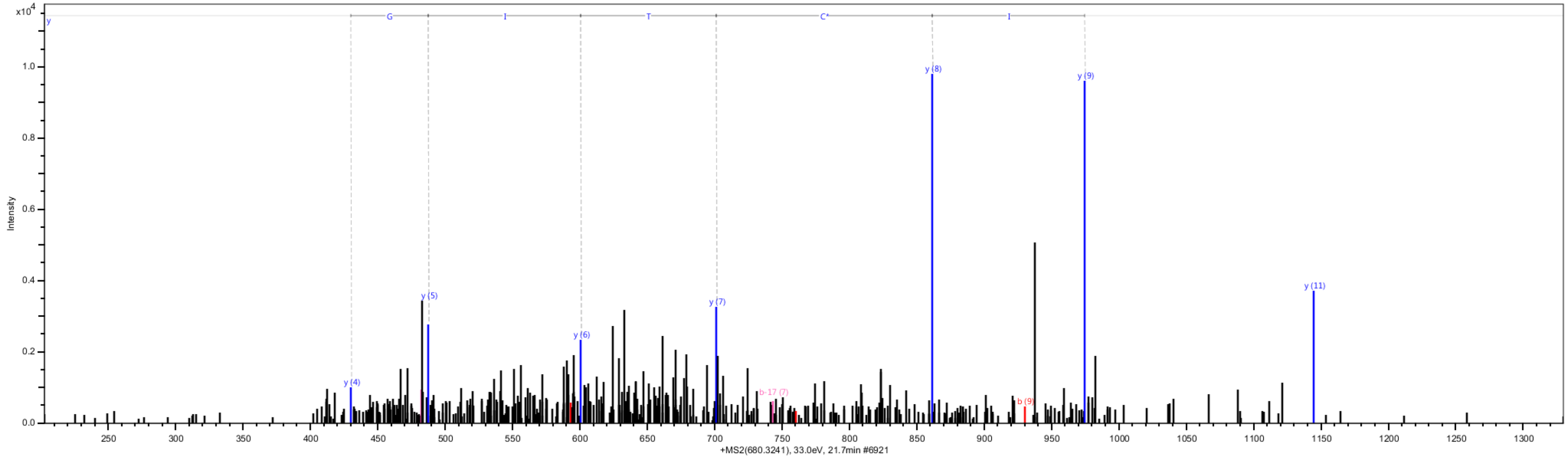

RBG10_HUMAN

Accession: RBG10_HUMAN
Database: SwissProt
MW [kDa] / pI: 29.0 / 5.3
Score: 23.5
Seq. Coverage [%]: 2.8
No. of Peptides: 1

Peptide Match

Sequence: K.AEYEIKK.T
Score: 23.5
Modifications:

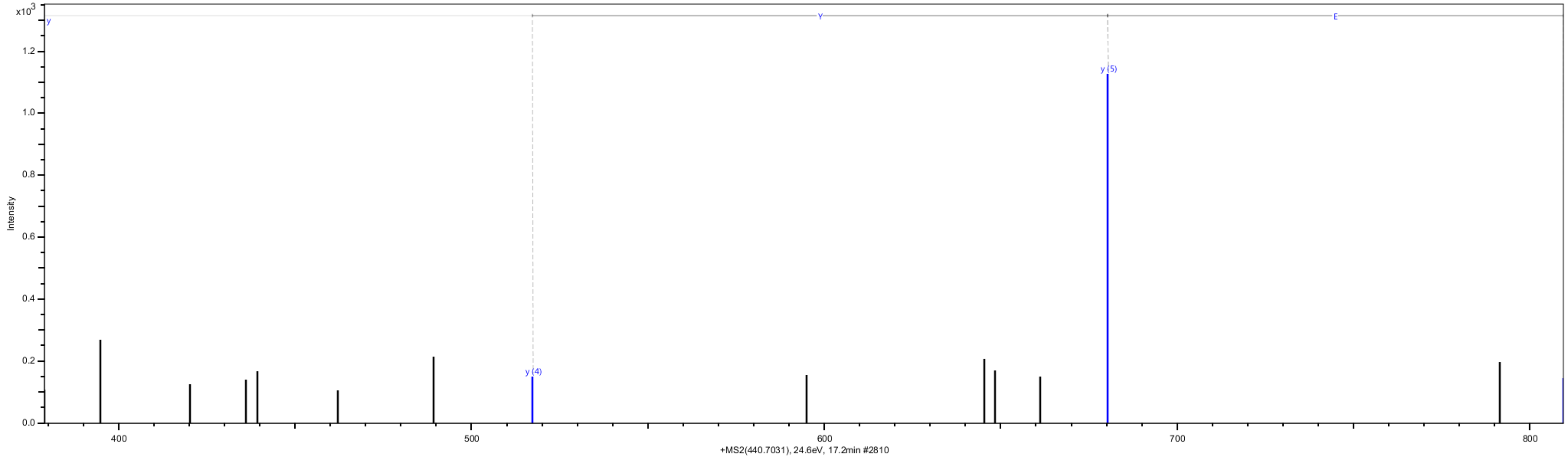

GGYF2_HUMAN

Accession: GGYF2_HUMAN
Database: SwissProt
MW [kDa] / pI: 150.0 / 5.4
Score: 23.5
Seq. Coverage [%]: 1.0
No. of Peptides: 1

Peptide Match

Sequence: K.GVSIPLMHEAMQK.W
Score: 23.5
Modifications: Oxidation: 7, 11

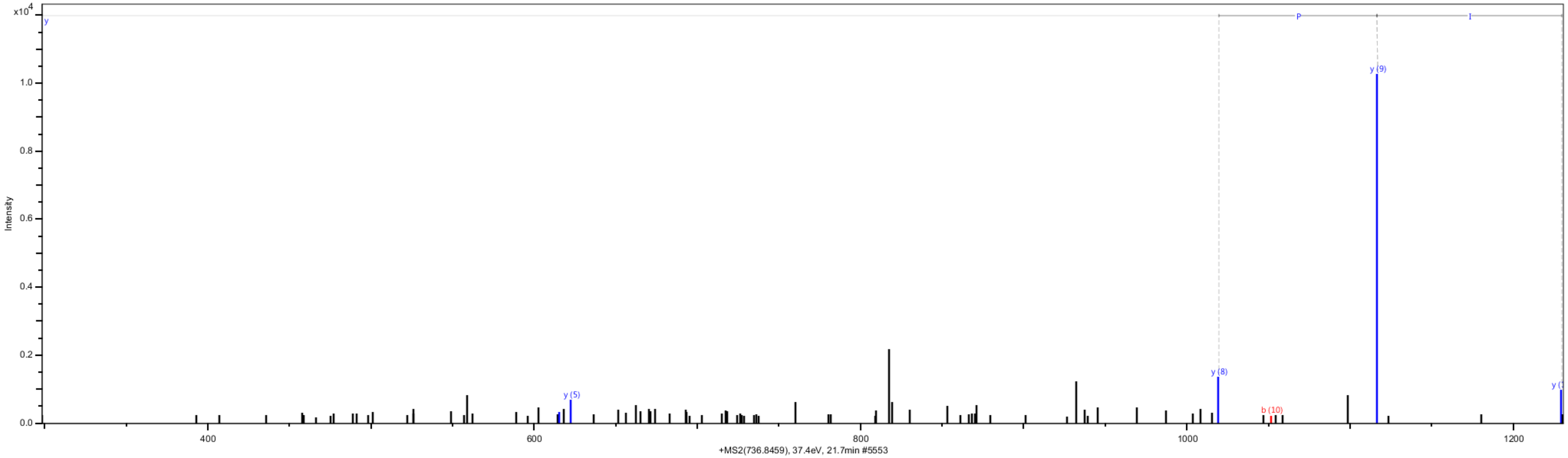

THOC6_HUMAN

Accession: THOC6_HUMAN
Database: SwissProt
MW [kDa] / pI: 37.5 / 7.1

Score: 23.7
Seq. Coverage [%]: 4.4
No. of Peptides: 1

Peptide Match

Sequence: R.LHMTIFSQSVSPCGK.F
Score: 23.7
Modifications: Carbamidomethyl: 13; Oxidation: 3

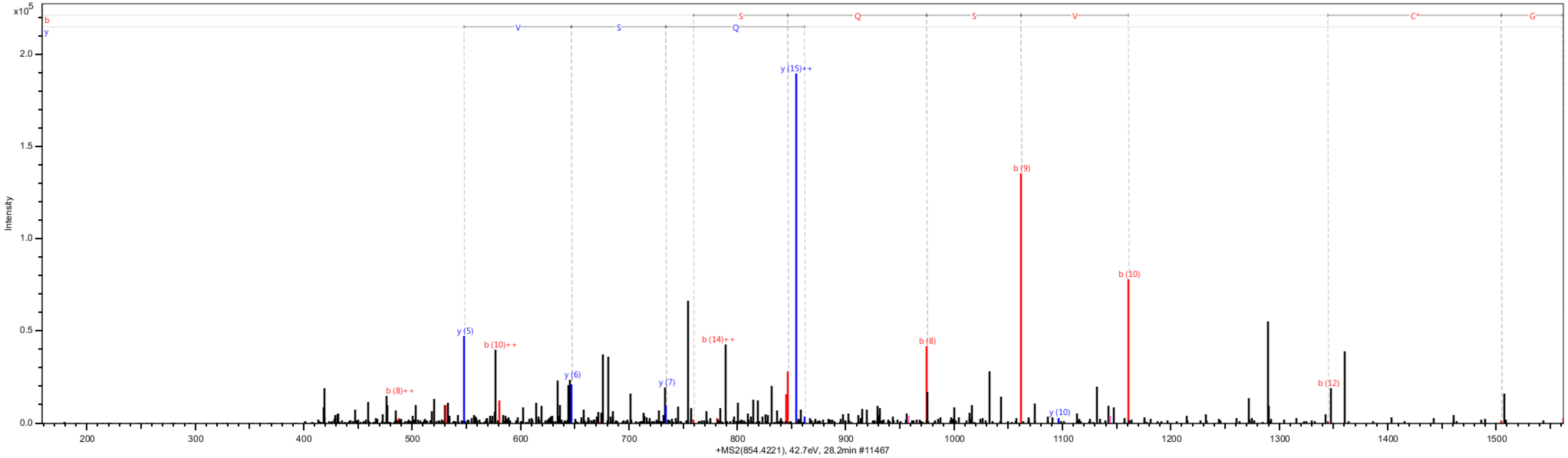

ZN622_HUMAN

**Accession:**	ZN622_HUMAN	**Score:**	23.8
**Database:**	SwissProt	**Seq. Coverage [%]:**	2.5
**MW [kDa] / pI:**	54.2 / 5.8	**No. of Peptides:**	1

Peptide Match

**Sequence:**	K.DAMNAAIQQAIK.A	**Score:**	23.8
**Modifications:**	Oxidation: 3		

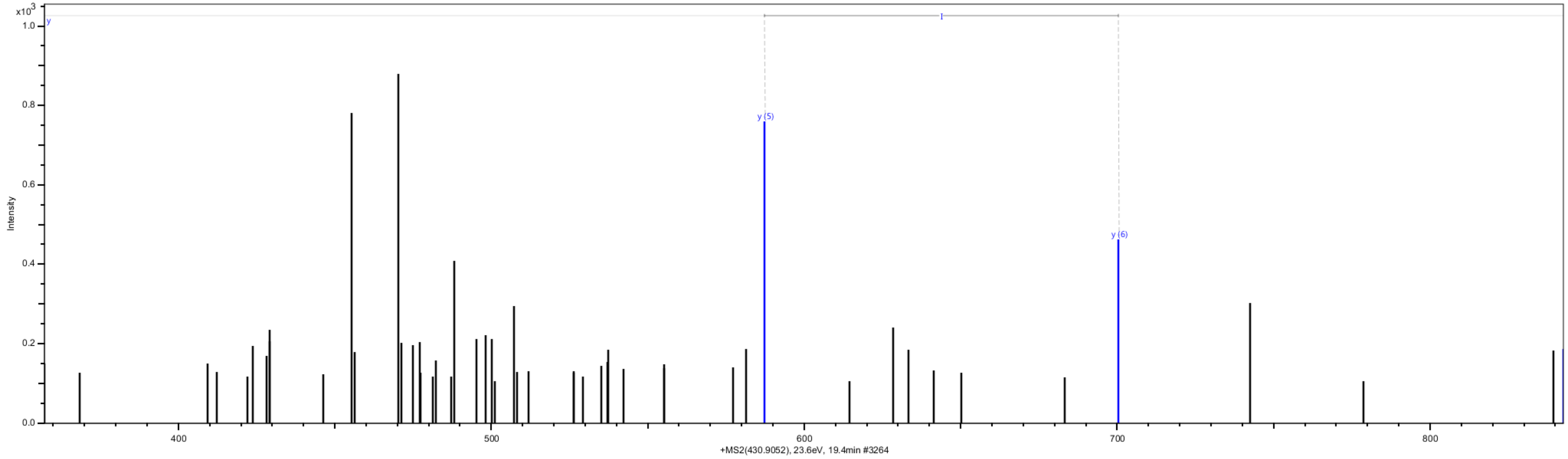

P5F1B_HUMAN

Accession: P5F1B_HUMAN
Database: SwissProt
MW [kDa] / pI: 38.6 / 6.3
Score: 19.6
Seq. Coverage [%]: 2.5
No. of Peptides: 1

Peptide Match

Sequence: K.EKLEQNPEK.S
Score: 19.6
Modifications:

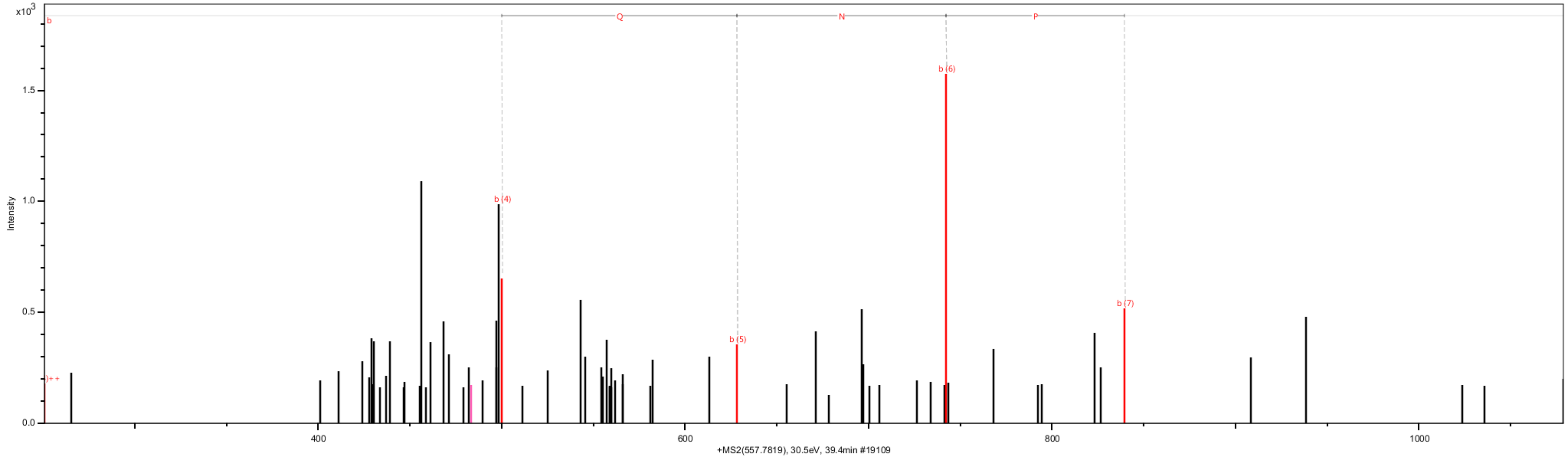

STX8_HUMAN

Accession: STX8_HUMAN

Database: SwissProt

MW [kDa] / pI: 26.9 / 4.9

Score: 19.6

Seq. Coverage [%]: 4.2

No. of Peptides: 1

Peptide Match

Sequence: R.QNLLDDLVTR.E

Score: 19.6

Modifications:

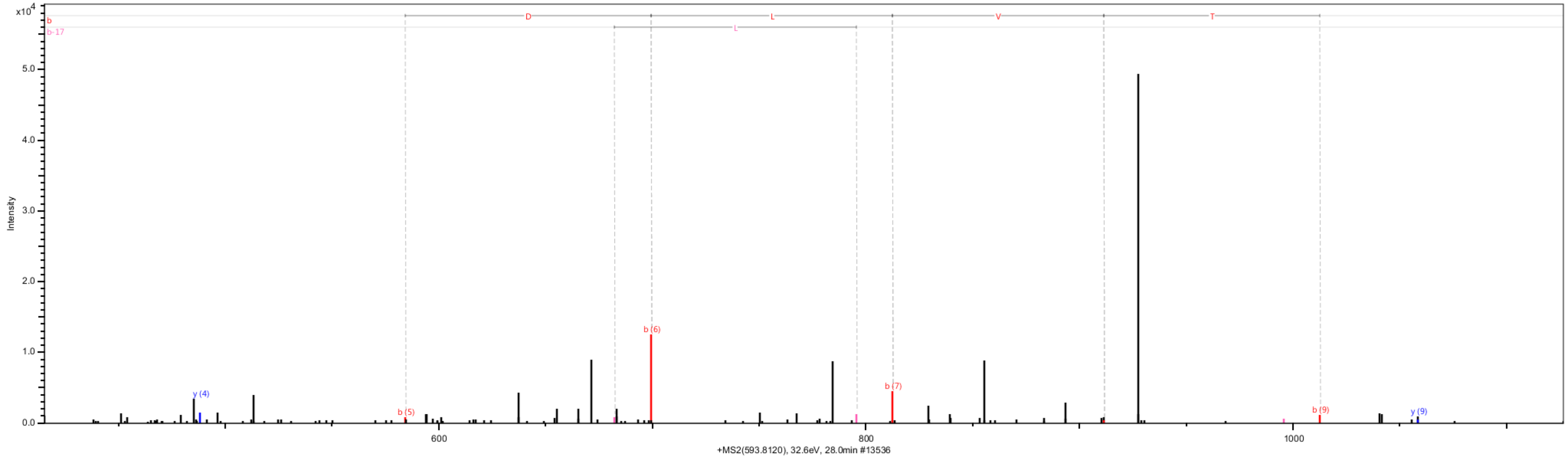

MTUS1_HUMAN

**Accession:**	MTUS1_HUMAN	**Score:**	19.6
**Database:**	SwissProt	**Seq. Coverage [%]:**	0.8
**MW [kDa] / pI:**	141.3 / 7.3	**No. of Peptides:**	1

Peptide Match

**Sequence:**	R.QQTVLSRTPR.S	**Score:**	19.6
**Modifications:**			

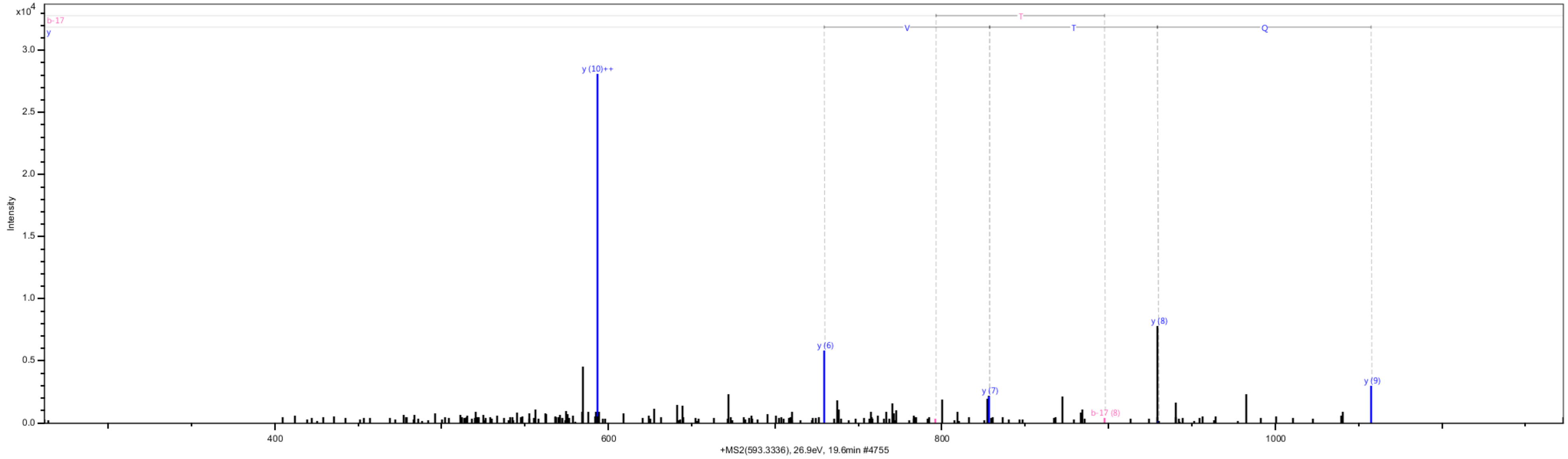

DMD_HUMAN

Accession: DMD_HUMAN **Score:** 19.5
Database: SwissProt **Seq. Coverage [%]:** 0.2
MW [kDa] / pI: 426.5 / 5.6 **No. of Peptides:** 1

Peptide Match

Sequence: R.IEHYASR.L **Score:** 19.5
Modifications:

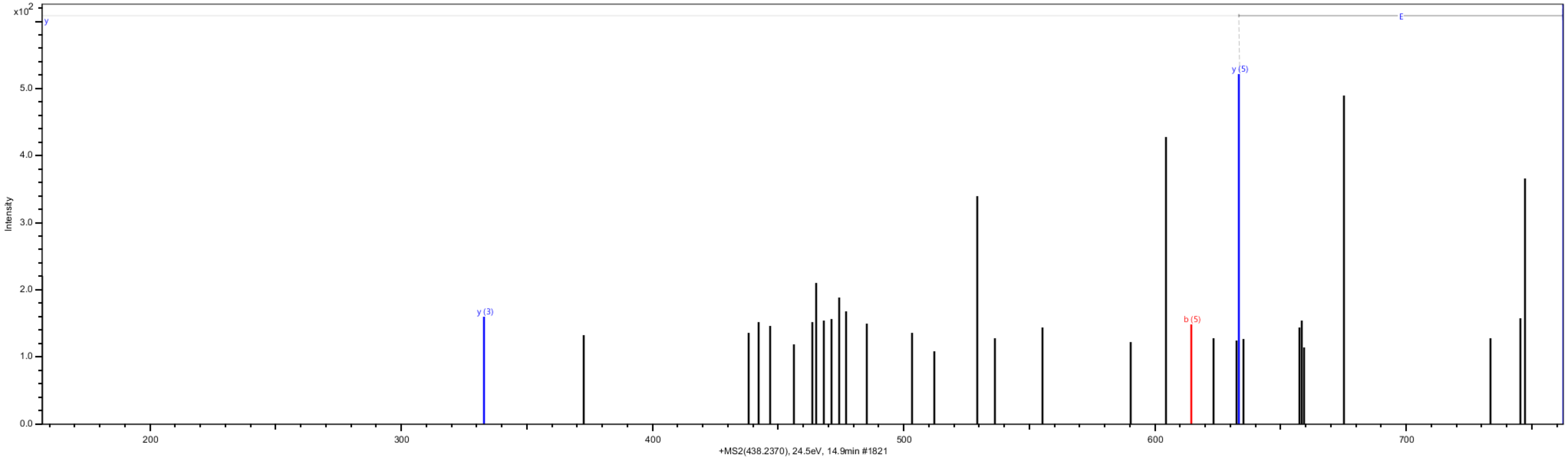

SLN11_HUMAN

**Accession:**	SLN11_HUMAN	**Score:**	19.5
**Database:**	SwissProt	**Seq. Coverage [%]:**	1.2
**MW [kDa] / pI:**	102.8 / 8.0	**No. of Peptides:**	1

Peptide Match

**Sequence:**	K.QQGRCFYIFVK.S	**Score:**	19.5
**Modifications:**	Carbamidomethyl: 5		

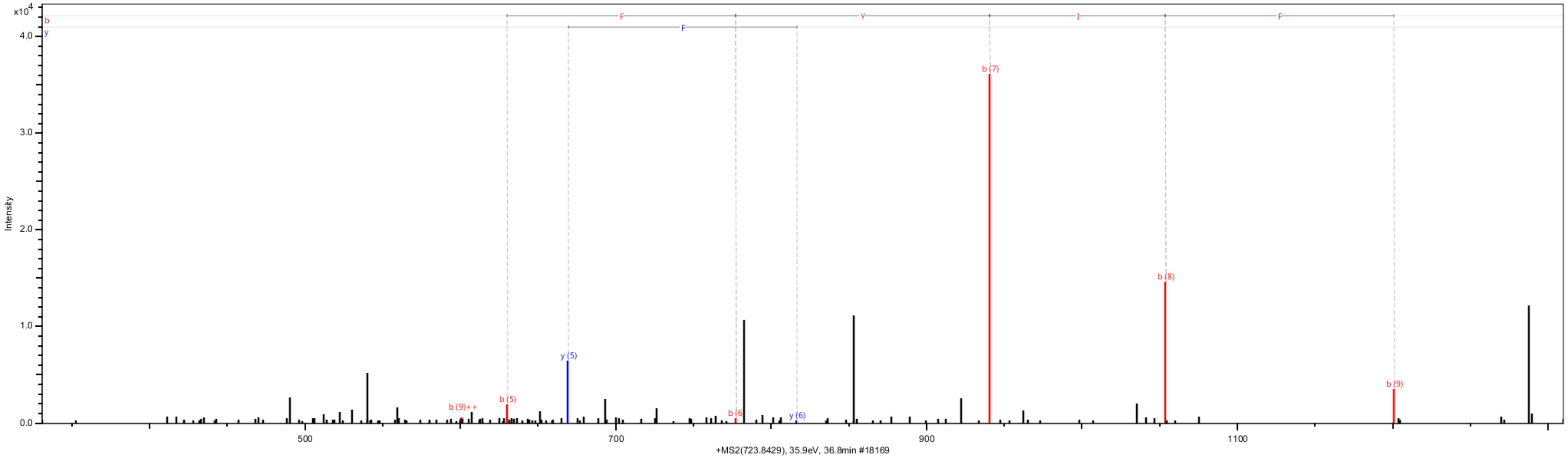

PKN2_HUMAN

Accession: PKN2_HUMAN
Database: SwissProt
MW [kDa] / pI: 112.0 / 5.9
Score: 19.5
Seq. Coverage [%]: 0.9
No. of Peptides: 1

Peptide Match

Sequence: R.DFDYIADWC.-
Score: 19.5
Modifications: Carbamidomethyl: 9

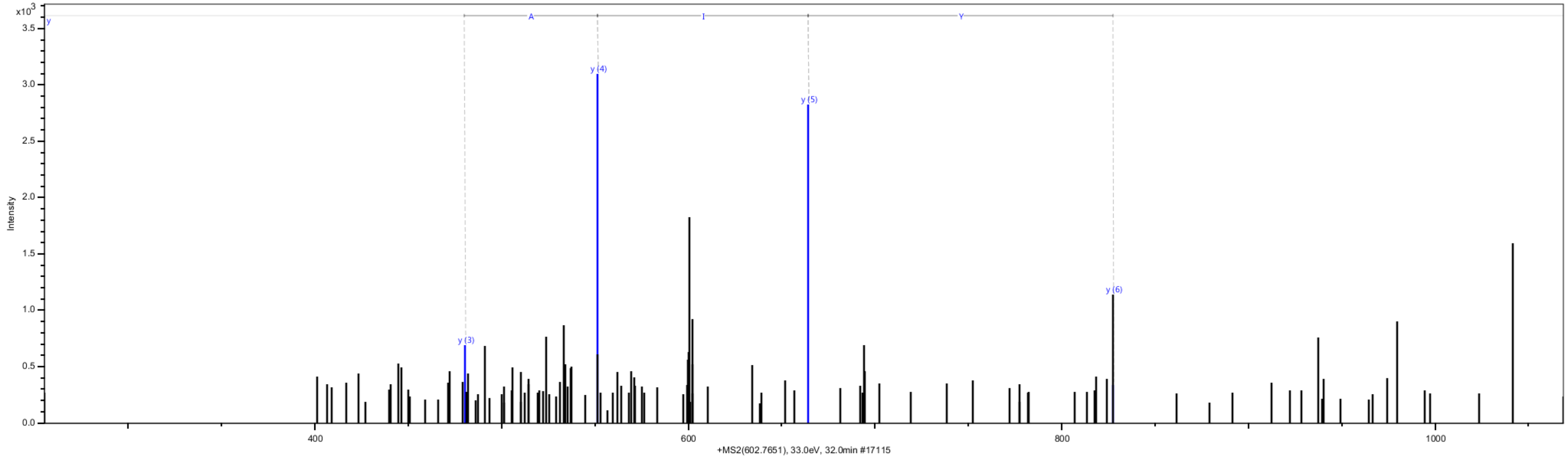

CHSTF_HUMAN

Accession: CHSTF_HUMAN
Database: SwissProt
MW [kDa] / pI: 64.9 / 8.6
Score: 19.5
Seq. Coverage [%]: 1.4
No. of Peptides: 1

Peptide Match

Sequence: K.ILFRVDSK.Q
Score: 19.4
Modifications:

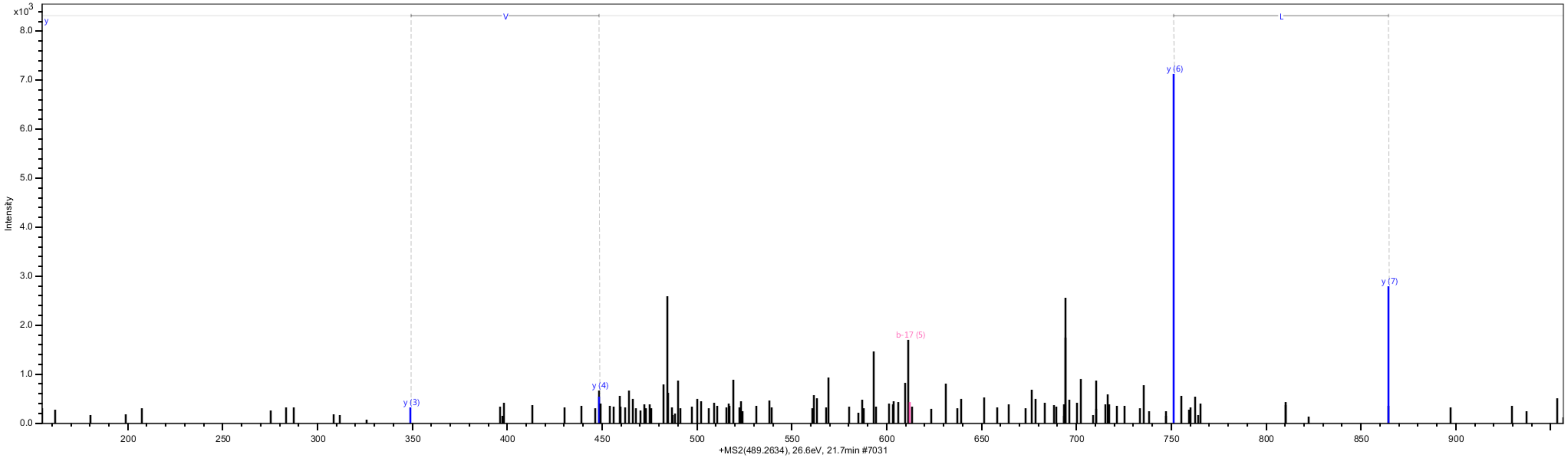

CTIP_HUMAN

**Accession:**	CTIP_HUMAN	**Score:**	19.4
**Database:**	SwissProt	**Seq. Coverage [%]:**	1.0
**MW [kDa] / pI:**	101.9 / 5.9	**No. of Peptides:**	1

Peptide Match

**Sequence:**	R.CAVTEEHMR.K	**Score:**	19.4
**Modifications:**	Carbamidomethyl: 1; Oxidation: 8		

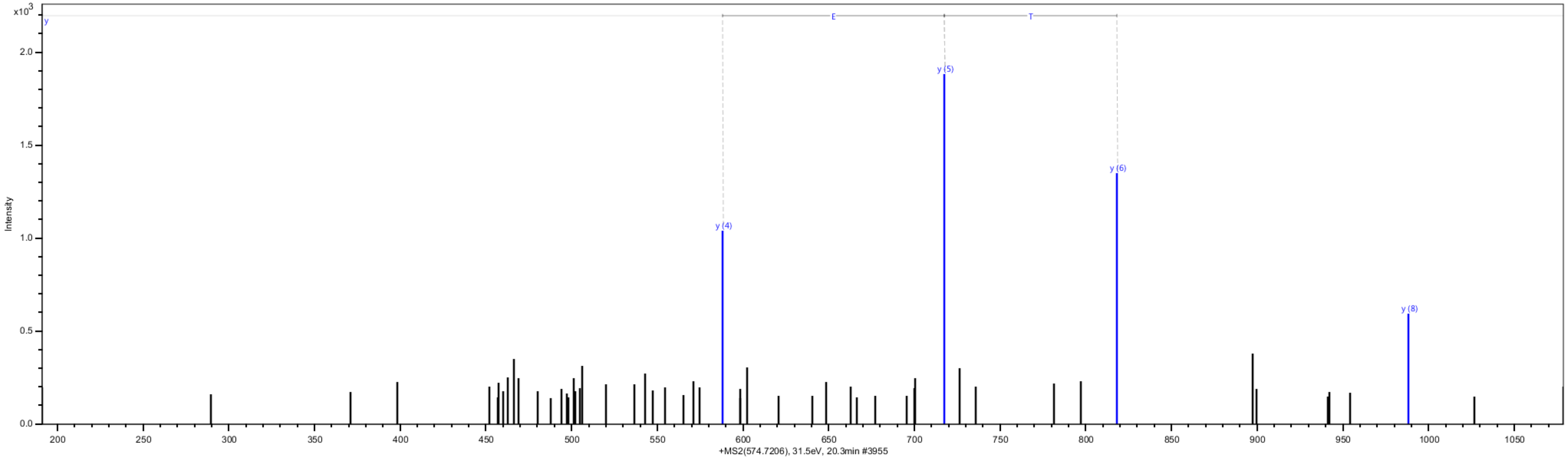

MS18B_HUMAN

Accession: MS18B_HUMAN
Database: SwissProt
MW [kDa] / pI: 24.7 / 7.0
Score: 19.4
Seq. Coverage [%]: 3.1
No. of Peptides: 1

Peptide Match

Sequence: -.MAAQPLR.H
Score: 19.4
Modifications:

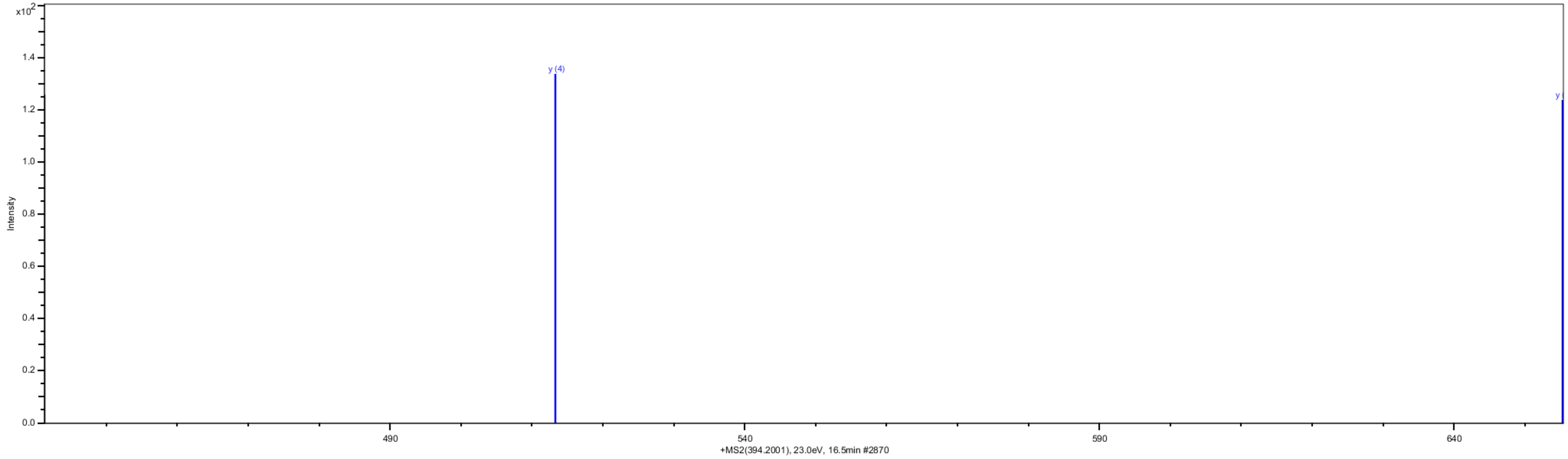

I20RB_HUMAN

**Accession:**	I20RB_HUMAN	**Score:**	19.4
**Database:**	SwissProt	**Seq. Coverage [%]:**	2.3
**MW [kDa] / pI:**	35.1 / 5.0	**No. of Peptides:**	1

Peptide Match

**Sequence:**	K.ITNSPQK.L	**Score:**	19.4
**Modifications:**			

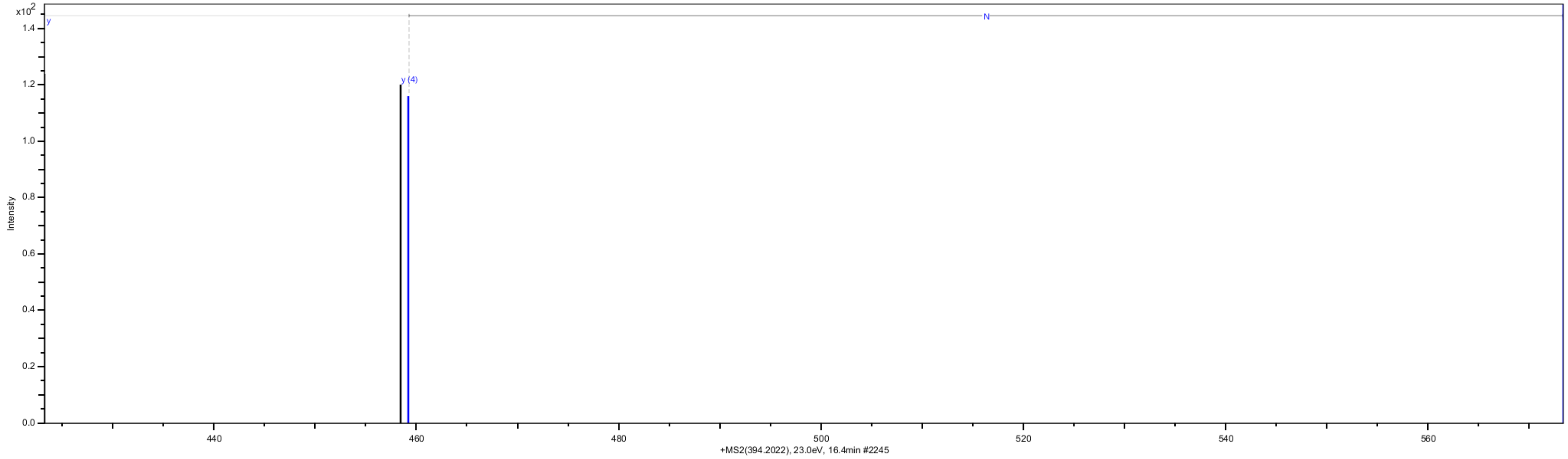

TLRN1_HUMAN

Accession: TLRN1_HUMAN
Database: SwissProt
MW [kDa] / pI: 37.7 / 8.5
Score: 19.4
Seq. Coverage [%]: 2.2
No. of Peptides: 1

Peptide Match

Sequence: K.VAPSELAR.S
Score: 19.4
Modifications:

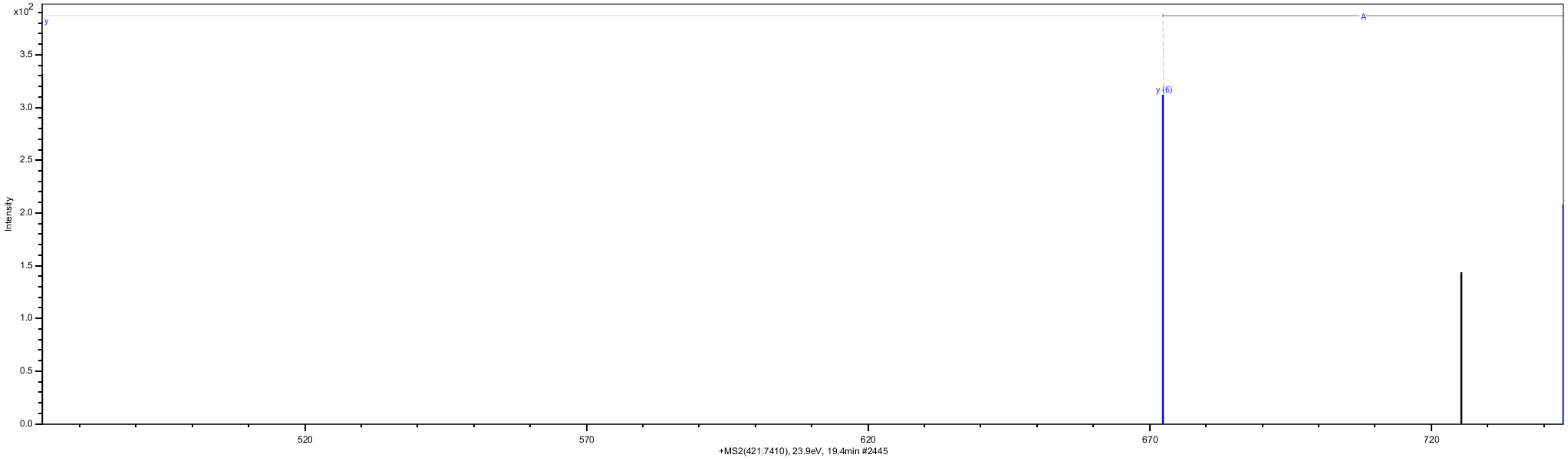

AFG32_HUMAN

Accession: AFG32_HUMAN
Database: SwissProt
MW [kDa] / pI: 88.5 / 8.8
Score: 19.4
Seq. Coverage [%]: 1.0
No. of Peptides: 1

Peptide Match

Sequence: R.RGPAGIGR.T
Score: 19.4
Modifications:

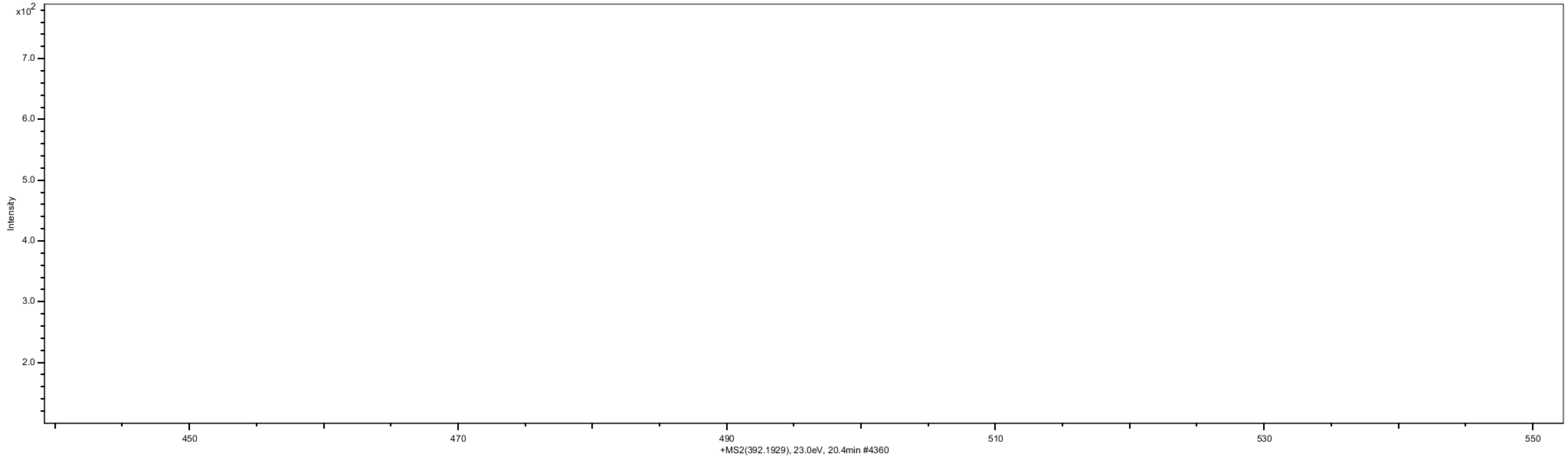

GRM4_HUMAN

Accession: GRM4_HUMAN
Database: SwissProt
MW [kDa] / pI: 101.8 / 9.1
Score: 19.4
Seq. Coverage [%]: 1.2
No. of Peptides: 1

Peptide Match

Sequence: K.AVVTAATMSNK.F
Score: 19.4
Modifications:

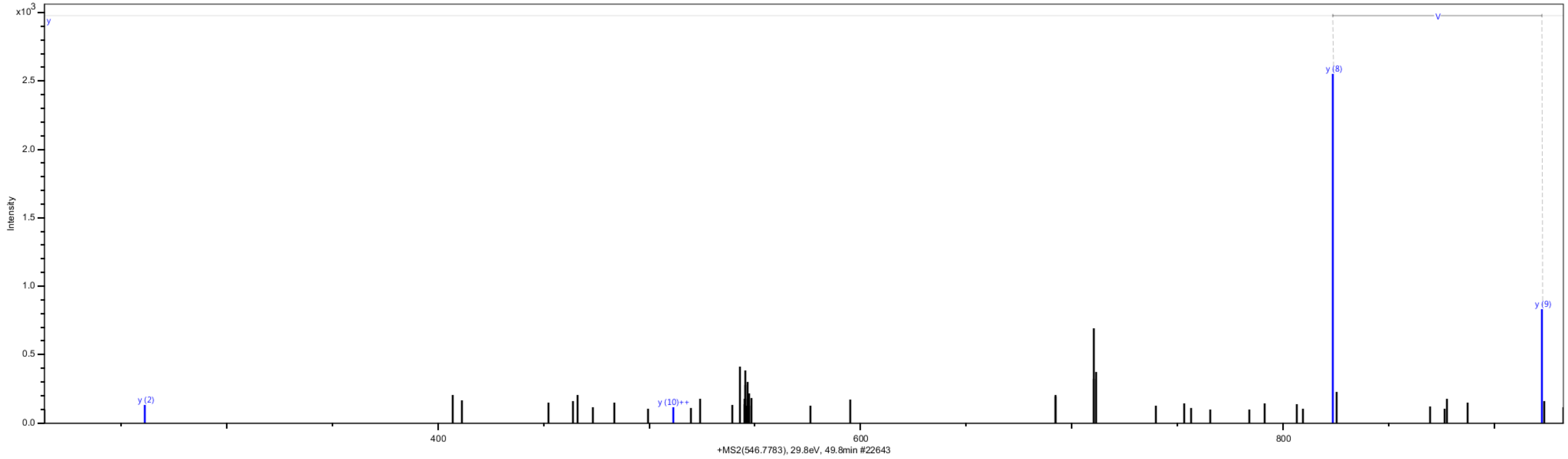

FCGBP_HUMAN

Accession: FCGBP_HUMAN
Database: SwissProt
MW [kDa] / pI: 571.6 / 5.1
Score: 19.4
Seq. Coverage [%]: 0.1
No. of Peptides: 1

Peptide Match

Sequence: K.DEIGKVR.V
Score: 19.4
Modifications:

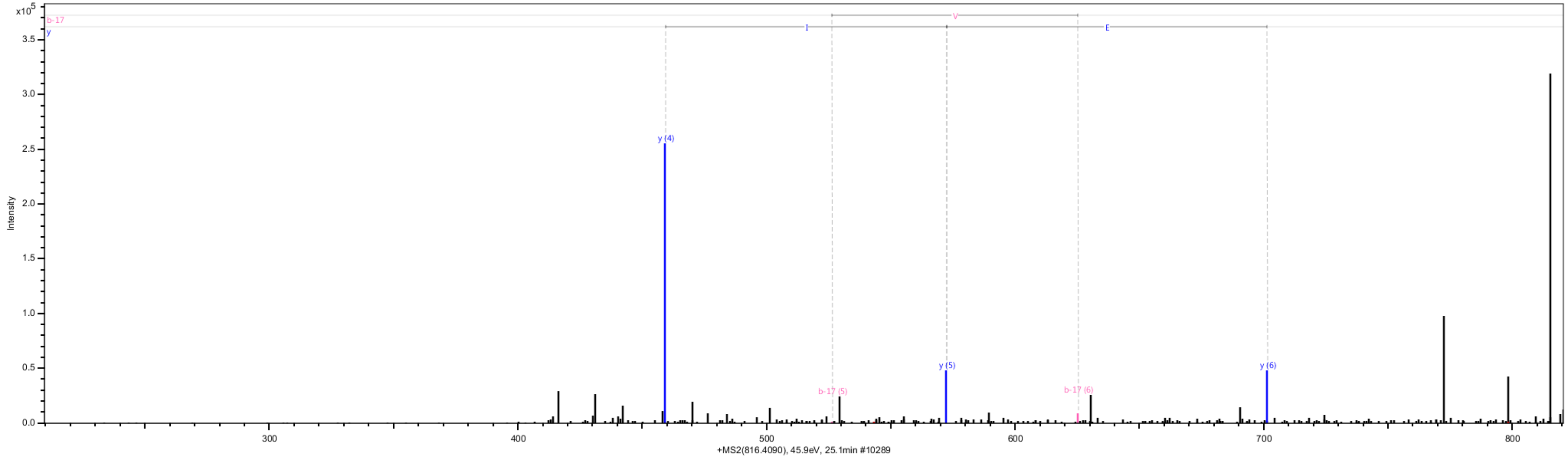

C19L2_HUMAN

Accession: C19L2_HUMAN
Database: SwissProt
MW [kDa] / pI: 103.7 / 8.8
Score: 19.4
Seq. Coverage [%]: 1.6
No. of Peptides: 1

Peptide Match

Sequence: K.AEMMGNMELAEQLK.V
Score: 19.4
Modifications: Oxidation: 3, 4

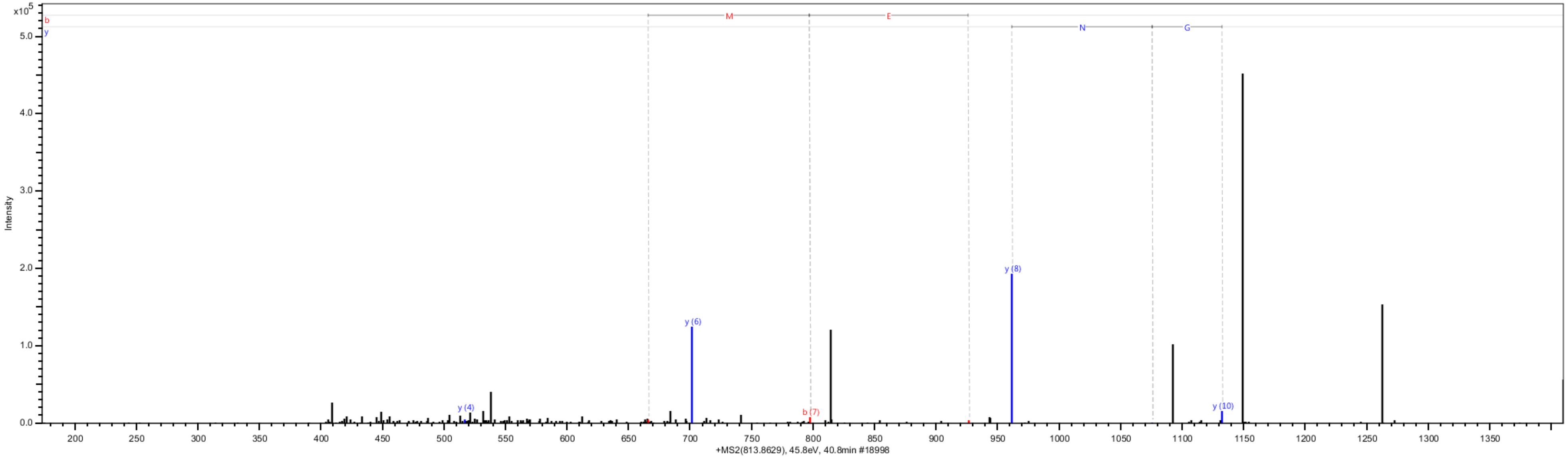

PPIH_HUMAN

Accession: PPIH_HUMAN
Database: SwissProt
MW [kDa] / pI: 19.2 / 8.3
Score: 19.3
Seq. Coverage [%]: 6.2
No. of Peptides: 1

Peptide Match

Sequence: K.LPVVISQCGEM.-
Score: 19.3
Modifications: Carbamidomethyl: 8; Oxidation: 11

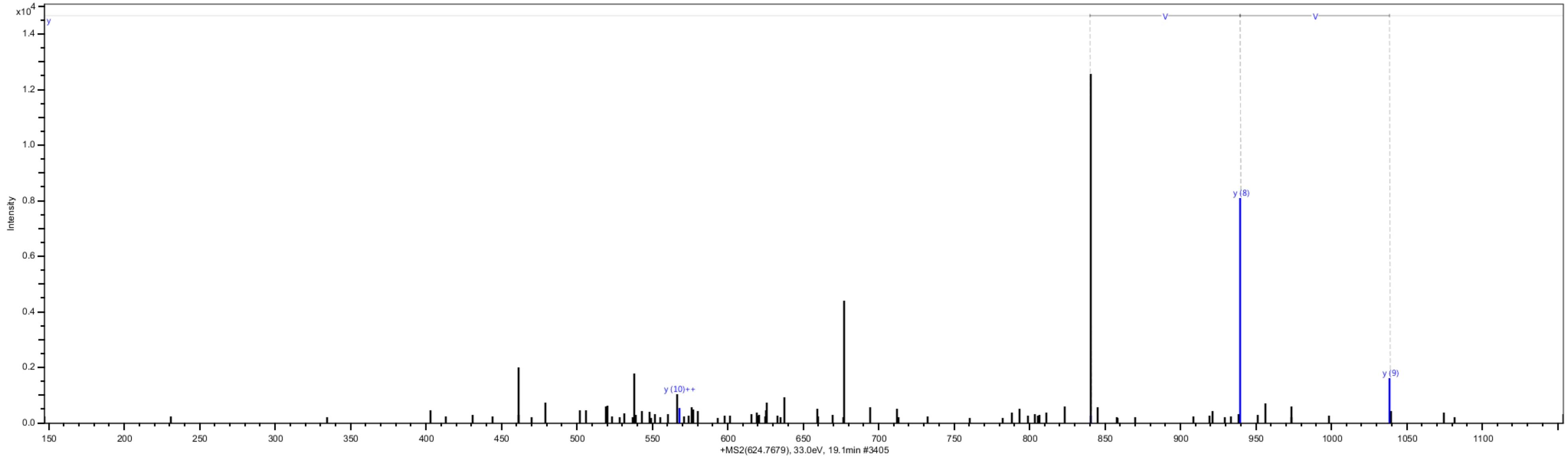

ATP4A_HUMAN

Accession: ATP4A_HUMAN
Database: SwissProt
MW [kDa] / pI: 114.0 / 5.6
Score: 19.9
Seq. Coverage [%]: 2.0
No. of Peptides: 1

Peptide Match

Sequence: K.DMDPSELVEALRTHPEMVFAR.T
Score: 19.9
Modifications: Oxidation: 2, 17

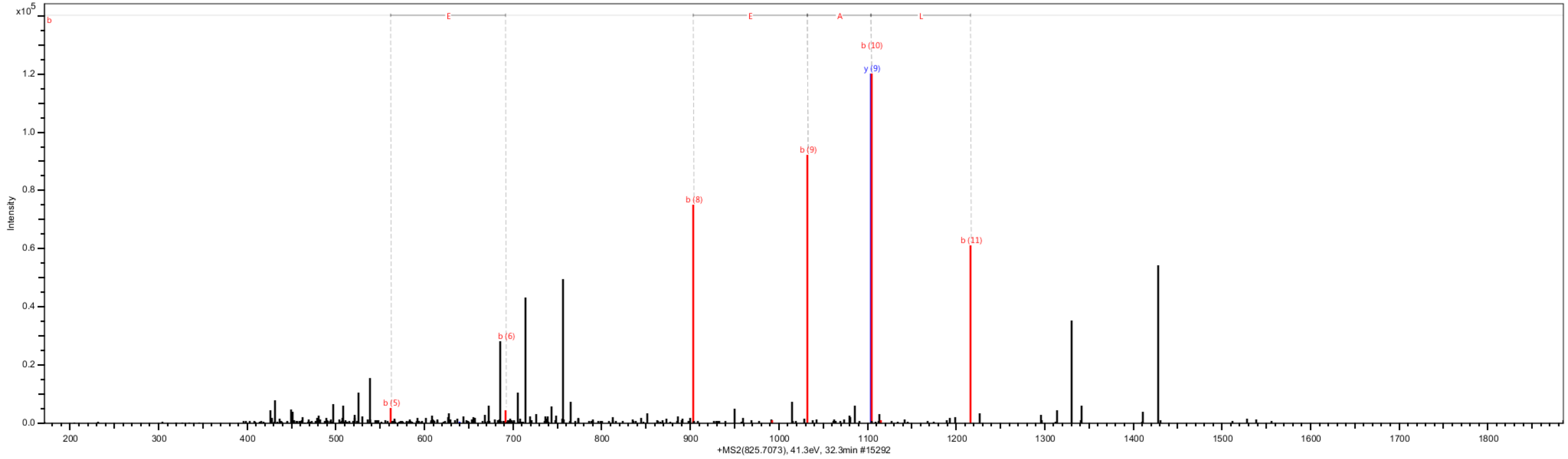

CCHCR_HUMAN

Accession: CCHCR_HUMAN
Database: SwissProt
MW [kDa] / pI: 88.6 / 5.8
Score: 19.9
Seq. Coverage [%]: 1.2
No. of Peptides: 1

Peptide Match

Sequence: R.EQLSDTERR.L
Score: 19.9
Modifications:

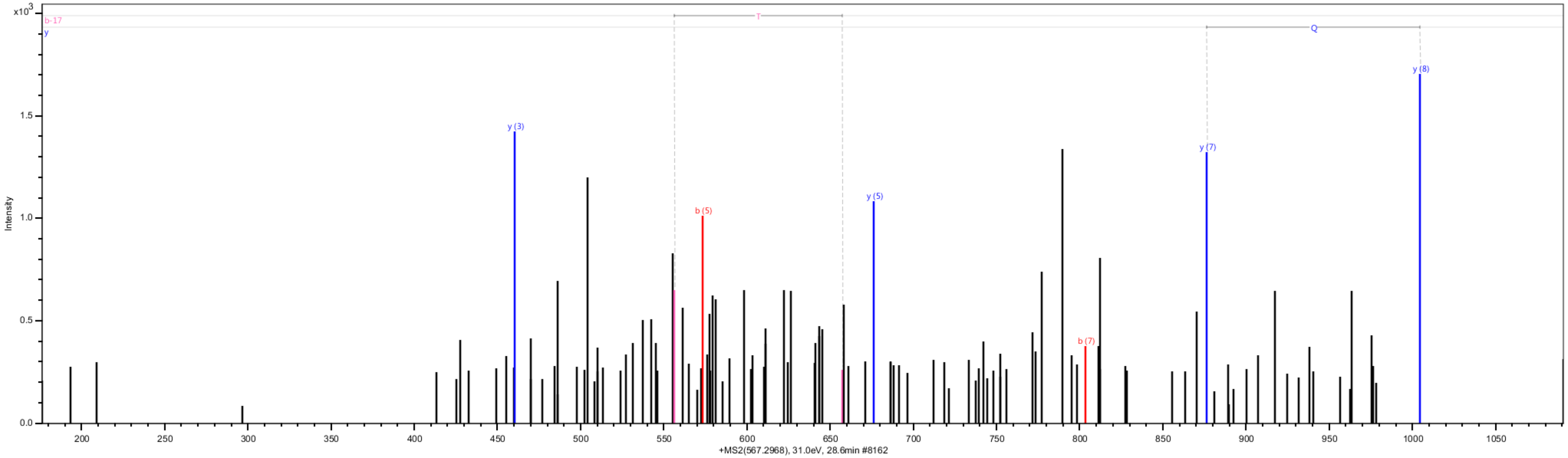

U520_HUMAN

Accession: U520_HUMAN
Database: SwissProt
MW [kDa] / pI: 244.4 / 5.7
Score: 19.8
Seq. Coverage [%]: 0.5
No. of Peptides: 1

Peptide Match

Sequence: R.FYDDAIVSQKK.A
Score: 19.8
Modifications:

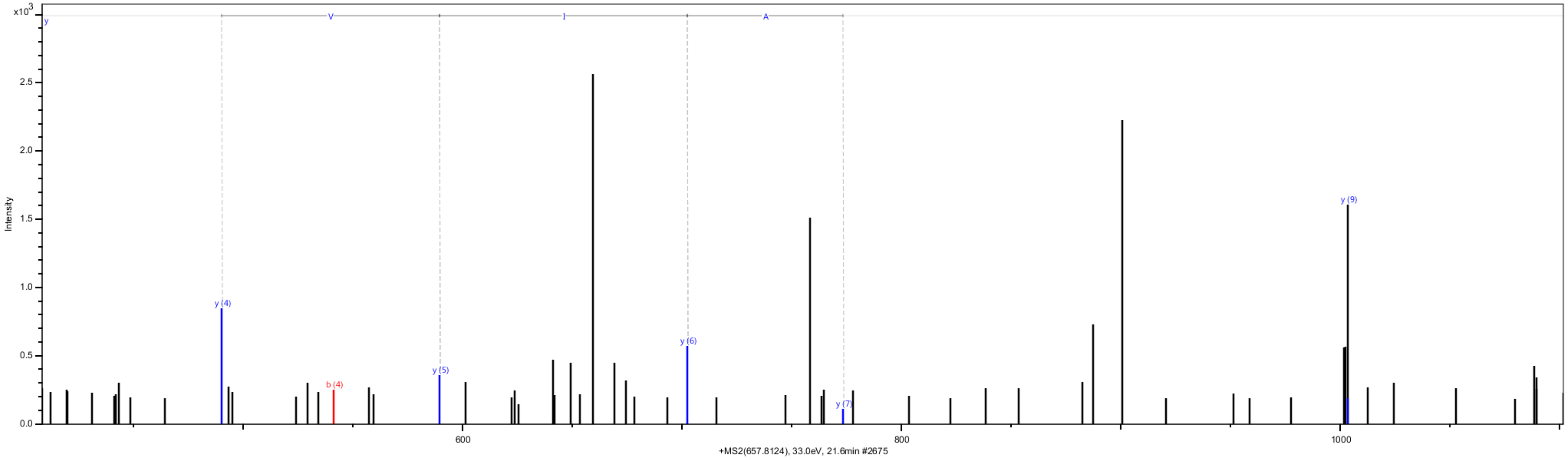

KANL1_HUMAN

Accession: KANL1_HUMAN
Database: SwissProt
MW [kDa] / pI: 121.0 / 9.0
Score: 19.9
Seq. Coverage [%]: 1.5
No. of Peptides: 1

Peptide Match

Sequence: K.KSPLSSILFSALDSDTR.I
Score: 19.9
Modifications:

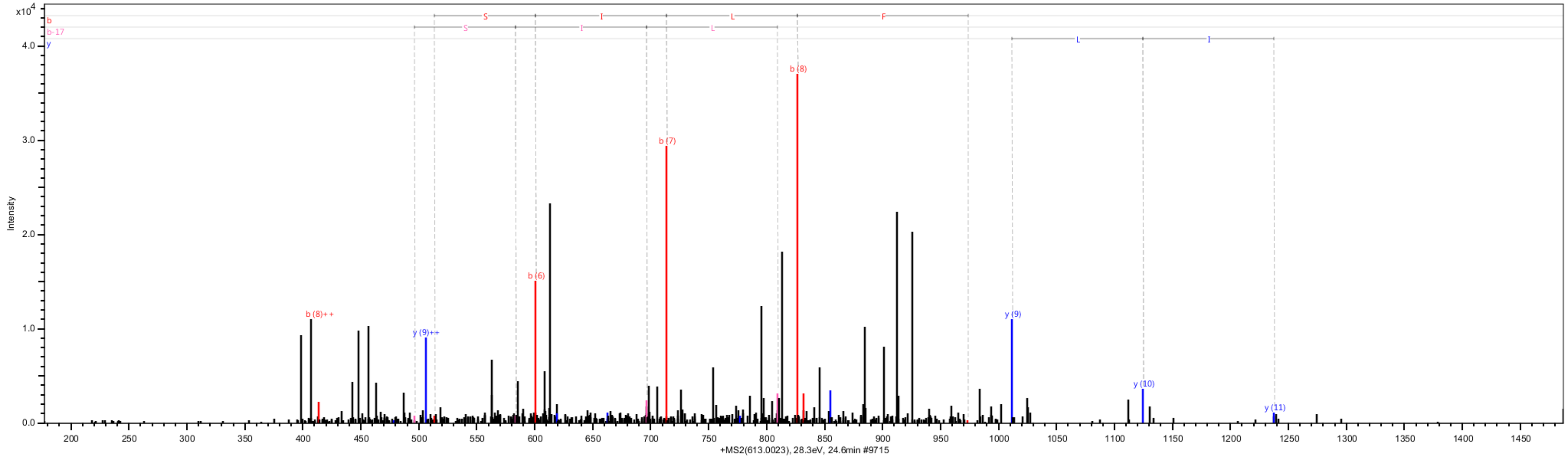

GFAP_HUMAN

Accession: GFAP_HUMAN
Database: SwissProt
MW [kDa] / pI: 49.8 / 5.4
Score: 19.8
Seq. Coverage [%]: 2.5
No. of Peptides: 1

Peptide Match

Sequence: K.LALDIEIATYR.K
Score: 19.8
Modifications:

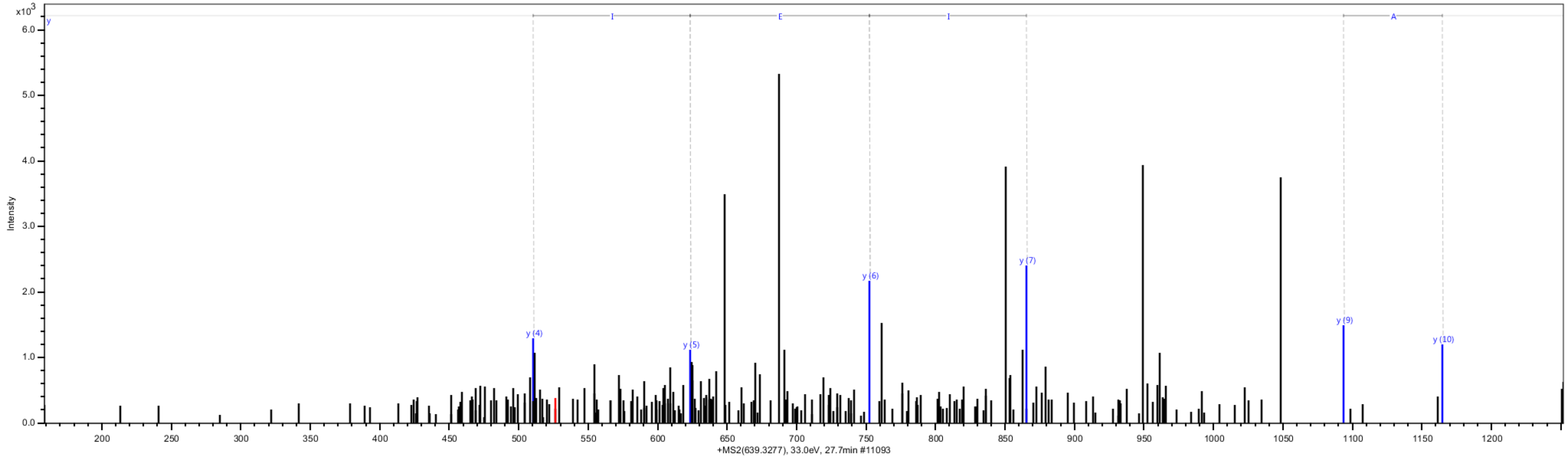

VIR_HUMAN

**Accession:**	VIR_HUMAN	**Score:**	19.7
**Database:**	SwissProt	**Seq. Coverage [%]:**	0.4
**MW [kDa] / pI:**	201.9 / 4.9	**No. of Peptides:**	1

Peptide Match

**Sequence:**	K.LLQEIVR.S	**Score:**	19.7
**Modifications:**			

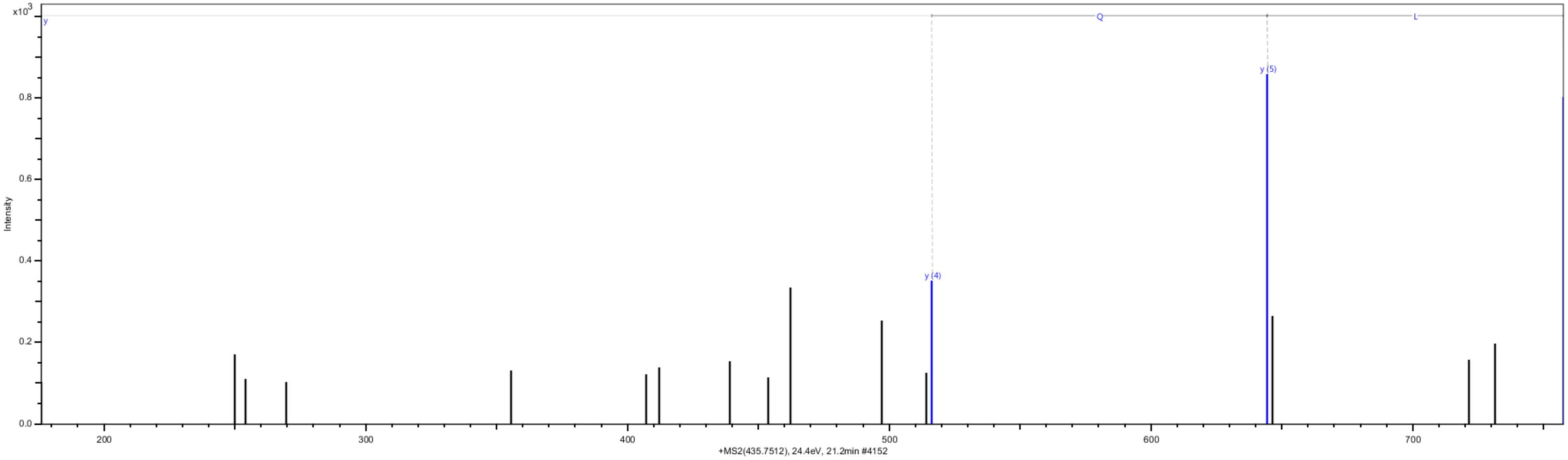

IPRI_HUMAN

**Accession:**	IPRI_HUMAN	**Score:**	19.7
**Database:**	SwissProt	**Seq. Coverage [%]:**	2.2
**MW [kDa] / pI:**	62.0 / 5.6	**No. of Peptides:**	1

Peptide Match

**Sequence:**	R.ADGDTLSCICGK.T	**Score:**	19.7
**Modifications:**	Carbamidomethyl: 8, 10		

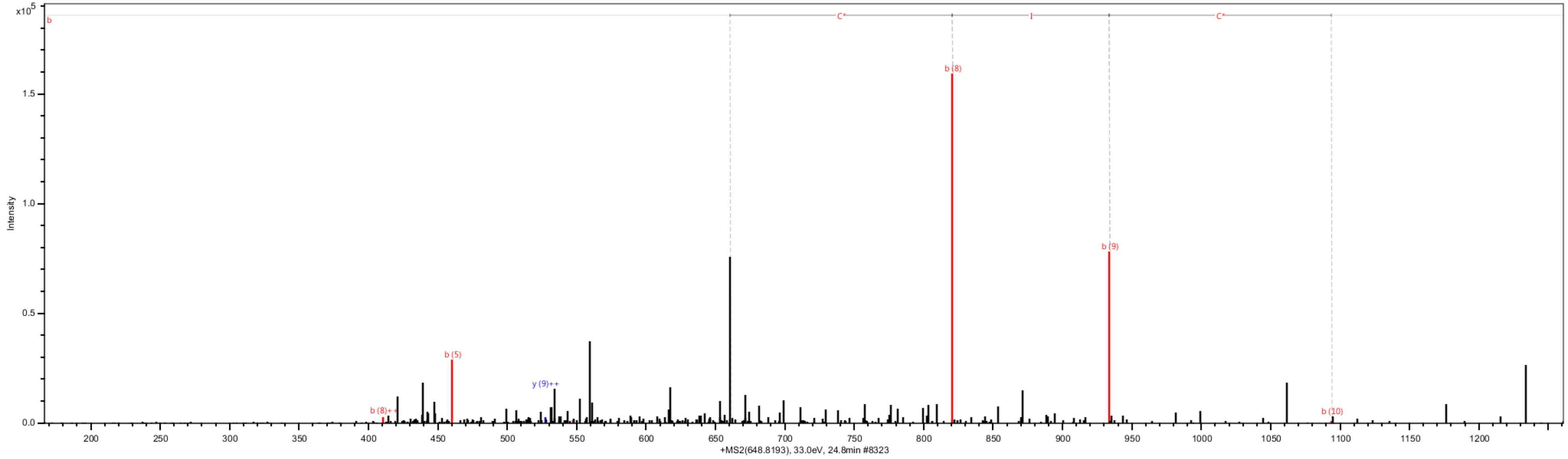

KVD13_HUMAN

**Accession:**	KVD13_HUMAN	**Score:**	19.7
**Database:**	SwissProt	**Seq. Coverage [%]:**	13.7
**MW [kDa] / pI:**	12.6 / 7.7	**No. of Peptides:**	1

Peptide Match

**Sequence:**	K.LLIYDASSLESGVPSR.F	**Score:**	19.7
**Modifications:**			

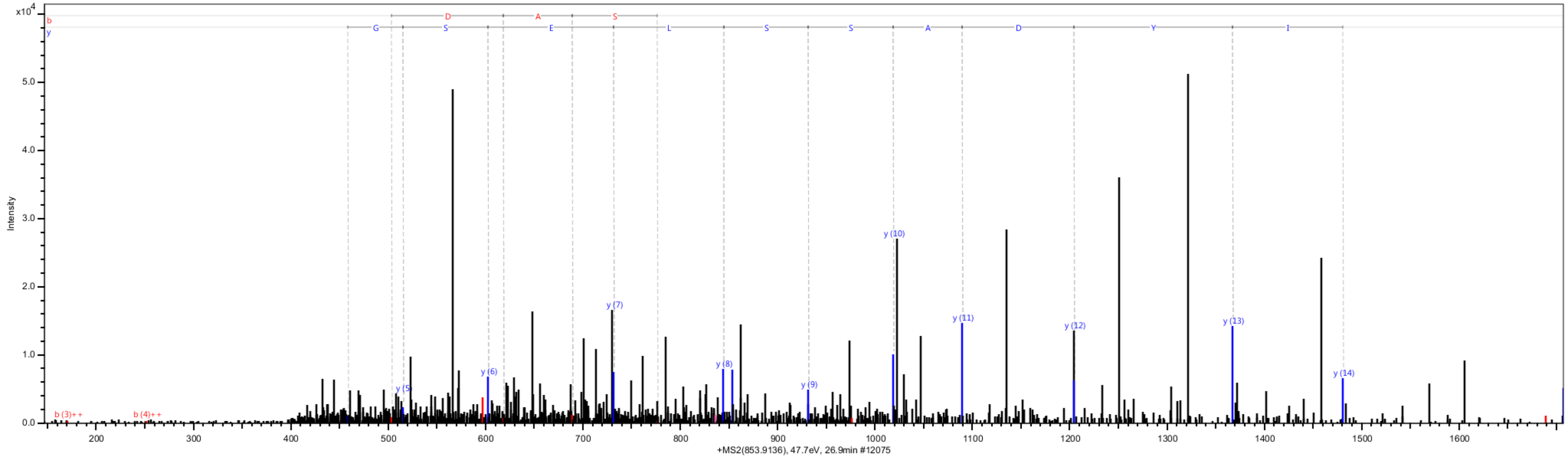

NUMBL_HUMAN

**Accession:**	NUMBL_HUMAN	**Score:**	19.7
**Database:**	SwissProt	**Seq. Coverage [%]:**	2.0
**MW [kDa] / pI:**	64.9 / 9.1	**No. of Peptides:**	1

Peptide Match

**Sequence:**	K.ECGVTAAFDASR.T	**Score:**	19.7
**Modifications:**	Carbamidomethyl: 2		

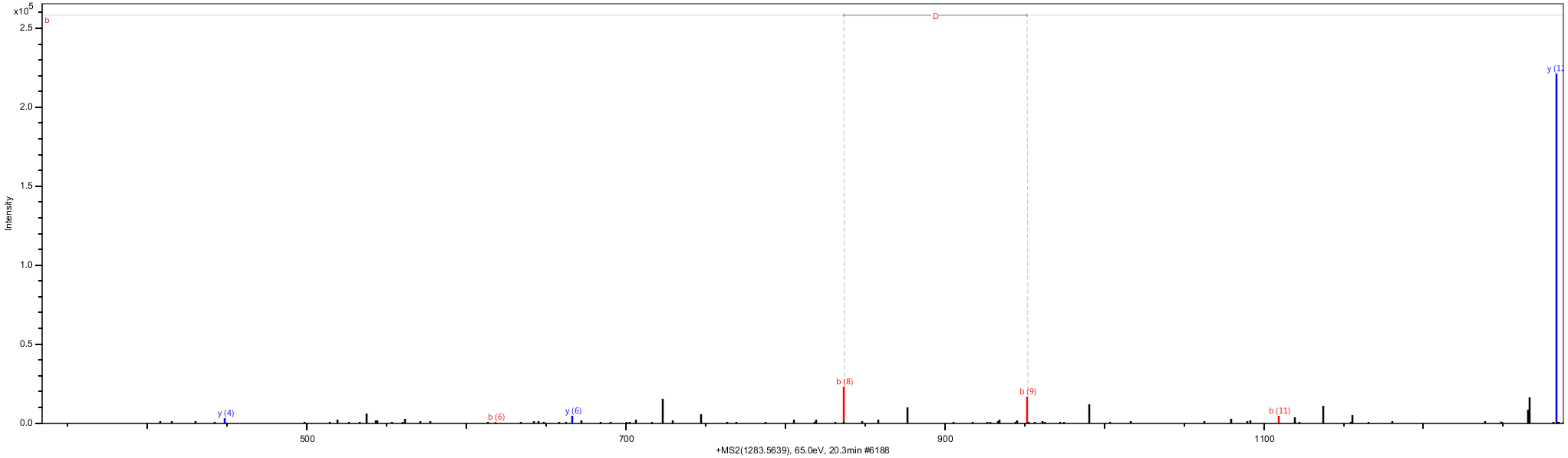

MECP2_HUMAN

Accession: MECP2_HUMAN
Database: SwissProt
MW [kDa] / pI: 52.4 / 10.0
Score: 19.7
Seq. Coverage [%]: 2.1
No. of Peptides: 1

Peptide Match

Sequence: R.SVQETVLPIK.K
Score: 19.7
Modifications:

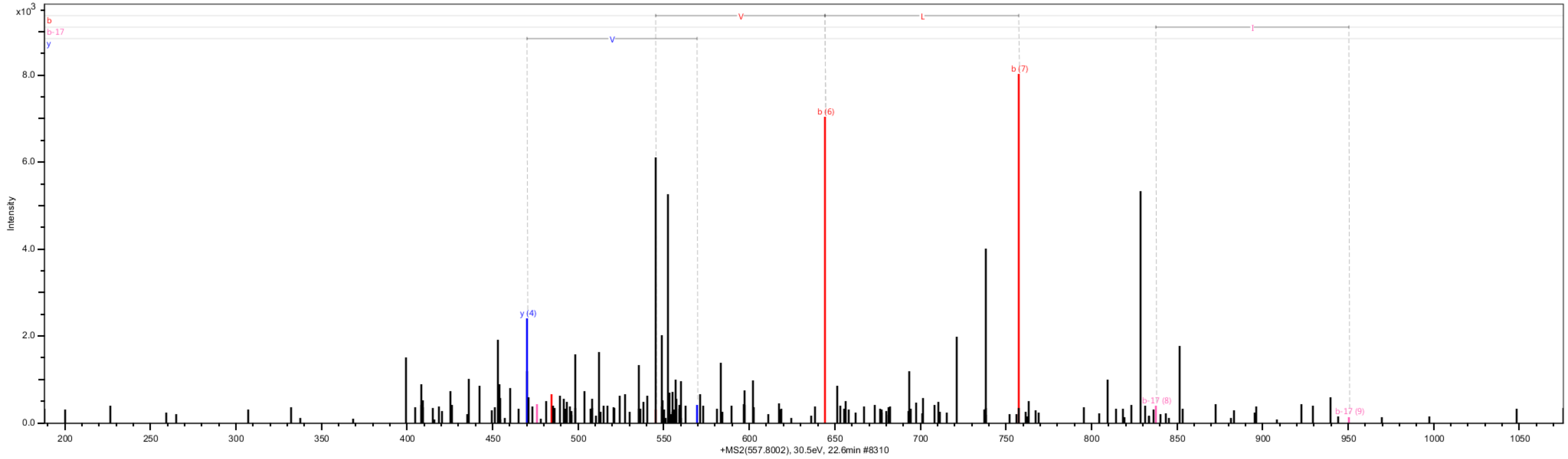

RL21_HUMAN

Accession: RL21_HUMAN
Database: SwissProt
MW [kDa] / pI: 18.6 / 10.5
Score: 19.6
Seq. Coverage [%]: 4.4
No. of Peptides: 1

Peptide Match

Sequence: K.GTWVQLK.R
Score: 19.6
Modifications:

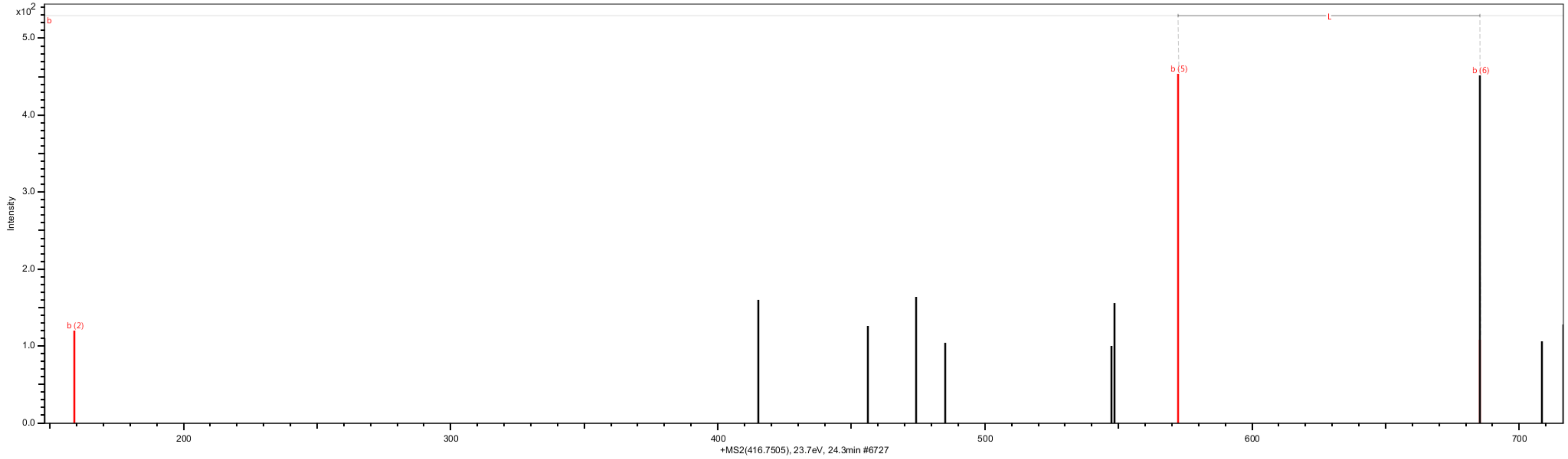

SC61G_HUMAN

**Accession:**	SC61G_HUMAN	**Score:**	19.6
**Database:**	SwissProt	**Seq. Coverage [%]:**	17.6
**MW [kDa] / pI:**	7.7 / 10.0	**No. of Peptides:**	1

Peptide Match

**Sequence:**	-.MDQVMQFVEPSR.Q	**Score:**	19.6
**Modifications:**	Oxidation: 1, 5		

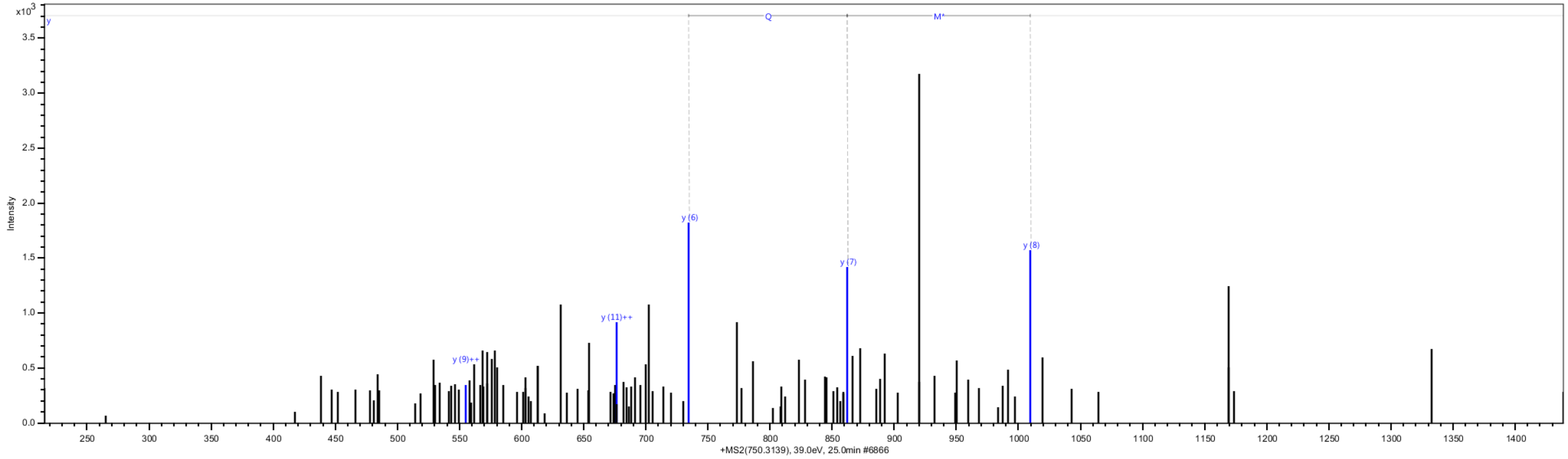

RLGPB_HUMAN

**Accession:**	RLGPB_HUMAN	**Score:**	20.3
**Database:**	SwissProt	**Seq. Coverage [%]:**	0.7
**MW [kDa] / pI:**	166.7 / 6.3	**No. of Peptides:**	1

Peptide Match

**Sequence:**	R.AVTVNKATMK.T	**Score:**	20.2
**Modifications:**	Oxidation: 9		

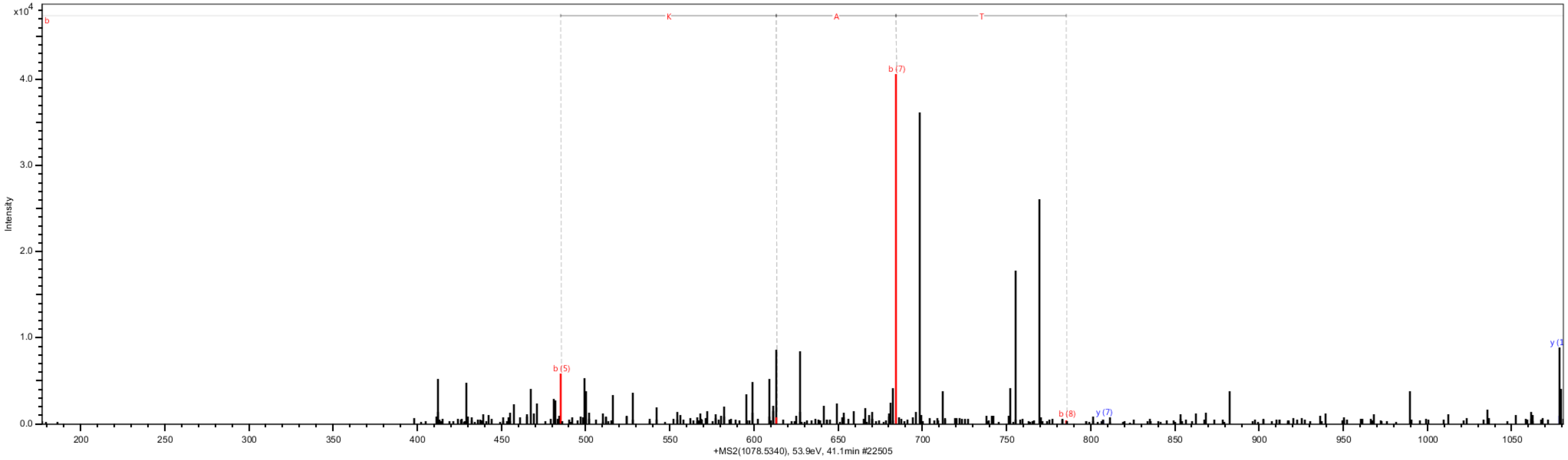

KR87P_HUMAN

Accession: KR87P_HUMAN
Database: SwissProt
MW [kDa] / pI: 29.1 / 5.6
Score: 20.2
Seq. Coverage [%]: 4.3
No. of Peptides: 1

Peptide Match

Sequence: K.AQYDDIATRSR.A
Score: 20.2
Modifications:

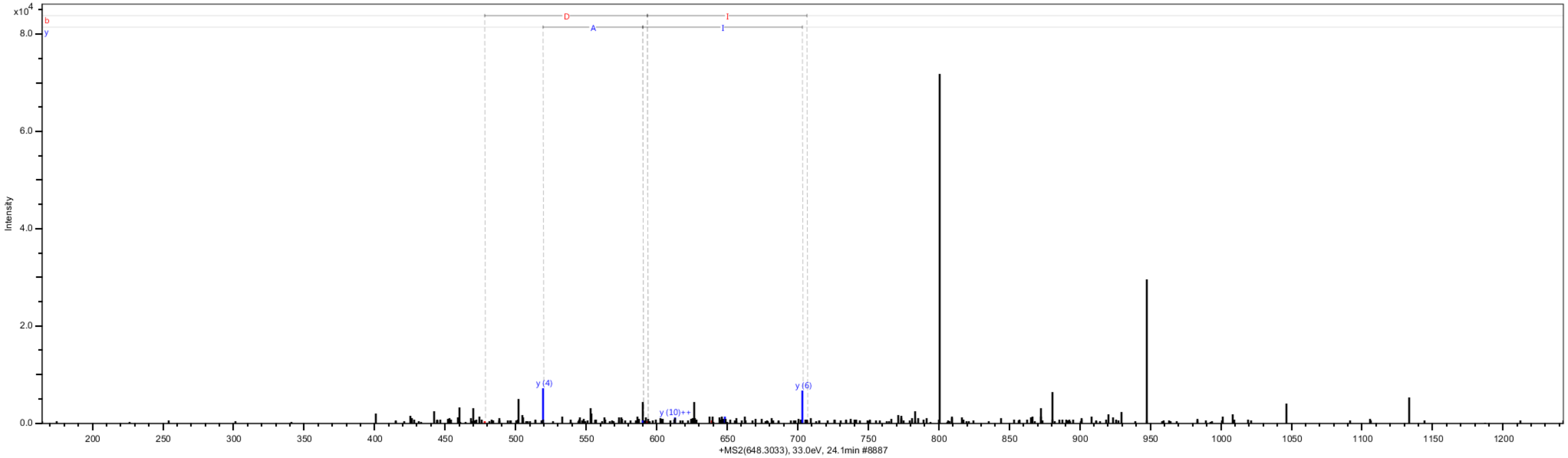

LCORL_HUMAN

Accession: LCORL_HUMAN
Database: SwissProt
MW [kDa] / pI: 66.9 / 8.2

Score: 20.3
Seq. Coverage [%]: 1.5
No. of Peptides: 1

Peptide Match

Sequence: R.QFAIEYISK.S
Score: 20.3
Modifications:

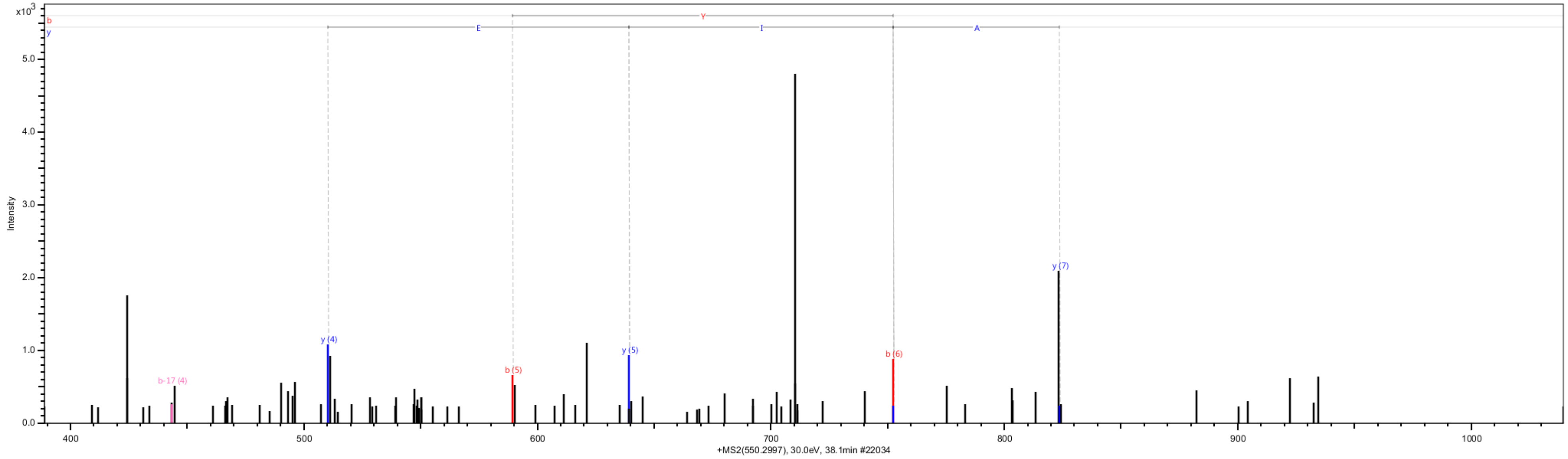

LIMK1_HUMAN

Accession: LIMK1_HUMAN
Database: SwissProt
MW [kDa] / pI: 72.5 / 6.5
Score: 20.3
Seq. Coverage [%]: 3.1
No. of Peptides: 1

Peptide Match

Sequence: R.SCSIDRSPGAGSLGSPASQR.K
Score: 20.3
Modifications: Carbamidomethyl: 2

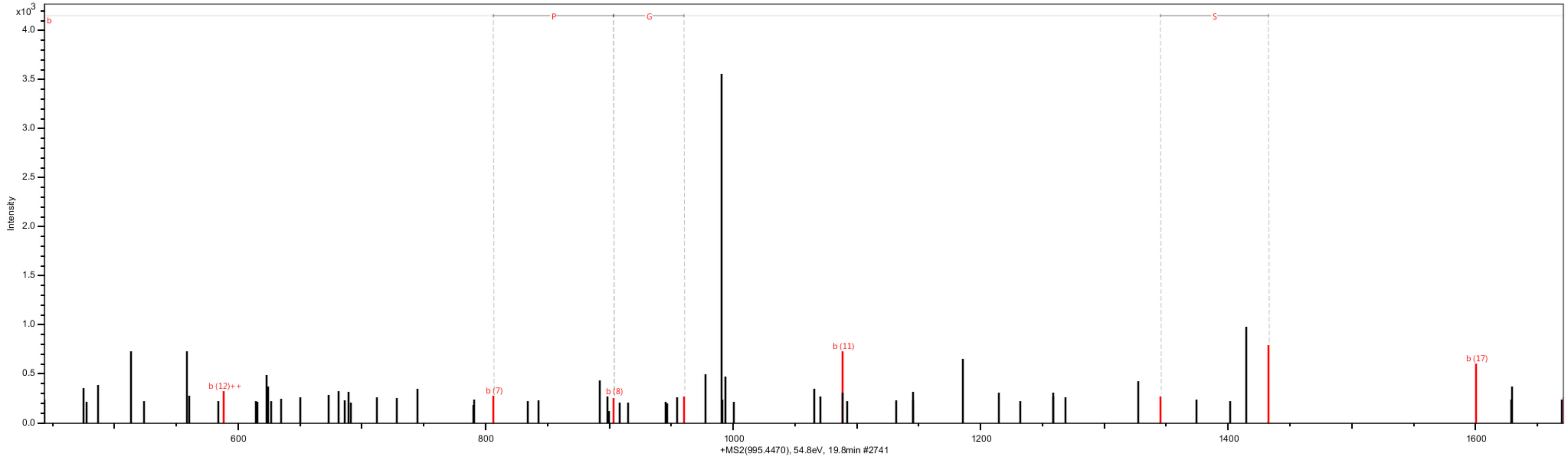

TLK1_HUMAN

Accession: TLK1_HUMAN
Database: SwissProt
MW [kDa] / pI: 86.6 / 8.9
Score: 20.1
Seq. Coverage [%]: 1.0
No. of Peptides: 1

Peptide Match

Sequence: K.ESETPEKK.Q
Score: 20.1
Modifications:

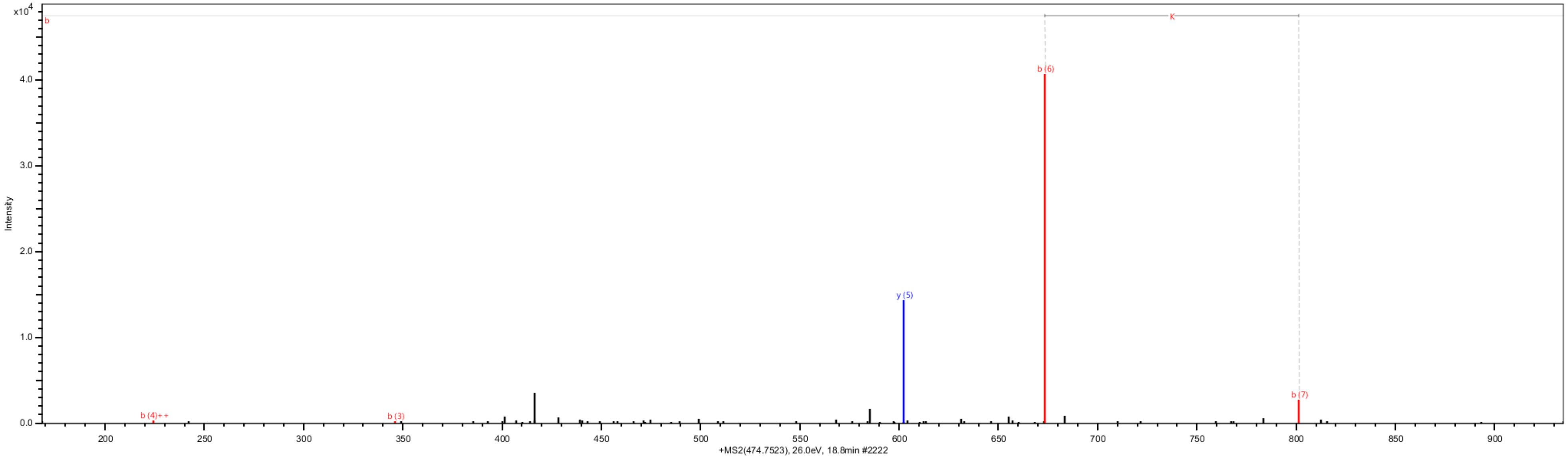

CLMP_HUMAN

**Accession:**	CLMP_HUMAN	**Score:**	20.0
**Database:**	SwissProt	**Seq. Coverage [%]:**	3.5
**MW [kDa] / pI:**	41.3 / 8.1	**No. of Peptides:**	1

Peptide Match

**Sequence:**	K.AETTPSMIPSQSR.A	**Score:**	20
**Modifications:**	Oxidation: 7		

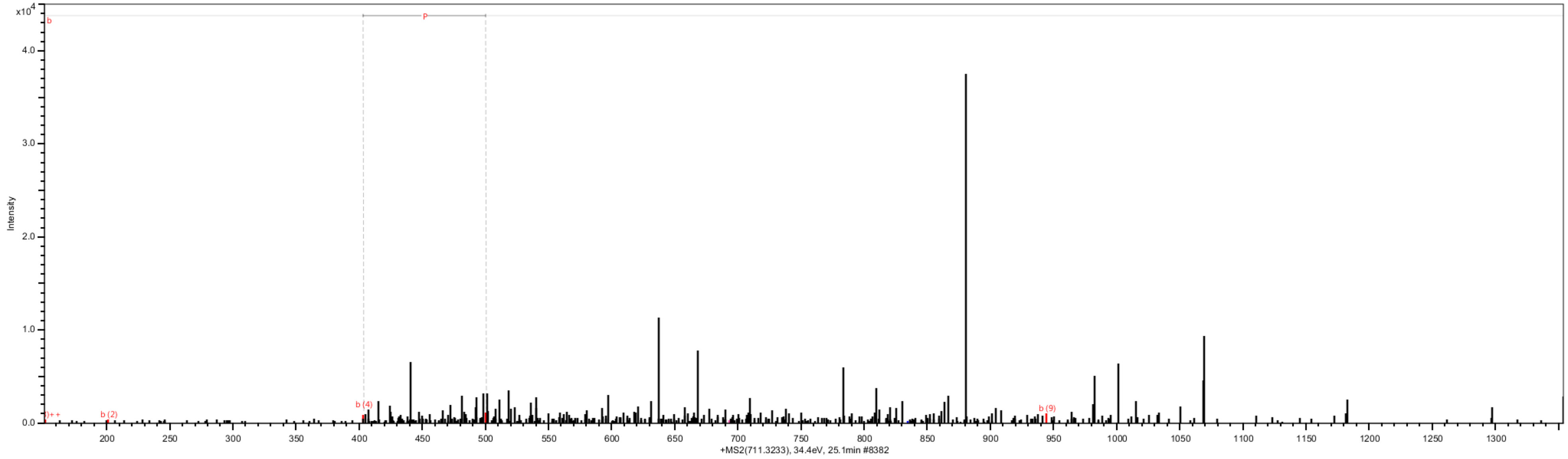

DPOD2_HUMAN

**Accession:**	DPOD2_HUMAN	**Score:**	19.9
**Database:**	SwissProt	**Seq. Coverage [%]:**	1.9
**MW [kDa] / pI:**	51.3 / 5.3	**No. of Peptides:**	1

Peptide Match

**Sequence:**	-.MFSEQAAQR.A	**Score:**	19.9
**Modifications:**	Oxidation: 1		

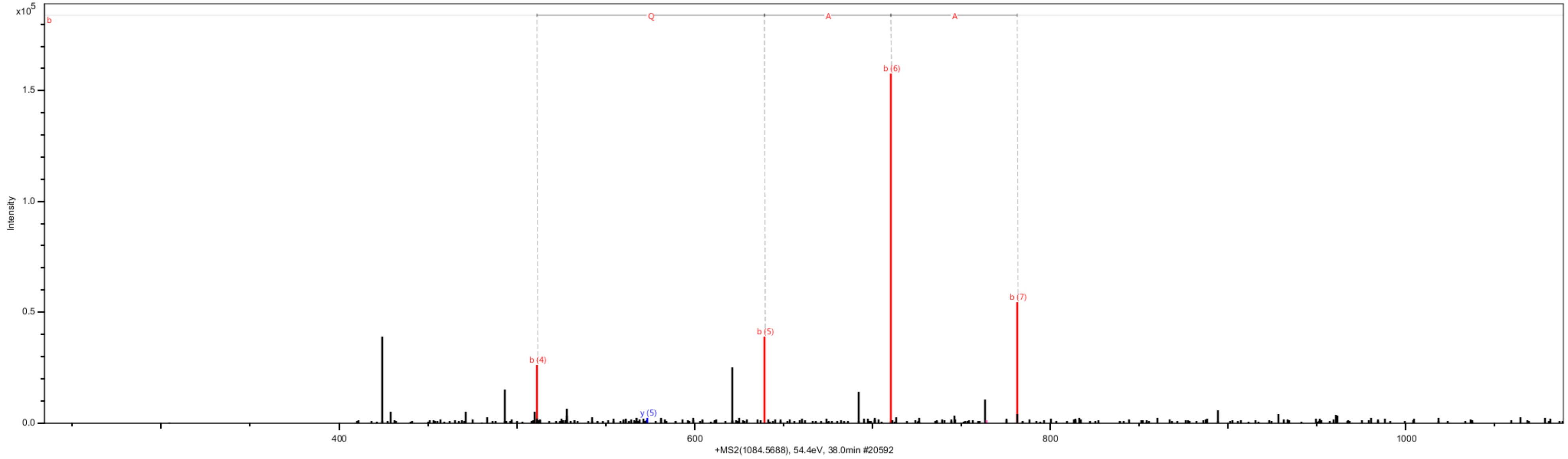

FBLN1_HUMAN

Accession: FBLN1_HUMAN
Database: SwissProt
MW [kDa] / pI: 77.2 / 5.1
Score: 19.9
Seq. Coverage [%]: 1.4
No. of Peptides: 1

Peptide Match

Sequence: K.TGYYFDGISR.M
Score: 19.9
Modifications:

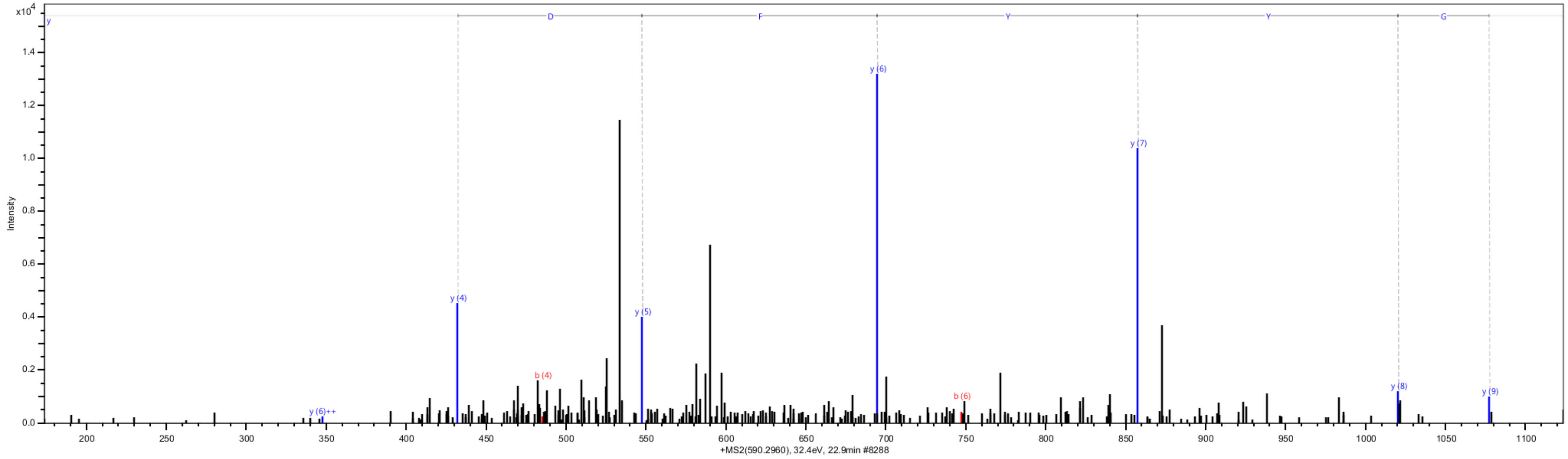

NUD15_HUMAN

Accession: NUD15_HUMAN
Database: SwissProt
MW [kDa] / pI: 18.6 / 5.7
Score: 20.0
Seq. Coverage [%]: 5.5
No. of Peptides: 1

Peptide Match

Sequence: M.TASAQPRGR.R
Score: 20
Modifications:

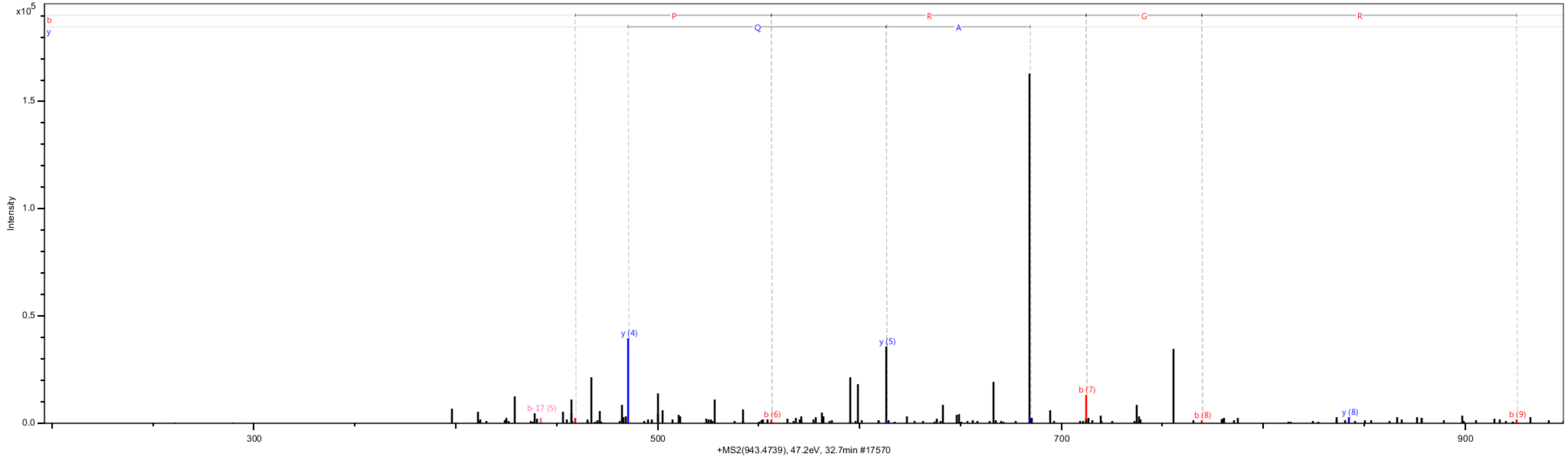

TGT_HUMAN

Accession: TGT_HUMAN
Database: SwissProt
MW [kDa] / pI: 44.0 / 6.8
Score: 20.0
Seq. Coverage [%]: 2.2
No. of Peptides: 1

Peptide Match

Sequence: R.ATCLEEMTK.R
Score: 20
Modifications: Carbamidomethyl: 3; Oxidation: 7

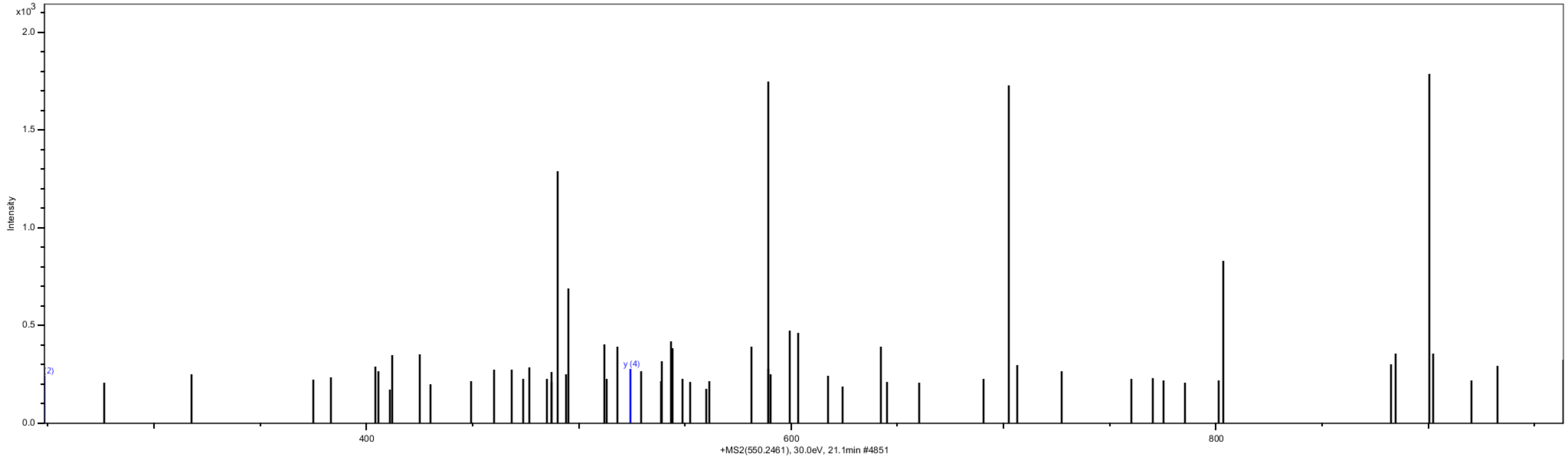

XPO1_HUMAN

Accession: XPO1_HUMAN
Database: SwissProt
MW [kDa] / pI: 123.3 / 5.7
Score: 20.6
Seq. Coverage [%]: 0.8
No. of Peptides: 1

Peptide Match

Sequence: R.QLYLPMLFK.V
Score: 20.6
Modifications: Oxidation: 6

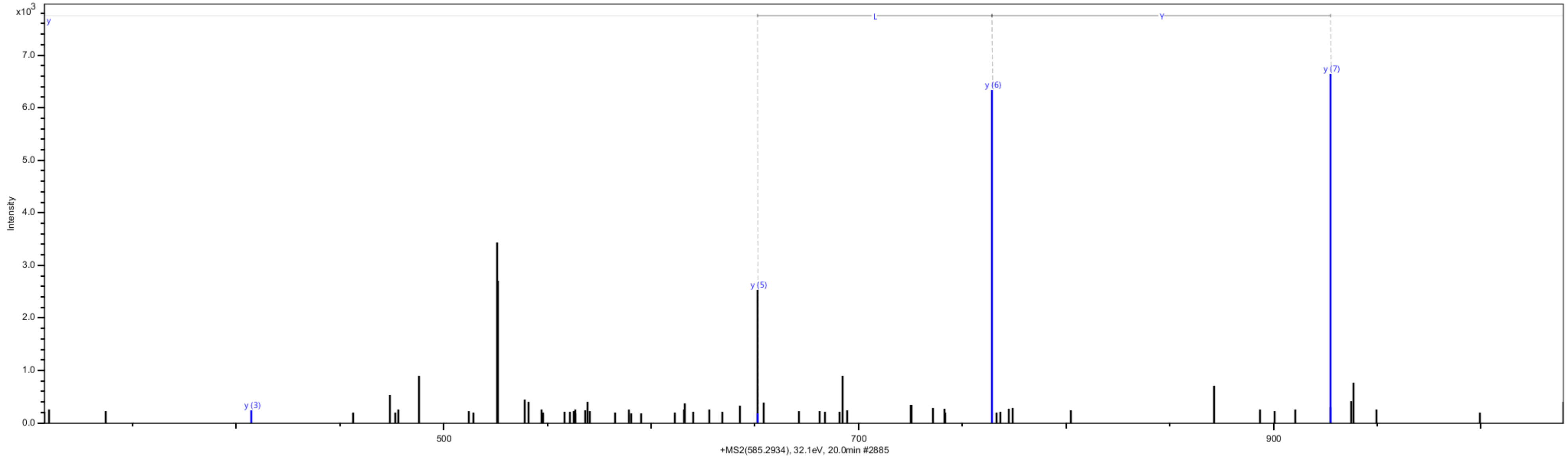

MRM2_HUMAN

Accession: MRM2_HUMAN
Database: SwissProt
MW [kDa] / pI: 27.4 / 9.6
Score: 20.6
Seq. Coverage [%]: 2.8
No. of Peptides: 1

Peptide Match

Sequence: K.LLEVNER.H
Score: 20.6
Modifications:

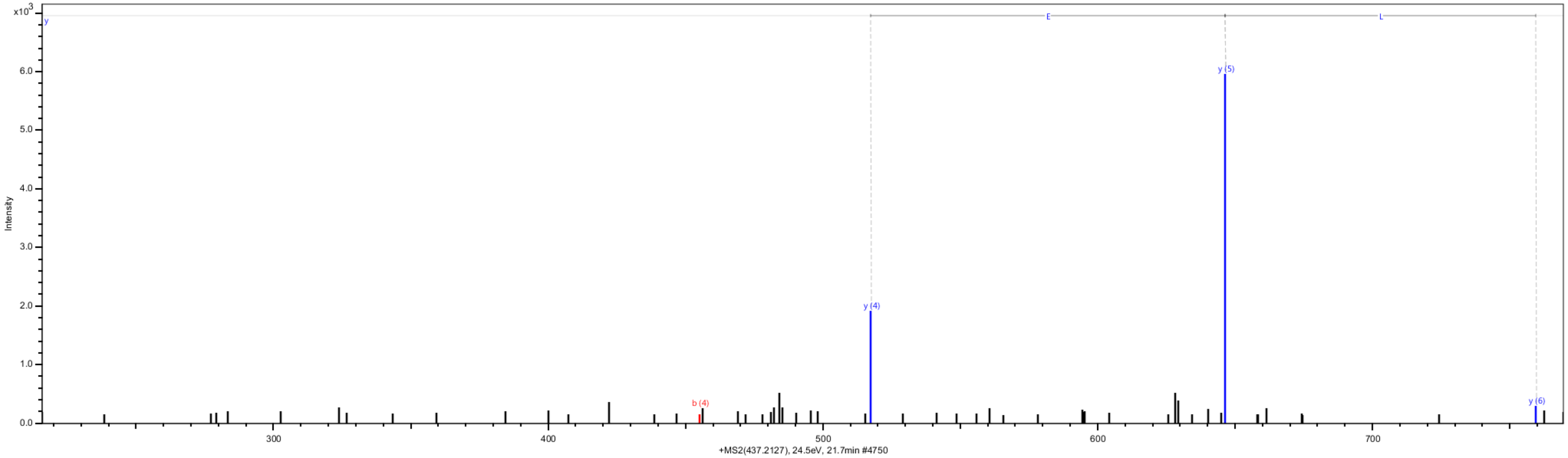

RS4X_HUMAN

**Accession:**	RS4X_HUMAN	**Score:**	20.7
**Database:**	SwissProt	**Seq. Coverage [%]:**	3.8
**MW [kDa] / pI:**	29.6 / 10.2	**No. of Peptides:**	1

Peptide Match

**Sequence:**	R.ECLPLIIFLR.N	**Score:**	20.7
**Modifications:**	Carbamidomethyl: 2		

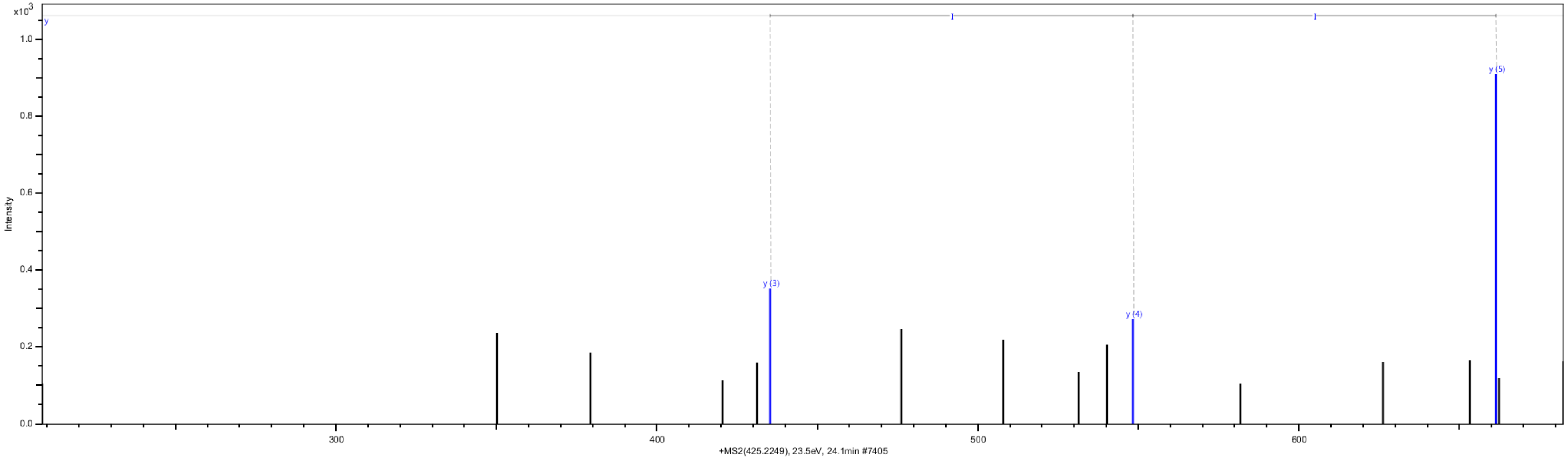

ZN197_HUMAN

Accession: ZN197_HUMAN
Database: SwissProt
MW [kDa] / pI: 118.8 / 8.9
Score: 20.7
Seq. Coverage [%]: 0.7
No. of Peptides: 1

Peptide Match

Sequence: K.DQDTLQK.V
Score: 20.7
Modifications:

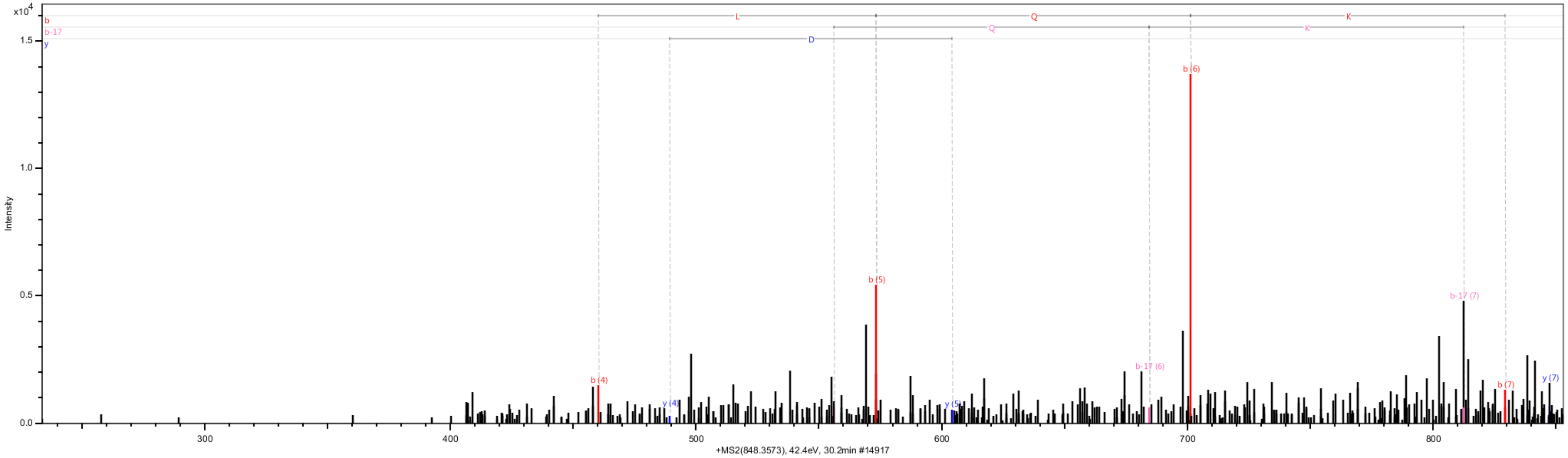

ZN184_HUMAN

Accession: ZN184_HUMAN
Database: SwissProt
MW [kDa] / pI: 86.1 / 8.4
Score: 20.5
Seq. Coverage [%]: 1.2
No. of Peptides: 1

Peptide Match

Sequence: R.SSSLAKHER.I
Score: 20.5
Modifications:

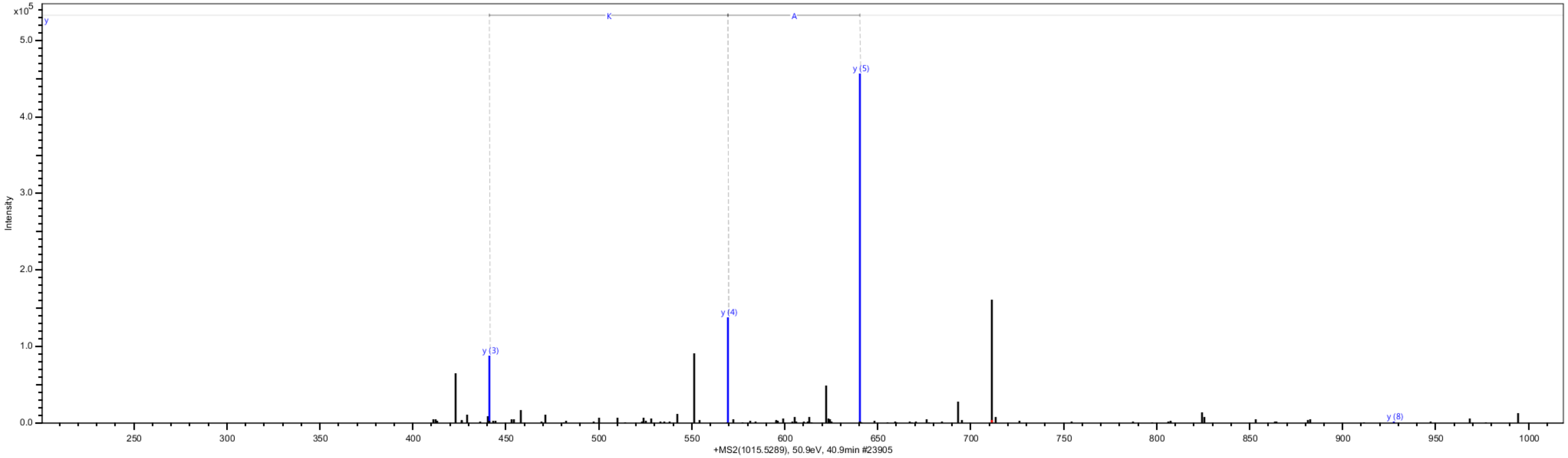

DCR1A_HUMAN

Accession: DCR1A_HUMAN
Database: SwissProt
MW [kDa] / pI: 116.3 / 8.2
Score: 20.5
Seq. Coverage [%]: 1.3
No. of Peptides: 1

Peptide Match

Sequence: -.MLEDISEEDIWEYK.S
Score: 20.5
Modifications:

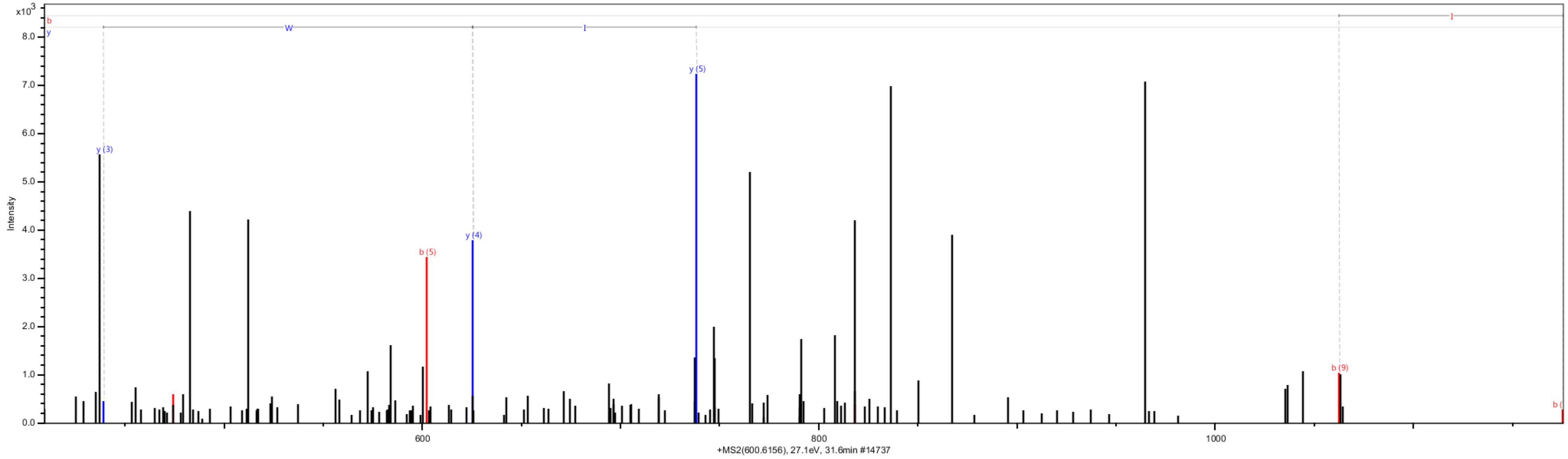

TM246_HUMAN

Accession: TM246_HUMAN
Database: SwissProt
MW [kDa] / pI: 46.6 / 7.3
Score: 20.5
Seq. Coverage [%]: 2.2
No. of Peptides: 1

Peptide Match

Sequence: K.DMALYSLLR.A
Modifications: Oxidation: 2
Score: 20.5

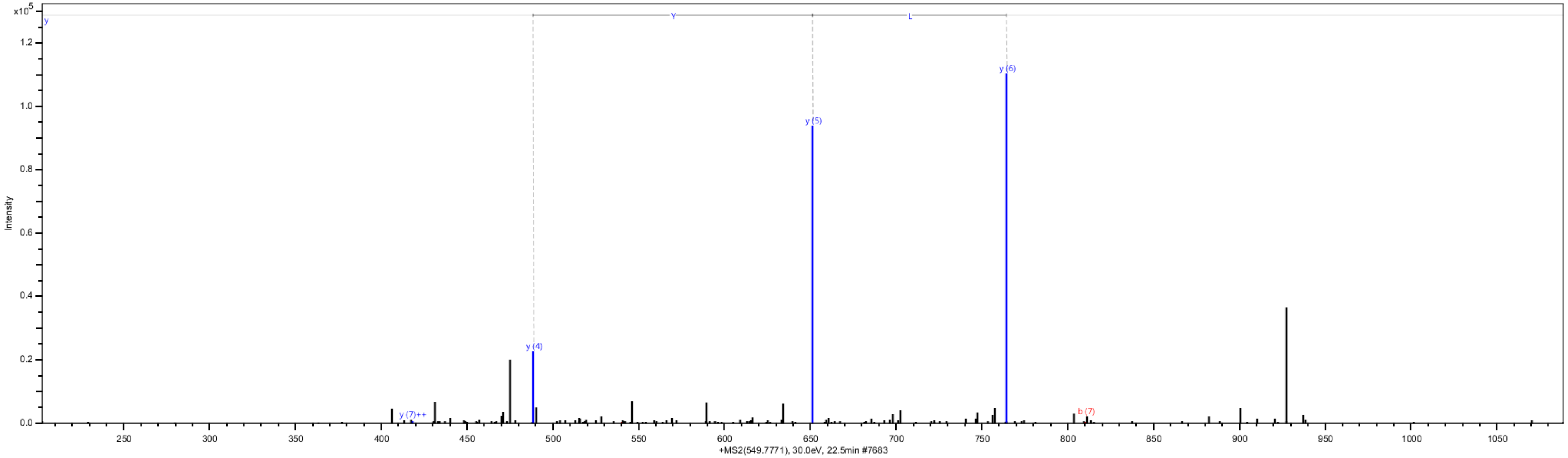

UT14C_HUMAN

Accession: UT14C_HUMAN
Database: SwissProt
MW [kDa] / pI: 87.1 / 6.7
Score: 20.6
Seq. Coverage [%]: 1.0
No. of Peptides: 1

Peptide Match

Sequence: K.MHRAELQR.A
Score: 20.6
Modifications:

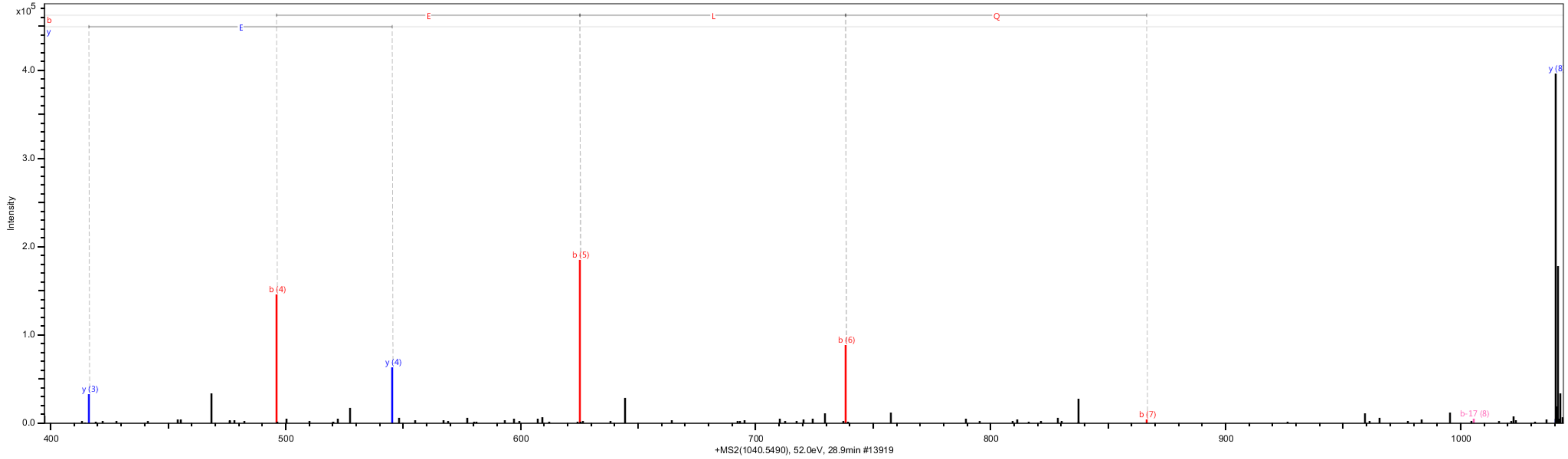

MEF2B_HUMAN

Accession: MEF2B_HUMAN
Database: SwissProt
MW [kDa] / pI: 38.6 / 9.6
Score: 20.3
Seq. Coverage [%]: 2.5
No. of Peptides: 1

Peptide Match

Sequence: R.SPGGTSPER.S
Score: 20.3
Modifications:

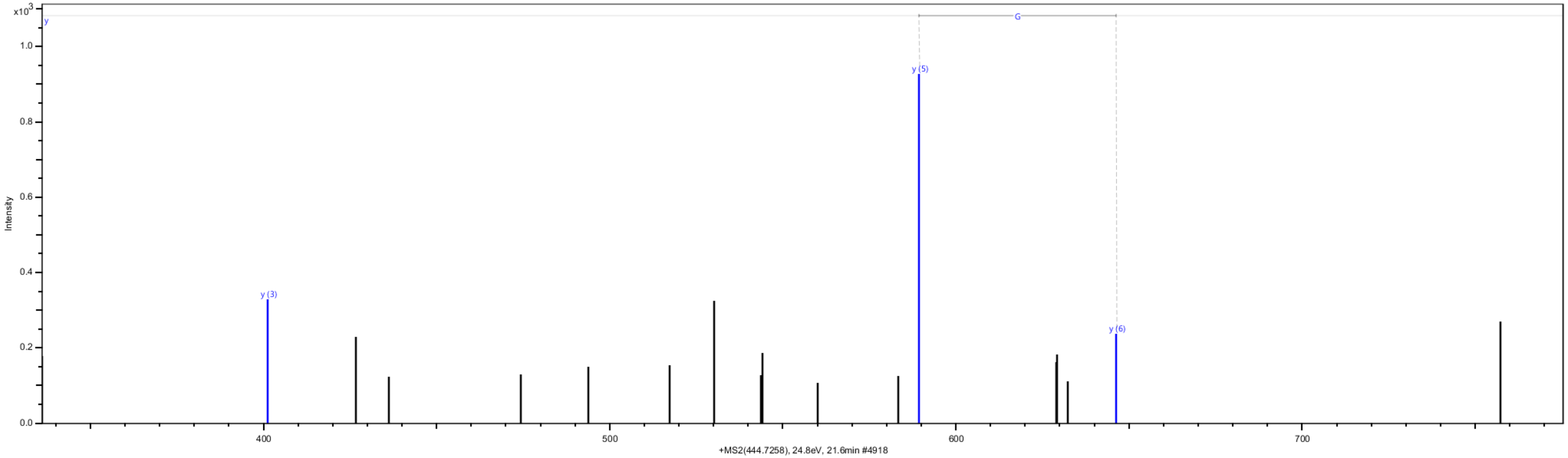

IMPG1_HUMAN

Accession: IMPG1_HUMAN
Database: SwissProt
MW [kDa] / pI: 89.3 / 4.8
Score: 20.3
Seq. Coverage [%]: 1.0
No. of Peptides: 1

Peptide Match

Sequence: R.KDEISAEK.T
Score: 20.3
Modifications:

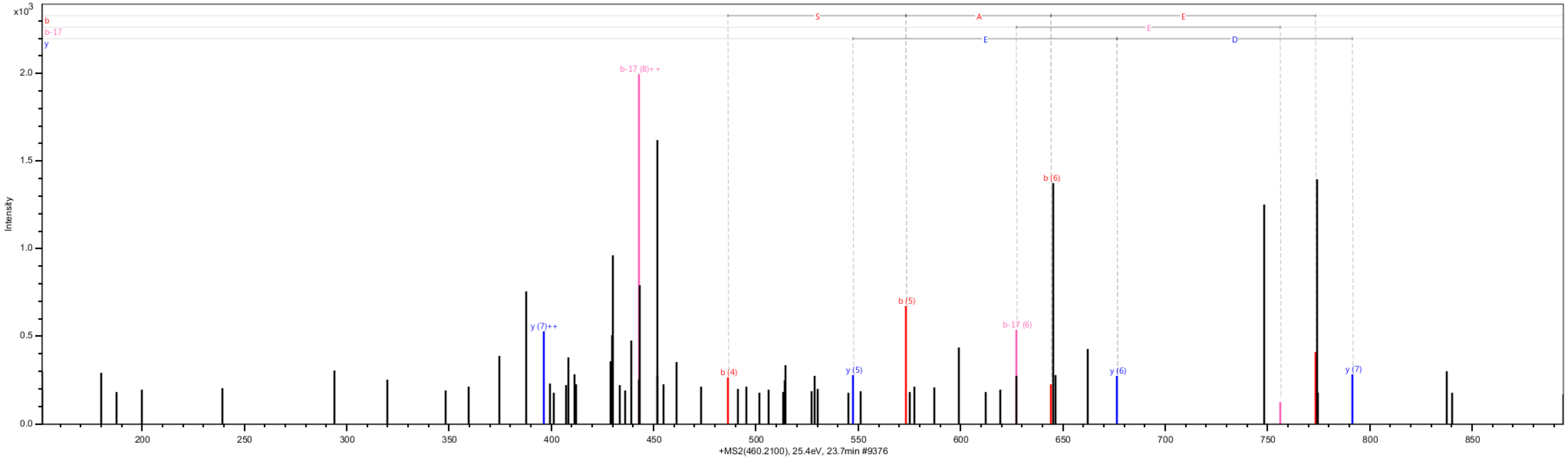

AP1S3_HUMAN

Accession: AP1S3_HUMAN
Database: SwissProt
MW [kDa] / pI: 18.3 / 6.3

Score: 20.3
Seq. Coverage [%]: 5.8
No. of Peptides: 1

Peptide Match

Sequence: R.EIVQIILSR.G
Score: 20.3
Modifications:

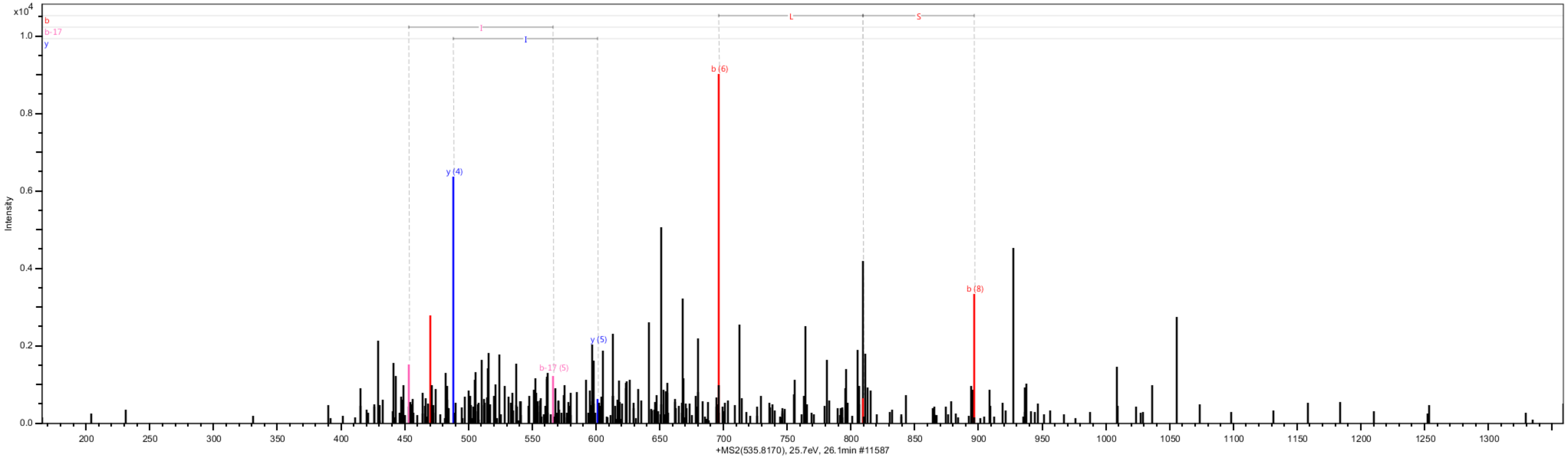

SNP29_HUMAN

**Accession:**	SNP29_HUMAN	**Score:**	20.3
**Database:**	SwissProt	**Seq. Coverage [%]:**	4.7
**MW [kDa] / pI:**	29.0 / 5.6	**No. of Peptides:**	1

Peptide Match

**Sequence:**	K.EAISTSKEQEAK.Y	**Score:**	20.3
**Modifications:**			

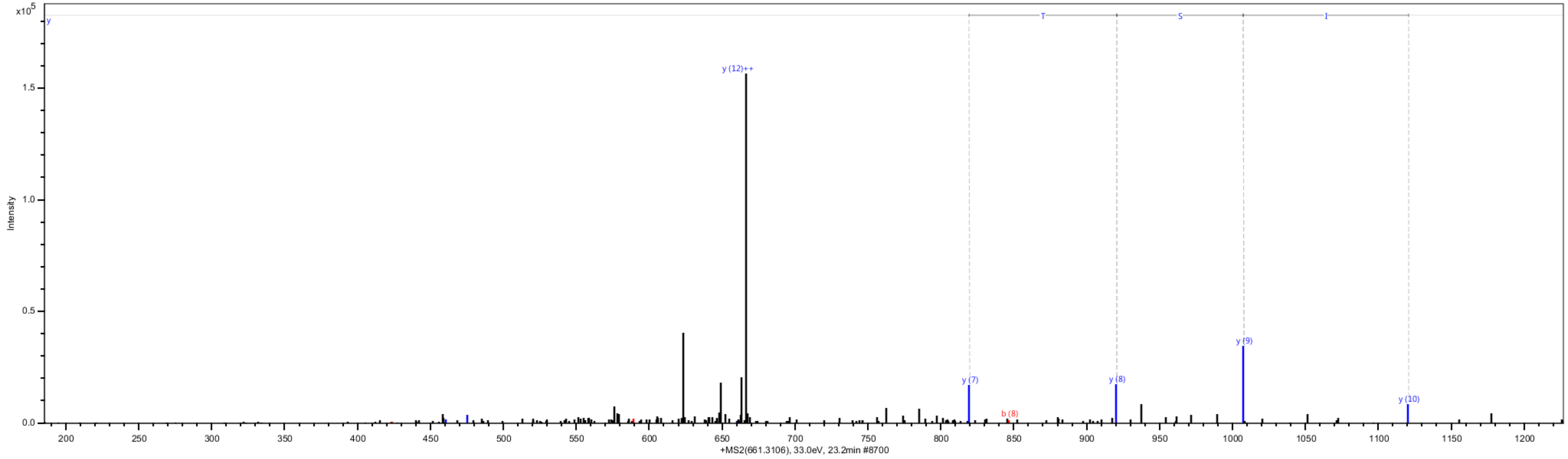

LAMA3_HUMAN

**Accession:**	LAMA3_HUMAN	**Score:**	33.3
**Database:**	SwissProt	**Seq. Coverage [%]:**	0.2
**MW [kDa] / pI:**	366.4 / 7.0	**No. of Peptides:**	1

Peptide Match

**Sequence:**	K.QLEEIKR.N	**Score:**	18.2
**Modifications:**			

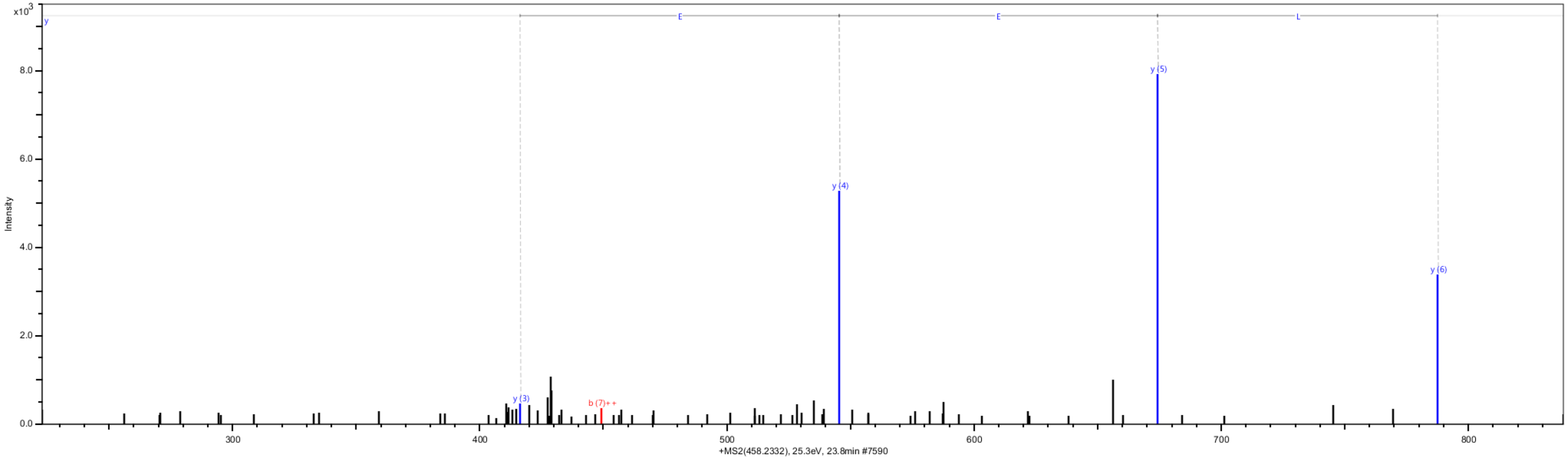

ASGR2_HUMAN

Accession: ASGR2_HUMAN
Database: SwissProt
MW [kDa] / pI: 35.1 / 5.8
Score: 33.3
Seq. Coverage [%]: 2.3
No. of Peptides: 1

Peptide Match

Sequence: R.RGNPFLK.G
Score: 16.2
Modifications:

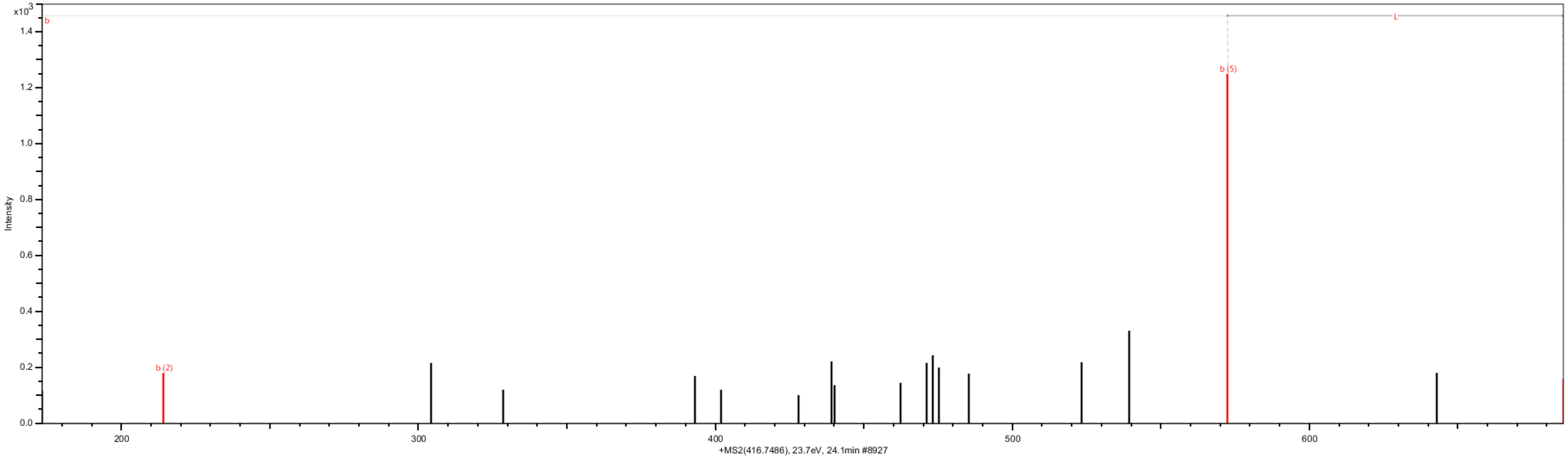

FOG2_HUMAN

Accession: FOG2_HUMAN
Database: SwissProt
MW [kDa] / pI: 128.1 / 6.0
Score: 33.1
Seq. Coverage [%]: 1.0
No. of Peptides: 1

Peptide Match

Sequence: K.NRGMVIVNGGLK.Q
Score: 33.1
Modifications: Oxidation: 4

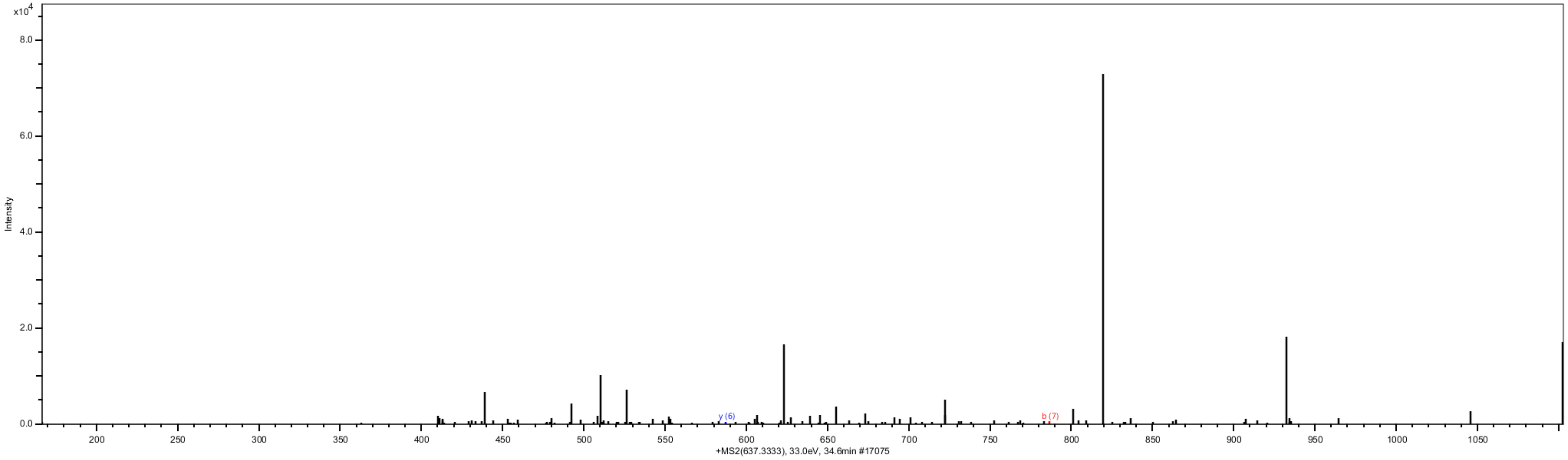

LV147_HUMAN

Accession: LV147_HUMAN
Database: SwissProt
MW [kDa] / pI: 12.3 / 5.6
Score: 33.3
Seq. Coverage [%]: 11.1
No. of Peptides: 1

Peptide Match

Sequence: K.SGTSASLAISGLR.S
Score: 33.3
Modifications:

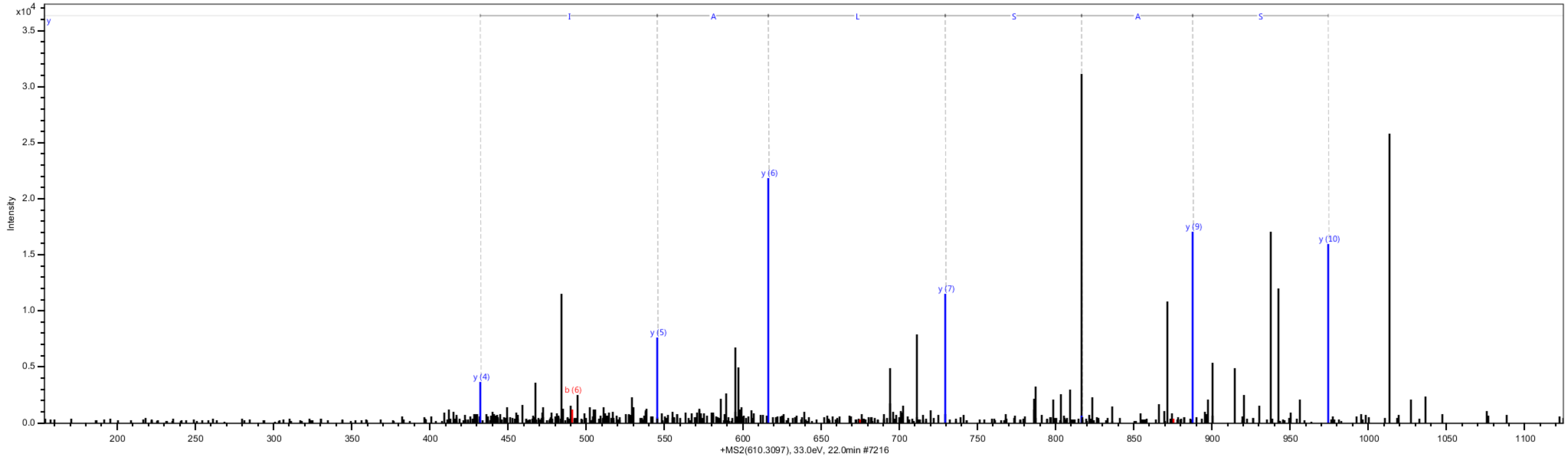

FBN1_HUMAN

Accession: FBN1_HUMAN
Database: SwissProt
MW [kDa] / pI: 312.1 / 4.8
Score: 33.7
Seq. Coverage [%]: 0.4
No. of Peptides: 1

Peptide Match

Sequence: R.SCKDLDECATK.Q
Score: 33.7
Modifications: Carbamidomethyl: 2, 8

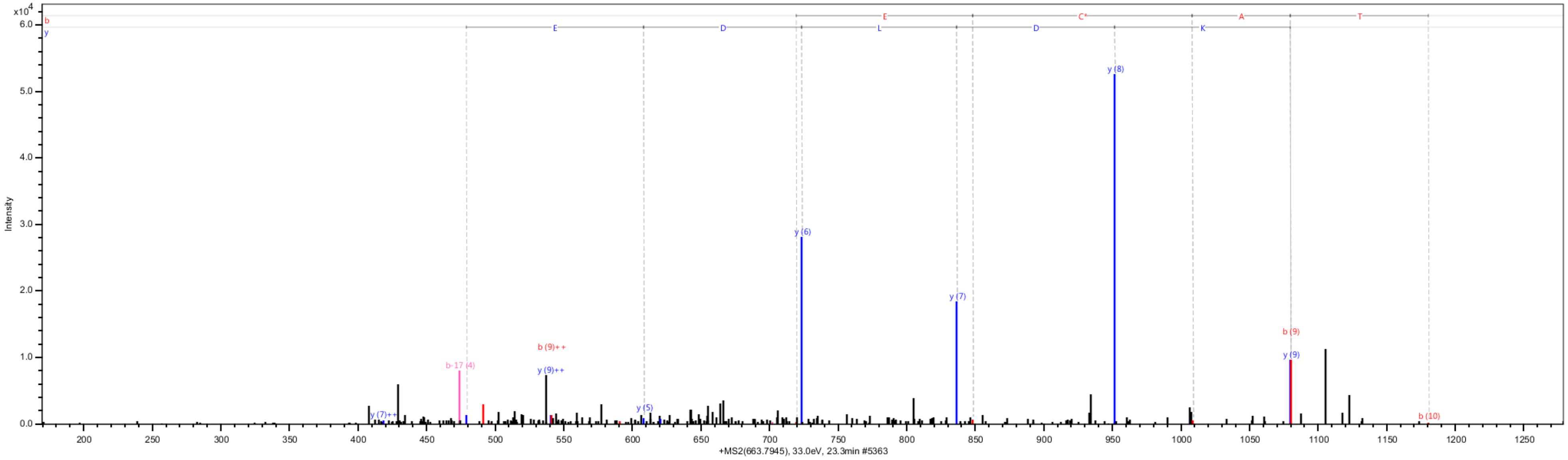

PHLB2_HUMAN

Accession: PHLB2_HUMAN
Database: SwissProt
MW [kDa] / pI: 142.1 / 7.1
Score: 33.8
Seq. Coverage [%]: 0.9
No. of Peptides: 1

Peptide Match

Sequence: R.SQELAAMEETR.I
Score: 15.9
Modifications: Oxidation: 7

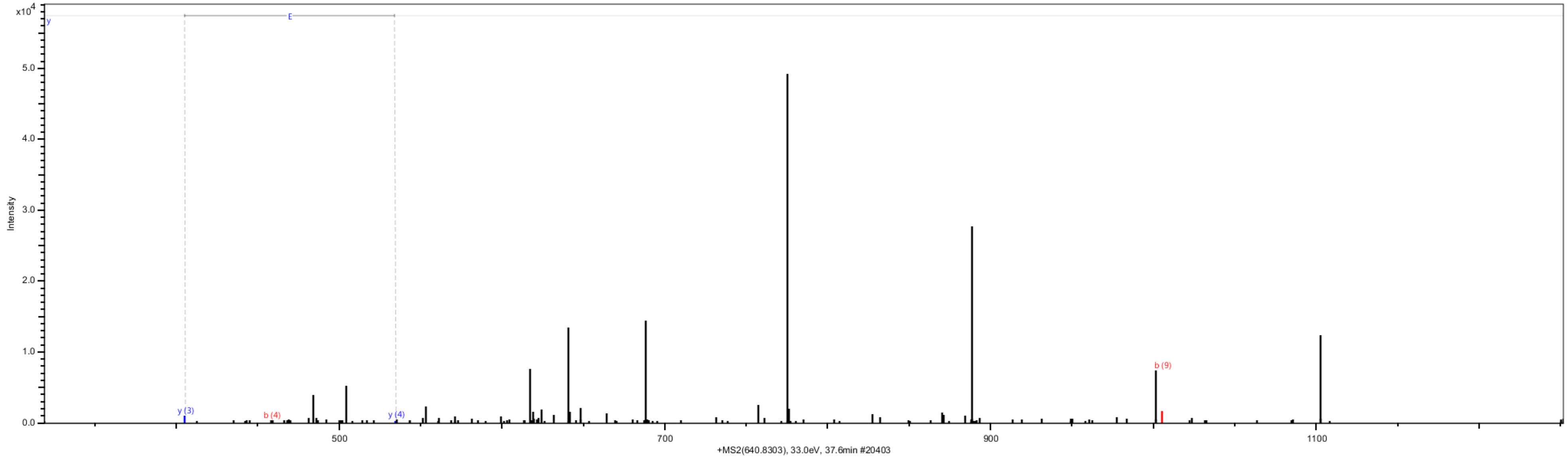

DCAF1_HUMAN

Accession: DCAF1_HUMAN
Database: SwissProt
MW [kDa] / pI: 168.9 / 4.9
Score: 33.4
Seq. Coverage [%]: 0.7
No. of Peptides: 1

Peptide Match

Sequence: R.ERPSPCNGRK.I
Score: 33.4
Modifications: Carbamidomethyl: 6

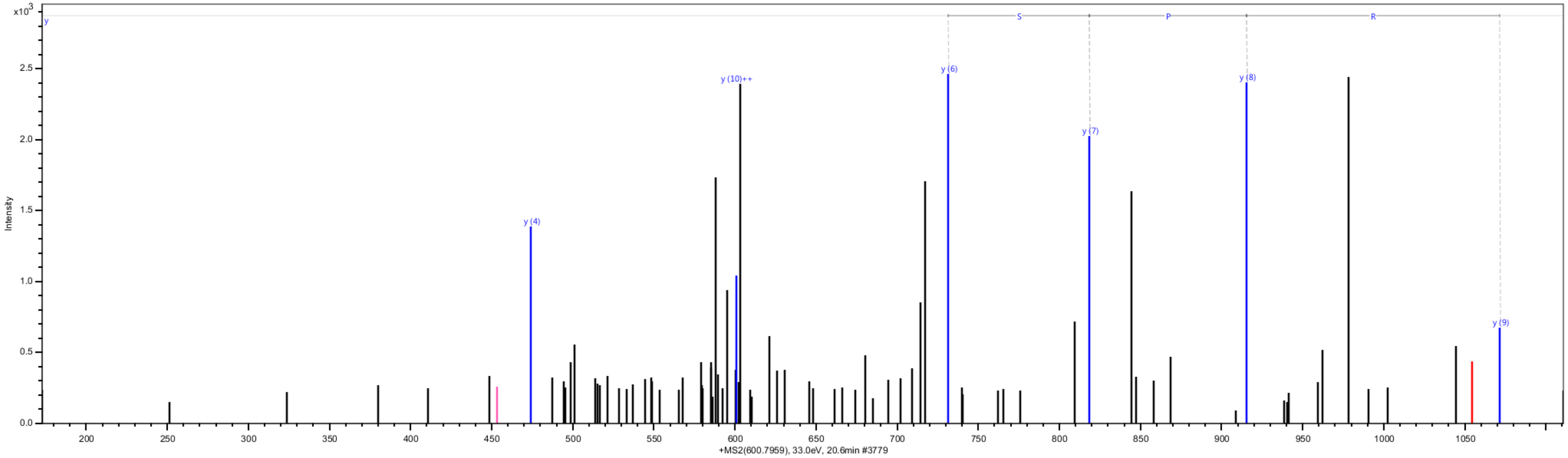

LAMB2_HUMAN

**Accession:**	LAMB2_HUMAN	**Score:**	33.5
**Database:**	SwissProt	**Seq. Coverage [%]:**	0.8
**MW [kDa] / pI:**	195.9 / 6.1	**No. of Peptides:**	1

Peptide Match

**Sequence:**	R.SCDCDPMGSQDGGR.C	**Score:**	16
**Modifications:**	Carbamidomethyl: 2, 4		

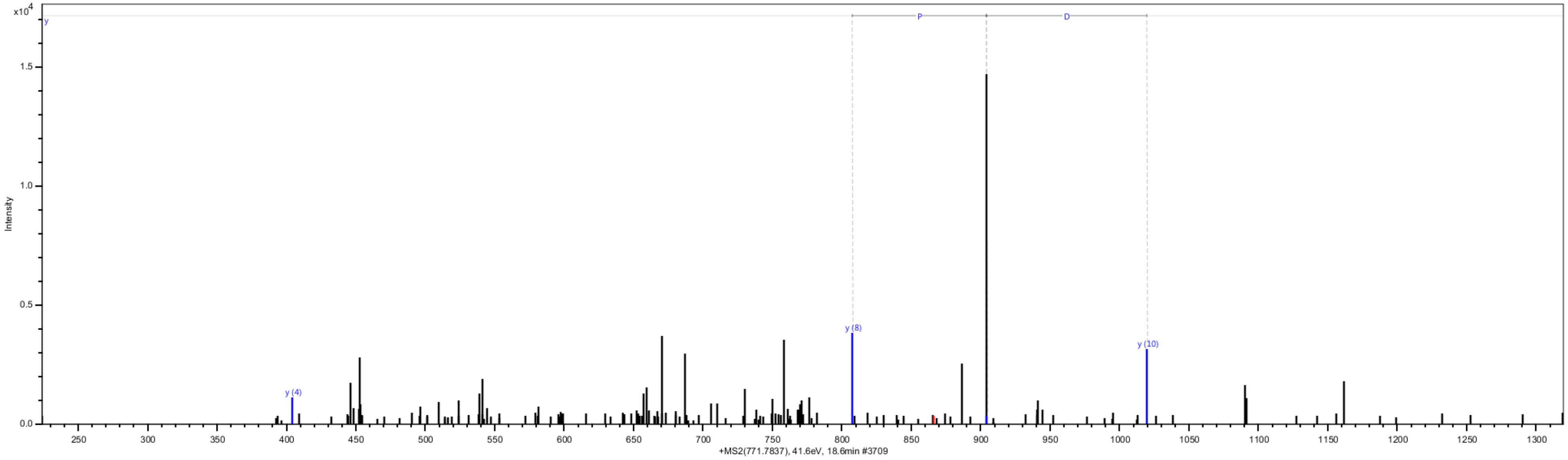

CO2_HUMAN

Accession: CO2_HUMAN
Database: SwissProt
MW [kDa] / pI: 83.2 / 7.2
Score: 33.9
Seq. Coverage [%]: 1.6
No. of Peptides: 1

Peptide Match

Sequence: R.QHLGDVLNFLPL.-
Score: 33.9
Modifications:

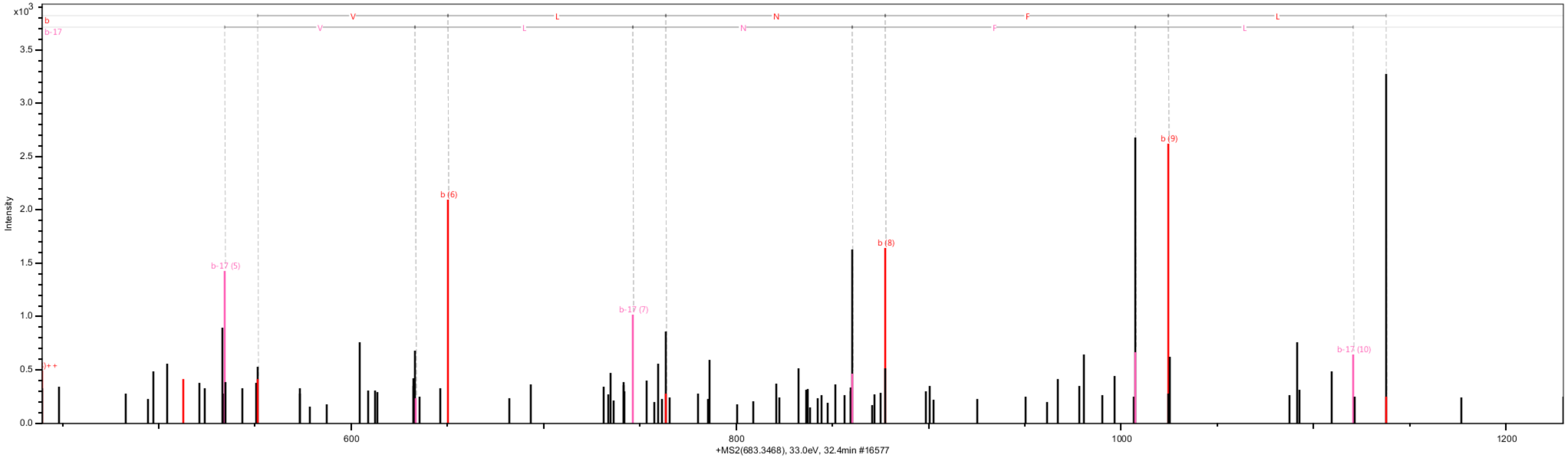

NAV1_HUMAN

Accession: NAV1_HUMAN
Database: SwissProt
MW [kDa] / pI: 202.3 / 8.2
Score: 33.9
Seq. Coverage [%]: 1.1
No. of Peptides: 2

Peptide Match

Sequence: R.SPSKLSHISR.L
Score: 18.3
Modifications:

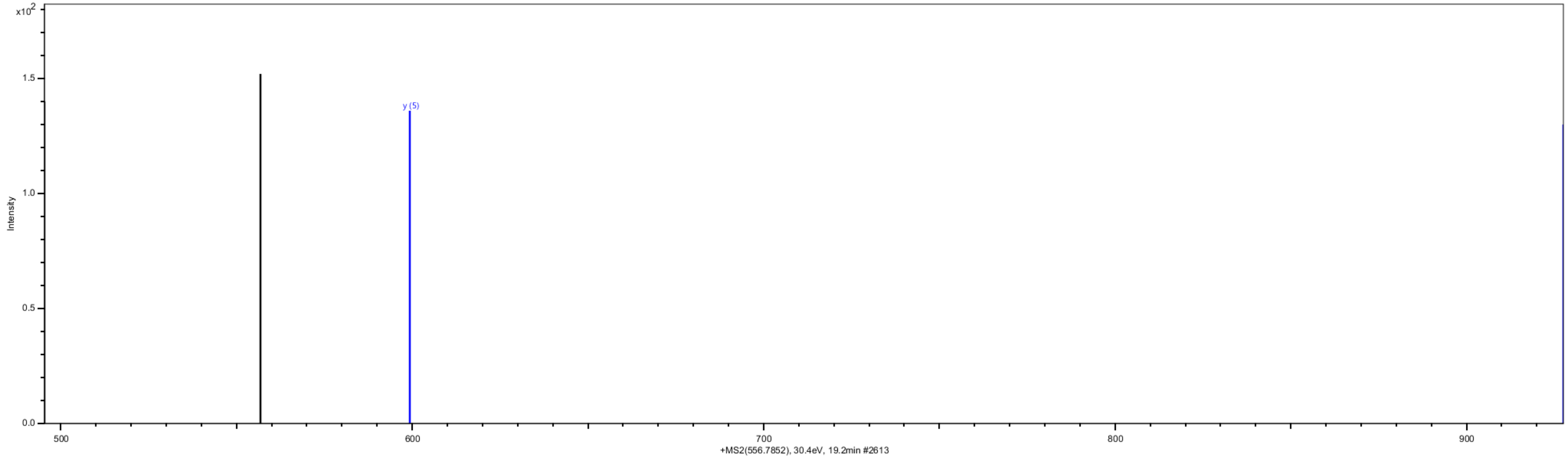

Peptide Match

Sequence: K.QGGLTPSRLK.E
Score: 15.6
Modifications:

Spectrum Report
Combine Search_2020-10-19 07:53:31

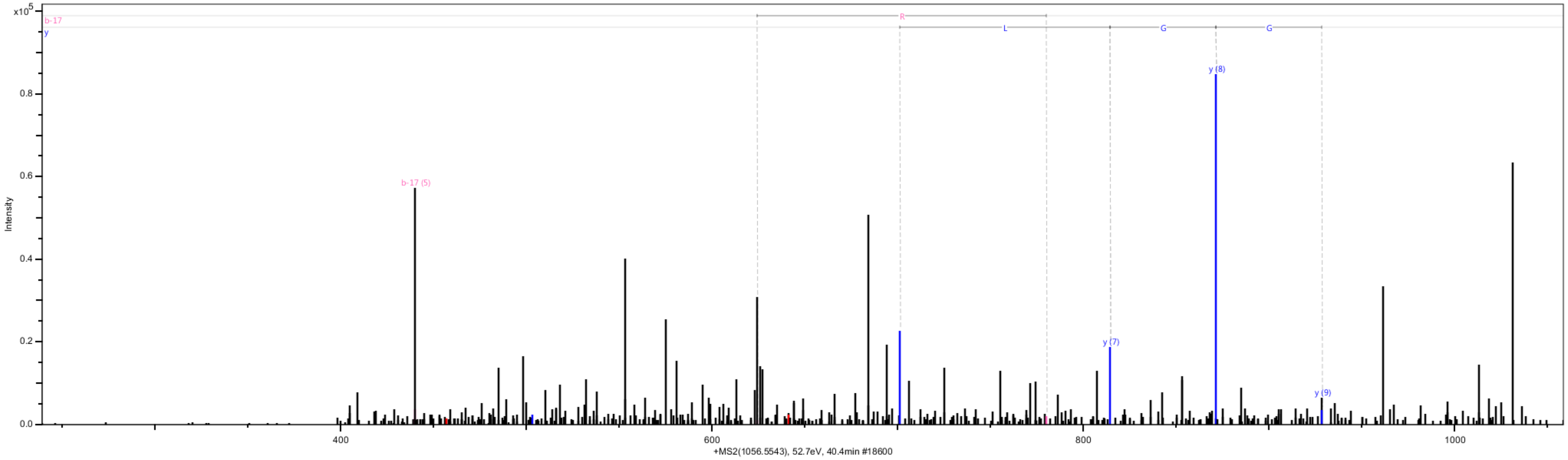

SZT2_HUMAN

**Accession:**	SZT2_HUMAN	**Score:**	33.8
**Database:**	SwissProt	**Seq. Coverage [%]:**	0.4
**MW [kDa] / pI:**	377.8 / 5.9	**No. of Peptides:**	2

Peptide Match

**Sequence:**	R.RVGEICR.E	**Score:**	17.1
**Modifications:**	Carbamidomethyl: 6		

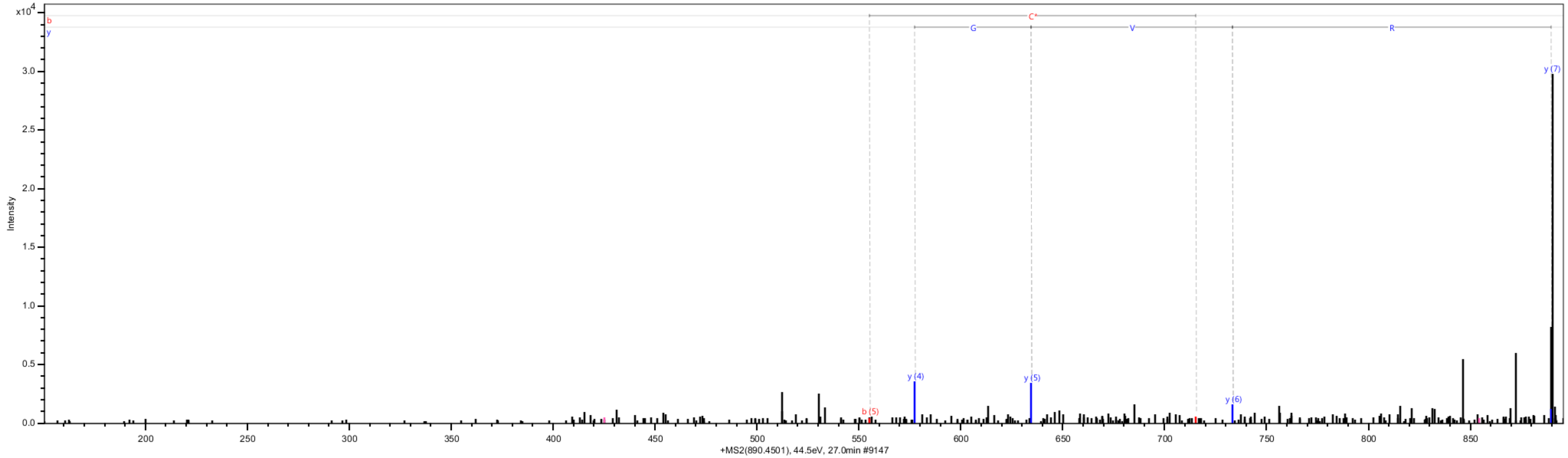

Peptide Match

**Sequence:**	K.NMFVYQER.A	**Score:**	16.7
**Modifications:**	Oxidation: 2		

Spectrum Report
Combine Search_2020-10-19 07:53:31

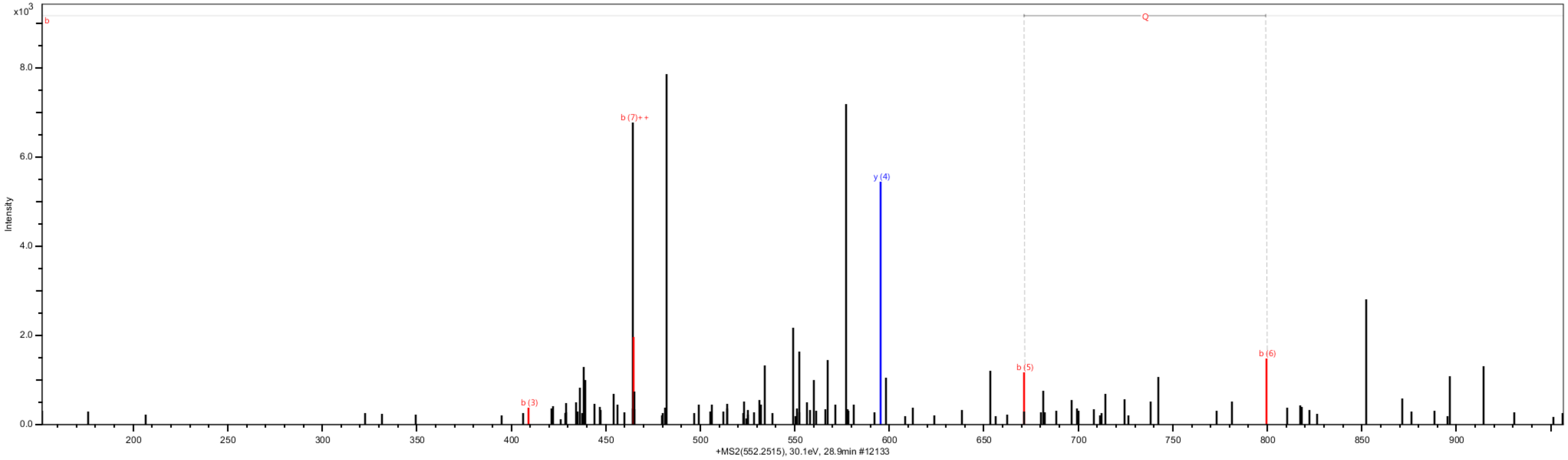

HSPB1_HUMAN

Accession: HSPB1_HUMAN
Database: SwissProt
MW [kDa] / pI: 22.8 / 6.0
Score: 34.2
Seq. Coverage [%]: 4.9
No. of Peptides: 1

Peptide Match

Sequence: R.LFDQAFGLPR.L
Score: 34.2
Modifications:

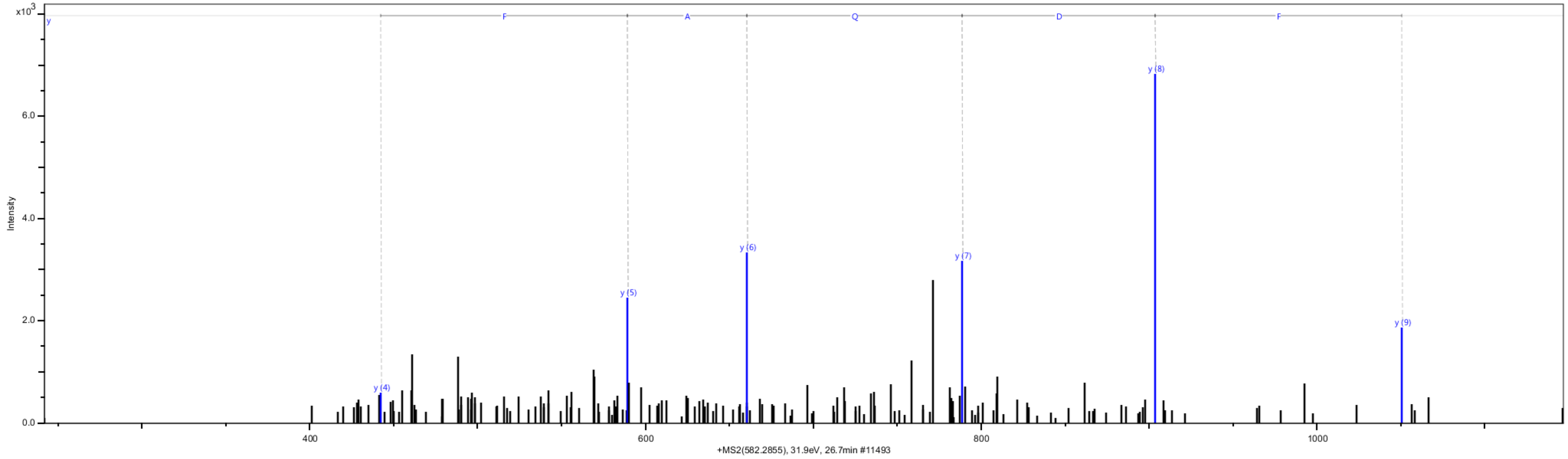

UN13B_HUMAN

**Accession:**	UN13B_HUMAN	**Score:**	34.0
**Database:**	SwissProt	**Seq. Coverage [%]:**	1.3
**MW [kDa] / pI:**	180.6 / 5.7	**No. of Peptides:**	2

Peptide Match

Sequence: R.TDKSAVSGAIR.L **Score:** 15.7

Modifications:

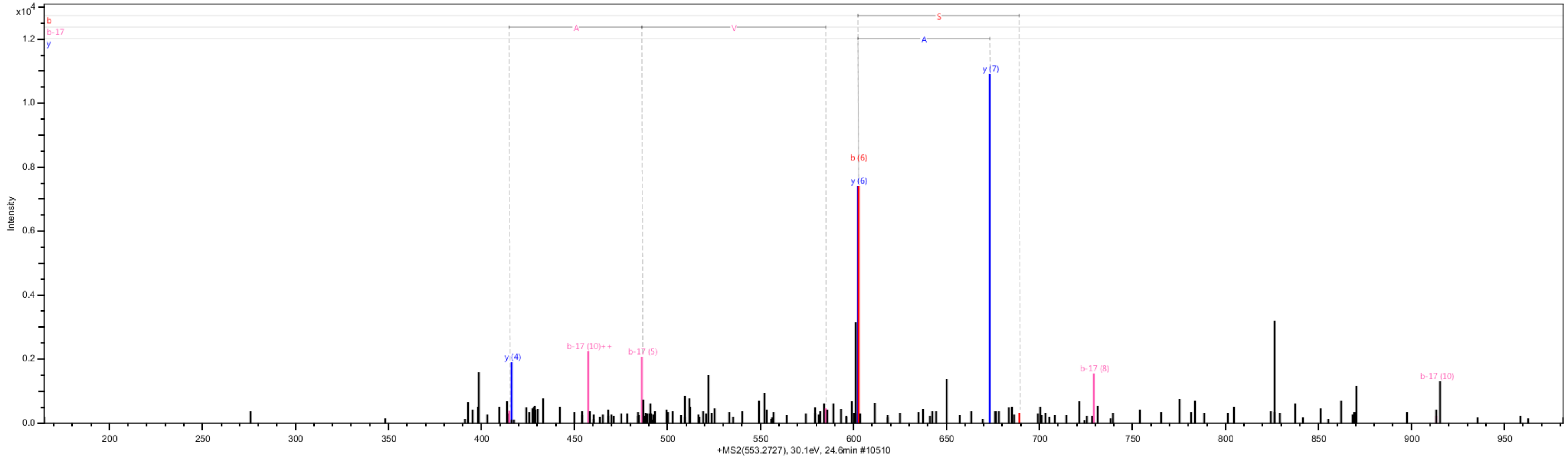

Peptide Match

Sequence: R.GTGNASPDAR.A **Score:** 18.4

Modifications:

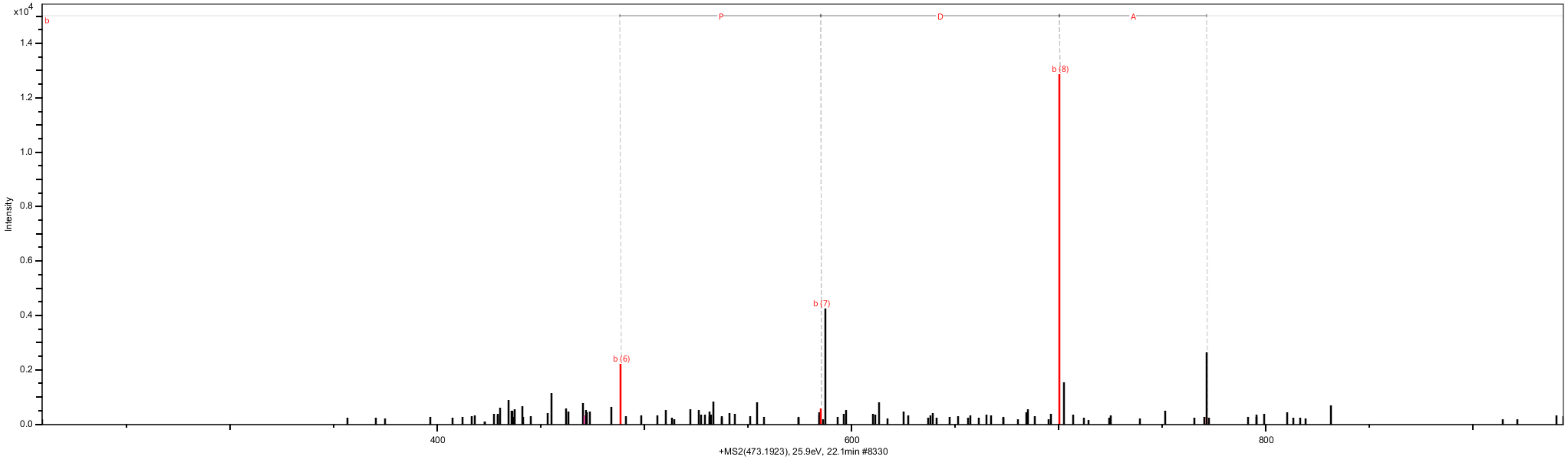
x10^4
1.4
1.2
1.0
0.8
0.6
0.4
0.2
0.0
Intensity
b
P
D
A
b (6)
b (7)
b (8)
400
600
800
+MS2(473.1923), 25.9eV, 22.1min #8330

ZN749_HUMAN

Accession: ZN749_HUMAN
Database: SwissProt
MW [kDa] / pI: 90.3 / 9.0
Score: 34.0
Seq. Coverage [%]: 1.9
No. of Peptides: 2

Peptide Match

Sequence: K.SHLVQHEK.I
Score: 17.2
Modifications:

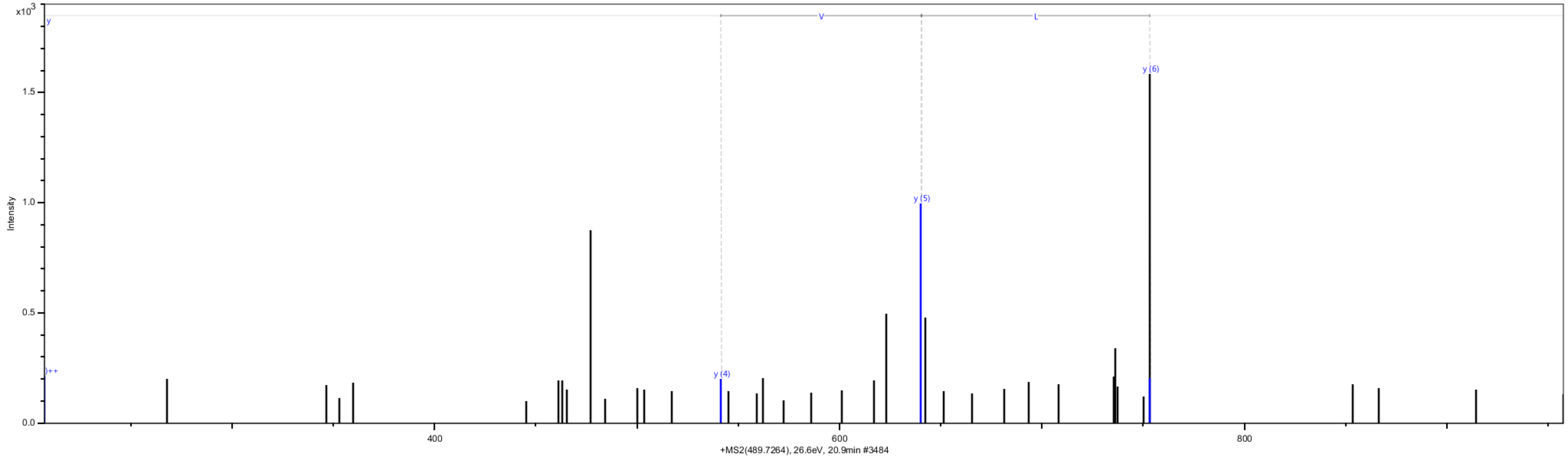

Peptide Match

Sequence: K.YNSSLIK.H
Score: 16.8
Modifications:

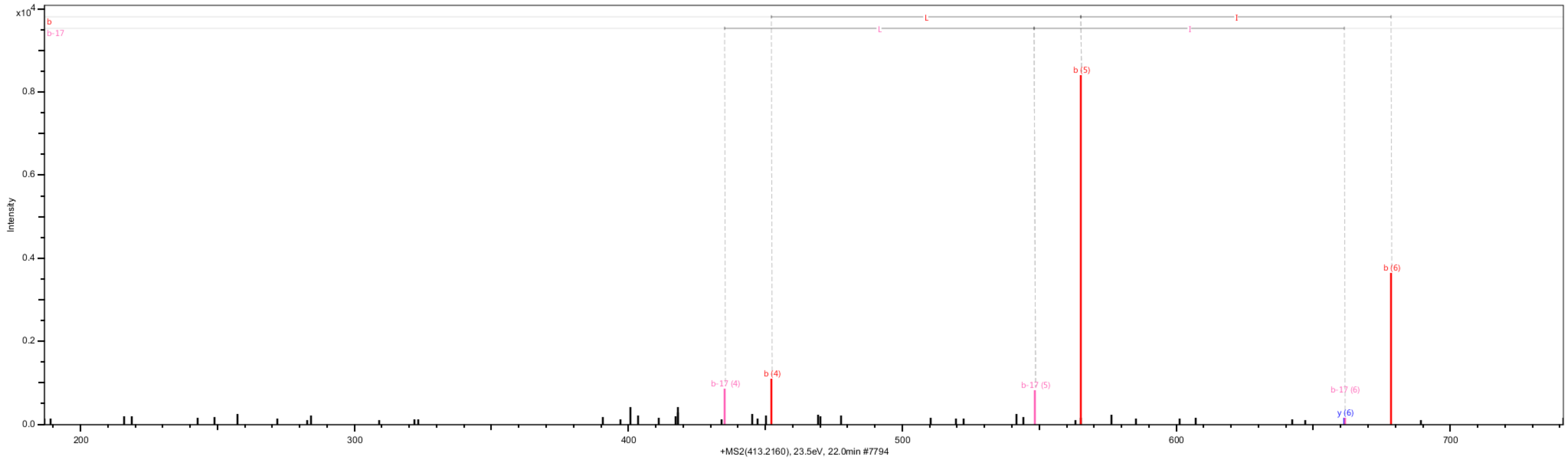
x10^4
b
b-17
L
I
b (5)
b (6)
b (4)
b-17 (4)
b-17 (5)
b-17 (6)
y (6)
Intensity
0.8
0.6
0.4
0.2
0.0
200
300
400
500
600
700
+MS2(413.2160), 23.5eV, 22.0min #7794

TBC15_HUMAN

Accession: TBC15_HUMAN
Database: SwissProt
MW [kDa] / pI: 79.4 / 5.4
Score: 34.3
Seq. Coverage [%]: 2.6
No. of Peptides: 2

Peptide Match

Sequence: -.MAAAGVVSGK.I
Score: 15.9
Modifications: Oxidation: 1

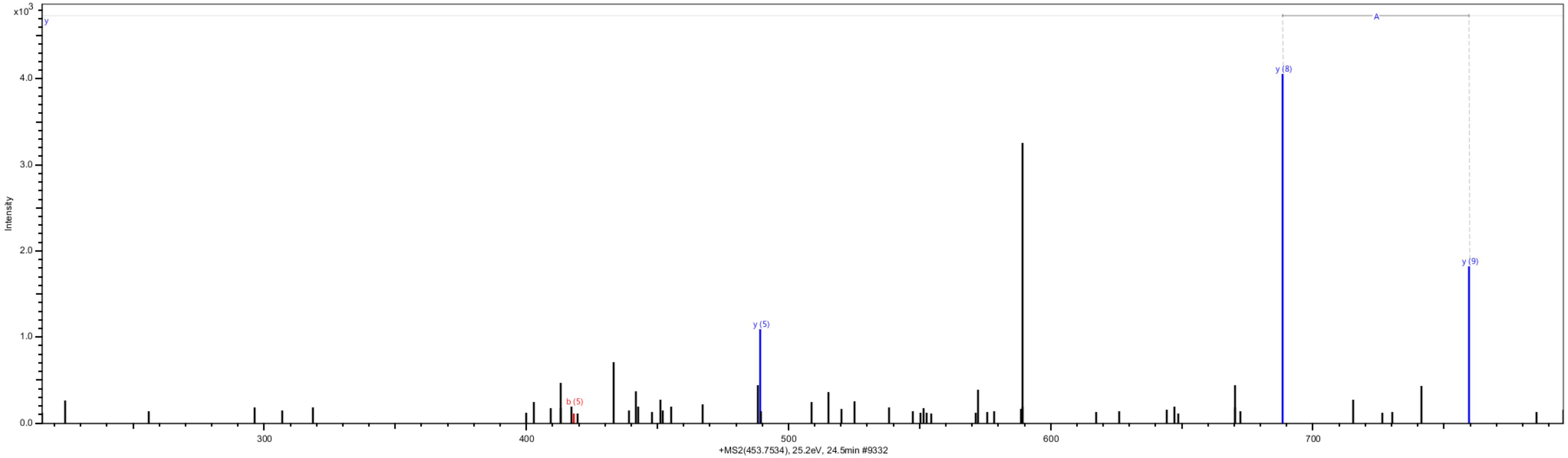

Peptide Match

Sequence: K.SISQEQEK.R
Score: 18.5
Modifications:

Spectrum Report
Combine Search_2020-10-19 07:53:31

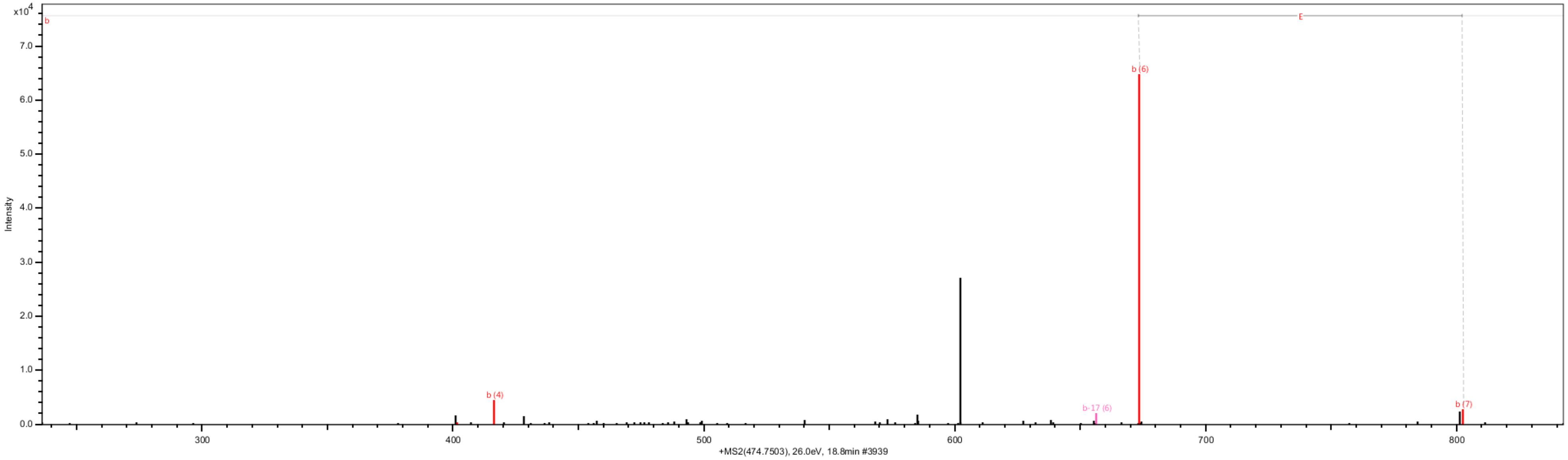

SAA2_HUMAN

**Accession:**	SAA2_HUMAN	**Score:**	34.3
**Database:**	SwissProt	**Seq. Coverage [%]:**	11.5
**MW [kDa] / pI:**	13.5 / 9.2	**No. of Peptides:**	1

Peptide Match

**Sequence:**	R.EANYIGSDKYFHAR.G	**Score:**	34.3
**Modifications:**			

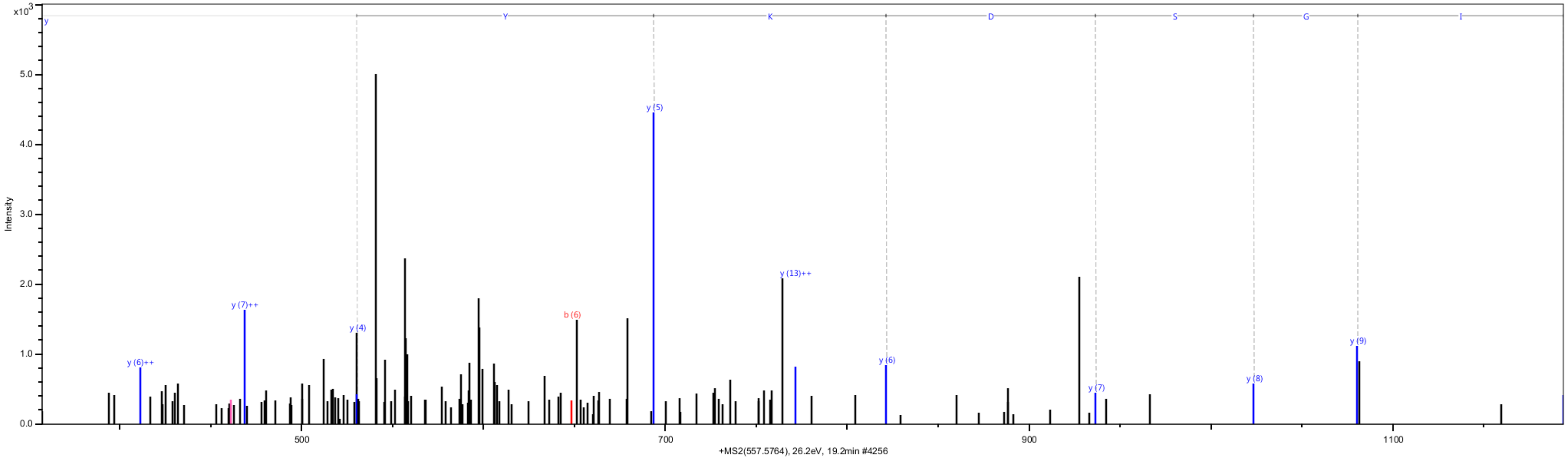

SRBP2_HUMAN

**Accession:**	SRBP2_HUMAN	**Score:**	34.2
**Database:**	SwissProt	**Seq. Coverage [%]:**	1.0
**MW [kDa] / pI:**	123.6 / 8.7	**No. of Peptides:**	1

Peptide Match

**Sequence:**	K.ALEVTESPLVK.A	**Score:**	34.2
**Modifications:**			

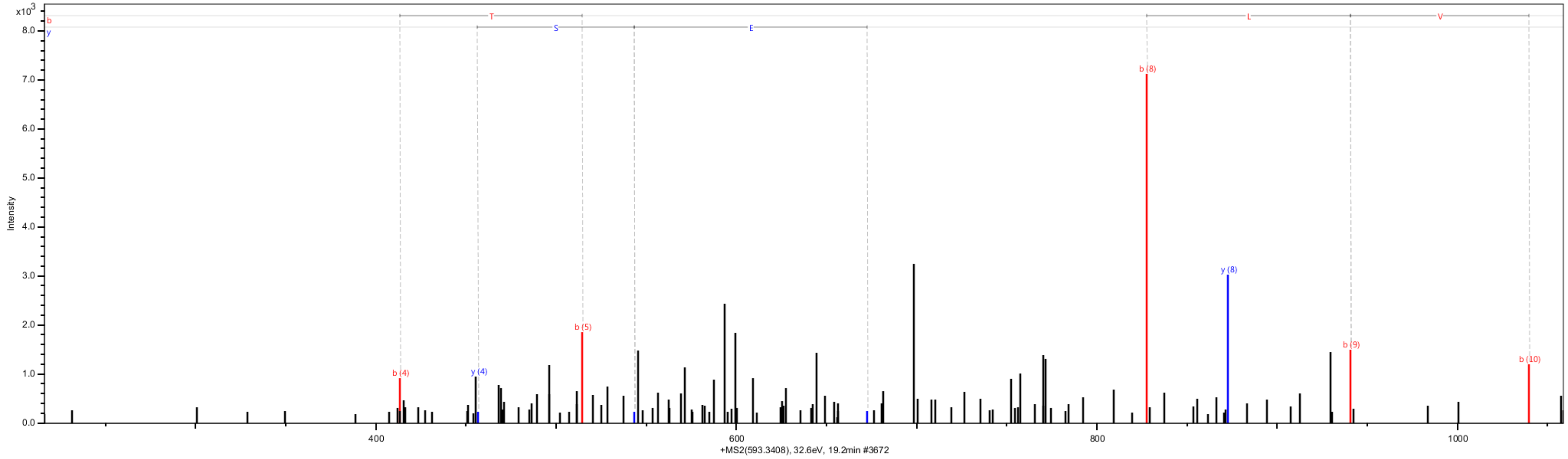

TET3_HUMAN

Accession: TET3_HUMAN
Database: SwissProt
MW [kDa] / pI: 193.6 / 8.0
Score: 34.9
Seq. Coverage [%]: 0.5
No. of Peptides: 1

Peptide Match

Sequence: R.CGTCEPCRR.L
Score: 16
Modifications: Carbamidomethyl: 1, 4, 7

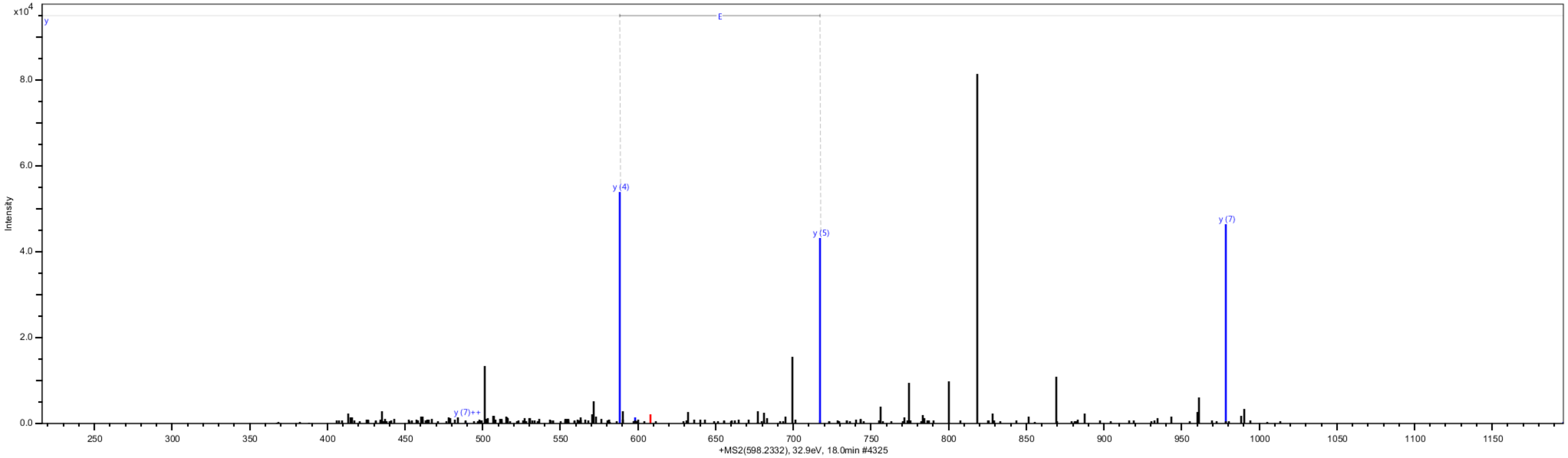

AMOT_HUMAN

Accession: AMOT_HUMAN
Database: SwissProt
MW [kDa] / pI: 118.0 / 7.3
Score: 34.7
Seq. Coverage [%]: 0.9
No. of Peptides: 1

Peptide Match

Sequence: R.ILALEADMTK.W
Score: 17.9
Modifications: Oxidation: 8

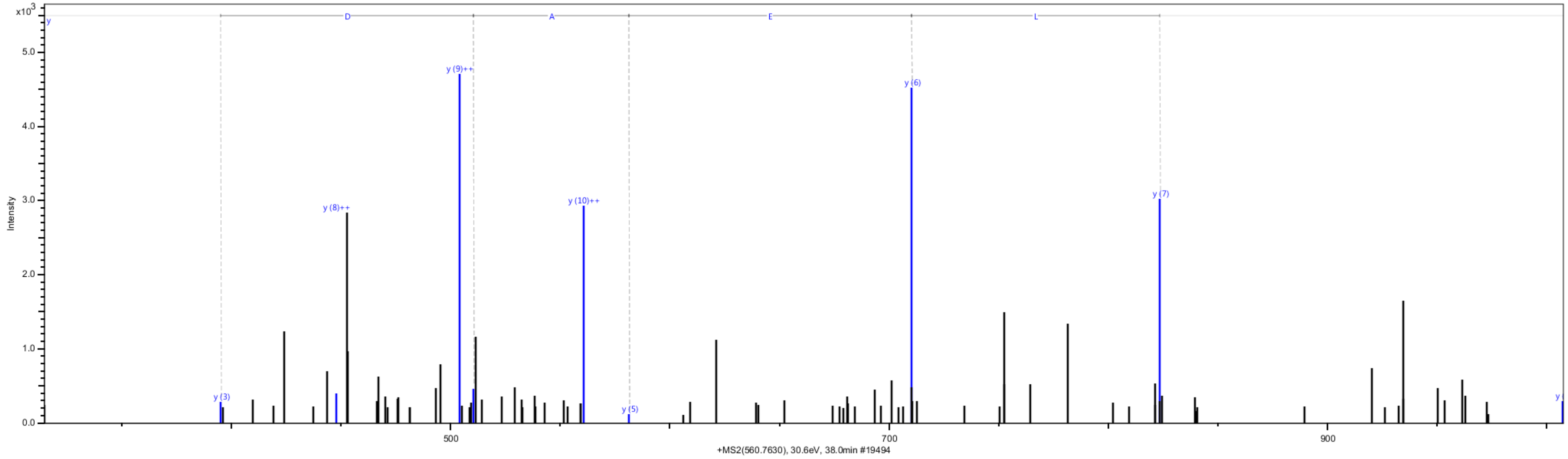

NAA25_HUMAN

Accession: NAA25_HUMAN
Database: SwissProt
MW [kDa] / pI: 112.2 / 6.2
Score: 34.6
Seq. Coverage [%]: 0.7
No. of Peptides: 1

Peptide Match

Sequence: K.CMAMYKK.L
Score: 17.8
Modifications: Carbamidomethyl: 1; Oxidation: 2

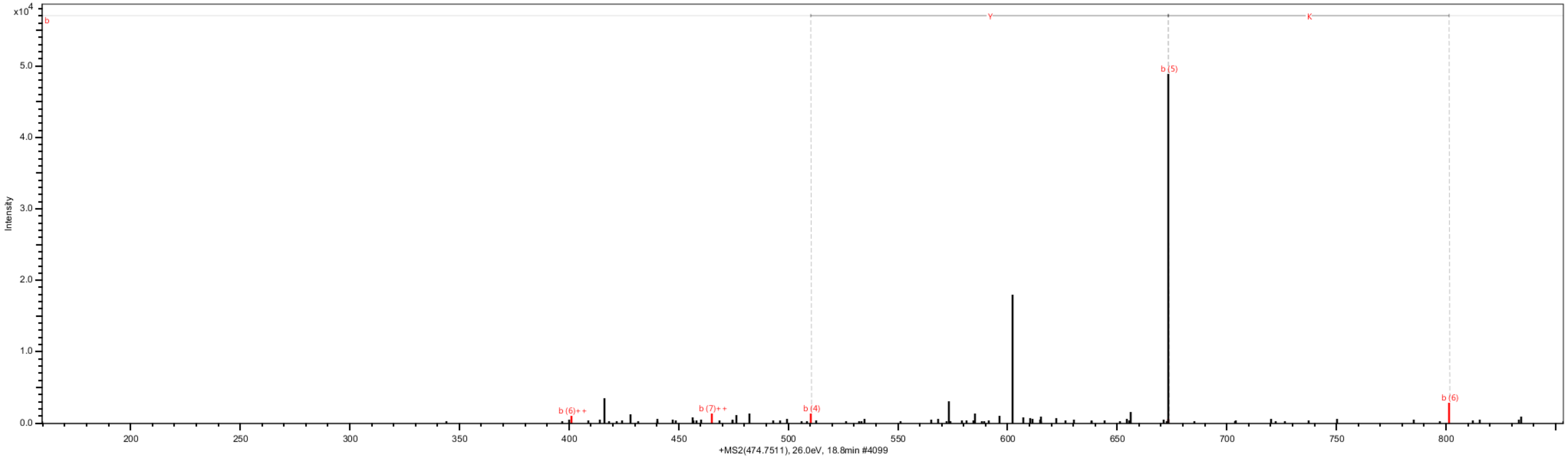

CE162_HUMAN

Accession: CE162_HUMAN
Database: SwissProt
MW [kDa] / pI: 161.8 / 5.4
Score: 34.9
Seq. Coverage [%]: 0.7
No. of Peptides: 2

Peptide Match

Sequence: R.KIATQEVLIR.H
Score: 17.6
Modifications:

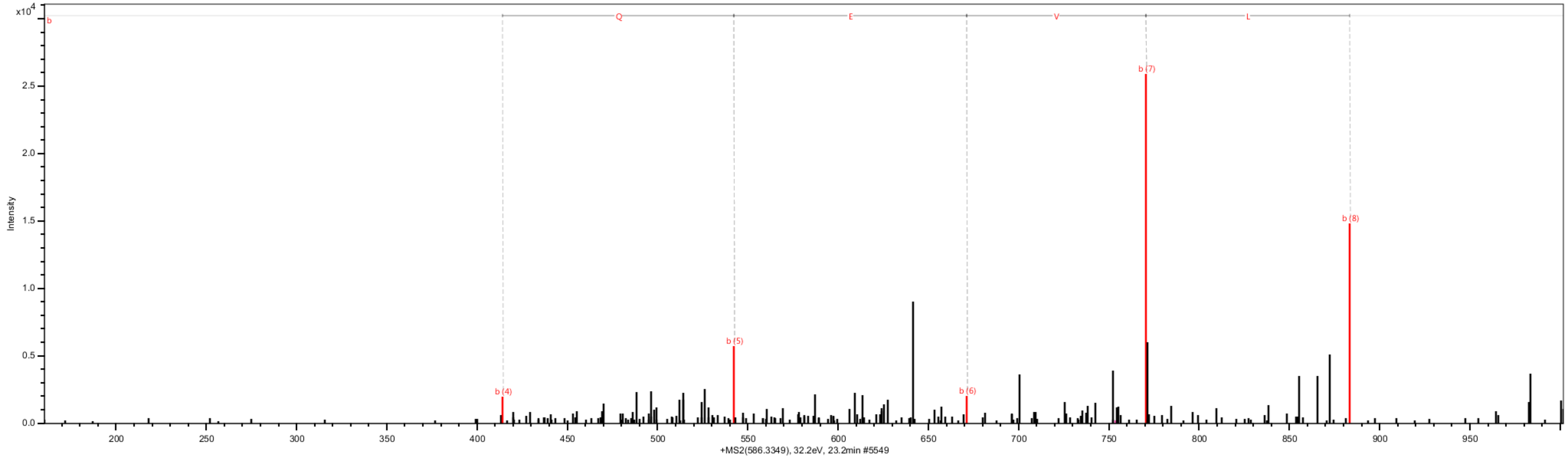

Peptide Match

Sequence: K.IATQEVLIR.H
Score: 17.3
Modifications:

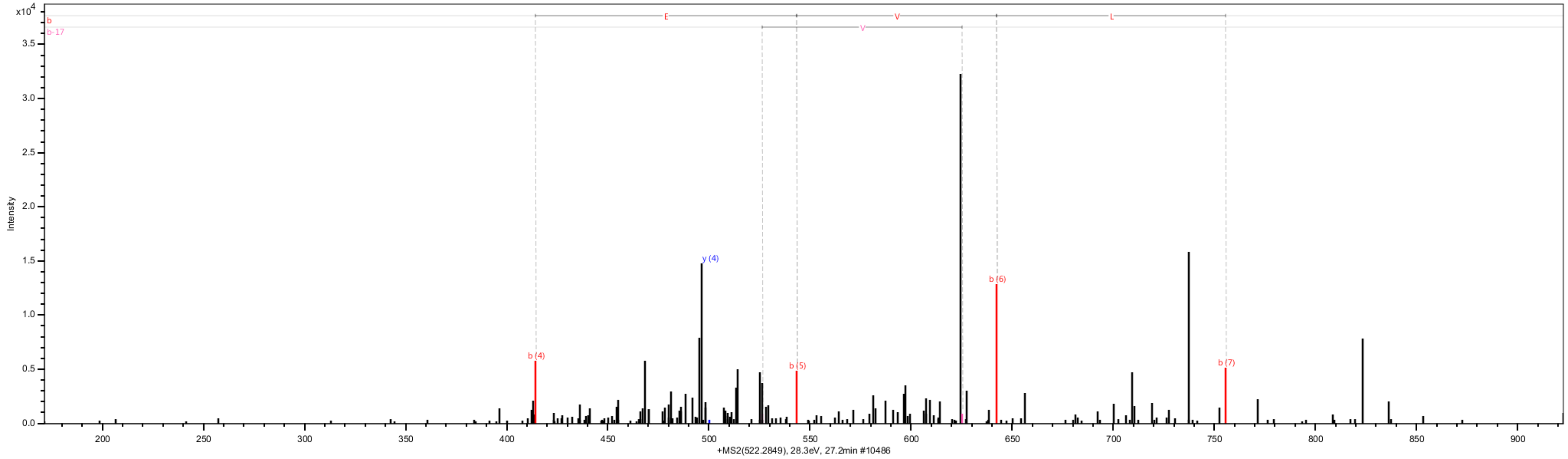
x10^4
b
b-17
E
V
V
L
Intensity
y (4)
b (6)
b (4)
b (5)
b (7)
+MS2(522.2849), 28.3eV, 27.2min #10486

S22A1_HUMAN

Accession: S22A1_HUMAN
Database: SwissProt
MW [kDa] / pI: 61.1 / 6.4
Score: 35.1
Seq. Coverage [%]: 4.5
No. of Peptides: 2

Peptide Match

Sequence: M.PTVDDILEQVGESGWFQK.Q **Score:** 16.6
Modifications:

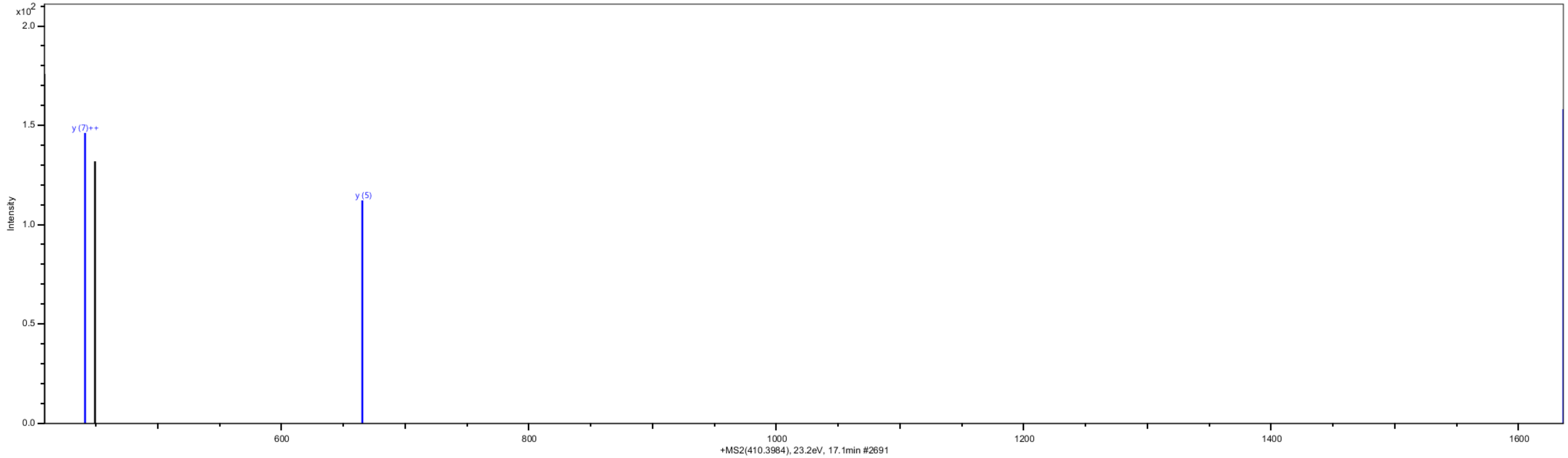

Peptide Match

Sequence: K.RNTEAIK.I **Score:** 18.6
Modifications:

Spectrum Report
Combine Search_2020-10-19 07:53:31

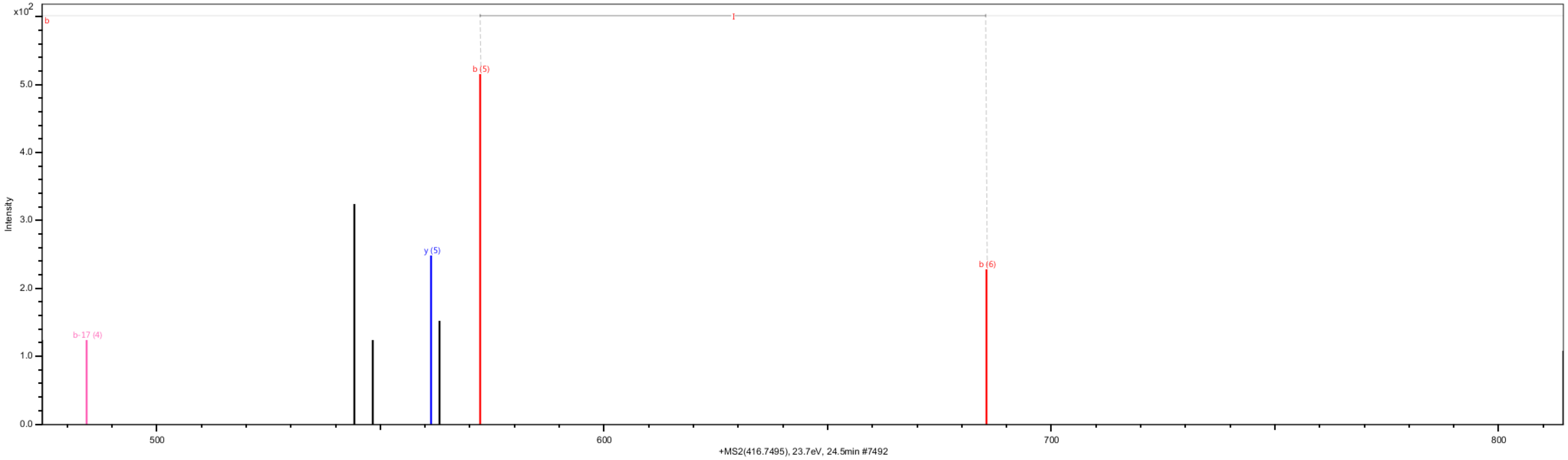

PRP16_HUMAN

Accession: PRP16_HUMAN
Database: SwissProt
MW [kDa] / pI: 140.4 / 6.1
Score: 35.3
Seq. Coverage [%]: 1.4
No. of Peptides: 2

Peptide Match

Sequence: R.RNEPESPR.H
Score: 15.8
Modifications:

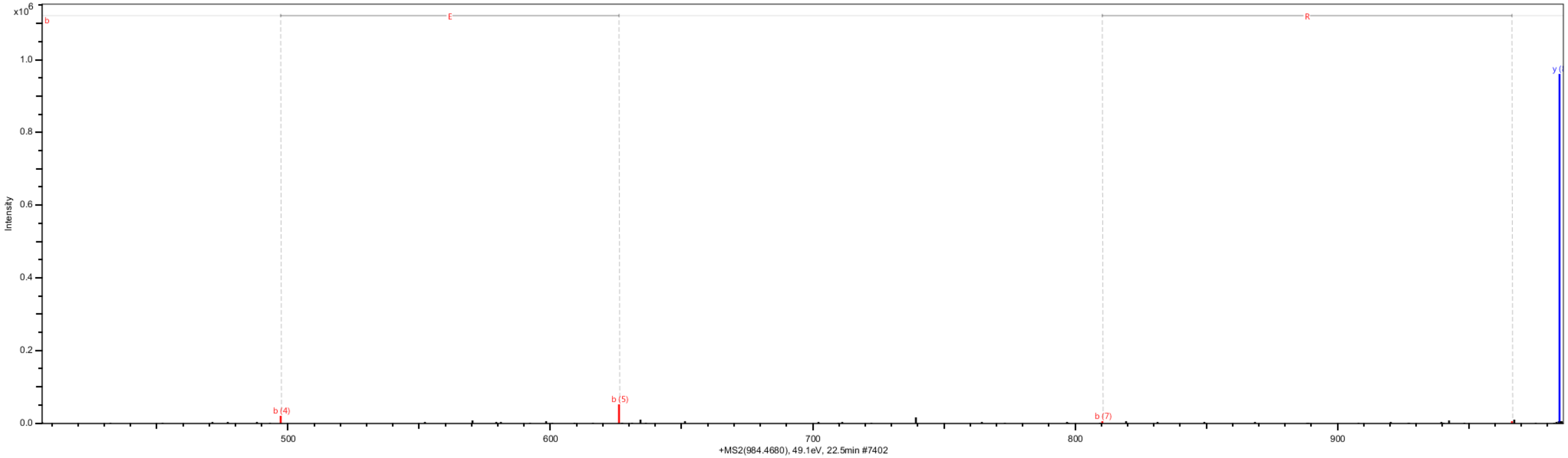

Peptide Match

Sequence: K.LGDIMGVKK.E
Score: 19.4
Modifications:

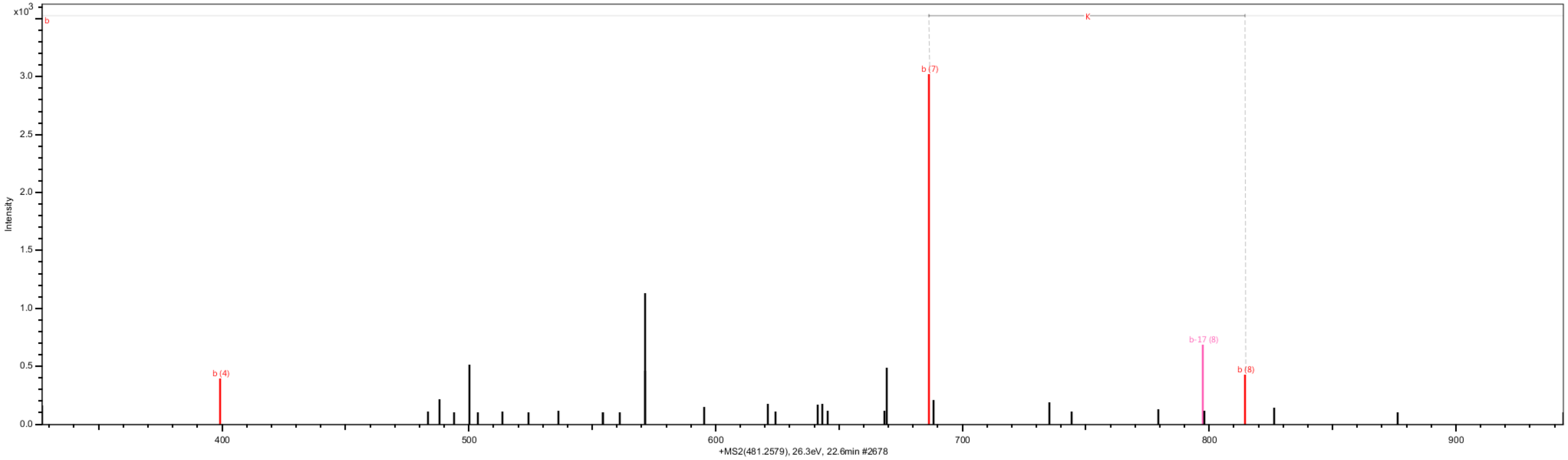
x10^3
b
K
b (7)
b-17 (8)
b (8)
b (4)
Intensity
3.0
2.5
2.0
1.5
1.0
0.5
0.0
400
500
600
700
800
900
+MS2(481.2579), 26.3eV, 22.6min #2678

CD44_HUMAN

**Accession:**	CD44_HUMAN	**Score:**	35.5
**Database:**	SwissProt	**Seq. Coverage [%]:**	1.6
**MW [kDa] / pI:**	81.5 / 5.1	**No. of Peptides:**	1

Peptide Match

**Sequence:**	R.YGFIEGHVVIPR.I	**Score:**	35.5
**Modifications:**			

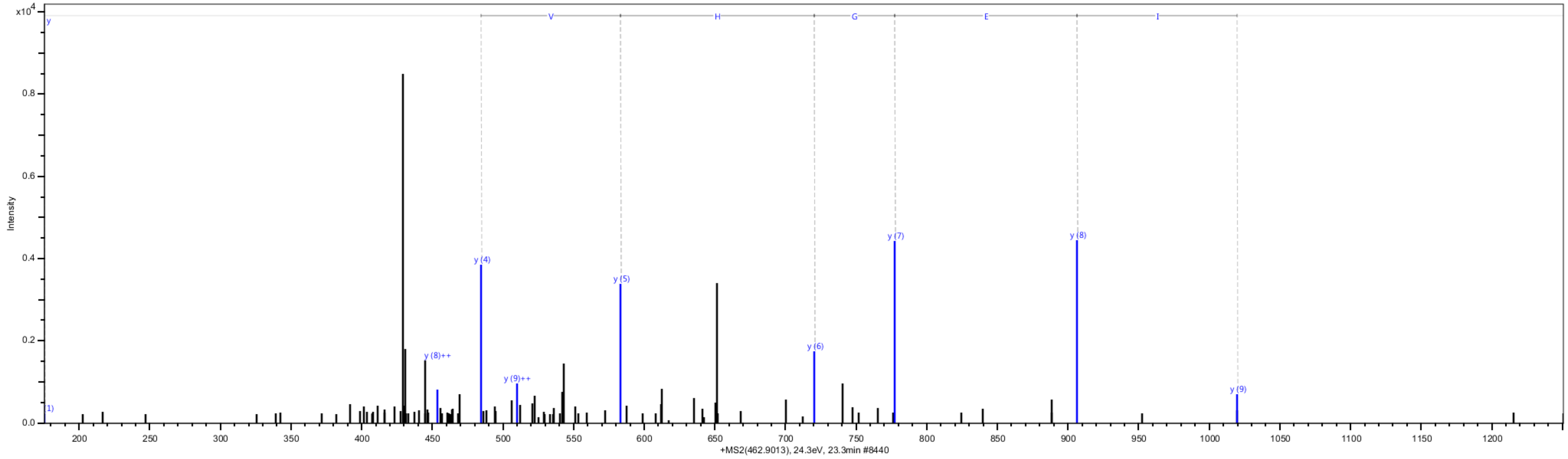

RET4_HUMAN

**Accession:**	RET4_HUMAN	**Score:**	35.6
**Database:**	SwissProt	**Seq. Coverage [%]:**	5.0
**MW [kDa] / pI:**	23.0 / 5.8	**No. of Peptides:**	1

Peptide Match

**Sequence:**	K.YWGVASFLQK.G	**Score:**	35.6
**Modifications:**			

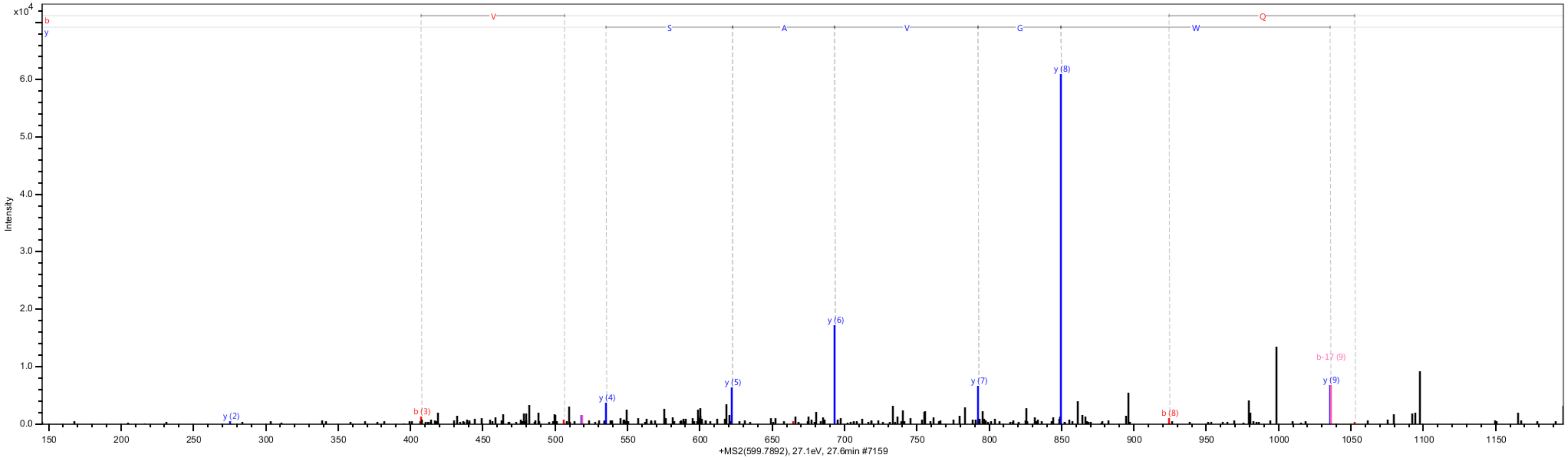

ZN512_HUMAN

**Accession:**	ZN512_HUMAN	**Score:**	35.9
**Database:**	SwissProt	**Seq. Coverage [%]:**	3.5
**MW [kDa] / pI:**	64.6 / 9.8	**No. of Peptides:**	2

Peptide Match

**Sequence:**	R.KSTYWMK.M	**Score:**	18.6
**Modifications:**	Oxidation: 6		

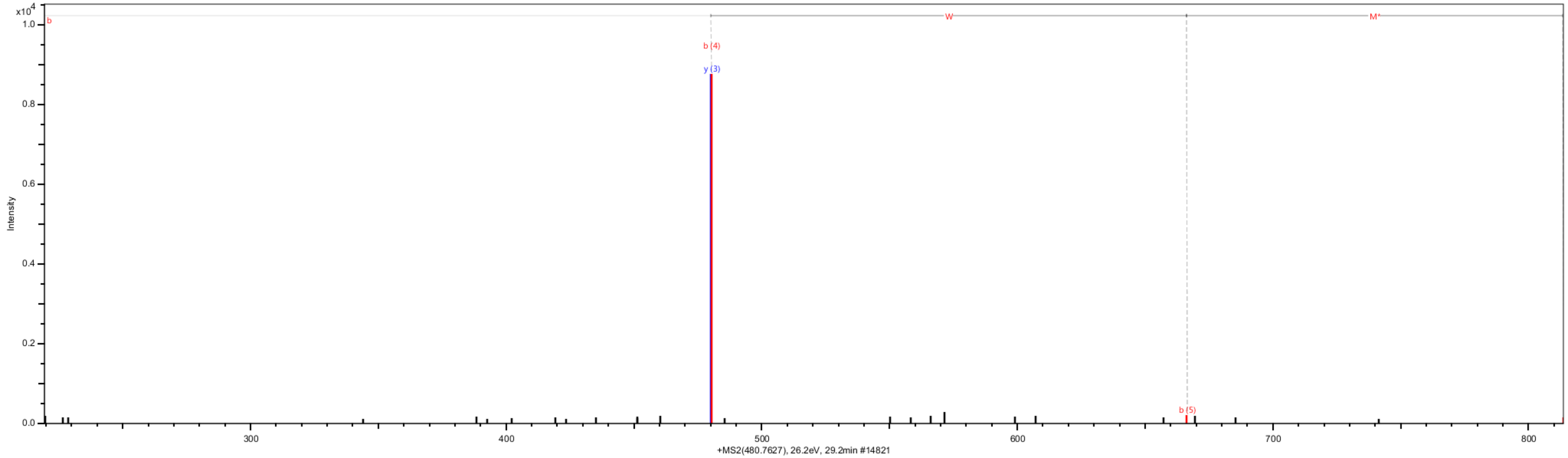

Peptide Match

**Sequence:**	R.RNNEELVVSASCK.E	**Score:**	17.2
**Modifications:**	Carbamidomethyl: 12		

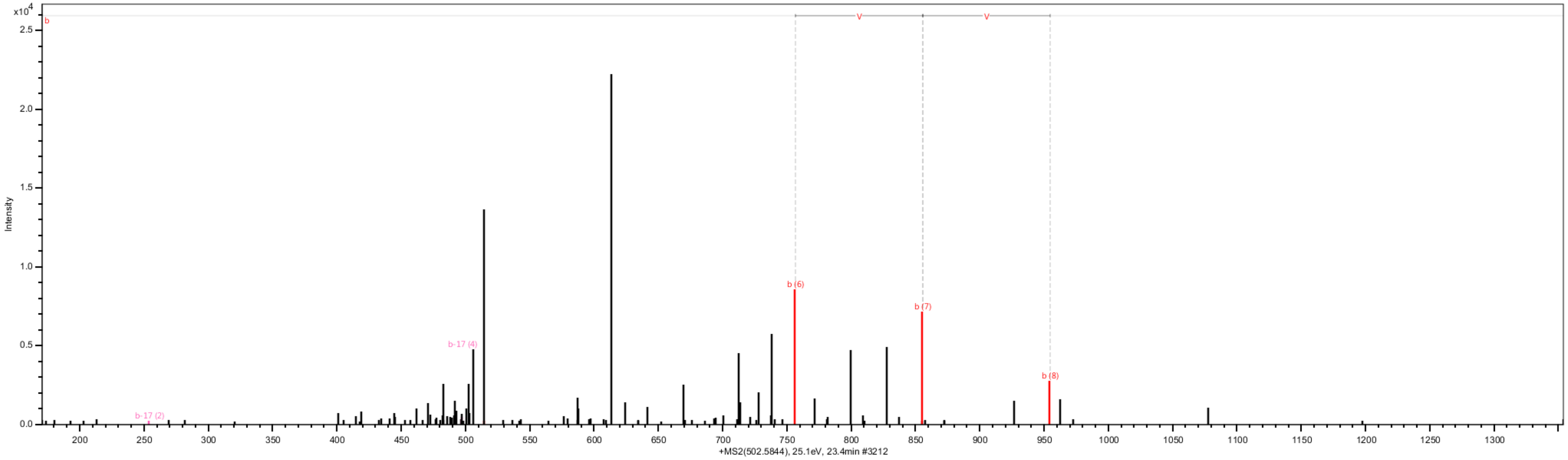
x10^4
Intensity
b
b-17 (2)
b-17 (4)
b (6)
b (7)
b (8)
V
V
+MS2(502.5844), 25.1eV, 23.4min #3212

CC178_HUMAN

Accession: CC178_HUMAN
Database: SwissProt
MW [kDa] / pI: 101.9 / 6.3
Score: 35.9
Seq. Coverage [%]: 2.1
No. of Peptides: 2

Peptide Match

Sequence: K.TELEEANAK.I
Score: 15.8
Modifications:

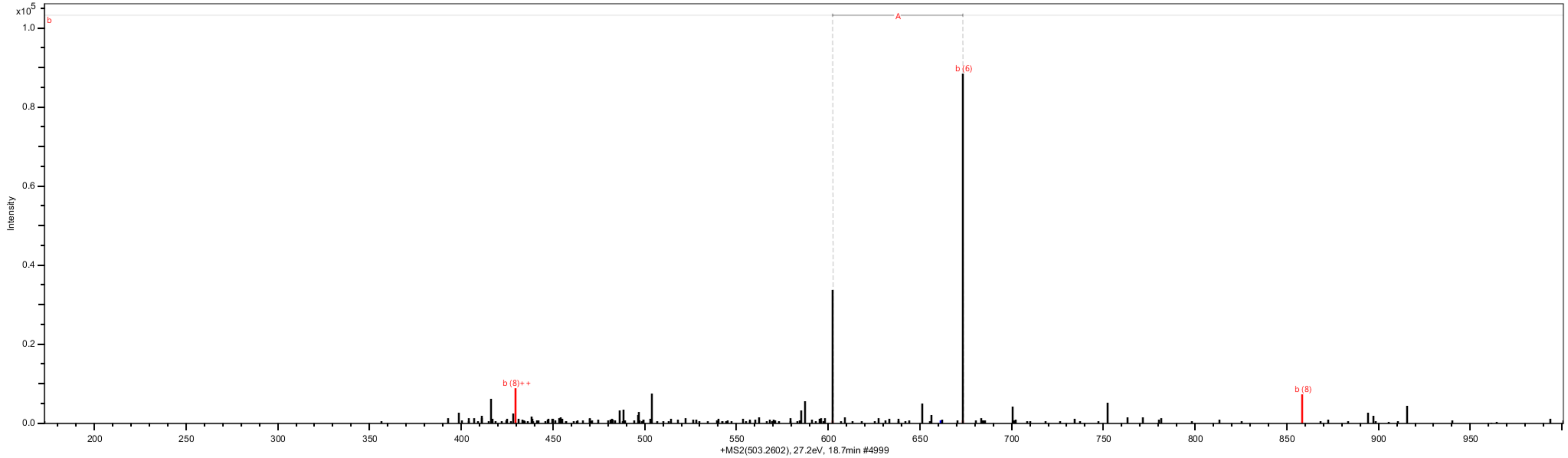

Peptide Match

Sequence: K.LVVLFSQMR.L
Score: 20.2
Modifications:

Spectrum Report
Combine Search_2020-10-19 07:53:31

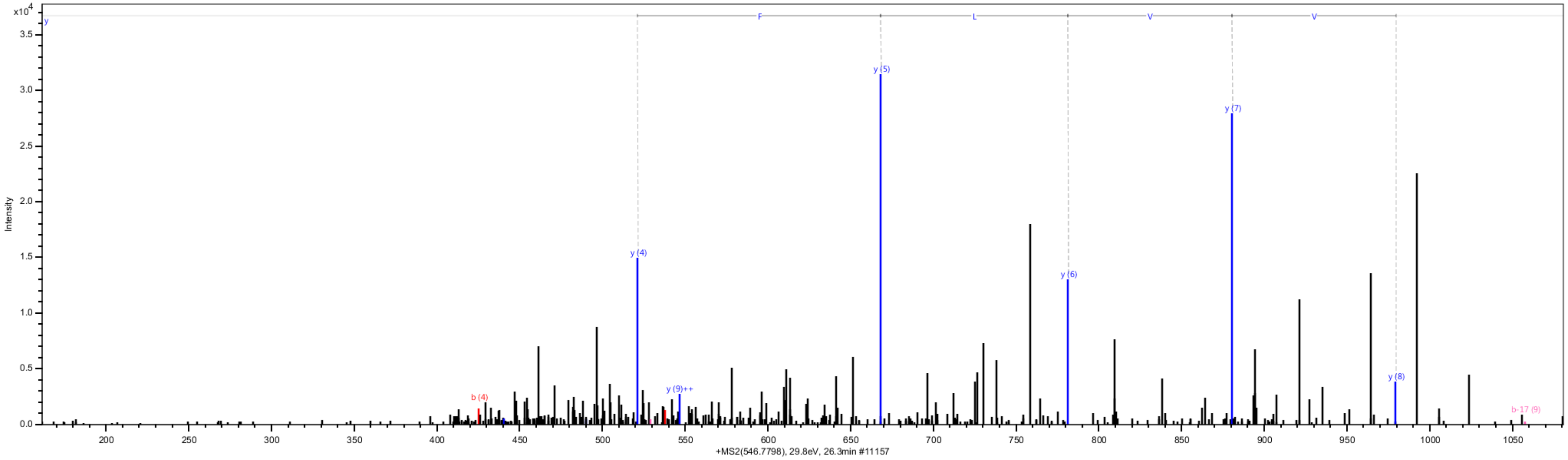

AHNK2_HUMAN

**Accession:**	AHNK2_HUMAN	**Score:**	35.9
**Database:**	SwissProt	**Seq. Coverage [%]:**	0.3
**MW [kDa] / pI:**	616.2 / 5.2	**No. of Peptides:**	2

Peptide Match

**Sequence:**	K.VQMPSLKMPK.V	**Score:**	17.3
**Modifications:**	Oxidation: 3, 8		

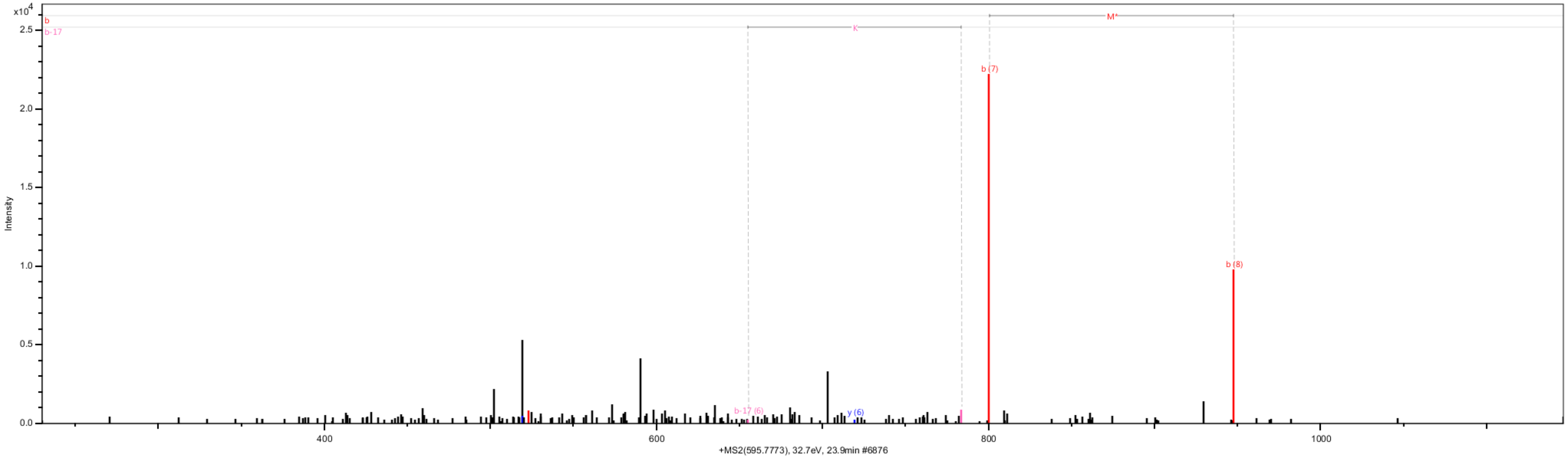

Peptide Match

**Sequence:**	K.VQTGQVDVK.L	**Score:**	18.6
**Modifications:**			

Spectrum Report
Combine Search_2020-10-19 07:53:31

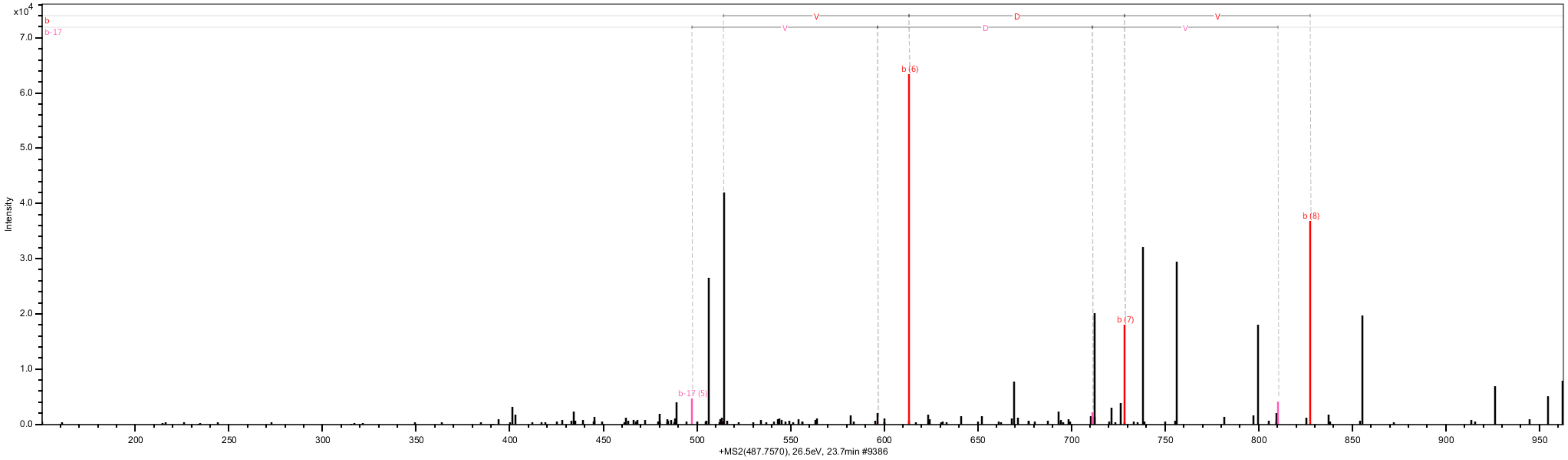

FMNL1_HUMAN

Accession: FMNL1_HUMAN
Database: SwissProt
MW [kDa] / pI: 121.8 / 5.6
Score: 36.1
Seq. Coverage [%]: 1.5
No. of Peptides: 2

Peptide Match

Sequence: R.RDSELGPGVK.A
Score: 17.6
Modifications:

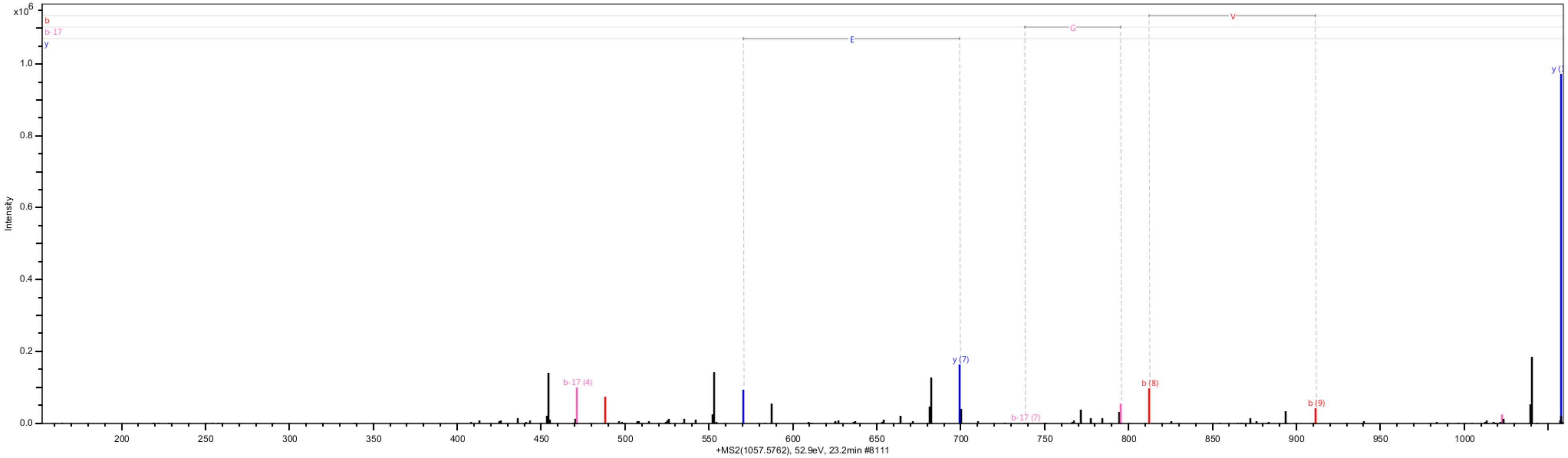

Peptide Match

Sequence: R.GLELTQR.E
Score: 18.5
Modifications:

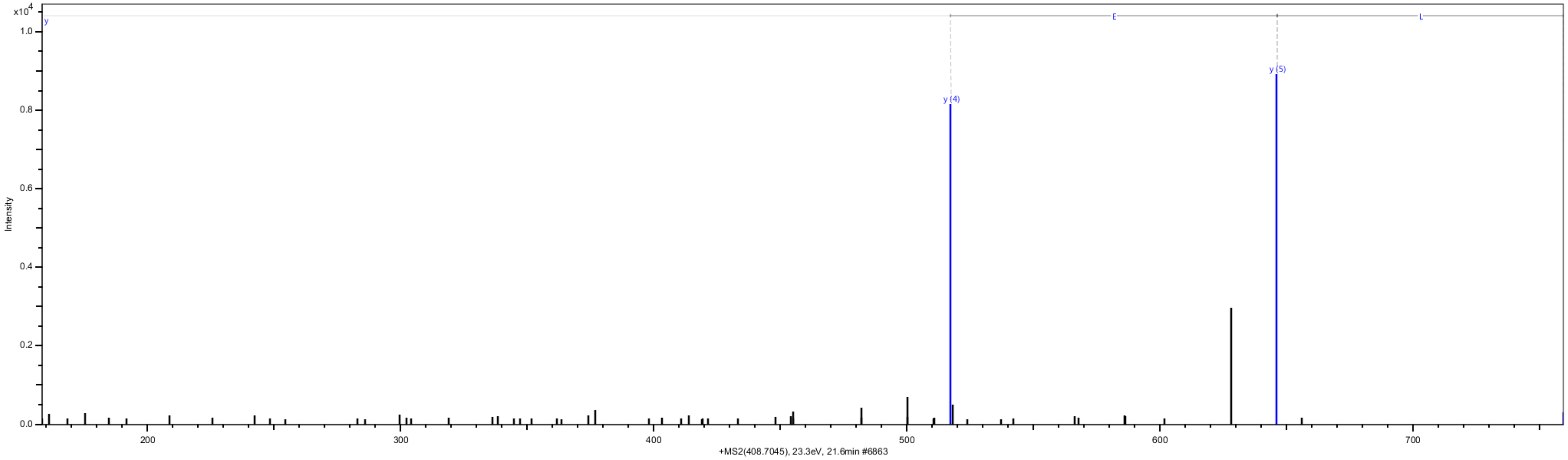
x10^4
1.0
0.8
0.6
0.4
0.2
0.0
Intensity
y
E
L
y (4)
y (5)
200
300
400
500
600
700
+MS2(408.7045), 23.3eV, 21.6min #6863

CBG_HUMAN

Accession: CBG_HUMAN
Database: SwissProt
MW [kDa] / pI: 45.1 / 5.6
Score: 36.6
Seq. Coverage [%]: 2.5
No. of Peptides: 1

Peptide Match

Sequence: K.MNTVIAALSR.D
Score: 36.6
Modifications:

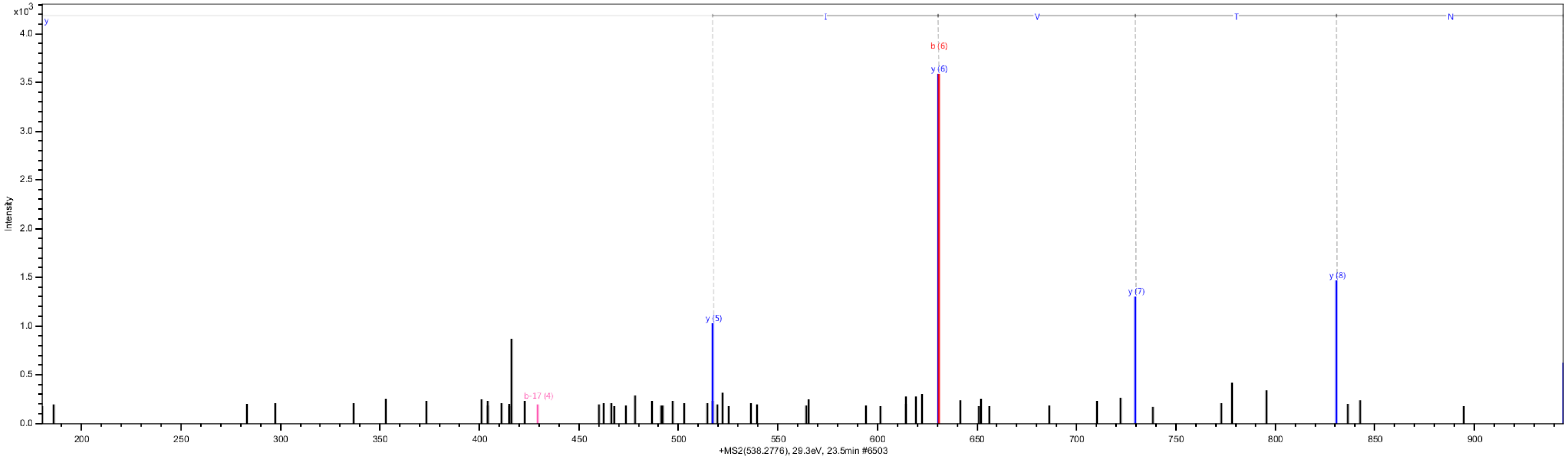

FBLN3_HUMAN

**Accession:**	FBLN3_HUMAN	**Score:**	36.7
**Database:**	SwissProt	**Seq. Coverage [%]:**	1.8
**MW [kDa] / pI:**	54.6 / 5.0	**No. of Peptides:**	1

Peptide Match

**Sequence:**	R.ADQVCINLR.G	**Score:**	36.7
**Modifications:**	Carbamidomethyl: 5		

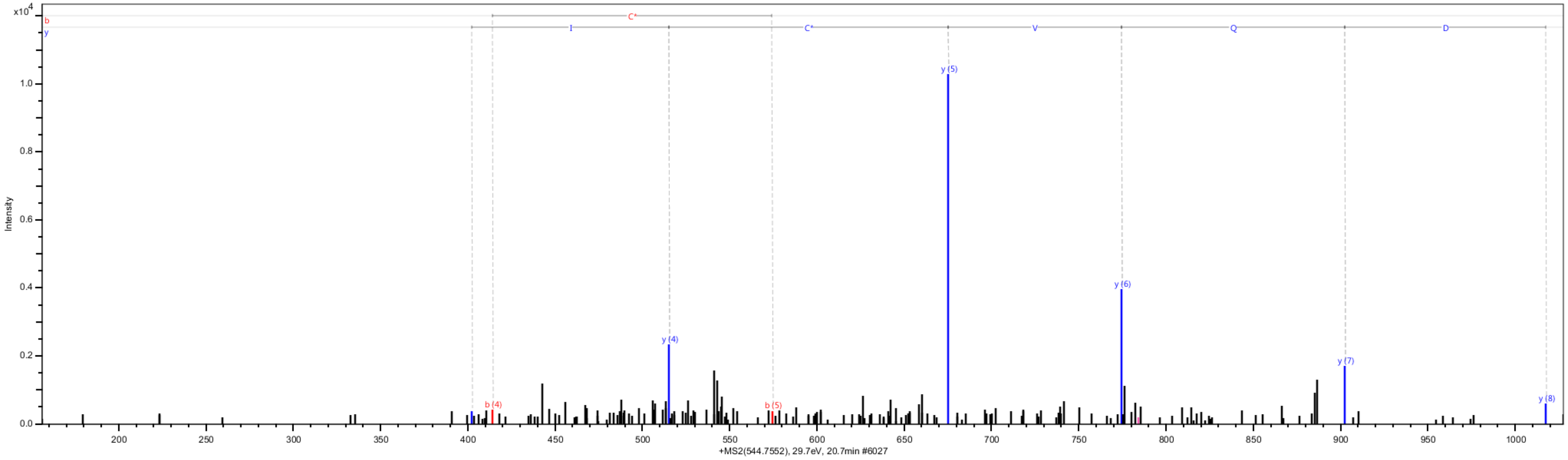

TETN_HUMAN

Accession: TETN_HUMAN
Database: SwissProt
MW [kDa] / pI: 22.5 / 5.5
Score: 36.9
Seq. Coverage [%]: 4.5
No. of Peptides: 1

Peptide Match

Sequence: K.CFLAFTQTK.T
Score: 36.9
Modifications: Carbamidomethyl: 1

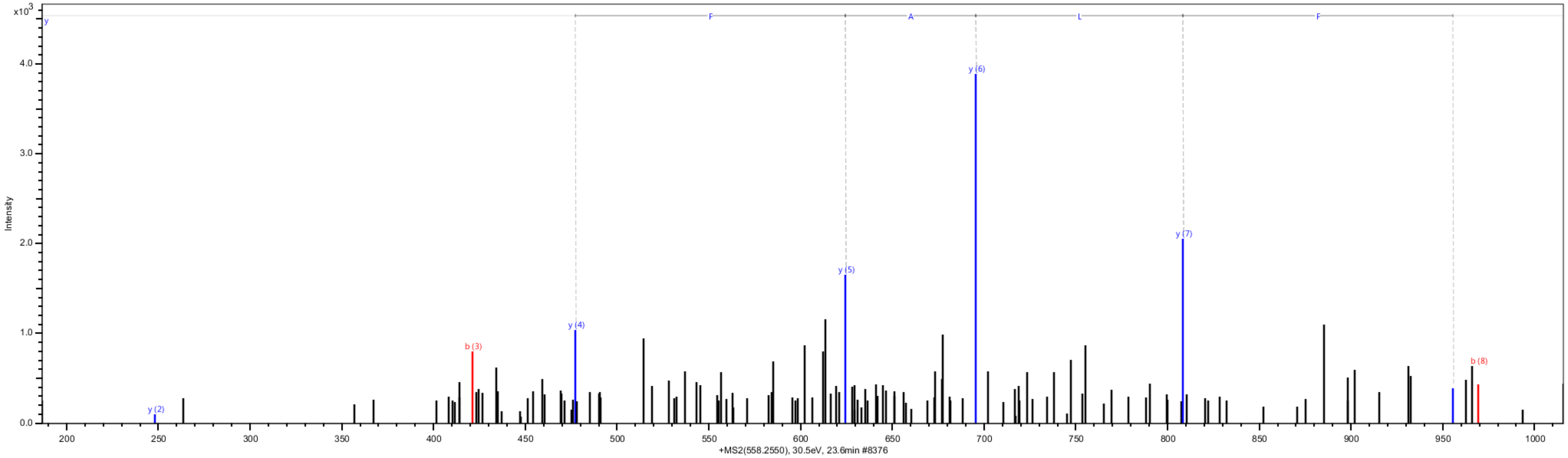

CK5P2_HUMAN

Accession: CK5P2_HUMAN
Database: SwissProt
MW [kDa] / pI: 214.9 / 5.4
Score: 37.2
Seq. Coverage [%]: 0.4
No. of Peptides: 1

Peptide Match

Sequence: R.IYFLEER.M
Score: 15.7
Modifications:

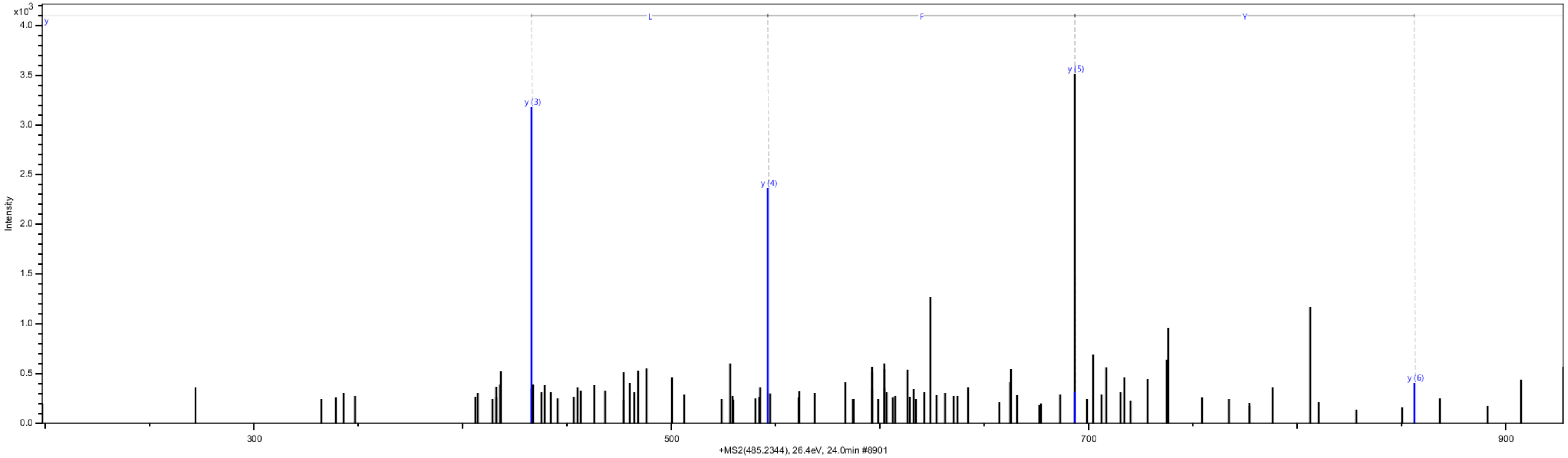

RRBP1_HUMAN

Accession: RRBP1_HUMAN
Database: SwissProt
MW [kDa] / pI: 152.4 / 8.7
Score: 37.3
Seq. Coverage [%]: 1.4
No. of Peptides: 2

Peptide Match

Sequence: K.KGEGAQNQGK.K **Score:** 20.5
Modifications:

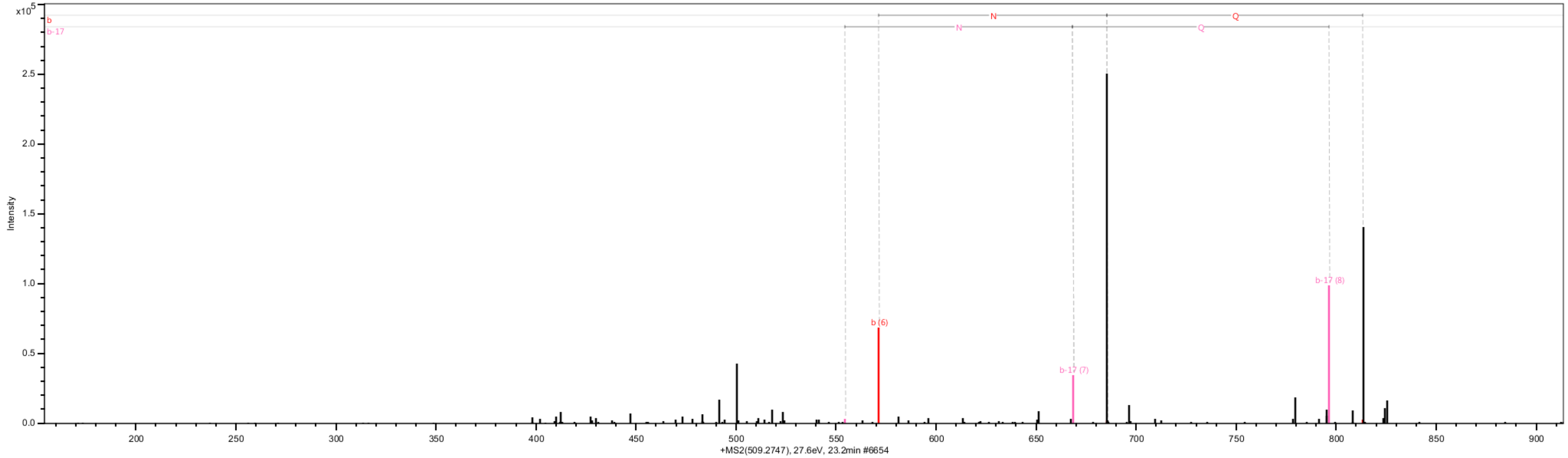

Peptide Match

Sequence: K.ELVEKSEAVR.Q **Score:** 16.8
Modifications:

Spectrum Report
Combine Search_2020-10-19 07:53:31

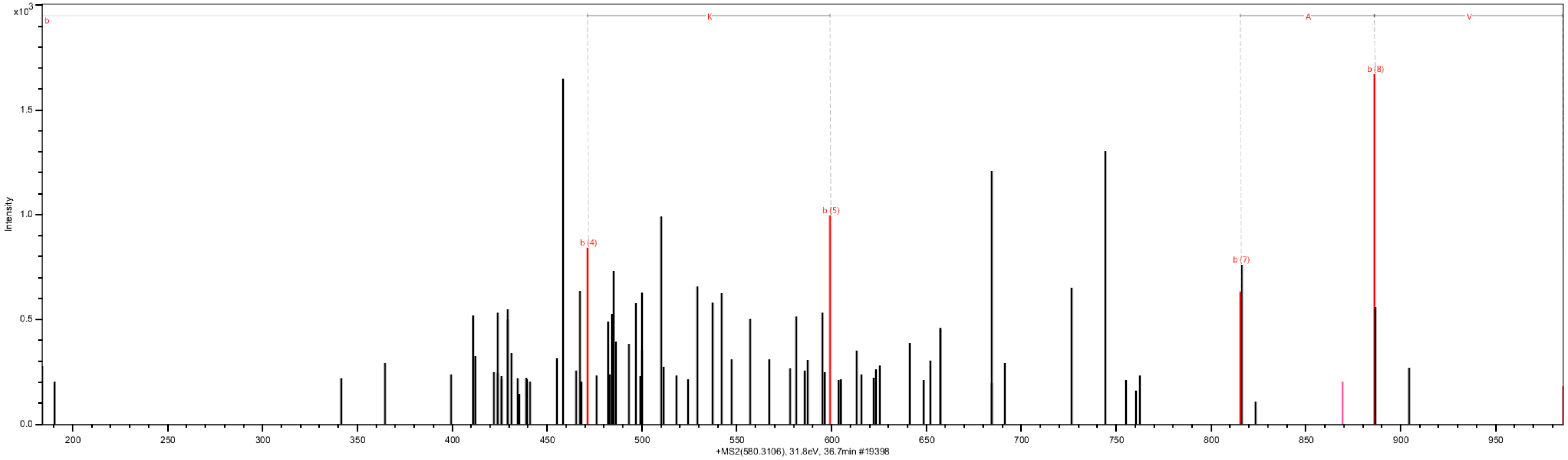

GLI2_HUMAN

Accession: GLI2_HUMAN
Database: SwissProt
MW [kDa] / pI: 167.7 / 6.9
Score: 37.7
Seq. Coverage [%]: 1.5
No. of Peptides: 2

Peptide Match

Sequence: R.KHMTTMHR.F
Score: 22.1
Modifications: Oxidation: 3, 6

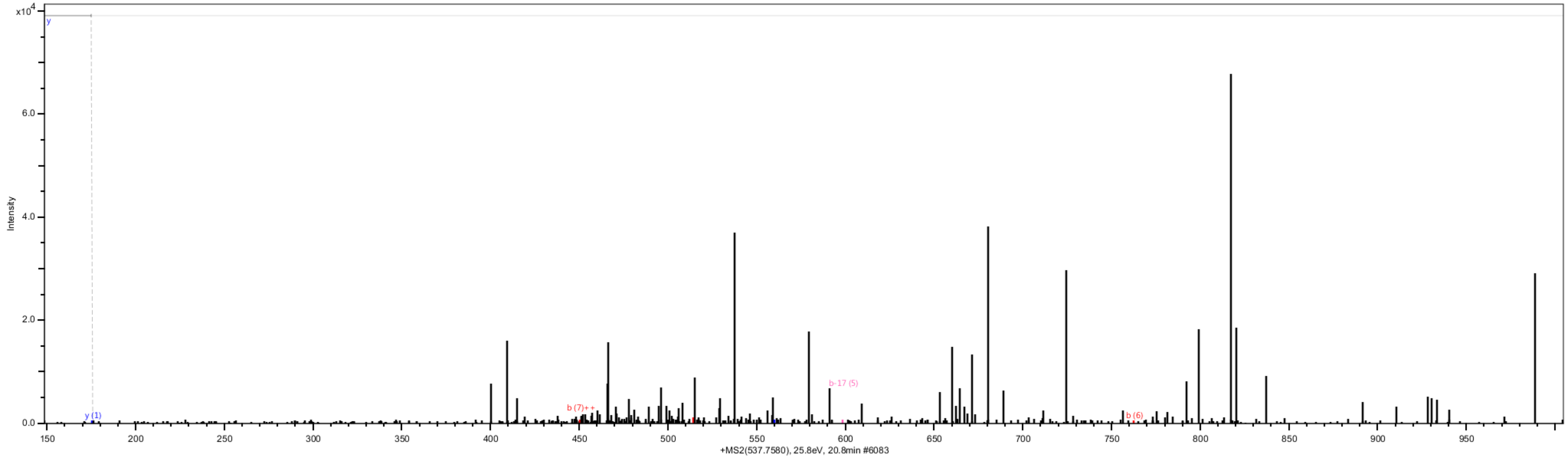

Peptide Match

Sequence: K.SLKDSCSWAGPTPHTR.N
Score: 15.6
Modifications: Carbamidomethyl: 6

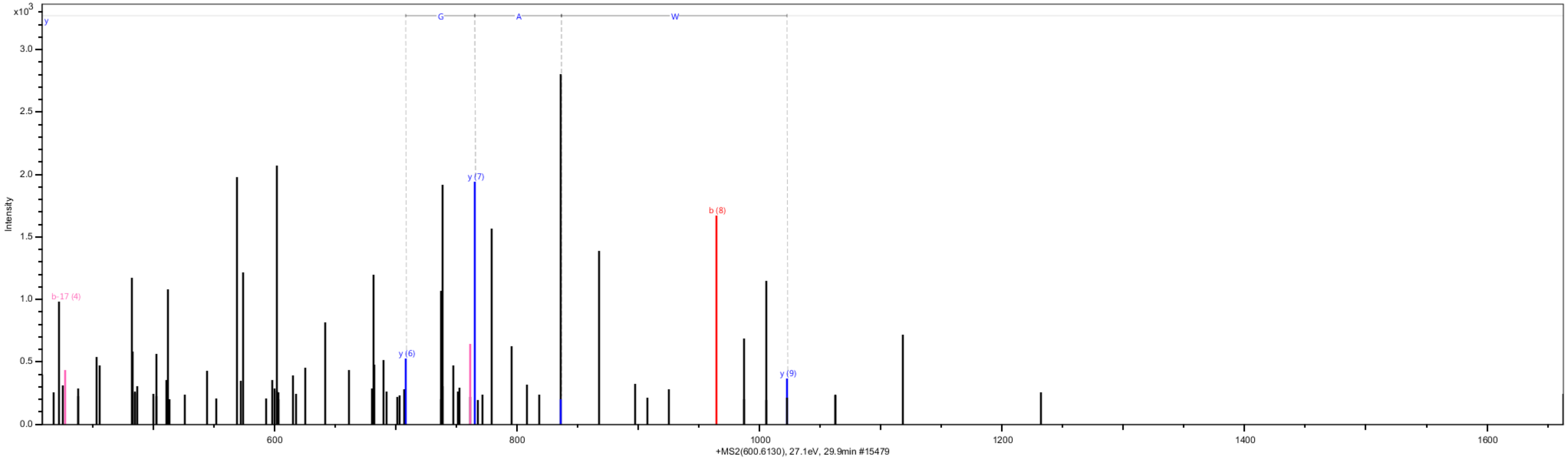
x10^3
y
G
A
W
Intensity
3.0
2.5
2.0
1.5
1.0
0.5
0.0
b-17 (4)
y (6)
y (7)
b (8)
y (9)
600
800
1000
1200
1400
1600
+MS2(600.6130), 27.1eV, 29.9min #15479

PEPL_HUMAN

Accession: PEPL_HUMAN
Database: SwissProt
MW [kDa] / pI: 204.6 / 5.5
Score: 38.0
Seq. Coverage [%]: 1.3
No. of Peptides: 2

Peptide Match

Sequence: R.GYSYTLQK.N
Score: 17.4
Modifications:

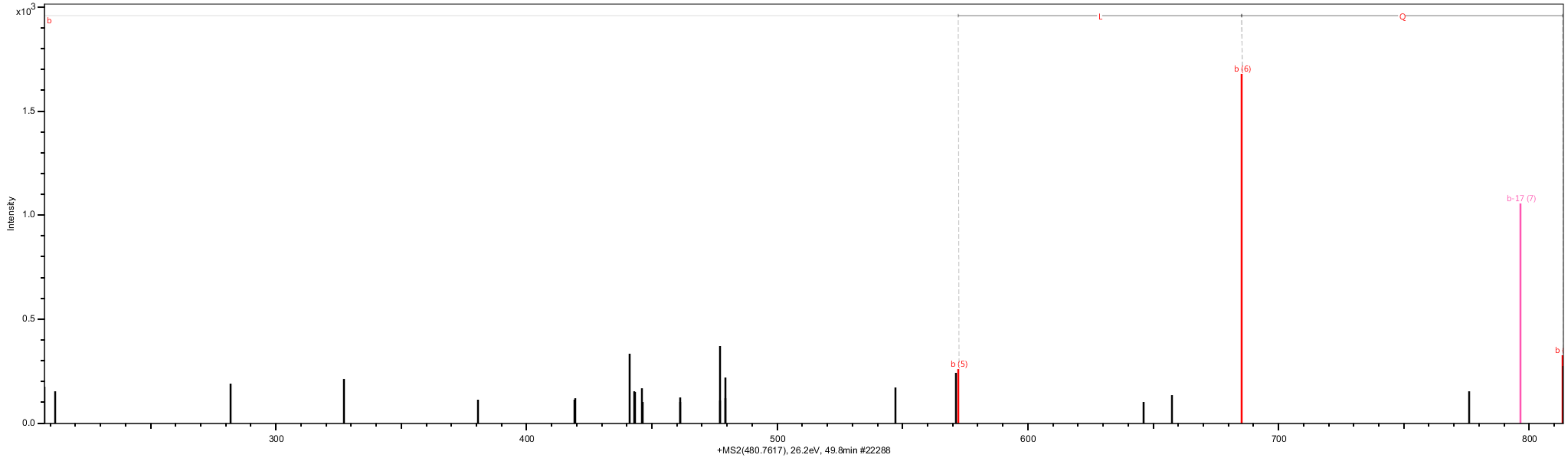

Peptide Match

Sequence: R.NRPDSGVEEAWKIR.K
Score: 20.6
Modifications:

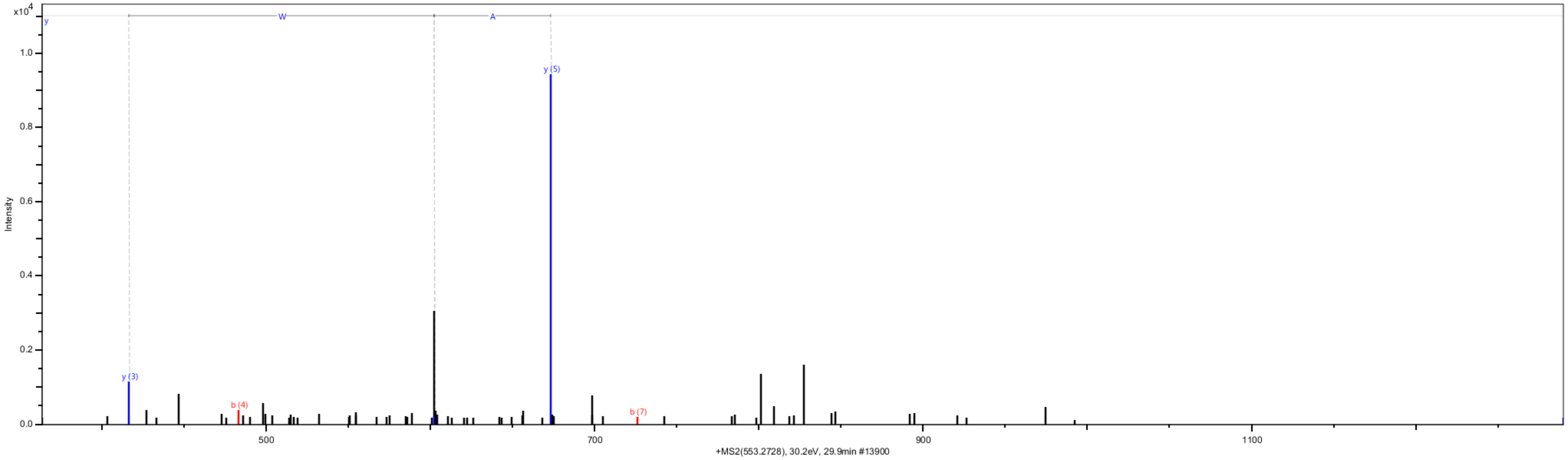
x10^4
y
W
A
1.0
0.8
0.6
0.4
0.2
0.0
Intensity
y (5)
y (3)
b (4)
b (7)
500
700
900
1100
+MS2(553.2728), 30.2eV, 29.9min #13900

FHAD1_HUMAN

Accession: FHAD1_HUMAN
Database: SwissProt
MW [kDa] / pI: 161.8 / 6.5
Score: 37.8
Seq. Coverage [%]: 0.6
No. of Peptides: 1

Peptide Match

Sequence: K.IEGEIATLK.D
Score: 21
Modifications:

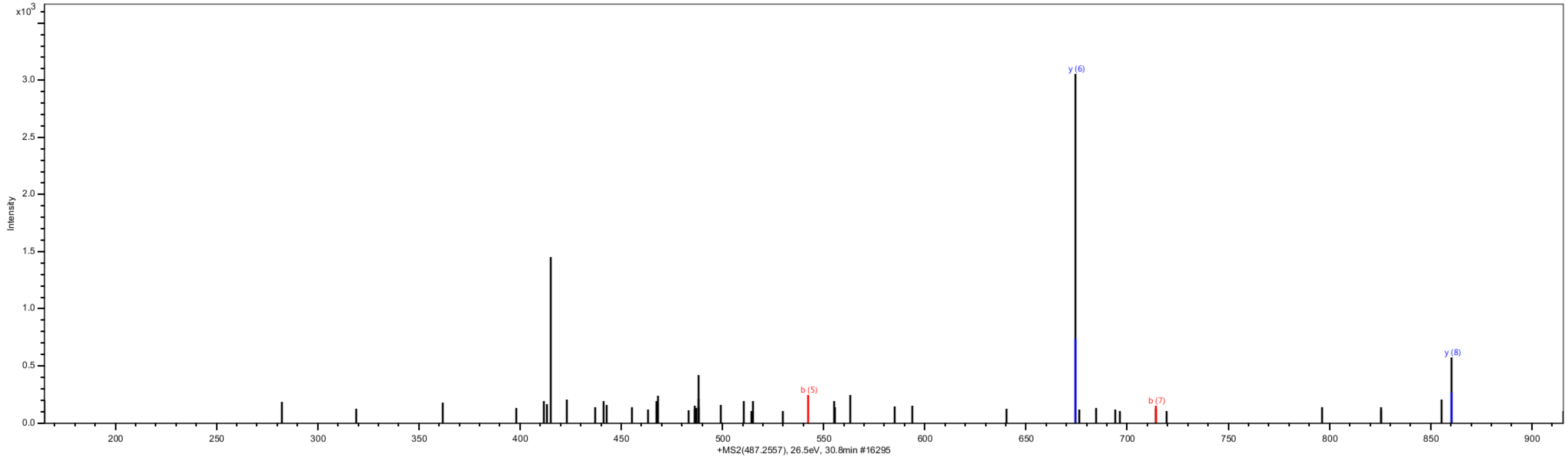

PRR11_HUMAN

Accession: PRR11_HUMAN
Database: SwissProt
MW [kDa] / pI: 40.1 / 10.1
Score: 38.2
Seq. Coverage [%]: 2.5
No. of Peptides: 1

Peptide Match

Sequence: K.DGPMQITVK.D
Score: 15.7
Modifications:

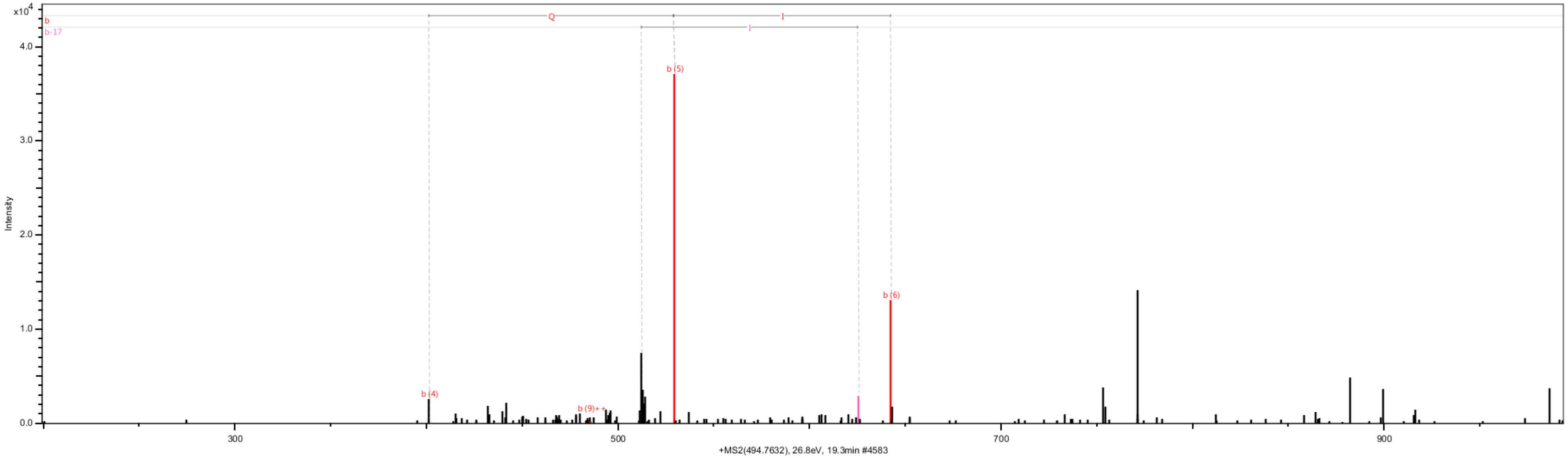

AR6P4_HUMAN

Accession: AR6P4_HUMAN
Database: SwissProt
MW [kDa] / pI: 44.9 / 10.9
Score: 38.2
Seq. Coverage [%]: 6.4
No. of Peptides: 2

Peptide Match

Sequence: R.KASTAPGAEASPSPCITER.S
Score: 19
Modifications: Carbamidomethyl: 15

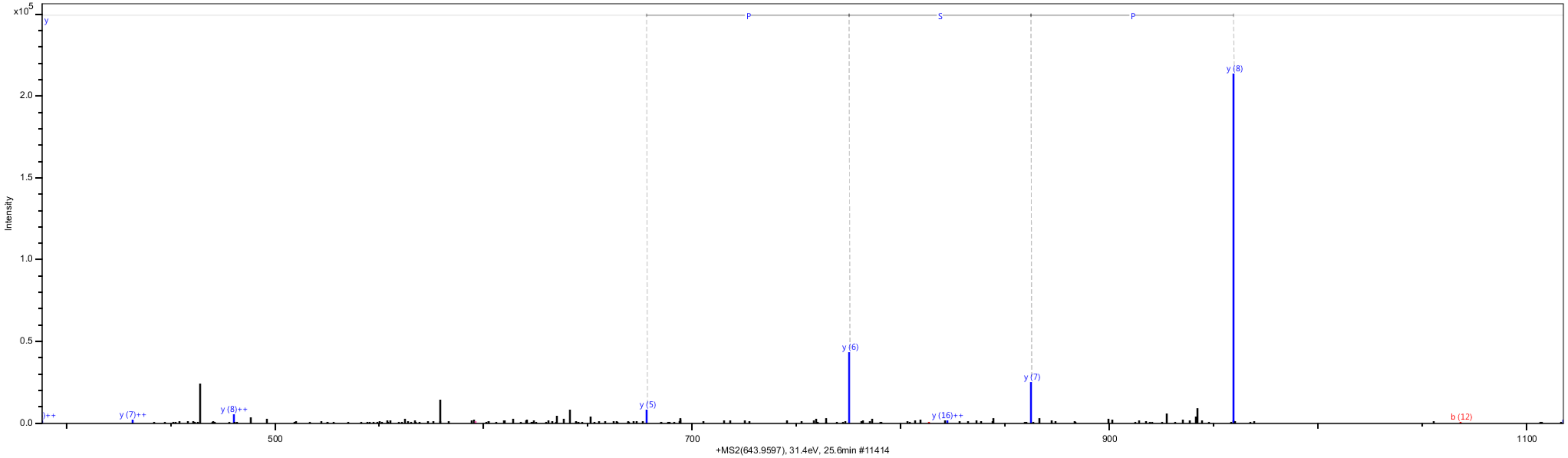

Peptide Match

Sequence: R.EINKQATR.G
Score: 19.2
Modifications:

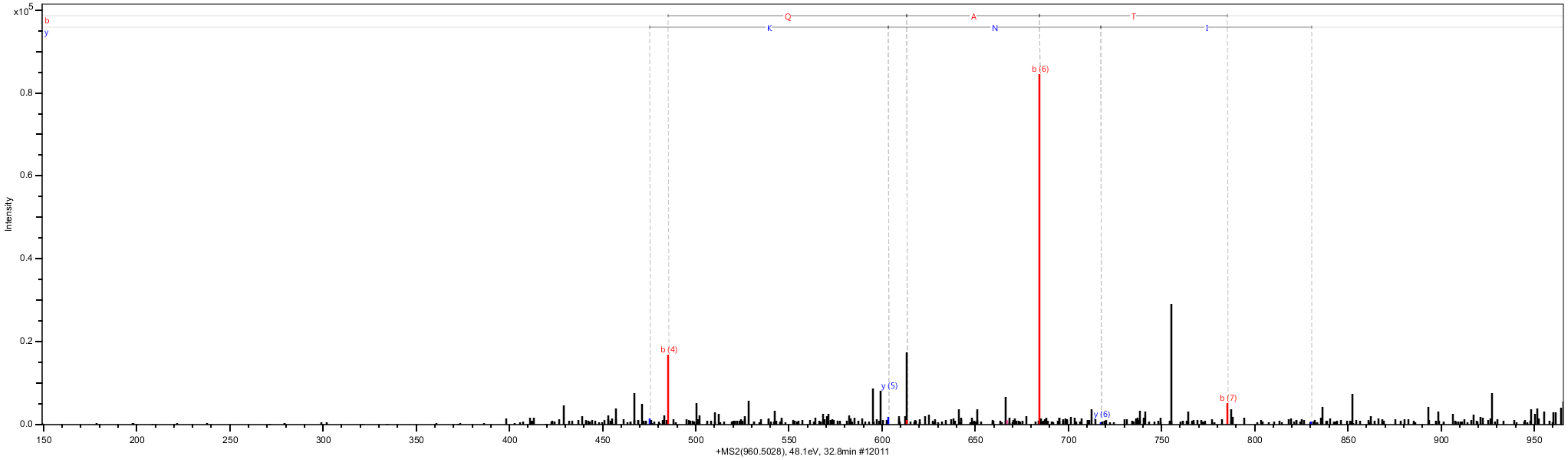

x10^5
Intensity
b
y
Q
A
T
K
N
I
b (4)
y (5)
b (6)
y (6)
b (7)
+MS2(960.5028), 48.1eV, 32.8min #12011

OSBL6_HUMAN

Accession: OSBL6_HUMAN
Database: SwissProt
MW [kDa] / pI: 106.2 / 6.5
Score: 38.4
Seq. Coverage [%]: 2.1
No. of Peptides: 2

Peptide Match

Sequence: R.TASSSTEPSVSR.Q
Score: 18
Modifications:

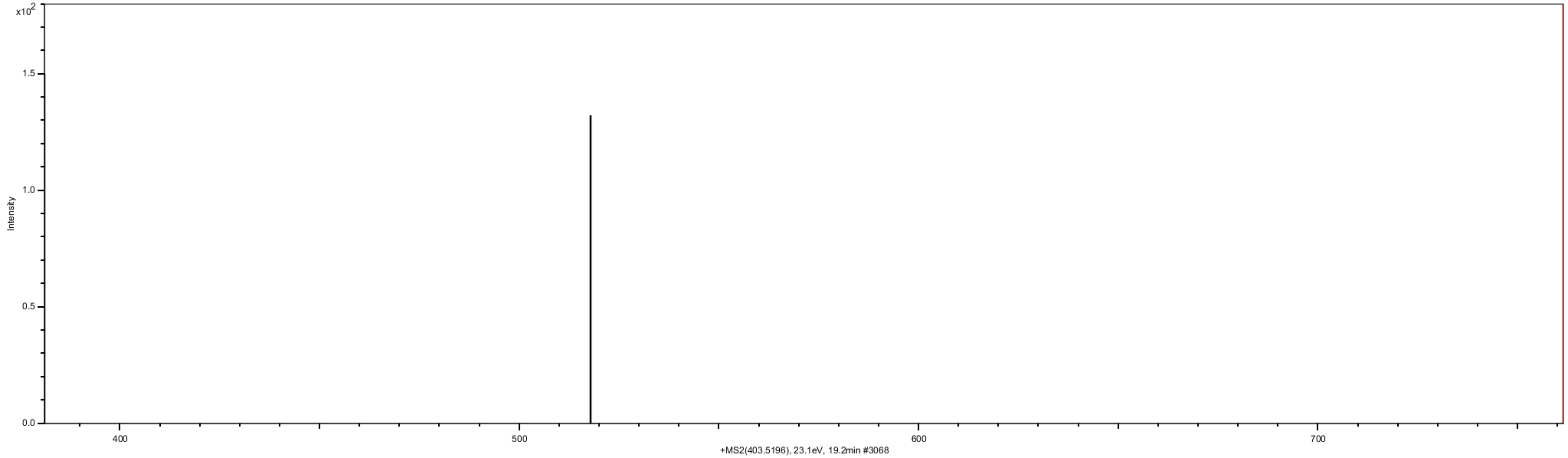

Peptide Match

Sequence: K.QRVEELQR.S
Score: 20.4
Modifications:

Spectrum Report
Combine Search_2020-10-19 07:53:31

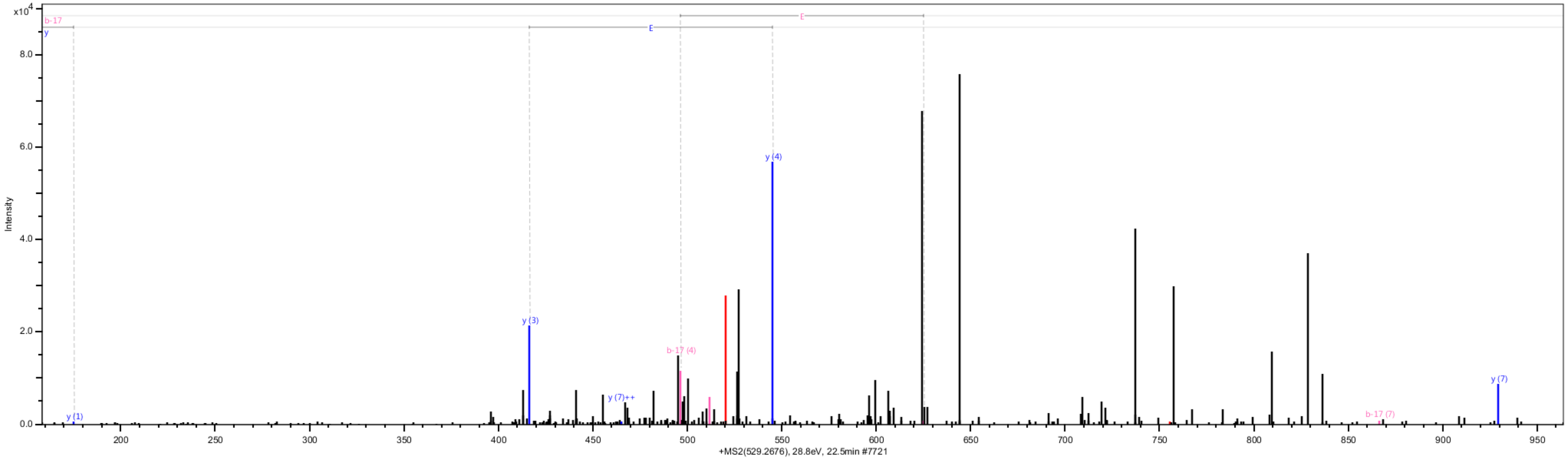

SKT_HUMAN

**Accession:**	SKT_HUMAN	**Score:**	38.3
**Database:**	SwissProt	**Seq. Coverage [%]:**	1.0
**MW [kDa] / pI:**	214.0 / 6.6	**No. of Peptides:**	2

Peptide Match

**Sequence:**	R.RQMQEQGK.G	**Score:**	17.6
**Modifications:**			

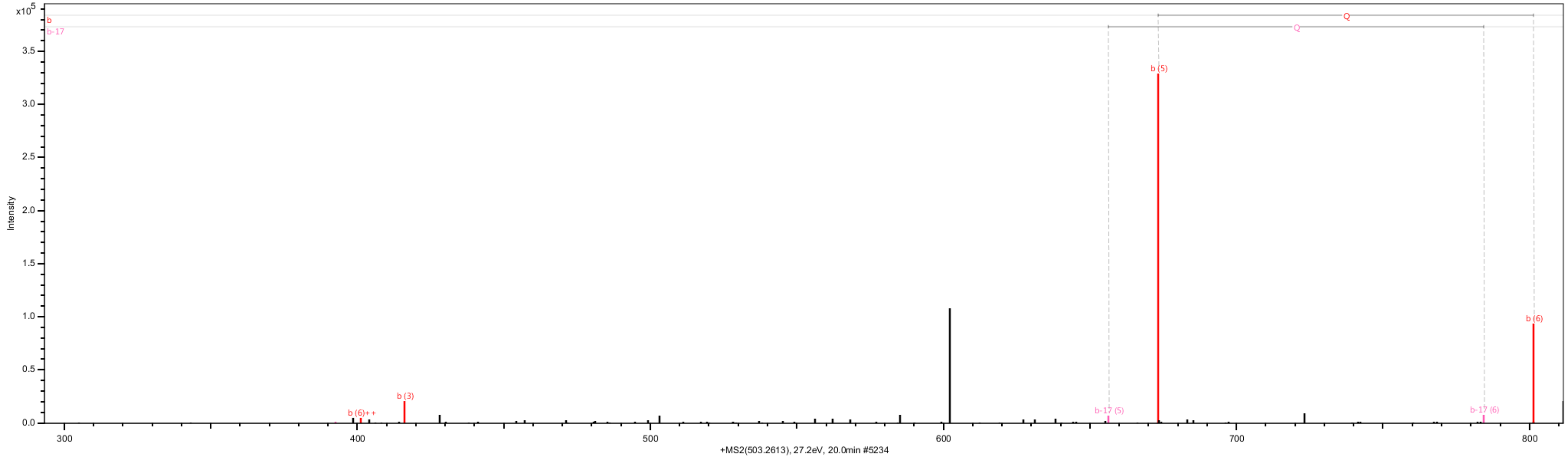

Peptide Match

**Sequence:**	R.DVKPDEDMSGK.N	**Score:**	20.7
**Modifications:**	Oxidation: 8		

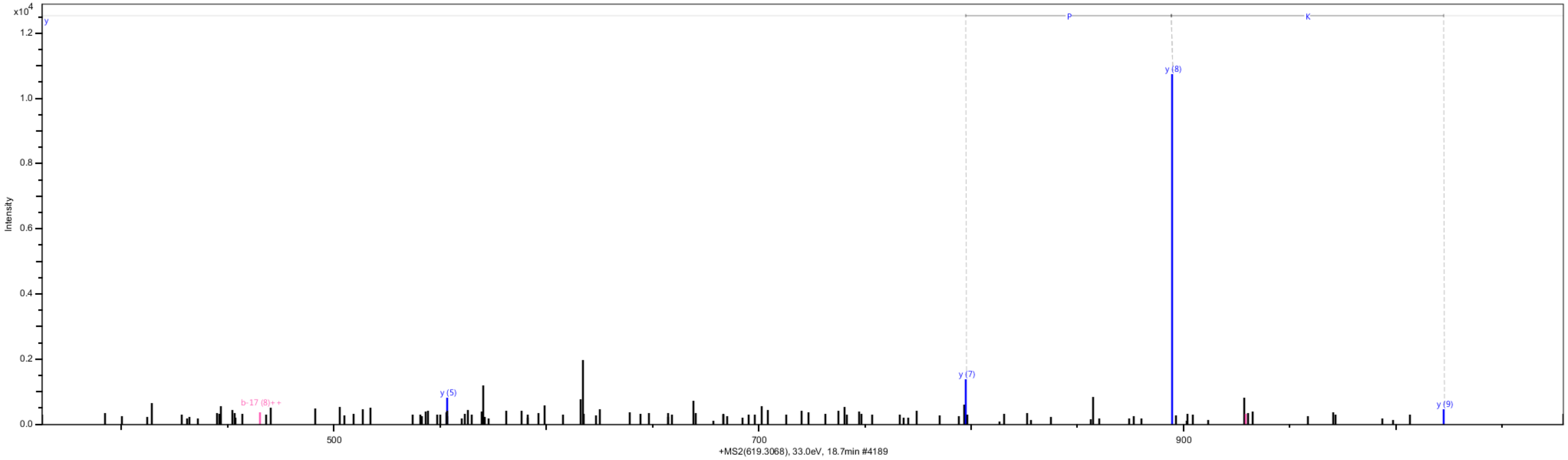
x10^4
Intensity
1.2
1.0
0.8
0.6
0.4
0.2
0.0
P
K
y (8)
y (7)
y (5)
y (9)
b-17 (8)++
500
700
900
+MS2(619.3068), 33.0eV, 18.7min #4189

S26A6_HUMAN

Accession: S26A6_HUMAN
Database: SwissProt
MW [kDa] / pI: 82.9 / 8.6
Score: 38.6
Seq. Coverage [%]: 1.3
No. of Peptides: 2

Peptide Match

Sequence: -.MGLADASGPR.D
Score: 22.5
Modifications: Oxidation: 1

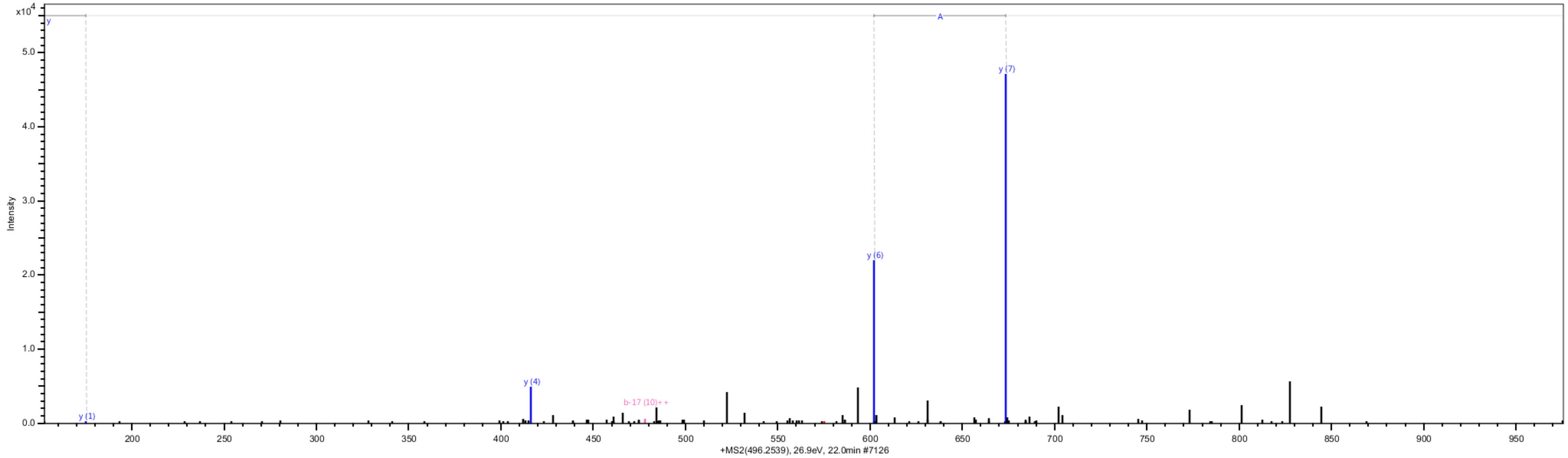

Peptide Match

Sequence: M.GLADASGPR.D
Score: 16.2
Modifications:

Spectrum Report
Combine Search_2020-10-19 07:53:31

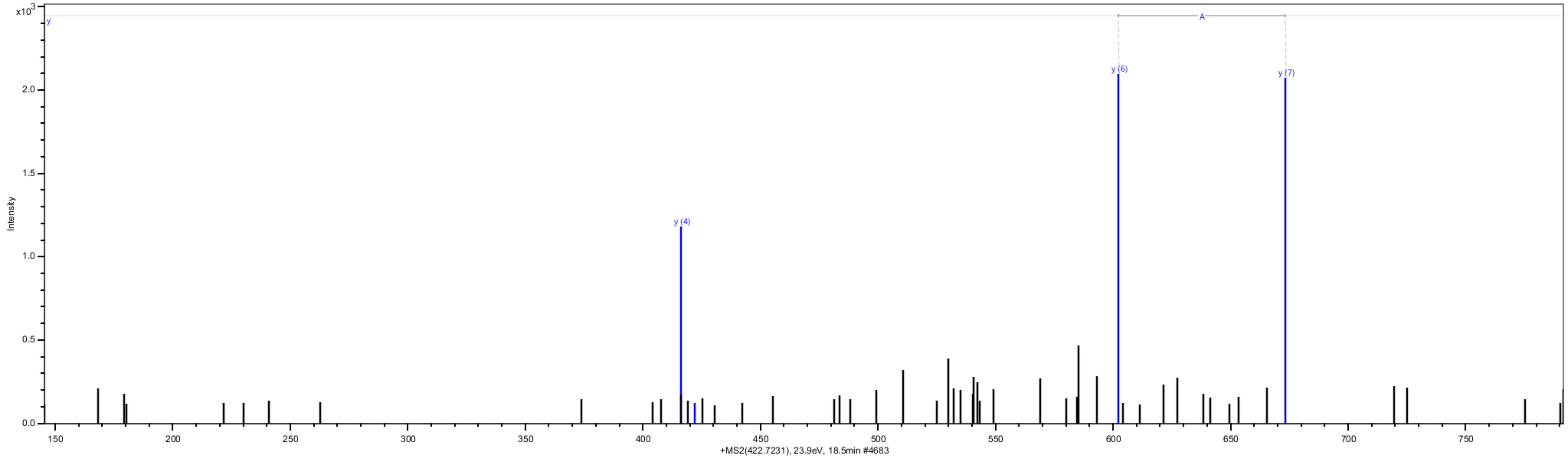

CP19A_HUMAN

Accession: CP19A_HUMAN
Database: SwissProt
MW [kDa] / pI: 57.8 / 7.2
Score: 38.5
Seq. Coverage [%]: 2.6
No. of Peptides: 2

Peptide Match

Sequence: K.LKVMENFIYESMR.Y
Score: 18.4
Modifications: Oxidation: 4, 12

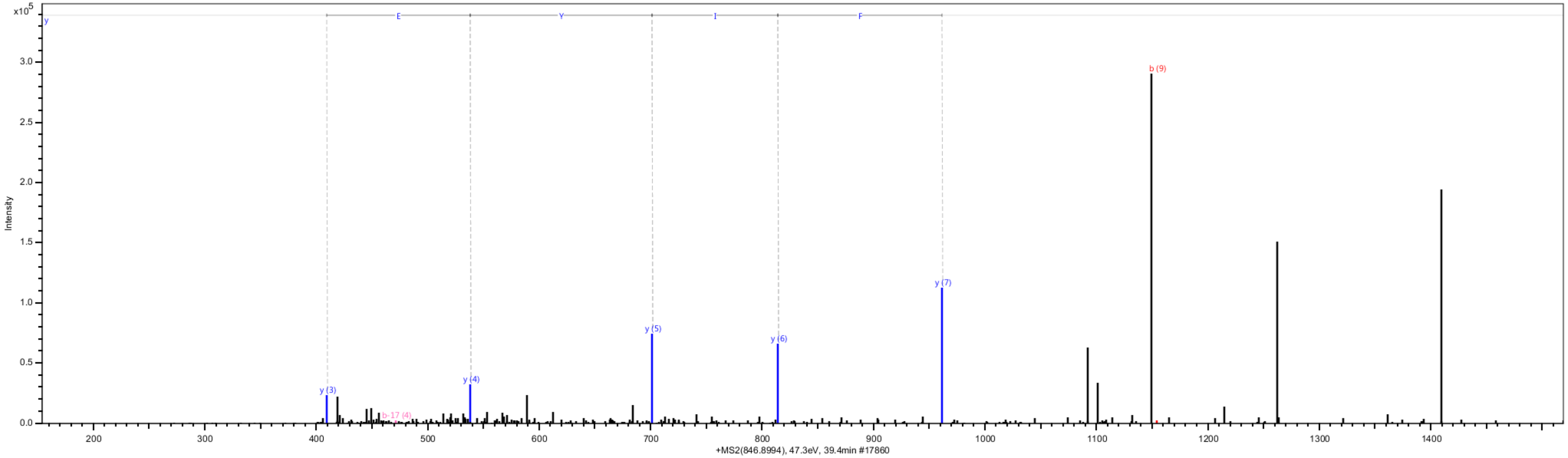

Peptide Match

Sequence: K.VMENFIYESMR.Y
Score: 20.2
Modifications: Oxidation: 10

Spectrum Report
Combine Search_2020-10-19 07:53:31

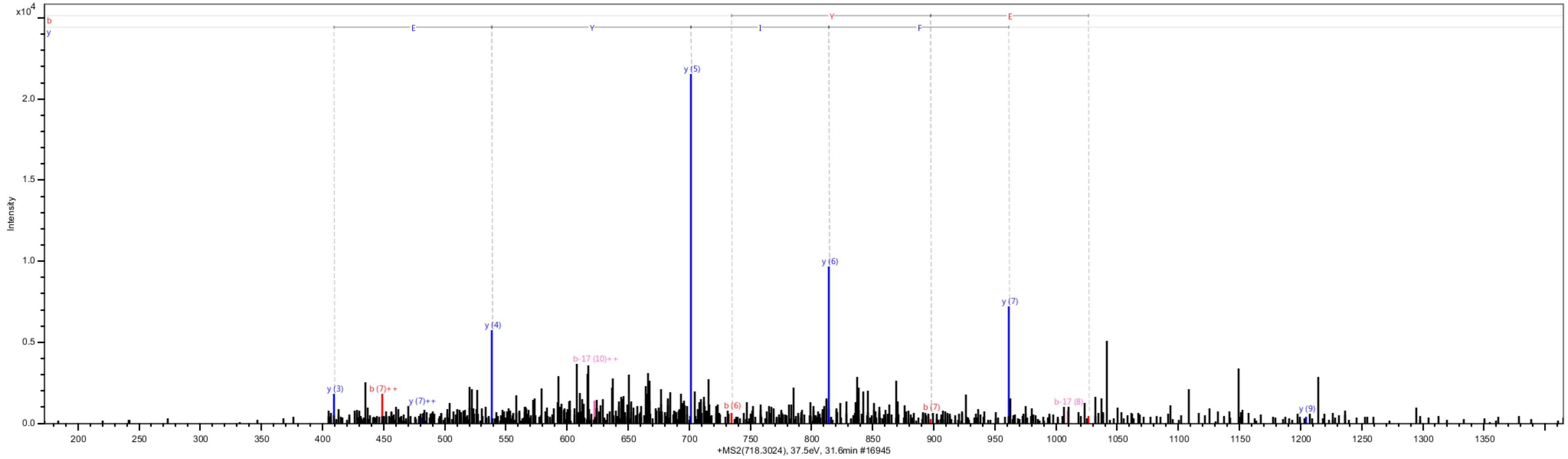

PKHG1_HUMAN

**Accession:**	PKHG1_HUMAN	**Score:**	39.0
**Database:**	SwissProt	**Seq. Coverage [%]:**	1.2
**MW [kDa] / pI:**	155.3 / 5.8	**No. of Peptides:**	2

Peptide Match

Sequence: R.TYVQDLK.S **Score:** 21.9

Modifications:

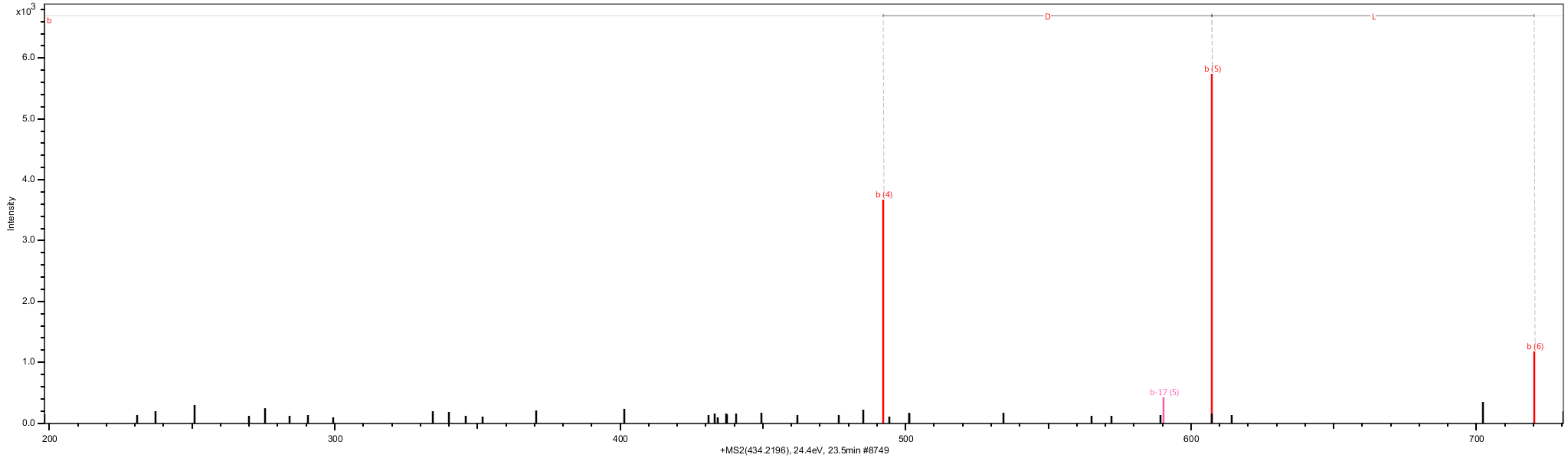

Peptide Match

Sequence: K.REEAESTPSK.S **Score:** 17

Modifications:

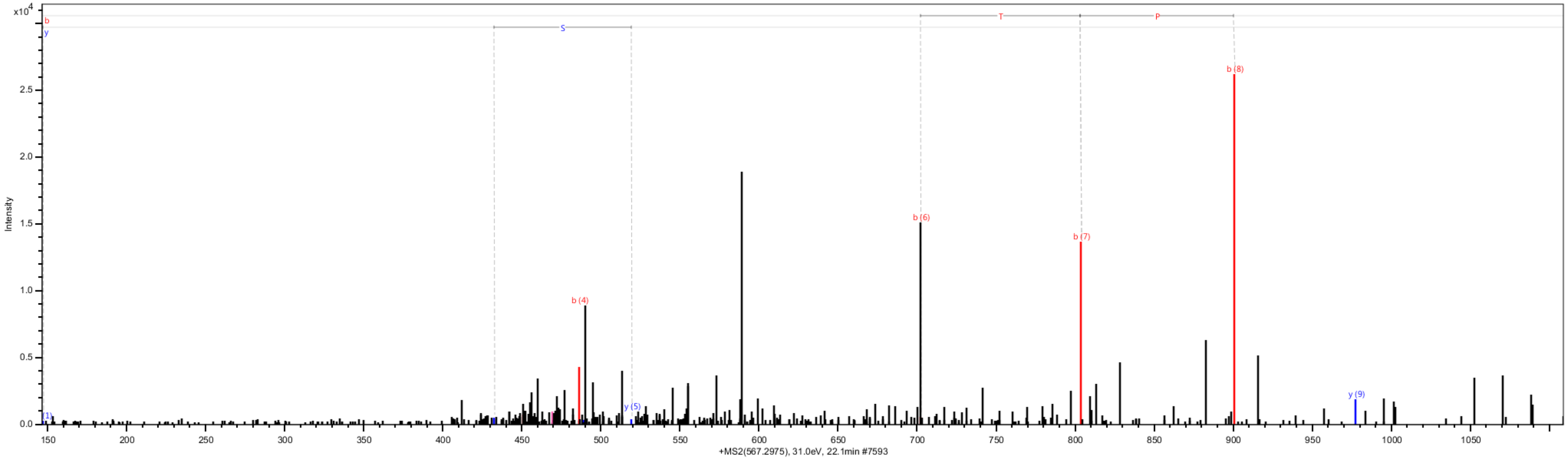
x10^4
Intensity
b
y
S
T
P
b (4)
b (6)
b (7)
b (8)
y (1)
y (5)
y (9)
0.0
0.5
1.0
1.5
2.0
2.5
150
200
250
300
350
400
450
500
550
600
650
700
750
800
850
900
950
1000
1050
+MS2(567.2975), 31.0eV, 22.1min #7593

ASTN2_HUMAN

Accession: ASTN2_HUMAN
Database: SwissProt
MW [kDa] / pI: 148.1 / 5.7
Score: 38.8
Seq. Coverage [%]: 1.3
No. of Peptides: 2

Peptide Match

Sequence: K.ETTELGSK.K
Score: 15.2
Modifications:

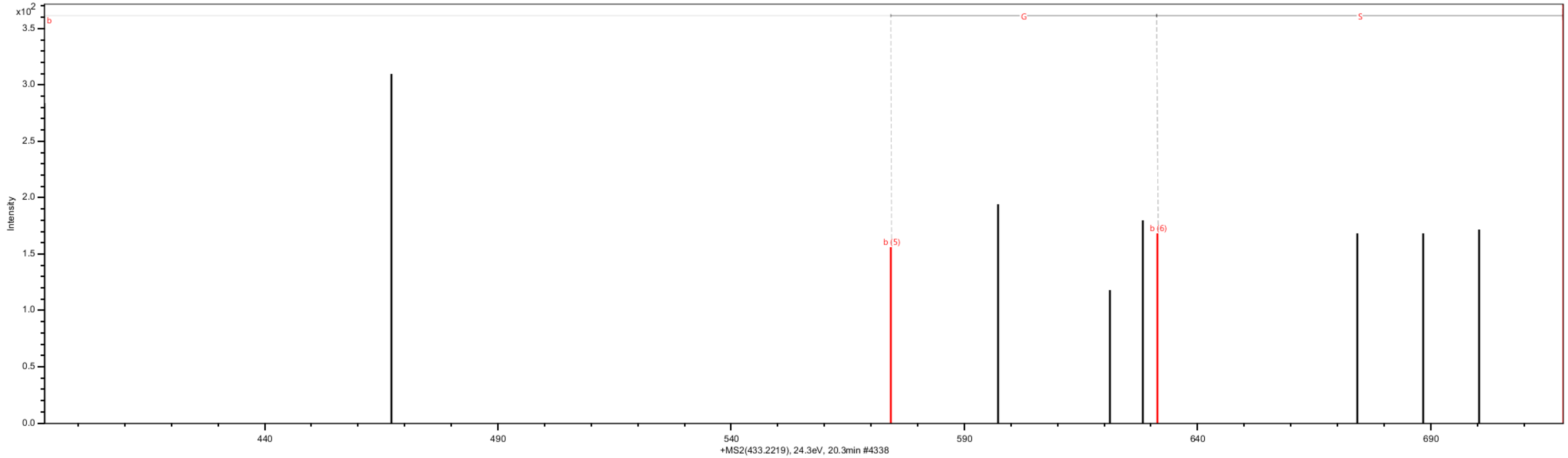

Peptide Match

Sequence: R.TACPLVDDNK.A
Score: 23.5
Modifications: Carbamidomethyl: 3

Spectrum Report
Combine Search_2020-10-19 07:53:31

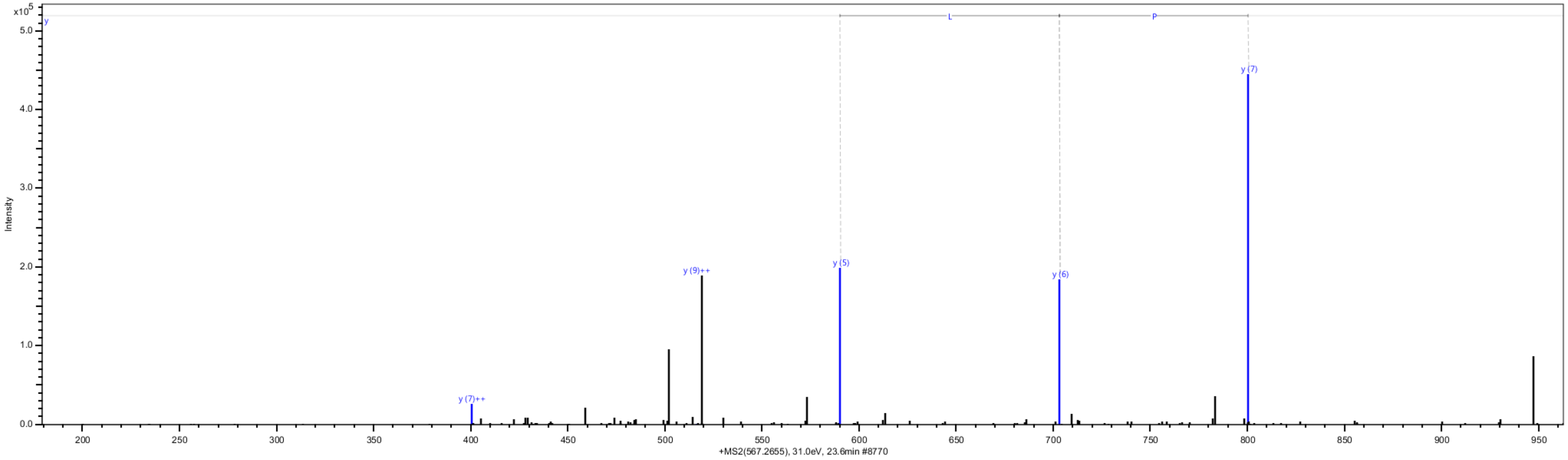

PEDF_HUMAN

Accession: PEDF_HUMAN
Database: SwissProt
MW [kDa] / pI: 46.3 / 6.0
Score: 39.2
Seq. Coverage [%]: 6.2
No. of Peptides: 2

Peptide Match

Sequence: K.LAAAVSNFGYDLYR.V
Score: 20.4
Modifications:

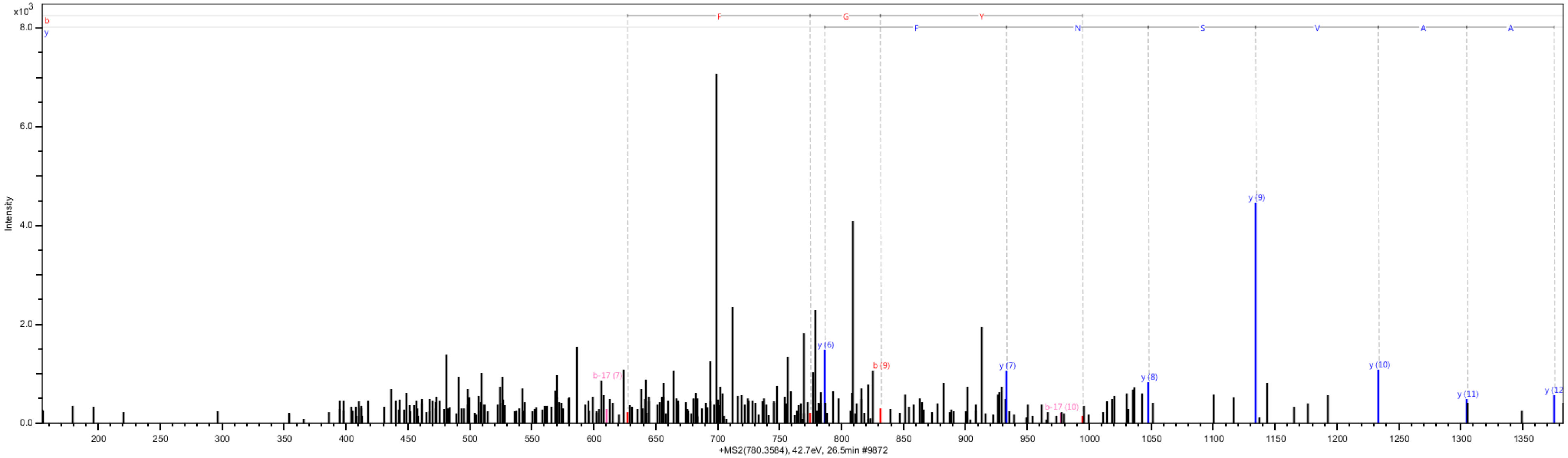

Peptide Match

Sequence: K.LQSLFDSPDFSK.I
Score: 18.8
Modifications:

Spectrum Report
Combine Search_2020-10-19 07:53:31

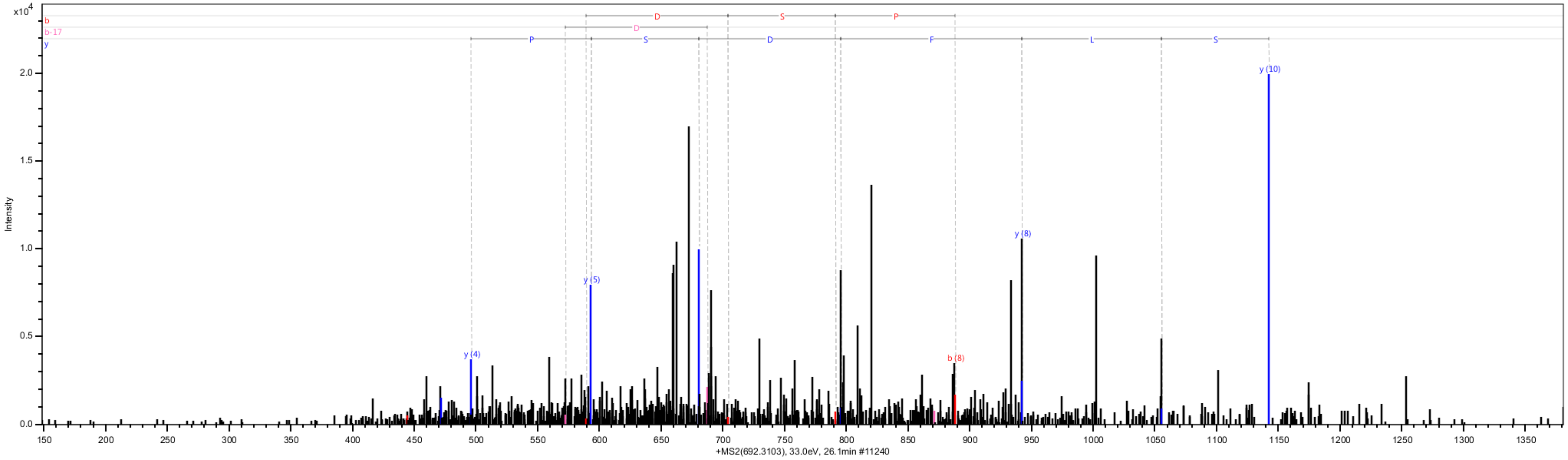

RGS9_HUMAN

Accession: RGS9_HUMAN
Database: SwissProt
MW [kDa] / pI: 76.9 / 9.4
Score: 39.8
Seq. Coverage [%]: 3.1
No. of Peptides: 2

Peptide Match

Sequence: K.GILEEYEK.E
Score: 16.8
Modifications:

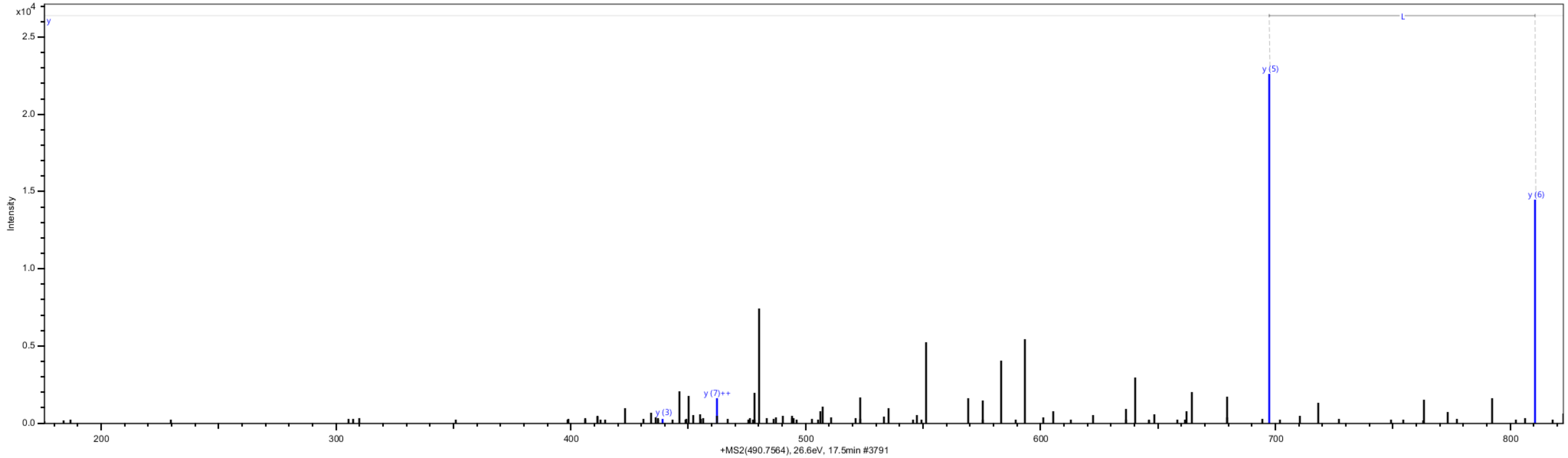

Peptide Match

Sequence: K.ENYNFLNQKMNYK.W
Score: 23
Modifications: Oxidation: 10

Spectrum Report
Combine Search_2020-10-19 07:53:31

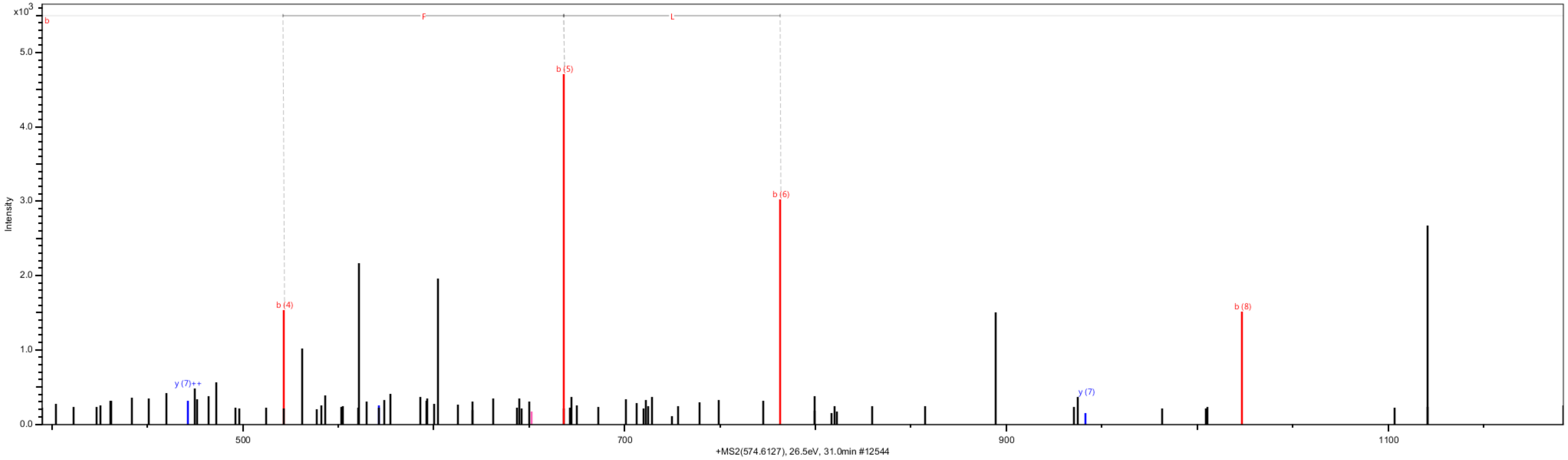

SPTN4_HUMAN

Accession: SPTN4_HUMAN
Database: SwissProt
MW [kDa] / pI: 288.8 / 5.7
Score: 39.5
Seq. Coverage [%]: 0.3
No. of Peptides: 1

Peptide Match

Sequence: K.WVNSHLAR.V
Score: 15.1
Modifications:

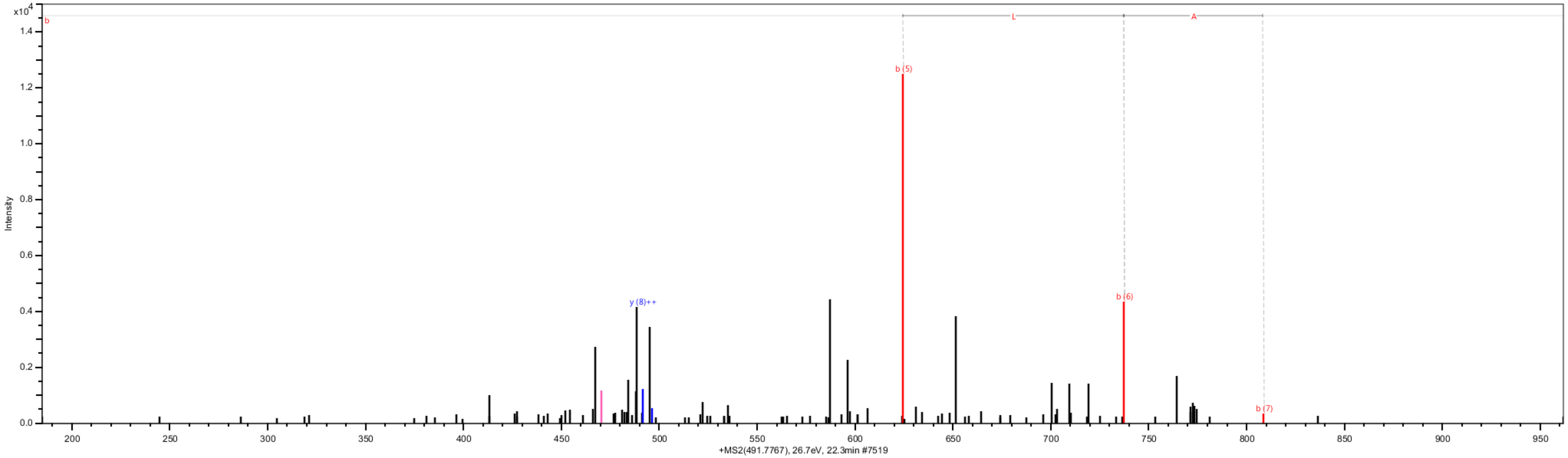

HV551_HUMAN

Accession: HV551_HUMAN
Database: SwissProt
MW [kDa] / pI: 12.7 / 8.4
Score: 24.6
Seq. Coverage [%]: 11.1
No. of Peptides: 1

Peptide Match

Sequence: K.SISTAYLQWSSLK.A
Score: 24.6
Modifications:

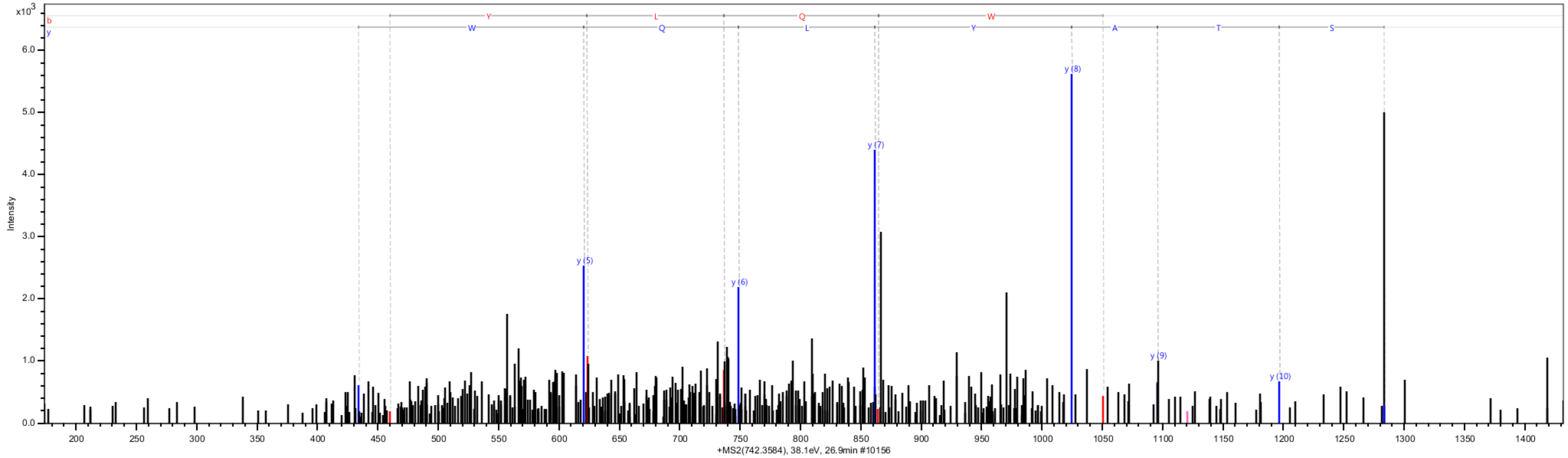

PTPRM_HUMAN

**Accession:**	PTPRM_HUMAN	**Score:**	24.5
**Database:**	SwissProt	**Seq. Coverage [%]:**	0.8
**MW [kDa] / pI:**	163.6 / 6.2	**No. of Peptides:**	1

Peptide Match

**Sequence:**	R.VEDCSIALLPR.N	**Score:**	24.5
**Modifications:**	Carbamidomethyl: 4		

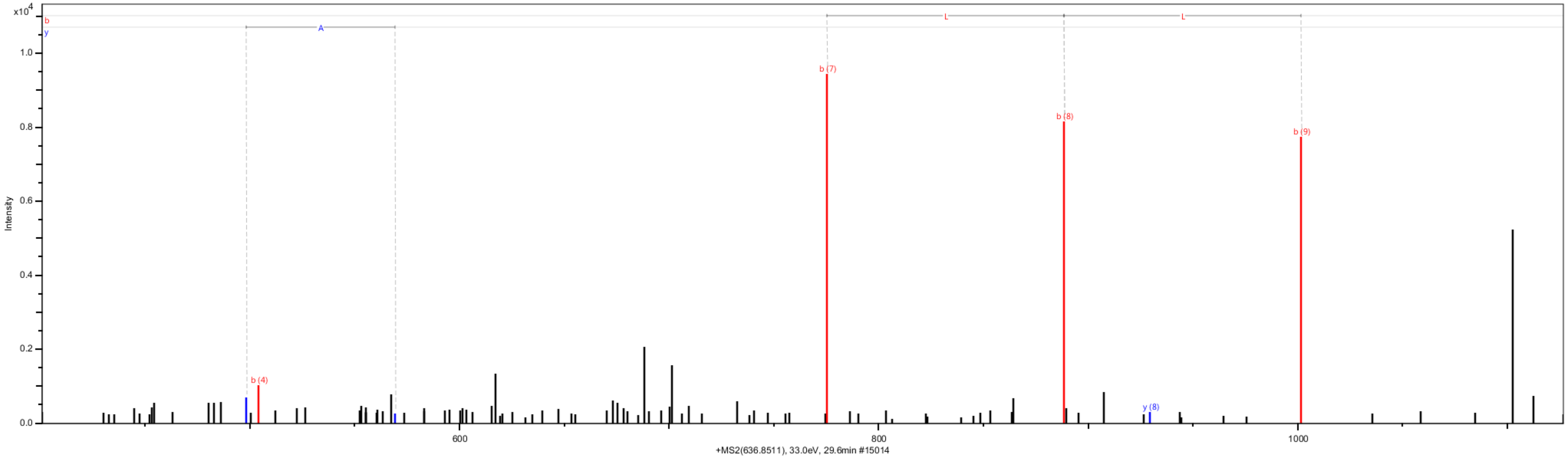

CSC2A_HUMAN

**Accession:**	CSC2A_HUMAN	**Score:**	24.5
**Database:**	SwissProt	**Seq. Coverage [%]:**	8.8
**MW [kDa] / pI:**	11.9 / 8.5	**No. of Peptides:**	1

Peptide Match

**Sequence:**	K.HFGSALRLR.Q	**Score:**	24.5
**Modifications:**			

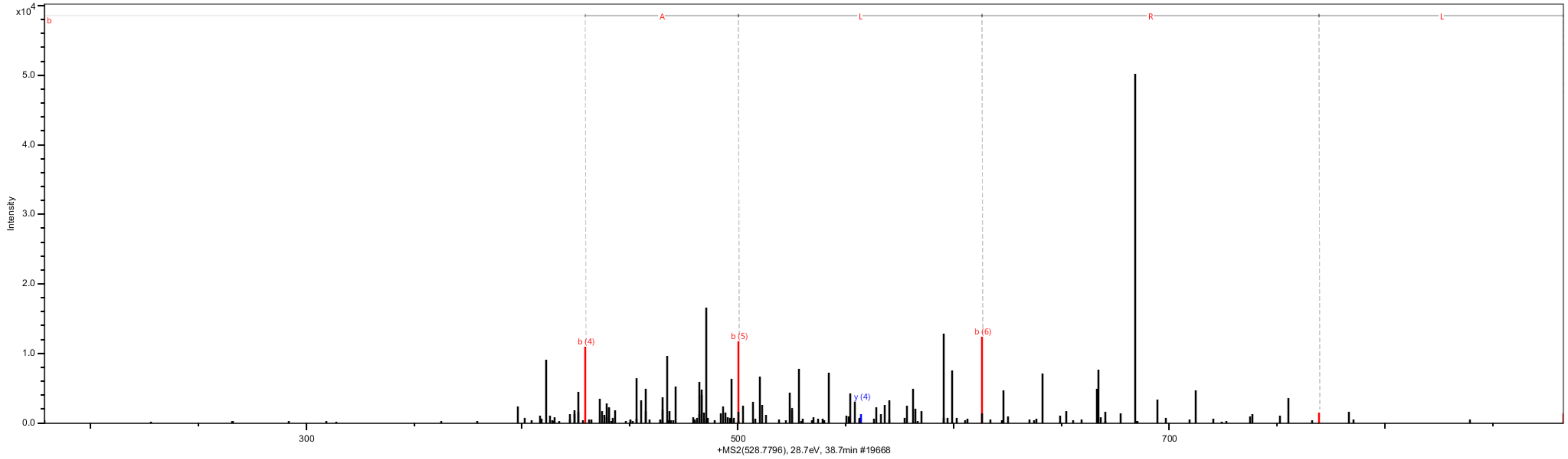

WDR7_HUMAN

Accession: WDR7_HUMAN
Database: SwissProt
MW [kDa] / pI: 163.7 / 6.5
Score: 24.4
Seq. Coverage [%]: 0.5
No. of Peptides: 1

Peptide Match

Sequence: R.IEQAGRK.E
Score: 24.4
Modifications:

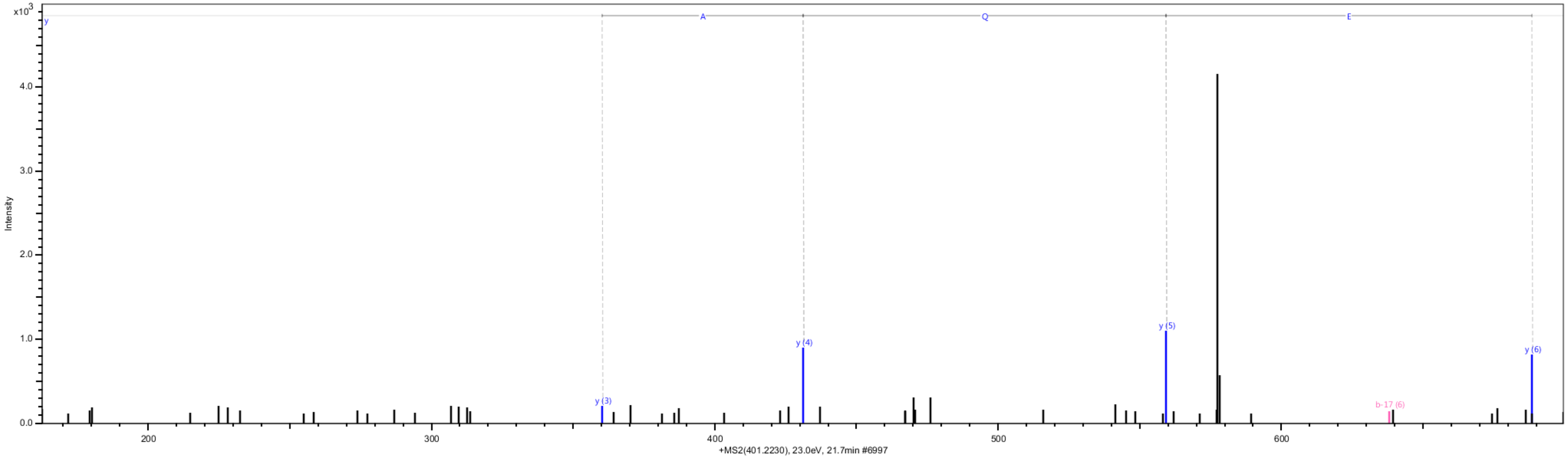

CD22_HUMAN

Accession: CD22_HUMAN
Database: SwissProt
MW [kDa] / pI: 95.3 / 6.2
Score: 24.5
Seq. Coverage [%]: 1.4
No. of Peptides: 1

Peptide Match

Sequence: K.VTTVIQNPMPIR.E
Score: 24.4
Modifications:

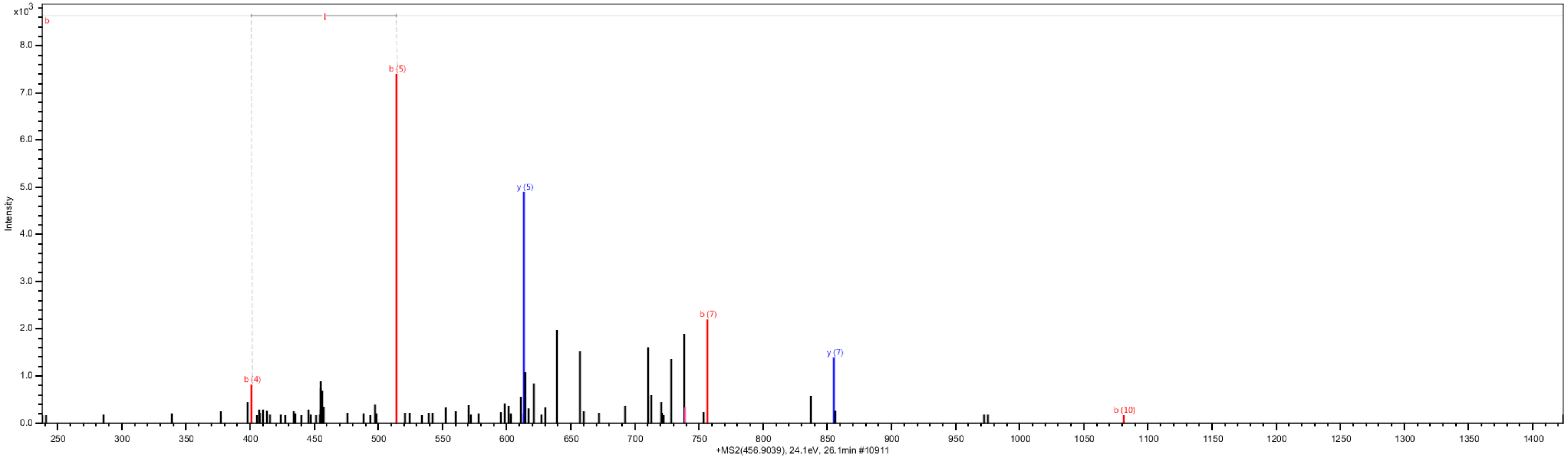

TSN14_HUMAN

Accession: TSN14_HUMAN
Database: SwissProt
MW [kDa] / pI: 30.7 / 6.4
Score: 24.3
Seq. Coverage [%]: 3.0
No. of Peptides: 1

Peptide Match

Sequence: K.GVLSDLTK.V
Score: 24.3
Modifications:

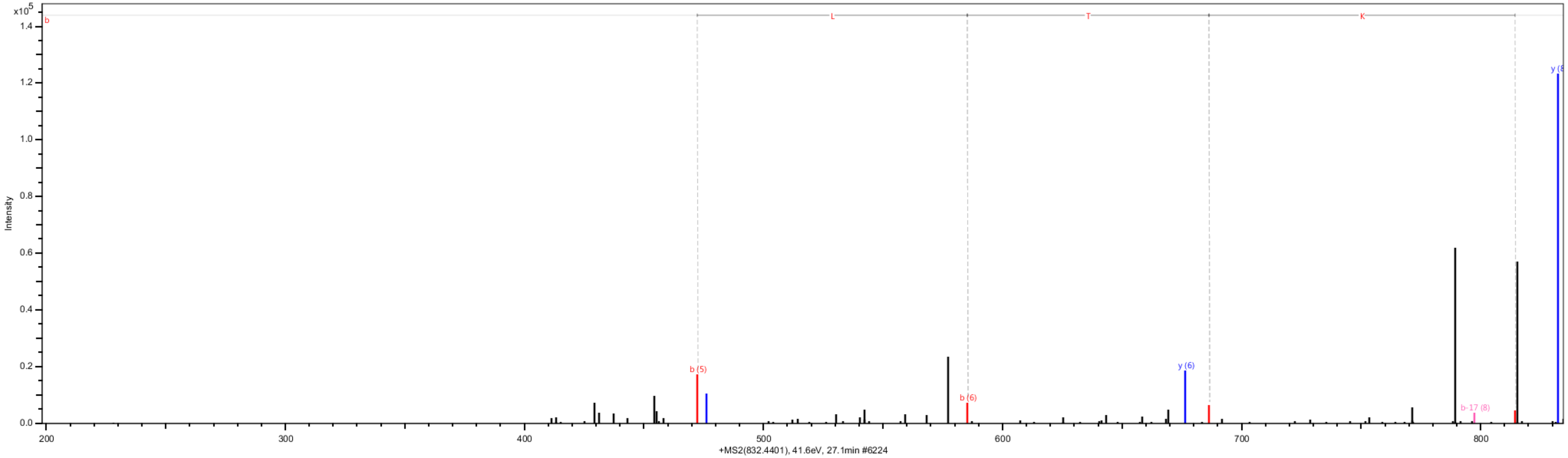

ZN554_HUMAN

Accession: ZN554_HUMAN
Database: SwissProt
MW [kDa] / pI: 60.5 / 7.6
Score: 24.4
Seq. Coverage [%]: 1.5
No. of Peptides: 1

Peptide Match

Sequence: R.SSLVRHER.T
Score: 24.4
Modifications:

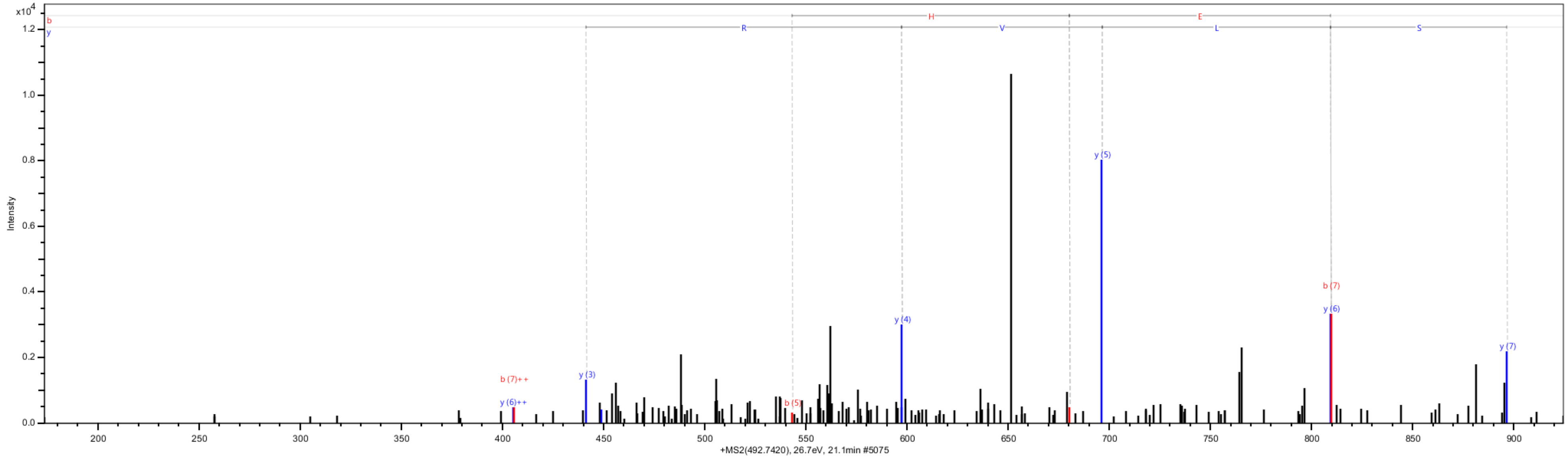

SPT6H_HUMAN

**Accession:**	SPT6H_HUMAN	**Score:**	25.3
**Database:**	SwissProt	**Seq. Coverage [%]:**	0.5
**MW [kDa] / pI:**	198.9 / 4.8	**No. of Peptides:**	1

Peptide Match

**Sequence:**	K.VGMTVHCR.I	**Score:**	25.3
**Modifications:**	Carbamidomethyl: 7; Oxidation: 3		

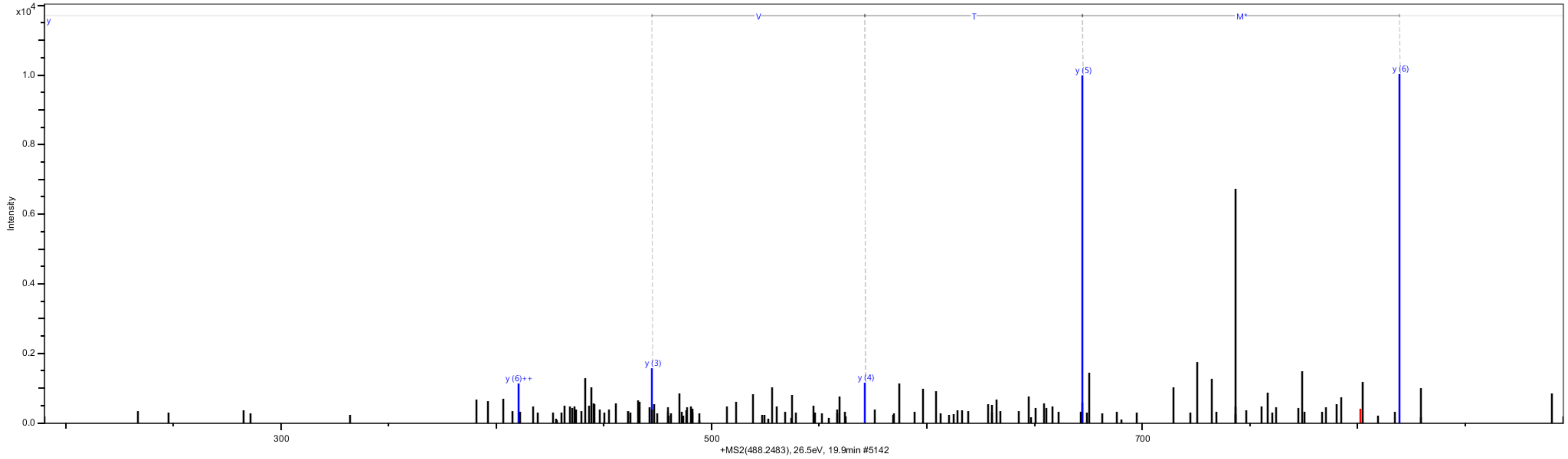

TM100_HUMAN

Accession: TM100_HUMAN
Database: SwissProt
MW [kDa] / pI: 14.4 / 9.6
Score: 25.3
Seq. Coverage [%]: 9.0
No. of Peptides: 1

Peptide Match

Sequence: R.RESQTALVANQR.S
Score: 25.3
Modifications:

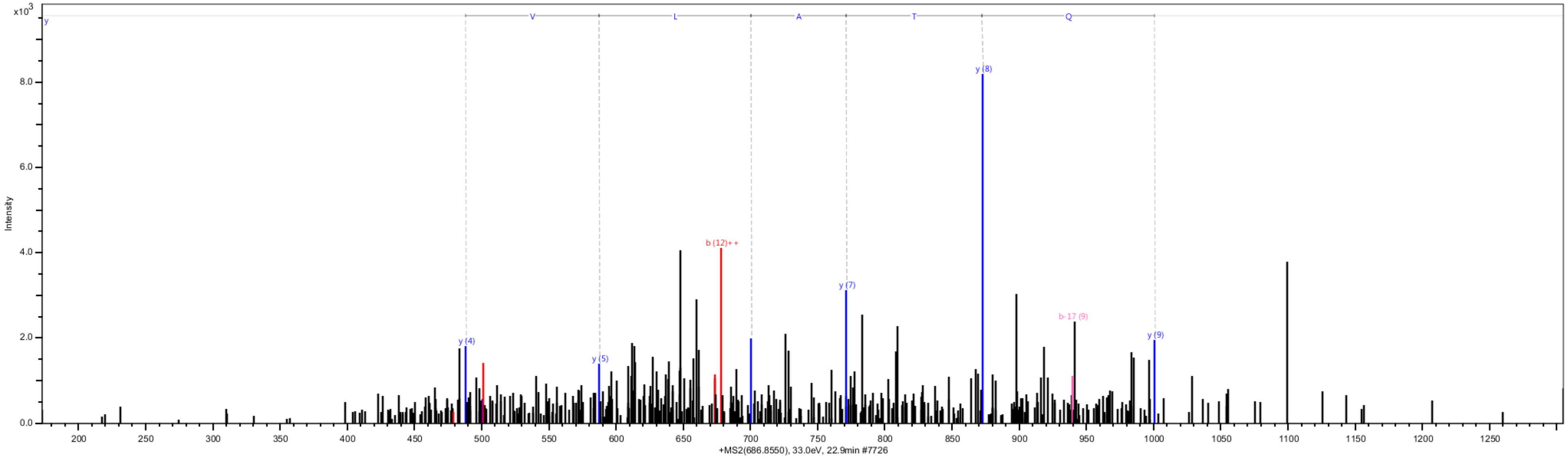

USP9Y_HUMAN

**Accession:**	USP9Y_HUMAN	**Score:**	25.2
**Database:**	SwissProt	**Seq. Coverage [%]:**	0.5
**MW [kDa] / pI:**	290.9 / 5.6	**No. of Peptides:**	1

Peptide Match

**Sequence:**	K.GDLLEGANAYHCEK.C	**Score:**	25.2
**Modifications:**	Carbamidomethyl: 12		

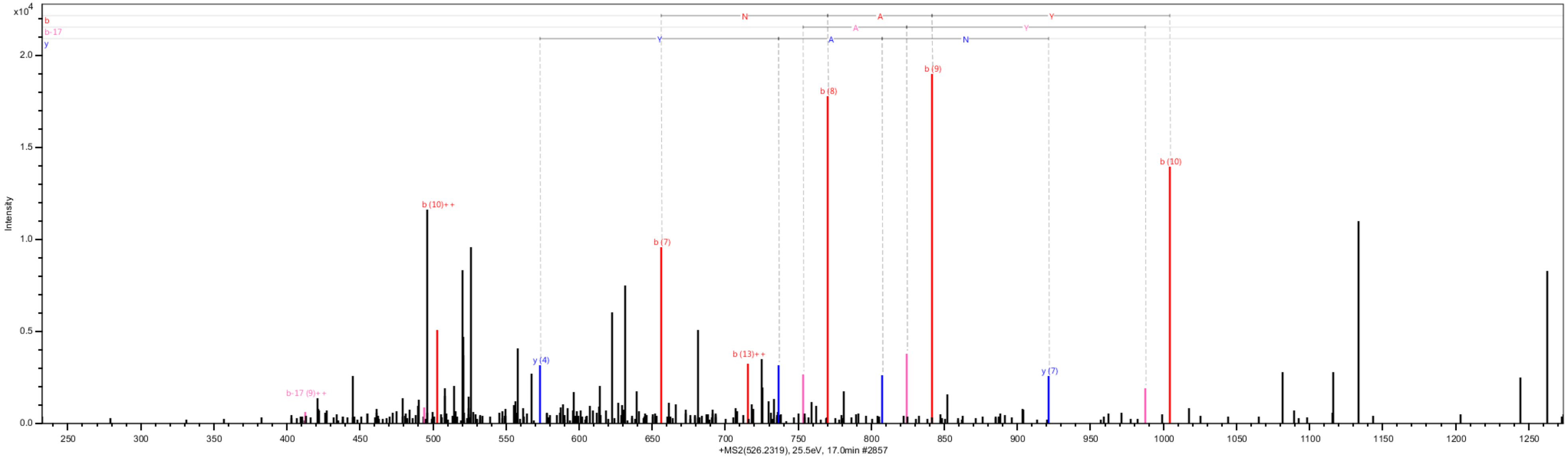

ZN831_HUMAN

Accession: ZN831_HUMAN
Database: SwissProt
MW [kDa] / pI: 177.8 / 8.7
Score: 25.7
Seq. Coverage [%]: 1.0
No. of Peptides: 1

Peptide Match

Sequence: R.TLTSSSPDSKVTEEGR.A
Score: 25.7
Modifications:

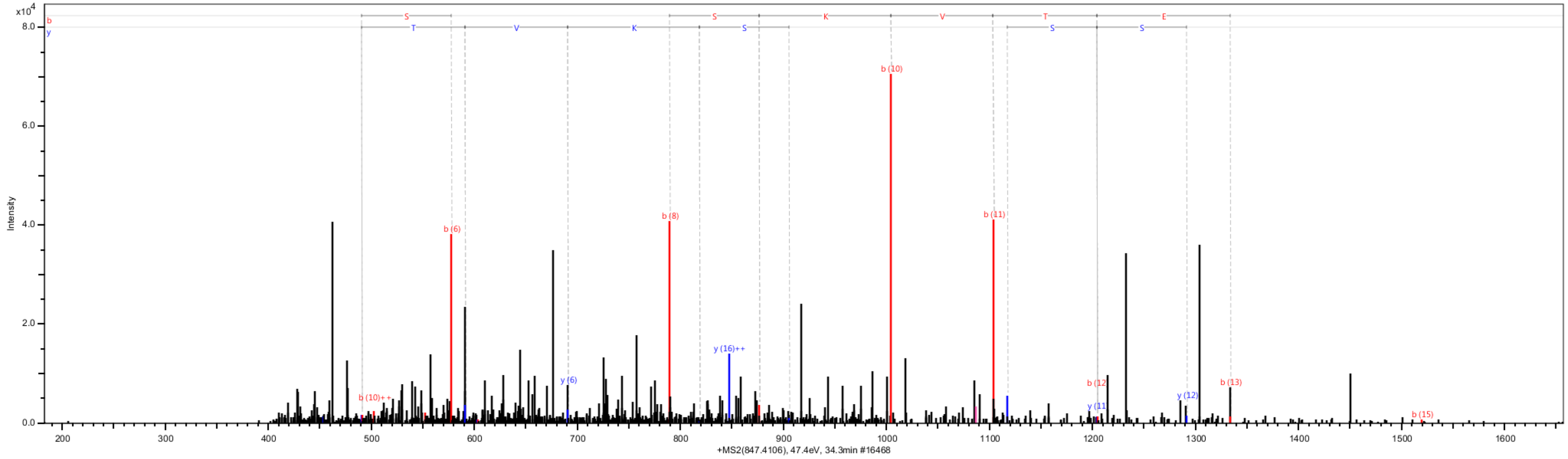

SPKAP_HUMAN

Accession: SPKAP_HUMAN
Database: SwissProt
MW [kDa] / pI: 186.3 / 5.0
Score: 25.7
Seq. Coverage [%]: 0.9
No. of Peptides: 1

Peptide Match

Sequence: R.ASSEGQRSPTVSQSR.S
Score: 25.6
Modifications:

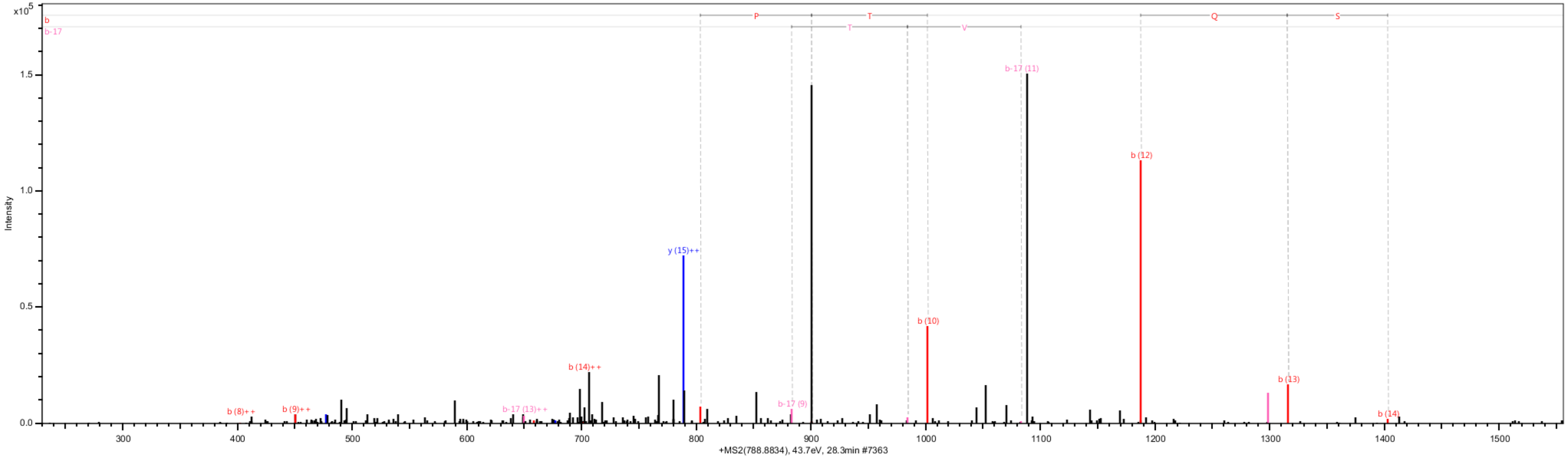

HV313_HUMAN

Accession: HV313_HUMAN
Database: SwissProt
MW [kDa] / pI: 12.5 / 6.5
Score: 25.5
Seq. Coverage [%]: 9.5
No. of Peptides: 1

Peptide Match

Sequence: K.NSLYLQMNSLR.A
Score: 25.5
Modifications:

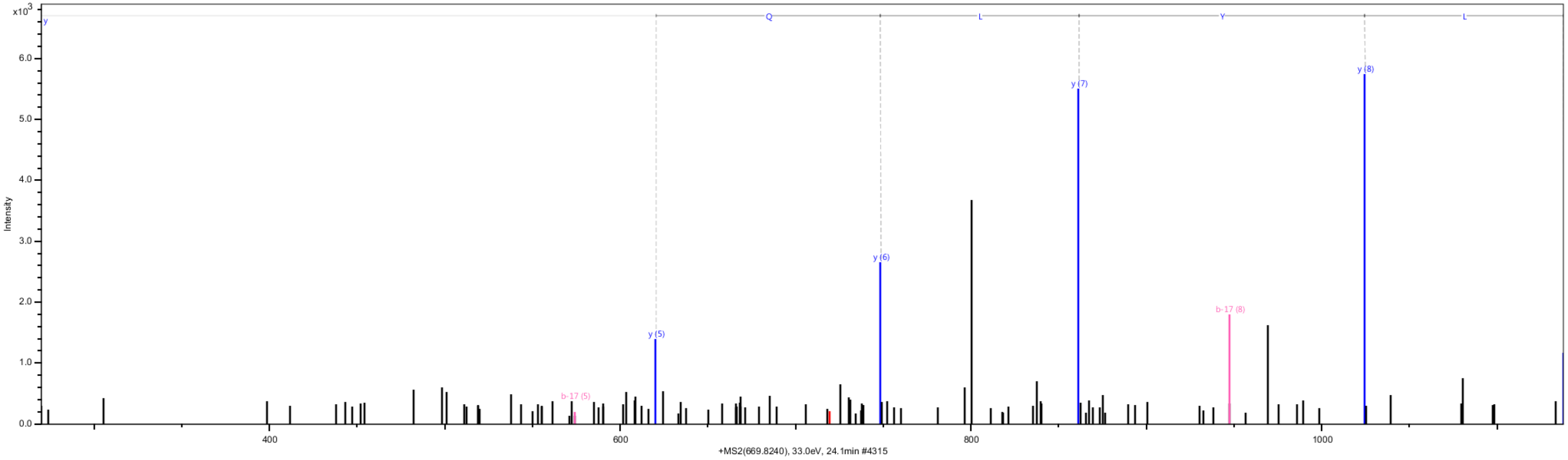

CO1A1_HUMAN

Accession: CO1A1_HUMAN
Database: SwissProt
MW [kDa] / pI: 138.9 / 5.6
Score: 26.0
Seq. Coverage [%]: 0.8
No. of Peptides: 1

Peptide Match

Sequence: R.EGAPGAEGSPGR.D
Score: 26
Modifications:

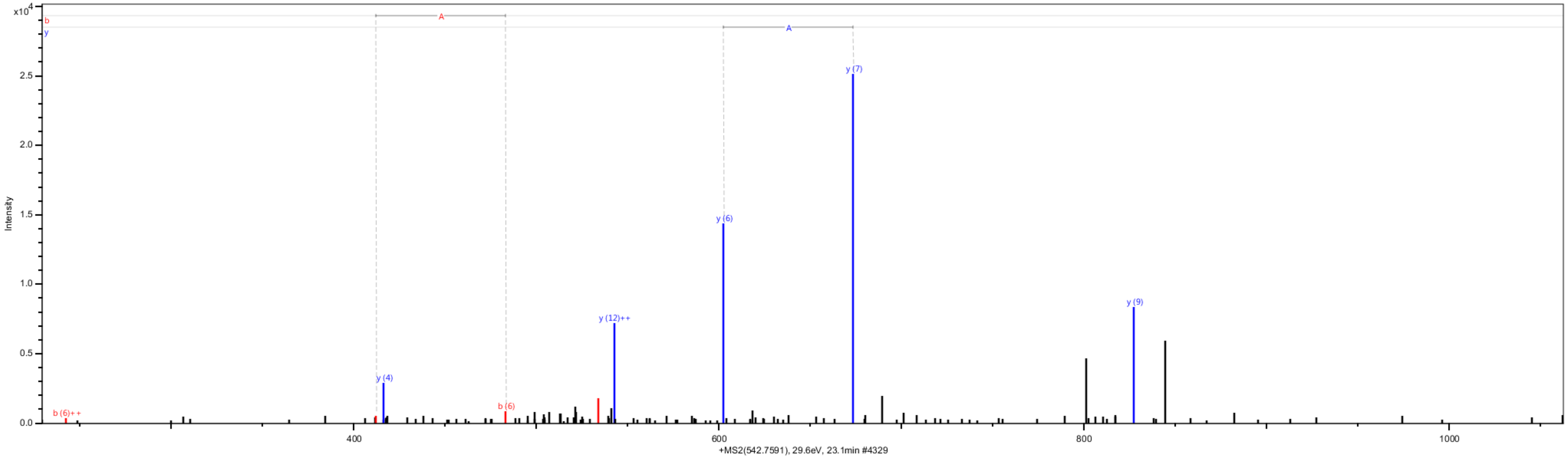

HOIL1_HUMAN

Accession: HOIL1_HUMAN
Database: SwissProt
MW [kDa] / pI: 57.5 / 5.5
Score: 26.0
Seq. Coverage [%]: 2.2
No. of Peptides: 1

Peptide Match

Sequence: R.NTSLNPQELQR.E
Score: 26
Modifications:

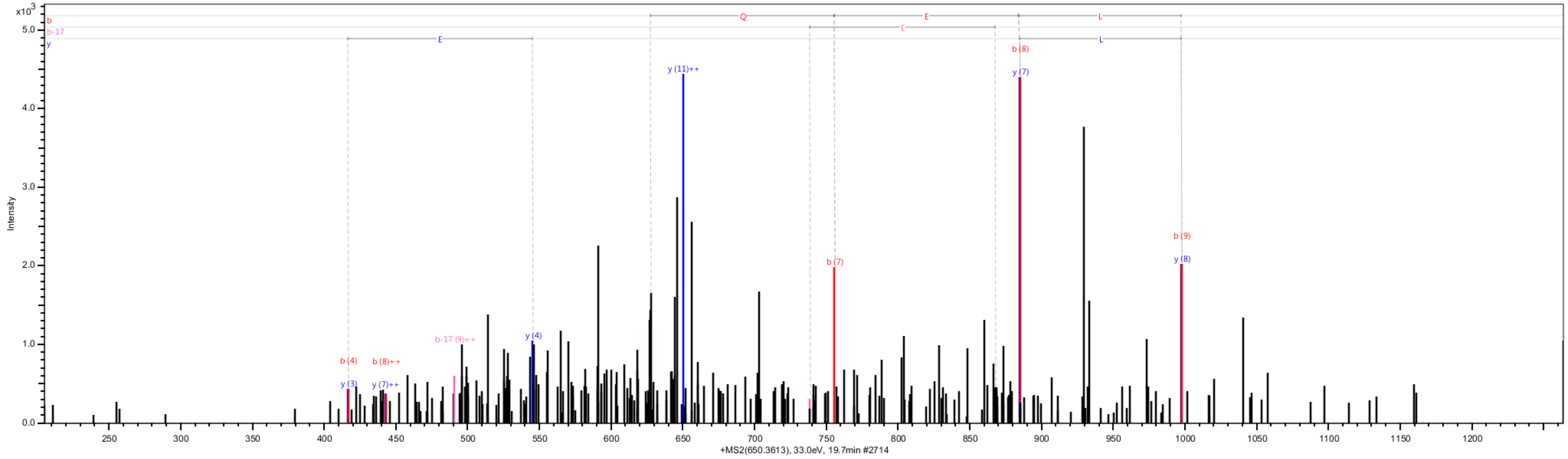

EZHIP_HUMAN

Accession: EZHIP_HUMAN
Database: SwissProt
MW [kDa] / pI: 51.9 / 10.1
Score: 26.0
Seq. Coverage [%]: 2.2
No. of Peptides: 1

Peptide Match

Sequence: R.DSAPVSAPRGR.D
Score: 26
Modifications:

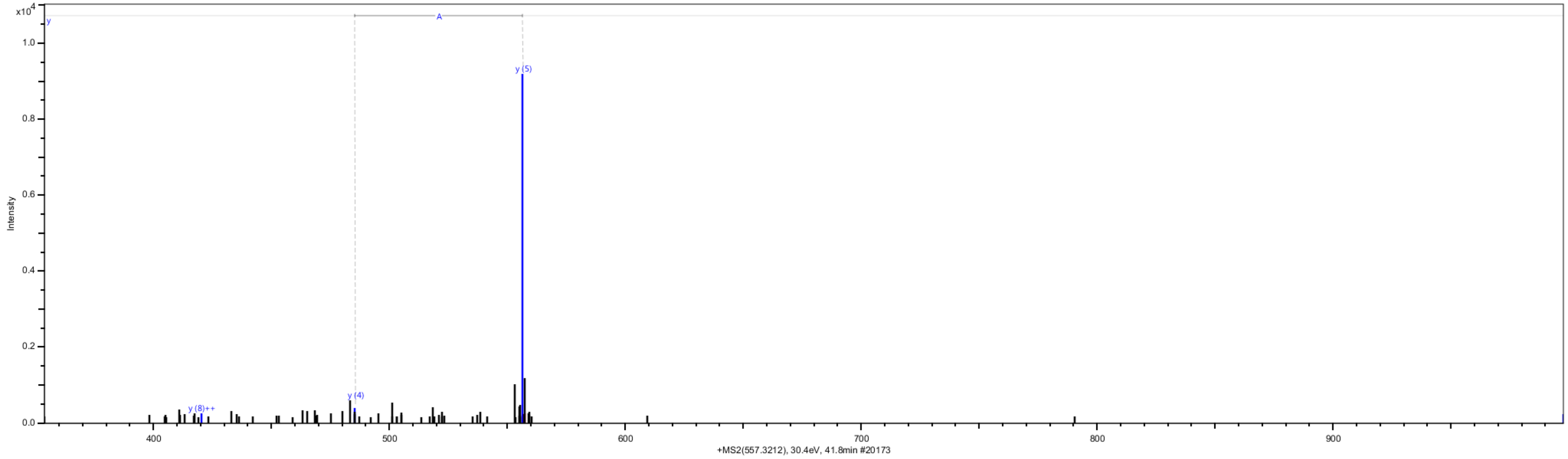

VWA3A_HUMAN

**Accession:**	VWA3A_HUMAN	**Score:**	26.0
**Database:**	SwissProt	**Seq. Coverage [%]:**	0.8
**MW [kDa] / pI:**	133.9 / 8.7	**No. of Peptides:**	1

Peptide Match

**Sequence:**	K.GLDSLVAIMR.S	**Score:**	26
**Modifications:**	Oxidation: 9		

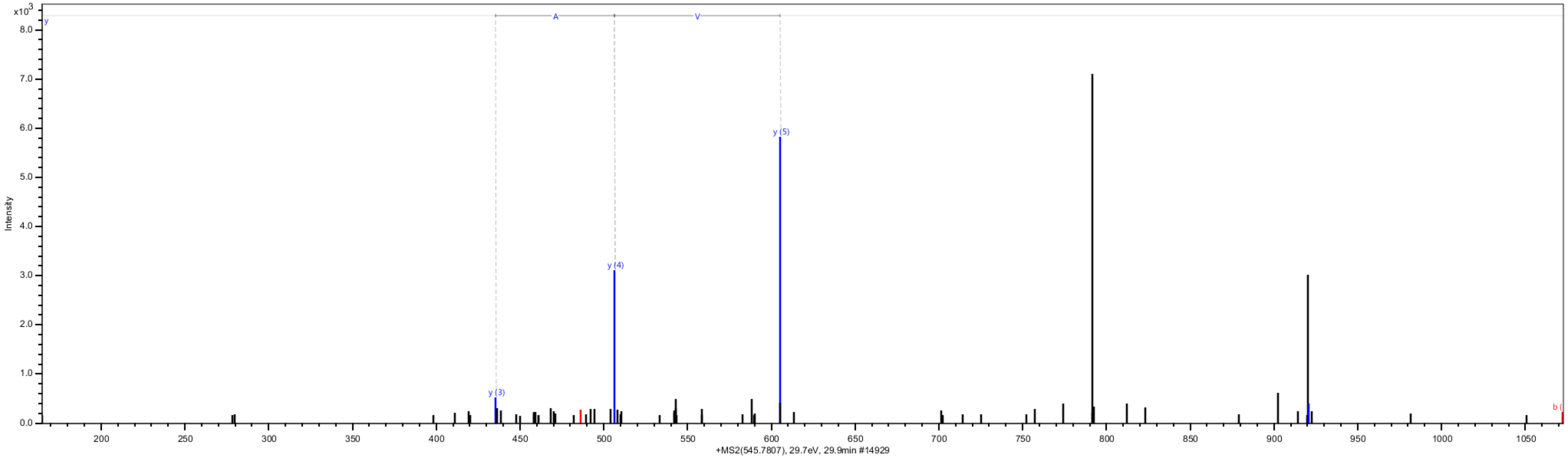

CRAD_HUMAN

Accession: CRAD_HUMAN
Database: SwissProt
MW [kDa] / pI: 136.7 / 5.5
Score: 25.7
Seq. Coverage [%]: 0.7
No. of Peptides: 1

Peptide Match

Sequence: K.ASPFGIKLR.R
Score: 25.7
Modifications:

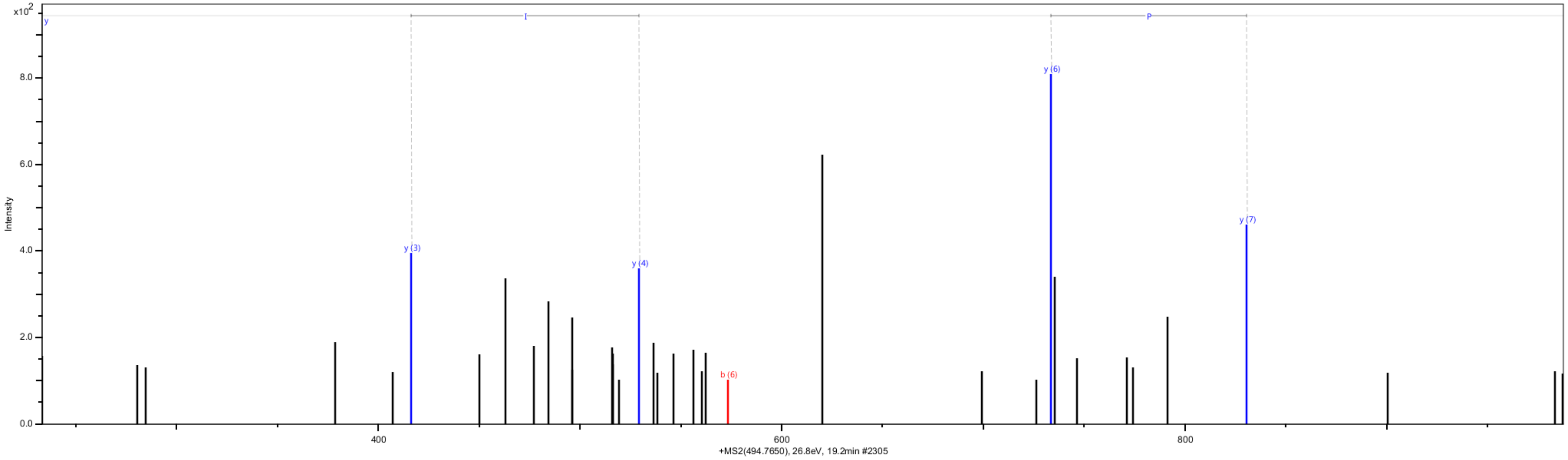

IGJ_HUMAN

Accession: IGJ_HUMAN
Database: SwissProt
MW [kDa] / pI: 18.1 / 5.1
Score: 25.7
Seq. Coverage [%]: 6.9
No. of Peptides: 1

Peptide Match

Sequence: R.FVYHLSDLCKK.C
Score: 25.7
Modifications: Carbamidomethyl: 9

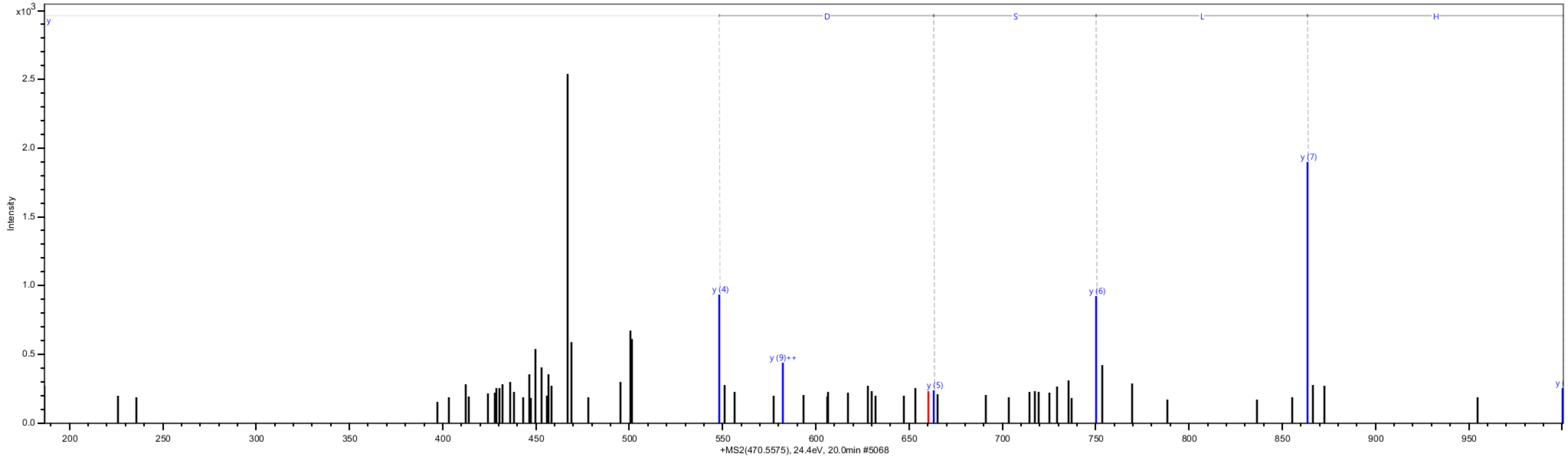

F120A_HUMAN

**Accession:**	F120A_HUMAN	**Score:**	25.8
**Database:**	SwissProt	**Seq. Coverage [%]:**	1.7
**MW [kDa] / pI:**	121.8 / 9.1	**No. of Peptides:**	1

Peptide Match

**Sequence:**	K.GSQMGTVQPIPCLLSMPTR.N	**Score:**	25.8
**Modifications:**	Carbamidomethyl: 12; Oxidation: 4		

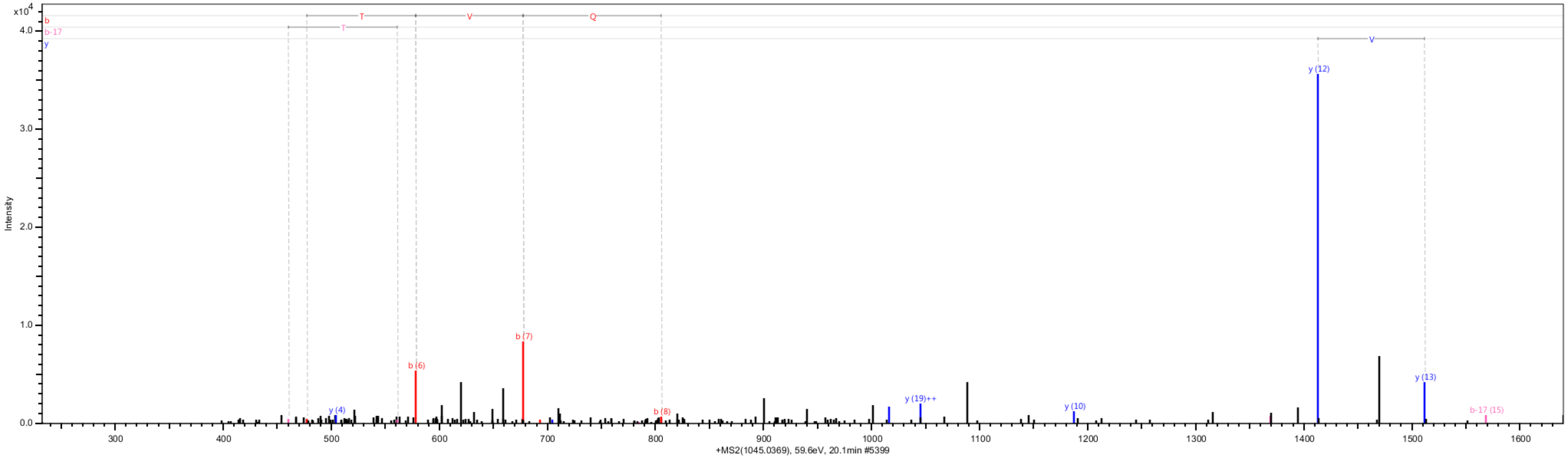

KCC1A_HUMAN

**Accession:**	KCC1A_HUMAN	**Score:**	26.0
**Database:**	SwissProt	**Seq. Coverage [%]:**	2.7
**MW [kDa] / pI:**	41.3 / 5.1	**No. of Peptides:**	1

Peptide Match

**Sequence:**	K.CIAKEALEGK.E	**Score:**	26
**Modifications:**	Carbamidomethyl: 1		

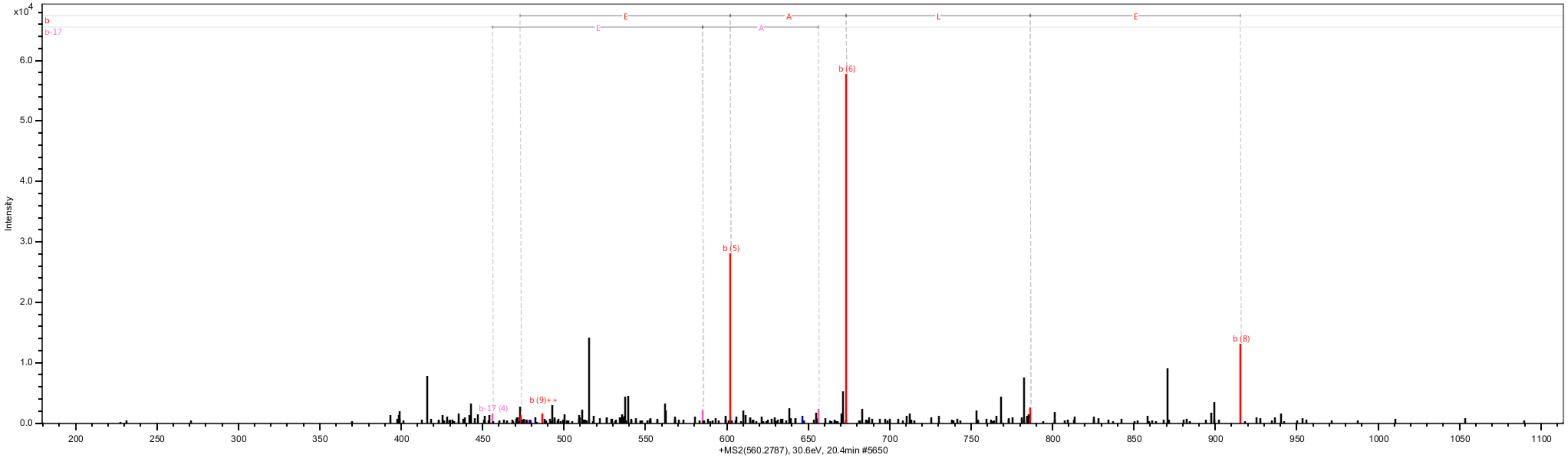

PCGF2_HUMAN

Accession: PCGF2_HUMAN
Database: SwissProt
MW [kDa] / pI: 37.8 / 8.2
Score: 27.5
Seq. Coverage [%]: 3.5
No. of Peptides: 1

Peptide Match

Sequence: K.MTVNGAPVPPLT.-
Score: 27.5
Modifications: Oxidation: 1

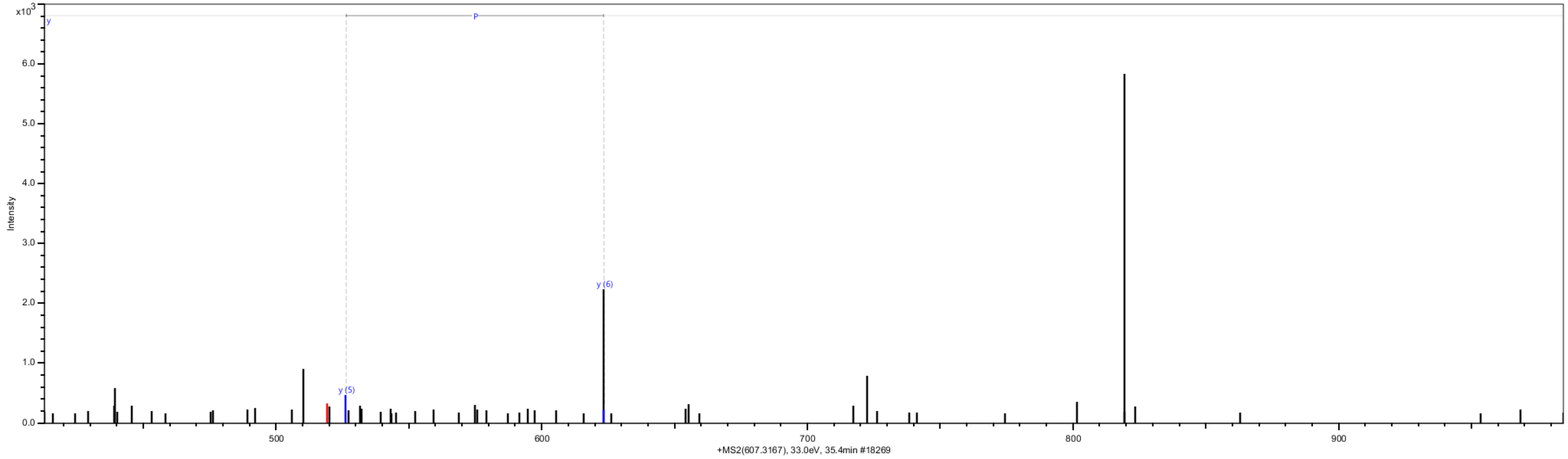

CAC1C_HUMAN

**Accession:**	CAC1C_HUMAN	**Score:**	27.6
**Database:**	SwissProt	**Seq. Coverage [%]:**	0.8
**MW [kDa] / pI:**	248.8 / 6.3	**No. of Peptides:**	1

Peptide Match

**Sequence:**	K.ADGANALGGKGAGFDVK.A	**Score:**	27.6
**Modifications:**			

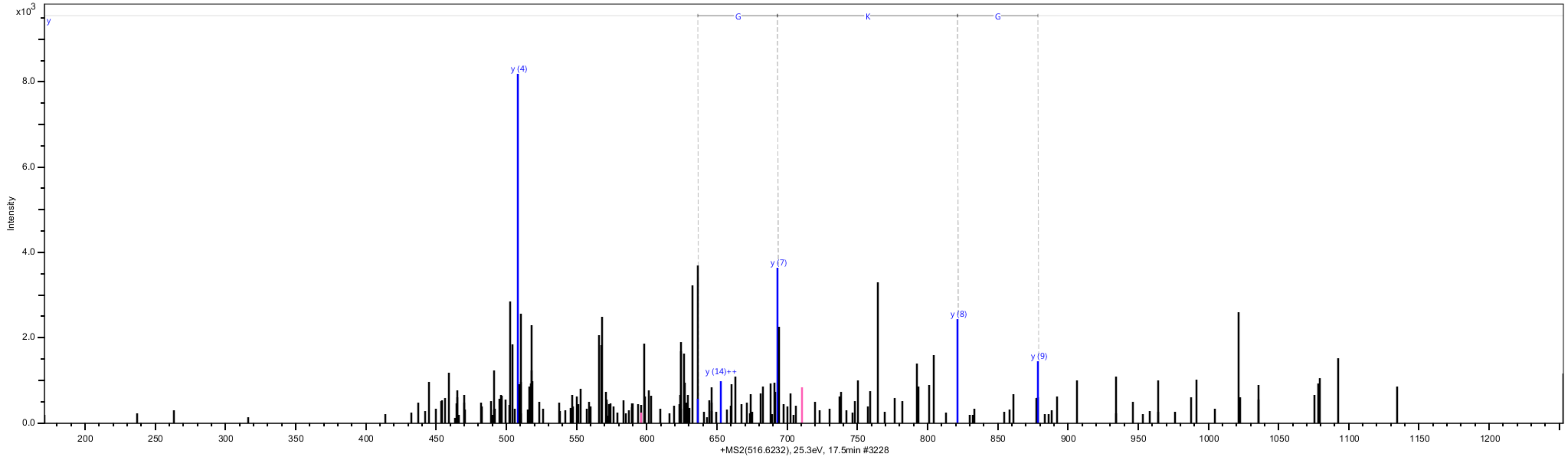

RNPC3_HUMAN

**Accession:**	RNPC3_HUMAN	**Score:**	27.7
**Database:**	SwissProt	**Seq. Coverage [%]:**	1.9
**MW [kDa] / pI:**	58.5 / 7.6	**No. of Peptides:**	1

Peptide Match

**Sequence:**	R.EEMETLSVFR.S	**Score:**	27.7
**Modifications:**			

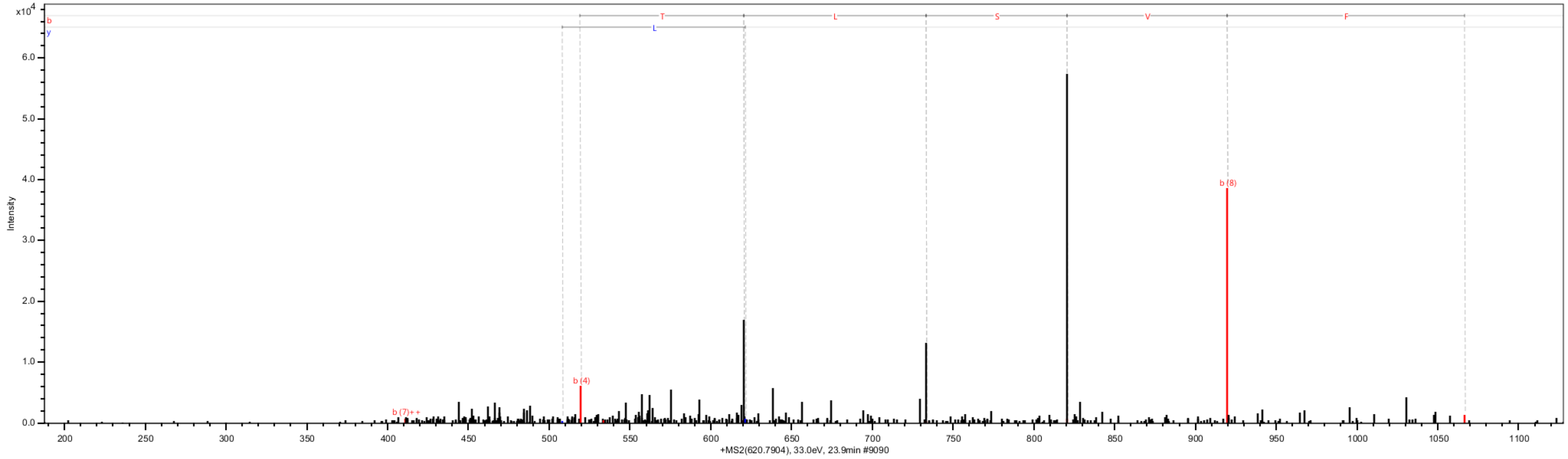

TIM_HUMAN

Accession: TIM_HUMAN
Database: SwissProt
MW [kDa] / pI: 138.6 / 5.3
Score: 27.9
Seq. Coverage [%]: 0.7
No. of Peptides: 1

Peptide Match

Sequence: R.GNLVVQNK.Q
Score: 27.9
Modifications:

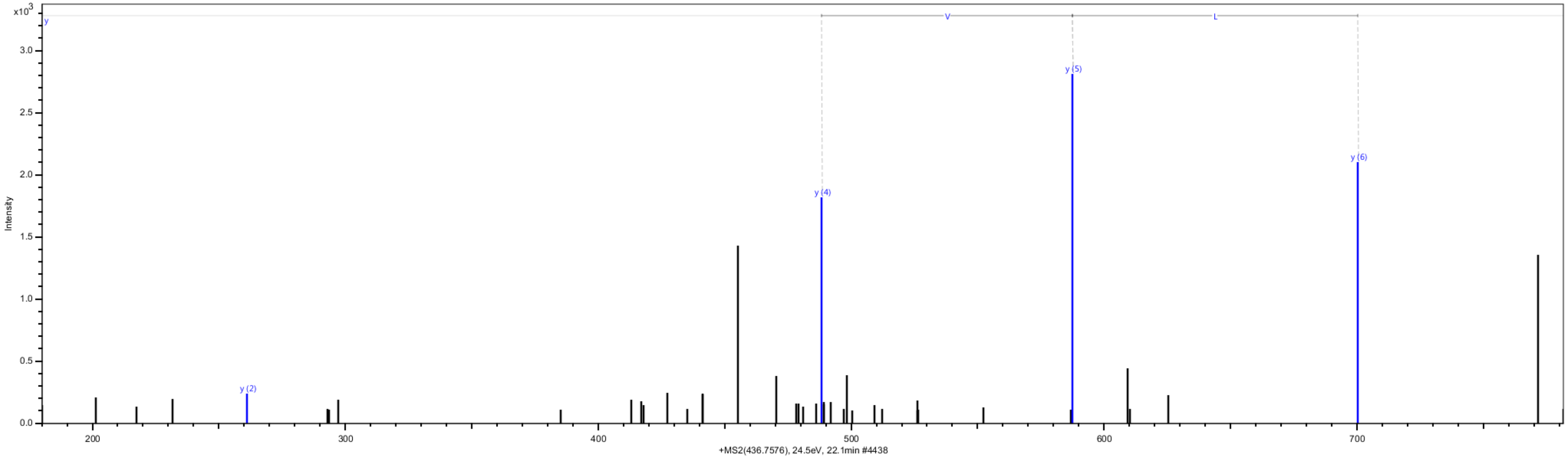

LIPR3_HUMAN

Accession: LIPR3_HUMAN
Database: SwissProt
MW [kDa] / pI: 52.2 / 8.6
Score: 26.1
Seq. Coverage [%]: 1.9
No. of Peptides: 1

Peptide Match

Sequence: K.LEPGMTYTK.L
Score: 26.1
Modifications: Oxidation: 5

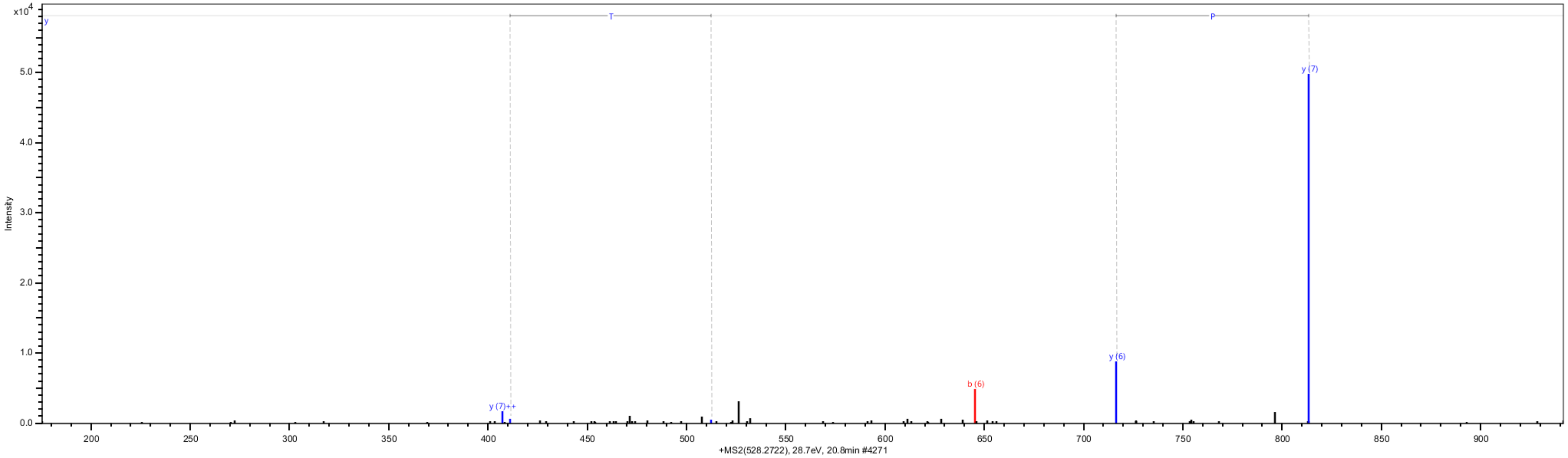

NDUF2_HUMAN

Accession: NDUF2_HUMAN
Database: SwissProt
MW [kDa] / pI: 19.8 / 8.9
Score: 26.5
Seq. Coverage [%]: 4.1
No. of Peptides: 1

Peptide Match

Sequence: K.SQDFYEK.E
Score: 26.5
Modifications:

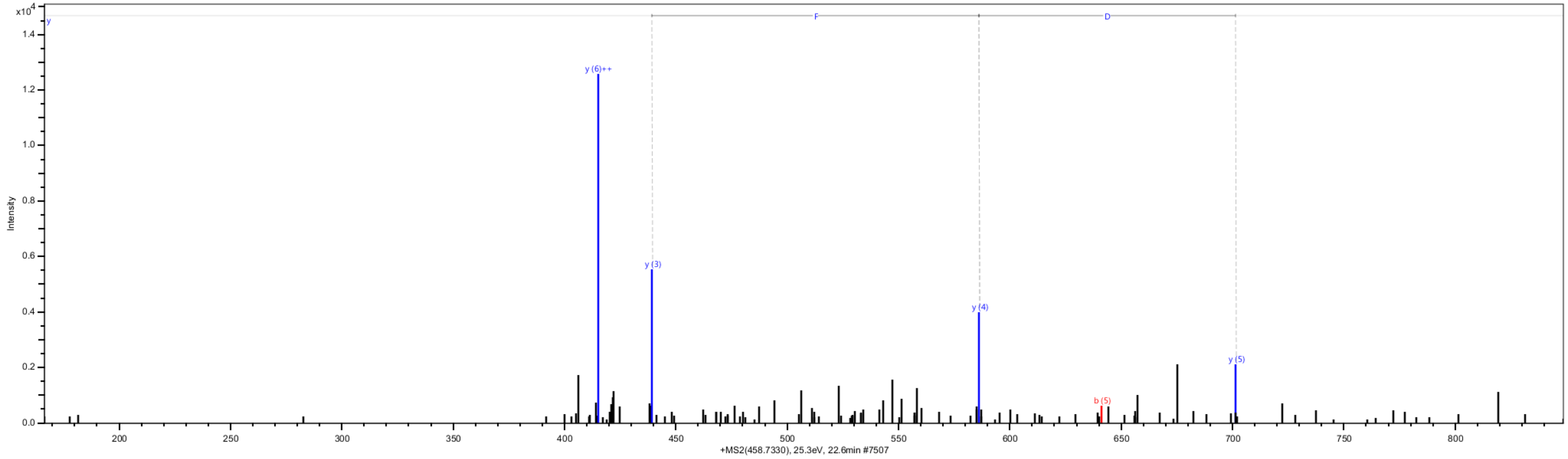

KIF15_HUMAN

Accession: KIF15_HUMAN
Database: SwissProt
MW [kDa] / pI: 160.1 / 5.7
Score: 26.6
Seq. Coverage [%]: 0.6
No. of Peptides: 1

Peptide Match

Sequence: R.LESQQLIEK.N
Score: 26.6
Modifications:

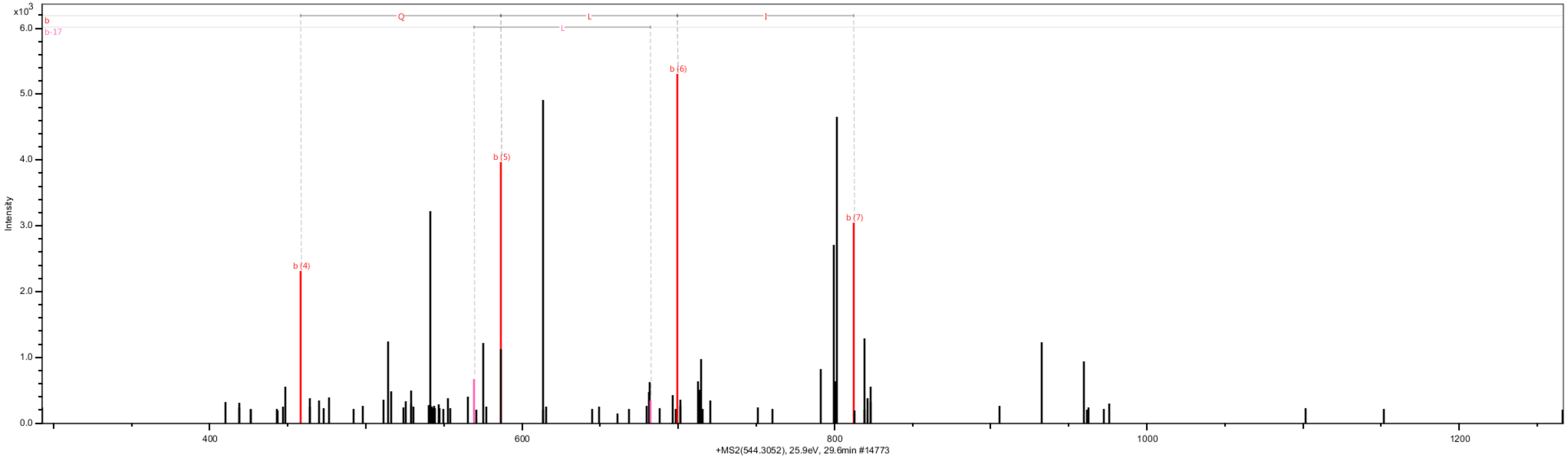

KI16B_HUMAN

Accession: KI16B_HUMAN
Database: SwissProt
MW [kDa] / pI: 151.9 / 5.9
Score: 27.4
Seq. Coverage [%]: 0.7
No. of Peptides: 1

Peptide Match

Sequence: K.LASLNSGSR.E
Score: 27.4
Modifications:

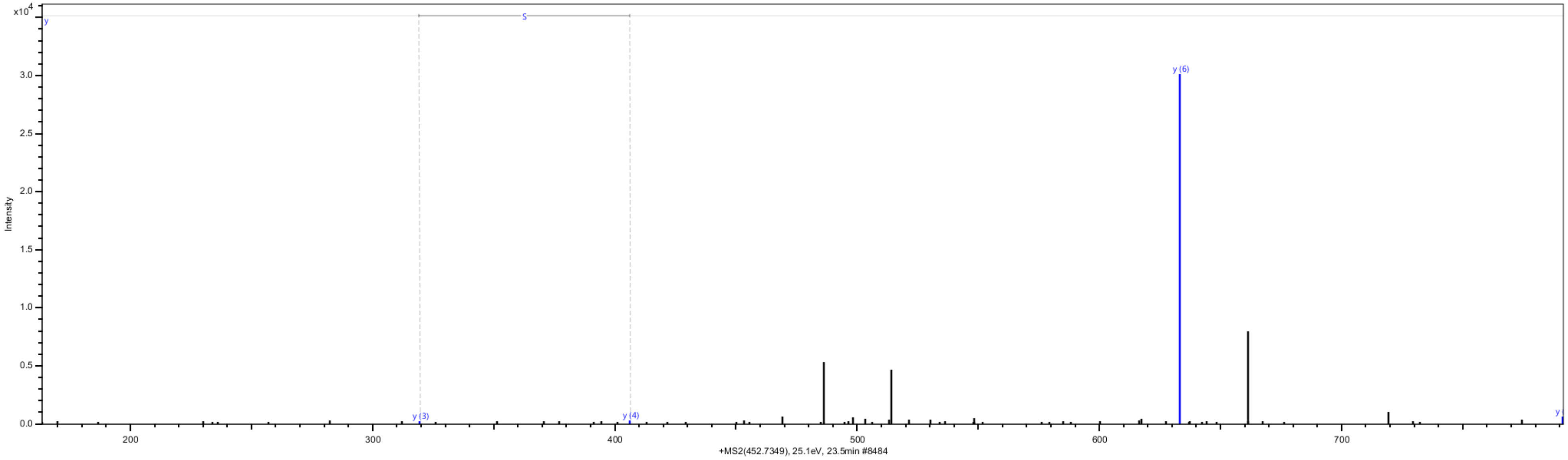

CC033_HUMAN

Accession: CC033_HUMAN
Database: SwissProt
MW [kDa] / pI: 33.7 / 9.8
Score: 30.1
Seq. Coverage [%]: 3.1
No. of Peptides: 1

Peptide Match

Sequence: K.EPRGALLVK.L
Score: 30
Modifications:

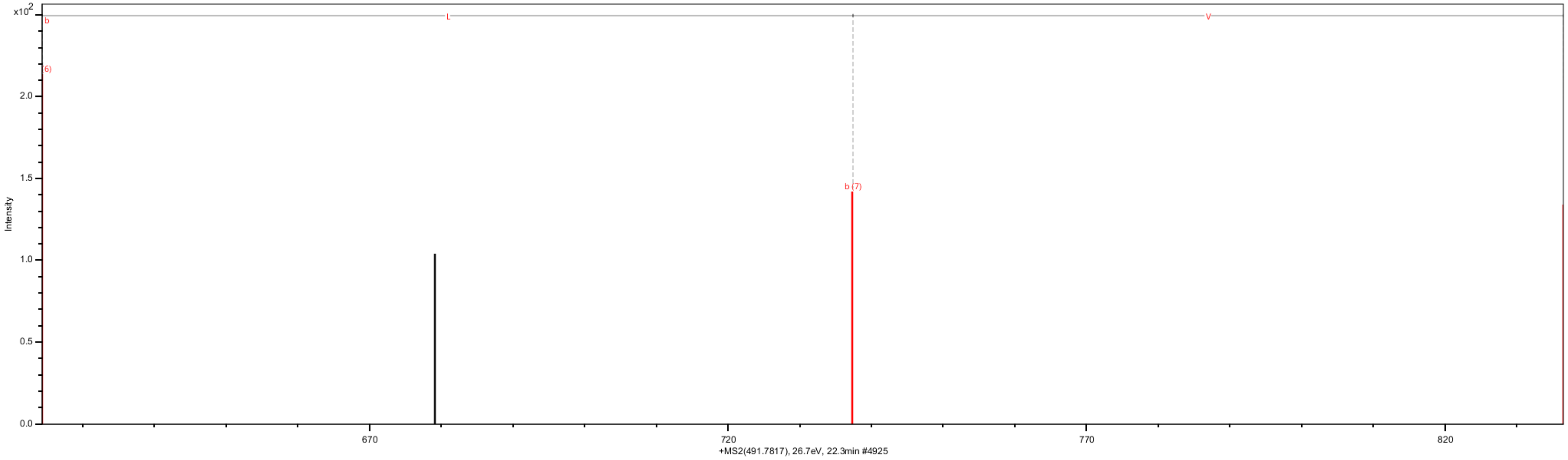

HV349_HUMAN

Accession: HV349_HUMAN
Database: SwissProt
MW [kDa] / pI: 13.0 / 8.8
Score: 29.9
Seq. Coverage [%]: 7.6
No. of Peptides: 1

Peptide Match

Sequence: K.GLEWVGFIR.S
Score: 29.9
Modifications:

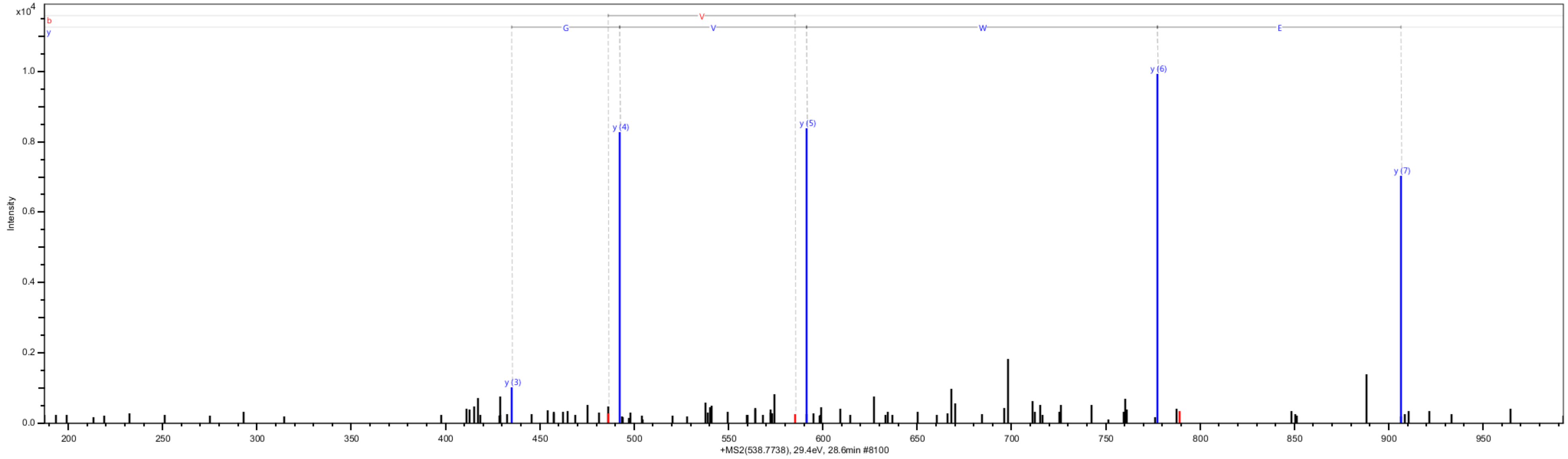

XPO2_HUMAN

Accession: XPO2_HUMAN
Database: SwissProt
MW [kDa] / pI: 110.3 / 5.5
Score: 31.3
Seq. Coverage [%]: 0.8
No. of Peptides: 1

Peptide Match

Sequence: K.IIIPEIQK.V
Score: 16.2
Modifications:

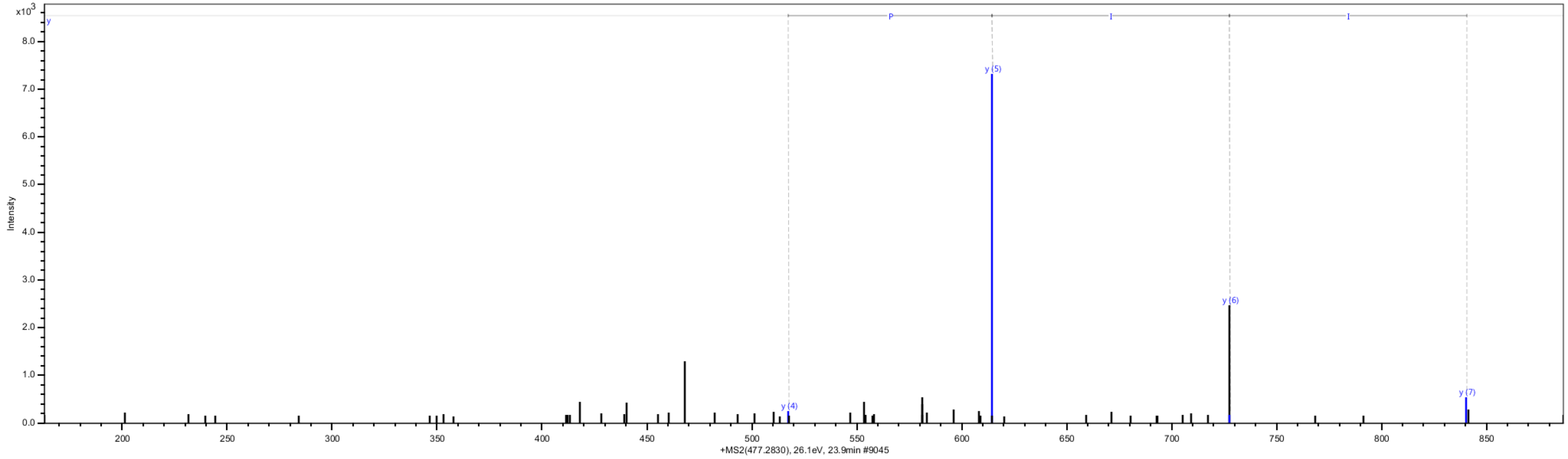

PHF1_HUMAN

Accession: PHF1_HUMAN
Database: SwissProt
MW [kDa] / pI: 62.1 / 9.3
Score: 31.2
Seq. Coverage [%]: 1.4
No. of Peptides: 1

Peptide Match

Sequence: R.LVSCEKCR.H
Score: 16
Modifications: Carbamidomethyl: 4, 7

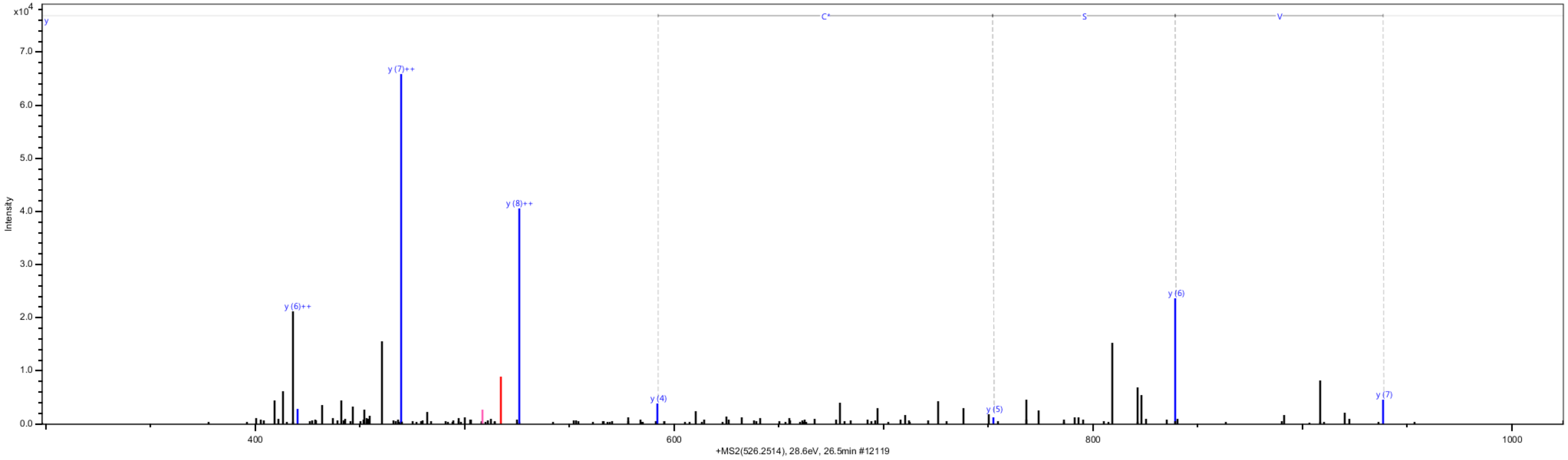

ATLA1_HUMAN

Accession: ATLA1_HUMAN
Database: SwissProt
MW [kDa] / pI: 63.5 / 5.8
Score: 28.5
Seq. Coverage [%]: 1.6
No. of Peptides: 1

Peptide Match

Sequence: K.KMGGEEFSR.R
Score: 28.5
Modifications: Oxidation: 2

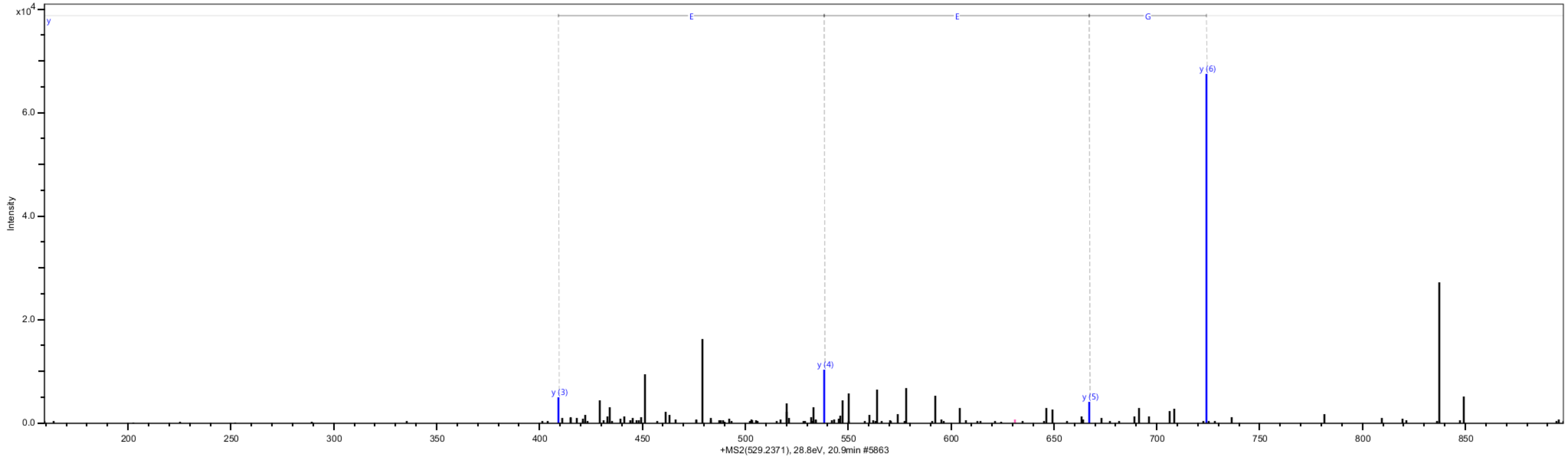

KV401_HUMAN

Accession: KV401_HUMAN
Database: SwissProt
MW [kDa] / pI: 13.4 / 5.1
Score: 28.3
Seq. Coverage [%]: 7.4
No. of Peptides: 1

Peptide Match

Sequence: K.LLIYWASTR.E
Score: 28.3
Modifications:

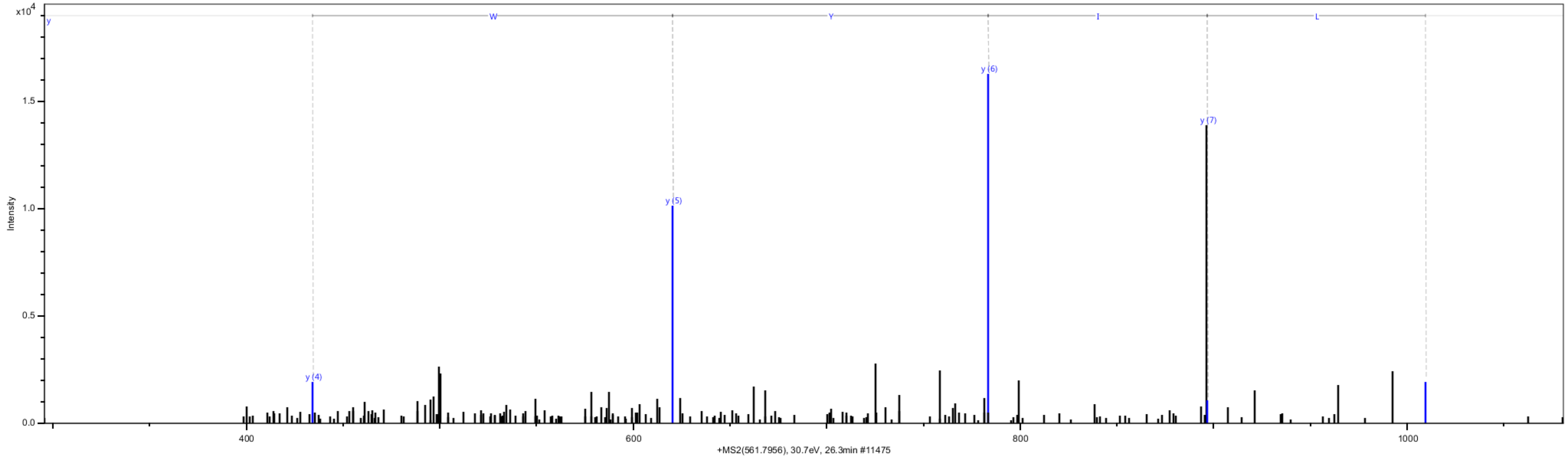

CDK5_HUMAN

**Accession:**	CDK5_HUMAN	**Score:**	29.4
**Database:**	SwissProt	**Seq. Coverage [%]:**	3.1
**MW [kDa] / pI:**	33.3 / 7.6	**No. of Peptides:**	1

Peptide Match

**Sequence:**	R.LHDVLHSDK.K	**Score:**	29.4
**Modifications:**			

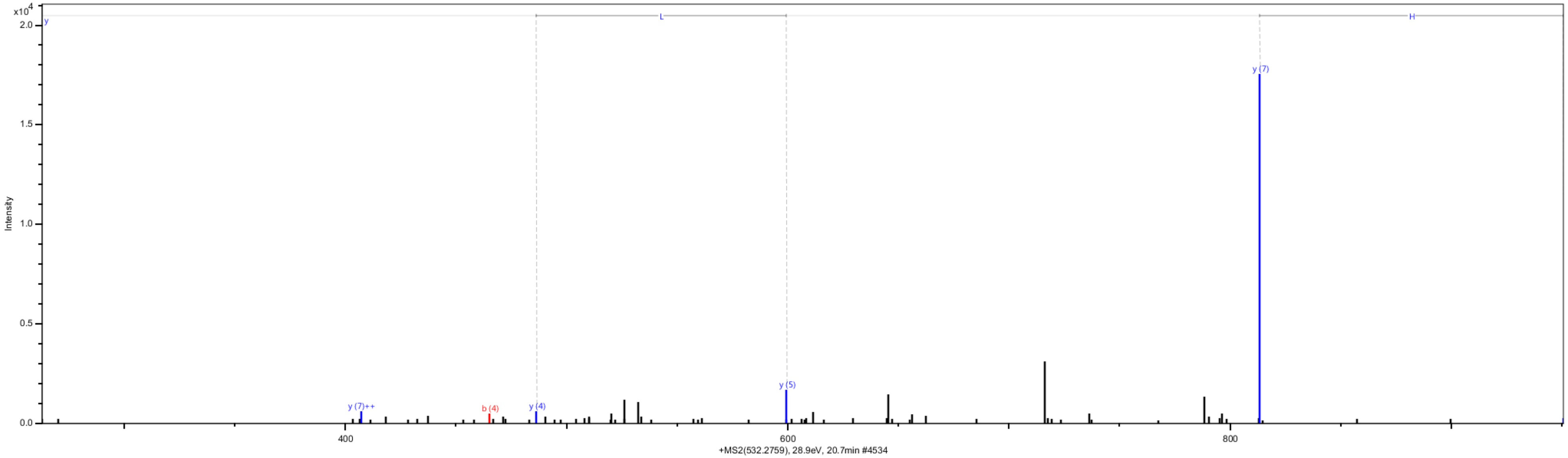

SH24B_HUMAN

Accession: SH24B_HUMAN
Database: SwissProt
MW [kDa] / pI: 51.2 / 5.9
Score: 28.9
Seq. Coverage [%]: 2.1
No. of Peptides: 1

Peptide Match

Sequence: K.FRDALANEK.A
Score: 28.8
Modifications:

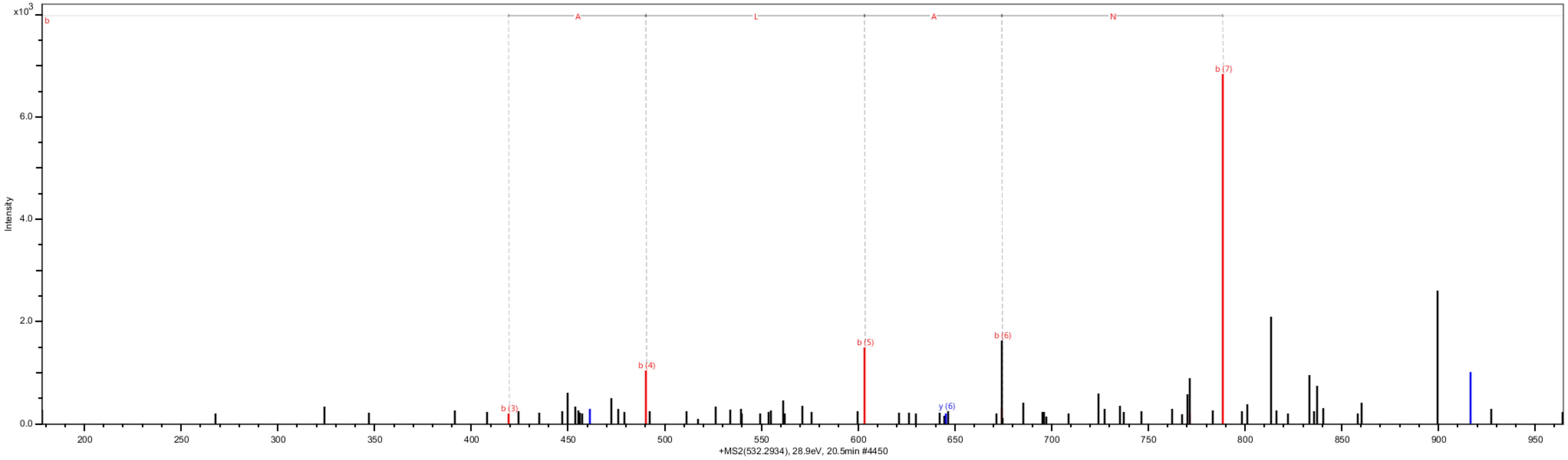

NID1_HUMAN

Accession: NID1_HUMAN
Database: SwissProt
MW [kDa] / pI: 136.3 / 5.1
Score: 31.9
Seq. Coverage [%]: 0.6
No. of Peptides: 1

Peptide Match

Sequence: R.GIVTDSVR.G
Score: 31.9
Modifications:

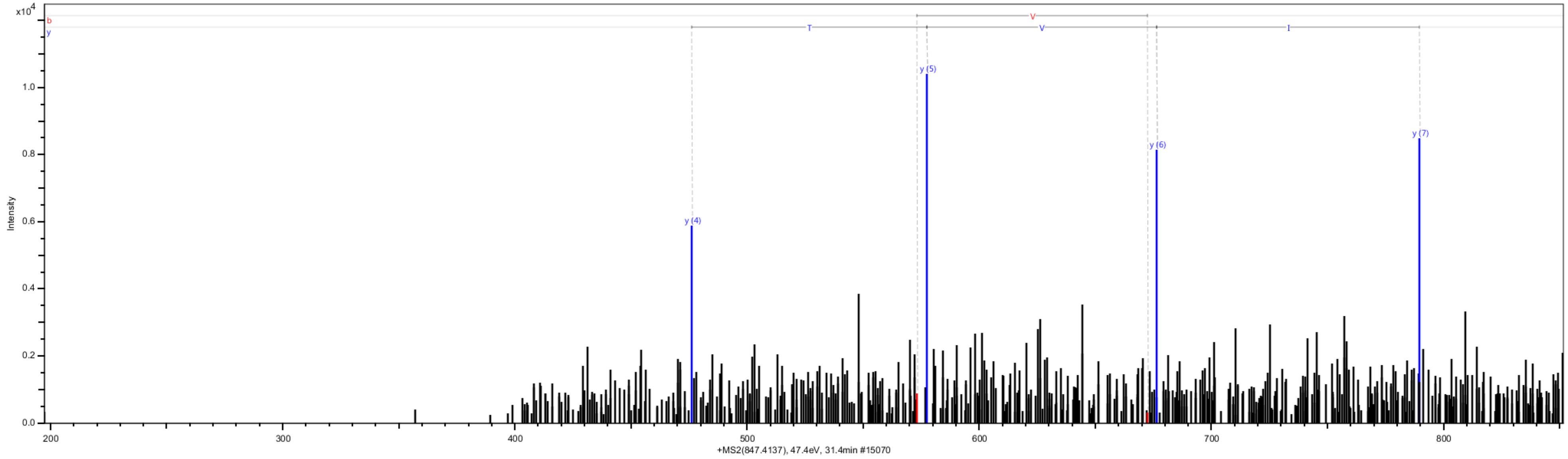

KV315_HUMAN

**Accession:**	KV315_HUMAN	**Score:**	31.9
**Database:**	SwissProt	**Seq. Coverage [%]:**	7.8
**MW [kDa] / pI:**	12.5 / 5.1	**No. of Peptides:**	1

Peptide Match

**Sequence:**	R.LLIYGASTR.A	**Score:**	31.9
**Modifications:**			

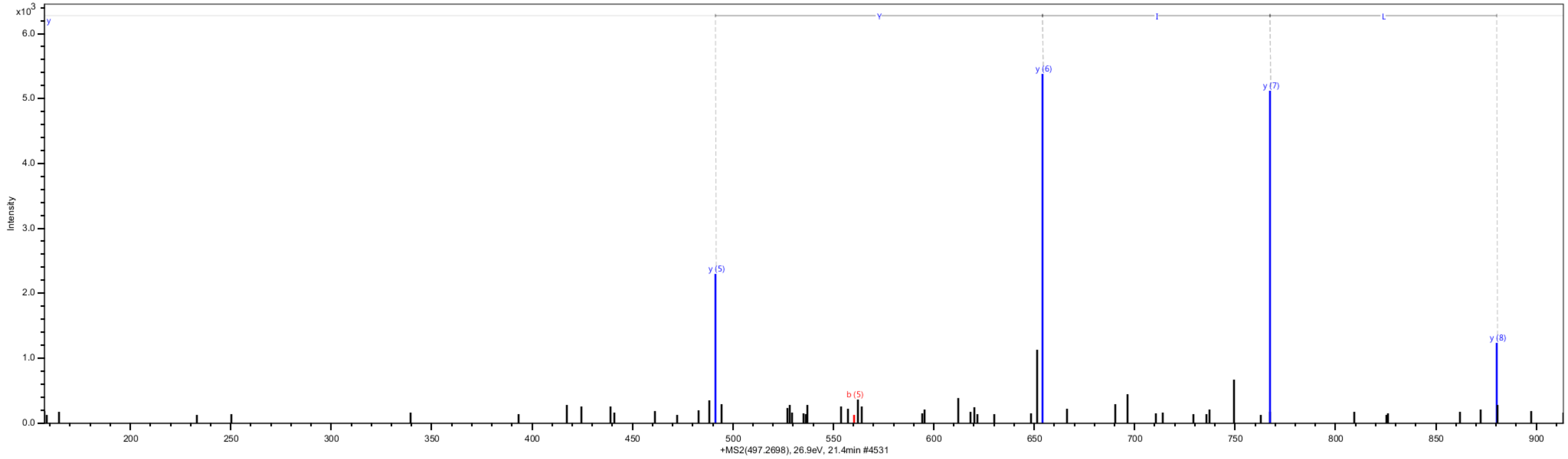

DYH11_HUMAN

**Accession:**	DYH11_HUMAN	**Score:**	31.5
**Database:**	SwissProt	**Seq. Coverage [%]:**	0.3
**MW [kDa] / pI:**	520.0 / 6.0	**No. of Peptides:**	1

Peptide Match

**Sequence:**	K.GKPLMLVGNAGVGK.T	**Score:**	15.8
**Modifications:**	Oxidation: 5		

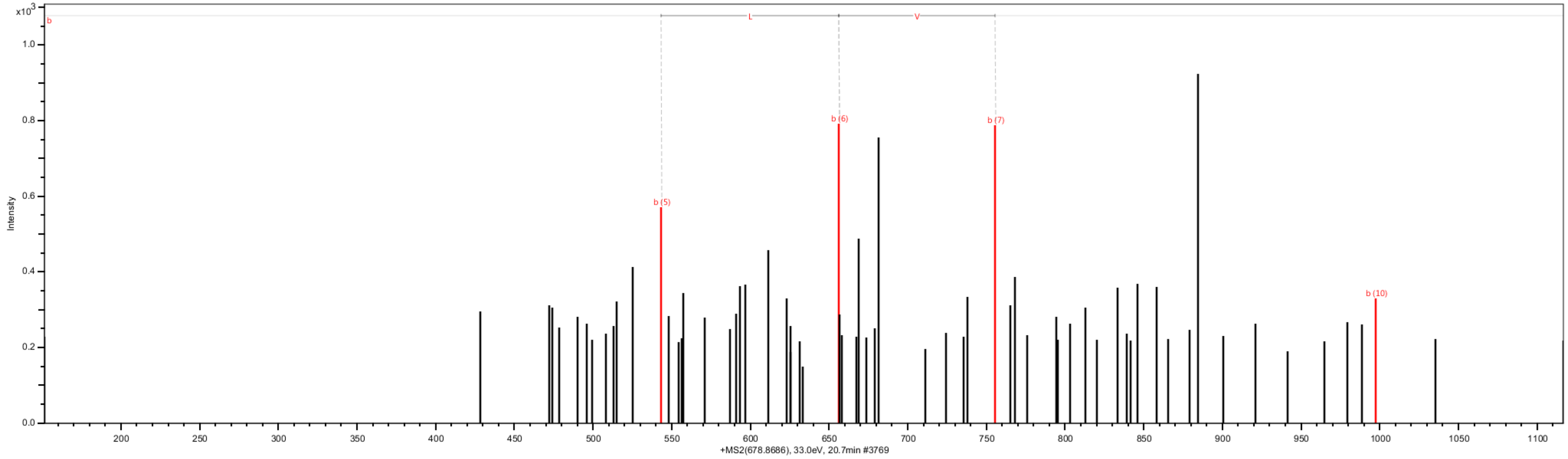

ERAP1_HUMAN

Accession: ERAP1_HUMAN
Database: SwissProt
MW [kDa] / pI: 107.2 / 6.0
Score: 31.3
Seq. Coverage [%]: 1.1
No. of Peptides: 1

Peptide Match

Sequence: R.DMNEVETQFK.A
Score: 31.3
Modifications: Oxidation: 2

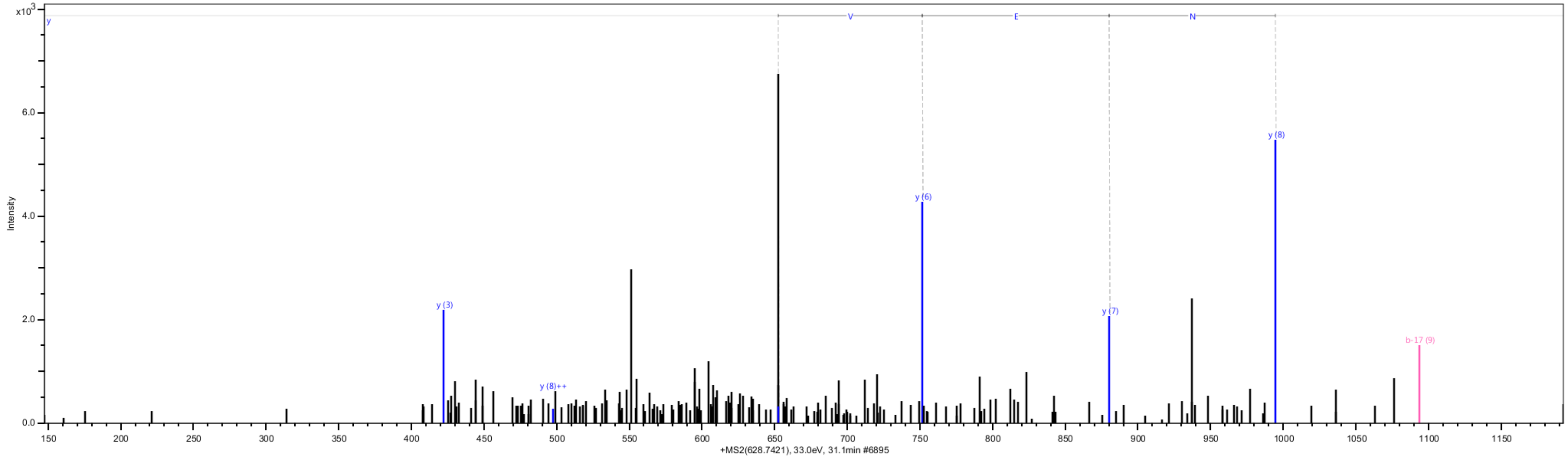

CO6A5_HUMAN

Accession: CO6A5_HUMAN
Database: SwissProt
MW [kDa] / pI: 289.7 / 6.5
Score: 31.8
Seq. Coverage [%]: 0.3
No. of Peptides: 1

Peptide Match

Sequence: K.QFEQIKR.F
Score: 15.7
Modifications:

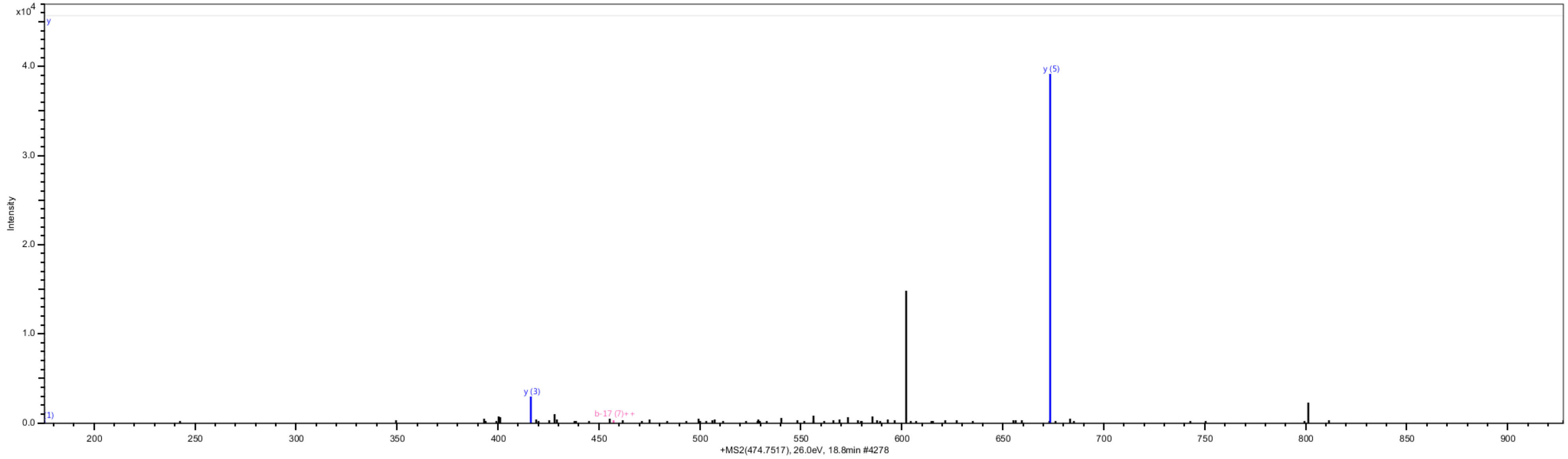

MBD5_HUMAN

**Accession:**	MBD5_HUMAN	**Score:**	31.7
**Database:**	SwissProt	**Seq. Coverage [%]:**	0.7
**MW [kDa] / pI:**	159.8 / 9.2	**No. of Peptides:**	2

Peptide Match

Sequence: R.QRLGSSEHGQK.S **Score:** 15.9
Modifications:

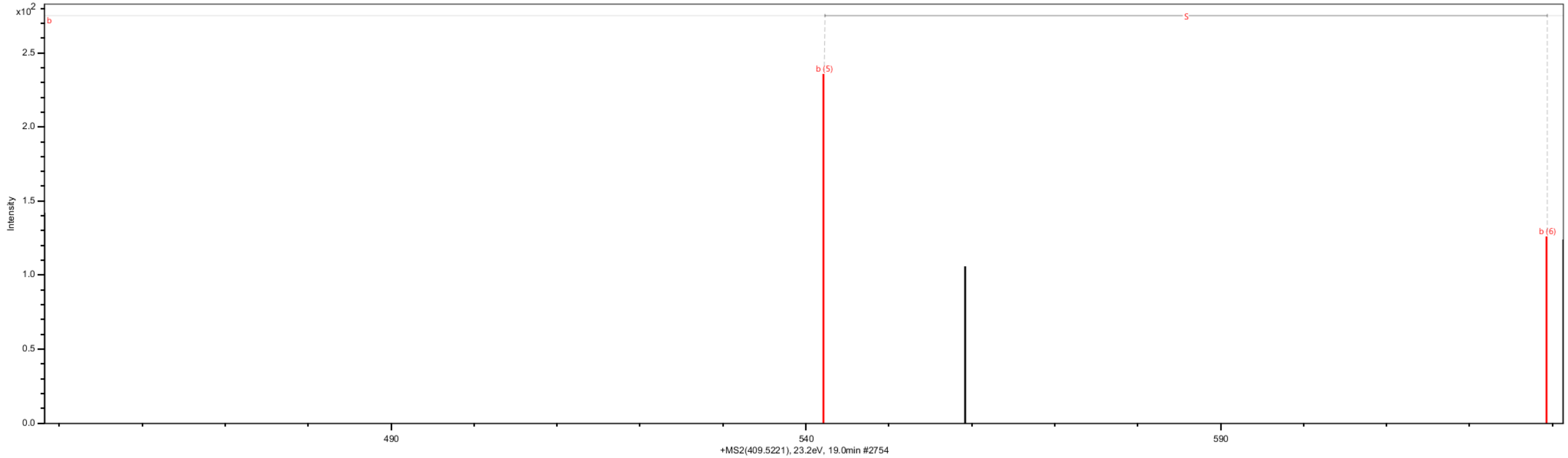

Peptide Match

Sequence: R.LGSSEHGQK.S **Score:** 15.8
Modifications:

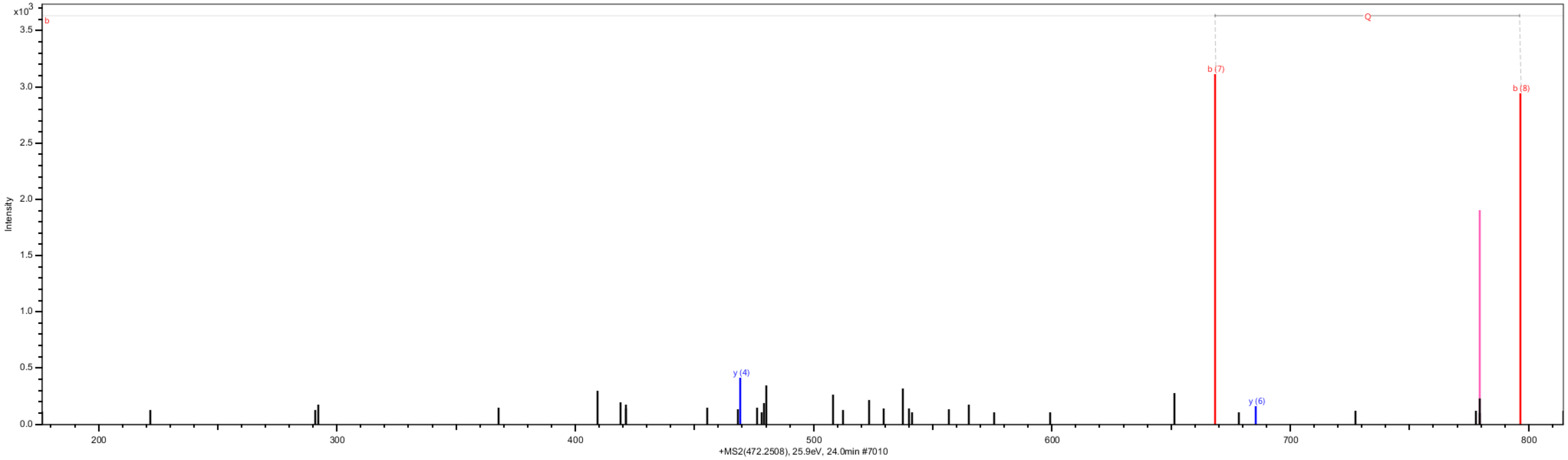
x10^3
3.5
3.0
2.5
2.0
1.5
1.0
0.5
0.0
Intensity
b
Q
b (7)
b (8)
y (4)
y (6)
200
300
400
500
600
700
800
+MS2(472.2508), 25.9eV, 24.0min #7010

PLXA1_HUMAN

Accession: PLXA1_HUMAN
Database: SwissProt
MW [kDa] / pI: 210.9 / 6.5
Score: 16.8
Seq. Coverage [%]: 0.8
No. of Peptides: 1

Peptide Match

Sequence: R.SRTPMITPDLESGTK.L
Score: 16.8
Modifications: Oxidation: 5

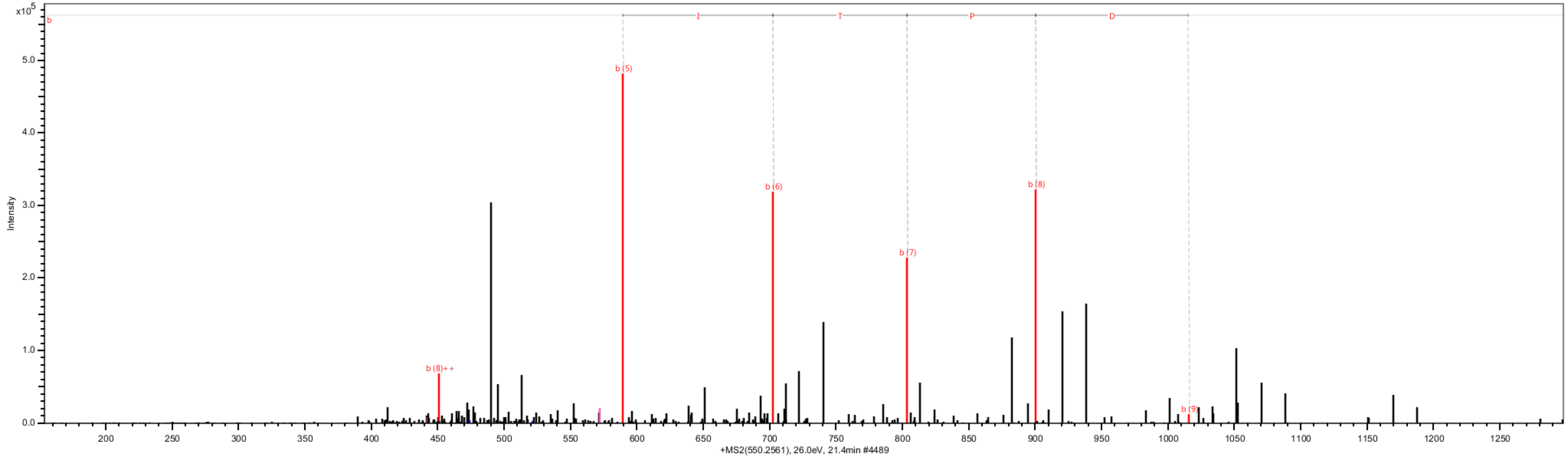

SYNP2_HUMAN

Accession: SYNP2_HUMAN
Database: SwissProt
MW [kDa] / pI: 117.4 / 8.8
Score: 16.8
Seq. Coverage [%]: 1.6
No. of Peptides: 1

Peptide Match

Sequence: K.MEMLPDTTGKGALMFAK.R
Score: 16.8
Modifications: Oxidation: 14

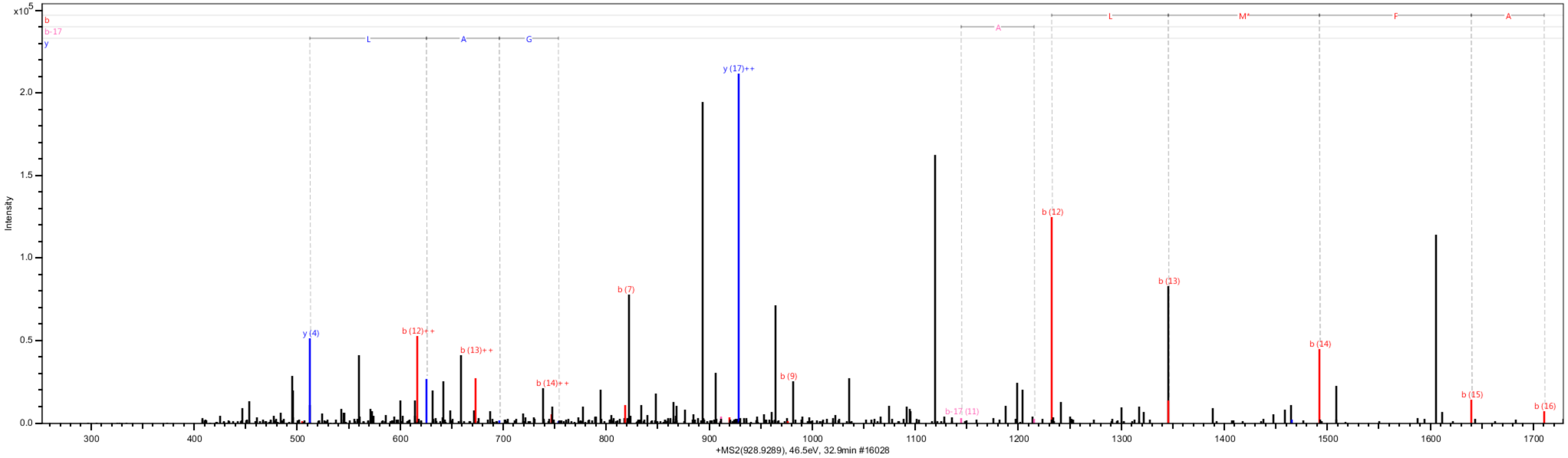

OFUT2_HUMAN

Accession: OFUT2_HUMAN
Database: SwissProt
MW [kDa] / pI: 49.9 / 6.1
Score: 16.8
Seq. Coverage [%]: 2.8
No. of Peptides: 1

Peptide Match

Sequence: R.LDKVFVATDAVR.K
Score: 16.8
Modifications:

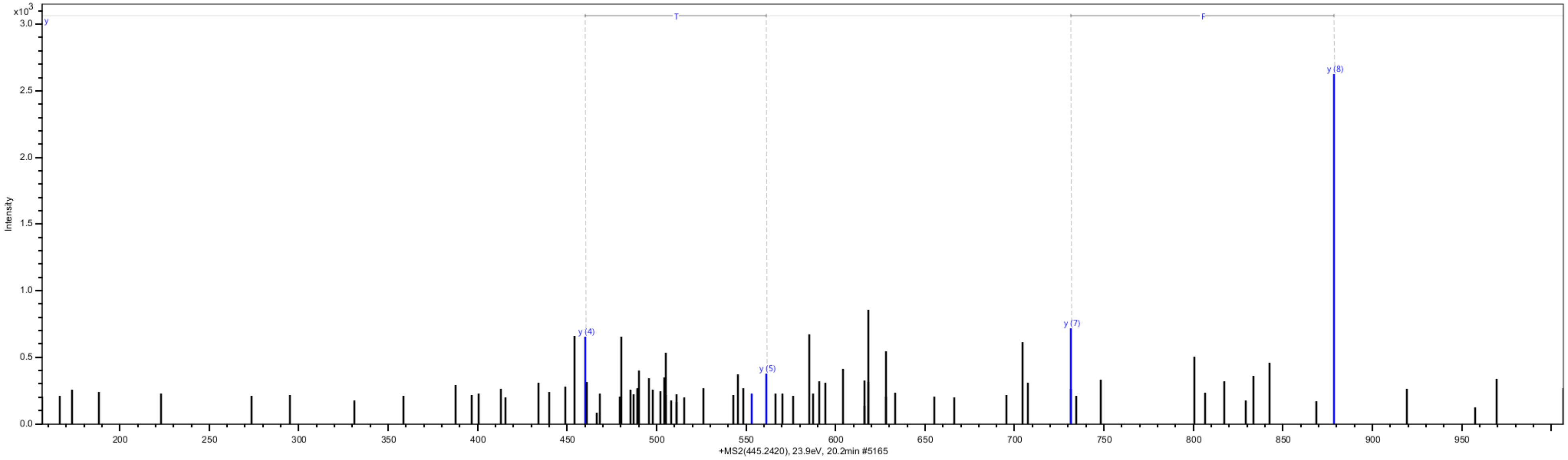

F102A_HUMAN

Accession: F102A_HUMAN
Database: SwissProt
MW [kDa] / pI: 41.8 / 8.8
Score: 16.8
Seq. Coverage [%]: 2.3
No. of Peptides: 1

Peptide Match

Sequence: R.IDADAIVEK.I
Score: 16.8
Modifications:

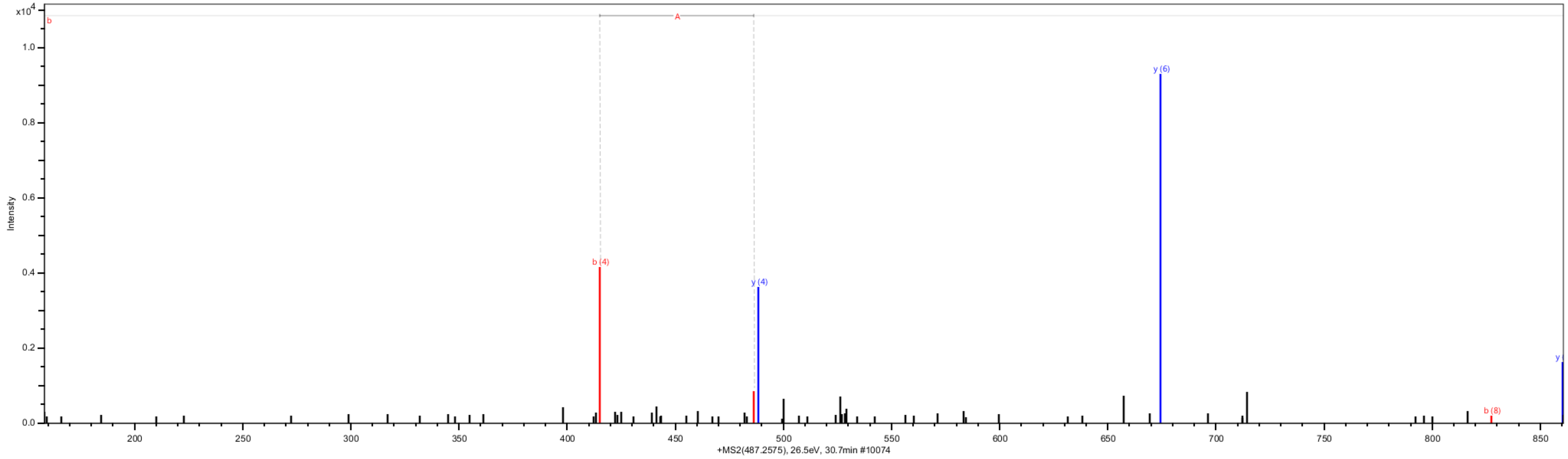

LRN4L_HUMAN

**Accession:**	LRN4L_HUMAN	**Score:**	16.9
**Database:**	SwissProt	**Seq. Coverage [%]:**	4.2
**MW [kDa] / pI:**	25.2 / 6.1	**No. of Peptides:**	1

Peptide Match

**Sequence:**	K.GPPLNATVRR.A	**Score:**	16.9
**Modifications:**			

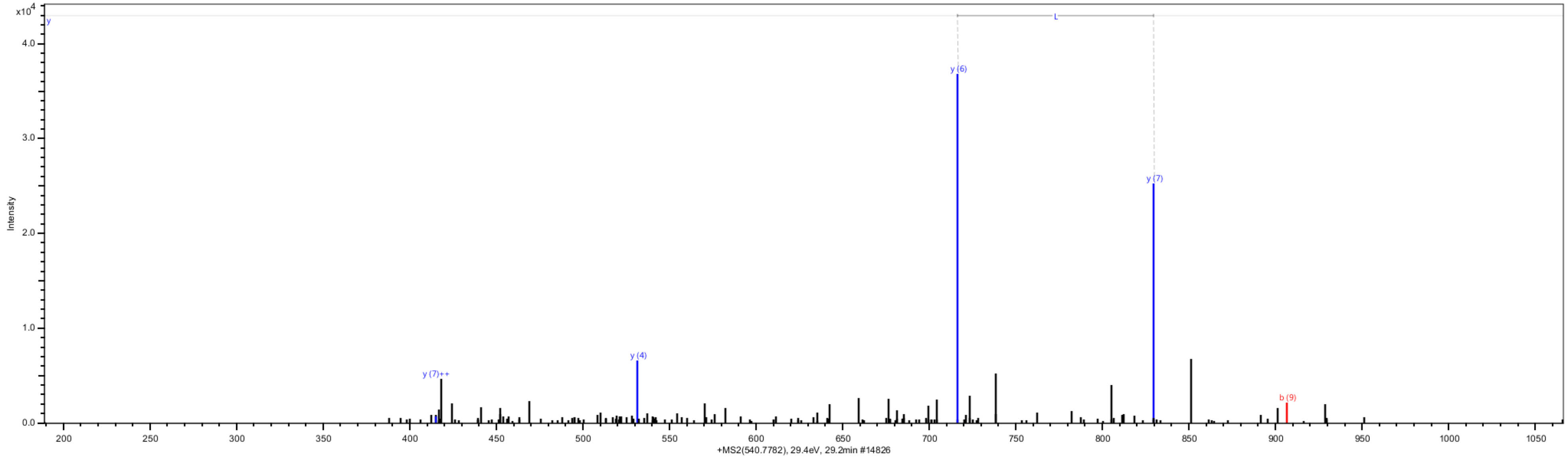

PCDGH_HUMAN

**Accession:**	PCDGH_HUMAN	**Score:**	16.9
**Database:**	SwissProt	**Seq. Coverage [%]:**	0.9
**MW [kDa] / pI:**	99.8 / 4.9	**No. of Peptides:**	1

Peptide Match

**Sequence:**	R.AFDYEQLR.T	**Score:**	16.9
**Modifications:**			

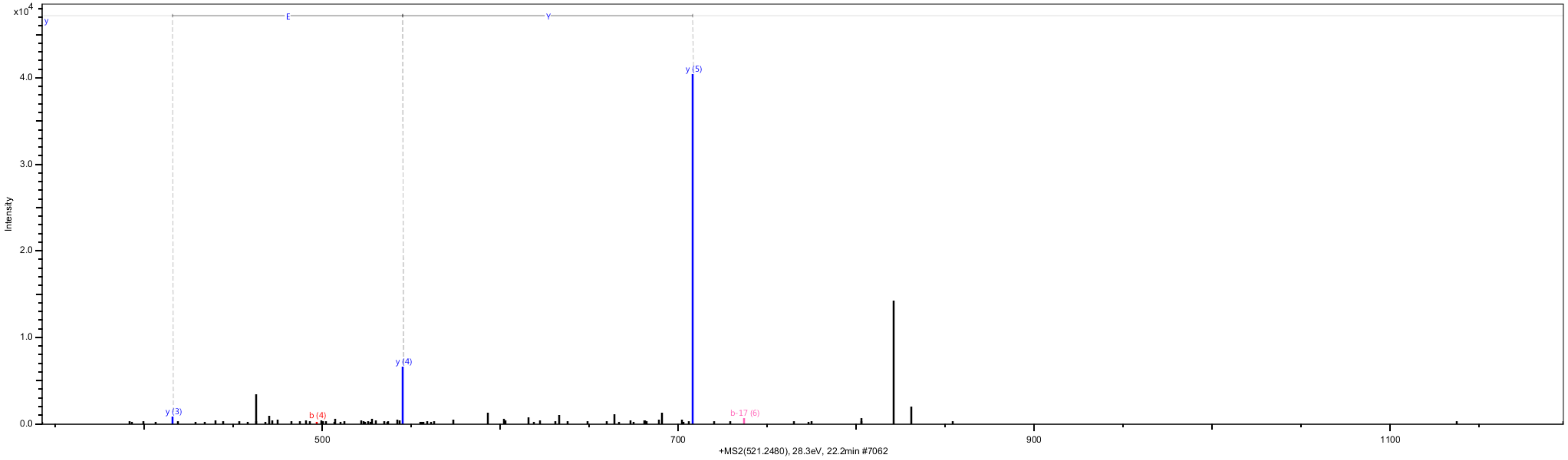

FA47E_HUMAN

Accession: FA47E_HUMAN
Database: SwissProt
MW [kDa] / pI: 45.6 / 9.5
Score: 16.9
Seq. Coverage [%]: 2.0
No. of Peptides: 1

Peptide Match

Sequence: K.LSPAQQAR.K
Score: 16.9
Modifications:

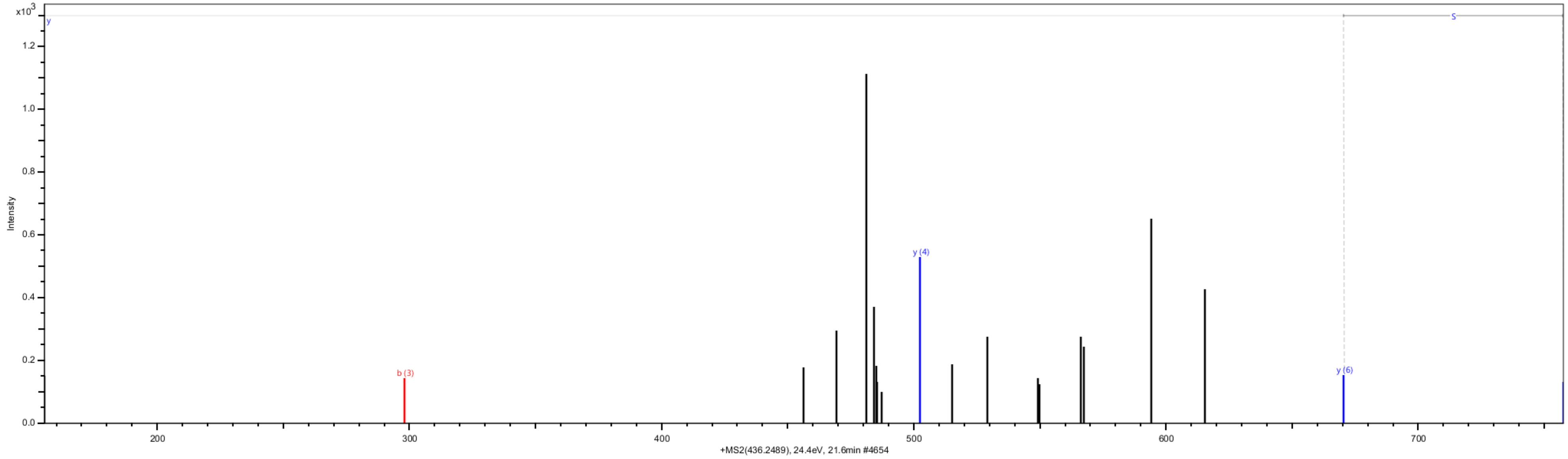

DOC11_HUMAN

**Accession:**	DOC11_HUMAN	**Score:**	16.9
**Database:**	SwissProt	**Seq. Coverage [%]:**	0.4
**MW [kDa] / pI:**	237.5 / 7.9	**No. of Peptides:**	1

Peptide Match

**Sequence:**	K.ILEVMHTKK.R	**Score:**	16.9
**Modifications:**	Oxidation: 5		

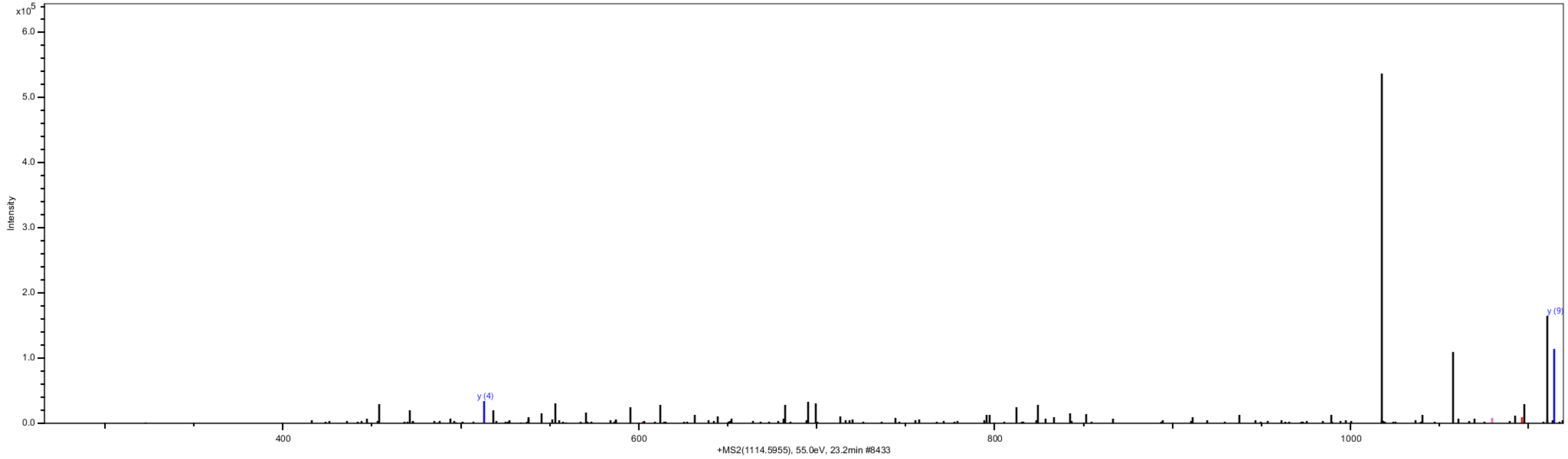

SARG_HUMAN

Accession: SARG_HUMAN
Database: SwissProt
MW [kDa] / pI: 63.9 / 8.8
Score: 16.7
Seq. Coverage [%]: 1.3
No. of Peptides: 1

Peptide Match

Sequence: R.LSYQGQSR.D
Score: 16.7
Modifications:

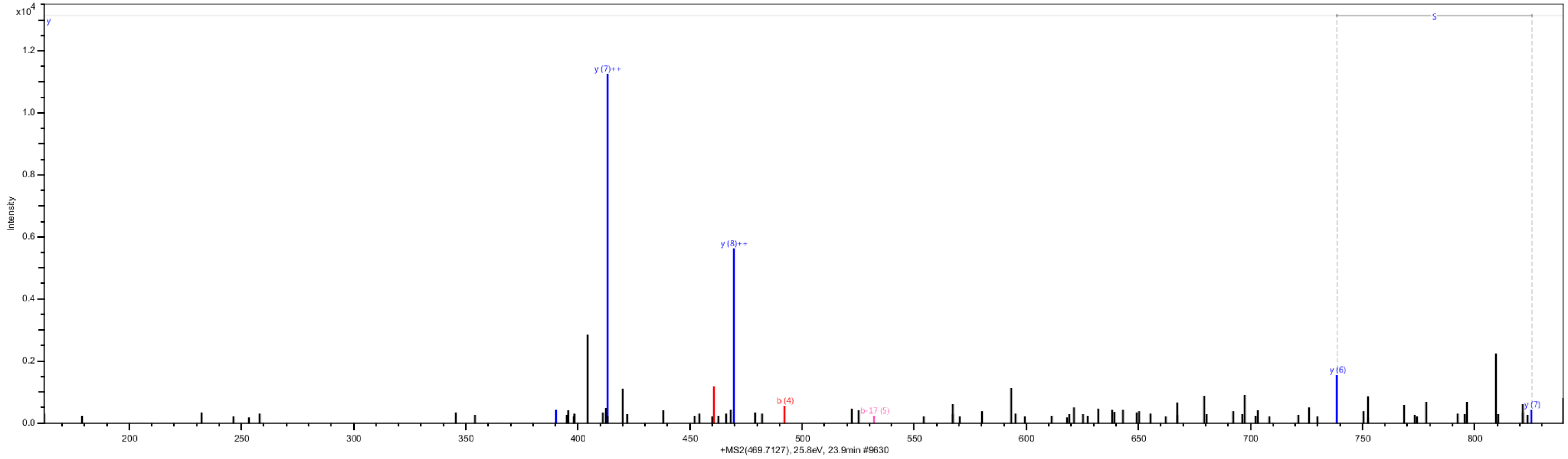

PYRD1_HUMAN

Accession: PYRD1_HUMAN
Database: SwissProt
MW [kDa] / pI: 55.8 / 5.6
Score: 16.7
Seq. Coverage [%]: 1.4
No. of Peptides: 1

Peptide Match

Sequence: K.KSFTFPR.D
Score: 16.7
Modifications:

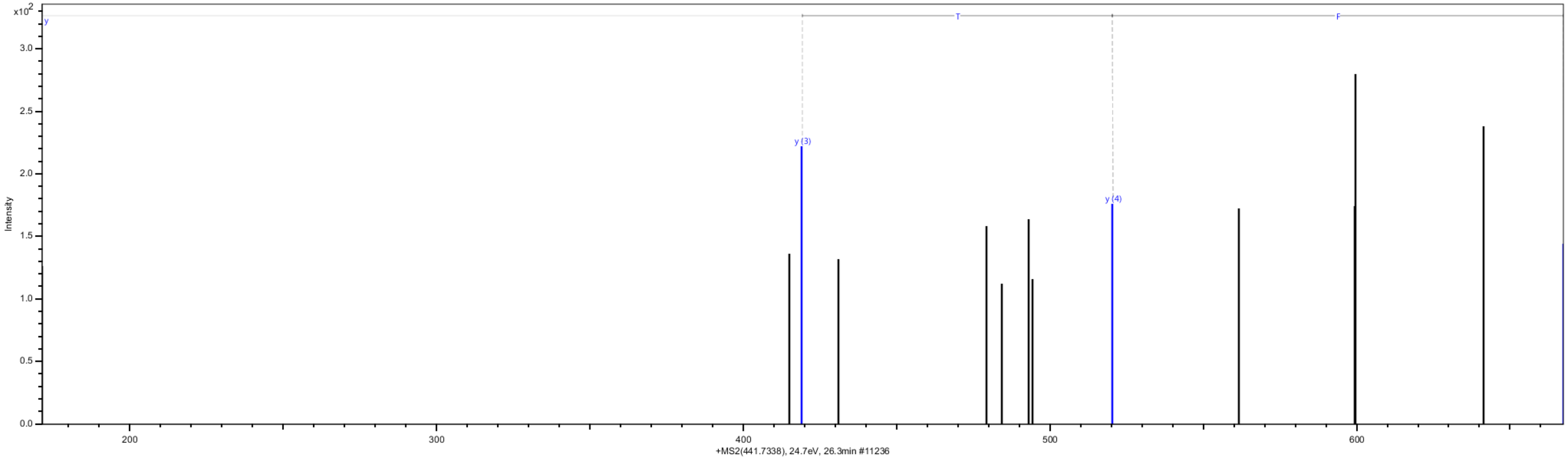

MED16_HUMAN

Accession: MED16_HUMAN
Database: SwissProt
MW [kDa] / pI: 96.7 / 7.1
Score: 16.7
Seq. Coverage [%]: 1.4
No. of Peptides: 1

Peptide Match

Sequence: K.ACTRCGCVTMLK.S
Score: 16.7
Modifications: Carbamidomethyl: 2, 5, 7

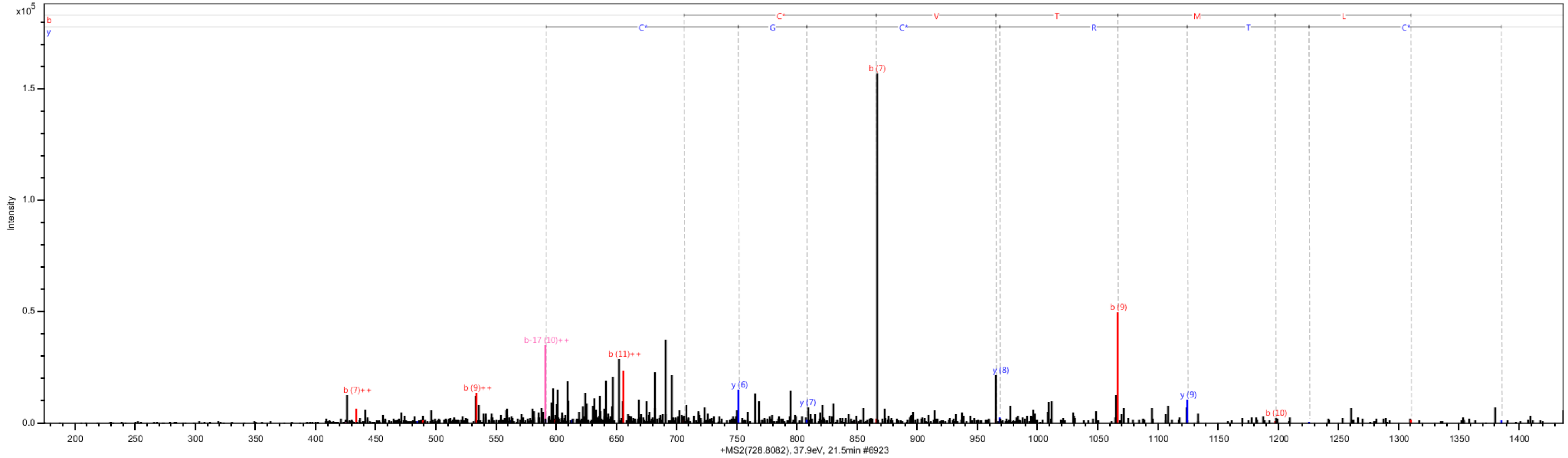

CEBOS_HUMAN

**Accession:**	CEBOS_HUMAN	**Score:**	16.7
**Database:**	SwissProt	**Seq. Coverage [%]:**	15.0
**MW [kDa] / pI:**	9.3 / 9.7	**No. of Peptides:**	1

Peptide Match

**Sequence:**	K.SGMYGIRELDQK.T	**Score:**	16.7
**Modifications:**	Oxidation: 3		

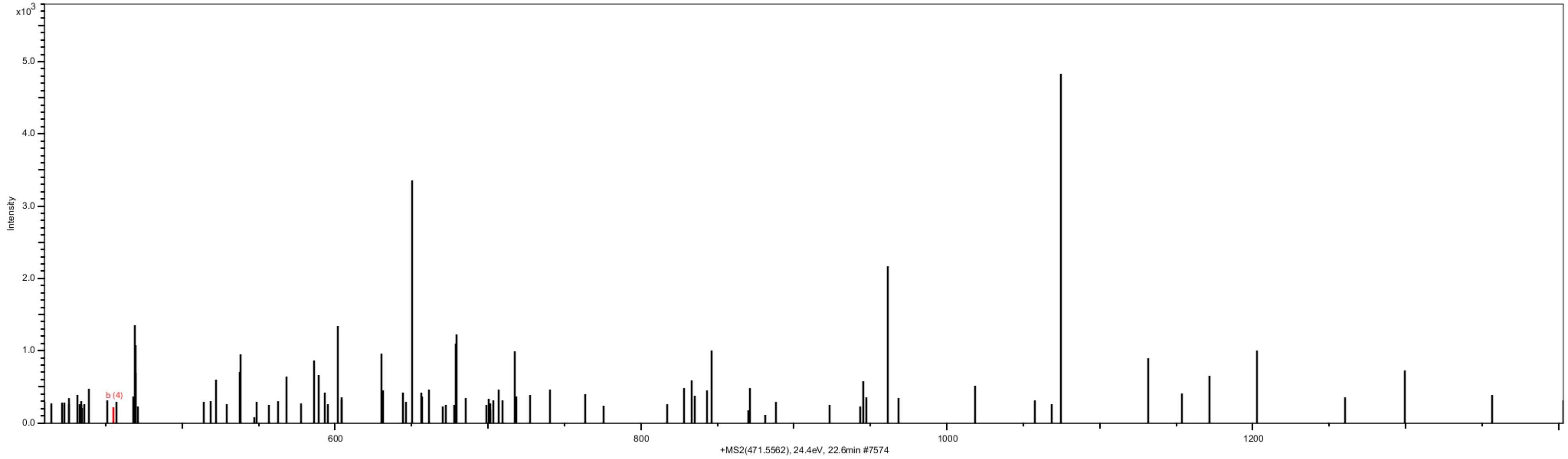

CG026_HUMAN

Accession: CG026_HUMAN
Database: SwissProt
MW [kDa] / pI: 50.0 / 7.6
Score: 16.8
Seq. Coverage [%]: 2.4
No. of Peptides: 1

Peptide Match

Sequence: K.ADDSRMSLLGK.L
Score: 16.8
Modifications: Oxidation: 6

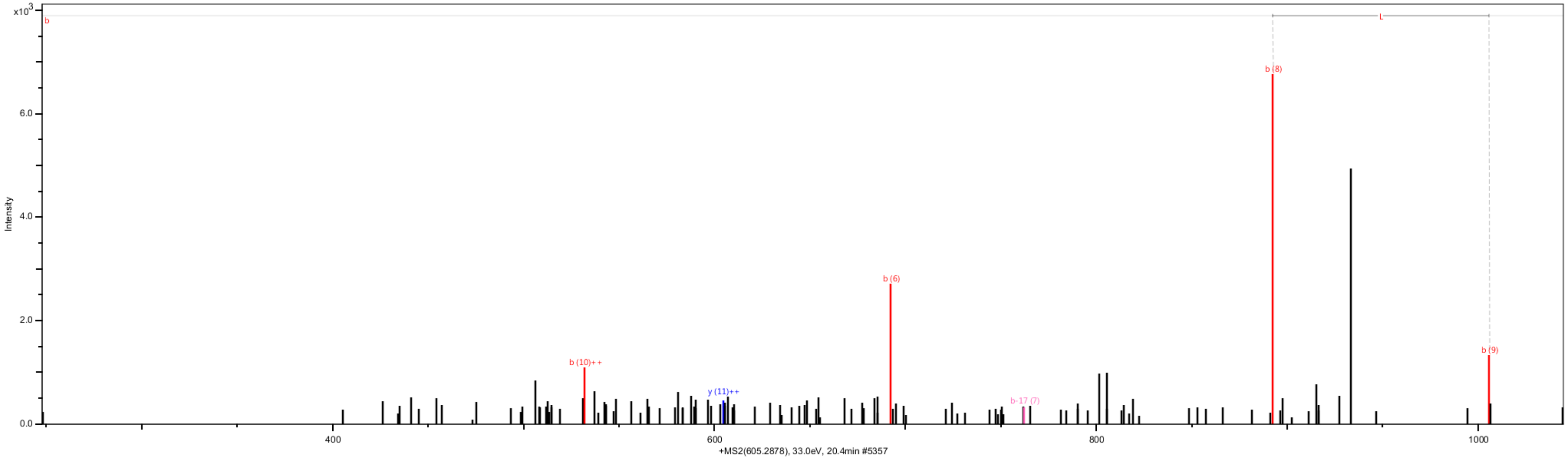

TICN2_HUMAN

Accession: TICN2_HUMAN
Database: SwissProt
MW [kDa] / pI: 46.7 / 4.7
Score: 16.8
Seq. Coverage [%]: 4.0
No. of Peptides: 1

Peptide Match

Sequence: K.QNGSASSVAGPASGLDK.S
Score: 16.8
Modifications:

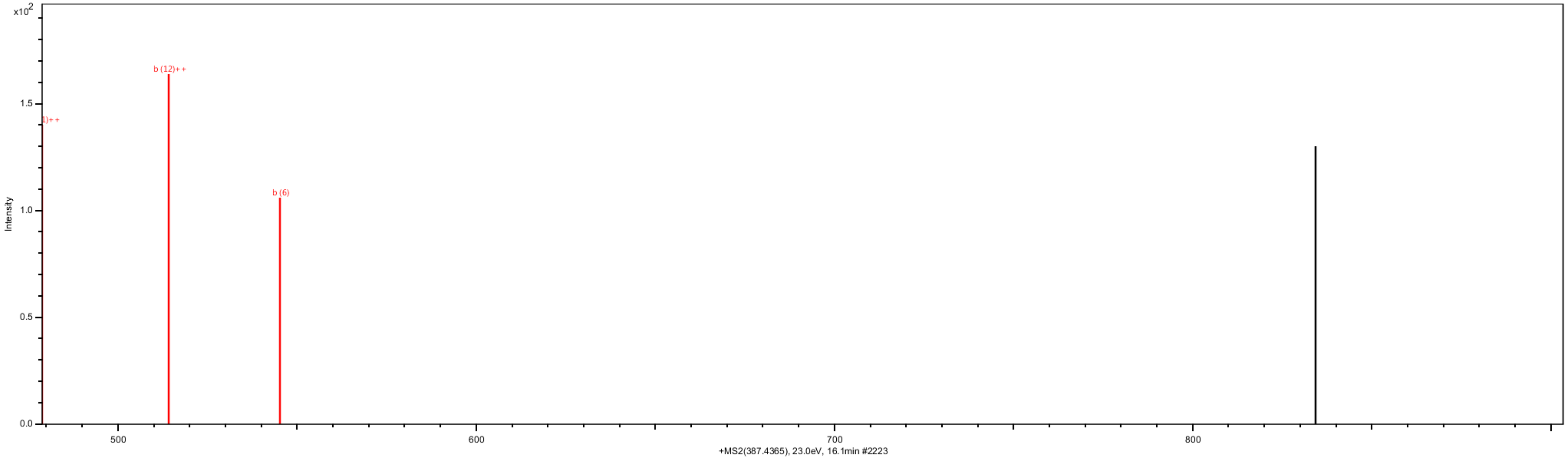

PRDX2_HUMAN

**Accession:**	PRDX2_HUMAN	**Score:**	16.8
**Database:**	SwissProt	**Seq. Coverage [%]:**	5.6
**MW [kDa] / pI:**	21.9 / 5.7	**No. of Peptides:**	1

Peptide Match

**Sequence:**	R.QITVNDLPVGR.S	**Score:**	16.8
**Modifications:**			

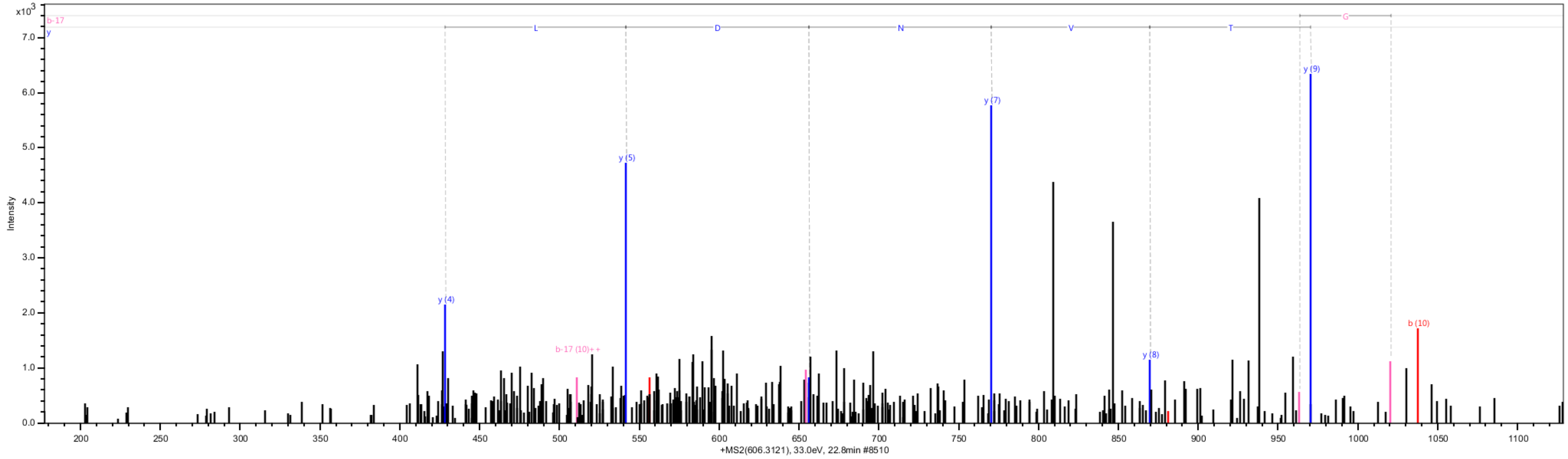

FHOD3_HUMAN

**Accession:**	FHOD3_HUMAN	**Score:**	16.6
**Database:**	SwissProt	**Seq. Coverage [%]:**	0.8
**MW [kDa] / pI:**	158.5 / 5.7	**No. of Peptides:**	1

Peptide Match

**Sequence:**	R.FMLDMLYAHNR.K	**Score:**	16.6
**Modifications:**	Oxidation: 2, 5		

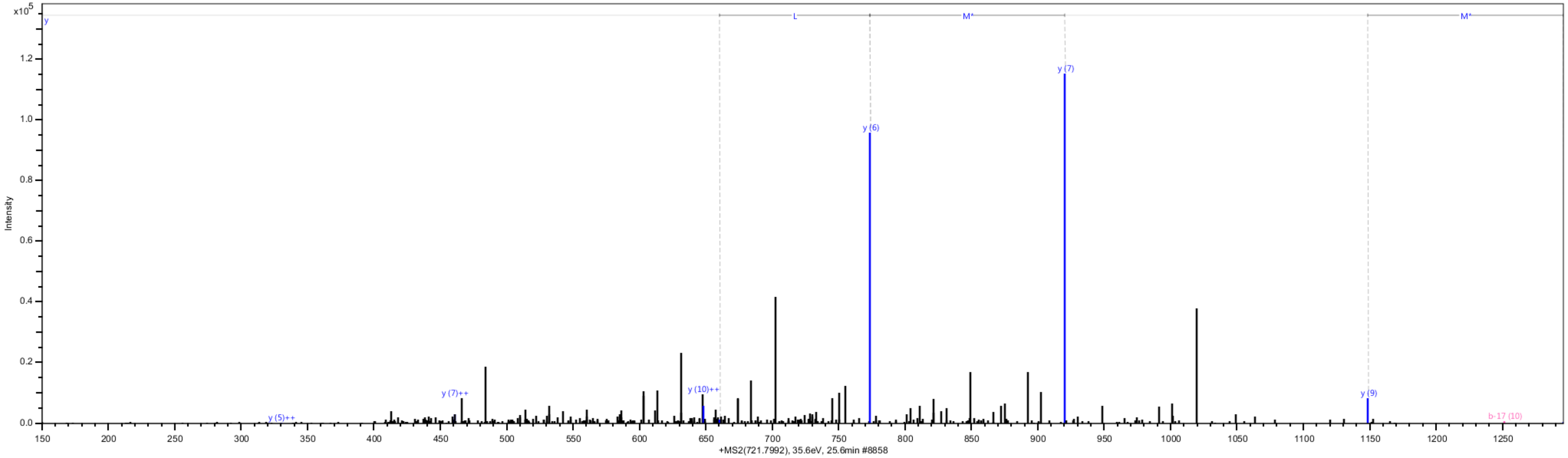

MRCKA_HUMAN

**Accession:**	MRCKA_HUMAN	**Score:**	16.6
**Database:**	SwissProt	**Seq. Coverage [%]:**	0.6
**MW [kDa] / pI:**	197.2 / 6.2	**No. of Peptides:**	1

Peptide Match

**Sequence:**	K.FEDRLPEDMAR.F	**Score:**	16.6
**Modifications:**			

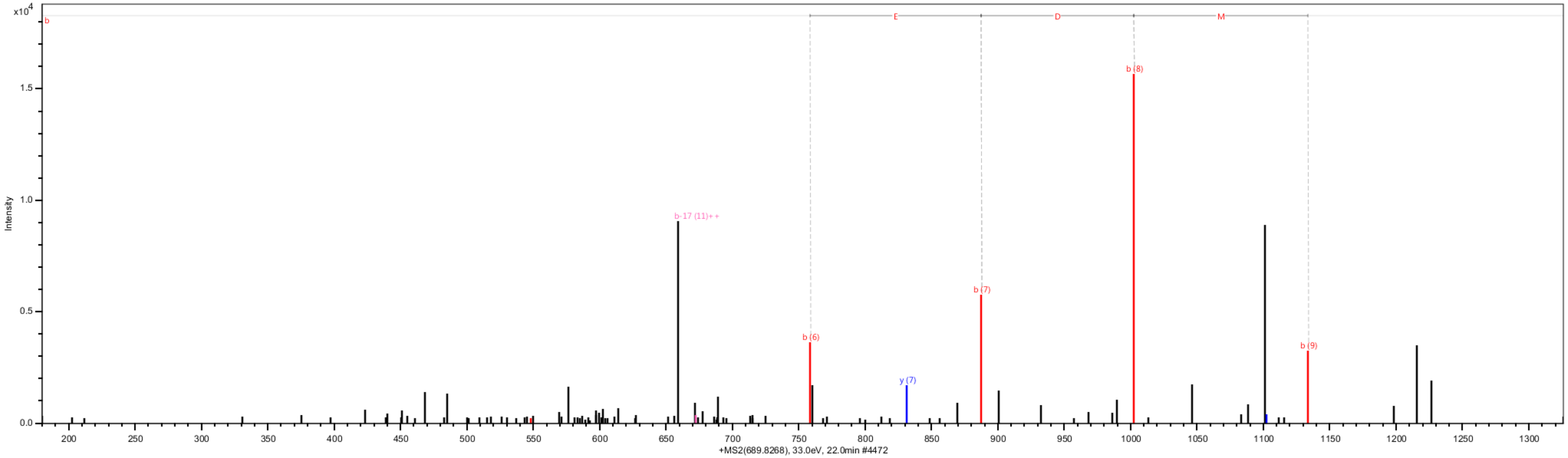

TGO1_HUMAN

**Accession:**	TGO1_HUMAN	**Score:**	16.6
**Database:**	SwissProt	**Seq. Coverage [%]:**	0.4
**MW [kDa] / pI:**	213.6 / 4.8	**No. of Peptides:**	1

Peptide Match

**Sequence:**	K.ENTELVQK.L	**Score:**	16.6
**Modifications:**			

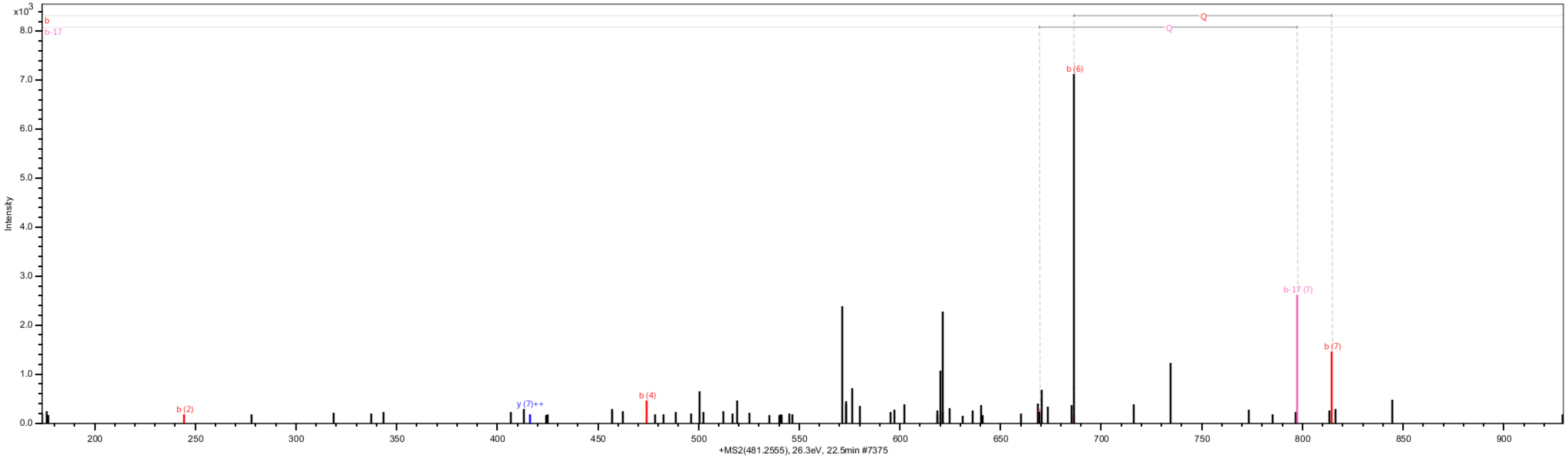

CARD8_HUMAN

Accession: CARD8_HUMAN
Database: SwissProt
MW [kDa] / pI: 48.9 / 5.1
Score: 16.6
Seq. Coverage [%]: 2.1
No. of Peptides: 1

Peptide Match

Sequence: R.DPYLVSYLR.Q
Score: 16.6
Modifications:

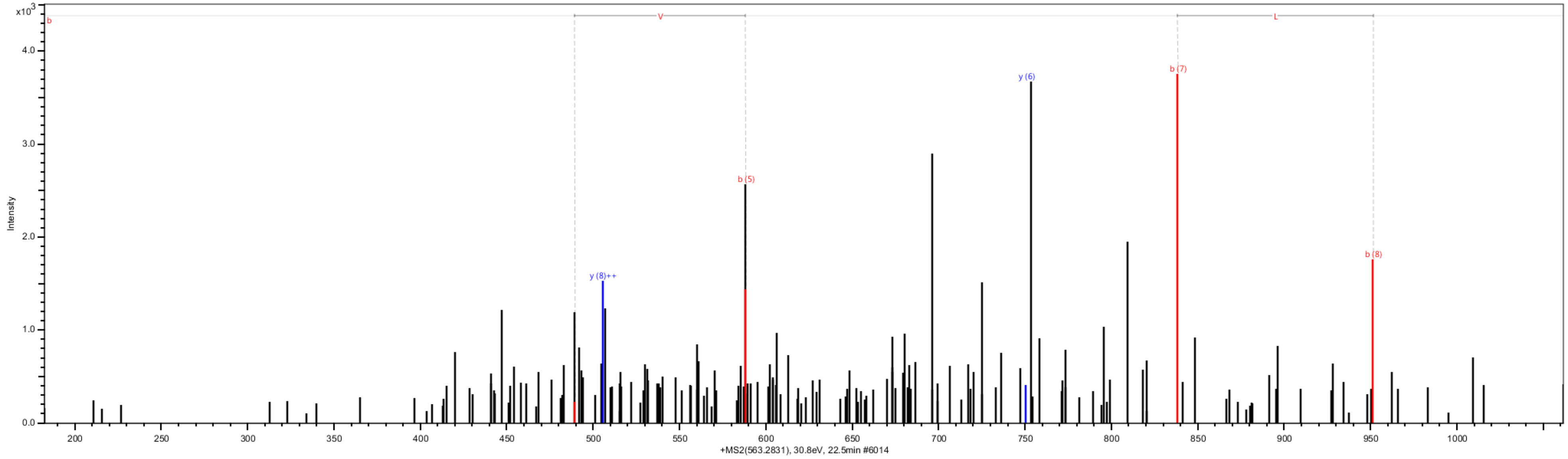

MUS81_HUMAN

Accession: MUS81_HUMAN
Database: SwissProt
MW [kDa] / pI: 61.1 / 9.8
Score: 16.7
Seq. Coverage [%]: 1.5
No. of Peptides: 1

Peptide Match

Sequence: R.LHVTHTVR.K
Score: 16.7
Modifications:

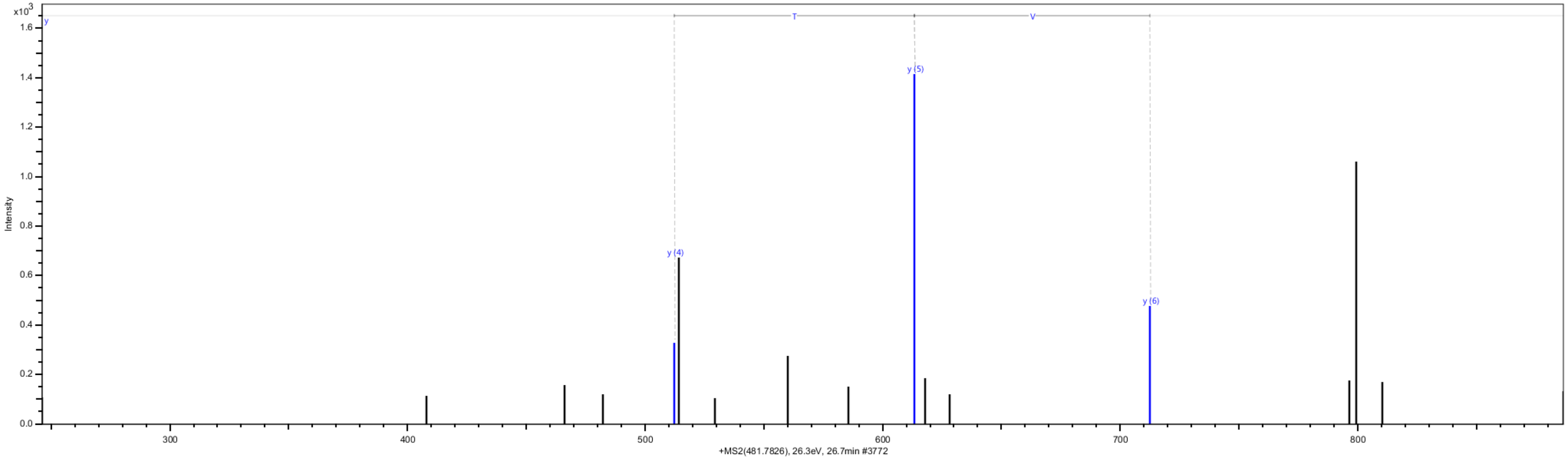

MMP16_HUMAN

Accession: MMP16_HUMAN
Database: SwissProt
MW [kDa] / pI: 69.5 / 8.7
Score: 16.7
Seq. Coverage [%]: 1.5
No. of Peptides: 1

Peptide Match

Sequence: M.ILLTFSTGR.R
Score: 16.6
Modifications:

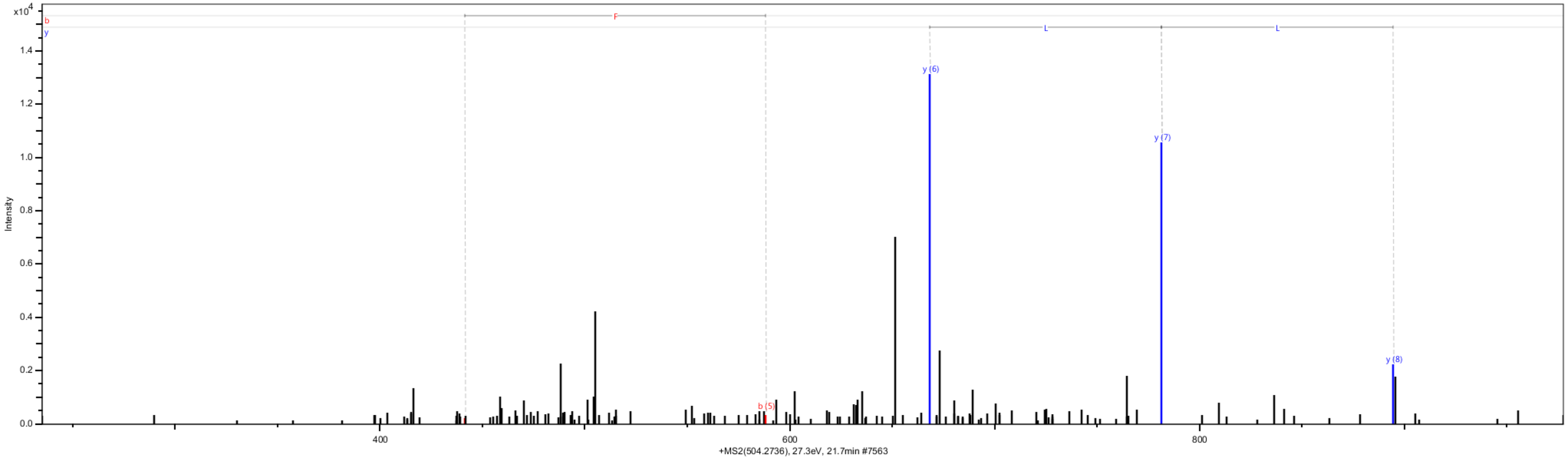

CD36_HUMAN

Accession: CD36_HUMAN
Database: SwissProt
MW [kDa] / pI: 53.0 / 8.2
Score: 16.7
Seq. Coverage [%]: 1.7
No. of Peptides: 1

Peptide Match

Sequence: K.VAIIDTYK.G
Score: 16.7
Modifications:

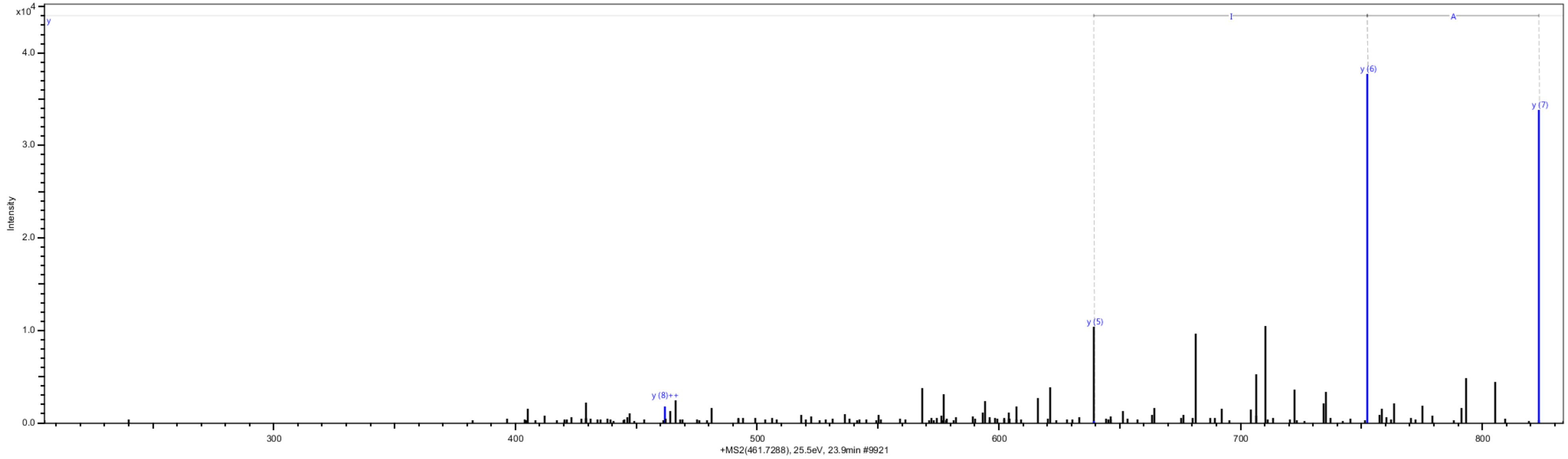

GALE_HUMAN

Accession: GALE_HUMAN
Database: SwissProt
MW [kDa] / pI: 38.3 / 6.3
Score: 16.7
Seq. Coverage [%]: 2.3
No. of Peptides: 1

Peptide Match

Sequence: K.TWNAVLLR.Y
Score: 16.7
Modifications:

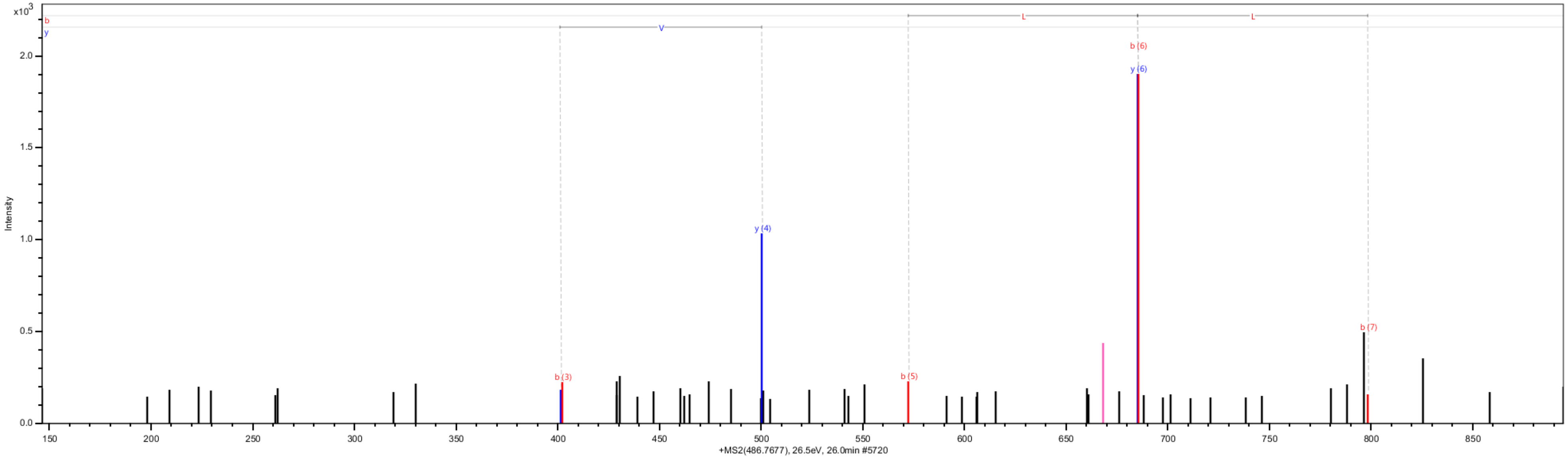

GMDS_HUMAN

**Accession:**	GMDS_HUMAN	**Score:**	16.5
**Database:**	SwissProt	**Seq. Coverage [%]:**	4.3
**MW [kDa] / pI:**	41.9 / 6.9	**No. of Peptides:**	1

Peptide Match

**Sequence:**	R.EMVHADVELMRTNPNA.-	**Score:**	16.5
**Modifications:**	Oxidation: 2		

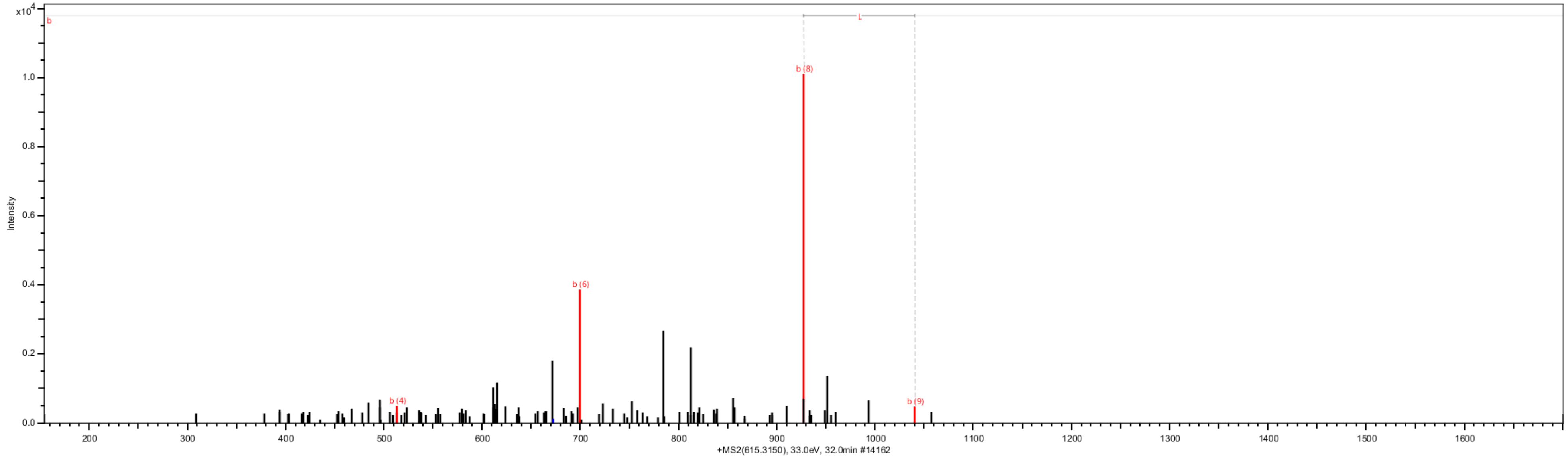

K1C14_HUMAN

Accession: K1C14_HUMAN
Database: SwissProt
MW [kDa] / pI: 51.5 / 5.1
Score: 16.5
Seq. Coverage [%]: 2.3
No. of Peptides: 1

Peptide Match

Sequence: R.ADLEMQIESLK.E
Score: 16.5
Modifications:

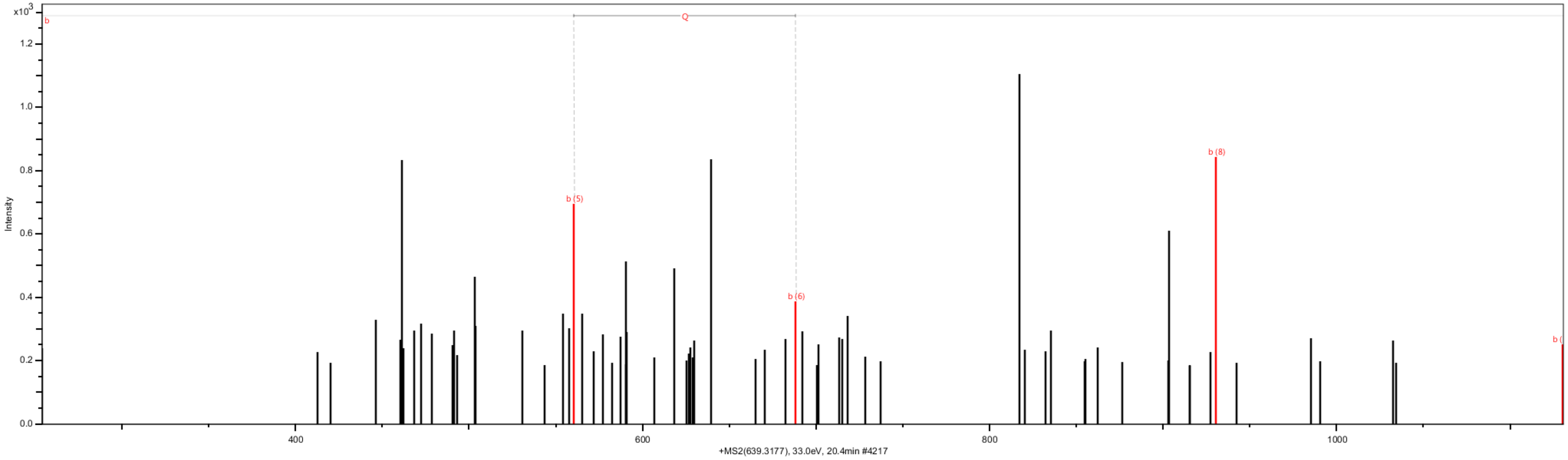

MCM2_HUMAN

**Accession:**	MCM2_HUMAN	**Score:**	16.5
**Database:**	SwissProt	**Seq. Coverage [%]:**	1.2
**MW [kDa] / pI:**	101.8 / 5.3	**No. of Peptides:**	1

Peptide Match

**Sequence:**	R.VMLESFIDTQK.F	**Score:**	16.5
**Modifications:**	Oxidation: 2		

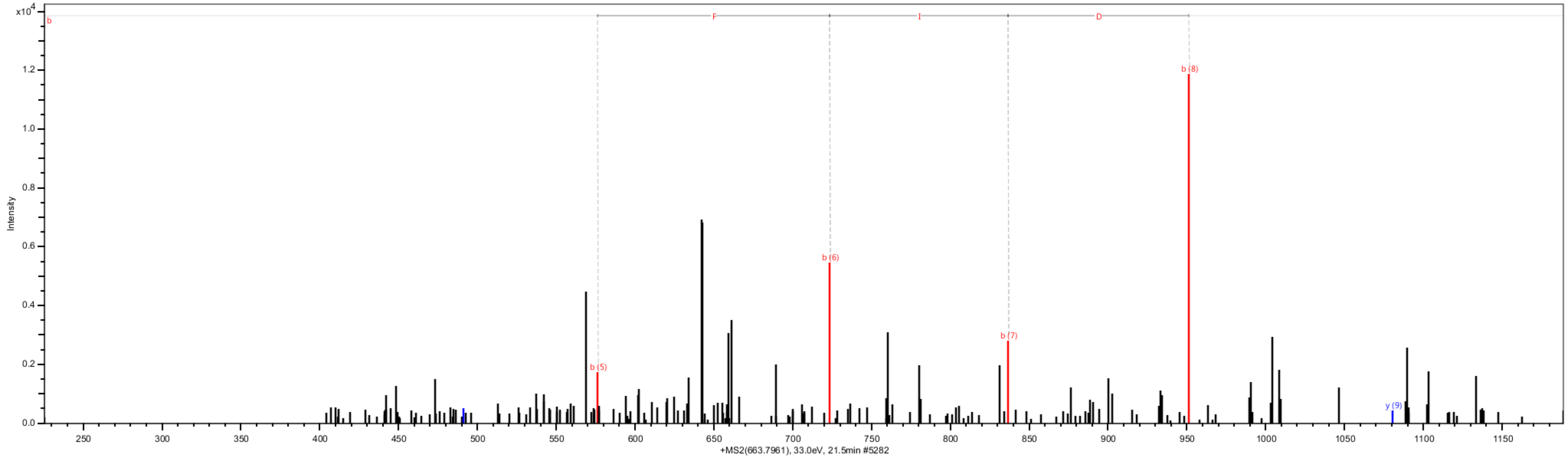

IQEC3_HUMAN

**Accession:**	IQEC3_HUMAN	**Score:**	16.5
**Database:**	SwissProt	**Seq. Coverage [%]:**	1.0
**MW [kDa] / pI:**	127.5 / 6.1	**No. of Peptides:**	1

Peptide Match

**Sequence:**	K.SPSALGKGVLSR.R	**Score:**	16.5
**Modifications:**			

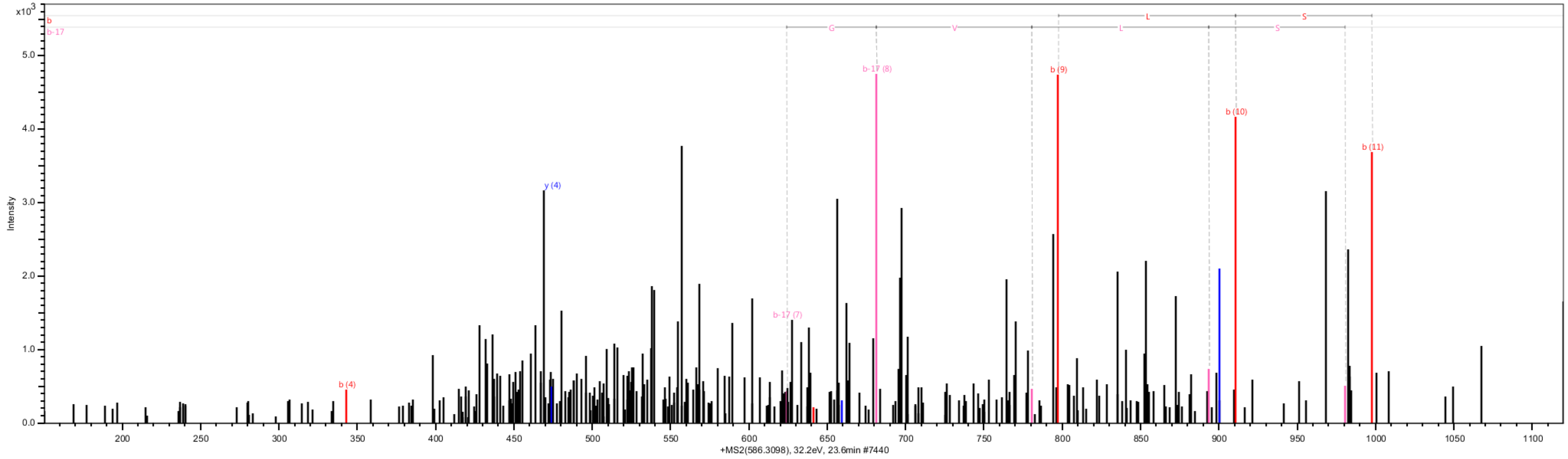

LYSM2_HUMAN

**Accession:**	LYSM2_HUMAN	**Score:**	16.5
**Database:**	SwissProt	**Seq. Coverage [%]:**	7.4
**MW [kDa] / pI:**	23.4 / 5.3	**No. of Peptides:**	1

Peptide Match

**Sequence:**	-.MADSSPALSLREGGPR.A	**Score:**	16.5
**Modifications:**	Oxidation: 1		

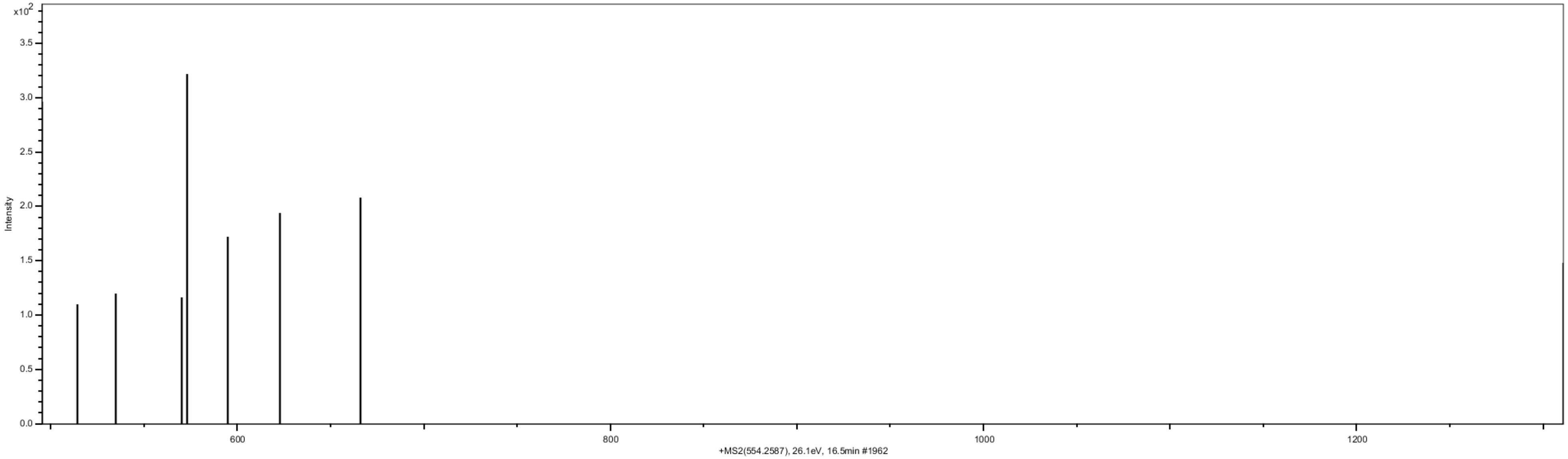

TTC29_HUMAN

Accession: TTC29_HUMAN
Database: SwissProt
MW [kDa] / pI: 55.0 / 5.5
Score: 16.5
Seq. Coverage [%]: 1.9
No. of Peptides: 1

Peptide Match

Sequence: R.CFKIAQLIK.I
Score: 16.5
Modifications: Carbamidomethyl: 1

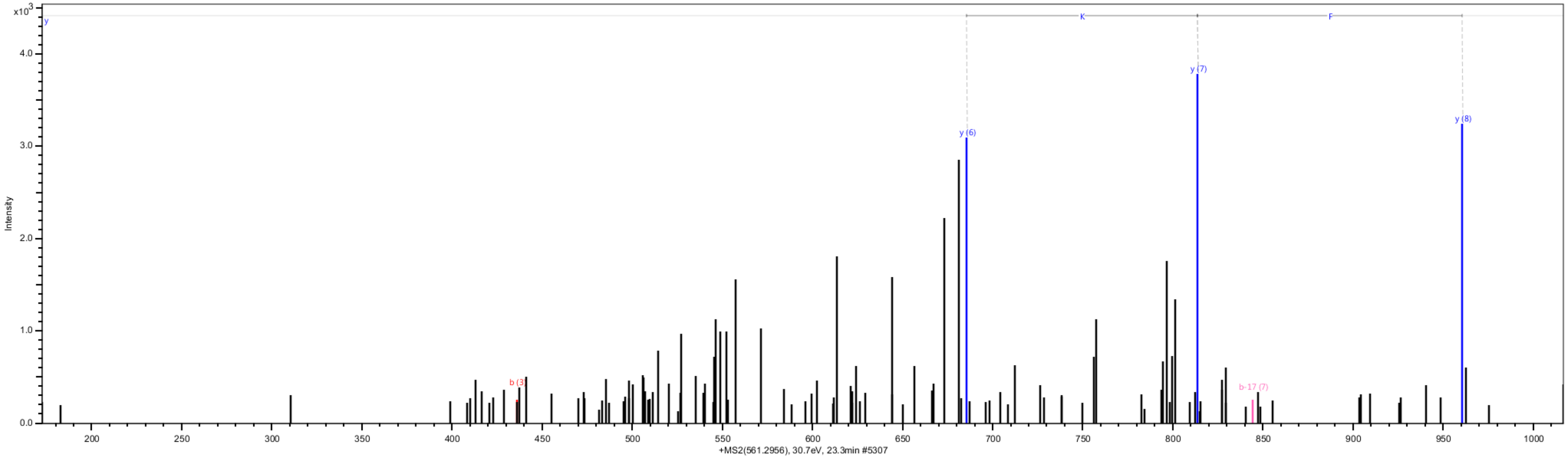

PARI_HUMAN

**Accession:**	PARI_HUMAN	**Score:**	16.6
**Database:**	SwissProt	**Seq. Coverage [%]:**	1.9
**MW [kDa] / pI:**	65.0 / 9.0	**No. of Peptides:**	1

Peptide Match

**Sequence:**	R.GLGREAFTDLK.H	**Score:**	16.6
**Modifications:**			

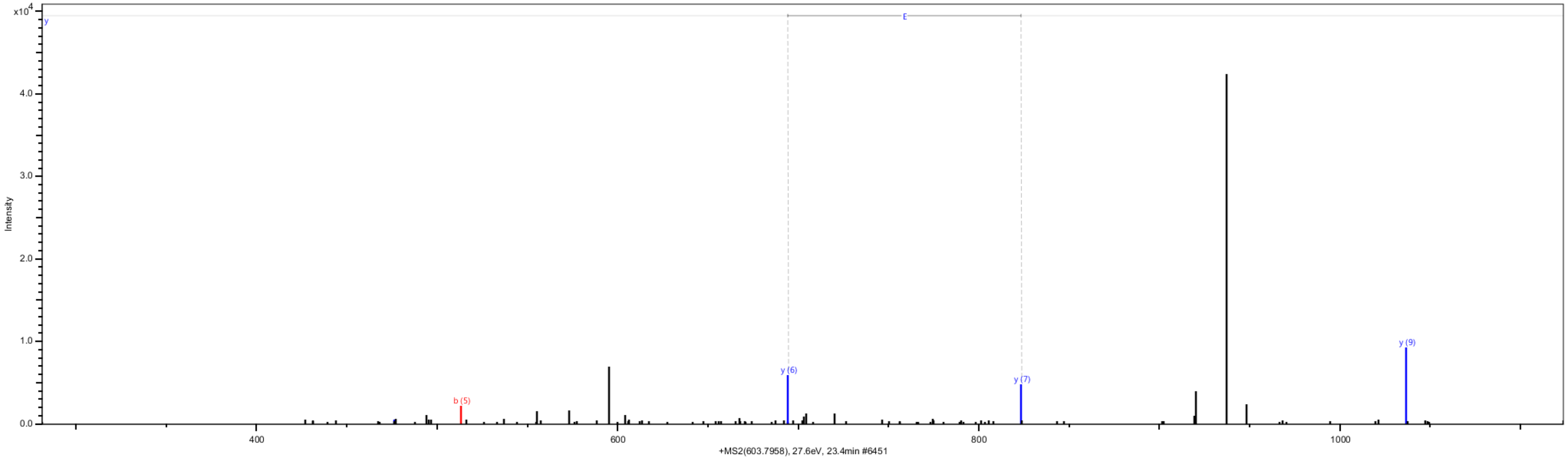

SNX13_HUMAN

**Accession:**	SNX13_HUMAN	**Score:**	16.6
**Database:**	SwissProt	**Seq. Coverage [%]:**	1.1
**MW [kDa] / pI:**	112.1 / 6.2	**No. of Peptides:**	1

Peptide Match

**Sequence:**	K.MSDNMGKMSER.L	**Score:**	16.6
**Modifications:**	Oxidation: 8		

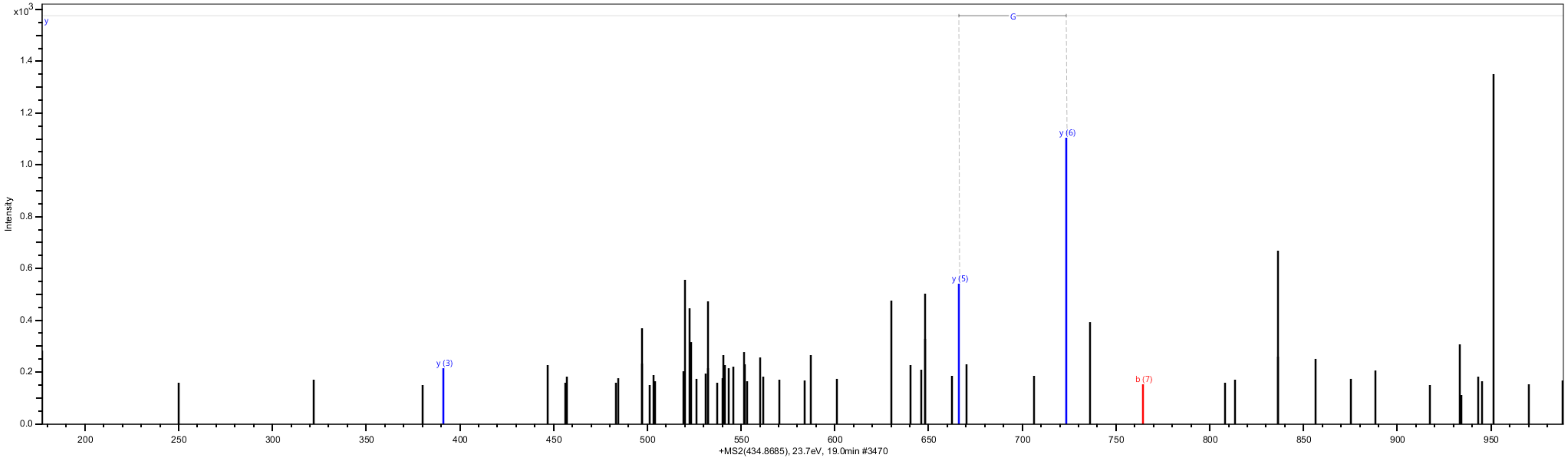

MYL6_HUMAN

**Accession:**	MYL6_HUMAN	**Score:**	17.2
**Database:**	SwissProt	**Seq. Coverage [%]:**	10.6
**MW [kDa] / pI:**	16.9 / 4.6	**No. of Peptides:**	1

Peptide Match

**Sequence:**	R.VFDKEGNGTVMGAEIR.H	**Score:**	17.2
**Modifications:**			

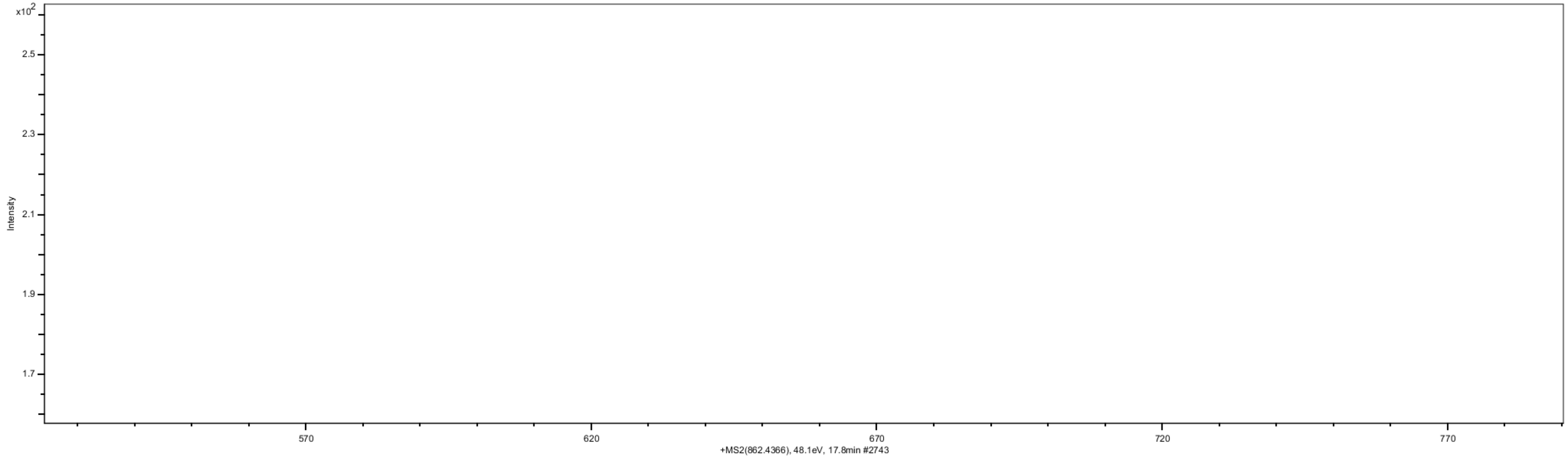

SKOR2_HUMAN

Accession: SKOR2_HUMAN
Database: SwissProt
MW [kDa] / pI: 104.2 / 6.0
Score: 17.2
Seq. Coverage [%]: 1.0
No. of Peptides: 1

Peptide Match

Sequence: R.FLQGRGPSEK.G
Score: 17.2
Modifications:

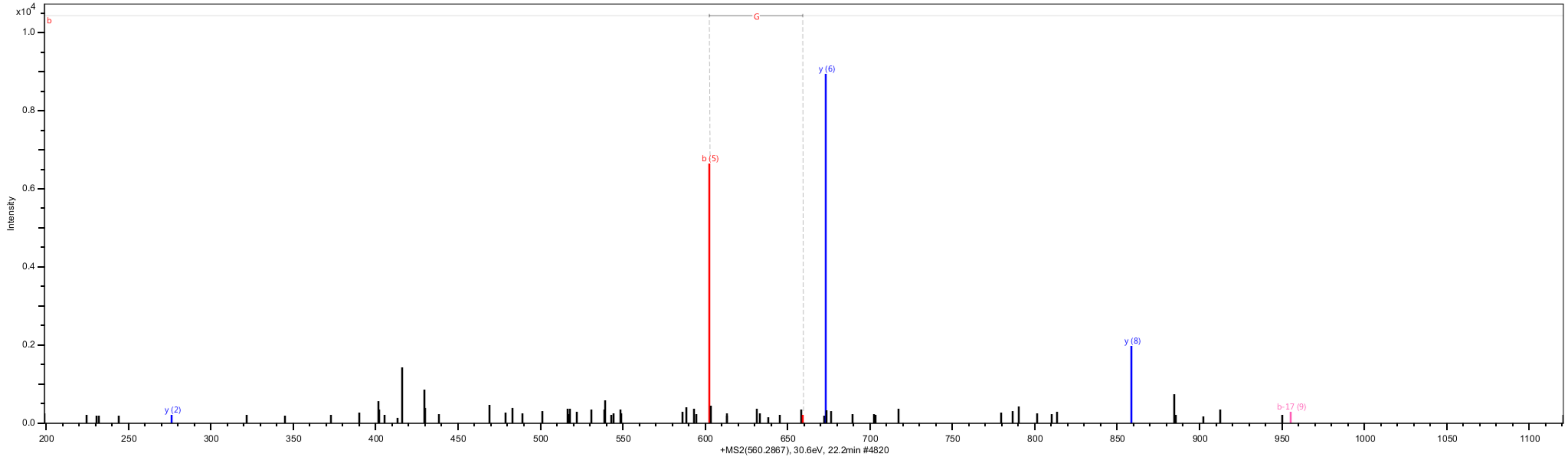

PALB2_HUMAN

Accession: PALB2_HUMAN
Database: SwissProt
MW [kDa] / pI: 131.2 / 6.0
Score: 17.2
Seq. Coverage [%]: 0.8
No. of Peptides: 1

Peptide Match

Sequence: K.AVAQAPGRR.Y
Score: 17.2
Modifications:

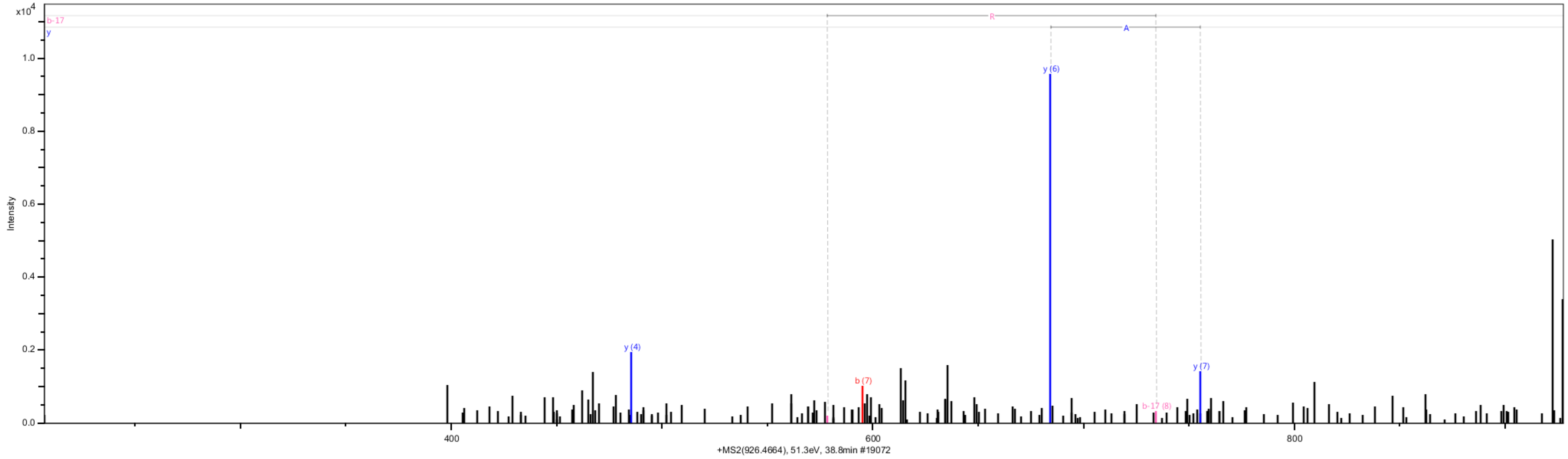

TNFL8_HUMAN

**Accession:**	TNFL8_HUMAN	**Score:**	17.2
**Database:**	SwissProt	**Seq. Coverage [%]:**	6.0
**MW [kDa] / pI:**	26.0 / 7.6	**No. of Peptides:**	1

Peptide Match

**Sequence:**	K.QALVTVCESGMQTK.H	**Score:**	17.2
**Modifications:**	Carbamidomethyl: 7; Oxidation: 11		

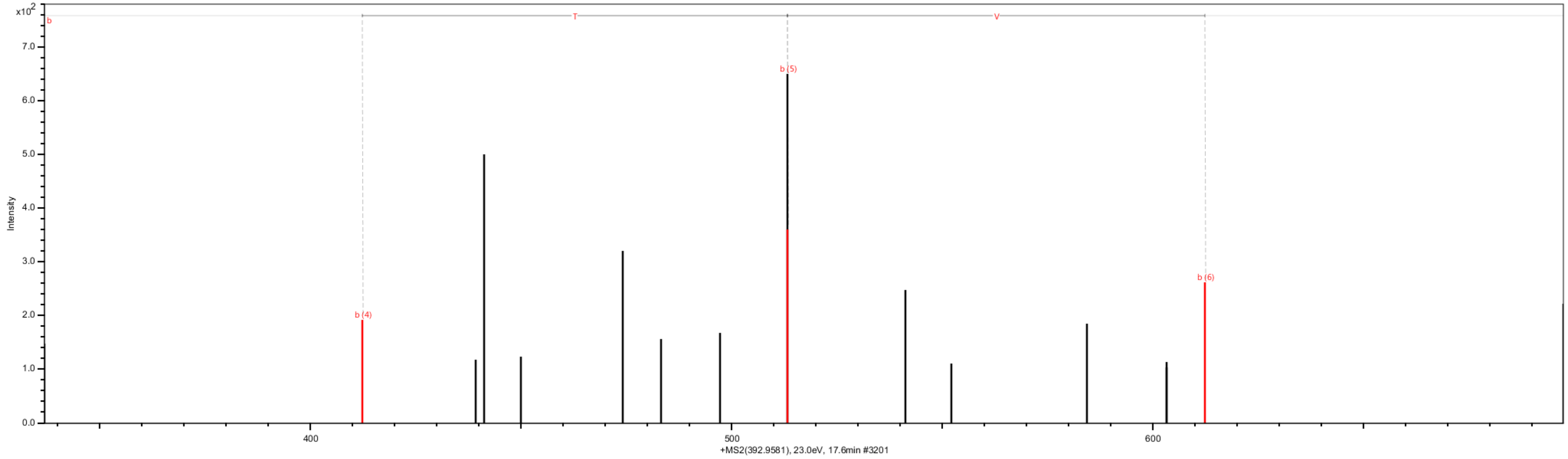

OR3A3_HUMAN

**Accession:**	OR3A3_HUMAN	**Score:**	17.3
**Database:**	SwissProt	**Seq. Coverage [%]:**	4.7
**MW [kDa] / pI:**	34.9 / 7.0	**No. of Peptides:**	1

Peptide Match

**Sequence:**	-.MSLQKLMEPEAGTNR.T	**Score:**	17.2
**Modifications:**	Oxidation: 1, 7		

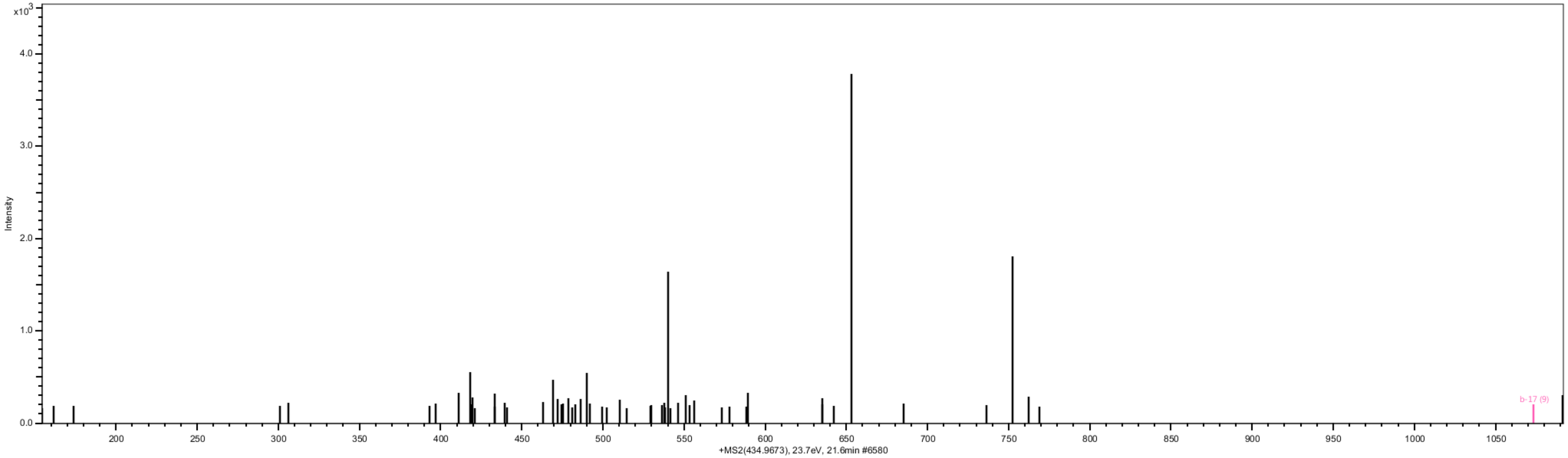

JADE2_HUMAN

Accession: JADE2_HUMAN
Database: SwissProt
MW [kDa] / pI: 87.4 / 5.1
Score: 17.2
Seq. Coverage [%]: 1.3
No. of Peptides: 1

Peptide Match

Sequence: R.TILADNDEVK.F
Score: 17.2
Modifications:

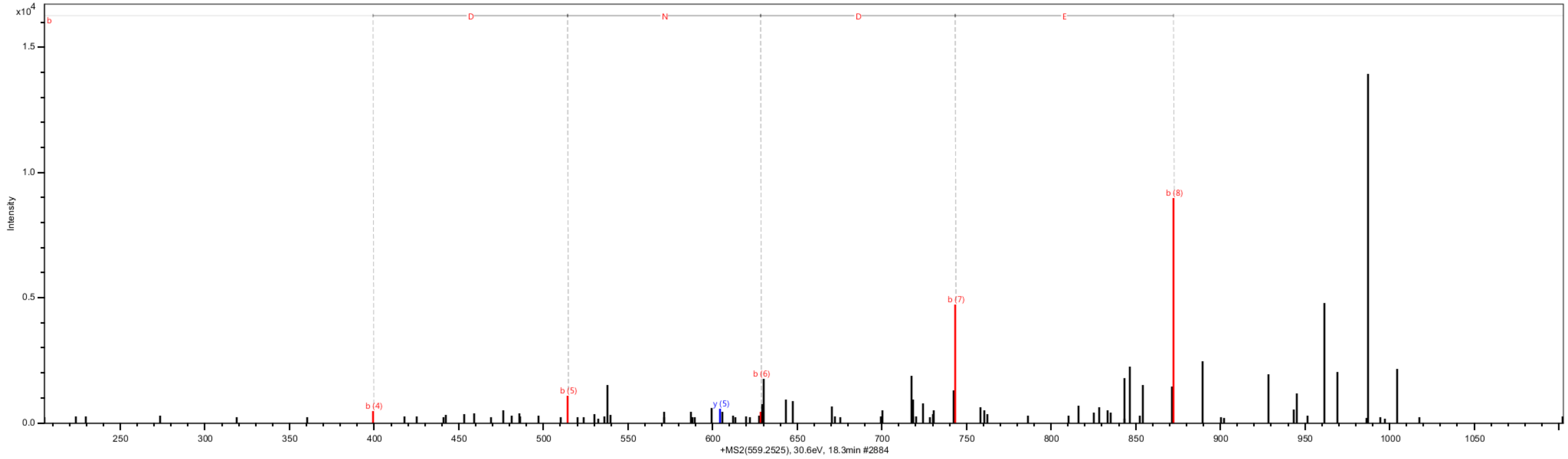

CNBD2_HUMAN

Accession: CNBD2_HUMAN
Database: SwissProt
MW [kDa] / pI: 67.5 / 9.3
Score: 17.3
Seq. Coverage [%]: 2.4
No. of Peptides: 1

Peptide Match

Sequence: K.LNIAFPSDEDMCQK.F
Score: 17.2
Modifications: Carbamidomethyl: 12; Oxidation: 11

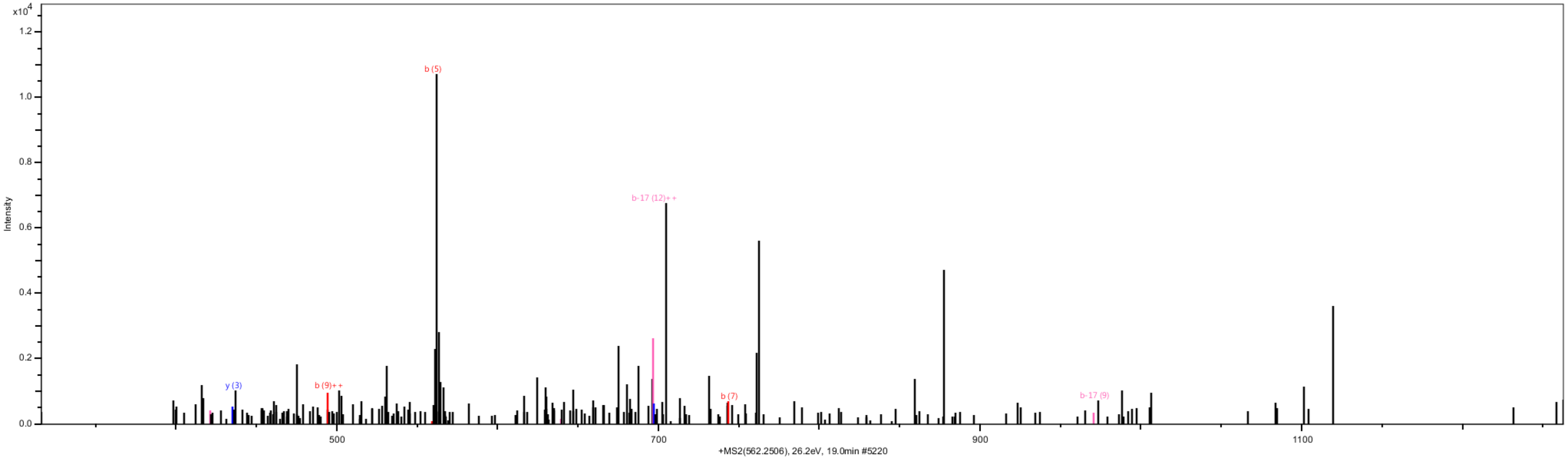

TM50A_HUMAN

Accession: TM50A_HUMAN
Database: SwissProt
MW [kDa] / pI: 17.4 / 5.6
Score: 17.1
Seq. Coverage [%]: 6.4
No. of Peptides: 1

Peptide Match

Sequence: R.CSECIDWGEK.R
Score: 17.1
Modifications: Carbamidomethyl: 1, 4

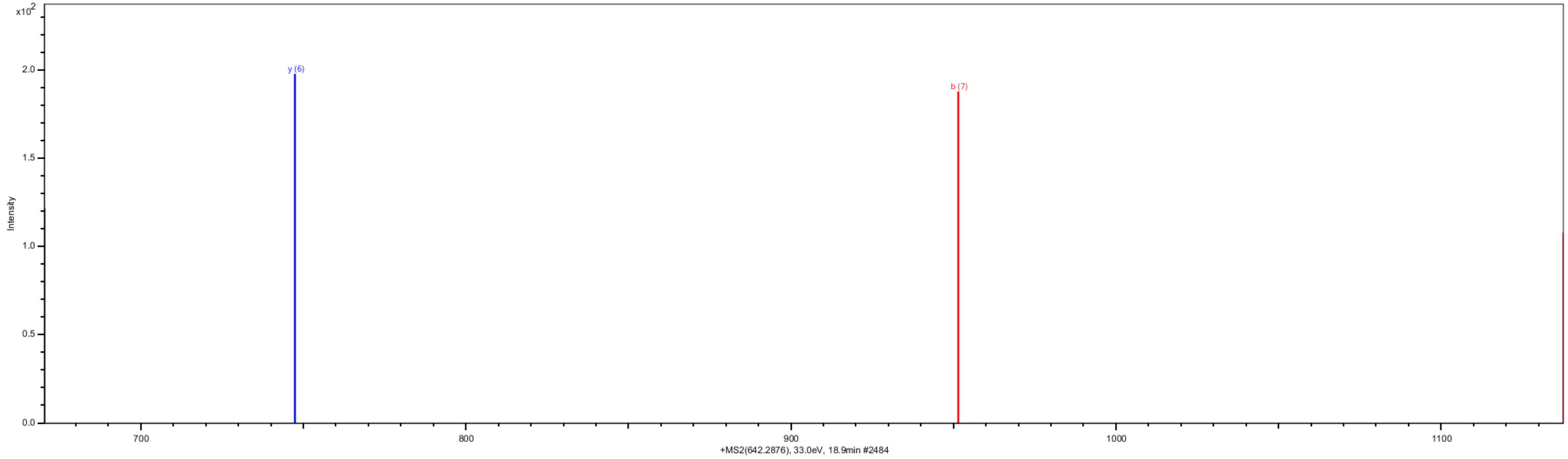

ABCC8_HUMAN

Accession: ABCC8_HUMAN
Database: SwissProt
MW [kDa] / pI: 176.9 / 8.0
Score: 17.1
Seq. Coverage [%]: 0.5
No. of Peptides: 1

Peptide Match

Sequence: R.LVLSSTFR.I
Score: 17.1
Modifications:

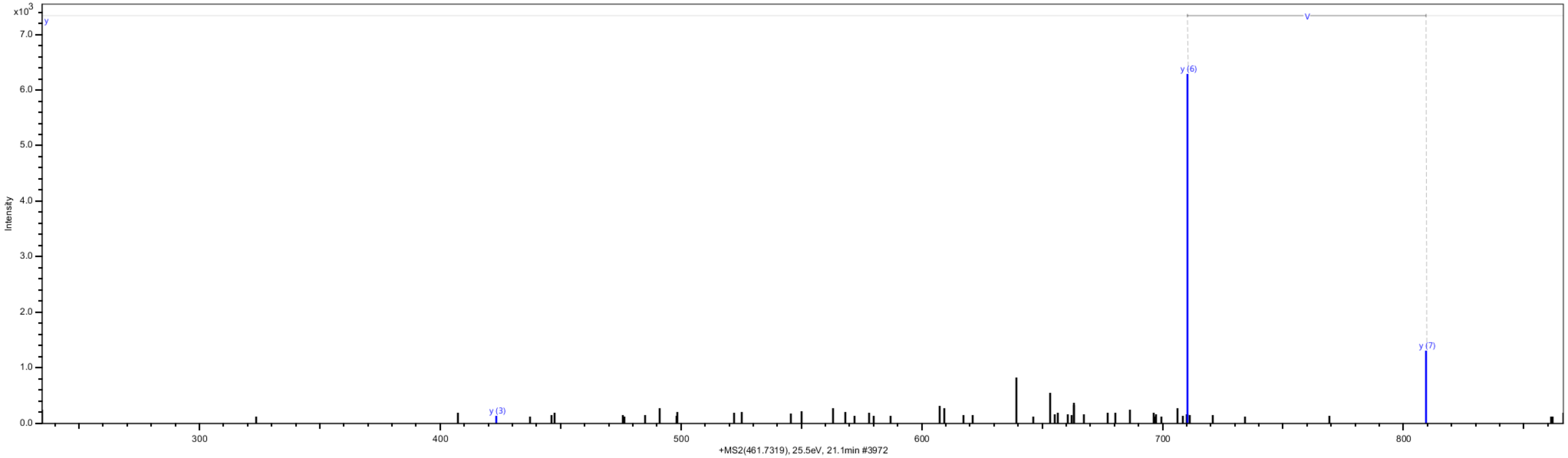

IGSF3_HUMAN

**Accession:**	IGSF3_HUMAN	**Score:**	17.1
**Database:**	SwissProt	**Seq. Coverage [%]:**	0.8
**MW [kDa] / pI:**	135.1 / 5.7	**No. of Peptides:**	1

Peptide Match

Sequence: K.EFTVRLETEK.R **Score:** 17

Modifications:

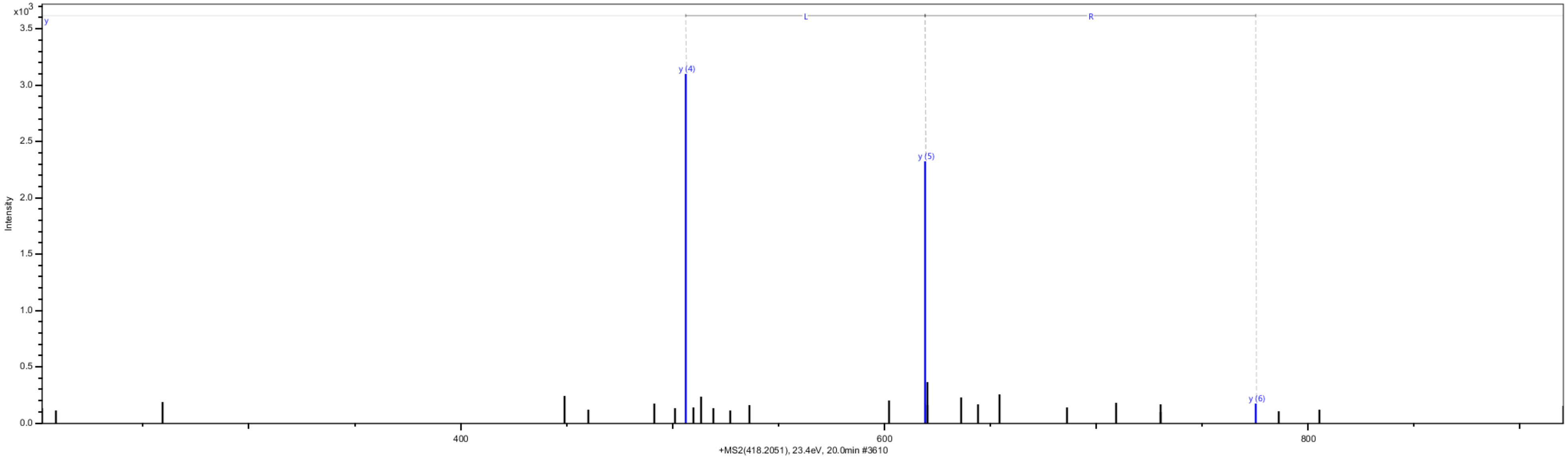

CCD40_HUMAN

**Accession:**	CCD40_HUMAN	**Score:**	17.1
**Database:**	SwissProt	**Seq. Coverage [%]:**	1.0
**MW [kDa] / pI:**	130.0 / 5.2	**No. of Peptides:**	1

Peptide Match

**Sequence:**	K.LDLQELVVATK.Q	**Score:**	17.1
**Modifications:**			

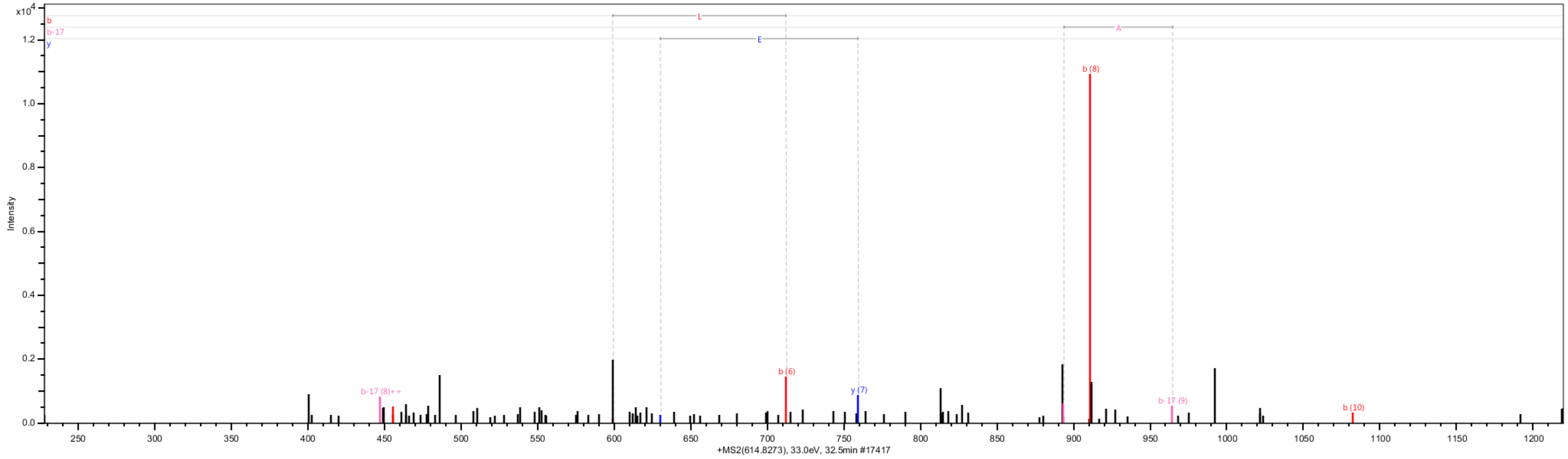

VACHT_HUMAN

Accession: VACHT_HUMAN
Database: SwissProt
MW [kDa] / pI: 56.9 / 5.8
Score: 16.2
Seq. Coverage [%]: 2.4
No. of Peptides: 1

Peptide Match

Sequence: K.LSEAVGAALQEPR.R
Score: 16.2
Modifications:

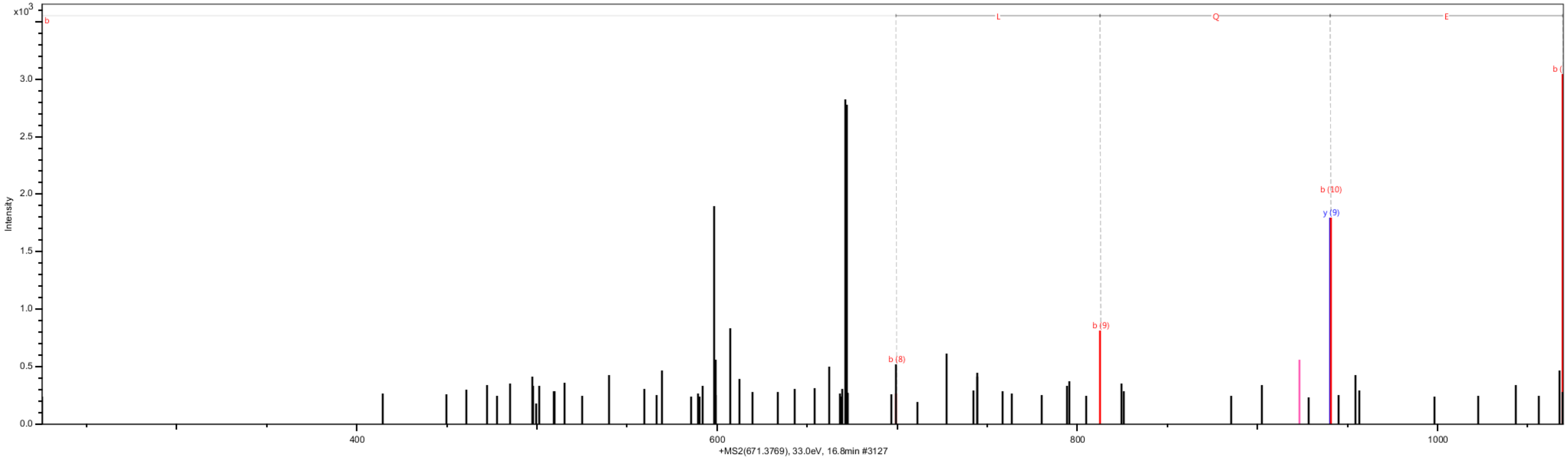

NHRF4_HUMAN

Accession: NHRF4_HUMAN
Database: SwissProt
MW [kDa] / pI: 61.0 / 6.2
Score: 16.1
Seq. Coverage [%]: 3.3
No. of Peptides: 1

Peptide Match

Sequence: R.IQGQGSCVSLTVVDPEADR.F
Score: 16.1
Modifications: Carbamidomethyl: 7

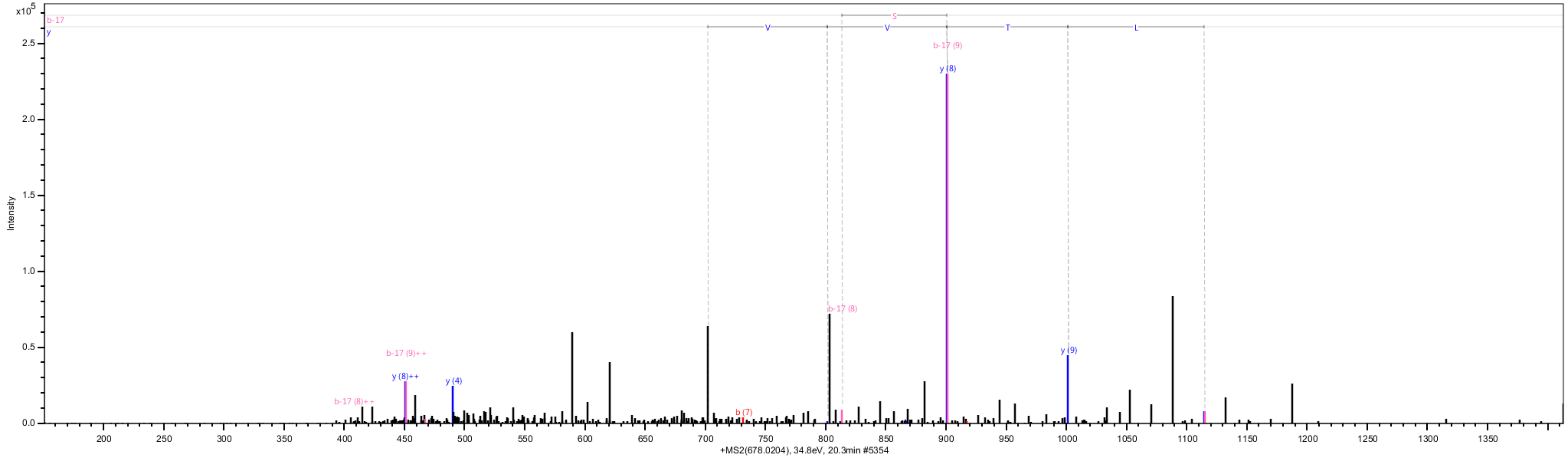

MRM3_HUMAN

**Accession:**	MRM3_HUMAN	**Score:**	16.2
**Database:**	SwissProt	**Seq. Coverage [%]:**	2.6
**MW [kDa] / pI:**	47.0 / 8.9	**No. of Peptides:**	1

Peptide Match

**Sequence:**	K.VLRAGMGAHFR.M	**Score:**	16.2
**Modifications:**			

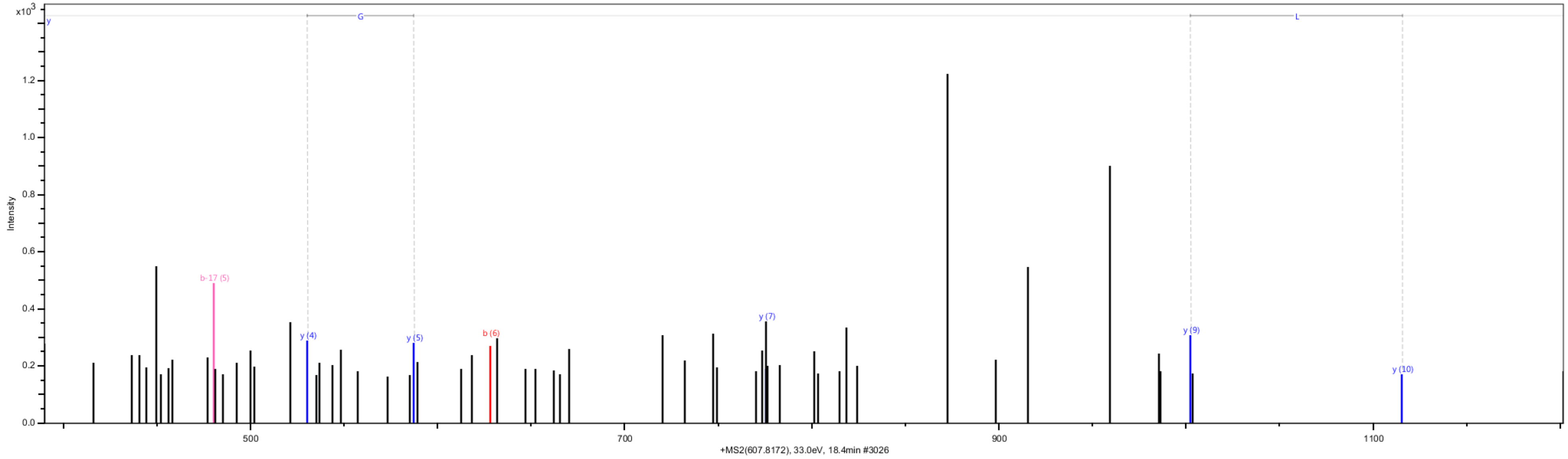

CYTC_HUMAN

Accession: CYTC_HUMAN
Database: SwissProt
MW [kDa] / pI: 15.8 / 9.0
Score: 16.2
Seq. Coverage [%]: 7.5
No. of Peptides: 1

Peptide Match

Sequence: R.ALDFAVGEYNK.A
Score: 16.2
Modifications:

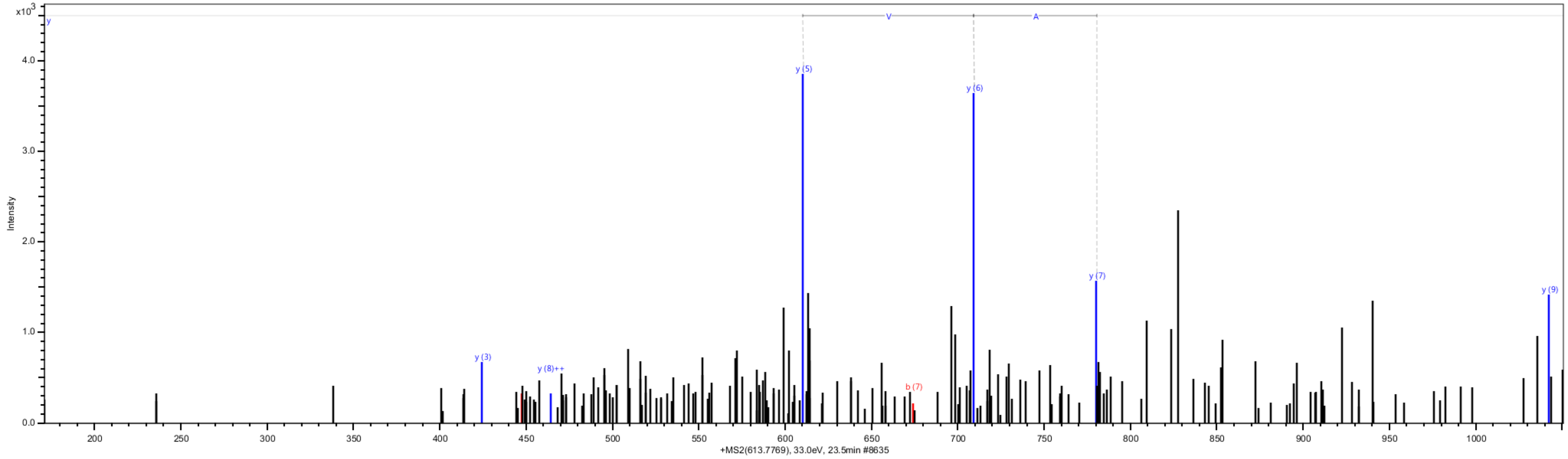

E41L1_HUMAN

Accession: E41L1_HUMAN
Database: SwissProt
MW [kDa] / pI: 98.4 / 5.4
Score: 16.2
Seq. Coverage [%]: 1.5
No. of Peptides: 1

Peptide Match

Sequence: K.IKELKPEQETTPR.H
Score: 16.2
Modifications:

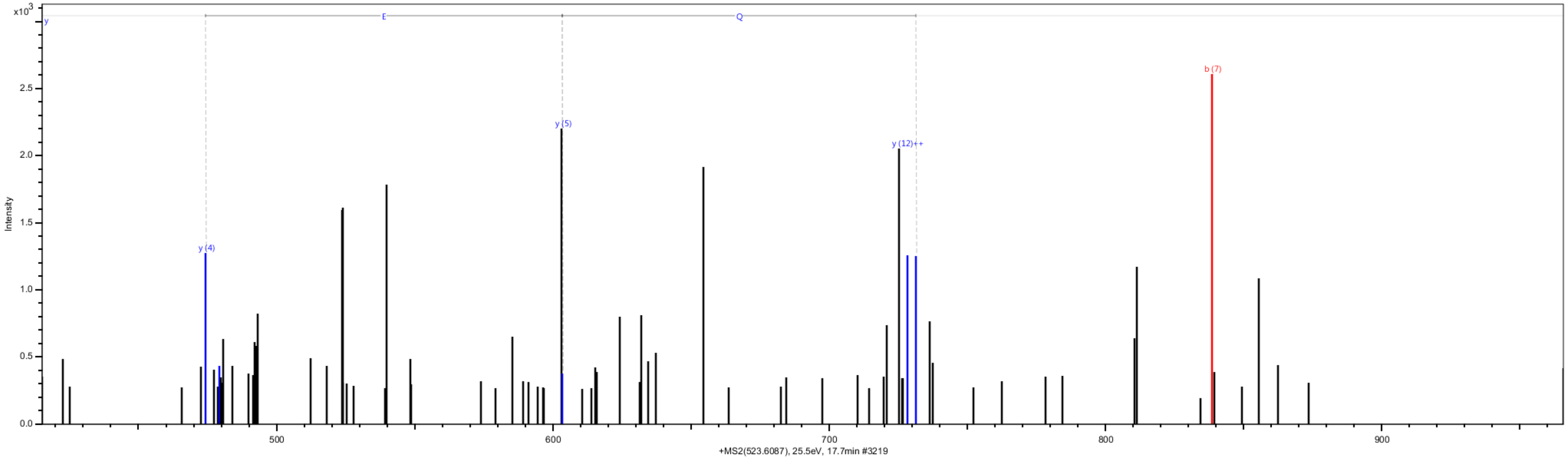

CQ047_HUMAN

Accession: CQ047_HUMAN
Database: SwissProt
MW [kDa] / pI: 63.1 / 9.3
Score: 16.1
Seq. Coverage [%]: 1.6
No. of Peptides: 1

Peptide Match

Sequence: M.VKTNKPGAK.V
Score: 16.1
Modifications:

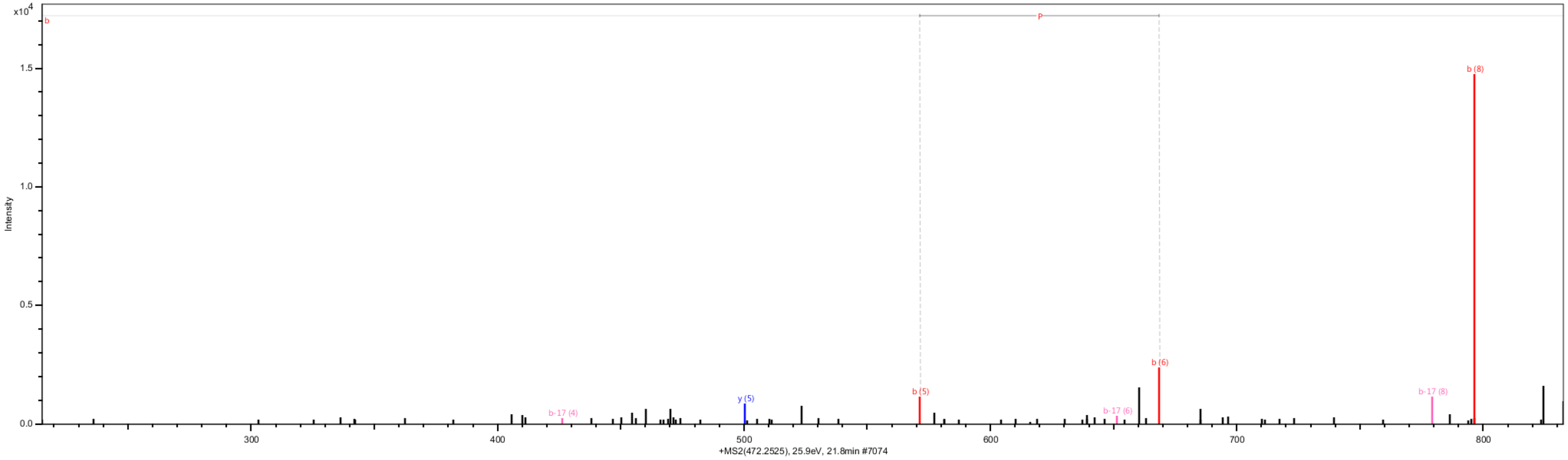

DPYL4_HUMAN

**Accession:**	DPYL4_HUMAN	**Score:**	16.1
**Database:**	SwissProt	**Seq. Coverage [%]:**	1.9
**MW [kDa] / pI:**	61.8 / 6.6	**No. of Peptides:**	1

Peptide Match

**Sequence:**	K.GAADAIAQAKR.R	**Score:**	16.1
**Modifications:**			

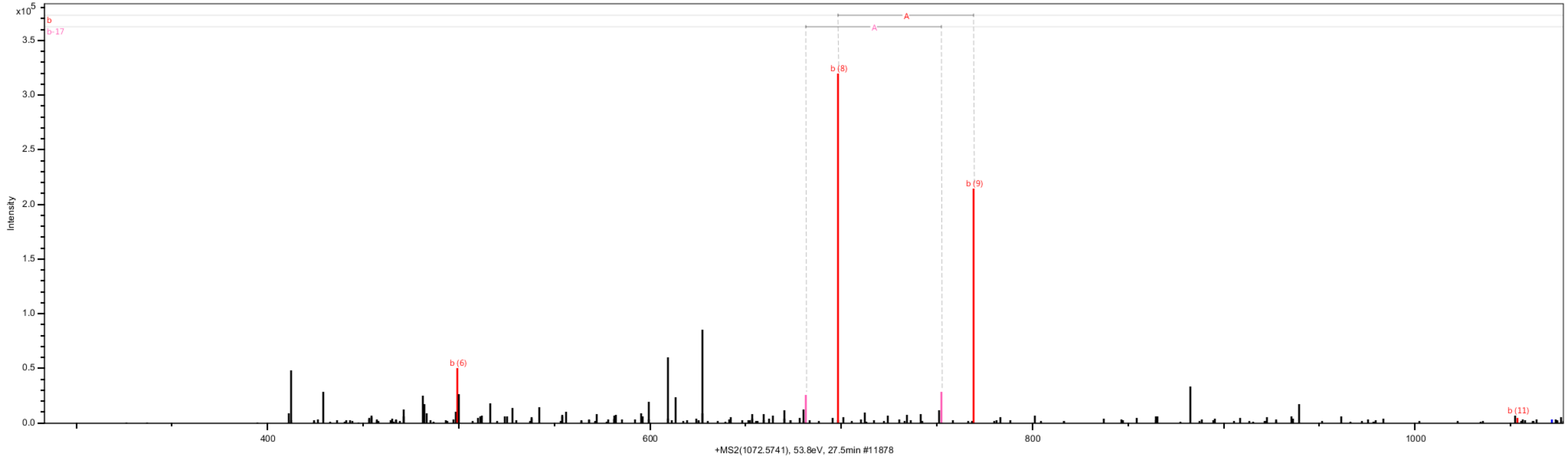

PLXA4_HUMAN

Accession: PLXA4_HUMAN
Database: SwissProt
MW [kDa] / pI: 212.3 / 6.4
Score: 16.1
Seq. Coverage [%]: 0.8
No. of Peptides: 1

Peptide Match

Sequence: R.SRTPMITPDLESGVK.M
Score: 16.1
Modifications: Oxidation: 5

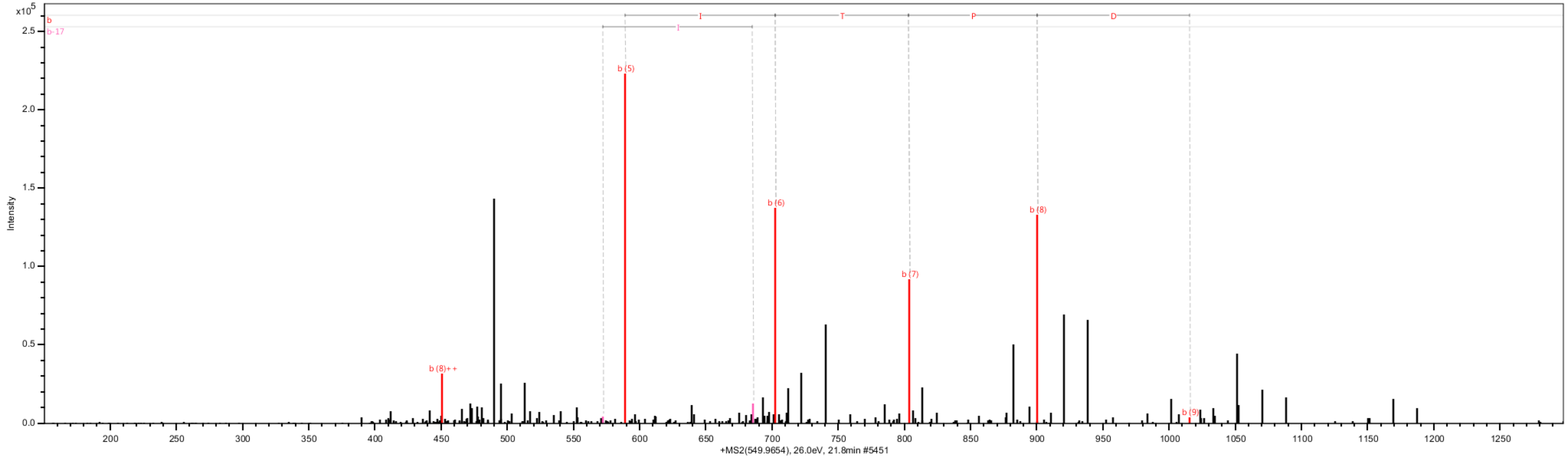

NEB1_HUMAN

Accession: NEB1_HUMAN
Database: SwissProt
MW [kDa] / pI: 123.3 / 5.0
Score: 16.1
Seq. Coverage [%]: 0.9
No. of Peptides: 1

Peptide Match

Sequence: K.TKLQAAENEK.V
Score: 16.1
Modifications:

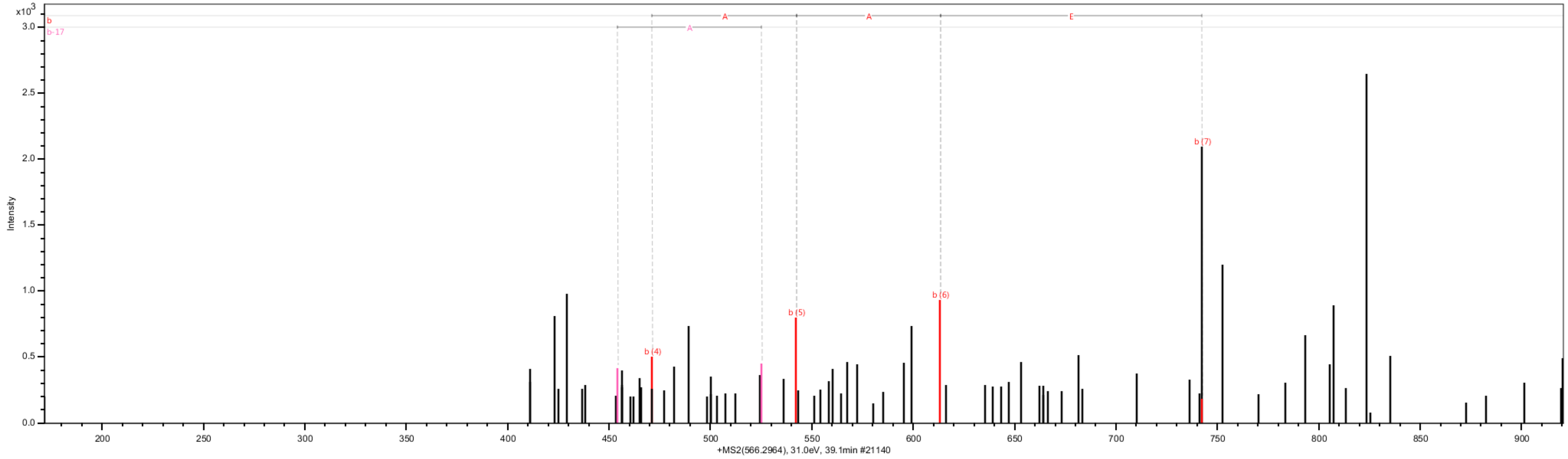

CD1D_HUMAN

Accession: CD1D_HUMAN
Database: SwissProt
MW [kDa] / pI: 37.7 / 8.4
Score: 16.1
Seq. Coverage [%]: 4.8
No. of Peptides: 1

Peptide Match

Sequence: R.ATLDVVAGEAAGLSCR.V
Score: 16.1
Modifications: Carbamidomethyl: 15

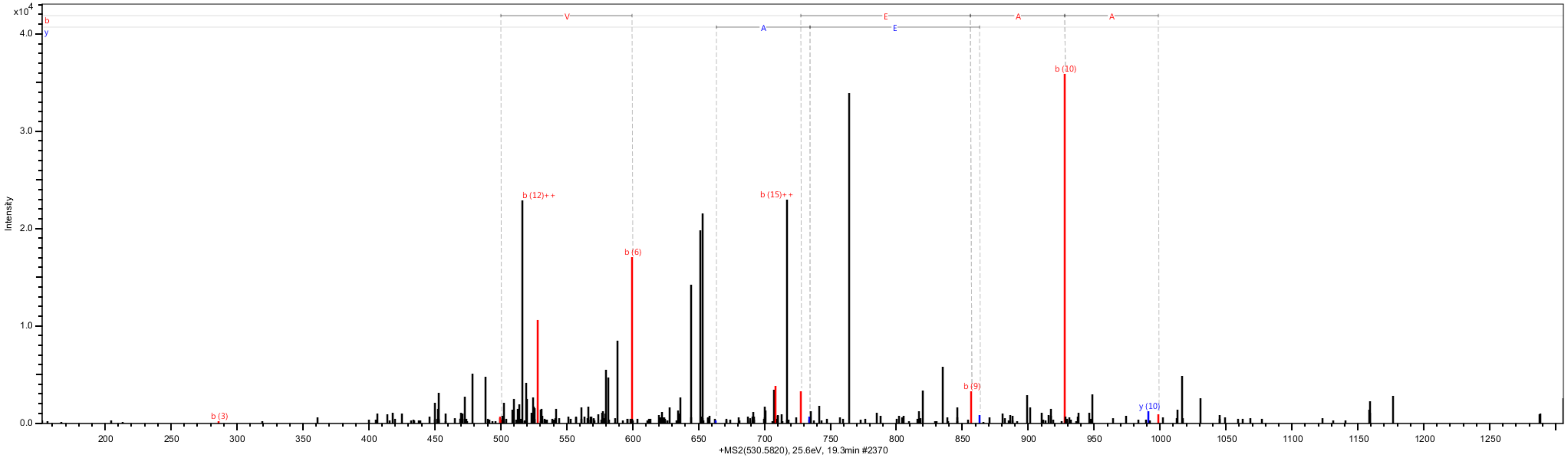

TRIM9_HUMAN

Accession: TRIM9_HUMAN
Database: SwissProt
MW [kDa] / pI: 79.1 / 6.4

Score: 16.1
Seq. Coverage [%]: 1.7
No. of Peptides: 1

Peptide Match

Sequence: R.SWFMHNNSHTNR.T
Modifications: Oxidation: 4
Score: 16.1

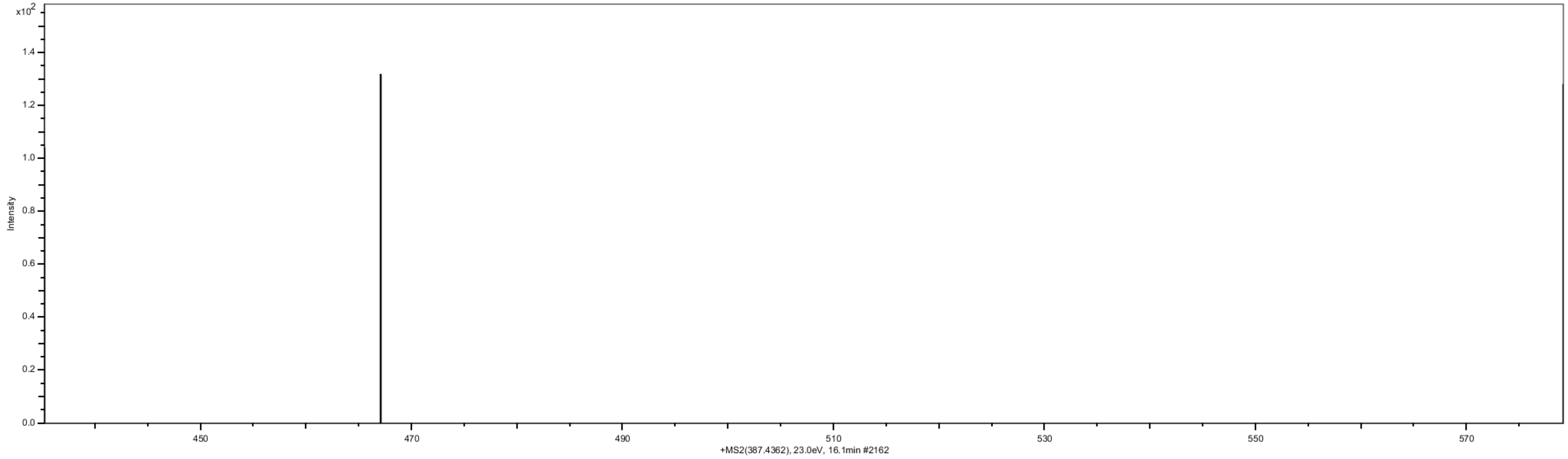

FA49B_HUMAN

Accession: FA49B_HUMAN
Database: SwissProt
MW [kDa] / pI: 36.7 / 5.8
Score: 16.1
Seq. Coverage [%]: 2.2
No. of Peptides: 1

Peptide Match

Sequence: K.TSKIDMK.G
Score: 16.1
Modifications: Oxidation: 6

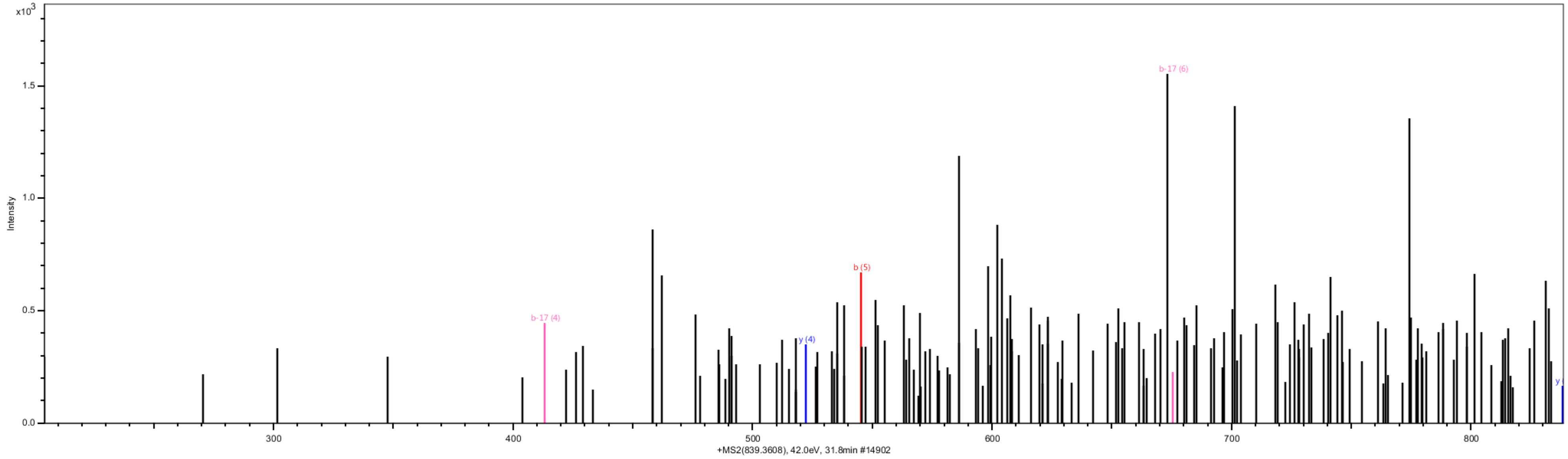

PRKDC_HUMAN

**Accession:**	PRKDC_HUMAN	**Score:**	16.1
**Database:**	SwissProt	**Seq. Coverage [%]:**	0.3
**MW [kDa] / pI:**	468.8 / 6.7	**No. of Peptides:**	1

Peptide Match

**Sequence:**	K.HDVGAYMLMYK.G	**Score:**	16.1
**Modifications:**	Oxidation: 7		

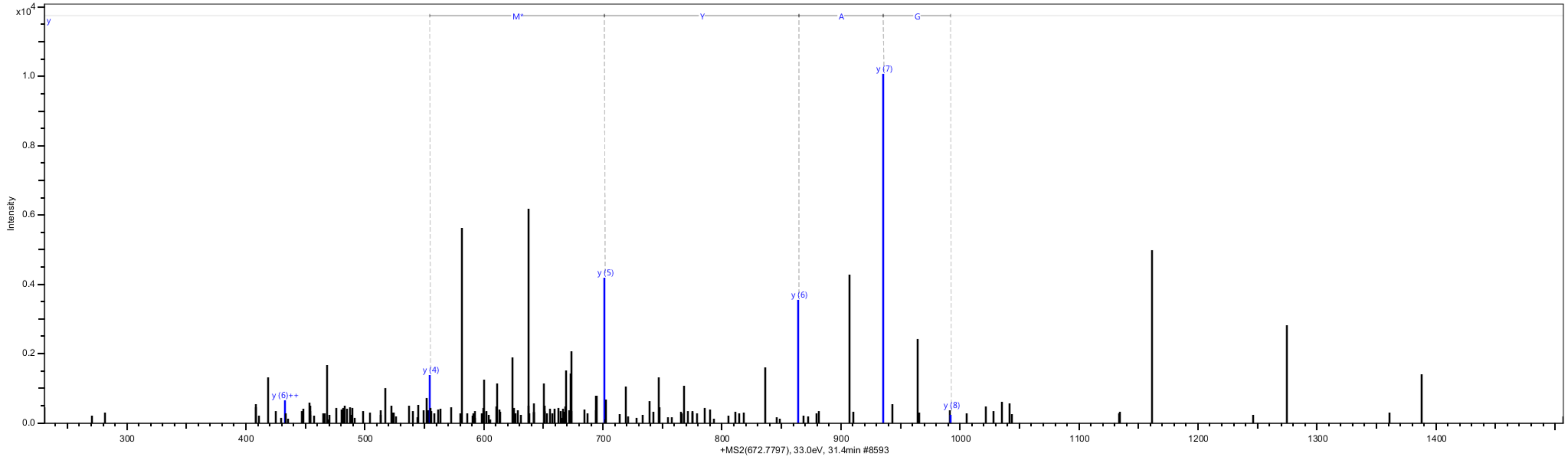

DYH1_HUMAN

Accession: DYH1_HUMAN
Database: SwissProt
MW [kDa] / pI: 487.2 / 5.6
Score: 16.1
Seq. Coverage [%]: 0.3
No. of Peptides: 1

Peptide Match

Sequence: K.NQLIISNAKMR.Q
Score: 16
Modifications:

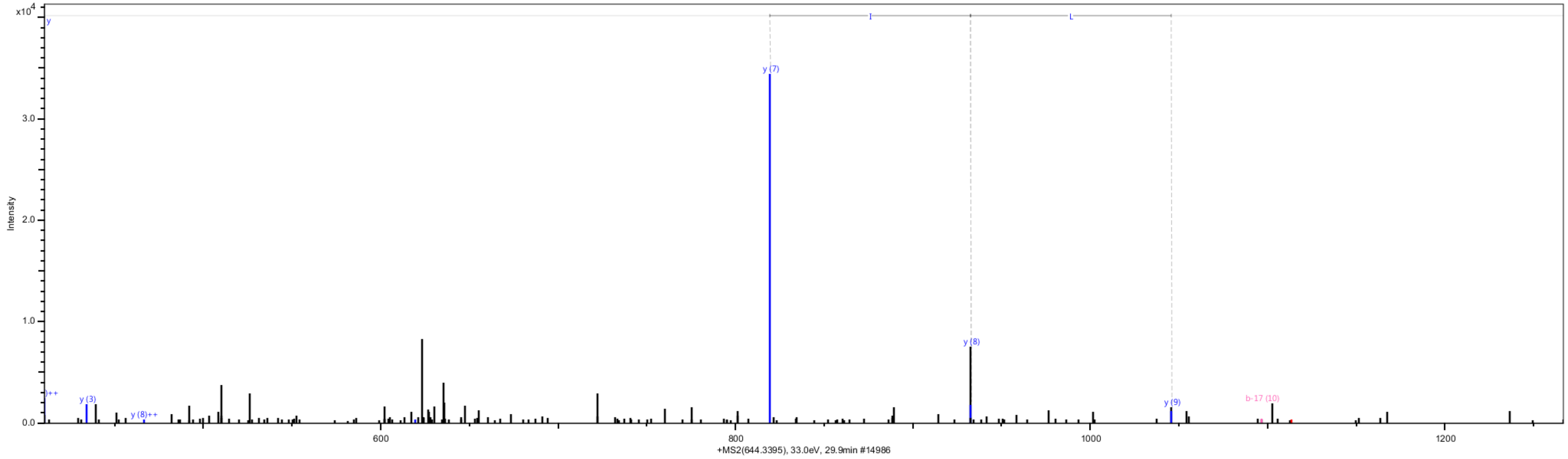

GAB1_HUMAN

**Accession:**	GAB1_HUMAN	**Score:**	16.1
**Database:**	SwissProt	**Seq. Coverage [%]:**	2.0
**MW [kDa] / pI:**	76.6 / 5.6	**No. of Peptides:**	1

Peptide Match

**Sequence:**	-.MSGGEVVCSGWLRK.S	**Score:**	16.1
**Modifications:**	Carbamidomethyl: 8		

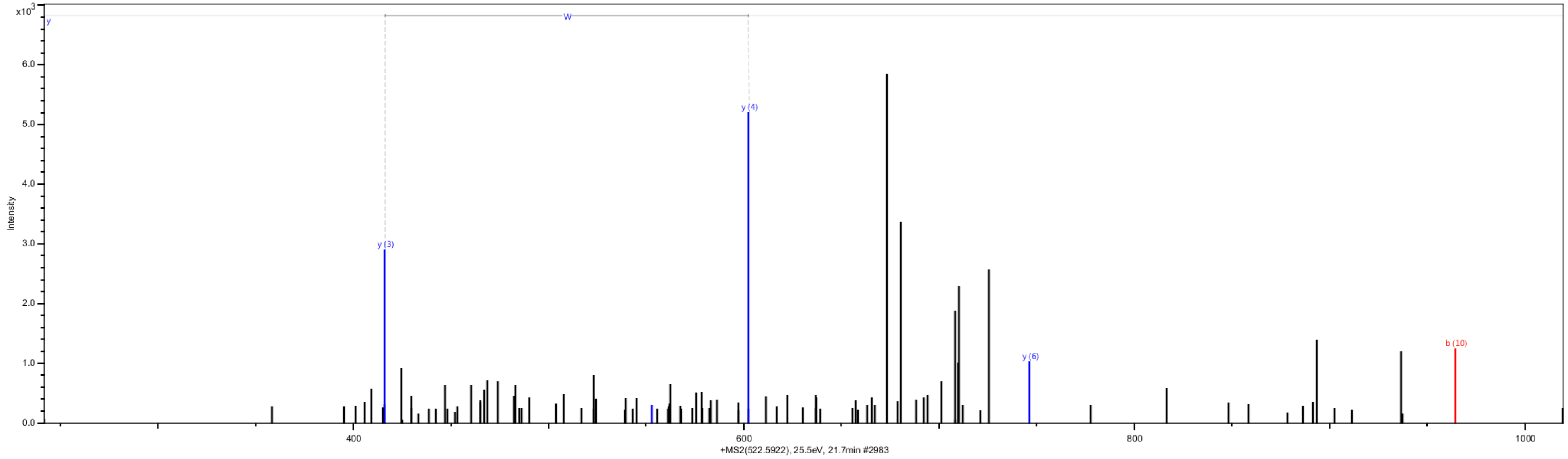

ANR35_HUMAN

Accession: ANR35_HUMAN
Database: SwissProt
MW [kDa] / pI: 109.9 / 5.8
Score: 16.1
Seq. Coverage [%]: 0.9
No. of Peptides: 1

Peptide Match

Sequence: R.LREAVAEER.R
Score: 16.1
Modifications:

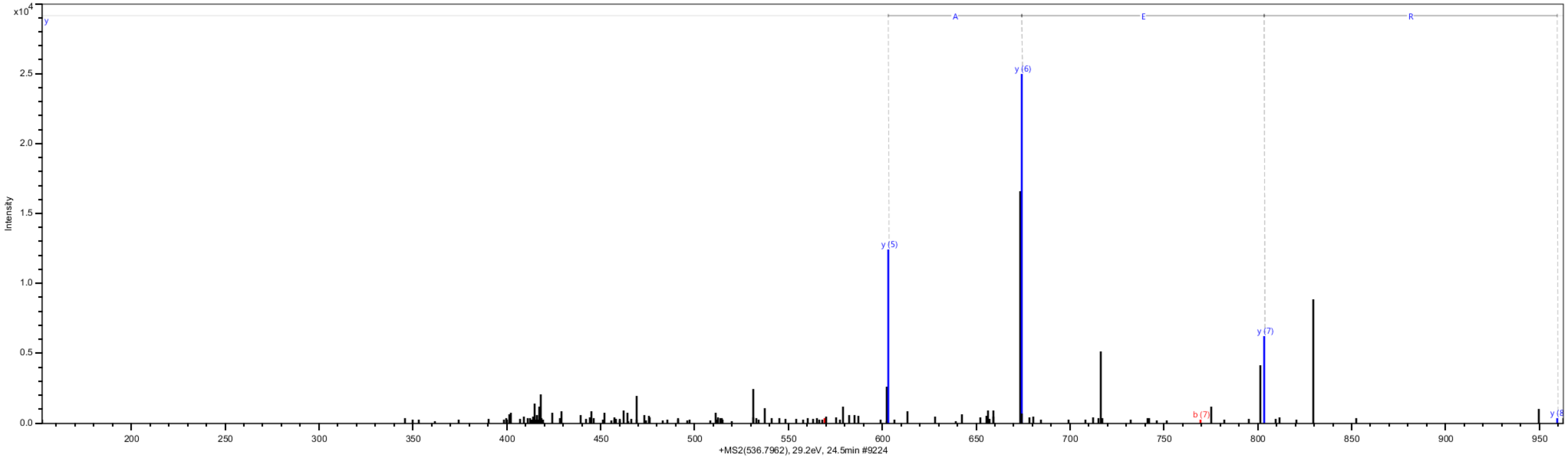

NRDE2_HUMAN

Accession: NRDE2_HUMAN
Database: SwissProt
MW [kDa] / pI: 132.6 / 7.7
Score: 16.0
Seq. Coverage [%]: 0.8
No. of Peptides: 1

Peptide Match

Sequence: R.WQIWLAAER.S
Score: 16
Modifications:

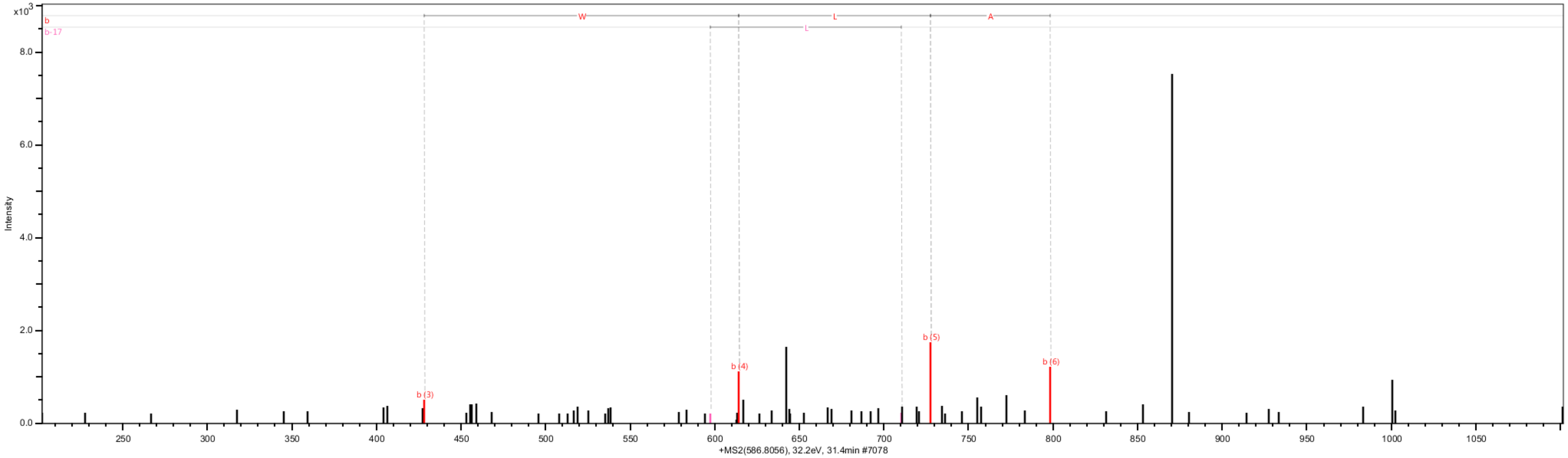

NAR3_HUMAN

Accession: NAR3_HUMAN
Database: SwissProt
MW [kDa] / pI: 43.9 / 5.7
Score: 16.0
Seq. Coverage [%]: 3.6
No. of Peptides: 1

Peptide Match

Sequence: K.ASHQQLDTVWENAK.A
Score: 16
Modifications:

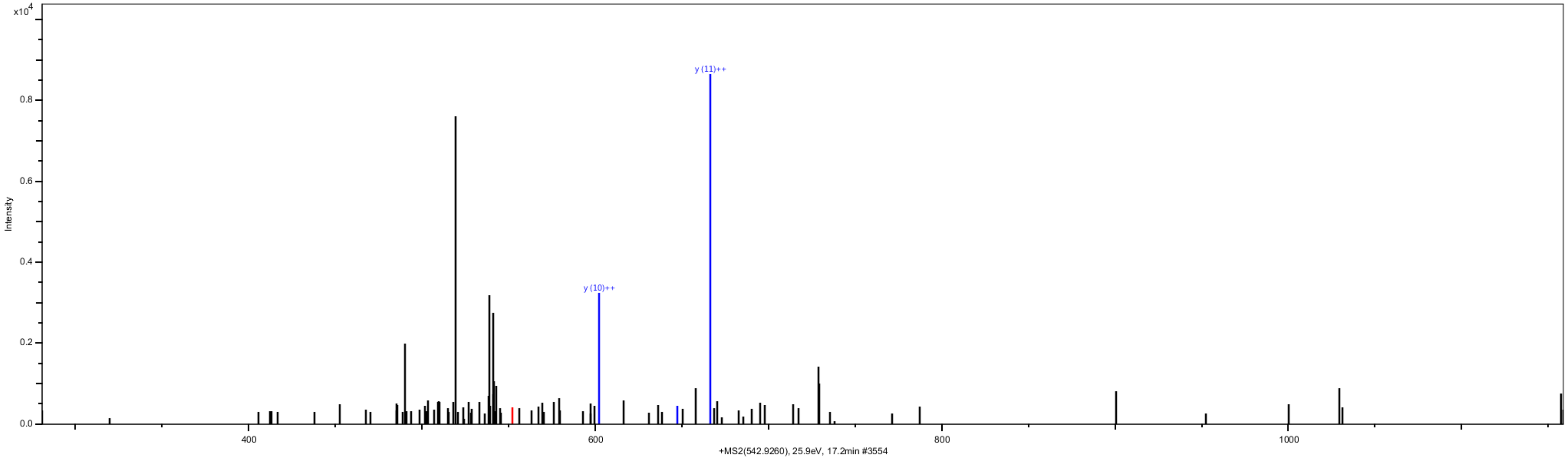

AP3M1_HUMAN

Accession: AP3M1_HUMAN
Database: SwissProt
MW [kDa] / pI: 46.9 / 6.5
Score: 16.0
Seq. Coverage [%]: 2.9
No. of Peptides: 1

Peptide Match

Sequence: R.LDMYGEKYKPFK.G
Score: 16
Modifications:

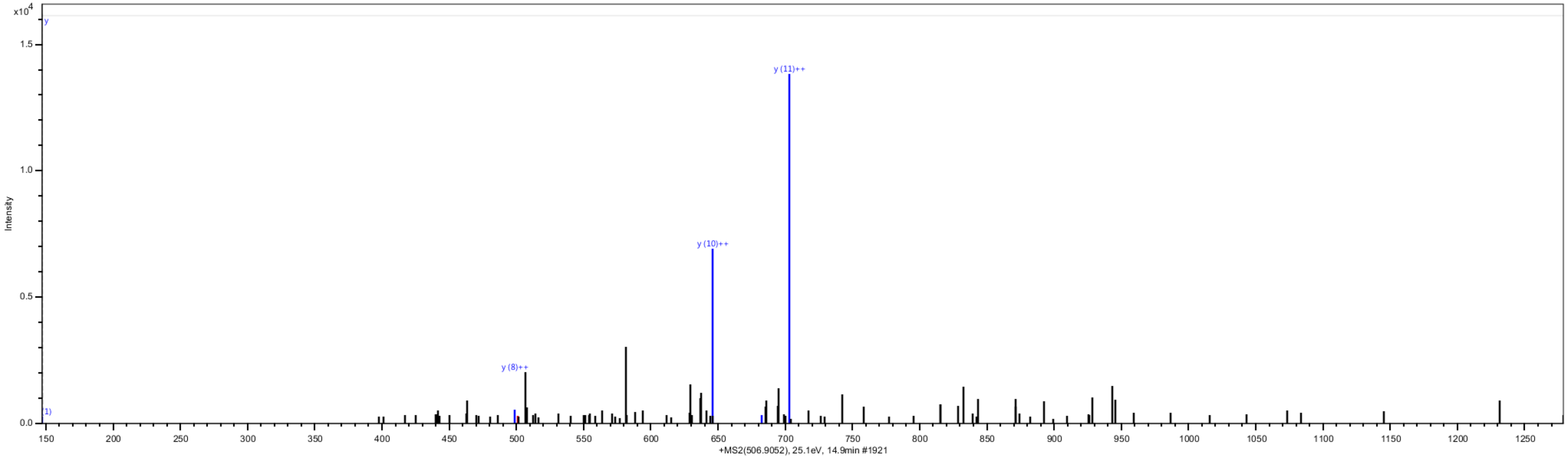

NCBP3_HUMAN

Accession: NCBP3_HUMAN
Database: SwissProt
MW [kDa] / pI: 70.5 / 5.6
Score: 16.0
Seq. Coverage [%]: 1.1
No. of Peptides: 1

Peptide Match

Sequence: R.RSQYYMK.Y
Score: 16
Modifications:

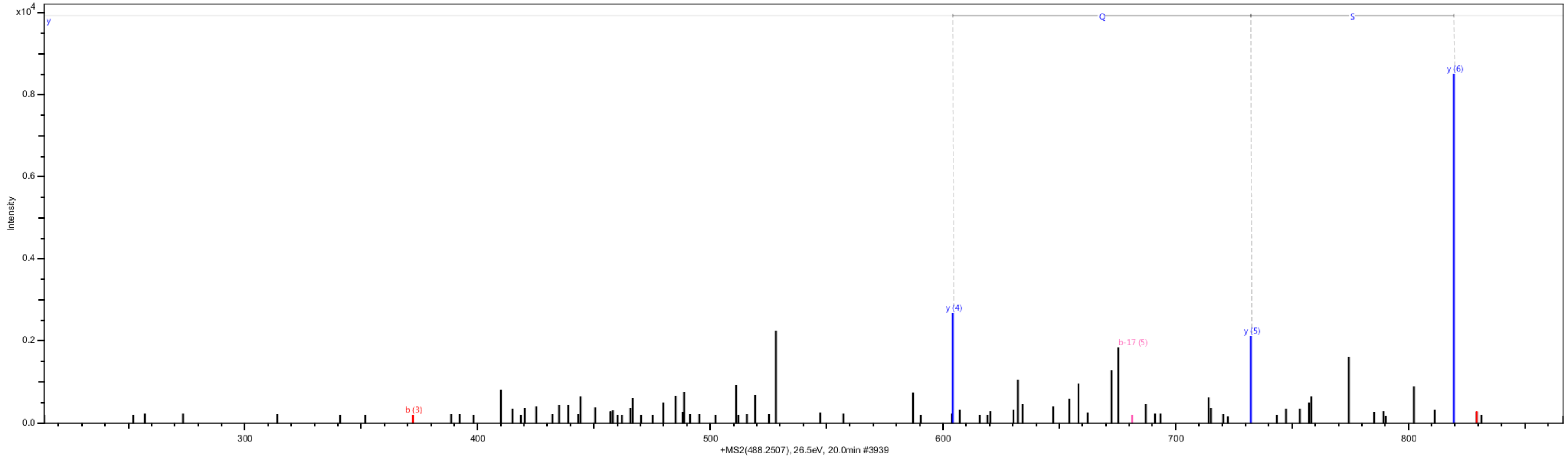

ADM2_HUMAN

Accession: ADM2_HUMAN
Database: SwissProt
MW [kDa] / pI: 15.9 / 11.8
Score: 16.0
Seq. Coverage [%]: 10.8
No. of Peptides: 1

Peptide Match

Sequence: R.VGCVLGTCQVQNLSHR.L
Score: 16
Modifications: Carbamidomethyl: 3, 8

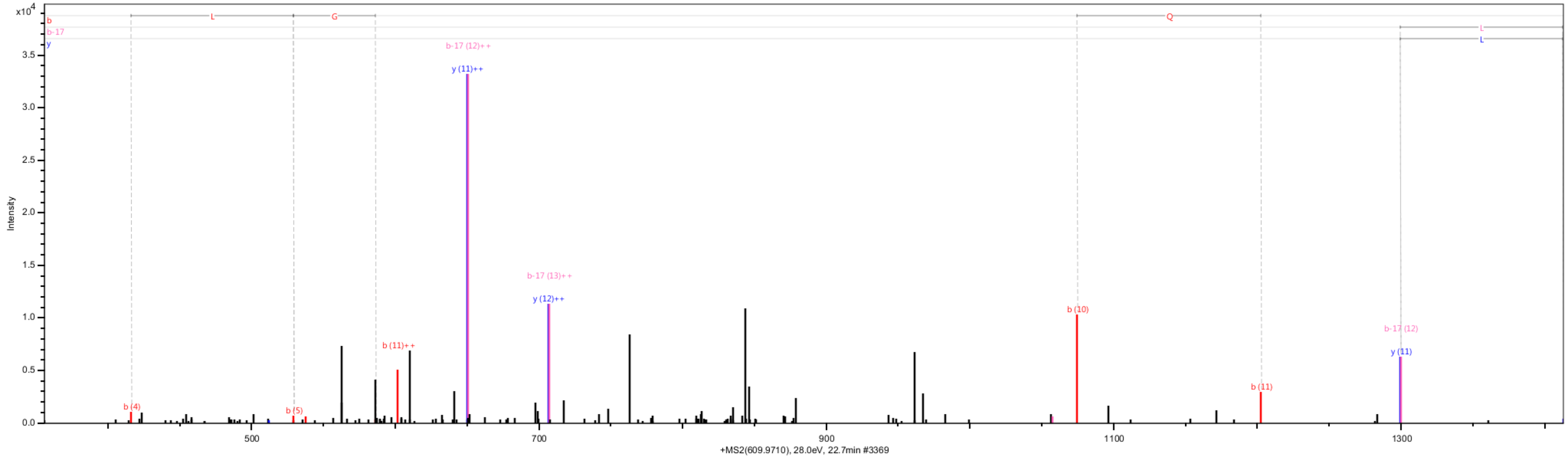

HPPD_HUMAN

Accession: HPPD_HUMAN
Database: SwissProt
MW [kDa] / pI: 44.9 / 6.5
Score: 16.0
Seq. Coverage [%]: 2.5
No. of Peptides: 1

Peptide Match

Sequence: K.MPINEPAPGK.K
Score: 16
Modifications: Oxidation: 1

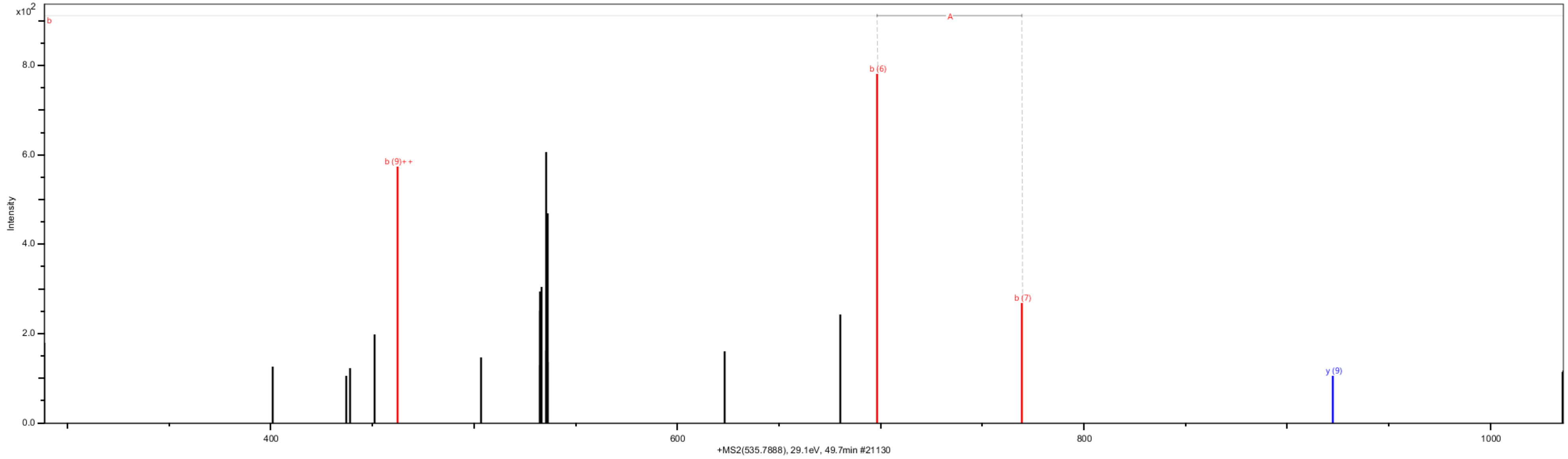

MKS1_HUMAN

Accession: MKS1_HUMAN
Database: SwissProt
MW [kDa] / pI: 64.5 / 6.0
Score: 16.0
Seq. Coverage [%]: 1.8
No. of Peptides: 1

Peptide Match

Sequence: R.GMEGGILKSR.I
Score: 16
Modifications:

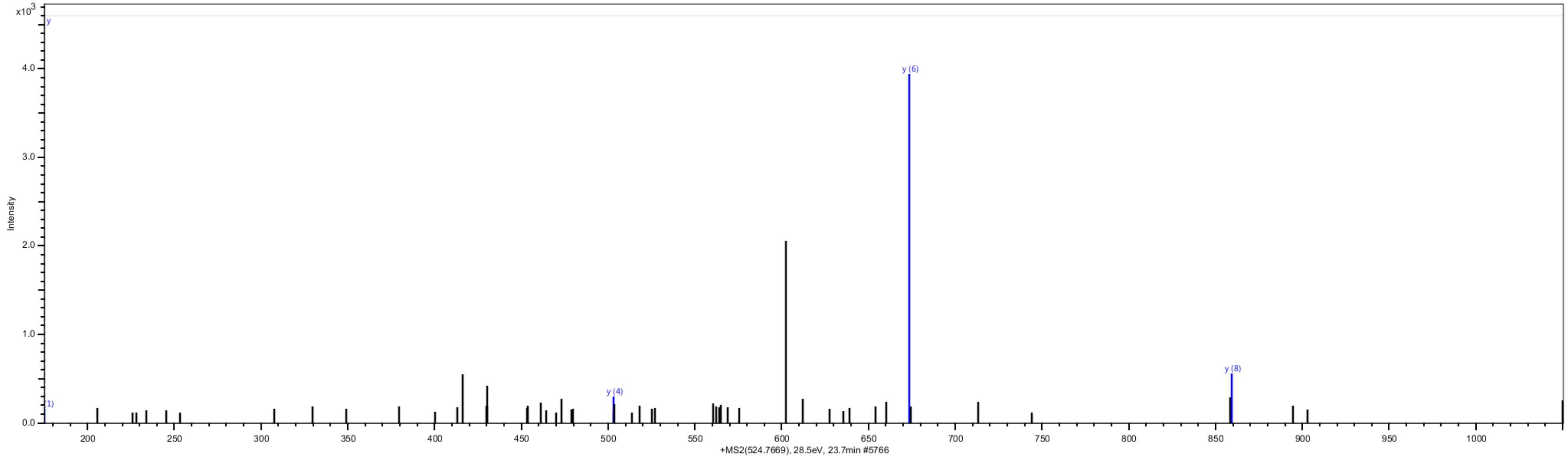

PTBP2_HUMAN

Accession: PTBP2_HUMAN
Database: SwissProt
MW [kDa] / pI: 57.5 / 8.7
Score: 16.0
Seq. Coverage [%]: 1.3
No. of Peptides: 1

Peptide Match

Sequence: K.MYGKIIR.V
Score: 16
Modifications:

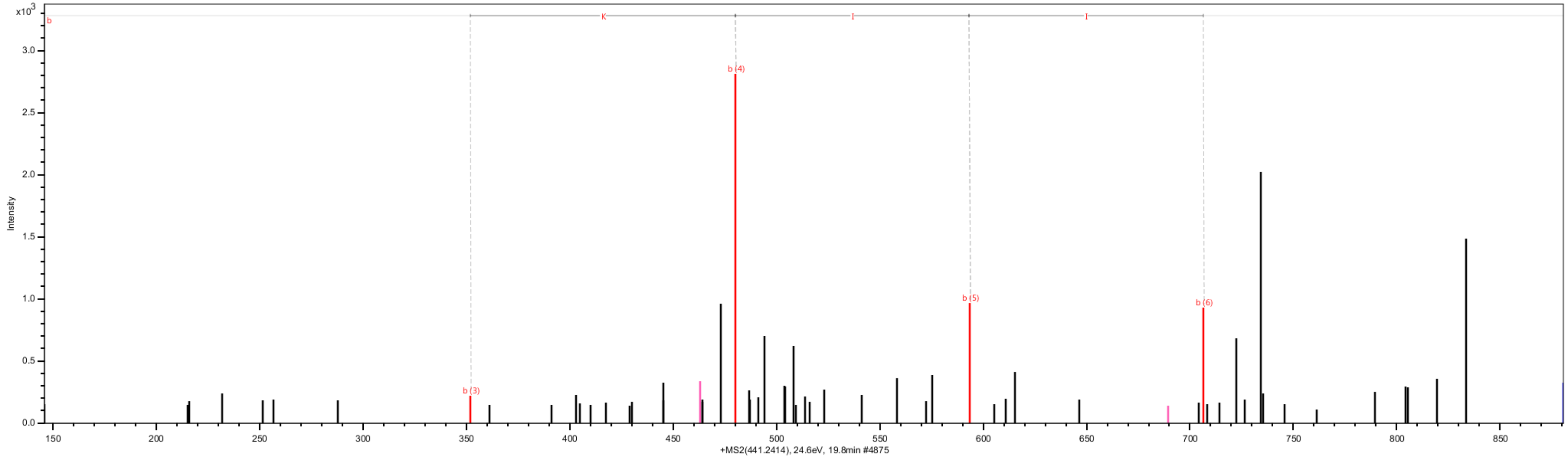

ABCG5_HUMAN

Accession: ABCG5_HUMAN
Database: SwissProt
MW [kDa] / pI: 72.5 / 9.2
Score: 16.0
Seq. Coverage [%]: 1.7
No. of Peptides: 1

Peptide Match

Sequence: K.HLKTLPMVPFK.T
Score: 16
Modifications: Oxidation: 7

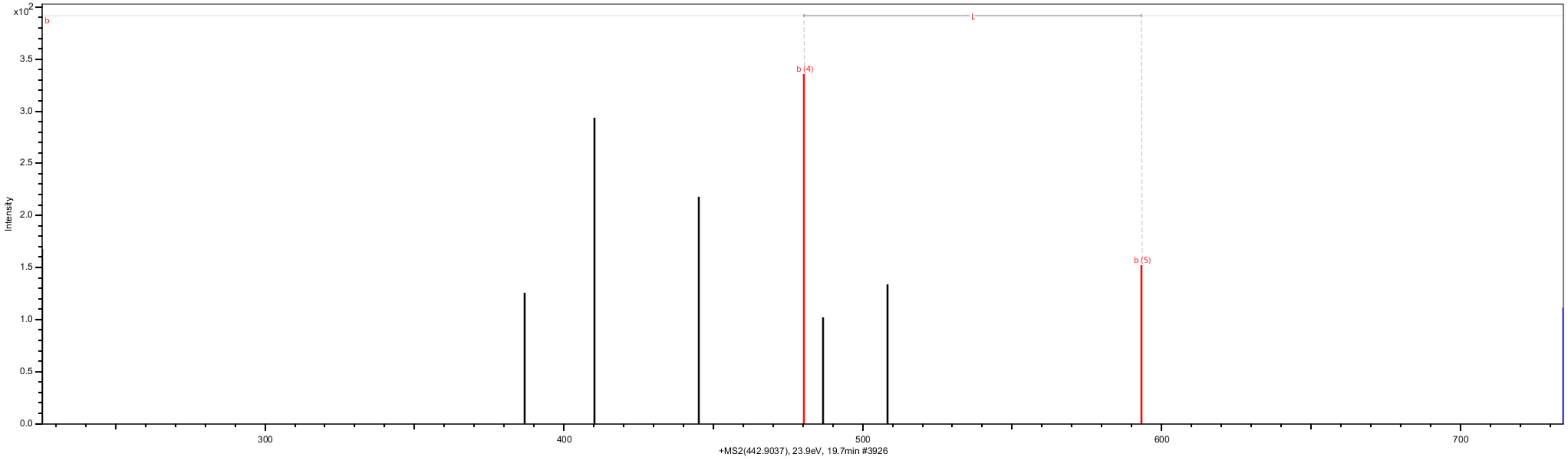

GTPB1_HUMAN

Accession: GTPB1_HUMAN
Database: SwissProt
MW [kDa] / pI: 72.4 / 8.6
Score: 16.0
Seq. Coverage [%]: 2.1
No. of Peptides: 1

Peptide Match

Sequence: K.SMAEQIEADVILLR.E
Score: 16
Modifications: Oxidation: 2

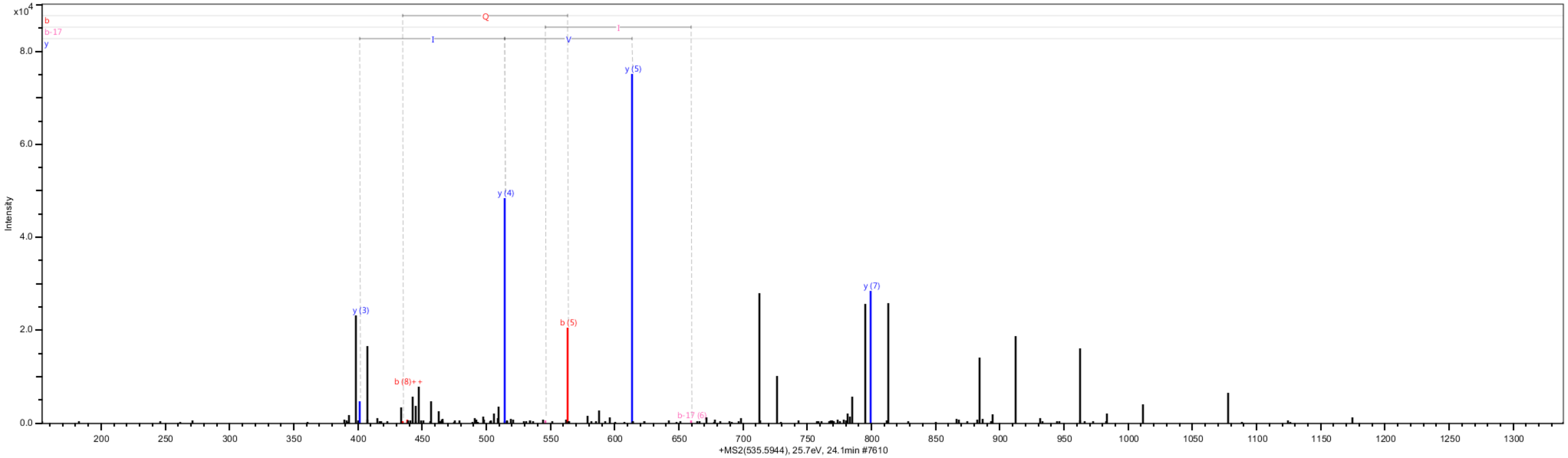

FGD5_HUMAN

Accession: FGD5_HUMAN
Database: SwissProt
MW [kDa] / pI: 159.8 / 4.9
Score: 16.0
Seq. Coverage [%]: 0.5
No. of Peptides: 1

Peptide Match

Sequence: R.AFTLYPR.S
Score: 16
Modifications:

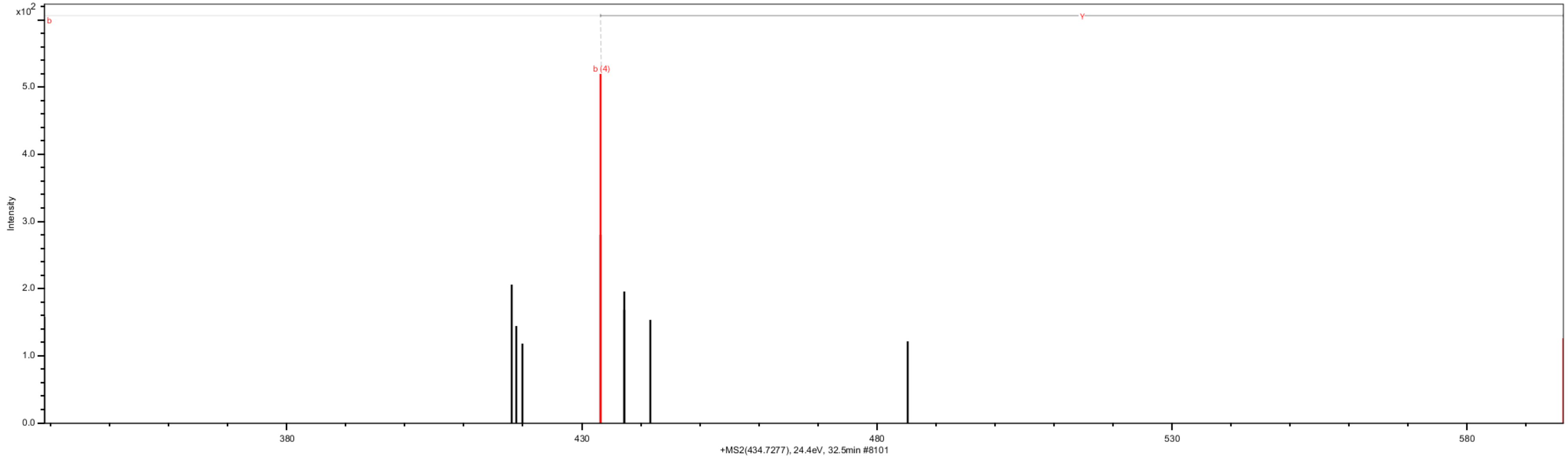

K1C12_HUMAN

Accession: K1C12_HUMAN
Database: SwissProt
MW [kDa] / pI: 53.5 / 4.7
Score: 16.5
Seq. Coverage [%]: 2.0
No. of Peptides: 1

Peptide Match

Sequence: K.SQAQSTDSSK.D
Score: 16.4
Modifications:

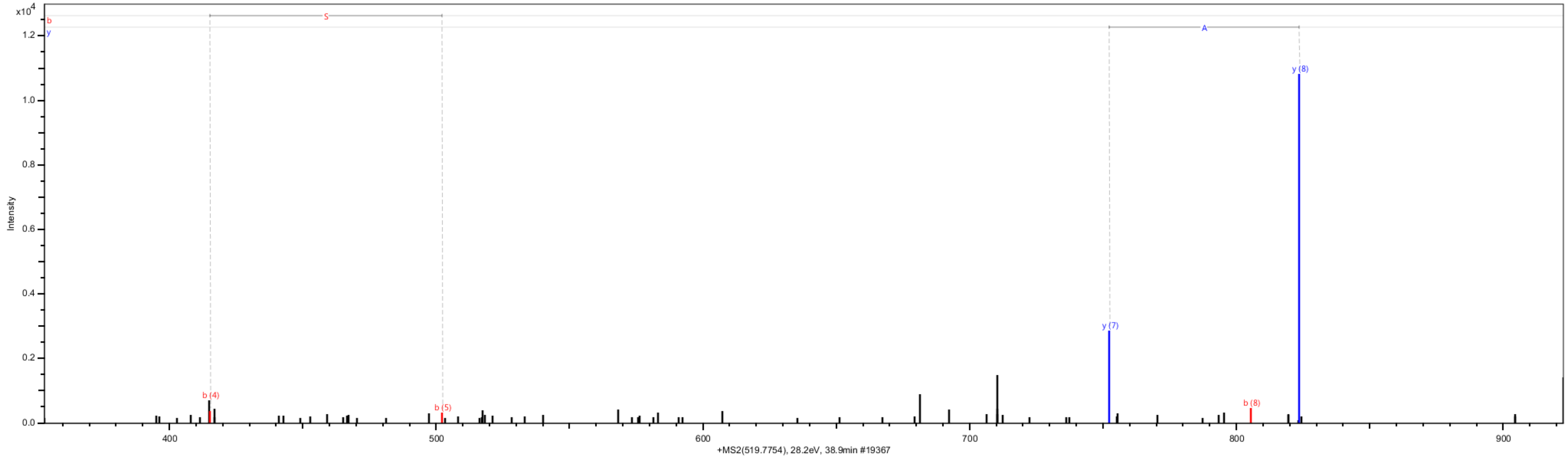

RETST_HUMAN

Accession: RETST_HUMAN **Score:** 16.5
Database: SwissProt **Seq. Coverage [%]:** 2.0
MW [kDa] / pI: 66.8 / 8.5 **No. of Peptides:** 1

Peptide Match

Sequence: R.NLYSDLKNLDSR.I **Score:** 16.5
Modifications:

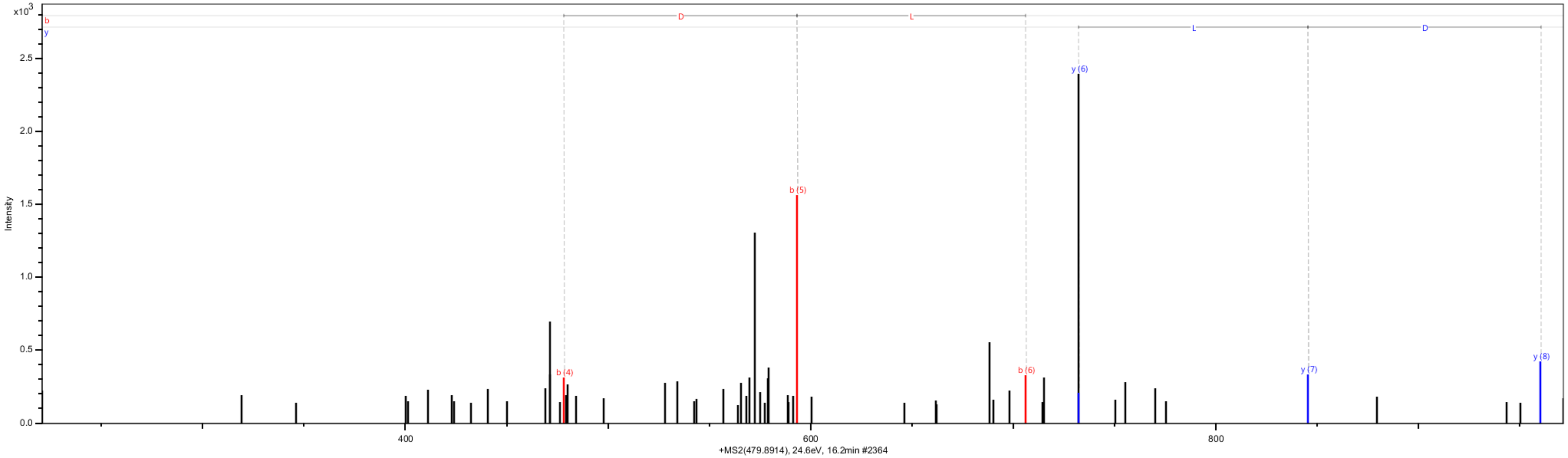

KS6C1_HUMAN

Accession: KS6C1_HUMAN
Database: SwissProt
MW [kDa] / pI: 118.6 / 4.8
Score: 16.5
Seq. Coverage [%]: 0.8
No. of Peptides: 1

Peptide Match

Sequence: R.EAAAMGPTK.F
Score: 16.4
Modifications:

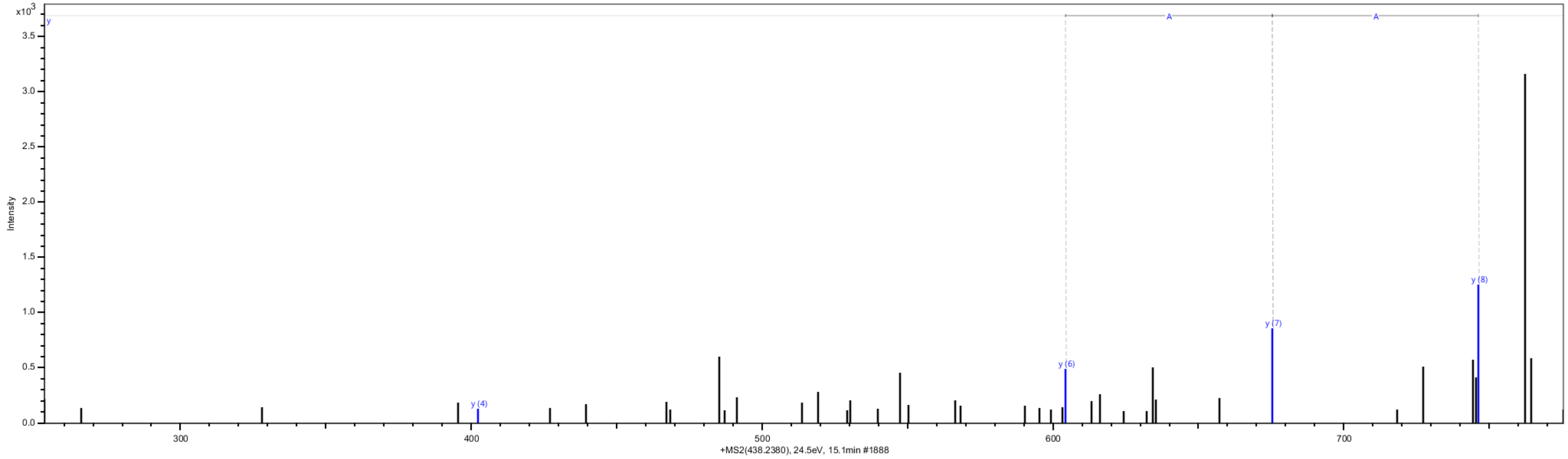

SCLT1_HUMAN

Accession: SCLT1_HUMAN
Database: SwissProt
MW [kDa] / pI: 80.9 / 5.8
Score: 16.5
Seq. Coverage [%]: 1.2
No. of Peptides: 1

Peptide Match

Sequence: K.LILEHQEK.A
Score: 16.4
Modifications:

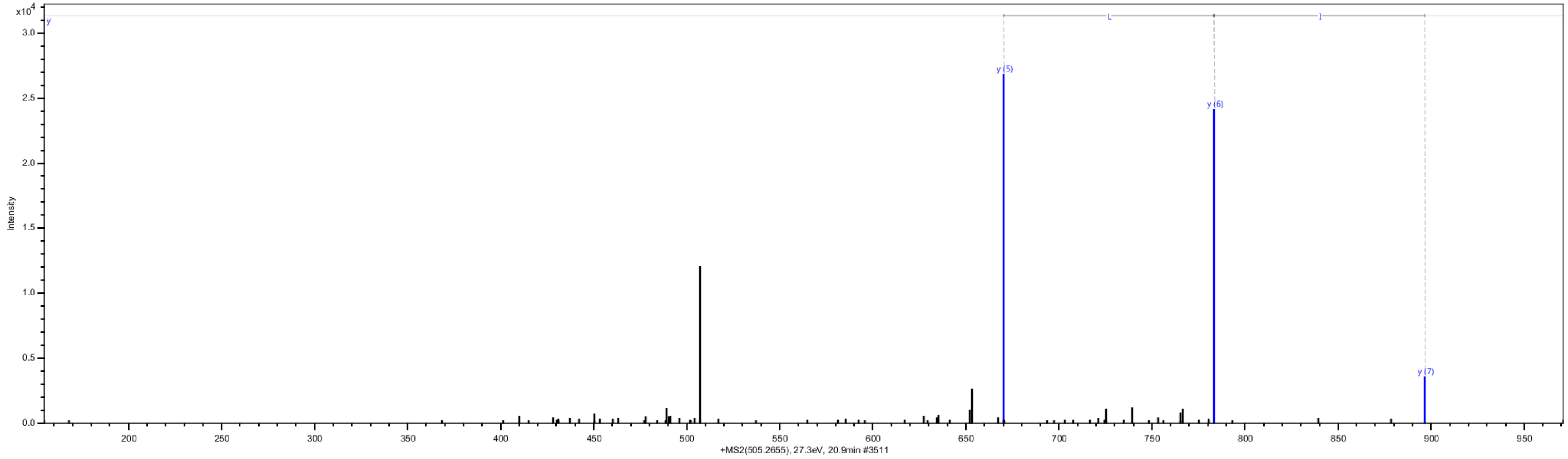

Z705D_HUMAN

Accession: Z705D_HUMAN
Database: SwissProt
MW [kDa] / pI: 34.7 / 9.1
Score: 16.4
Seq. Coverage [%]: 6.0
No. of Peptides: 1

Peptide Match

Sequence: R.HKMTHTGERPYACHLCGK.A
Score: 16.4
Modifications: Carbamidomethyl: 13, 16; Oxidation: 3

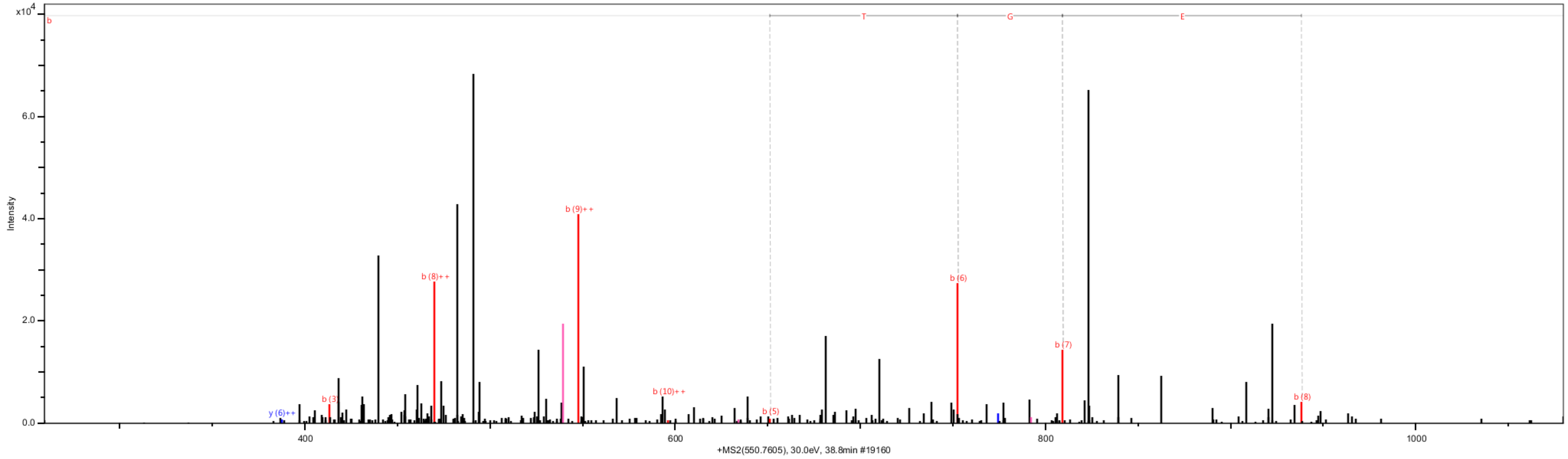

ARMC2_HUMAN

Accession: ARMC2_HUMAN
Database: SwissProt
MW [kDa] / pI: 96.8 / 8.5
Score: 16.4
Seq. Coverage [%]: 1.7
No. of Peptides: 1

Peptide Match

Sequence: K.LLVSNNMDGILEAVR.V
Score: 16.4
Modifications: Oxidation: 7

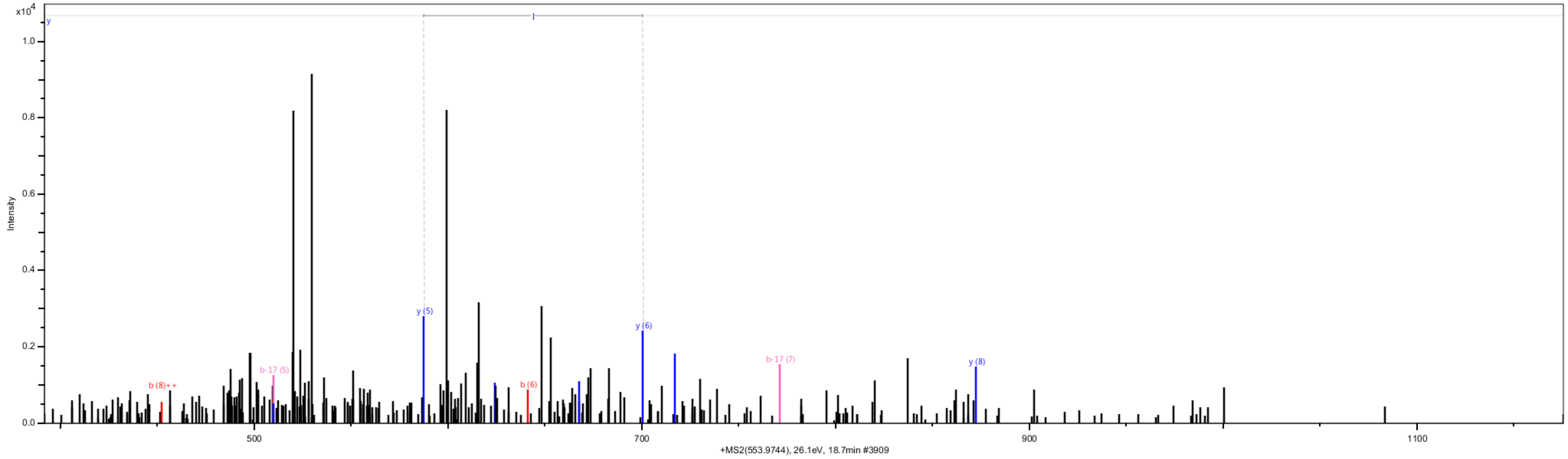

STRN4_HUMAN

Accession: STRN4_HUMAN
Database: SwissProt
MW [kDa] / pI: 80.5 / 5.2
Score: 16.4
Seq. Coverage [%]: 1.9
No. of Peptides: 1

Peptide Match

Sequence: K.KNAALDVEPIHAFR.A
Score: 16.4
Modifications:

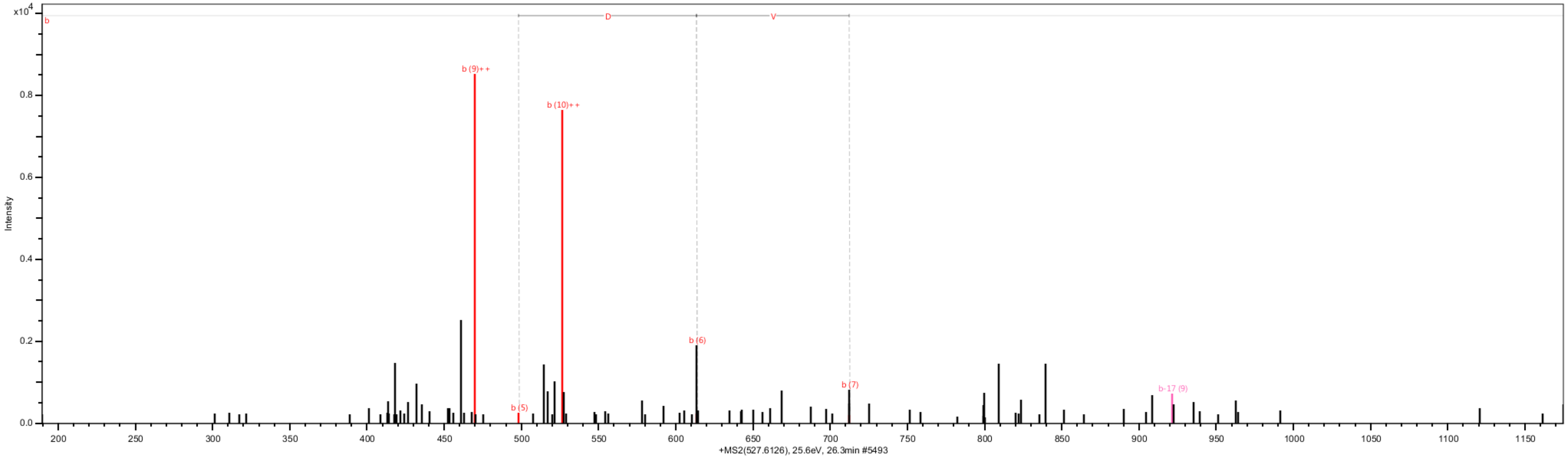

ABCB6_HUMAN

Accession: ABCB6_HUMAN
Database: SwissProt
MW [kDa] / pI: 93.8 / 8.7
Score: 16.4
Seq. Coverage [%]: 0.8
No. of Peptides: 1

Peptide Match

Sequence: K.YQGLEWK.S
Score: 16.4
Modifications:

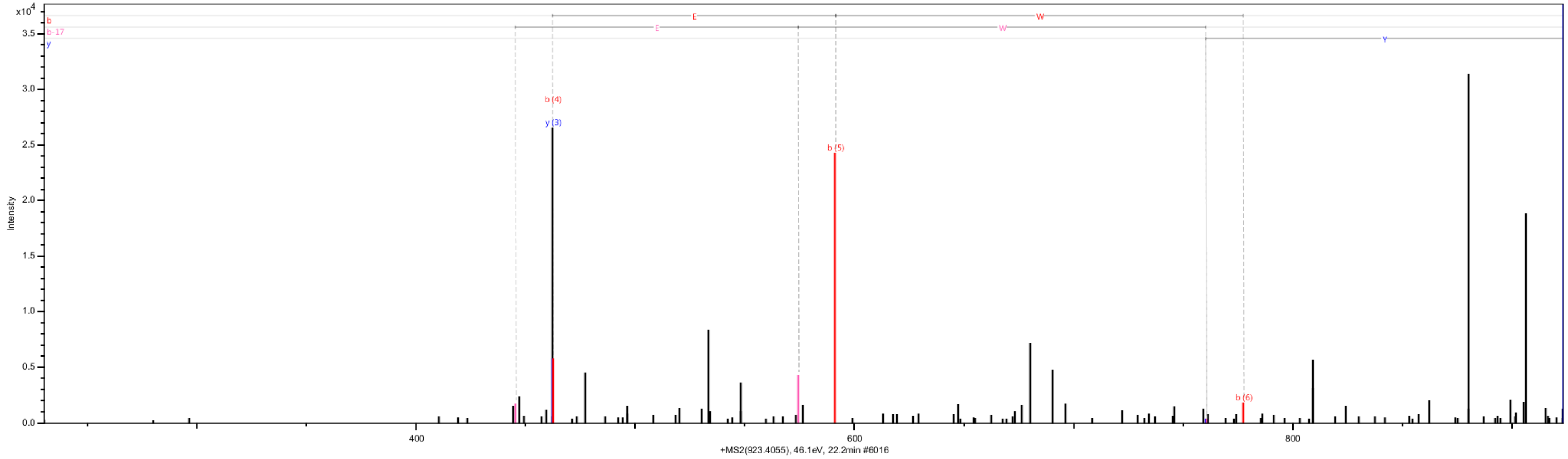

WWTR1_HUMAN

Accession: WWTR1_HUMAN
Database: SwissProt
MW [kDa] / pI: 44.1 / 5.5
Score: 16.4
Seq. Coverage [%]: 1.8
No. of Peptides: 1

Peptide Match

Sequence: R.IQMERER.I
Score: 16.4
Modifications: Oxidation: 3

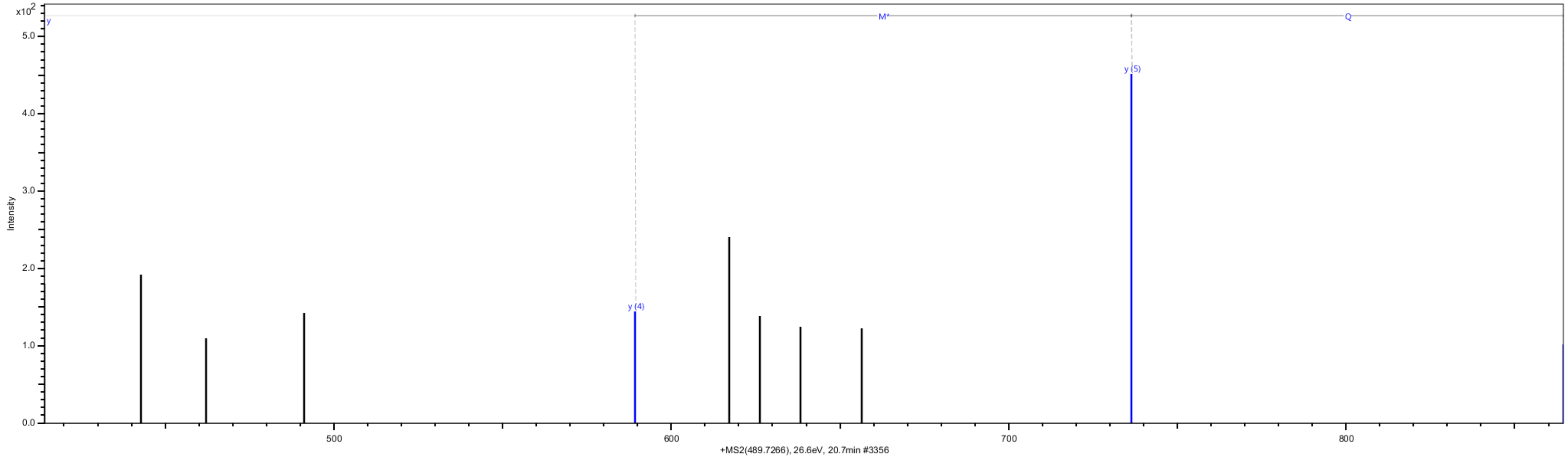

CDN1B_HUMAN

Accession: CDN1B_HUMAN
Database: SwissProt
MW [kDa] / pI: 22.1 / 6.5
Score: 16.4
Seq. Coverage [%]: 7.1
No. of Peptides: 1

Peptide Match

Sequence: R.VSNGSPSLERMDAR.Q
Score: 16.4
Modifications:

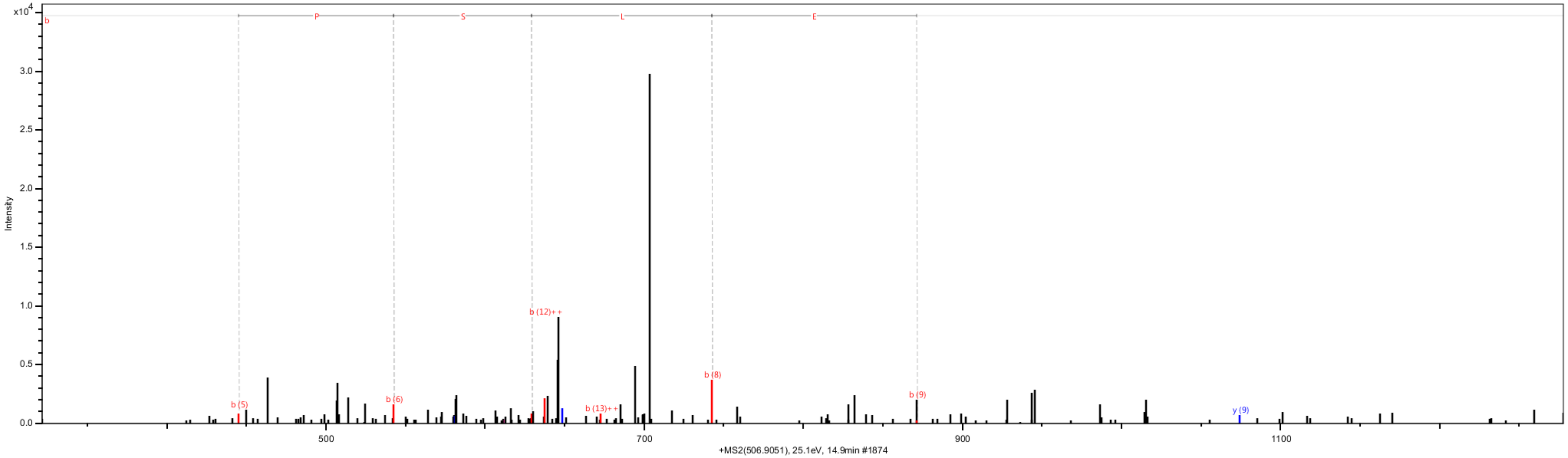

MSH2_HUMAN

Accession: MSH2_HUMAN
Database: SwissProt
MW [kDa] / pI: 104.7 / 5.6
Score: 16.4
Seq. Coverage [%]: 1.5
No. of Peptides: 1

Peptide Match

Sequence: K.QMFHIITGPNMGGK.S
Score: 16.4
Modifications: Oxidation: 11

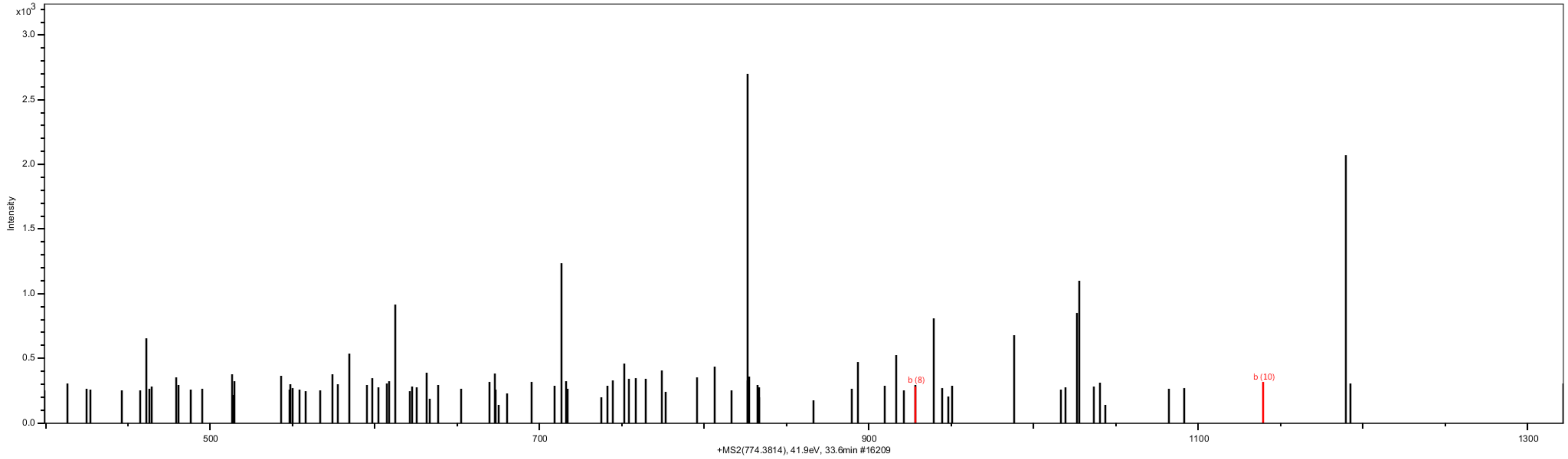

MOB3C_HUMAN

Accession: MOB3C_HUMAN
Database: SwissProt
MW [kDa] / pI: 25.6 / 9.0
Score: 16.4
Seq. Coverage [%]: 5.1
No. of Peptides: 1

Peptide Match

Sequence: R.INLIYGTMAER.C
Score: 16.4
Modifications:

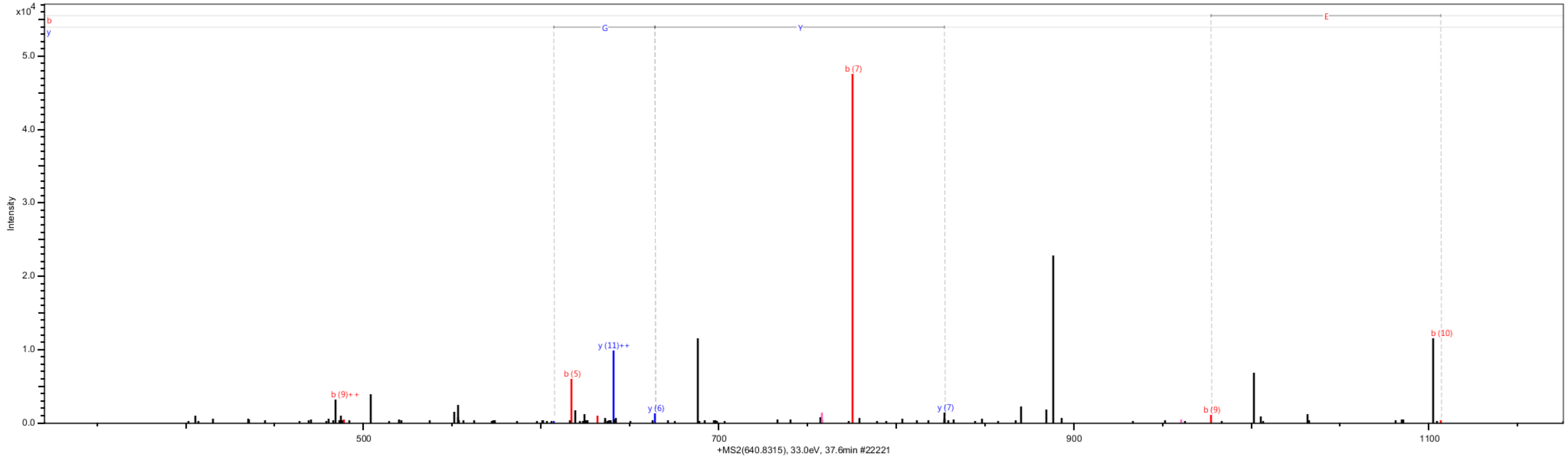

HD_HUMAN

Accession: HD_HUMAN
Database: SwissProt
MW [kDa] / pI: 347.4 / 5.8
Score: 16.4
Seq. Coverage [%]: 0.3
No. of Peptides: 1

Peptide Match

Sequence: R.NIIISLAR.L
Score: 16.4
Modifications:

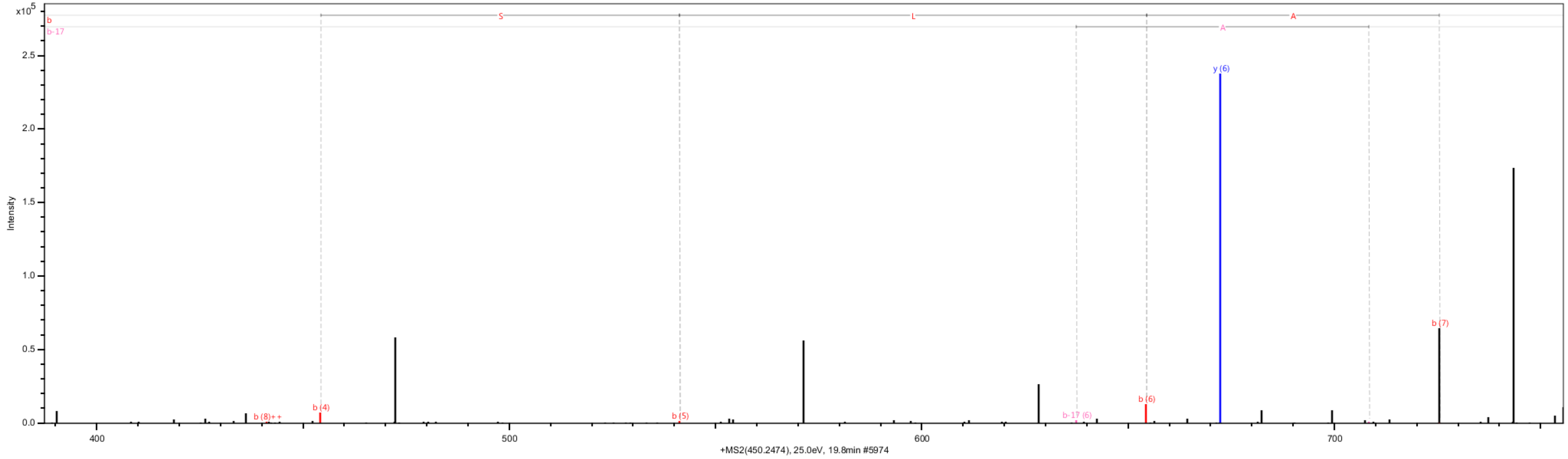

CRFR2_HUMAN

**Accession:**	CRFR2_HUMAN	**Score:**	16.4
**Database:**	SwissProt	**Seq. Coverage [%]:**	1.7
**MW [kDa] / pI:**	47.7 / 7.9	**No. of Peptides:**	1

Peptide Match

**Sequence:**	R.ISFHSIK.Q	**Score:**	16.4
**Modifications:**			

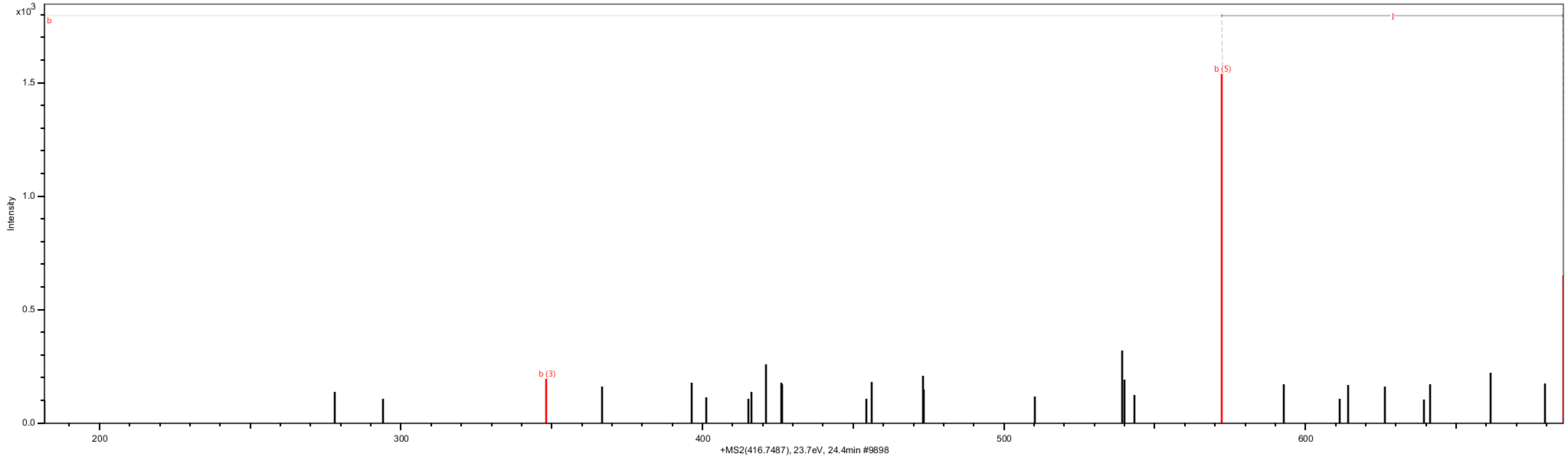

ZNT1_HUMAN

**Accession:**	ZNT1_HUMAN	**Score:**	16.4
**Database:**	SwissProt	**Seq. Coverage [%]:**	2.2
**MW [kDa] / pI:**	55.3 / 6.0	**No. of Peptides:**	1

Peptide Match

**Sequence:**	K.NMPNKQPESSL.-	**Score:**	16.4
**Modifications:**			

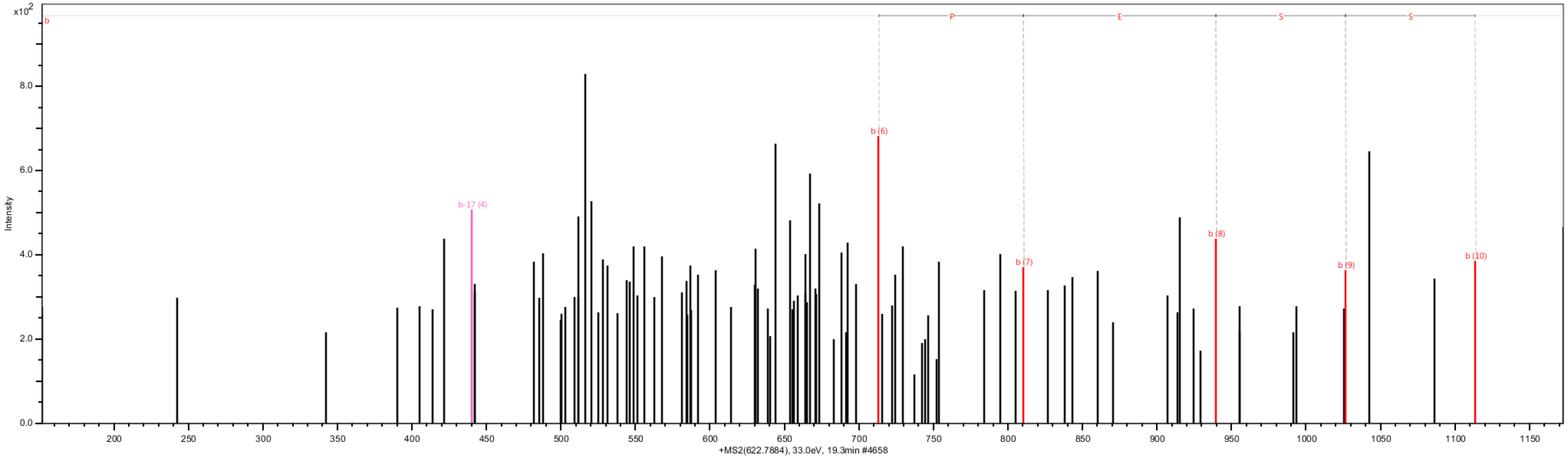

CLIC6_HUMAN

**Accession:**	CLIC6_HUMAN	**Score:**	16.4
**Database:**	SwissProt	**Seq. Coverage [%]:**	1.1
**MW [kDa] / pI:**	73.0 / 4.3	**No. of Peptides:**	1

Peptide Match

**Sequence:**	R.YLNNAYAR.D	**Score:**	16.4
**Modifications:**			

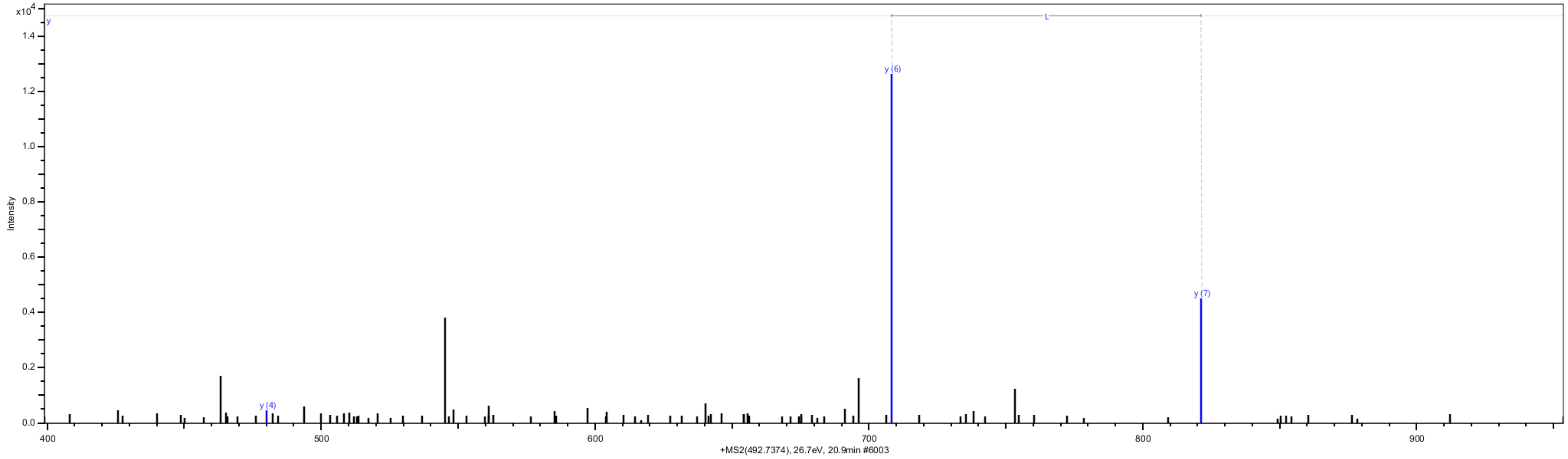

RET3_HUMAN

Accession: RET3_HUMAN
Database: SwissProt
MW [kDa] / pI: 135.3 / 5.0
Score: 16.3
Seq. Coverage [%]: 0.6
No. of Peptides: 1

Peptide Match

Sequence: R.RAIVVGER.T
Score: 16.3
Modifications:

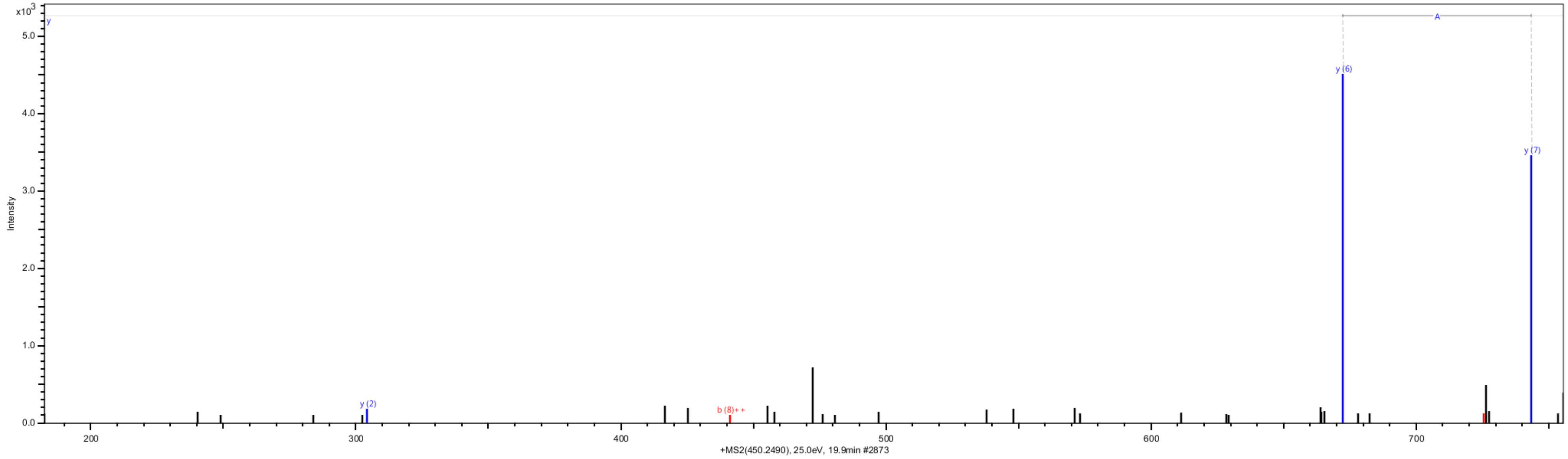

ELOA1_HUMAN

Accession: ELOA1_HUMAN
Database: SwissProt
MW [kDa] / pI: 89.9 / 9.6
Score: 16.3
Seq. Coverage [%]: 1.3
No. of Peptides: 1

Peptide Match

Sequence: K.NLDSVQKLPK.V
Score: 16.3
Modifications:

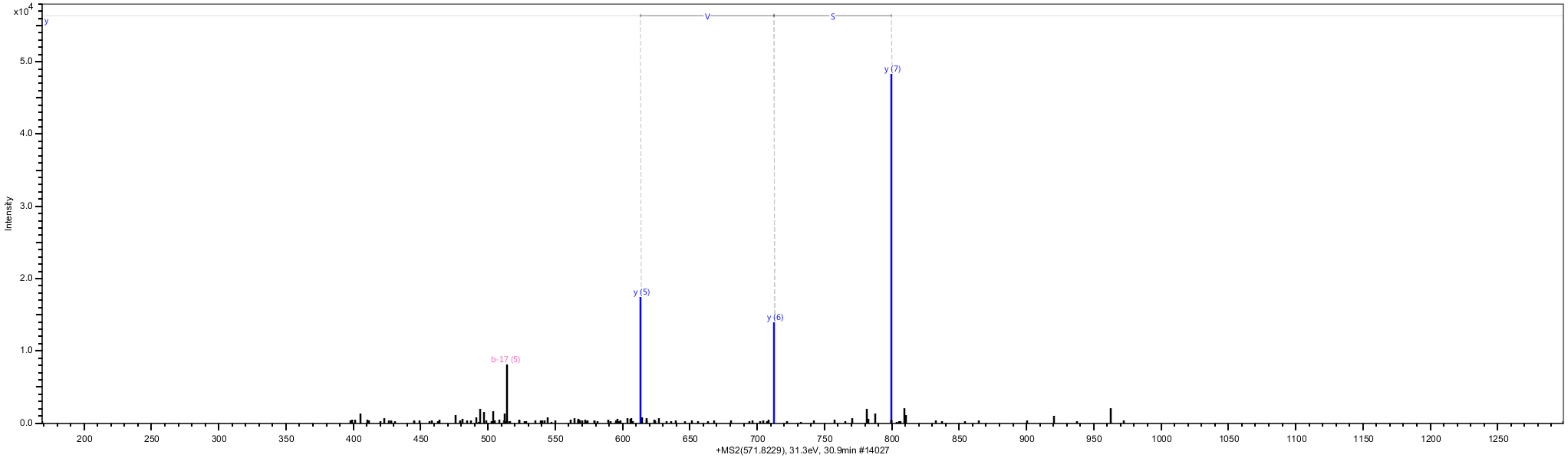

IFT1B_HUMAN

Accession: IFT1B_HUMAN
Database: SwissProt
MW [kDa] / pI: 55.0 / 7.6
Score: 16.3
Seq. Coverage [%]: 3.2
No. of Peptides: 1

Peptide Match

Sequence: R.AQMIQIKEATNWQPR.G
Score: 16.3
Modifications: Oxidation: 3

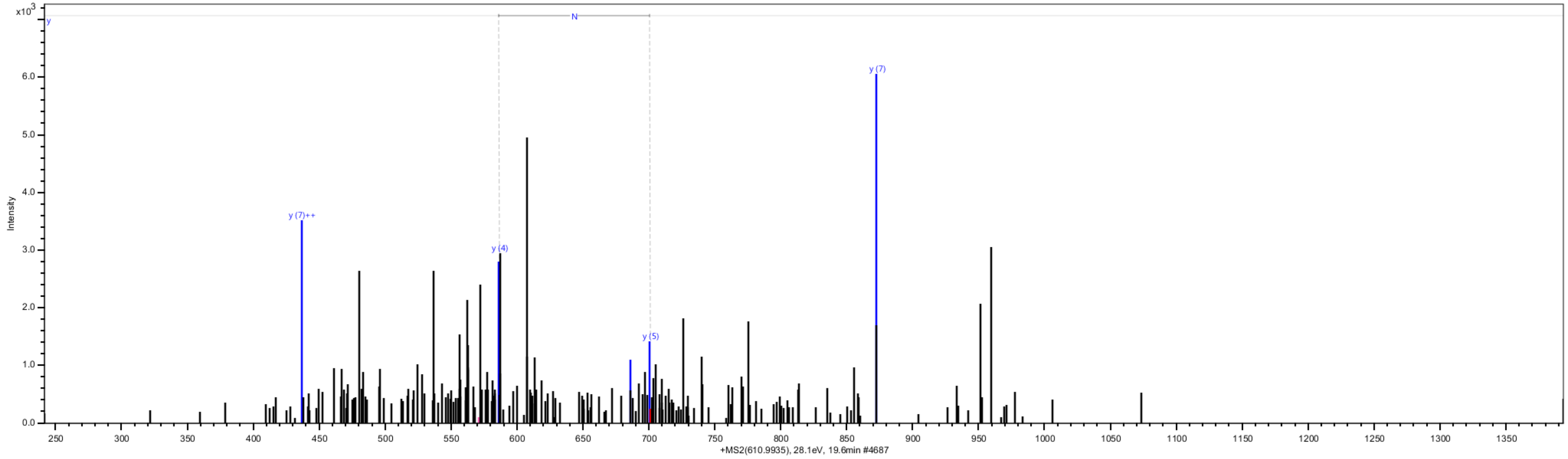

XRN2_HUMAN

**Accession:**	XRN2_HUMAN	**Score:**	16.3
**Database:**	SwissProt	**Seq. Coverage [%]:**	0.8
**MW [kDa] / pI:**	108.5 / 7.3	**No. of Peptides:**	1

Peptide Match

**Sequence:**	R.TLGHVMPR.G	**Score:**	16.3
**Modifications:**	Oxidation: 6		

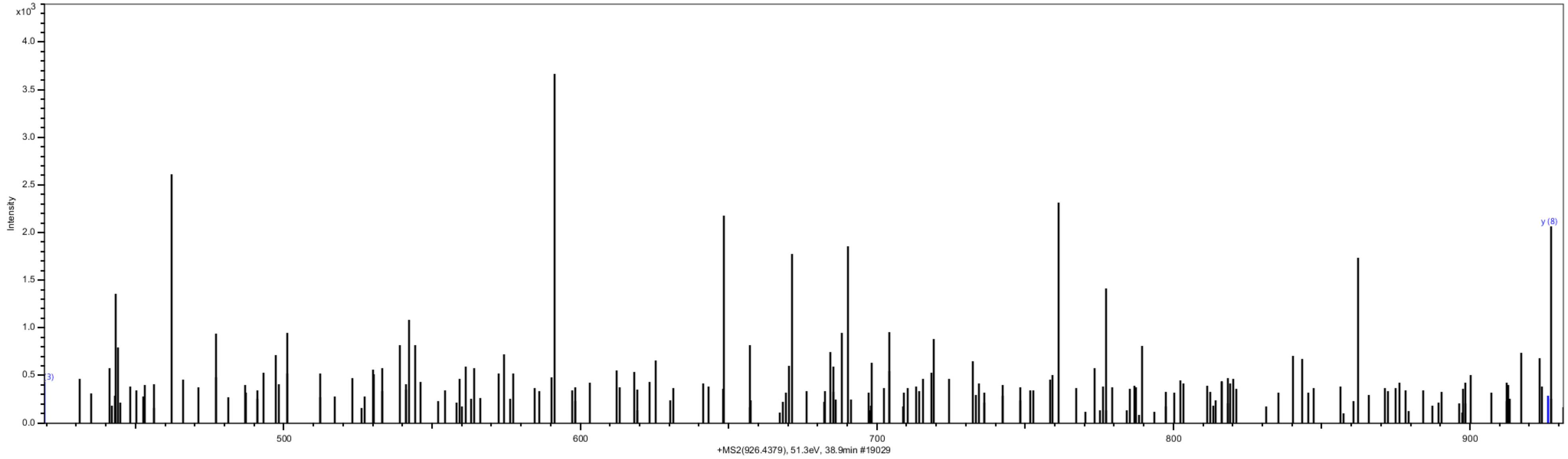

DYR_HUMAN

Accession: DYR_HUMAN
Database: SwissProt
MW [kDa] / pI: 21.4 / 6.8
Score: 16.3
Seq. Coverage [%]: 5.3
No. of Peptides: 1

Peptide Match

Sequence: R.MTTTSSVEGK.Q
Score: 16.3
Modifications: Oxidation: 1

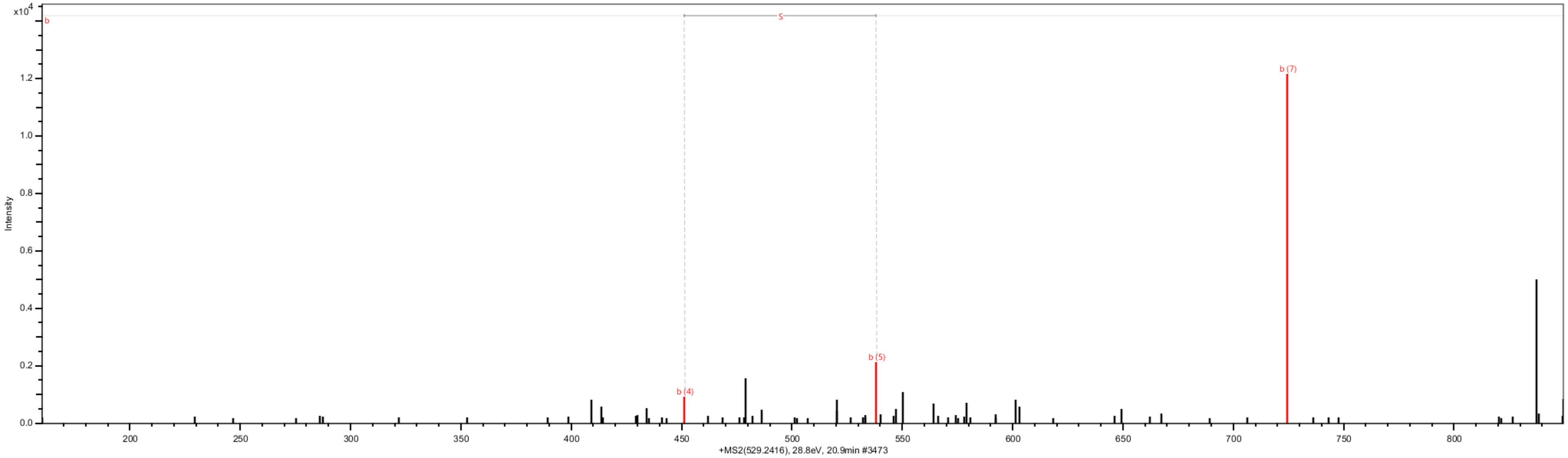

TUT4_HUMAN

Accession: TUT4_HUMAN
Database: SwissProt
MW [kDa] / pI: 185.0 / 8.3
Score: 16.3
Seq. Coverage [%]: 0.5
No. of Peptides: 1

Peptide Match

Sequence: K.SPNSVKAEK.A
Score: 16.3
Modifications:

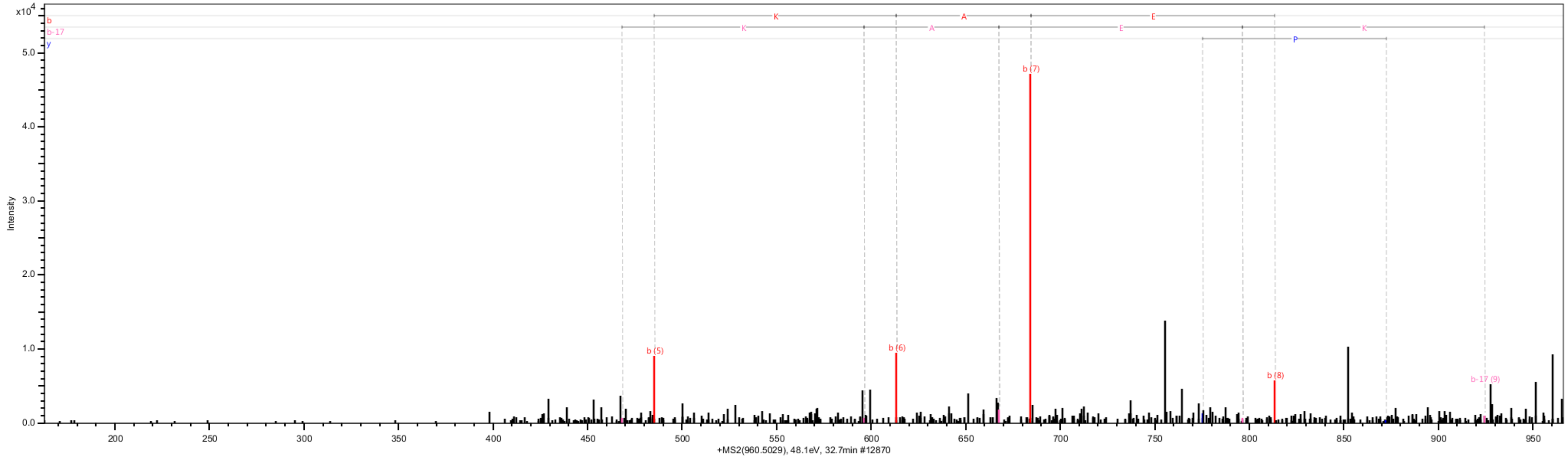

TR61B_HUMAN

**Accession:**	TR61B_HUMAN	**Score:**	16.3
**Database:**	SwissProt	**Seq. Coverage [%]:**	3.1
**MW [kDa] / pI:**	52.9 / 6.2	**No. of Peptides:**	1

Peptide Match

**Sequence:**	-.MLMAWCRGPVLLCLR.Q	**Score:**	16.3
**Modifications:**	Carbamidomethyl: 6, 13; Oxidation: 1		

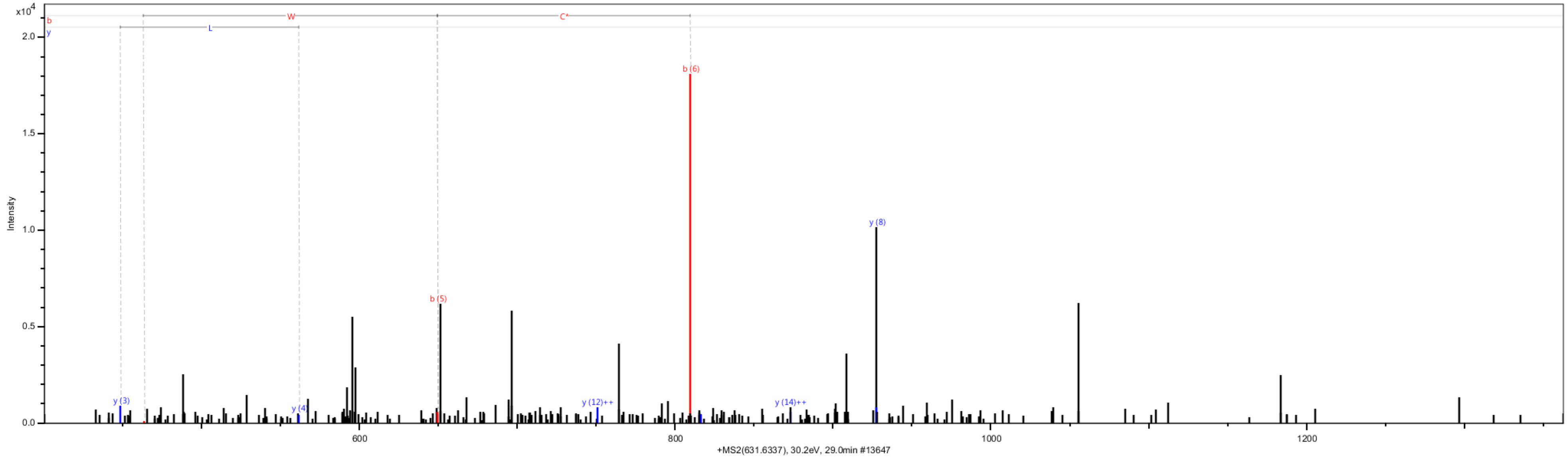

INHBA_HUMAN

**Accession:**	INHBA_HUMAN	**Score:**	16.3
**Database:**	SwissProt	**Seq. Coverage [%]:**	3.3
**MW [kDa] / pI:**	47.4 / 8.3	**No. of Peptides:**	1

Peptide Match

**Sequence:**	K.DVPNSQPEMVEAVK.K	**Score:**	16.3
**Modifications:**			

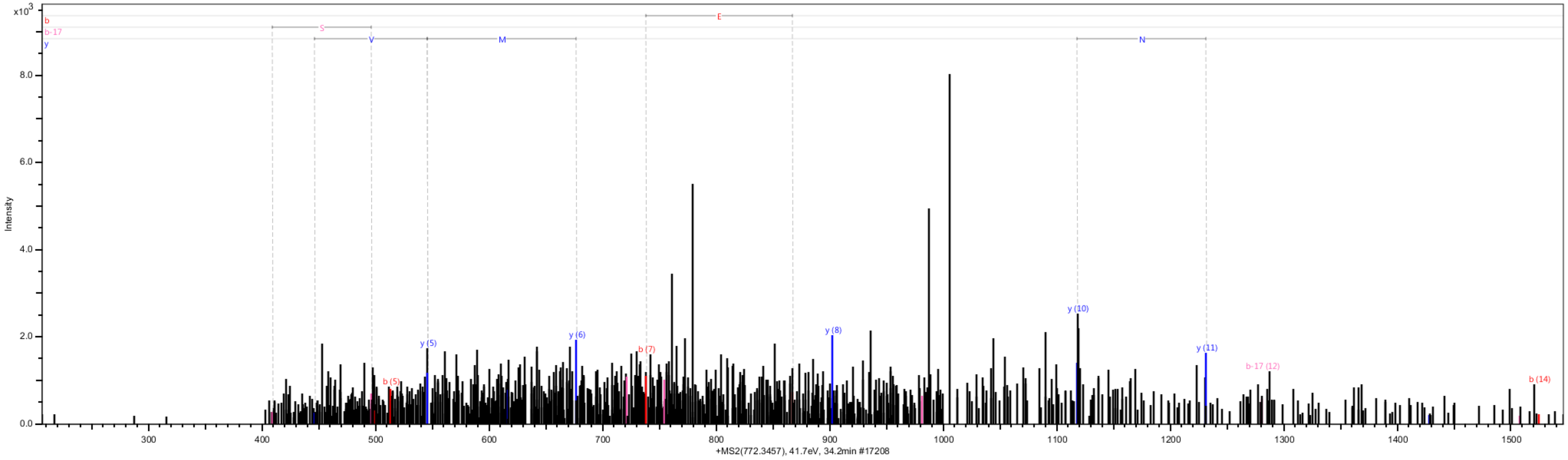

RGPS1_HUMAN

Accession: RGPS1_HUMAN
Database: SwissProt
MW [kDa] / pI: 62.1 / 9.2
Score: 16.3
Seq. Coverage [%]: 2.2
No. of Peptides: 1

Peptide Match

Sequence: K.LDYLMSKEDNYK.R
Score: 16.2
Modifications:

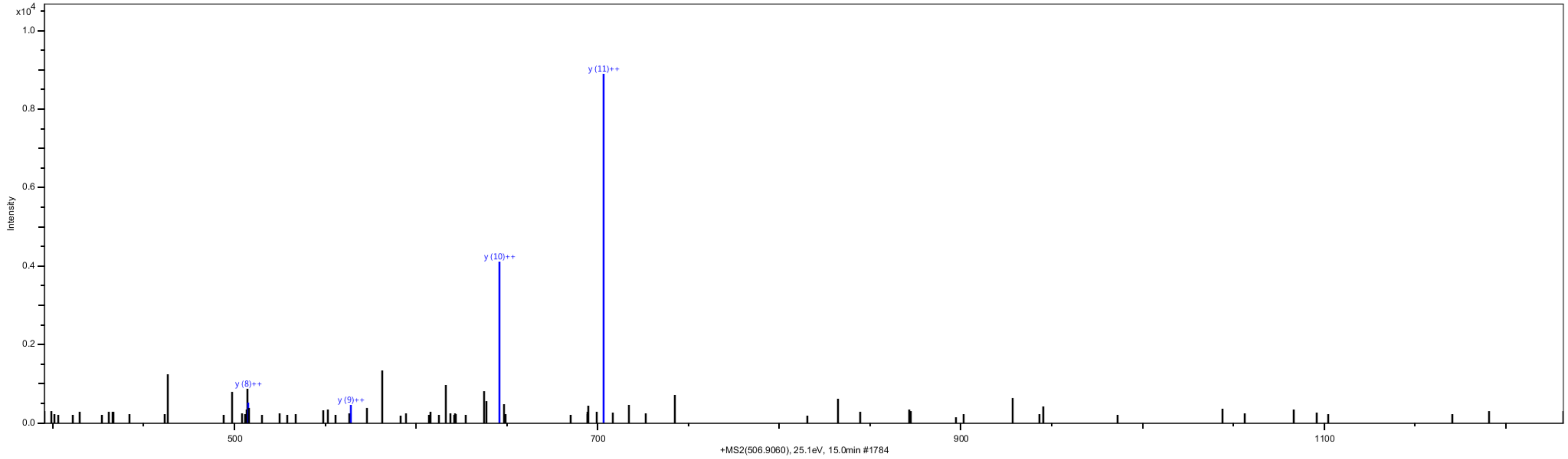

Spectrum Report
Combine Search_2020-10-19 07:53:31

UH1BL_HUMAN

Accession: UH1BL_HUMAN
Database: SwissProt
MW [kDa] / pI: 164.1 / 5.9
Score: 16.3
Seq. Coverage [%]: 0.8
No. of Peptides: 1

Peptide Match

Sequence: K.ITGVNGEIDIR.G
Score: 16.2
Modifications:

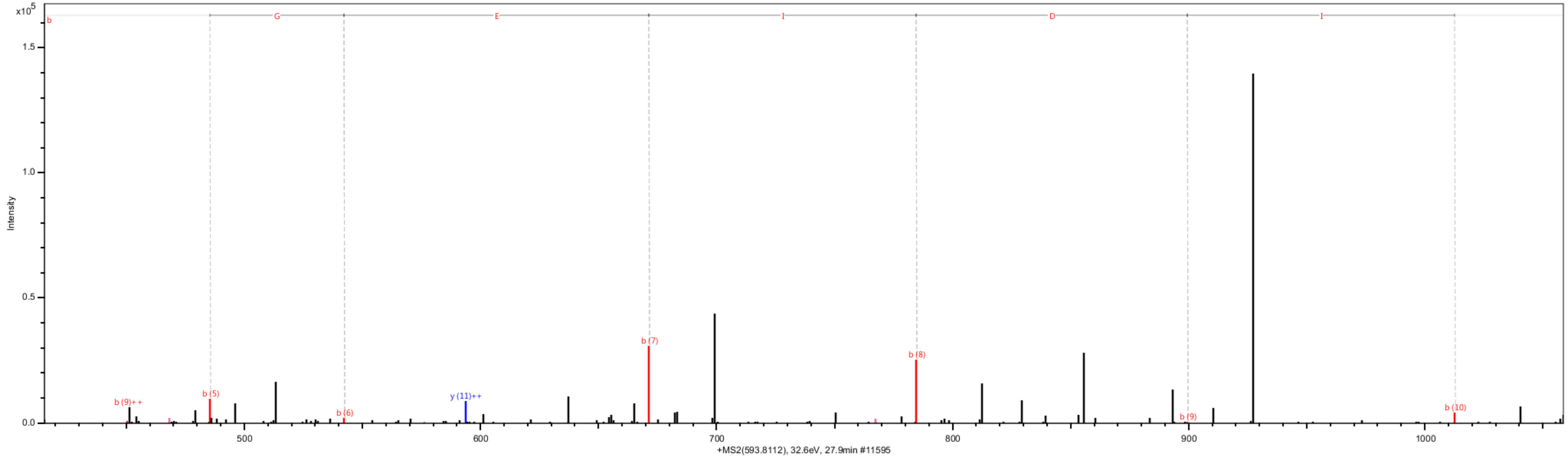

LMO7_HUMAN

**Accession:**	LMO7_HUMAN	**Score:**	16.2
**Database:**	SwissProt	**Seq. Coverage [%]:**	0.5
**MW [kDa] / pI:**	192.6 / 8.3	**No. of Peptides:**	1

Peptide Match

**Sequence:**	R.RISAVEPK.T	**Score:**	16.2
**Modifications:**			

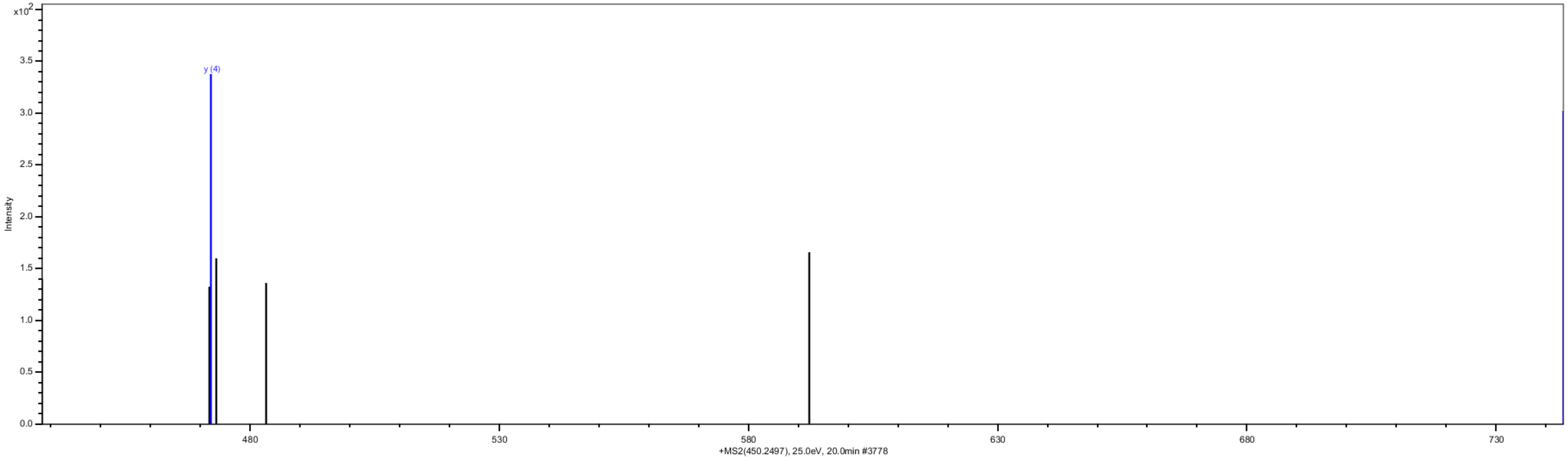

ZMYM4_HUMAN

**Accession:**	ZMYM4_HUMAN	**Score:**	16.2
**Database:**	SwissProt	**Seq. Coverage [%]:**	0.8
**MW [kDa] / pI:**	172.7 / 6.5	**No. of Peptides:**	1

Peptide Match

**Sequence:**	K.KINNVMAMCEYCK.I	**Score:**	16.2
**Modifications:**	Carbamidomethyl: 9, 12		

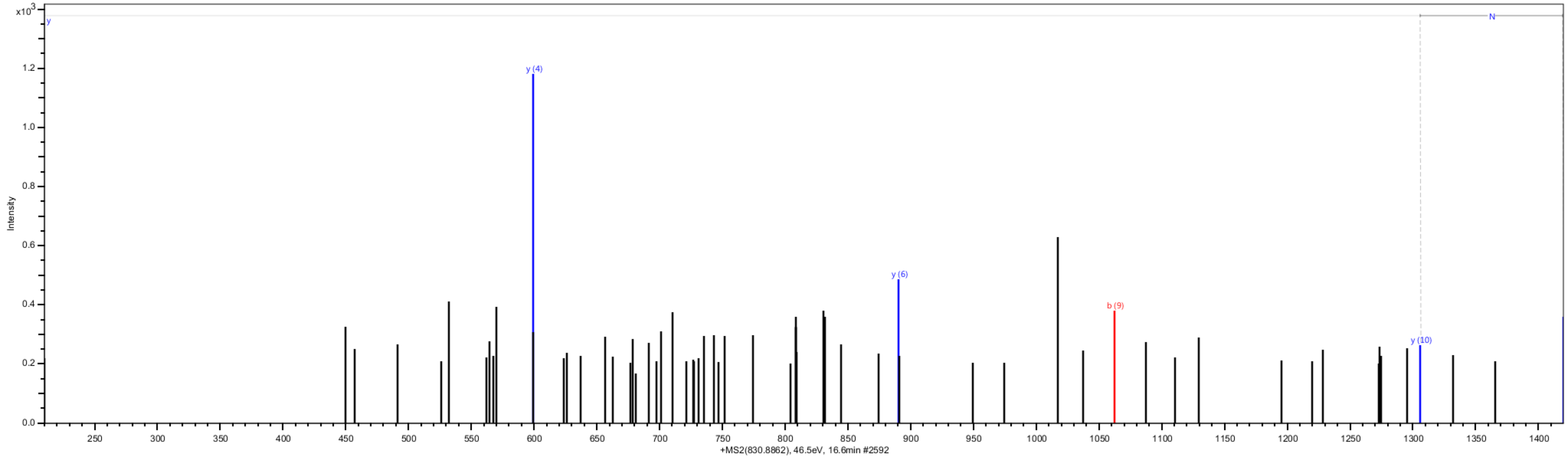

ICE2_HUMAN

Accession: ICE2_HUMAN
Database: SwissProt
MW [kDa] / pI: 109.9 / 6.7
Score: 18.5
Seq. Coverage [%]: 1.0
No. of Peptides: 1

Peptide Match

Sequence: K.VTRTAYNLYK.T
Score: 18.5
Modifications:

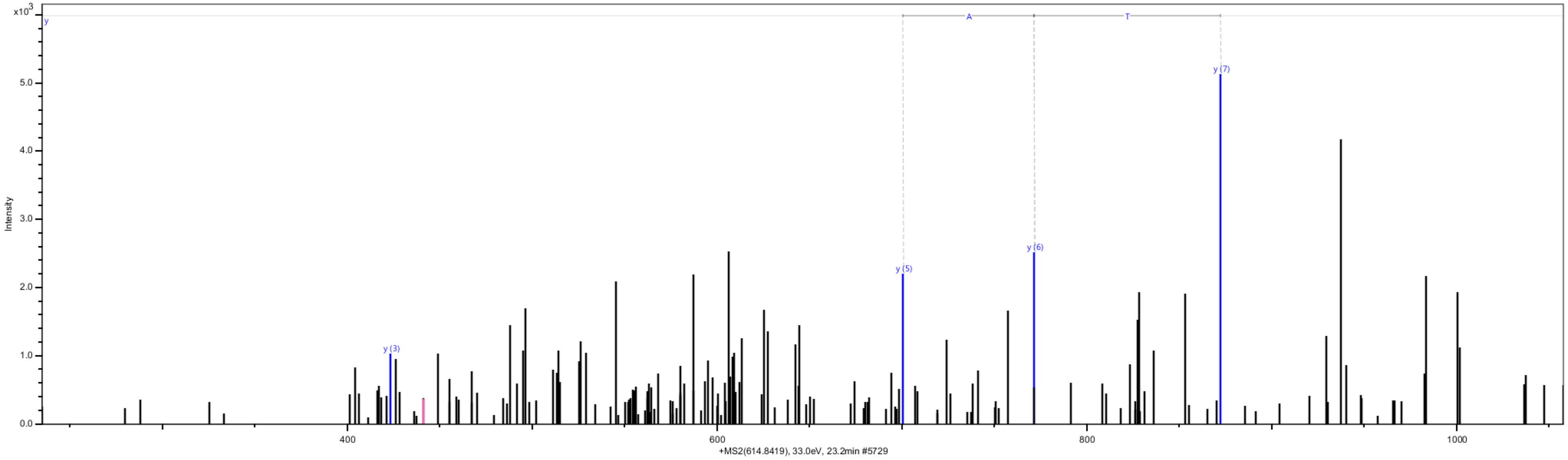

FRPD3_HUMAN

Accession: FRPD3_HUMAN
Database: SwissProt
MW [kDa] / pI: 199.1 / 8.4
Score: 18.4
Seq. Coverage [%]: 0.6
No. of Peptides: 1

Peptide Match

Sequence: R.SKLEETSLVPR.A
Score: 18.4
Modifications:

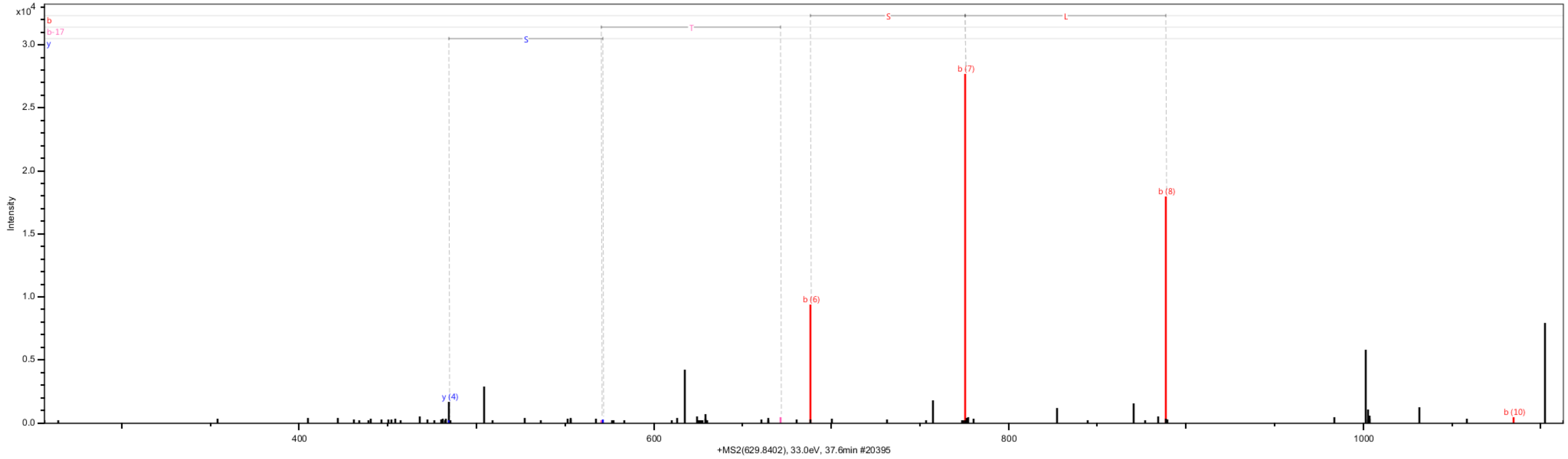

CYLC1_HUMAN

**Accession:**	CYLC1_HUMAN	**Score:**	18.5
**Database:**	SwissProt	**Seq. Coverage [%]:**	1.1
**MW [kDa] / pI:**	74.2 / 9.7	**No. of Peptides:**	1

Peptide Match

**Sequence:**	R.GFRMSSK.K	**Score:**	18.5
**Modifications:**			

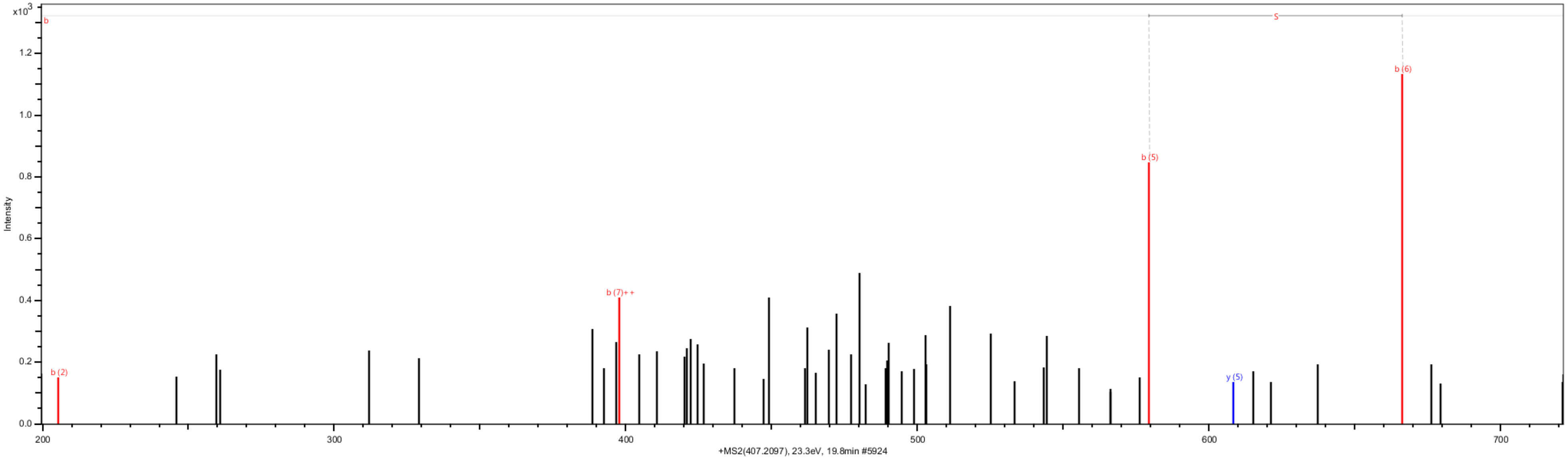

HACE1_HUMAN

**Accession:**	HACE1_HUMAN	**Score:**	18.5
**Database:**	SwissProt	**Seq. Coverage [%]:**	1.1
**MW [kDa] / pI:**	102.3 / 5.6	**No. of Peptides:**	1

Peptide Match

**Sequence:**	R.SVSELLSNSK.F	**Score:**	18.5
**Modifications:**			

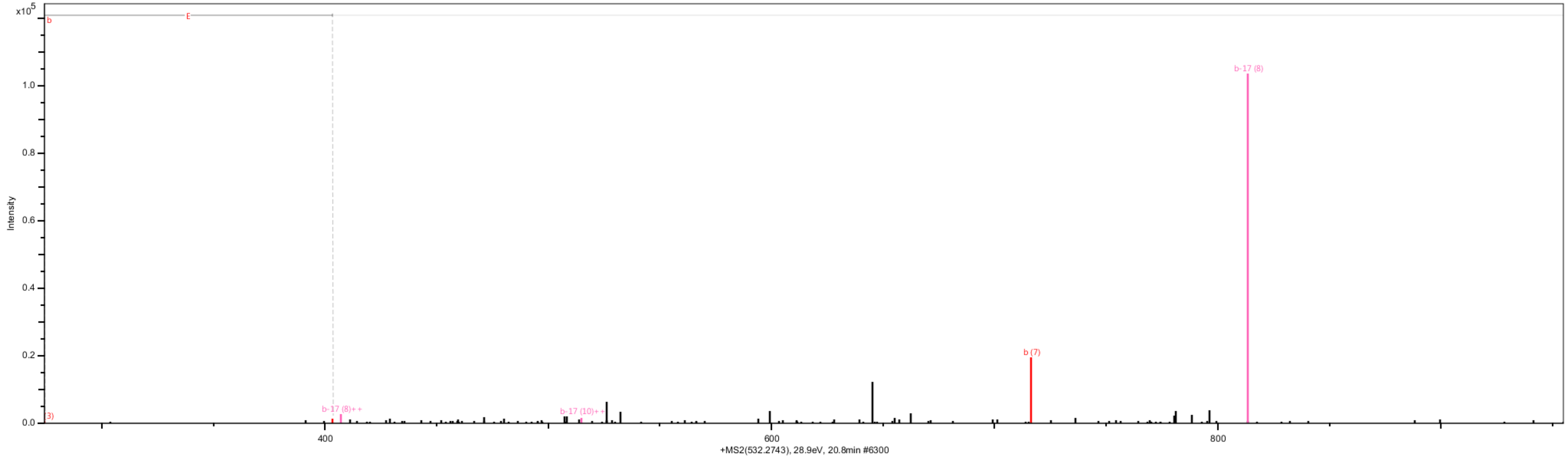

CQ082_HUMAN

Accession: CQ082_HUMAN
Database: SwissProt
MW [kDa] / pI: 25.4 / 10.4
Score: 18.6
Seq. Coverage [%]: 3.6
No. of Peptides: 1

Peptide Match

Sequence: R.EREIDCGPR.Q
Score: 18.6
Modifications: Carbamidomethyl: 6

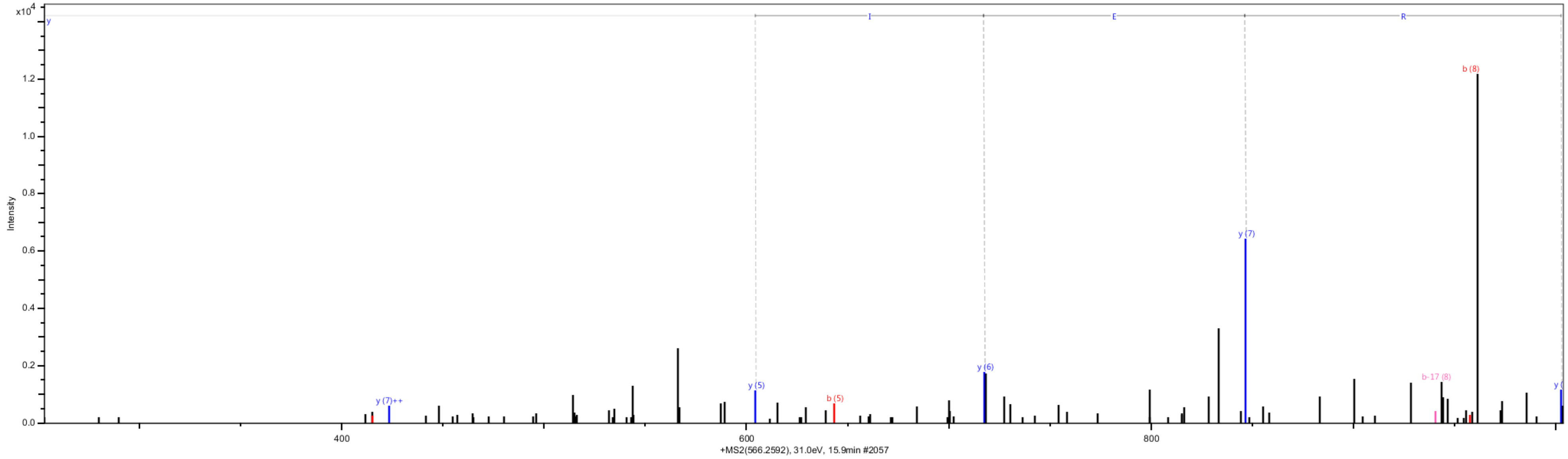

ULA1_HUMAN

**Accession:**	ULA1_HUMAN	**Score:**	18.5
**Database:**	SwissProt	**Seq. Coverage [%]:**	1.5
**MW [kDa] / pI:**	60.2 / 5.2	**No. of Peptides:**	1

Peptide Match

**Sequence:**	R.ALKEFVAK.E	**Score:**	18.5
**Modifications:**			

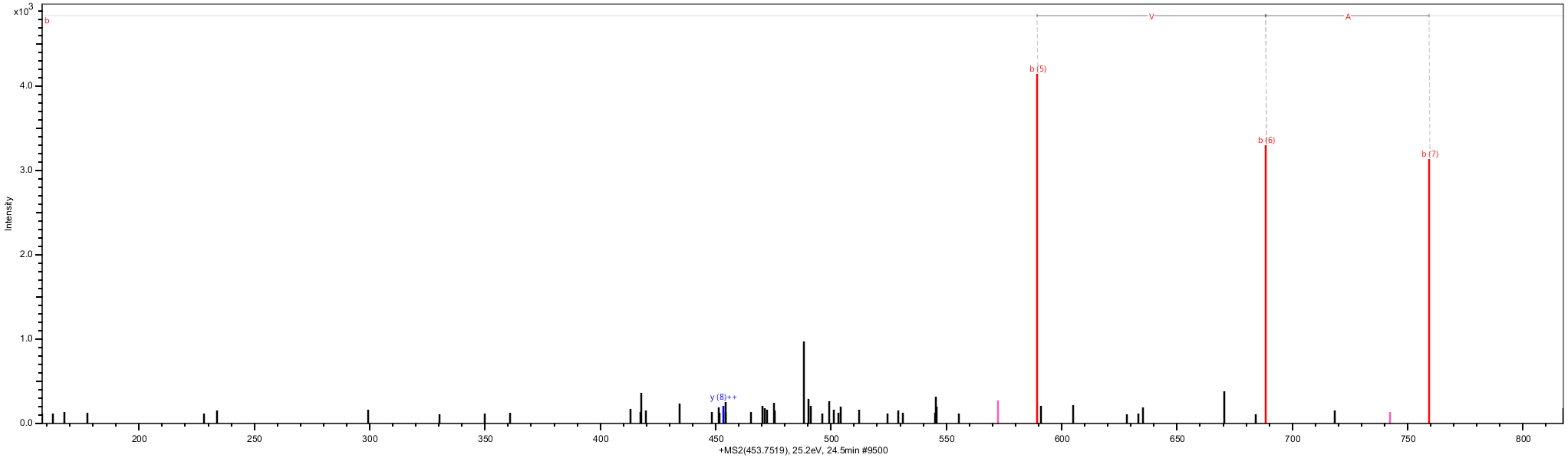

MGAP_HUMAN

**Accession:**	MGAP_HUMAN	**Score:**	18.6
**Database:**	SwissProt	**Seq. Coverage [%]:**	0.3
**MW [kDa] / pI:**	336.0 / 6.5	**No. of Peptides:**	1

Peptide Match

**Sequence:**	K.AQEFLPKK.I	**Score:**	18.6
**Modifications:**			

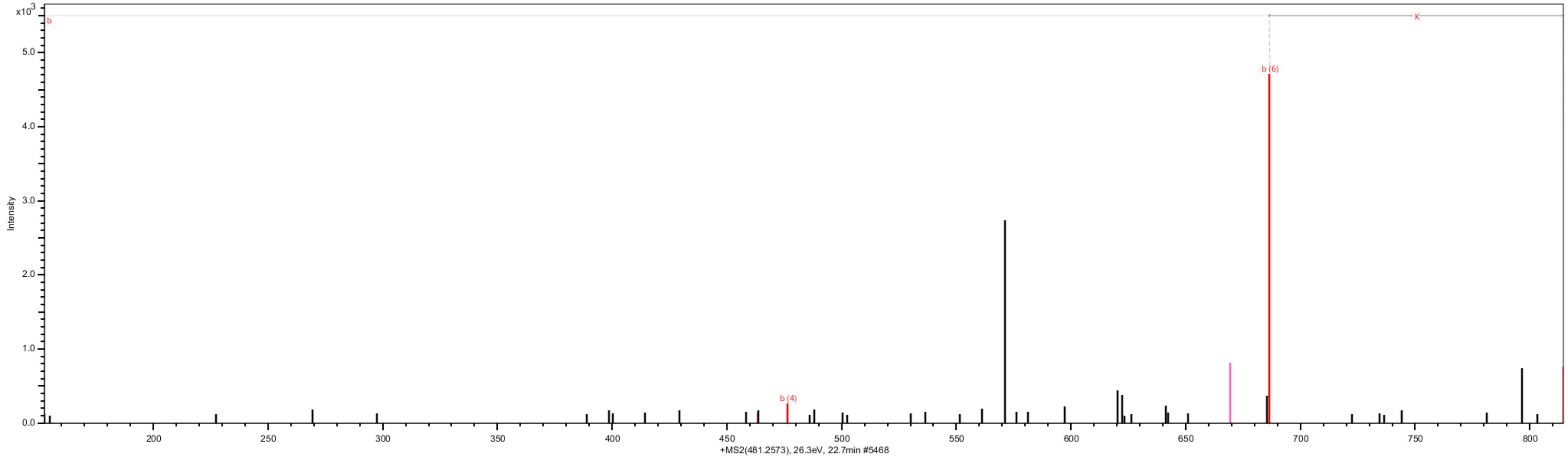

DCD_HUMAN

Accession: DCD_HUMAN
Database: SwissProt
MW [kDa] / pI: 11.3 / 6.1
Score: 18.6
Seq. Coverage [%]: 7.3
No. of Peptides: 1

Peptide Match

Sequence: K.QRSSLLEK.G
Score: 18.6
Modifications:

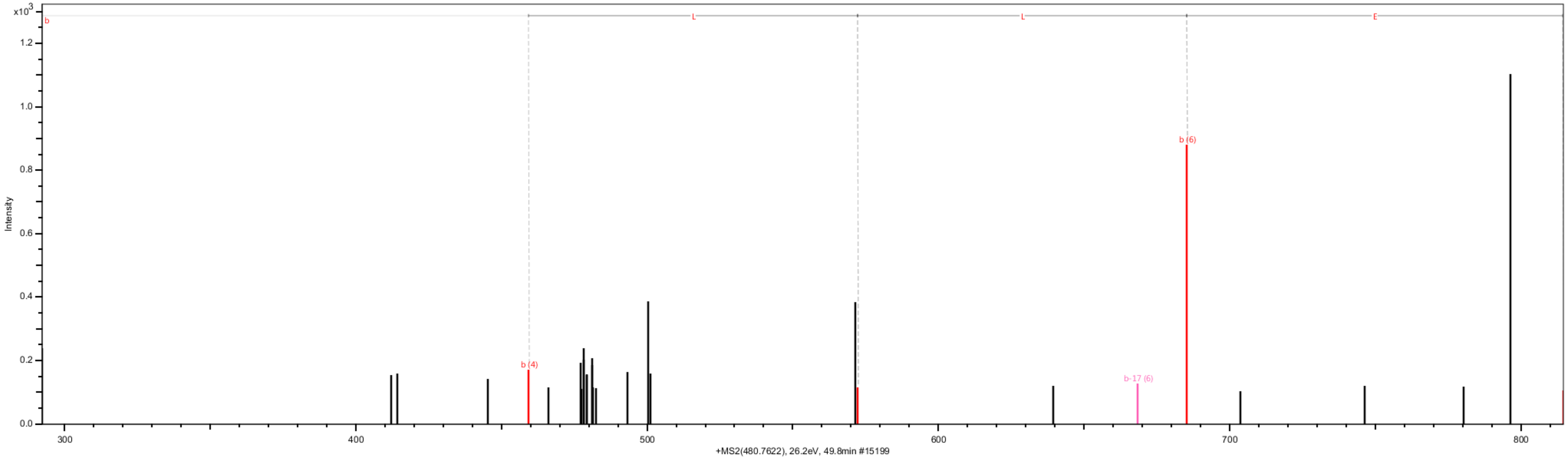

Spectrum Report
Combine Search_2020-10-19 07:53:31

PAR14_HUMAN

Accession: PAR14_HUMAN
Database: SwissProt
MW [kDa] / pI: 202.7 / 6.8
Score: 18.7
Seq. Coverage [%]: 0.7
No. of Peptides: 1

Peptide Match

Sequence: K.TMDAKNGQTMNEK.Q
Score: 18.6
Modifications: Oxidation: 2, 10

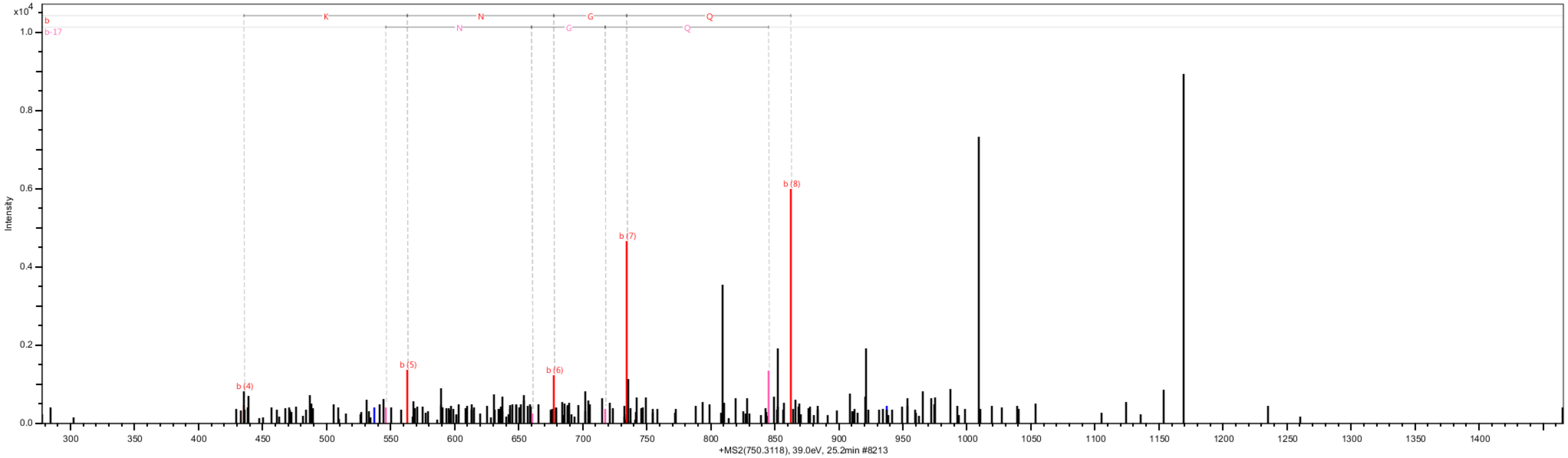

PAPP1_HUMAN

Accession: PAPP1_HUMAN
Database: SwissProt
MW [kDa] / pI: 180.9 / 5.8
Score: 18.6
Seq. Coverage [%]: 0.7
No. of Peptides: 1

Peptide Match

Sequence: R.SPAVITGLYDK.C
Score: 18.6
Modifications:

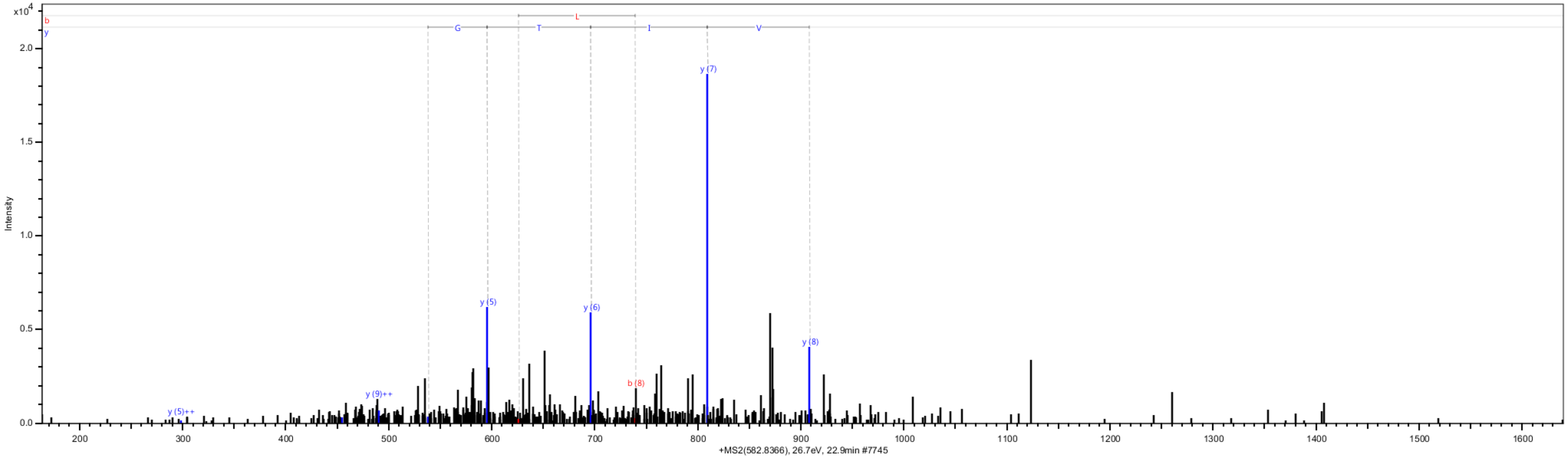

SLTM_HUMAN

Accession: SLTM_HUMAN
Database: SwissProt
MW [kDa] / pI: 117.1 / 7.7
Score: 18.7
Seq. Coverage [%]: 1.0
No. of Peptides: 1

Peptide Match

Sequence: K.ITDLRVIDLK.S
Score: 18.7
Modifications:

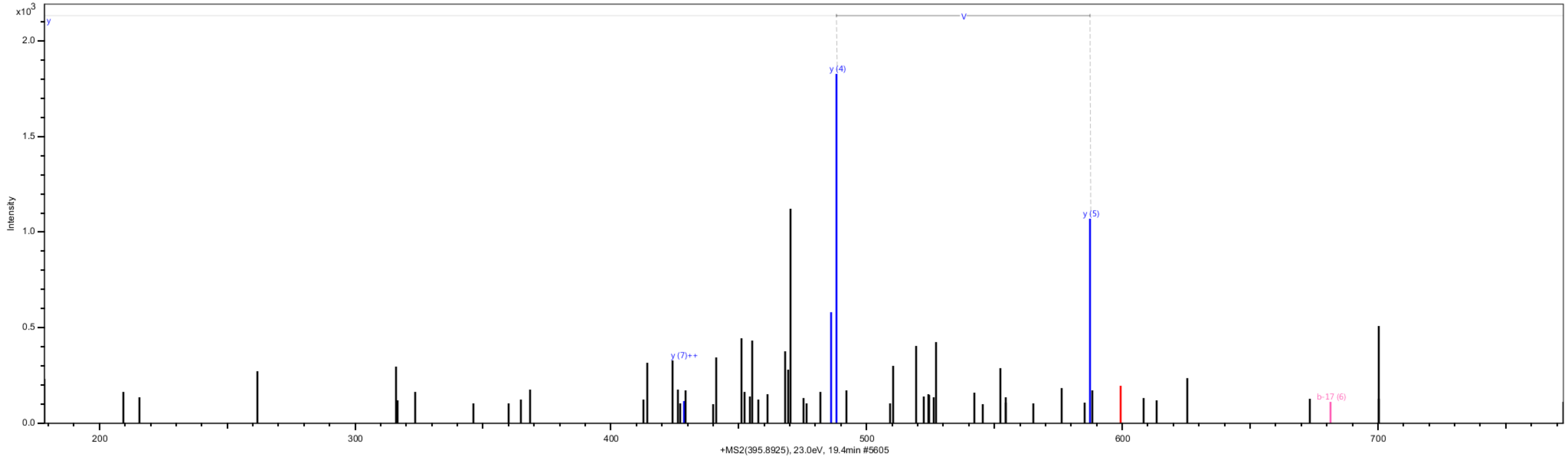

BIG3_HUMAN

Accession: BIG3_HUMAN
Database: SwissProt
MW [kDa] / pI: 240.5 / 5.5
Score: 18.7
Seq. Coverage [%]: 0.4
No. of Peptides: 1

Peptide Match

Sequence: K.LQKEASGSK.Y
Score: 18.7
Modifications:

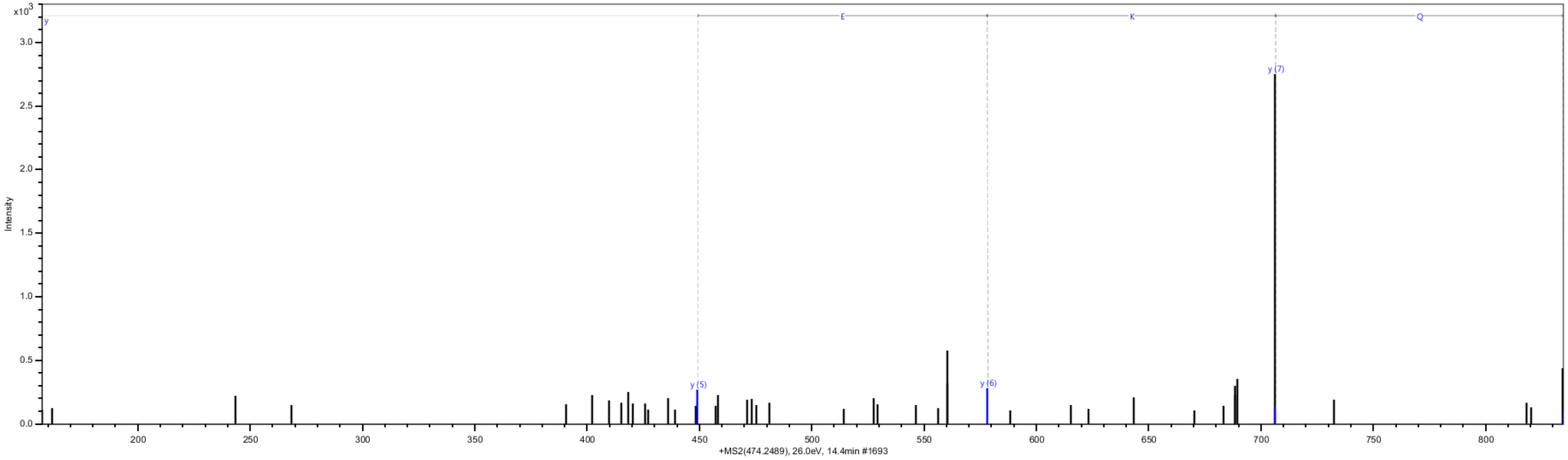

WAPL_HUMAN

Accession: WAPL_HUMAN
Database: SwissProt
MW [kDa] / pI: 132.9 / 5.3
Score: 18.7
Seq. Coverage [%]: 0.8
No. of Peptides: 1

Peptide Match

Sequence: K.SSQGASNFDK.L
Score: 18.7
Modifications:

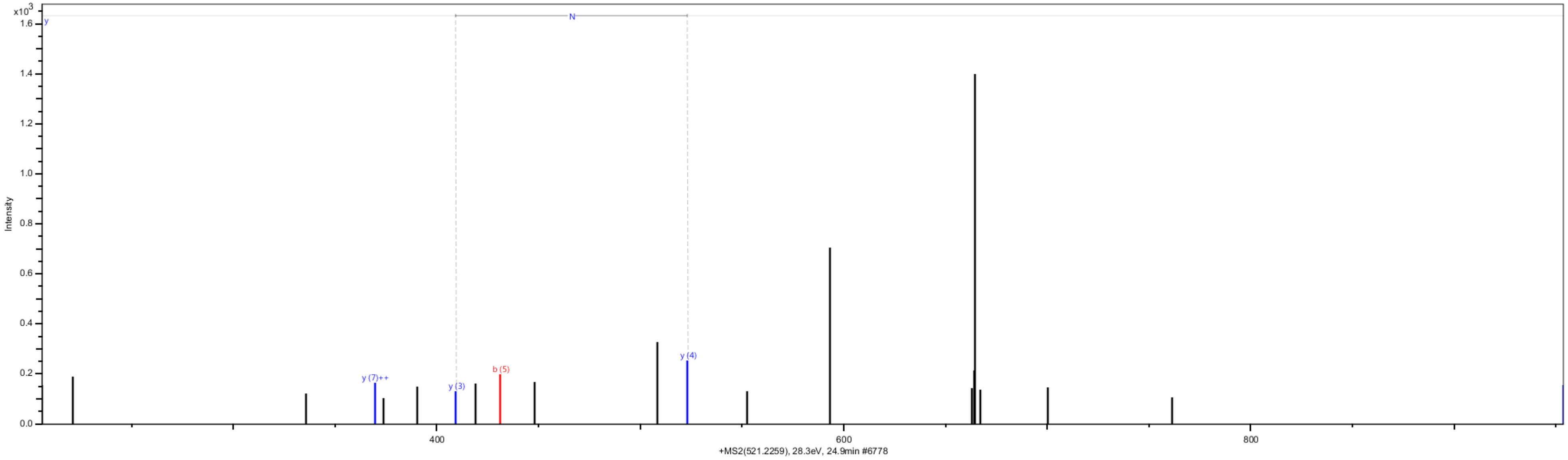

ARIP4_HUMAN

Accession: ARIP4_HUMAN
Database: SwissProt
MW [kDa] / pI: 162.7 / 5.7
Score: 18.7
Seq. Coverage [%]: 0.6
No. of Peptides: 1

Peptide Match

Sequence: R.LVADYTLEK.K
Score: 18.7
Modifications:

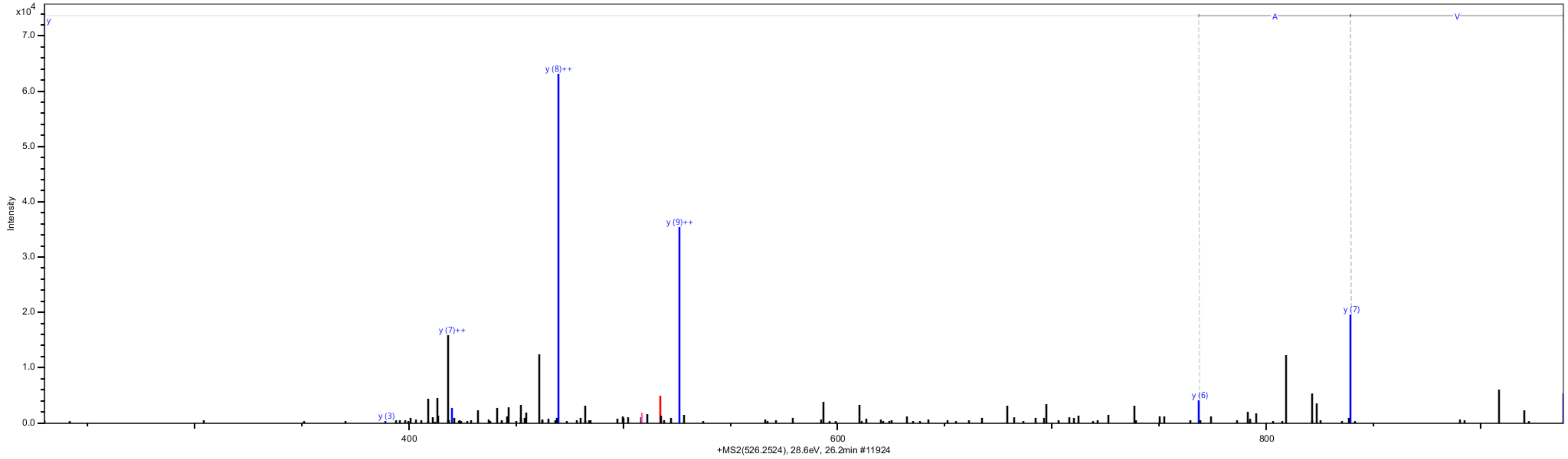

SGCG_HUMAN

**Accession:**	SGCG_HUMAN	**Score:**	18.7
**Database:**	SwissProt	**Seq. Coverage [%]:**	3.8
**MW [kDa] / pI:**	32.4 / 5.6	**No. of Peptides:**	1

Peptide Match

**Sequence:**	R.NSEGEVTGRLK.V	**Score:**	18.7
**Modifications:**			

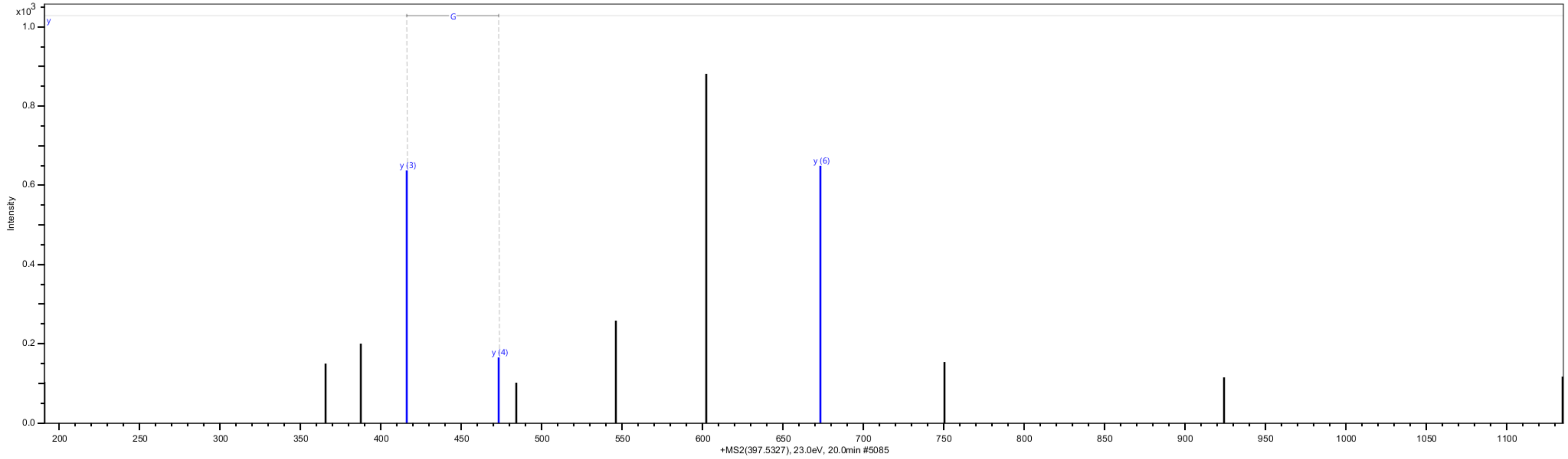

DOCK4_HUMAN

Accession: DOCK4_HUMAN
Database: SwissProt
MW [kDa] / pI: 225.1 / 7.6
Score: 18.7
Seq. Coverage [%]: 0.6
No. of Peptides: 1

Peptide Match

Sequence: R.DCMKMGEVDGK.K
Score: 18.7
Modifications: Carbamidomethyl: 2

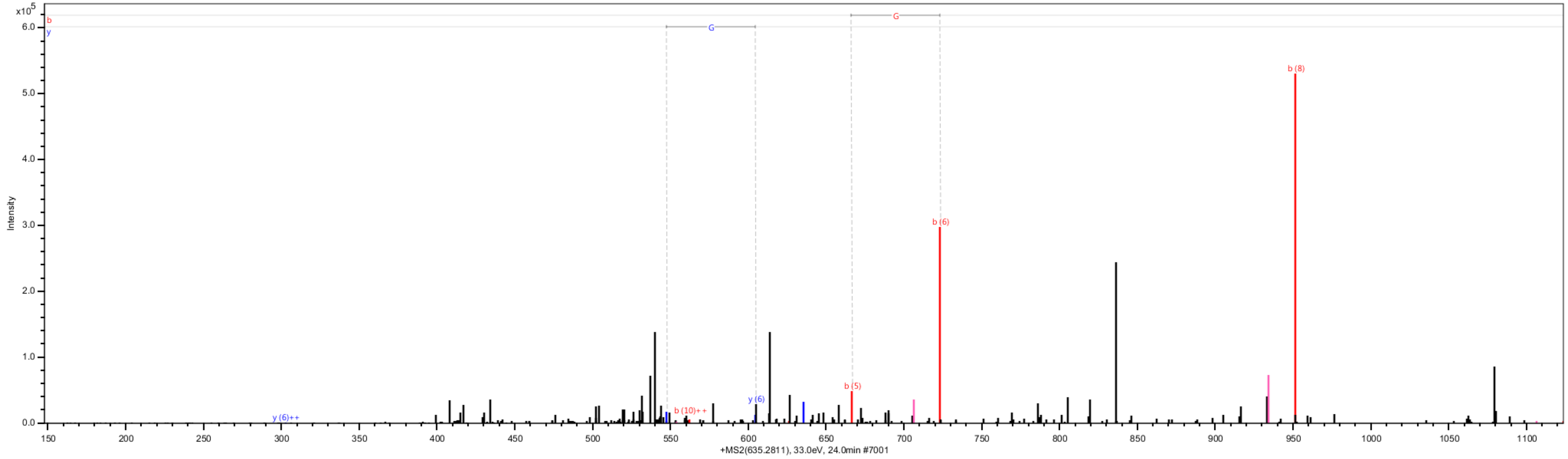

CBPQ_HUMAN

**Accession:**	CBPQ_HUMAN	**Score:**	18.2
**Database:**	SwissProt	**Seq. Coverage [%]:**	1.9
**MW [kDa] / pI:**	51.9 / 5.8	**No. of Peptides:**	1

Peptide Match

**Sequence:**	K.VGALASLIR.S	**Score:**	18.2
**Modifications:**			

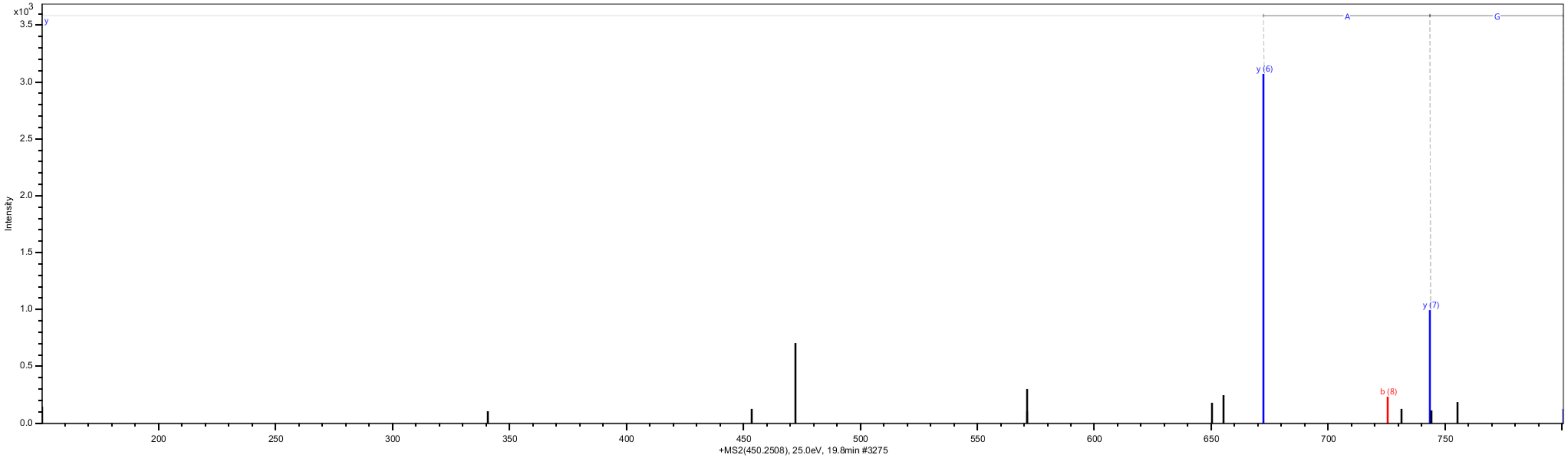

ZNT6_HUMAN

Accession: ZNT6_HUMAN
Database: SwissProt
MW [kDa] / pI: 51.1 / 9.3
Score: 18.2
Seq. Coverage [%]: 2.4
No. of Peptides: 1

Peptide Match

Sequence: R.FLEQPEIHTGR.L
Score: 18.2
Modifications:

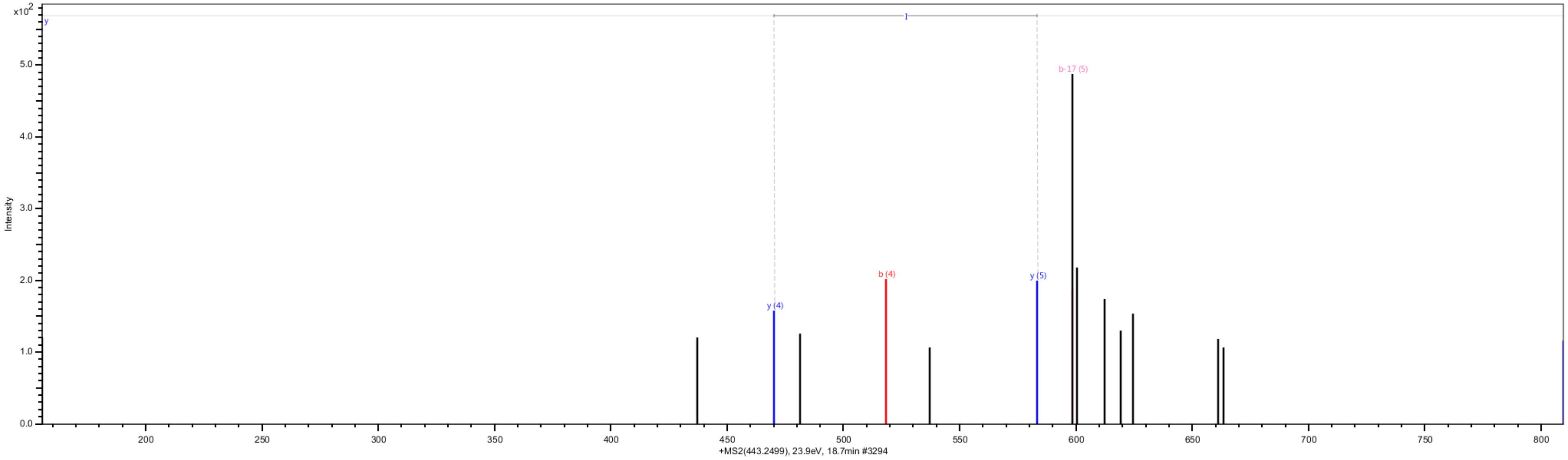

TM198_HUMAN

Accession: TM198_HUMAN
Database: SwissProt
MW [kDa] / pI: 39.4 / 10.0
Score: 18.3
Seq. Coverage [%]: 2.5
No. of Peptides: 1

Peptide Match

Sequence: -.MPGTVATLR.F
Score: 18.3
Modifications:

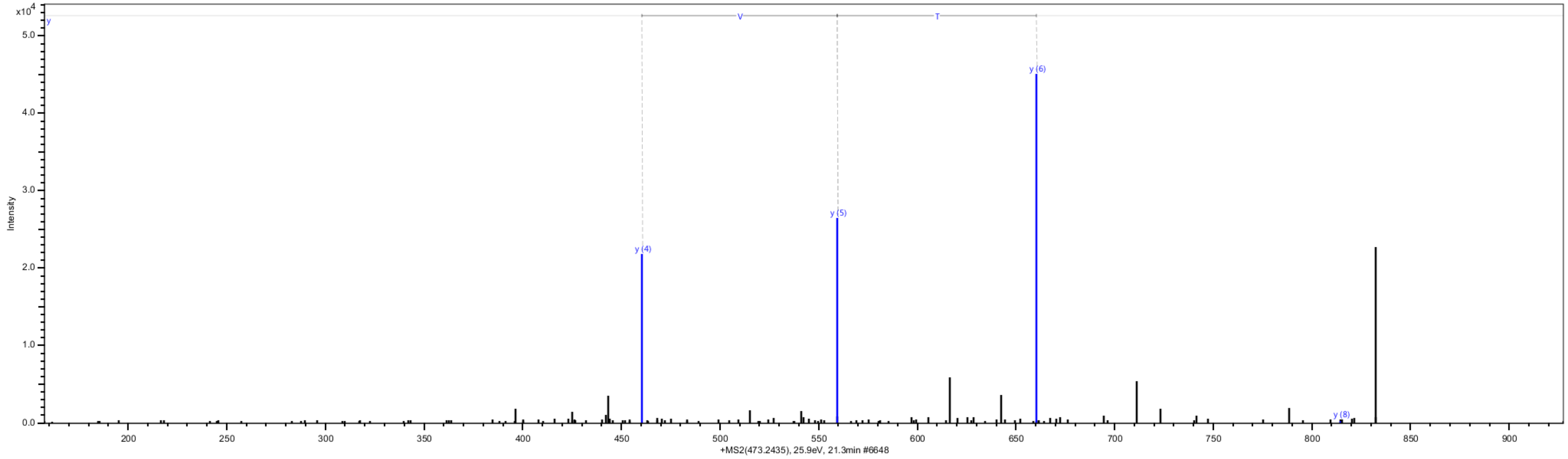

GPTC4_HUMAN

Accession: GPTC4_HUMAN
Database: SwissProt
MW [kDa] / pI: 50.4 / 9.6
Score: 18.3
Seq. Coverage [%]: 2.5
No. of Peptides: 1

Peptide Match

Sequence: R.GMKFAEEQLLK.H
Score: 18.3
Modifications: Oxidation: 2

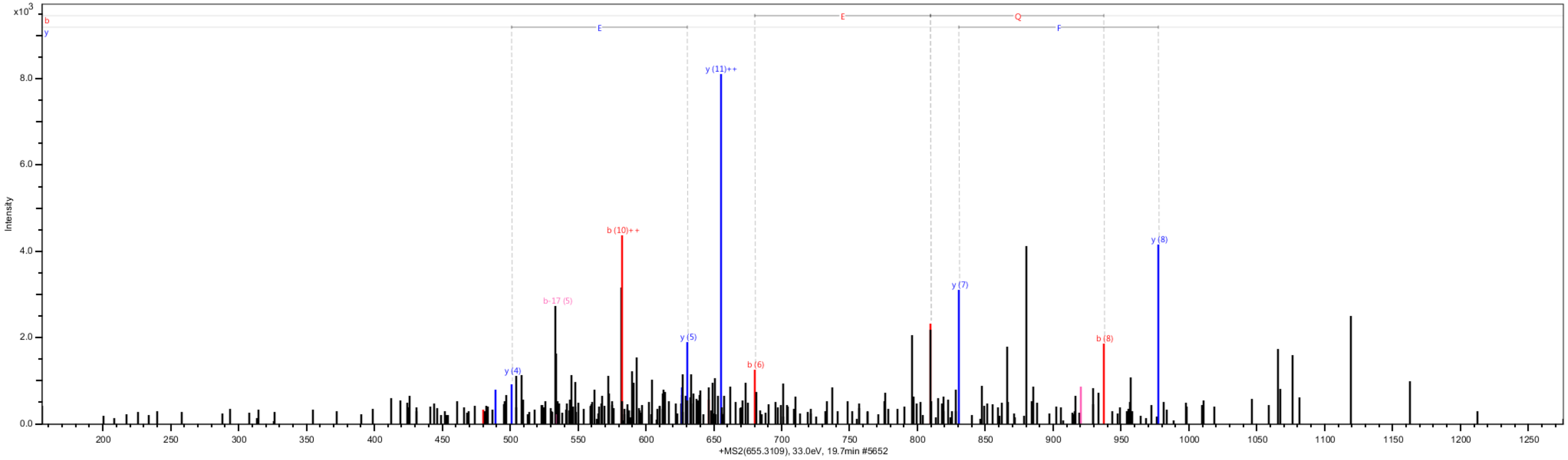

SSX9_HUMAN

**Accession:**	SSX9_HUMAN	**Score:**	18.3
**Database:**	SwissProt	**Seq. Coverage [%]:**	5.9
**MW [kDa] / pI:**	21.5 / 9.2	**No. of Peptides:**	1

Peptide Match

**Sequence:**	R.AGSQIPEKIQK.A	**Score:**	18.3
**Modifications:**			

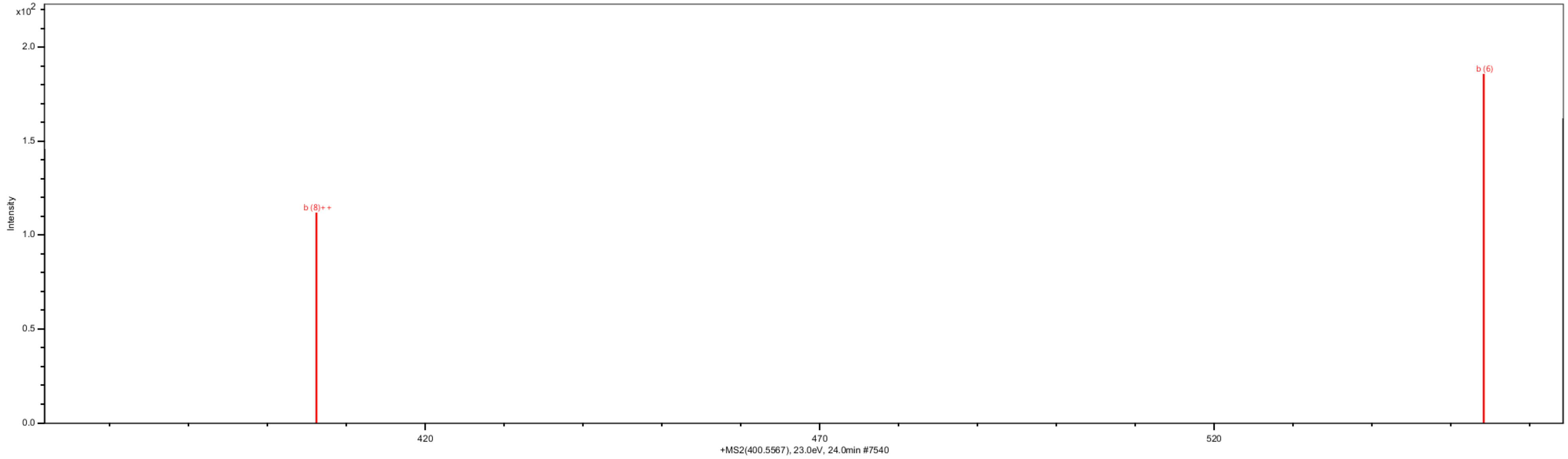

KIF7_HUMAN

**Accession:**	KIF7_HUMAN	**Score:**	18.3
**Database:**	SwissProt	**Seq. Coverage [%]:**	0.5
**MW [kDa] / pI:**	150.5 / 6.4	**No. of Peptides:**	1

Peptide Match

**Sequence:**	K.REAILAK.K	**Score:**	18.3
**Modifications:**			

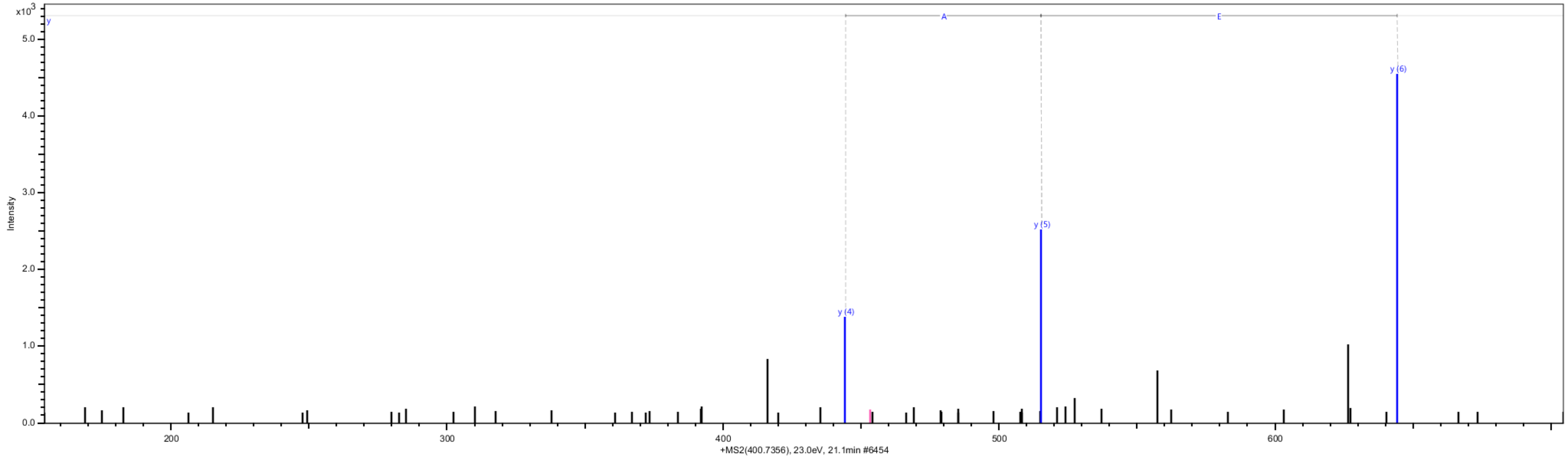

Spectrum Report
Combine Search_2020-10-19 07:53:31

LIPS_HUMAN

Accession: LIPS_HUMAN
Database: SwissProt
MW [kDa] / pI: 116.5 / 6.2

Score: 18.4
Seq. Coverage [%]: 1.5
No. of Peptides: 1

Peptide Match

Sequence: R.LGASSWLNSFLELSGR.K
Score: 18.4
Modifications:

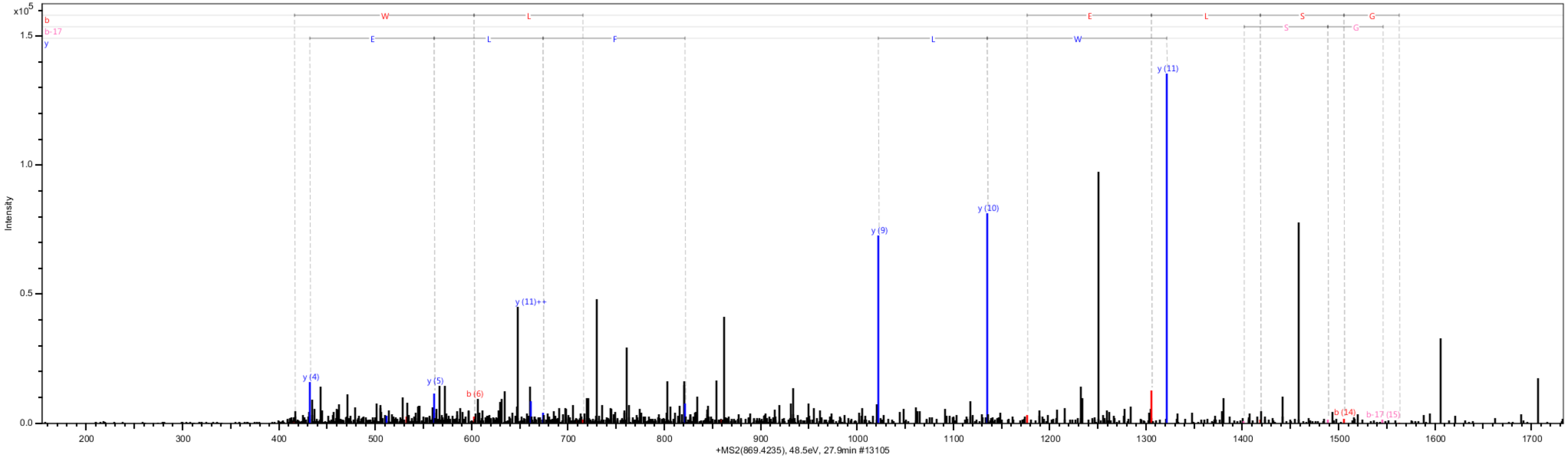

RUFY2_HUMAN

**Accession:**	RUFY2_HUMAN	**Score:**	18.4
**Database:**	SwissProt	**Seq. Coverage [%]:**	1.7
**MW [kDa] / pI:**	70.0 / 5.6	**No. of Peptides:**	1

Peptide Match

Sequence: K.QLVQLETDLK.I **Score:** 18.4

Modifications:

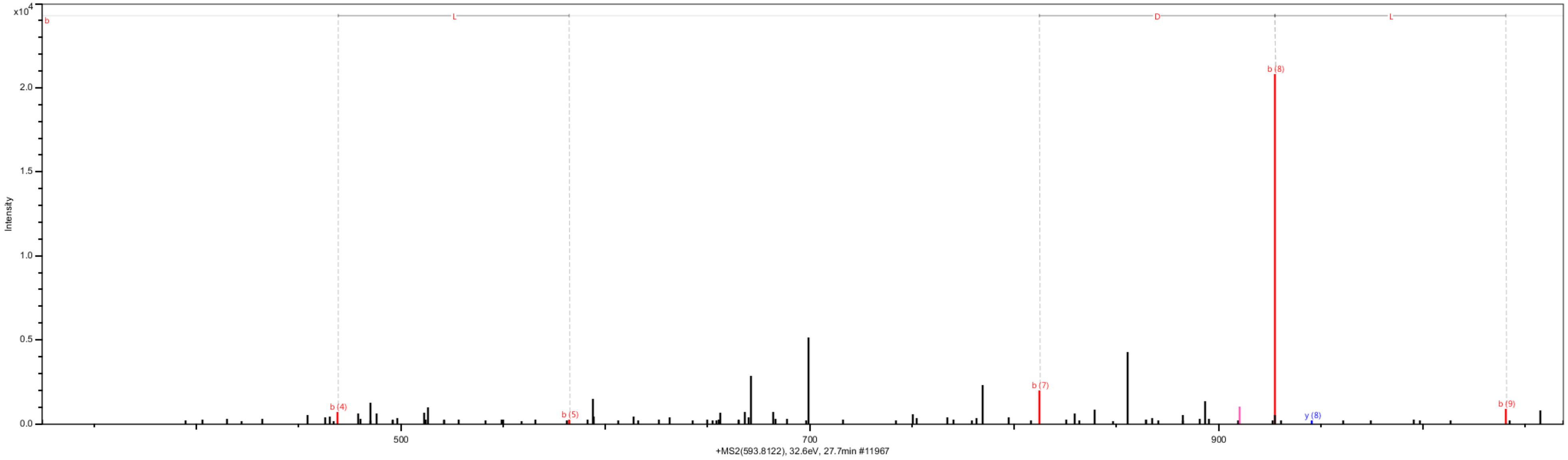

GHDC_HUMAN

**Accession:**	GHDC_HUMAN	**Score:**	18.9
**Database:**	SwissProt	**Seq. Coverage [%]:**	1.5
**MW [kDa] / pI:**	57.5 / 8.1	**No. of Peptides:**	1

Peptide Match

**Sequence:**	R.EALEQGPR.G	**Score:**	18.8
**Modifications:**			

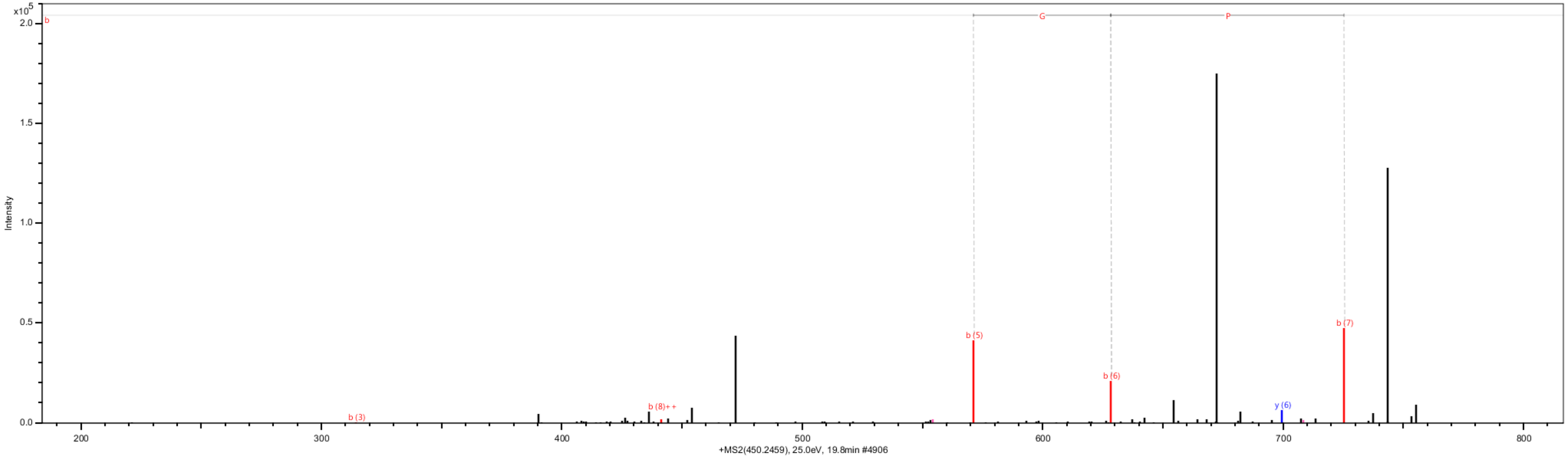

SUCB1_HUMAN

Accession: SUCB1_HUMAN
Database: SwissProt
MW [kDa] / pI: 50.3 / 7.0
Score: 18.9
Seq. Coverage [%]: 1.7
No. of Peptides: 1

Peptide Match

Sequence: R.LVAVATLR.N
Score: 18.9
Modifications:

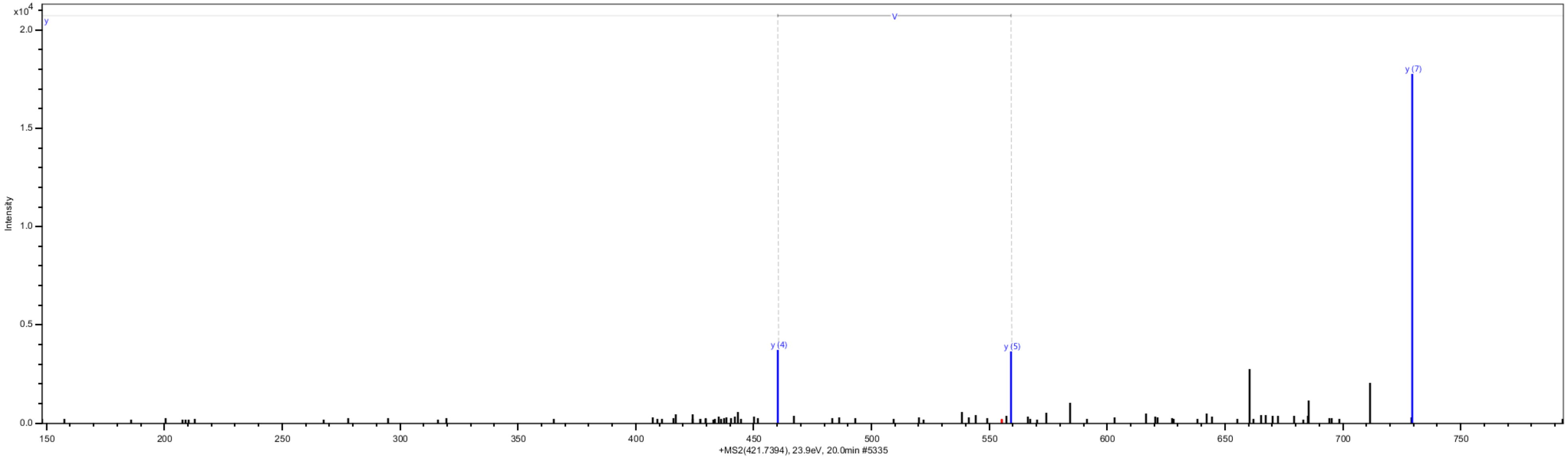

CJ067_HUMAN

Accession: CJ067_HUMAN
Database: SwissProt
MW [kDa] / pI: 63.6 / 8.9
Score: 18.8
Seq. Coverage [%]: 1.6
No. of Peptides: 1

Peptide Match

Sequence: K.EDQAVVEDK.H
Score: 18.8
Modifications:

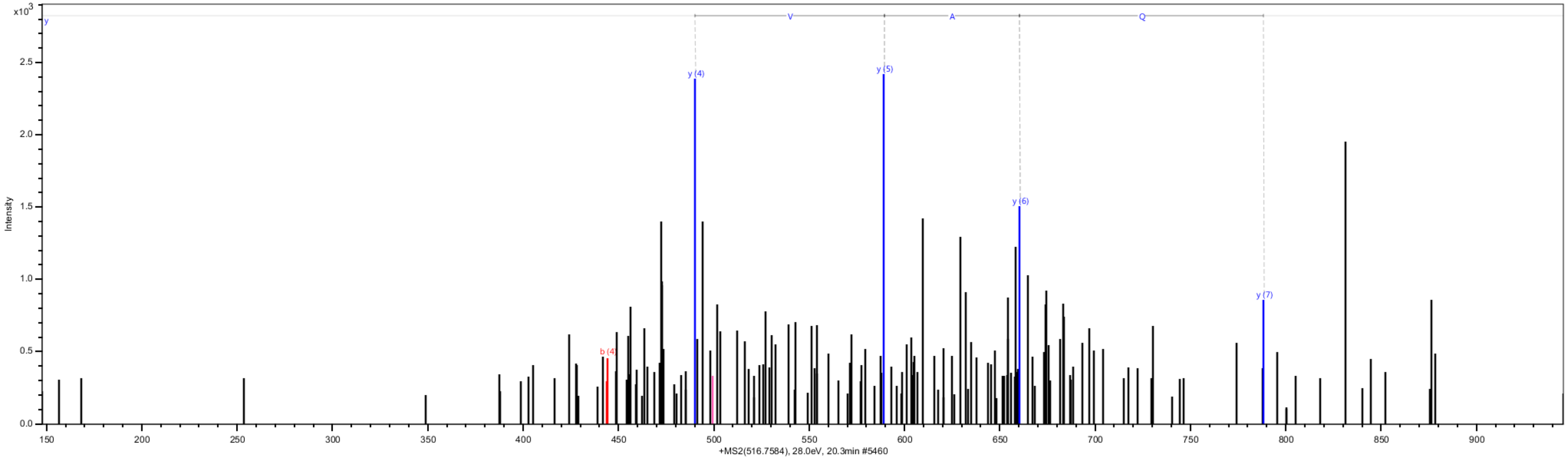

SOGA3_HUMAN

Accession: SOGA3_HUMAN
Database: SwissProt
MW [kDa] / pI: 103.1 / 5.8
Score: 18.8
Seq. Coverage [%]: 1.5
No. of Peptides: 1

Peptide Match

Sequence: K.YRSFYGDLDSPLPK.G
Score: 18.8
Modifications:

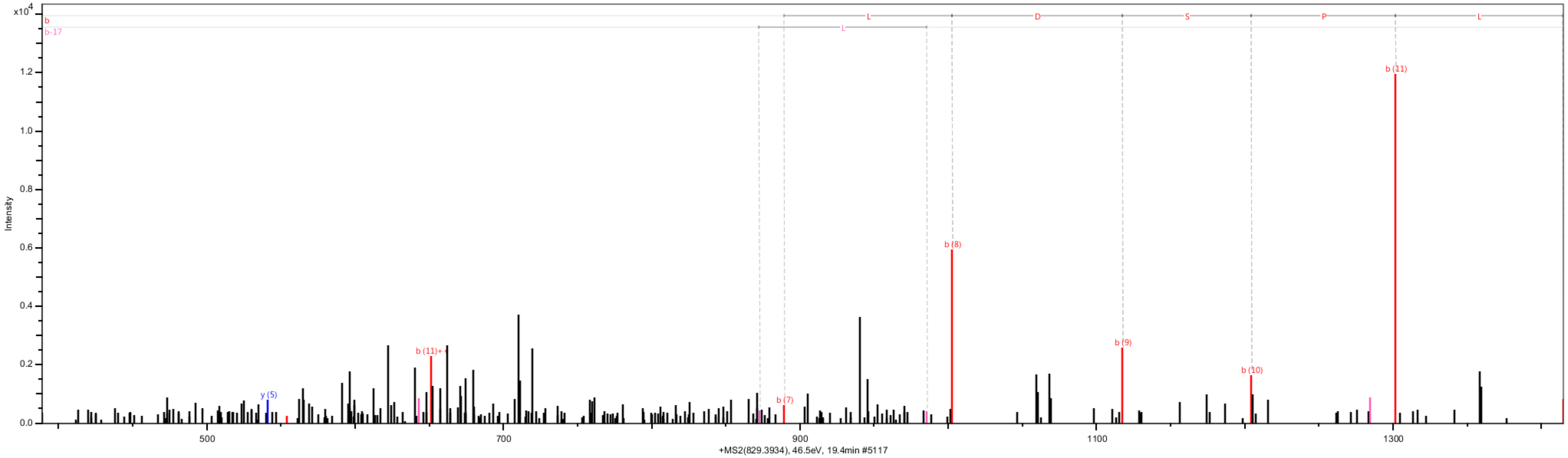

SMC1A_HUMAN

Accession: SMC1A_HUMAN
Database: SwissProt
MW [kDa] / pI: 143.1 / 7.5
Score: 18.9
Seq. Coverage [%]: 0.8
No. of Peptides: 1

Peptide Match

Sequence: K.MQDIKLPLSK.G
Score: 18.9
Modifications:

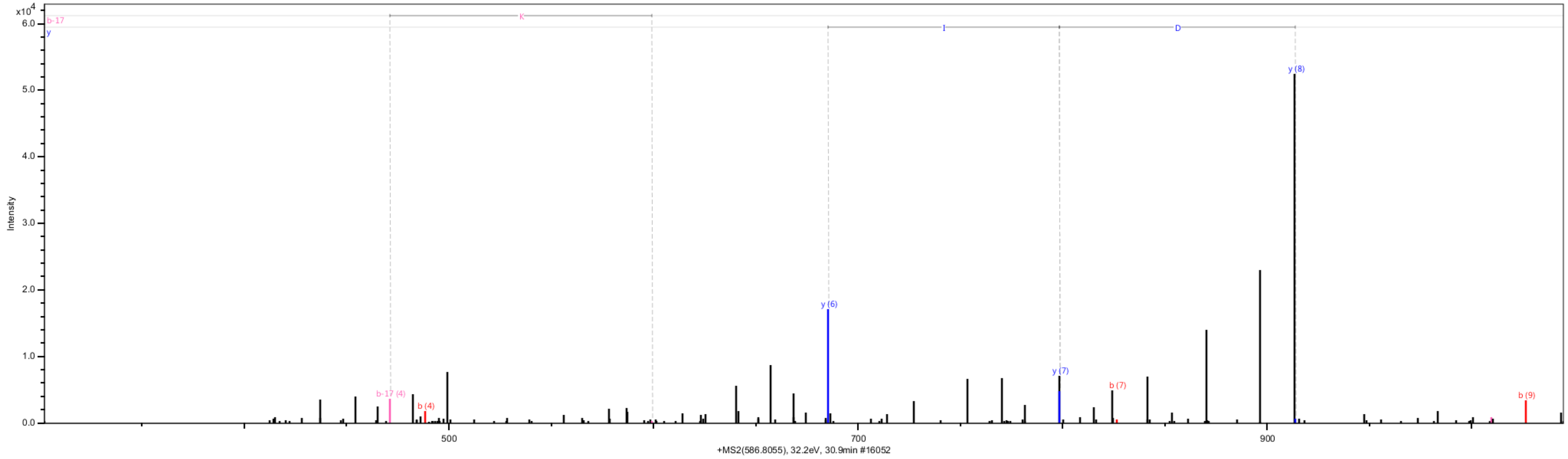

CP072_HUMAN

Accession: CP072_HUMAN
Database: SwissProt
MW [kDa] / pI: 30.9 / 7.8
Score: 18.9
Seq. Coverage [%]: 2.9
No. of Peptides: 1

Peptide Match

Sequence: R.LTVVSPNR.A
Score: 18.9
Modifications:

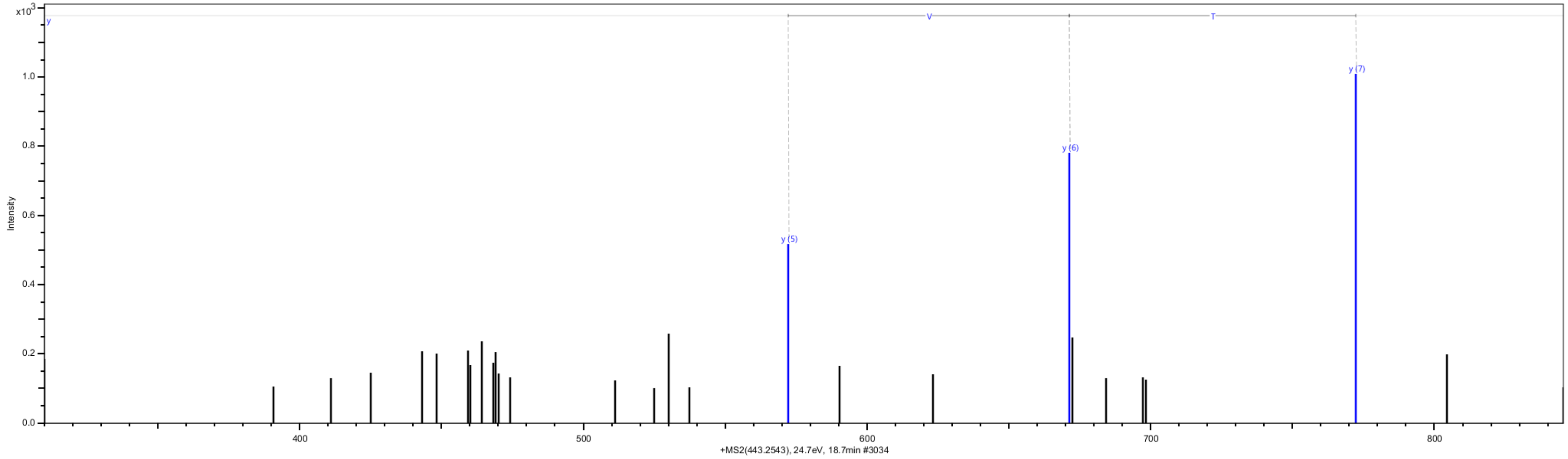

RIC3_HUMAN

**Accession:**	RIC3_HUMAN	**Score:**	18.9
**Database:**	SwissProt	**Seq. Coverage [%]:**	2.2
**MW [kDa] / pI:**	41.1 / 5.0	**No. of Peptides:**	1

Peptide Match

**Sequence:**	K.ETEAAMEK.L	**Score:**	18.9
**Modifications:**	Oxidation: 6		

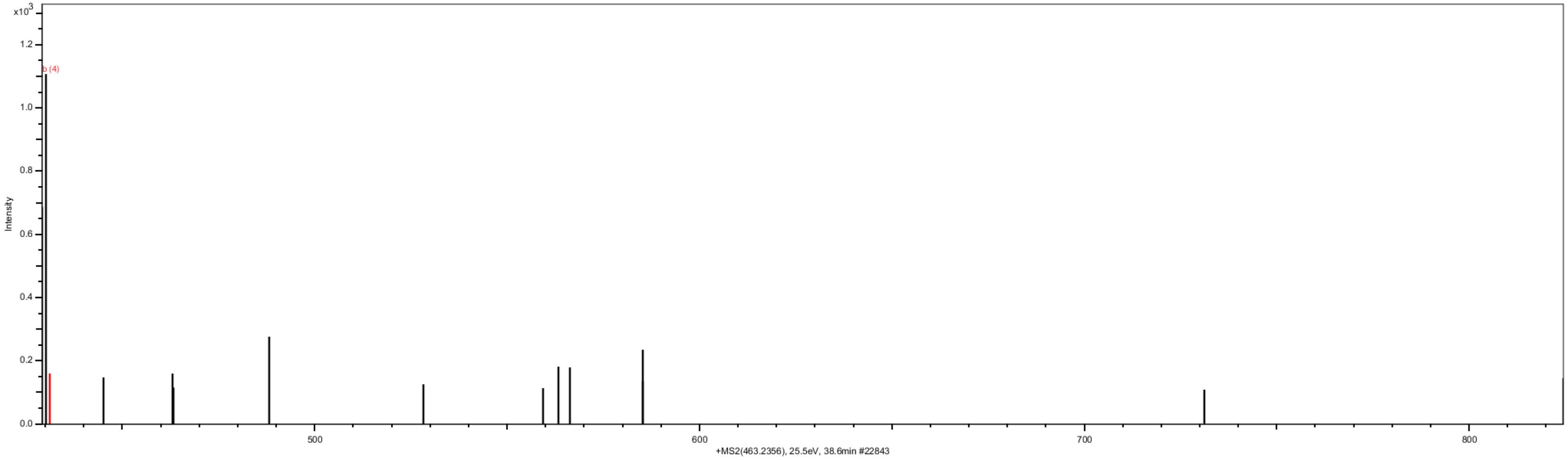

CRK_HUMAN

Accession: CRK_HUMAN
Database: SwissProt
MW [kDa] / pI: 33.8 / 5.4
Score: 18.9
Seq. Coverage [%]: 3.9
No. of Peptides: 1

Peptide Match

Sequence: K.TALALEVGELVK.V
Score: 18.9
Modifications:

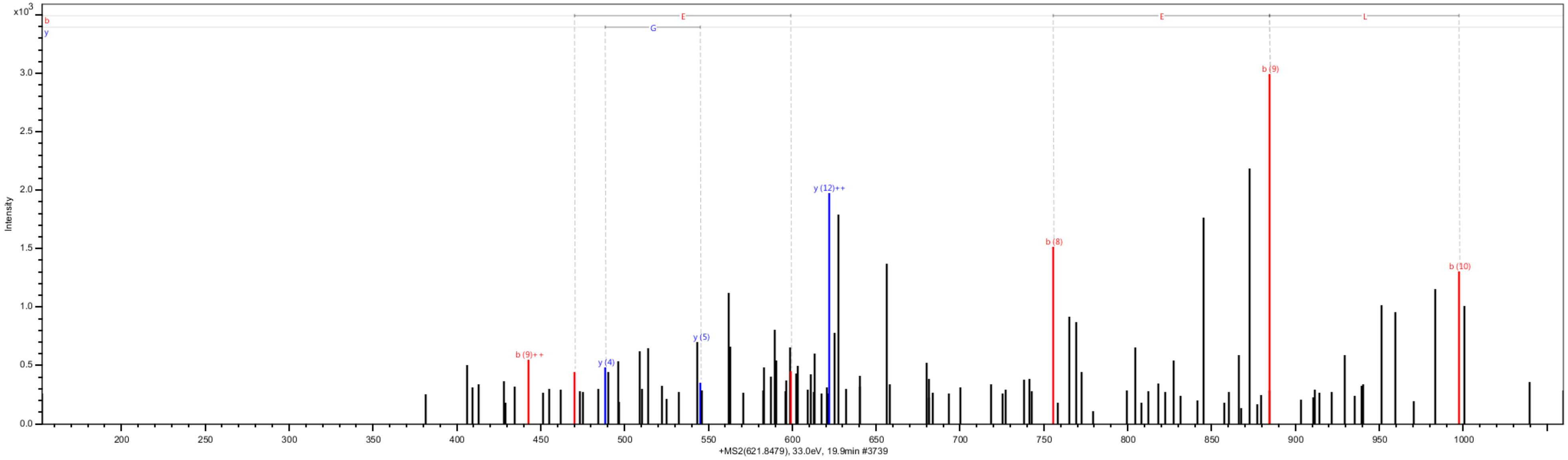

M3K6_HUMAN

**Accession:**	M3K6_HUMAN	**Score:**	19.0
**Database:**	SwissProt	**Seq. Coverage [%]:**	0.7
**MW [kDa] / pI:**	142.5 / 6.7	**No. of Peptides:**	1

Peptide Match

**Sequence:**	R.EPQPGLEPR.E	**Score:**	19
**Modifications:**			

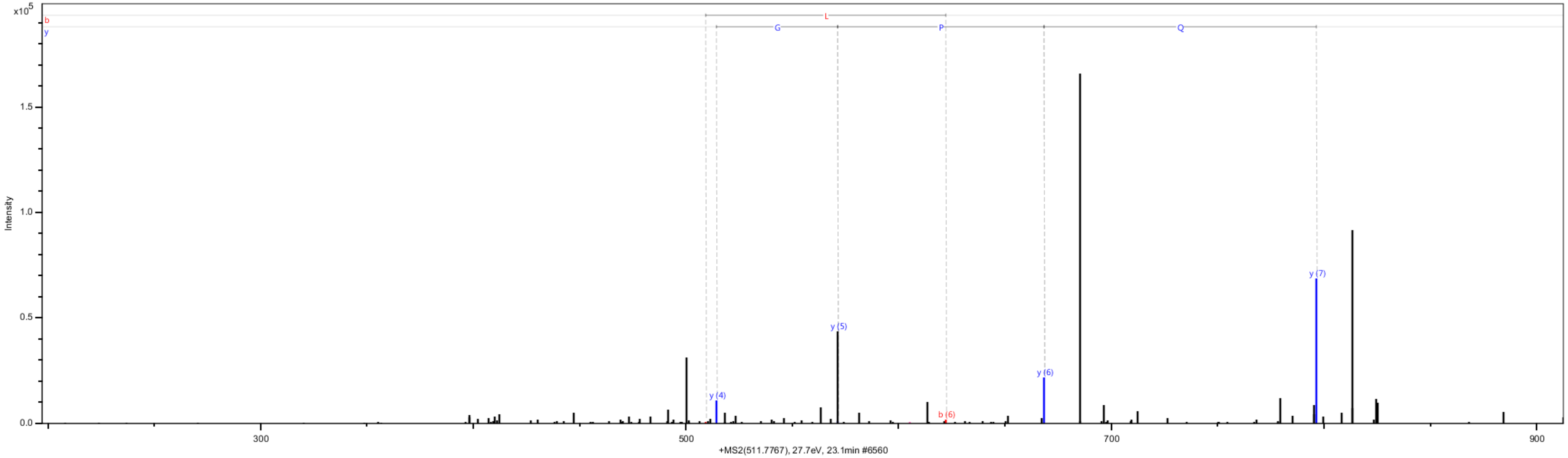

FXDC2_HUMAN

Accession: FXDC2_HUMAN
Database: SwissProt
MW [kDa] / pI: 39.0 / 9.0
Score: 19.0
Seq. Coverage [%]: 3.0
No. of Peptides: 1

Peptide Match

Sequence: R.LLHHPTFYKK.I
Score: 19
Modifications:

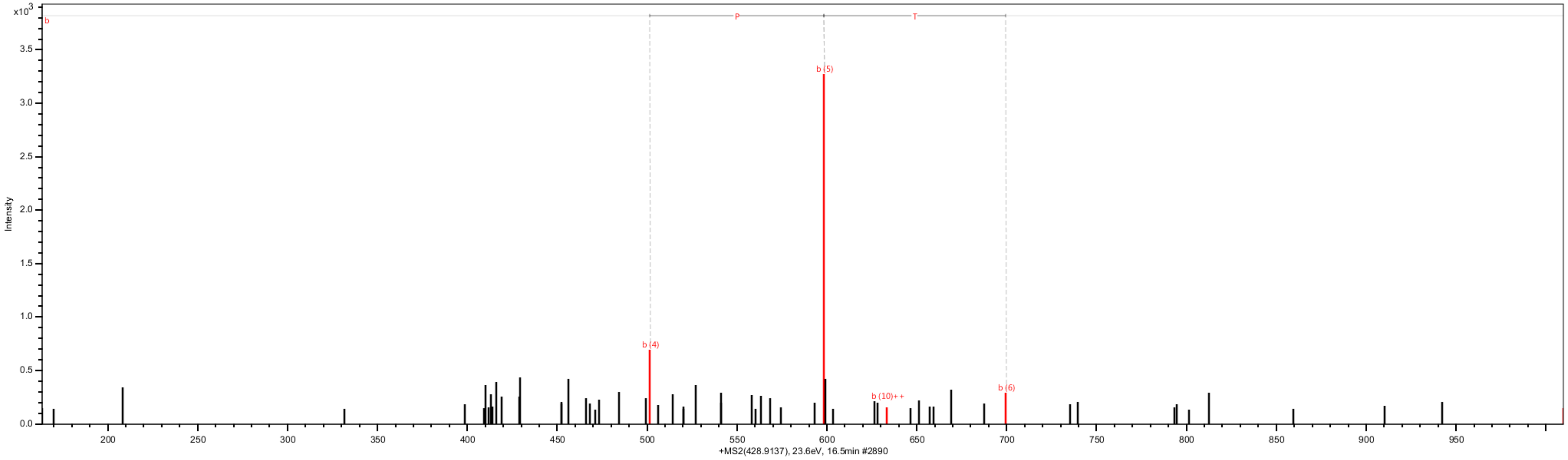

PAR15_HUMAN

Accession: PAR15_HUMAN
Database: SwissProt
MW [kDa] / pI: 74.5 / 9.0
Score: 19.0
Seq. Coverage [%]: 2.1
No. of Peptides: 1

Peptide Match

Sequence: R.IQNAFLWQSYQVKK.R
Score: 19
Modifications:

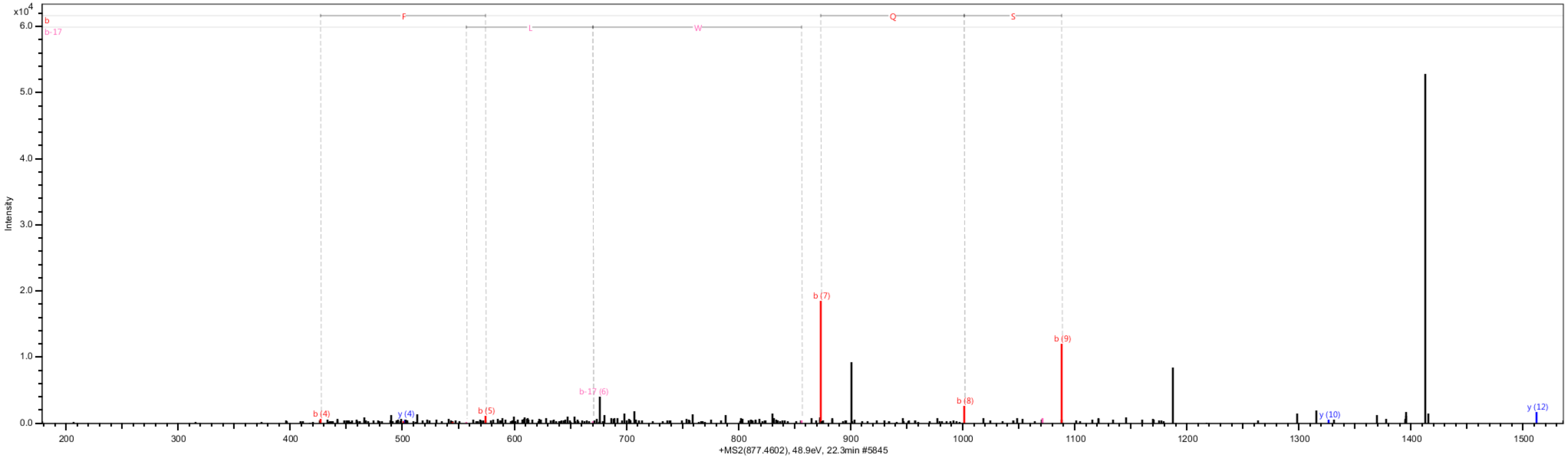

MYO5C_HUMAN

**Accession:**	MYO5C_HUMAN	**Score:**	19.0
**Database:**	SwissProt	**Seq. Coverage [%]:**	0.5
**MW [kDa] / pI:**	202.7 / 7.7	**No. of Peptides:**	1

Peptide Match

**Sequence:**	K.LPFEFDSK.R	**Score:**	19
**Modifications:**			

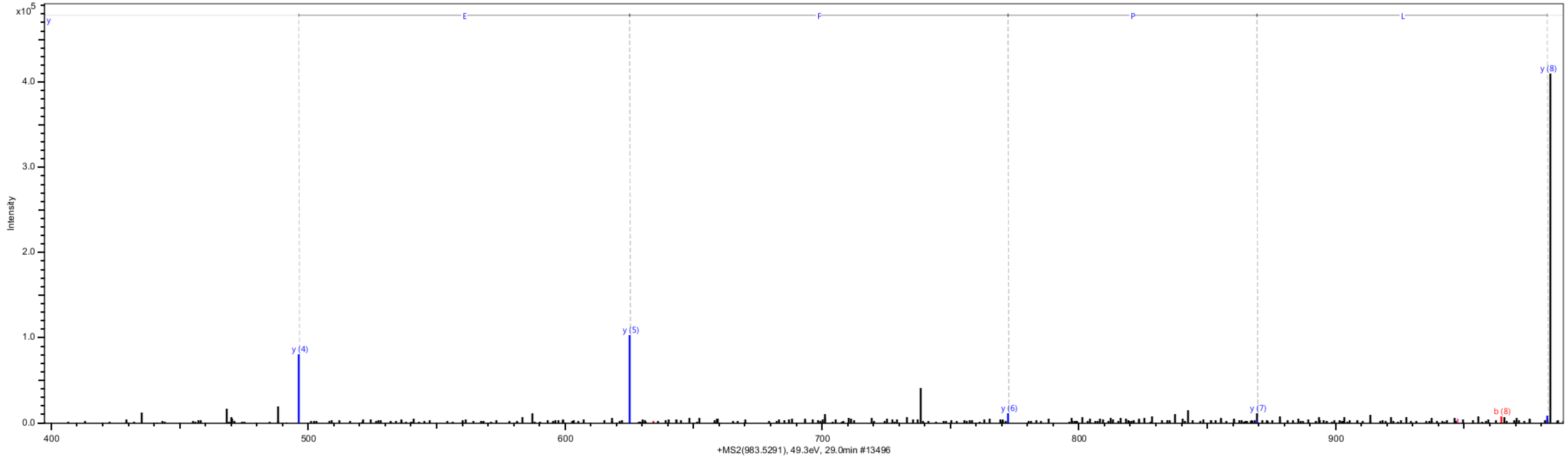

ANR39_HUMAN

**Accession:**	ANR39_HUMAN	**Score:**	17.5
**Database:**	SwissProt	**Seq. Coverage [%]:**	7.1
**MW [kDa] / pI:**	19.6 / 6.4	**No. of Peptides:**	1

Peptide Match

**Sequence:**	R.LACDLLPCNSDLR.D	**Score:**	17.5
**Modifications:**	Carbamidomethyl: 3, 8		

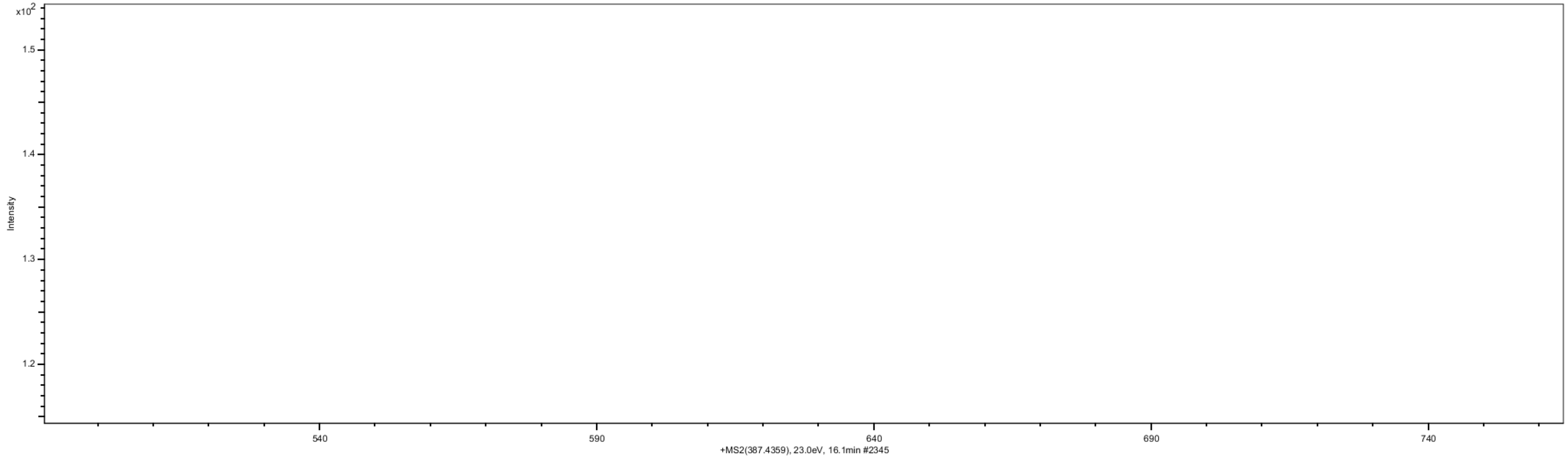

RL3_HUMAN

Accession: RL3_HUMAN
Database: SwissProt
MW [kDa] / pI: 46.1 / 10.2
Score: 17.5
Seq. Coverage [%]: 2.7
No. of Peptides: 1

Peptide Match

Sequence: R.VAFSVARAGQK.G
Score: 17.5
Modifications:

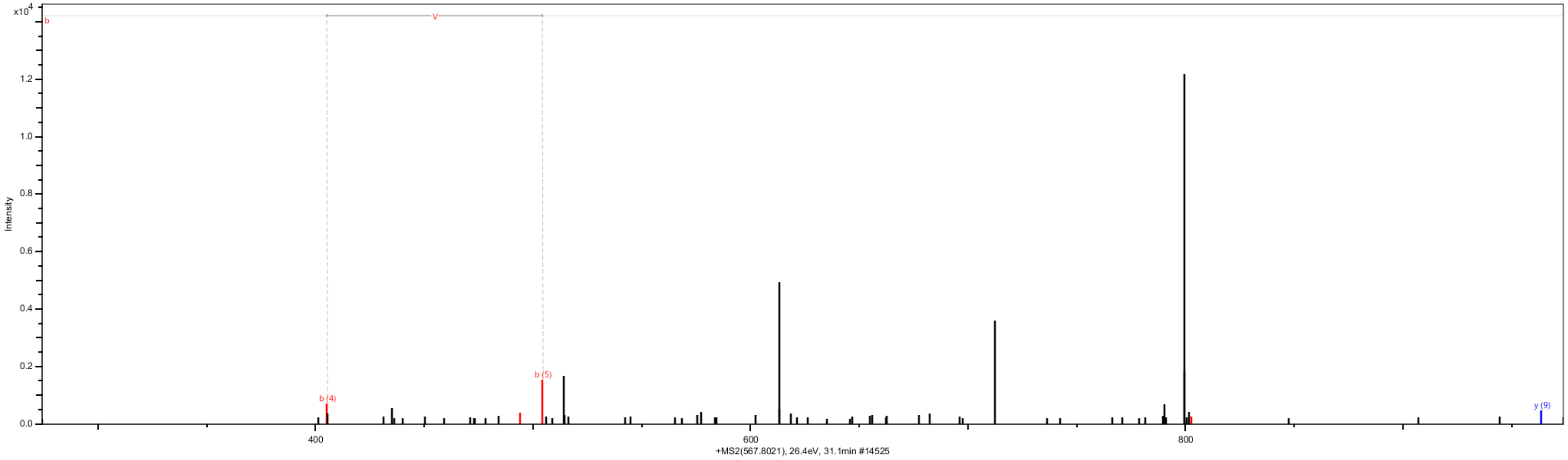

PUS10_HUMAN

Accession: PUS10_HUMAN
Database: SwissProt
MW [kDa] / pI: 60.2 / 6.1
Score: 17.6
Seq. Coverage [%]: 2.6
No. of Peptides: 1

Peptide Match

Sequence: K.TYSALIWTNKAIQK.K
Score: 17.6
Modifications:

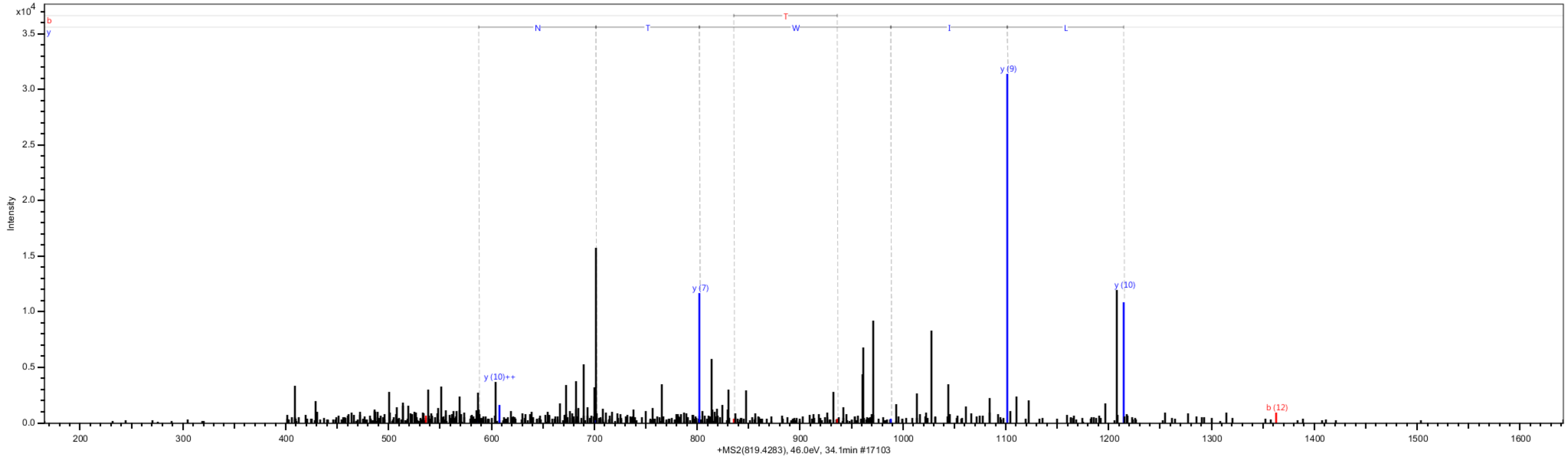

DDX6L_HUMAN

Accession: DDX6L_HUMAN
Database: SwissProt
MW [kDa] / pI: 197.5 / 8.6
Score: 17.6
Seq. Coverage [%]: 0.6
No. of Peptides: 1

Peptide Match

Sequence: K.DLSIAVQMMKR.I
Score: 17.6
Modifications: Oxidation: 8

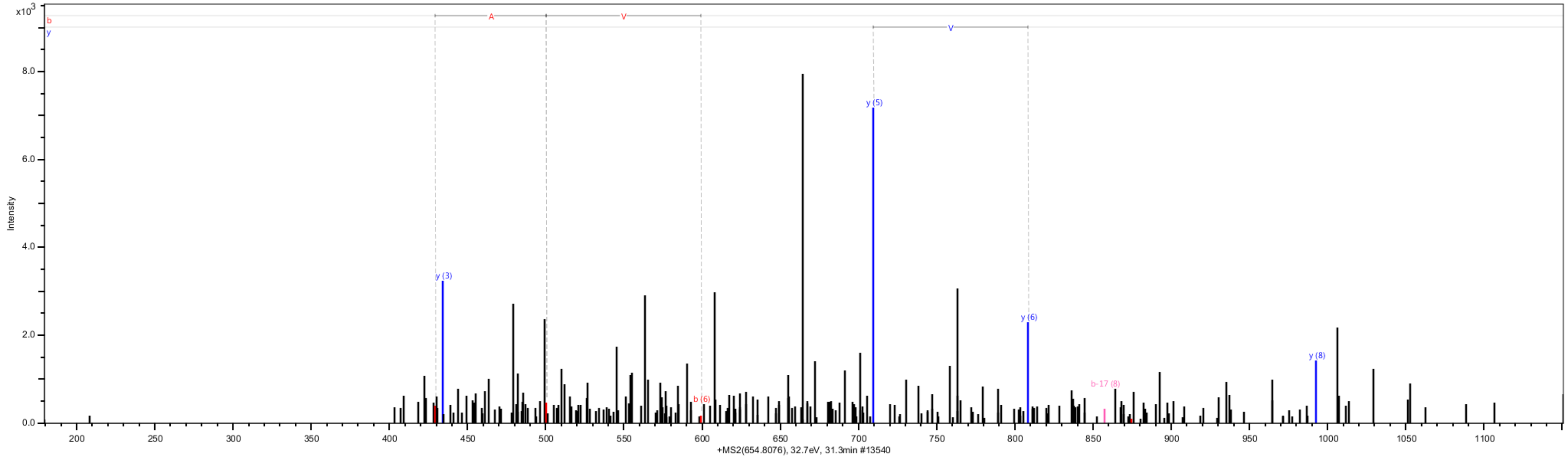

HXA10_HUMAN

Accession: HXA10_HUMAN
Database: SwissProt
MW [kDa] / pI: 42.4 / 8.6

Score: 17.5
Seq. Coverage [%]: 2.2
No. of Peptides: 1

Peptide Match

Sequence: R.LSQAYGTAK.G
Score: 17.5
Modifications:

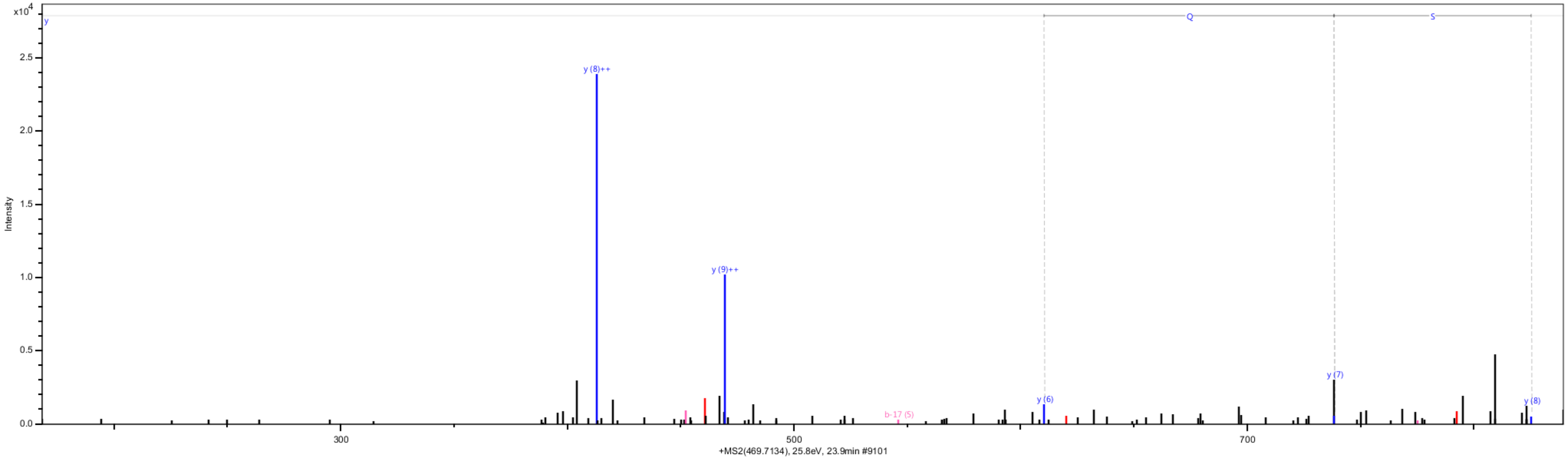

OSBL1_HUMAN

Accession: OSBL1_HUMAN
Database: SwissProt
MW [kDa] / pI: 108.4 / 6.0
Score: 17.5
Seq. Coverage [%]: 1.1
No. of Peptides: 1

Peptide Match

Sequence: K.STDSCLFFIK.C
Modifications: Carbamidomethyl: 5
Score: 17.5

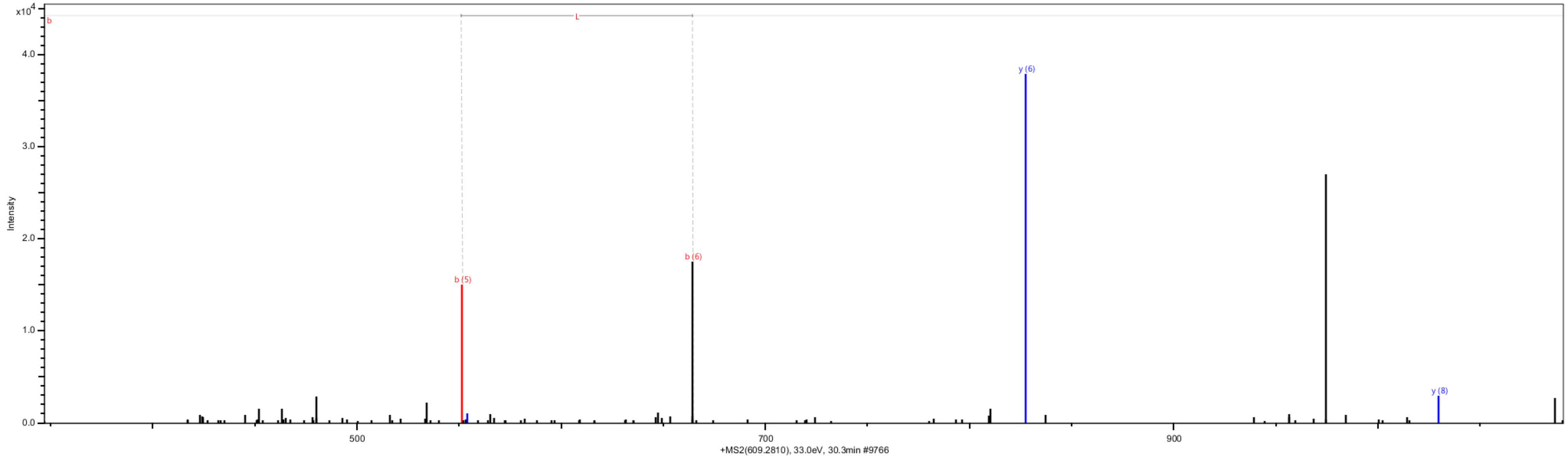

MYBPP_HUMAN

Accession: MYBPP_HUMAN
Database: SwissProt
MW [kDa] / pI: 108.1 / 7.1
Score: 17.5
Seq. Coverage [%]: 1.1
No. of Peptides: 1

Peptide Match

Sequence: K.ASVNAELLPR.F
Score: 17.5
Modifications:

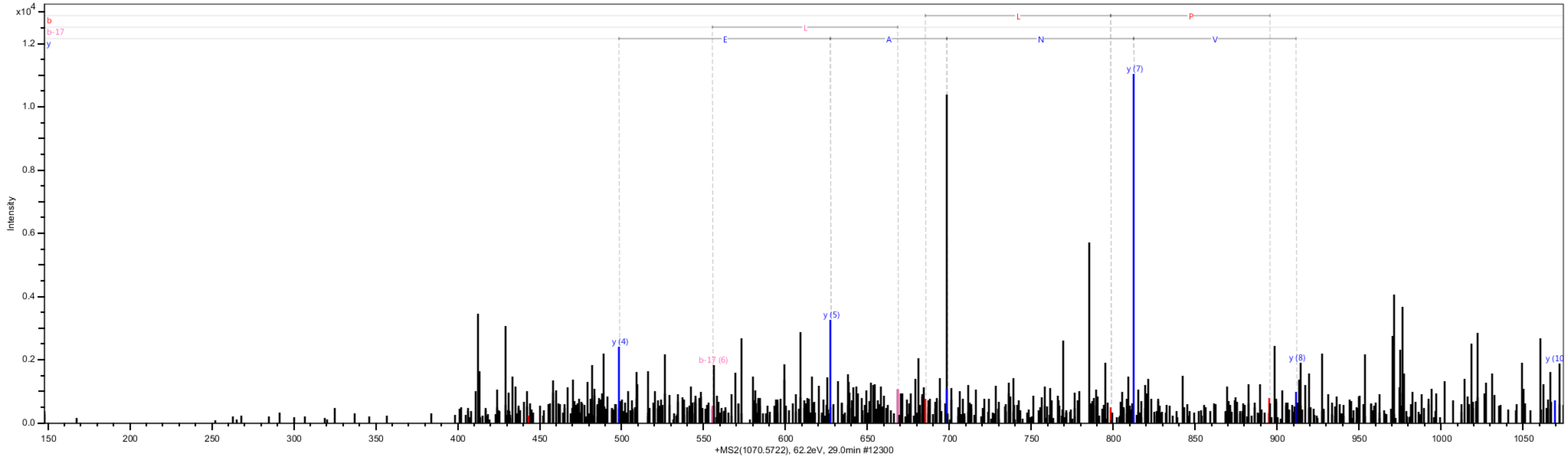

SPT22_HUMAN

Accession: SPT22_HUMAN
Database: SwissProt
MW [kDa] / pI: 41.3 / 9.4
Score: 17.5
Seq. Coverage [%]: 1.9
No. of Peptides: 1

Peptide Match

Sequence: K.ISGCTMR.G
Score: 17.5
Modifications: Carbamidomethyl: 4

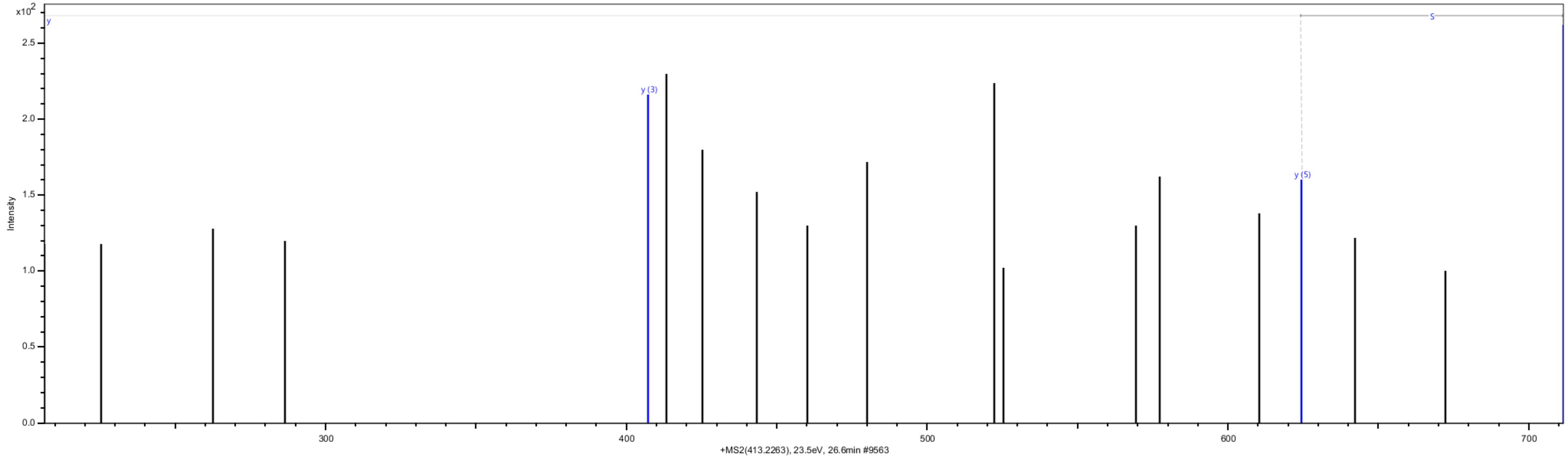

ARF5_HUMAN

**Accession:**	ARF5_HUMAN	**Score:**	17.6
**Database:**	SwissProt	**Seq. Coverage [%]:**	8.3
**MW [kDa] / pI:**	20.5 / 6.3	**No. of Peptides:**	1

Peptide Match

**Sequence:**	K.QDMPNAMPVSELTDK.L	**Score:**	17.6
**Modifications:**	Oxidation: 3, 7		

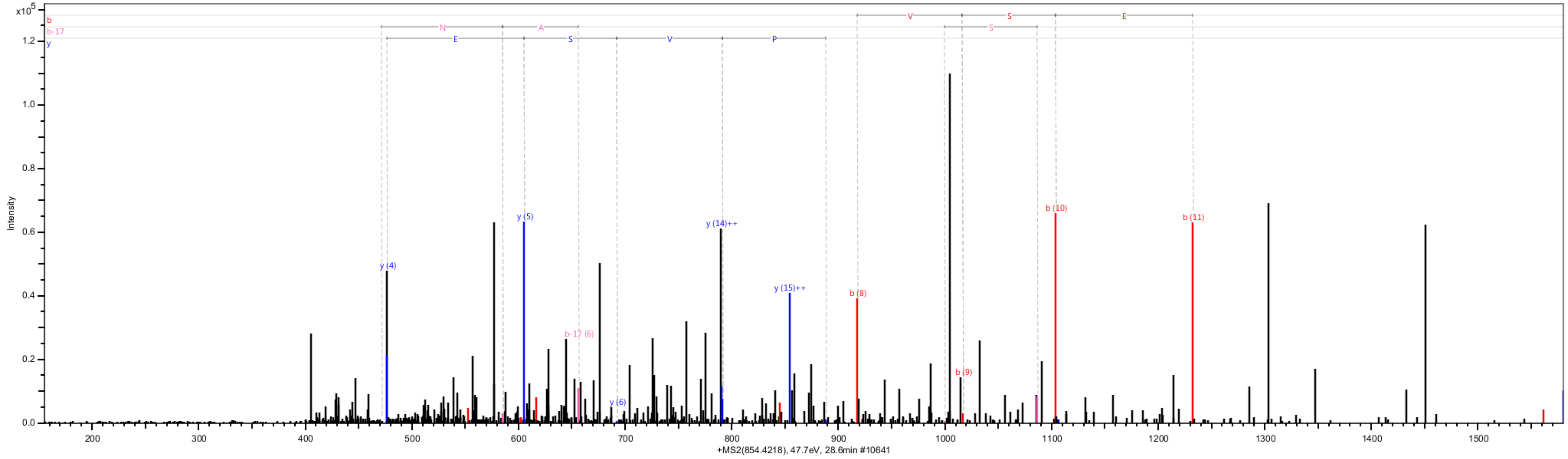

OTC_HUMAN

Accession: OTC_HUMAN
Database: SwissProt
MW [kDa] / pI: 39.9 / 8.7
Score: 17.6
Seq. Coverage [%]: 2.5
No. of Peptides: 1

Peptide Match

Sequence: K.QSDLDTLAK.E
Score: 17.6
Modifications:

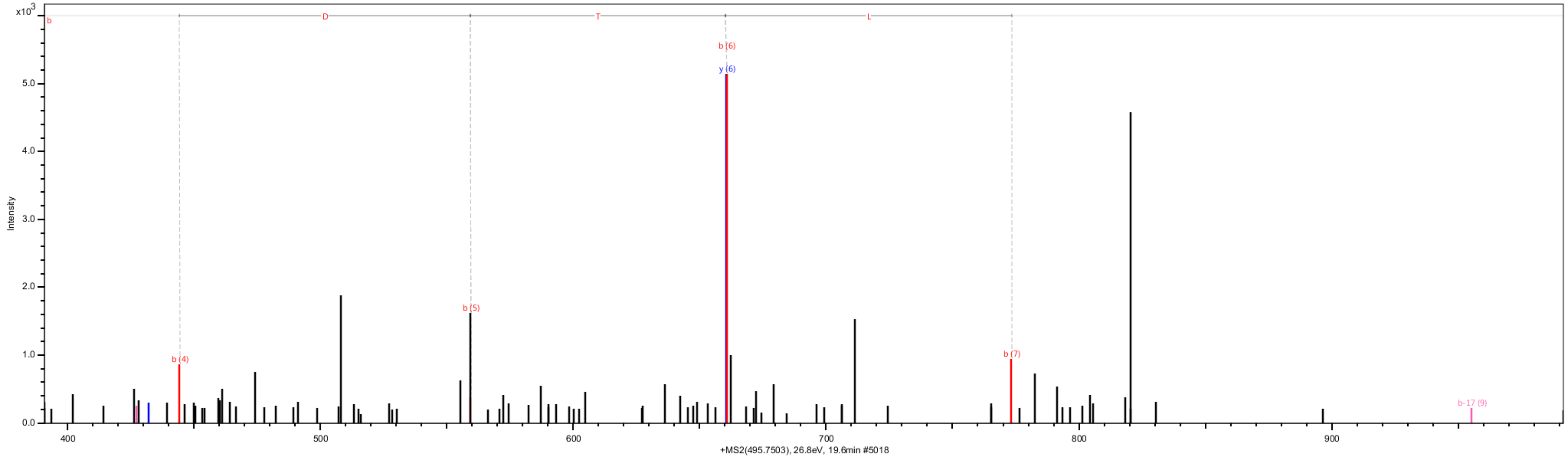

COE2_HUMAN

Accession: COE2_HUMAN
Database: SwissProt
MW [kDa] / pI: 62.6 / 9.2
Score: 17.6
Seq. Coverage [%]: 3.0
No. of Peptides: 1

Peptide Match

Sequence: R.FIYTALNEPTIDYGFQR.L
Score: 17.6
Modifications:

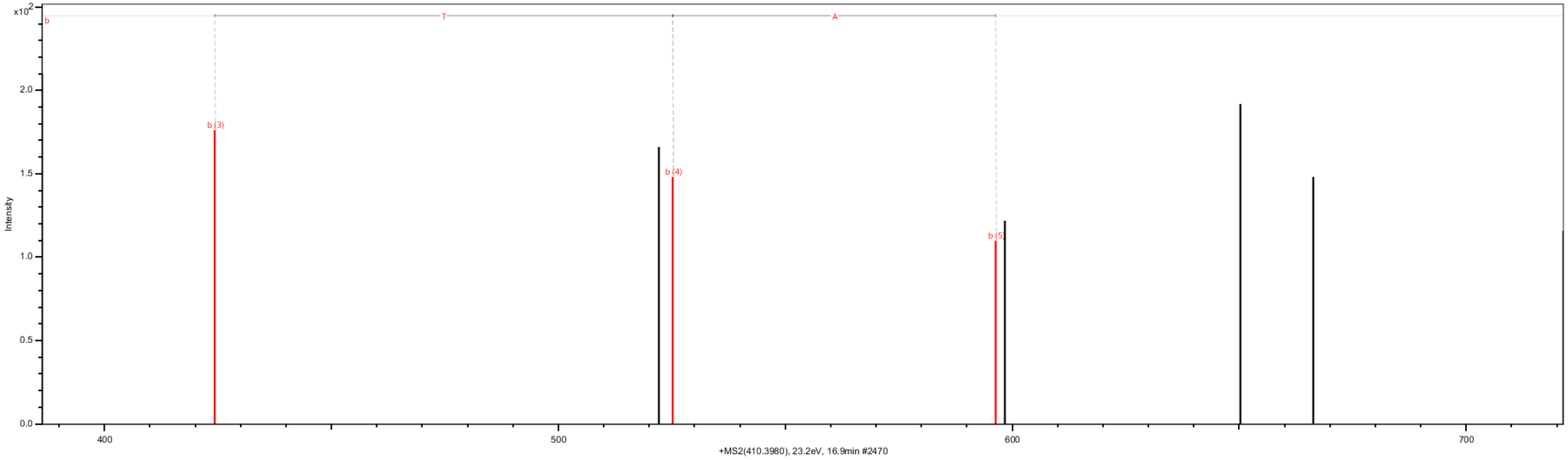

EIF3G_HUMAN

Accession: EIF3G_HUMAN
Database: SwissProt
MW [kDa] / pI: 35.6 / 5.9
Score: 17.6
Seq. Coverage [%]: 2.5
No. of Peptides: 1

Peptide Match

Sequence: K.IVSCRICK.G
Score: 17.6
Modifications: Carbamidomethyl: 4, 7

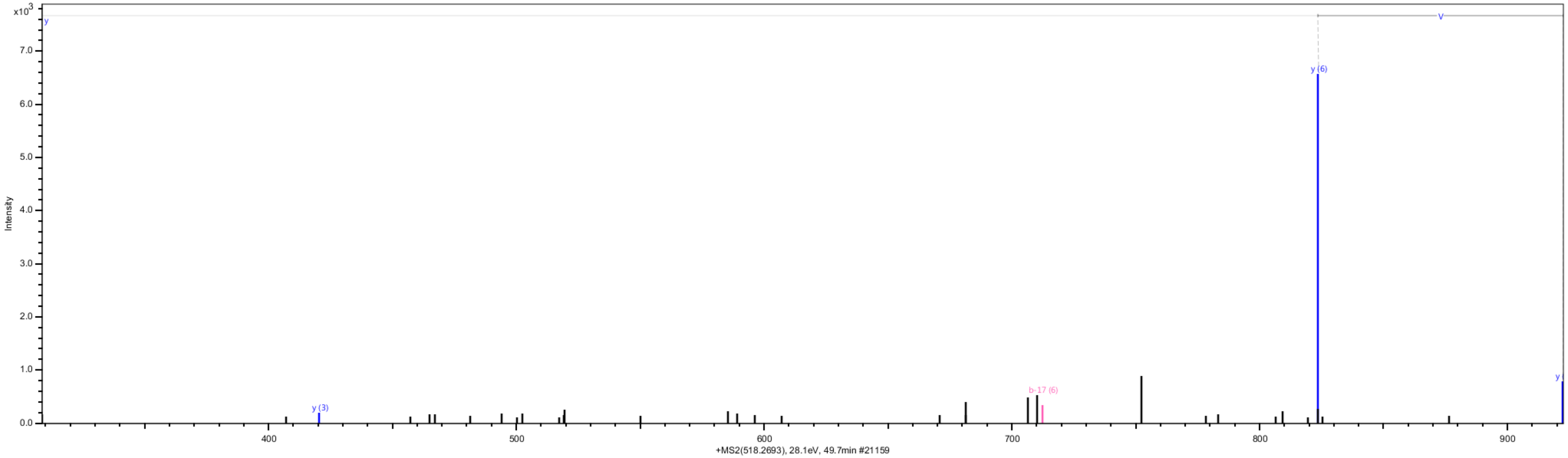

TXND2_HUMAN

Accession: TXND2_HUMAN
Database: SwissProt
MW [kDa] / pI: 60.4 / 4.8
Score: 17.6
Seq. Coverage [%]: 2.4
No. of Peptides: 1

Peptide Match

Sequence: -.MDVDKELGMESVK.A
Score: 17.6
Modifications:

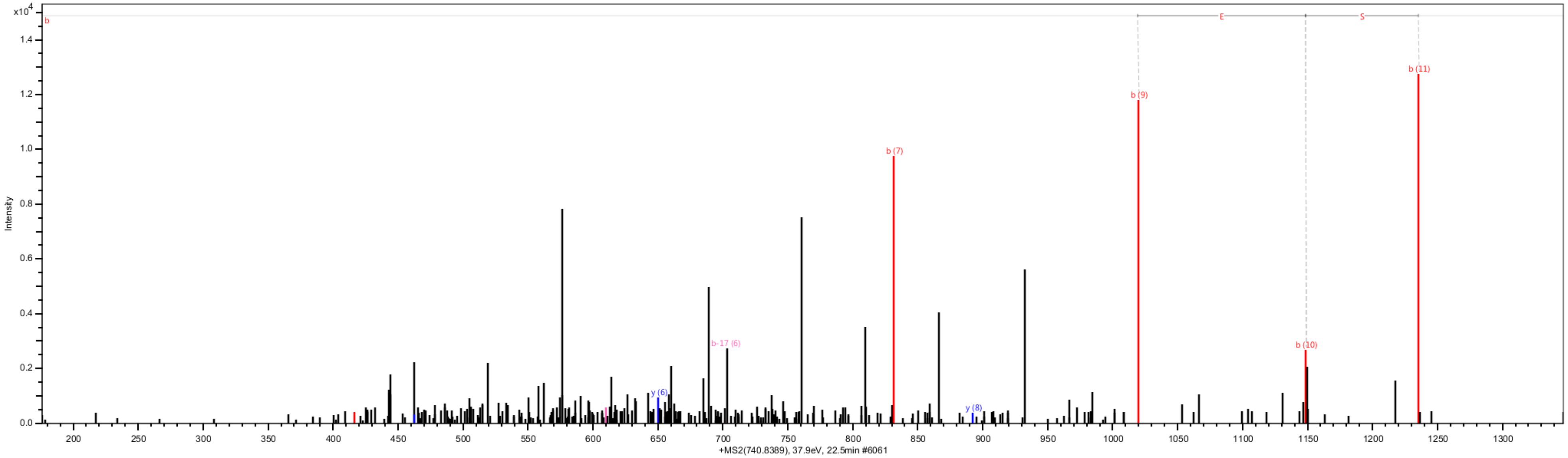

HHIP_HUMAN

Accession: HHIP_HUMAN
Database: SwissProt
MW [kDa] / pI: 78.8 / 8.2
Score: 17.6
Seq. Coverage [%]: 2.0
No. of Peptides: 1

Peptide Match

Sequence: K.IVDPKRPLMPEECR.A
Score: 17.6
Modifications: Carbamidomethyl: 13

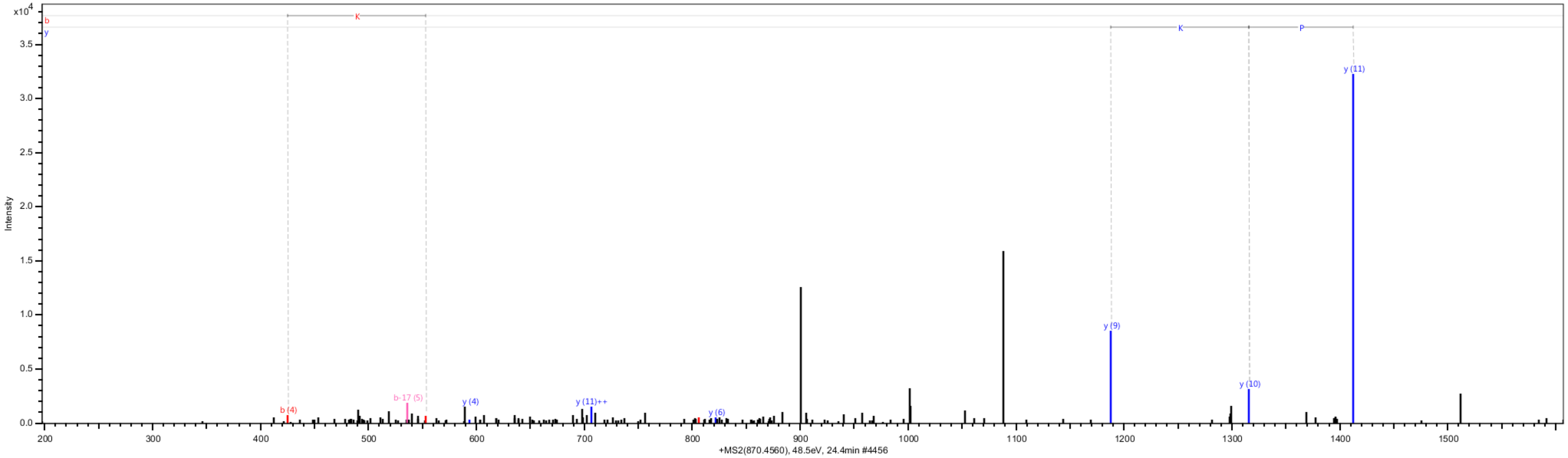

EEA1_HUMAN

Accession: EEA1_HUMAN
Database: SwissProt
MW [kDa] / pI: 162.4 / 5.5

Score: 17.6
Seq. Coverage [%]: 0.6
No. of Peptides: 1

Peptide Match

Sequence: R.SLREAAEQK.V
Score: 17.6
Modifications:

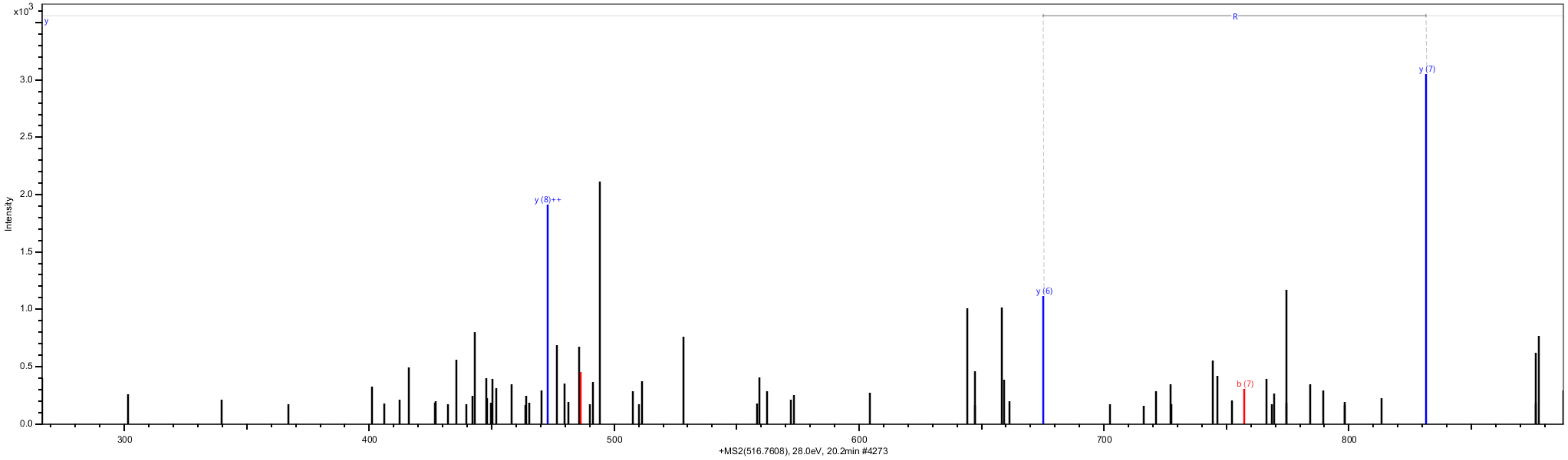

S22A3_HUMAN

**Accession:**	S22A3_HUMAN	**Score:**	17.3
**Database:**	SwissProt	**Seq. Coverage [%]:**	1.4
**MW [kDa] / pI:**	61.2 / 8.9	**No. of Peptides:**	1

Peptide Match

**Sequence:**	R.SAPLVPCR.G	**Score:**	17.3
**Modifications:**	Carbamidomethyl: 7		

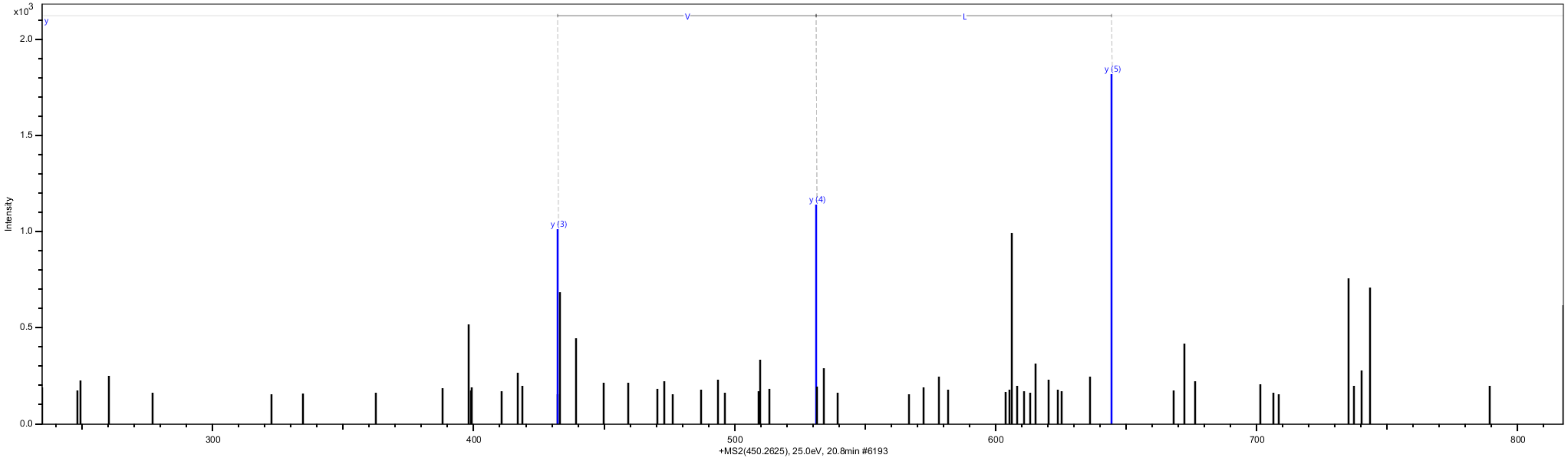

KAAG1_HUMAN

**Accession:**	KAAG1_HUMAN	**Score:**	17.3
**Database:**	SwissProt	**Seq. Coverage [%]:**	16.7
**MW [kDa] / pI:**	9.0 / 9.5	**No. of Peptides:**	1

Peptide Match

Sequence: R.QVAGPGAAAAHLPR.W **Score:** 17.3

Modifications:

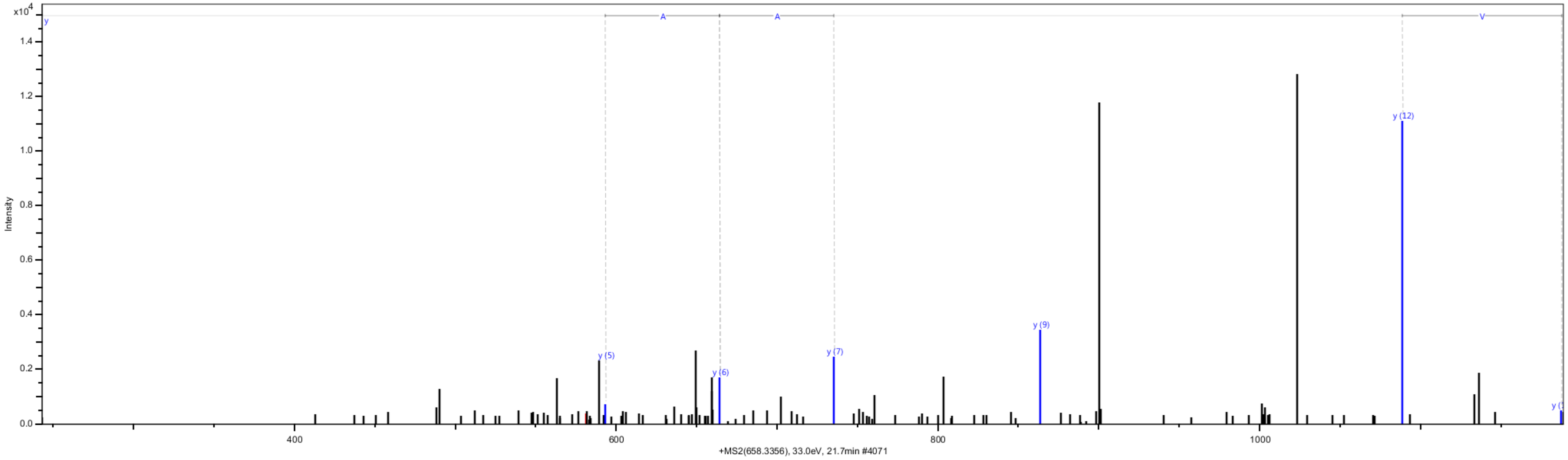

MEIOB_HUMAN

**Accession:**	MEIOB_HUMAN	**Score:**	17.3
**Database:**	SwissProt	**Seq. Coverage [%]:**	1.6
**MW [kDa] / pI:**	49.3 / 6.0	**No. of Peptides:**	1

Peptide Match

**Sequence:**	K.ALKNEGK.A	**Score:**	17.3
**Modifications:**			

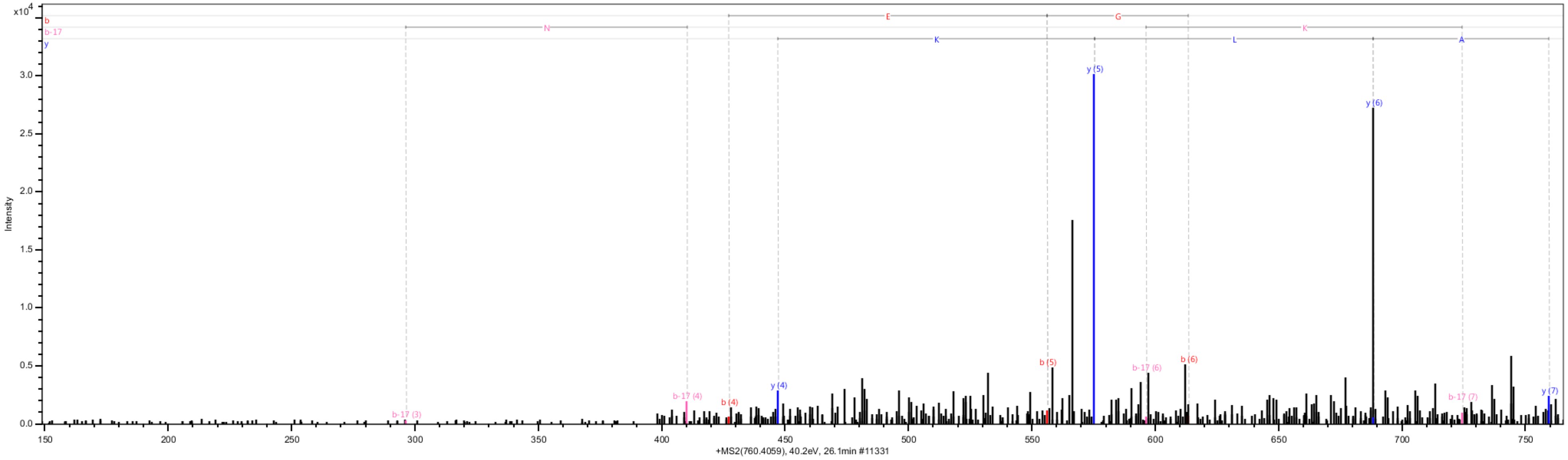

C4BPA_HUMAN

Accession: C4BPA_HUMAN
Database: SwissProt
MW [kDa] / pI: 67.0 / 7.2
Score: 17.4
Seq. Coverage [%]: 2.3
No. of Peptides: 1

Peptide Match

Sequence: R.FSAICQGDGTWSPR.T
Score: 17.4
Modifications: Carbamidomethyl: 5

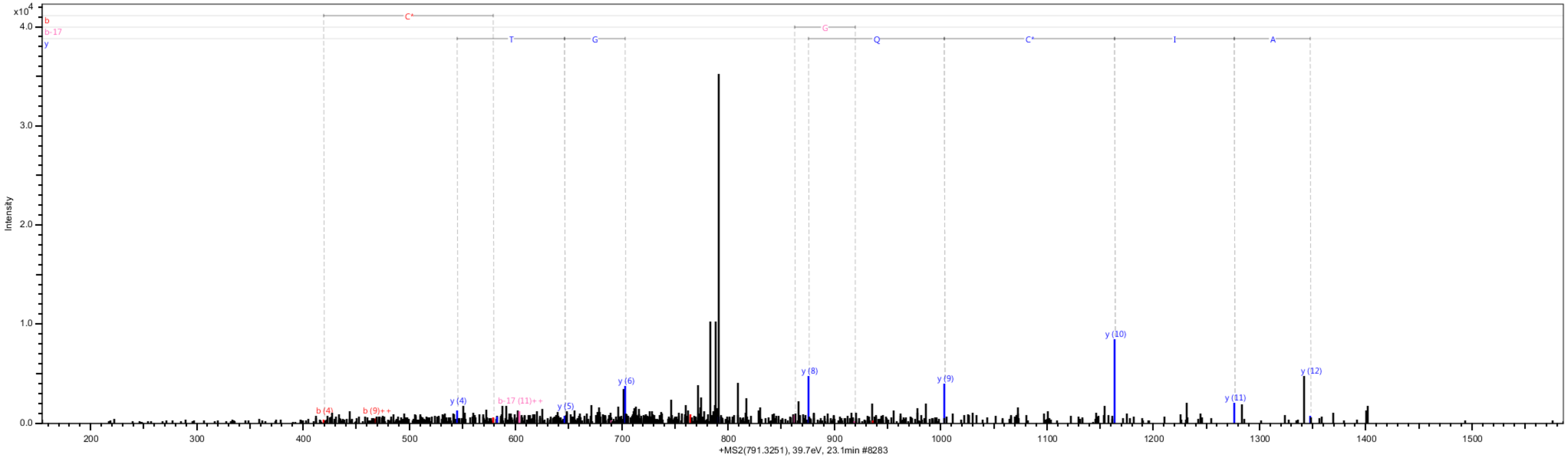

M3K11_HUMAN

Accession: M3K11_HUMAN
Database: SwissProt
MW [kDa] / pI: 92.6 / 8.4
Score: 17.3
Seq. Coverage [%]: 1.8
No. of Peptides: 1

Peptide Match

Sequence: R.GIDCLAVAYGVAVNK.L
Score: 17.3
Modifications: Carbamidomethyl: 4

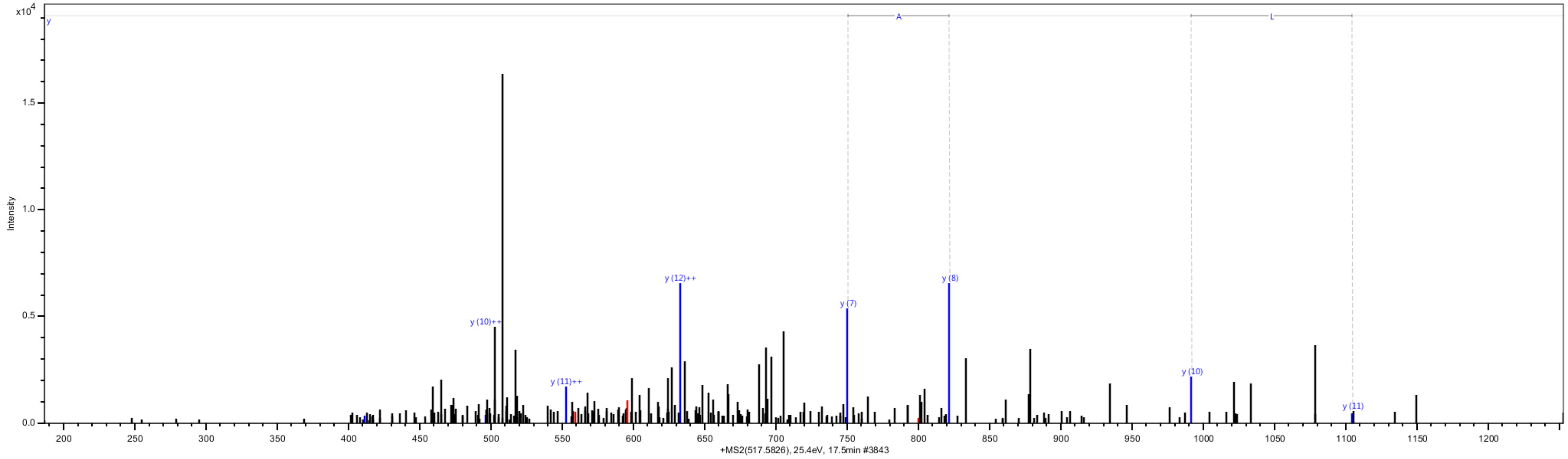

KCNJ1_HUMAN

Accession: KCNJ1_HUMAN
Database: SwissProt
MW [kDa] / pI: 44.8 / 9.0

Score: 17.3
Seq. Coverage [%]: 2.0
No. of Peptides: 1

Peptide Match

Sequence: R.VLTESMFK.H
Modifications: Oxidation: 6

Score: 17.3

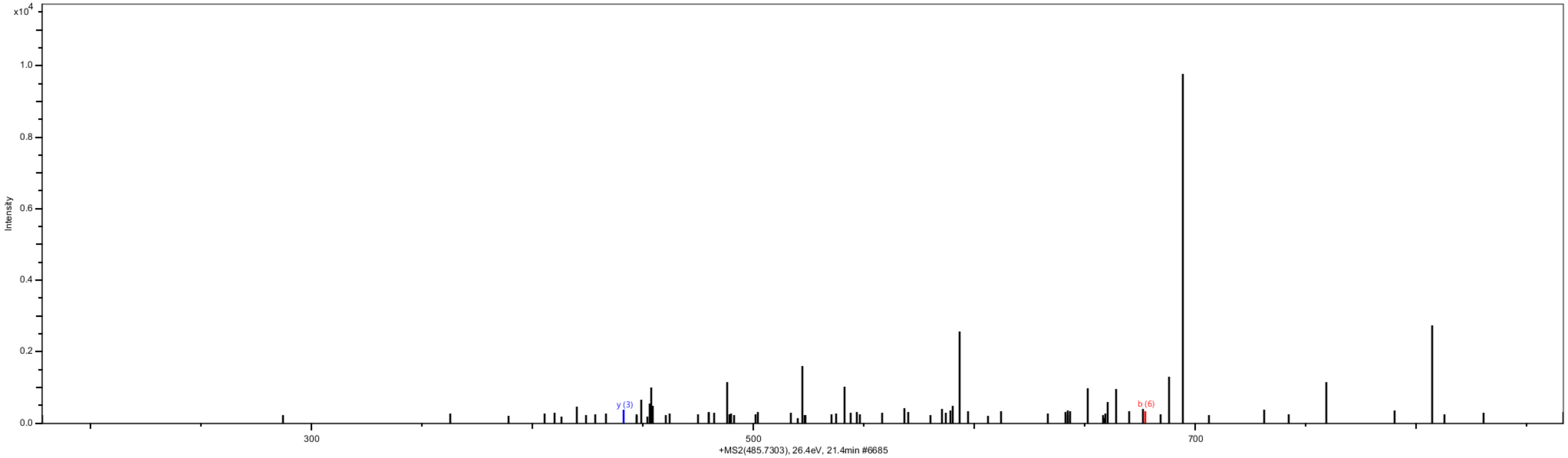

Spectrum Report
Combine Search_2020-10-19 07:53:31

SVIL_HUMAN

Accession: SVIL_HUMAN
Database: SwissProt
MW [kDa] / pI: 247.6 / 6.5

Score: 17.3
Seq. Coverage [%]: 0.9
No. of Peptides: 1

Peptide Match

Sequence: R.GEVPVEGNLLEVACHCSSLR.S
Score: 17.3
Modifications: Carbamidomethyl: 14, 16

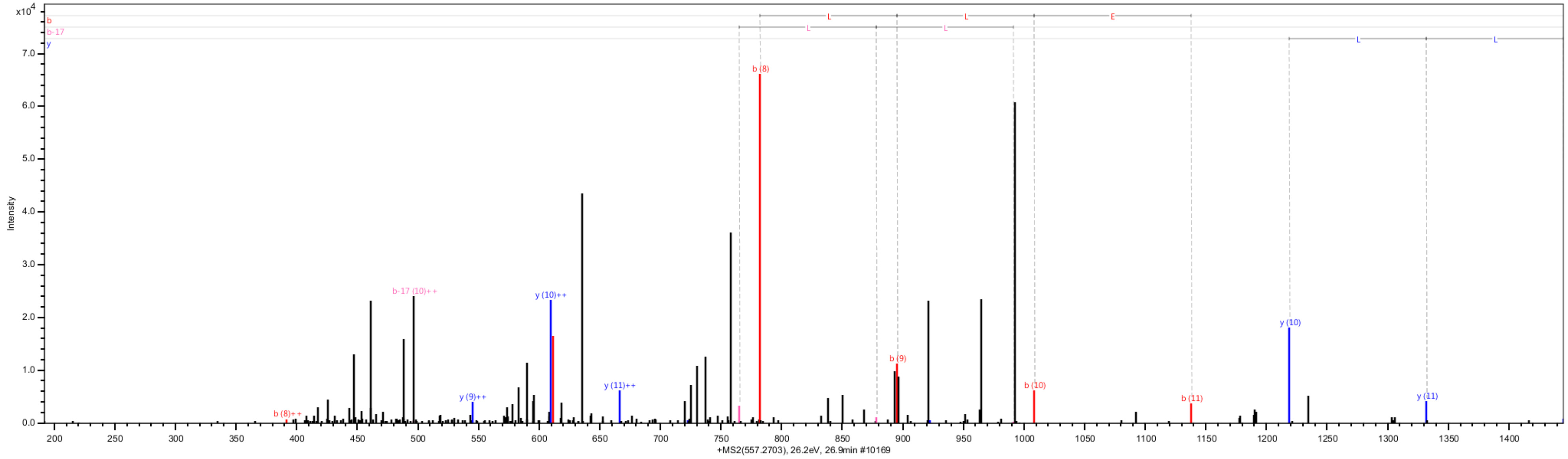

ZN730_HUMAN

Accession: ZN730_HUMAN
Database: SwissProt
MW [kDa] / pI: 59.0 / 9.5
Score: 17.3
Seq. Coverage [%]: 2.2
No. of Peptides: 1

Peptide Match

Sequence: R.HNQCLTTSHSK.I
Score: 17.3
Modifications: Carbamidomethyl: 4

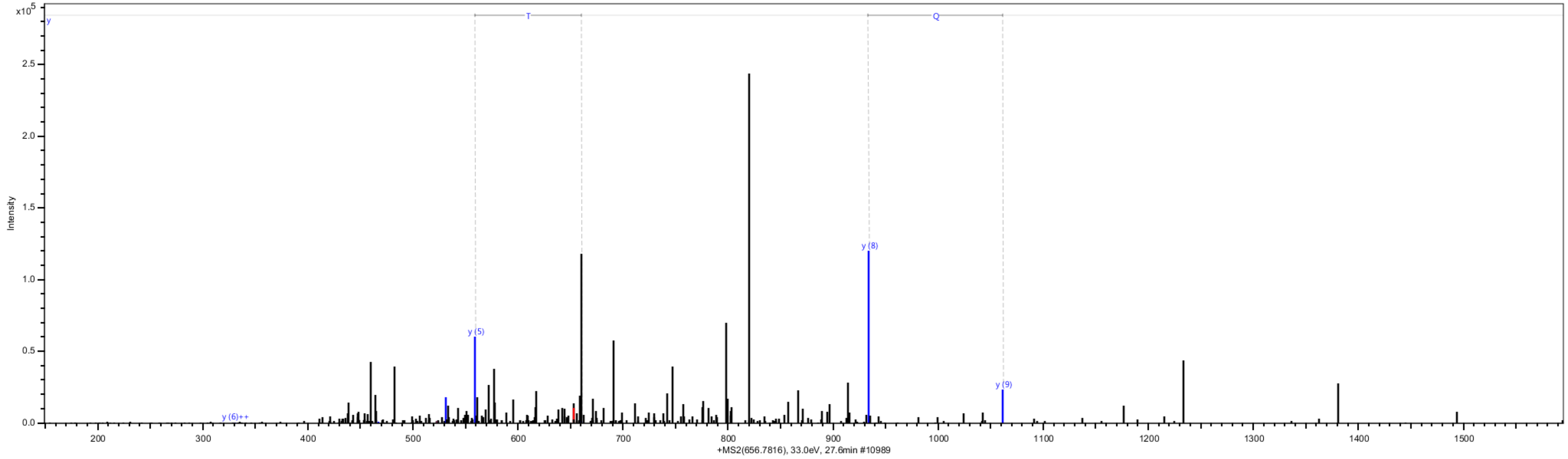

AINX_HUMAN

Accession: AINX_HUMAN
Database: SwissProt
MW [kDa] / pI: 55.4 / 5.3
Score: 17.4
Seq. Coverage [%]: 1.8
No. of Peptides: 1

Peptide Match

Sequence: R.STEAIRASR.E
Score: 17.4
Modifications:

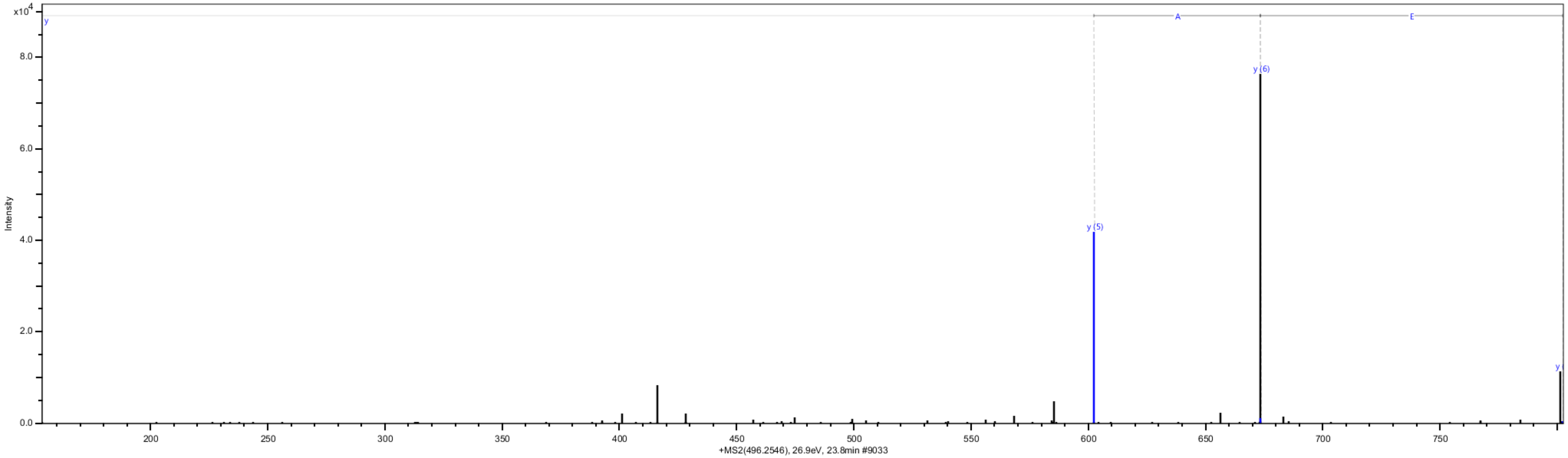

PTPRE_HUMAN

Accession: PTPRE_HUMAN
Database: SwissProt
MW [kDa] / pI: 80.6 / 6.6
Score: 17.5
Seq. Coverage [%]: 1.1
No. of Peptides: 1

Peptide Match

Sequence: K.FIAAQGPK.Q
Score: 17.4
Modifications:

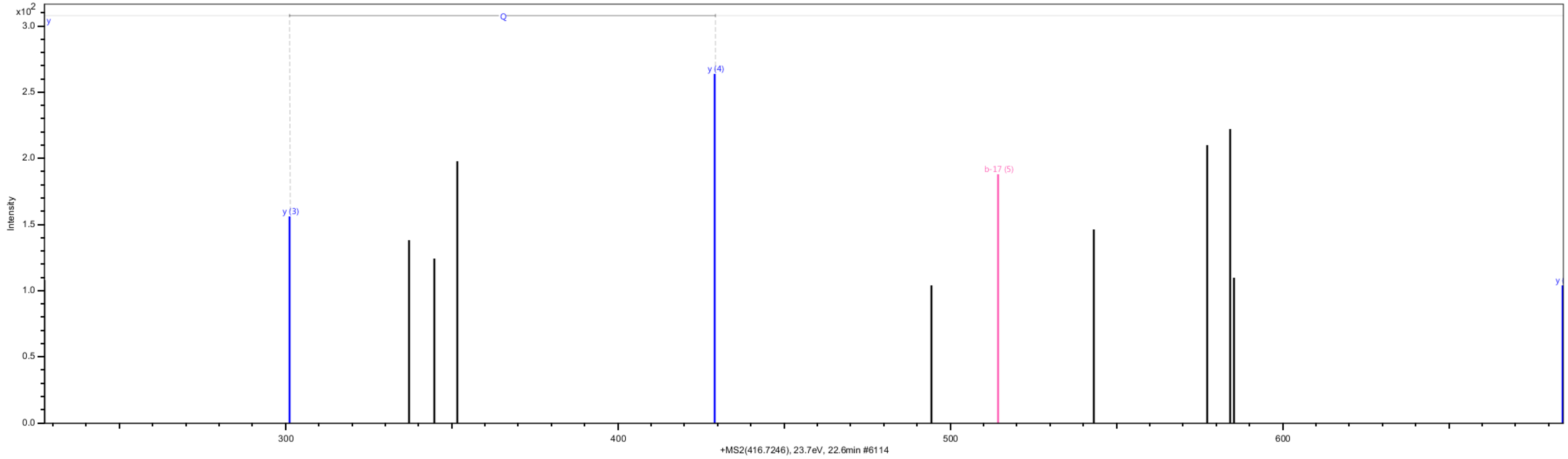

FGF14_HUMAN

Accession: FGF14_HUMAN
Database: SwissProt
MW [kDa] / pI: 27.7 / 10.1
Score: 17.5
Seq. Coverage [%]: 5.7
No. of Peptides: 1

Peptide Match

Sequence: R.QAREQHWDRPSASR.R
Score: 17.4
Modifications:

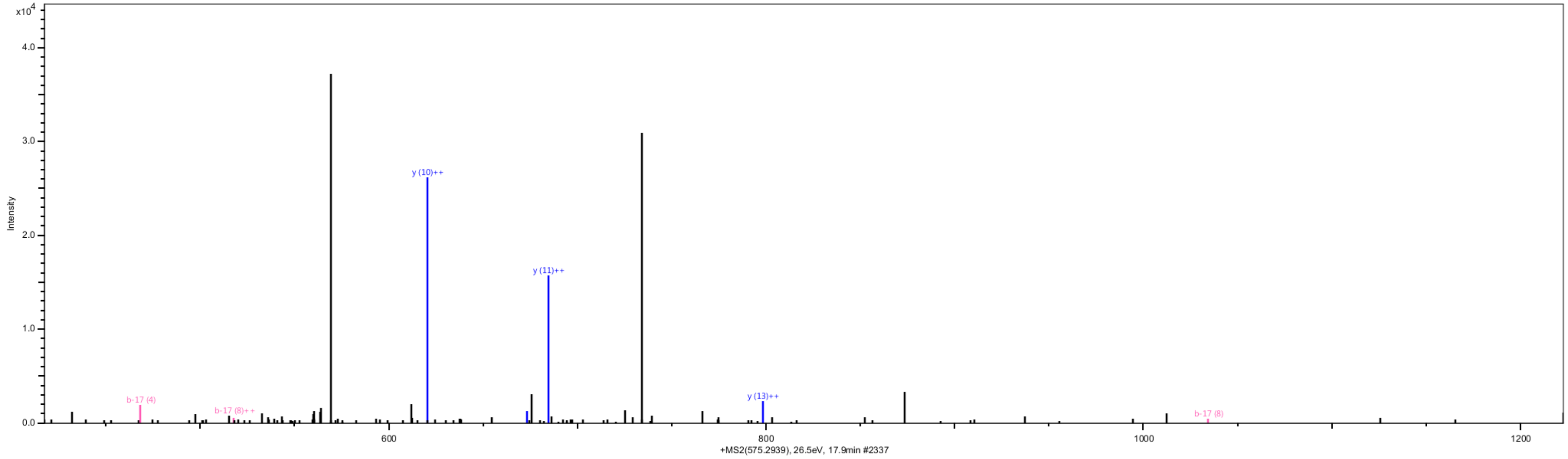

ERVV2_HUMAN

Accession: ERVV2_HUMAN
Database: SwissProt
MW [kDa] / pI: 59.3 / 8.9
Score: 17.4
Seq. Coverage [%]: 1.3
No. of Peptides: 1

Peptide Match

Sequence: R.IKQFHMK.S
Score: 17.4
Modifications: Oxidation: 6

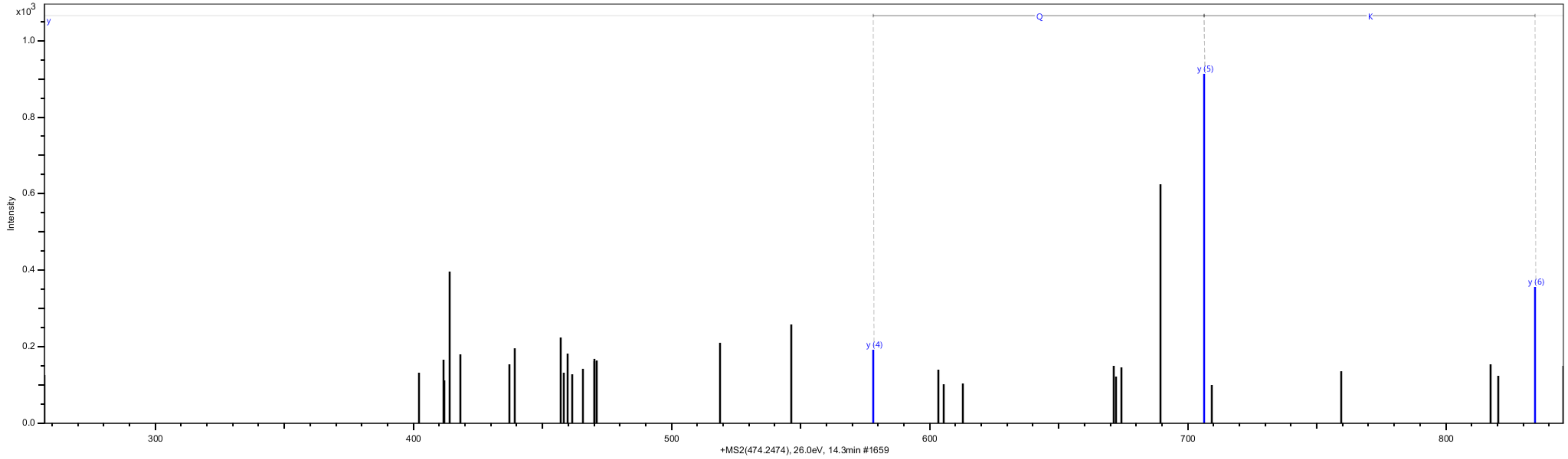

GP1BA_HUMAN

Accession: GP1BA_HUMAN
Database: SwissProt
MW [kDa] / pI: 71.5 / 5.9
Score: 17.4
Seq. Coverage [%]: 1.2
No. of Peptides: 1

Peptide Match

Sequence: R.GRQVTVPR.A
Score: 17.4
Modifications:

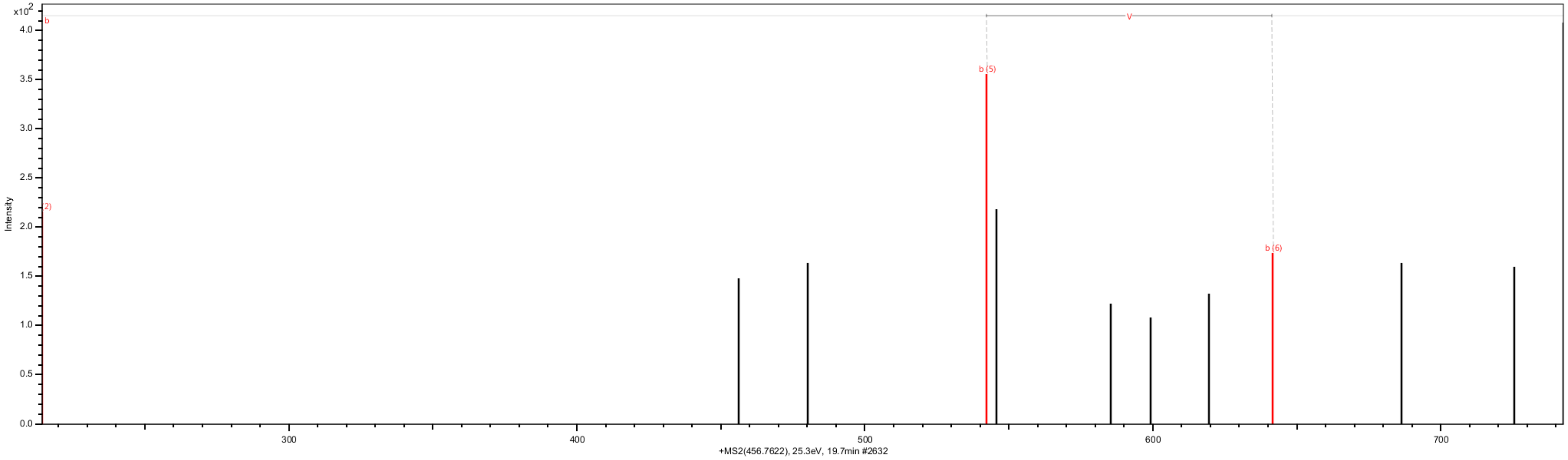

NUDC3_HUMAN

**Accession:**	NUDC3_HUMAN	**Score:**	17.4
**Database:**	SwissProt	**Seq. Coverage [%]:**	4.4
**MW [kDa] / pI:**	40.8 / 5.2	**No. of Peptides:**	1

Peptide Match

**Sequence:**	R.VAMLEENGERVLMEGK.L	**Score:**	17.4
**Modifications:**	Oxidation: 3		

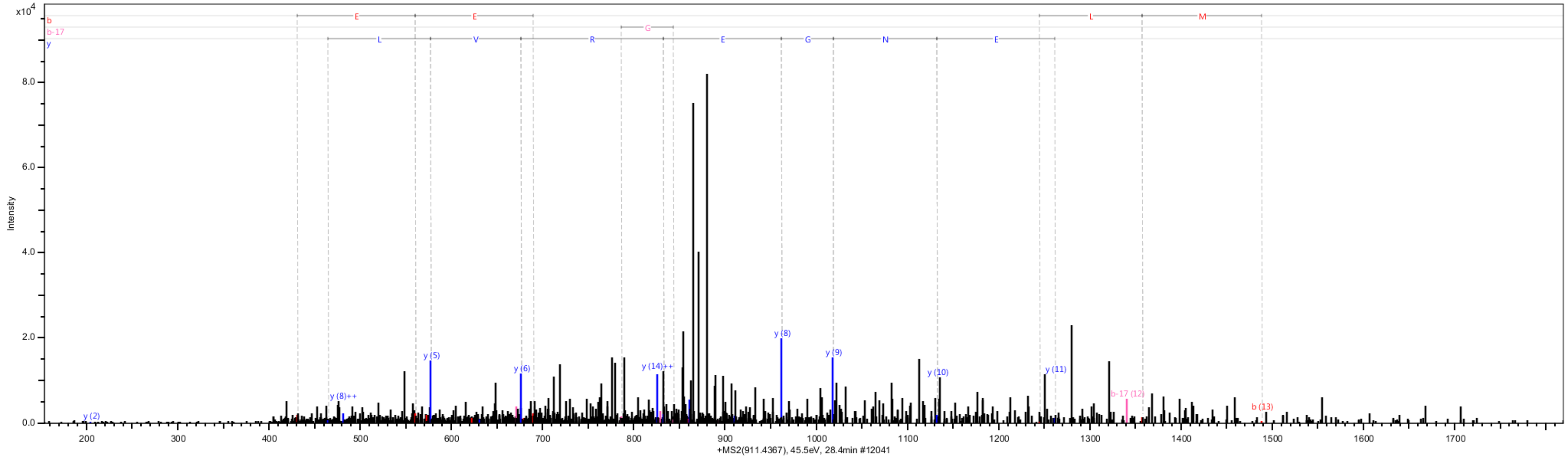

NAT8_HUMAN

Accession: NAT8_HUMAN
Database: SwissProt
MW [kDa] / pI: 25.6 / 9.1
Score: 17.4
Seq. Coverage [%]: 4.0
No. of Peptides: 1

Peptide Match

Sequence: R.QWVVGLLSR.G
Score: 17.4
Modifications:

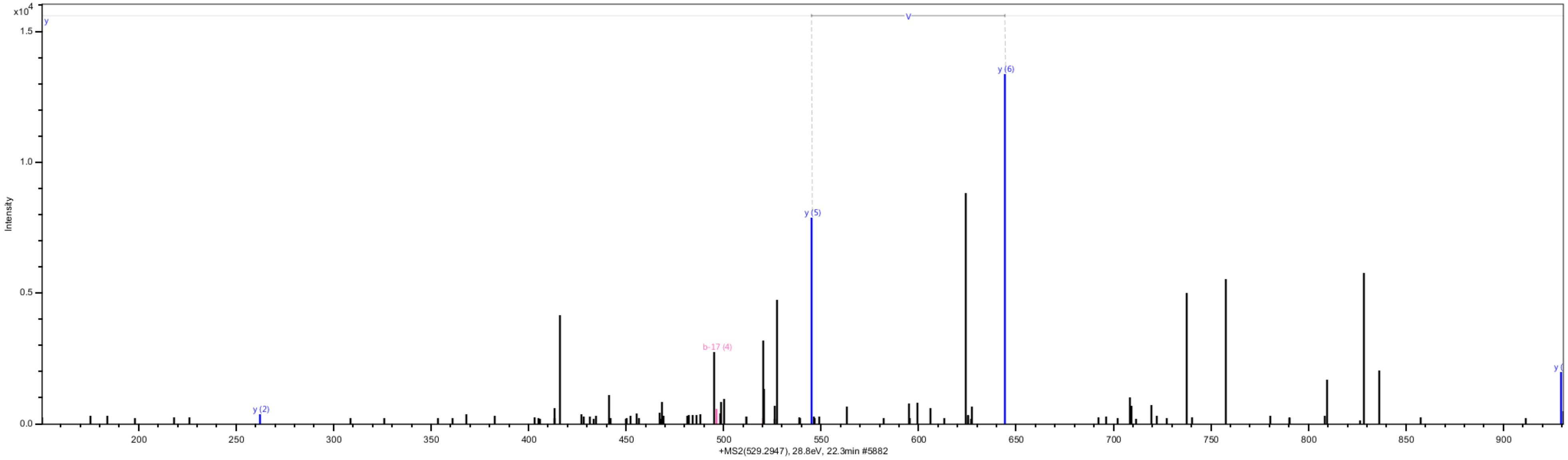

BORC8_HUMAN

Accession: BORC8_HUMAN
Database: SwissProt
MW [kDa] / pI: 13.4 / 5.5
Score: 18.0
Seq. Coverage [%]: 9.2
No. of Peptides: 1

Peptide Match

Sequence: -.MEEPEMQLKGK.K
Score: 18
Modifications: Oxidation: 1, 6

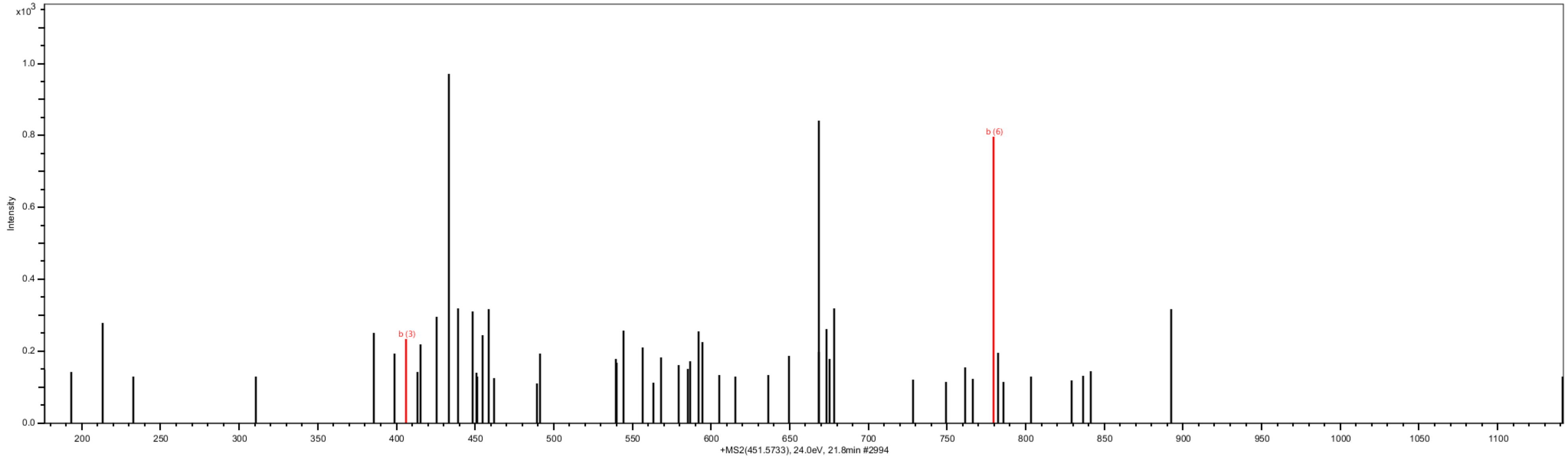

TR49D_HUMAN

Accession: TR49D_HUMAN
Database: SwissProt
MW [kDa] / pI: 52.5 / 8.7
Score: 18.0
Seq. Coverage [%]: 1.5
No. of Peptides: 1

Peptide Match

Sequence: K.GKDIFHR.L
Score: 18
Modifications:

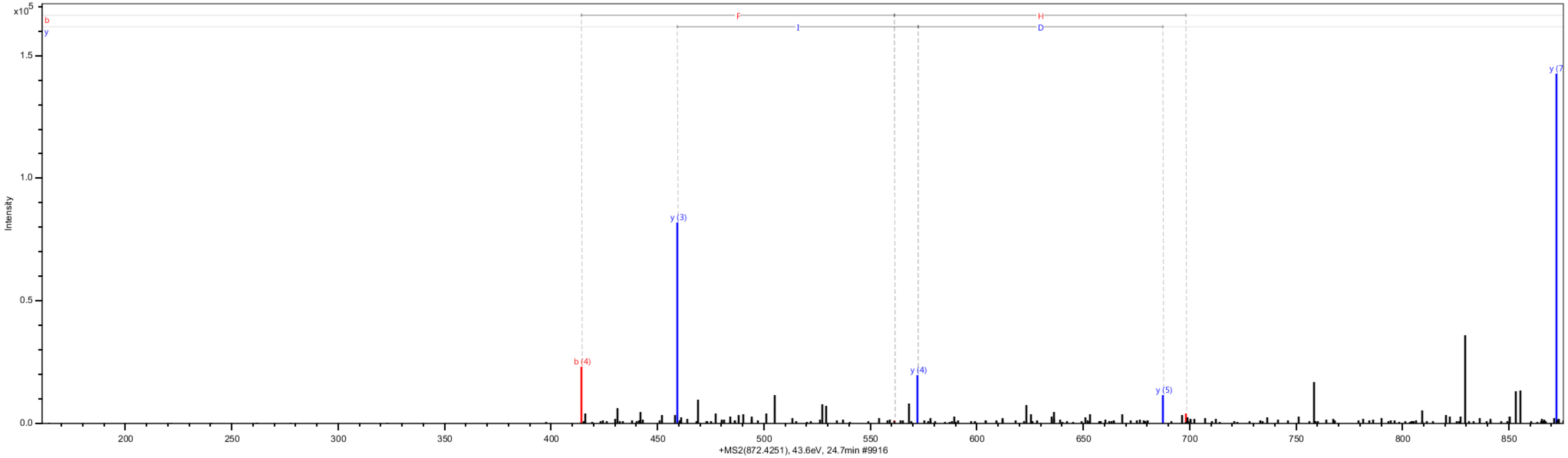

UBE3C_HUMAN

**Accession:**	UBE3C_HUMAN	**Score:**	18.0
**Database:**	SwissProt	**Seq. Coverage [%]:**	1.3
**MW [kDa] / pI:**	123.8 / 6.3	**No. of Peptides:**	1

Peptide Match

**Sequence:**	K.HSSLFVKQLDGSER.L	**Score:**	18
**Modifications:**			

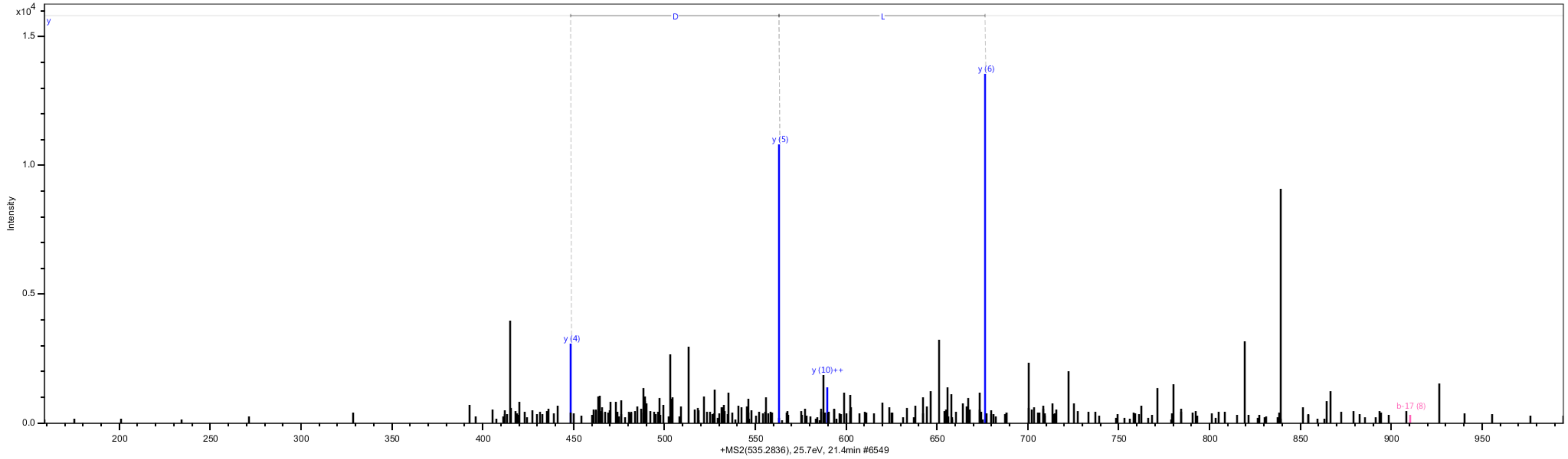

CK2N1_HUMAN

**Accession:**	CK2N1_HUMAN	**Score:**	18.0
**Database:**	SwissProt	**Seq. Coverage [%]:**	14.1
**MW [kDa] / pI:**	8.5 / 5.2	**No. of Peptides:**	1

Peptide Match

**Sequence:**	-.MSEVLPYGDEK.L	**Score:**	18
**Modifications:**	Oxidation: 1		

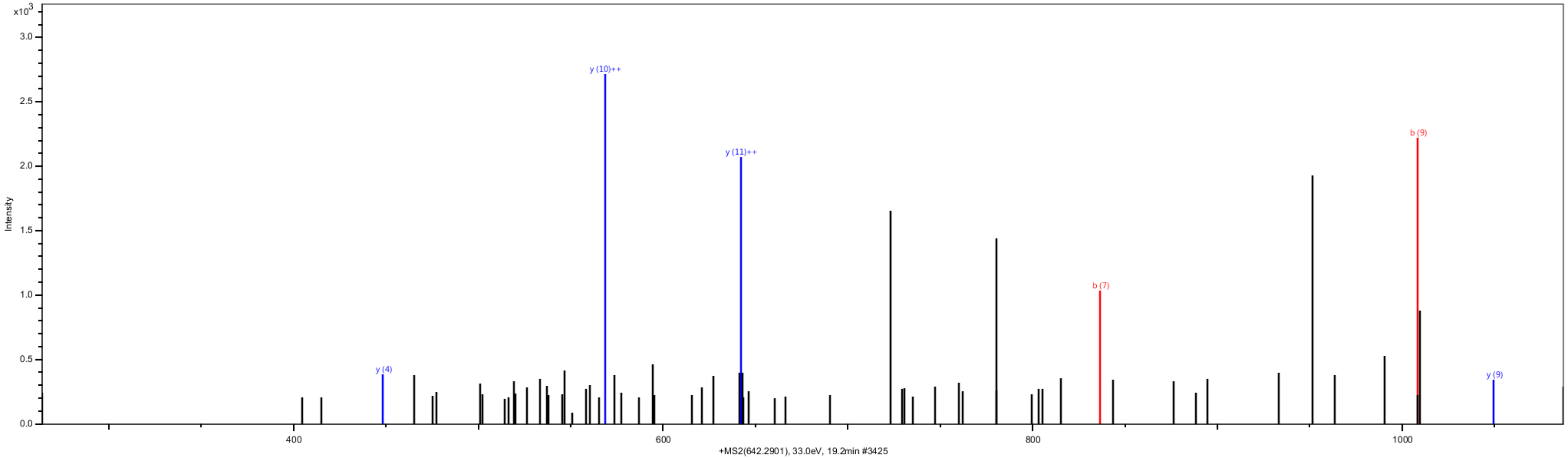

ES8L2_HUMAN

Accession: ES8L2_HUMAN
Database: SwissProt
MW [kDa] / pI: 80.6 / 6.4
Score: 18.0
Seq. Coverage [%]: 1.4
No. of Peptides: 1

Peptide Match

Sequence: R.SDGVAKMSPK.D
Score: 18
Modifications: Oxidation: 7

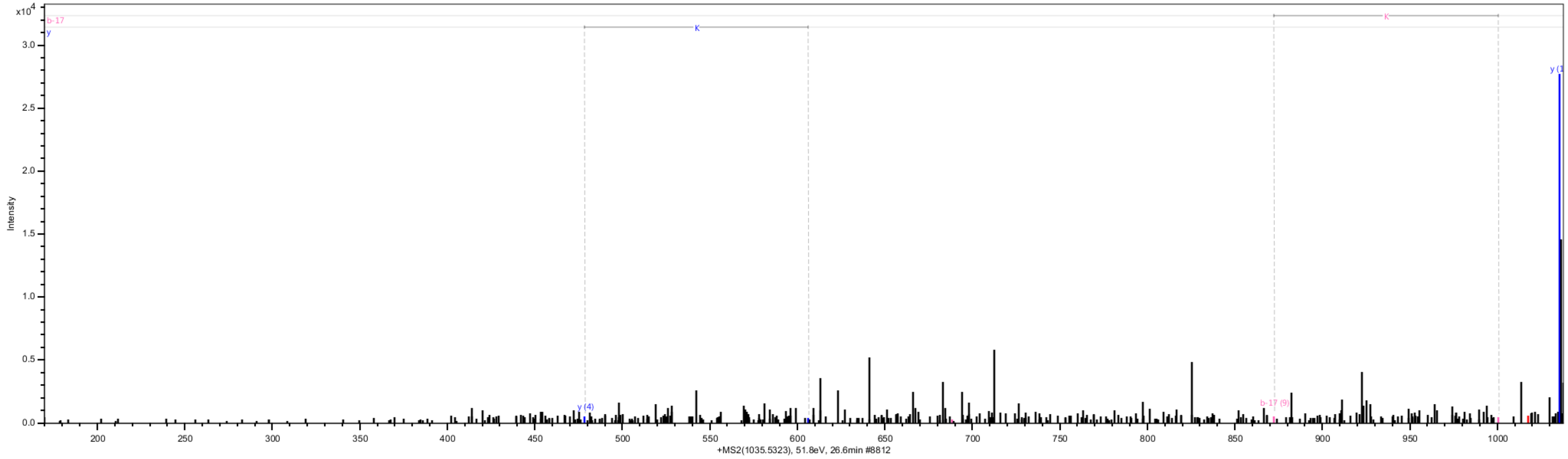

BRWD1_HUMAN

**Accession:**	BRWD1_HUMAN	**Score:**	17.9
**Database:**	SwissProt	**Seq. Coverage [%]:**	0.4
**MW [kDa] / pI:**	262.8 / 8.7	**No. of Peptides:**	1

Peptide Match

**Sequence:**	R.MGADQDTIPR.G	**Score:**	17.9
**Modifications:**			

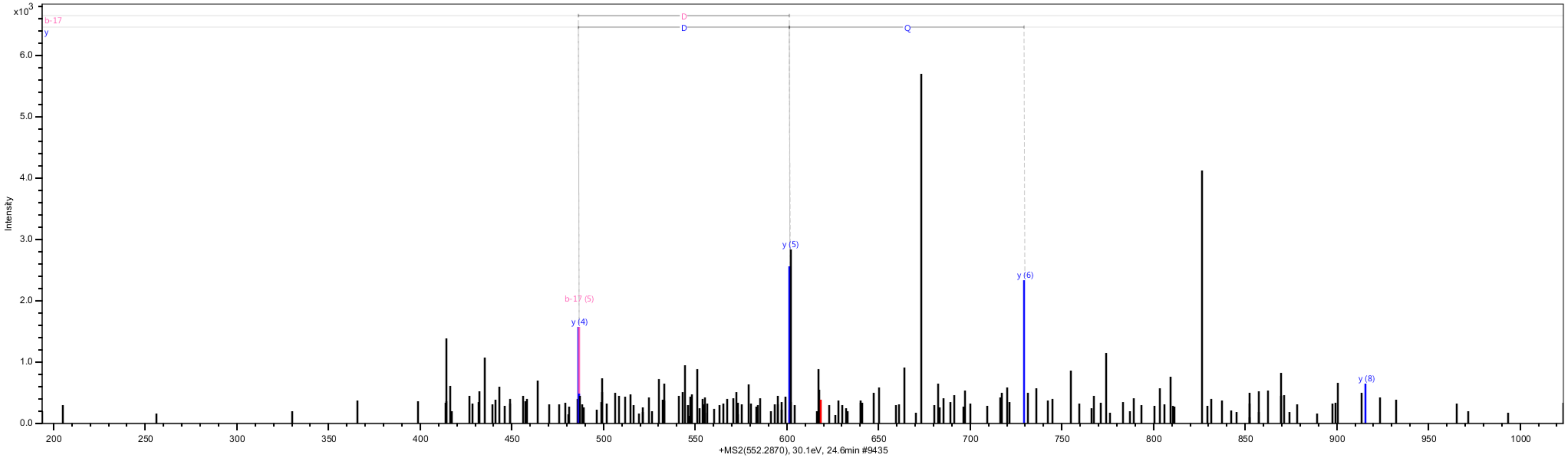

STAT6_HUMAN

**Accession:**	STAT6_HUMAN	**Score:**	17.9
**Database:**	SwissProt	**Seq. Coverage [%]:**	0.9
**MW [kDa] / pI:**	94.1 / 5.8	**No. of Peptides:**	1

Peptide Match

**Sequence:**	K.MCETLNLK.F	**Score:**	17.9
**Modifications:**	Carbamidomethyl: 2; Oxidation: 1		

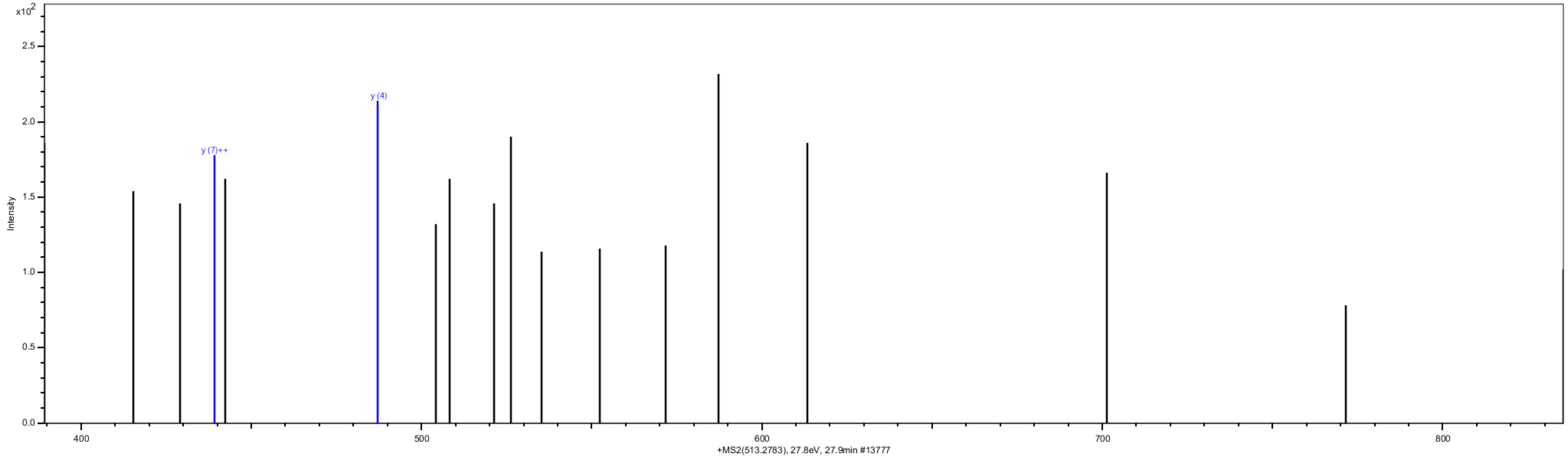

ARHG8_HUMAN

**Accession:**	ARHG8_HUMAN	**Score:**	18.2
**Database:**	SwissProt	**Seq. Coverage [%]:**	2.3
**MW [kDa] / pI:**	67.7 / 9.3	**No. of Peptides:**	1

Peptide Match

**Sequence:**	R.SSALWSEMLDITMK.E	**Score:**	18.2
**Modifications:**	Oxidation: 8		

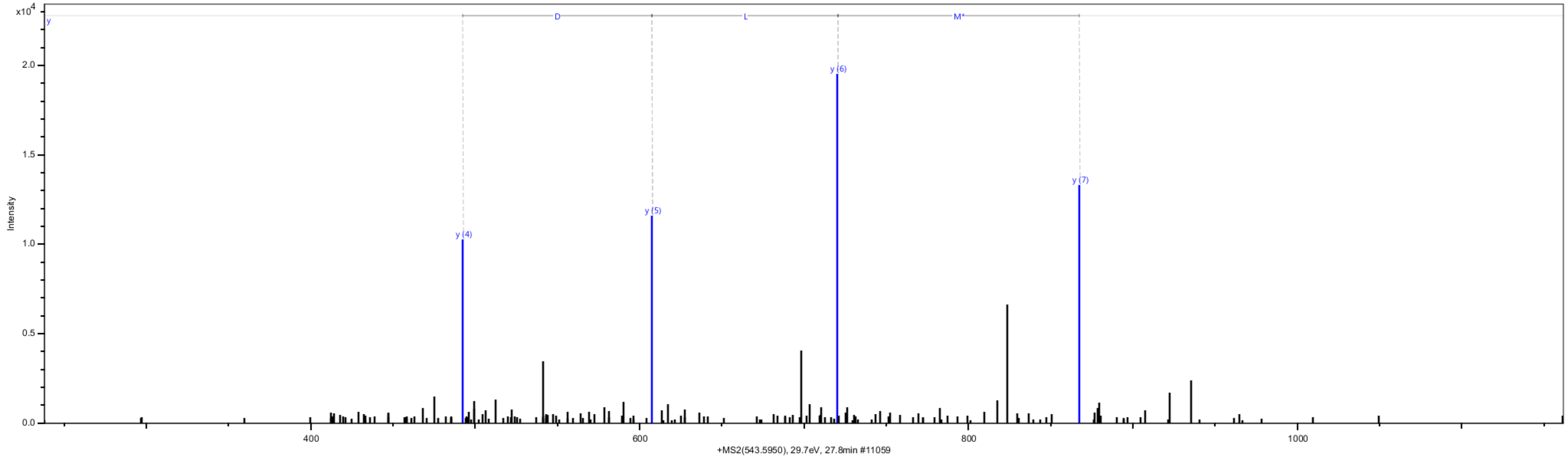

CLD10_HUMAN

**Accession:**	CLD10_HUMAN	**Score:**	18.1
**Database:**	SwissProt	**Seq. Coverage [%]:**	4.4
**MW [kDa] / pI:**	24.5 / 8.3	**No. of Peptides:**	1

Peptide Match

**Sequence:**	R.TKYHGGEDFK.T	**Score:**	18.1
**Modifications:**			

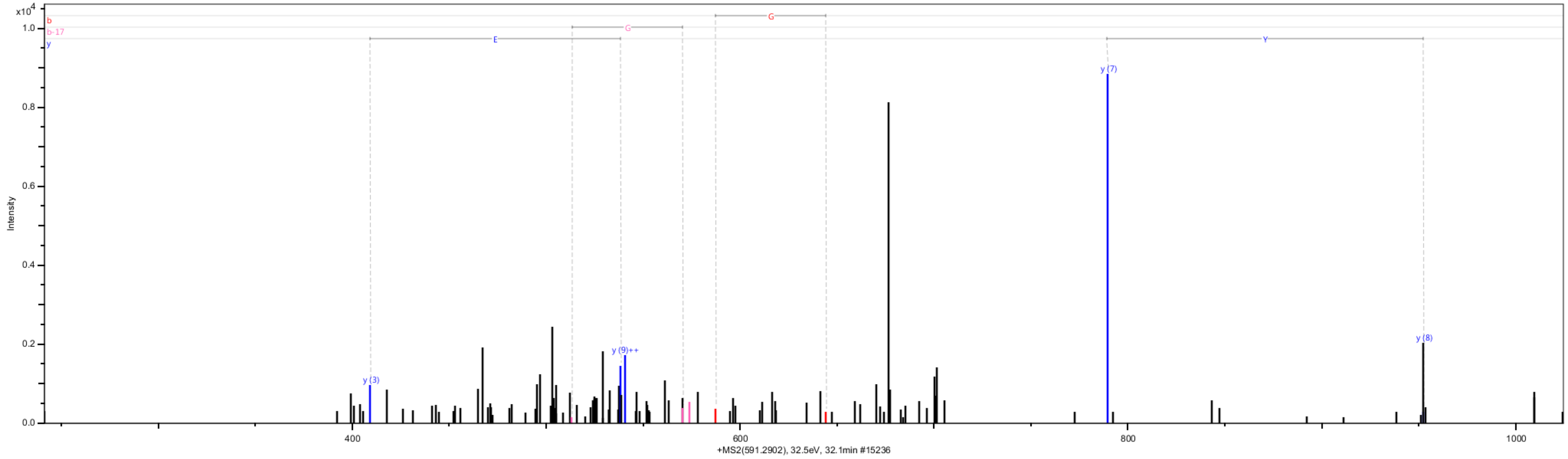

RFC1_HUMAN

Accession: RFC1_HUMAN
Database: SwissProt
MW [kDa] / pI: 128.2 / 9.4

Score: 18.1
Seq. Coverage [%]: 0.7
No. of Peptides: 1

Peptide Match

Sequence: K.IGEVSSPK.A
Score: 18.1
Modifications:

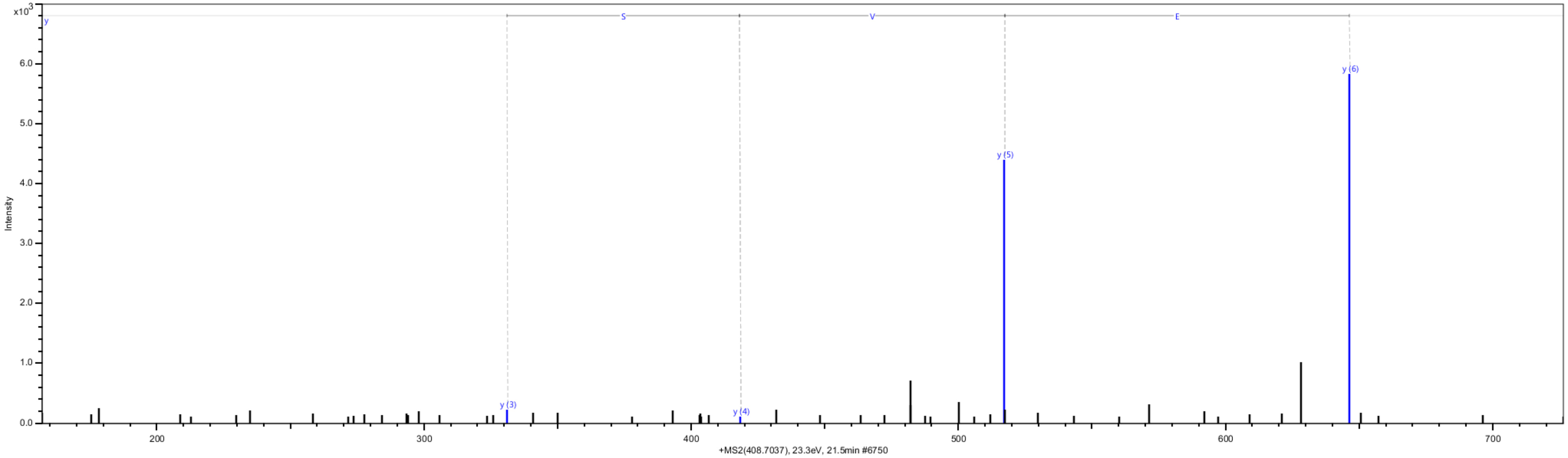

RMC1_HUMAN

**Accession:**	RMC1_HUMAN	**Score:**	18.0
**Database:**	SwissProt	**Seq. Coverage [%]:**	1.2
**MW [kDa] / pI:**	74.9 / 7.9	**No. of Peptides:**	1

Peptide Match

**Sequence:**	R.LMDFLLQR.K	**Score:**	18
**Modifications:**	Oxidation: 2		

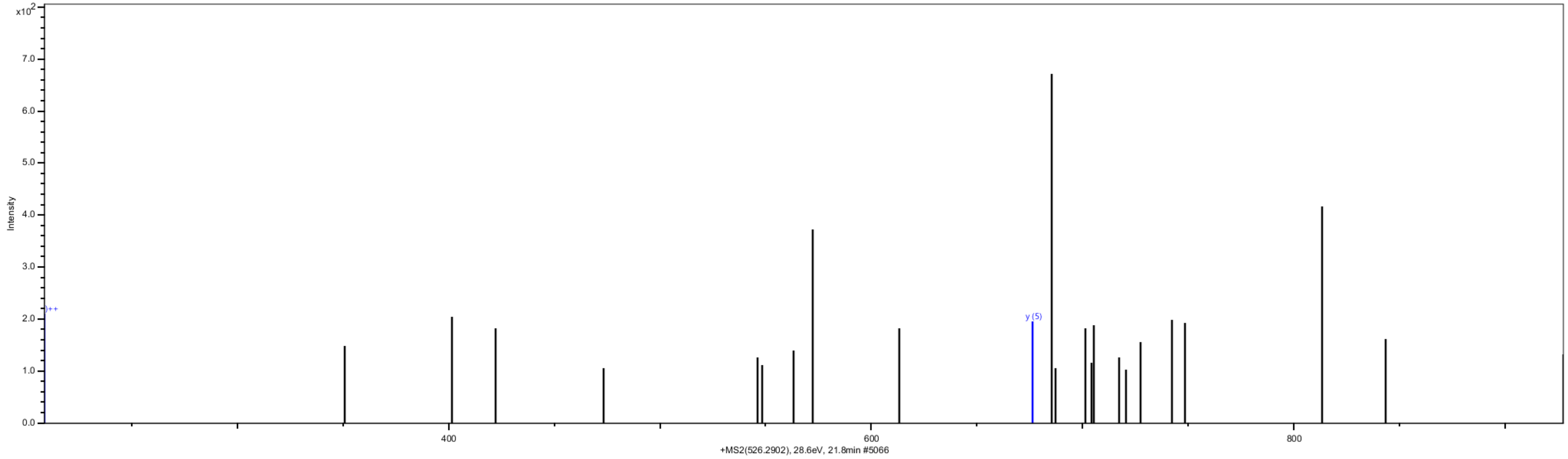

ENOL_HUMAN

Accession: ENOL_HUMAN
Database: SwissProt
MW [kDa] / pI: 9.0 / 8.5
Score: 18.0
Seq. Coverage [%]: 14.5
No. of Peptides: 1

Peptide Match

Sequence: -.MASTPMGNEGEK.K
Score: 18
Modifications: Oxidation: 1, 6

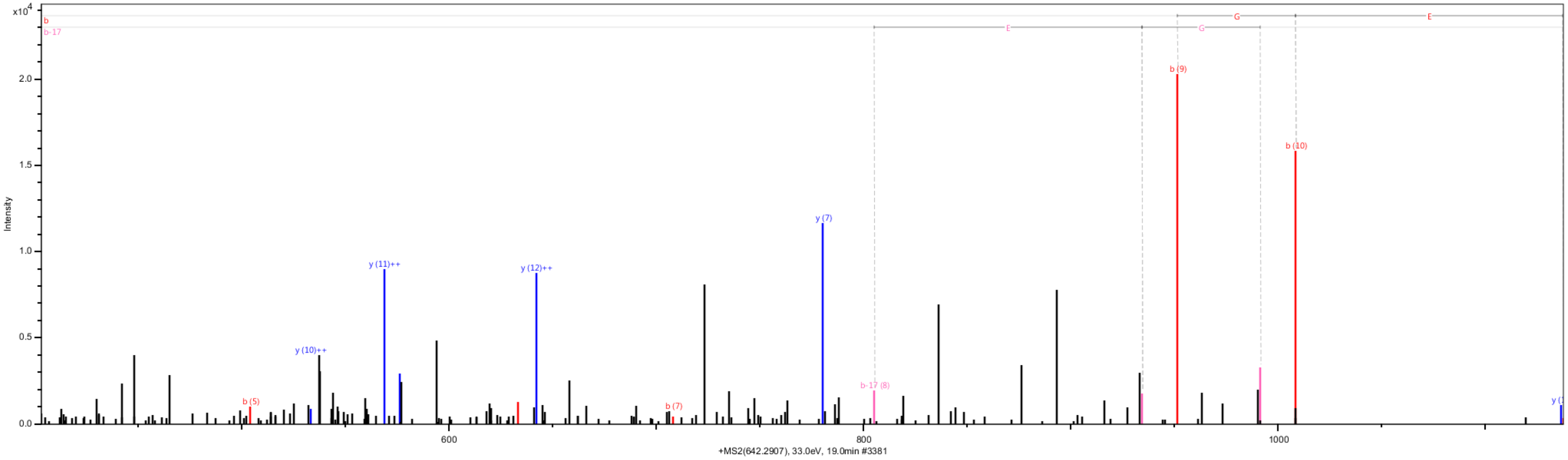

CHSTB_HUMAN

Accession: CHSTB_HUMAN
Database: SwissProt
MW [kDa] / pI: 41.5 / 9.0
Score: 18.0
Seq. Coverage [%]: 2.3
No. of Peptides: 1

Peptide Match

Sequence: R.DQVTDTCR.A
Score: 18
Modifications: Carbamidomethyl: 7

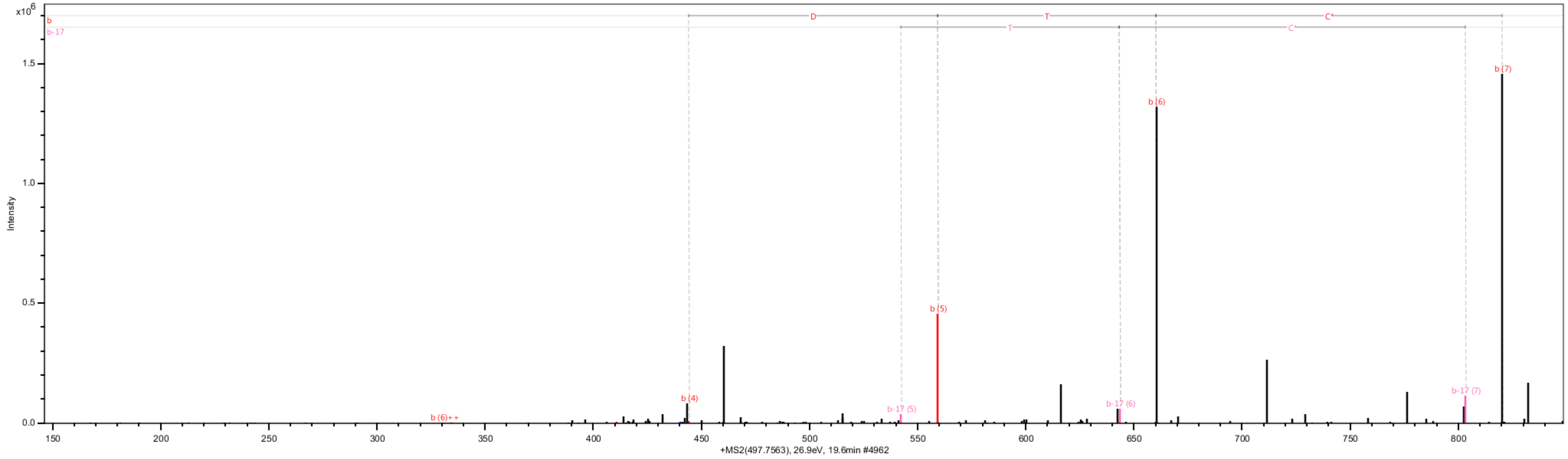

TTF2_HUMAN

Accession: TTF2_HUMAN
Database: SwissProt
MW [kDa] / pI: 129.5 / 8.6
Score: 18.0
Seq. Coverage [%]: 0.8
No. of Peptides: 1

Peptide Match

Sequence: K.VEPSDPVAR.R
Score: 18
Modifications:

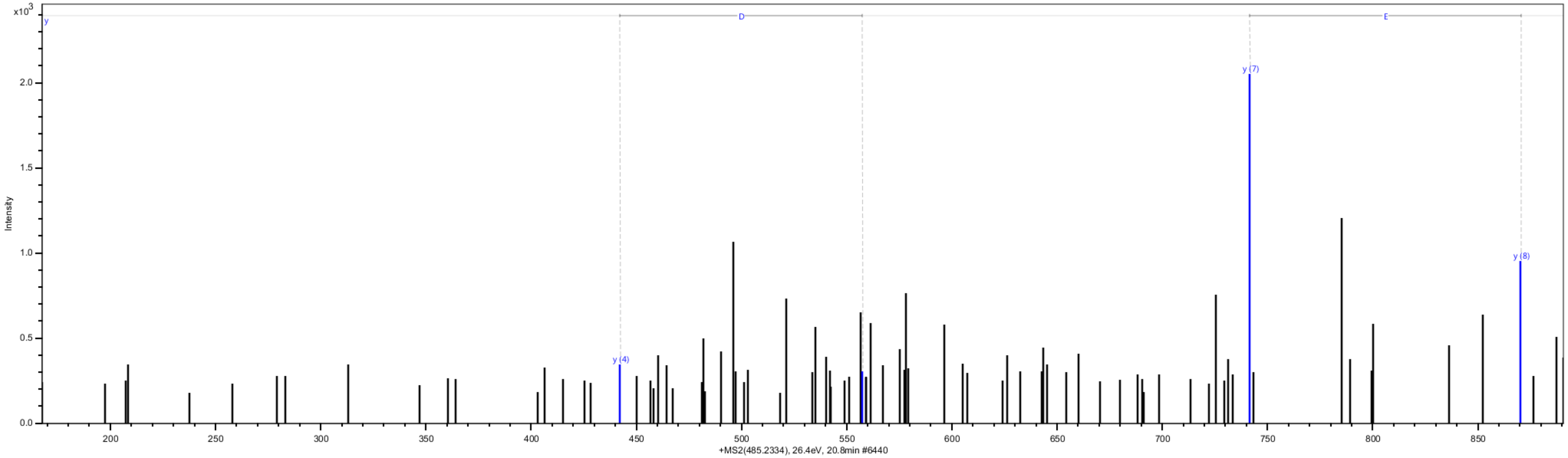

F184A_HUMAN

Accession: F184A_HUMAN
Database: SwissProt
MW [kDa] / pI: 132.9 / 5.6
Score: 18.0
Seq. Coverage [%]: 1.0
No. of Peptides: 1

Peptide Match

Sequence: R.LQQQASDLVLK.A
Score: 18
Modifications:

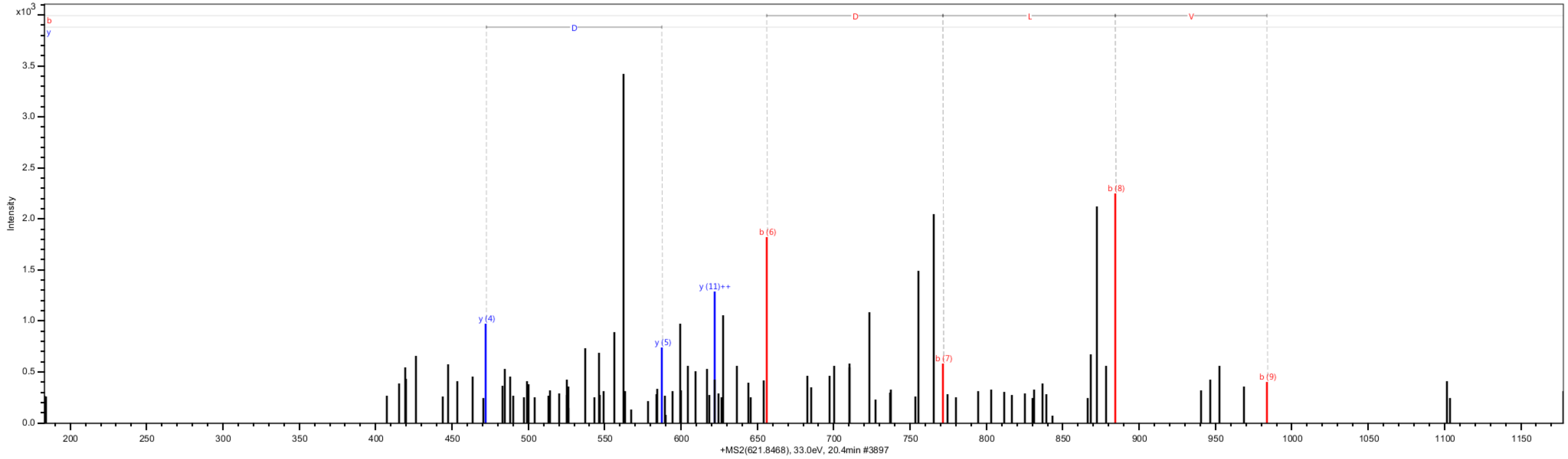

FZD2_HUMAN

Accession: FZD2_HUMAN
Database: SwissProt
MW [kDa] / pI: 63.5 / 8.5
Score: 17.7
Seq. Coverage [%]: 1.9
No. of Peptides: 1

Peptide Match

Sequence: R.ARQGCEALMNK.F
Score: 17.7
Modifications: Carbamidomethyl: 5; Oxidation: 9

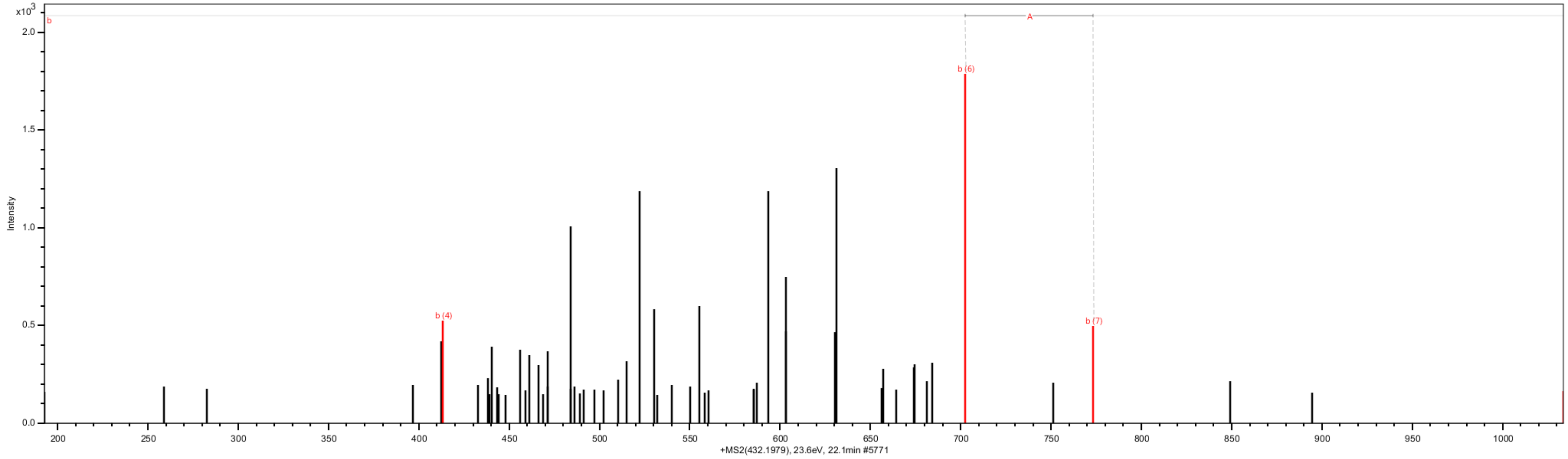

MON2_HUMAN

Accession: MON2_HUMAN
Database: SwissProt
MW [kDa] / pI: 190.2 / 5.7
Score: 17.7
Seq. Coverage [%]: 0.6
No. of Peptides: 1

Peptide Match

Sequence: K.KLLENMQSDLR.A
Score: 17.7
Modifications:

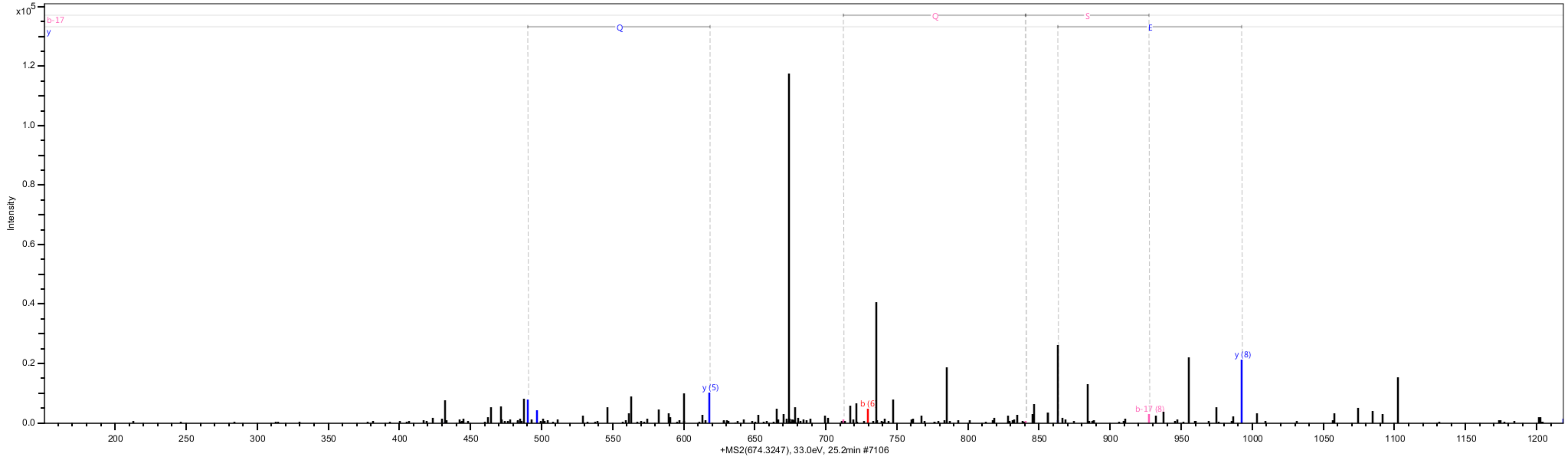

ENY2_HUMAN

Accession: ENY2_HUMAN
Database: SwissProt
MW [kDa] / pI: 11.5 / 9.4
Score: 17.8
Seq. Coverage [%]: 7.9
No. of Peptides: 1

Peptide Match

Sequence: K.MNKDAQMR.A
Score: 17.8
Modifications: Oxidation: 7

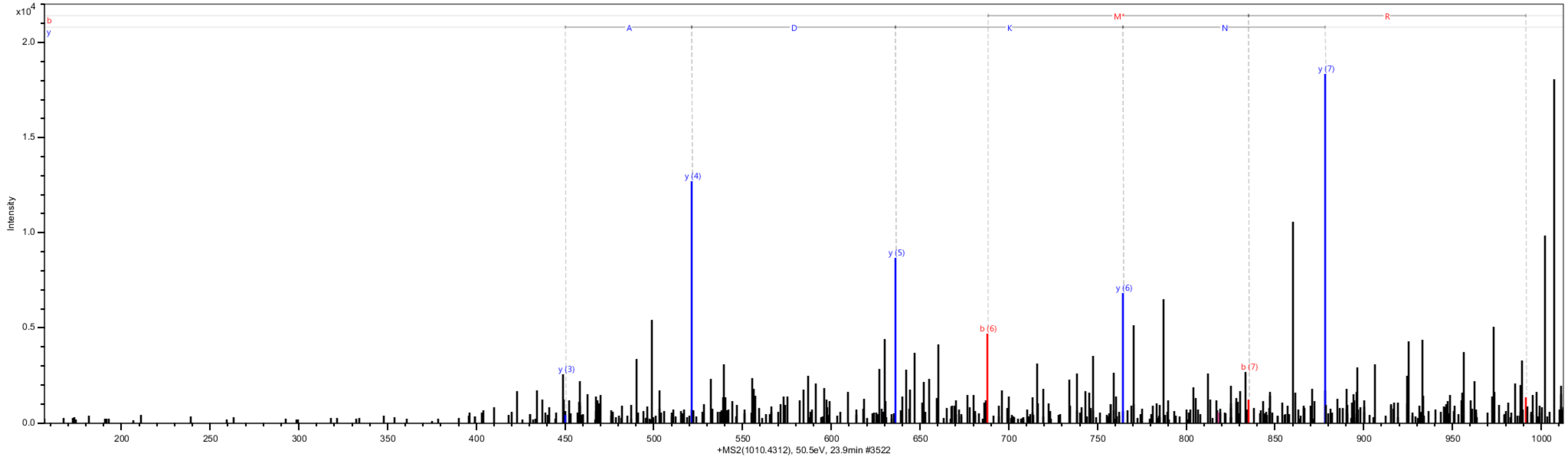

CP2CI_HUMAN

**Accession:**	CP2CI_HUMAN	**Score:**	17.8
**Database:**	SwissProt	**Seq. Coverage [%]:**	1.8
**MW [kDa] / pI:**	55.7 / 6.8	**No. of Peptides:**	1

Peptide Match

**Sequence:**	R.MCMGEGLAR.M	**Score:**	17.8
**Modifications:**	Carbamidomethyl: 2; Oxidation: 1		

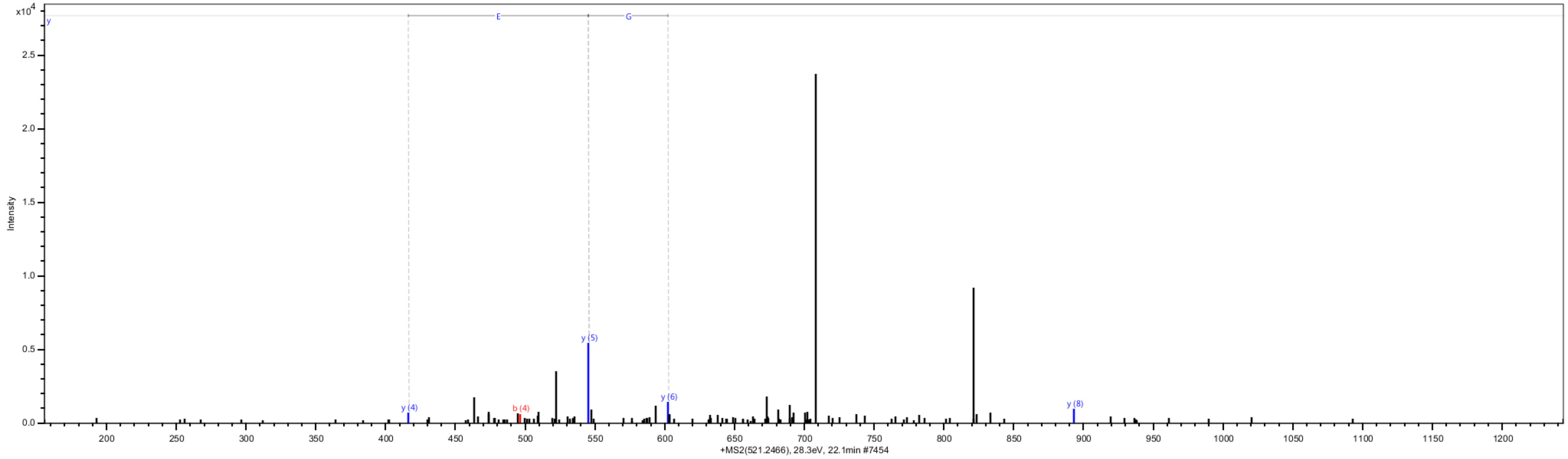

MYOZ3_HUMAN

**Accession:**	MYOZ3_HUMAN	**Score:**	17.7
**Database:**	SwissProt	**Seq. Coverage [%]:**	5.6
**MW [kDa] / pI:**	27.1 / 9.4	**No. of Peptides:**	1

Peptide Match

**Sequence:**	K.GVPPEKFNHTAISK.G	**Score:**	17.7
**Modifications:**			

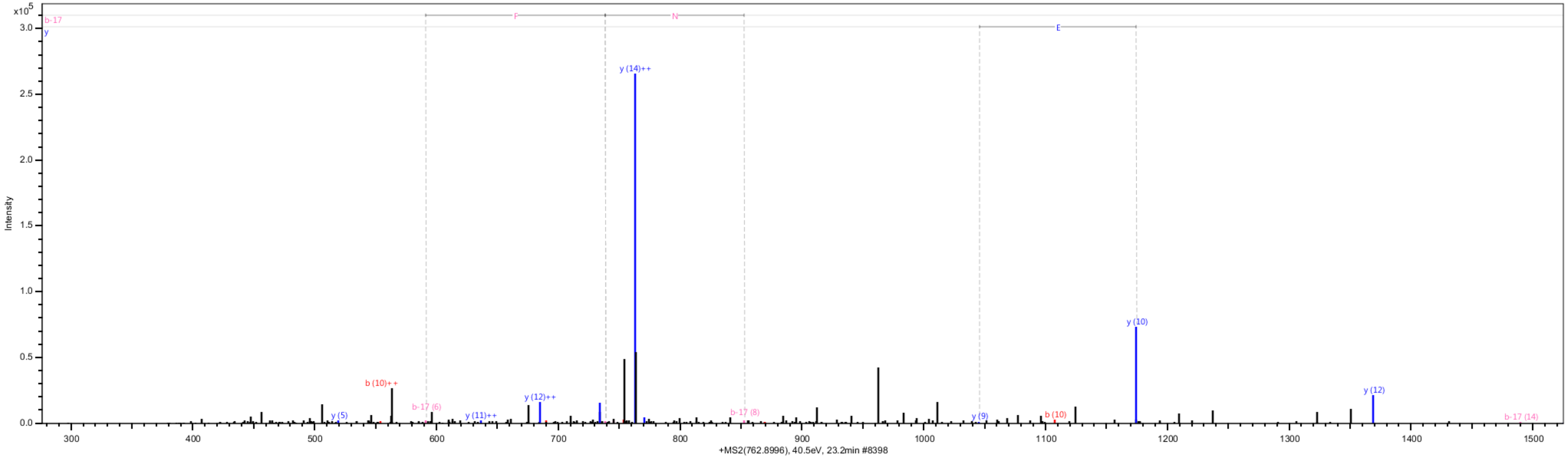

LAMA2_HUMAN

Accession: LAMA2_HUMAN
Database: SwissProt
MW [kDa] / pI: 343.7 / 6.0
Score: 17.7
Seq. Coverage [%]: 0.5
No. of Peptides: 1

Peptide Match

Sequence: R.TPYNILSSPDYVGVTK.G
Score: 17.7
Modifications:

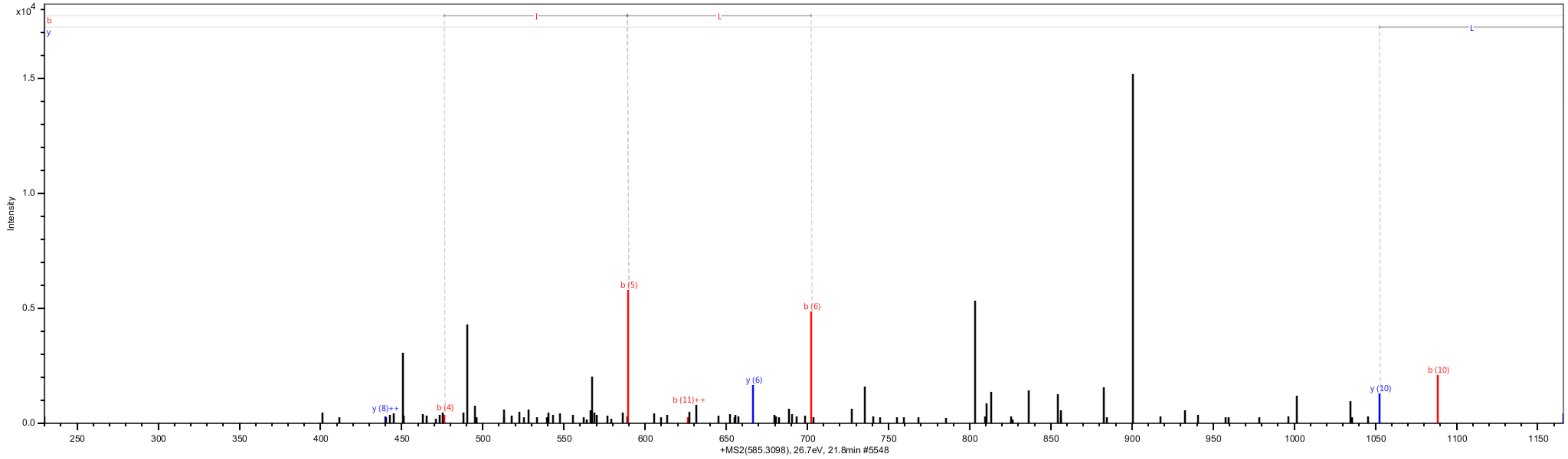

NOD2_HUMAN

**Accession:**	NOD2_HUMAN	**Score:**	15.0
**Database:**	SwissProt	**Seq. Coverage [%]:**	1.1
**MW [kDa] / pI:**	115.2 / 6.3	**No. of Peptides:**	1

Peptide Match

**Sequence:**	K.LTDGCAHSMAK.L	**Score:**	15
**Modifications:**	Carbamidomethyl: 5; Oxidation: 9		

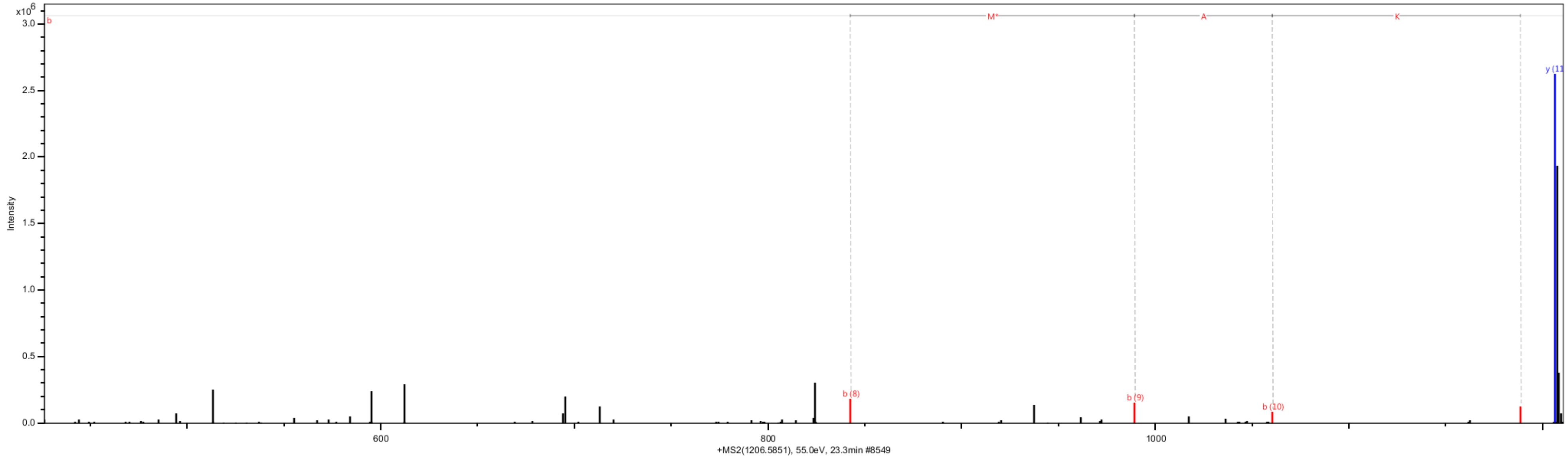

AT5L2_HUMAN

Accession: AT5L2_HUMAN
Database: SwissProt
MW [kDa] / pI: 11.0 / 9.9
Score: 15.1
Seq. Coverage [%]: 10.0
No. of Peptides: 1

Peptide Match

Sequence: M.APFVRNLVEK.T
Score: 15.1
Modifications:

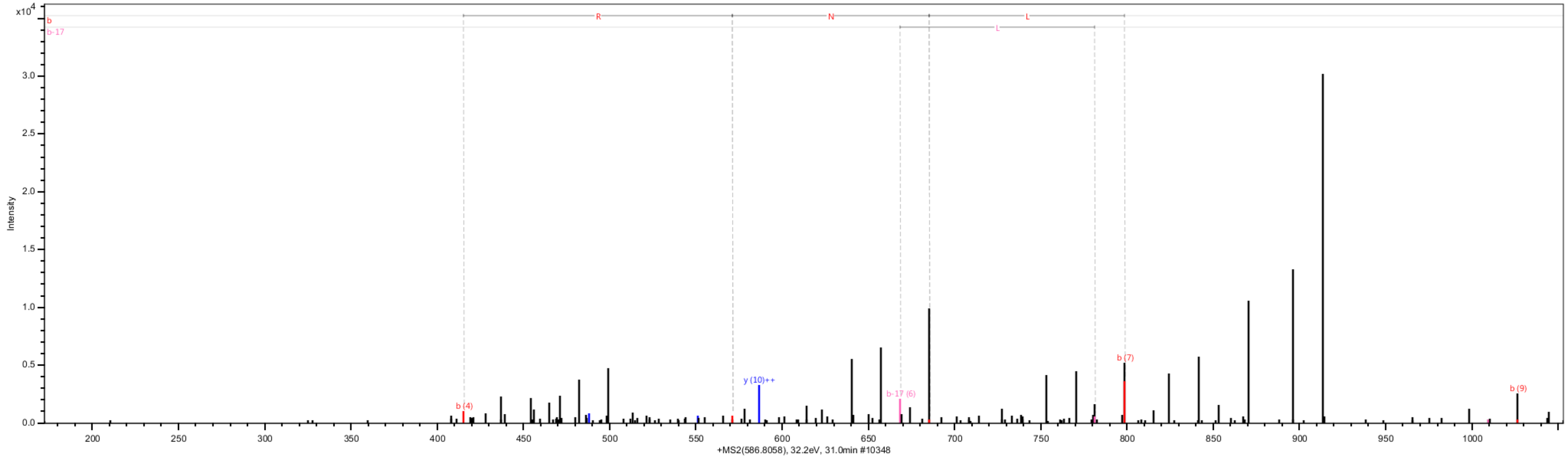

TAF2_HUMAN

Accession: TAF2_HUMAN
Database: SwissProt
MW [kDa] / pI: 136.9 / 8.5

Score: 15.1
Seq. Coverage [%]: 1.0
No. of Peptides: 1

Peptide Match

Sequence: K.LLSLASTASSQK.F
Score: 15.1
Modifications:

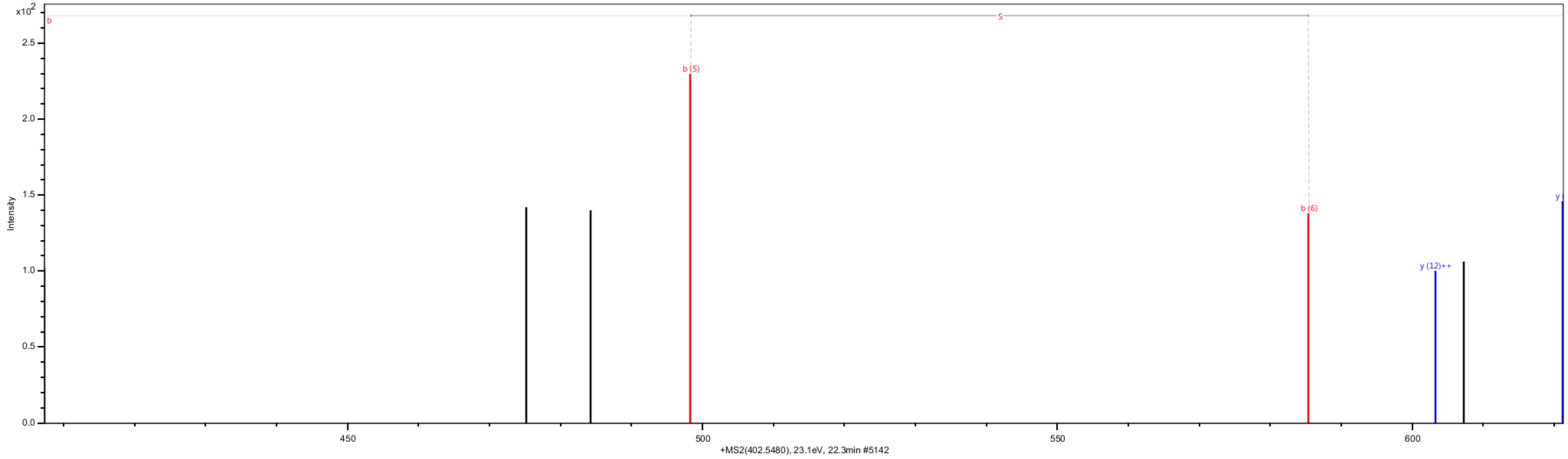

RIN2_HUMAN

Accession: RIN2_HUMAN
Database: SwissProt
MW [kDa] / pI: 100.1 / 6.2
Score: 15.0
Seq. Coverage [%]: 1.5
No. of Peptides: 1

Peptide Match

Sequence: R.VAFQEVNSGCTGK.T
Score: 15
Modifications: Carbamidomethyl: 10

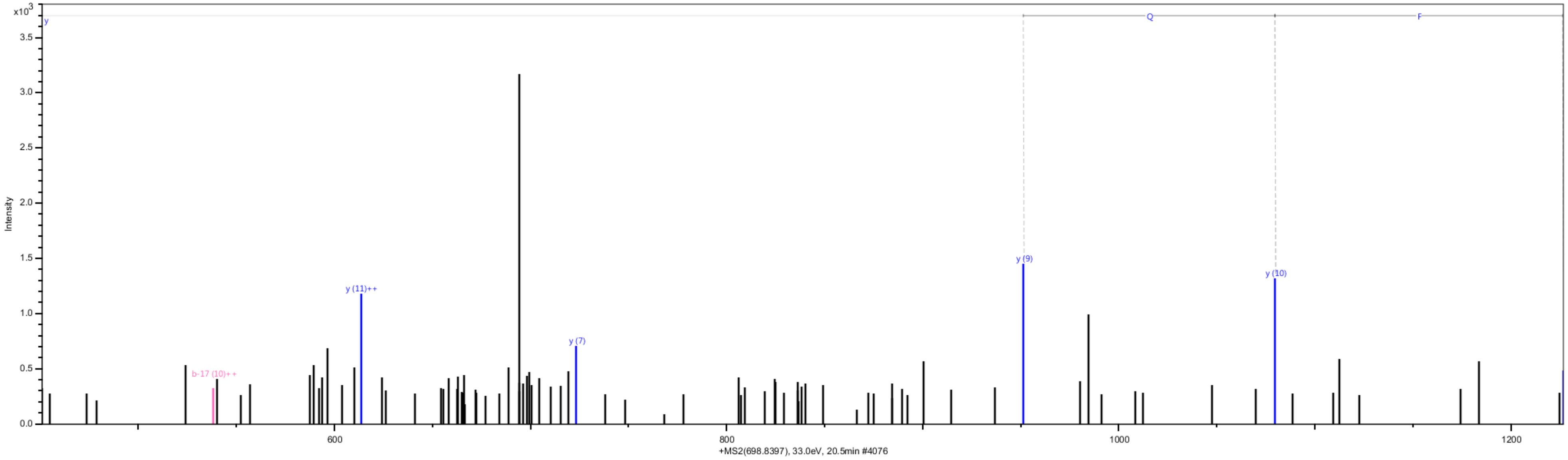

SYLM_HUMAN

Accession: SYLM_HUMAN
Database: SwissProt
MW [kDa] / pI: 101.9 / 8.5
Score: 15.0
Seq. Coverage [%]: 1.1
No. of Peptides: 1

Peptide Match

Sequence: K.SPQPQLLSNK.E
Score: 15
Modifications:

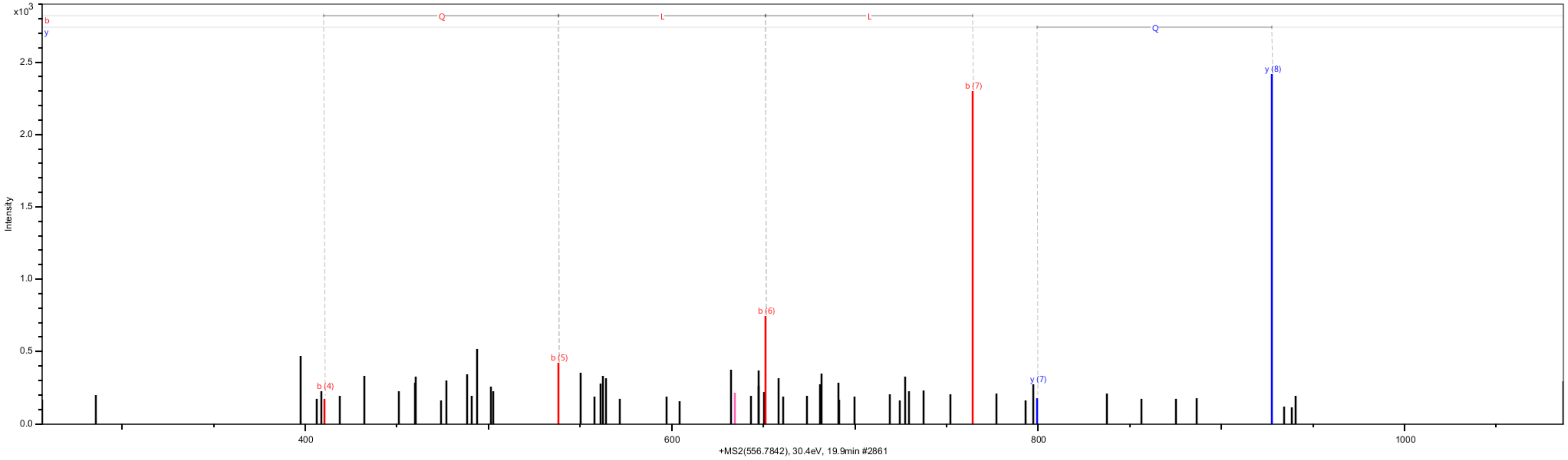

ACO11_HUMAN

Accession: ACO11_HUMAN
Database: SwissProt
MW [kDa] / pI: 68.4 / 8.7

Score: 15.0
Seq. Coverage [%]: 1.3
No. of Peptides: 1

Peptide Match

Sequence: R.LVYADTIK.D
Score: 15
Modifications:

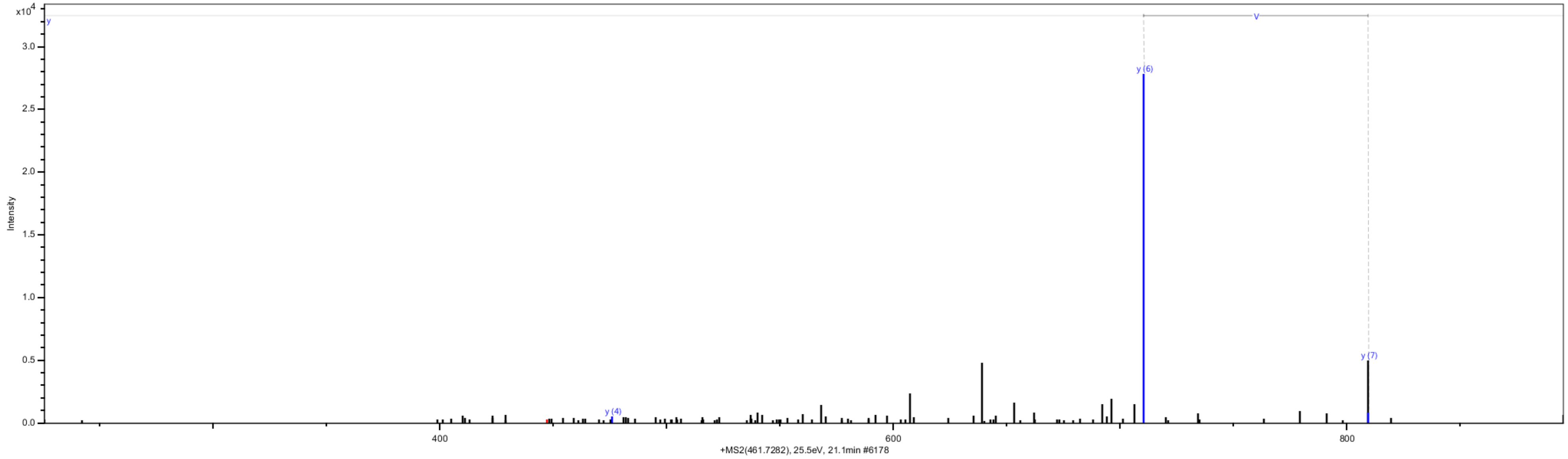

PZRN3_HUMAN

**Accession:**	PZRN3_HUMAN	**Score:**	15.7
**Database:**	SwissProt	**Seq. Coverage [%]:**	0.8
**MW [kDa] / pI:**	119.5 / 5.7	**No. of Peptides:**	1

Peptide Match

**Sequence:**	R.CVAAPPGGK.G	**Score:**	15.7
**Modifications:**	Carbamidomethyl: 1		

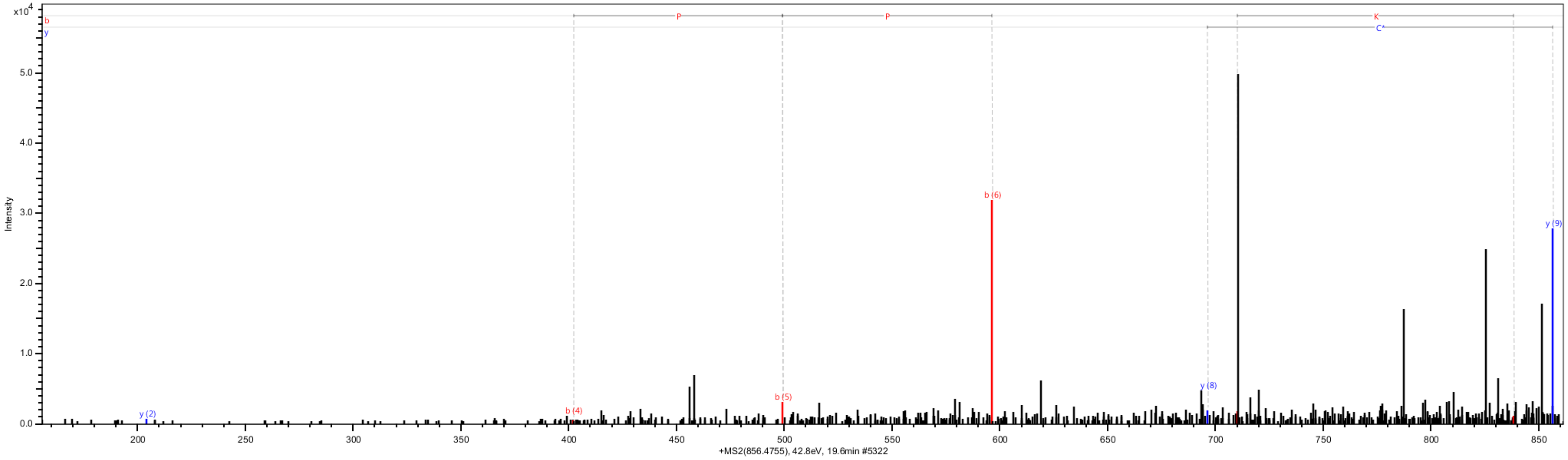

LAR4B_HUMAN

**Accession:**	LAR4B_HUMAN	**Score:**	15.7
**Database:**	SwissProt	**Seq. Coverage [%]:**	1.9
**MW [kDa] / pI:**	80.5 / 6.5	**No. of Peptides:**	1

Peptide Match

**Sequence:**	R.TSKEPPSSPLQPQK.E	**Score:**	15.7
**Modifications:**			

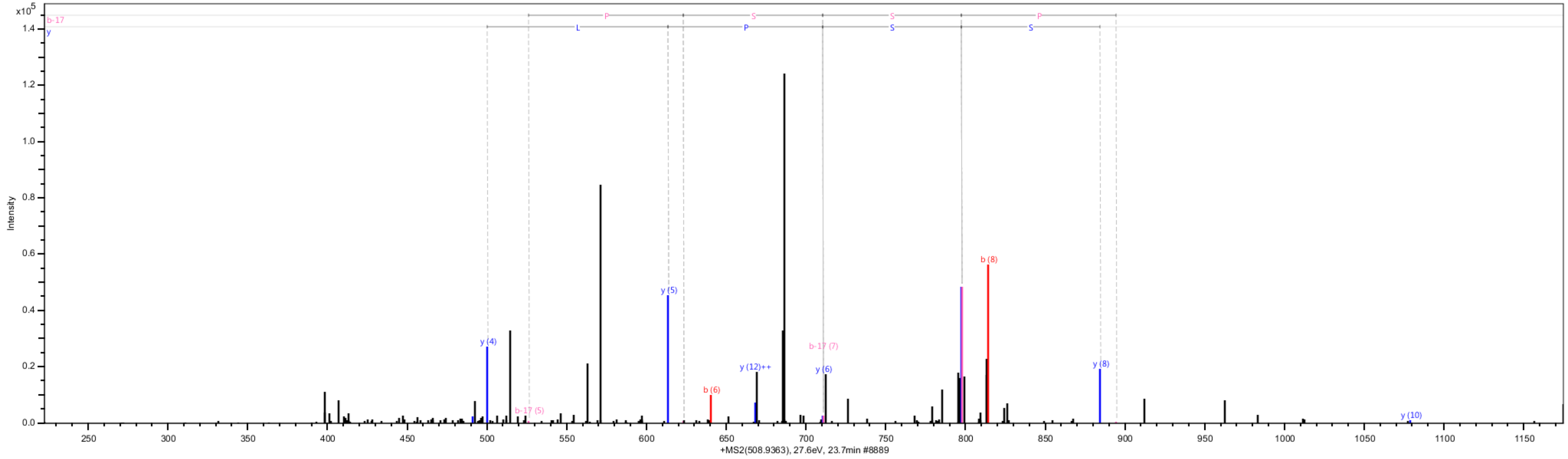

SIA4C_HUMAN

Accession: SIA4C_HUMAN
Database: SwissProt
MW [kDa] / pI: 38.0 / 9.4

Score: 15.7
Seq. Coverage [%]: 3.0
No. of Peptides: 1

Peptide Match

Sequence: K.LLSLPMQQPR.K
Score: 15.7
Modifications: Oxidation: 6

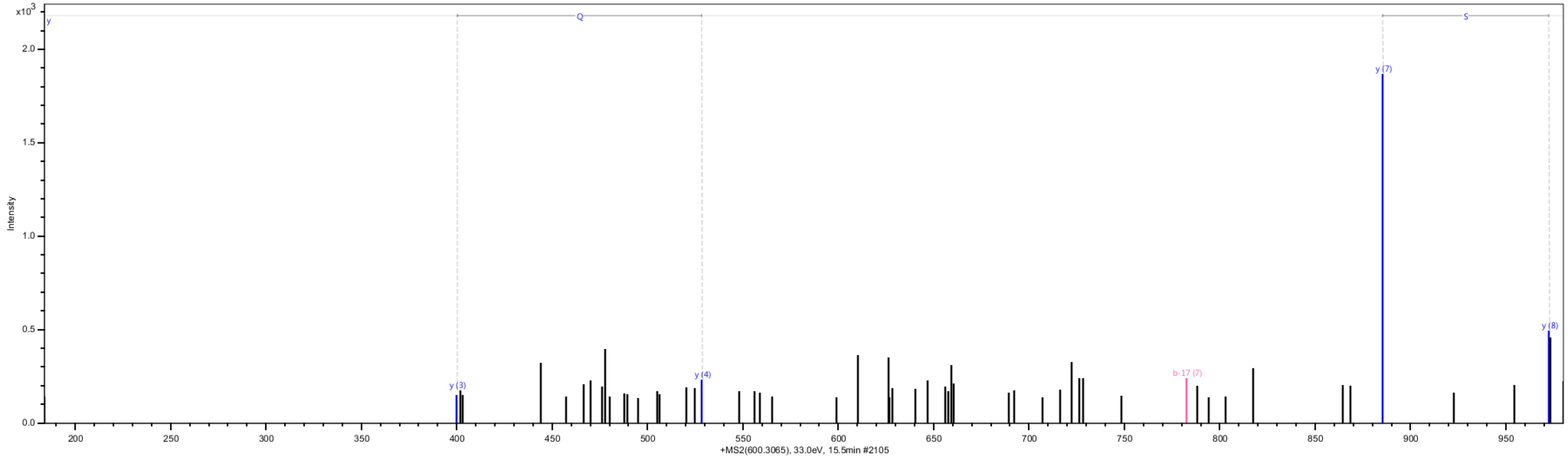

F86C2_HUMAN

**Accession:**	F86C2_HUMAN	**Score:**	15.7
**Database:**	SwissProt	**Seq. Coverage [%]:**	11.5
**MW [kDa] / pI:**	18.5 / 8.2	**No. of Peptides:**	1

Peptide Match

**Sequence:**	R.WSGSCGGWLPAGSTSGLLK.S	**Score:**	15.7
**Modifications:**	Carbamidomethyl: 5		

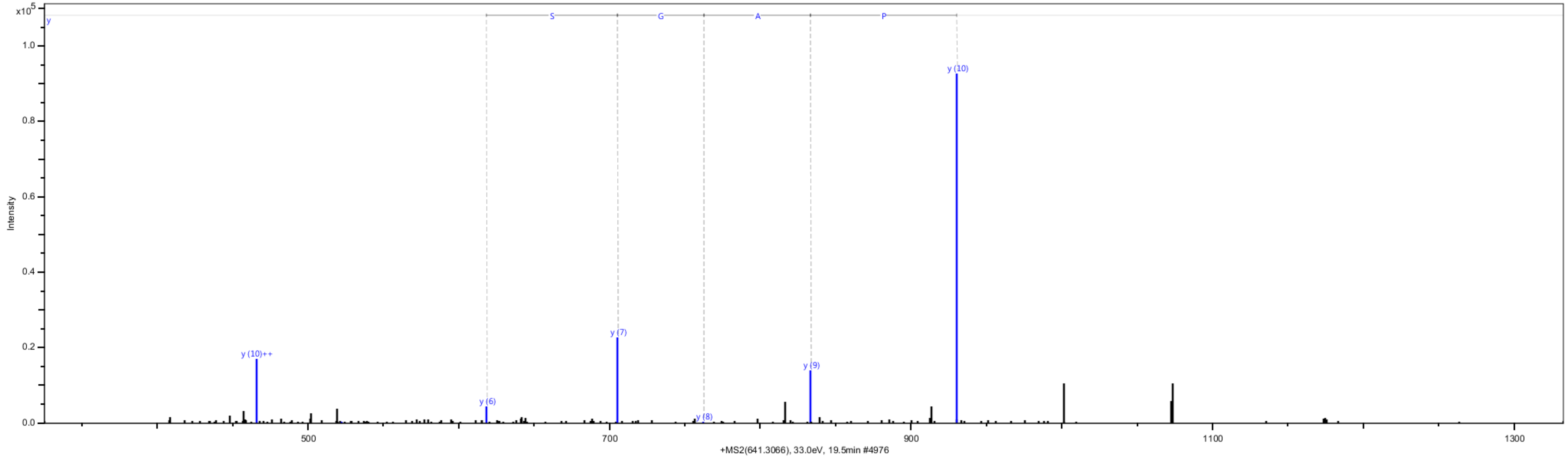

IDLC_HUMAN

Accession: IDLC_HUMAN
Database: SwissProt
MW [kDa] / pI: 29.6 / 8.7

Score: 15.7
Seq. Coverage [%]: 6.6
No. of Peptides: 1

Peptide Match

Sequence: K.VSPQQPGPSGSAPQPPK.T
Score: 15.6
Modifications:

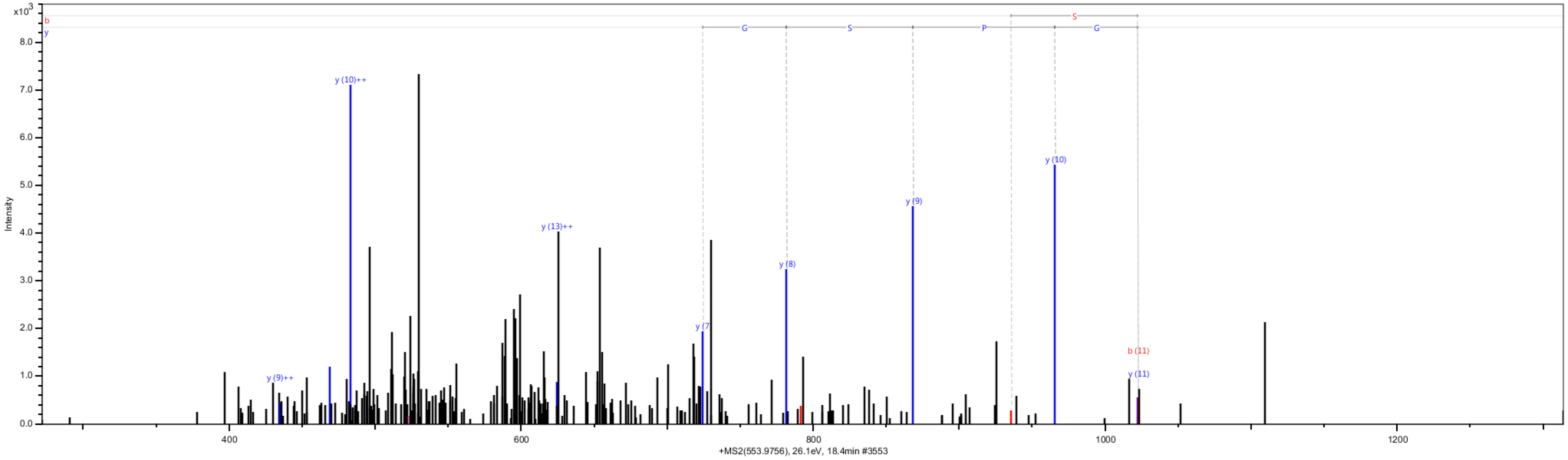

MYO5B_HUMAN

Accession: MYO5B_HUMAN
Database: SwissProt
MW [kDa] / pI: 213.5 / 6.8
Score: 15.7
Seq. Coverage [%]: 0.5
No. of Peptides: 1

Peptide Match

Sequence: R.SAELTKDYK.E
Score: 15.7
Modifications:

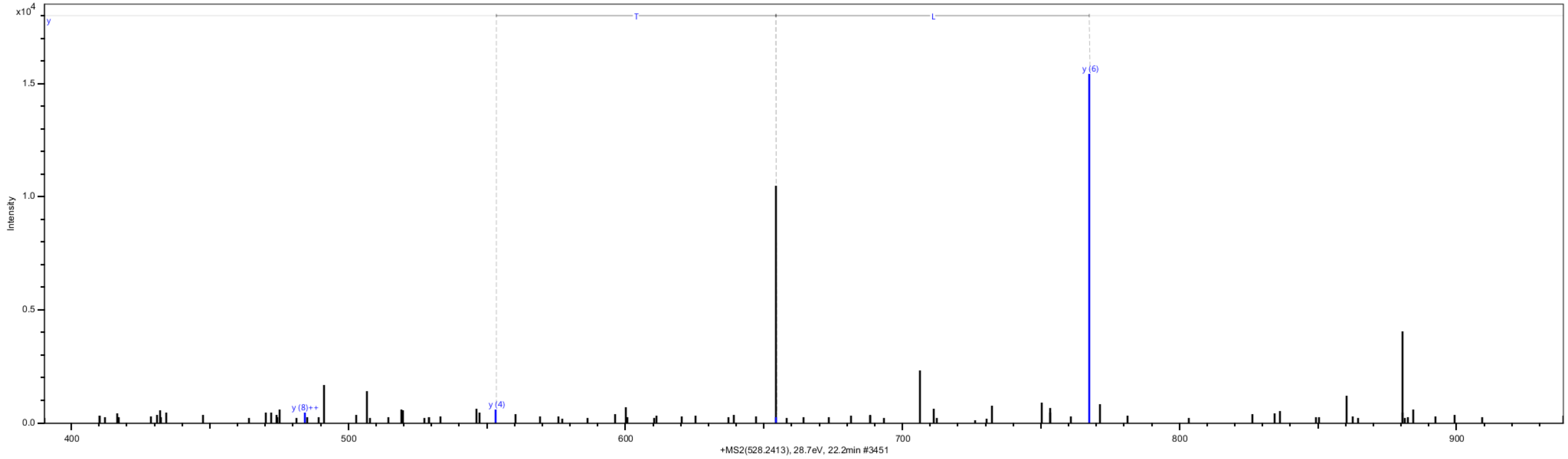

MAGD2_HUMAN

Accession: MAGD2_HUMAN
Database: SwissProt
MW [kDa] / pI: 64.9 / 9.3
Score: 15.7
Seq. Coverage [%]: 1.8
No. of Peptides: 1

Peptide Match

Sequence: R.SSEAVIWEVLR.K
Score: 15.7
Modifications:

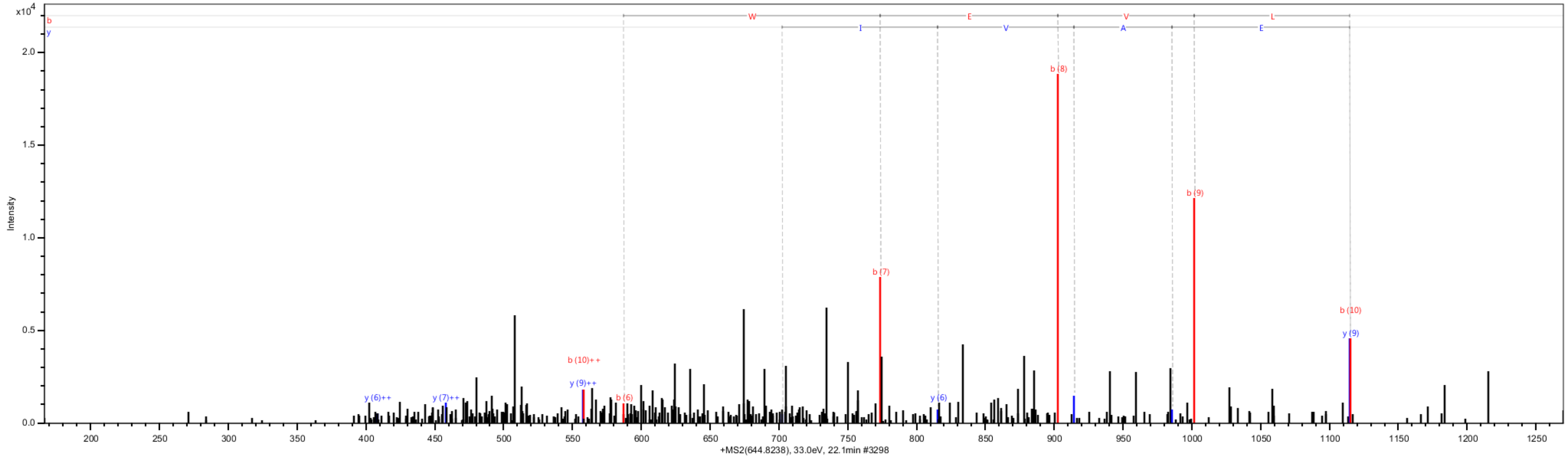

REV1_HUMAN

**Accession:**	REV1_HUMAN	**Score:**	15.7
**Database:**	SwissProt	**Seq. Coverage [%]:**	0.8
**MW [kDa] / pI:**	138.2 / 8.8	**No. of Peptides:**	1

Peptide Match

**Sequence:**	K.DLEKLDLVIK.Y	**Score:**	15.7
**Modifications:**			

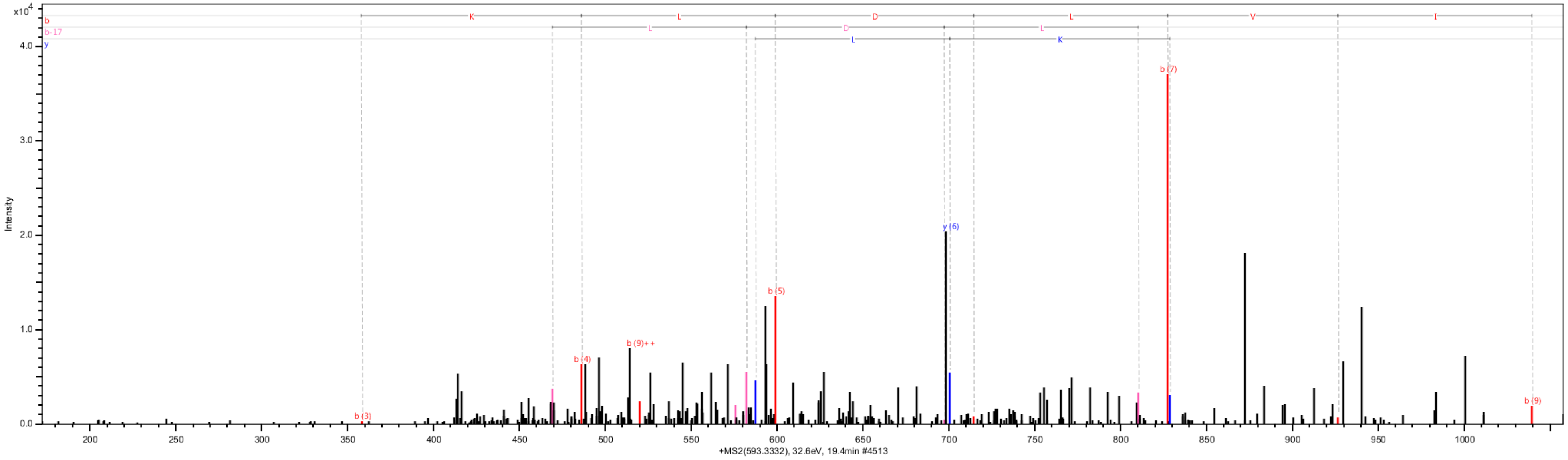

COCA1_HUMAN

Accession: COCA1_HUMAN
Database: SwissProt
MW [kDa] / pI: 332.9 / 5.4
Score: 15.8
Seq. Coverage [%]: 0.5
No. of Peptides: 1

Peptide Match

Sequence: R.NNVILQPLQPDTPYK.I
Score: 15.8
Modifications:

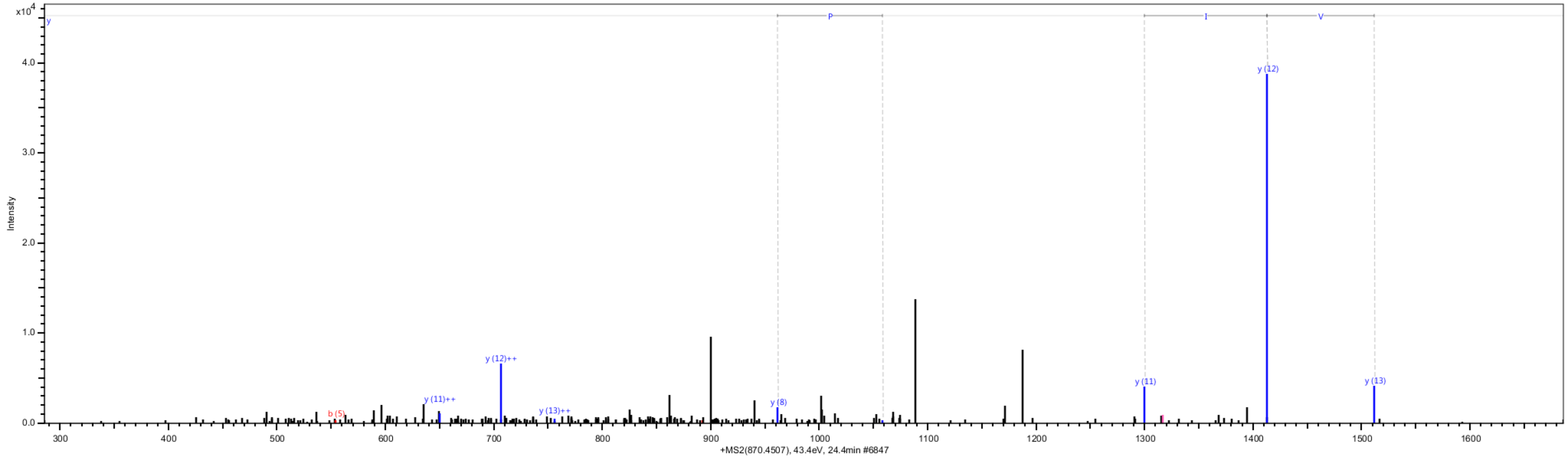

COBA2_HUMAN

Accession: COBA2_HUMAN
Database: SwissProt
MW [kDa] / pI: 171.7 / 5.9
Score: 15.8
Seq. Coverage [%]: 1.0
No. of Peptides: 1

Peptide Match

Sequence: K.GNEGPSGPPGPAGSPGER.G
Score: 15.8
Modifications:

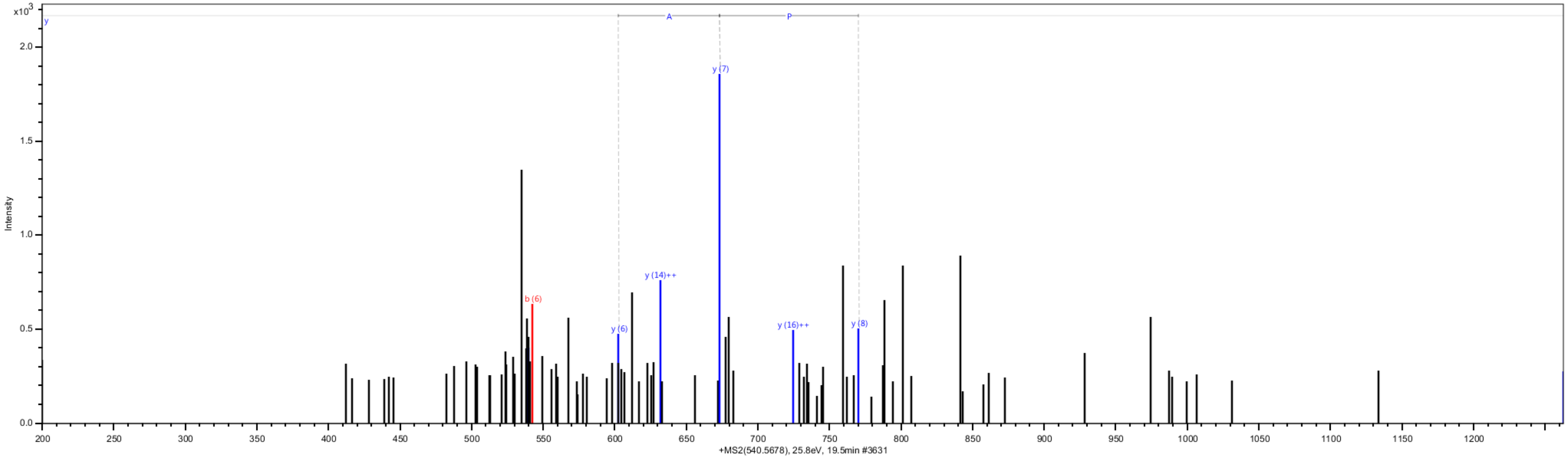

PDILT_HUMAN

**Accession:**	PDILT_HUMAN	**Score:**	15.8
**Database:**	SwissProt	**Seq. Coverage [%]:**	1.9
**MW [kDa] / pI:**	66.6 / 6.4	**No. of Peptides:**	1

Peptide Match

**Sequence:**	K.MLFPLLEELGR.K	**Score:**	15.8
**Modifications:**	Oxidation: 1		

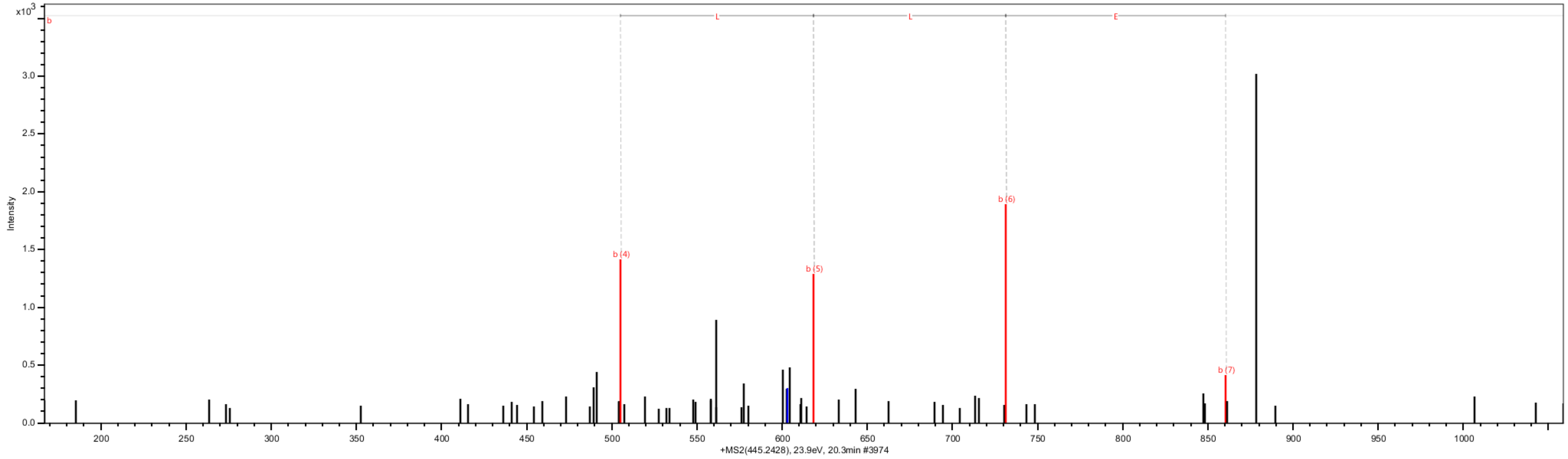

CP2CJ_HUMAN

Accession: CP2CJ_HUMAN
Database: SwissProt
MW [kDa] / pI: 55.9 / 7.1
Score: 15.8
Seq. Coverage [%]: 4.3
No. of Peptides: 1

Peptide Match

Sequence: K.EALIDLGEEFSGRGHFPLAER.A
Score: 15.8
Modifications:

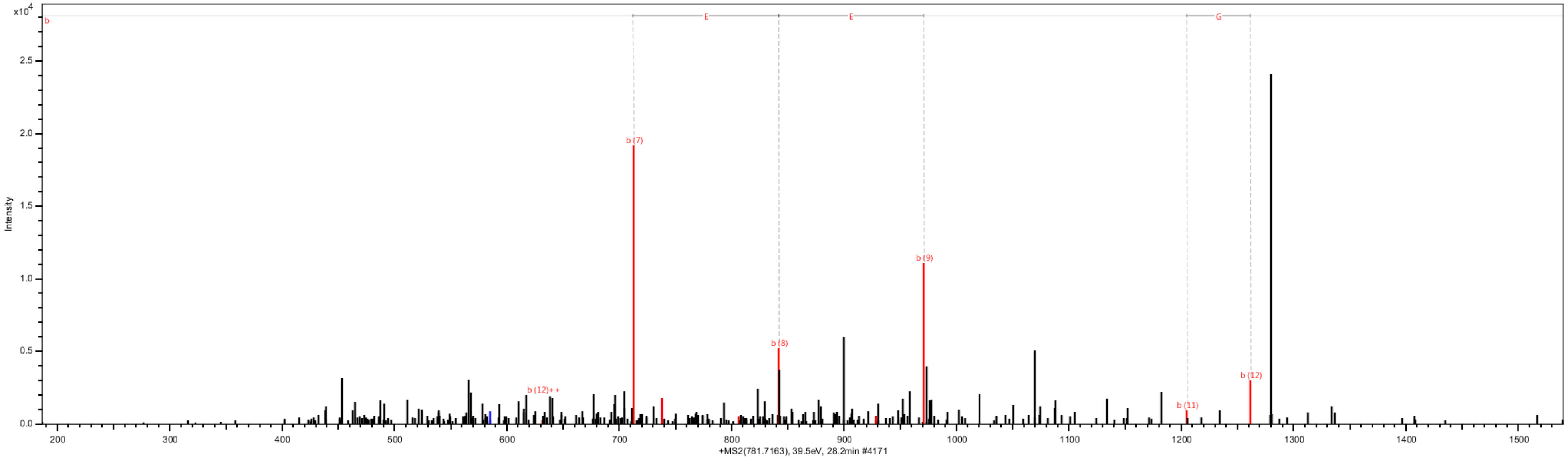

MUC12_HUMAN

**Accession:**	MUC12_HUMAN	**Score:**	15.7
**Database:**	SwissProt	**Seq. Coverage [%]:**	0.2
**MW [kDa] / pI:**	557.8 / 5.3	**No. of Peptides:**	1

Peptide Match

**Sequence:**	K.STILYSSSR.S	**Score:**	15.7
**Modifications:**			

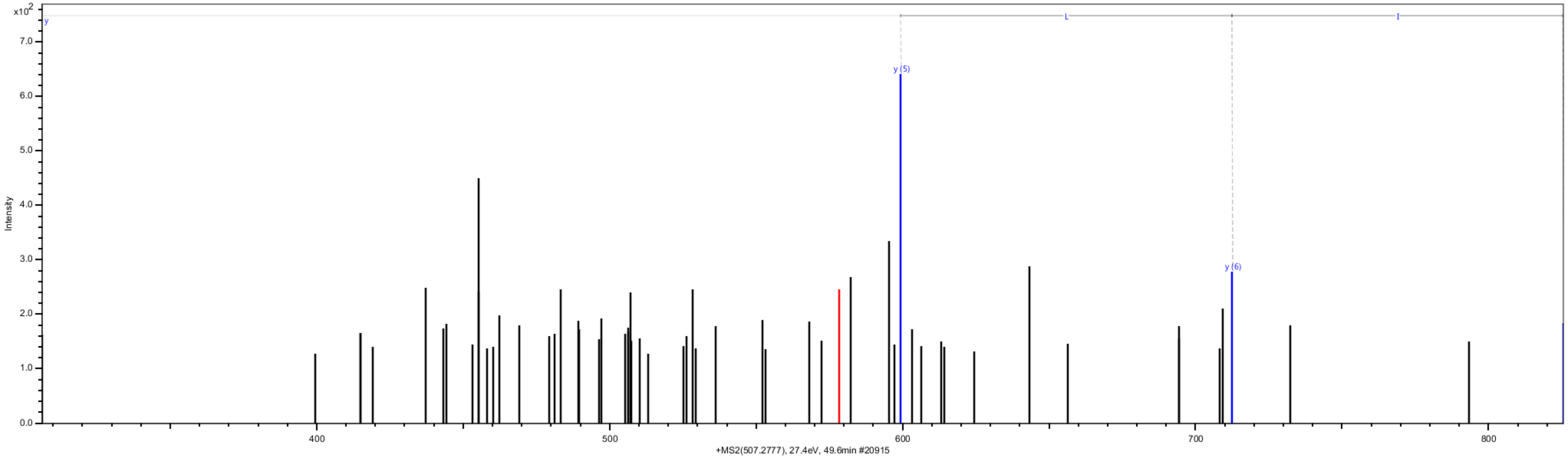

DYH10_HUMAN

**Accession:**	DYH10_HUMAN	**Score:**	15.7
**Database:**	SwissProt	**Seq. Coverage [%]:**	0.2
**MW [kDa] / pI:**	514.5 / 5.6	**No. of Peptides:**	1

Peptide Match

**Sequence:**	K.SVLVMAGELK.R	**Score:**	15.7
**Modifications:**	Oxidation: 5		

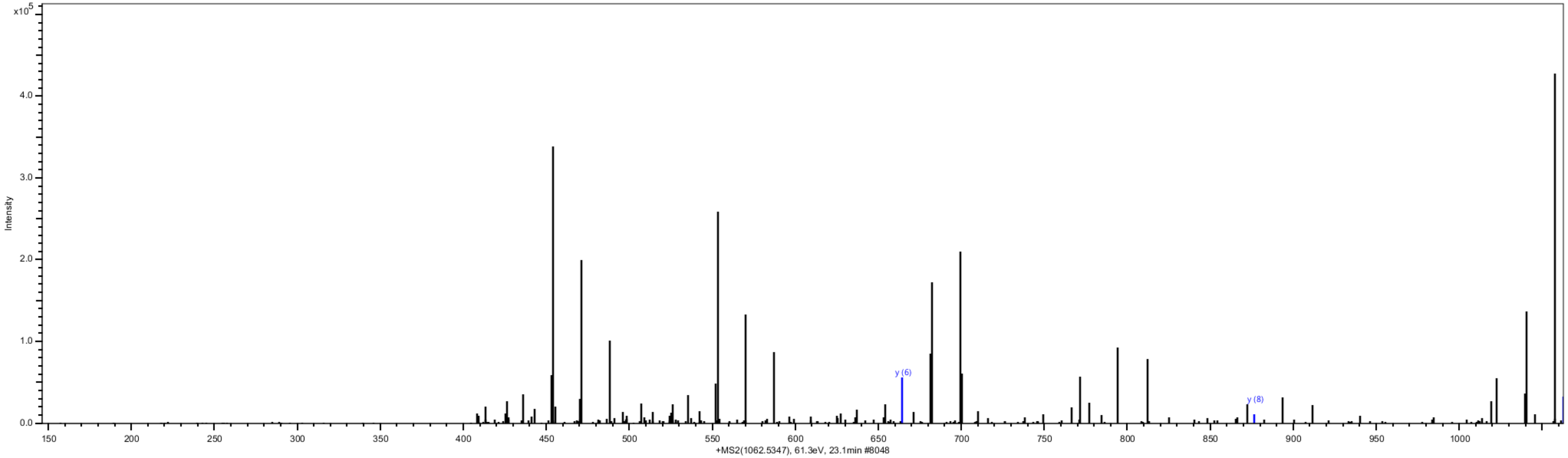

ZN816_HUMAN

Accession: ZN816_HUMAN
Database: SwissProt
MW [kDa] / pI: 75.7 / 9.4
Score: 15.7
Seq. Coverage [%]: 1.2
No. of Peptides: 1

Peptide Match

Sequence: K.GILAQHQR.V
Score: 15.7
Modifications:

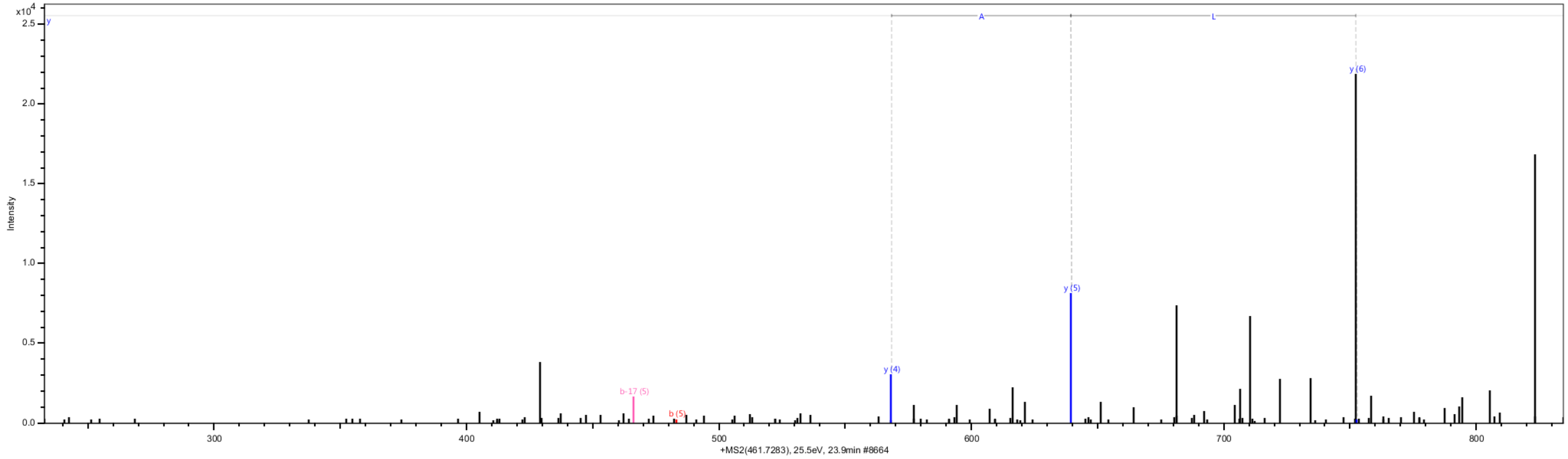

FA11_HUMAN

**Accession:**	FA11_HUMAN	**Score:**	15.7
**Database:**	SwissProt	**Seq. Coverage [%]:**	1.1
**MW [kDa] / pI:**	70.1 / 8.5	**No. of Peptides:**	1

Peptide Match

**Sequence:**	R.WFTCVLK.D	**Score:**	15.7
**Modifications:**	Carbamidomethyl: 4		

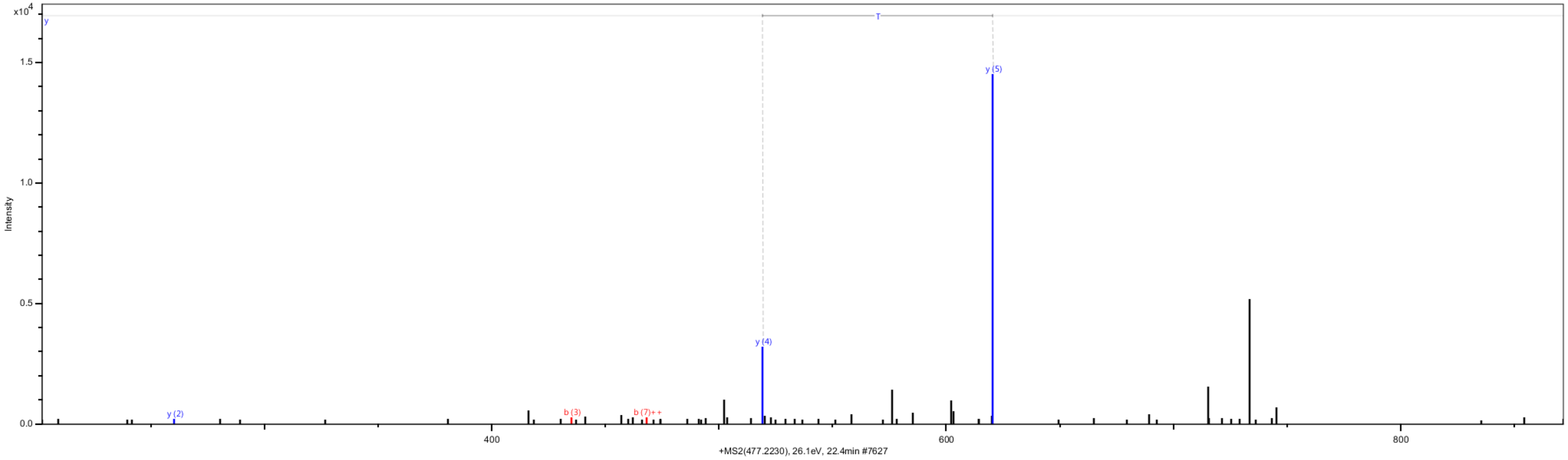

RBMX2_HUMAN

Accession: RBMX2_HUMAN
Database: SwissProt
MW [kDa] / pI: 37.3 / 9.8
Score: 15.9
Seq. Coverage [%]: 3.1
No. of Peptides: 1

Peptide Match

Sequence: R.ERESSNPSDR.W
Score: 15.8
Modifications:

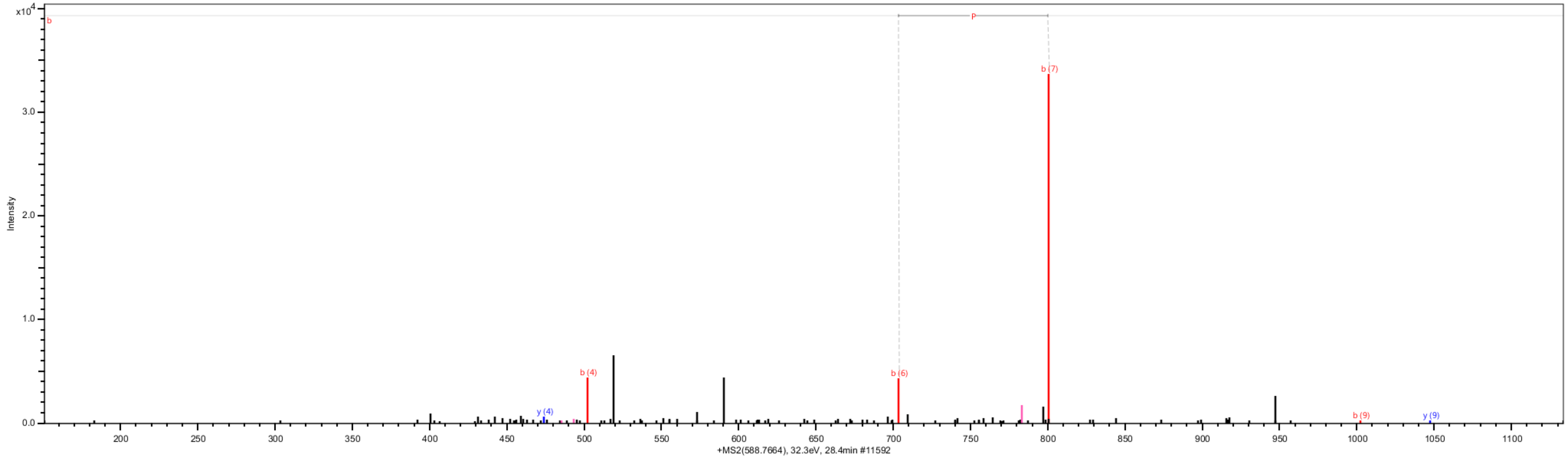

MCM8_HUMAN

Accession: MCM8_HUMAN
Database: SwissProt
MW [kDa] / pI: 93.6 / 7.8
Score: 15.8
Seq. Coverage [%]: 1.2
No. of Peptides: 1

Peptide Match

Sequence: K.GPKVYQLQTM.-
Score: 15.8
Modifications:

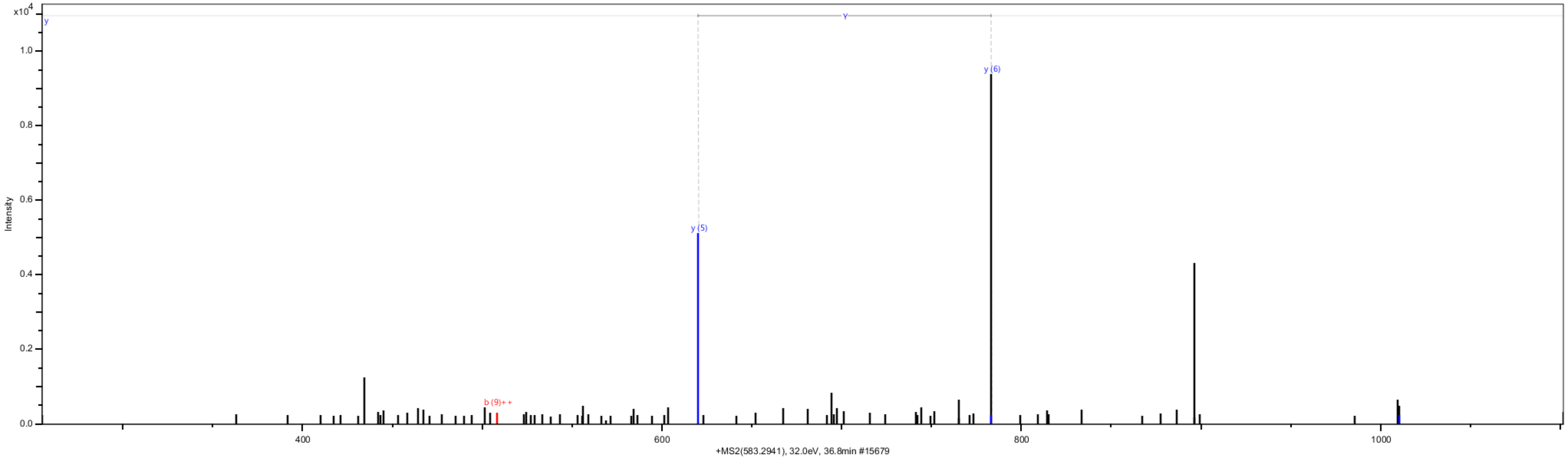

ARI5A_HUMAN

Accession: ARI5A_HUMAN | **Score:** 15.9
Database: SwissProt | **Seq. Coverage [%]:** 1.7
MW [kDa] / pI: 64.0 / 9.3 | **No. of Peptides:** 1

Peptide Match

Sequence: R.GDDGATERPK.K | **Score:** 15.9
Modifications:

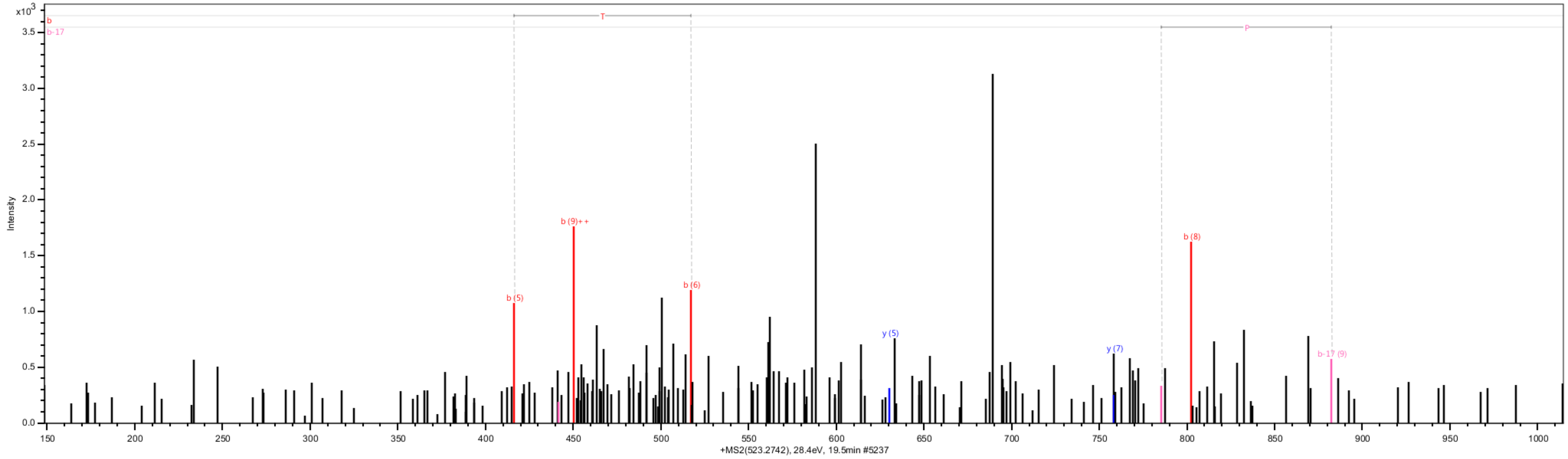

C1GLC_HUMAN

Accession: C1GLC_HUMAN
Database: SwissProt
MW [kDa] / pI: 36.4 / 6.4
Score: 15.9
Seq. Coverage [%]: 2.2
No. of Peptides: 1

Peptide Match

Sequence: K.QLAVCLK.Y
Score: 15.9
Modifications: Carbamidomethyl: 5

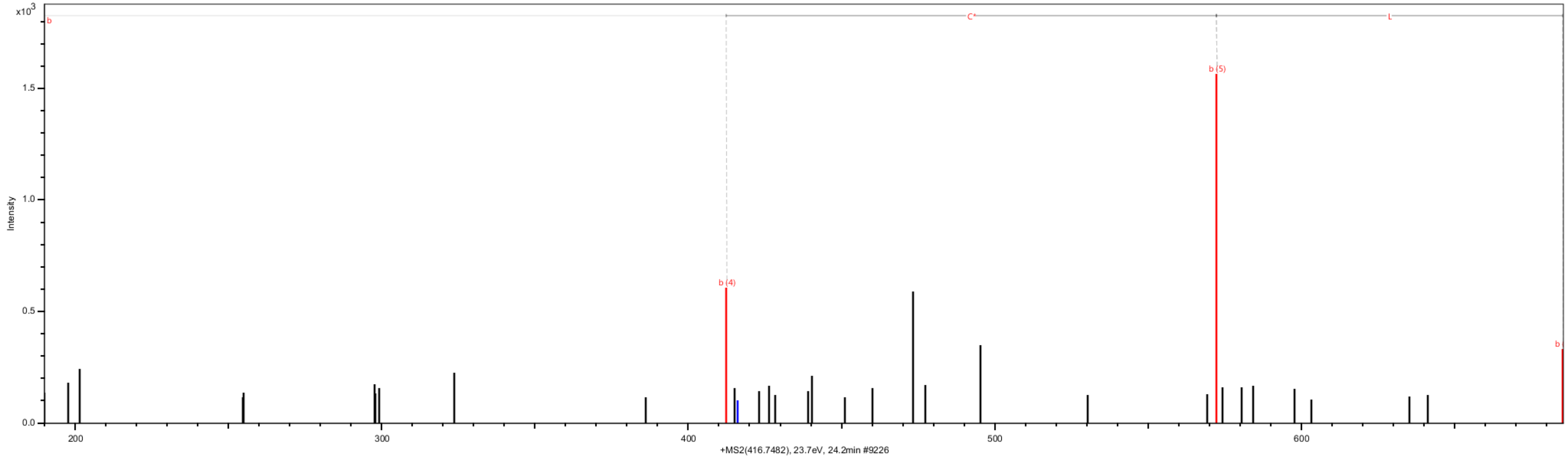

ZN473_HUMAN

Accession: ZN473_HUMAN
Database: SwissProt
MW [kDa] / pI: 100.1 / 8.6
Score: 15.8
Seq. Coverage [%]: 0.9
No. of Peptides: 1

Peptide Match

Sequence: K.QGFFVSGK.I
Score: 15.8
Modifications:

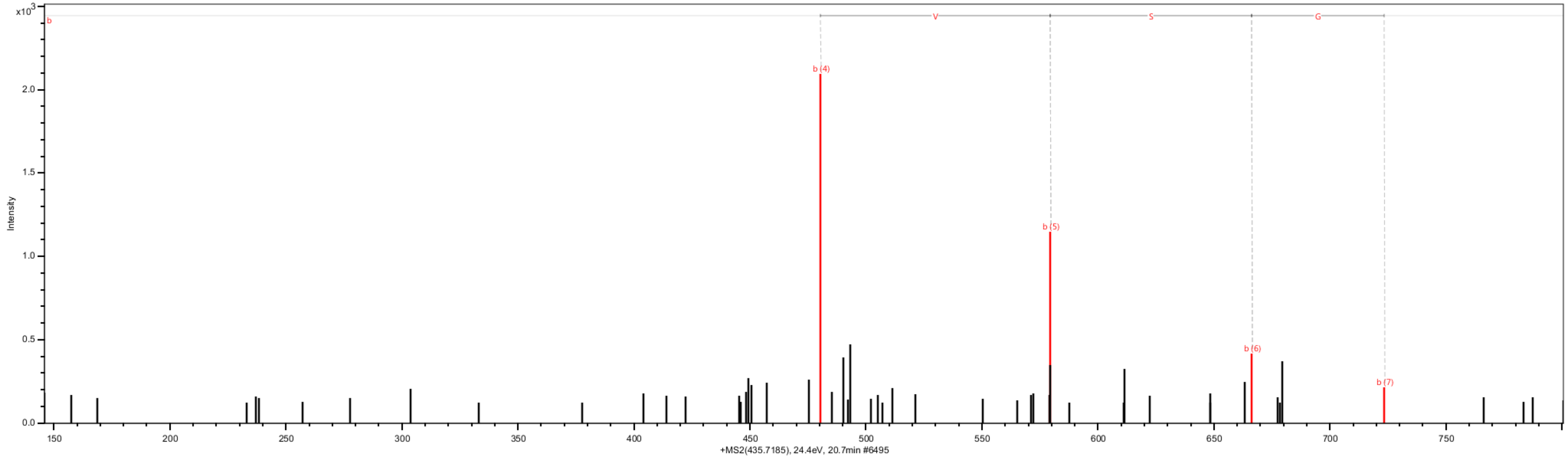

MAST3_HUMAN

**Accession:**	MAST3_HUMAN	**Score:**	15.8
**Database:**	SwissProt	**Seq. Coverage [%]:**	0.9
**MW [kDa] / pI:**	143.0 / 8.2	**No. of Peptides:**	1

Peptide Match

**Sequence:**	R.TRGPEAELVVMR.R	**Score:**	15.8
**Modifications:**	Oxidation: 11		

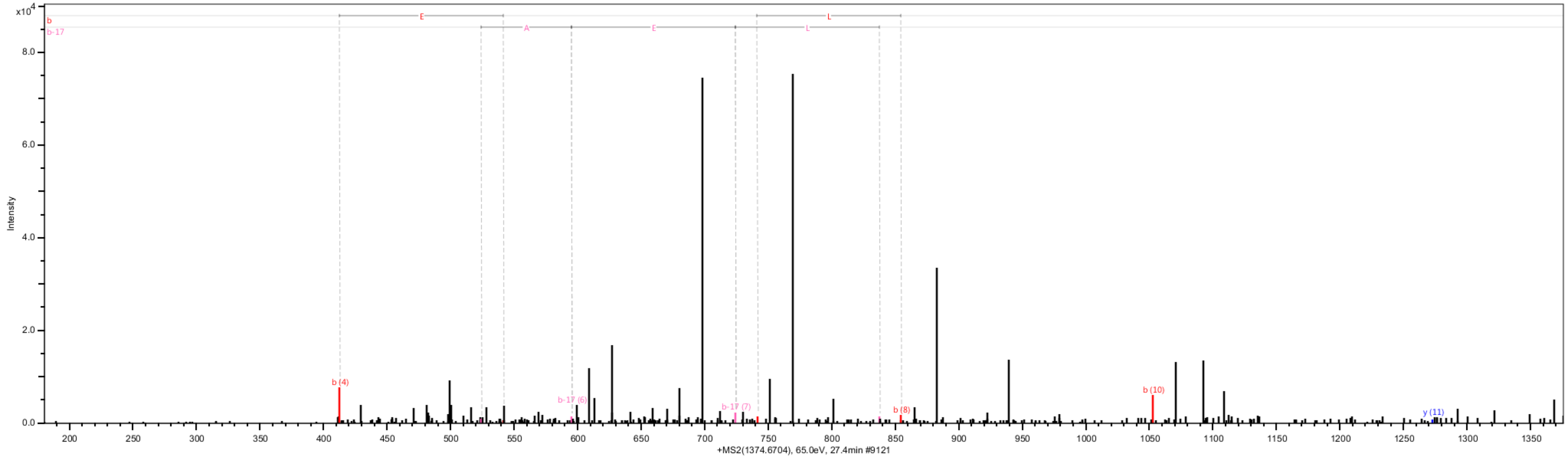

IDD_HUMAN

**Accession:**	IDD_HUMAN	**Score:**	15.8
**Database:**	SwissProt	**Seq. Coverage [%]:**	1.3
**MW [kDa] / pI:**	60.8 / 5.1	**No. of Peptides:**	1

Peptide Match

**Sequence:**	K.DPKECCK.F	**Score:**	15.8
**Modifications:**	Carbamidomethyl: 5, 6		

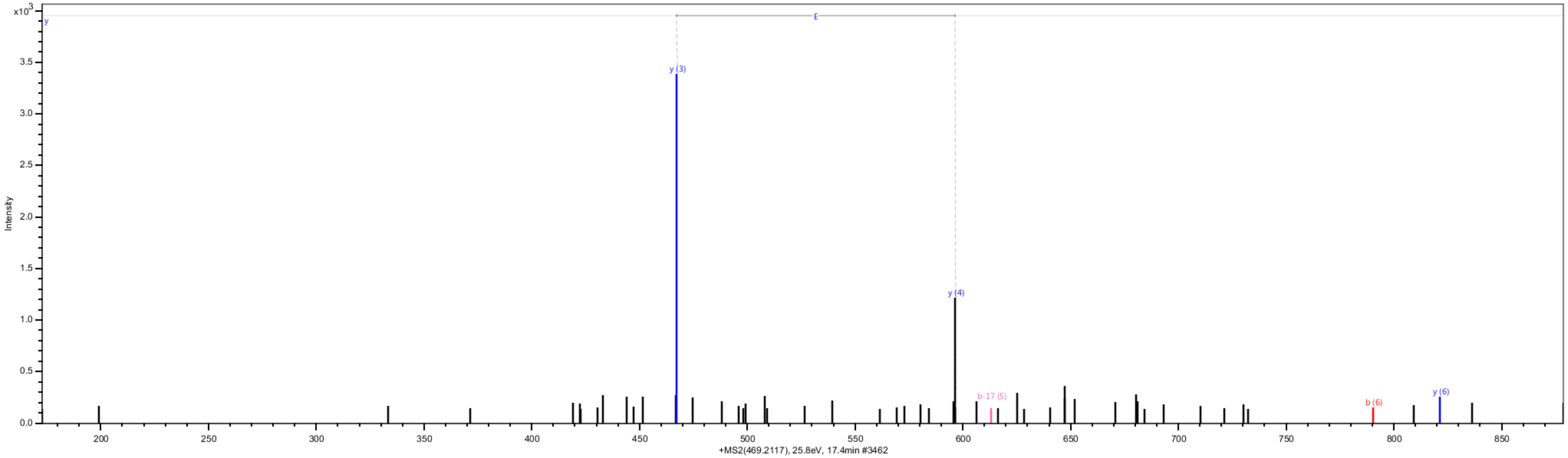

TTLL8_HUMAN

Accession: TTLL8_HUMAN
Database: SwissProt
MW [kDa] / pI: 94.6 / 8.5
Score: 15.8
Seq. Coverage [%]: 1.6
No. of Peptides: 1

Peptide Match

Sequence: R.SFCAAVLQGASFVR.L
Score: 15.8
Modifications: Carbamidomethyl: 3

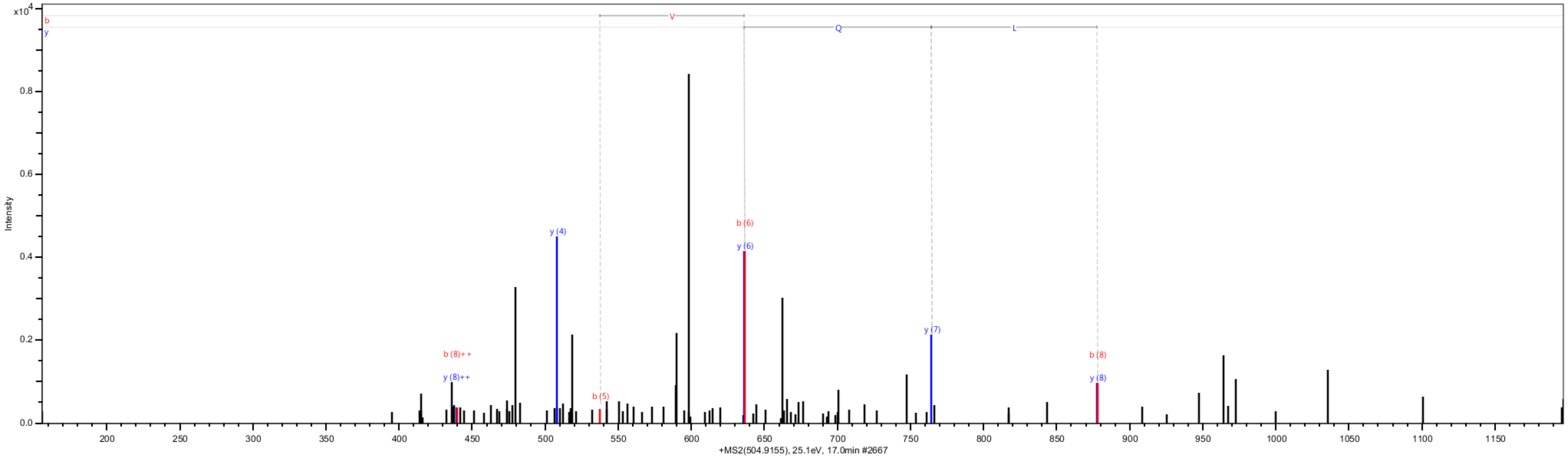

SMAG1_HUMAN

Accession: SMAG1_HUMAN
Database: SwissProt
MW [kDa] / pI: 79.4 / 8.6
Score: 16.0
Seq. Coverage [%]: 1.3
No. of Peptides: 1

Peptide Match

Sequence: K.TLLDISGYR.Q
Score: 16
Modifications:

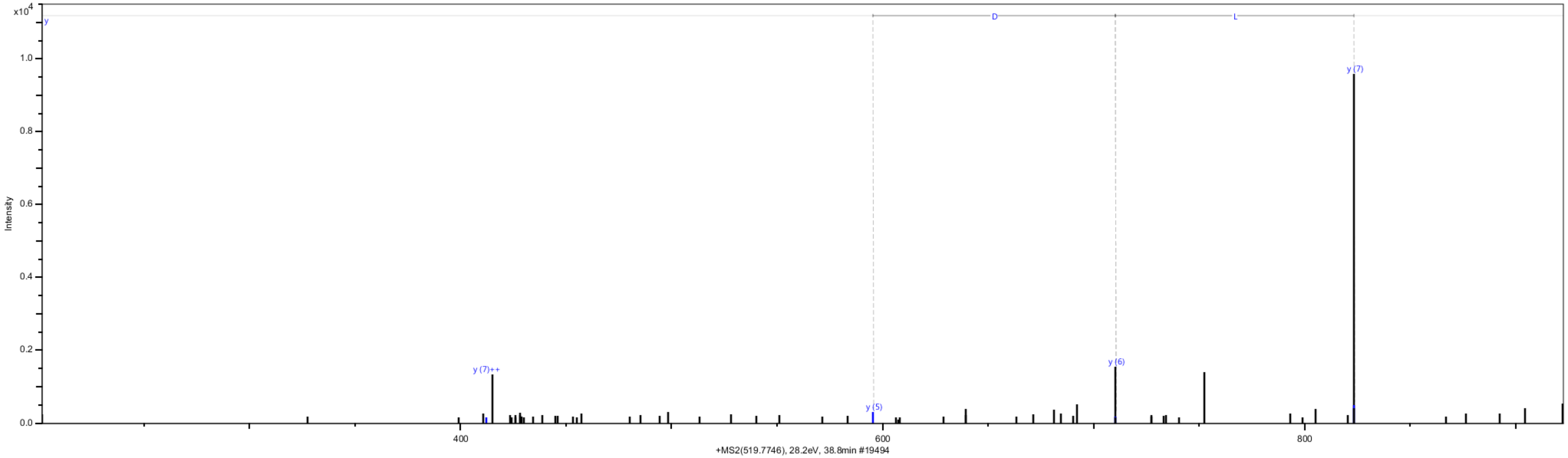

ICAL_HUMAN

Accession: ICAL_HUMAN
Database: SwissProt
MW [kDa] / pI: 76.5 / 5.0
Score: 15.9
Seq. Coverage [%]: 1.1
No. of Peptides: 1

Peptide Match

Sequence: K.LSDSLGQR.Q
Score: 15.9
Modifications:

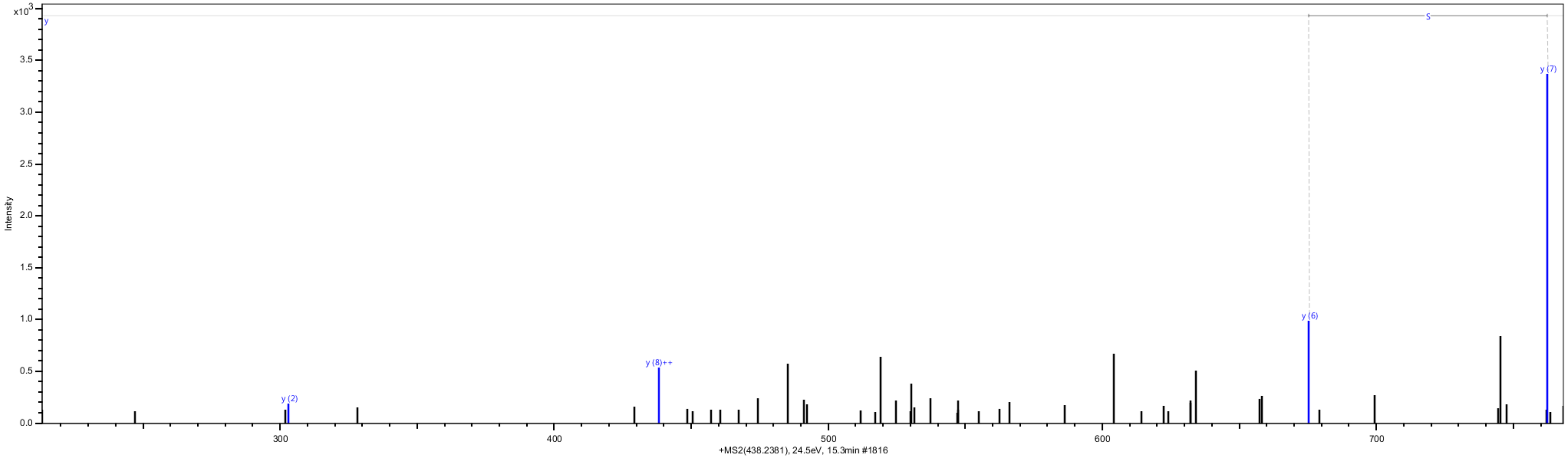

ZHX2_HUMAN

**Accession:**	ZHX2_HUMAN	**Score:**	16.0
**Database:**	SwissProt	**Seq. Coverage [%]:**	0.8
**MW [kDa] / pI:**	92.2 / 6.4	**No. of Peptides:**	1

Peptide Match

**Sequence:**	R.VETKLSR.R	**Score:**	16
**Modifications:**			

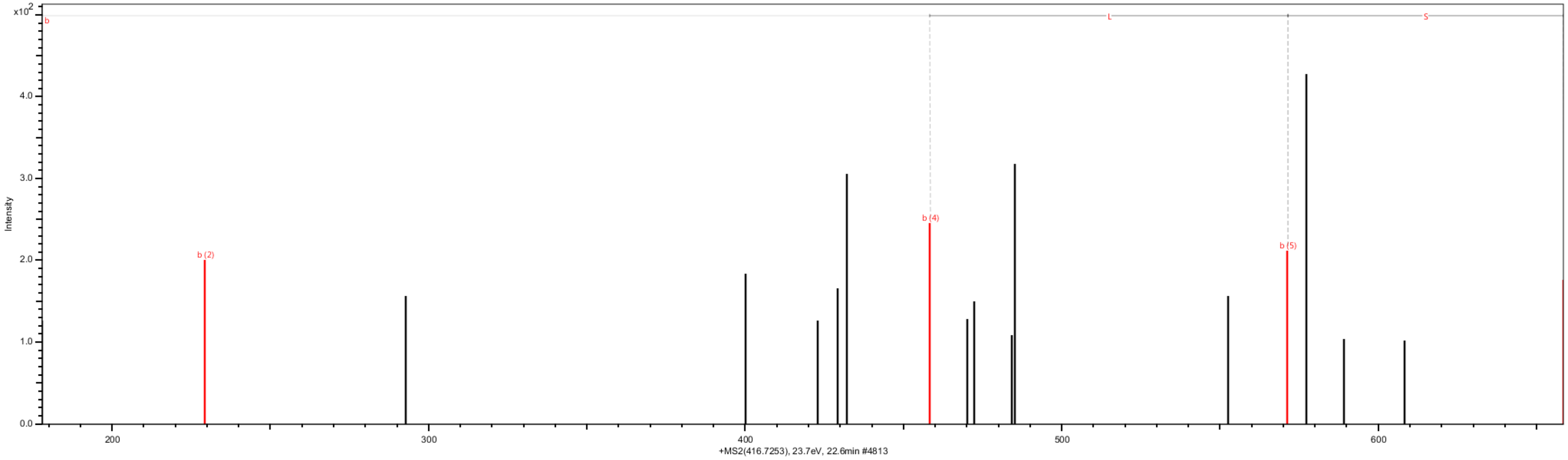

SO1B1_HUMAN

Accession: SO1B1_HUMAN
Database: SwissProt
MW [kDa] / pI: 76.4 / 8.9
Score: 16.0
Seq. Coverage [%]: 1.2
No. of Peptides: 1

Peptide Match

Sequence: R.ASPEIVGK.G
Score: 16
Modifications:

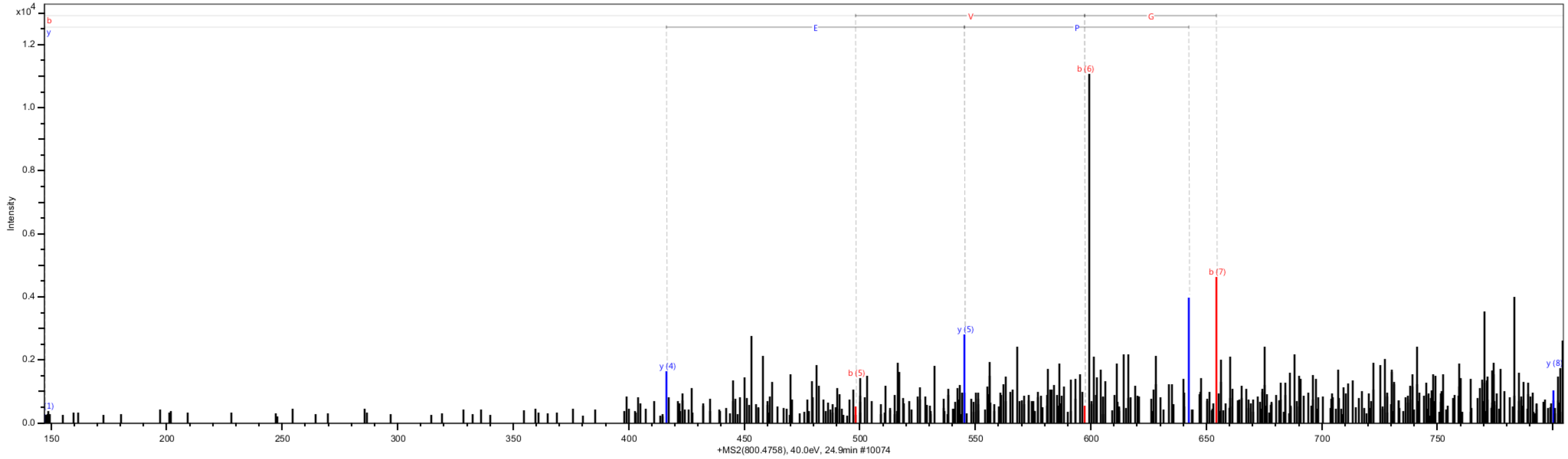

PLXC1_HUMAN

Accession: PLXC1_HUMAN
Database: SwissProt
MW [kDa] / pI: 175.6 / 7.8
Score: 15.9
Seq. Coverage [%]: 0.5
No. of Peptides: 1

Peptide Match

Sequence: K.CPKIQIIR.S
Score: 15.9
Modifications: Carbamidomethyl: 1

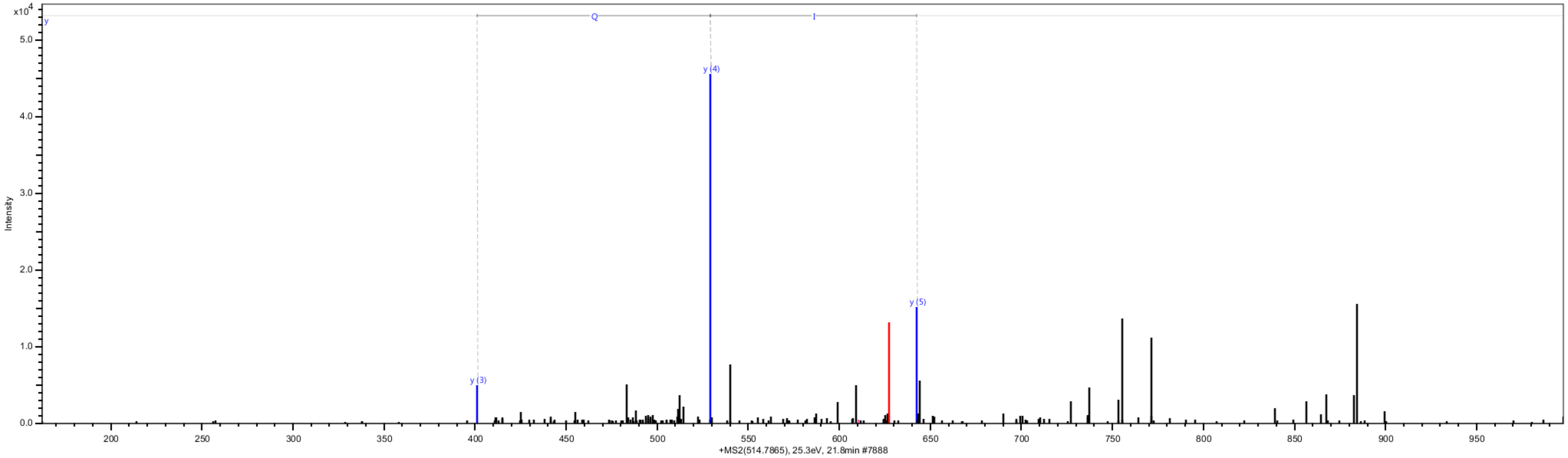

PKHN1_HUMAN

Accession: PKHN1_HUMAN
Database: SwissProt
MW [kDa] / pI: 66.4 / 9.1
Score: 15.9
Seq. Coverage [%]: 1.6
No. of Peptides: 1

Peptide Match

Sequence: K.GALQSRAAQR.H
Score: 15.9
Modifications:

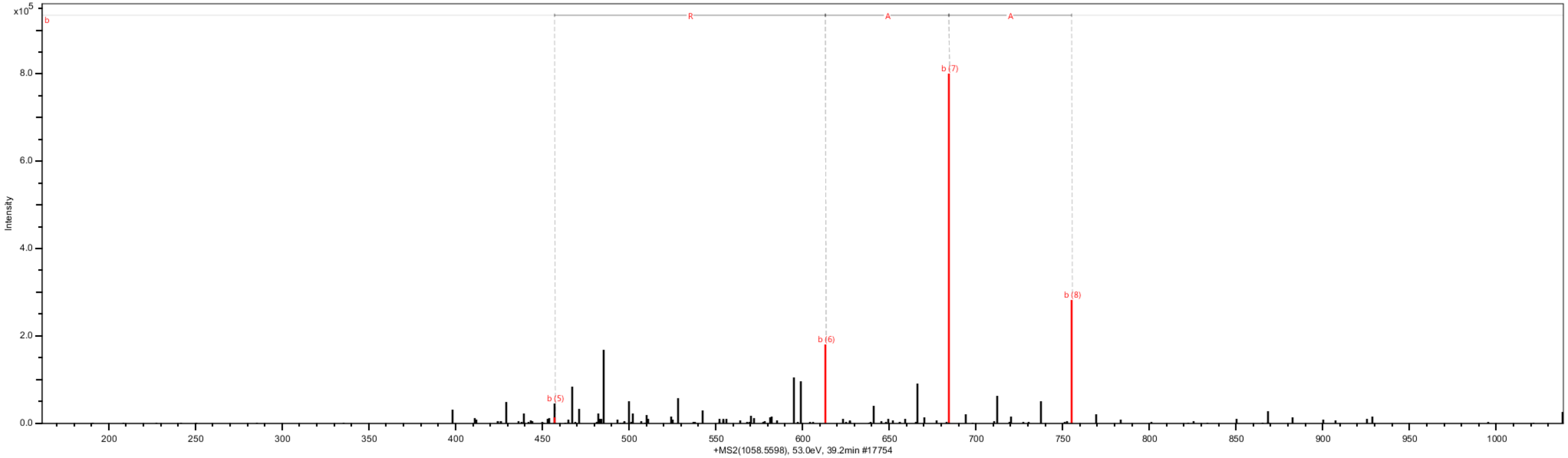

CBLB_HUMAN

**Accession:**	CBLB_HUMAN	**Score:**	15.9
**Database:**	SwissProt	**Seq. Coverage [%]:**	1.2
**MW [kDa] / pI:**	109.4 / 8.1	**No. of Peptides:**	1

Peptide Match

**Sequence:**	M.ANSMNGRNPGGR.G	**Score:**	15.9
**Modifications:**	Oxidation: 4		

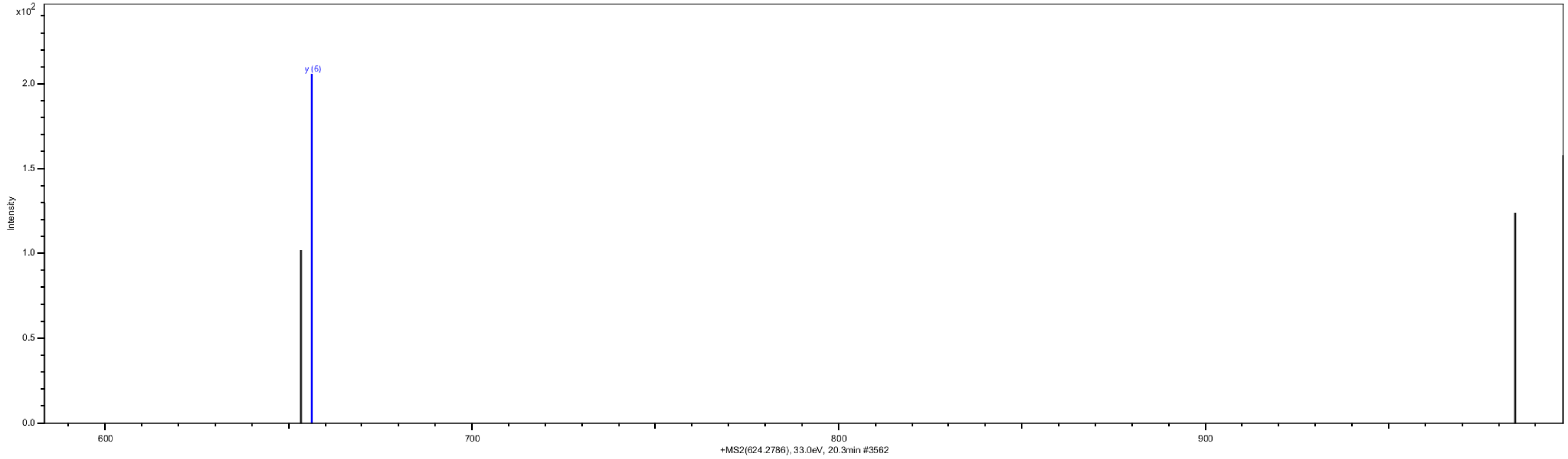

CP2C8_HUMAN

Accession: CP2C8_HUMAN
Database: SwissProt
MW [kDa] / pI: 55.8 / 8.8
Score: 15.9
Seq. Coverage [%]: 2.0
No. of Peptides: 1

Peptide Match

Sequence: K.GLGIISSNGK.R
Score: 15.9
Modifications:

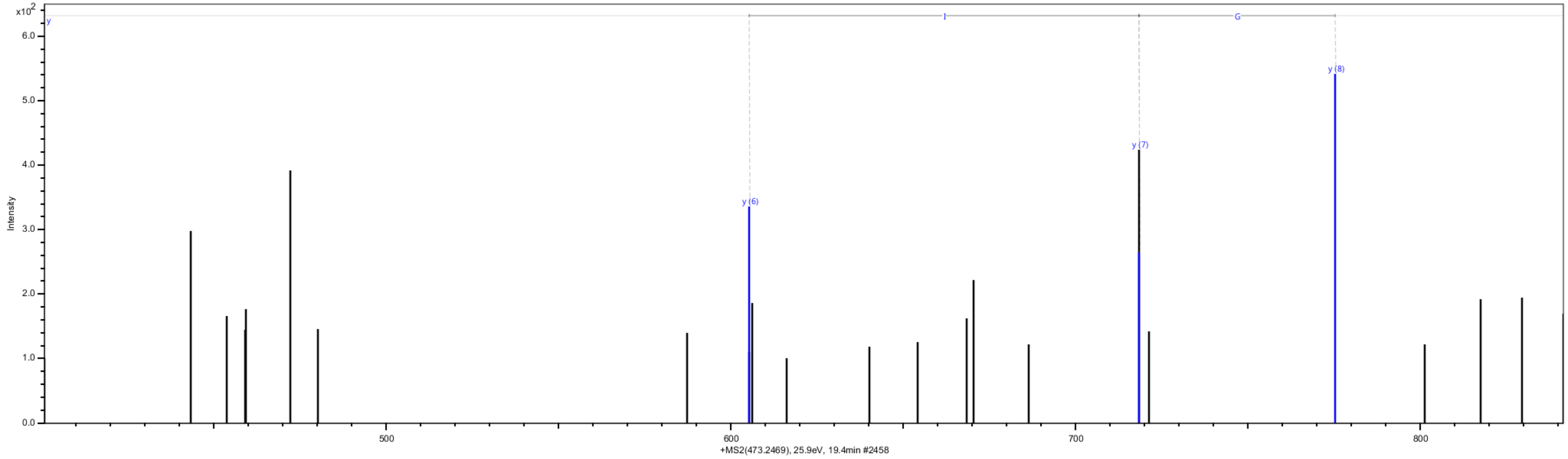

KVD12_HUMAN

**Accession:**	KVD12_HUMAN	**Score:**	15.5
**Database:**	SwissProt	**Seq. Coverage [%]:**	13.7
**MW [kDa] / pI:**	12.6 / 7.7	**No. of Peptides:**	1

Peptide Match

**Sequence:**	K.LLIYAASSLQSGVPSR.F	**Score:**	15.5
**Modifications:**			

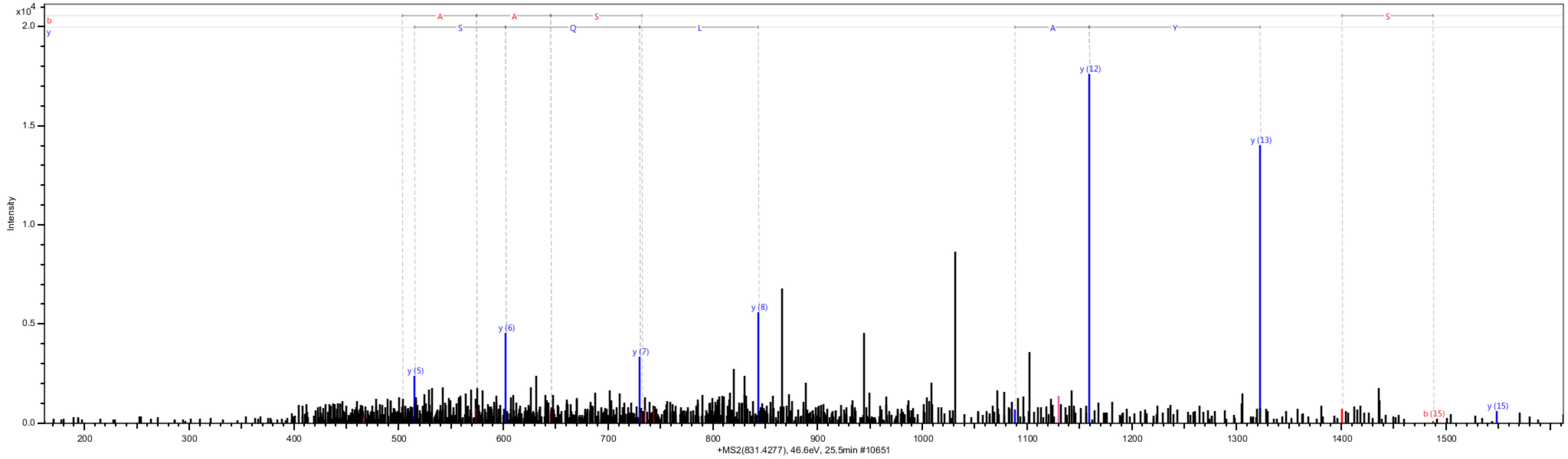

NAL13_HUMAN

**Accession:**	NAL13_HUMAN	**Score:**	15.5
**Database:**	SwissProt	**Seq. Coverage [%]:**	0.9
**MW [kDa] / pI:**	118.8 / 5.4	**No. of Peptides:**	1

Peptide Match

**Sequence:**	R.ELEDTLHCK.I	**Score:**	15.5
**Modifications:**	Carbamidomethyl: 8		

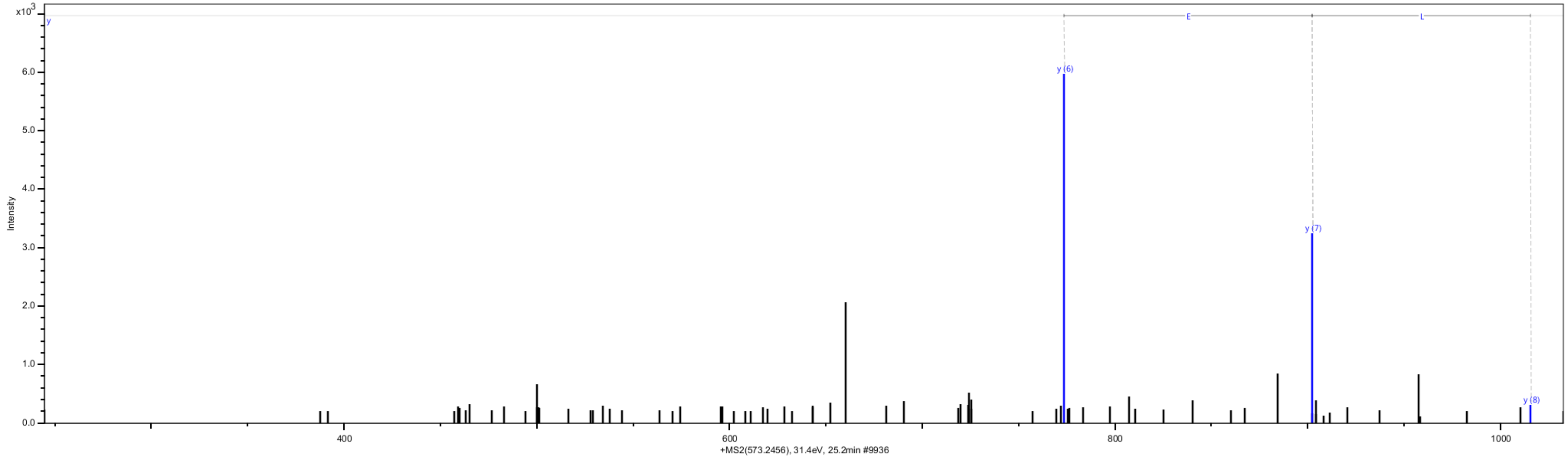

AL9A1_HUMAN

**Accession:**	AL9A1_HUMAN	**Score:**	15.5
**Database:**	SwissProt	**Seq. Coverage [%]:**	2.4
**MW [kDa] / pI:**	53.8 / 5.7	**No. of Peptides:**	1

Peptide Match

**Sequence:**	K.VSFTGSVPTGMK.I	**Score:**	15.5
**Modifications:**	Oxidation: 11		

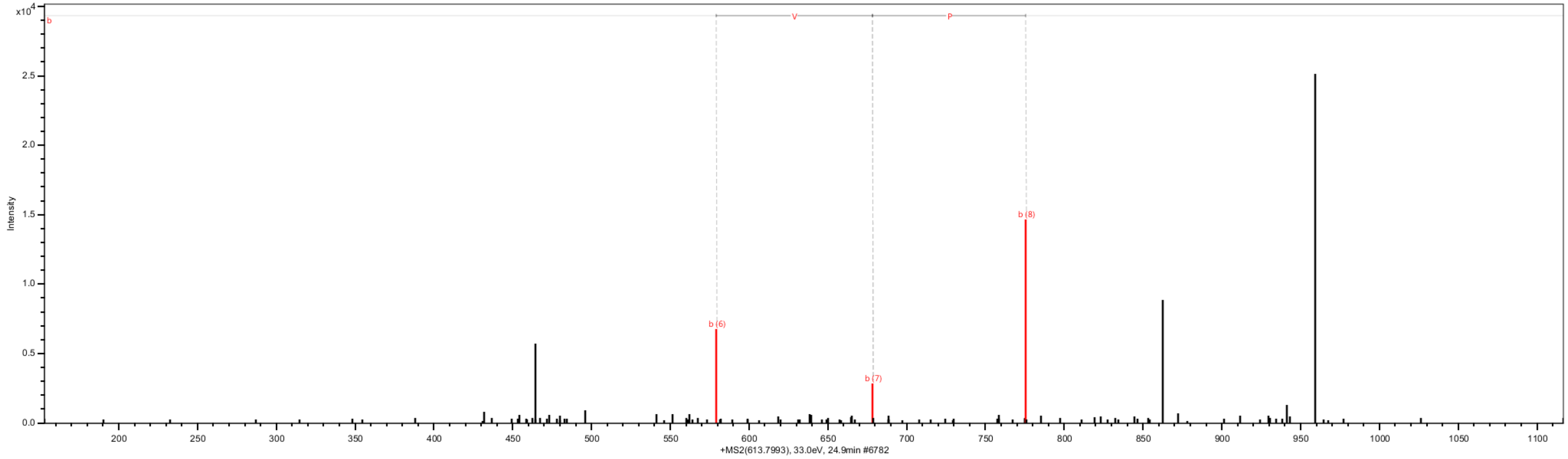

NHLC2_HUMAN

**Accession:**	NHLC2_HUMAN	**Score:**	15.5
**Database:**	SwissProt	**Seq. Coverage [%]:**	1.9
**MW [kDa] / pI:**	79.4 / 5.3	**No. of Peptides:**	1

Peptide Match

**Sequence:**	R.NLLYVADSYNHKIK.V	**Score:**	15.4
**Modifications:**			

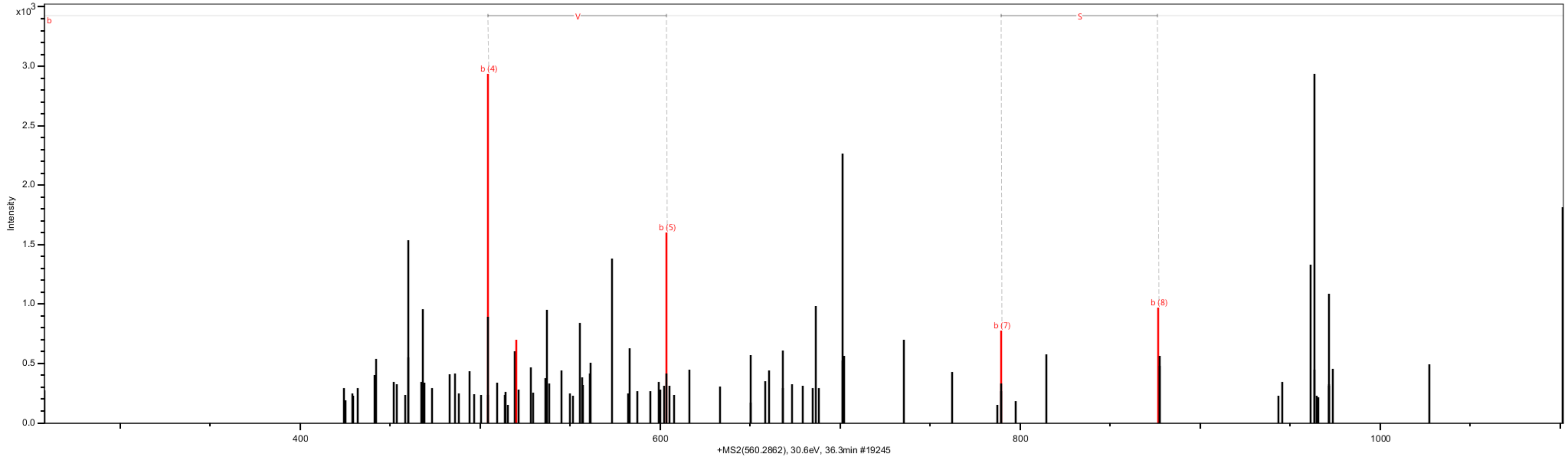

SC5A9_HUMAN

**Accession:**	SC5A9_HUMAN	**Score:**	15.5
**Database:**	SwissProt	**Seq. Coverage [%]:**	2.5
**MW [kDa] / pI:**	74.0 / 6.7	**No. of Peptides:**	1

Peptide Match

**Sequence:**	M.SKELAAMGPGASGDGVR.T	**Score:**	15.4
**Modifications:**			

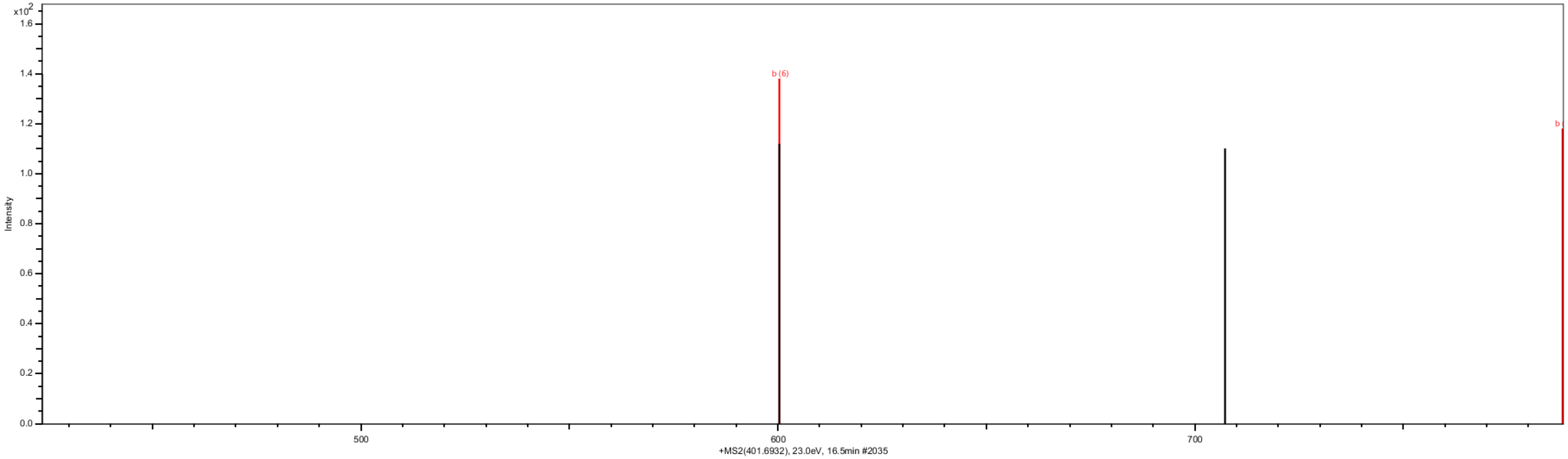

SFI1_HUMAN

Accession: SFI1_HUMAN
Database: SwissProt
MW [kDa] / pI: 147.6 / 10.8
Score: 15.5
Seq. Coverage [%]: 0.9
No. of Peptides: 1

Peptide Match

Sequence: K.EEWWVFQHEWK.L
Score: 15.5
Modifications:

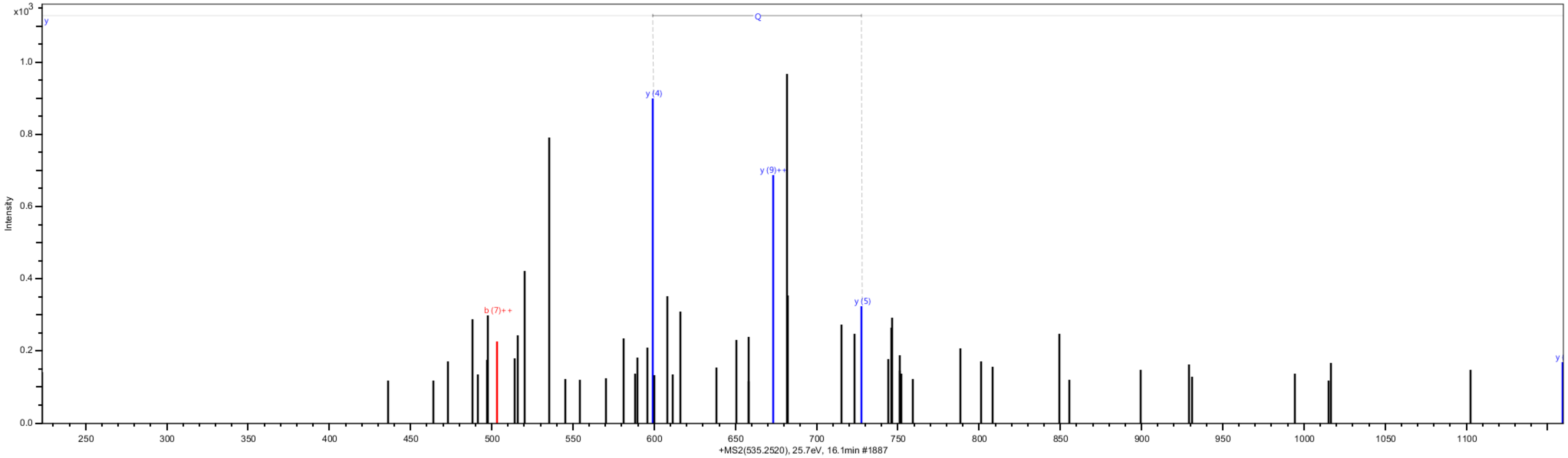

CHERP_HUMAN

**Accession:**	CHERP_HUMAN	**Score:**	15.5
**Database:**	SwissProt	**Seq. Coverage [%]:**	1.7
**MW [kDa] / pI:**	103.6 / 9.1	**No. of Peptides:**	1

Peptide Match

**Sequence:**	R.LHLIYLINDVLHHCQR.K	**Score:**	15.5
**Modifications:**	Carbamidomethyl: 14		

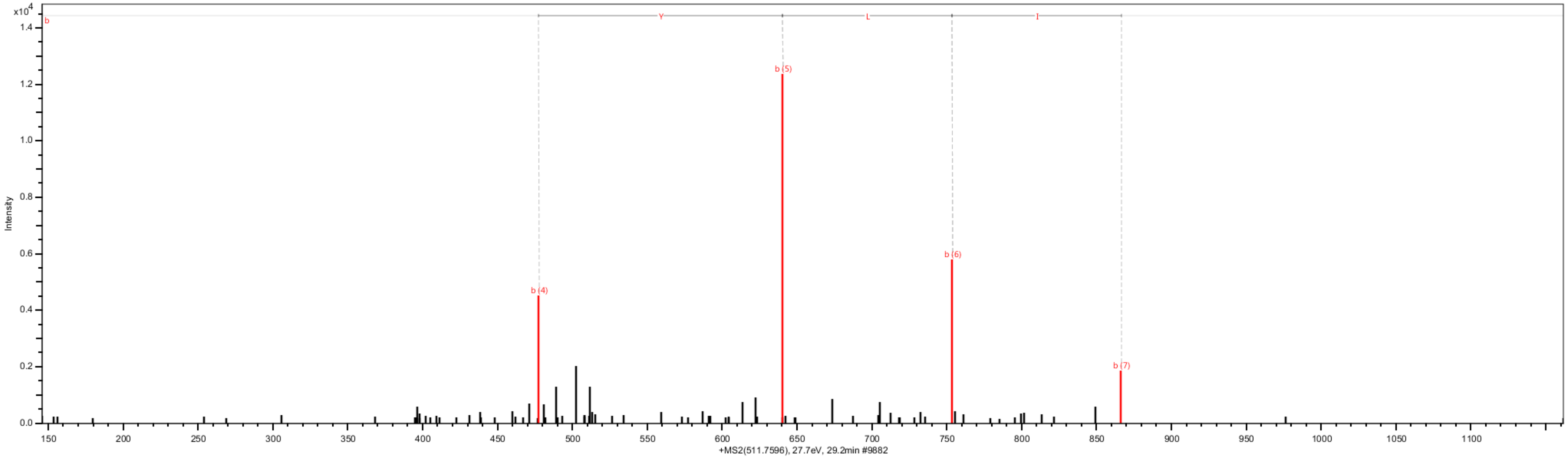

OAT_HUMAN

Accession: OAT_HUMAN
Database: SwissProt
MW [kDa] / pI: 48.5 / 6.6
Score: 15.5
Seq. Coverage [%]: 2.3
No. of Peptides: 1

Peptide Match

Sequence: K.LPSDVVTAVR.G
Score: 15.5
Modifications:

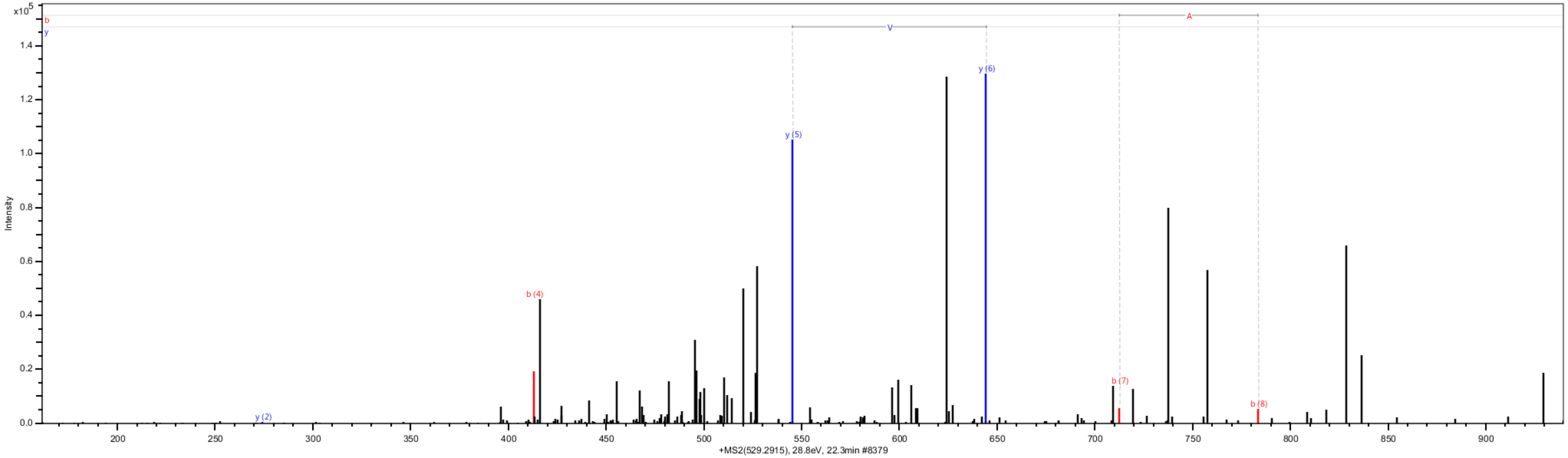

MET25_HUMAN

Accession: MET25_HUMAN
Database: SwissProt
MW [kDa] / pI: 68.2 / 6.7
Score: 15.5
Seq. Coverage [%]: 1.2
No. of Peptides: 1

Peptide Match

Sequence: K.DCYGITK.C
Score: 15.5
Modifications: Carbamidomethyl: 2

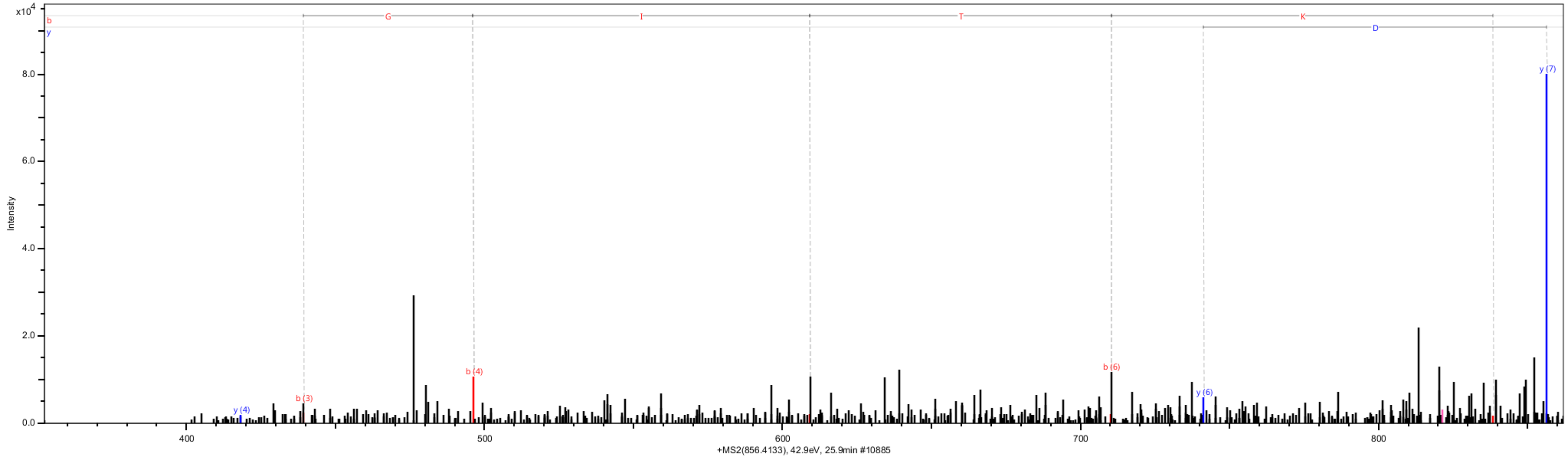

Spectrum Report
Combine Search_2020-10-19 07:53:31

CHK2_HUMAN

Accession: CHK2_HUMAN
Database: SwissProt
MW [kDa] / pI: 60.9 / 5.7
Score: 15.5
Seq. Coverage [%]: 1.7
No. of Peptides: 1

Peptide Match

Sequence: K.ILGETSLMR.T
Score: 15.5
Modifications: Oxidation: 8

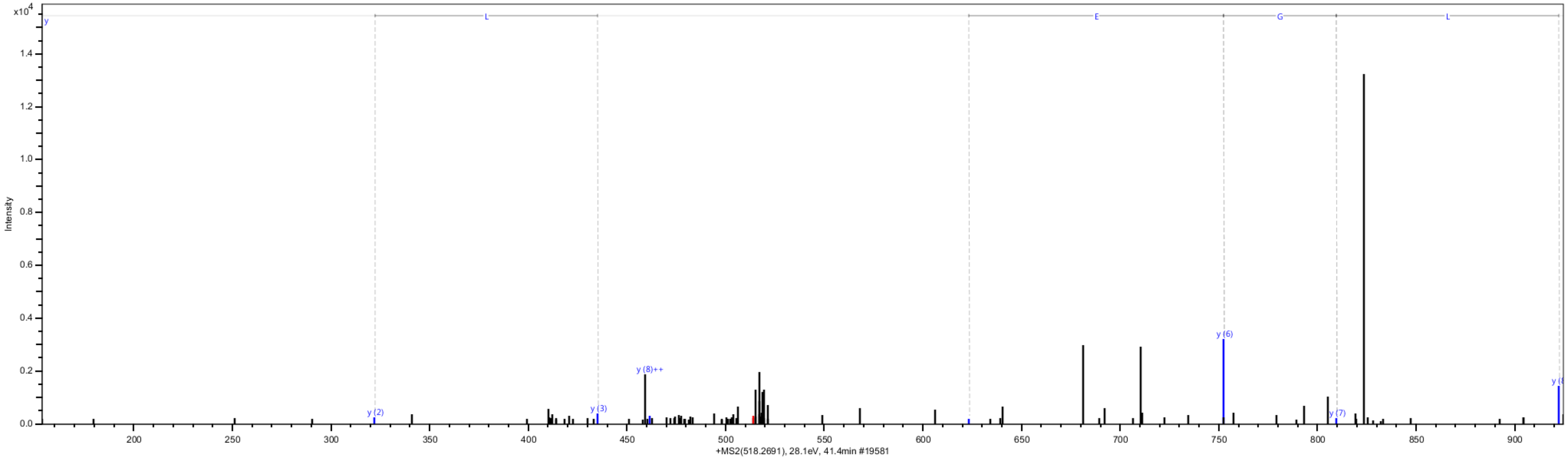

IWS1_HUMAN

Accession: IWS1_HUMAN
Database: SwissProt
MW [kDa] / pI: 91.9 / 4.6
Score: 15.5
Seq. Coverage [%]: 1.1
No. of Peptides: 1

Peptide Match

Sequence: K.EWLSPLPDR.S
Score: 15.5
Modifications:

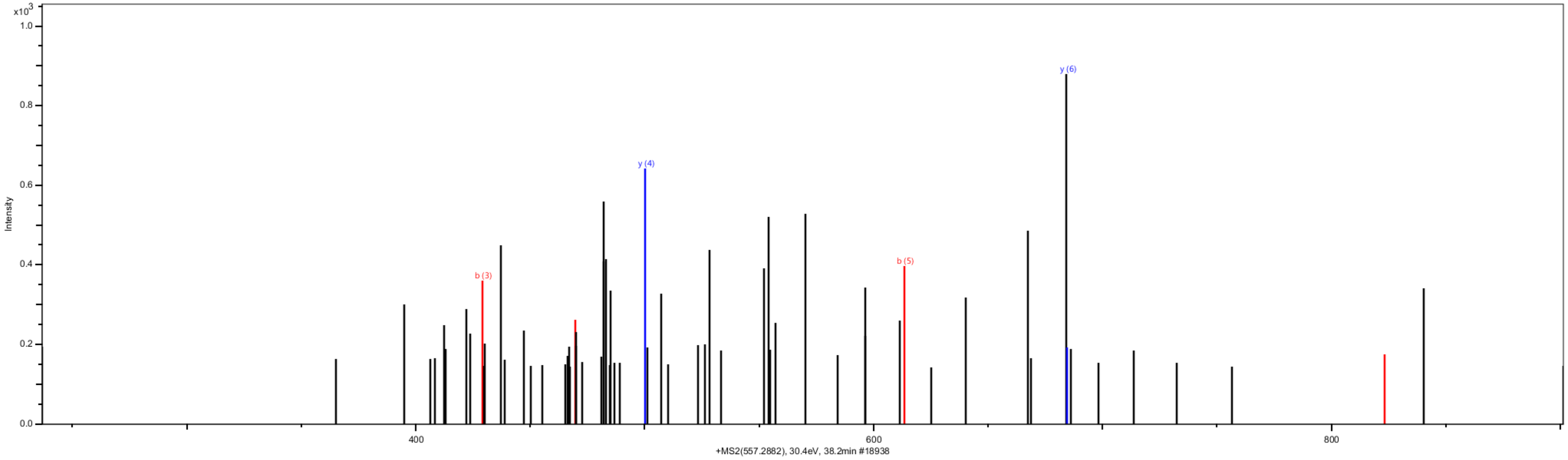

PDE7A_HUMAN

Accession: PDE7A_HUMAN
Database: SwissProt
MW [kDa] / pI: 55.5 / 7.1
Score: 15.5
Seq. Coverage [%]: 3.3
No. of Peptides: 1

Peptide Match

Sequence: R.GAISYDSSDQTALYIR.M
Score: 15.5
Modifications:

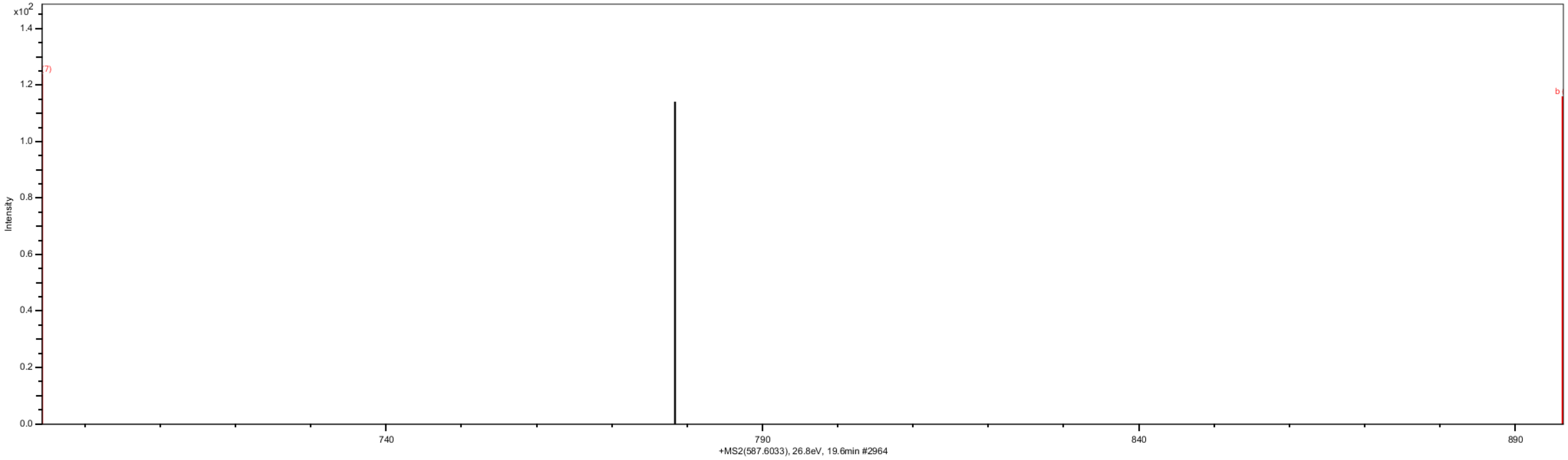

GRPE1_HUMAN

Accession: GRPE1_HUMAN
Database: SwissProt
MW [kDa] / pI: 24.3 / 8.2
Score: 15.5
Seq. Coverage [%]: 4.1
No. of Peptides: 1

Peptide Match

Sequence: K.LYGIQAFCK.D
Score: 15.5
Modifications: Carbamidomethyl: 8

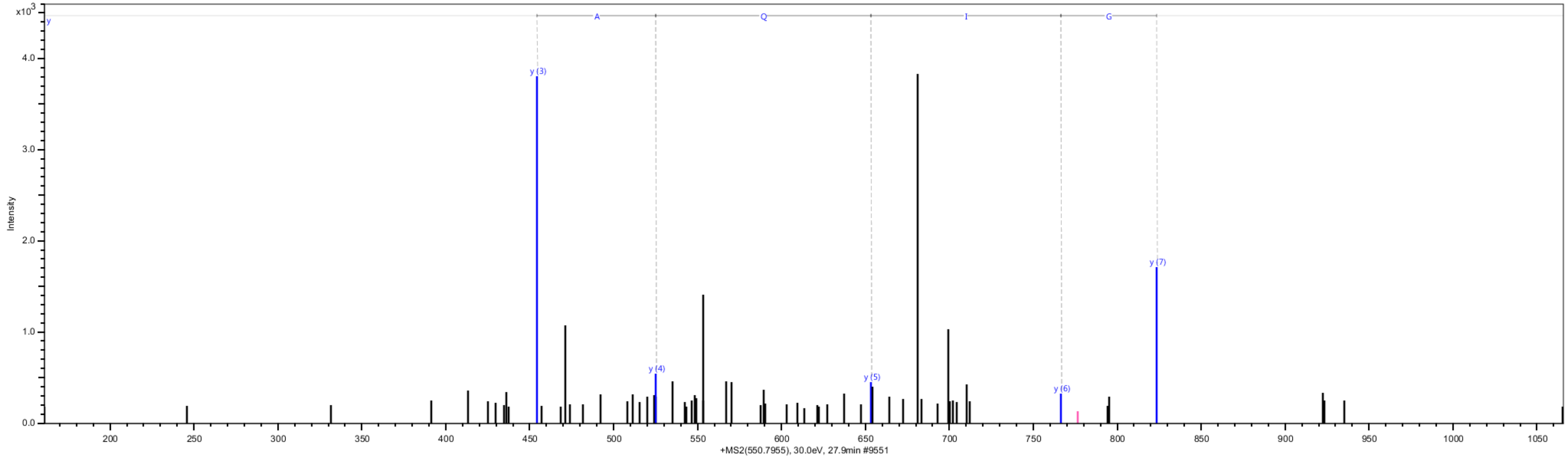

ANR40_HUMAN

Accession: ANR40_HUMAN
Database: SwissProt
MW [kDa] / pI: 41.1 / 4.9
Score: 15.6
Seq. Coverage [%]: 2.7
No. of Peptides: 1

Peptide Match

Sequence: K.GEMPVQLTSR.R
Score: 15.6
Modifications: Oxidation: 3

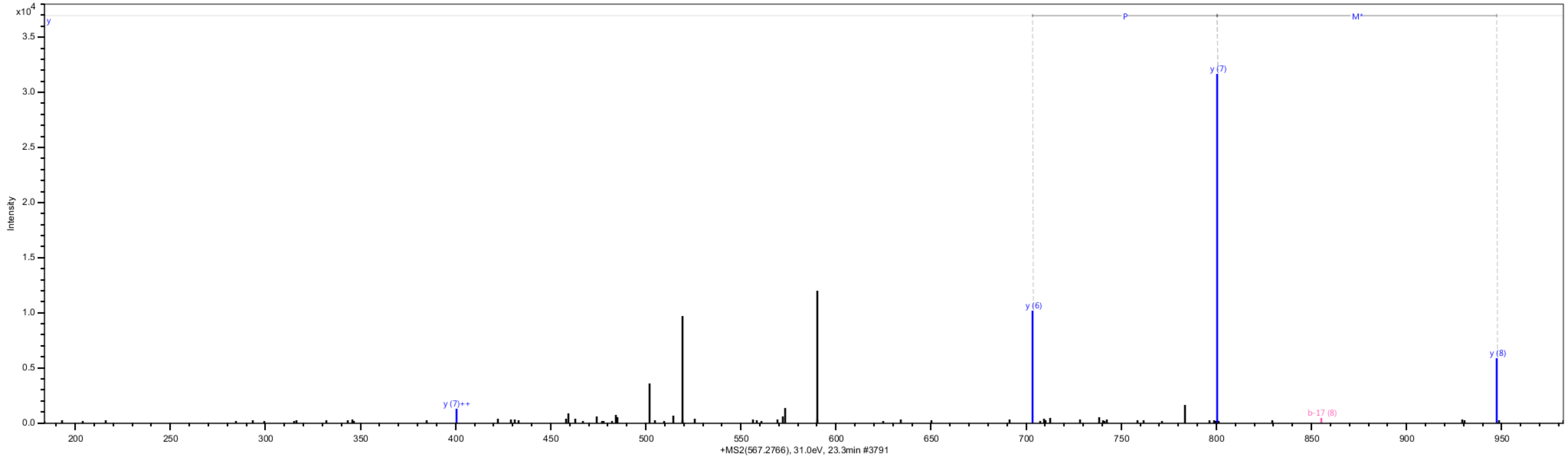

TENA_HUMAN

**Accession:**	TENA_HUMAN	**Score:**	15.6
**Database:**	SwissProt	**Seq. Coverage [%]:**	0.4
**MW [kDa] / pI:**	240.7 / 4.8	**No. of Peptides:**	1

Peptide Match

**Sequence:**	R.NLTVPGSLR.A	**Score:**	15.6
**Modifications:**			

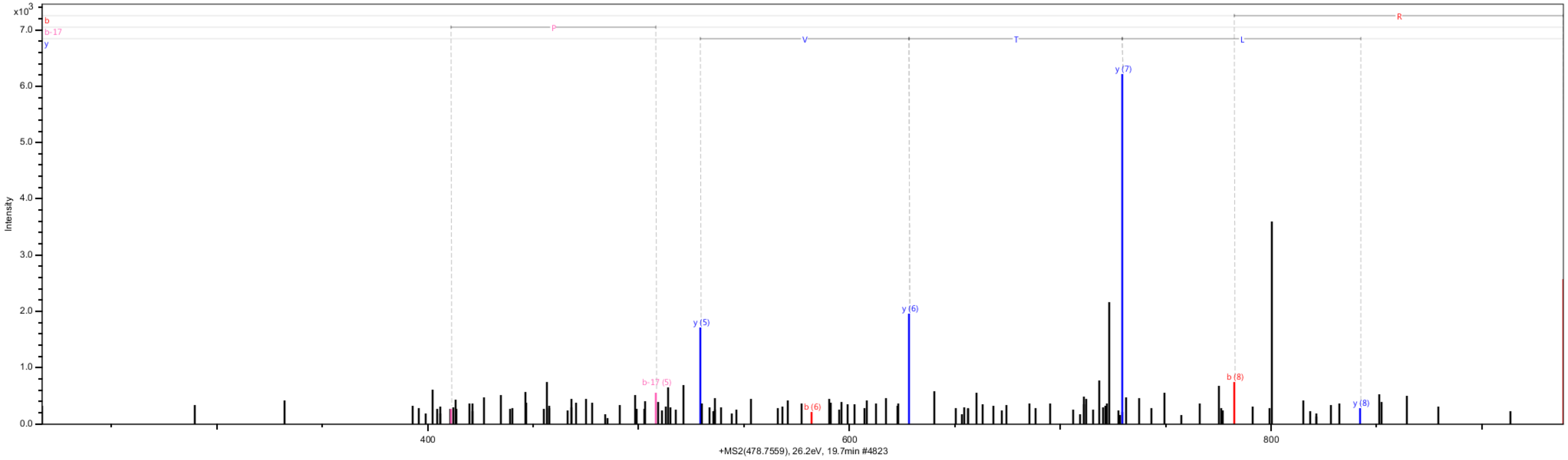

MYZAP_HUMAN

**Accession:**	MYZAP_HUMAN	**Score:**	15.6
**Database:**	SwissProt	**Seq. Coverage [%]:**	1.9
**MW [kDa] / pI:**	54.2 / 5.9	**No. of Peptides:**	1

Peptide Match

**Sequence:**	R.TREIVMPSR.N	**Score:**	15.6
**Modifications:**			

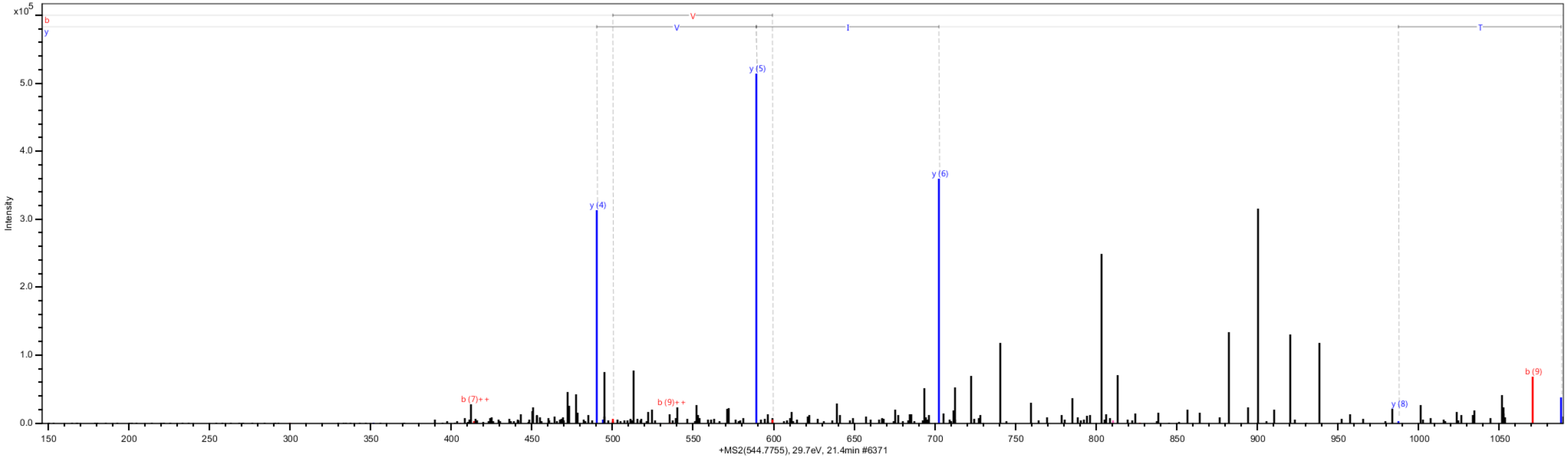

ZN703_HUMAN

**Accession:**	ZN703_HUMAN	**Score:**	15.6
**Database:**	SwissProt	**Seq. Coverage [%]:**	2.0
**MW [kDa] / pI:**	58.2 / 9.0	**No. of Peptides:**	1

Peptide Match

**Sequence:**	R.FATSEELLSHLR.T	**Score:**	15.6
**Modifications:**			

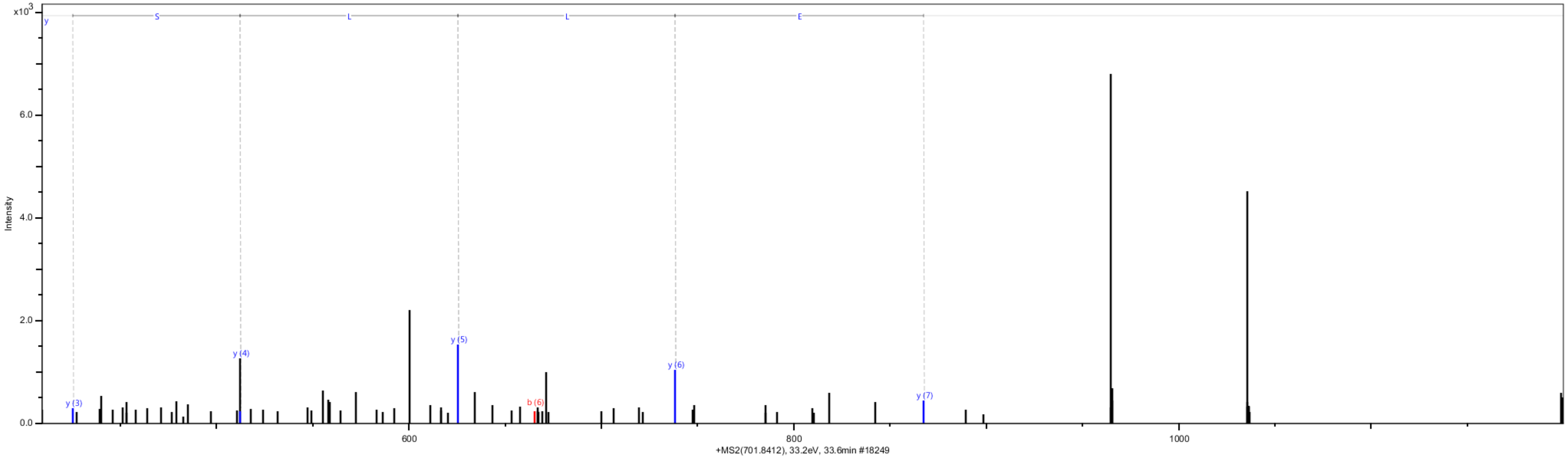

ARI4A_HUMAN

Accession: ARI4A_HUMAN
Database: SwissProt
MW [kDa] / pI: 142.7 / 5.0
Score: 15.6
Seq. Coverage [%]: 1.0
No. of Peptides: 1

Peptide Match

Sequence: K.DELEKNENLNDDK.L
Score: 15.6
Modifications:

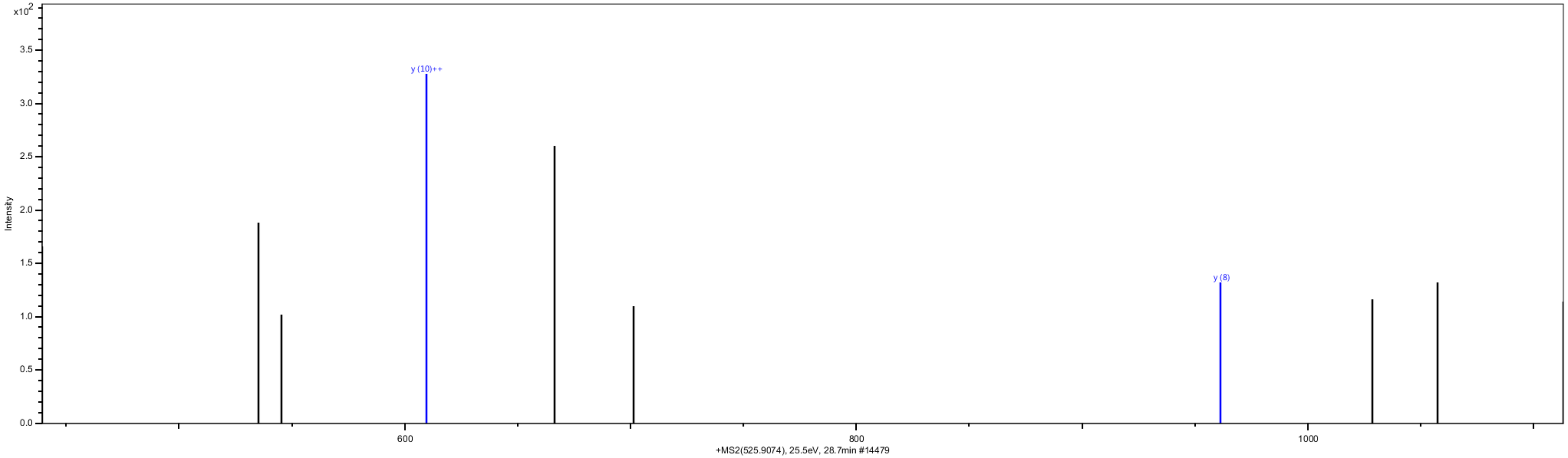

CHD4_HUMAN

Accession: CHD4_HUMAN
Database: SwissProt
MW [kDa] / pI: 217.9 / 5.6
Score: 15.6
Seq. Coverage [%]: 0.5
No. of Peptides: 1

Peptide Match

Sequence: R.GEEGRPGKK.L
Score: 15.6
Modifications:

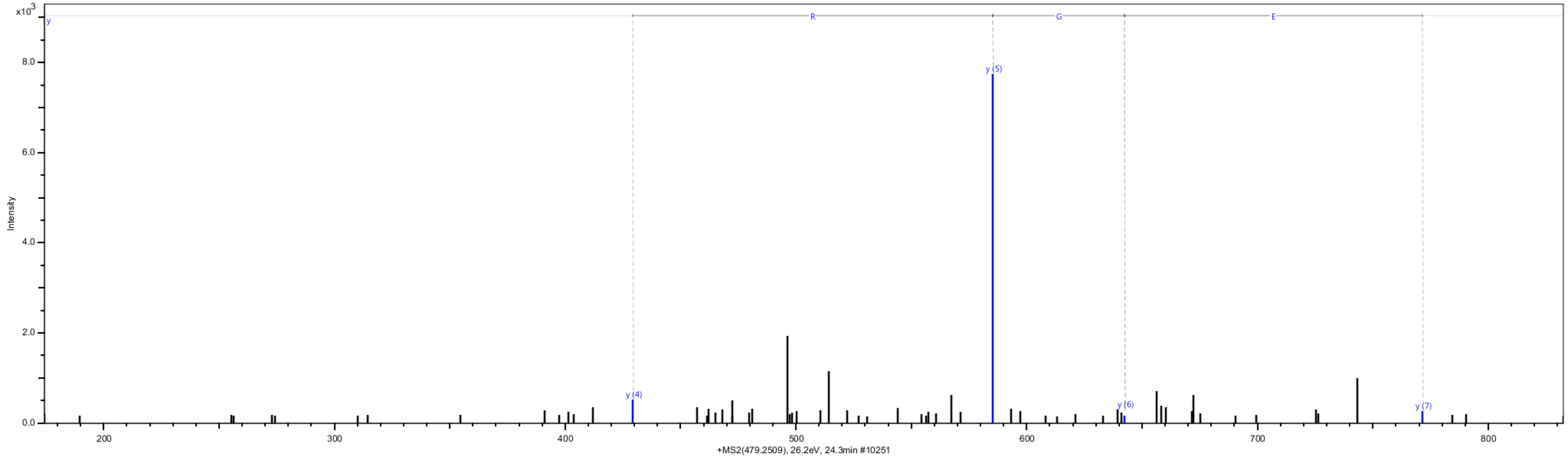

DGKK_HUMAN

**Accession:**	DGKK_HUMAN	**Score:**	15.5
**Database:**	SwissProt	**Seq. Coverage [%]:**	0.6
**MW [kDa] / pI:**	141.7 / 5.4	**No. of Peptides:**	1

Peptide Match

**Sequence:**	K.ITLAAPNR.K	**Score:**	15.5
**Modifications:**			

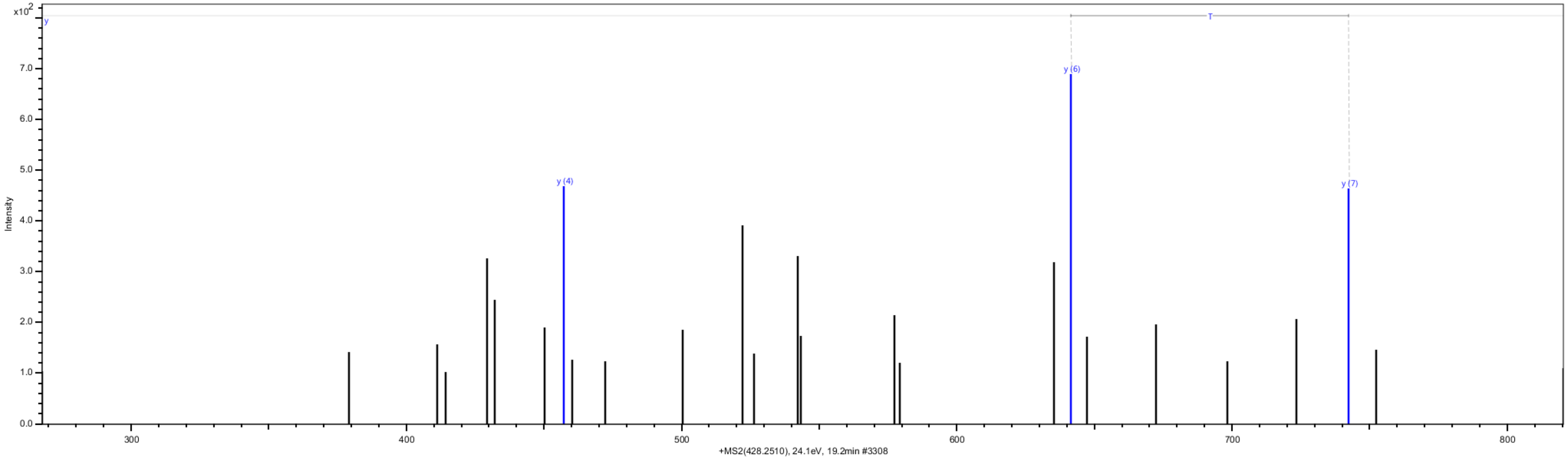

CRBB2_HUMAN

Accession: CRBB2_HUMAN
Database: SwissProt
MW [kDa] / pI: 23.4 / 6.5
Score: 15.6
Seq. Coverage [%]: 3.4
No. of Peptides: 1

Peptide Match

Sequence: K.VDSQEHK.I
Score: 15.6
Modifications:

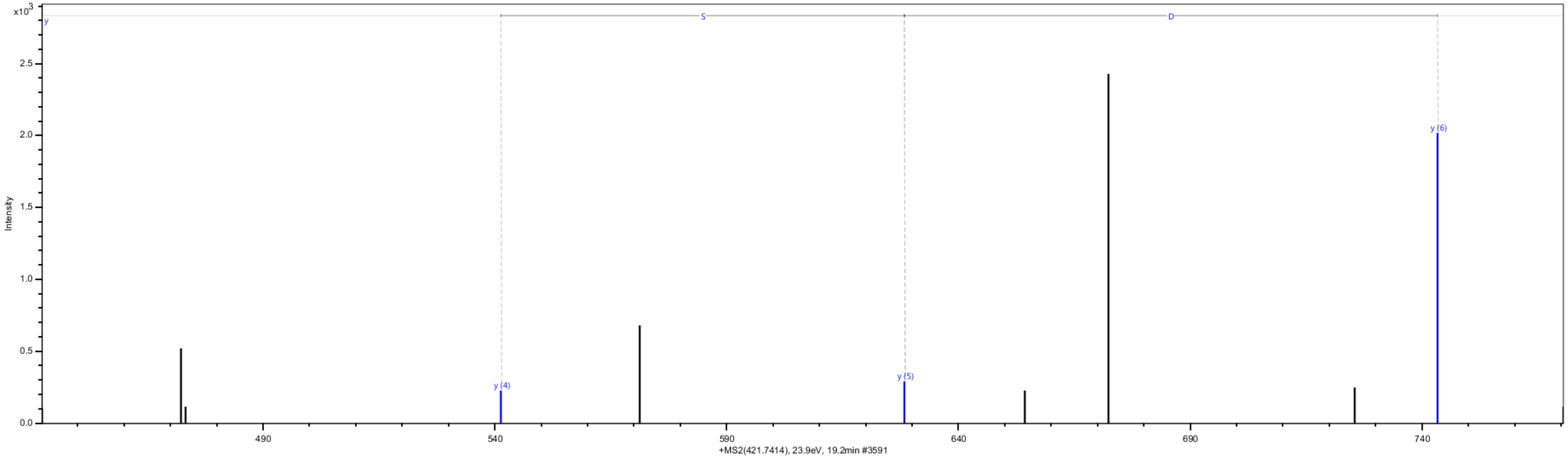

GBB3_HUMAN

**Accession:**	GBB3_HUMAN	**Score:**	15.6
**Database:**	SwissProt	**Seq. Coverage [%]:**	2.9
**MW [kDa] / pI:**	37.2 / 5.4	**No. of Peptides:**	1

Peptide Match

**Sequence:**	K.LLVSASQDGK.L	**Score:**	15.6
**Modifications:**			

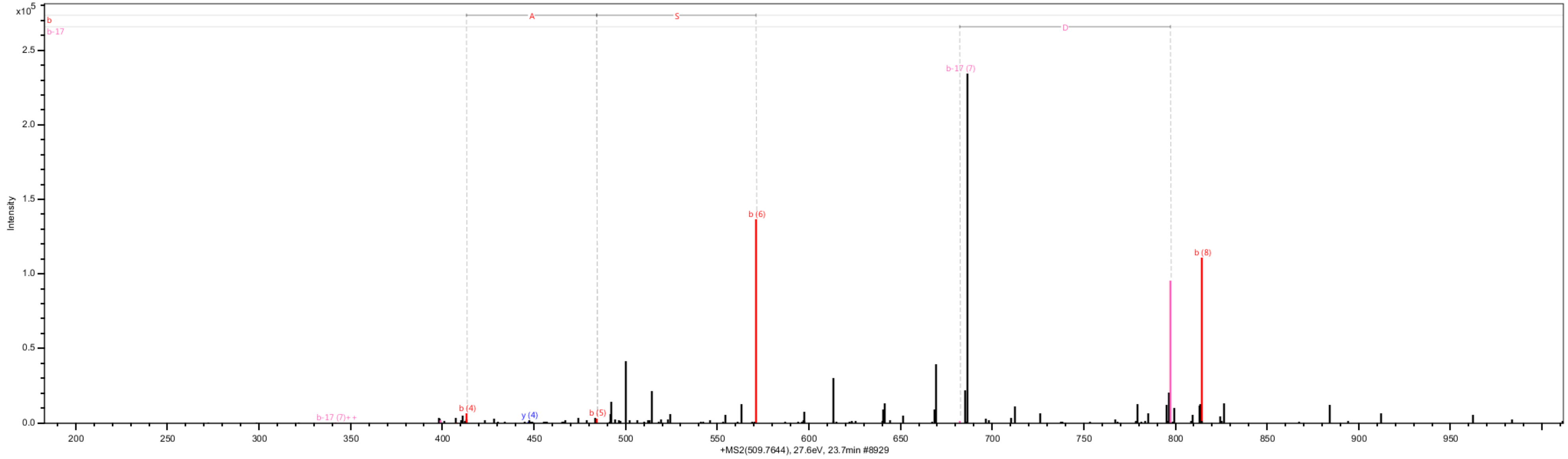

FA98B_HUMAN

Accession: FA98B_HUMAN
Database: SwissProt
MW [kDa] / pI: 45.5 / 8.9
Score: 15.6
Seq. Coverage [%]: 2.1
No. of Peptides: 1

Peptide Match

Sequence: R.TSSGTSREK.T
Score: 15.6
Modifications:

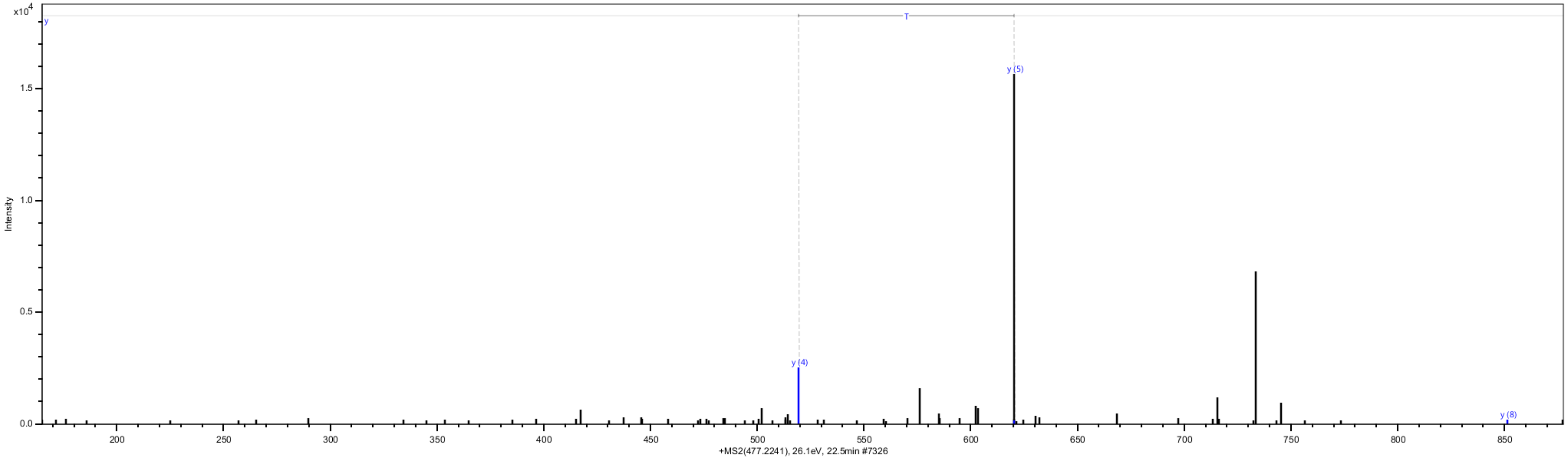

PIMRE_HUMAN

Accession: PIMRE_HUMAN
Database: SwissProt
MW [kDa] / pI: 27.5 / 10.2
Score: 15.6
Seq. Coverage [%]: 5.6
No. of Peptides: 1

Peptide Match

Sequence: K.LSQELDEAIMAEER.K
Score: 15.6
Modifications:

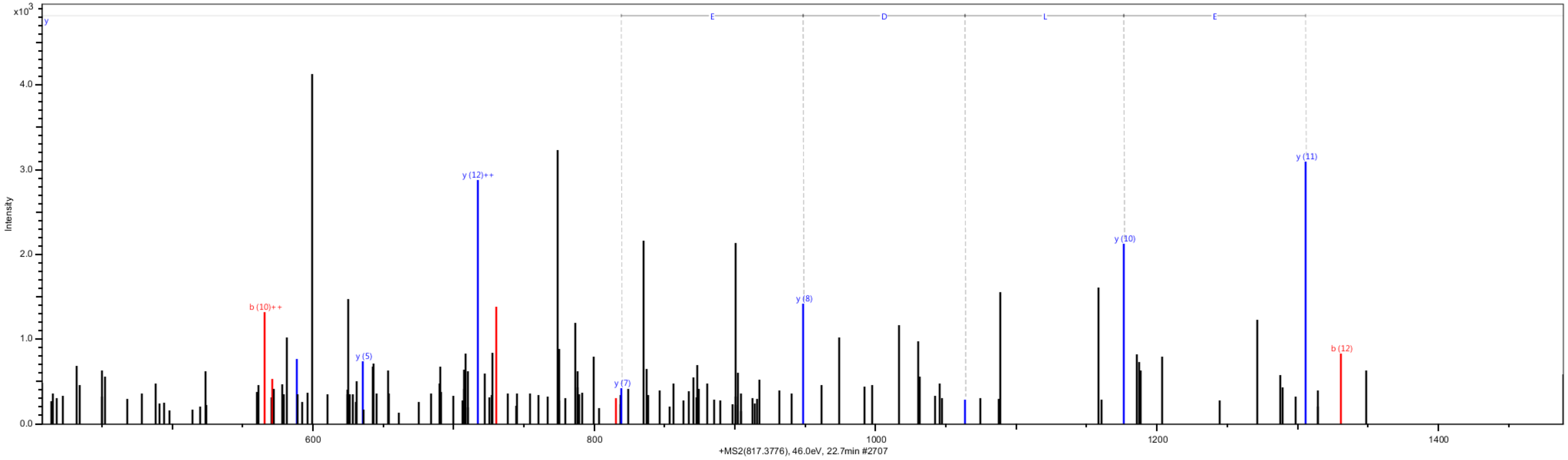

PLEC_HUMAN

Accession: PLEC_HUMAN
Database: SwissProt
MW [kDa] / pI: 531.5 / 5.7
Score: 15.6
Seq. Coverage [%]: 0.2
No. of Peptides: 1

Peptide Match

Sequence: K.MSAAQALKK.G
Score: 15.6
Modifications:

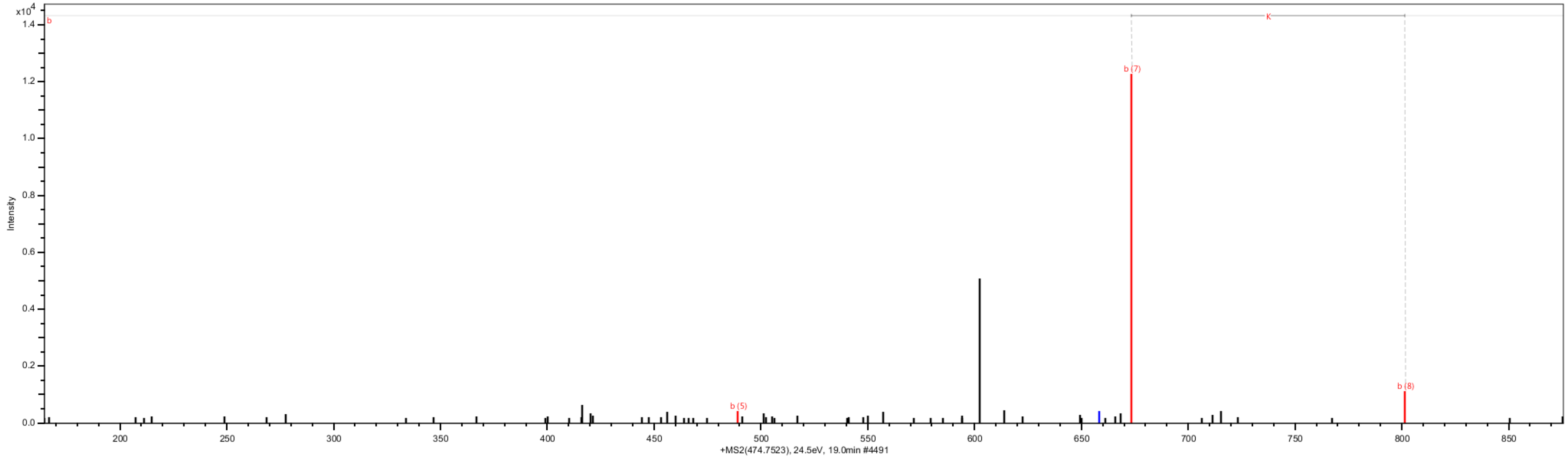

YDJC_HUMAN

Accession: YDJC_HUMAN
Database: SwissProt
MW [kDa] / pI: 34.4 / 6.0
Score: 15.2
Seq. Coverage [%]: 4.0
No. of Peptides: 1

Peptide Match

Sequence: R.LVVTADDFGYCPR.R
Score: 15.2
Modifications: Carbamidomethyl: 11

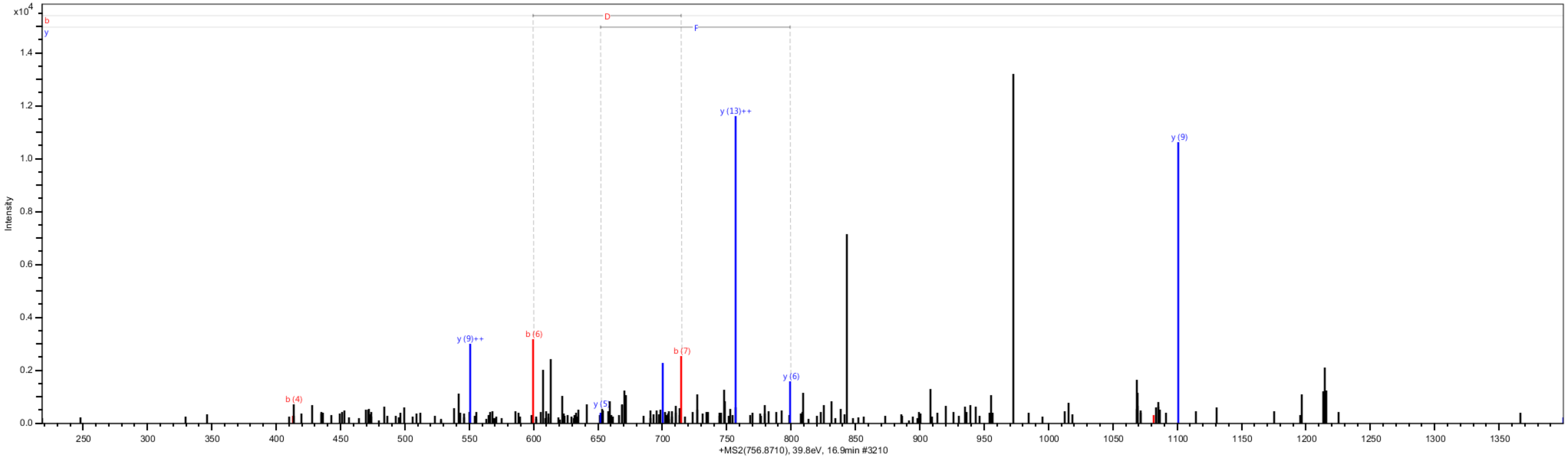

SAXO1_HUMAN

Accession: SAXO1_HUMAN
Database: SwissProt
MW [kDa] / pI: 54.6 / 8.7
Score: 15.3
Seq. Coverage [%]: 2.7
No. of Peptides: 1

Peptide Match

Sequence: K.GPIPMEGLTTSRR.D
Score: 15.2
Modifications: Oxidation: 5

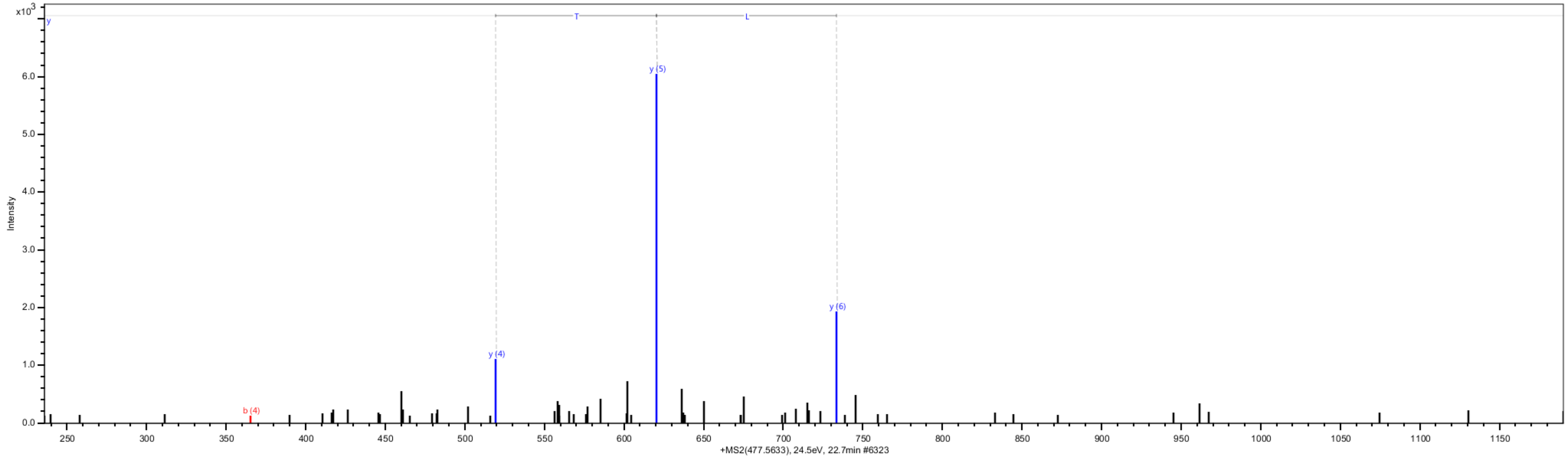

RAD9B_HUMAN

Accession: RAD9B_HUMAN
Database: SwissProt
MW [kDa] / pI: 47.8 / 6.5
Score: 15.3
Seq. Coverage [%]: 3.1
No. of Peptides: 1

Peptide Match

Sequence: K.MSENELDTTLHLK.C
Score: 15.2
Modifications:

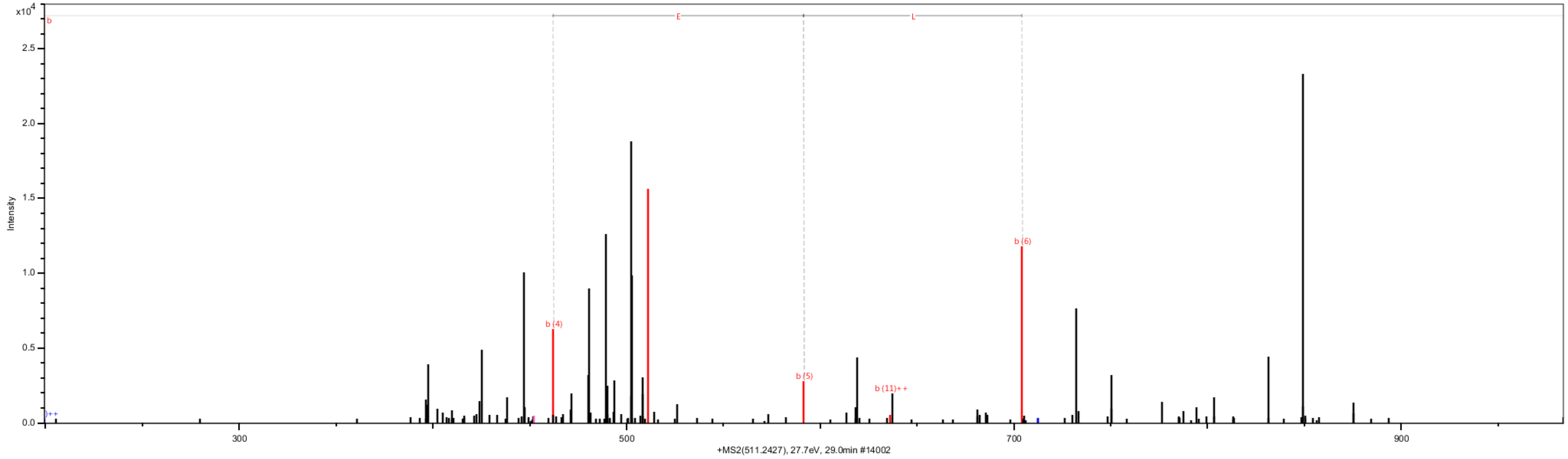

PRCC_HUMAN

**Accession:**	PRCC_HUMAN	**Score:**	15.3
**Database:**	SwissProt	**Seq. Coverage [%]:**	4.1
**MW [kDa] / pI:**	52.4 / 5.0	**No. of Peptides:**	1

Peptide Match

Sequence: K.GDDQLSGAQQWMTKSLTEEK.T **Score:** 15.3

Modifications:

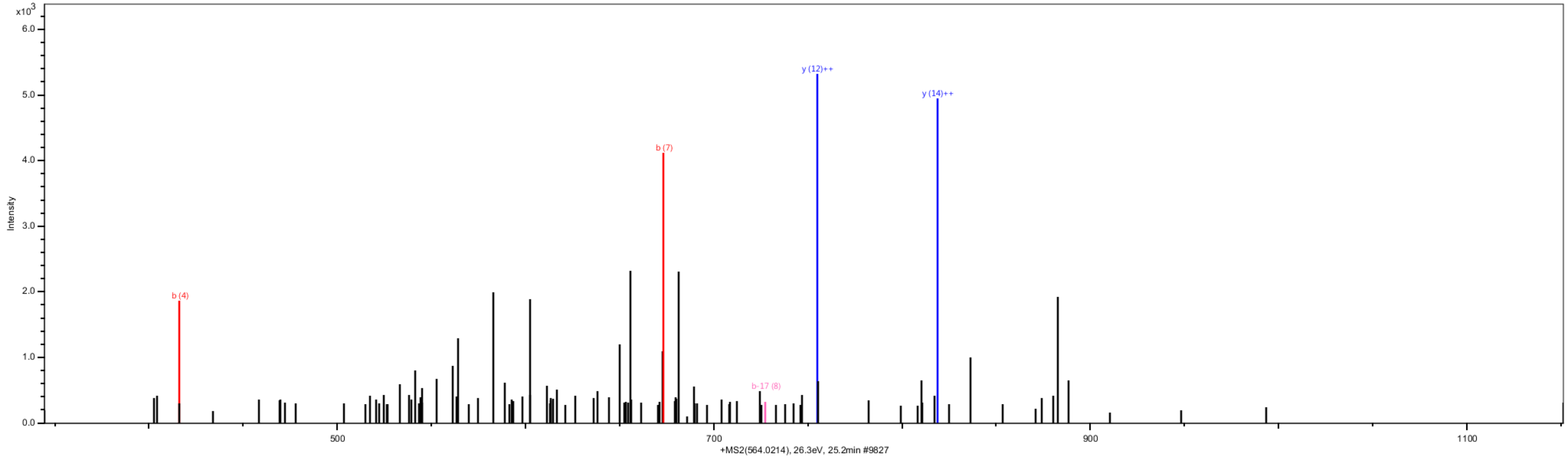

F1711_HUMAN

**Accession:**	F1711_HUMAN	**Score:**	15.3
**Database:**	SwissProt	**Seq. Coverage [%]:**	1.0
**MW [kDa] / pI:**	97.8 / 6.1	**No. of Peptides:**	1

Peptide Match

**Sequence:**	R.RPTECMMSR.S	**Score:**	15.3
**Modifications:**	Carbamidomethyl: 5; Oxidation: 6, 7		

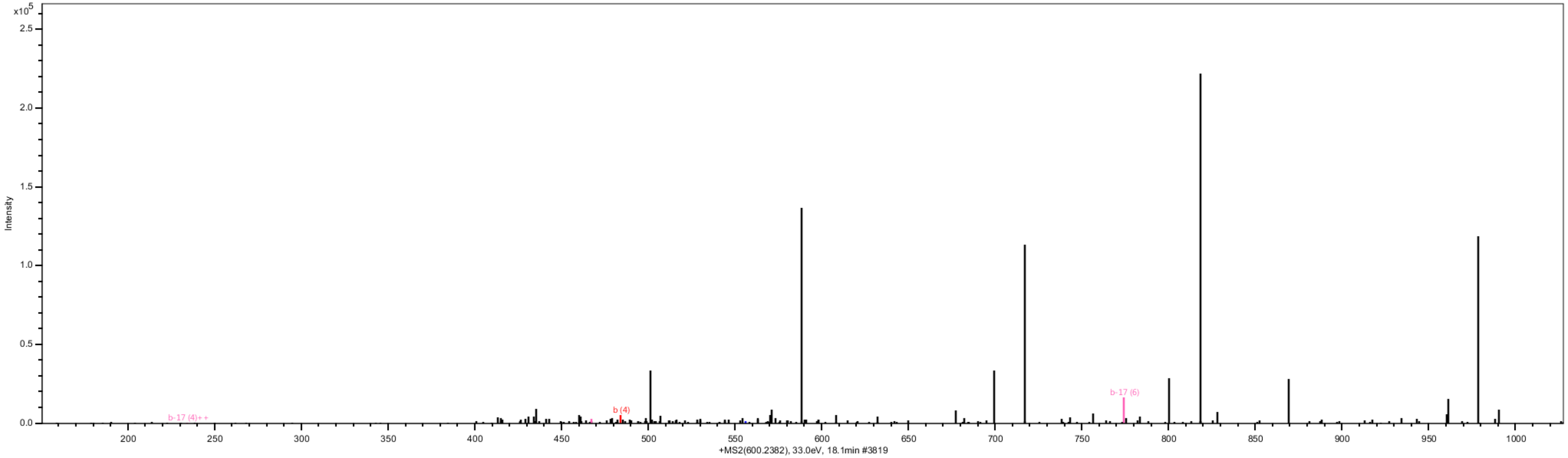

LV151_HUMAN

Accession: LV151_HUMAN
Database: SwissProt
MW [kDa] / pI: 12.2 / 6.5
Score: 15.3
Seq. Coverage [%]: 13.7
No. of Peptides: 1

Peptide Match

Sequence: K.LLIYDNNKRPSGIPDR.F
Score: 15.3
Modifications:

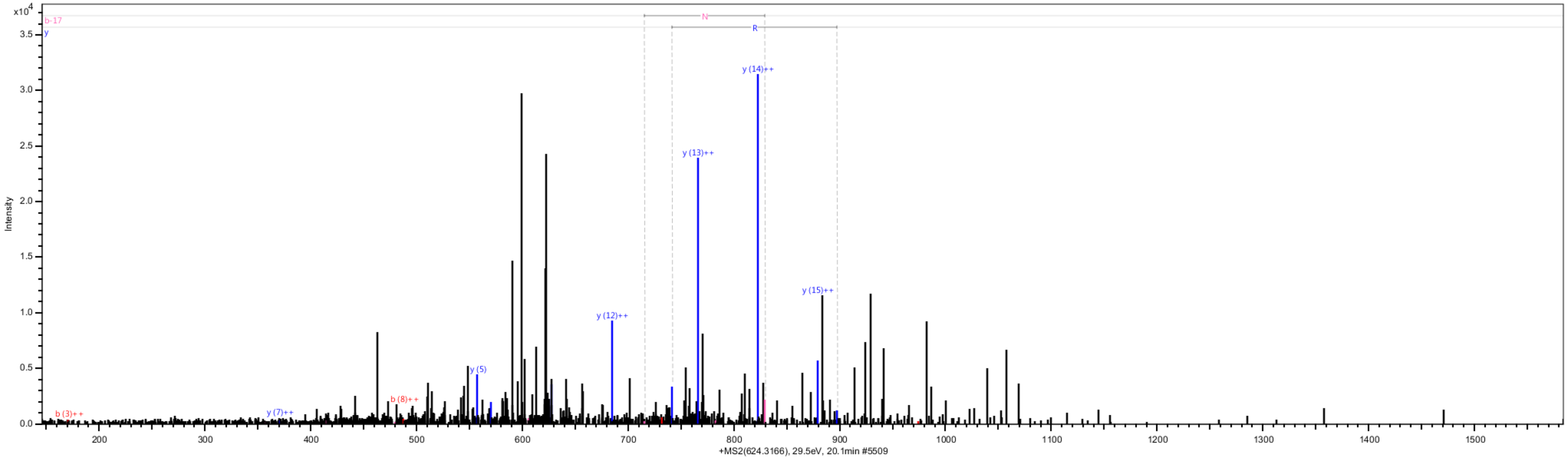

PATL1_HUMAN

Accession: PATL1_HUMAN
Database: SwissProt
MW [kDa] / pI: 86.8 / 6.2
Score: 15.3
Seq. Coverage [%]: 1.4
No. of Peptides: 1

Peptide Match

Sequence: K.LTVSSVNNPRK.M
Score: 15.3
Modifications:

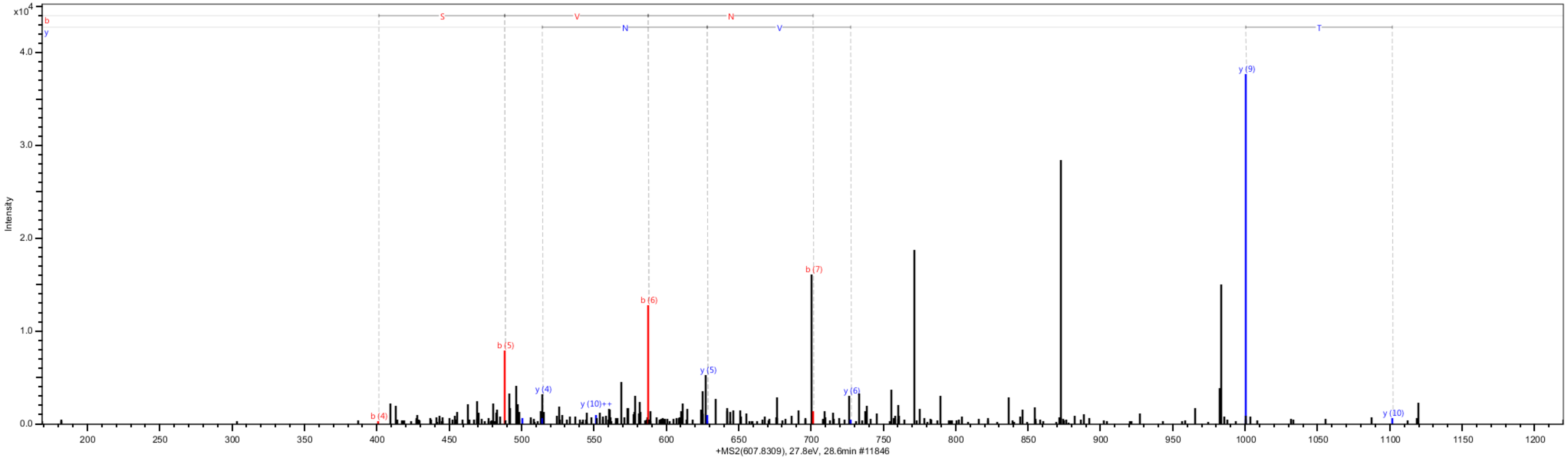

RM52_HUMAN

Accession: RM52_HUMAN
Database: SwissProt
MW [kDa] / pI: 13.7 / 10.3
Score: 15.4
Seq. Coverage [%]: 7.3
No. of Peptides: 1

Peptide Match

Sequence: R.KQENALKPK.G
Score: 15.4
Modifications:

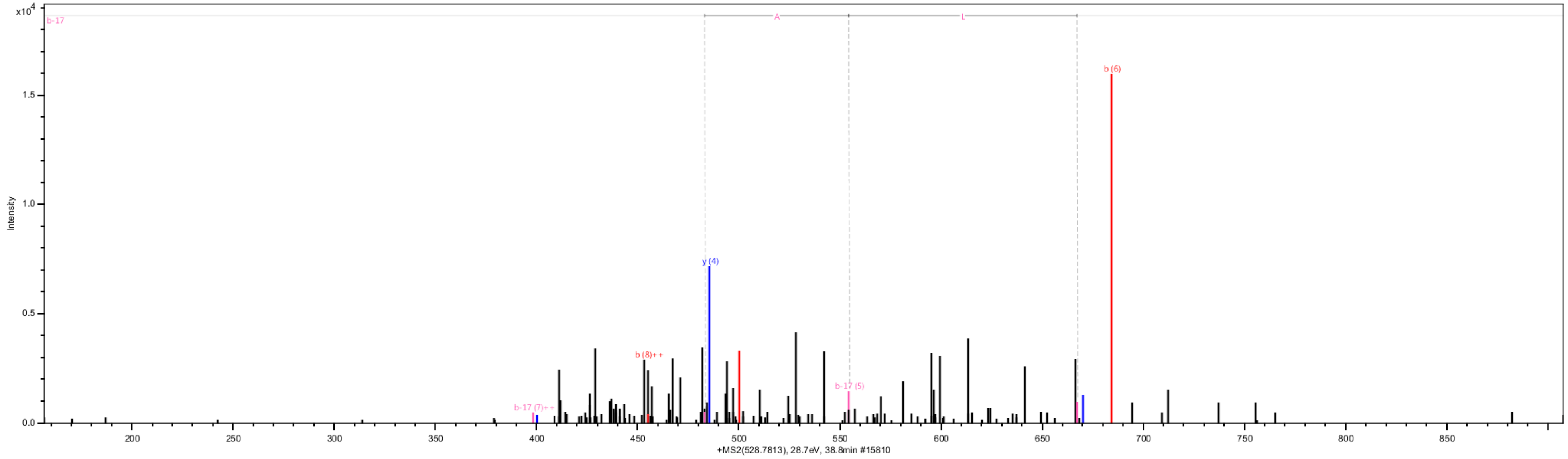

PKHD1_HUMAN

**Accession:**	PKHD1_HUMAN	**Score:**	15.4
**Database:**	SwissProt	**Seq. Coverage [%]:**	0.3
**MW [kDa] / pI:**	446.4 / 6.1	**No. of Peptides:**	1

Peptide Match

**Sequence:**	R.NIQIQPDVSCR.G	**Score:**	15.4
**Modifications:**	Carbamidomethyl: 10		

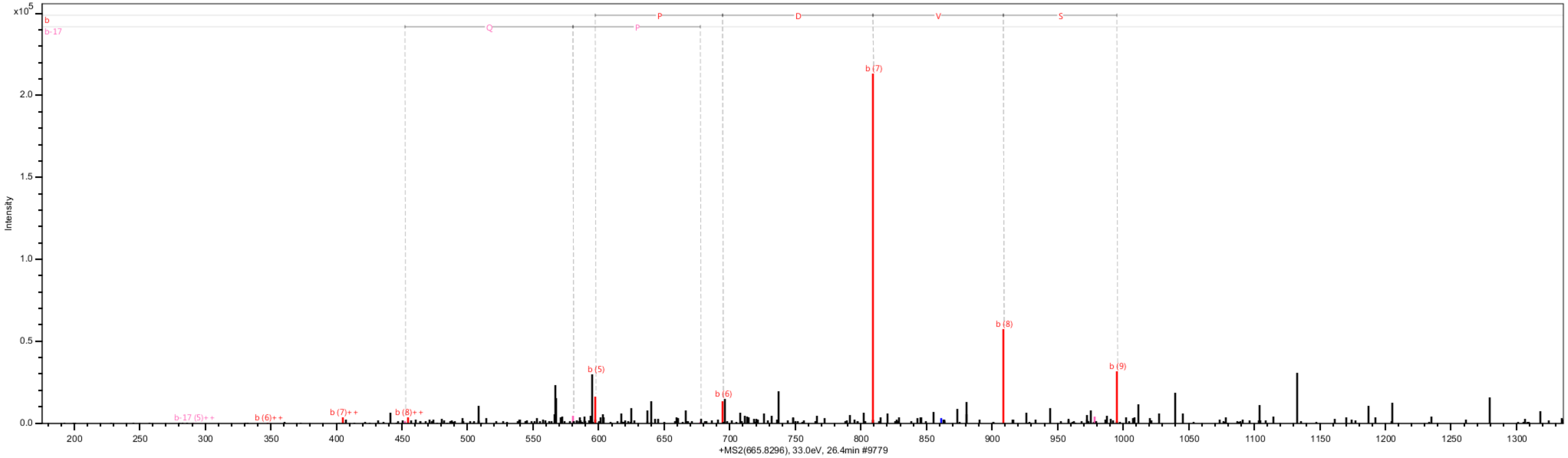

KAD9_HUMAN

Accession: KAD9_HUMAN
Database: SwissProt
MW [kDa] / pI: 221.3 / 5.0
Score: 15.4
Seq. Coverage [%]: 0.4
No. of Peptides: 1

Peptide Match

Sequence: K.NVQMVNK.Y
Score: 15.4
Modifications:

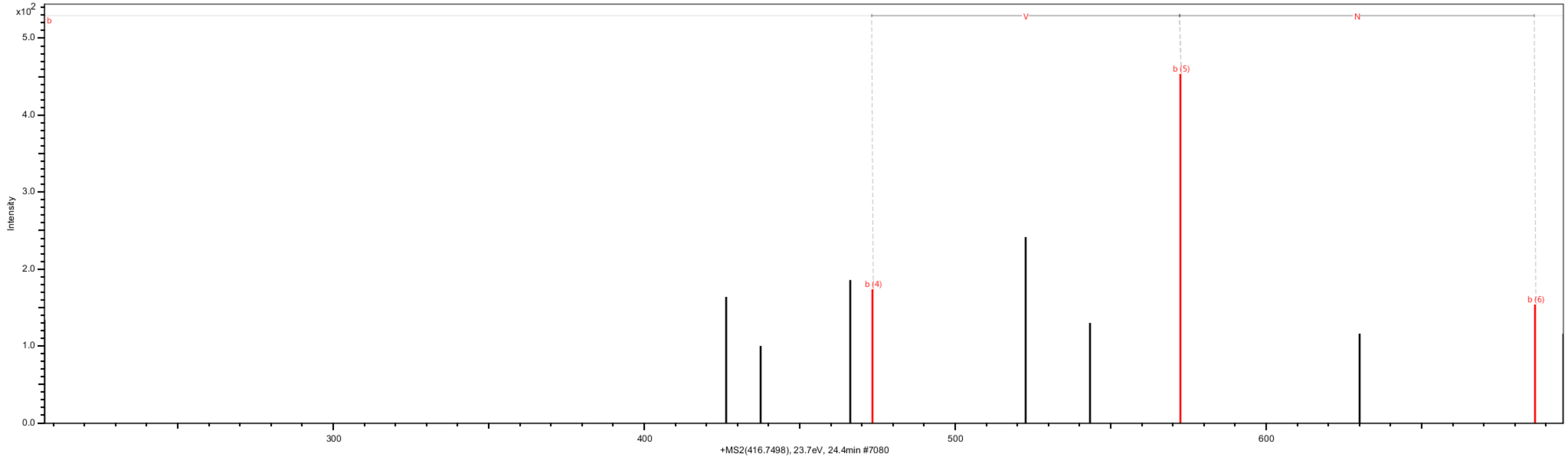

RREB1_HUMAN

**Accession:**	RREB1_HUMAN	**Score:**	15.4
**Database:**	SwissProt	**Seq. Coverage [%]:**	0.7
**MW [kDa] / pI:**	181.3 / 6.5	**No. of Peptides:**	1

Peptide Match

**Sequence:**	R.EQPSEGATELR.Q	**Score:**	15.4
**Modifications:**			

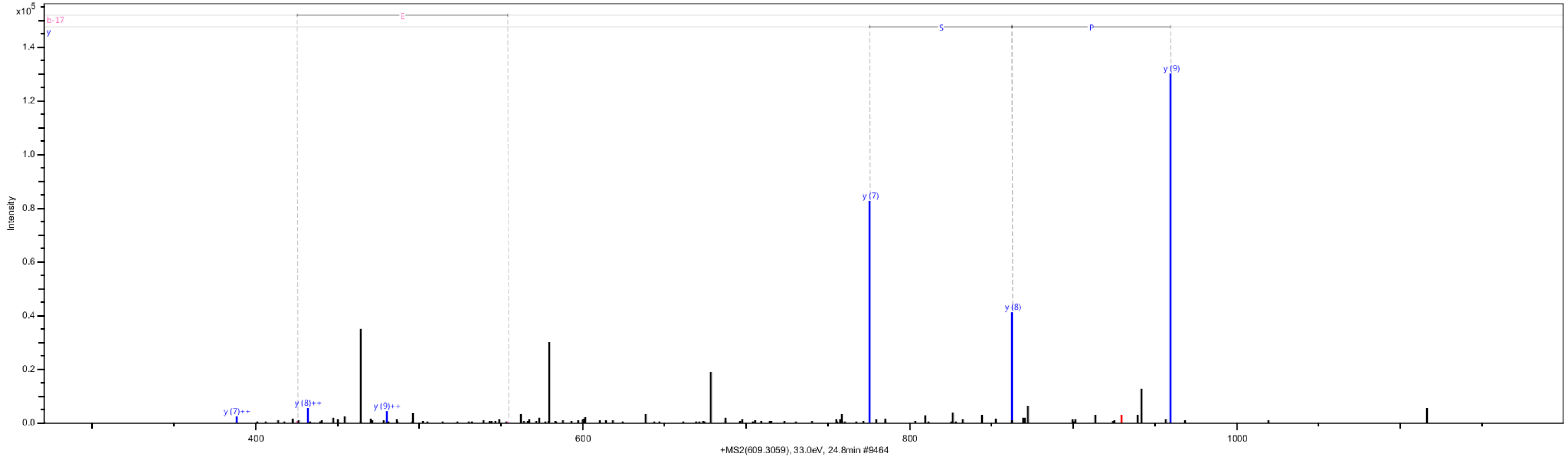

TNS2_HUMAN

Accession: TNS2_HUMAN
Database: SwissProt
MW [kDa] / pI: 152.5 / 8.7
Score: 15.4
Seq. Coverage [%]: 0.5
No. of Peptides: 1

Peptide Match

Sequence: K.FVQDTSK.F
Score: 15.4
Modifications:

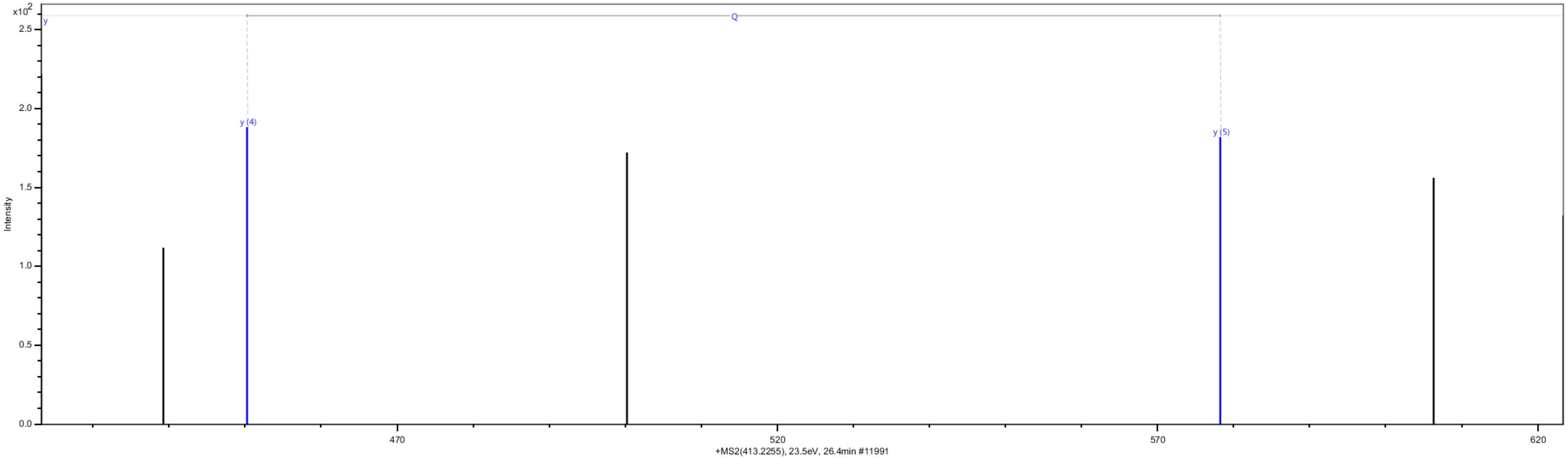

PDPR_HUMAN

**Accession:**	PDPR_HUMAN	**Score:**	15.4
**Database:**	SwissProt	**Seq. Coverage [%]:**	1.4
**MW [kDa] / pI:**	99.3 / 5.9	**No. of Peptides:**	1

Peptide Match

**Sequence:**	R.RMPELETLEIMK.L	**Score:**	15.4
**Modifications:**	Oxidation: 11		

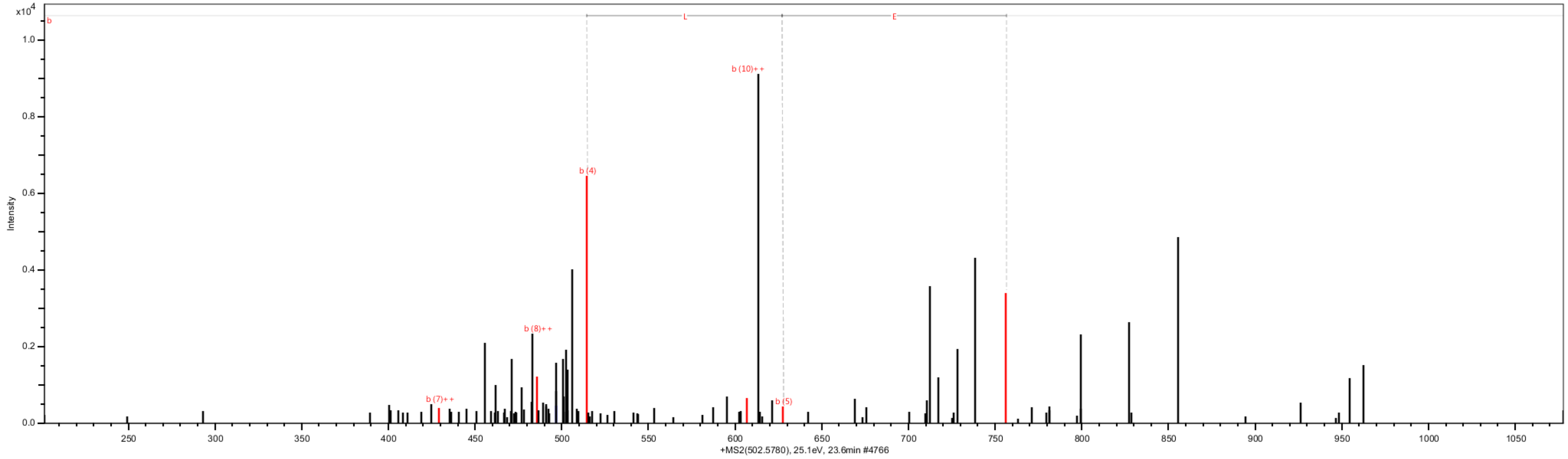

TSAP1_HUMAN

**Accession:**	TSAP1_HUMAN	**Score:**	15.4
**Database:**	SwissProt	**Seq. Coverage [%]:**	4.5
**MW [kDa] / pI:**	32.5 / 4.6	**No. of Peptides:**	1

Peptide Match

**Sequence:**	R.AFATMGETVMSVK.I	**Score:**	15.4
**Modifications:**	Oxidation: 10		

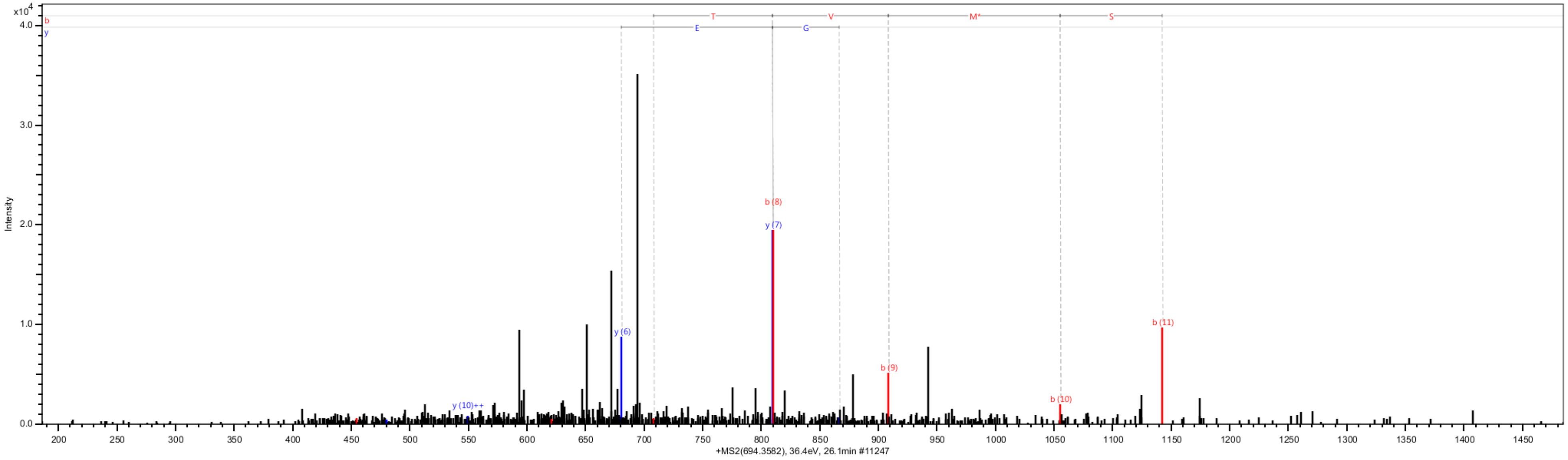

NIP7_HUMAN

Accession: NIP7_HUMAN
Database: SwissProt
MW [kDa] / pI: 20.4 / 8.7
Score: 15.4
Seq. Coverage [%]: 4.4
No. of Peptides: 1

Peptide Match

Sequence: R.VMFEKIAK.Y
Score: 15.4
Modifications: Oxidation: 2

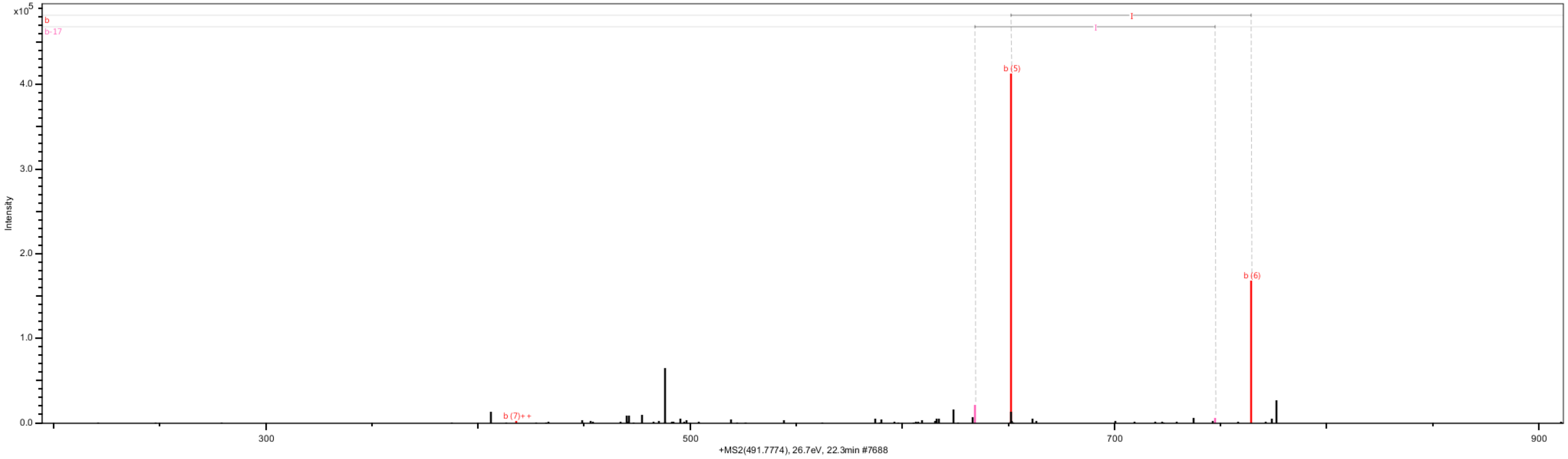

CDC5L_HUMAN

**Accession:**	CDC5L_HUMAN	**Score:**	15.4
**Database:**	SwissProt	**Seq. Coverage [%]:**	1.1
**MW [kDa] / pI:**	92.2 / 8.2	**No. of Peptides:**	1

Peptide Match

**Sequence:**	K.ILLGGYQSR.A	**Score:**	15.4
**Modifications:**			

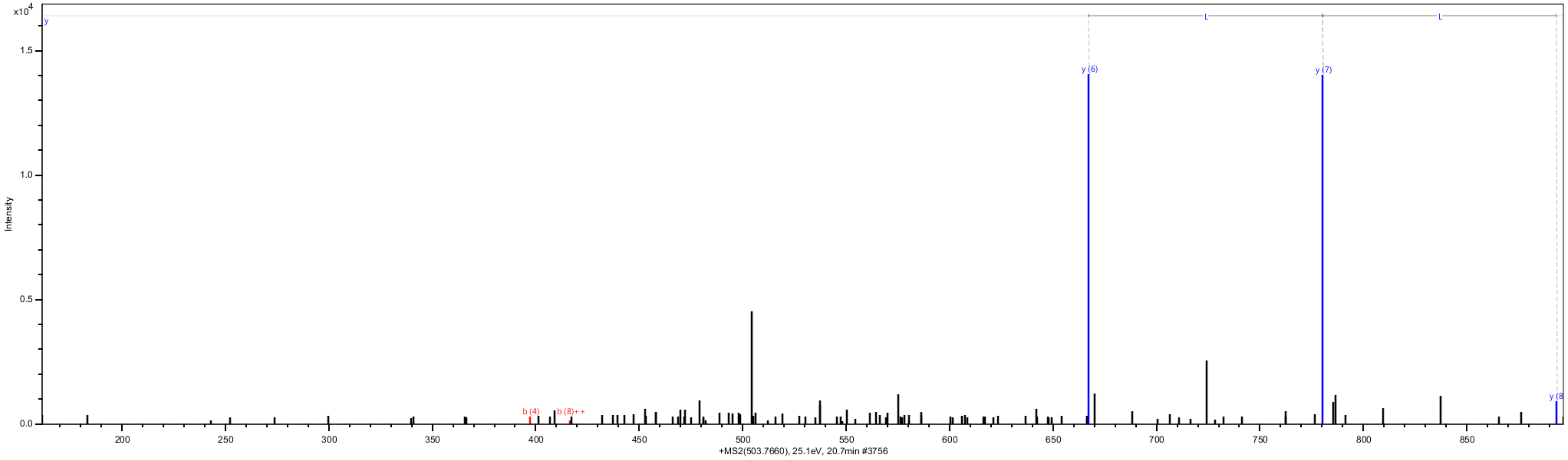

IF3M_HUMAN

Accession: IF3M_HUMAN
Database: SwissProt
MW [kDa] / pI: 31.7 / 9.7
Score: 15.4
Seq. Coverage [%]: 3.2
No. of Peptides: 1

Peptide Match

Sequence: K.TKQIQQWIK.K
Score: 15.4
Modifications:

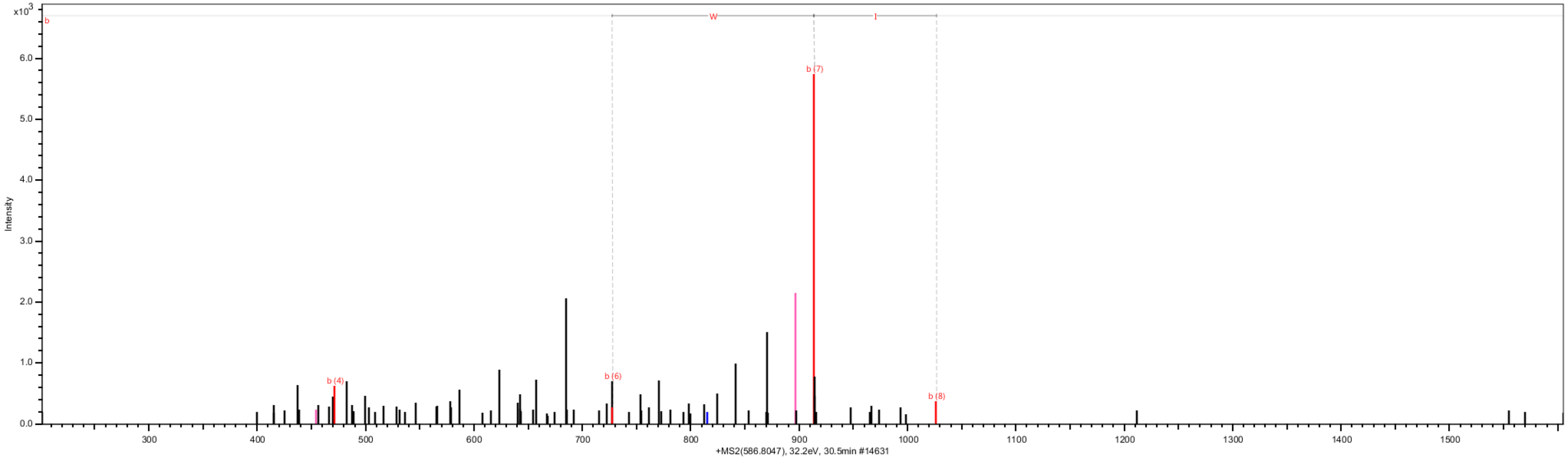

FBX33_HUMAN

**Accession:**	FBX33_HUMAN	**Score:**	15.4
**Database:**	SwissProt	**Seq. Coverage [%]:**	2.5
**MW [kDa] / pI:**	62.6 / 7.0	**No. of Peptides:**	1

Peptide Match

**Sequence:**	K.STSFRVYIMAFDIK.S	**Score:**	15.4
**Modifications:**	Oxidation: 9		

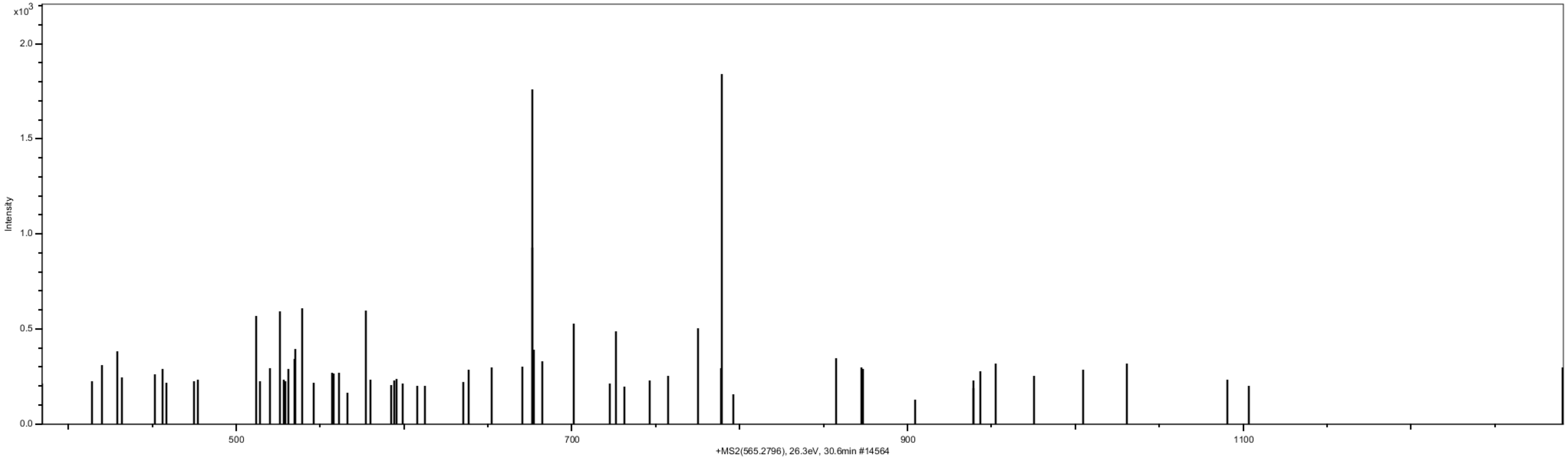

NAT14_HUMAN

Accession: NAT14_HUMAN
Database: SwissProt
MW [kDa] / pI: 21.6 / 10.7
Score: 15.4
Seq. Coverage [%]: 5.8
No. of Peptides: 1

Peptide Match

Sequence: R.AWAGGMGEPRAR.L
Score: 15.4
Modifications:

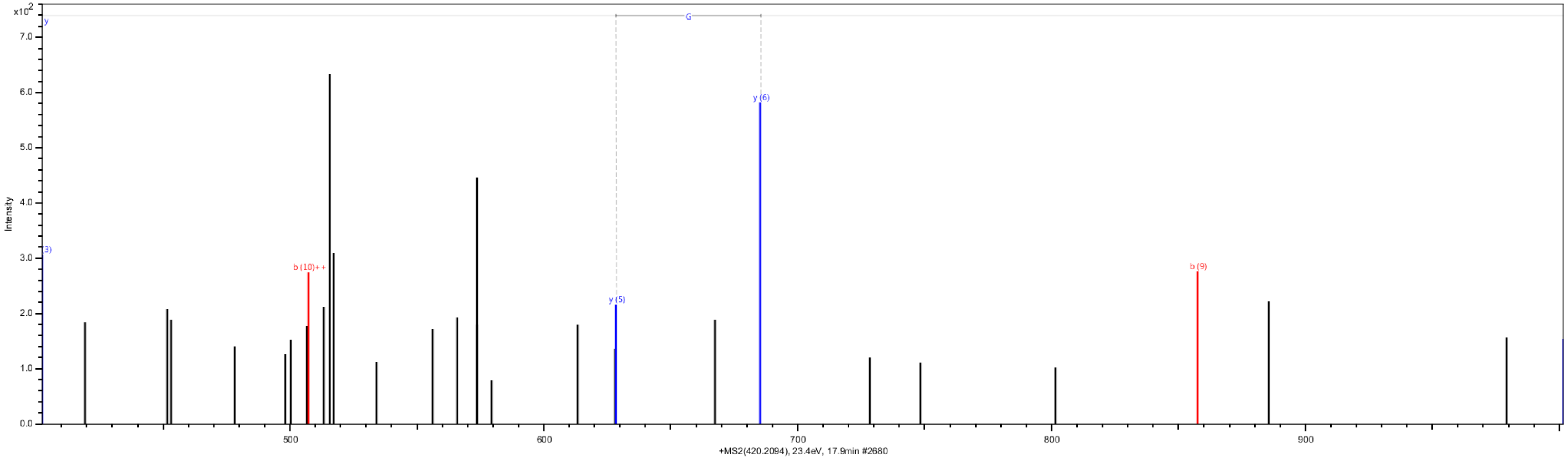

EMAL5_HUMAN

Accession: EMAL5_HUMAN
Database: SwissProt
MW [kDa] / pI: 219.3 / 7.9
Score: 15.4
Seq. Coverage [%]: 0.5
No. of Peptides: 1

Peptide Match

Sequence: R.NNLYYTAAK.E
Score: 15.4
Modifications:

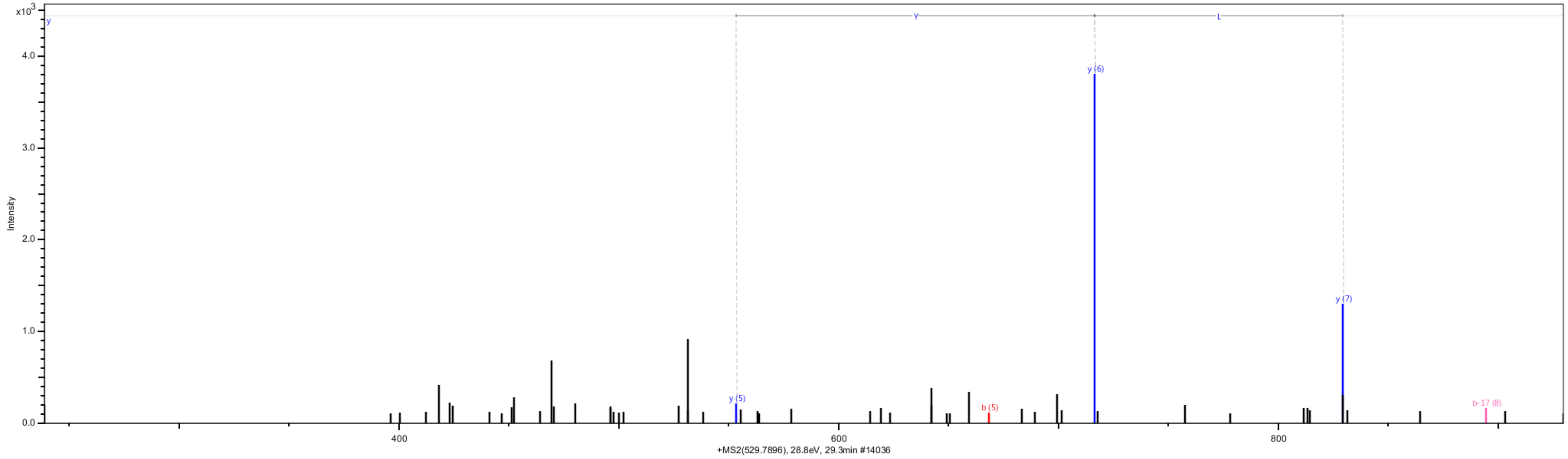

FA8_HUMAN

Accession: FA8_HUMAN
Database: SwissProt
MW [kDa] / pI: 266.8 / 6.9
Score: 15.4
Seq. Coverage [%]: 0.5
No. of Peptides: 1

Peptide Match

Sequence: R.MLMDKNATALR.L
Score: 15.4
Modifications: Oxidation: 1, 3

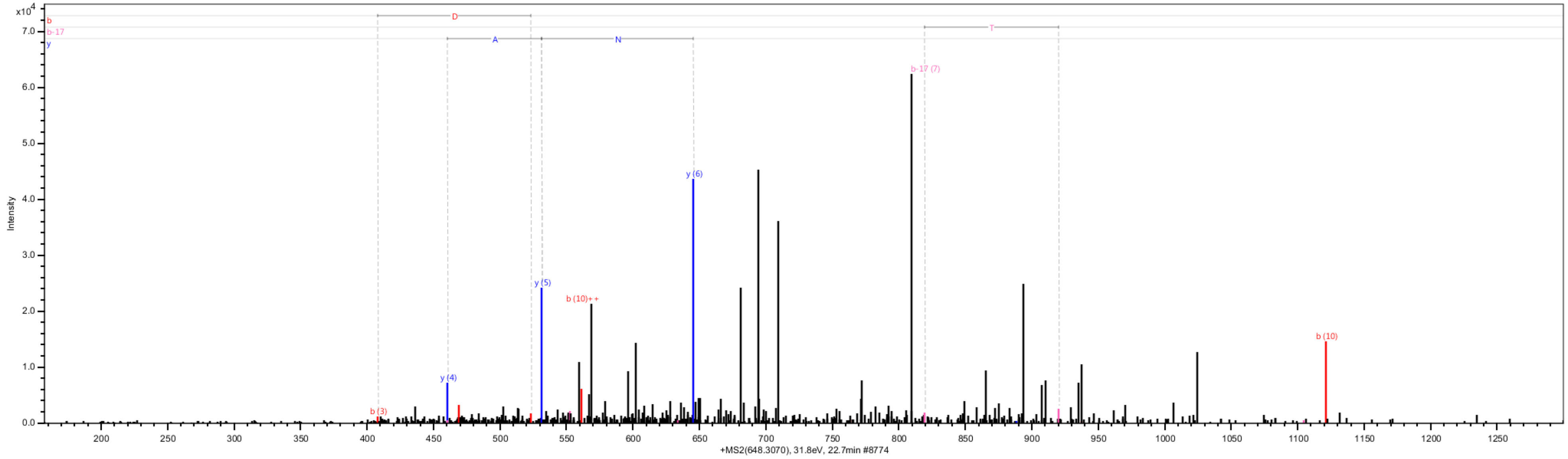

MPRI_HUMAN

Accession: MPRI_HUMAN
Database: SwissProt
MW [kDa] / pI: 274.2 / 5.6
Score: 15.4
Seq. Coverage [%]: 0.3
No. of Peptides: 1

Peptide Match

Sequence: K.LECKFVQK.H
Score: 15.4
Modifications: Carbamidomethyl: 3

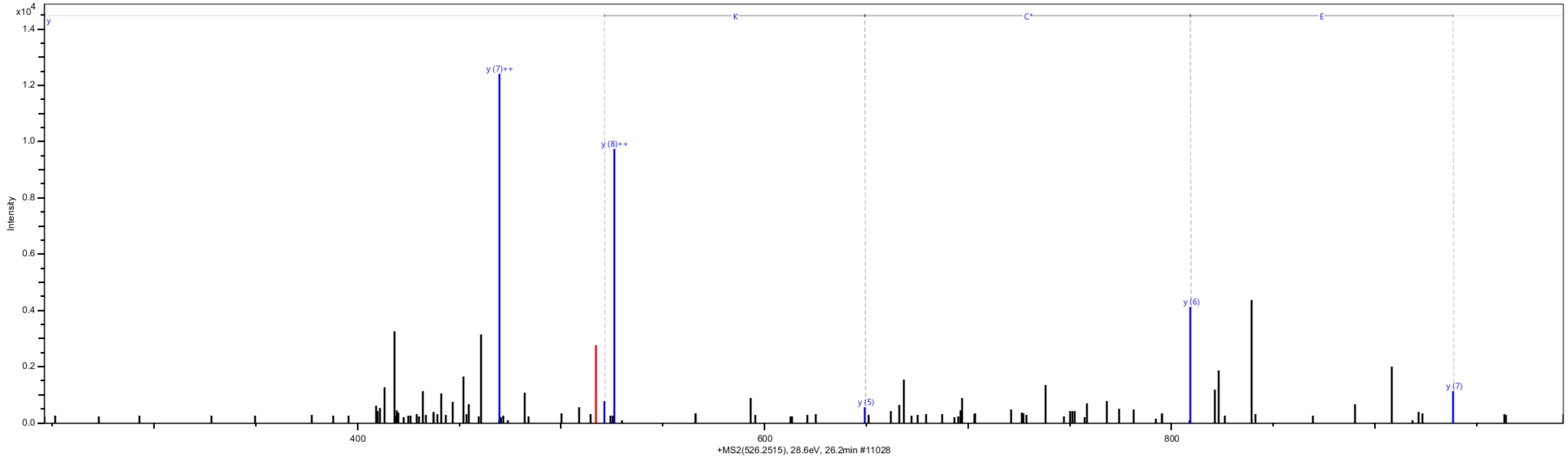

HIP1R_HUMAN

Accession: HIP1R_HUMAN
Database: SwissProt
MW [kDa] / pI: 119.3 / 6.2
Score: 15.4
Seq. Coverage [%]: 0.8
No. of Peptides: 1

Peptide Match

Sequence: R.IILGTHHEK.G
Score: 15.4
Modifications:

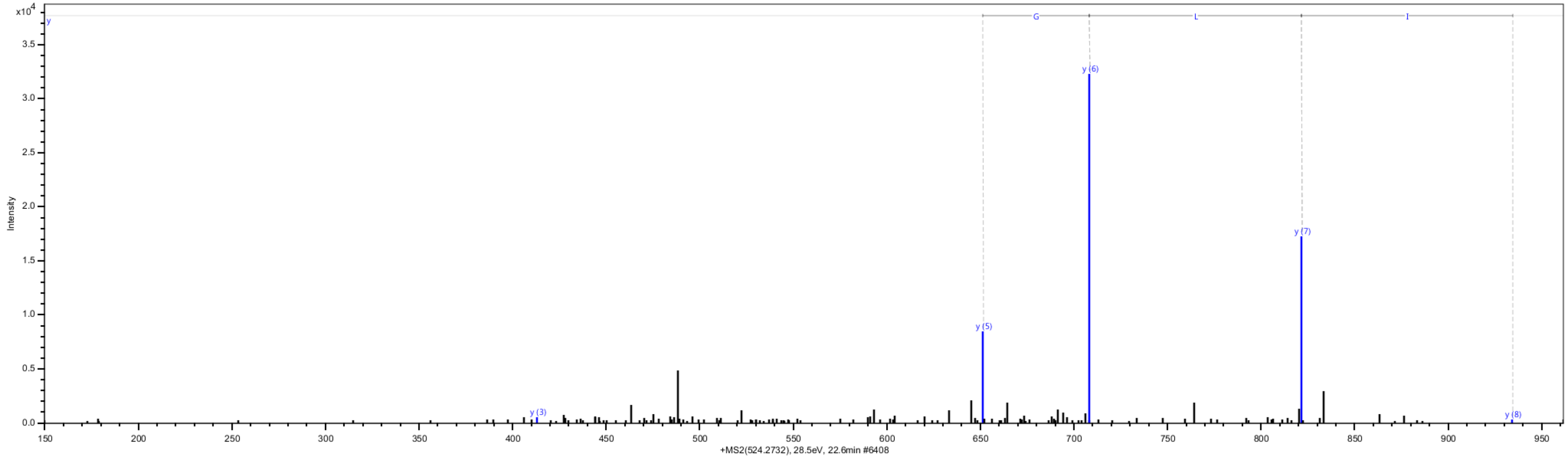

RHDF1_HUMAN

Accession: RHDF1_HUMAN
Database: SwissProt
MW [kDa] / pI: 97.3 / 8.8
Score: 15.4
Seq. Coverage [%]: 1.1
No. of Peptides: 1

Peptide Match

Sequence: R.AAAALMKGR.S
Score: 15.4
Modifications: Oxidation: 6

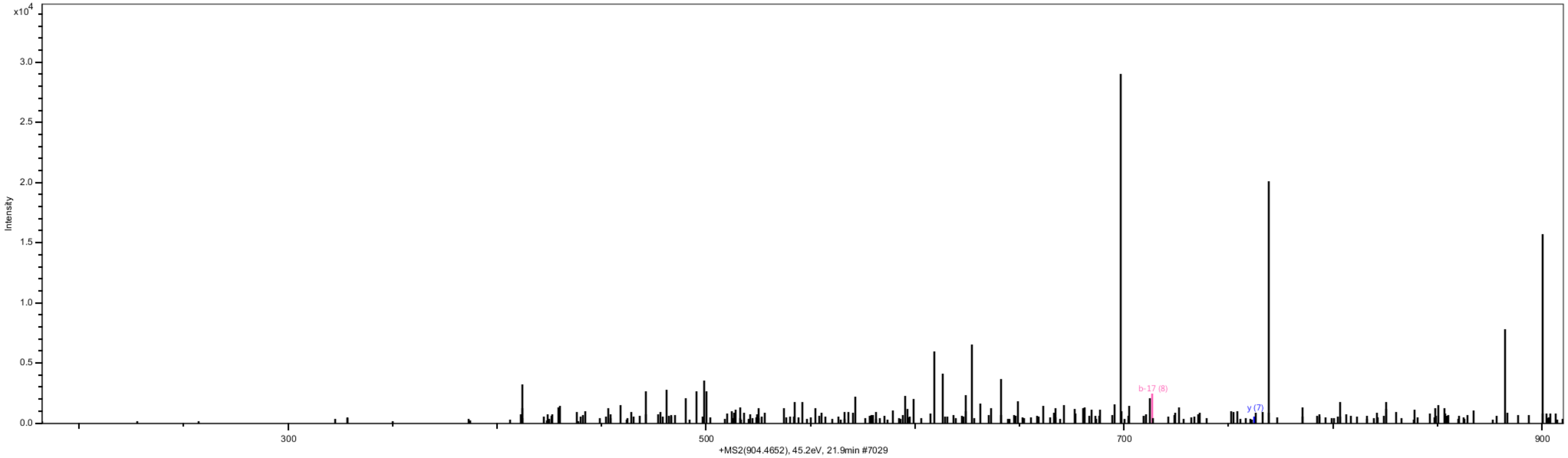

SAMD7_HUMAN

Accession: SAMD7_HUMAN
Database: SwissProt
MW [kDa] / pI: 49.1 / 6.3
Score: 15.1
Seq. Coverage [%]: 2.7
No. of Peptides: 1

Peptide Match

Sequence: R.SMLPAGDLHFHR.S
Score: 15.1
Modifications:

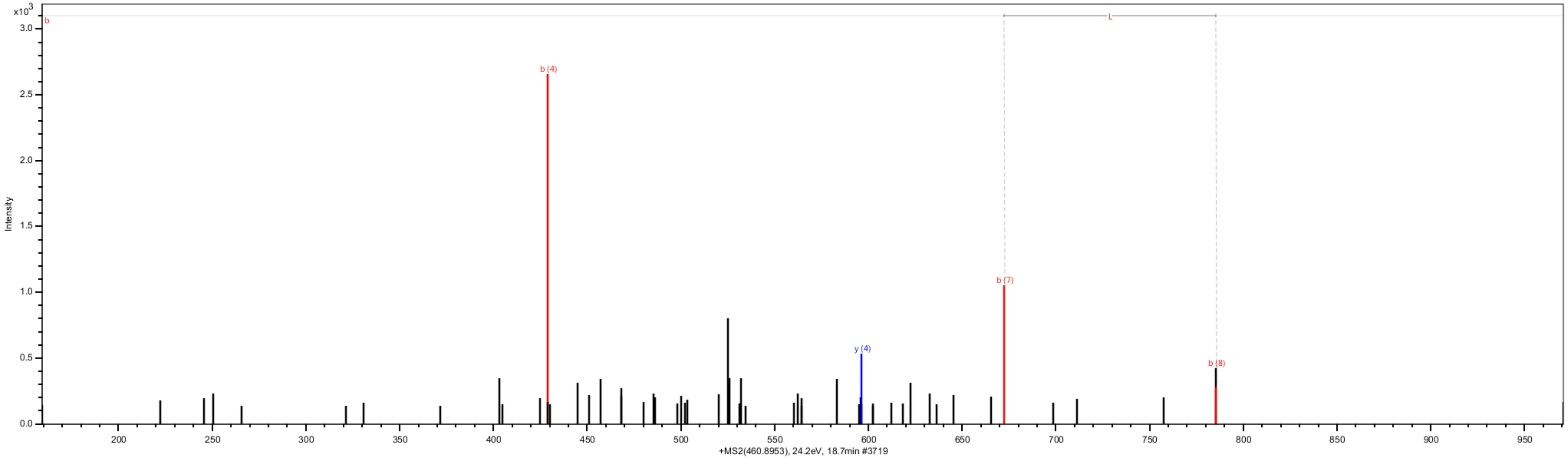

ARVC_HUMAN

Accession: ARVC_HUMAN
Database: SwissProt
MW [kDa] / pI: 104.6 / 6.4
Score: 15.1
Seq. Coverage [%]: 0.9
No. of Peptides: 1

Peptide Match

Sequence: R.NFDTLDLPK.R
Score: 15.1
Modifications:

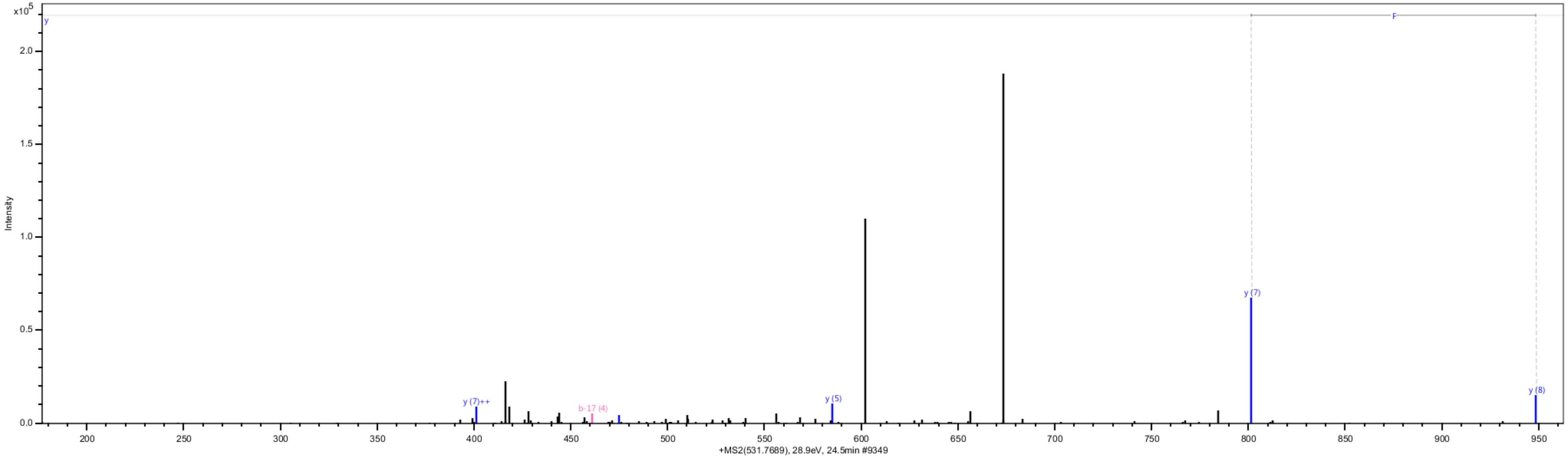

NEBL_HUMAN

**Accession:**	NEBL_HUMAN	**Score:**	15.1
**Database:**	SwissProt	**Seq. Coverage [%]:**	1.3
**MW [kDa] / pI:**	116.4 / 7.9	**No. of Peptides:**	1

Peptide Match

**Sequence:**	K.GMQVSTDTLDVQR.A	**Score:**	15.1
**Modifications:**	Oxidation: 2		

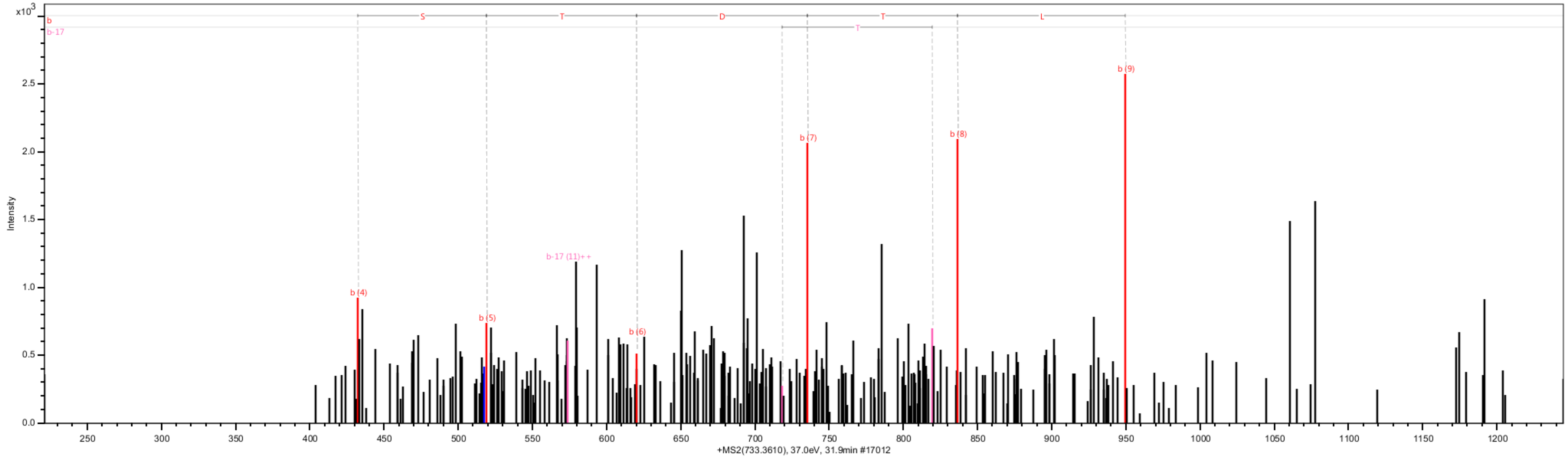

FAD1_HUMAN

Accession: FAD1_HUMAN
Database: SwissProt
MW [kDa] / pI: 65.2 / 6.5
Score: 15.1
Seq. Coverage [%]: 1.5
No. of Peptides: 1

Peptide Match

Sequence: K.ALGGEGWEK.L
Score: 15.1
Modifications:

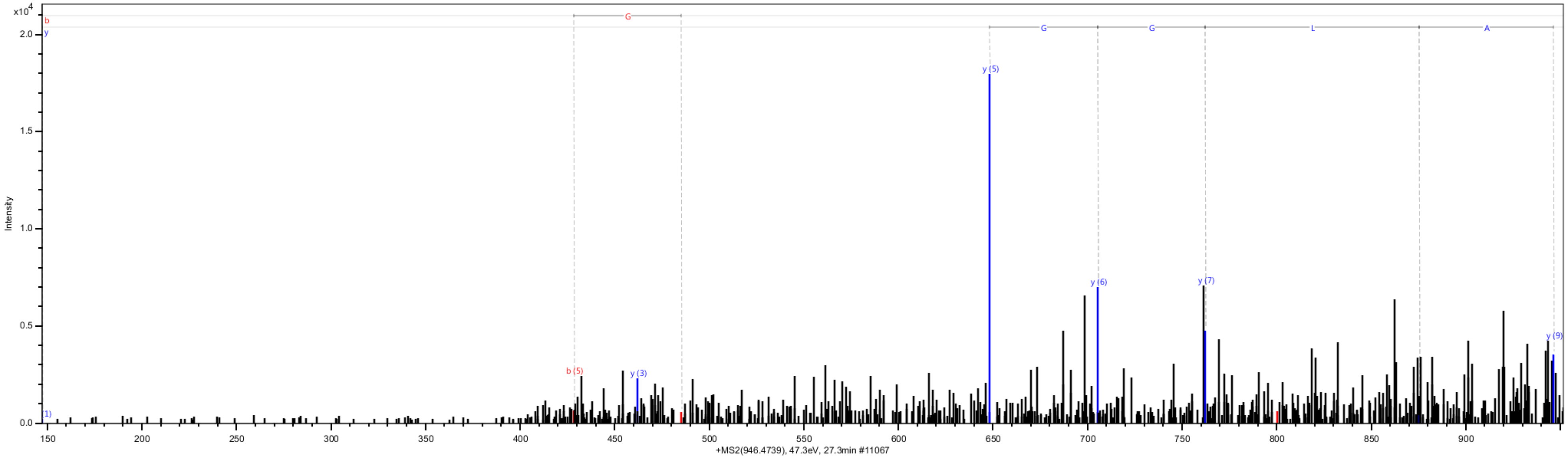

ASXL1_HUMAN

**Accession:**	ASXL1_HUMAN	**Score:**	15.1
**Database:**	SwissProt	**Seq. Coverage [%]:**	0.5
**MW [kDa] / pI:**	165.3 / 5.8	**No. of Peptides:**	1

Peptide Match

**Sequence:**	R.QEMEKEK.K	**Score:**	15.1
**Modifications:**	Oxidation: 3		

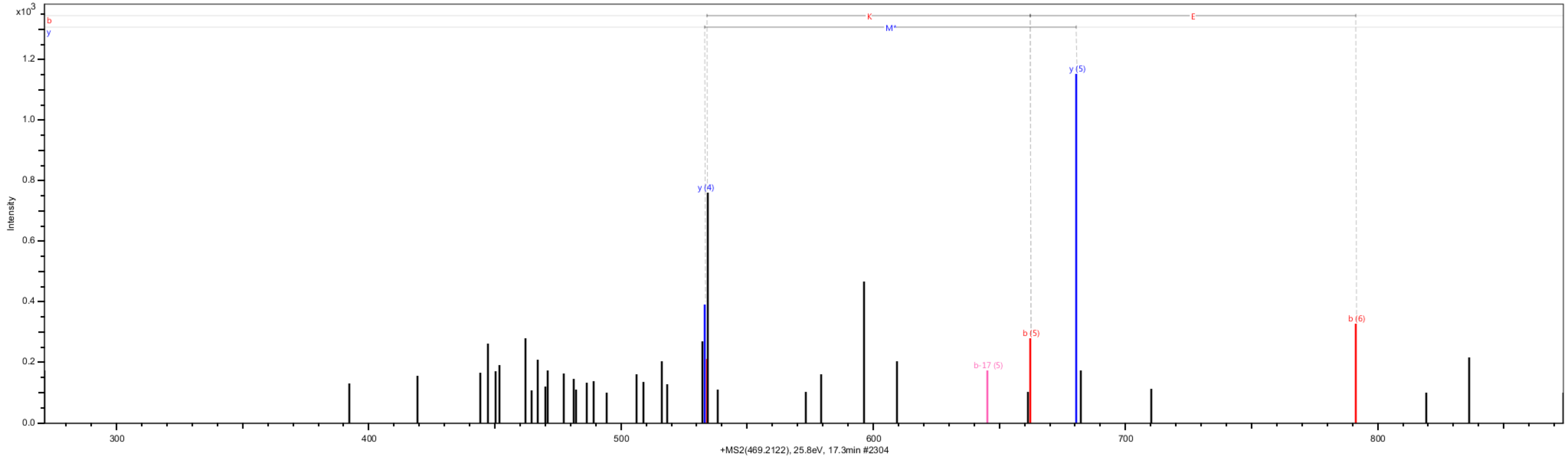

PAAT_HUMAN

**Accession:**	PAAT_HUMAN	**Score:**	15.1
**Database:**	SwissProt	**Seq. Coverage [%]:**	3.6
**MW [kDa] / pI:**	49.2 / 5.9	**No. of Peptides:**	1

Peptide Match

**Sequence:**	R.IDLDKVQTIMESMGSK.L	**Score:**	15.1
**Modifications:**	Oxidation: 10, 13		

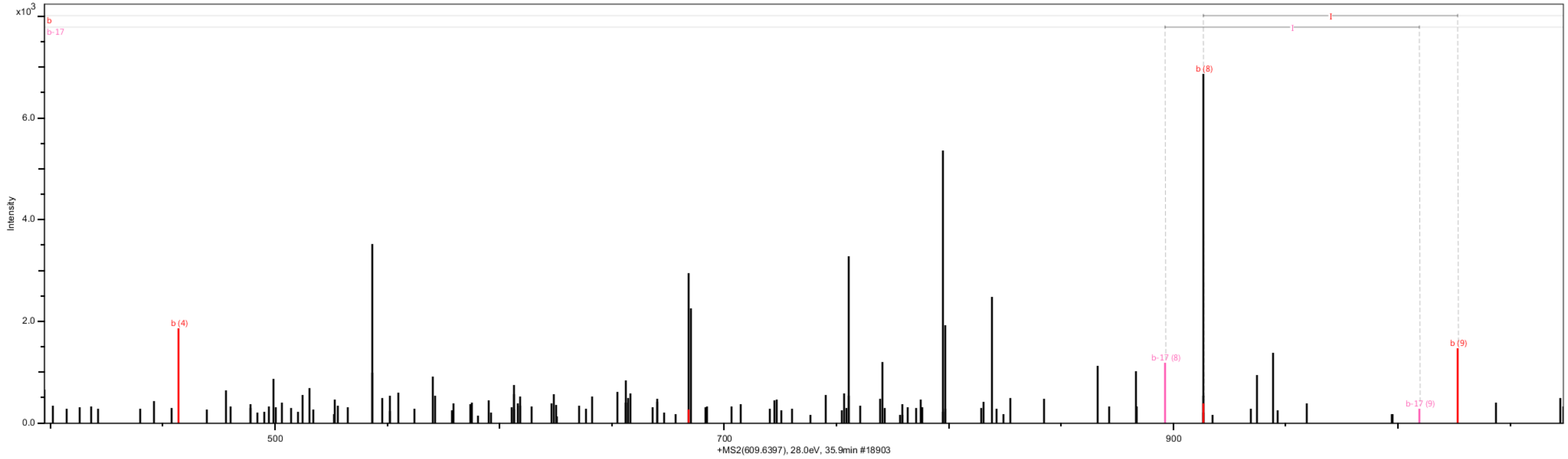

AT7L2_HUMAN

Accession: AT7L2_HUMAN
Database: SwissProt
MW [kDa] / pI: 77.1 / 9.4
Score: 15.1
Seq. Coverage [%]: 2.5
No. of Peptides: 1

Peptide Match

Sequence: K.DSPGKPPMAPPSKEPPGR.E
Score: 15.1
Modifications: Oxidation: 8

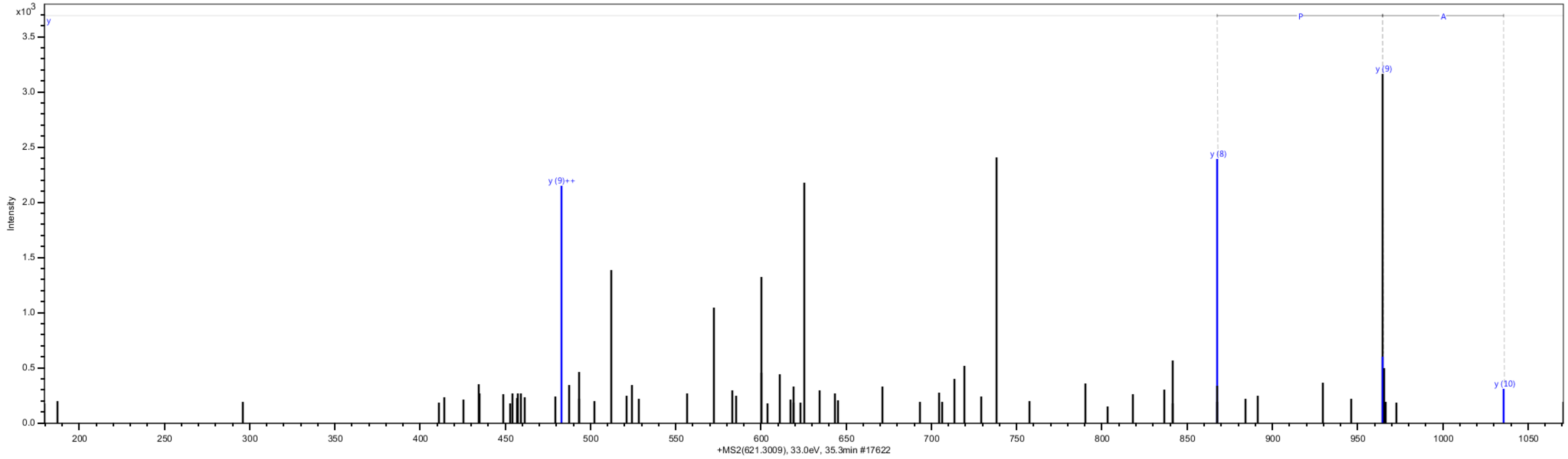

CSN7A_HUMAN

**Accession:**	CSN7A_HUMAN	**Score:**	15.1
**Database:**	SwissProt	**Seq. Coverage [%]:**	8.0
**MW [kDa] / pI:**	30.3 / 8.3	**No. of Peptides:**	1

Peptide Match

**Sequence:**	R.TLQEWCVGCEVVLSGIEEQVSR.A	**Score:**	15.1
**Modifications:**	Carbamidomethyl: 6, 9		

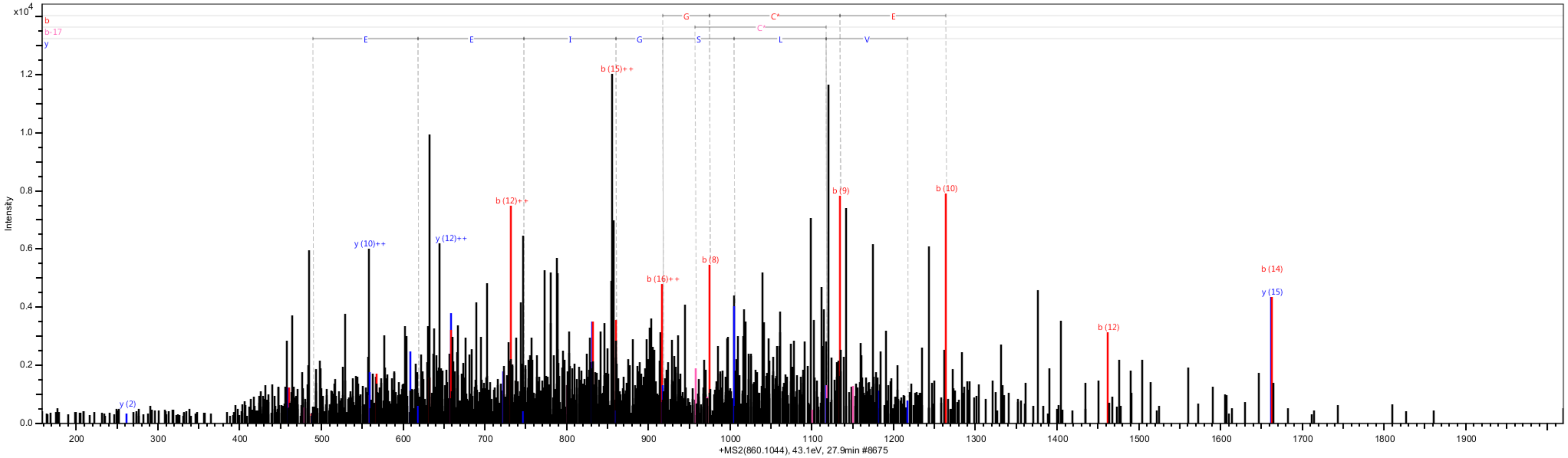

CLUS_HUMAN

Accession: CLUS_HUMAN
Database: SwissProt
MW [kDa] / pI: 52.5 / 5.9
Score: 15.2
Seq. Coverage [%]: 2.4
No. of Peptides: 1

Peptide Match

Sequence: R.ELDESLQVAER.L
Score: 15.2
Modifications:

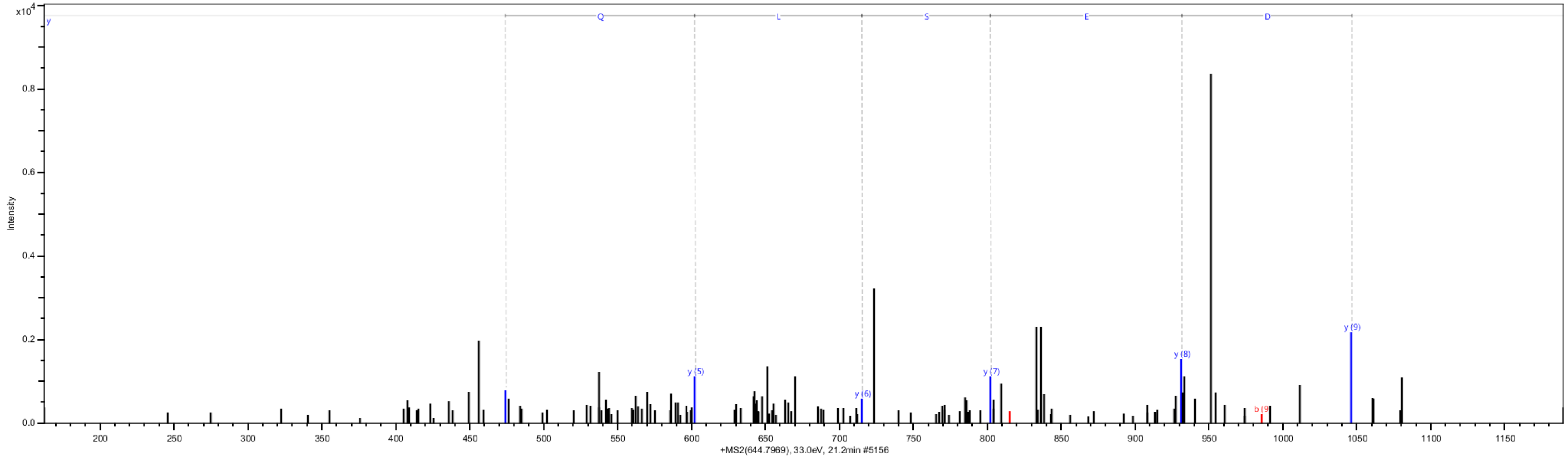

LYPA2_HUMAN

Accession: LYPA2_HUMAN
Database: SwissProt
MW [kDa] / pI: 24.7 / 6.7
Score: 15.2
Seq. Coverage [%]: 3.5
No. of Peptides: 1

Peptide Match

Sequence: K.ALIEHEMK.N
Score: 15.2
Modifications: Oxidation: 7

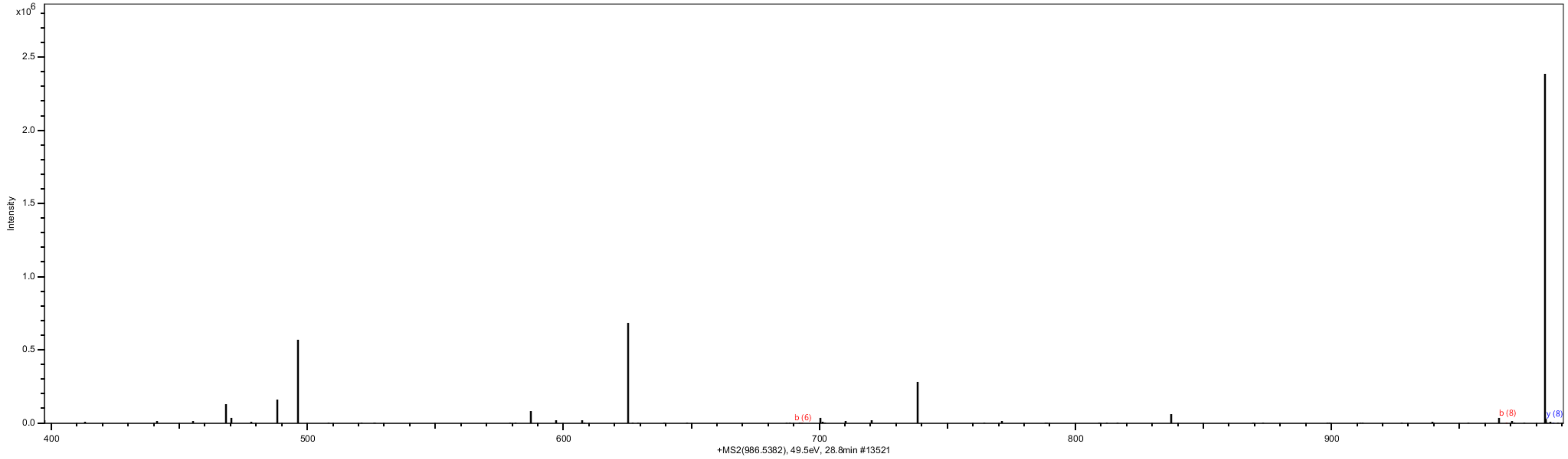

MOBP_HUMAN

Accession: MOBP_HUMAN
Database: SwissProt
MW [kDa] / pI: 20.9 / 11.3
Score: 15.1
Seq. Coverage [%]: 6.0
No. of Peptides: 1

Peptide Match

Sequence: R.SSPLRGPGASR.G
Score: 15.1
Modifications:

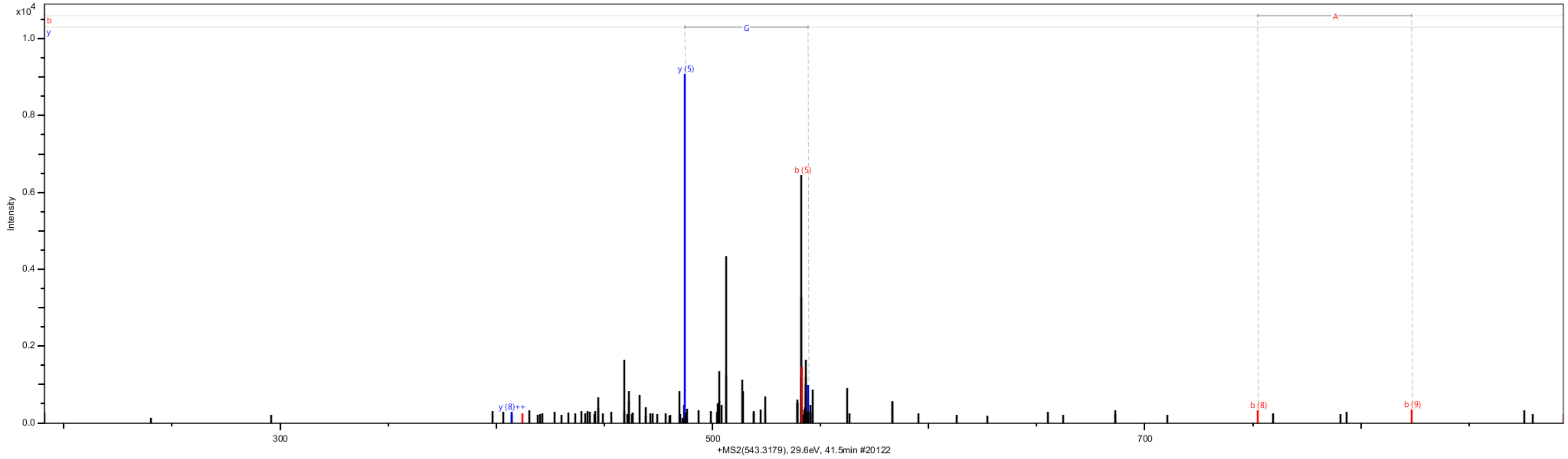

TOP1M_HUMAN

**Accession:**	TOP1M_HUMAN	**Score:**	15.2
**Database:**	SwissProt	**Seq. Coverage [%]:**	2.3
**MW [kDa] / pI:**	69.8 / 9.5	**No. of Peptides:**	1

Peptide Match

**Sequence:**	R.LSVAAEEVATFYGR.M	**Score:**	15.2
**Modifications:**			

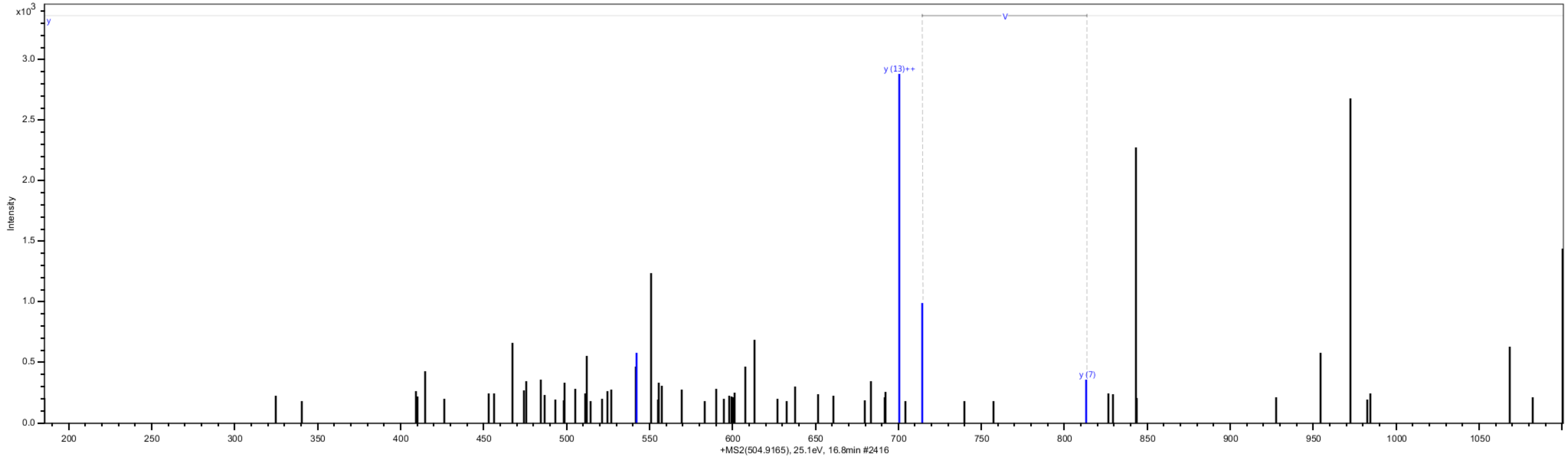

EXOC1_HUMAN

Accession: EXOC1_HUMAN
Database: SwissProt
MW [kDa] / pI: 101.9 / 6.2
Score: 15.2
Seq. Coverage [%]: 1.2
No. of Peptides: 1

Peptide Match

Sequence: K.ENPEFDLHFEK.I
Score: 15.2
Modifications:

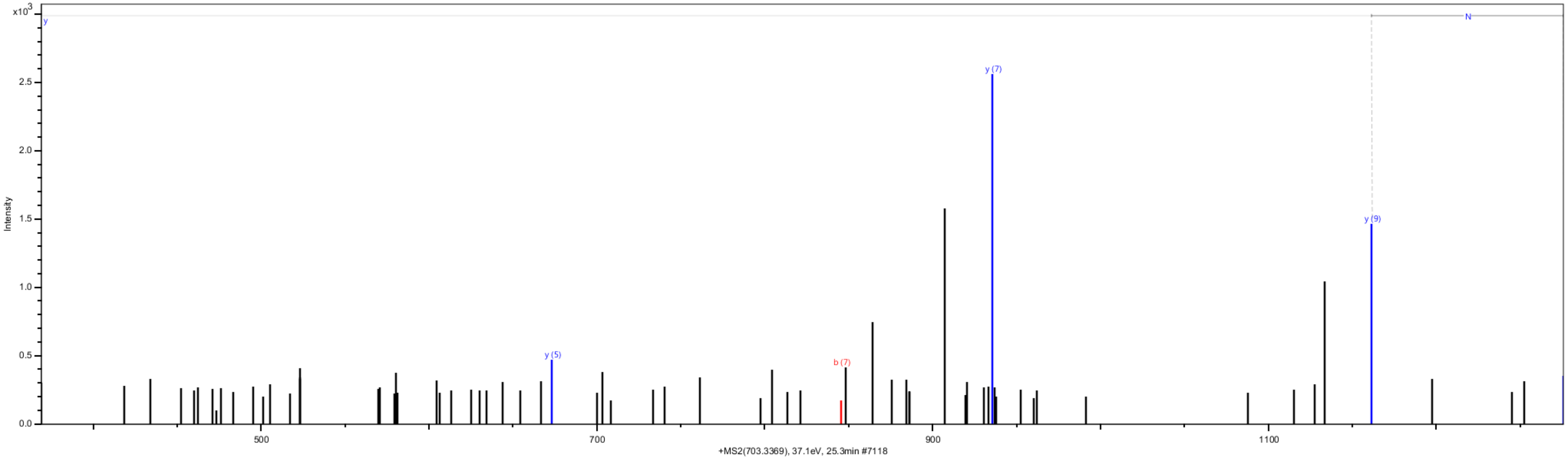

MND1_HUMAN

Accession: MND1_HUMAN
Database: SwissProt
MW [kDa] / pI: 23.7 / 8.3
Score: 15.2
Seq. Coverage [%]: 3.9
No. of Peptides: 1

Peptide Match

Sequence: K.GLSAEEKR.T
Score: 15.2
Modifications:

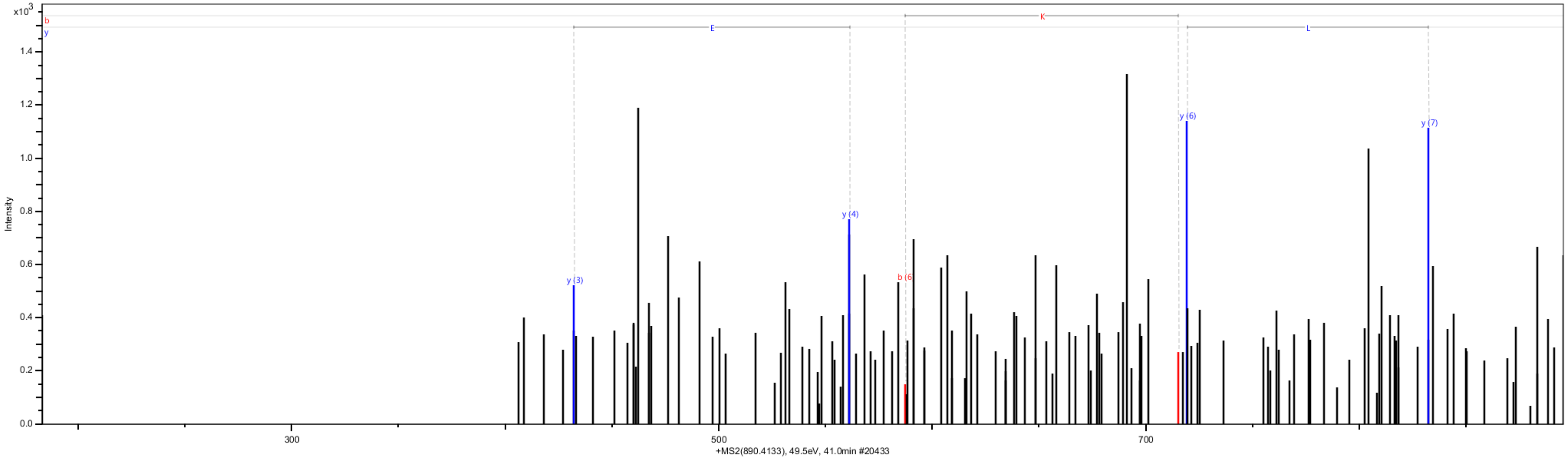

SYVM_HUMAN

Accession: SYVM_HUMAN
Database: SwissProt
MW [kDa] / pI: 118.4 / 6.6
Score: 15.2
Seq. Coverage [%]: 1.6
No. of Peptides: 1

Peptide Match

Sequence: R.SGNLDPAELAIVAAAQK.K
Score: 15.2
Modifications:

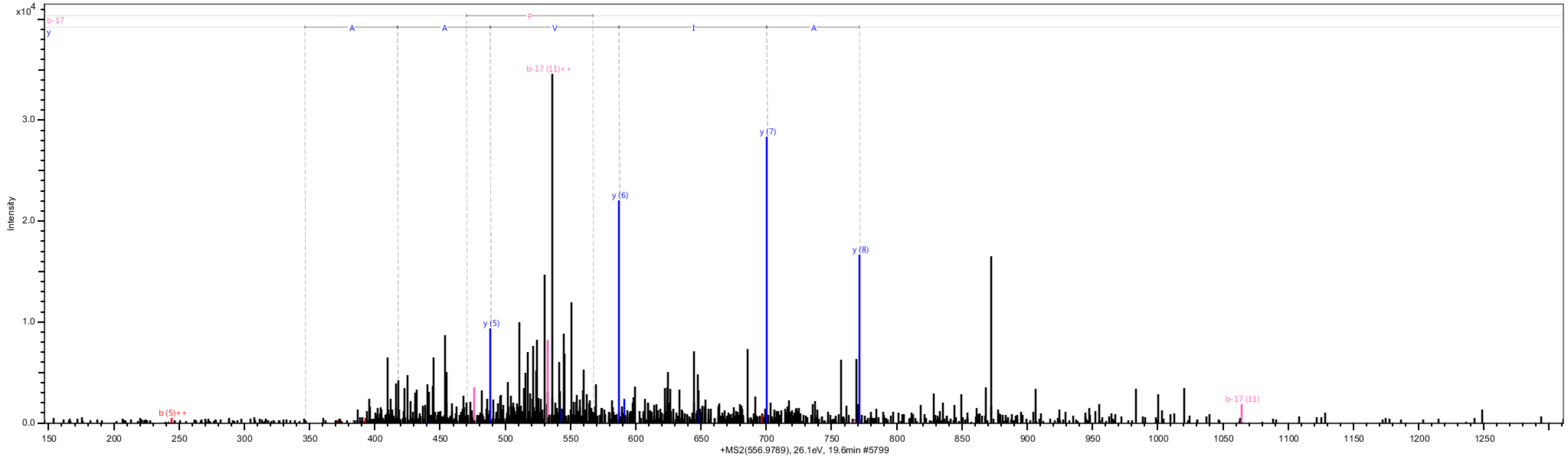

NR2E3_HUMAN

Accession: NR2E3_HUMAN
Database: SwissProt
MW [kDa] / pI: 44.7 / 8.2
Score: 15.2
Seq. Coverage [%]: 2.2
No. of Peptides: 1

Peptide Match

Sequence: R.LTLASMETR.V
Modifications: Oxidation: 6
Score: 15.2

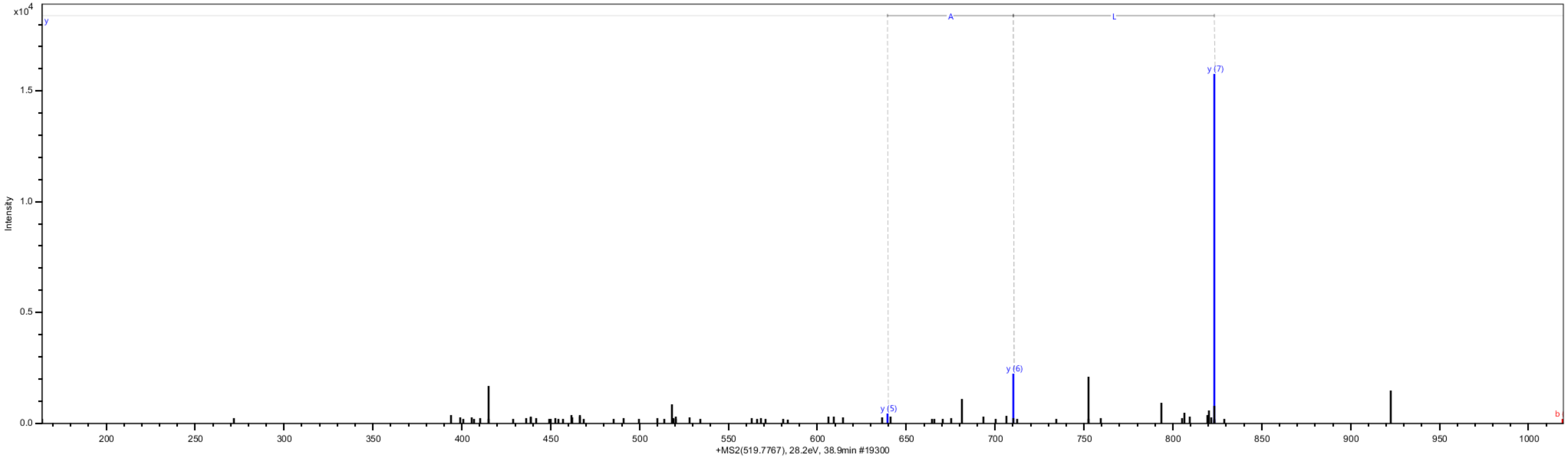

LRC34_HUMAN

Accession: LRC34_HUMAN
Database: SwissProt
MW [kDa] / pI: 51.2 / 6.3
Score: 15.2
Seq. Coverage [%]: 2.6
No. of Peptides: 1

Peptide Match

Sequence: -.MAAQPPRPVGER.S
Score: 15.2
Modifications:

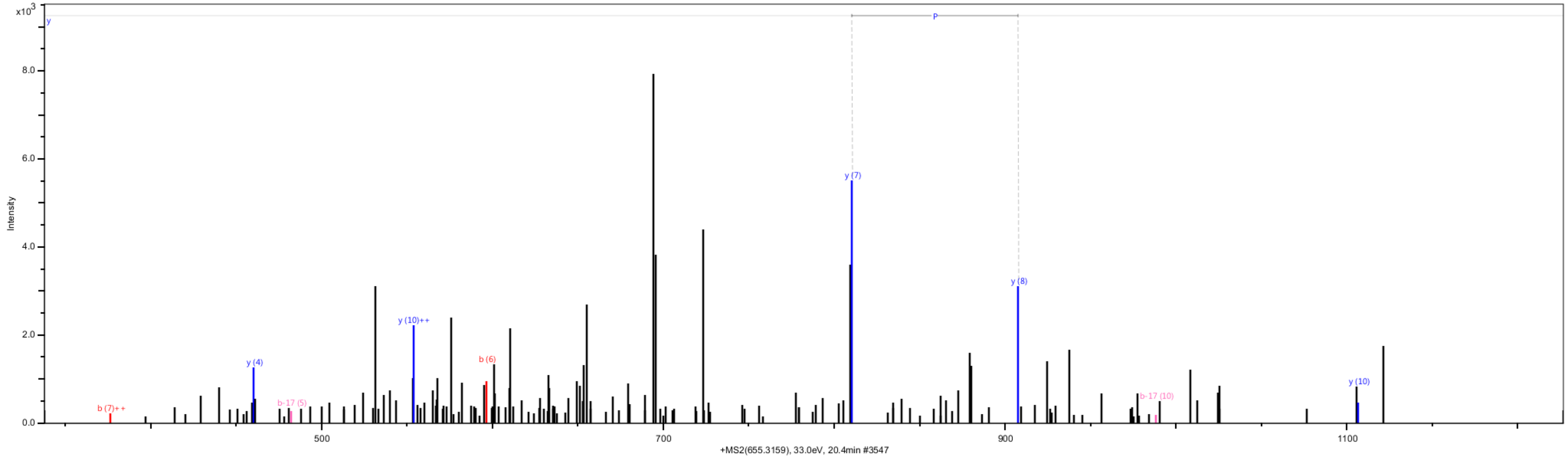

HUNK_HUMAN

**Accession:**	HUNK_HUMAN	**Score:**	15.2
**Database:**	SwissProt	**Seq. Coverage [%]:**	1.3
**MW [kDa] / pI:**	79.6 / 9.2	**No. of Peptides:**	1

Peptide Match

**Sequence:**	R.ALYQKMVDK.E	**Score:**	15.2
**Modifications:**	Oxidation: 6		

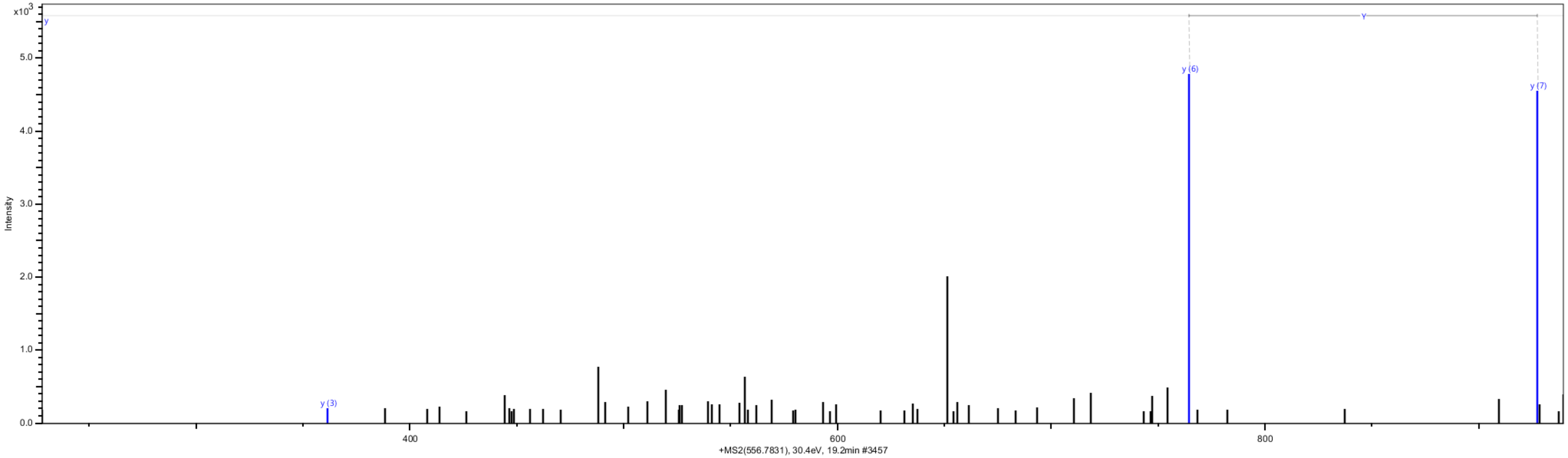

ZN806_HUMAN

Accession: ZN806_HUMAN
Database: SwissProt
MW [kDa] / pI: 67.8 / 8.8
Score: 15.2
Seq. Coverage [%]: 1.5
No. of Peptides: 1

Peptide Match

Sequence: R.VHTGEMPYK.C
Score: 15.2
Modifications:

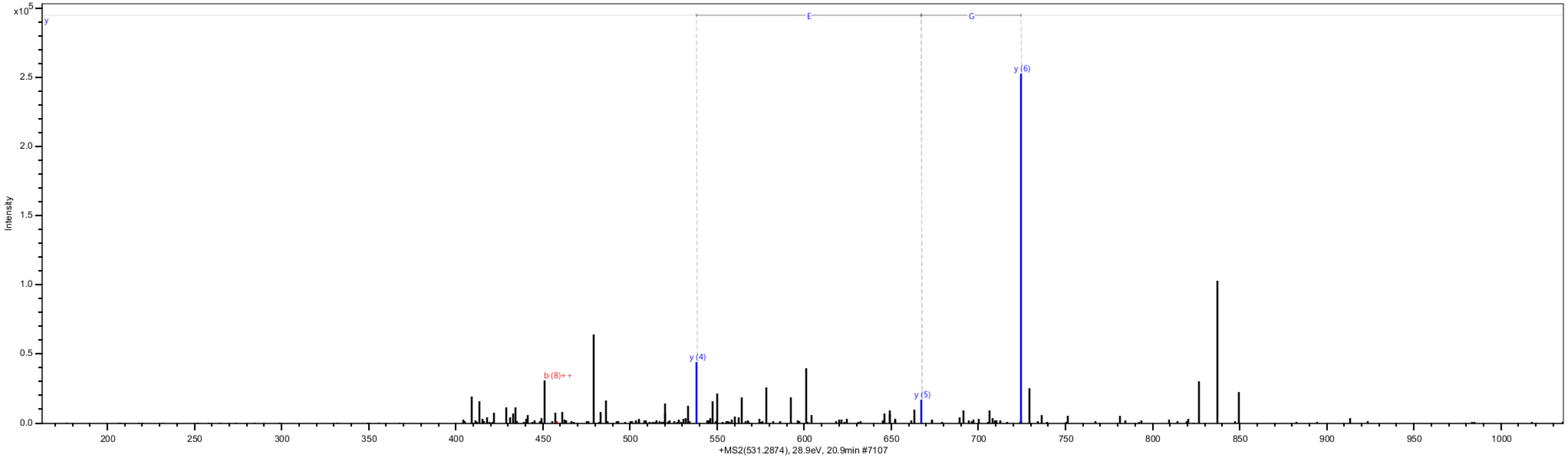

ROCK2_HUMAN

**Accession:**	ROCK2_HUMAN	**Score:**	15.2
**Database:**	SwissProt	**Seq. Coverage [%]:**	0.7
**MW [kDa] / pI:**	160.8 / 5.8	**No. of Peptides:**	1

Peptide Match

**Sequence:**	K.KPPAPDPFAR.S	**Score:**	15.2
**Modifications:**			

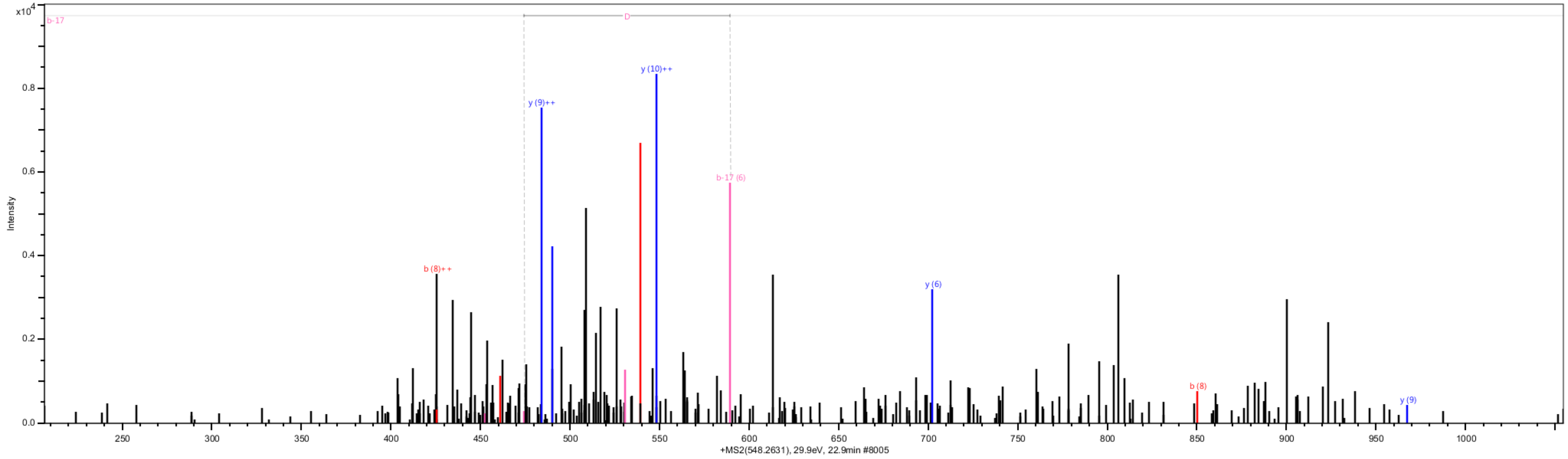

MEGF6_HUMAN

Accession: MEGF6_HUMAN
Database: SwissProt
MW [kDa] / pI: 161.1 / 5.9
Score: 15.2
Seq. Coverage [%]: 0.9
No. of Peptides: 1

Peptide Match

Sequence: R.GCEPGSFGEGCHQR.C
Score: 15.2
Modifications: Carbamidomethyl: 2, 11

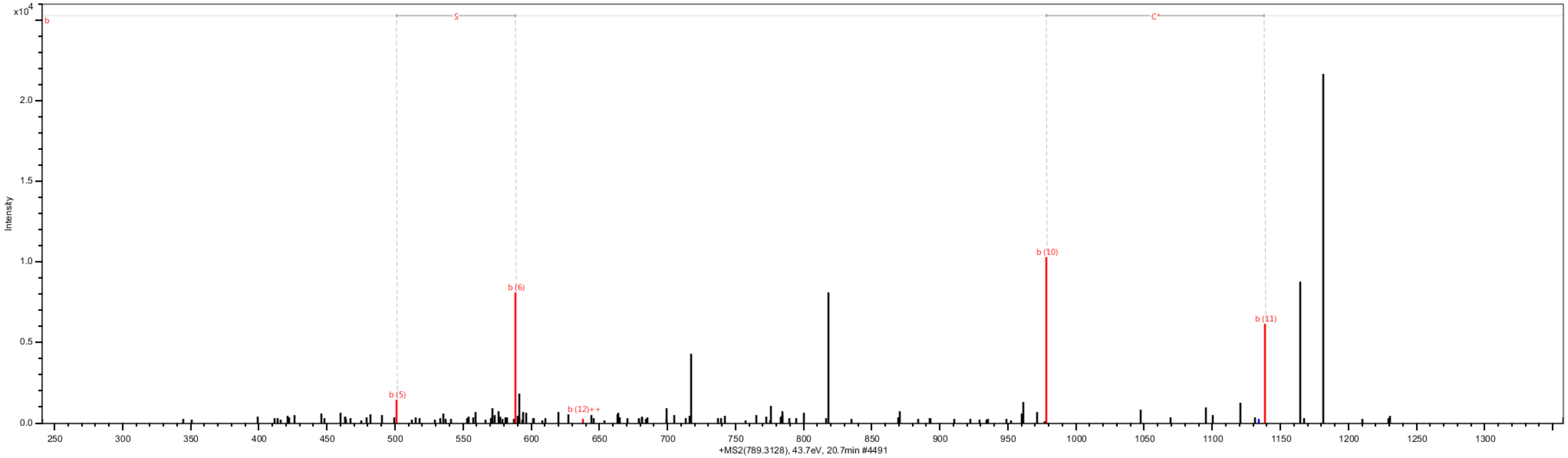

ZN608_HUMAN

Accession: ZN608_HUMAN
Database: SwissProt
MW [kDa] / pI: 162.1 / 8.9
Score: 15.2
Seq. Coverage [%]: 1.0
No. of Peptides: 1

Peptide Match

Sequence: K.APASPGAGNPPGTPK.G
Score: 15.2
Modifications:

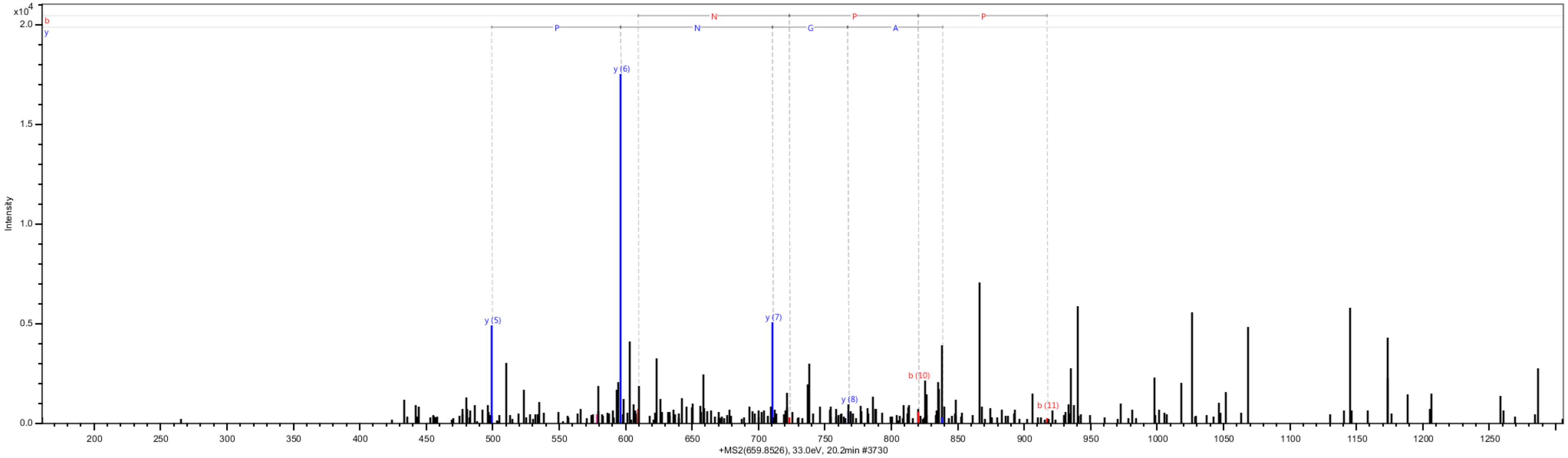

ZDH15_HUMAN

Accession: ZDH15_HUMAN
Database: SwissProt
MW [kDa] / pI: 39.3 / 8.4
Score: 15.2
Seq. Coverage [%]: 3.3
No. of Peptides: 1

Peptide Match

Sequence: R.GWKMALSGGLR.C
Score: 15.2
Modifications:

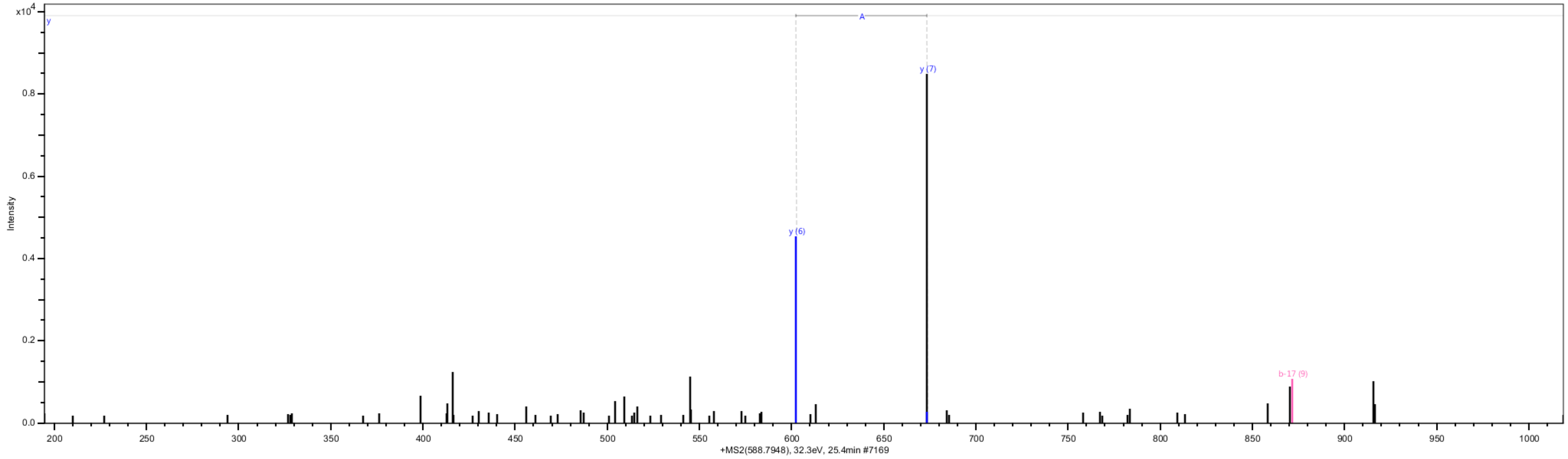

LEG2_HUMAN

**Accession:**	LEG2_HUMAN	**Score:**	15.2
**Database:**	SwissProt	**Seq. Coverage [%]:**	8.3
**MW [kDa] / pI:**	14.6 / 5.9	**No. of Peptides:**	1

Peptide Match

**Sequence:**	K.NMDMKPGSTLK.I	**Score:**	15.2
**Modifications:**	Oxidation: 2		

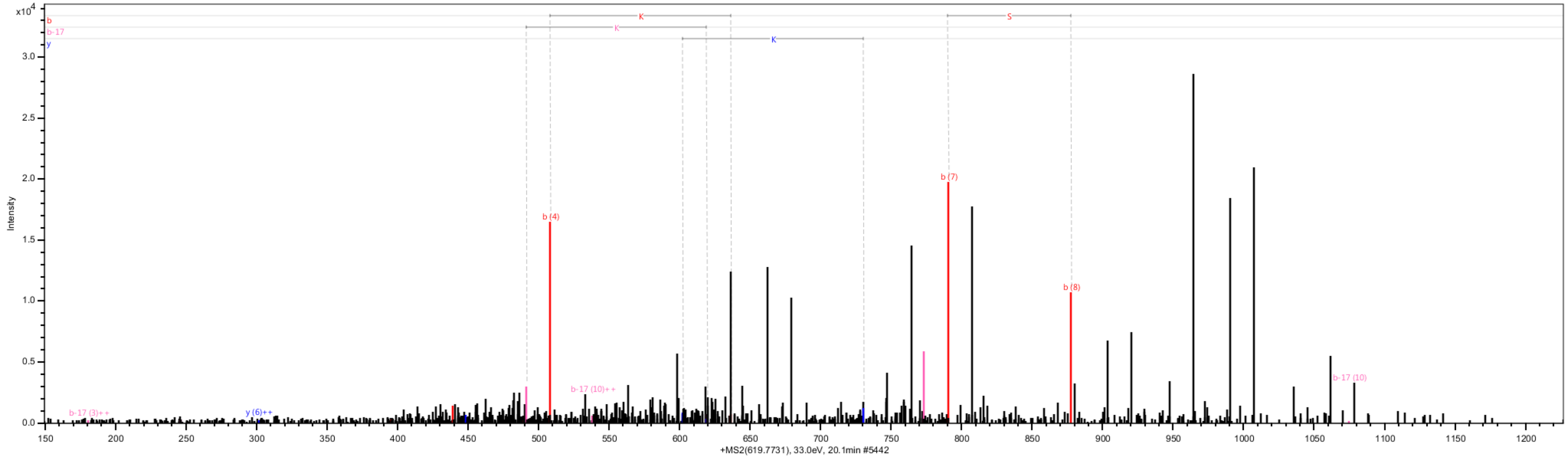

SI1L2_HUMAN

**Accession:**	SI1L2_HUMAN	**Score:**	15.2
**Database:**	SwissProt	**Seq. Coverage [%]:**	1.0
**MW [kDa] / pI:**	190.3 / 6.3	**No. of Peptides:**	1

Peptide Match

**Sequence:**	K.EDFLKLMLPDSPLVEEGR.R	**Score:**	15.2
**Modifications:**			

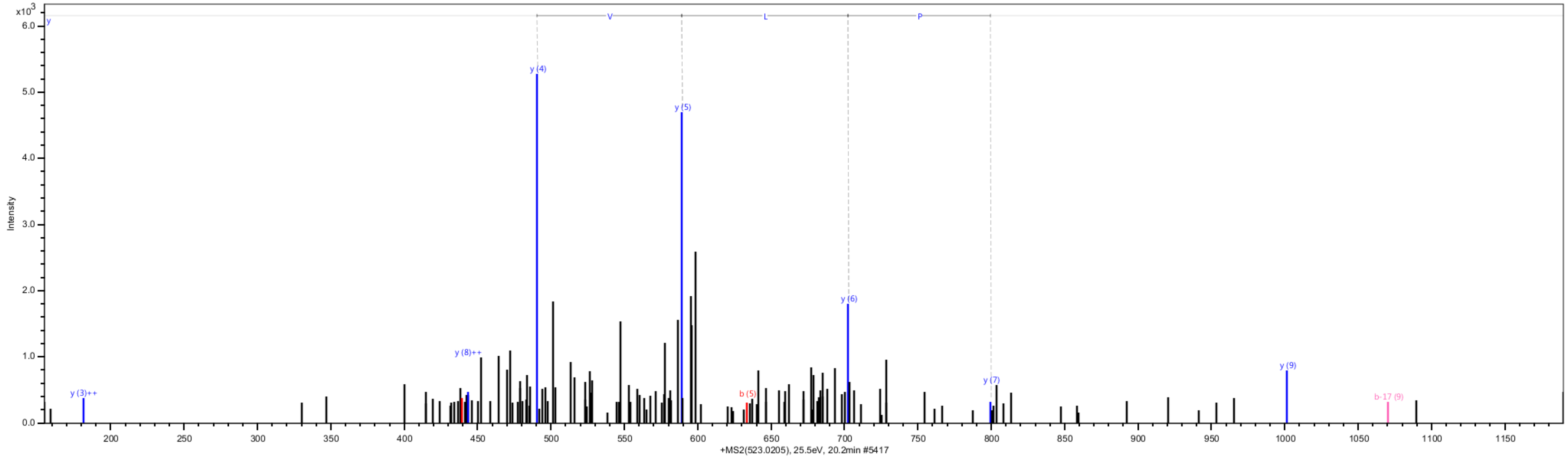

B3GT2_HUMAN

Accession: B3GT2_HUMAN | **Score:** 15.2
Database: SwissProt | **Seq. Coverage [%]:** 2.8
MW [kDa] / pI: 49.2 / 9.5 | **No. of Peptides:** 1

Peptide Match

Sequence: K.WYMPPDLYPSER.Y | **Score:** 15.2
Modifications: Oxidation: 3

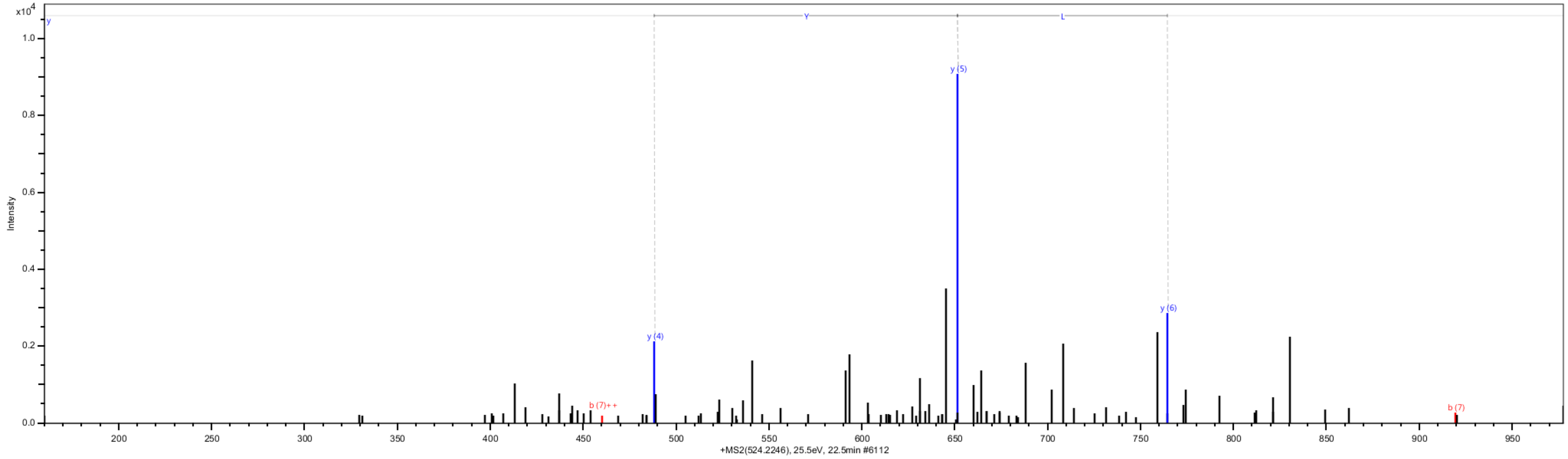

FBH1_HUMAN

Accession: FBH1_HUMAN
Database: SwissProt
MW [kDa] / pI: 117.6 / 8.6
Score: 15.2
Seq. Coverage [%]: 0.8
No. of Peptides: 1

Peptide Match

Sequence: R.YVTAAEDK.E
Score: 15.2
Modifications:

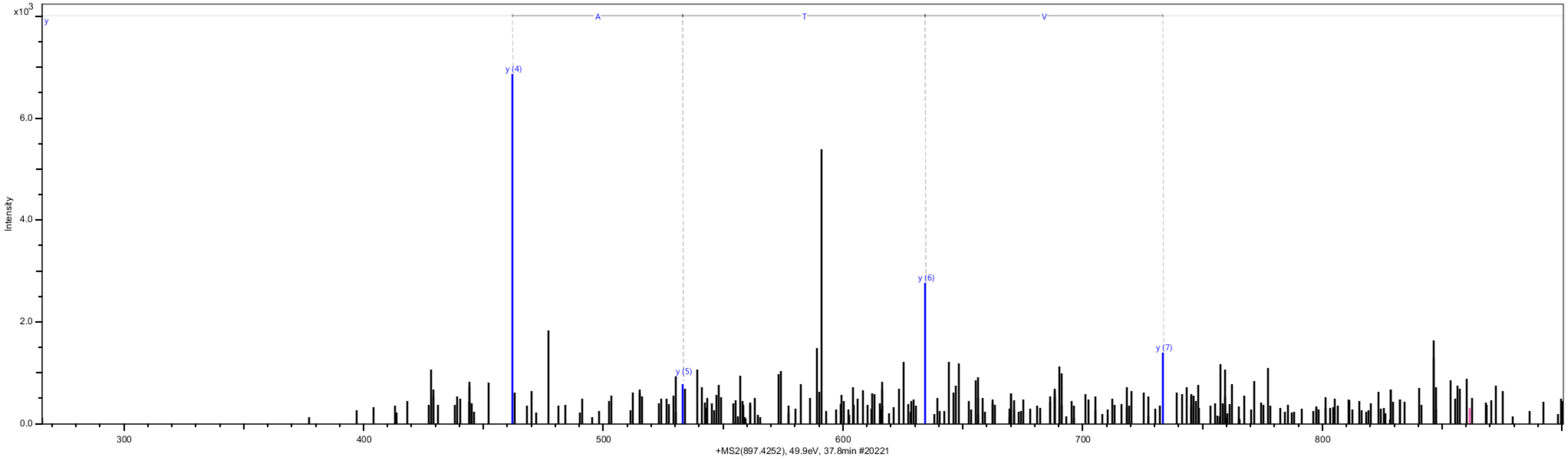

T3HPD_HUMAN

Accession: T3HPD_HUMAN
Database: SwissProt
MW [kDa] / pI: 38.1 / 6.2
Score: 15.2
Seq. Coverage [%]: 2.3
No. of Peptides: 1

Peptide Match

Sequence: M.ESALAVPR.L
Score: 15.2
Modifications:

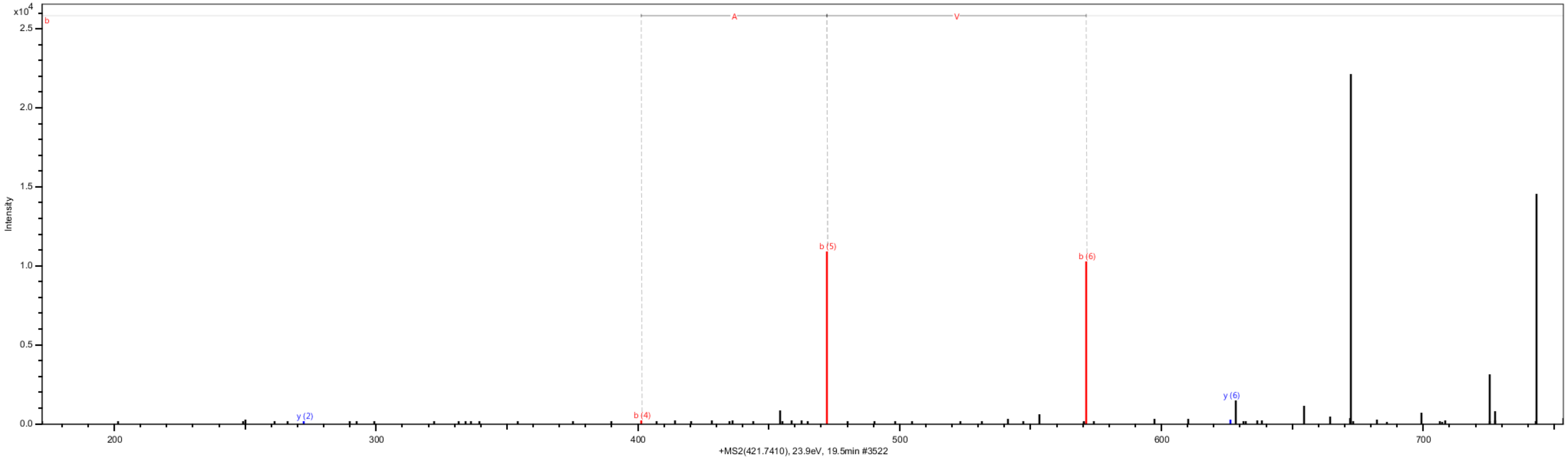

DIAP1_HUMAN

Accession: DIAP1_HUMAN
Database: SwissProt
MW [kDa] / pI: 141.3 / 5.3
Score: 15.2
Seq. Coverage [%]: 0.6
No. of Peptides: 1

Peptide Match

Sequence: K.EKPNSAHR.N
Score: 15.2
Modifications:

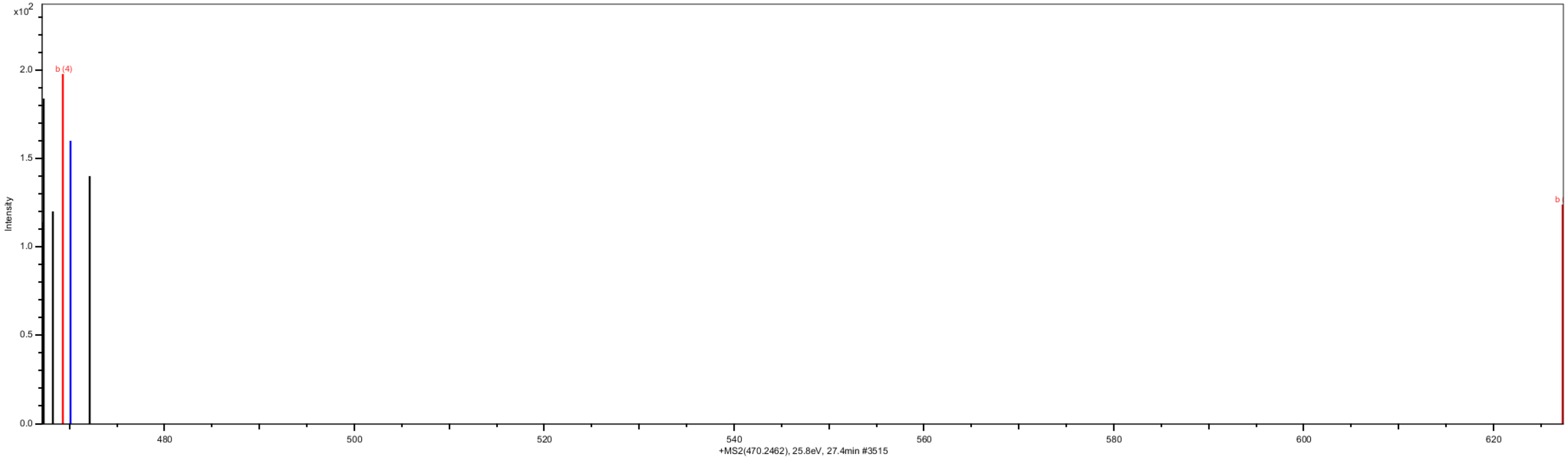

UCKL1_HUMAN

**Accession:**	UCKL1_HUMAN	**Score:**	15.2
**Database:**	SwissProt	**Seq. Coverage [%]:**	1.5
**MW [kDa] / pI:**	61.1 / 6.9	**No. of Peptides:**	1

Peptide Match

**Sequence:**	R.LADIVVPR.G	**Score:**	15.2
**Modifications:**			

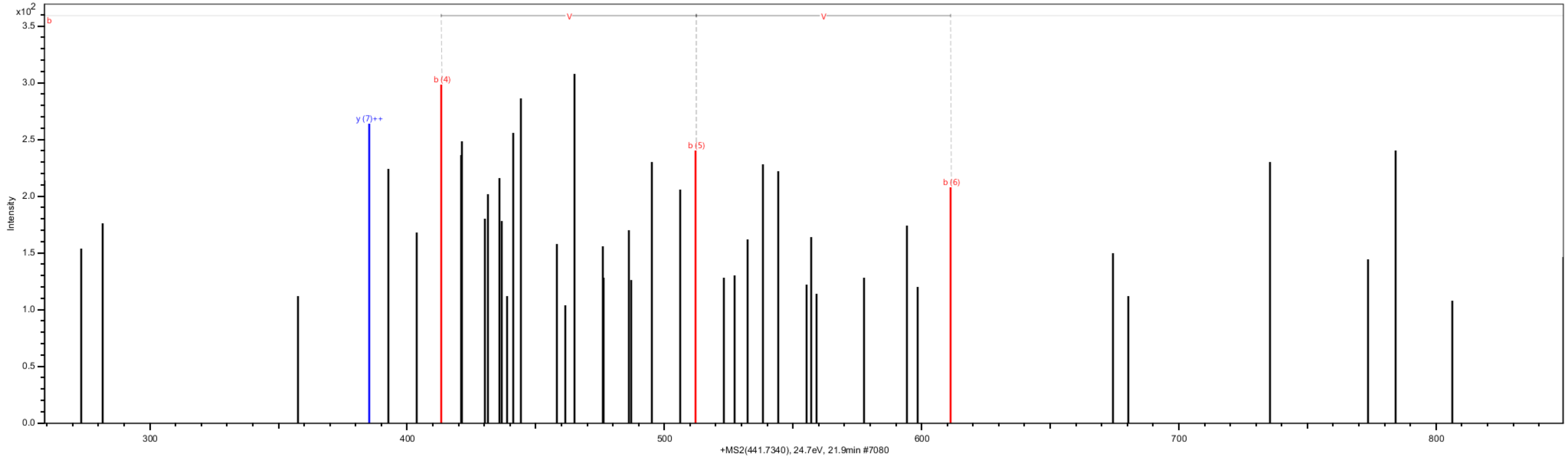

MRP6_HUMAN

**Accession:**	MRP6_HUMAN	**Score:**	15.2
**Database:**	SwissProt	**Seq. Coverage [%]:**	0.7
**MW [kDa] / pI:**	164.8 / 8.8	**No. of Peptides:**	1

Peptide Match

**Sequence:**	R.ISFPRLVADR.W	**Score:**	15.2
**Modifications:**			

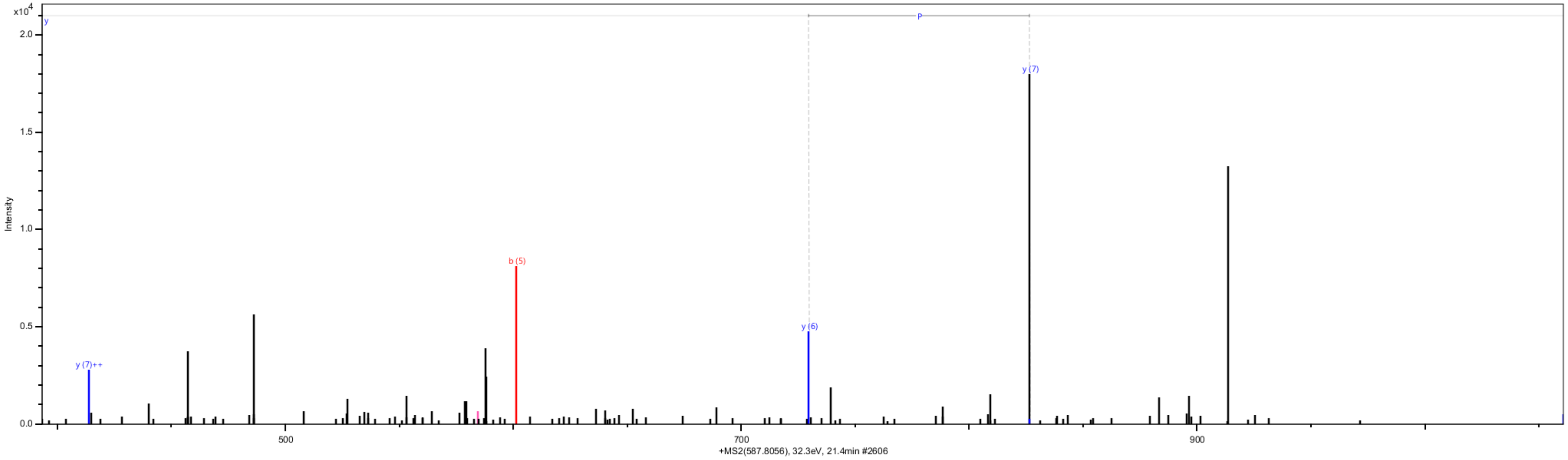

KVD28_HUMAN

Accession: KVD28_HUMAN
Database: SwissProt
MW [kDa] / pI: 12.9 / 5.6
Score: 64.4
Seq. Coverage [%]: 10.8
No. of Peptides: 1

Peptide Match

Sequence: R.FSGSGSGTDFTLK.I
Score: 64.4
Modifications:

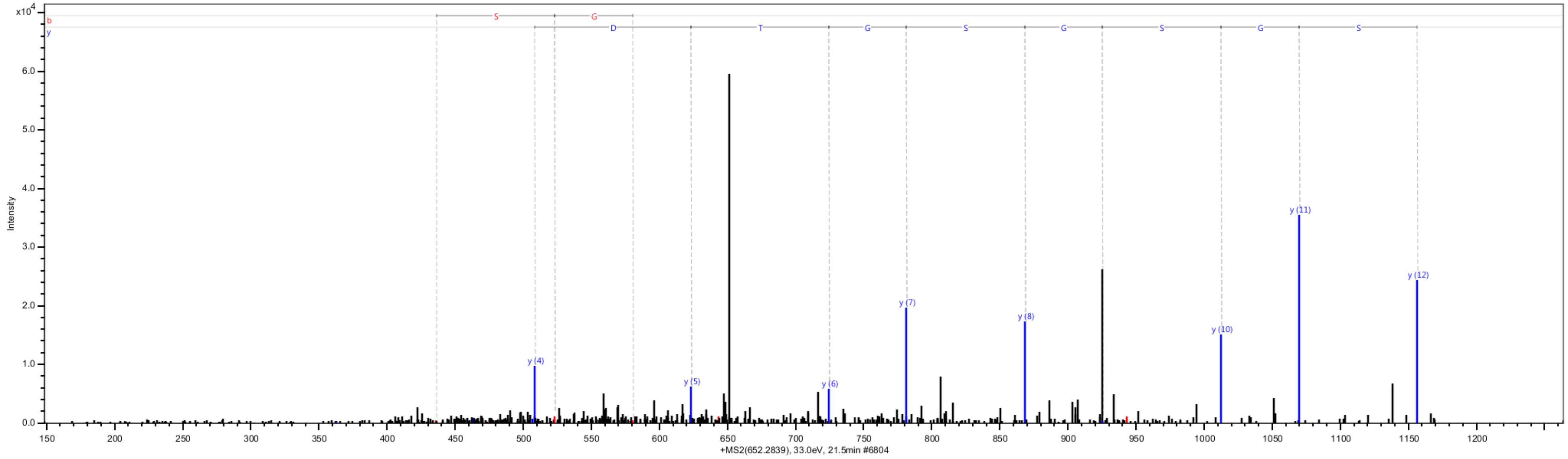

VIME_HUMAN

Accession: VIME_HUMAN
Database: SwissProt
MW [kDa] / pI: 53.6 / 5.1
Score: 64.5
Seq. Coverage [%]: 5.2
No. of Peptides: 2

Peptide Match

Sequence: K.ILLAELEQLKGQGK.S **Score:** 37.8
Modifications:

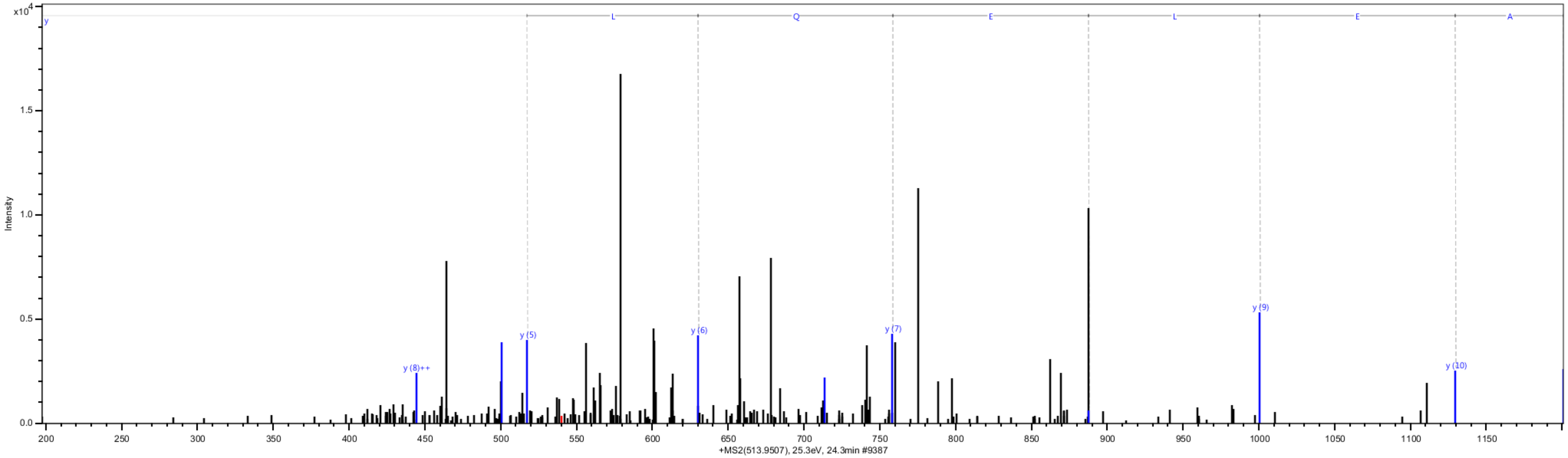

Peptide Match

Sequence: K.NLQEAEEWYK.S **Score:** 26.7
Modifications:

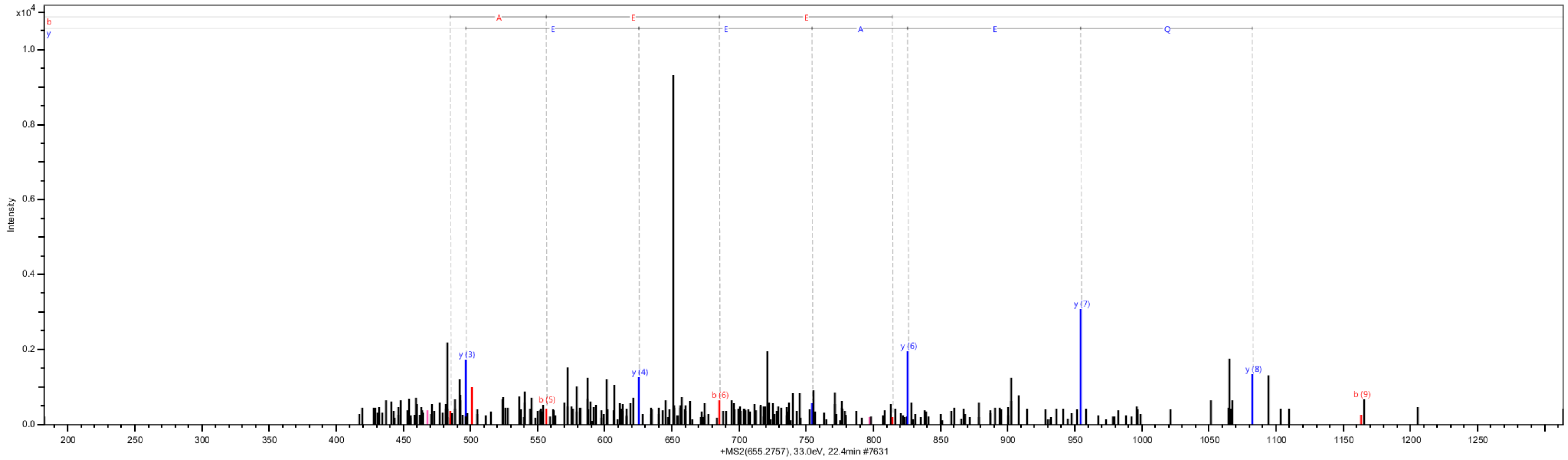
x10^4
b
y
A
E
E
E
E
A
E
Q
1.0
0.8
0.6
0.4
0.2
0.0
Intensity
y (3)
y (4)
b (5)
b (6)
y (6)
y (7)
y (8)
b (9)
200
250
300
350
400
450
500
550
600
650
700
750
800
850
900
950
1000
1050
1100
1150
1200
1250
+MS2(655.2757), 33.0eV, 22.4min #7631

WDR87_HUMAN

**Accession:**	WDR87_HUMAN	**Score:**	67.1
**Database:**	SwissProt	**Seq. Coverage [%]:**	0.9
**MW [kDa] / pI:**	333.0 / 6.9	**No. of Peptides:**	3

Peptide Match

Sequence: R.LNENLTLSHR.D **Score:** 15.9
Modifications:

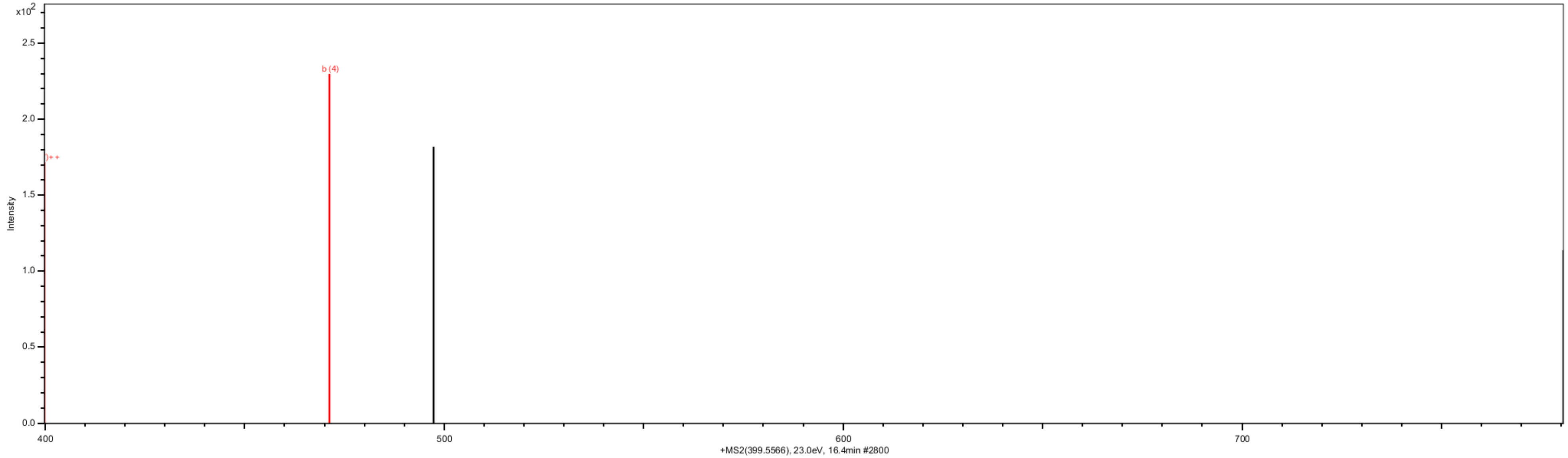

Peptide Match

Sequence: K.QFKLQEQR.R **Score:** 18
Modifications:

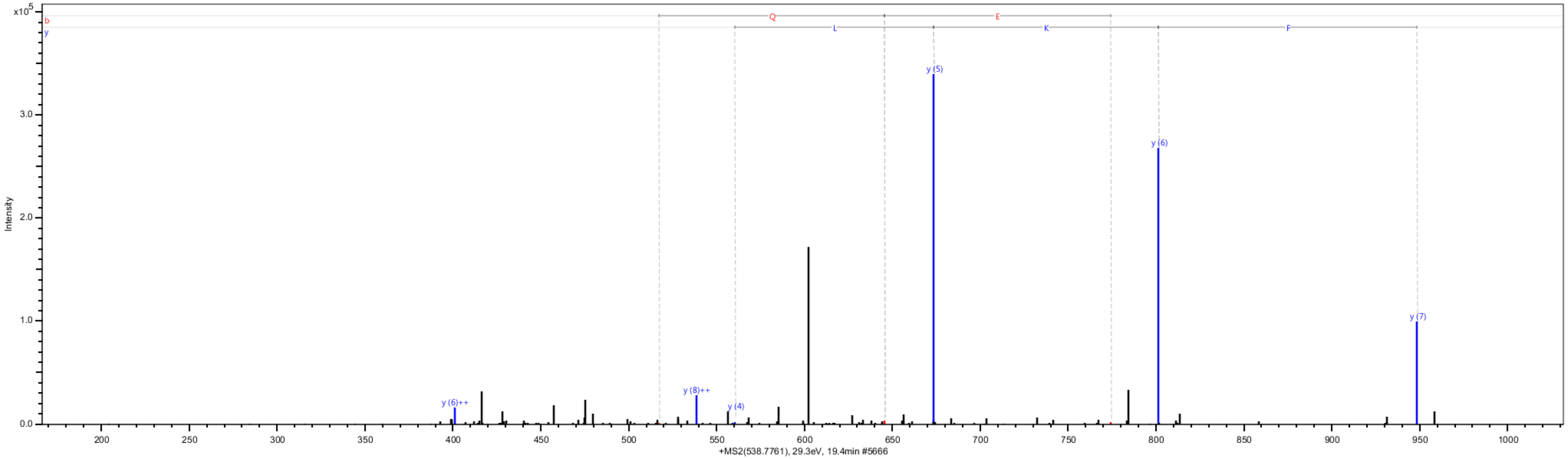

Peptide Match

Sequence: K.KATVVEIPR.K **Score:** 33.2
Modifications:

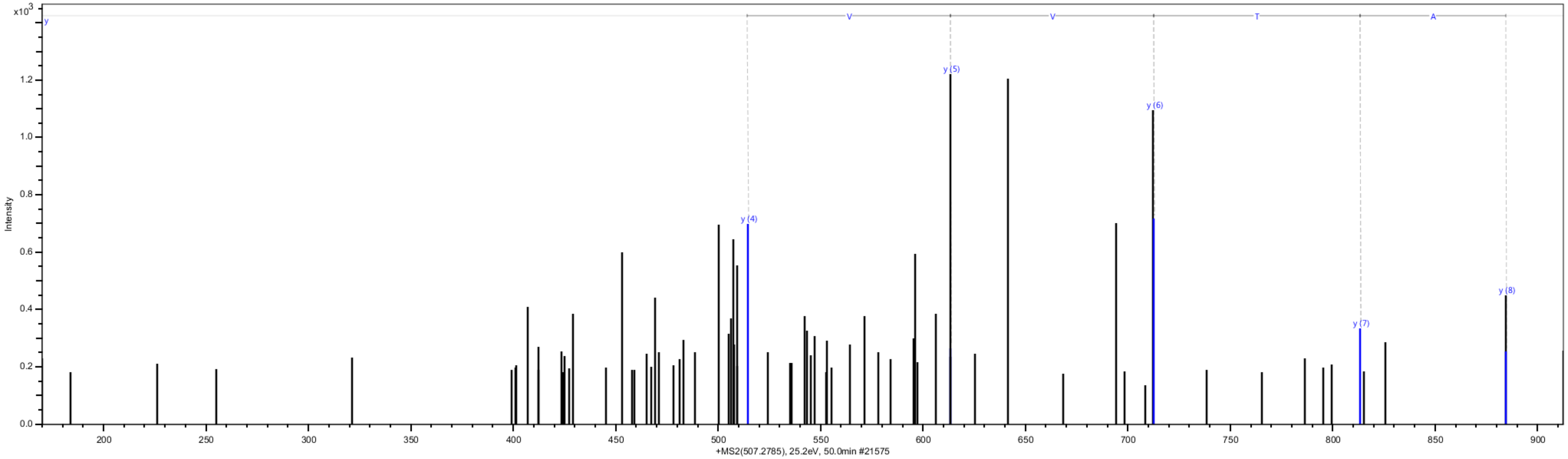
x10^3
y
V
V
T
A
y (4)
y (5)
y (6)
y (7)
y (8)
Intensity
1.2
1.0
0.8
0.6
0.4
0.2
0.0
200
250
300
350
400
450
500
550
600
650
700
750
800
850
900
+MS2(507.2785), 25.2eV, 50.0min #21575

PRG4_HUMAN

Accession: PRG4_HUMAN
Database: SwissProt
MW [kDa] / pI: 151.0 / 9.5
Score: 64.5
Seq. Coverage [%]: 2.4
No. of Peptides: 2

Peptide Match

Sequence: K.GFGGLTGQIVAALSTAK.Y
Score: 37.6
Modifications:

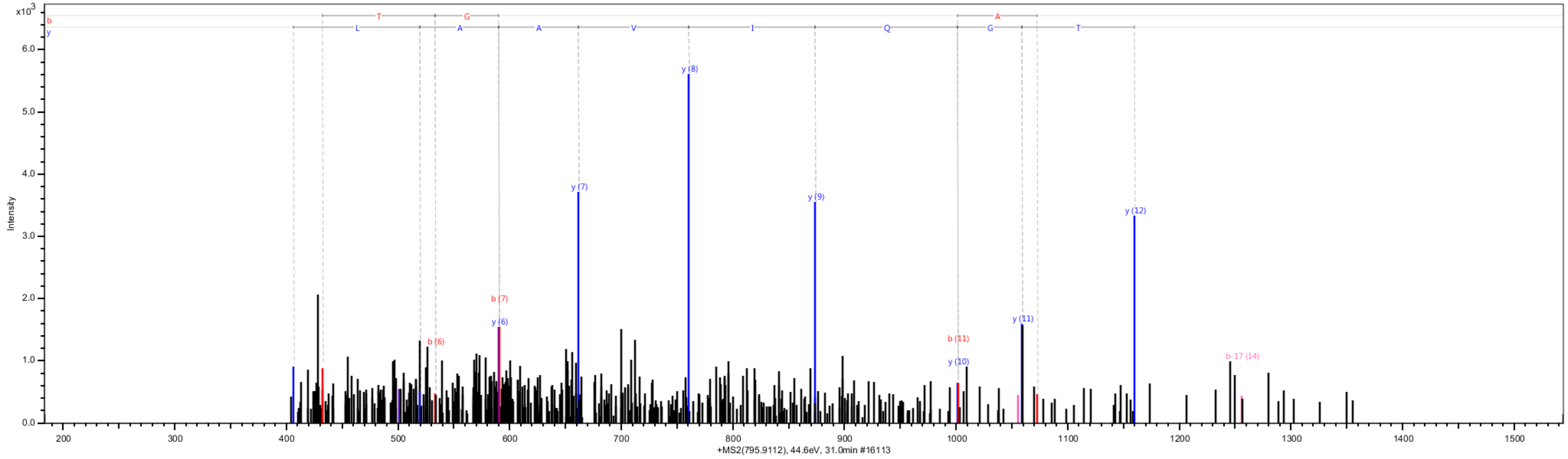

Peptide Match

Sequence: R.GLPNVVTSAISLPNIR.K
Score: 26.8
Modifications:

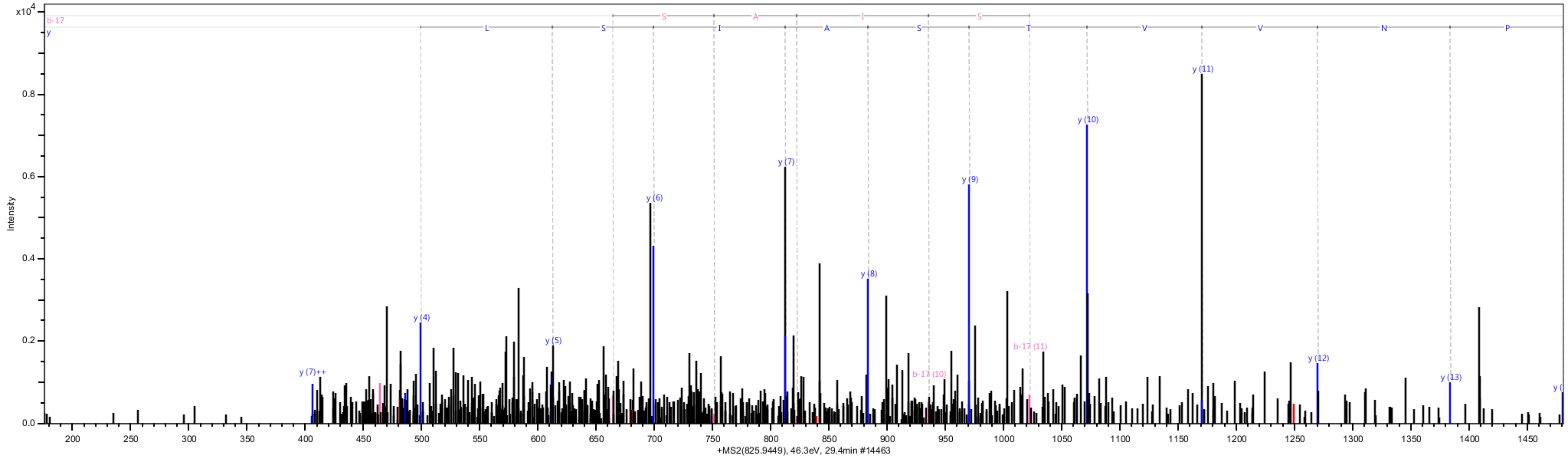
x10^4
Intensity
b-17
y
S A I S
L S I A S T V V N P
y (11)
y (10)
y (7)
y (9)
y (6)
y (8)
y (4)
y (5)
b-17 (11)
y (12)
b-17 (10)
y (7)++
y (13)
0.0
0.2
0.4
0.6
0.8
200 250 300 350 400 450 500 550 600 650 700 750 800 850 900 950 1000 1050 1100 1150 1200 1250 1300 1350 1400 1450
+MS2(825.9449), 46.3eV, 29.4min #14463

TITIN_HUMAN

Accession: TITIN_HUMAN
Database: SwissProt
MW [kDa] / pI: 3813.7 / 6.0
Score: 71.3
Seq. Coverage [%]: 0.1
No. of Peptides: 3

Peptide Match

Sequence: R.ISEEITKK.Q
Score: 15.3
Modifications:

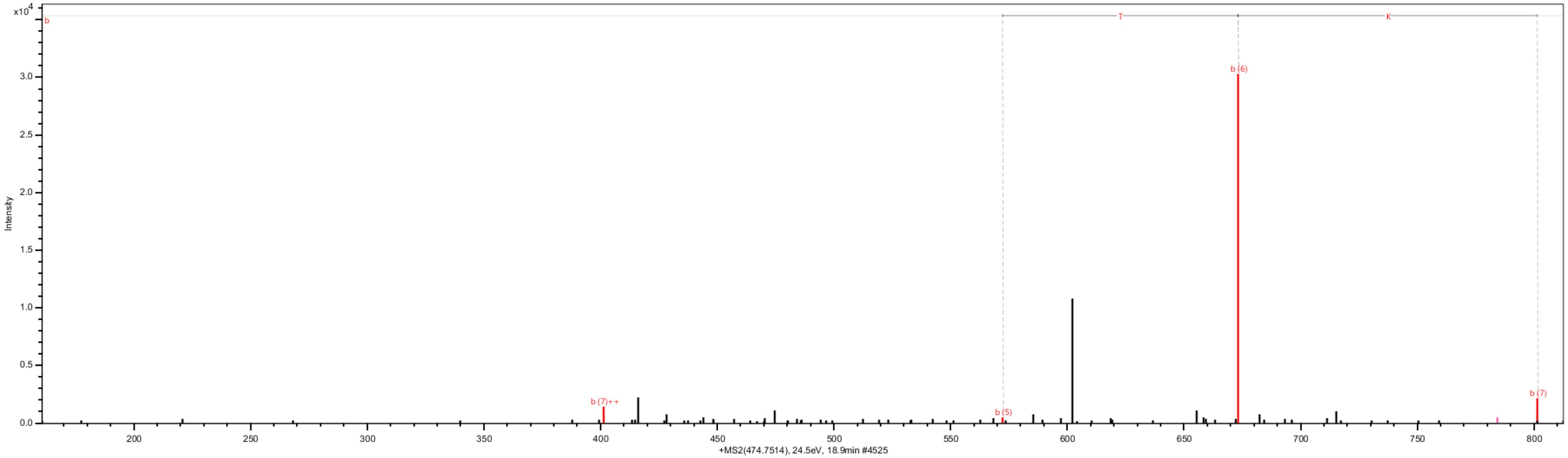

Peptide Match

Sequence: R.TTIKITK.L
Score: 15.2
Modifications:

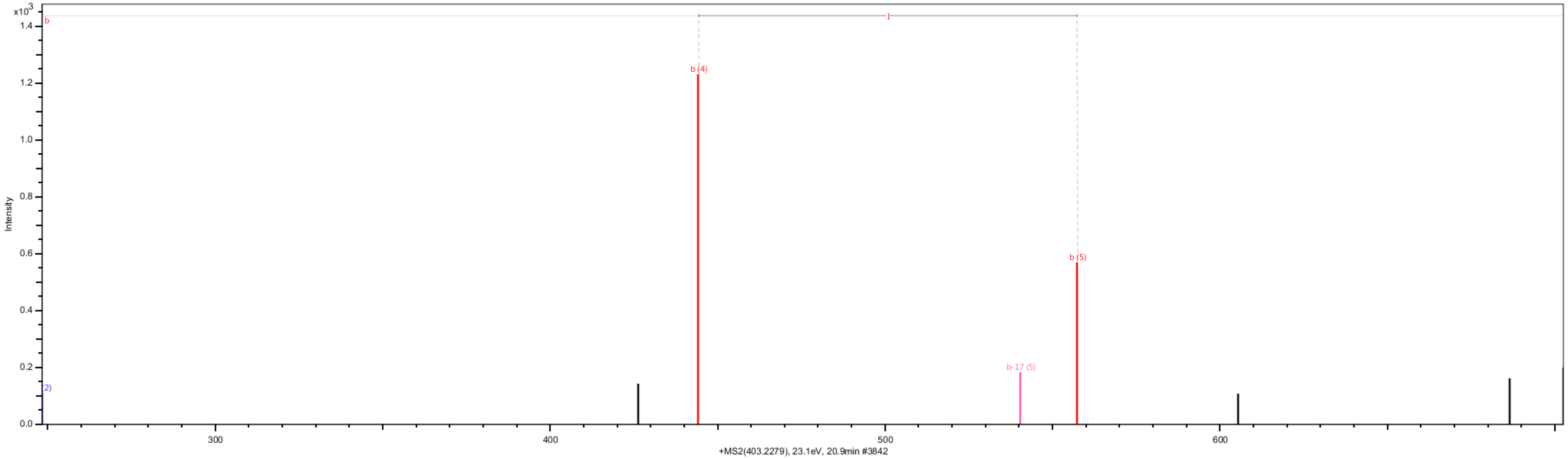

Peptide Match

Sequence: R.SLSRFER.S **Score:** 16.1

Modifications:

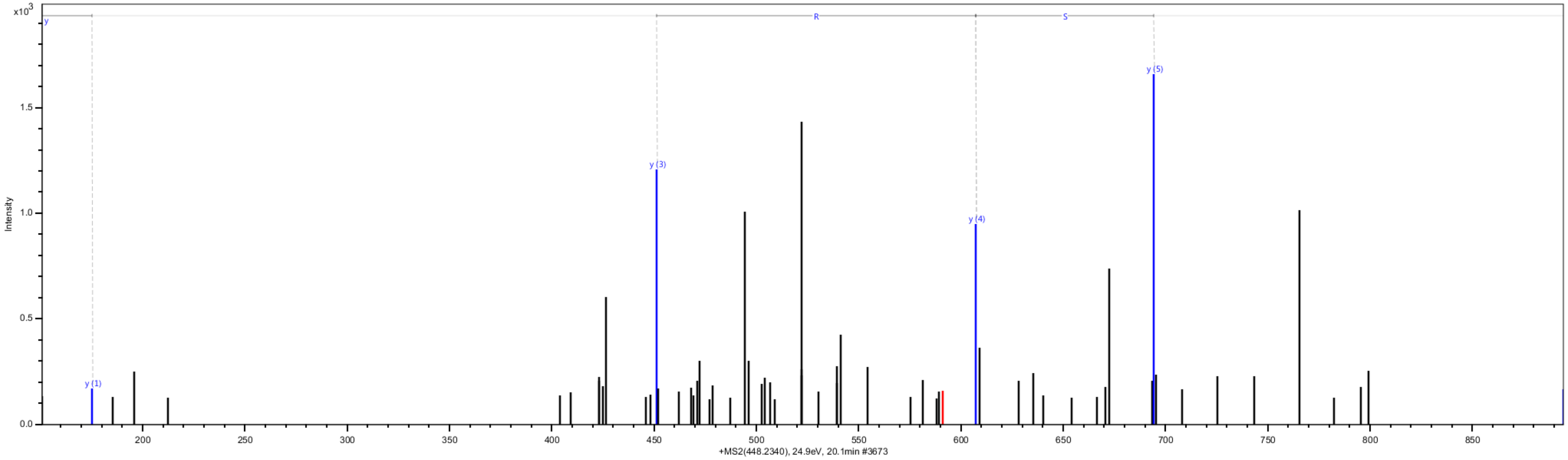
x10^3
Intensity
y
R
S
y (1)
y (3)
y (4)
y (5)
0.0
0.5
1.0
1.5
200
250
300
350
400
450
500
550
600
650
700
750
800
850
+MS2(448.2340), 24.9eV, 20.1min #3673

KV320_HUMAN

**Accession:**	KV320_HUMAN	**Score:**	71.8
**Database:**	SwissProt	**Seq. Coverage [%]:**	7.8
**MW [kDa] / pI:**	12.5 / 4.9	**No. of Peptides:**	1

Peptide Match

**Sequence:**	R.LLIYGASSR.A	**Score:**	34
**Modifications:**			

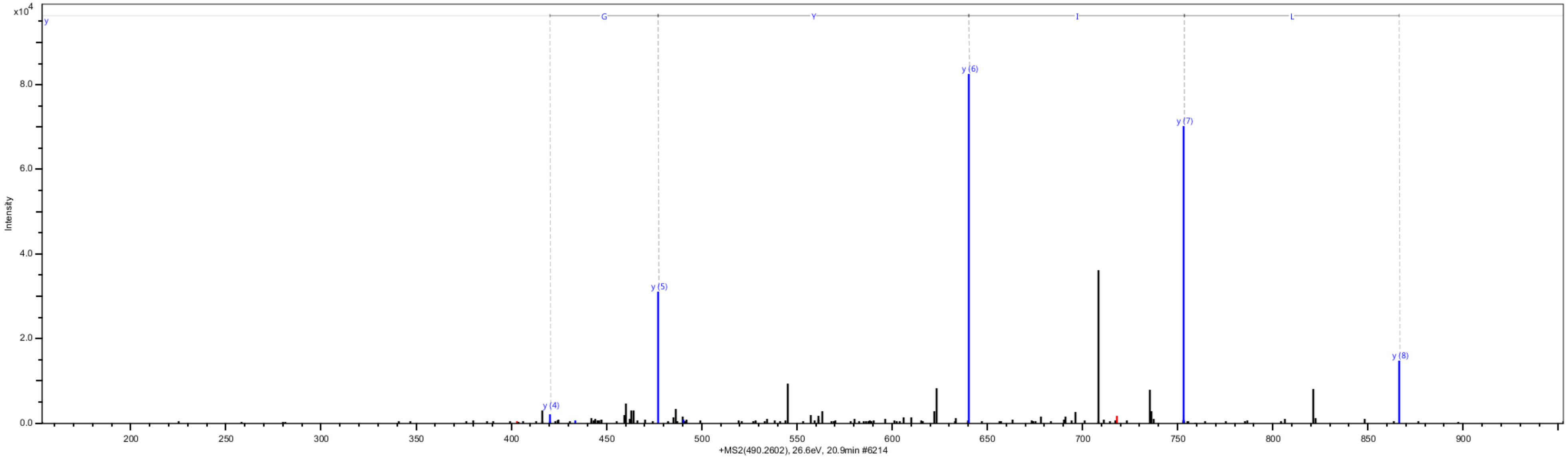

KDM5C_HUMAN

Accession: KDM5C_HUMAN
Database: SwissProt
MW [kDa] / pI: 175.6 / 5.4
Score: 68.0
Seq. Coverage [%]: 1.7
No. of Peptides: 3

Peptide Match

Sequence: R.ILDLYSLSK.I
Score: 20.3
Modifications:

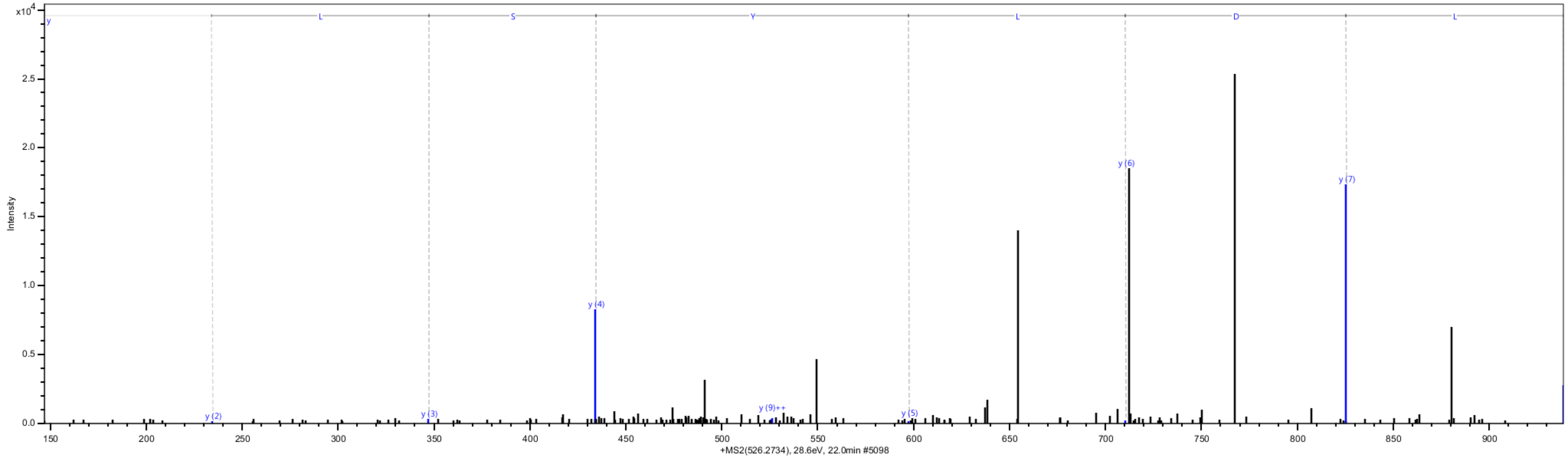

Peptide Match

Sequence: K.MMGLGLMAKDK.T
Score: 15.4
Modifications: Oxidation: 1, 2

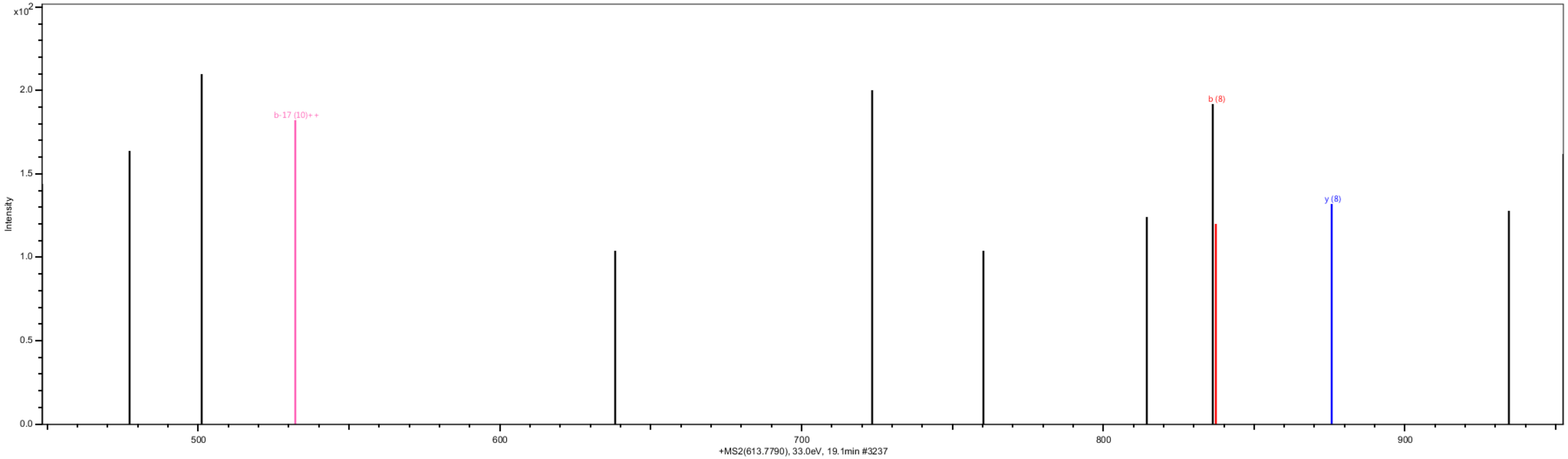

Peptide Match

**Sequence:**	R.LAELRQR.L	**Score:**	15.2
**Modifications:**			

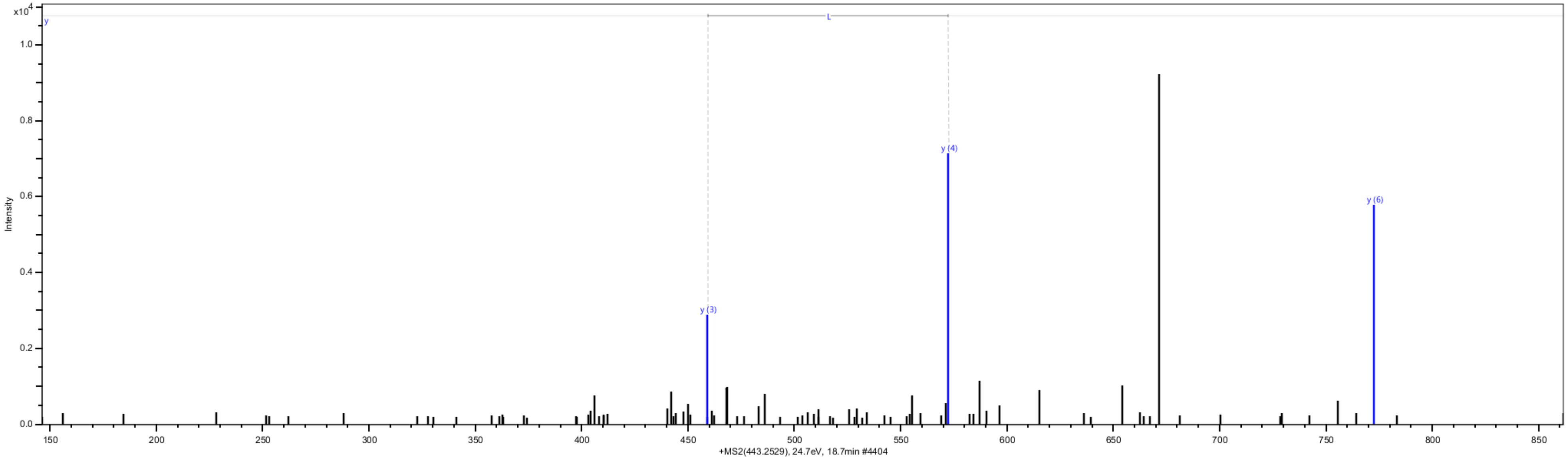
x10^4
y
L
1.0
0.8
0.6
0.4
0.2
0.0
Intensity
y (3)
y (4)
y (6)
150
200
250
300
350
400
450
500
550
600
650
700
750
800
850
+MS2(443.2529), 24.7eV, 18.7min #4404

TTHY_HUMAN

Accession: TTHY_HUMAN
Database: SwissProt
MW [kDa] / pI: 15.9 / 5.5
Score: 73.2
Seq. Coverage [%]: 18.4
No. of Peptides: 2

Peptide Match

Sequence: R.GSPAINVAVHVFRK.A **Score:** 50.5
Modifications:

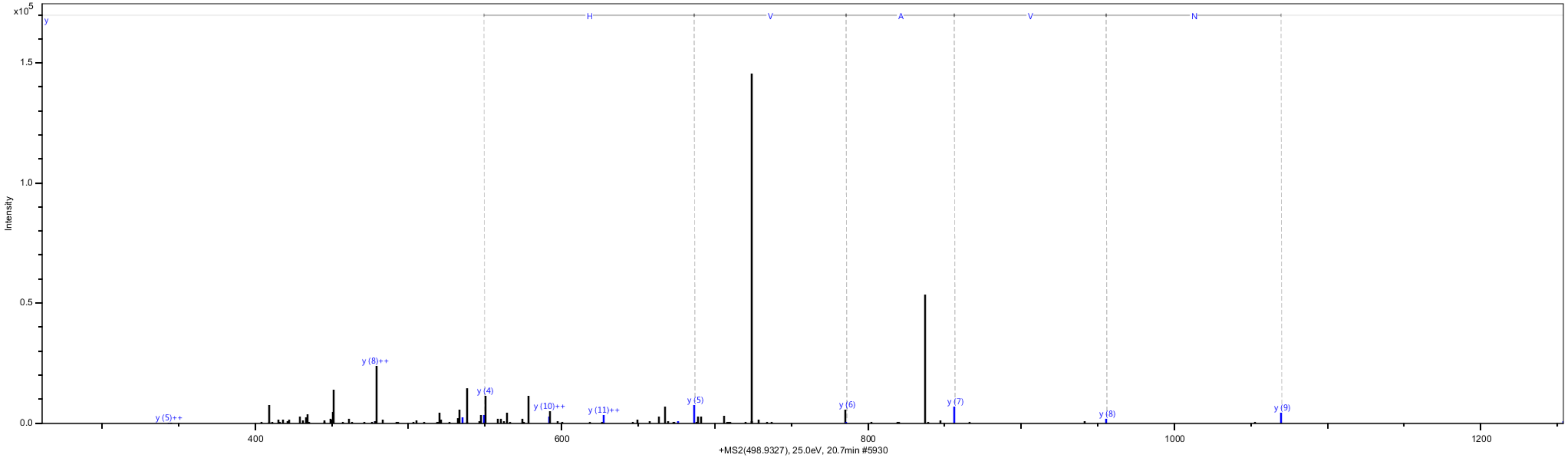

Peptide Match

Sequence: K.AADDTWEPFASGK.T **Score:** 22.6
Modifications:

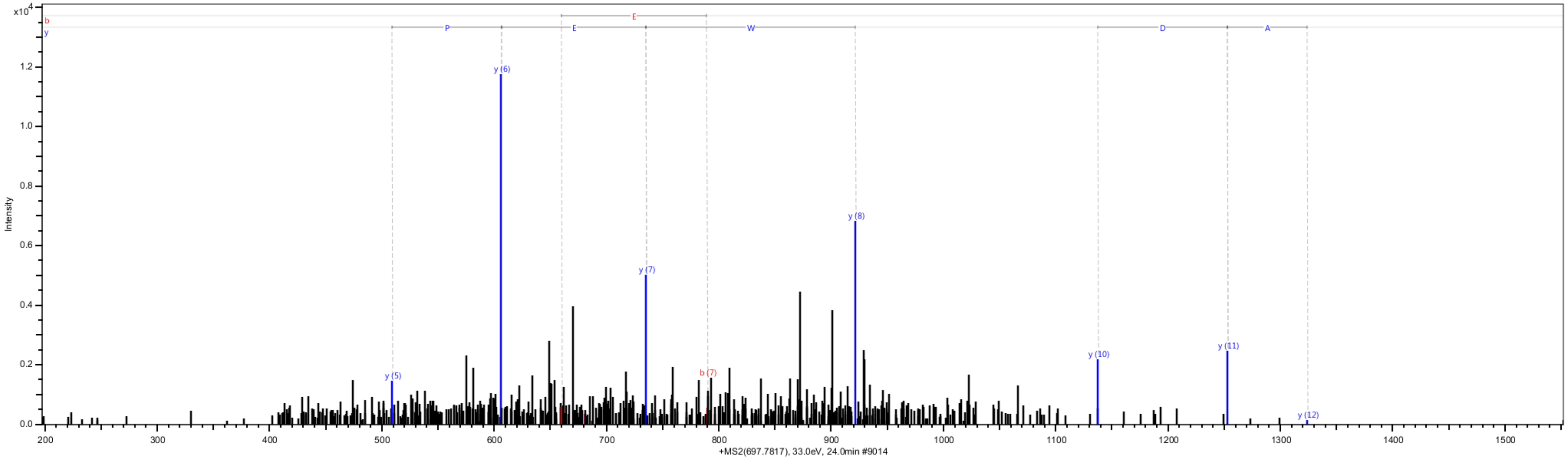
x10^4
b
y
P
E
E
W
D
A
y (6)
y (8)
y (7)
y (5)
b (7)
y (10)
y (11)
y (12)
Intensity
1.2
1.0
0.8
0.6
0.4
0.2
0.0
200
300
400
500
600
700
800
900
1000
1100
1200
1300
1400
1500
+MS2(697.7817), 33.0eV, 24.0min #9014

KVD20_HUMAN

**Accession:**	KVD20_HUMAN	**Score:**	74.0
**Database:**	SwissProt	**Seq. Coverage [%]:**	27.6
**MW [kDa] / pI:**	12.5 / 4.5	**No. of Peptides:**	2

Peptide Match

**Sequence:**	R.LLIYDASSR.A	**Score:**	36.3
**Modifications:**			

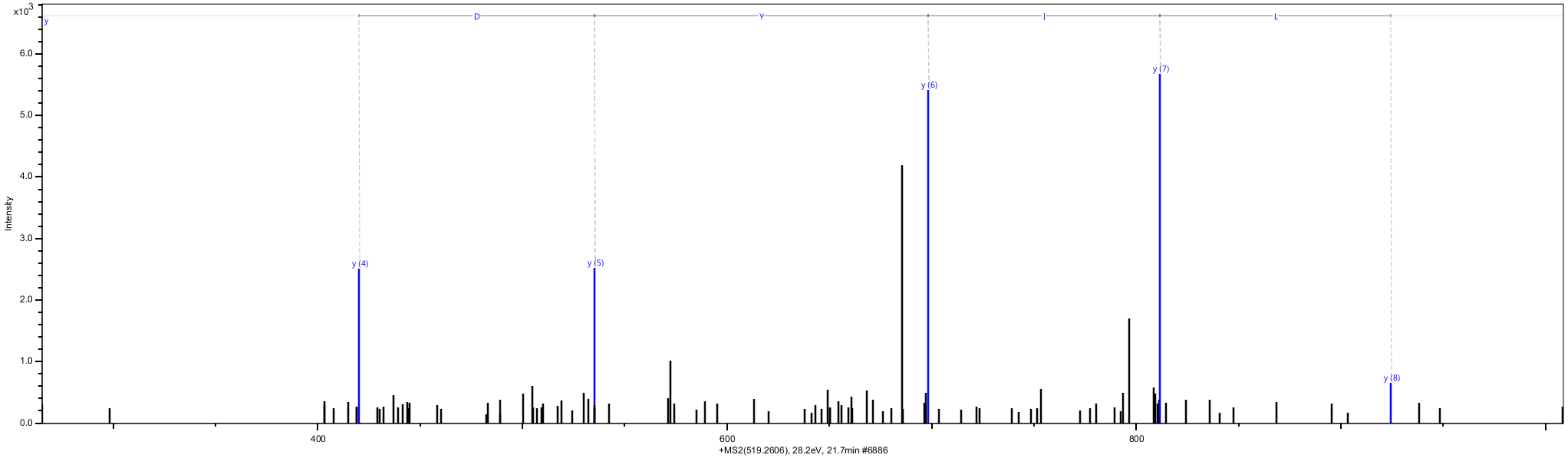

Peptide Match

**Sequence:**	R.ATGIPDRFSGSGSGTDFTLTISR.L	**Score:**	37.7
**Modifications:**			

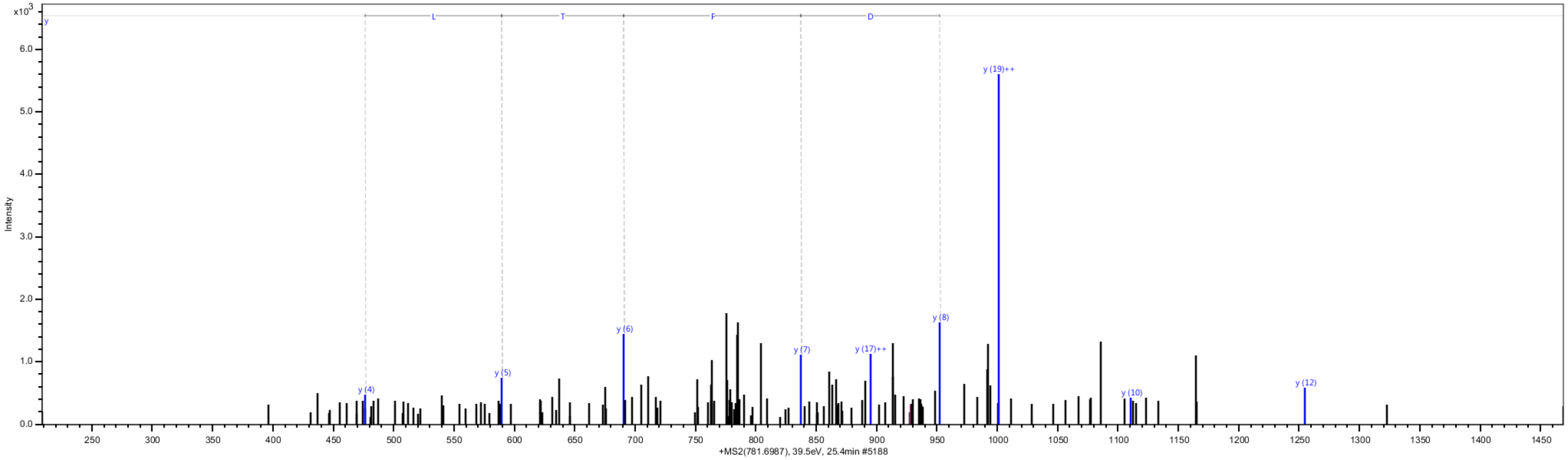
x10^3
Intensity
y
L
T
F
D
y (19)++
y (6)
y (7)
y (17)++
y (8)
y (5)
y (4)
y (10)
y (12)
+MS2(781.6987), 39.5eV, 25.4min #5188

MACF1_HUMAN

Accession: MACF1_HUMAN
Database: SwissProt
MW [kDa] / pI: 837.8 / 5.3
Score: 71.8
Seq. Coverage [%]: 0.5
No. of Peptides: 3

Peptide Match

Sequence: K.LVNIRNDDITDGNPK.L
Score: 16
Modifications:

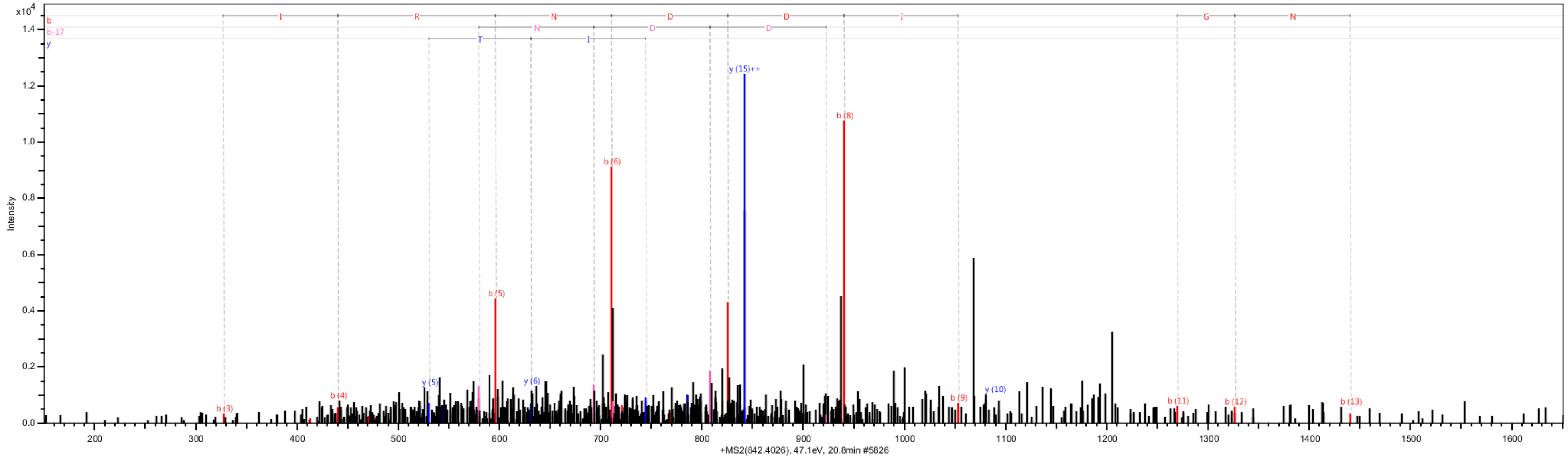

Peptide Match

Sequence: K.TFPQNPVELK.A
Score: 16.1
Modifications:

Spectrum Report
Combine Search_2020-10-19 07:53:31

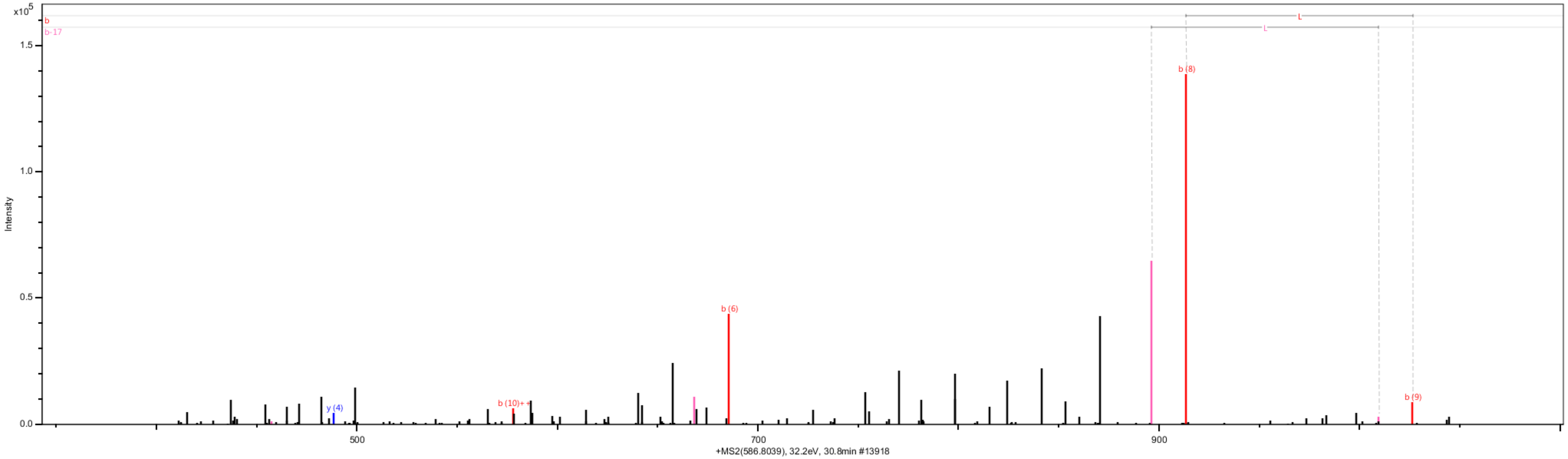

Peptide Match

Sequence: K.AKEETYNQLLDK.G **Score:** 15.2

Modifications:

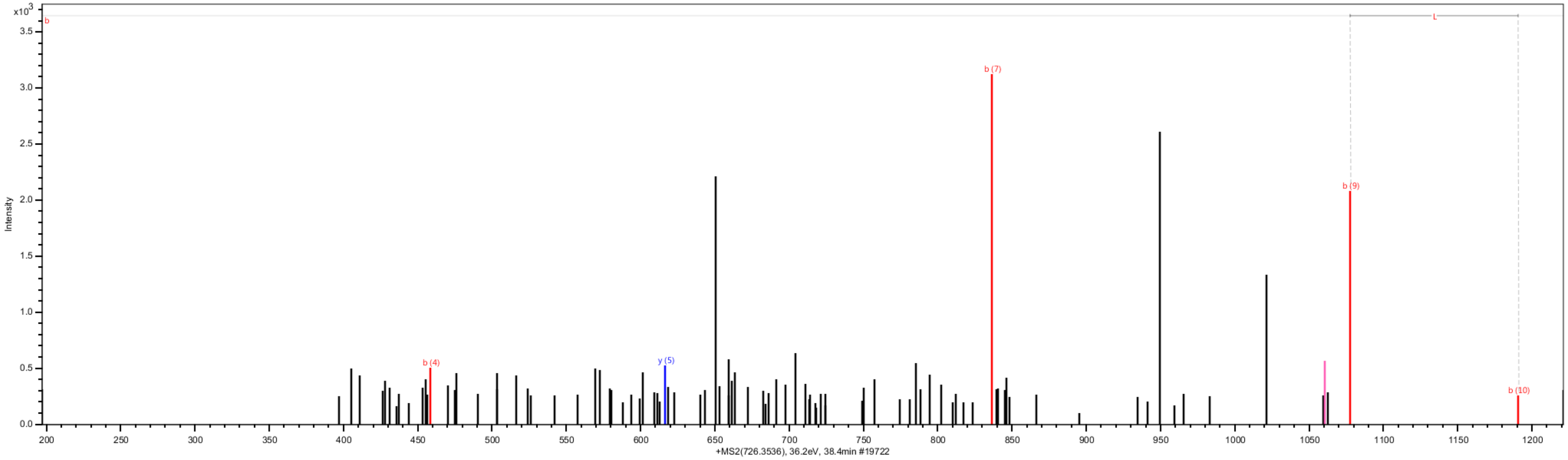
x10^3
Intensity
b (7)
b (9)
b (4)
y (5)
b (10)
L
+MS2(726.3536), 36.2eV, 38.4min #19722

P3C2B_HUMAN

Accession: P3C2B_HUMAN
Score: 53.0
Database: SwissProt
Seq. Coverage [%]: 0.9
MW [kDa] / pI: 184.7 / 6.9
No. of Peptides: 2

Peptide Match

Sequence: K.LKDIMQK.E
Score: 23.6
Modifications:

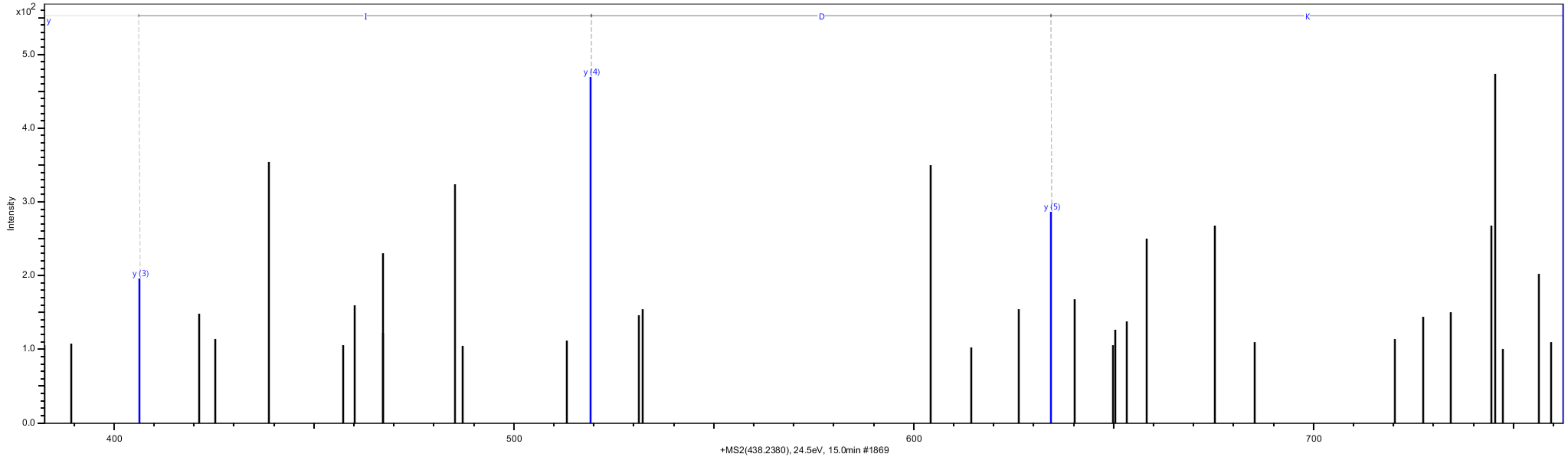

Peptide Match

Sequence: R.ISDVFLCR.H
Score: 29.4
Modifications: Carbamidomethyl: 7

Spectrum Report
Combine Search_2020-10-19 07:53:31

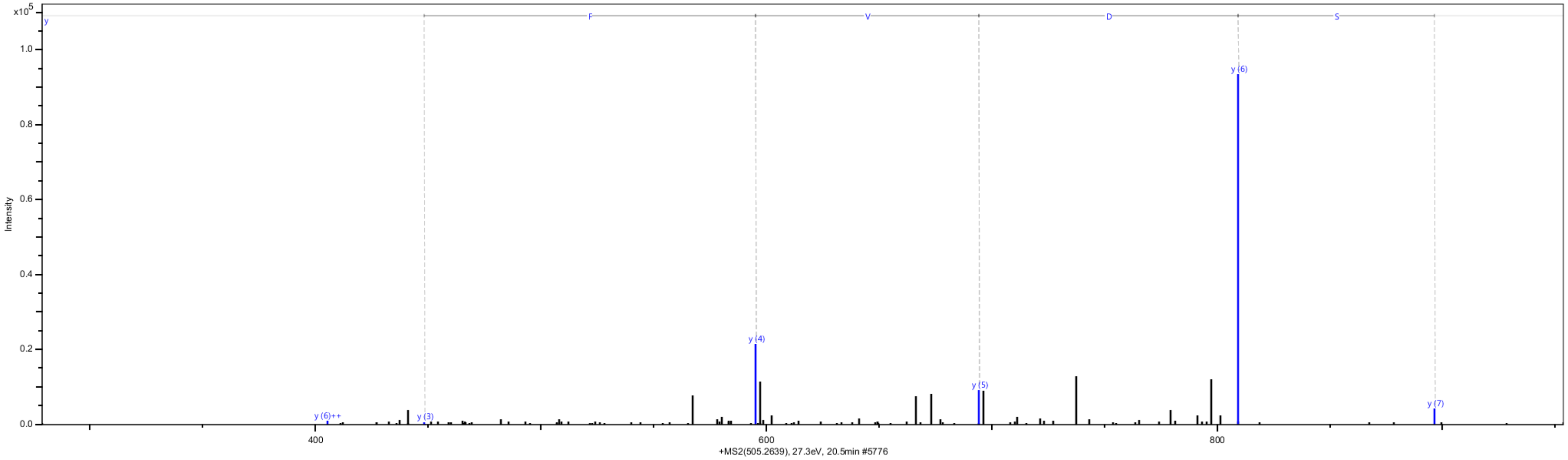

IDH3A_HUMAN

Accession: IDH3A_HUMAN
Database: SwissProt
MW [kDa] / pI: 39.6 / 6.5
Score: 51.5
Seq. Coverage [%]: 2.7
No. of Peptides: 1

Peptide Match

Sequence: R.IAEFAFEYAR.N
Score: 51.5
Modifications:

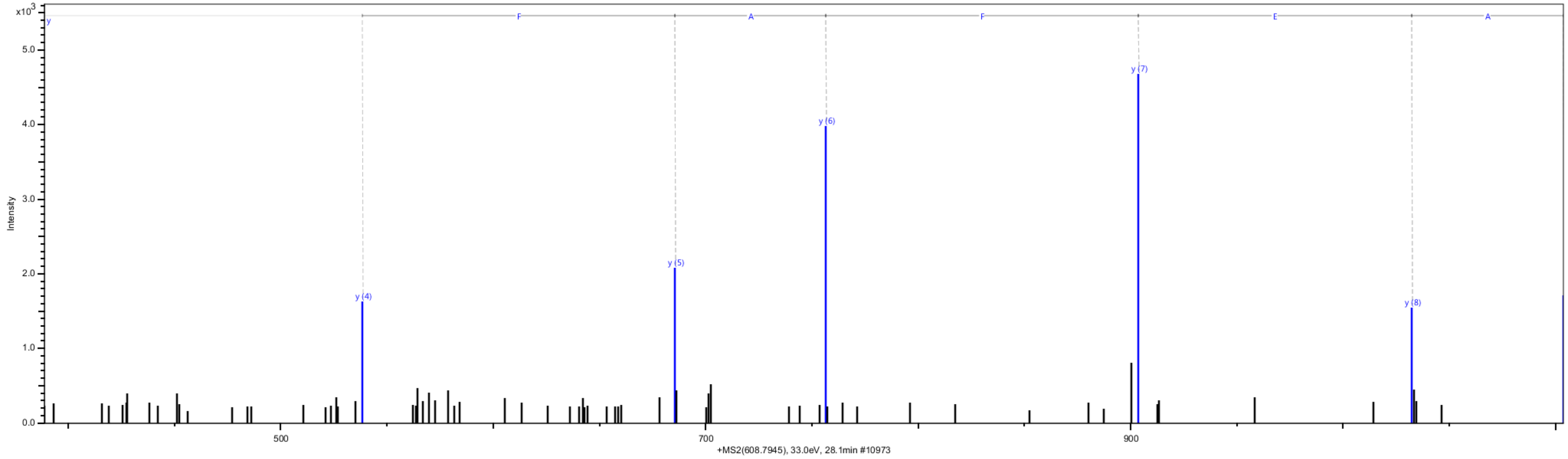

EVPL_HUMAN

Accession: EVPL_HUMAN
Database: SwissProt
MW [kDa] / pI: 231.5 / 6.6
Score: 51.1
Seq. Coverage [%]: 0.8
No. of Peptides: 2

Peptide Match

Sequence: R.LQSLGTEK.E
Score: 20.3
Modifications:

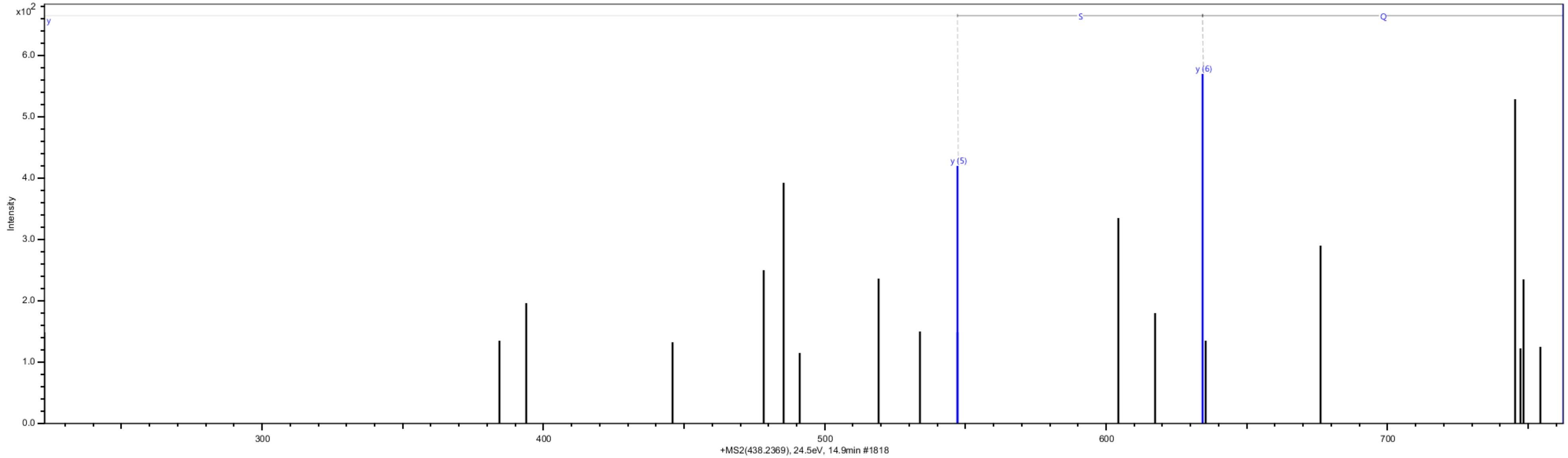

Peptide Match

Sequence: R.GEDAVISAR.L
Score: 15.4
Modifications:

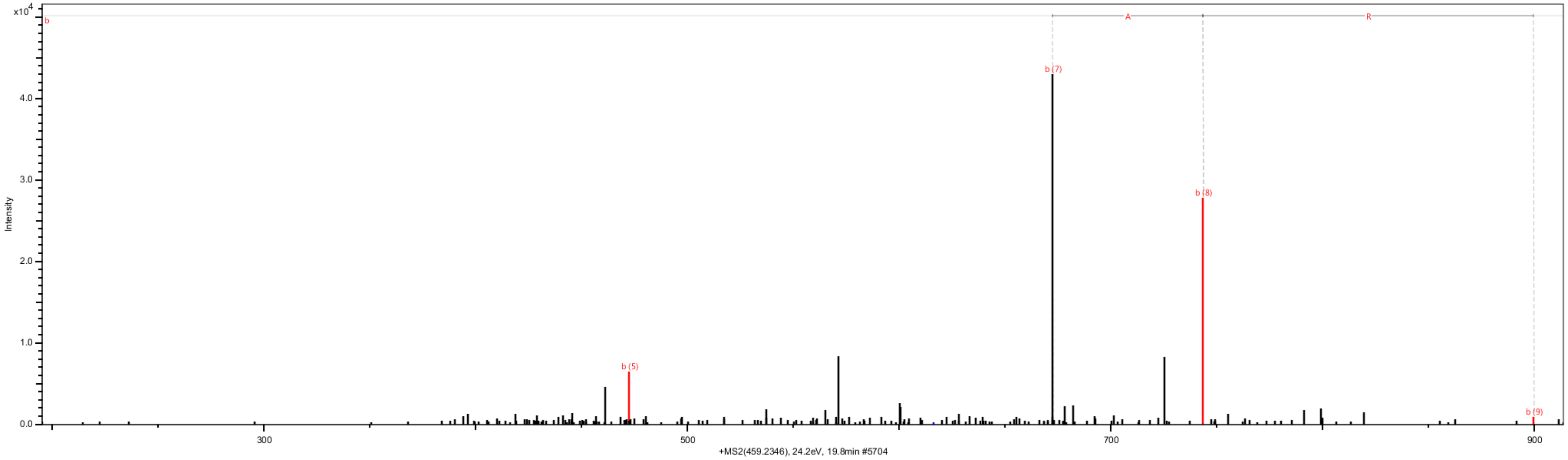
x10^4
Intensity
4.0
3.0
2.0
1.0
0.0
b
A
R
b (7)
b (8)
b (5)
b (9)
300
500
700
900
+MS2(459.2346), 24.2eV, 19.8min #5704

RHG44_HUMAN

Accession: RHG44_HUMAN
Database: SwissProt
MW [kDa] / pI: 89.2 / 6.1
Score: 51.1
Seq. Coverage [%]: 1.1
No. of Peptides: 2

Peptide Match

Sequence: K.LVLDMDSSR.T
Modifications: Oxidation: 5
Score: 21.6

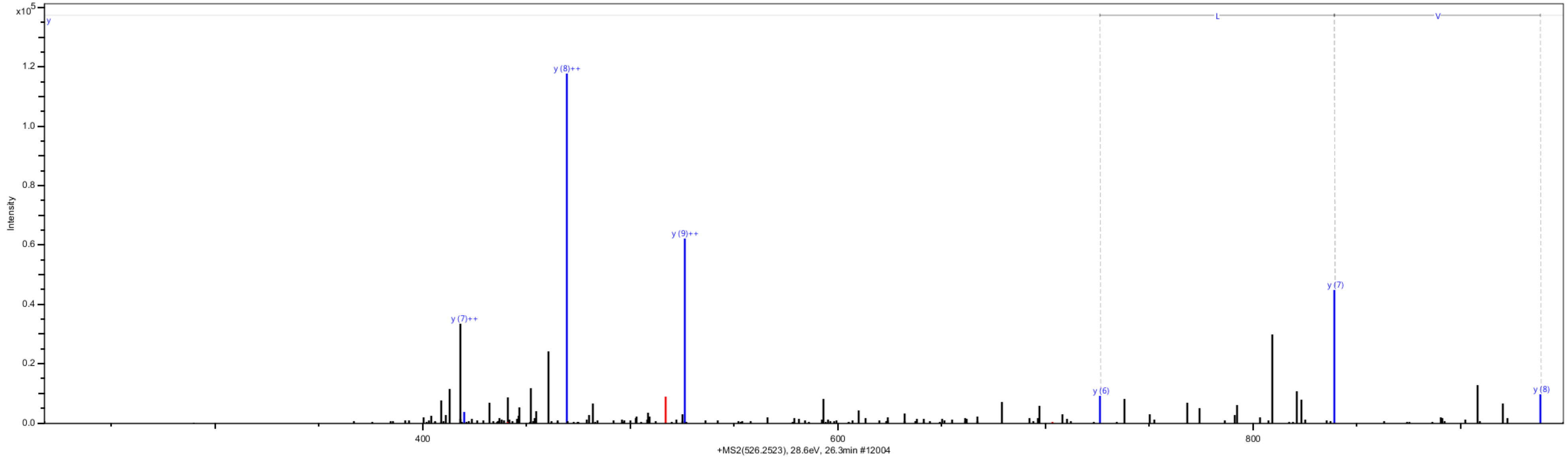

Peptide Match

Sequence: K.LVLDMDSSR.T
Modifications:
Score: 29.5

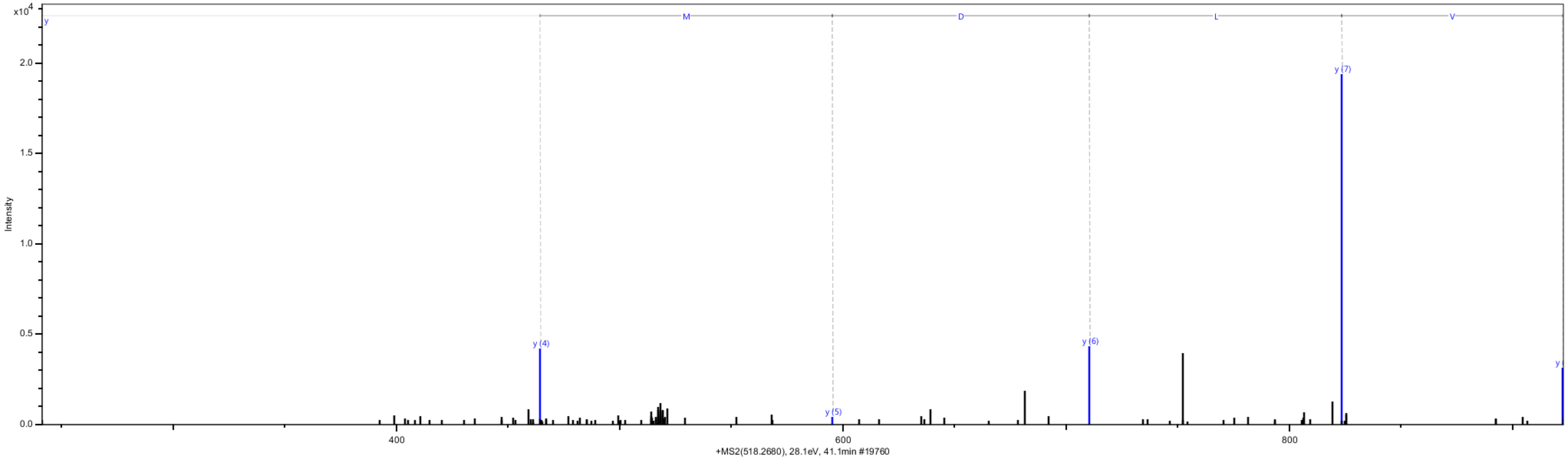
x10^4
2.0
1.5
1.0
0.5
0.0
Intensity
M
D
L
V
y (4)
y (5)
y (6)
y (7)
400
600
800
+MS2(518.2680), 28.1eV, 41.1min #19760

NEBU_HUMAN

Accession: NEBU_HUMAN
Database: SwissProt
MW [kDa] / pI: 772.4 / 9.1
Score: 56.3
Seq. Coverage [%]: 0.4
No. of Peptides: 3

Peptide Match

Sequence: K.VTNQVSKQK.Y
Score: 15.8
Modifications:

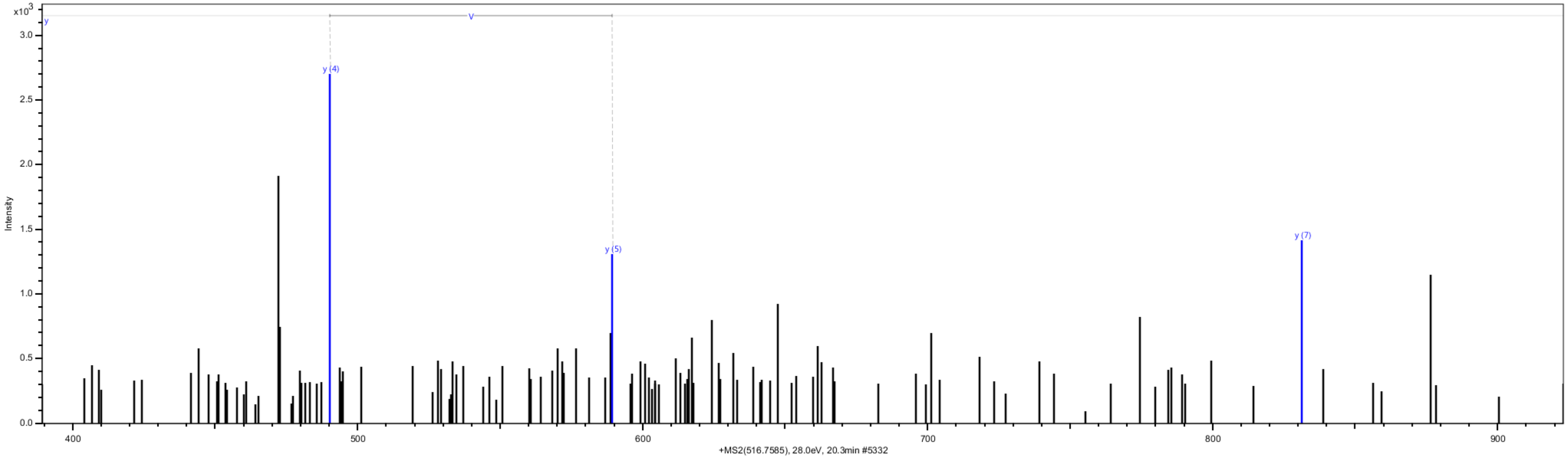

Peptide Match

Sequence: R.ASDILNEKK.Y
Score: 23.5
Modifications:

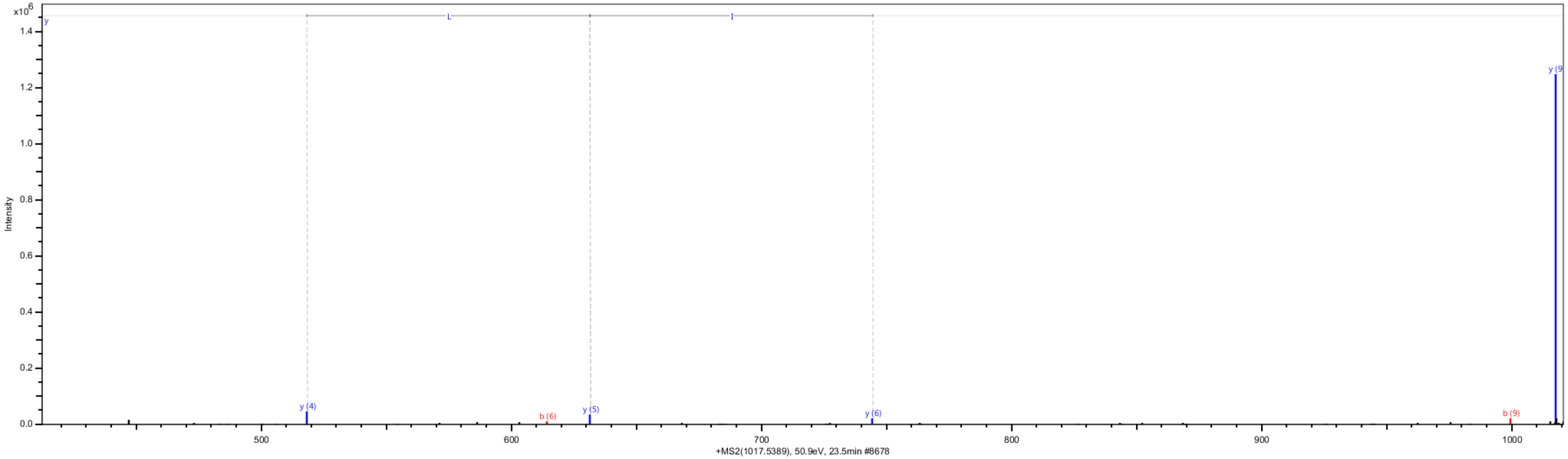

Peptide Match

**Sequence:**	K.AGDALNER.K	**Score:**	17.1
**Modifications:**			

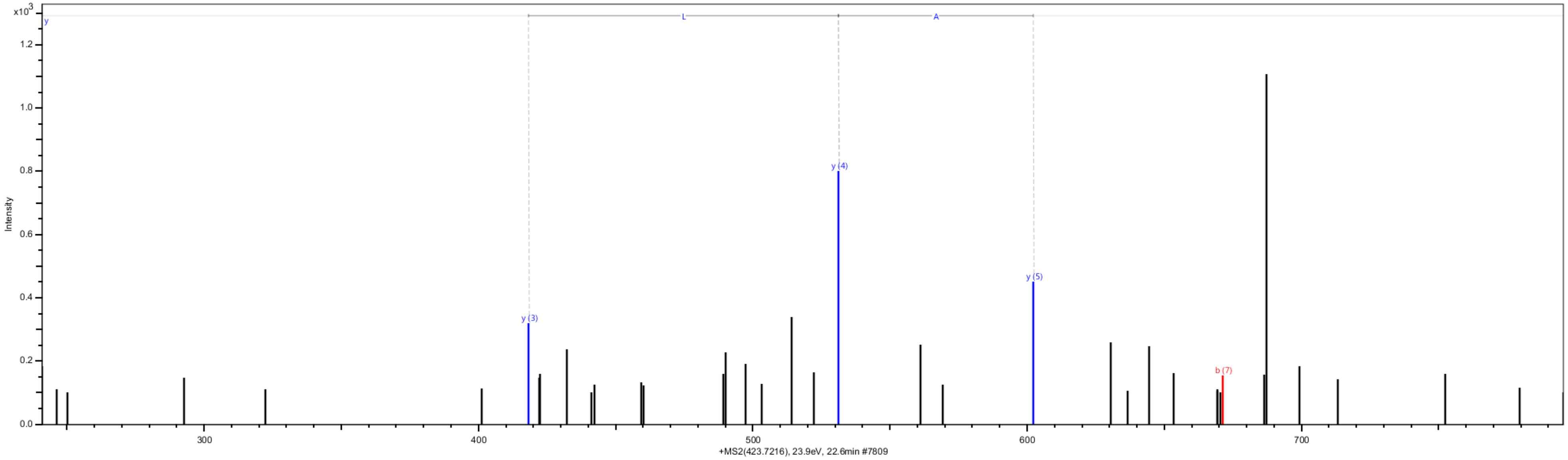
x10^3
y
L
A
1.2
1.0
0.8
0.6
0.4
0.2
0.0
Intensity
y (4)
y (5)
y (3)
b (7)
300
400
500
600
700
+MS2(423.7216), 23.9eV, 22.6min #7809

ANGT_HUMAN

Accession: ANGT_HUMAN
Database: SwissProt
MW [kDa] / pI: 53.1 / 5.9
Score: 56.1
Seq. Coverage [%]: 6.0
No. of Peptides: 2

Peptide Match

Sequence: K.ALQDQLVLVAAK.L
Score: 29.1
Modifications:

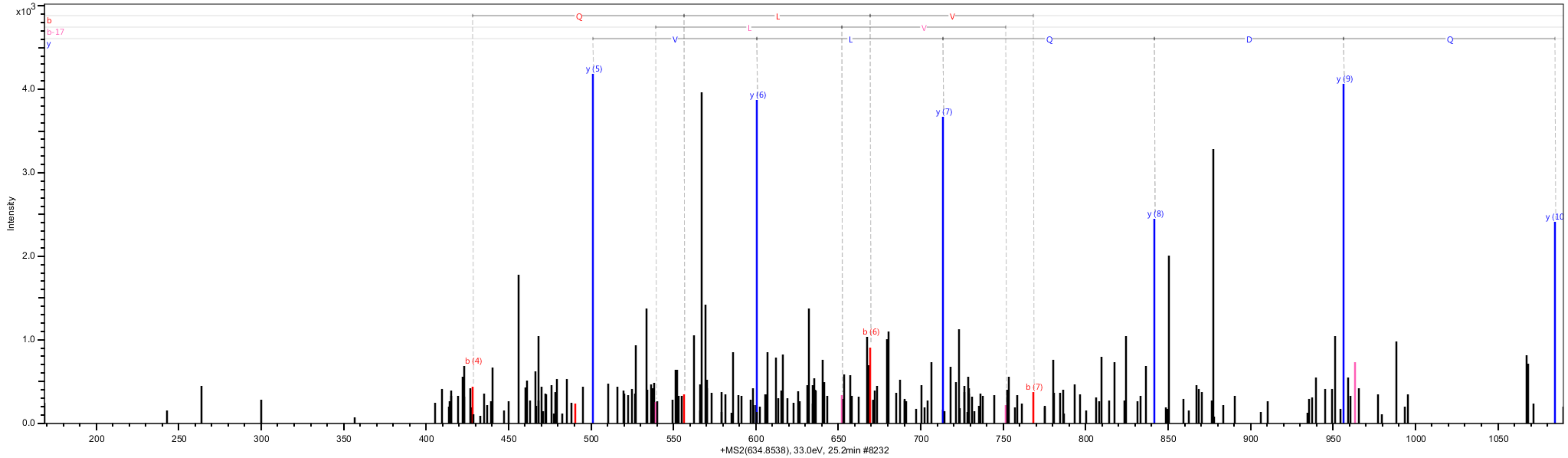

Peptide Match

Sequence: K.VLSALQAVQGLLVAQGR.A
Score: 27
Modifications:

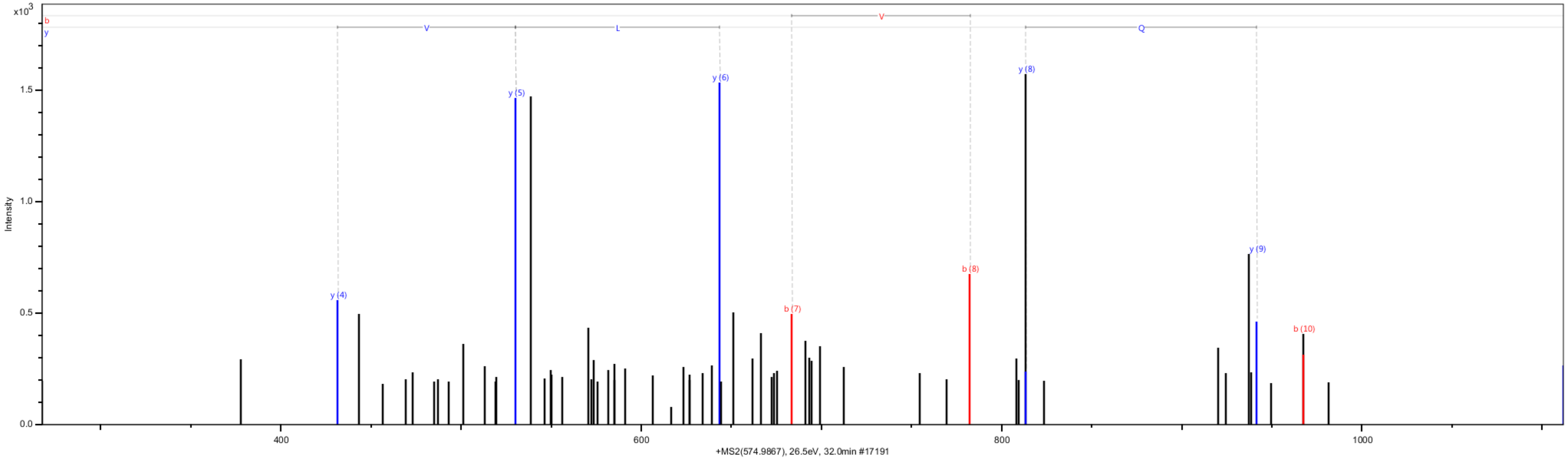
x10^3
b
y
V
L
V
Q
y (4)
y (5)
y (6)
b (7)
b (8)
y (8)
y (9)
b (10)
Intensity
1.5
1.0
0.5
0.0
400
600
800
1000
+MS2(574.9867), 26.5eV, 32.0min #17191

KV311_HUMAN

Accession: KV311_HUMAN
Database: SwissProt
MW [kDa] / pI: 12.6 / 4.9
Score: 53.8
Seq. Coverage [%]: 7.8
No. of Peptides: 1

Peptide Match

Sequence: R.LLIYDASNR.A
Score: 53.8
Modifications:

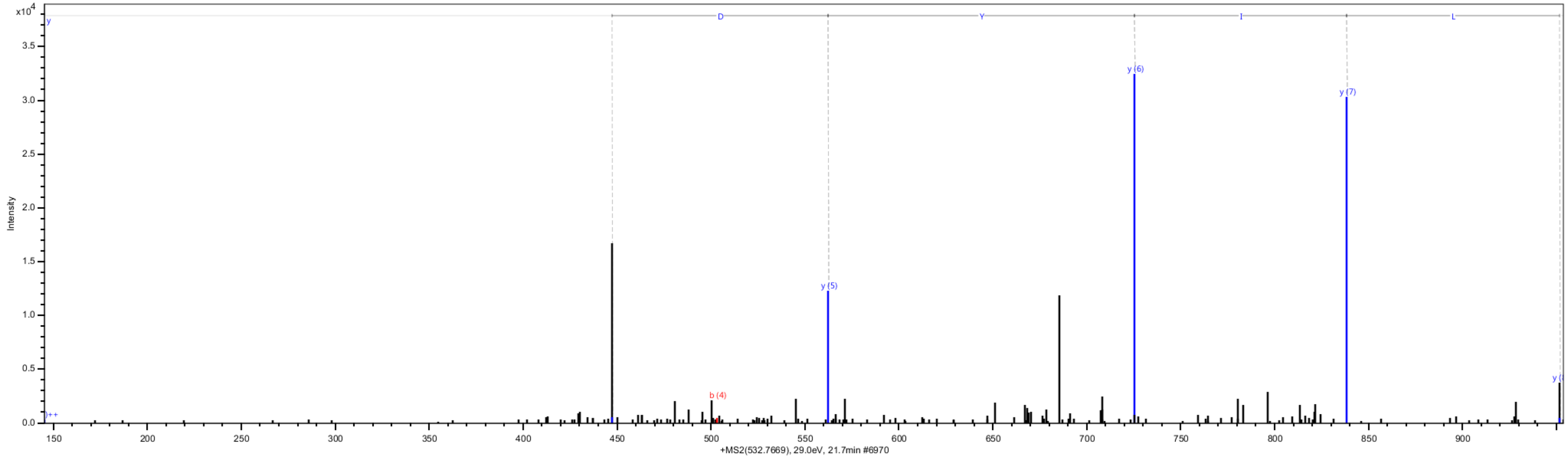

CO5_HUMAN

Accession: CO5_HUMAN
Database: SwissProt
MW [kDa] / pI: 188.2 / 6.1
Score: 59.7
Seq. Coverage [%]: 1.1
No. of Peptides: 2

Peptide Match

Sequence: K.TLLPVSKPEIR.S
Score: 39.1
Modifications:

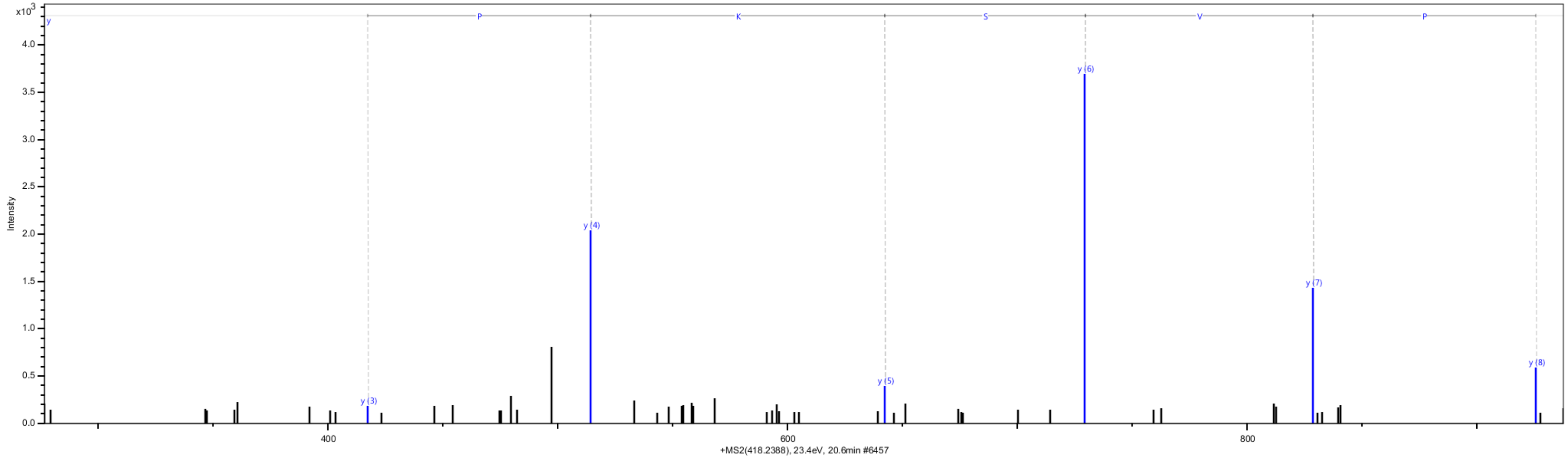

Peptide Match

Sequence: K.WLSEEQR.Y
Score: 20.6
Modifications:

Spectrum Report
Combine Search_2020-10-19 07:53:31

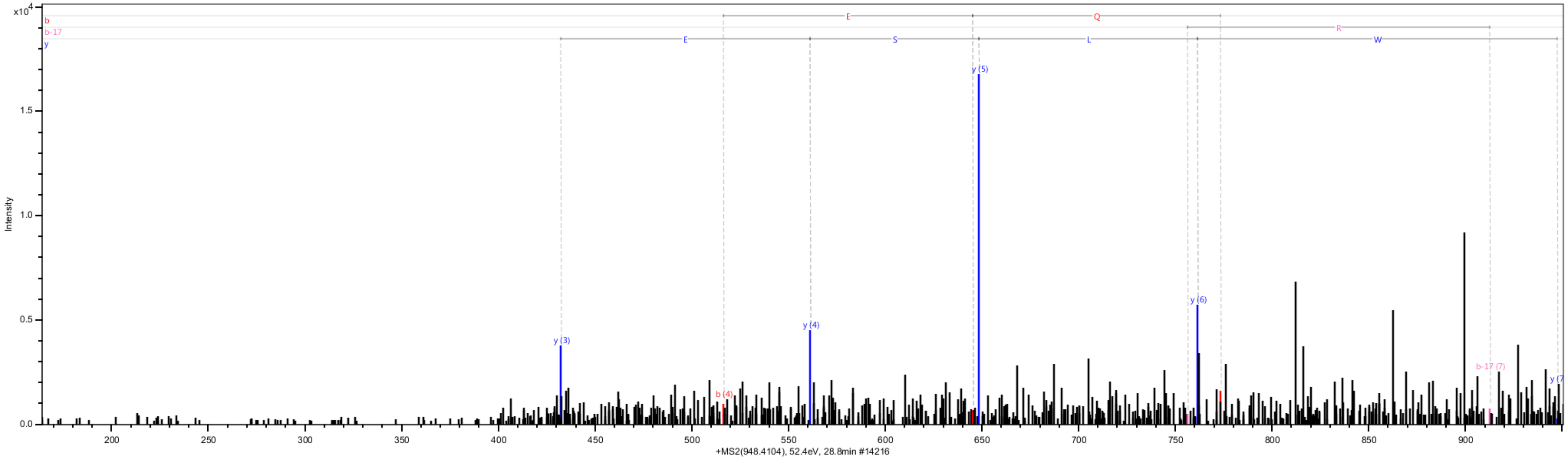

C1QC_HUMAN

Accession: C1QC_HUMAN
Database: SwissProt
MW [kDa] / pI: 25.8 / 8.6
Score: 59.5
Seq. Coverage [%]: 9.0
No. of Peptides: 2

Peptide Match

Sequence: K.FQSVFTVTR.Q
Score: 25.5
Modifications:

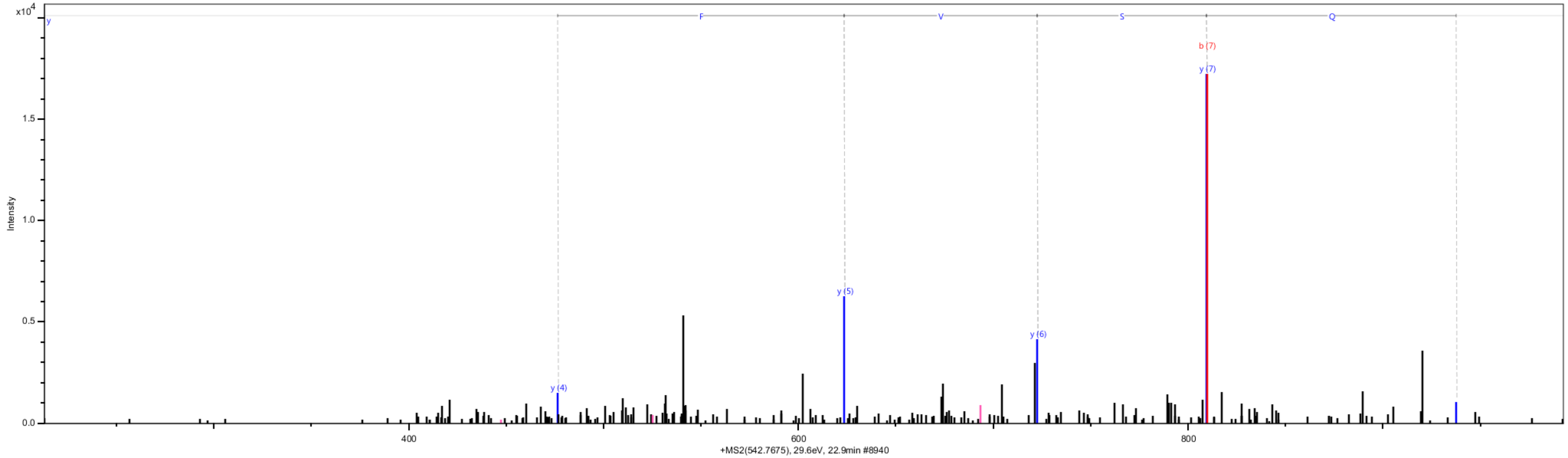

Peptide Match

Sequence: R.QTHQPPAPNSLIR.F
Score: 34
Modifications:

Spectrum Report
Combine Search_2020-10-19 07:53:31

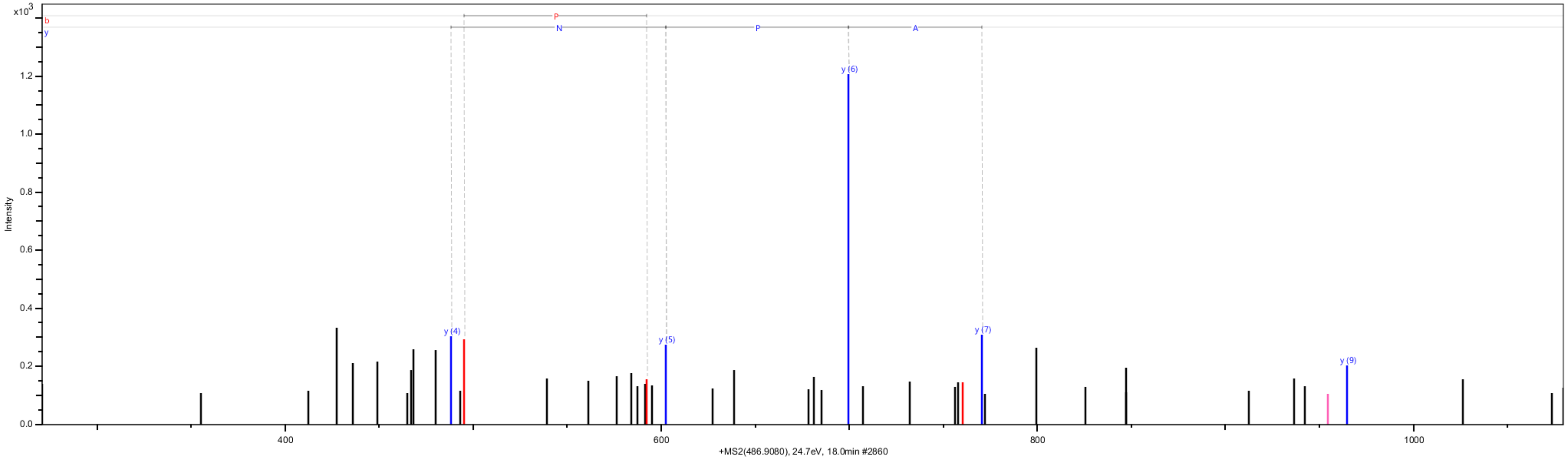

SYNE2_HUMAN

Accession: SYNE2_HUMAN
Database: SwissProt
MW [kDa] / pI: 795.9 / 5.3
Score: 58.6
Seq. Coverage [%]: 0.1
No. of Peptides: 1

Peptide Match

Sequence: K.RLYHQLIK.S
Score: 22.1
Modifications:

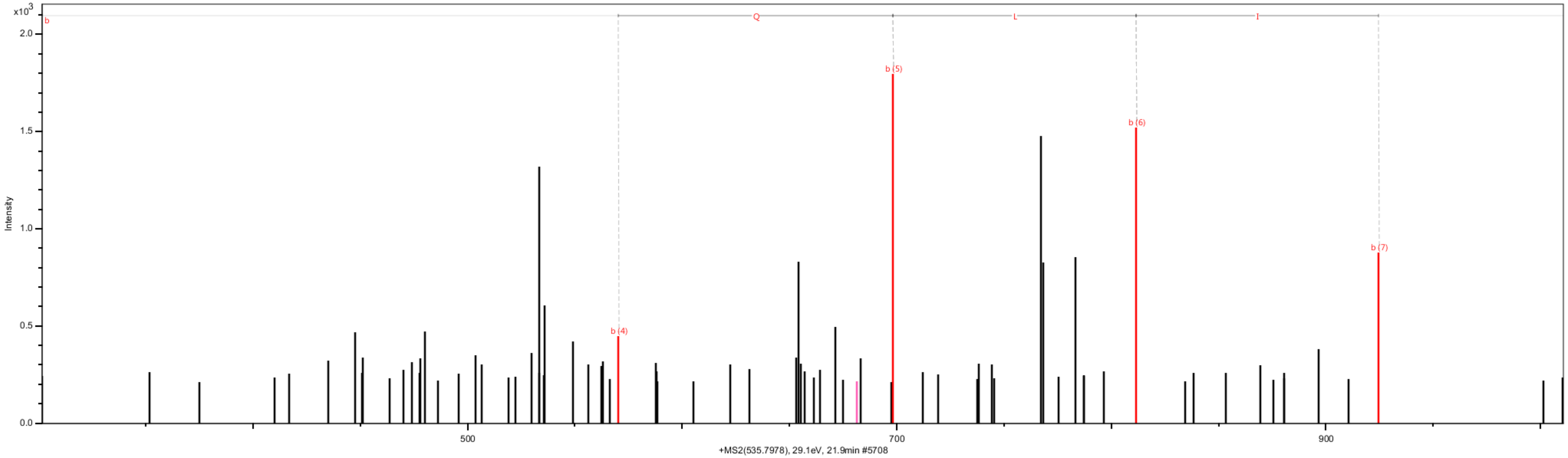

CFAI_HUMAN

Accession: CFAI_HUMAN
Database: SwissProt
MW [kDa] / pI: 65.7 / 7.7

Score: 57.8
Seq. Coverage [%]: 3.9
No. of Peptides: 2

Peptide Match

Sequence: R.GLETSLAECTFTK.R
Score: 24.1
Modifications: Carbamidomethyl: 9

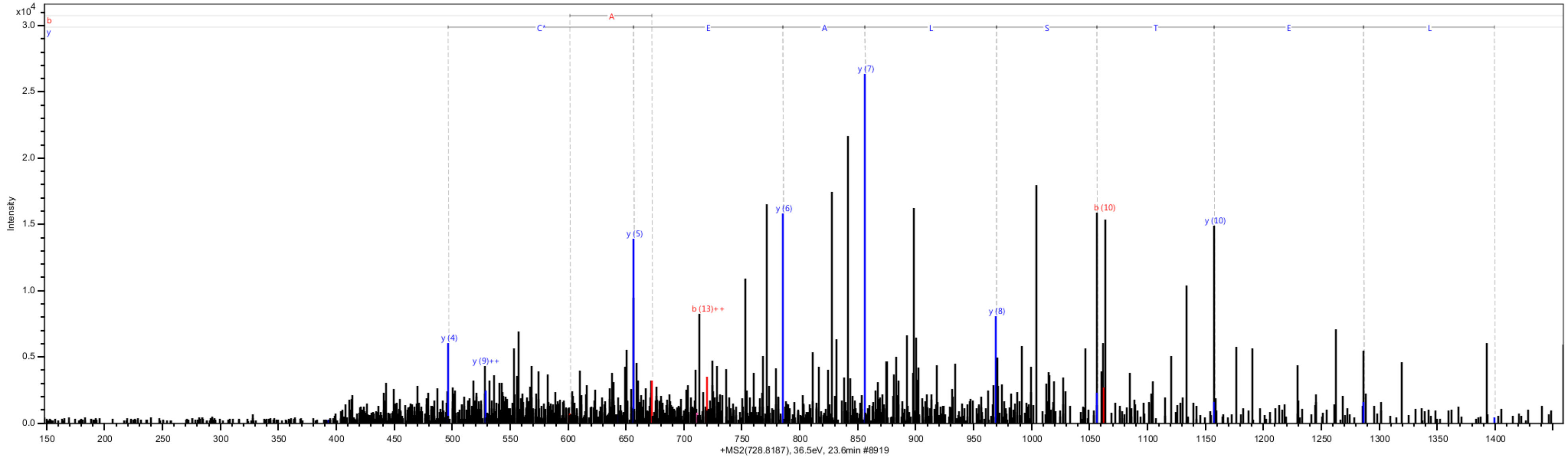

Peptide Match

Sequence: R.VFSLQWGEVK.L
Score: 33.7
Modifications:

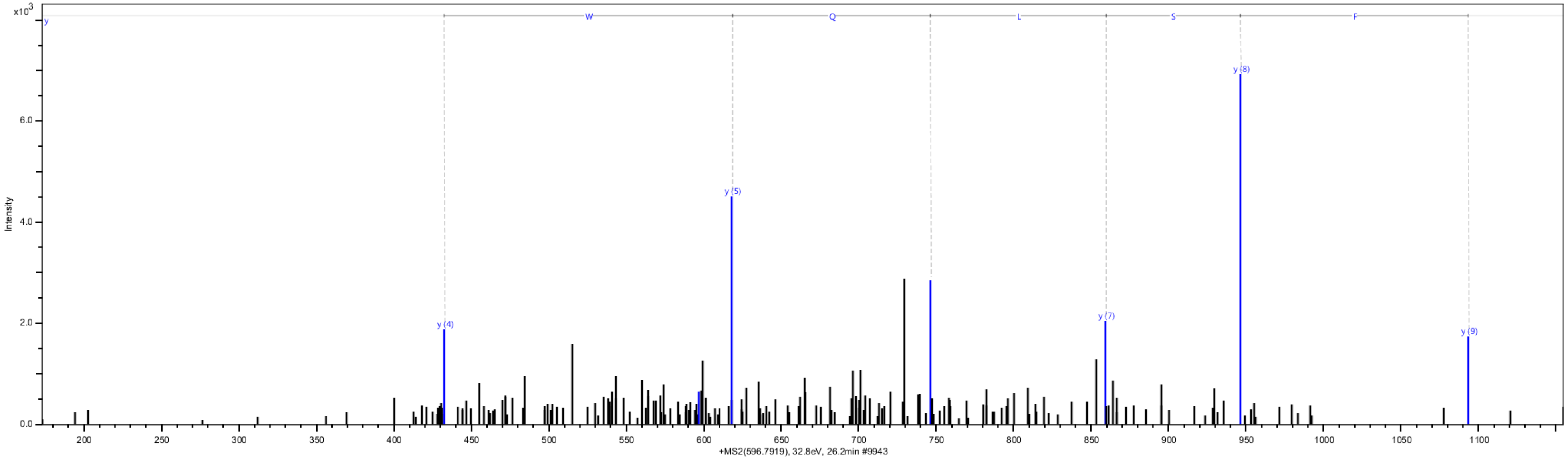
x10^3
Intensity
y
W
Q
L
S
F
y (4)
y (5)
y (7)
y (8)
y (9)
6.0
4.0
2.0
0.0
200
250
300
350
400
450
500
550
600
650
700
750
800
850
900
950
1000
1050
1100
+MS2(596.7919), 32.8eV, 26.2min #9943

IC1_HUMAN

Accession: IC1_HUMAN
Database: SwissProt
MW [kDa] / pI: 55.1 / 6.1
Score: 60.1
Seq. Coverage [%]: 4.2
No. of Peptides: 2

Peptide Match

Sequence: K.TNLESILSYPK.D
Score: 15.5
Modifications:

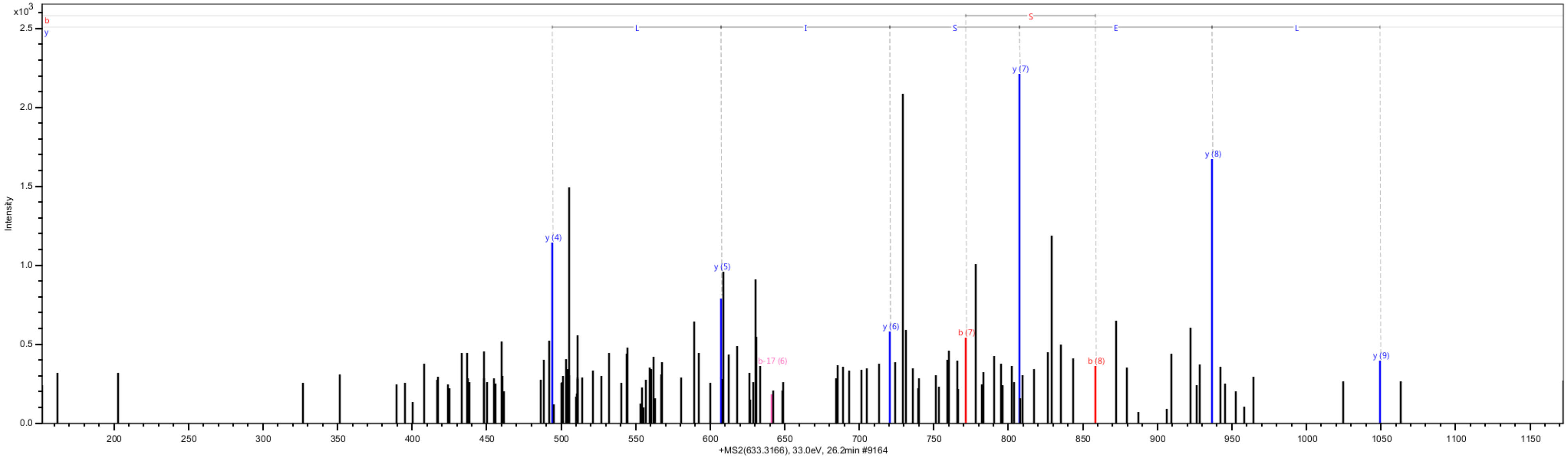

Peptide Match

Sequence: K.FQPTLLTLPR.I
Score: 44.6
Modifications:

Spectrum Report
Combine Search_2020-10-19 07:53:31

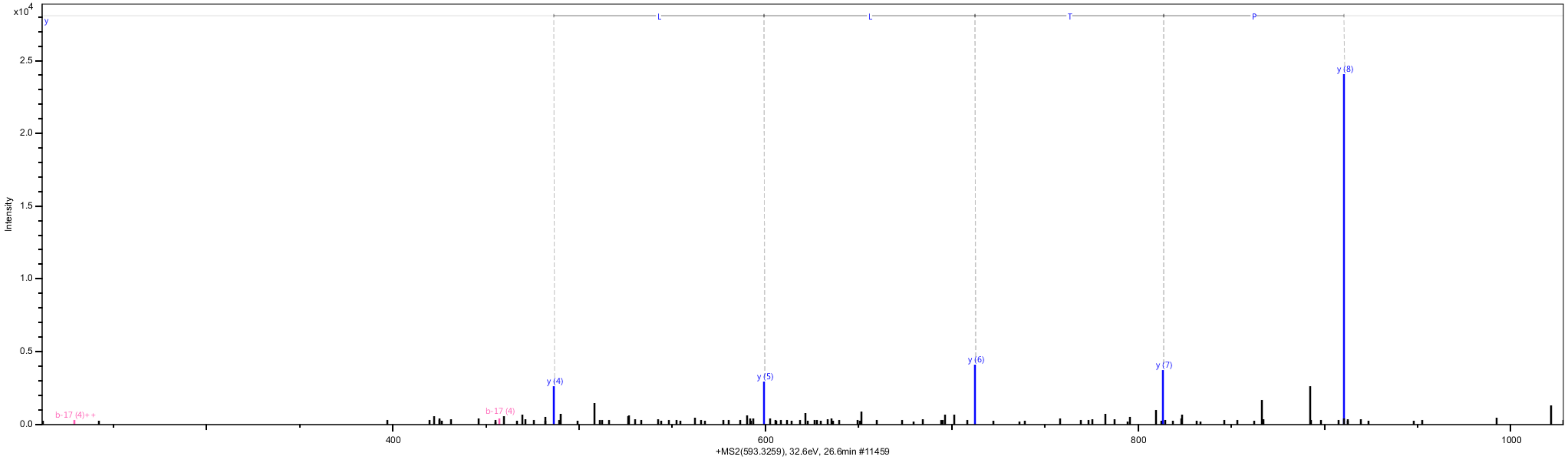

HV323_HUMAN

Accession: HV323_HUMAN
Database: SwissProt
MW [kDa] / pI: 12.6 / 8.5
Score: 42.5
Seq. Coverage [%]: 18.8
No. of Peptides: 2

Peptide Match

Sequence: K.NTLYLQMNSLR.A
Score: 25.8
Modifications:

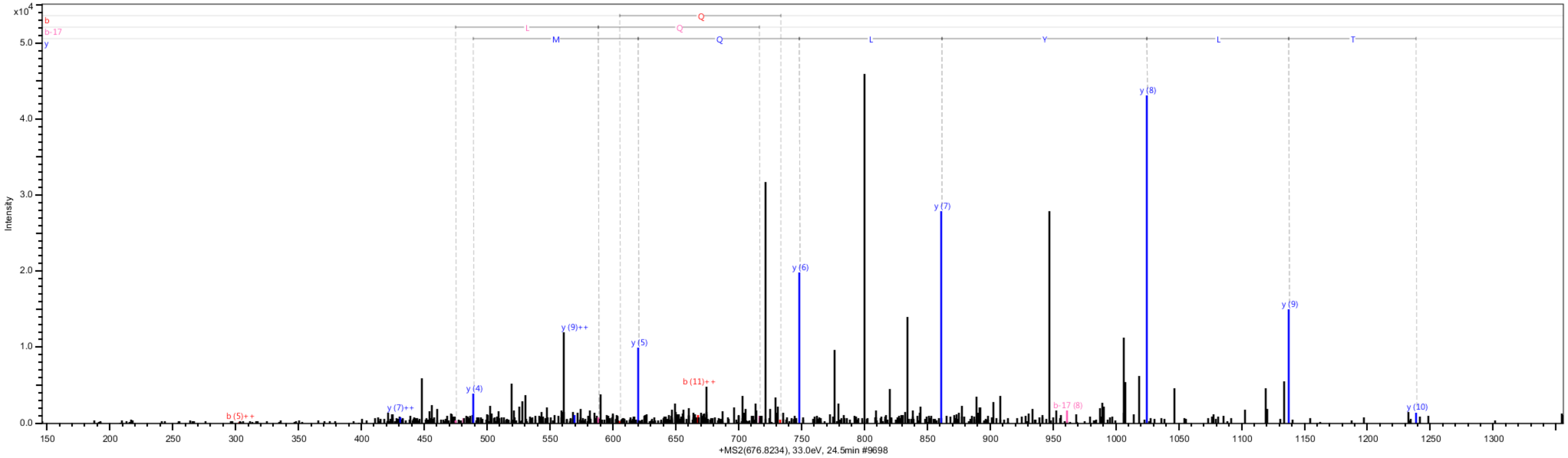

Peptide Match

Sequence: R.AEDTAVYYCAK.-
Score: 16.6
Modifications: Carbamidomethyl: 9

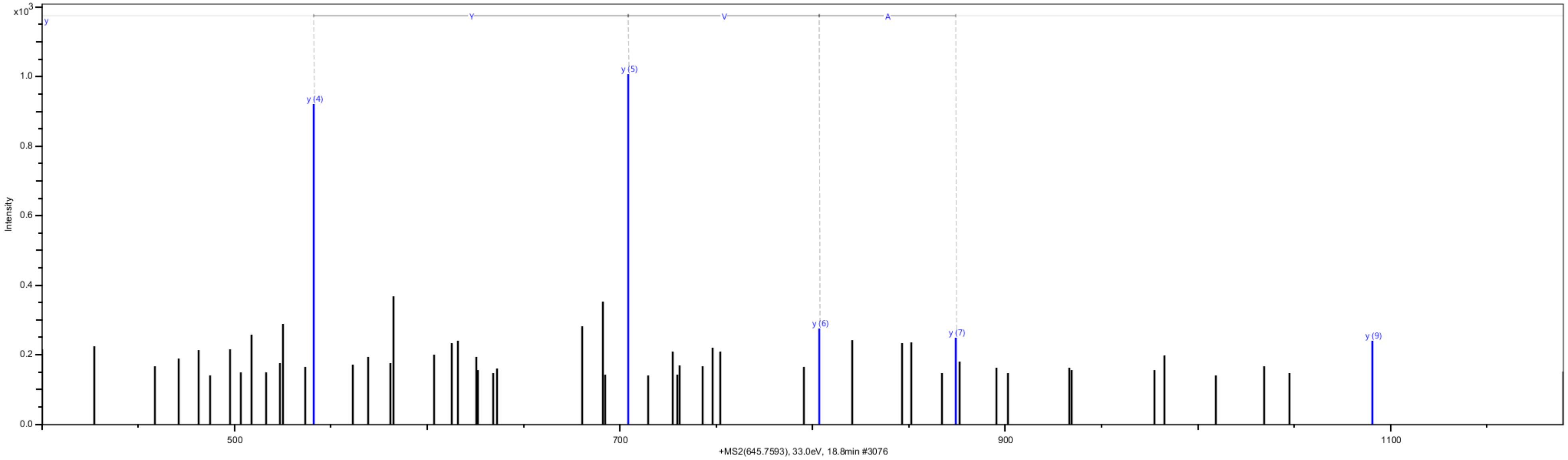
x10^3
Intensity
y
Y
V
A
y (4)
y (5)
y (6)
y (7)
y (9)
1.0
0.8
0.6
0.4
0.2
0.0
500
700
900
1100
+MS2(645.7593), 33.0eV, 18.8min #3076

CHD9_HUMAN

**Accession:**	CHD9_HUMAN	**Score:**	45.2
**Database:**	SwissProt	**Seq. Coverage [%]:**	0.3
**MW [kDa] / pI:**	325.8 / 6.6	**No. of Peptides:**	1

Peptide Match

**Sequence:**	K.NFMNVSGPHR.V	**Score:**	18.3
**Modifications:**	Oxidation: 3		

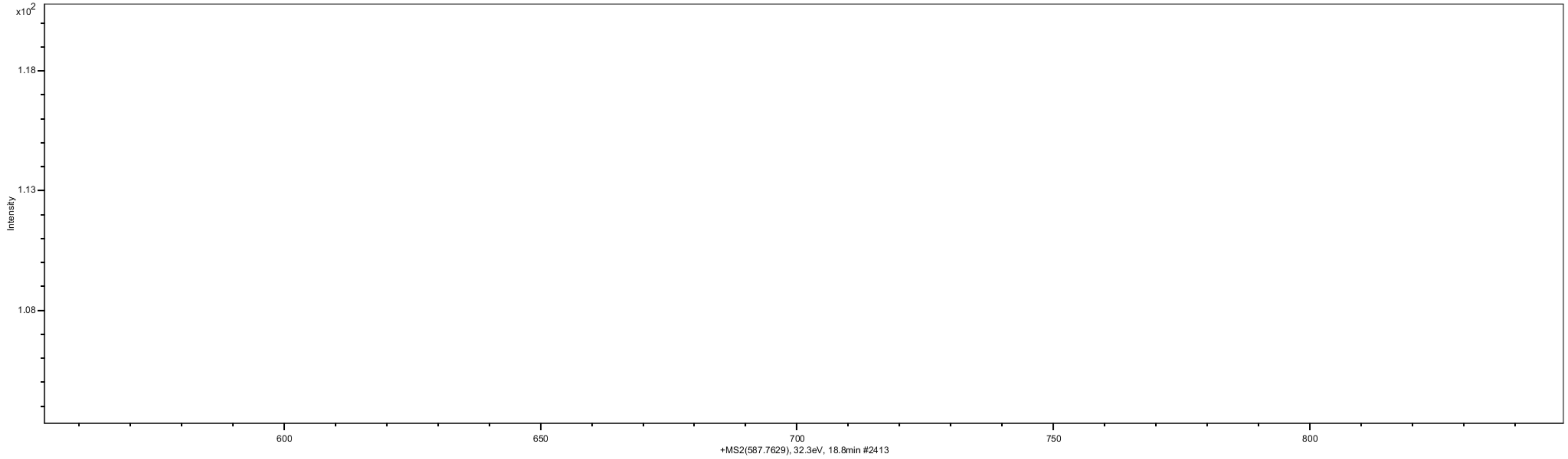

FHR1_HUMAN

**Accession:**	FHR1_HUMAN	**Score:**	46.2
**Database:**	SwissProt	**Seq. Coverage [%]:**	3.9
**MW [kDa] / pI:**	37.6 / 7.4	**No. of Peptides:**	1

Peptide Match

**Sequence:**	R.ITCTEEGWSPTPK.C	**Score:**	46.2
**Modifications:**	Carbamidomethyl: 3		

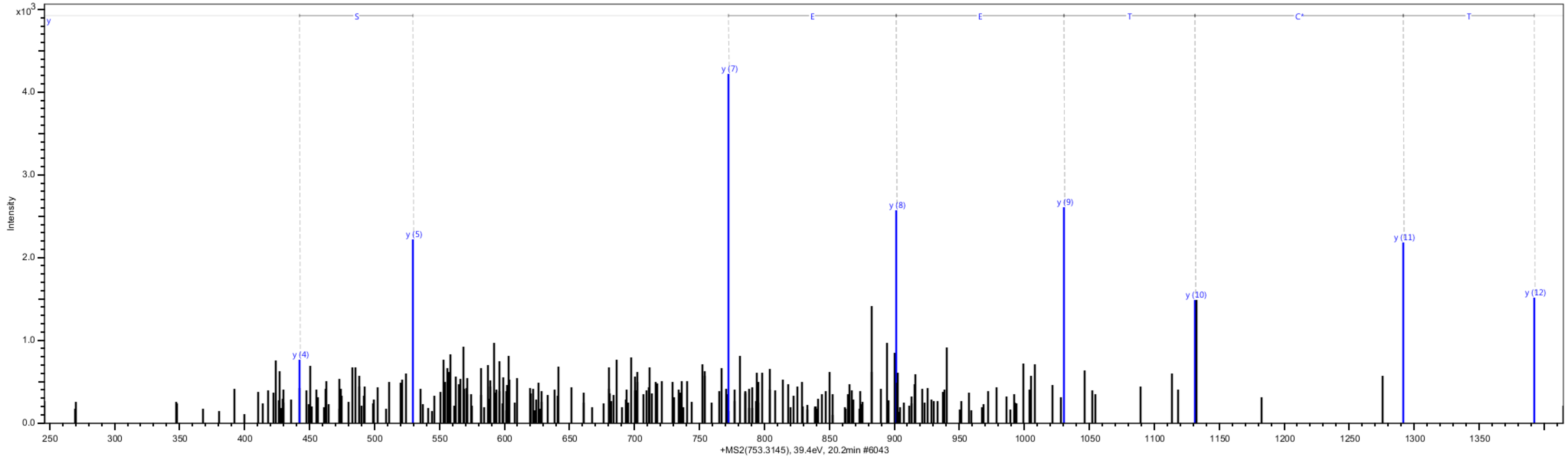

CO7_HUMAN

**Accession:**	CO7_HUMAN	**Score:**	46.3
**Database:**	SwissProt	**Seq. Coverage [%]:**	2.0
**MW [kDa] / pI:**	93.5 / 6.1	**No. of Peptides:**	2

Peptide Match

**Sequence:**	K.LTPLYELVK.E	**Score:**	17.6
**Modifications:**			

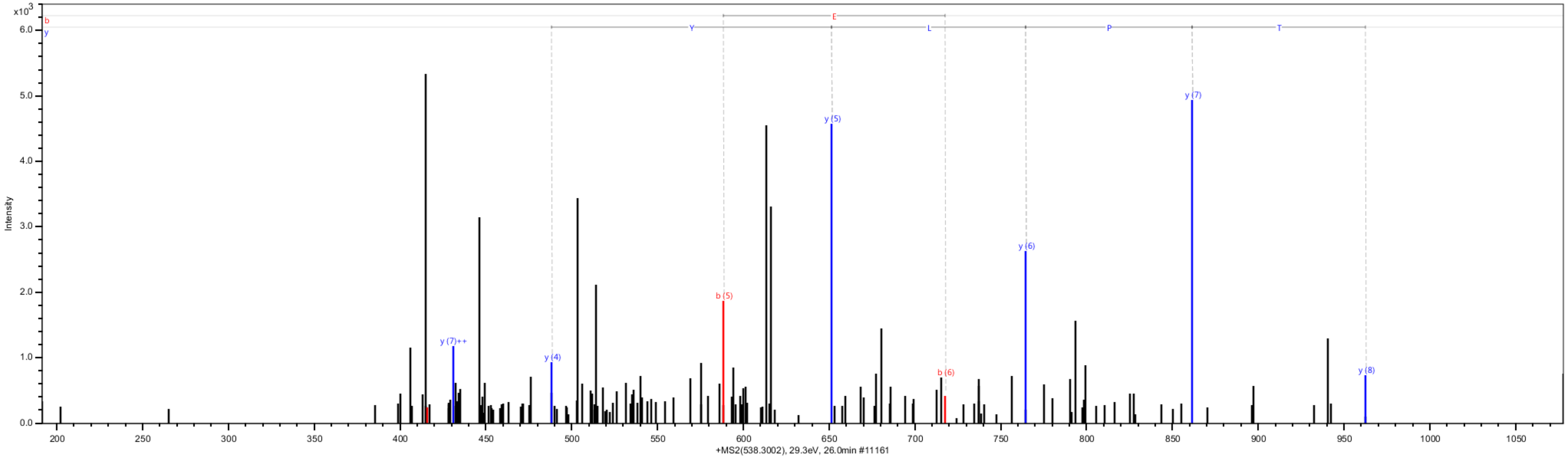

Peptide Match

**Sequence:**	R.ILPLTVCK.M	**Score:**	28.7
**Modifications:**	Carbamidomethyl: 7		

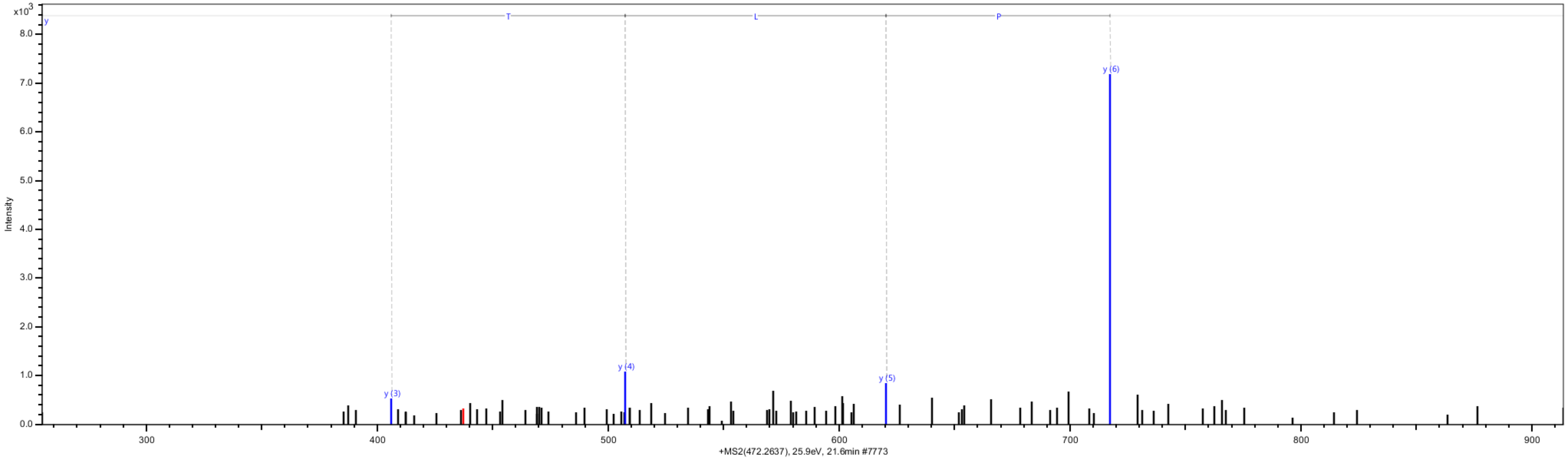
x10^3
y
T
L
P
y (6)
y (4)
y (5)
y (3)
Intensity
8.0
7.0
6.0
5.0
4.0
3.0
2.0
1.0
0.0
300
400
500
600
700
800
900
+MS2(472.2637), 25.9eV, 21.6min #7773

ZFHX4_HUMAN

Accession: ZFHX4_HUMAN
Database: SwissProt
MW [kDa] / pI: 393.5 / 5.9
Score: 46.4
Seq. Coverage [%]: 0.2
No. of Peptides: 1

Peptide Match

Sequence: K.SALESHIR.S
Score: 15.2
Modifications:

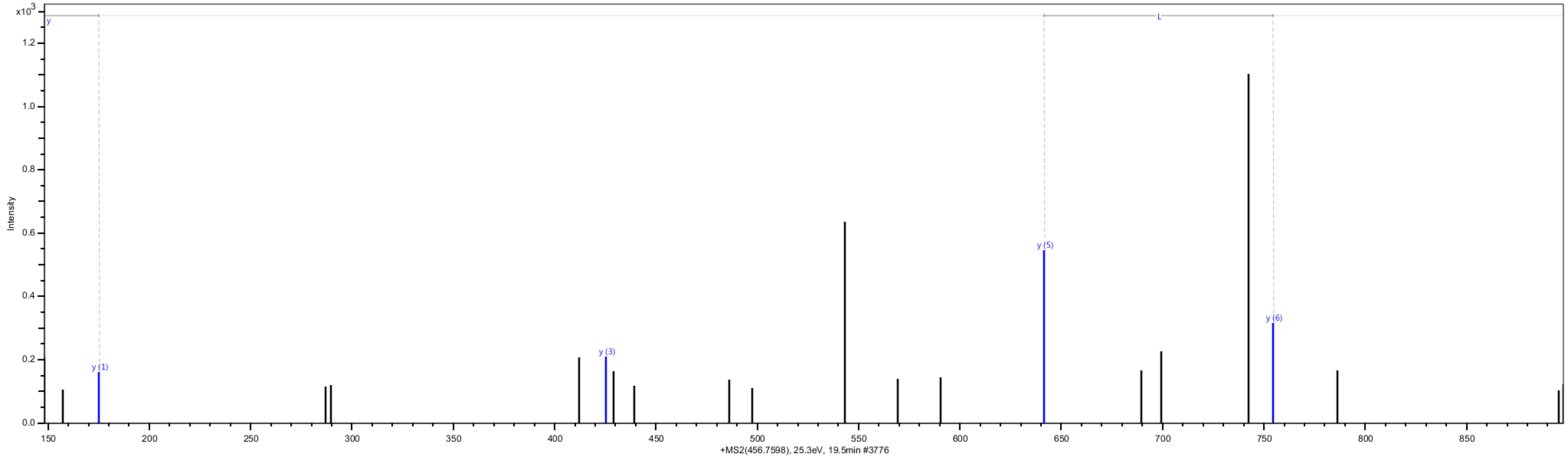

KVD33_HUMAN

**Accession:**	KVD33_HUMAN	**Score:**	46.5
**Database:**	SwissProt	**Seq. Coverage [%]:**	13.7
**MW [kDa] / pI:**	12.8 / 4.5	**No. of Peptides:**	1

Peptide Match

**Sequence:**	K.LLIYDASNLETGVPSR.F	**Score:**	46.5
**Modifications:**			

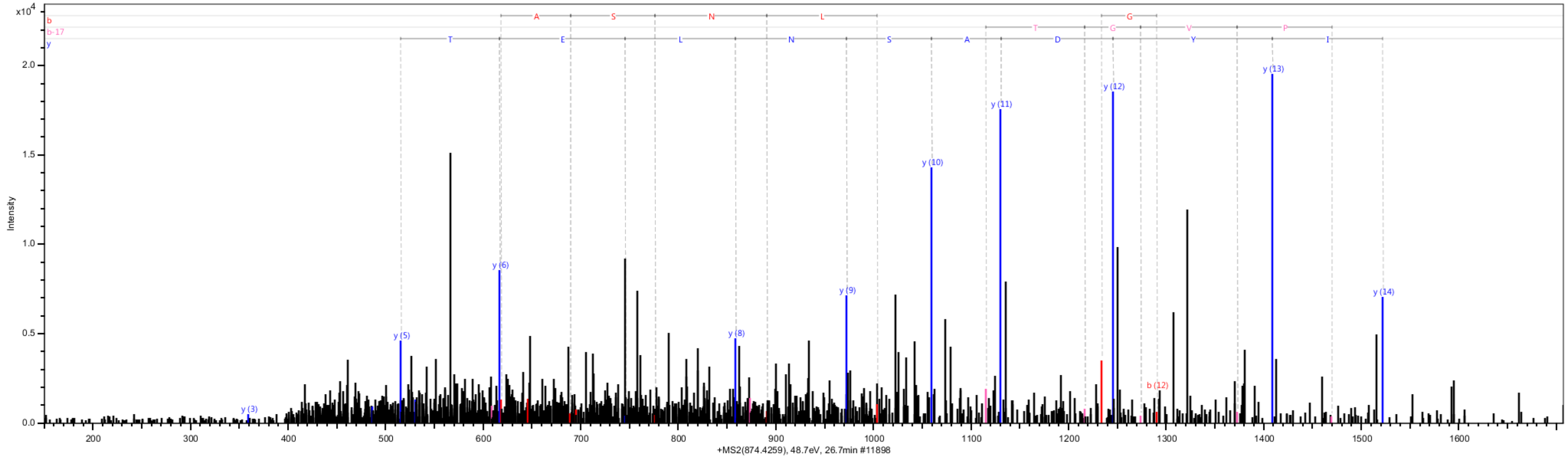

CAPS1_HUMAN

**Accession:**	CAPS1_HUMAN	**Score:**	42.3
**Database:**	SwissProt	**Seq. Coverage [%]:**	2.2
**MW [kDa] / pI:**	152.7 / 5.5	**No. of Peptides:**	2

Peptide Match

**Sequence:**	R.LQMADQIAR.E	**Score:**	27.1
**Modifications:**	Oxidation: 3		

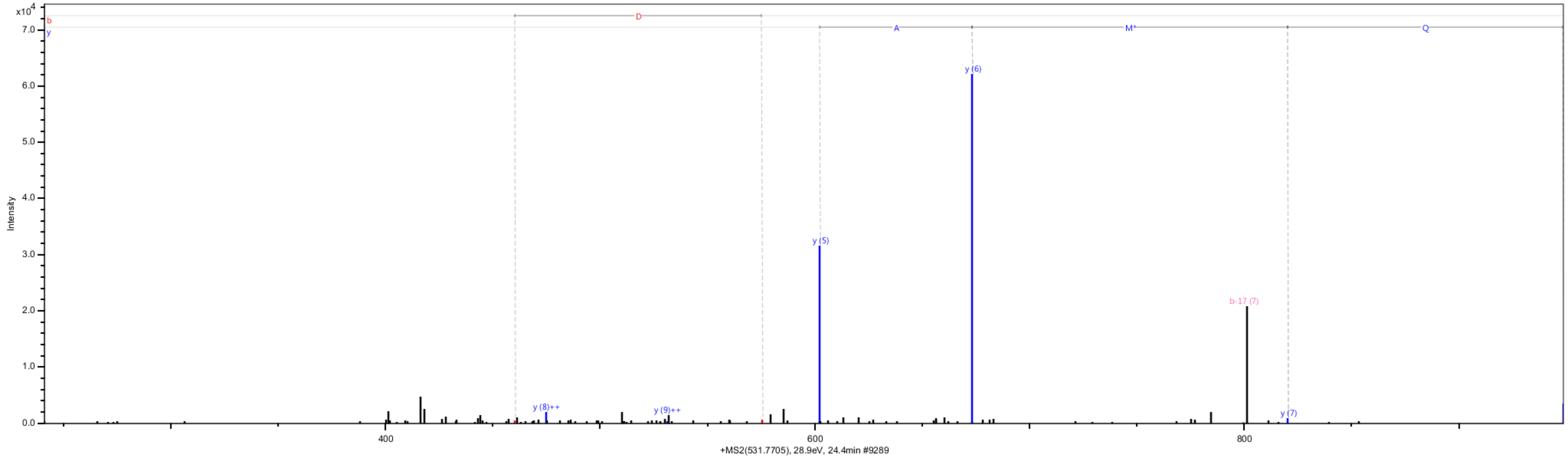

Peptide Match

**Sequence:**	R.STDFRVPQSICTMFNVMVDAK.A	**Score:**	15.2
**Modifications:**	Carbamidomethyl: 11; Oxidation: 17		

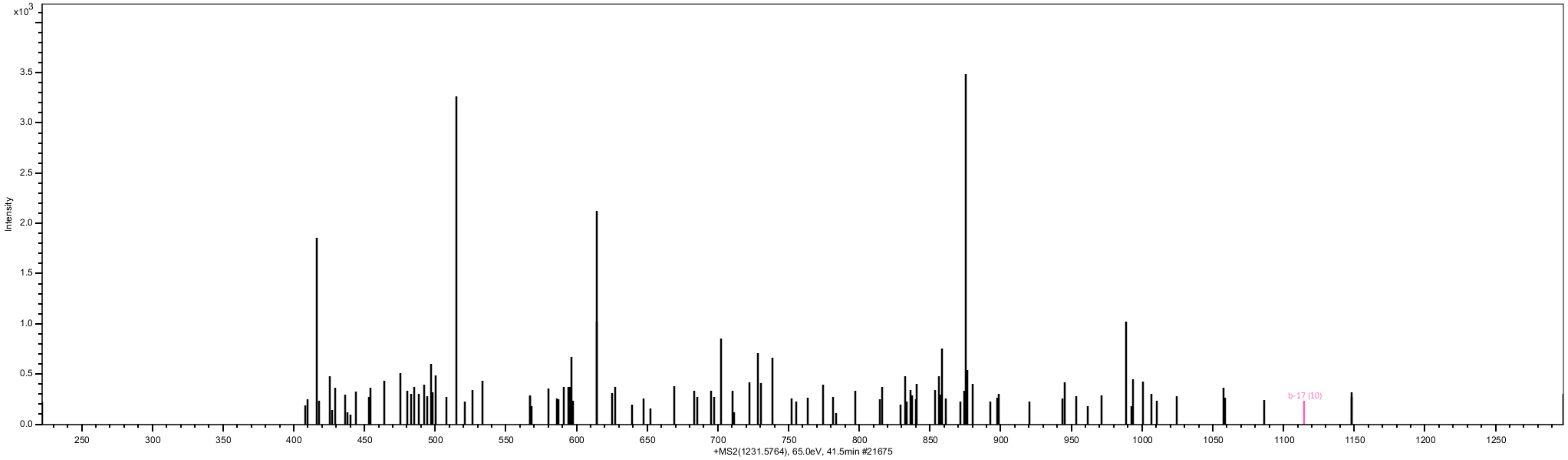
x10^3
Intensity
3.5
3.0
2.5
2.0
1.5
1.0
0.5
0.0
250
300
350
400
450
500
550
600
650
700
750
800
850
900
950
1000
1050
1100
1150
1200
1250
b-17 (10)
+MS2(1231.5764), 65.0eV, 41.5min #21675

EZRI_HUMAN

Accession: EZRI_HUMAN
Database: SwissProt
MW [kDa] / pI: 69.4 / 5.9
Score: 42.4
Seq. Coverage [%]: 2.7
No. of Peptides: 2

Peptide Match

Sequence: K.IGFPWSEIR.N
Score: 23.2
Modifications:

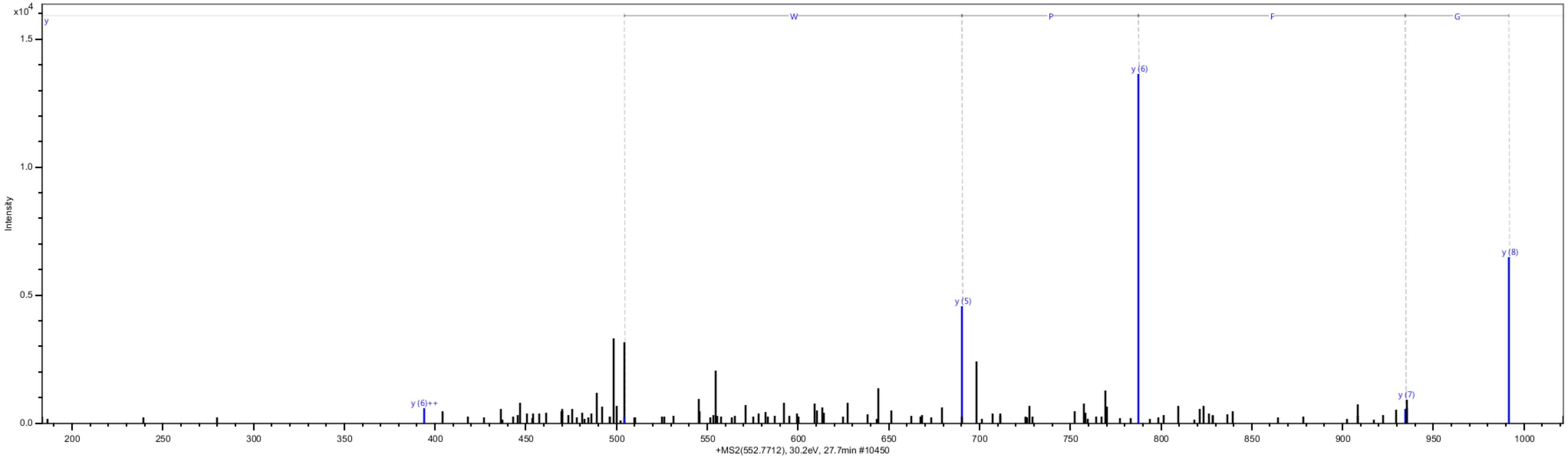

Peptide Match

Sequence: R.QAVDQIK.S
Score: 19.2
Modifications:

Spectrum Report
Combine Search_2020-10-19 07:53:31

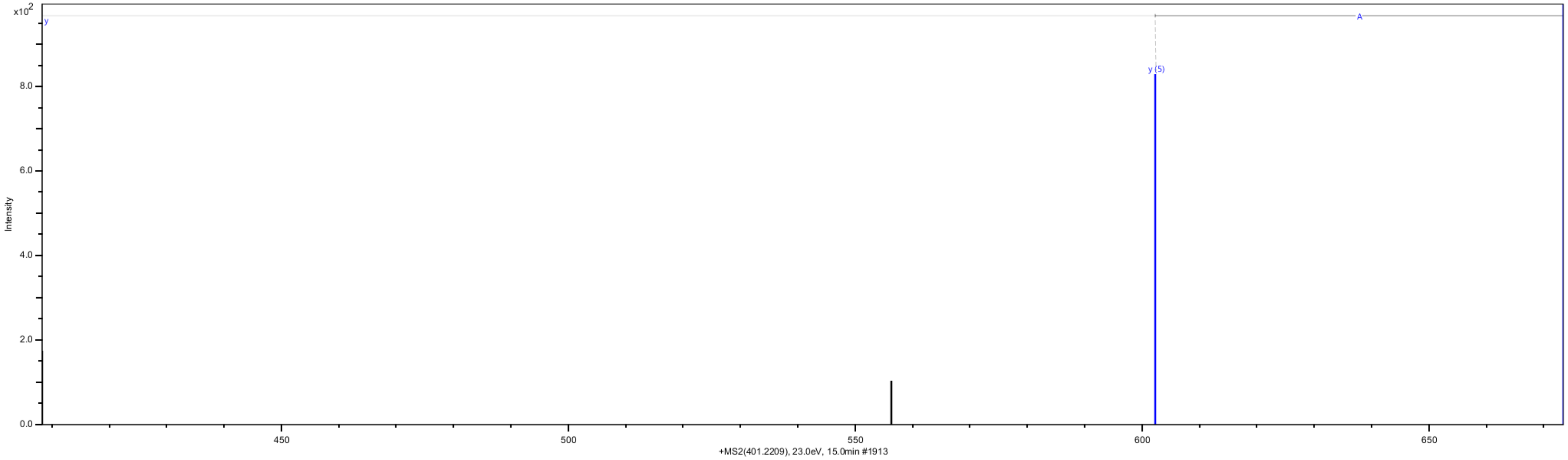

IGLC2_HUMAN

Accession: IGLC2_HUMAN
Database: SwissProt
MW [kDa] / pI: 11.3 / 6.9
Score: 244.6
Seq. Coverage [%]: 62.3
No. of Peptides: 5

Peptide Match

Sequence: K.AAPSVTLFPPSSEELQANK.A
Score: 34.4
Modifications:

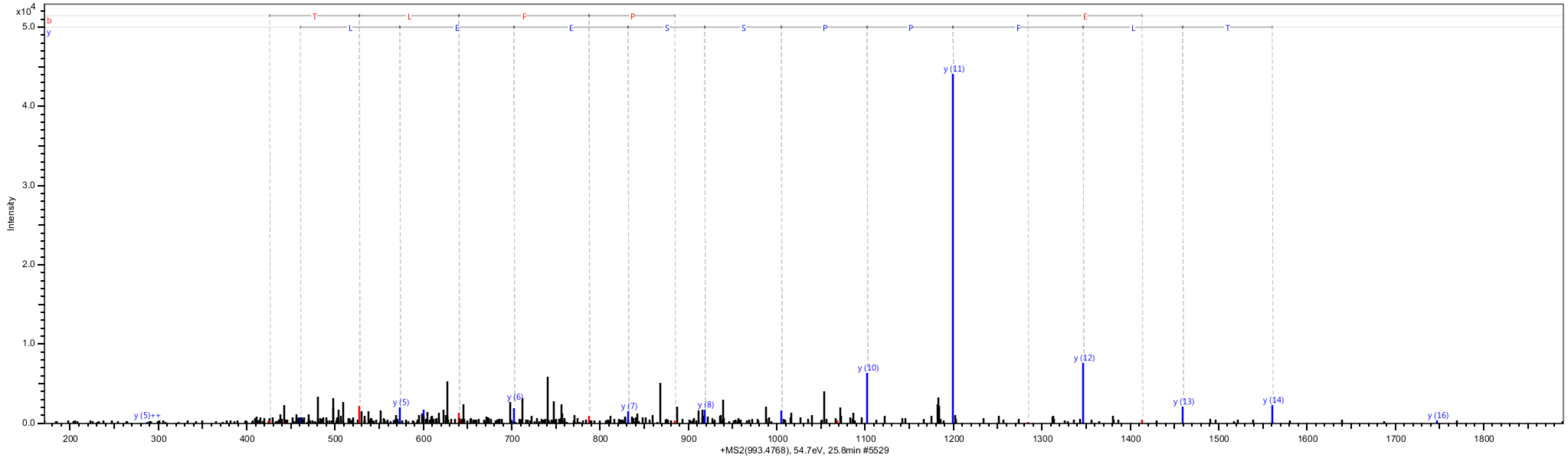

Peptide Match

Sequence: K.ADSSPVKAGVETTTPSK.Q
Score: 37.3
Modifications:

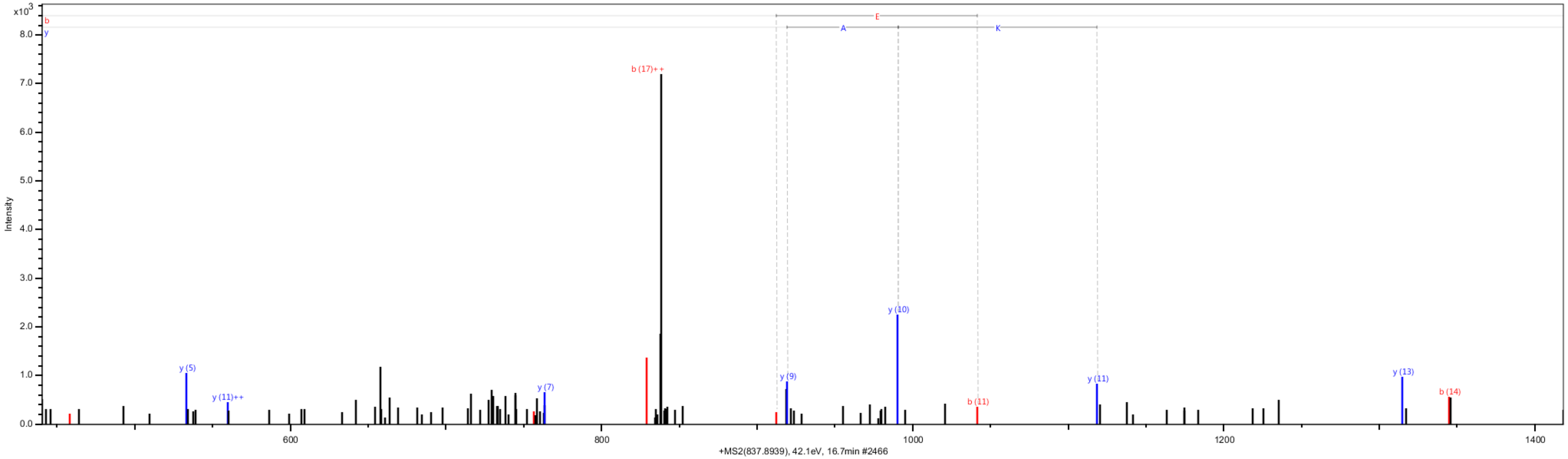

Peptide Match

Sequence: K.AGVETTTPSK.Q **Score:** 16.6

Modifications:

Spectrum Report
Combine Search_2020-10-19 07:53:31

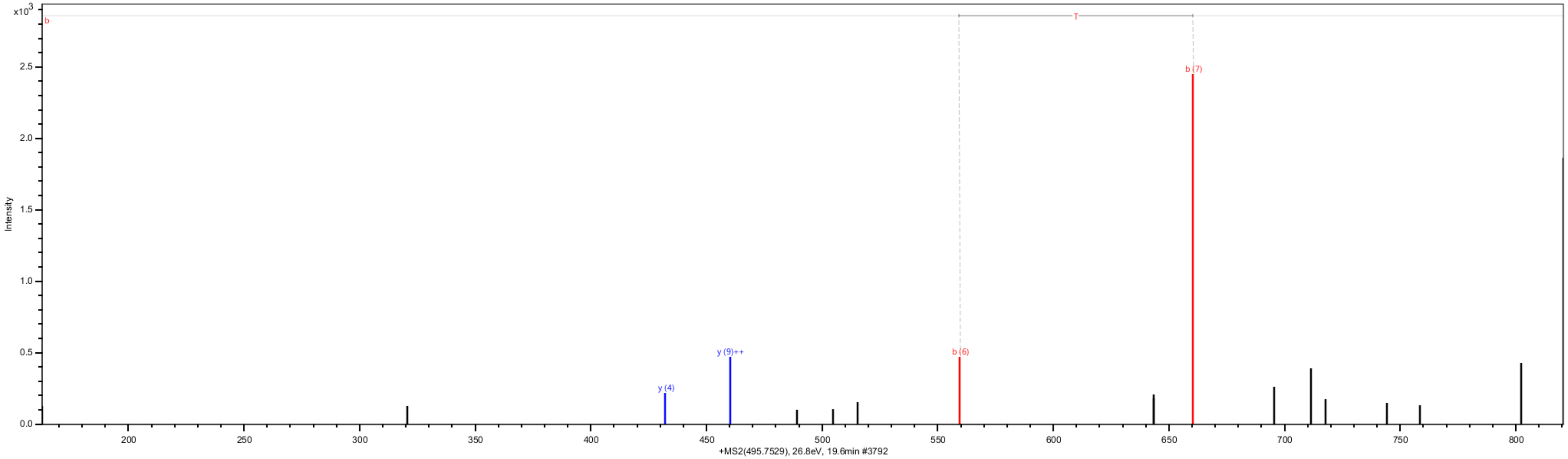

Peptide Match

Sequence: K.YAASSYLSLTPEQWK.S **Score:** 118.4

Modifications:

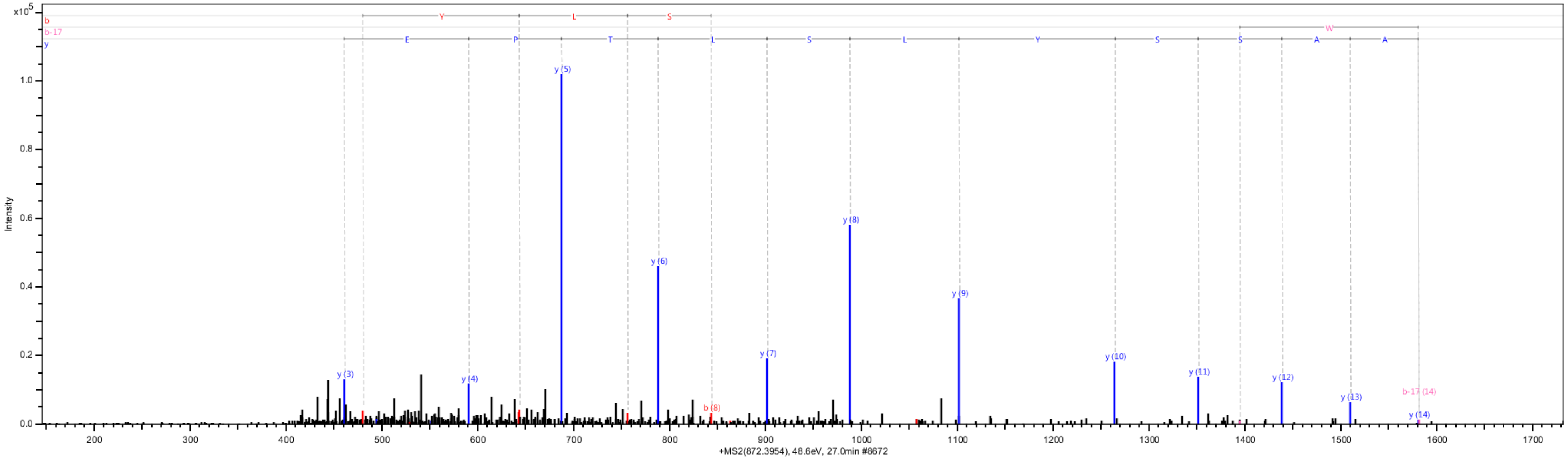

Peptide Match

Sequence: R.SYSCQVTHEGSTVEK.T **Score:** 37.9

Modifications: Carbamidomethyl: 4

Spectrum Report
Combine Search_2020-10-19 07:53:31

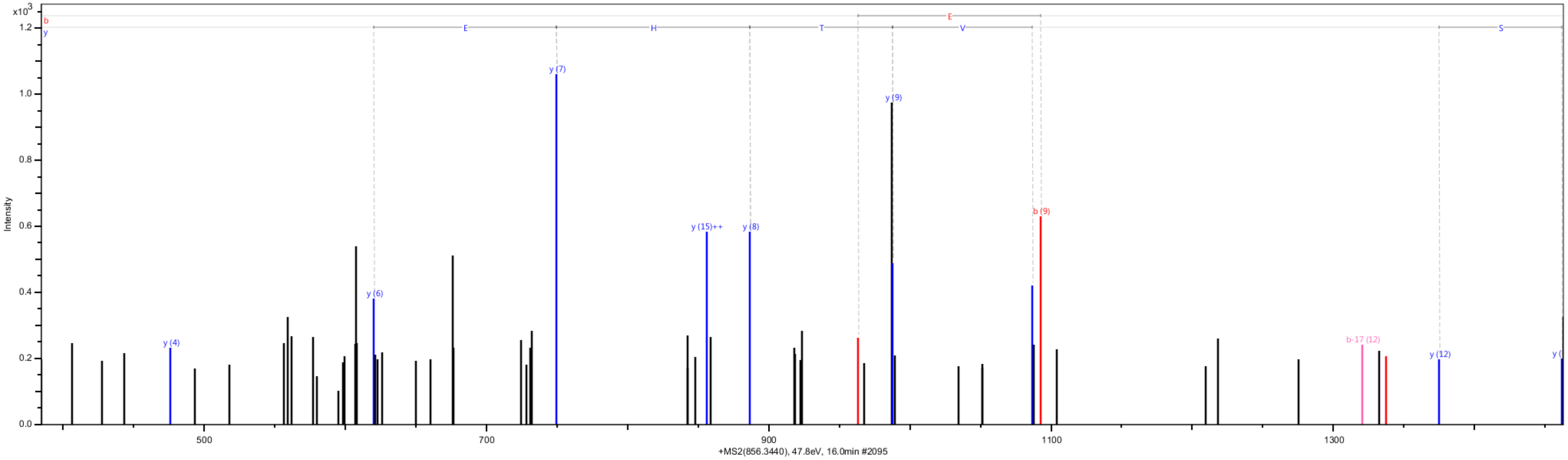

FETUA_HUMAN

Accession: FETUA_HUMAN
Database: SwissProt
MW [kDa] / pI: 39.3 / 5.4
Score: 236.3
Seq. Coverage [%]: 21.5
No. of Peptides: 6

Peptide Match

Sequence: K.EHAVEGDCDFQLLK.L
Score: 27.4
Modifications: Carbamidomethyl: 8

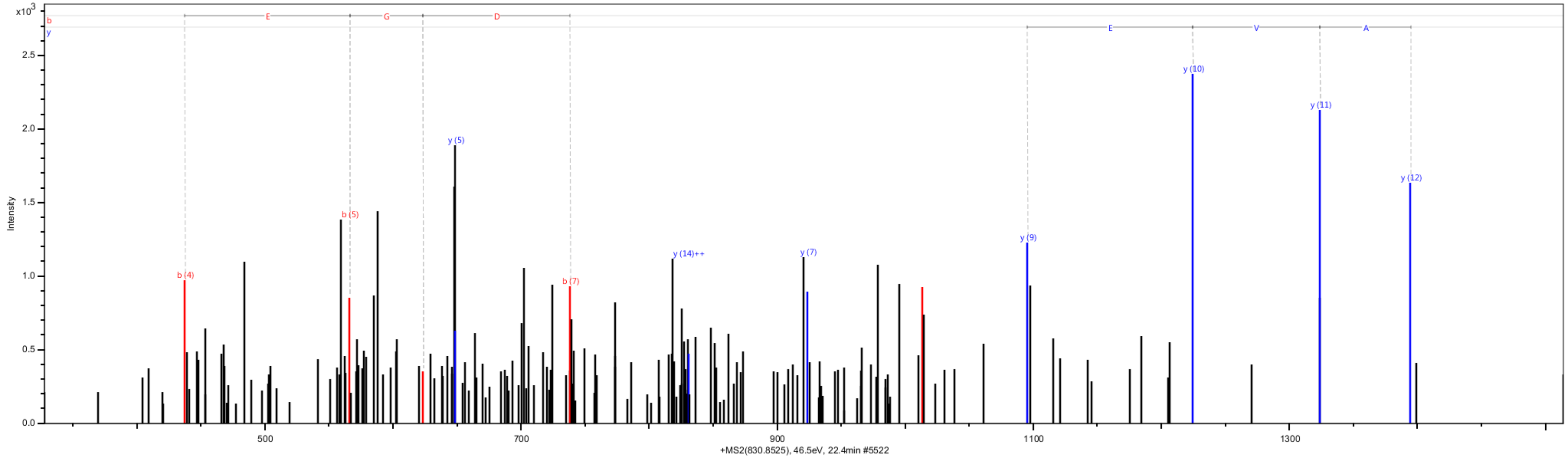

Peptide Match

Sequence: K.LDGKFSVVYAK.C
Score: 19.8
Modifications:

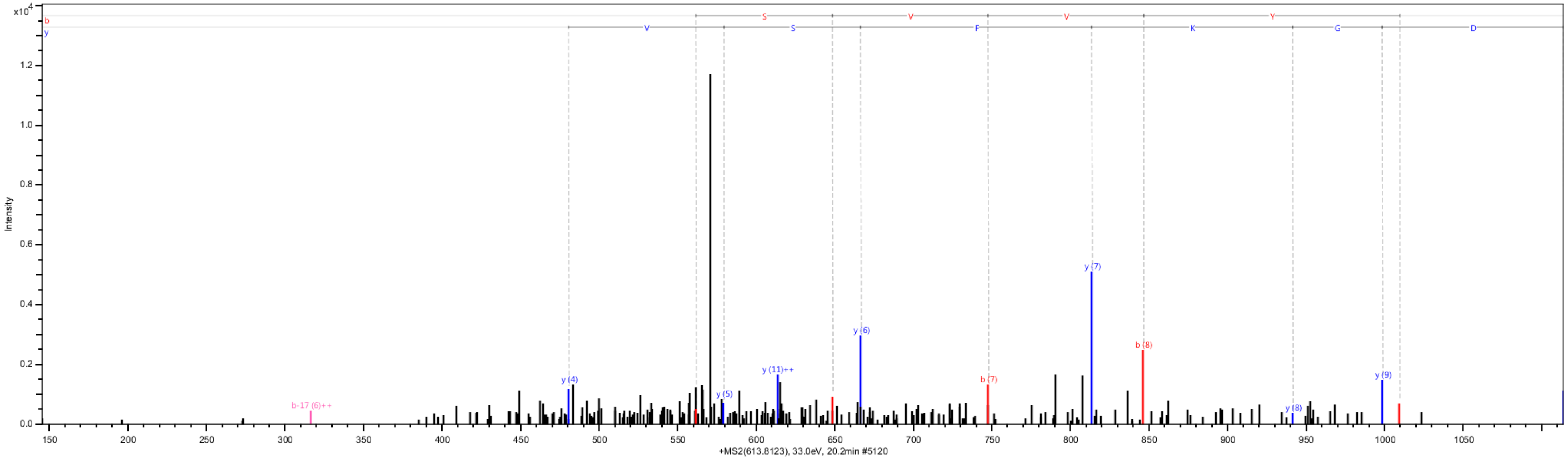

Peptide Match

Sequence: K.FSVVYAK.C **Score:** 24.9

Modifications:

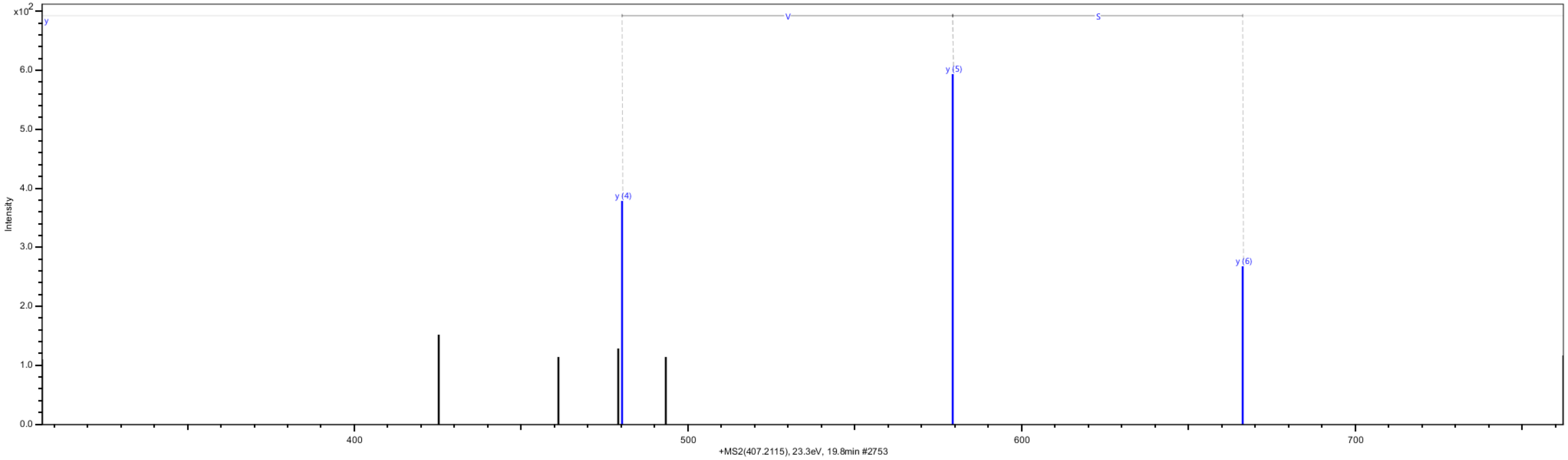

Peptide Match

**Sequence:**	K.CNLLAEKQYGFCK.A	**Score:**	37.7
**Modifications:**	Carbamidomethyl: 1, 12		

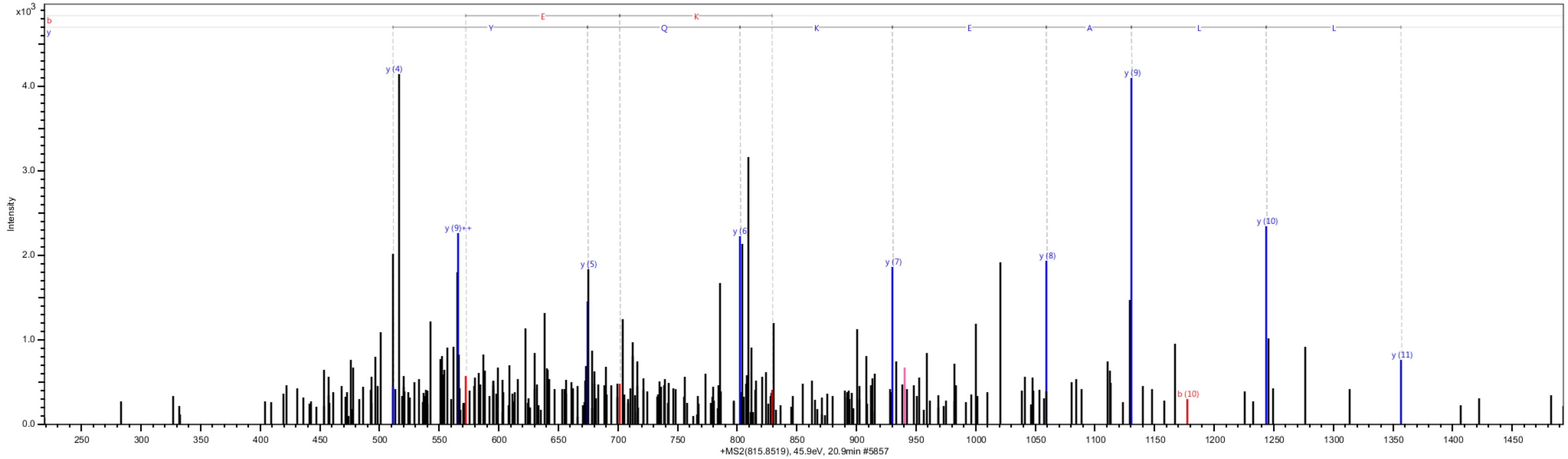

Peptide Match

Sequence: R.HTFMGVVSLGSPSGEVSHPR.K **Score:** 43.3

Modifications:

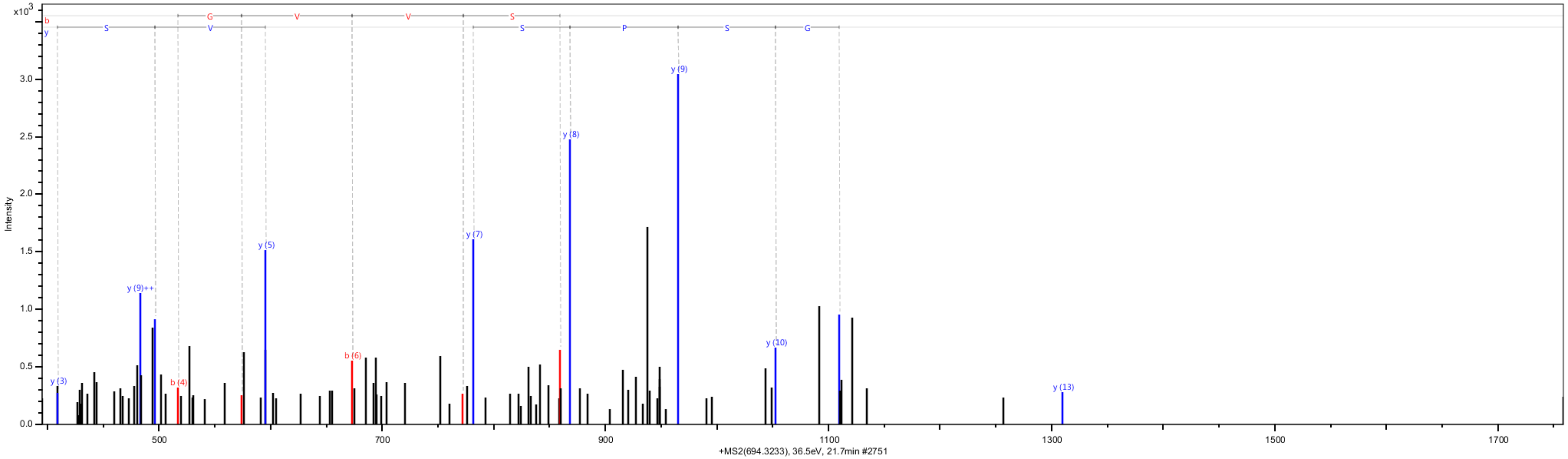

Peptide Match

**Sequence:**	R.TVVQPSVGAAAGPVVPPCPGR.I	**Score:**	83.3
**Modifications:**	Carbamidomethyl: 18		

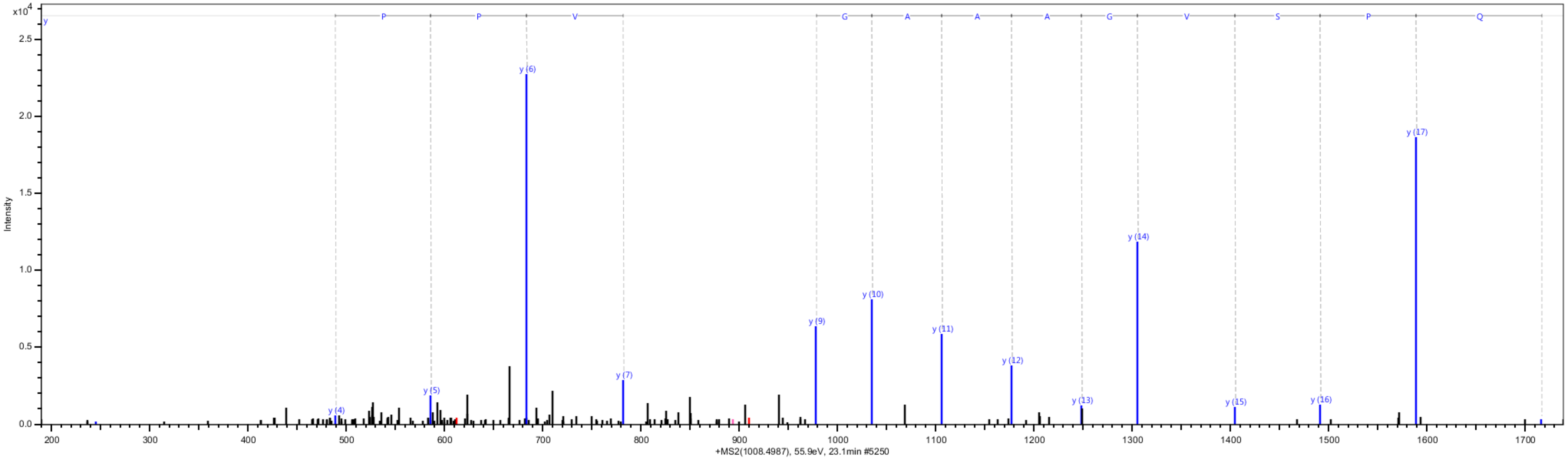
x10^4
Intensity
y (4)
y (5)
y (6)
y (7)
y (9)
y (10)
y (11)
y (12)
y (13)
y (14)
y (15)
y (16)
y (17)
P P V G A A A G V S P Q
+MS2(1008.4987), 55.9eV, 23.1min #5250

HBA_HUMAN

Accession: HBA_HUMAN
Database: SwissProt
MW [kDa] / pI: 15.2 / 8.7
Score: 332.1
Seq. Coverage [%]: 50.7
No. of Peptides: 8

Peptide Match

Sequence: M.VLSPADKTNVK.A
Score: 34.4
Modifications:

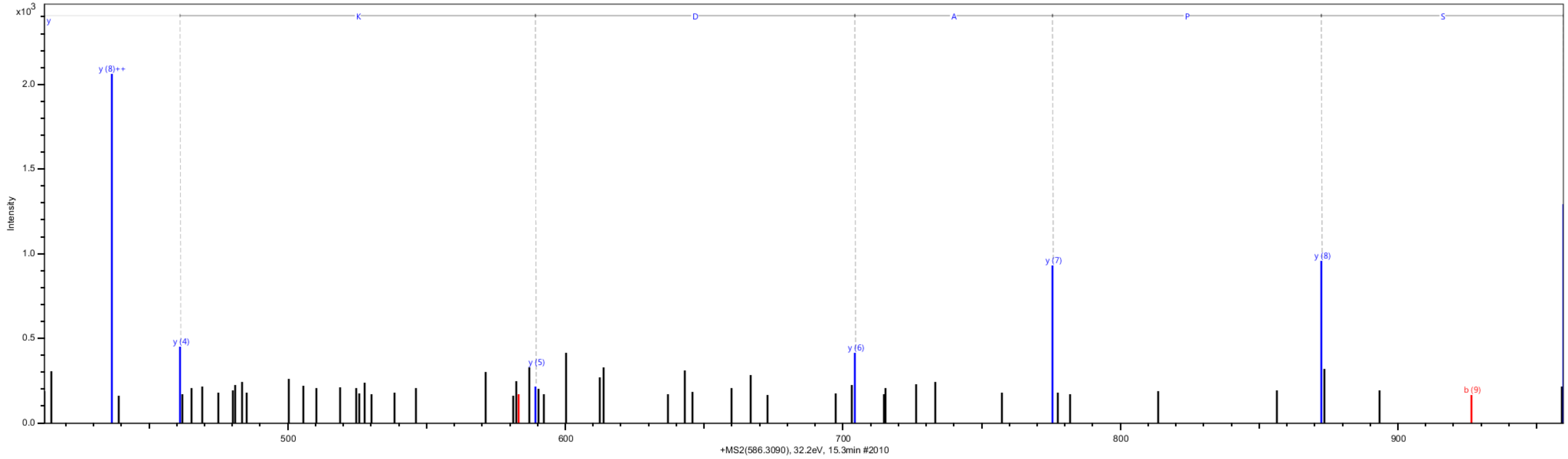

Peptide Match

Sequence: K.VGAHAGEYGAEALER.M
Score: 74
Modifications:

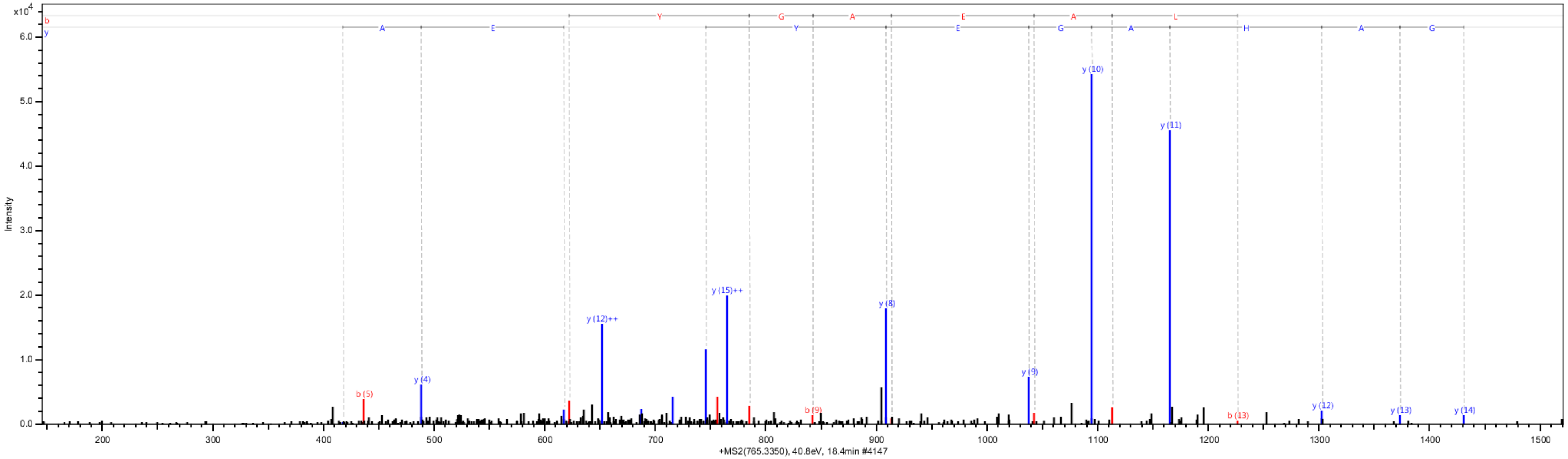

Peptide Match

**Sequence:**	R.MFLSFPTTK.T	**Score:**	31.6
**Modifications:**	Oxidation: 1		

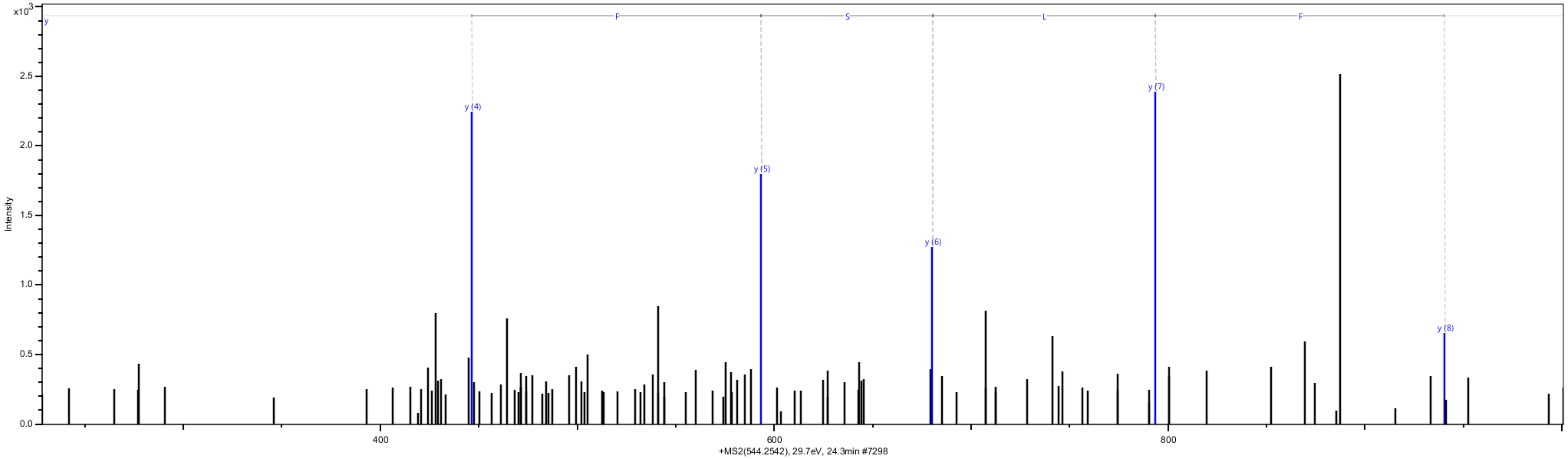

Peptide Match

Sequence: R.MFLSFPTTK.T **Score:** 54.5

Modifications:

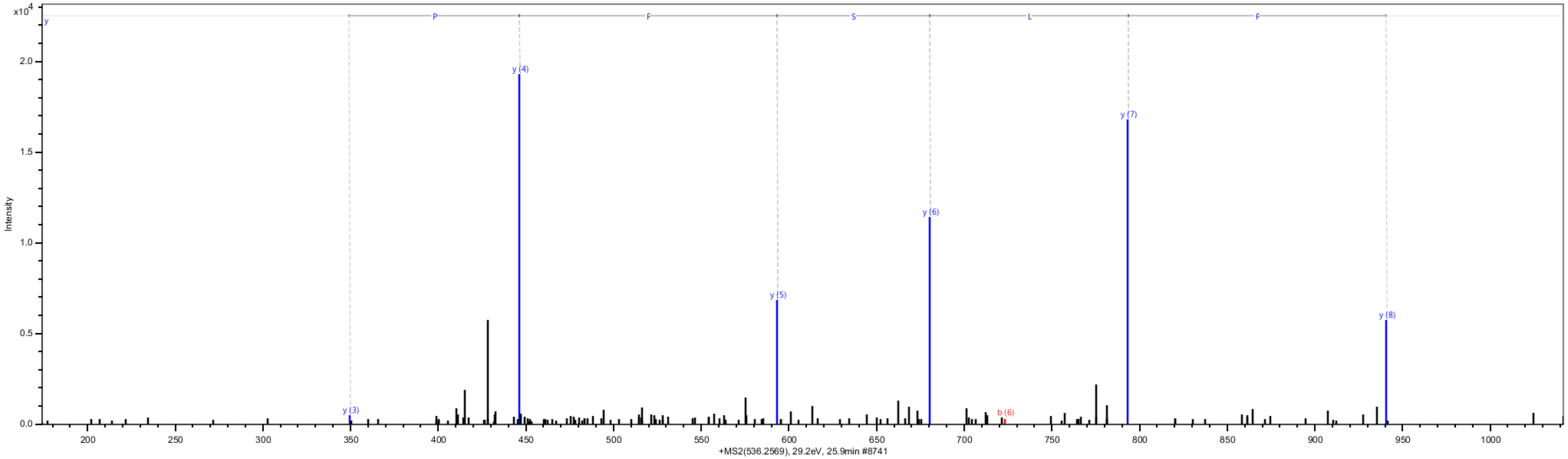

Peptide Match

Sequence: K.TYFPHFDLSHGSAQVK.G **Score:** 82.4

Modifications:

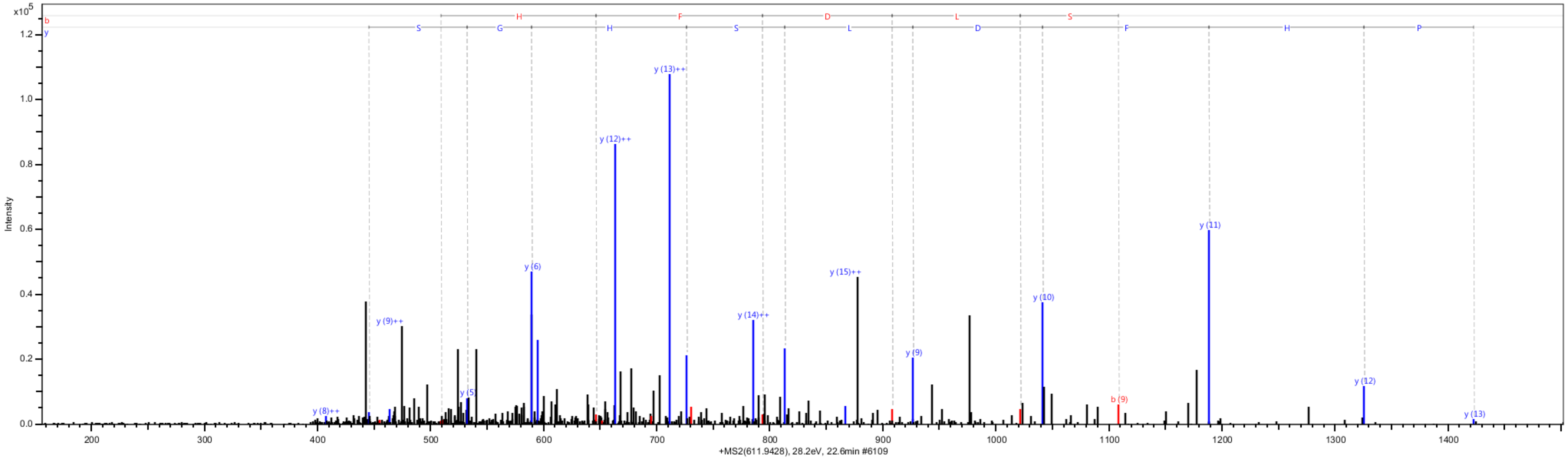

Peptide Match

Sequence: K.LRVDPVNFK.L **Score:** 20.4

Modifications:

Spectrum Report
Combine Search_2020-10-19 07:53:31

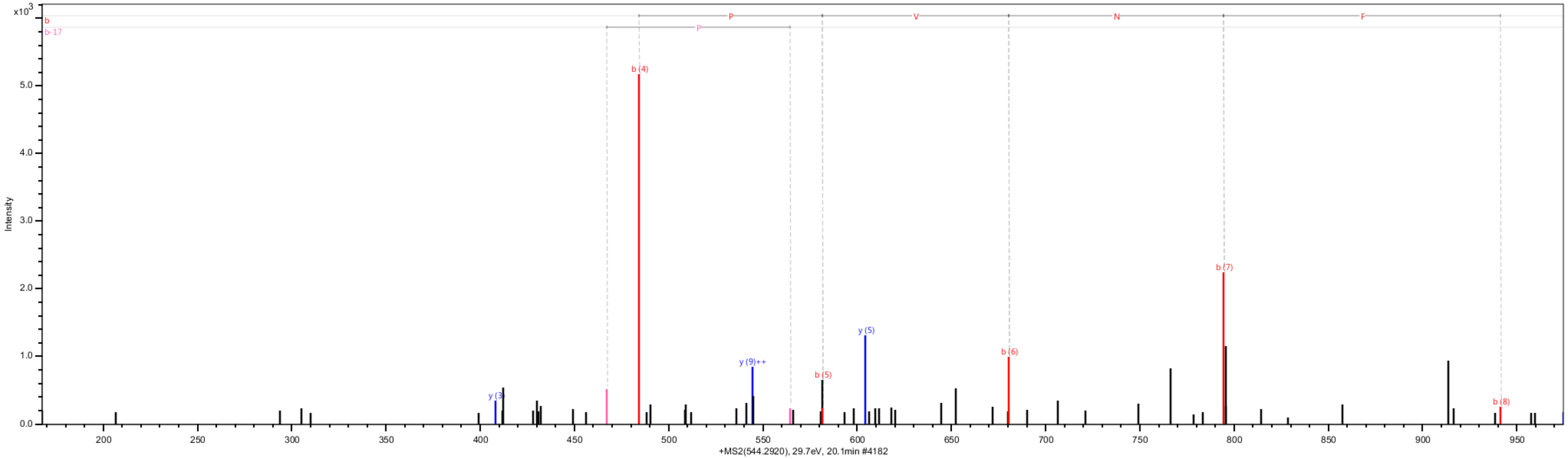

Peptide Match

Sequence: R.VDPVNFK.L **Score:** 18

Modifications:

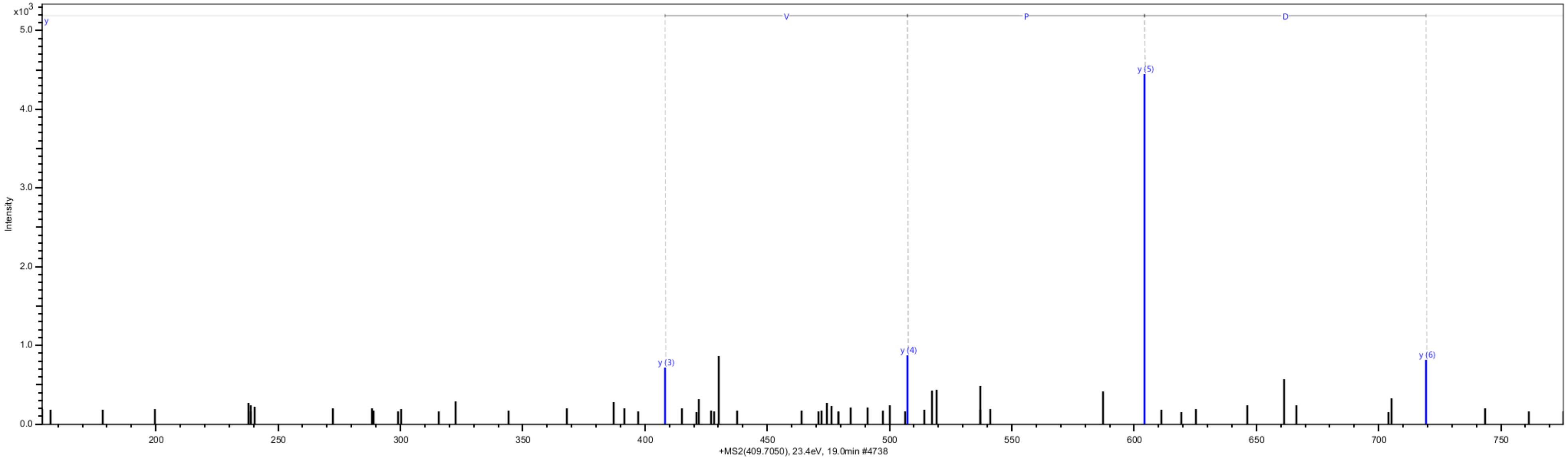

Peptide Match

Sequence: K.FLASVSTVLTSK.Y **Score:** 16.9

Modifications:

Spectrum Report
Combine Search_2020-10-19 07:53:31

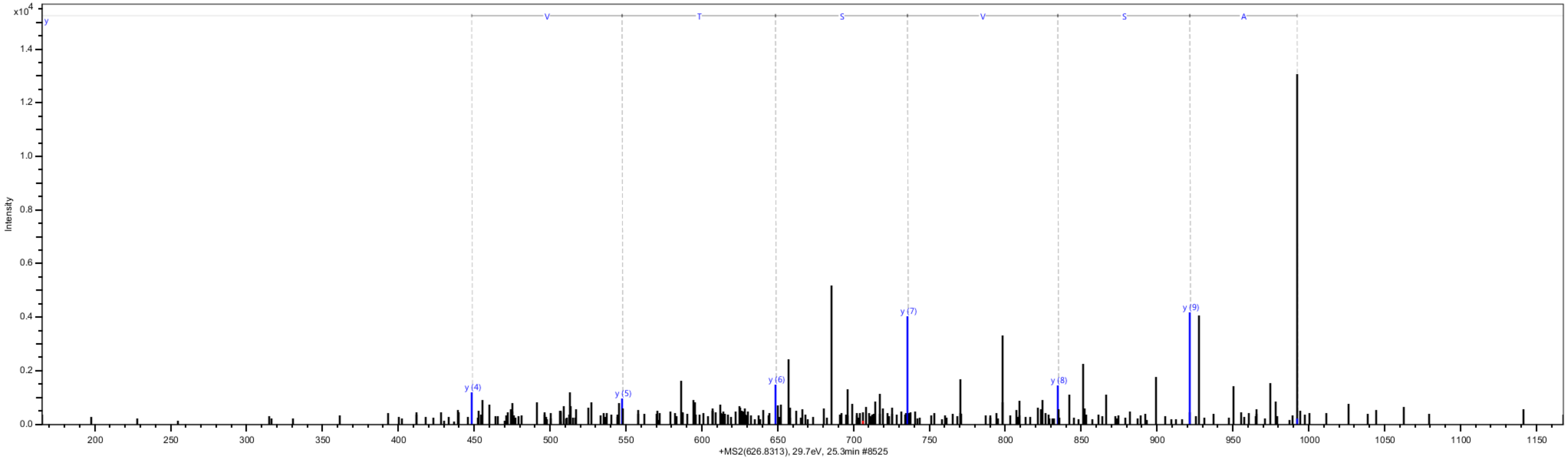

VTDB_HUMAN

Accession: VTDB_HUMAN
Database: SwissProt
MW [kDa] / pI: 52.9 / 5.3
Score: 299.5
Seq. Coverage [%]: 22.2
No. of Peptides: 10

Peptide Match

Sequence: K.EDFTSLSLVLYSR.K **Score:** 17.3
Modifications:

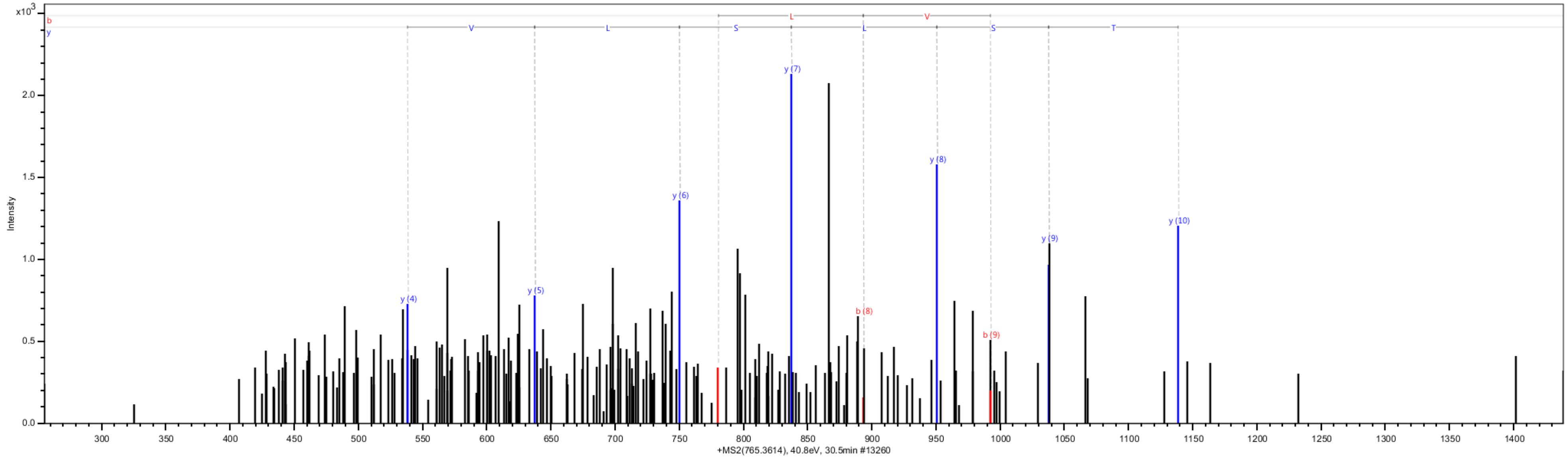

Peptide Match

Sequence: K.SCESNSPFPVHPGTAECCTKEGLER.K **Score:** 16.2
Modifications: Carbamidomethyl: 2, 17, 18

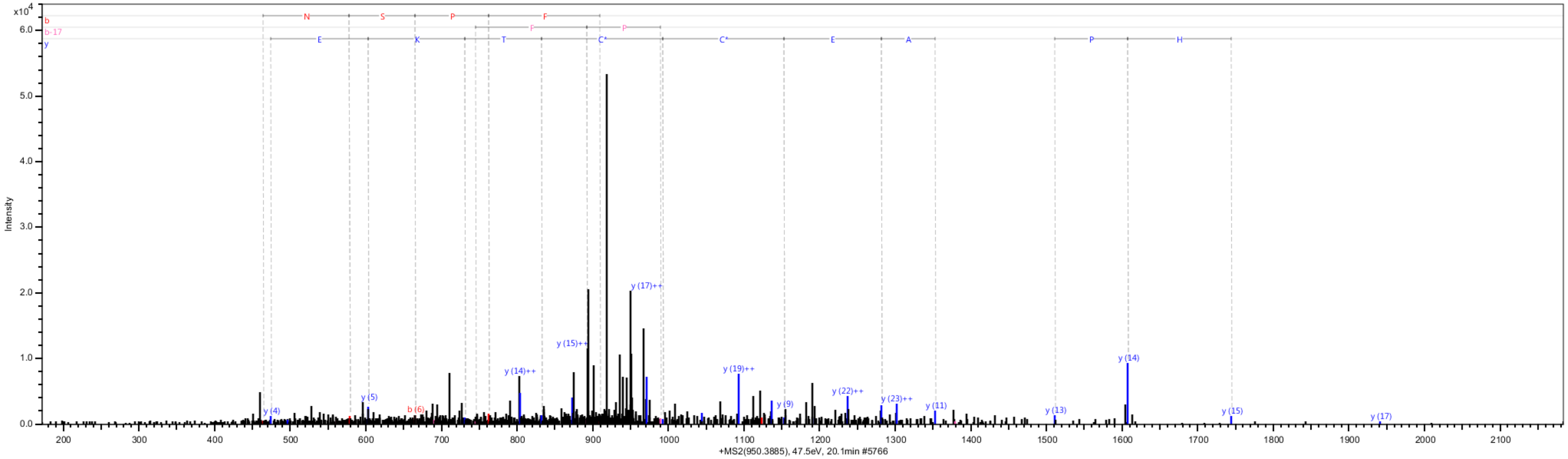

Peptide Match

Sequence: K.HLSLLTTLSNR.V **Score:** 50.9

Modifications:

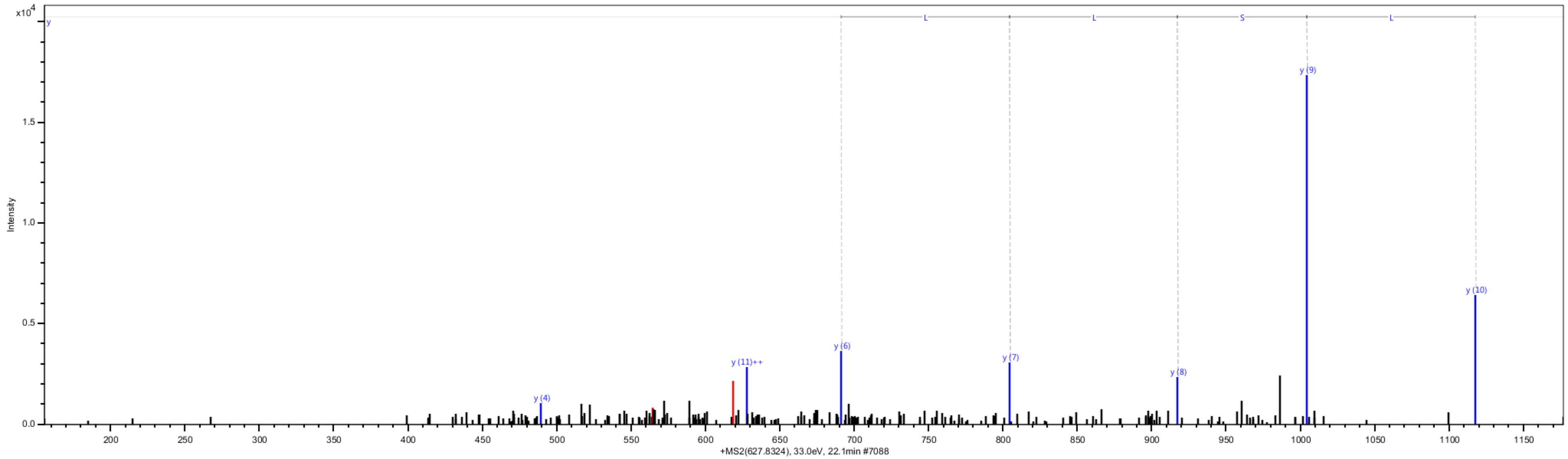

Peptide Match

Sequence: K.YTFELSR.R **Score:** 18.8

Modifications:

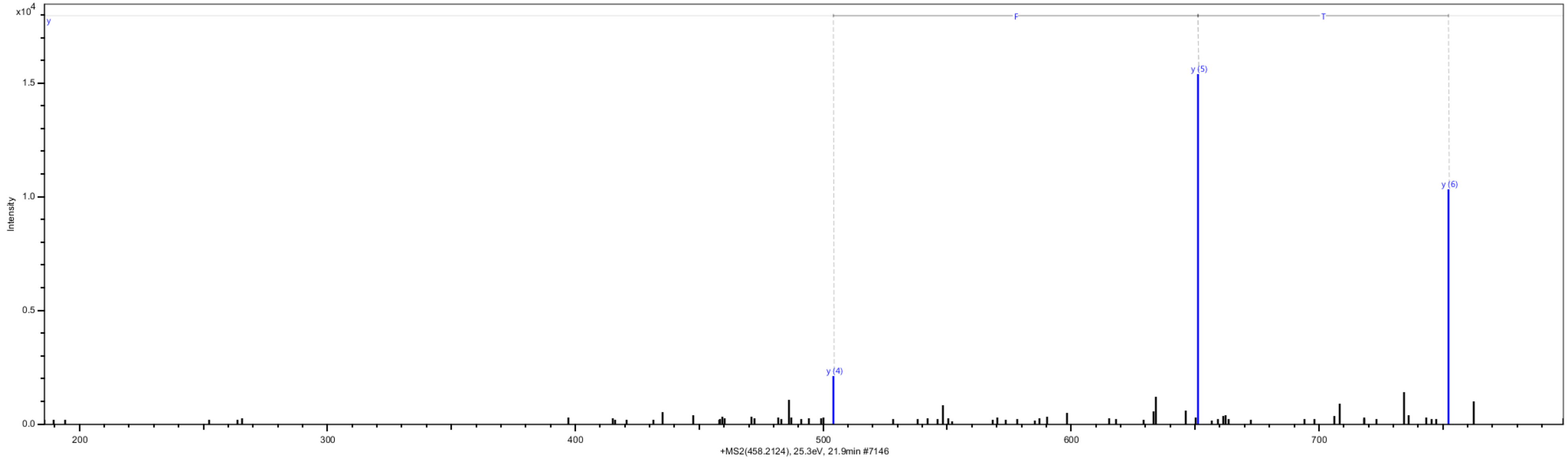

Peptide Match

Sequence: R.RTHLPEVFLSK.V **Score:** 33.5

Modifications:

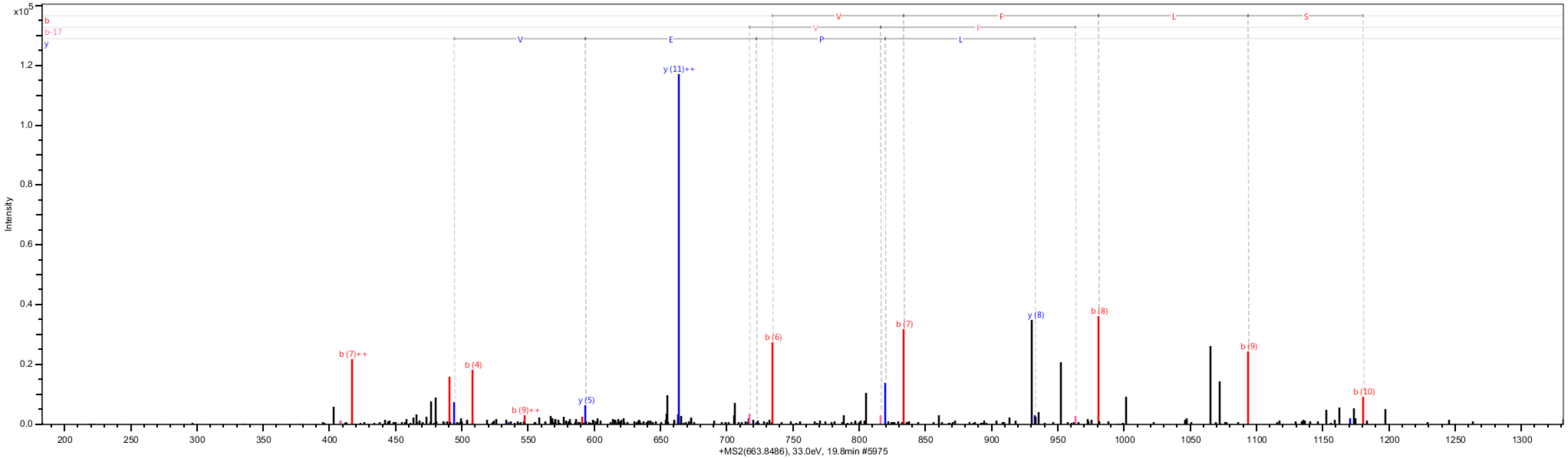

Peptide Match

Sequence: R.THLPEVFLSK.V **Score:** 26.2

Modifications:

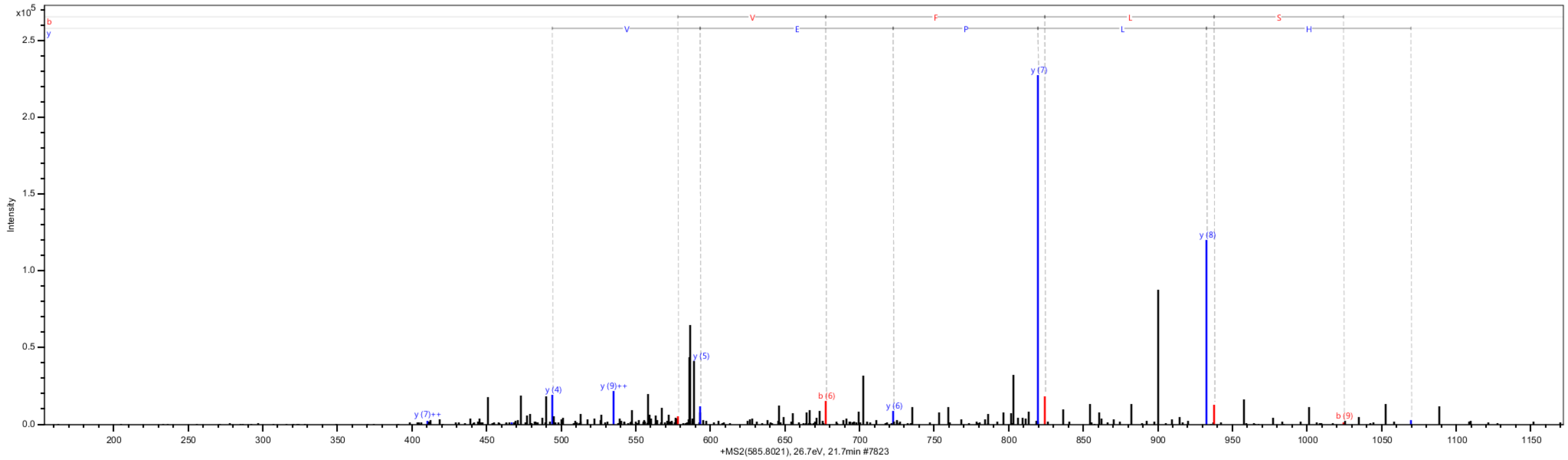

Peptide Match

Sequence: K.VLEPTLK.S **Score:** 29.8

Modifications:

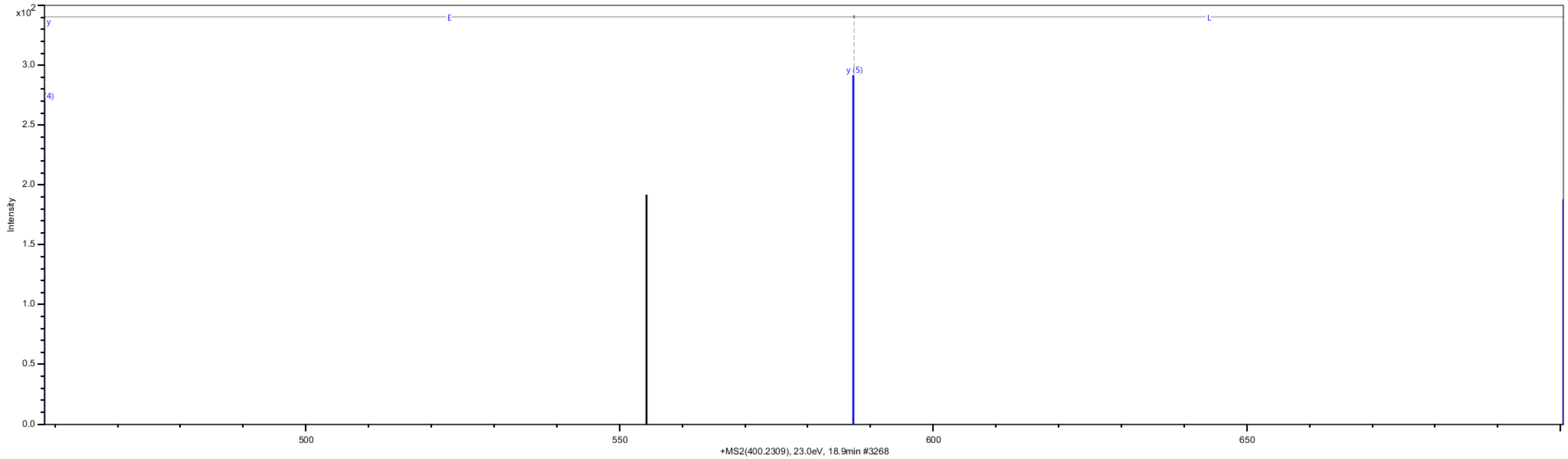

Peptide Match

**Sequence:**	K.SLGECCDVEDSTTCFNAK.G	**Score:**	42.4
**Modifications:**	Carbamidomethyl: 5, 6, 14		

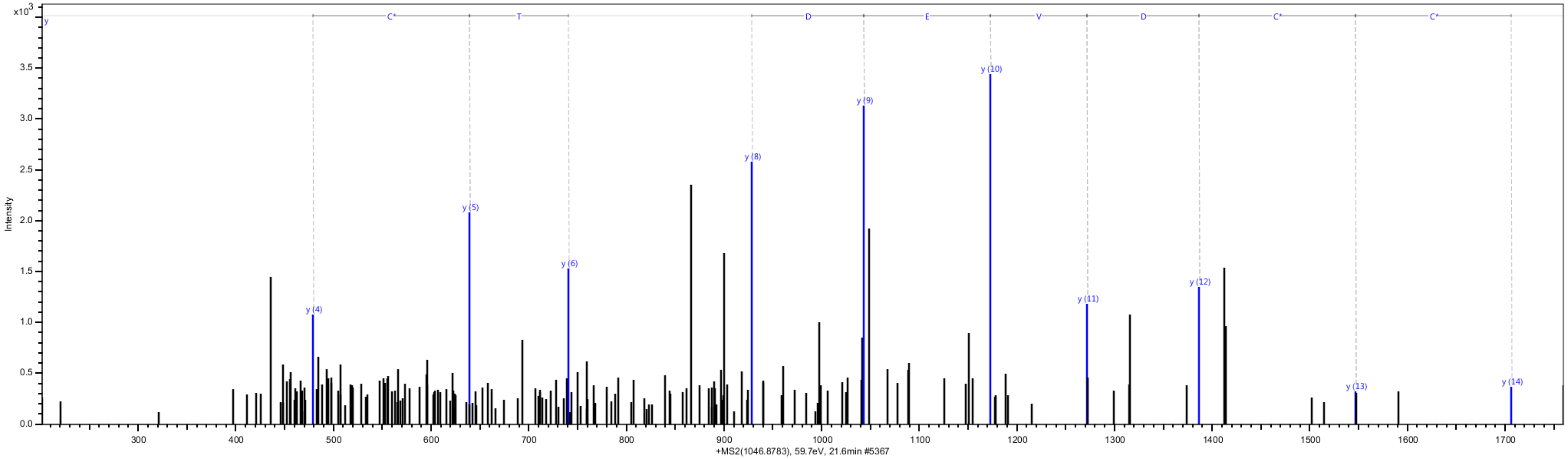

Peptide Match

Sequence: K.AKLPDATPTELAK.L **Score:** 24.1

Modifications:

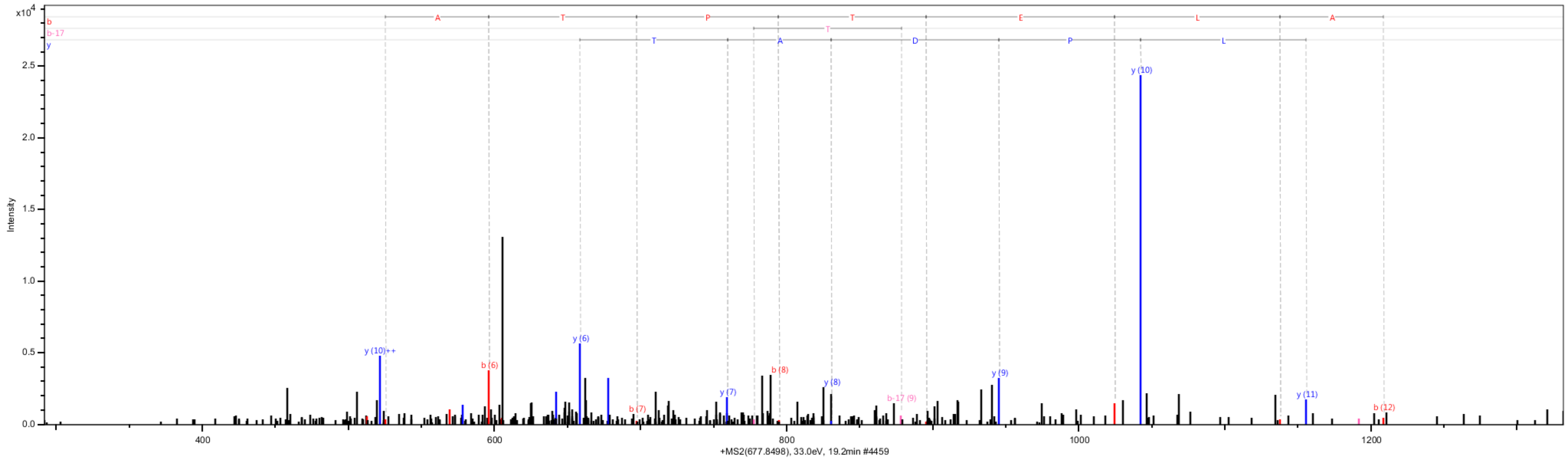

Peptide Match

Sequence: K.LPDATPTELAK.L **Score:** 40.4

Modifications:

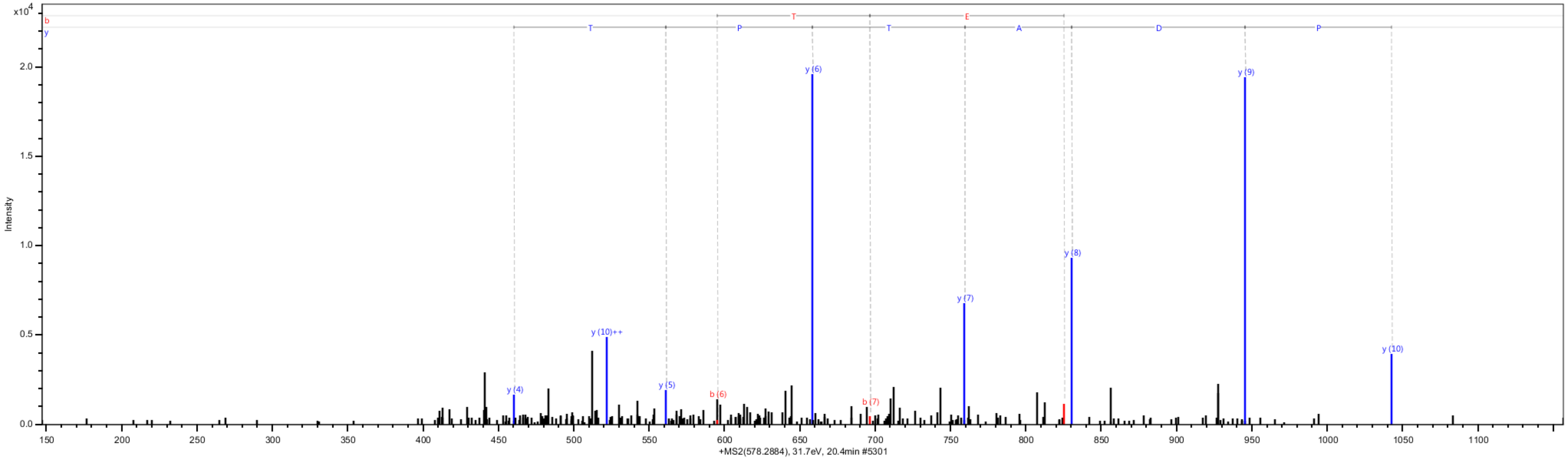
x10^4
Intensity
y (6)
y (9)
y (8)
y (7)
y (10)++
y (10)
y (4)
y (5)
b (6)
b (7)
+MS2(578.2884), 31.7eV, 20.4min #5301

A1AG1_HUMAN

Accession: A1AG1_HUMAN
Database: SwissProt
MW [kDa] / pI: 23.5 / 4.9
Score: 302.9
Seq. Coverage [%]: 27.9
No. of Peptides: 6

Peptide Match

Sequence: K.WFYIASAFR.N
Score: 41.6
Modifications:

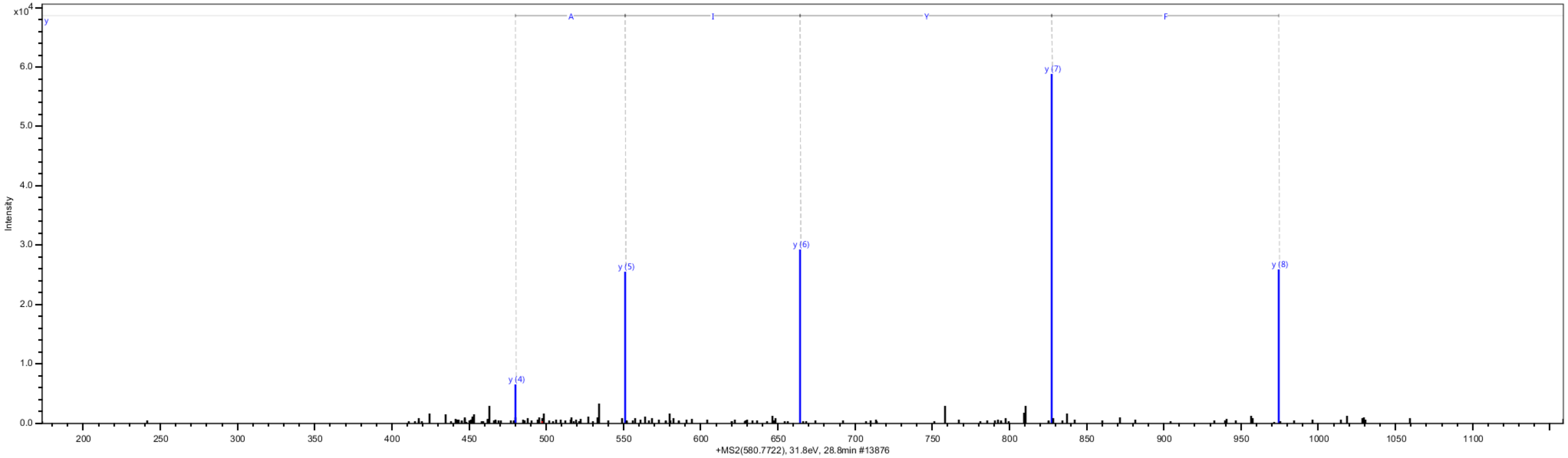

Peptide Match

Sequence: K.TEDTIFLR.E
Score: 23.3
Modifications:

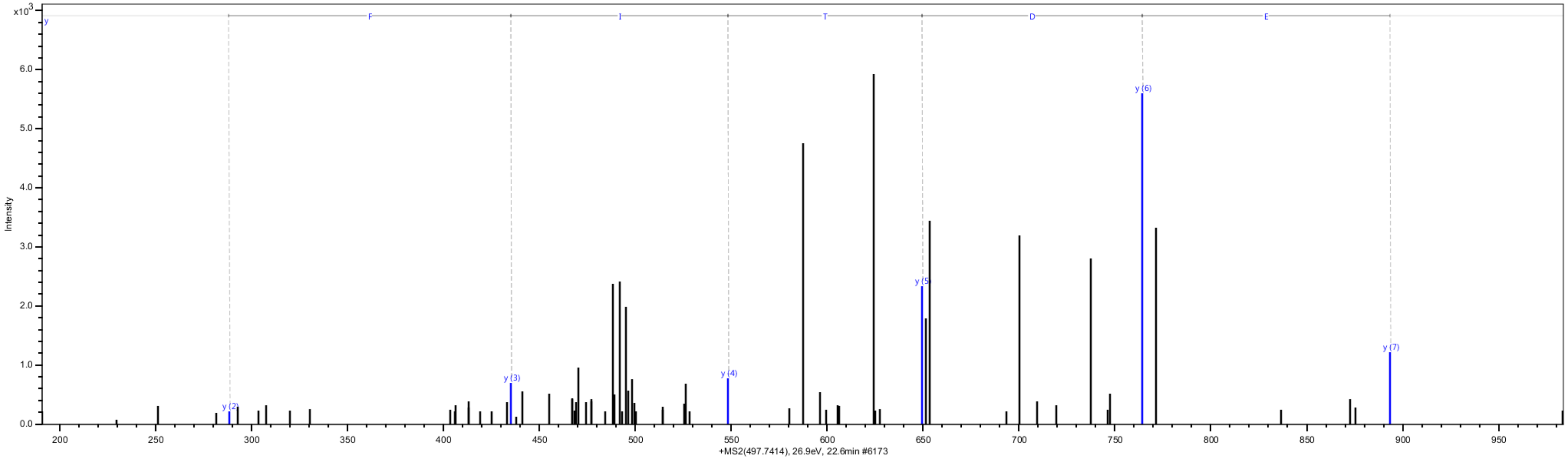

Peptide Match

Sequence: R.YVGGQEHFAHLLILR.D **Score:** 102

Modifications:

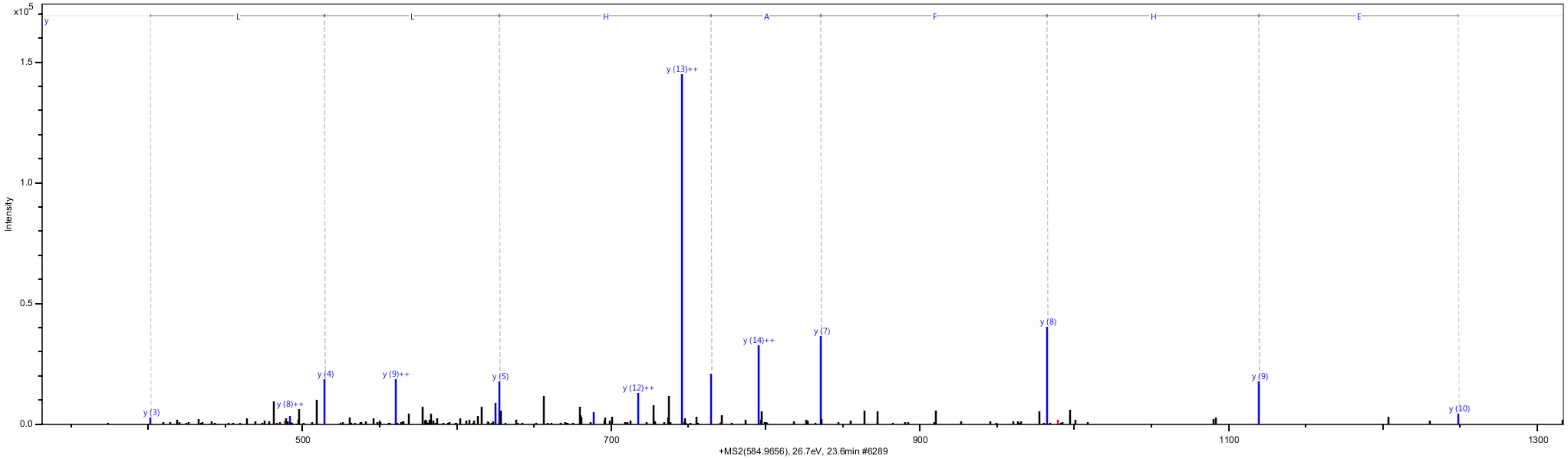

Peptide Match

**Sequence:**	K.EQLGEFYEALDCLR.I	**Score:**	40.6
**Modifications:**	Carbamidomethyl: 12		

Spectrum Report
Combine Search_2020-10-19 07:53:31

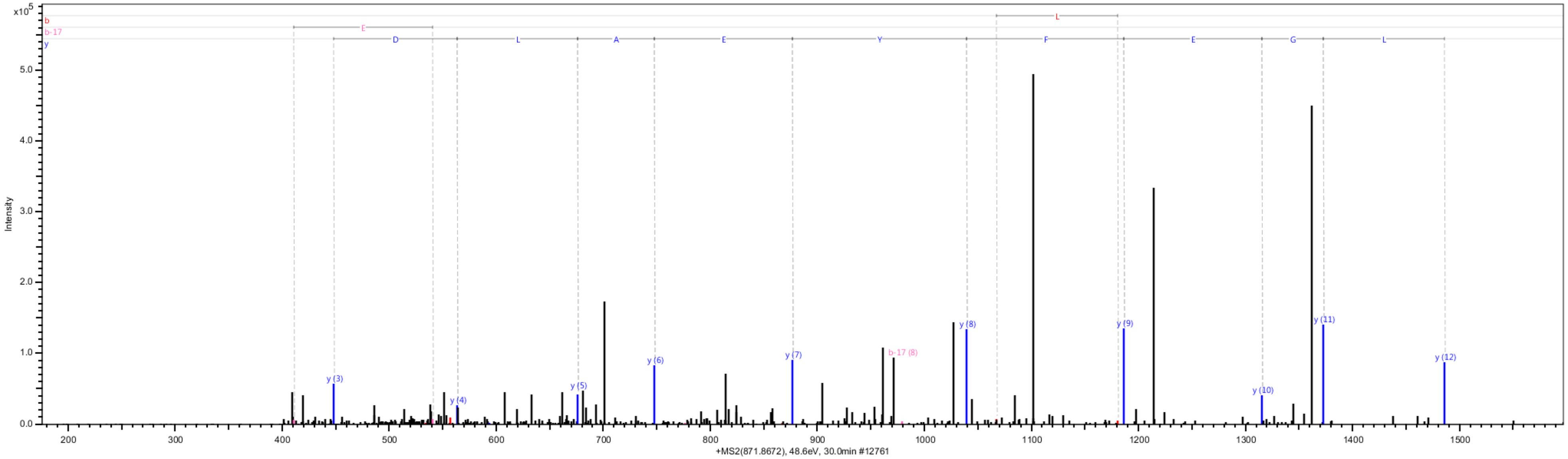

Peptide Match

Sequence: K.SDVVYTDWK.K **Score:** 35.7

Modifications:

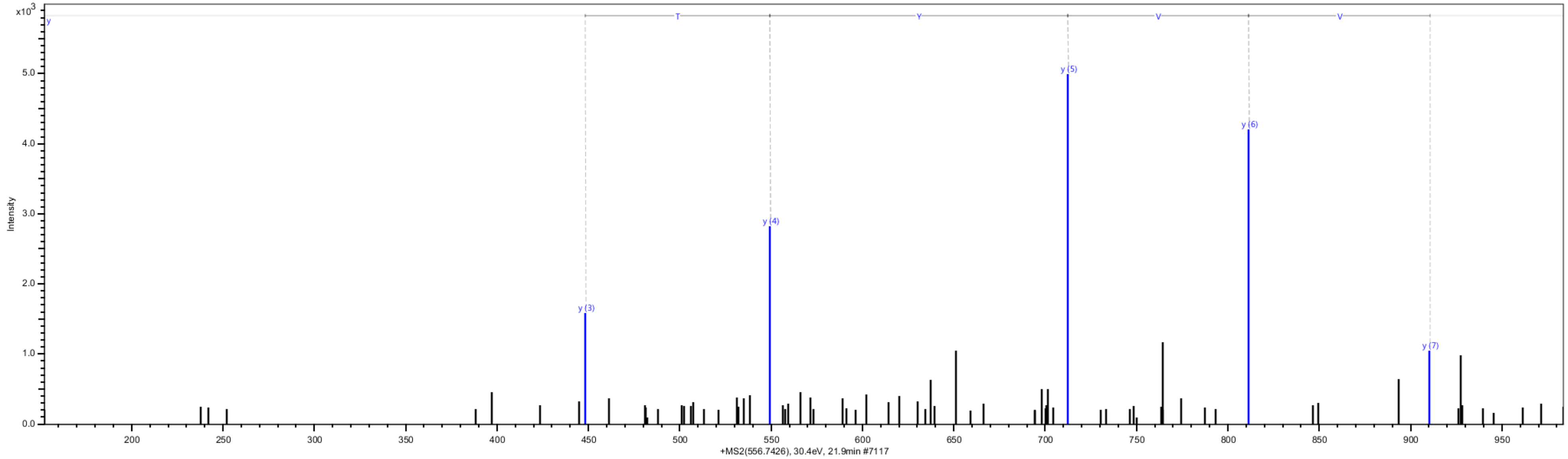

Peptide Match

Sequence: K.SDVVYTDWKK.D **Score:** 59.7

Modifications:

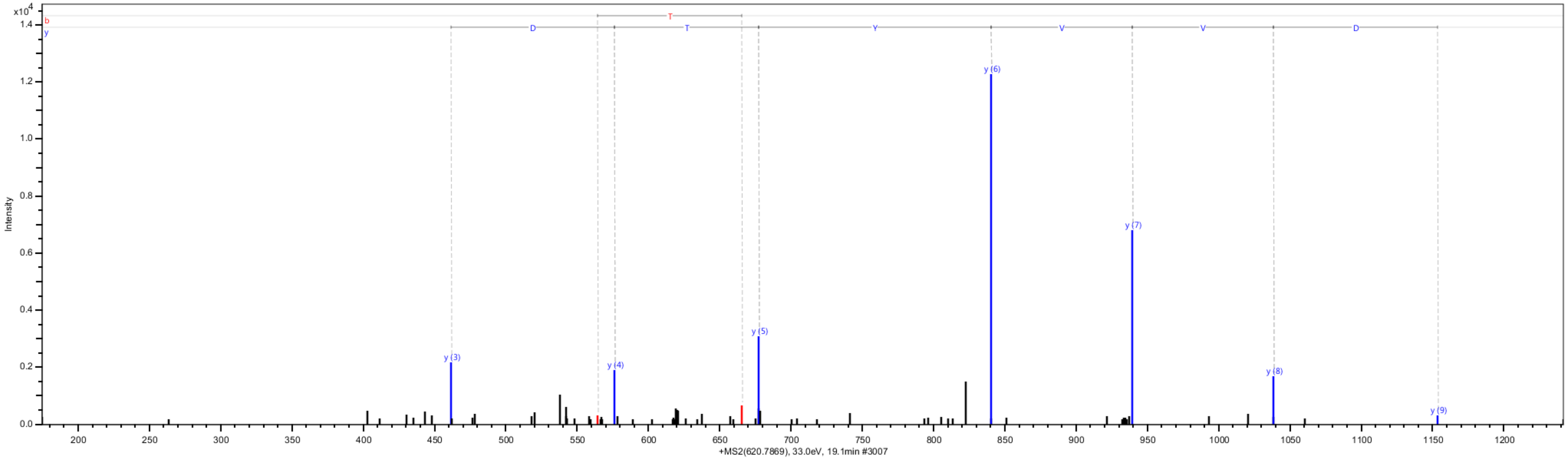
x10^4
Intensity
b
y
D
T
T
Y
V
V
D
y (3)
y (4)
y (5)
y (6)
y (7)
y (8)
y (9)
+MS2(620.7869), 33.0eV, 19.1min #3007

HPTR_HUMAN

Accession: HPTR_HUMAN
Database: SwissProt
MW [kDa] / pI: 39.0 / 6.6
Score: 271.5
Seq. Coverage [%]: 4.0
No. of Peptides: 1

Peptide Match

Sequence: M.SDLGAVISLLLWGR.Q
Score: 45.4
Modifications:

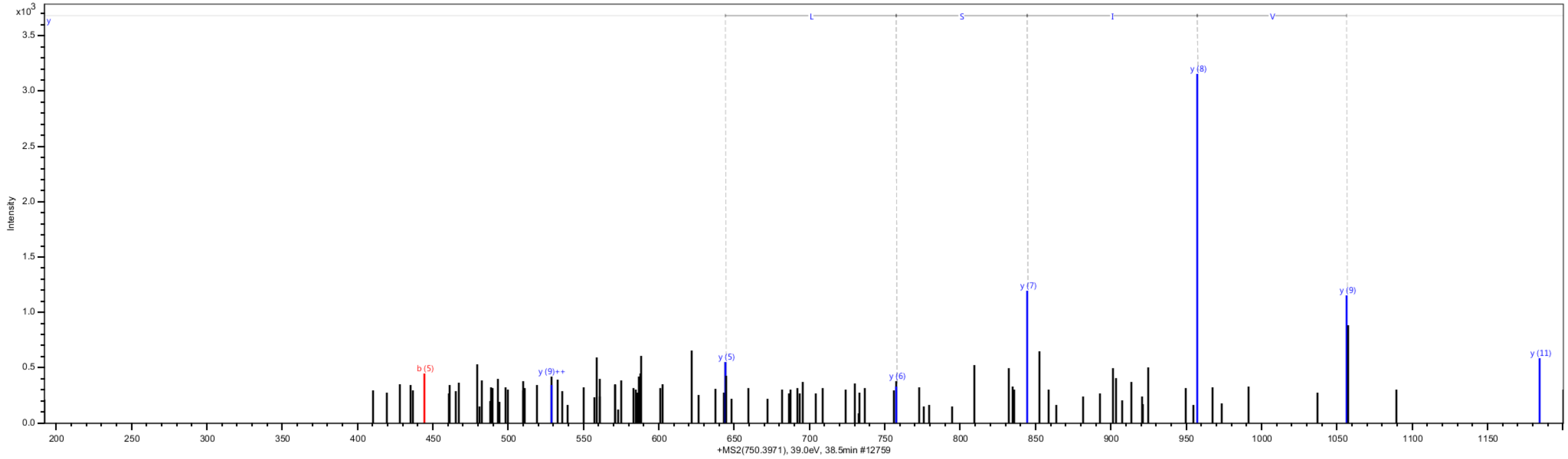

CERU_HUMAN

Accession: CERU_HUMAN
Database: SwissProt
MW [kDa] / pI: 122.1 / 5.4
Score: 277.1
Seq. Coverage [%]: 7.6
No. of Peptides: 7

Peptide Match

Sequence: K.DIASGLIGPLIICK.K
Modifications: Carbamidomethyl: 13
Score: 59.2

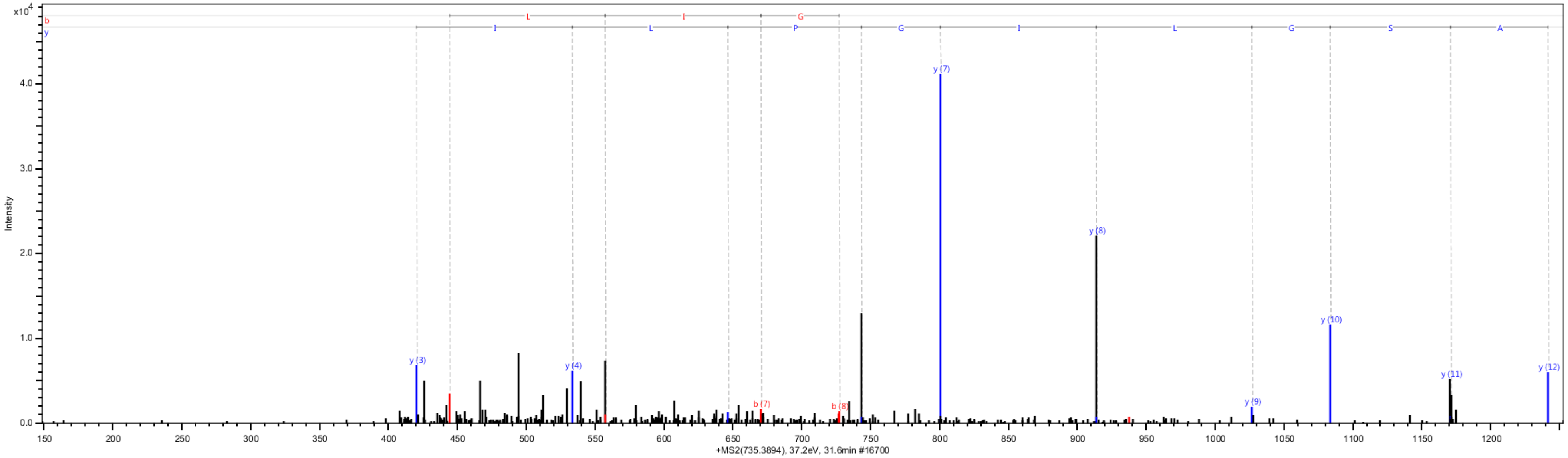

Peptide Match

Sequence: K.DIASGLIGPLIICKK.D
Modifications: Carbamidomethyl: 13
Score: 28.6

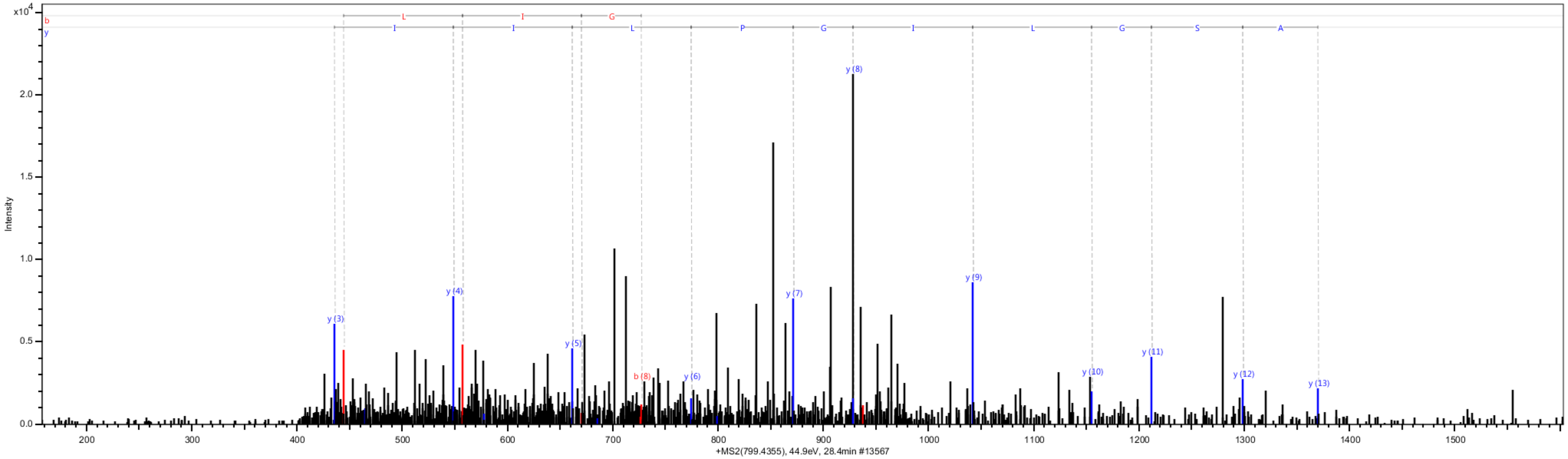

Peptide Match

Sequence: K.GAYPLSIEPIGVR.F **Score:** 51.8

Modifications:

Spectrum Report
Combine Search_2020-10-19 07:53:31

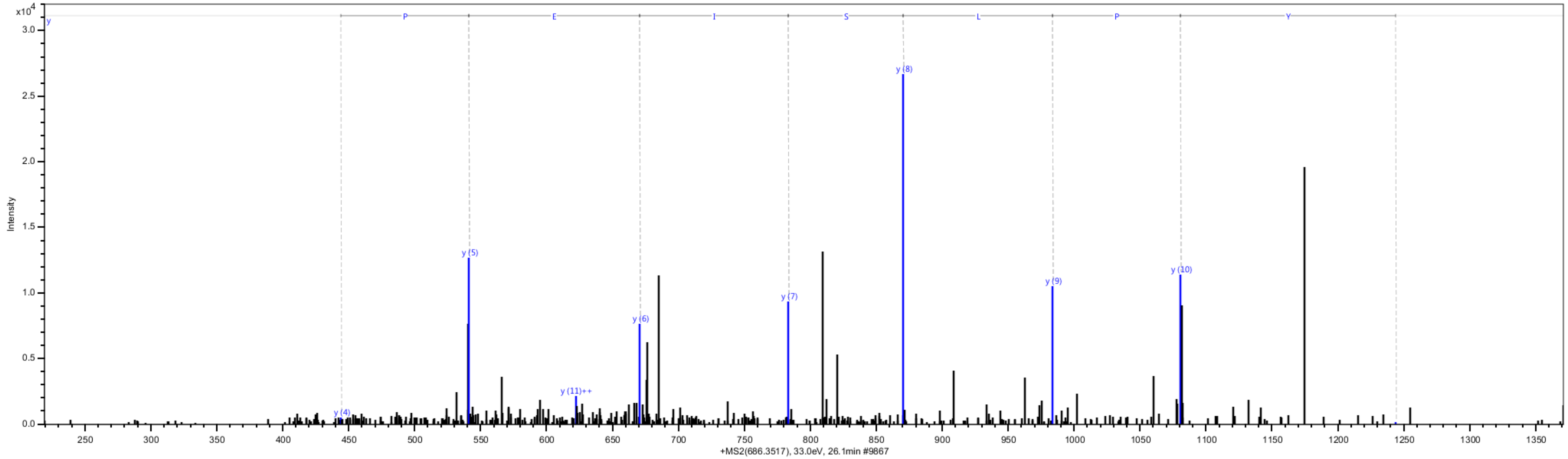

Peptide Match

Sequence: K.EVGPTNADPVCLAK.M **Score:** 26.7

Modifications: Carbamidomethyl: 11

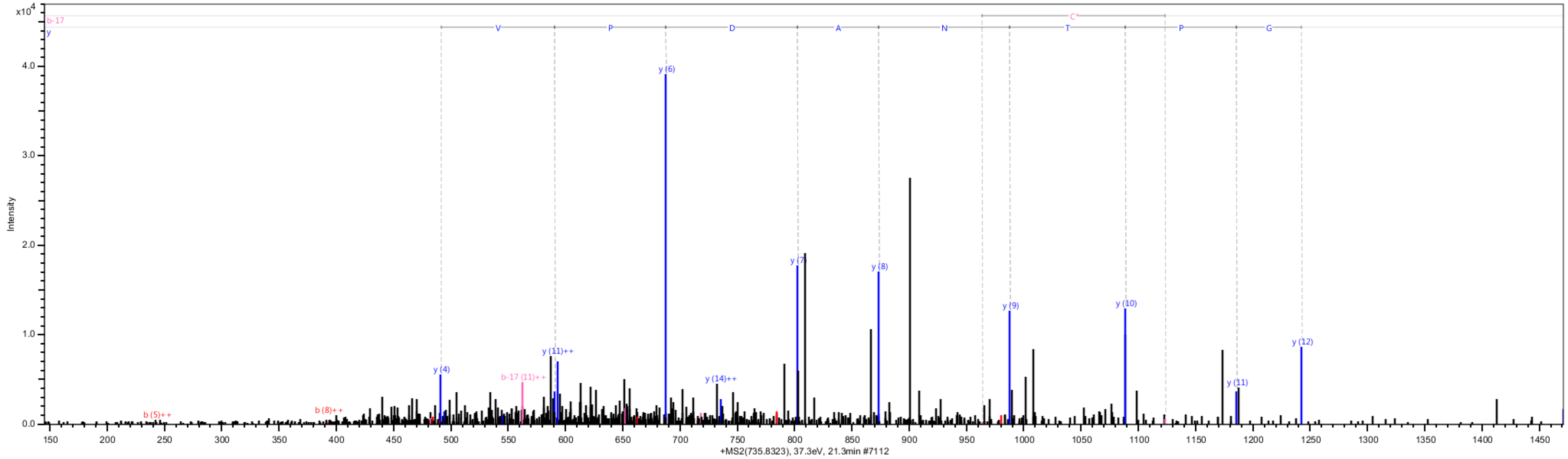

Peptide Match

Sequence: R.QSEDSTFYLGER.T **Score:** 17.2

Modifications:

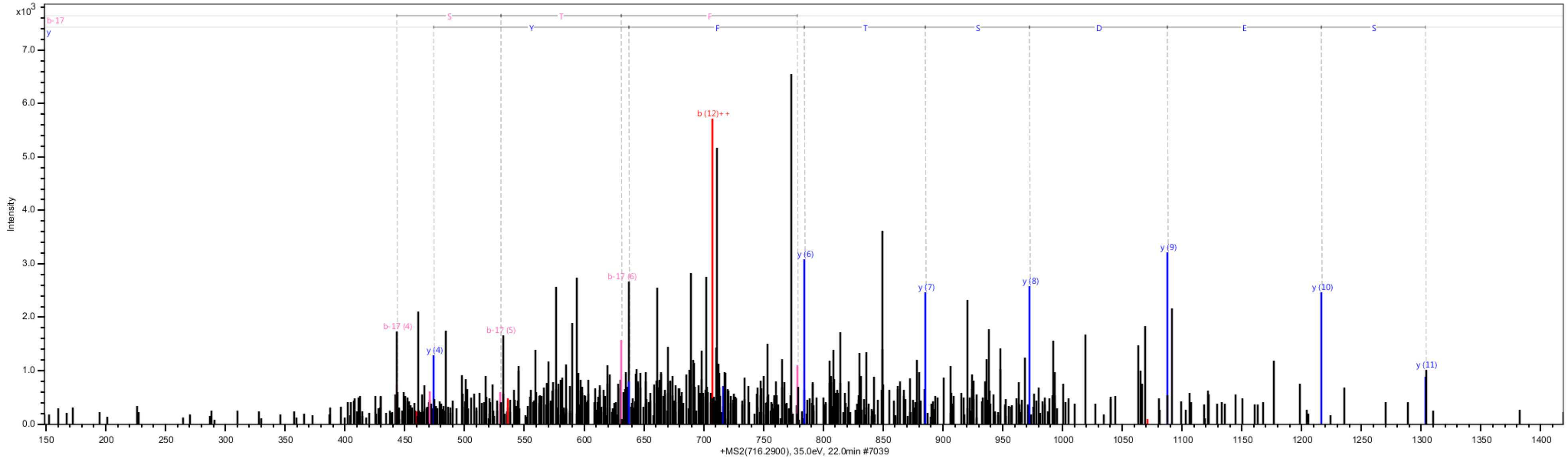

Peptide Match

Sequence: R.QYTDSTFRVPVER.K **Score:** 44.1

Modifications:

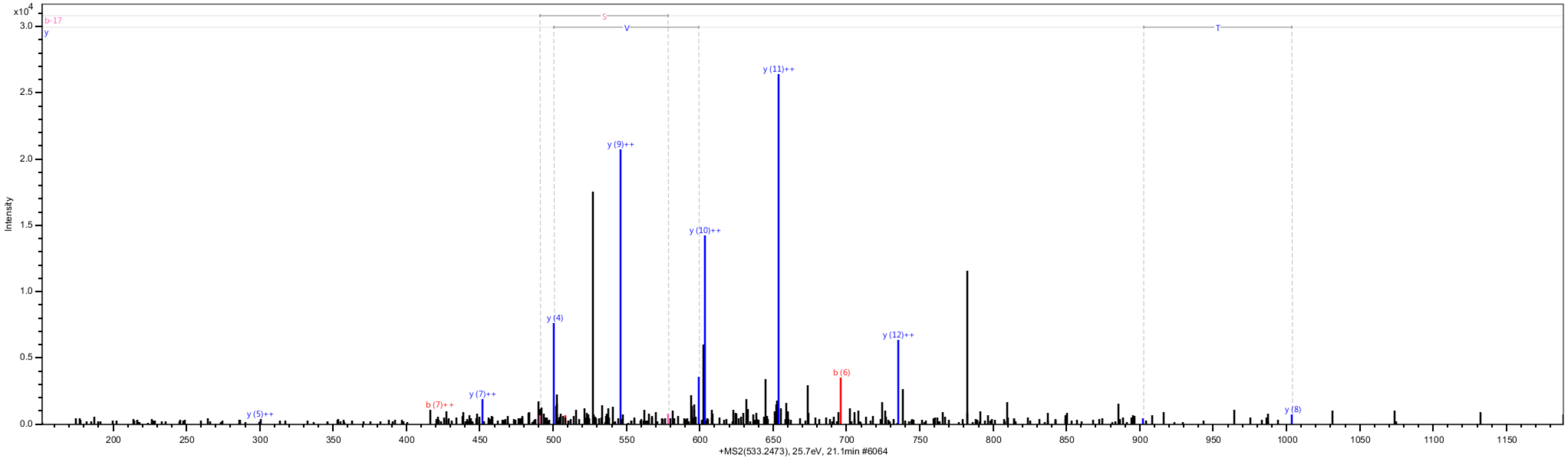

Peptide Match

**Sequence:**	K.DLYSGLIGPLIVCR.R	**Score:**	49.6
**Modifications:**	Carbamidomethyl: 13		

Spectrum Report
Combine Search_2020-10-19 07:53:31

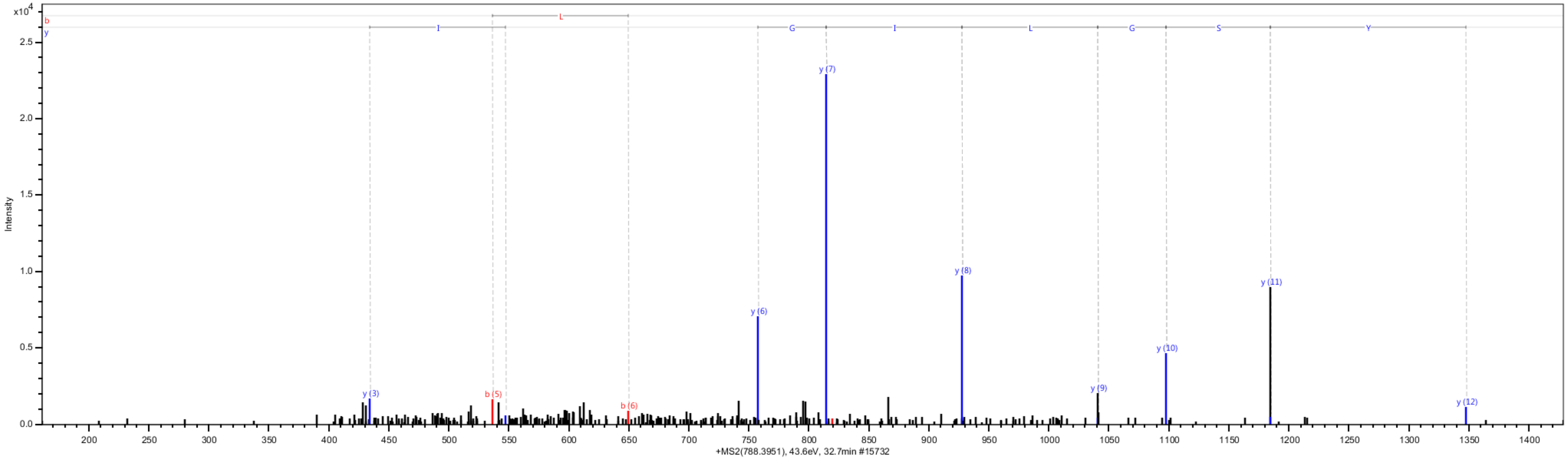

CFAB_HUMAN

Accession: CFAB_HUMAN
Database: SwissProt
MW [kDa] / pI: 85.5 / 6.7
Score: 259.8
Seq. Coverage [%]: 7.6
No. of Peptides: 5

Peptide Match

Sequence: K.CLVNLIEK.V
Score: 23.2
Modifications: Carbamidomethyl: 1

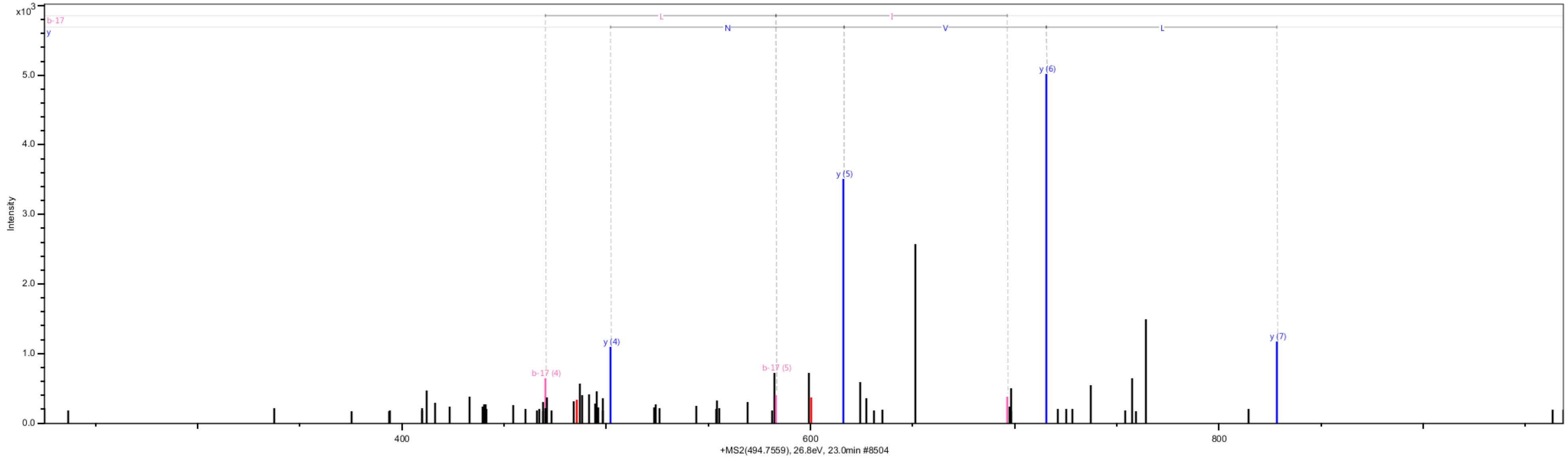

Peptide Match

Sequence: R.YGLVTYATYPK.I
Score: 54
Modifications:

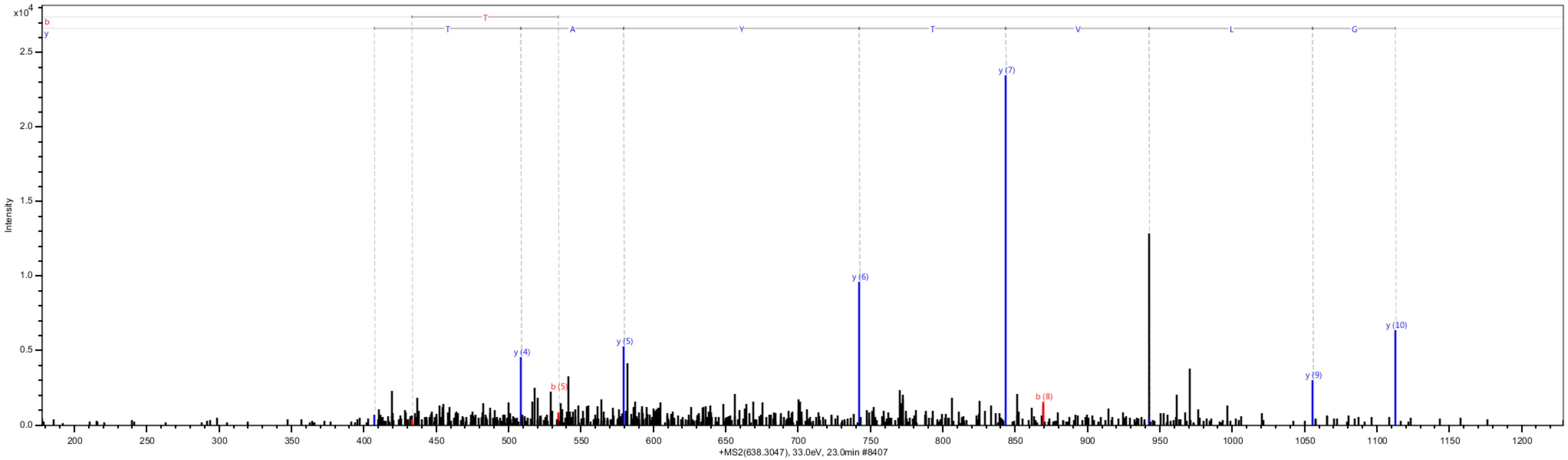

Peptide Match

**Sequence:**	K.VSEADSSNADWVTK.Q	**Score:**	88.8
**Modifications:**			

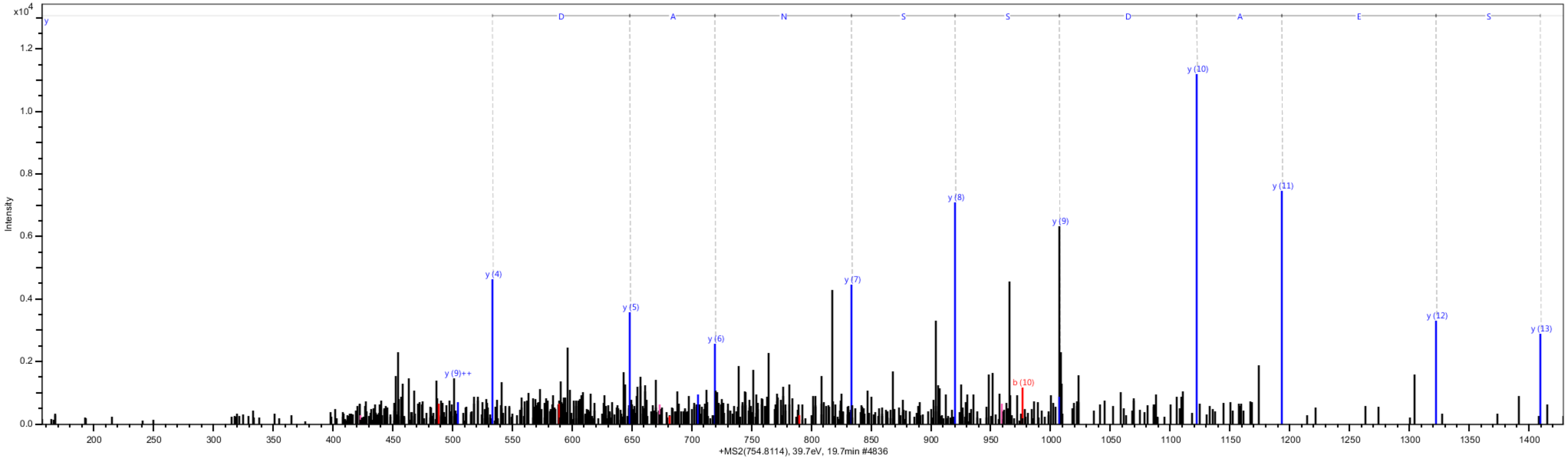

Peptide Match

Sequence: K.VKDISEVVTPR.F **Score:** 40.2

Modifications:

Spectrum Report
Combine Search_2020-10-19 07:53:31

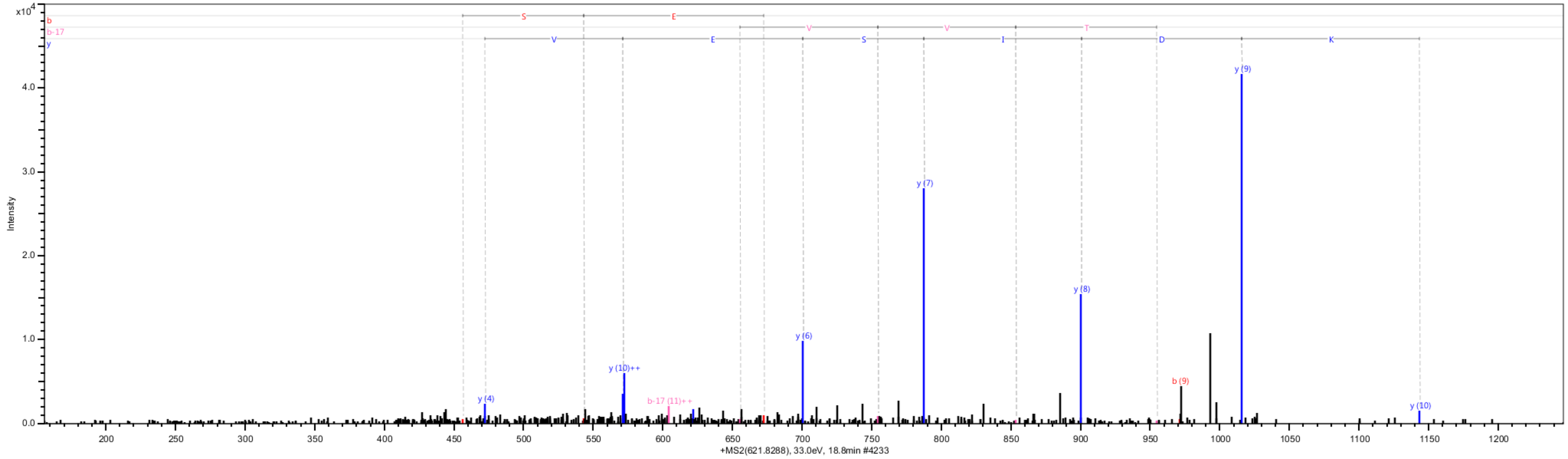

Peptide Match

Sequence: R.DFHINLFQVLPWLK.E **Score:** 53.7
Modifications:

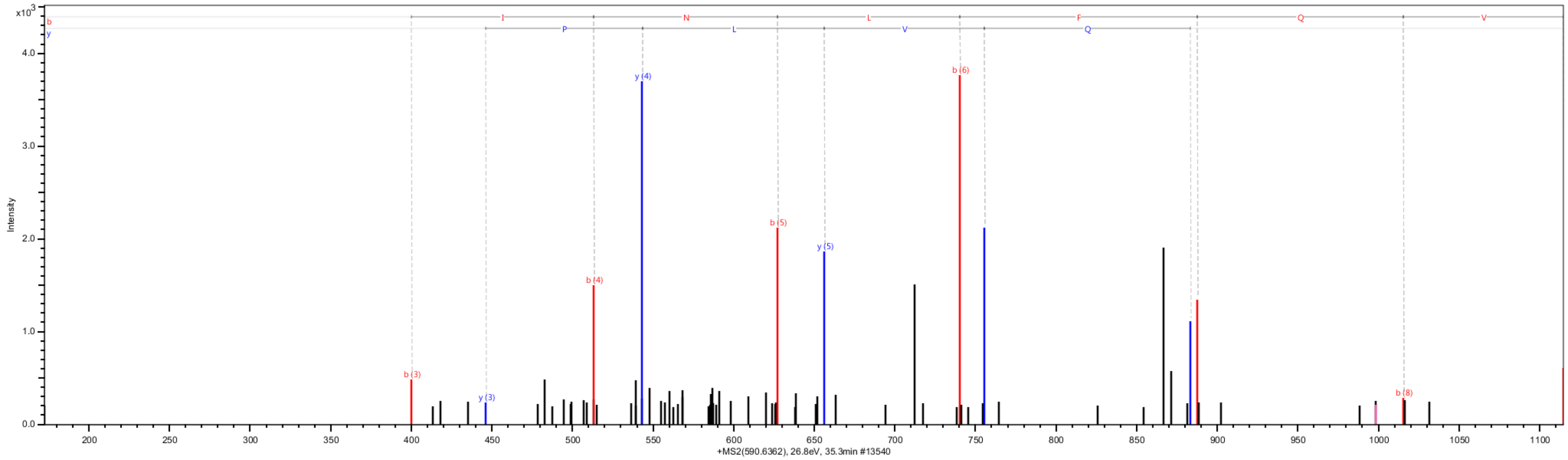
x10^3
b
y
I
N
L
F
Q
V
P
L
V
Q
Intensity
b (3)
y (3)
b (4)
y (4)
b (5)
y (5)
b (6)
b (8)
+MS2(590.6362), 26.8eV, 35.3min #13540

A1BG_HUMAN

Accession: A1BG_HUMAN
Database: SwissProt
MW [kDa] / pI: 54.2 / 5.6
Score: 270.3
Seq. Coverage [%]: 17.8
No. of Peptides: 8

Peptide Match

Sequence: K.HQFLLTGDTQGR.Y
Score: 25
Modifications:

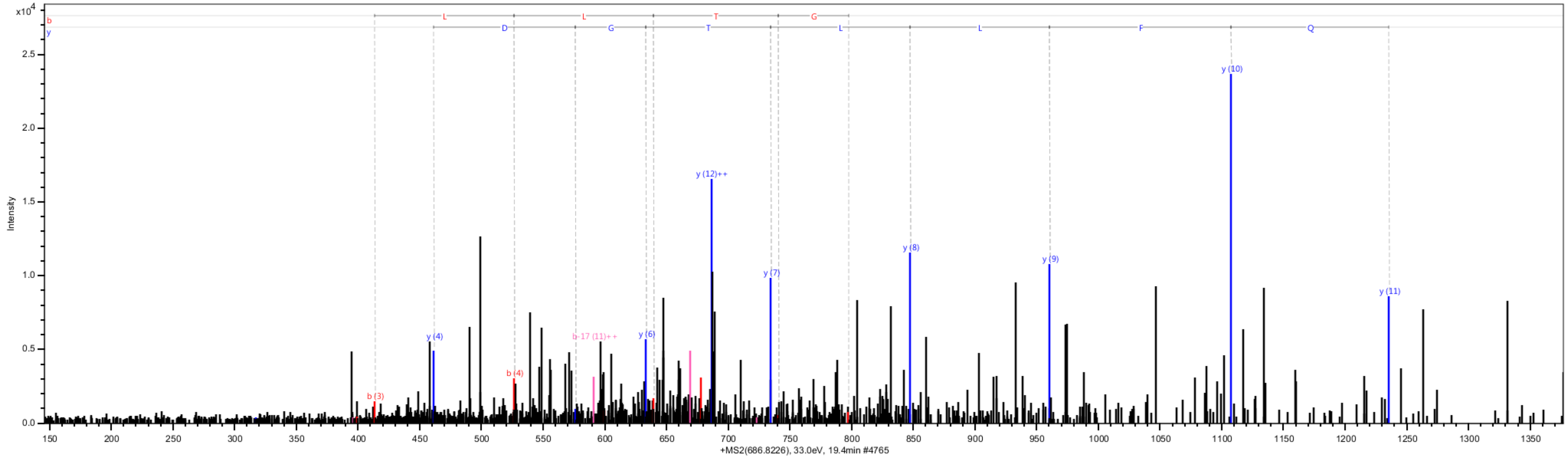

Peptide Match

Sequence: R.SGLSTGWTQLSK.L
Score: 26.1
Modifications:

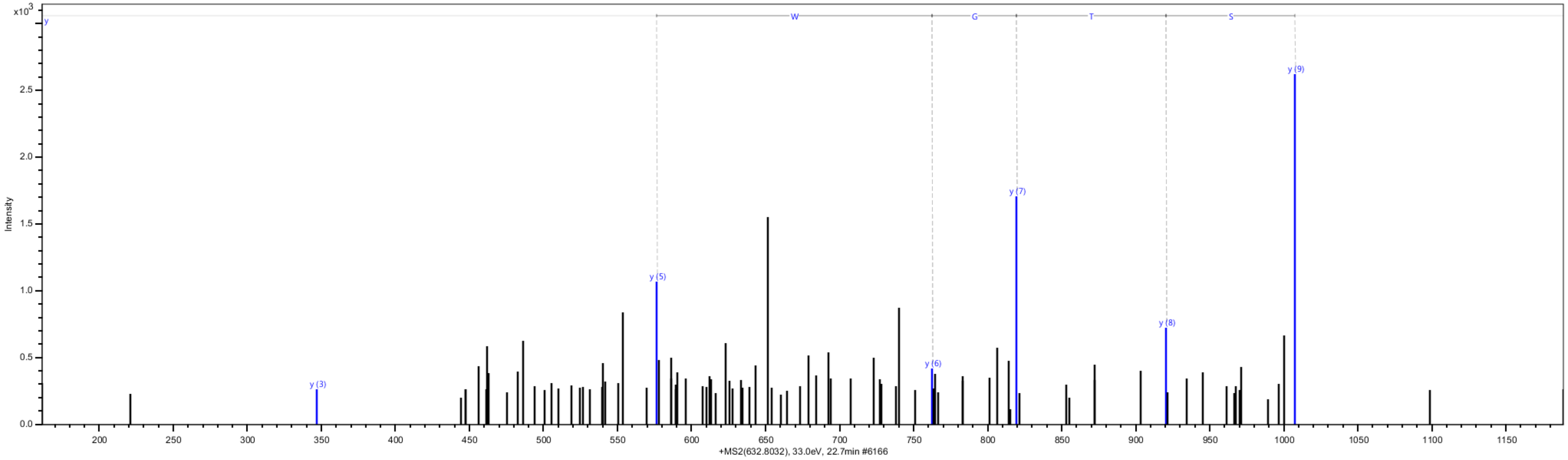

Peptide Match

**Sequence:**	K.LLELTGPK.S	**Score:**	35.6
**Modifications:**			

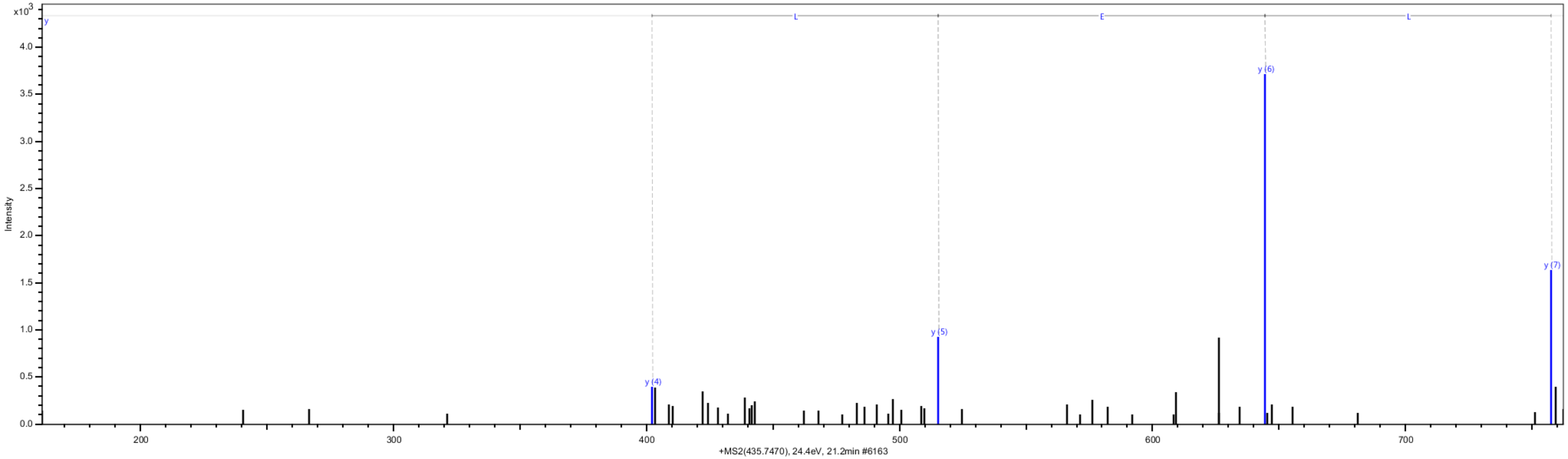

Peptide Match

Sequence: R.GVTFLLR.R **Score:** 24.1

Modifications:

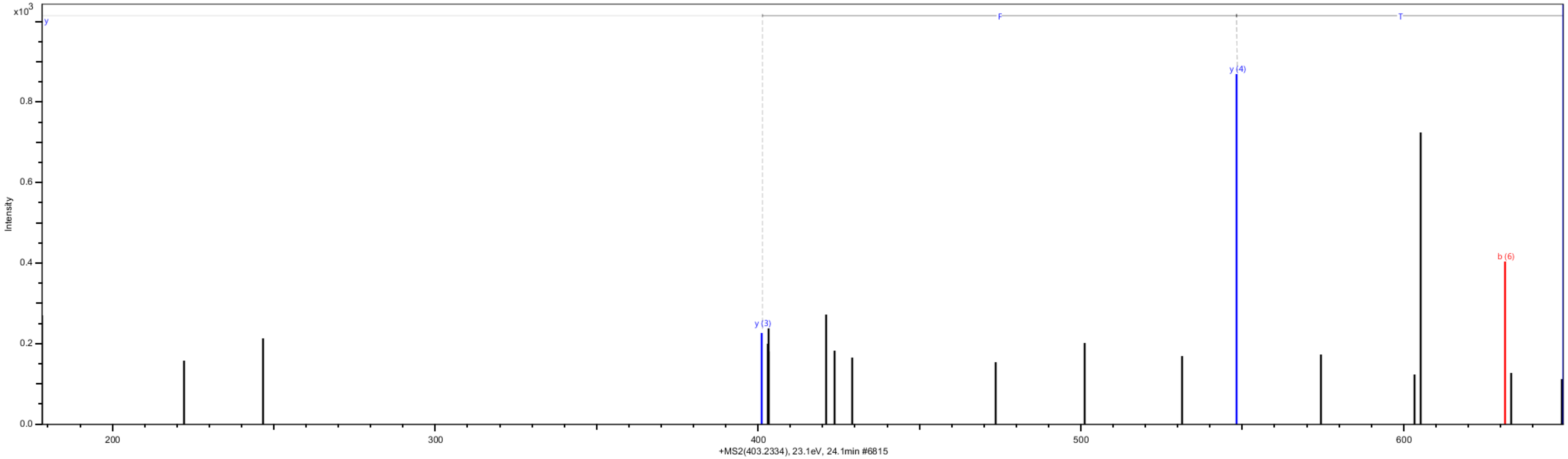

Peptide Match

**Sequence:**	R.CLAPLEGAR.F	**Score:**	30.4
**Modifications:**	Carbamidomethyl: 1		

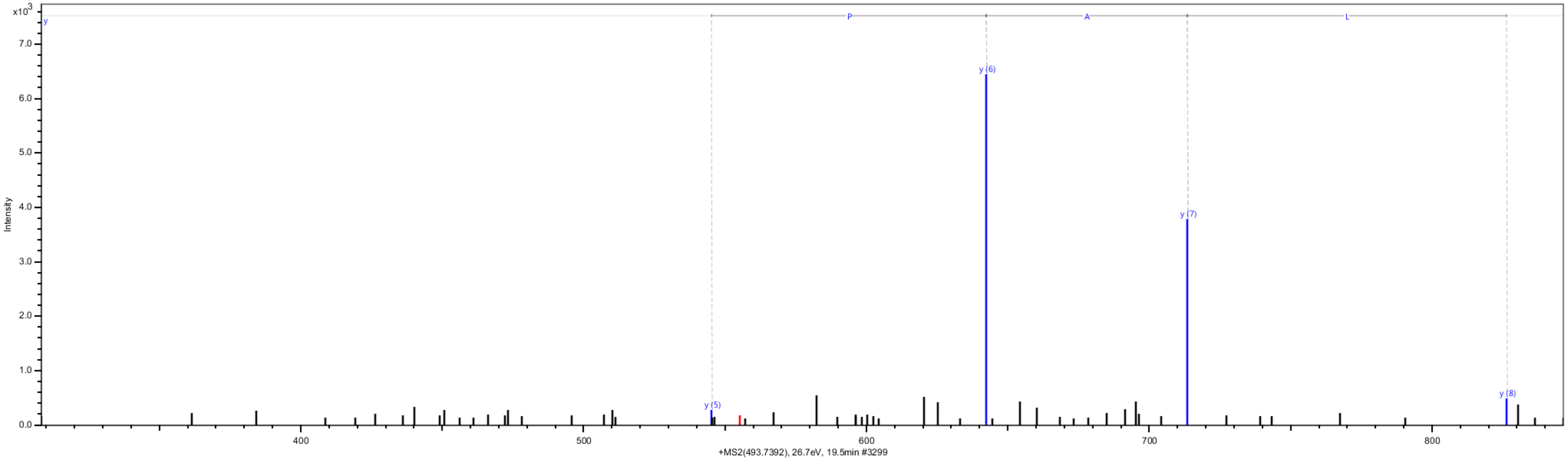

Peptide Match

Sequence: R.LELHVDGPPPRPQLR.A **Score:** 36.6

Modifications:

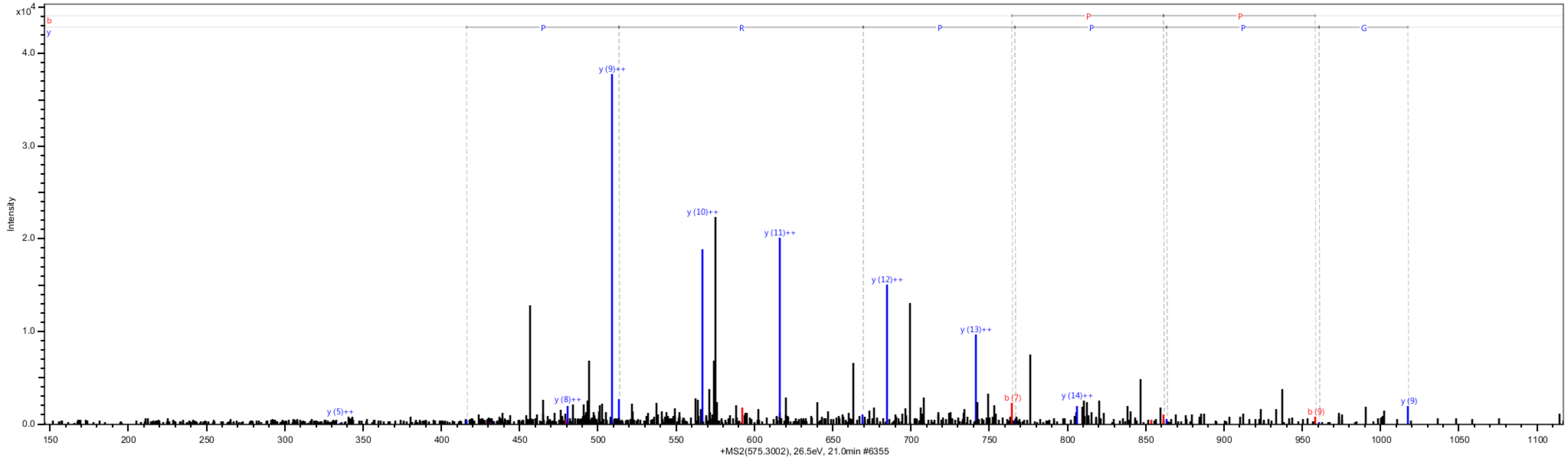

Peptide Match

**Sequence:**	R.ATWSGAVLAGR.D	**Score:**	52.8
**Modifications:**			

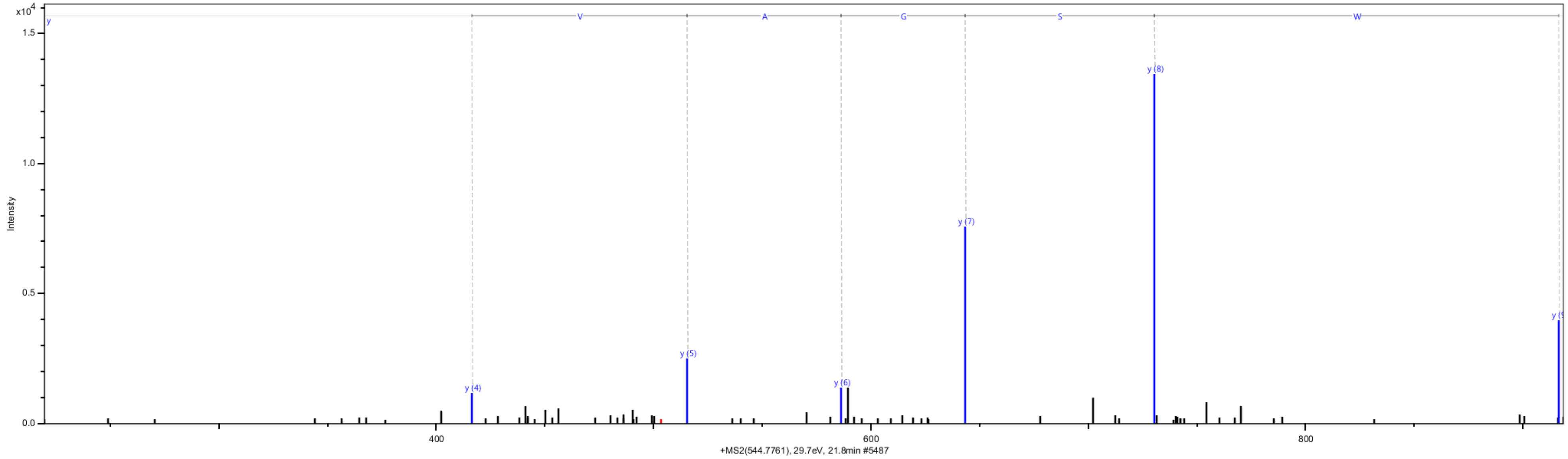

Peptide Match

Sequence: R.CEGPIPDVTFELLR.E **Score:** 39.8

Modifications: Carbamidomethyl: 1

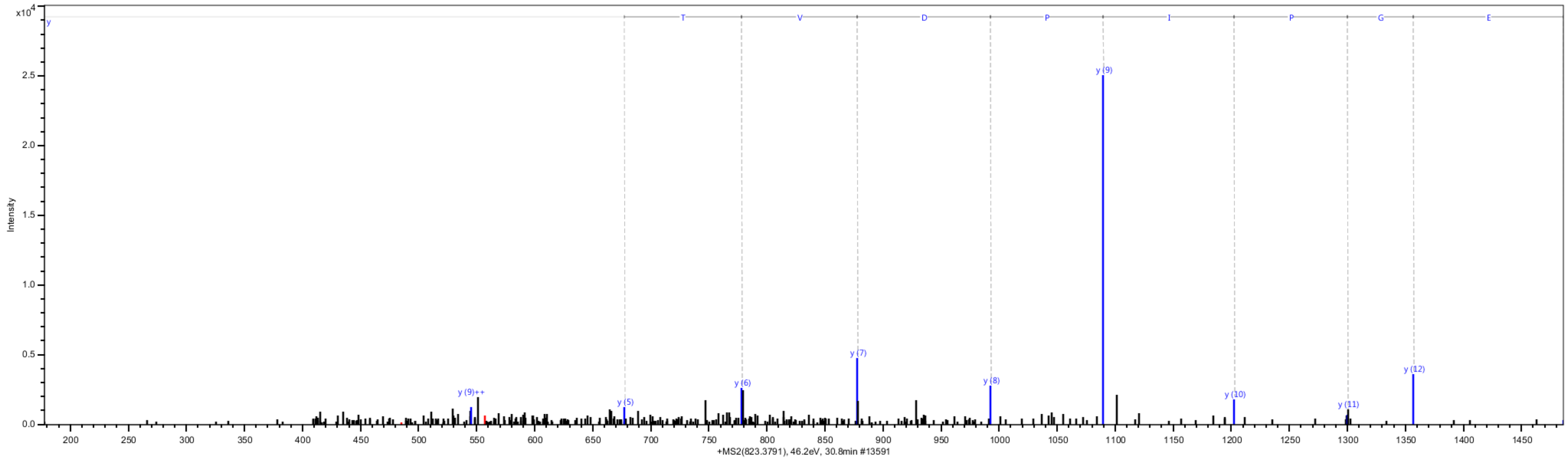
x10^4
Intensity
y
T
V
D
P
I
P
G
E
y (9)
y (7)
y (6)
y (8)
y (9)++
y (5)
y (10)
y (11)
y (12)
+MS2(823.3791), 46.2eV, 30.8min #13591

ANT3_HUMAN

Accession: ANT3_HUMAN
Database: SwissProt
MW [kDa] / pI: 52.6 / 6.3
Score: 185.9
Seq. Coverage [%]: 14.7
No. of Peptides: 6

Peptide Match

Sequence: R.DIPMNPMCIYR.S
Score: 22.2
Modifications: Carbamidomethyl: 8

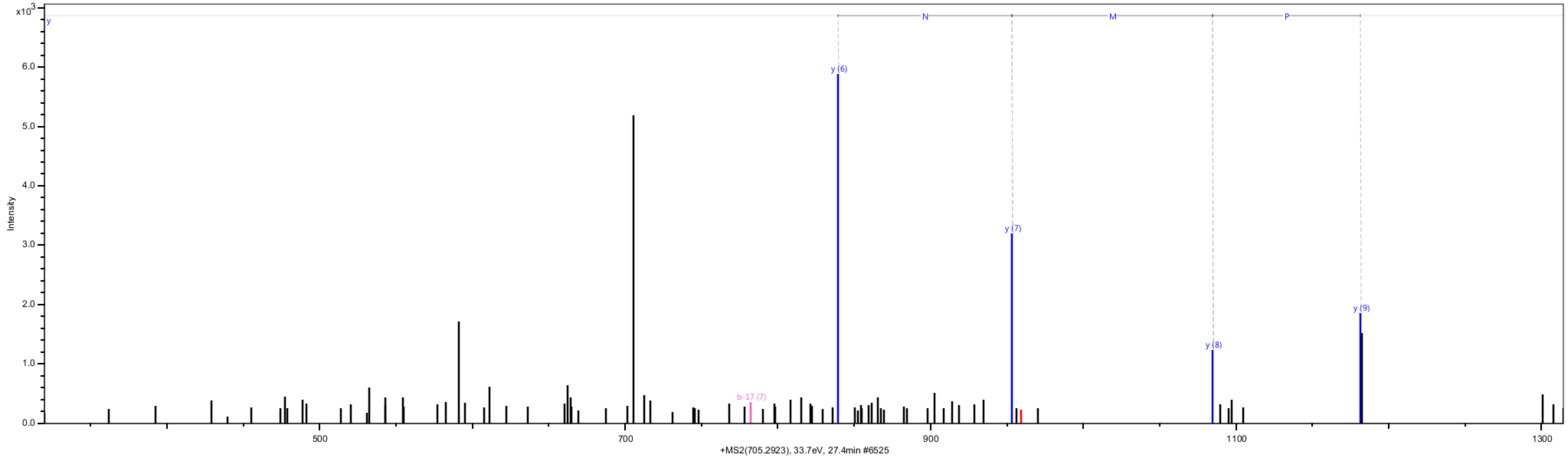

Peptide Match

Sequence: K.TSDQIHFFFAK.L
Score: 21.2
Modifications:

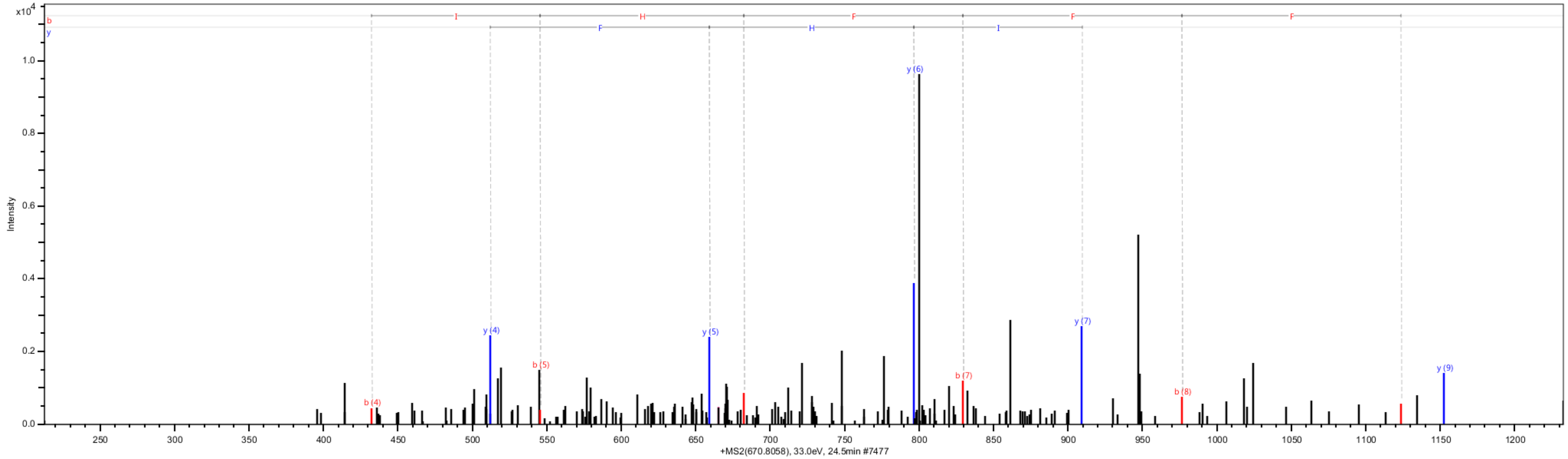

Peptide Match

Sequence: K.LQPLDFKENAEQSR.A **Score:** 39.1

Modifications:

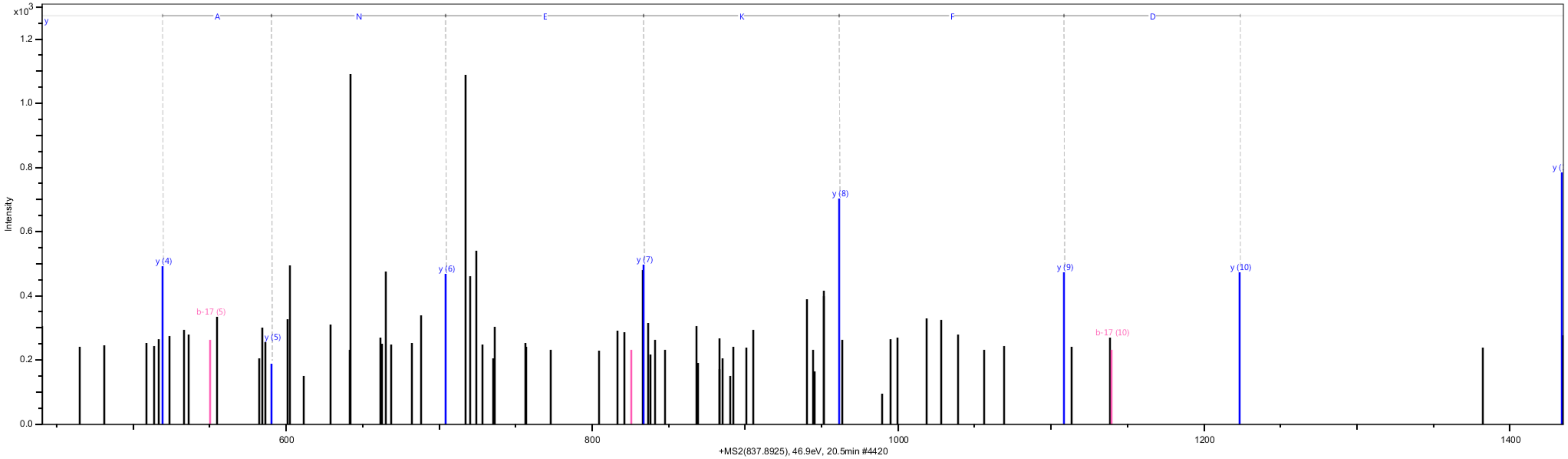

Peptide Match

Sequence: R.FRIEDGFSLK.E **Score:** 16

Modifications:

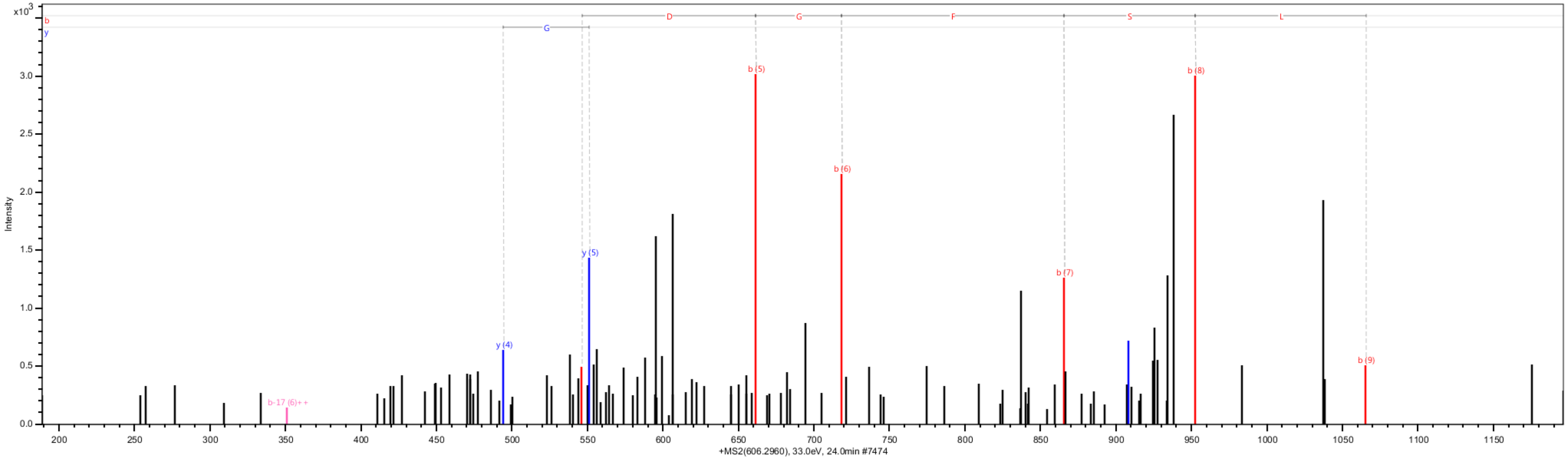

Peptide Match

Sequence: K.ANRPFLVFIR.E **Score:** 28.8
Modifications:

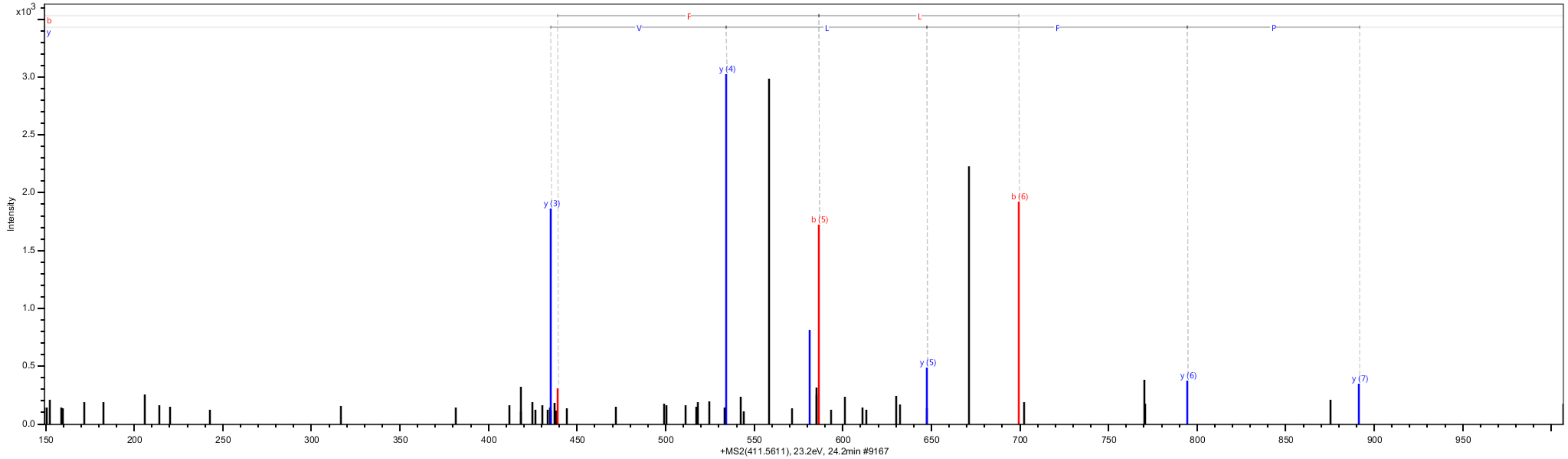

Peptide Match

Sequence: R.EVPLNTIIFMGR.V **Score:** 58.5

Modifications:

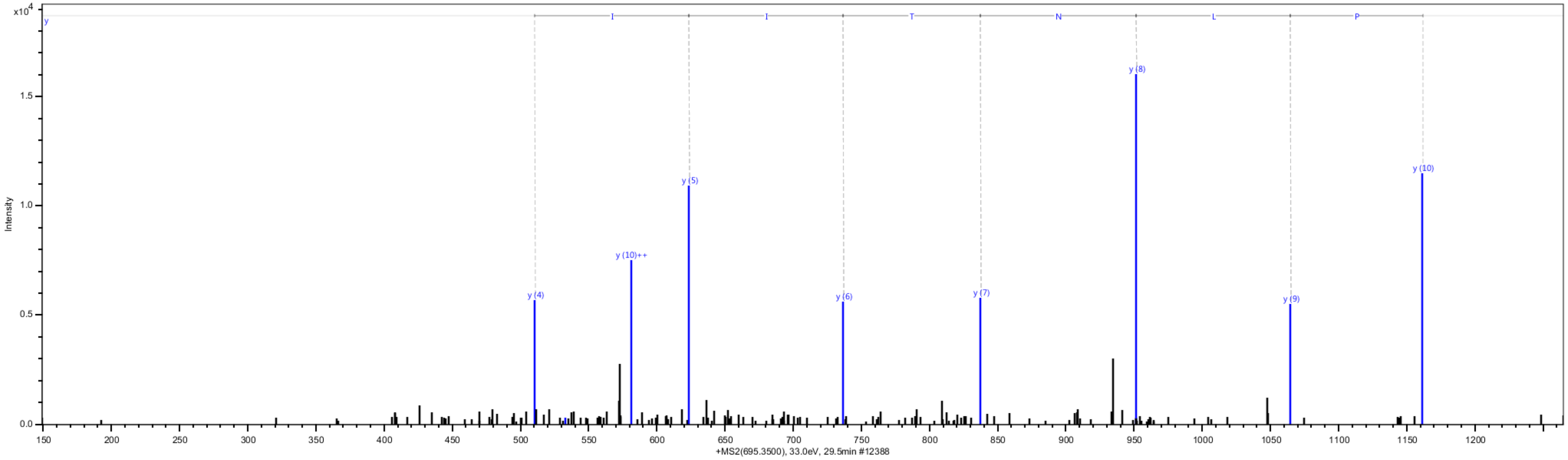
x10^4
Intensity
y
I
I
T
N
L
P
y (4)
y (10)++
y (5)
y (6)
y (7)
y (8)
y (9)
y (10)
+MS2(695.3500), 33.0eV, 29.5min #12388

ITLN1_HUMAN

Accession: ITLN1_HUMAN
Database: SwissProt
MW [kDa] / pI: 34.9 / 5.7

Score: 185.4
Seq. Coverage [%]: 10.9
No. of Peptides: 3

Peptide Match

Sequence: K.EWTCSSSPSLPR.S
Score: 39.8
Modifications: Carbamidomethyl: 4

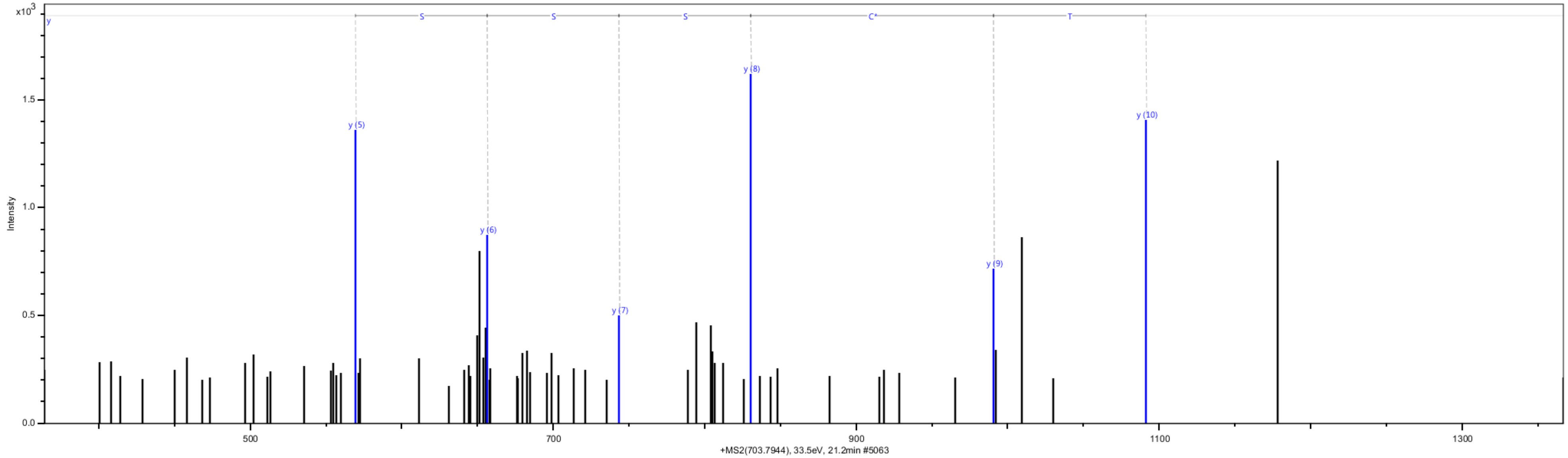

Peptide Match

Sequence: R.EFTAGFVQFR.V
Score: 77.5
Modifications:

Spectrum Report
Combine Search_2020-10-19 07:53:31

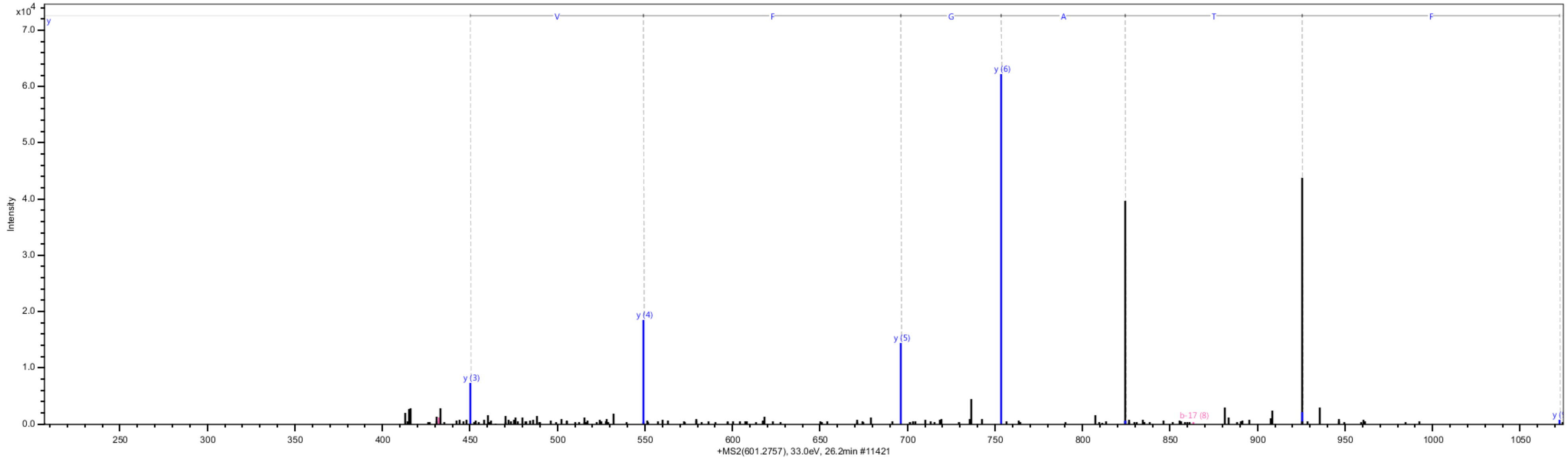

Peptide Match

**Sequence:**	R.EITEAAVLLFYR.-	**Score:**	68.1
**Modifications:**			

Spectrum Report
Combine Search_2020-10-19 07:53:31

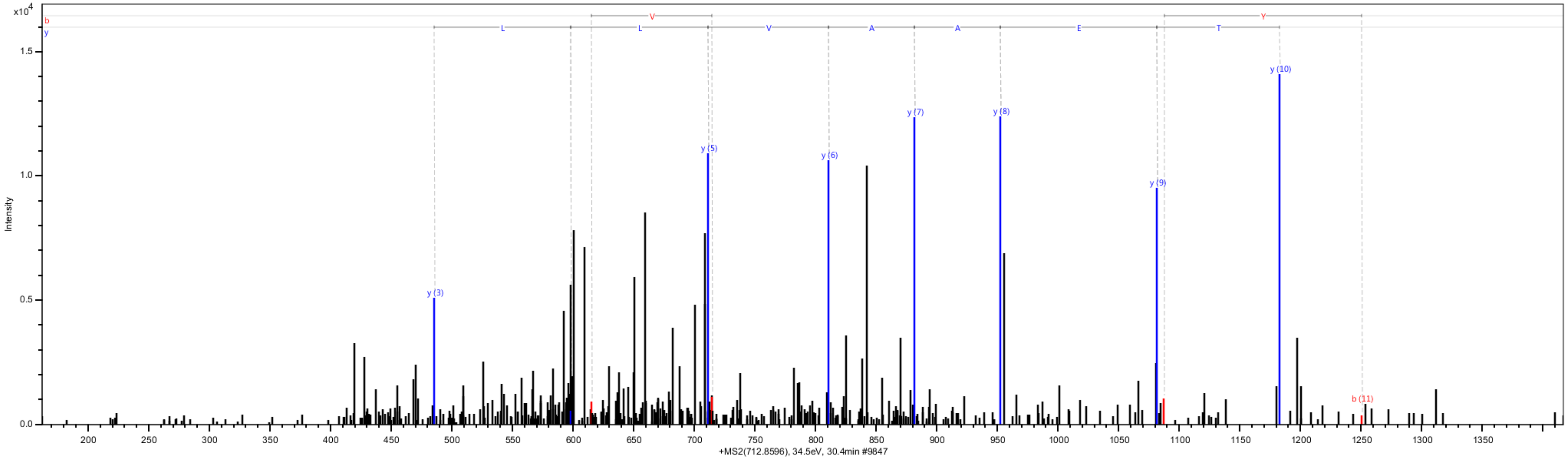

FIBG_HUMAN

**Accession:**	FIBG_HUMAN	**Score:**	183.9
**Database:**	SwissProt	**Seq. Coverage [%]:**	13.2
**MW [kDa] / pI:**	51.5 / 5.4	**No. of Peptides:**	5

Peptide Match

**Sequence:**	R.DNCCILDER.F	**Score:**	33.4
**Modifications:**	Carbamidomethyl: 3, 4		

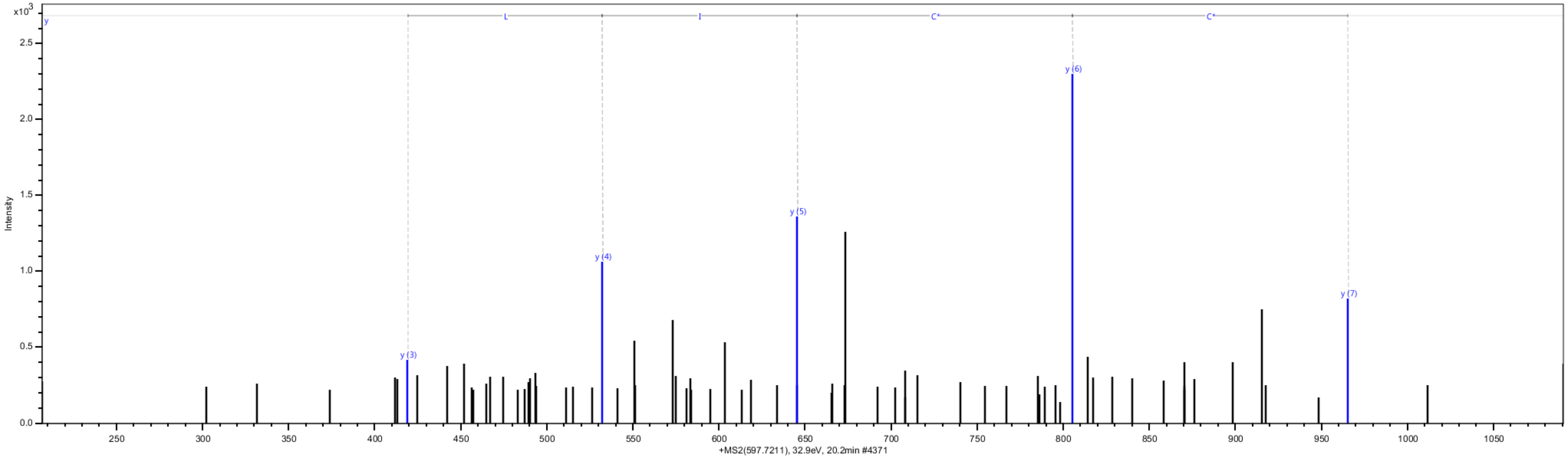

Peptide Match

**Sequence:**	K.YEASILTHDSSIR.Y	**Score:**	57.6
**Modifications:**			

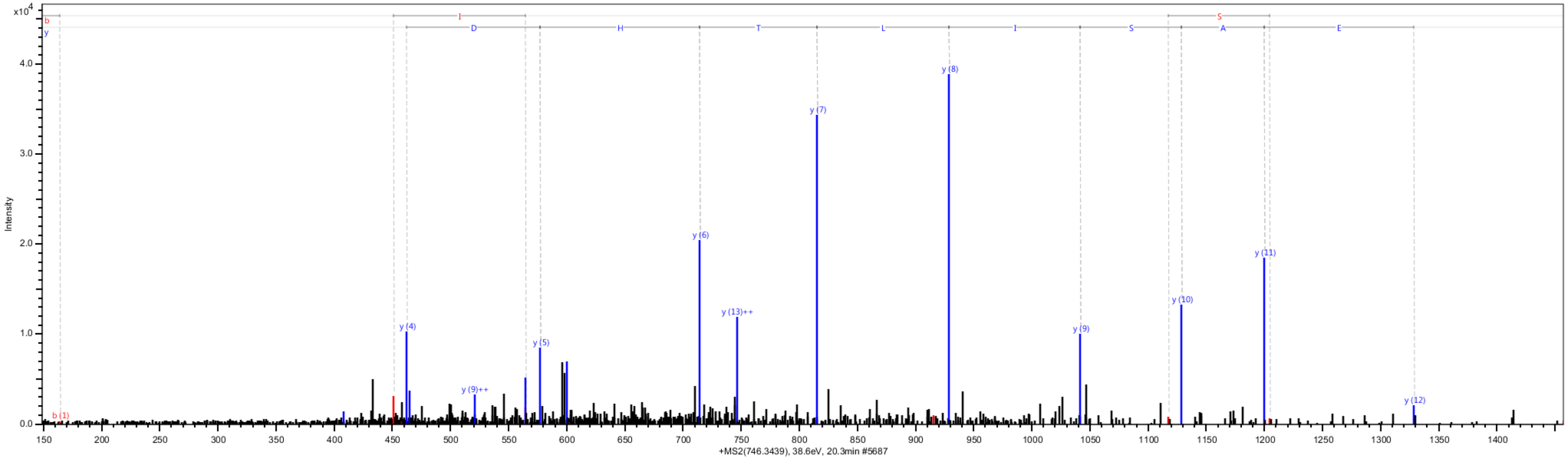

Peptide Match

Sequence: R.YLQEIYNSNNQK.I **Score:** 29.3

Modifications:

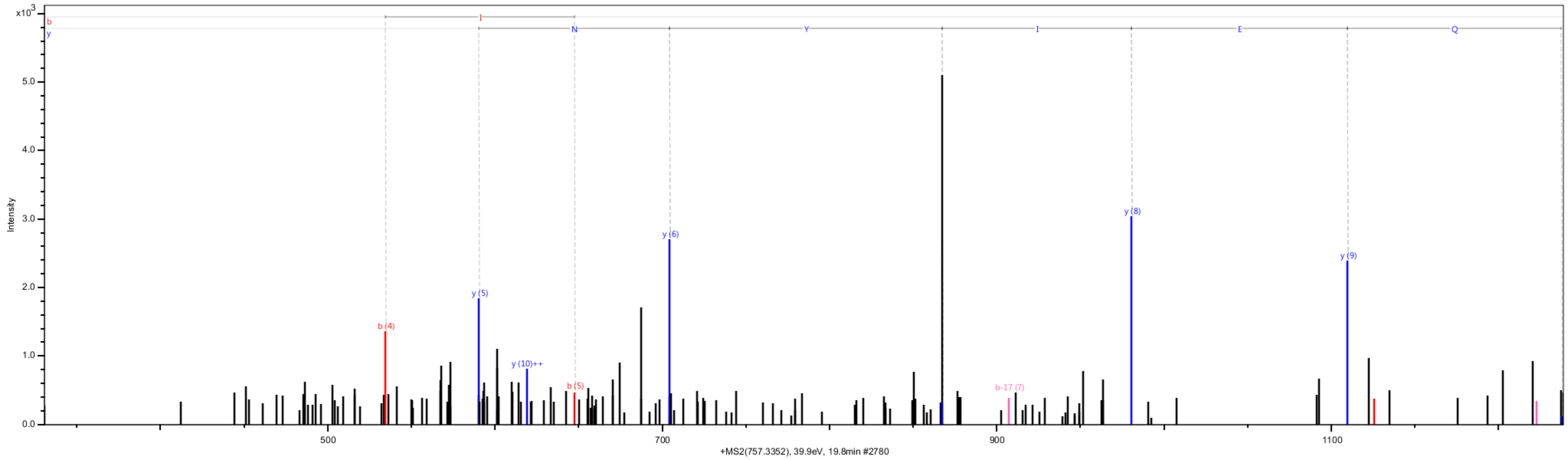

Peptide Match

**Sequence:**	K.QSGLYFIKPLK.A	**Score:**	38.5
**Modifications:**			

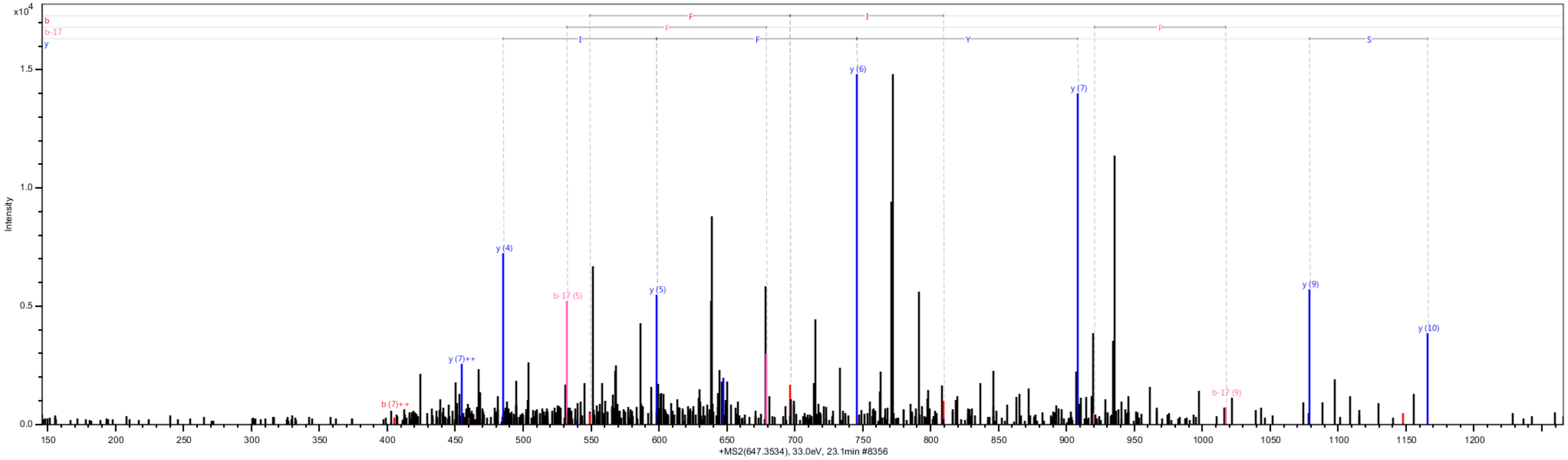

Peptide Match

Sequence: K.IHLISTQSAIPYALR.V **Score:** 25.1

Modifications:

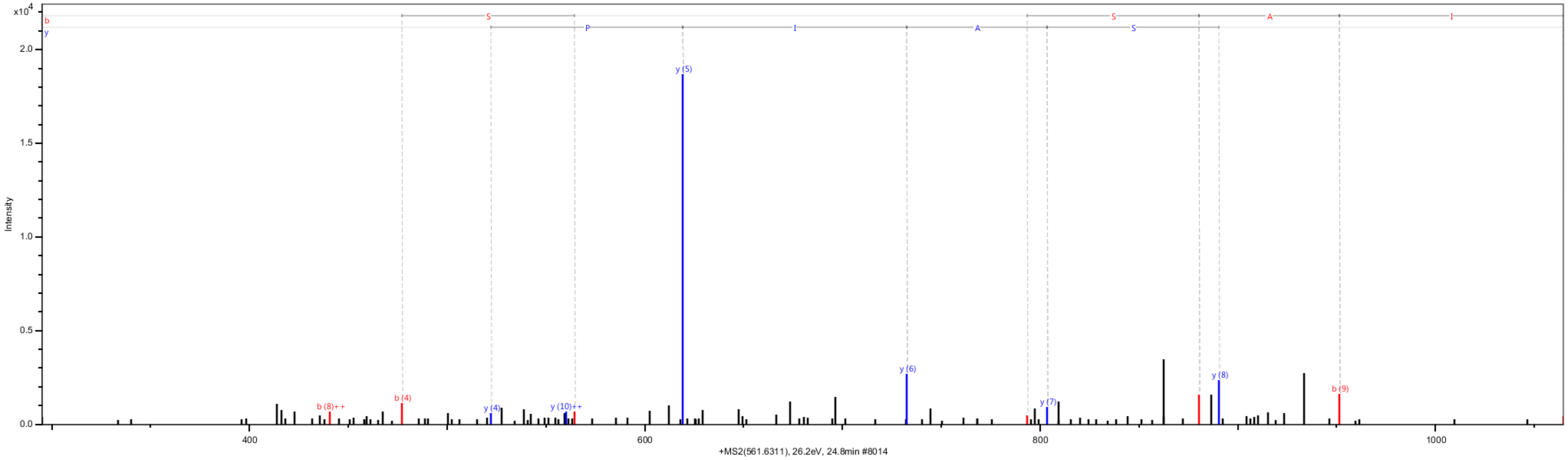
x10^4
b
y
S
P
I
A
S
A
I
2.0
1.5
1.0
0.5
0.0
Intensity
y (5)
y (6)
y (8)
b (9)
y (7)
b (8)++
b (4)
y (4)
y (10)++
400
600
800
1000
+MS2(561.6311), 26.2eV, 24.8min #8014

AFAM_HUMAN

**Accession:**	AFAM_HUMAN	**Score:**	173.7
**Database:**	SwissProt	**Seq. Coverage [%]:**	9.2
**MW [kDa] / pI:**	69.0 / 5.6	**No. of Peptides:**	5

Peptide Match

Sequence: R.ESLLNHFLYEVAR.R **Score:** 42.3

Modifications:

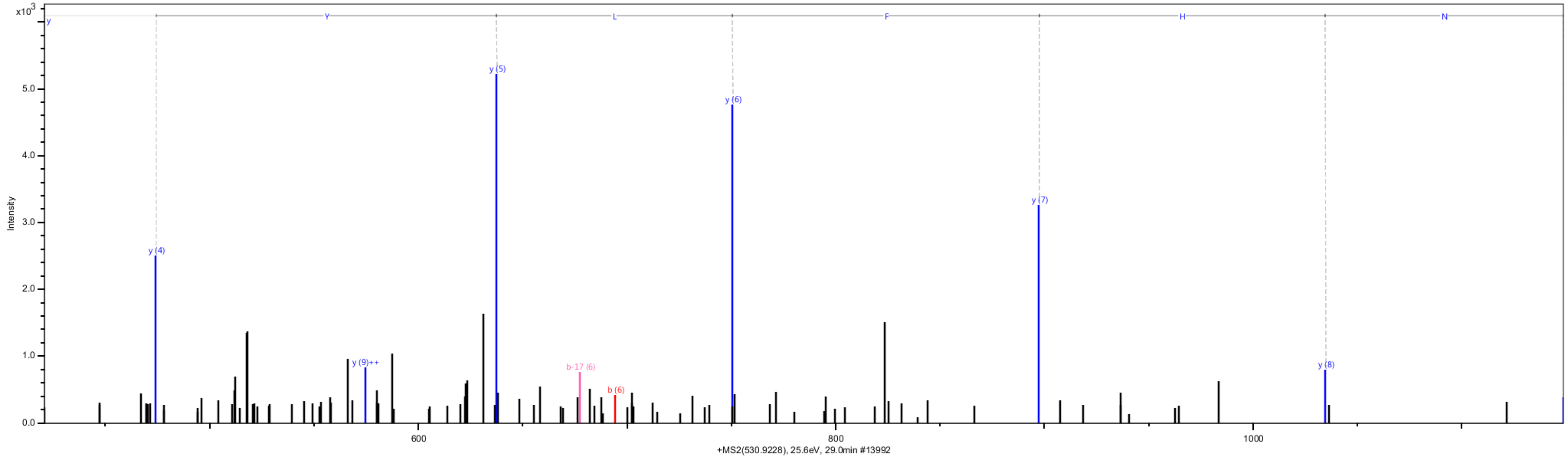

Peptide Match

Sequence: K.FTFEYSR.R **Score:** 28.7

Modifications:

Spectrum Report
Combine Search_2020-10-19 07:53:31

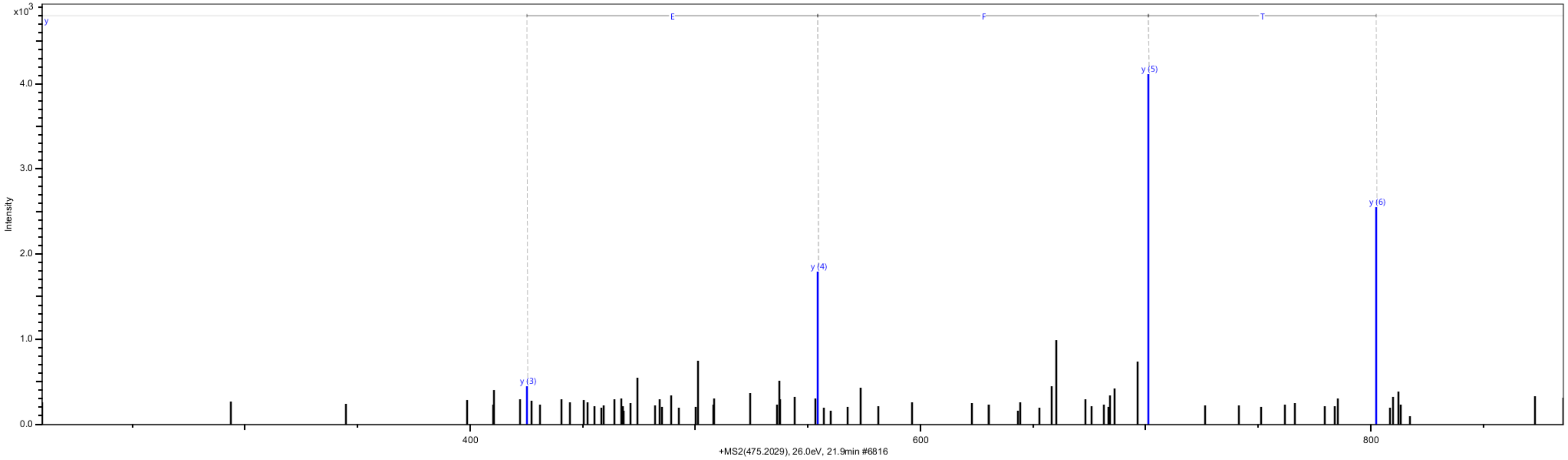

Peptide Match

Sequence: R.RHPDLSIPELLR.I **Score:** 23.7

Modifications:

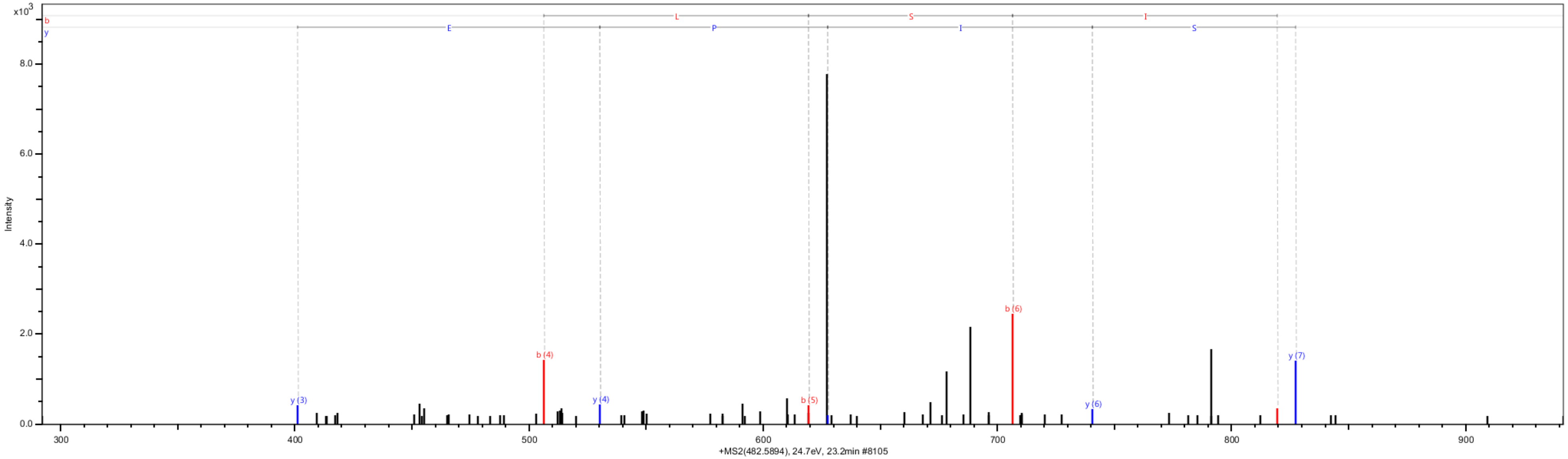

Peptide Match

Sequence: K.IAPQLSTEELVSLGEK.M **Score:** 51.1

Modifications:

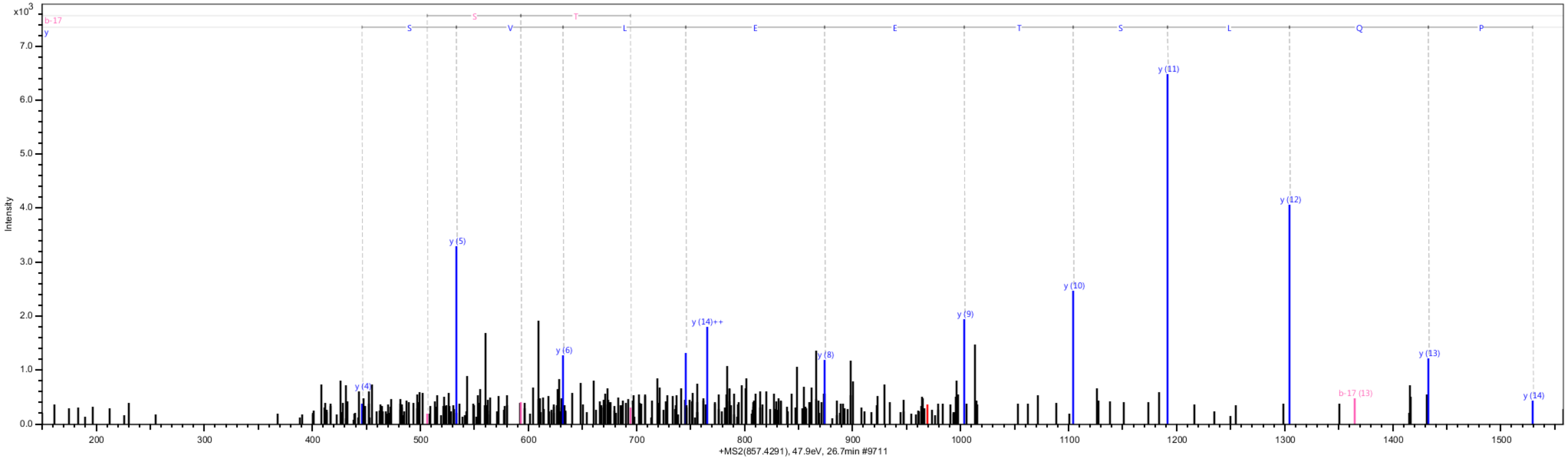

Peptide Match

Sequence: R.FLVNLVK.L

Score: 27.9

Modifications:

Spectrum Report
Combine Search_2020-10-19 07:53:31

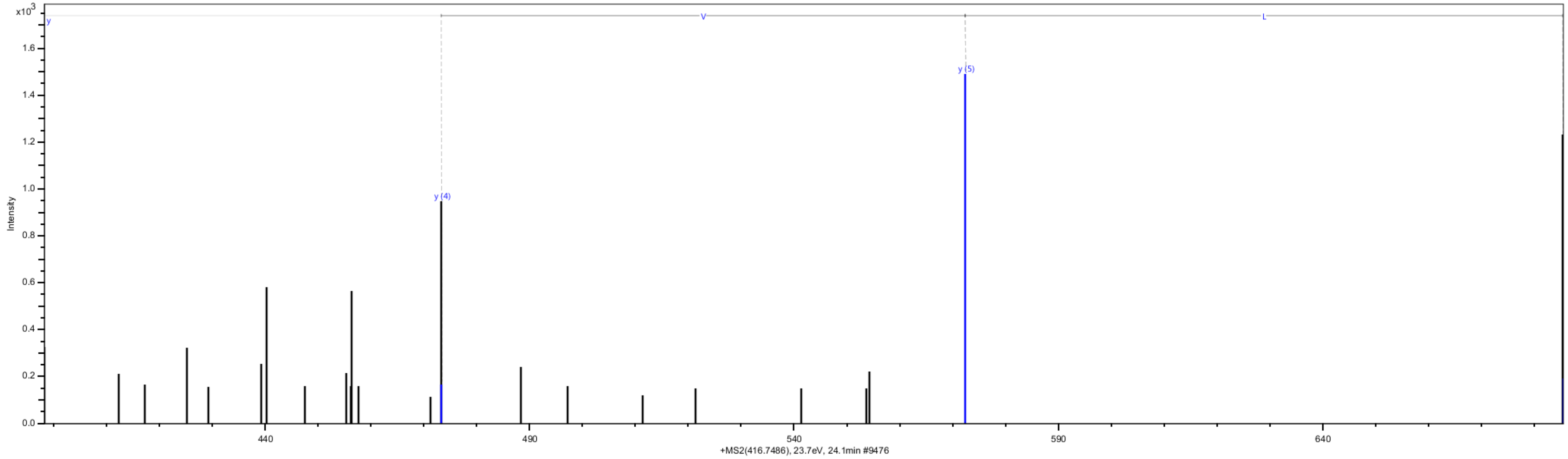

HRG_HUMAN

**Accession:**	HRG_HUMAN	**Score:**	167.7
**Database:**	SwissProt	**Seq. Coverage [%]:**	7.0
**MW [kDa] / pI:**	59.5 / 7.1	**No. of Peptides:**	3

Peptide Match

**Sequence:**	R.DGYLFQLLR.I	**Score:**	38
**Modifications:**			

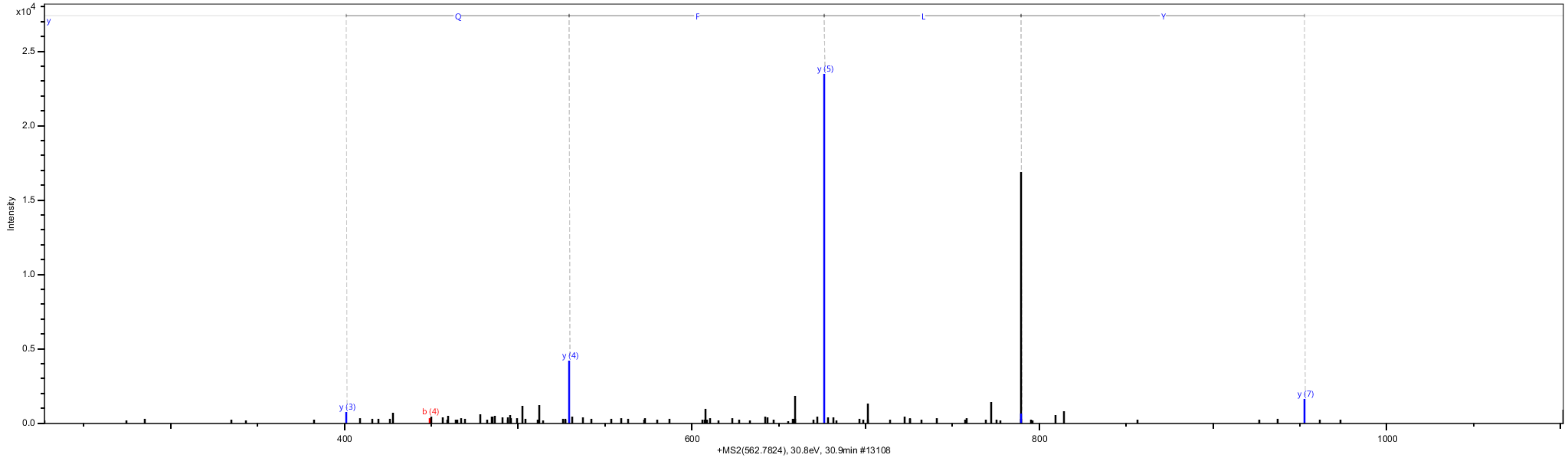

Peptide Match

**Sequence:**	K.DSPVLIDFFEDTER.Y	**Score:**	91
**Modifications:**			

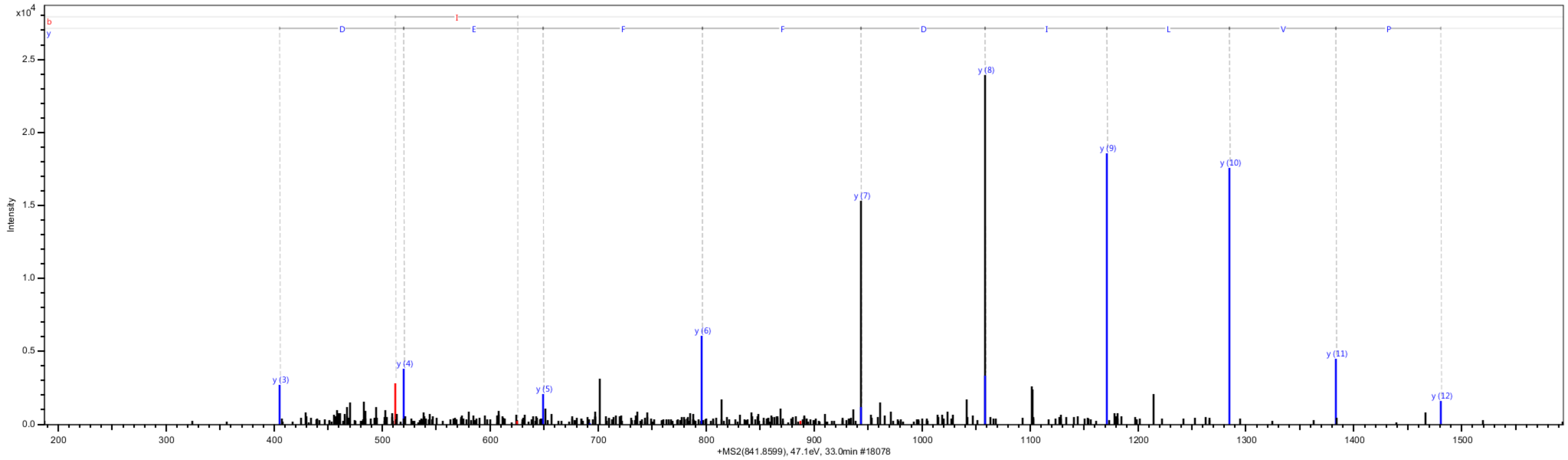

Peptide Match

Sequence: R.GGEGTGYFVDFSVR.N **Score:** 38.7

Modifications:

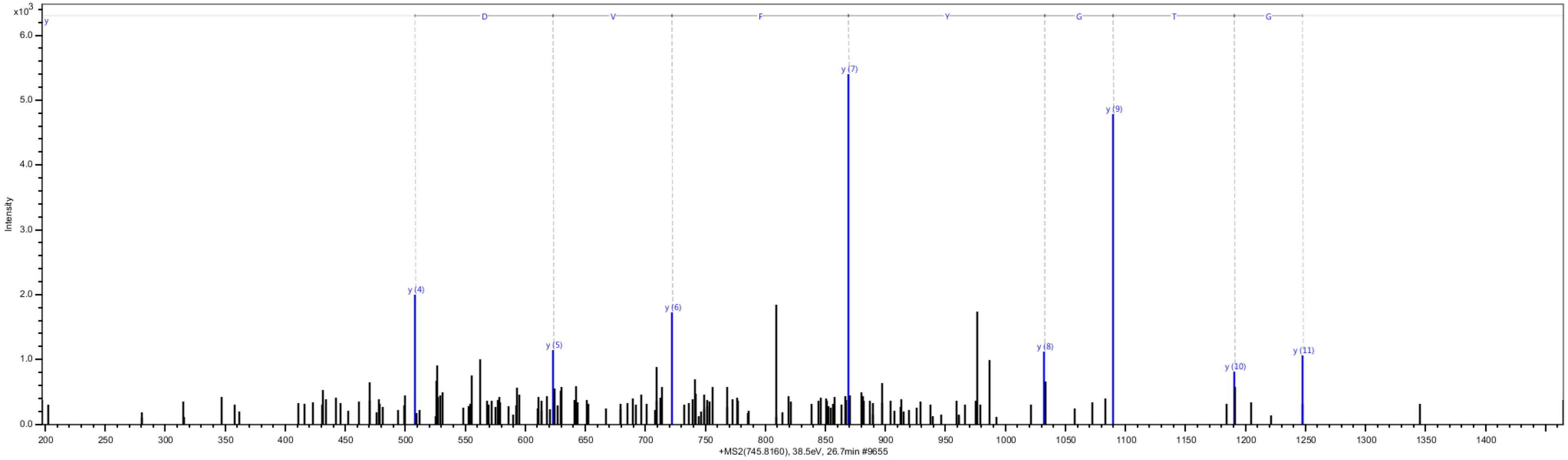
x10^3
Intensity
D
V
F
Y
G
T
G
y (4)
y (5)
y (6)
y (7)
y (8)
y (9)
y (10)
y (11)
+MS2(745.8160), 38.5eV, 26.7min #9655

VTNC_HUMAN

Accession: VTNC_HUMAN
Database: SwissProt
MW [kDa] / pI: 54.3 / 5.6
Score: 154.5
Seq. Coverage [%]: 8.4
No. of Peptides: 3

Peptide Match

Sequence: R.DVWGIEGPIDAAFTR.I **Score:** 79.1
Modifications:

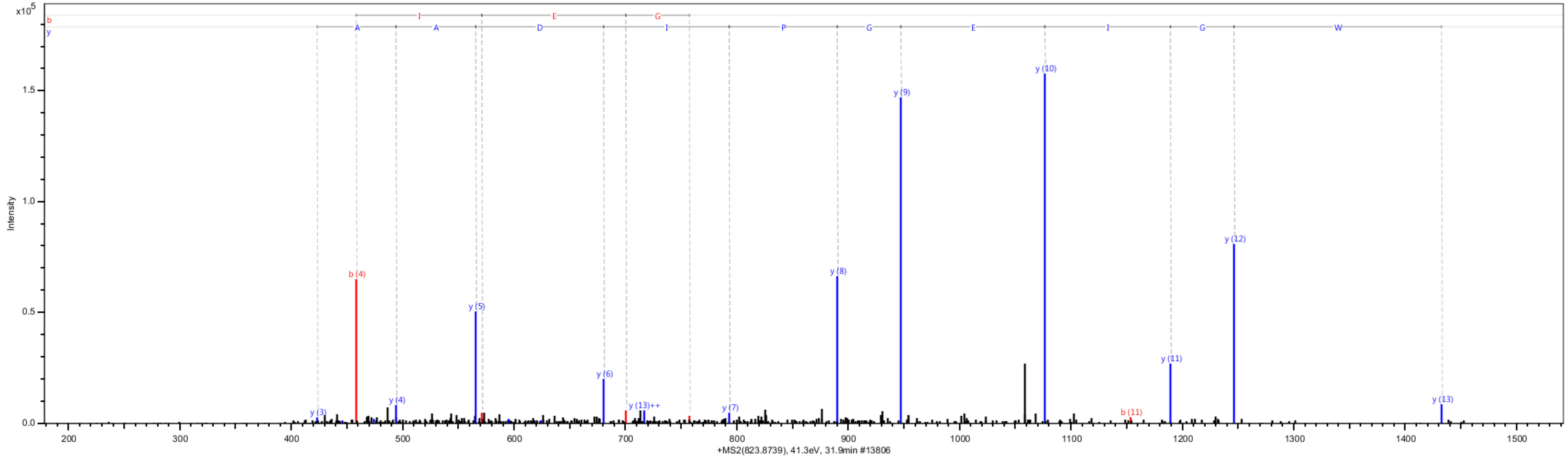

Peptide Match

Sequence: R.VDTVDPPYPR.S **Score:** 24
Modifications:

Spectrum Report
Combine Search_2020-10-19 07:53:31

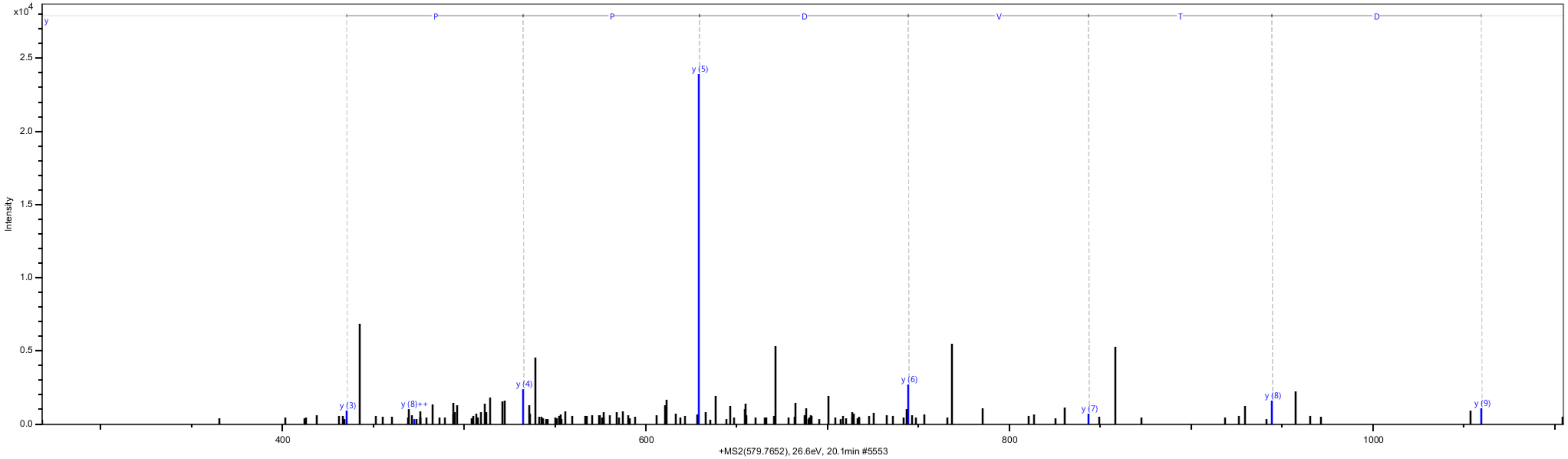

Peptide Match

**Sequence:**	R.SIAQYWLGCPAPGHL.-	**Score:**	51.4
**Modifications:**	Carbamidomethyl: 9		

Spectrum Report
Combine Search_2020-10-19 07:53:31

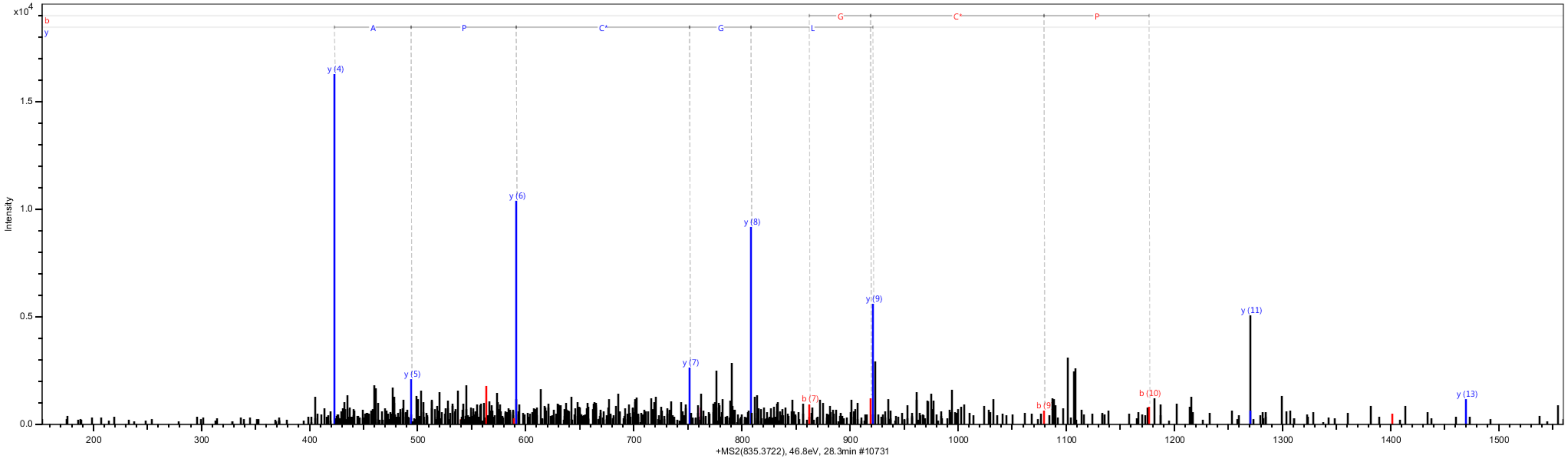

FIBB_HUMAN

Accession: FIBB_HUMAN
Database: SwissProt
MW [kDa] / pI: 55.9 / 8.5
Score: 148.2
Seq. Coverage [%]: 7.3
No. of Peptides: 3

Peptide Match

Sequence: R.EEAPSLRPAPPPISGGGYR.A
Score: 71.3
Modifications:

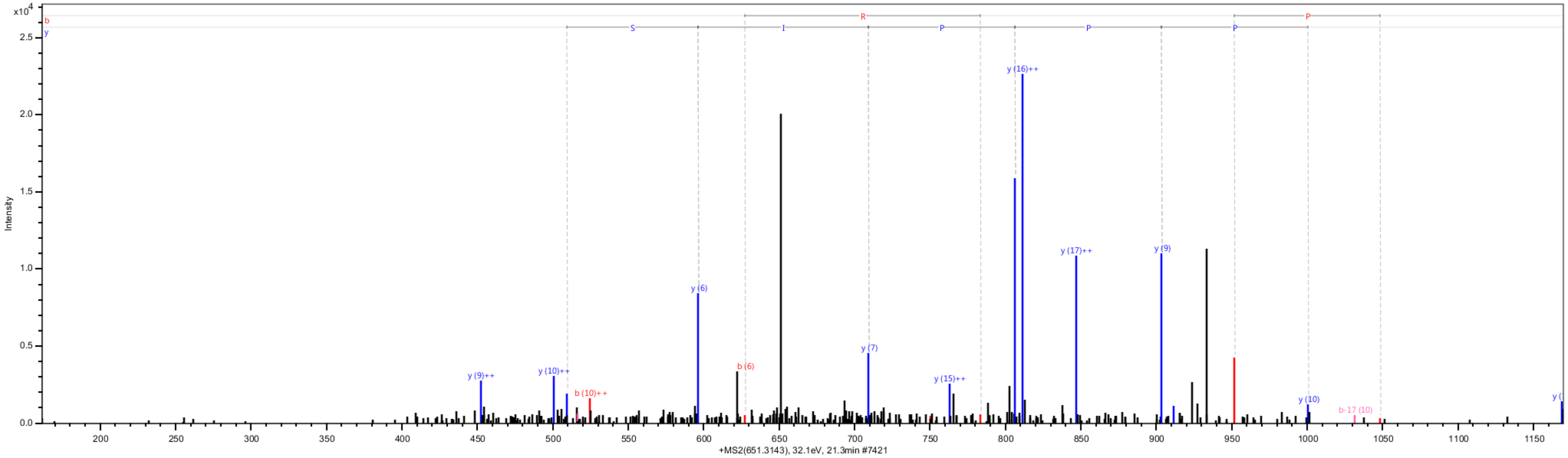

Peptide Match

Sequence: R.SILENLR.S
Score: 32.7
Modifications:

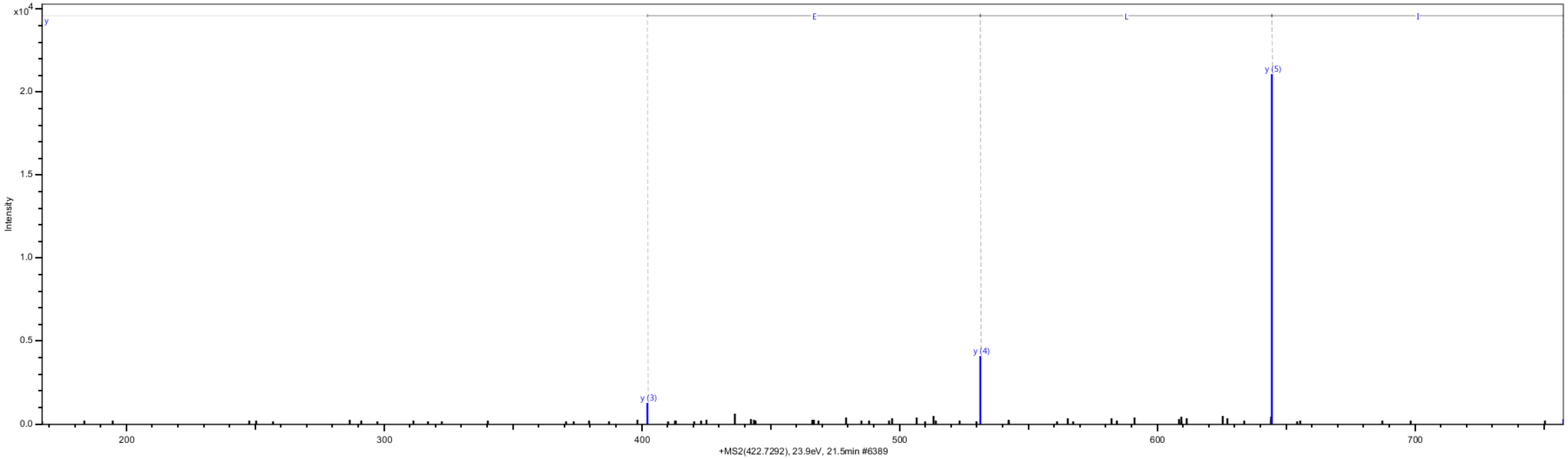

Peptide Match

Sequence: K.EDGGGWWYNR.C **Score:** 44.2

Modifications:

Spectrum Report
Combine Search_2020-10-19 07:53:31

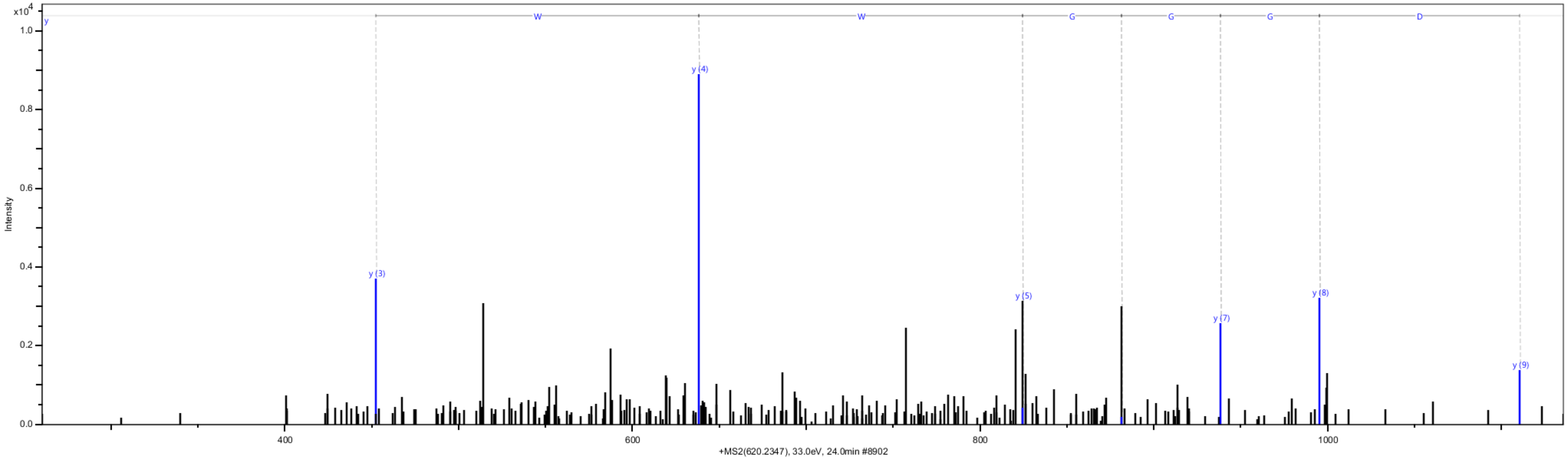

KNG1_HUMAN

Accession: KNG1_HUMAN
Database: SwissProt
MW [kDa] / pI: 71.9 / 6.3
Score: 222.4
Seq. Coverage [%]: 6.8
No. of Peptides: 5

Peptide Match

Sequence: K.YNSQNQSNNQFVLYR.I **Score:** 73.8
Modifications:

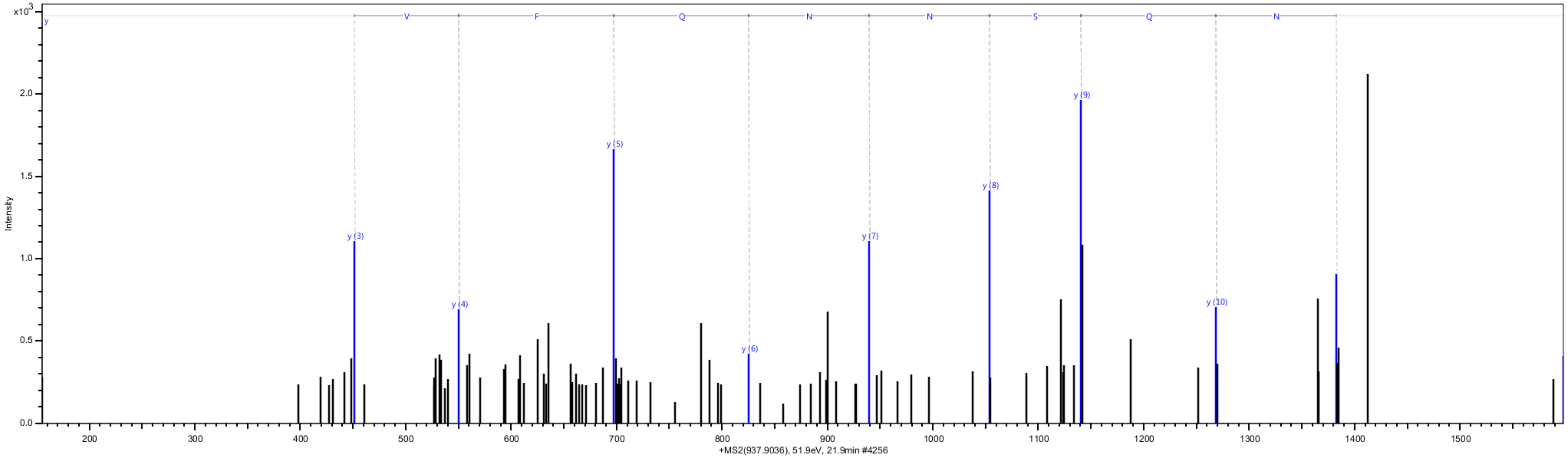

Peptide Match

Sequence: R.QVVAGLNFR.I **Score:** 54.6
Modifications:

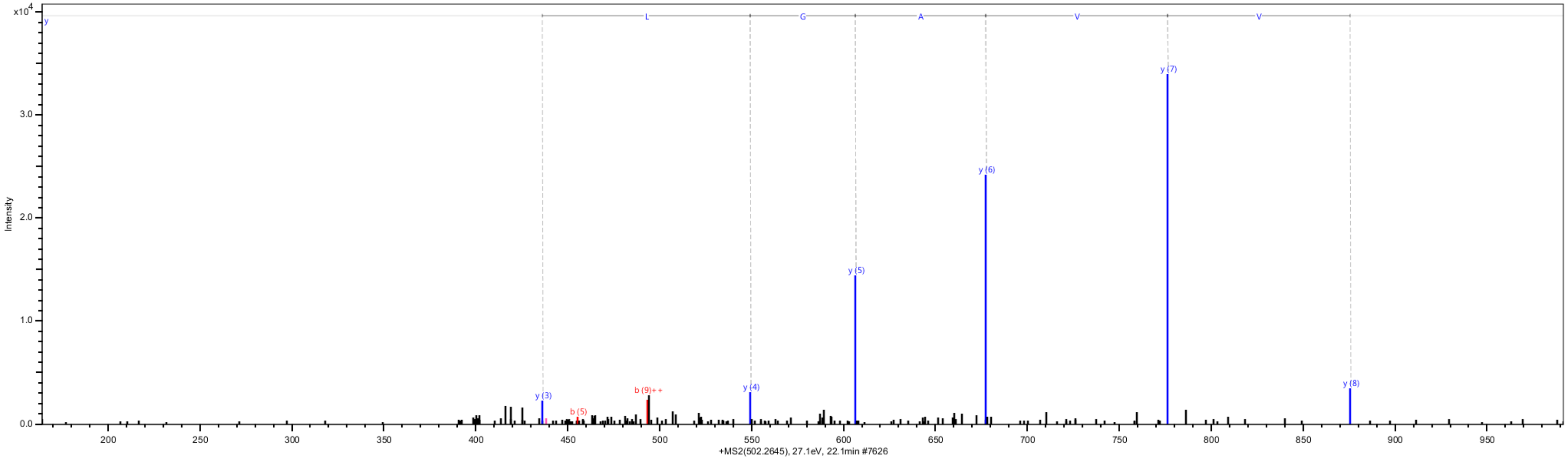

Peptide Match

**Sequence:**	K.ENFLFLTPDCK.S	**Score:**	25.9
**Modifications:**	Carbamidomethyl: 10		

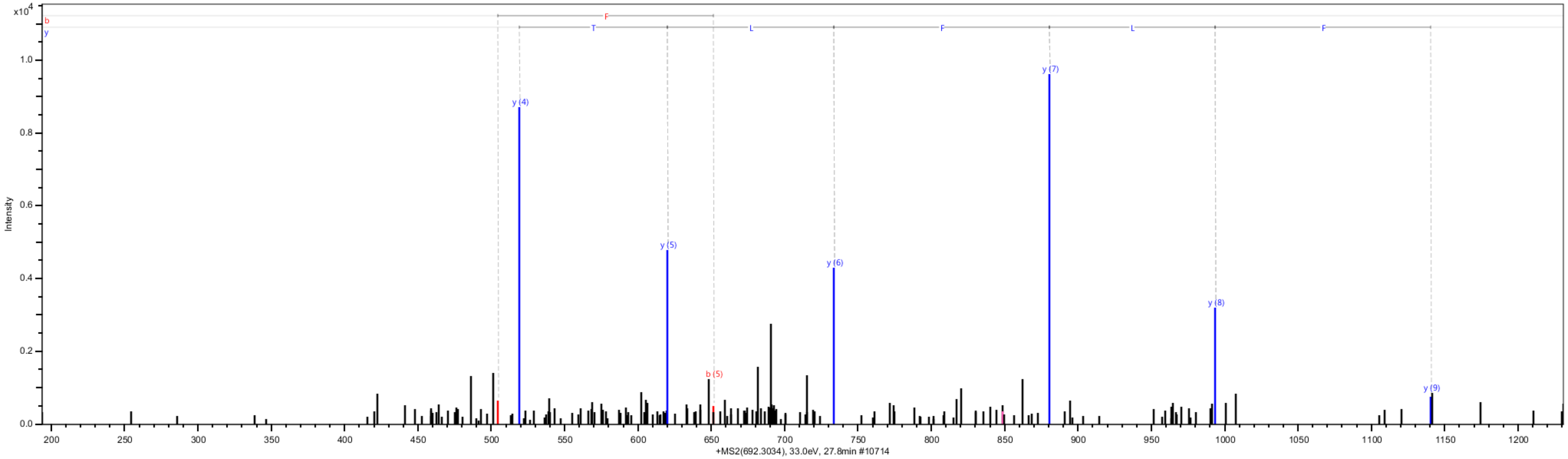

Peptide Match

Sequence: K.KYFIDFVAR.E **Score:** 39.4

Modifications:

Spectrum Report
Combine Search_2020-10-19 07:53:31

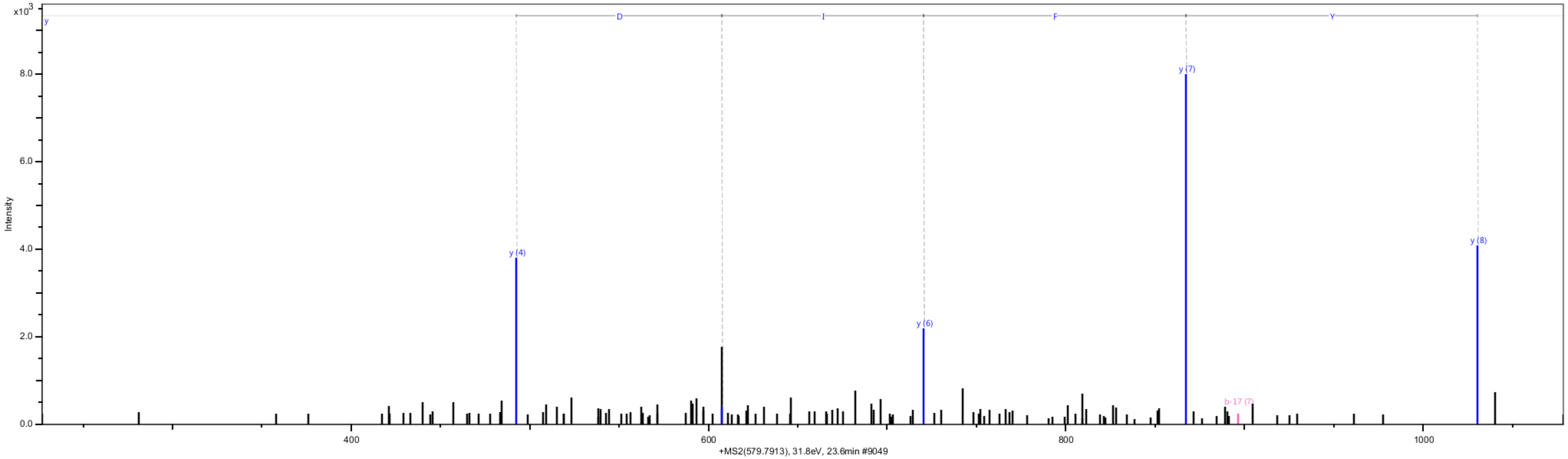

Peptide Match

**Sequence:**	K.YFIDFVAR.E	**Score:**	28.8
**Modifications:**			

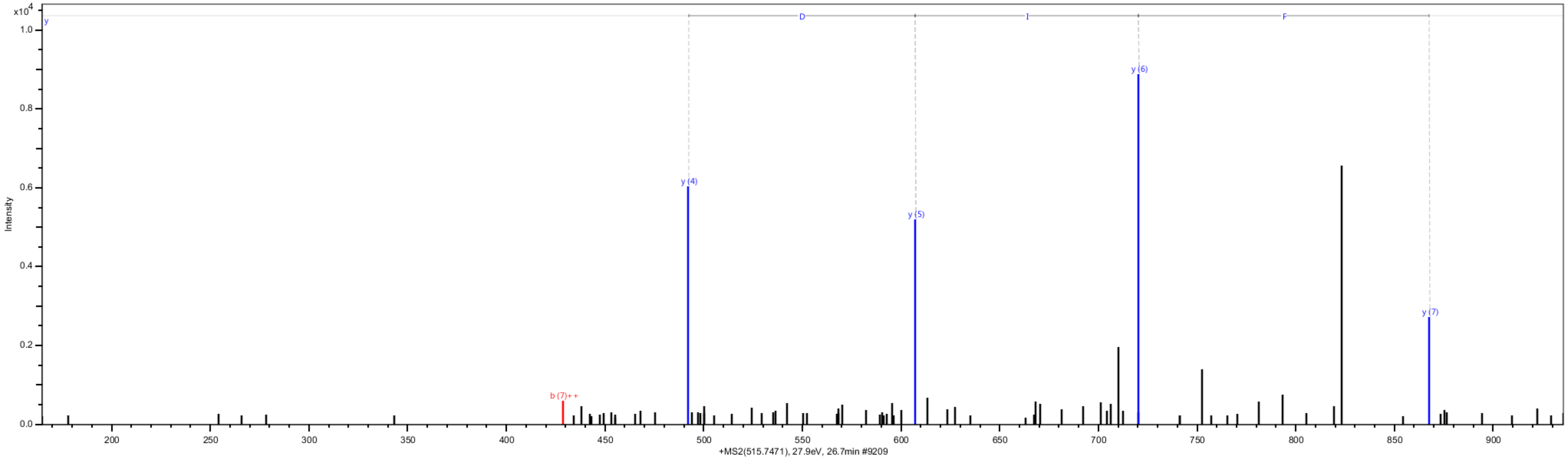
x10^4
Intensity
D
I
F
y (4)
y (5)
y (6)
y (7)
b (7)++
+MS2(515.7471), 27.9eV, 26.7min #9209

AACT_HUMAN

**Accession:**	AACT_HUMAN	**Score:**	221.4
**Database:**	SwissProt	**Seq. Coverage [%]:**	11.1
**MW [kDa] / pI:**	47.6 / 5.3	**No. of Peptides:**	5

Peptide Match

Sequence: K.EQLSLLDR.F **Score:** 15.2

Modifications:

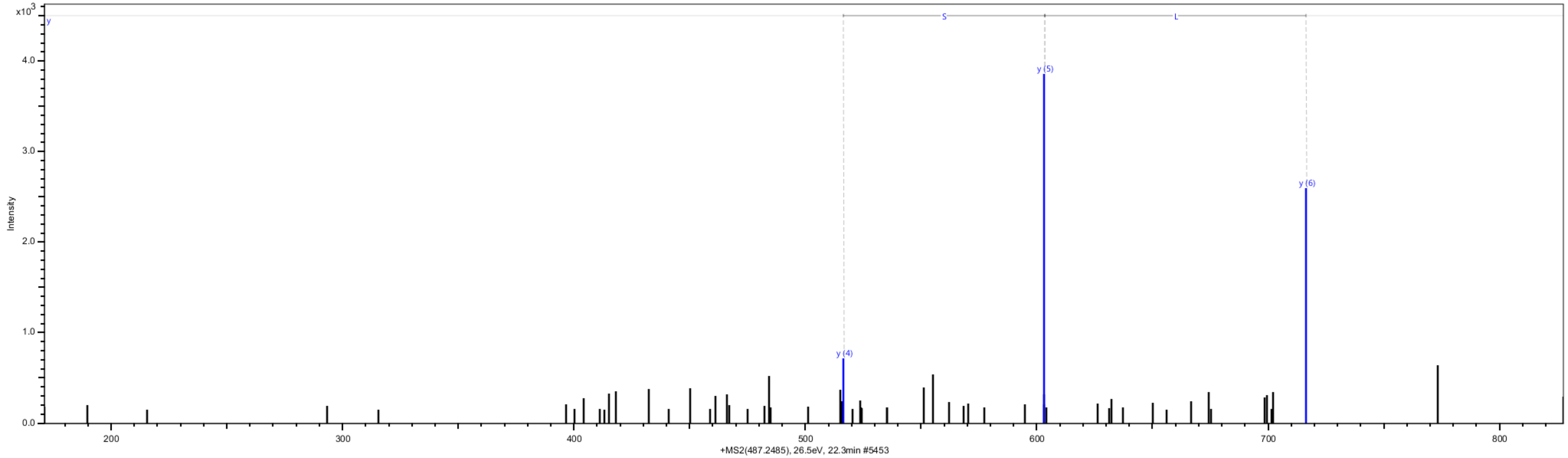

Peptide Match

Sequence: R.WRDSLEFR.E **Score:** 23.2

Modifications:

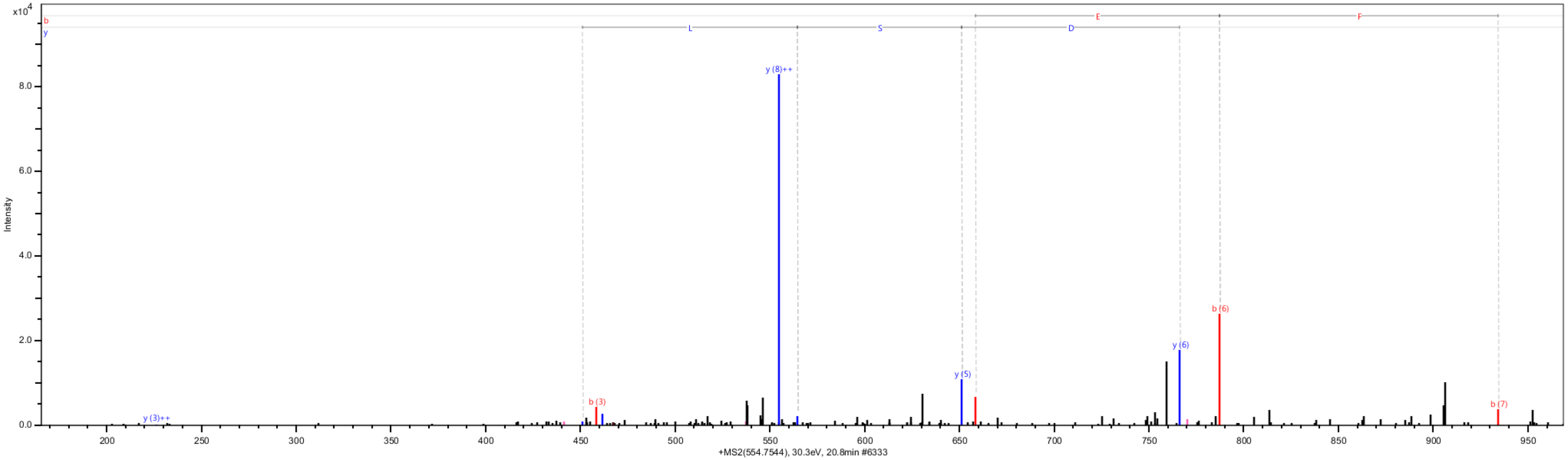

Peptide Match

**Sequence:**	K.ADLSGITGAR.N	**Score:**	53.9
**Modifications:**			

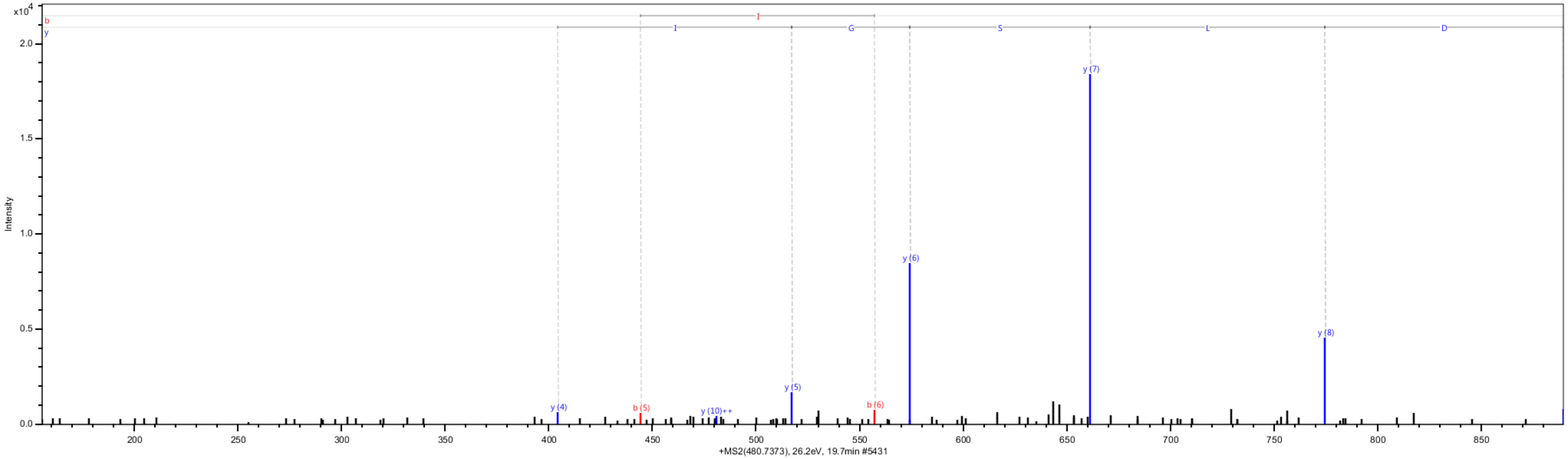

Peptide Match

**Sequence:**	R.NLAVSQVVHK.A	**Score:**	37.5
**Modifications:**			

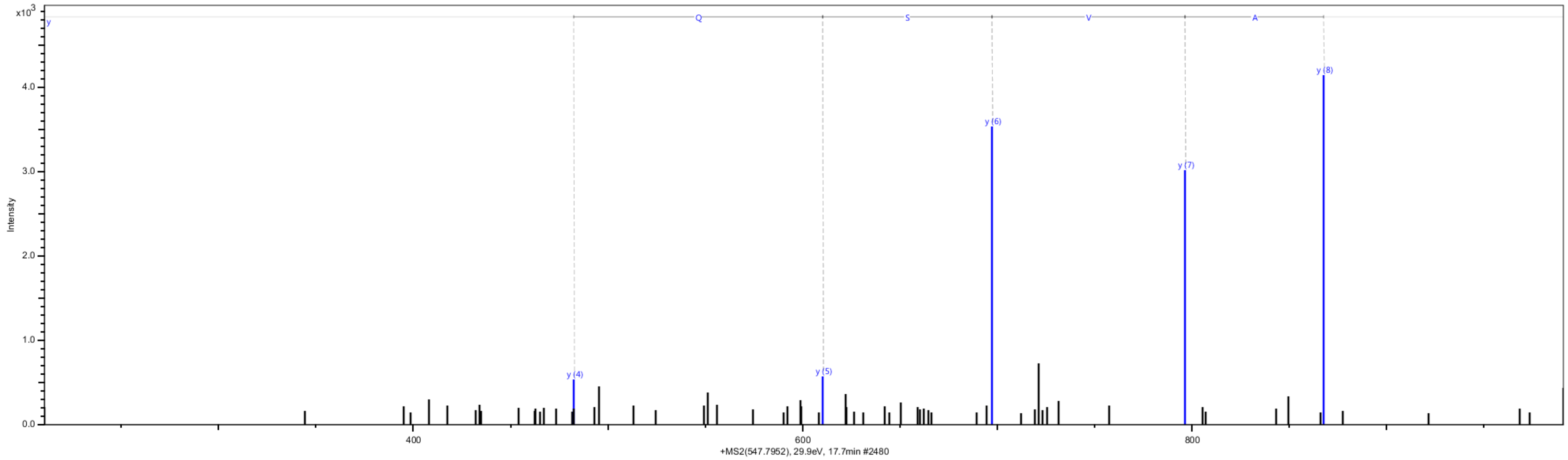

Peptide Match

**Sequence:**	K.ITLLSALVETR.T	**Score:**	91.5
**Modifications:**			

Spectrum Report
Combine Search_2020-10-19 07:53:31

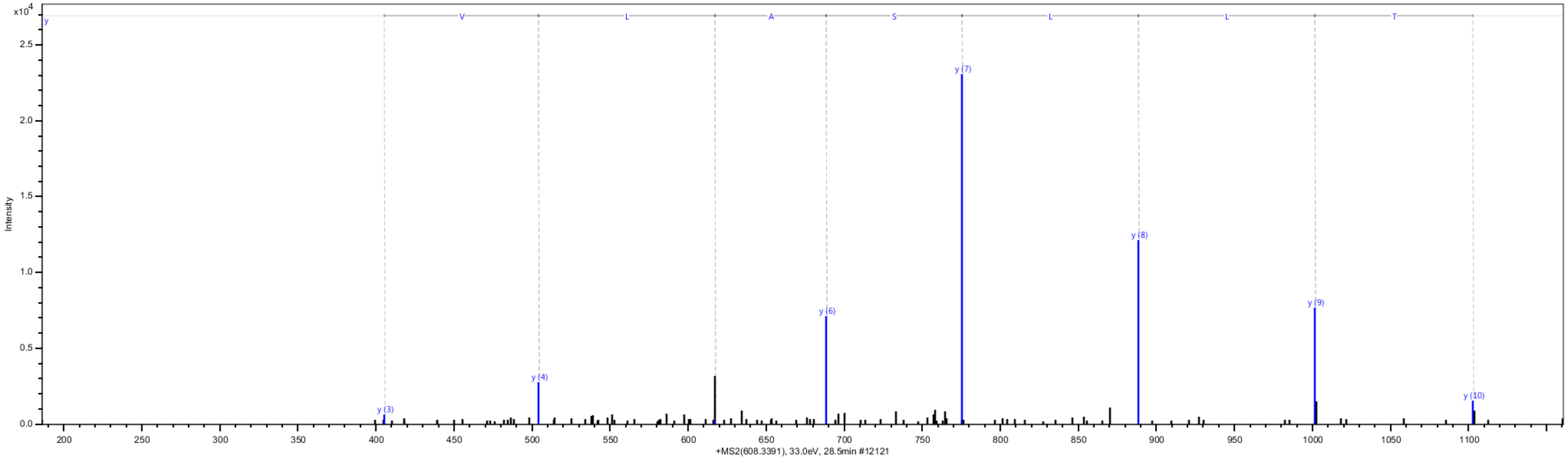

IGHM_HUMAN

Accession: IGHM_HUMAN
Database: SwissProt
MW [kDa] / pI: 49.4 / 6.3
Score: 211.2
Seq. Coverage [%]: 17.9
No. of Peptides: 7

Peptide Match

Sequence: K.VSVFVPPR.D
Score: 26.1
Modifications:

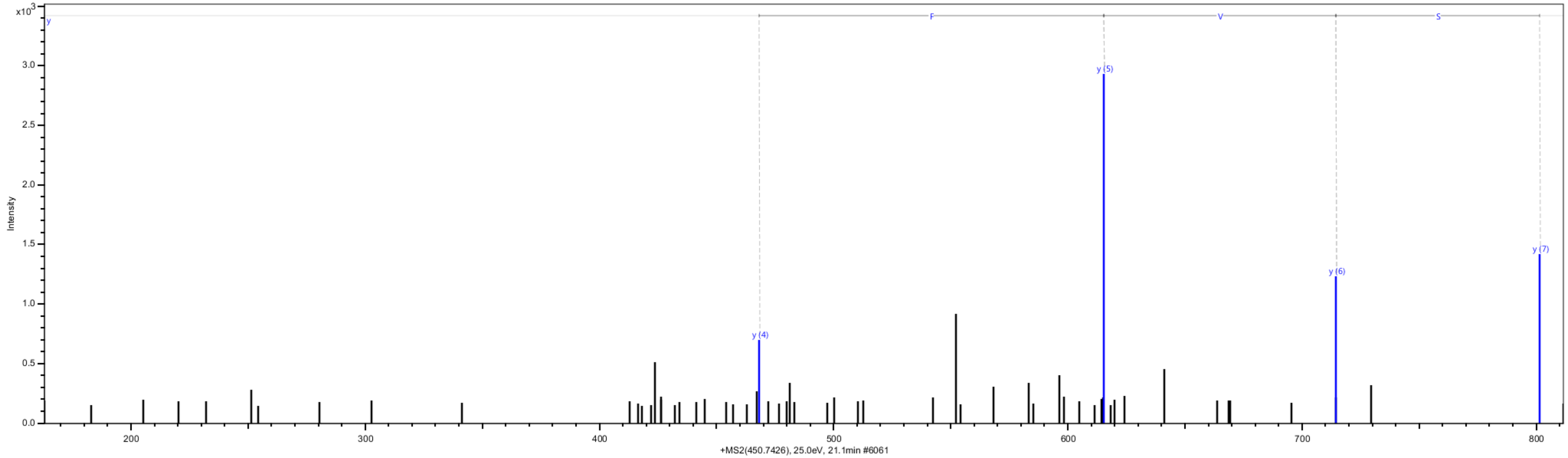

Peptide Match

Sequence: R.DGFFGNPR.K
Score: 18.6
Modifications:

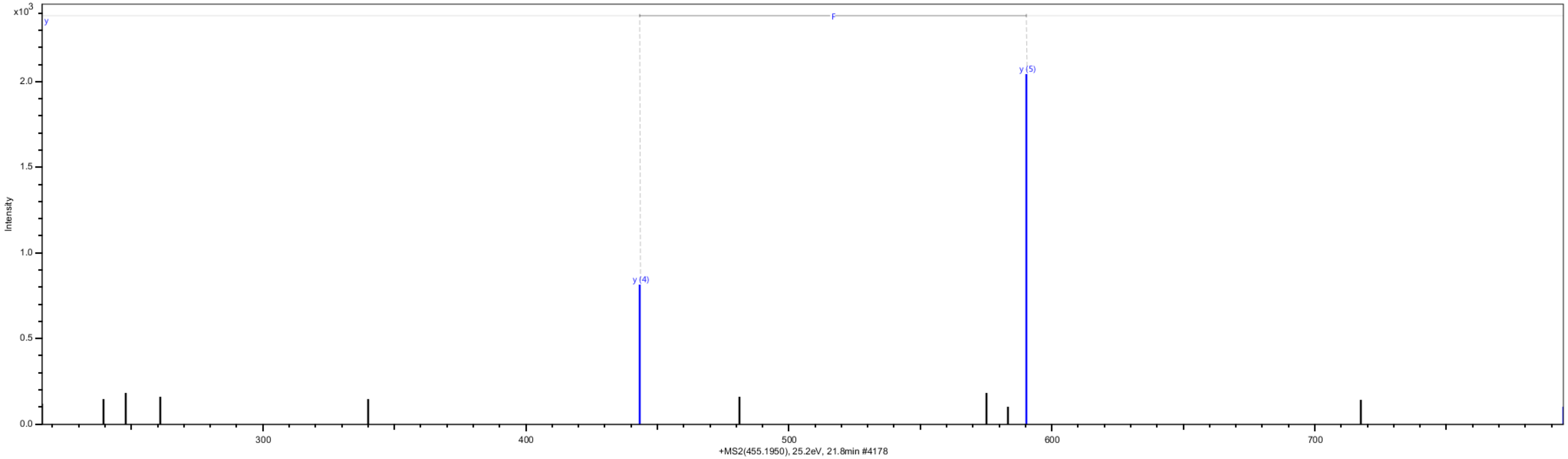

Peptide Match

**Sequence:**	K.LICQATGFSPR.Q	**Score:**	38
**Modifications:**	Carbamidomethyl: 3		

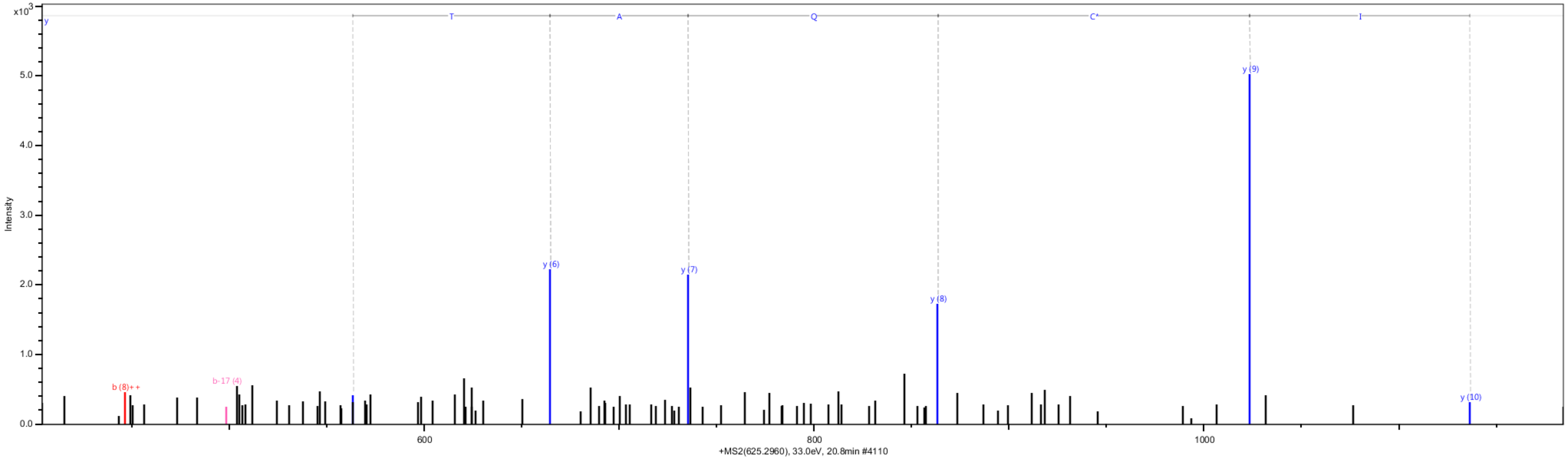

Peptide Match

Sequence: R.QIQVSWLR.E **Score:** 46.4

Modifications:

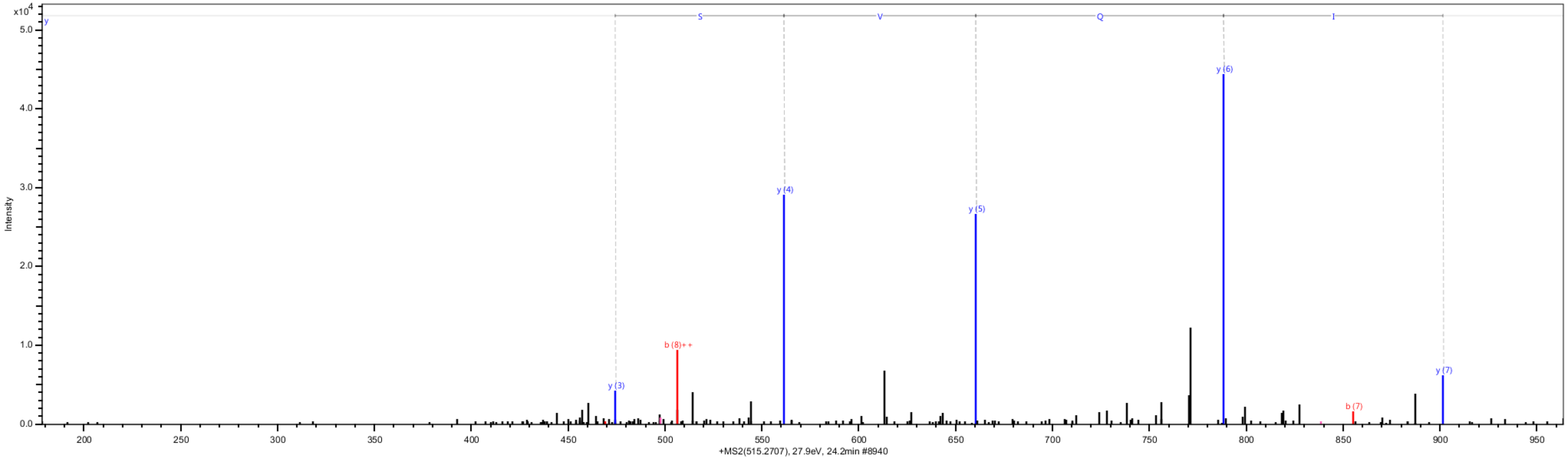

Peptide Match

Sequence: K.QVGSGVTTDQVQAEAK.E **Score:** 42

Modifications:

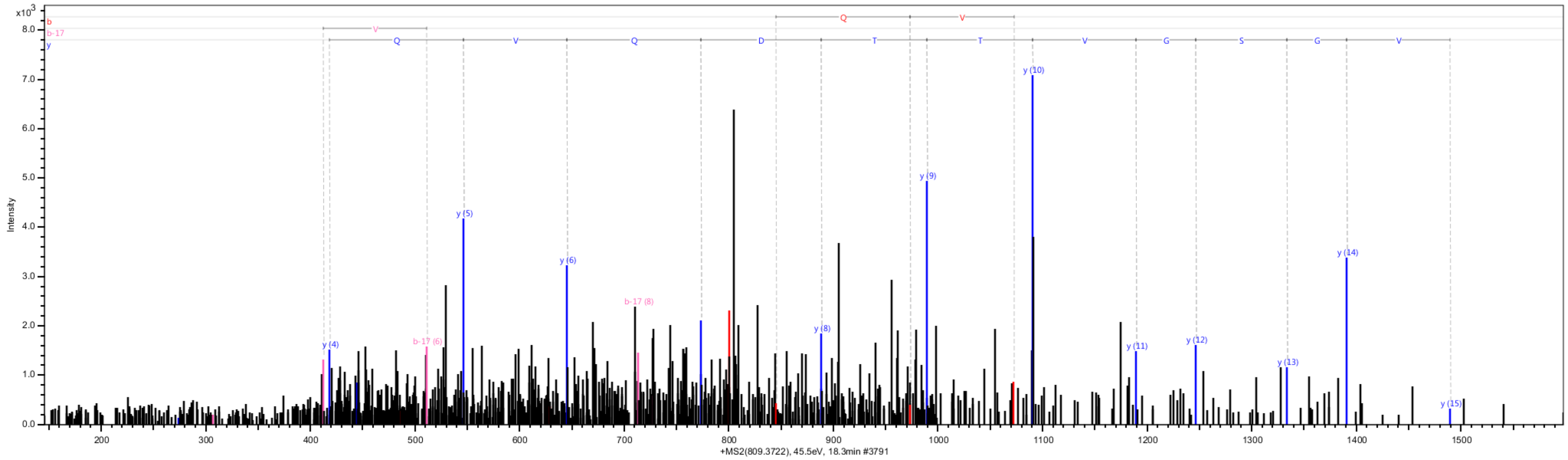

Peptide Match

**Sequence:**	R.VFAIPPSFASIFLTK.S	**Score:**	20.3
**Modifications:**			

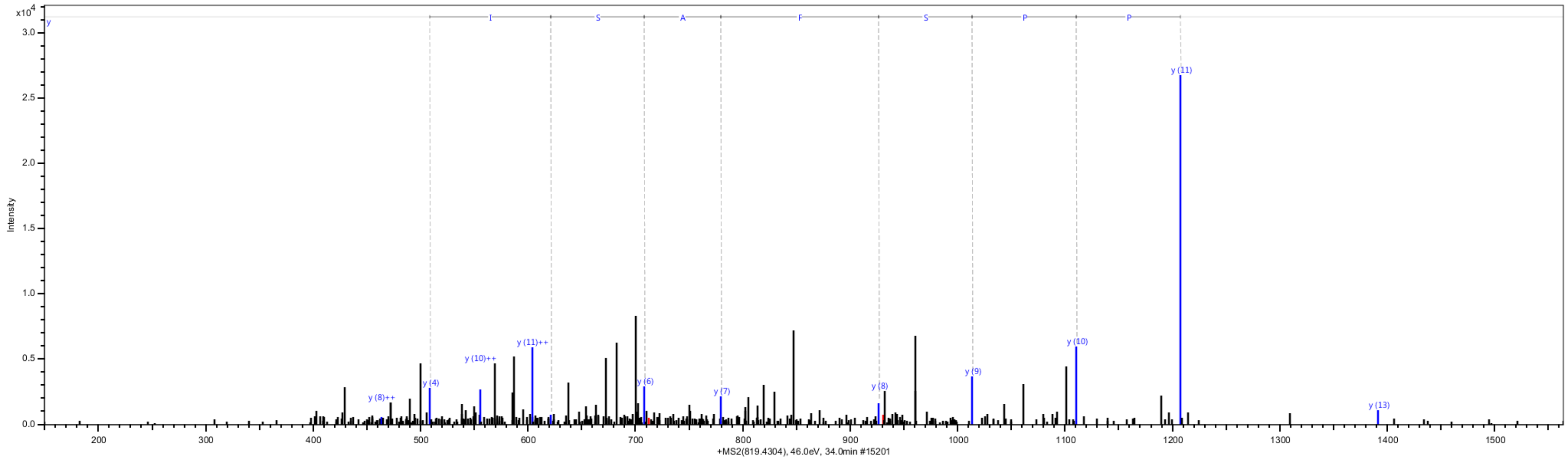

Peptide Match

Sequence: R.FTCTVTHTDLPSPLK.Q **Score:** 19.9
Modifications: Carbamidomethyl: 3

Spectrum Report
Combine Search_2020-10-19 07:53:31

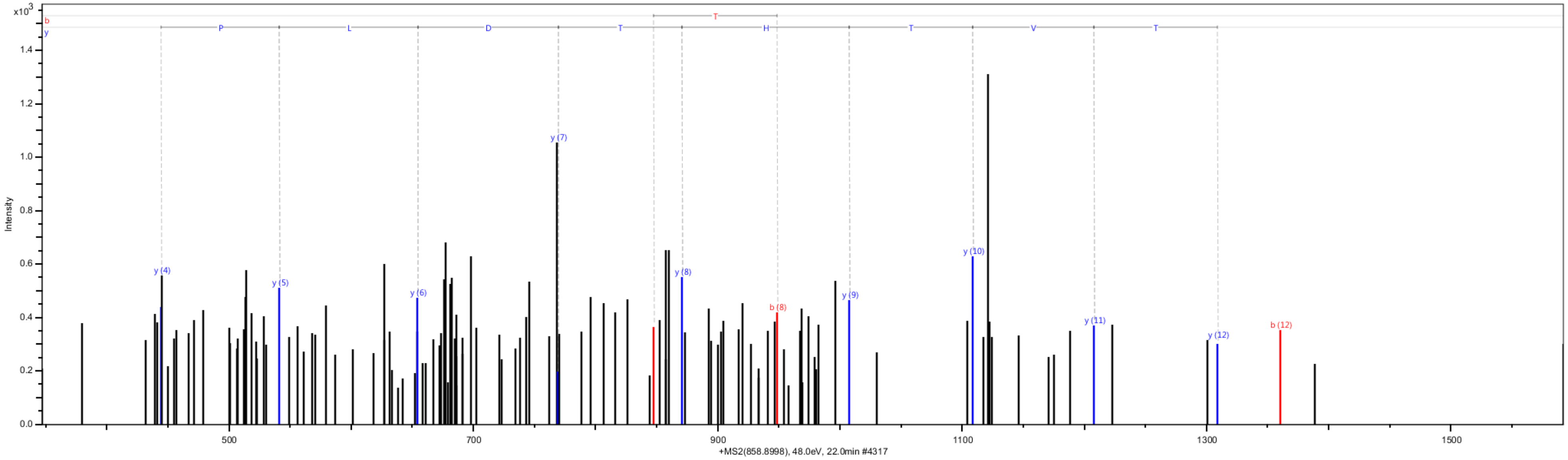

ZA2G_HUMAN

Accession: ZA2G_HUMAN
Database: SwissProt
MW [kDa] / pI: 34.2 / 5.7
Score: 206.5
Seq. Coverage [%]: 23.5
No. of Peptides: 6

Peptide Match

Sequence: R.YSLTYIYTGLSK.H
Score: 38.3
Modifications:

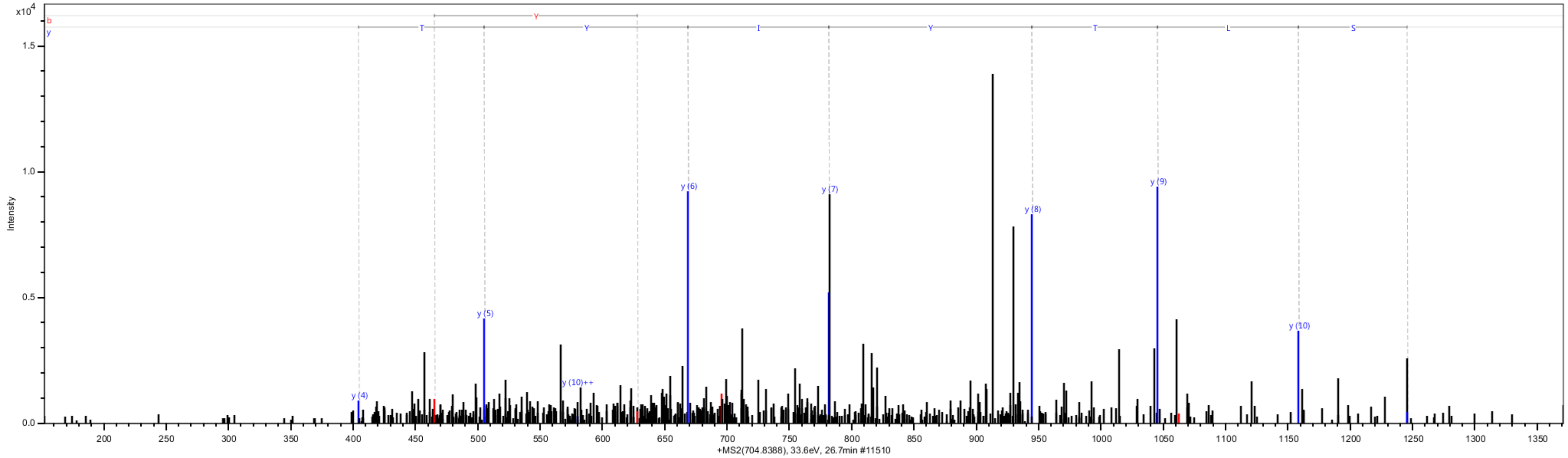

Peptide Match

Sequence: K.EIPAWVPFDPAAQITK.Q
Score: 20.7
Modifications:

Spectrum Report
Combine Search_2020-10-19 07:53:31

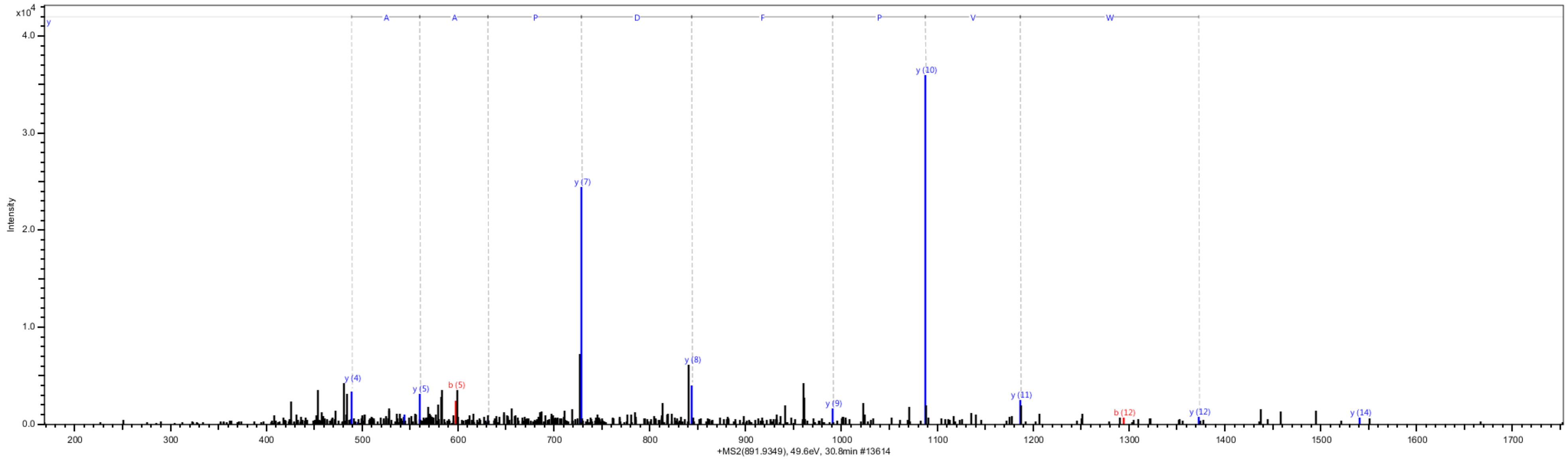

Peptide Match

**Sequence:**	K.QKWEAEPVYVQR.A	**Score:**	37
**Modifications:**			

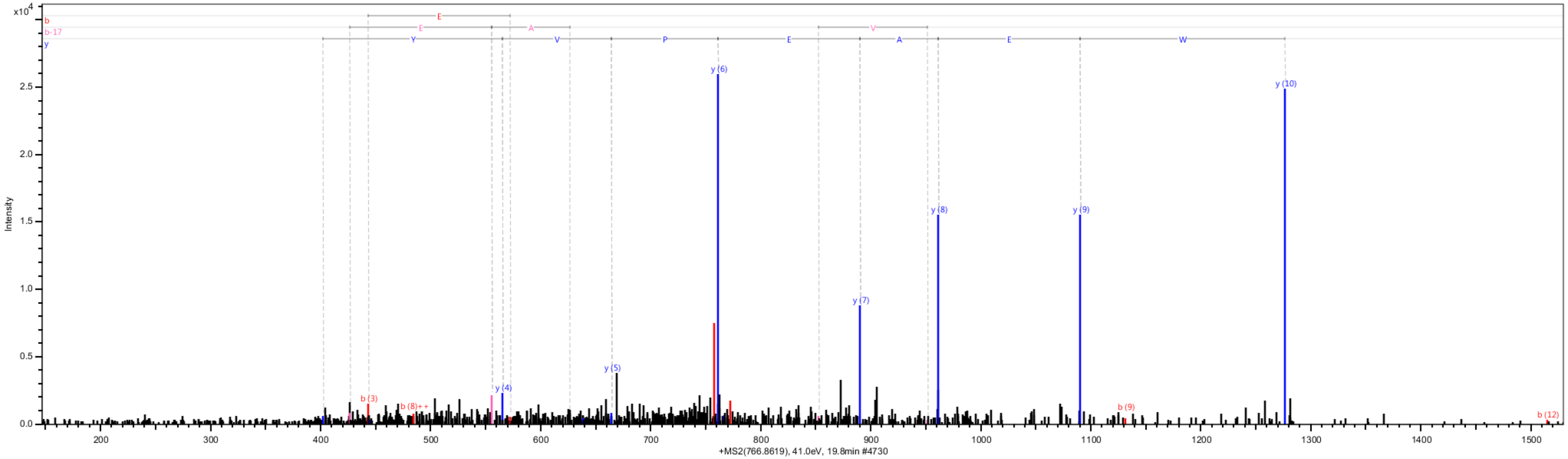

Peptide Match

**Sequence:**	K.AYLEEECPATLRK.Y	**Score:**	38.5
**Modifications:**	Carbamidomethyl: 7		

Spectrum Report
Combine Search_2020-10-19 07:53:31

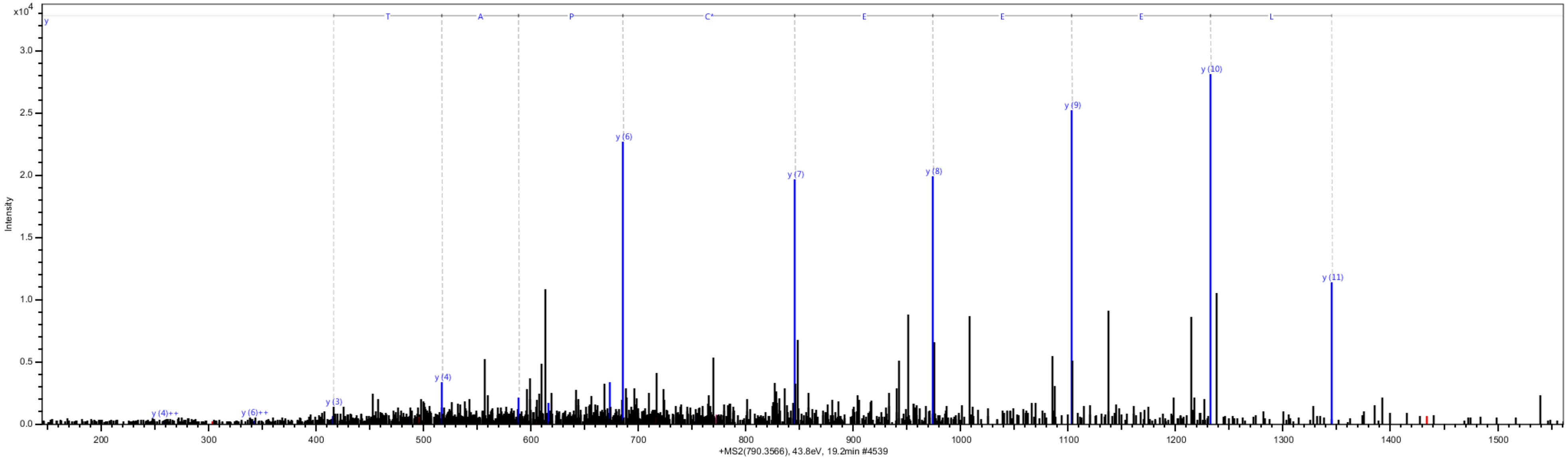

Peptide Match

Sequence: K.IDVHWTR.A **Score:** 17.8

Modifications:

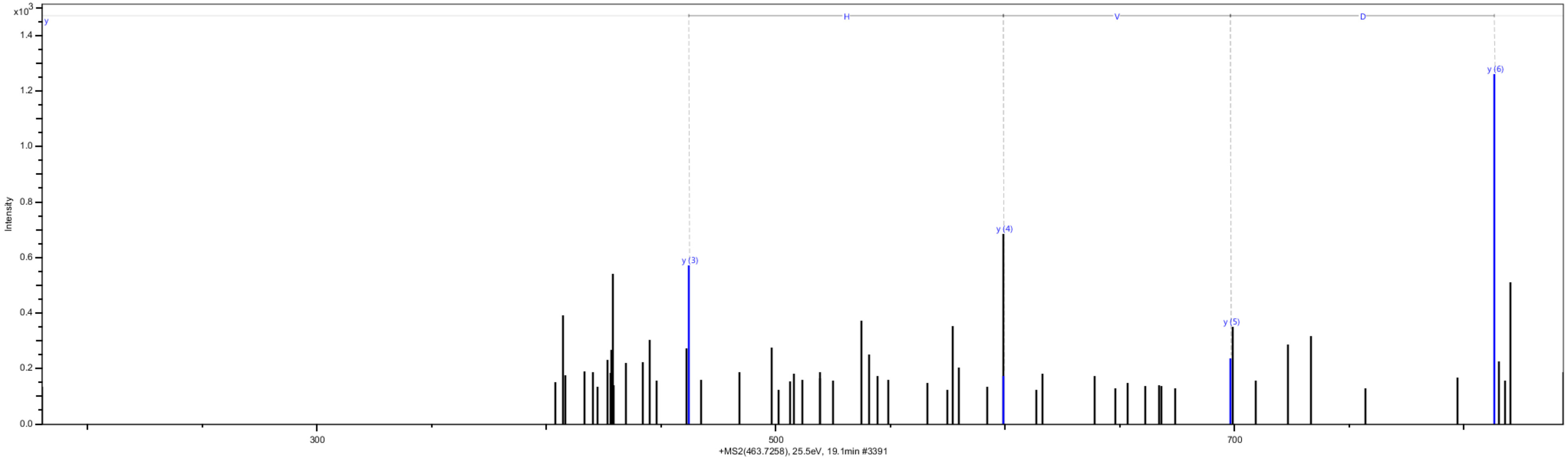

Peptide Match

**Sequence:**	R.AGEVQEPELR.G	**Score:**	54.2
**Modifications:**			

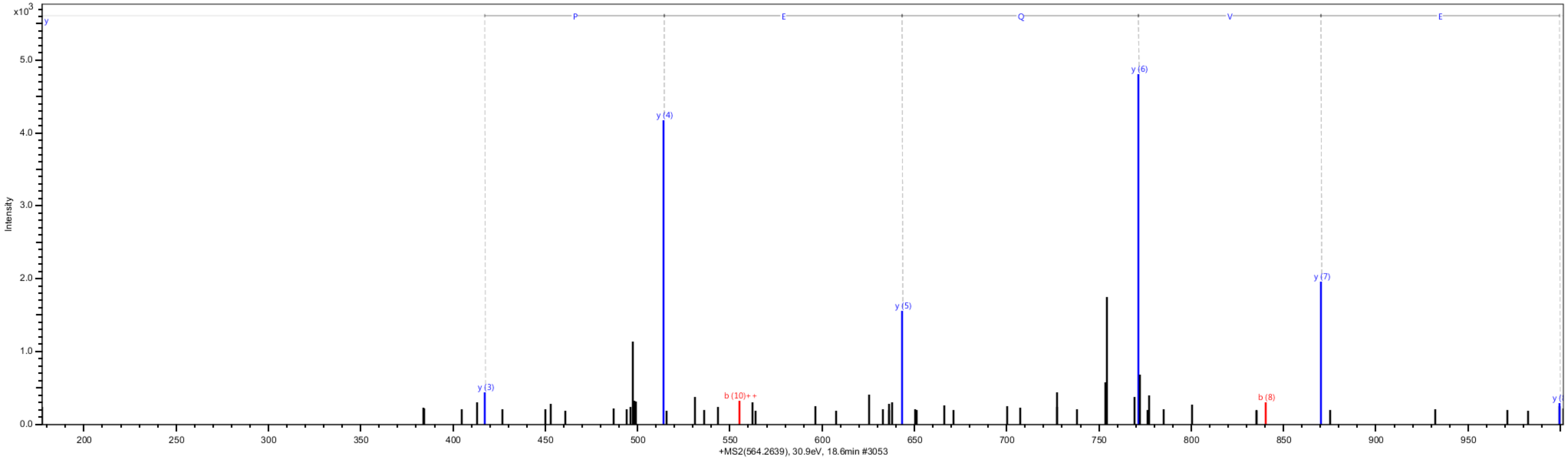
x10^3
Intensity
y
P
E
Q
V
E
y (3)
y (4)
b (10)++
y (5)
y (6)
y (7)
b (8)
5.0
4.0
3.0
2.0
1.0
0.0
200
250
300
350
400
450
500
550
600
650
700
750
800
850
900
950
+MS2(564.2639), 30.9eV, 18.6min #3053

IGM_HUMAN

**Accession:**	IGM_HUMAN	**Score:**	189.7
**Database:**	SwissProt	**Seq. Coverage [%]:**	1.2
**MW [kDa] / pI:**	63.4 / 8.1	**No. of Peptides:**	1

Peptide Match

**Sequence:**	K.ALEWLAR.I	**Score:**	24.9
**Modifications:**			

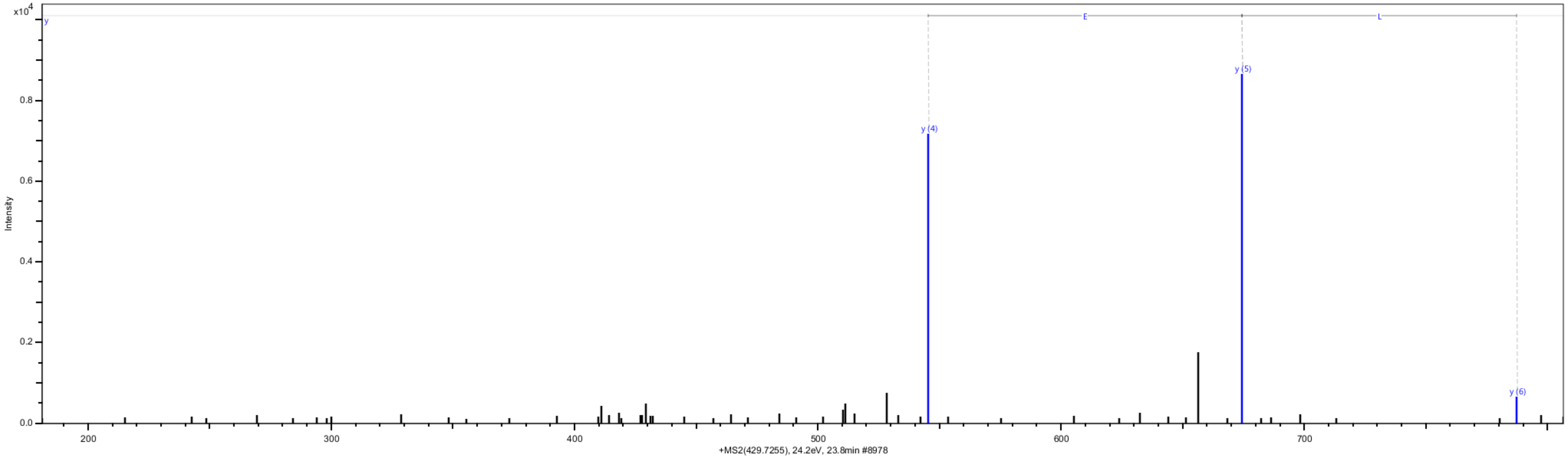

CFAH_HUMAN

Accession: CFAH_HUMAN
Database: SwissProt
MW [kDa] / pI: 139.0 / 6.2
Score: 186.9
Seq. Coverage [%]: 5.5
No. of Peptides: 6

Peptide Match

Sequence: K.CTSTGWIPAPR.C
Score: 48.7
Modifications: Carbamidomethyl: 1

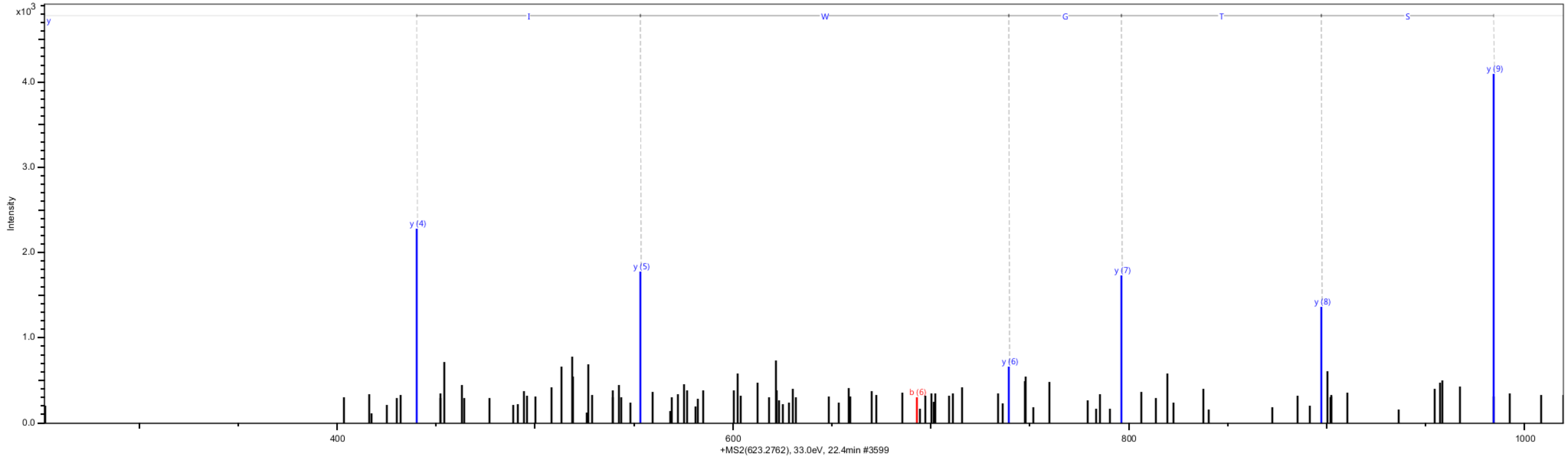

Peptide Match

Sequence: R.RPYFPVAVGK.Y
Score: 19.1
Modifications:

Spectrum Report
Combine Search_2020-10-19 07:53:31

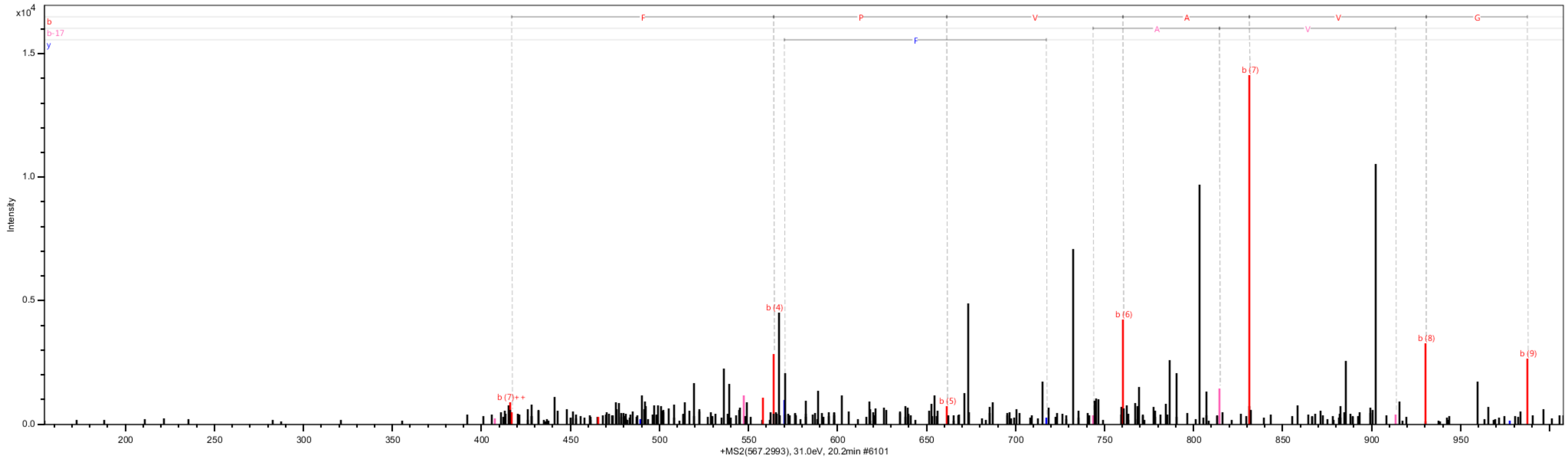

Peptide Match

**Sequence:**	K.SIDVACHPGYALPK.A	**Score:**	38.1
**Modifications:**	Carbamidomethyl: 6		

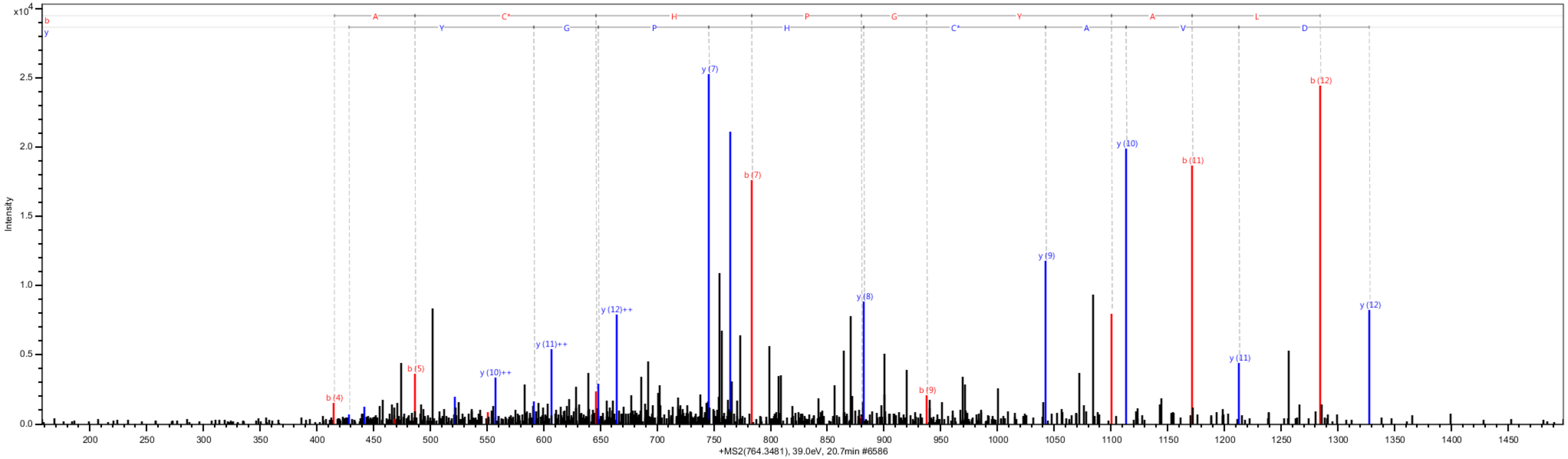

Peptide Match

**Sequence:**	K.SCDIPVFMNAR.T	**Score:**	30.6
**Modifications:**	Carbamidomethyl: 2		

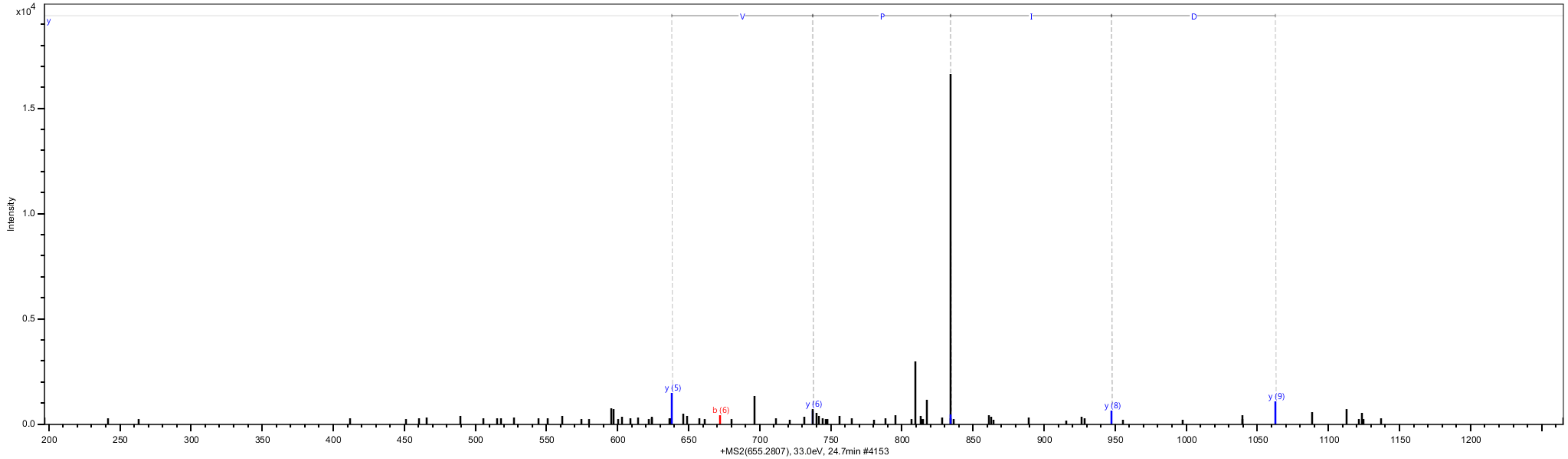

Peptide Match

**Sequence:**	K.SSNLIILEEHLK.N	**Score:**	23.5
**Modifications:**			

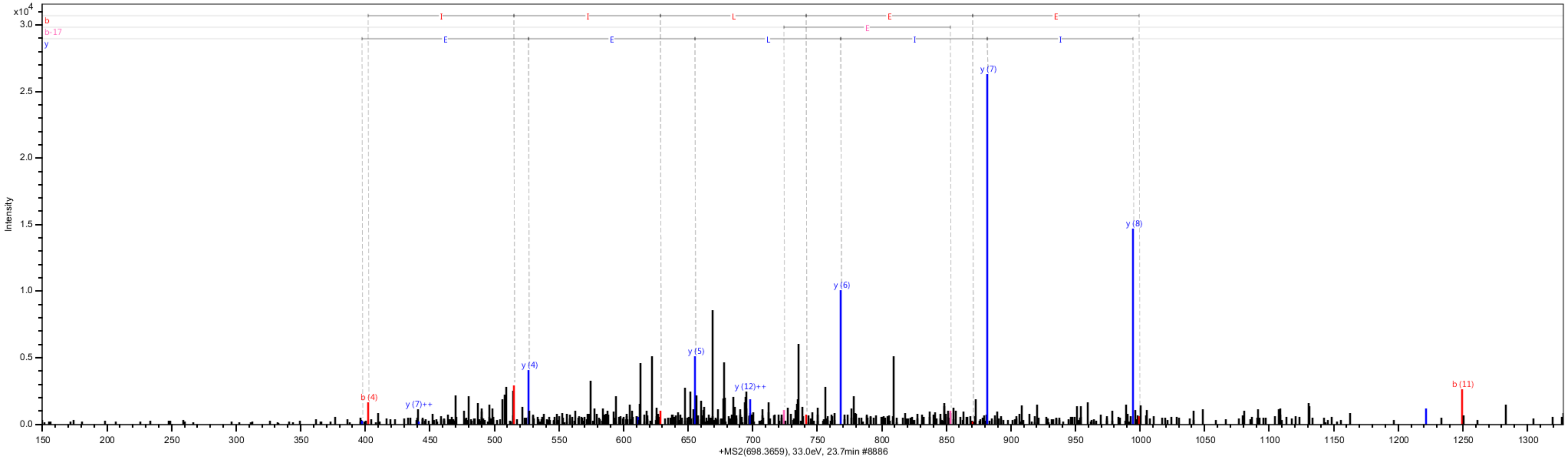

Peptide Match

Sequence: K.LSYTCEGGFR.I **Score:** 27
Modifications: Carbamidomethyl: 5

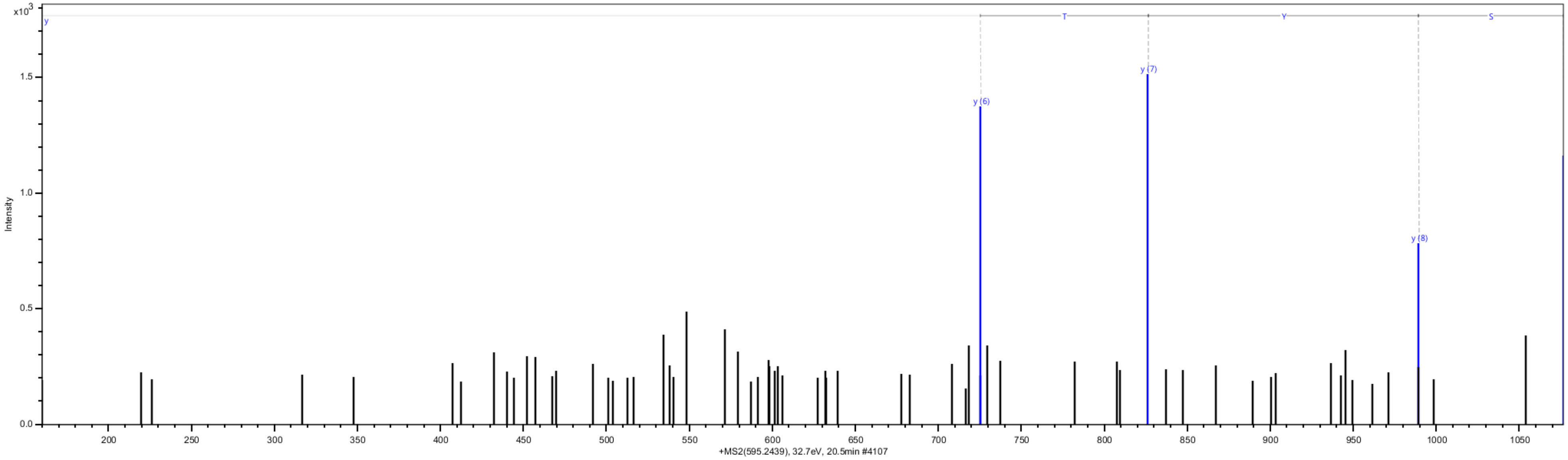
x10^3
Intensity
y
T
Y
S
y (6)
y (7)
y (8)
1.5
1.0
0.5
0.0
200
250
300
350
400
450
500
550
600
650
700
750
800
850
900
950
1000
1050
+MS2(595.2439), 32.7eV, 20.5min #4107

AMBP_HUMAN

Accession: AMBP_HUMAN
Database: SwissProt
MW [kDa] / pI: 39.0 / 5.9

Score: 132.0
Seq. Coverage [%]: 7.7
No. of Peptides: 3

Peptide Match

Sequence: R.TVAACNLPIVR.G
Score: 47.6
Modifications: Carbamidomethyl: 5

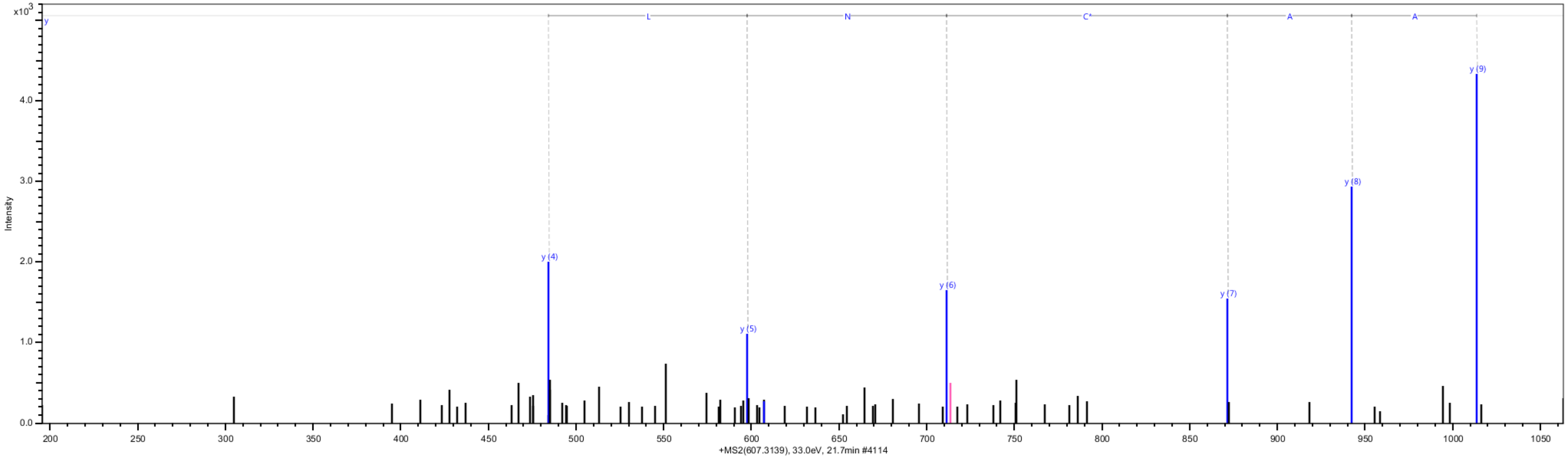

Peptide Match

Sequence: R.TVAACNLPIVRGPCR.A
Score: 18.2
Modifications: Carbamidomethyl: 5, 14

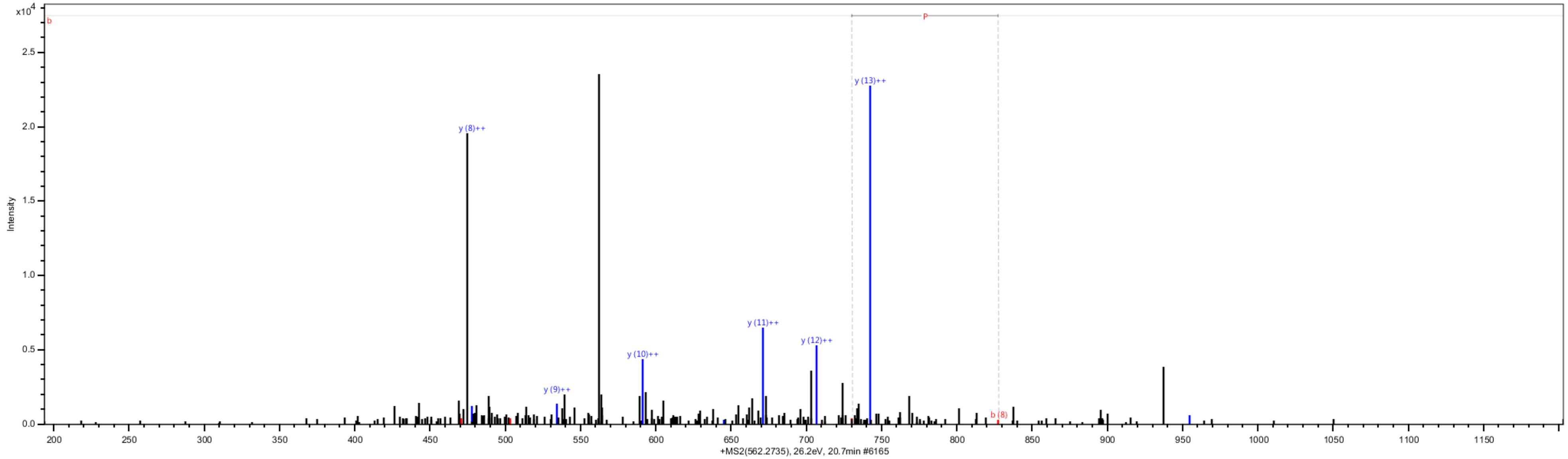

Peptide Match

**Sequence:**	R.AFIQLWAFDAVK.G	**Score:**	66.2
**Modifications:**			

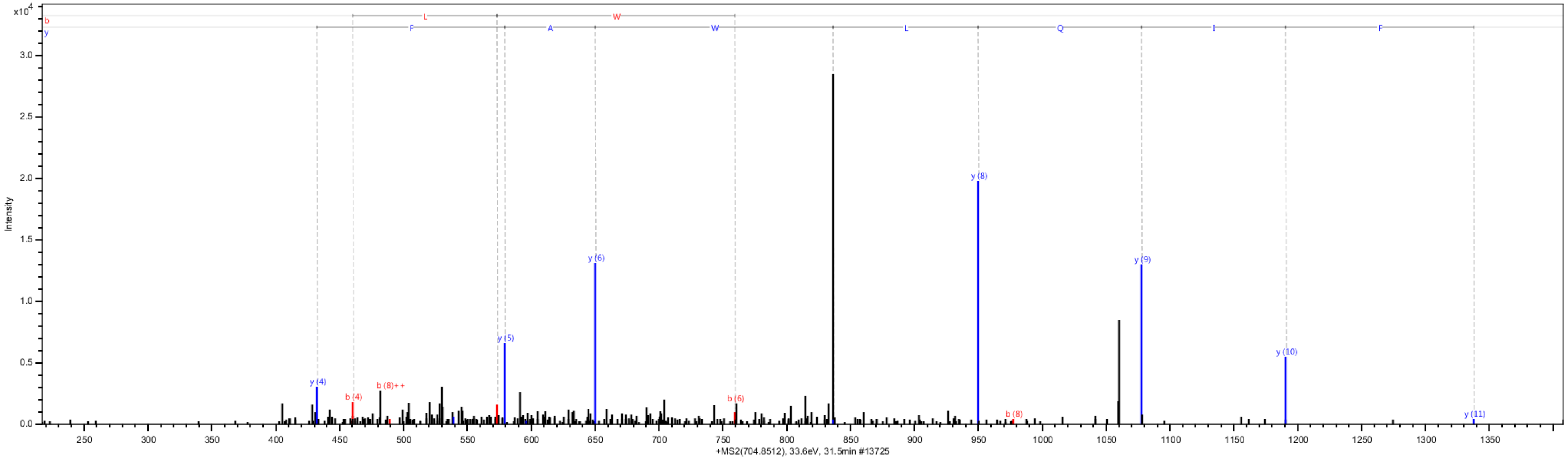
x10^4
b
y
L
W
F
A
W
L
Q
I
F
Intensity
y (4)
b (4)
b (8)++
y (5)
y (6)
b (6)
y (8)
b (8)
y (9)
y (10)
y (11)
+MS2(704.8512), 33.6eV, 31.5min #13725

THRB_HUMAN

Accession: THRB_HUMAN
Database: SwissProt
MW [kDa] / pI: 70.0 / 5.6
Score: 133.8
Seq. Coverage [%]: 5.1
No. of Peptides: 3

Peptide Match

Sequence: R.SGIECQLWR.S
Score: 63.3
Modifications: Carbamidomethyl: 5

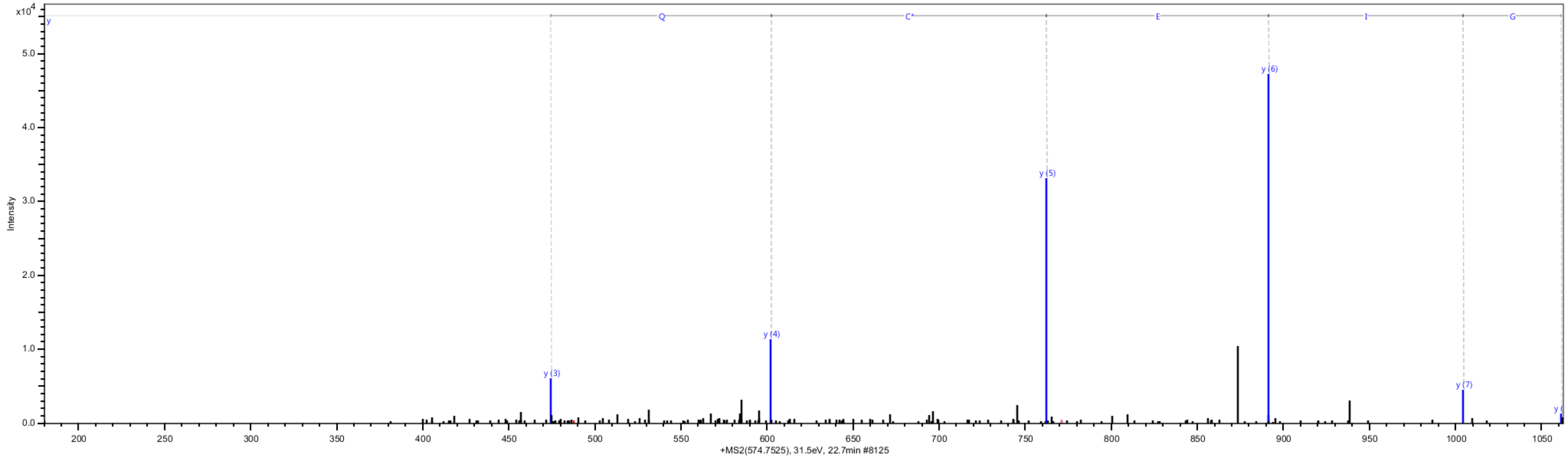

Peptide Match

Sequence: R.TATSEYQTFFNPR.T
Score: 44.3
Modifications:

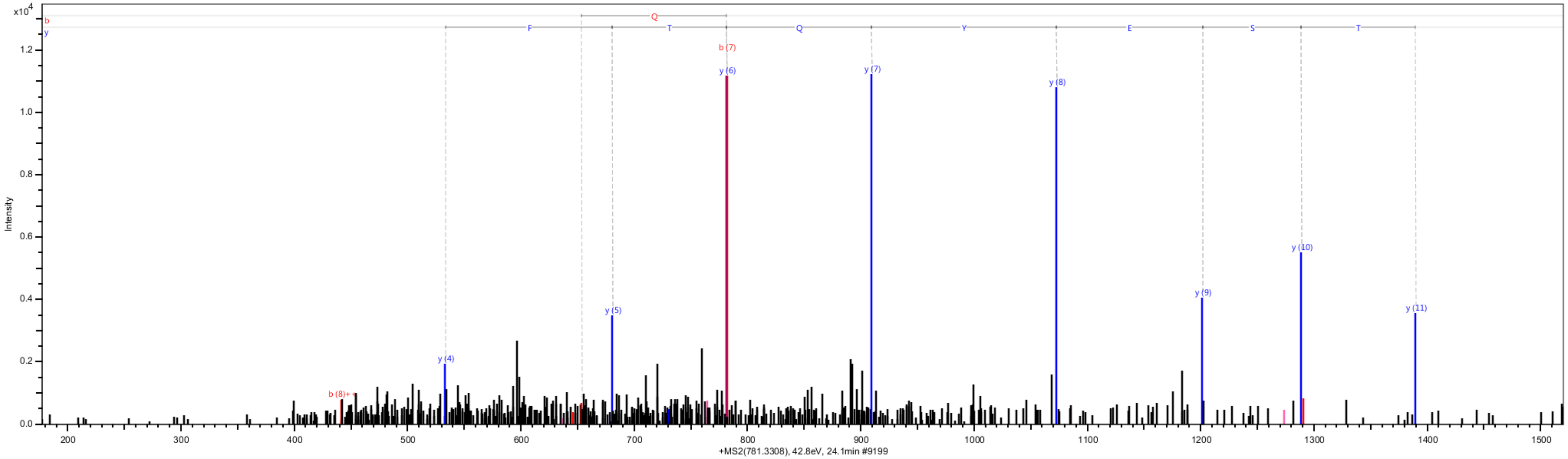

Peptide Match

**Sequence:**	R.ELLESYIDGR.I	**Score:**	26.2
**Modifications:**			

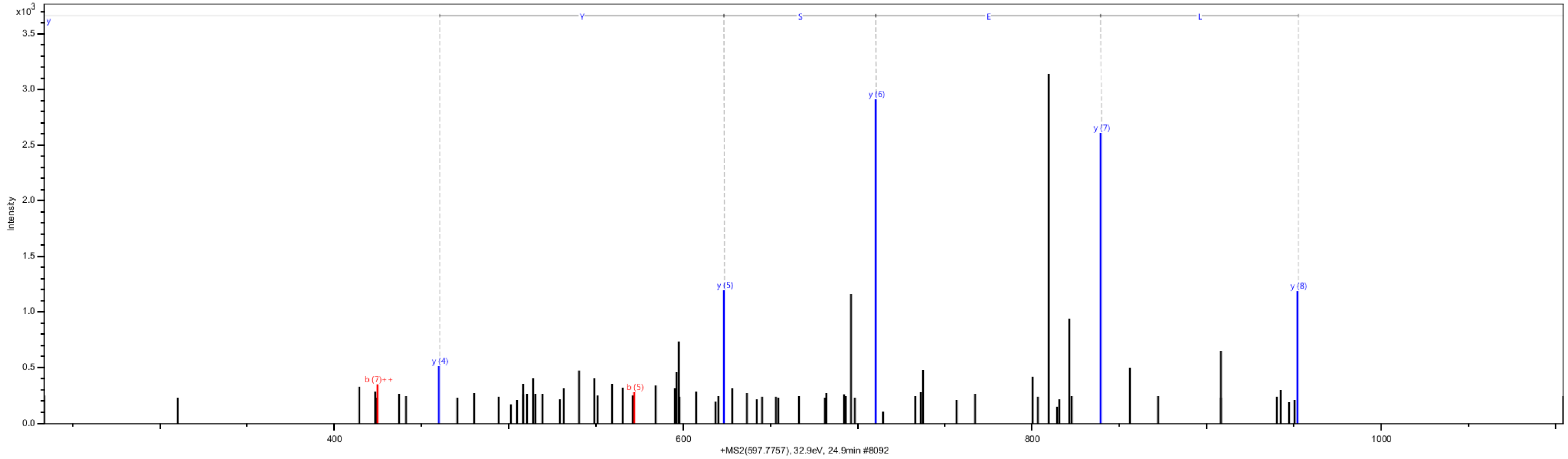
x10^3
Intensity
3.5
3.0
2.5
2.0
1.5
1.0
0.5
0.0
Y
S
E
L
y
b (7)++
y (4)
b (5)
y (5)
y (6)
y (7)
y (8)
400
600
800
1000
+MS2(597.7757), 32.9eV, 24.9min #8092

ITIH2_HUMAN

**Accession:**	ITIH2_HUMAN	**Score:**	139.8
**Database:**	SwissProt	**Seq. Coverage [%]:**	4.1
**MW [kDa] / pI:**	106.4 / 6.4	**No. of Peptides:**	3

Peptide Match

**Sequence:**	K.AHVSFKPTVAQQR.I	**Score:**	58.2
**Modifications:**			

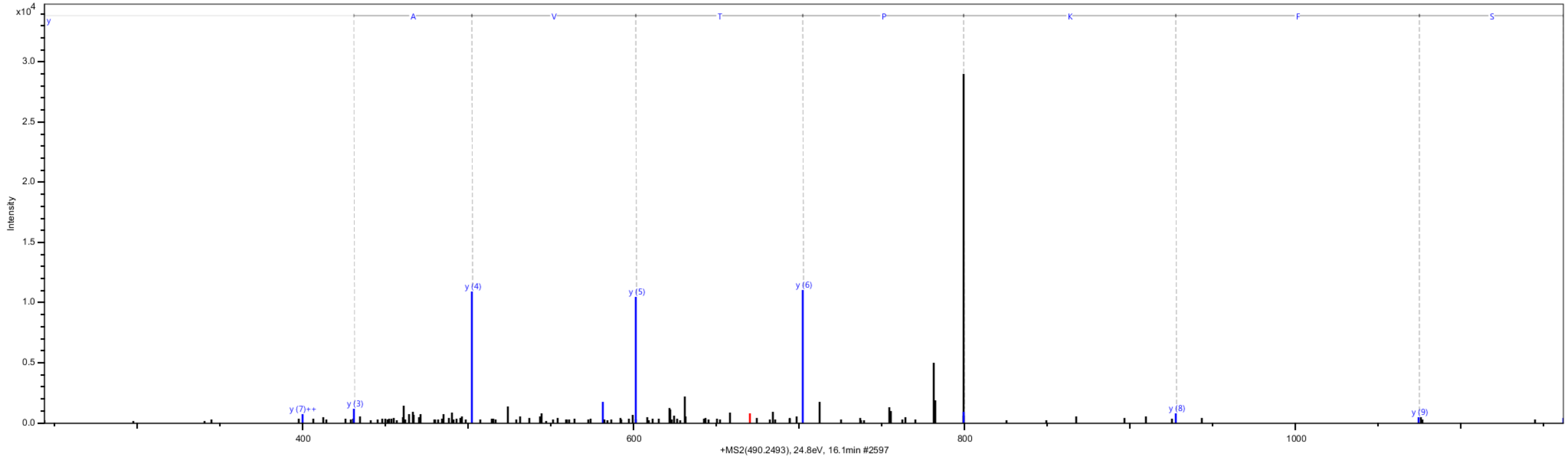

Peptide Match

**Sequence:**	K.IQPSGGTNINEALLR.A	**Score:**	30.2
**Modifications:**			

Spectrum Report
Combine Search_2020-10-19 07:53:31

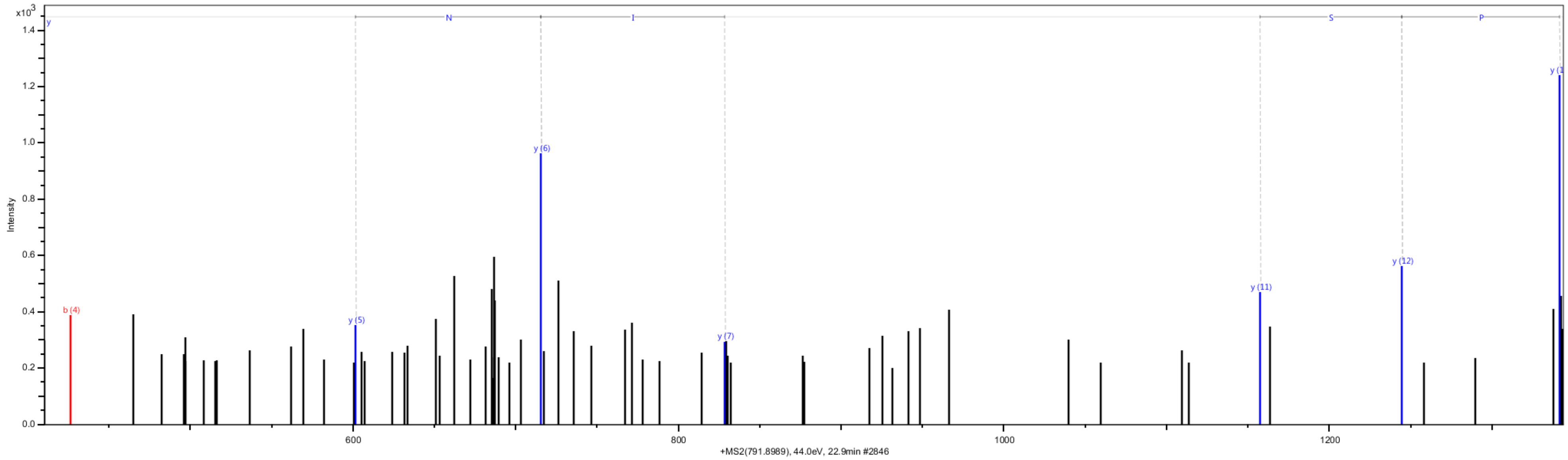

Peptide Match

Sequence: K.FYNQVSTPLLR.N **Score:** 51.4

Modifications:

Spectrum Report
Combine Search_2020-10-19 07:53:31

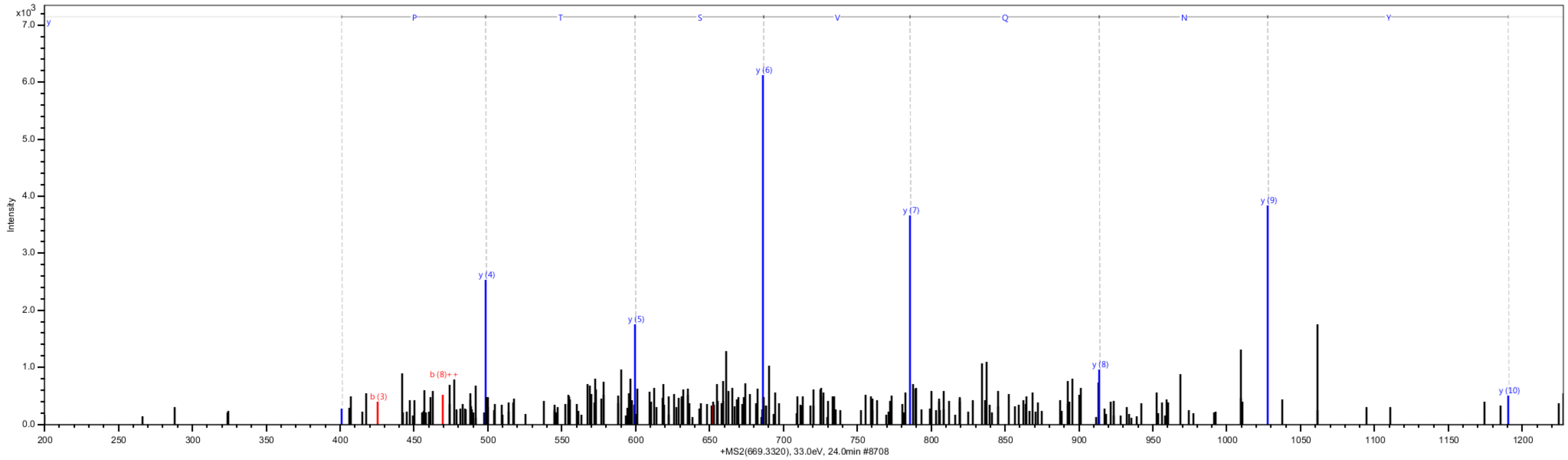

A1AG2_HUMAN

Accession: A1AG2_HUMAN
Database: SwissProt
MW [kDa] / pI: 23.6 / 5.0
Score: 141.6
Seq. Coverage [%]: 11.9
No. of Peptides: 3

Peptide Match

Sequence: R.YEGGREHVAHLLFLR.D
Score: 17.1
Modifications:

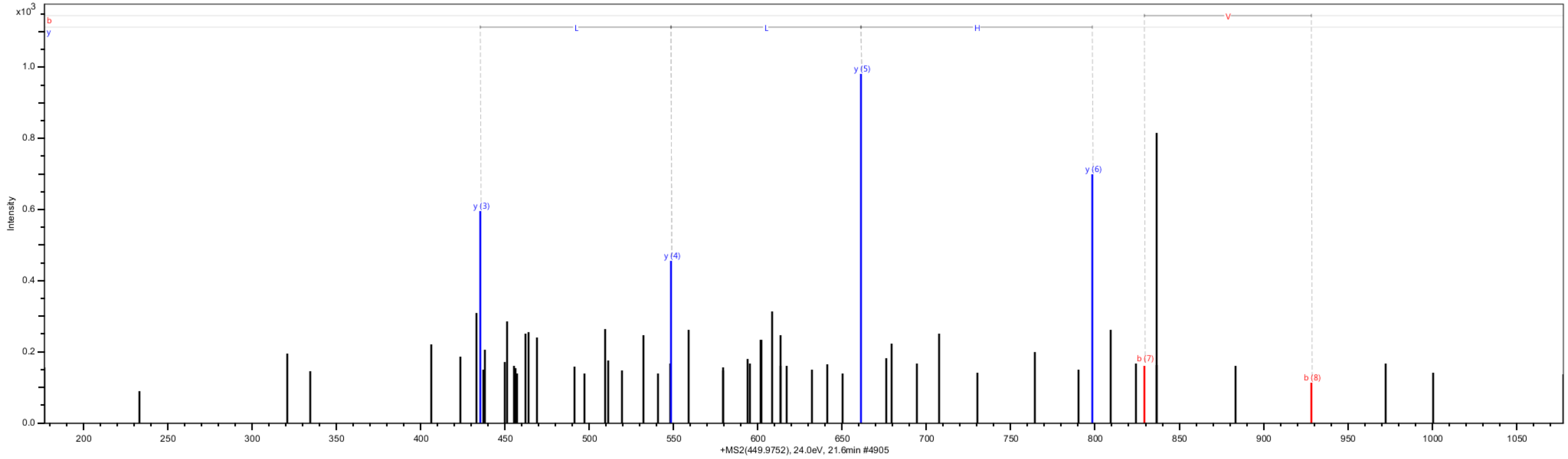

Peptide Match

Sequence: R.EHVAHLLFLR.D
Score: 42.5
Modifications:

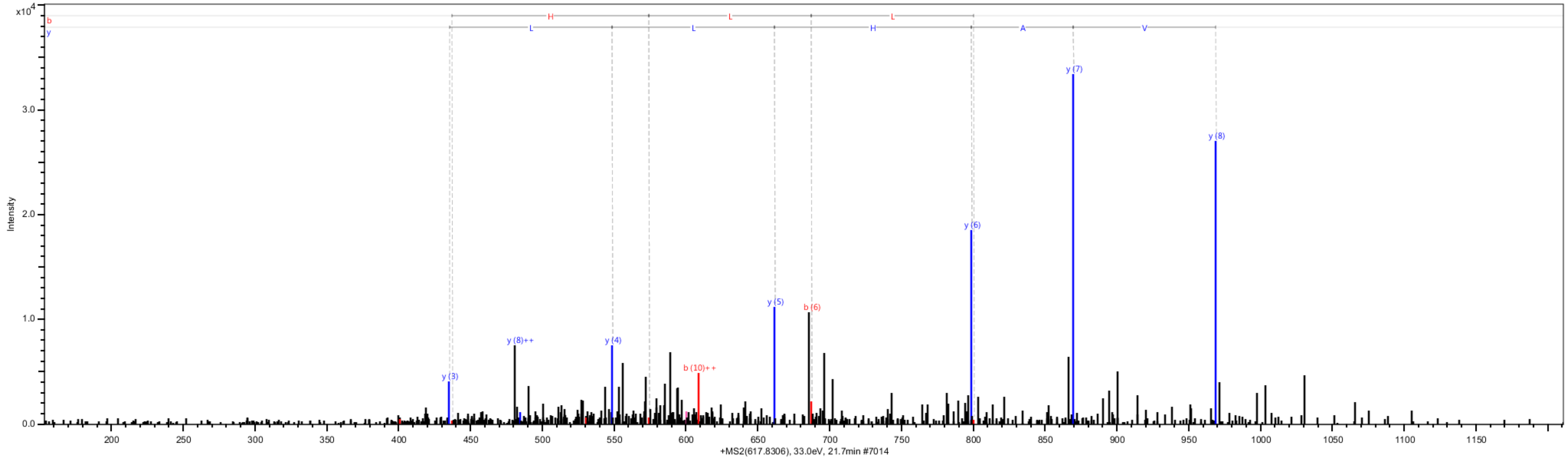

Peptide Match

**Sequence:**	R.SDVMYTDWK.K	**Score:**	17.1
**Modifications:**			

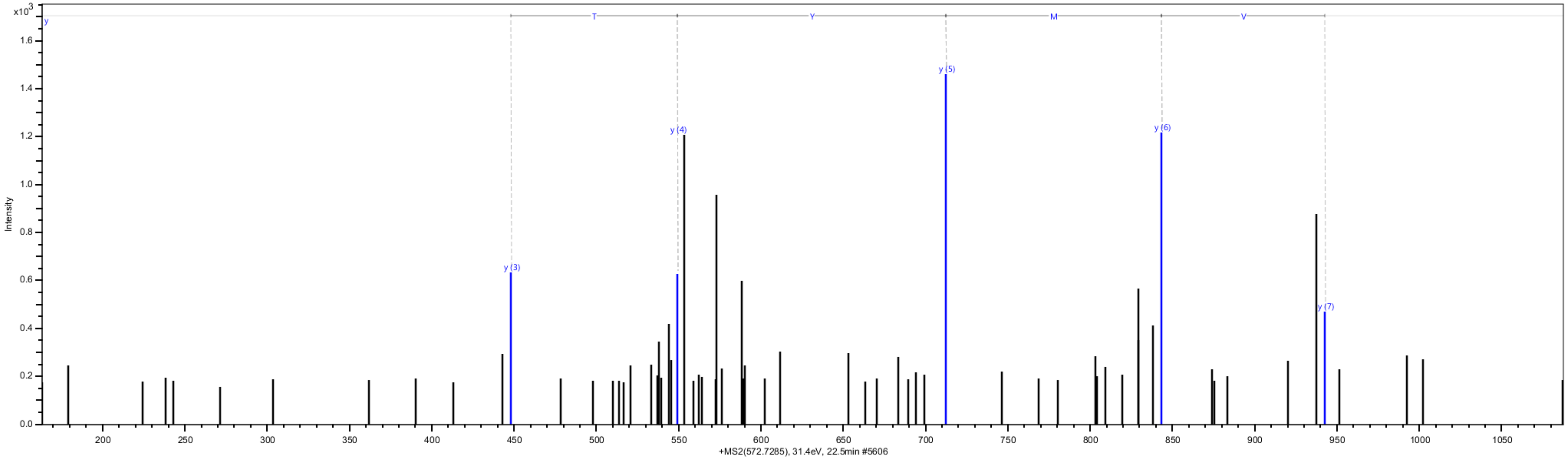
x10^3
Intensity
y
T
Y
M
V
y (3)
y (4)
y (5)
y (6)
y (7)
+MS2(572.7285), 31.4eV, 22.5min #5606

APOH_HUMAN

**Accession:**	APOH_HUMAN	**Score:**	143.8
**Database:**	SwissProt	**Seq. Coverage [%]:**	11.9
**MW [kDa] / pI:**	38.3 / 8.3	**No. of Peptides:**	4

Peptide Match

**Sequence:**	K.FICPLTGLWPINTLK.C	**Score:**	34.2
**Modifications:**	Carbamidomethyl: 3		

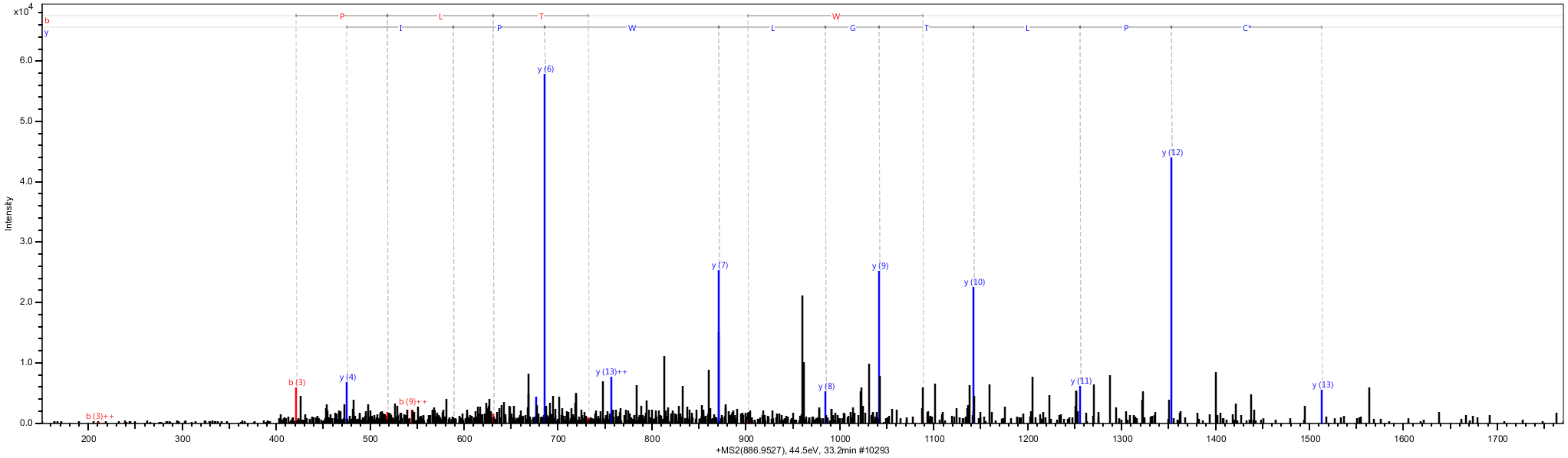

Peptide Match

**Sequence:**	R.VCPFAGILENGAVR.Y	**Score:**	68.8
**Modifications:**	Carbamidomethyl: 2		

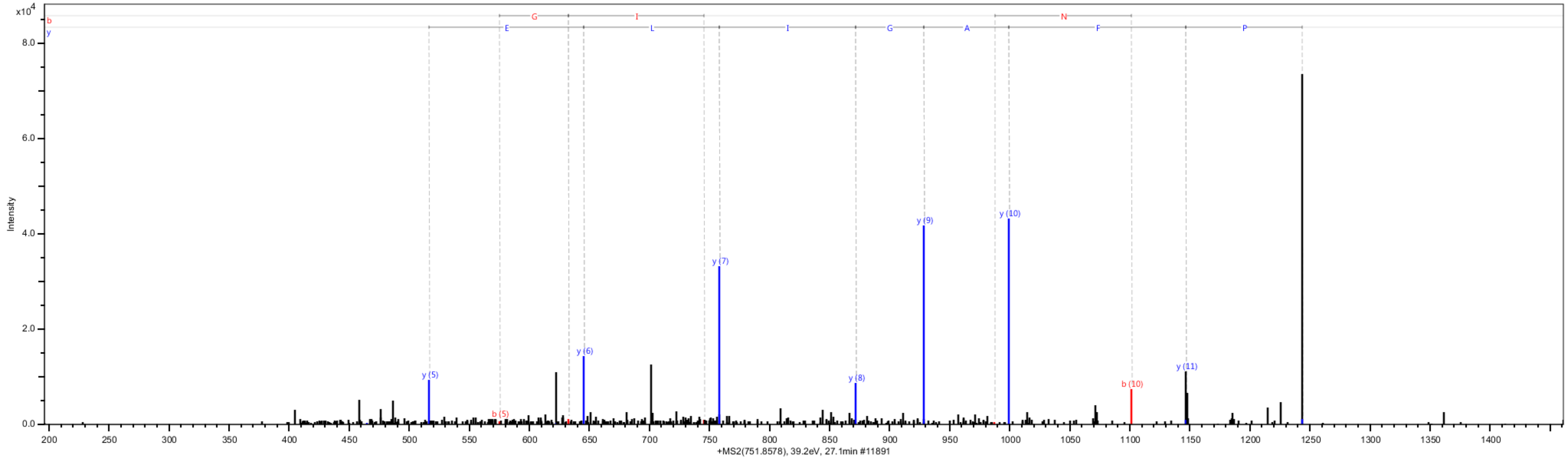

Peptide Match

**Sequence:**	K.CFKEHSSLAFWK.T	**Score:**	20.3
**Modifications:**	Carbamidomethyl: 1		

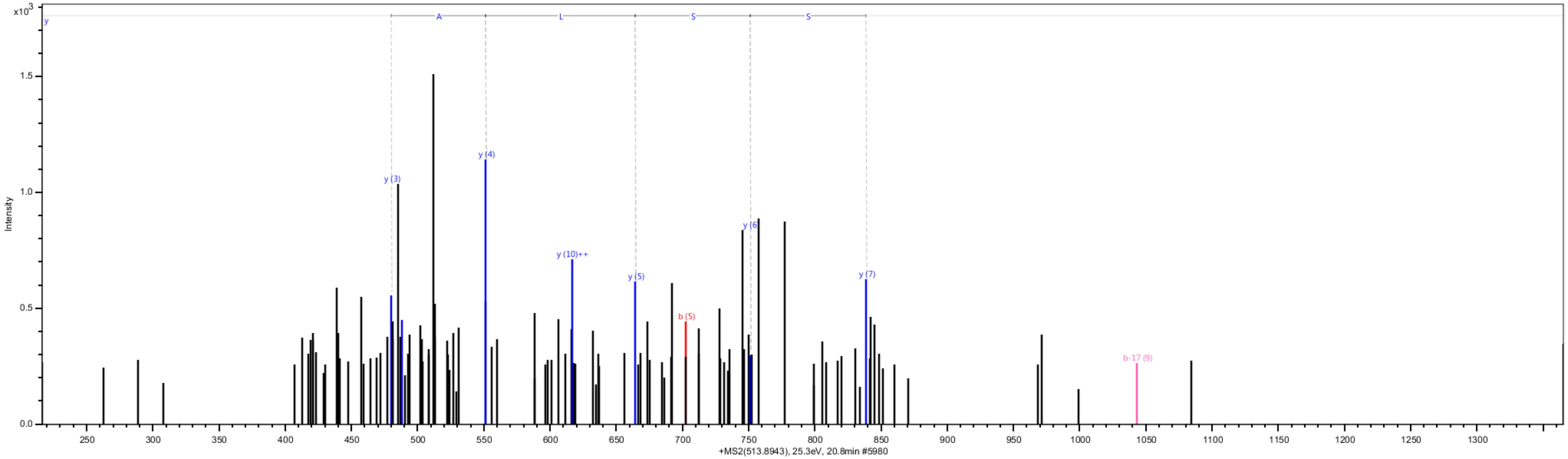

Peptide Match

Sequence: K.EHSSLAFWK.T **Score:** 20.5

Modifications:

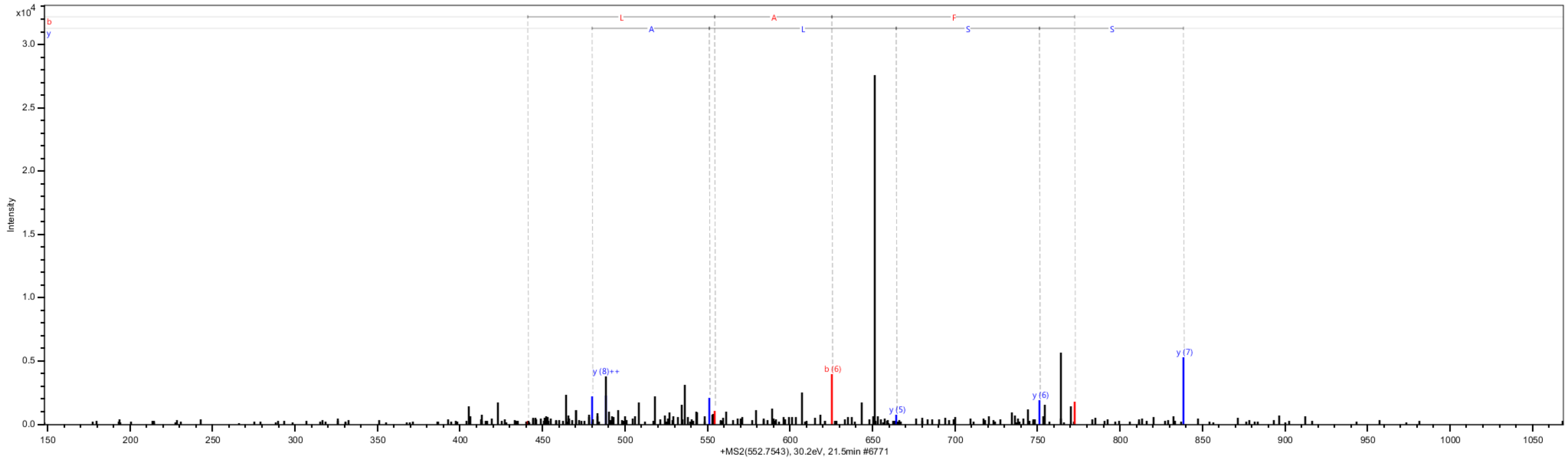
x10^4
Intensity
b
y
L
A
F
A
L
S
S
y (8)++
b (6)
y (5)
y (6)
y (7)
+MS2(552.7543), 30.2eV, 21.5min #6771

POTEE_HUMAN

Accession: POTEE_HUMAN
Database: SwissProt
MW [kDa] / pI: 121.3 / 5.8
Score: 132.5
Seq. Coverage [%]: 4.0
No. of Peptides: 4

Peptide Match

Sequence: K.AGFAGDDAPR.A
Score: 26.3
Modifications:

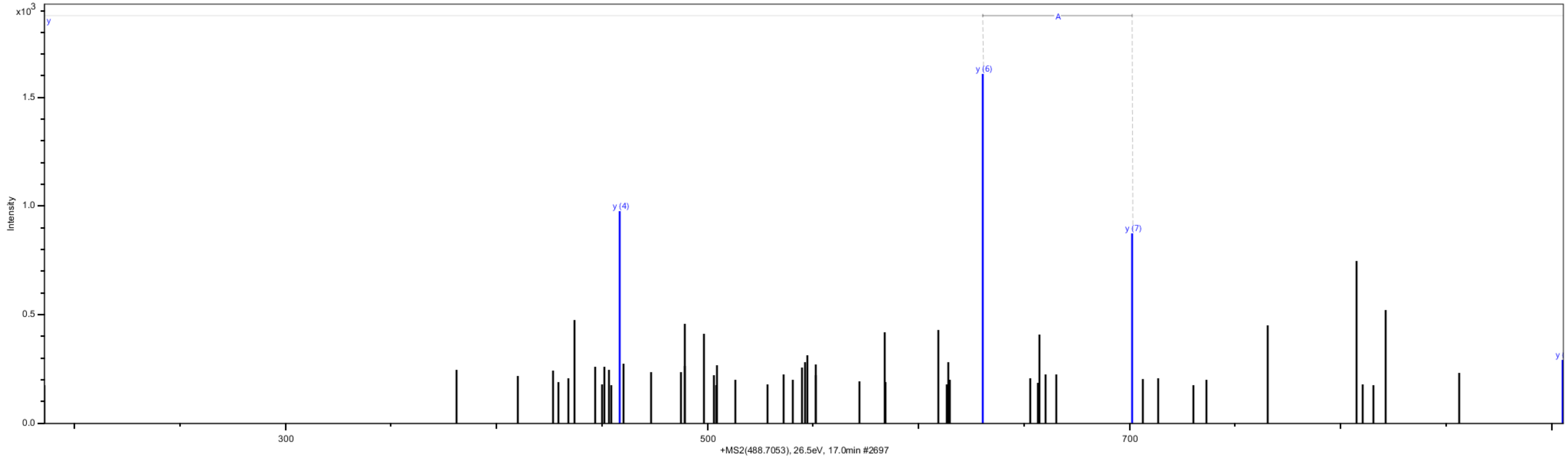

Peptide Match

Sequence: R.AVFPSIVGRPR.Q
Score: 45.3
Modifications:

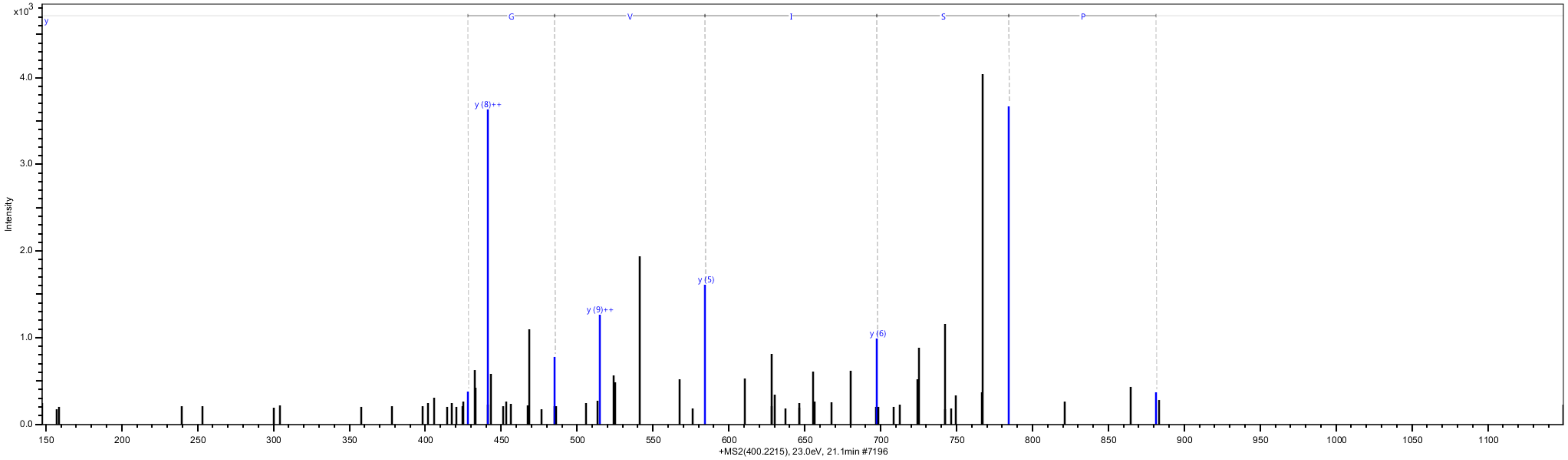

Peptide Match

**Sequence:**	K.IWHHTFYNELR.V	**Score:**	19.1
**Modifications:**			

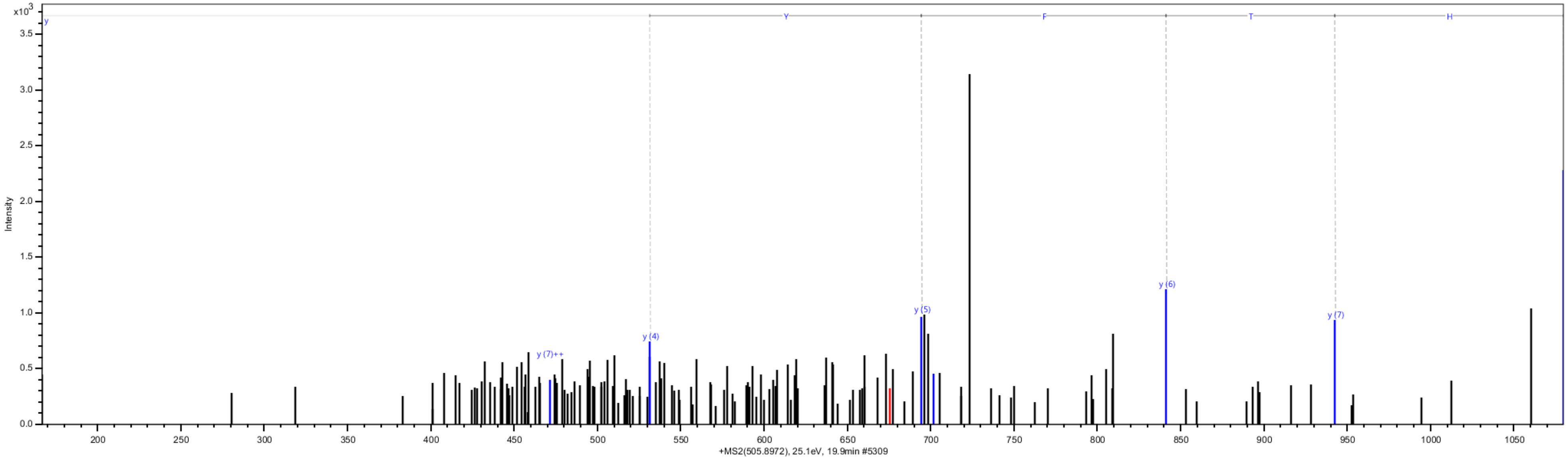

Peptide Match

**Sequence:**	K.EIAALAPSMMK.I	**Score:**	19.4
**Modifications:**	Oxidation: 9, 10		

Spectrum Report
Combine Search_2020-10-19 07:53:31

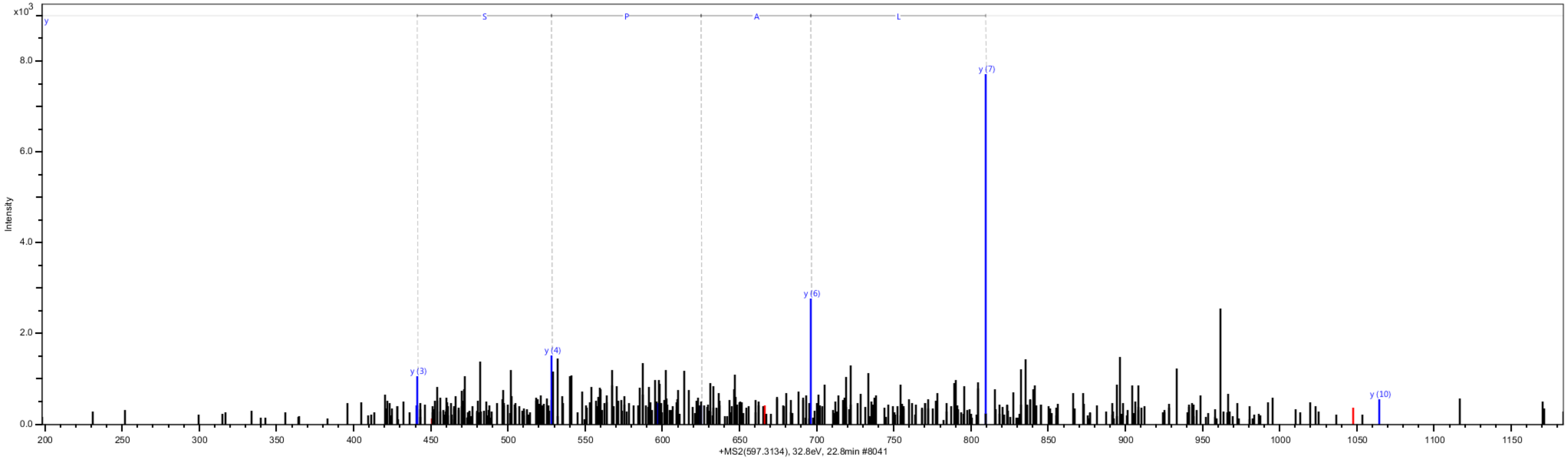

KV108_HUMAN

Accession: KV108_HUMAN
Database: SwissProt
MW [kDa] / pI: 12.5 / 9.2
Score: 78.6
Seq. Coverage [%]: 13.9
No. of Peptides: 1

Peptide Match

Sequence: K.LLIYAASTLQSGVPSR.F
Score: 78.6
Modifications:

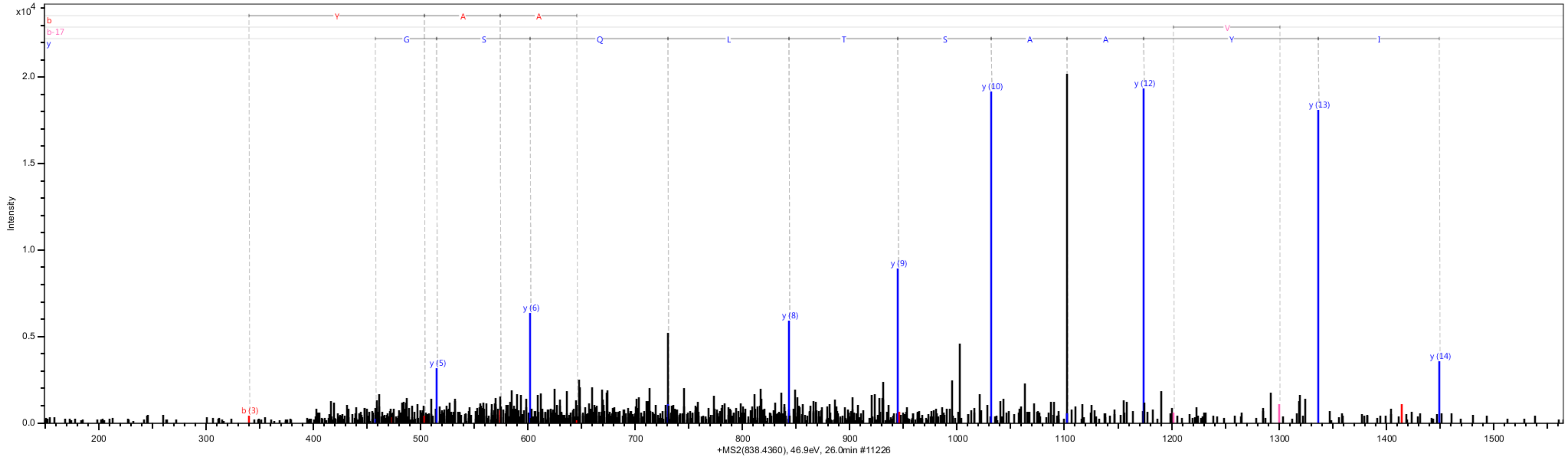

FIBA_HUMAN

Accession: FIBA_HUMAN
Database: SwissProt
MW [kDa] / pI: 94.9 / 5.7
Score: 75.8
Seq. Coverage [%]: 4.0
No. of Peptides: 3

Peptide Match

Sequence: K.GLIDEVNQDFTNR.I
Score: 24.2
Modifications:

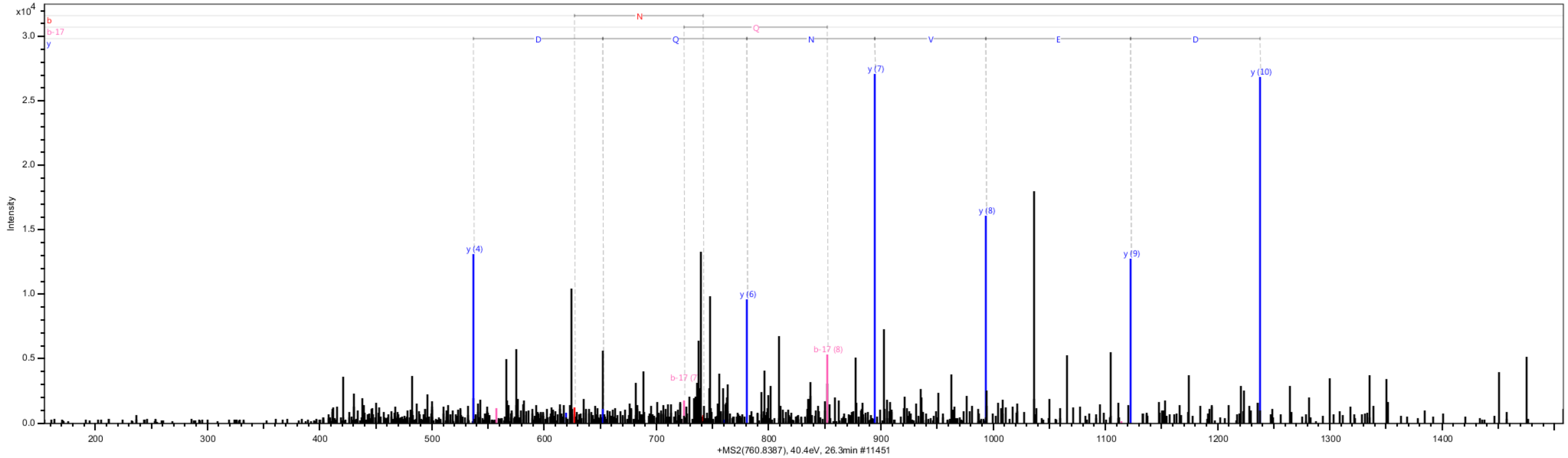

Peptide Match

Sequence: K.NSLFEYQK.N
Score: 22.8
Modifications:

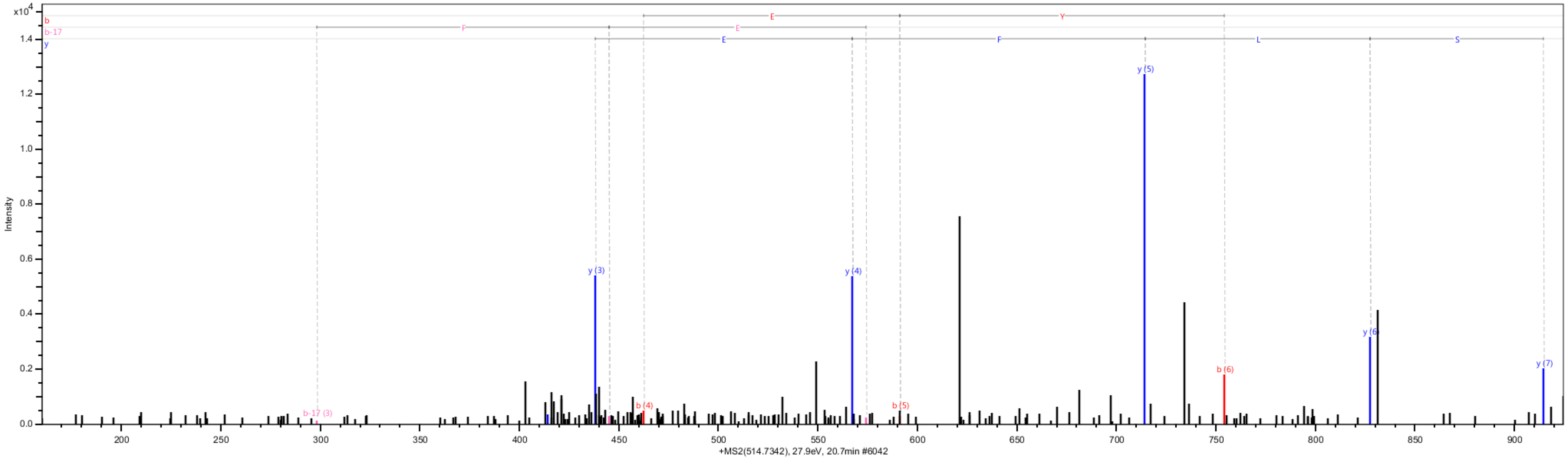

Peptide Match

Sequence: K.DSHSLTTNIMEILR.G **Score:** 28.8

Modifications:

Spectrum Report
Combine Search_2020-10-19 07:53:31

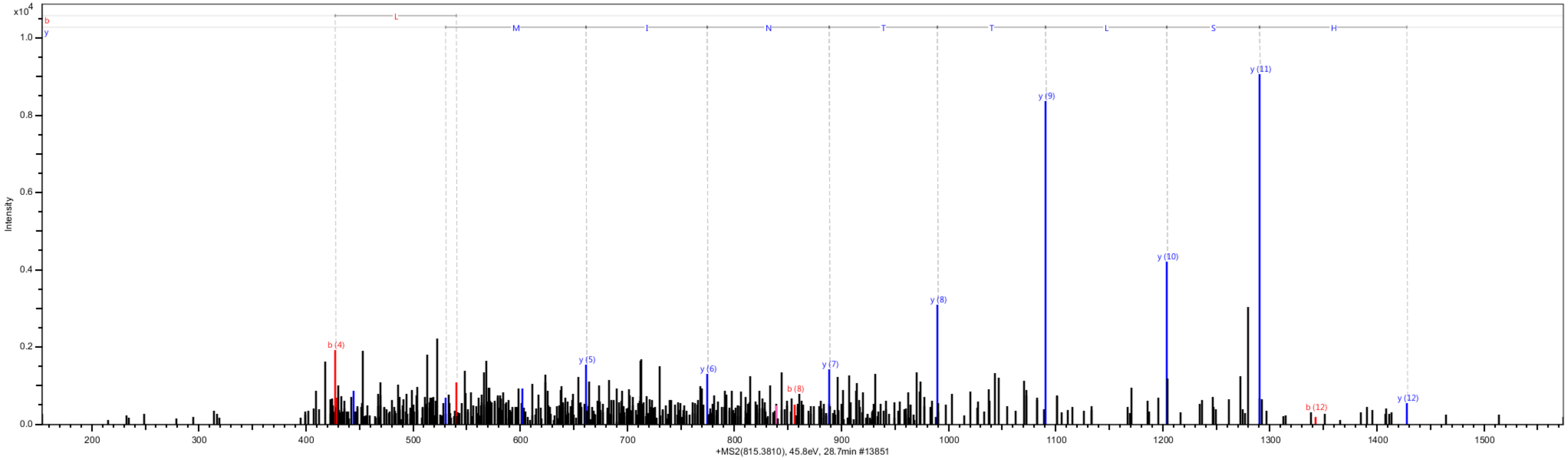

FA12_HUMAN

Accession: FA12_HUMAN
Database: SwissProt
MW [kDa] / pI: 67.7 / 8.0
Score: 74.5
Seq. Coverage [%]: 5.7
No. of Peptides: 3

Peptide Match

Sequence: R.NWGLGGHAFCR.N
Score: 29.4
Modifications: Carbamidomethyl: 10

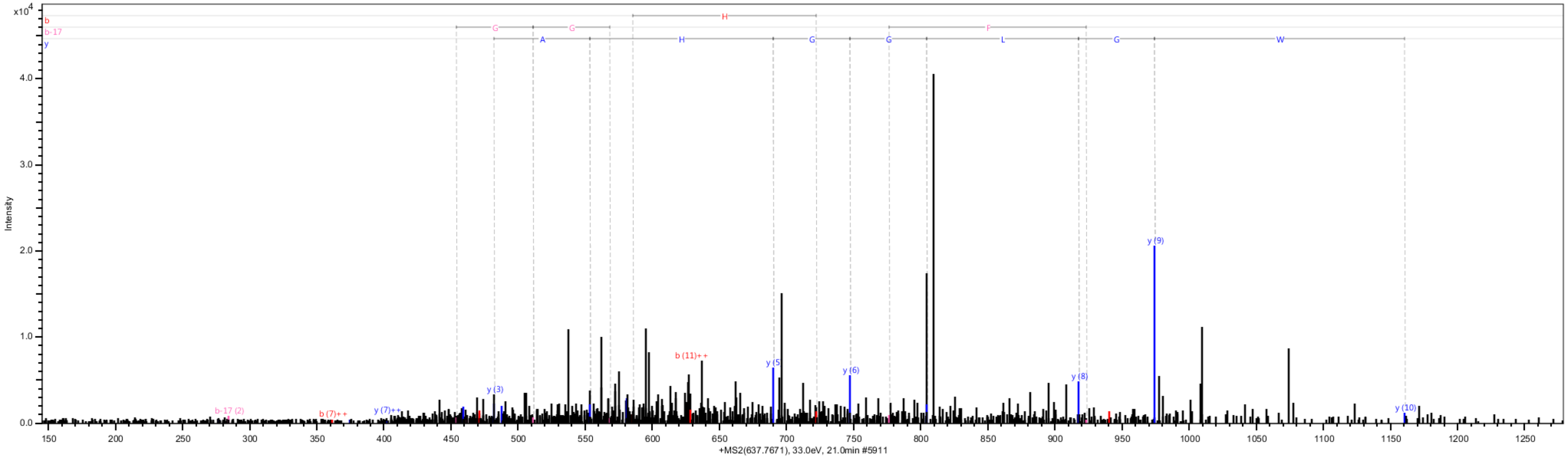

Peptide Match

Sequence: R.NPDNDIRPWCFVLNR.D
Score: 17.1
Modifications: Carbamidomethyl: 10

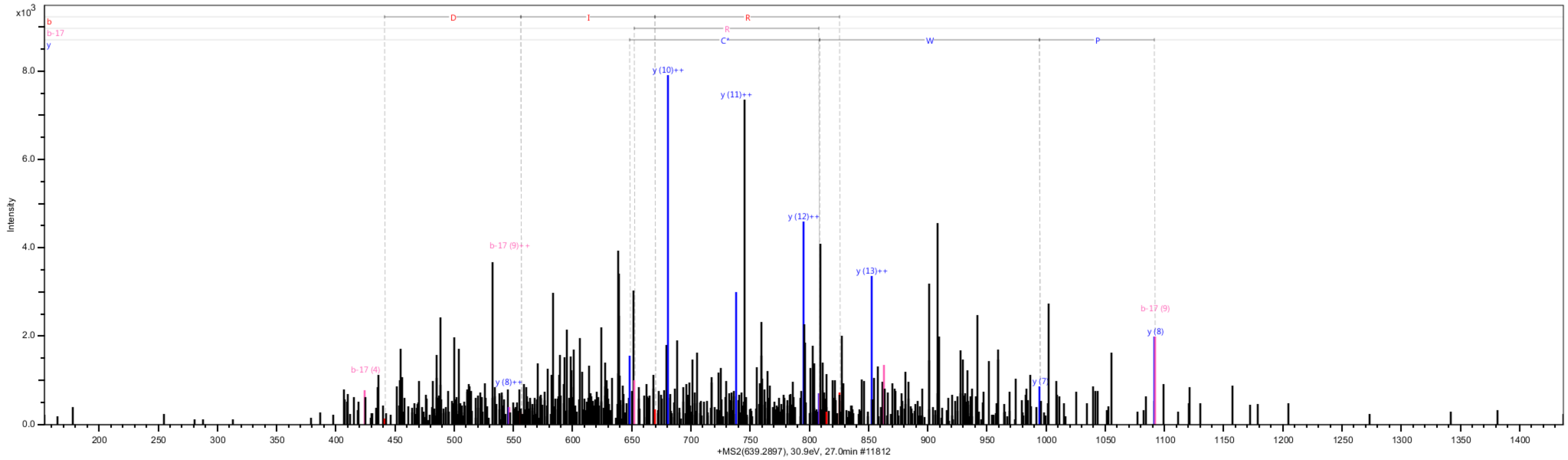

Peptide Match

Sequence: R.VVGGLVALR.G **Score:** 28

Modifications:

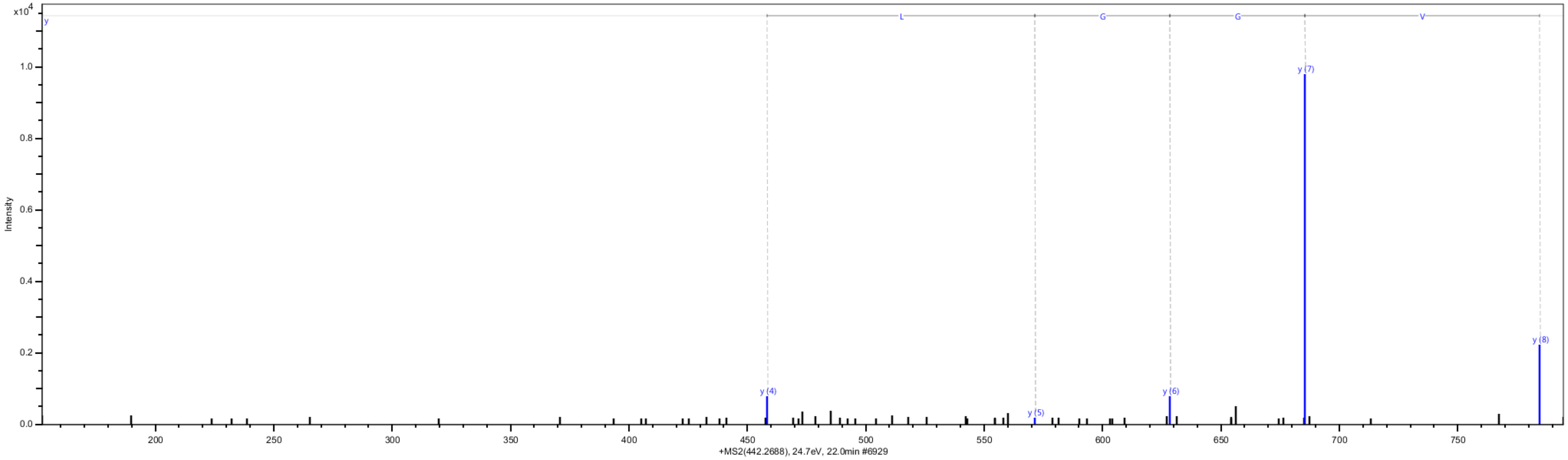
x10^4
y
L
G
G
V
y (7)
y (8)
y (4)
y (6)
y (5)
Intensity
1.0
0.8
0.6
0.4
0.2
0.0
200
250
300
350
400
450
500
550
600
650
700
750
+MS2(442.2688), 24.7eV, 22.0min #6929

ITIH4_HUMAN

Accession: ITIH4_HUMAN
Database: SwissProt
MW [kDa] / pI: 103.3 / 6.5
Score: 78.3
Seq. Coverage [%]: 3.3
No. of Peptides: 3

Peptide Match

Sequence: K.ITFELVYEELLK.R
Score: 41
Modifications:

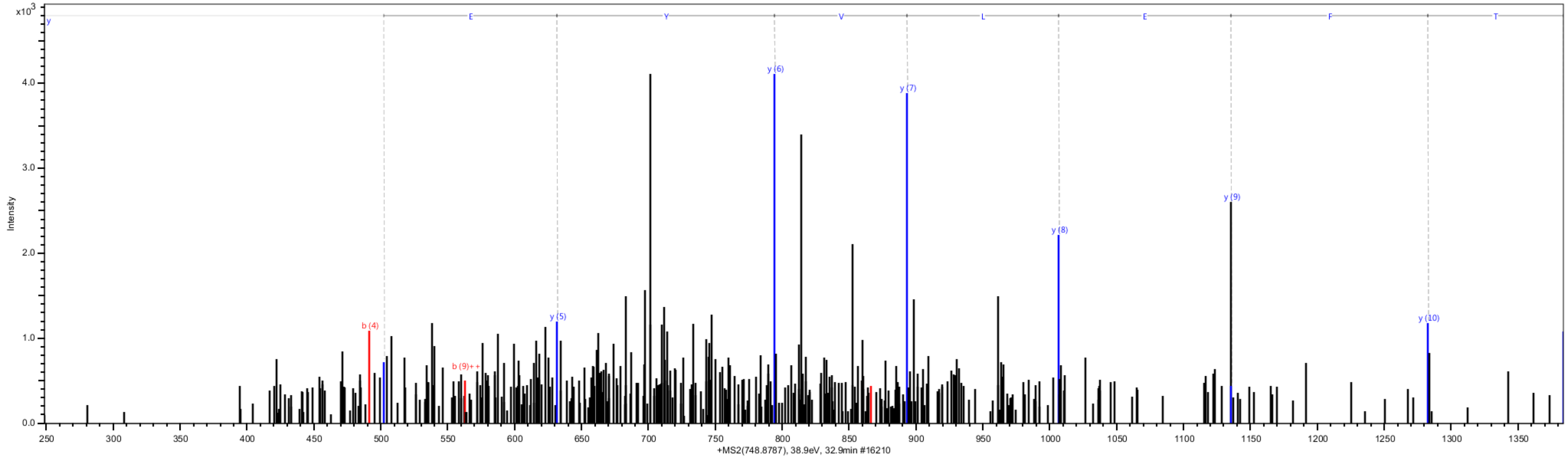

Peptide Match

Sequence: R.LGVYELLLK.V
Score: 16.6
Modifications:

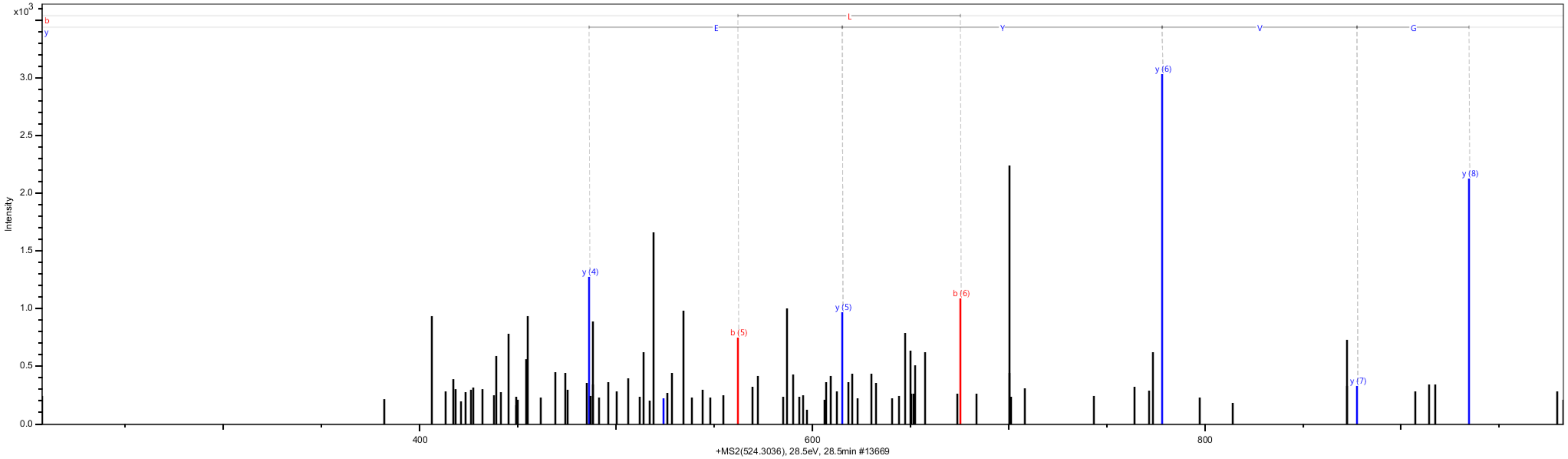

Peptide Match

**Sequence:**	K.LALDNGGLAR.R	**Score:**	20.7
**Modifications:**			

Spectrum Report
Combine Search_2020-10-19 07:53:31

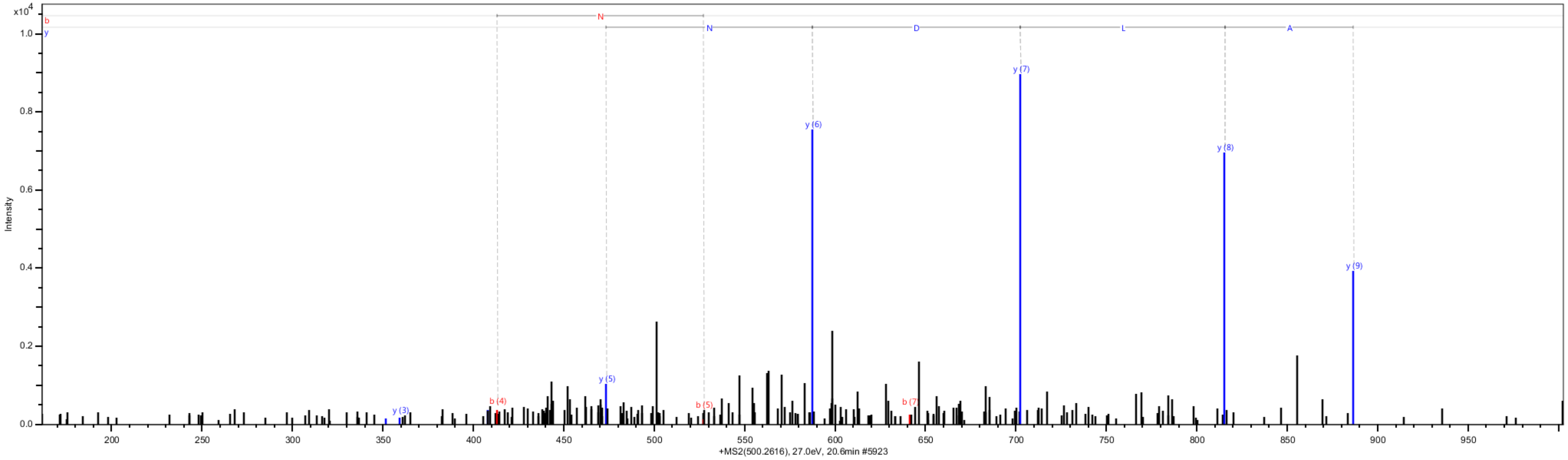

MSLN_HUMAN

Accession: MSLN_HUMAN
Database: SwissProt
MW [kDa] / pI: 68.9 / 6.0
Score: 76.2
Seq. Coverage [%]: 5.4
No. of Peptides: 3

Peptide Match

Sequence: R.ELAVALAQK.N
Score: 15
Modifications:

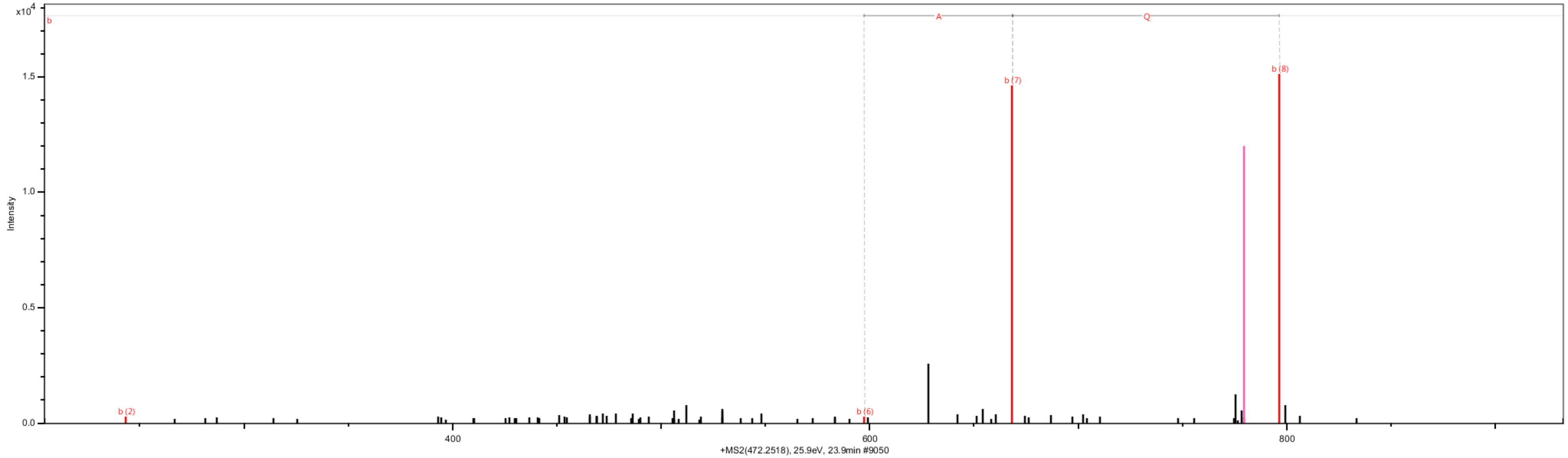

Peptide Match

Sequence: R.SIPQGIVAAWR.Q
Score: 17.6
Modifications:

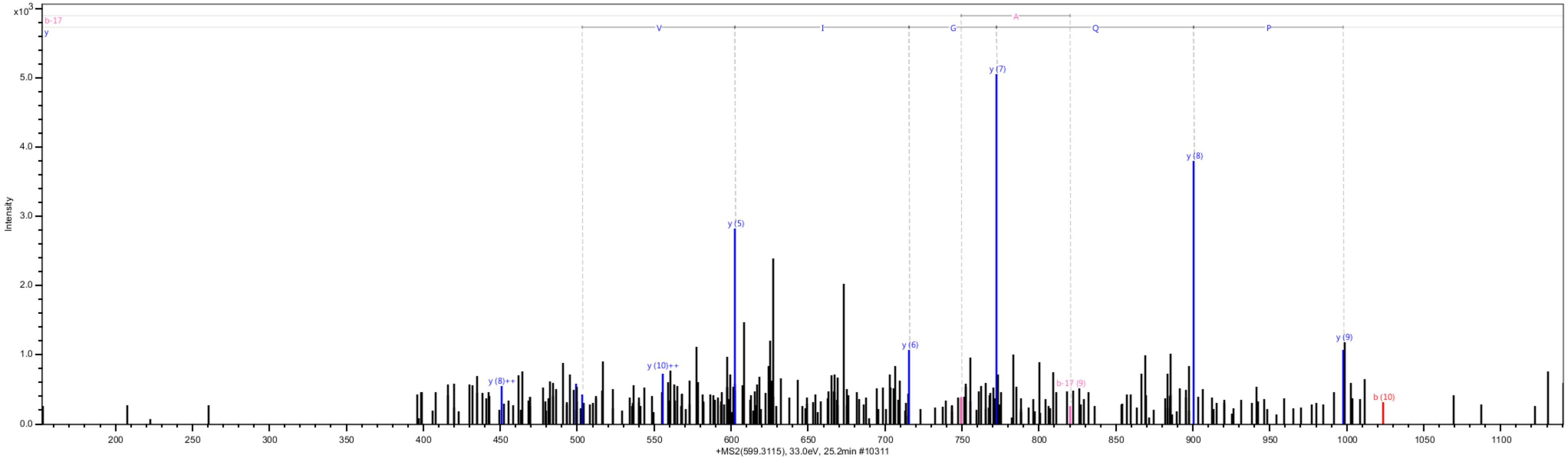

Peptide Match

Sequence: K.LLGPHVEGLKAEER.H

Score: 43.6

Modifications:

Spectrum Report
Combine Search_2020-10-19 07:53:31

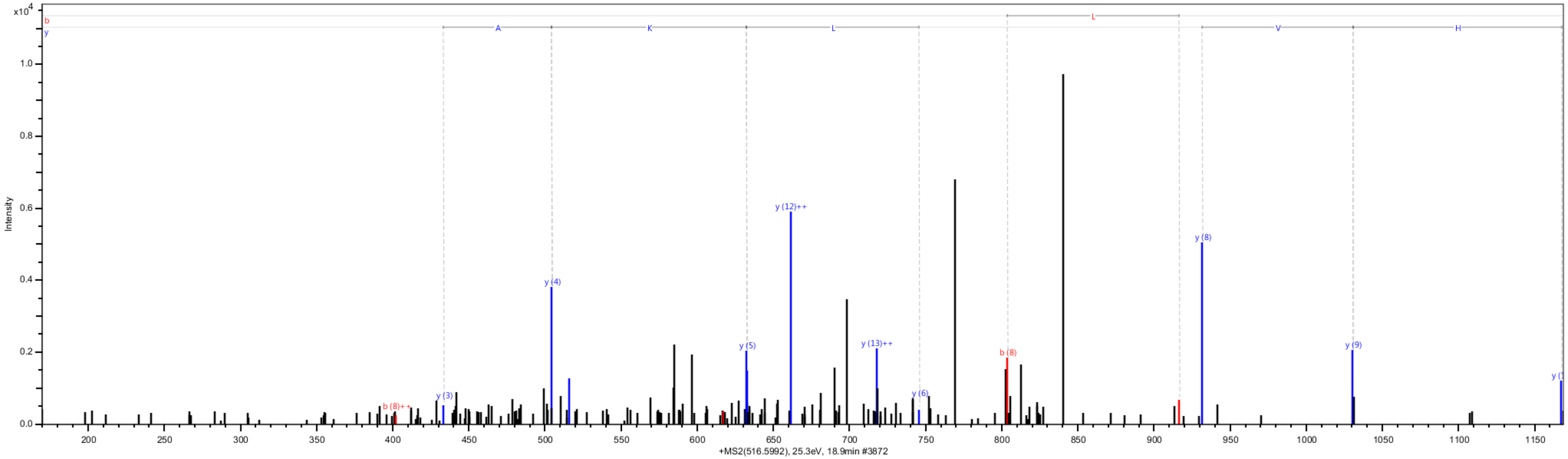

PGRP2_HUMAN

Accession: PGRP2_HUMAN
Database: SwissProt
MW [kDa] / pI: 62.2 / 7.3
Score: 115.0
Seq. Coverage [%]: 4.7
No. of Peptides: 2

Peptide Match

Sequence: R.AGLLRPDYALLGHR.Q
Score: 33.7
Modifications:

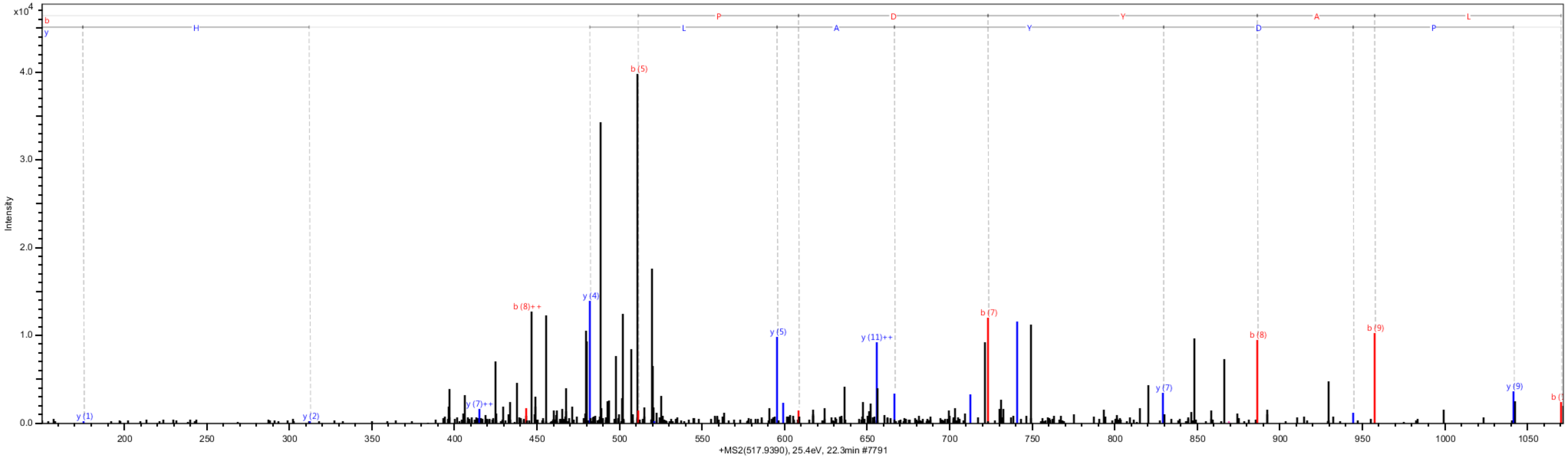

Peptide Match

Sequence: R.TDCPGDALFDLLR.T
Score: 81.3
Modifications: Carbamidomethyl: 3

Spectrum Report
Combine Search_2020-10-19 07:53:31

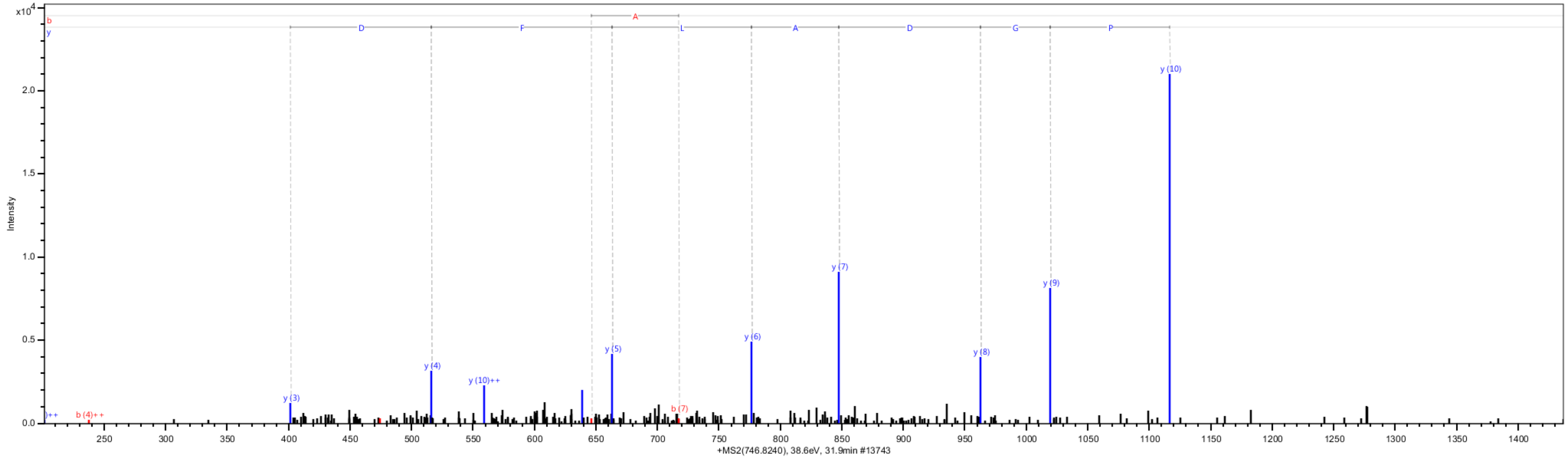

FINC_HUMAN

**Accession:**	FINC_HUMAN	**Score:**	114.1
**Database:**	SwissProt	**Seq. Coverage [%]:**	1.9
**MW [kDa] / pI:**	272.1 / 5.3	**No. of Peptides:**	4

Peptide Match

**Sequence:**	R.WSRPQAPITGYR.I	**Score:**	19.6
**Modifications:**			

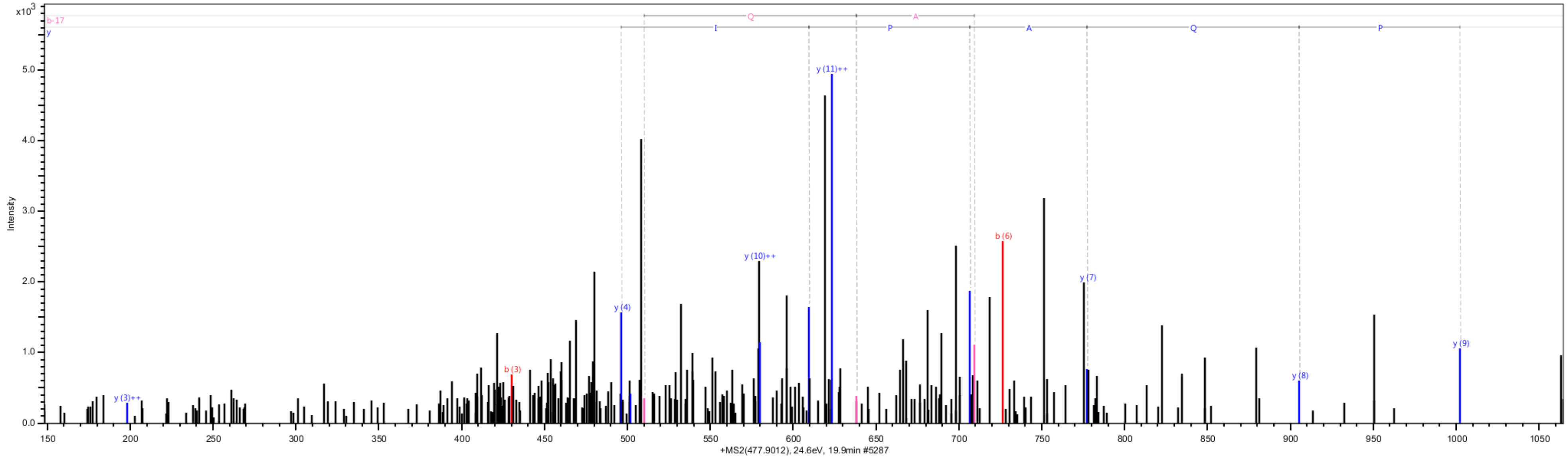

Peptide Match

**Sequence:**	R.STTPDITGYR.I	**Score:**	27.5
**Modifications:**			

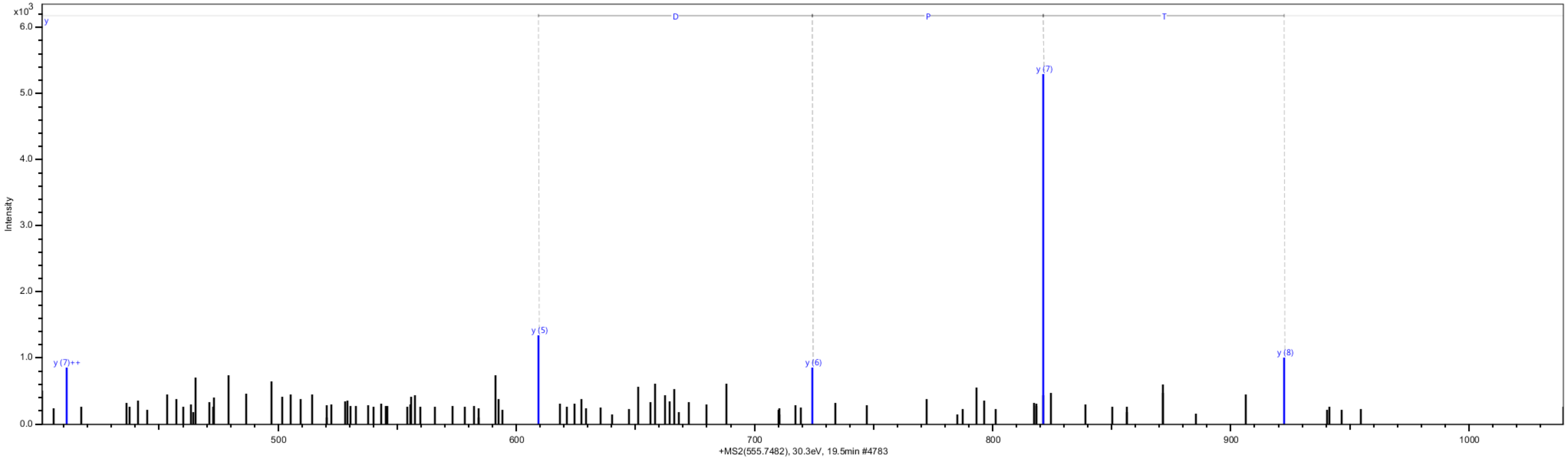

Peptide Match

**Sequence:**	K.WLPSSSPVTGYR.V	**Score:**	21.9
**Modifications:**			

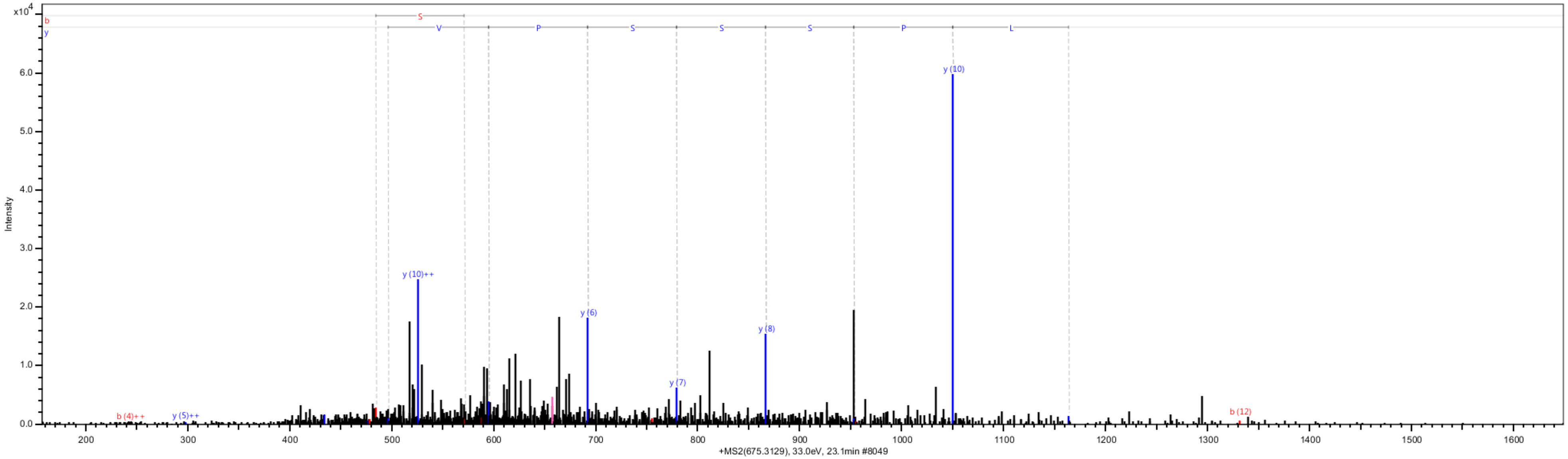

Peptide Match

Sequence: K.LLCQCLGFGSGHFR.C **Score:** 45.2
Modifications: Carbamidomethyl: 3, 5

Spectrum Report
Combine Search_2020-10-19 07:53:31

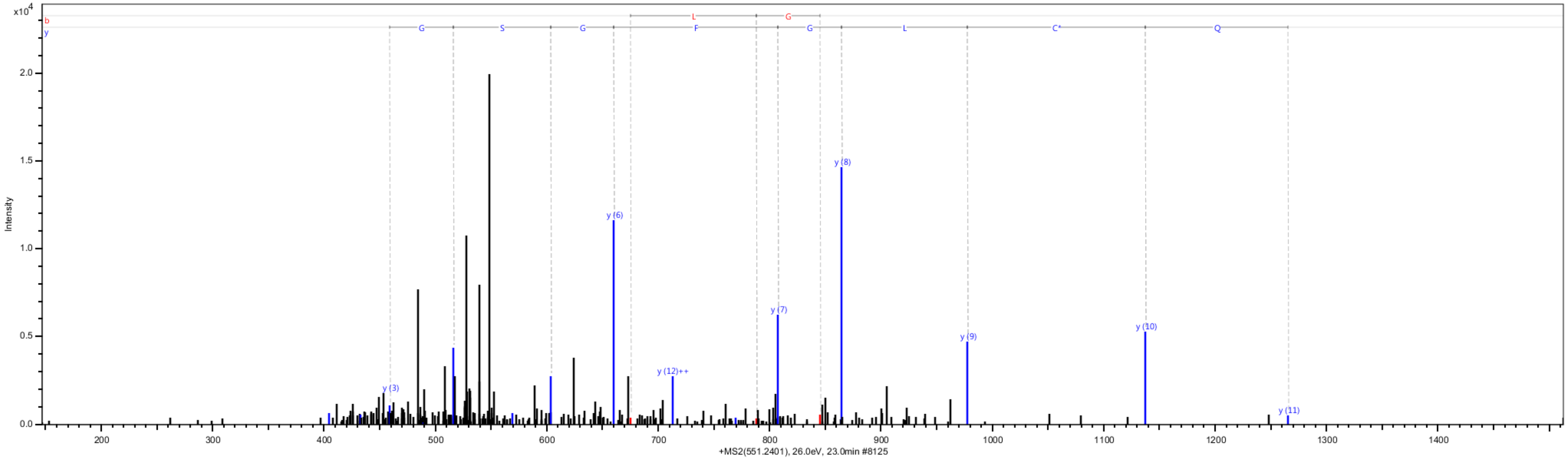

A2GL_HUMAN

Accession: A2GL_HUMAN
Database: SwissProt
MW [kDa] / pI: 38.2 / 6.5
Score: 130.6
Seq. Coverage [%]: 15.6
No. of Peptides: 4

Peptide Match

Sequence: K.ALGHLDLSGNRLR.K
Score: 27.8
Modifications:

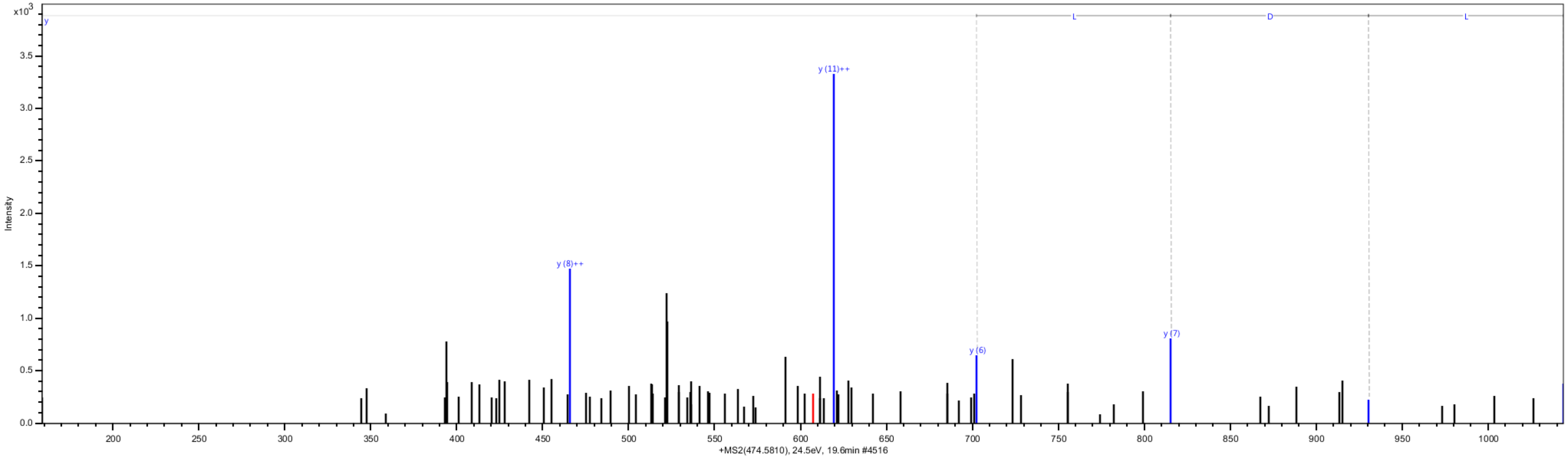

Peptide Match

Sequence: K.DLLLPQPDLR.Y
Score: 26.7
Modifications:

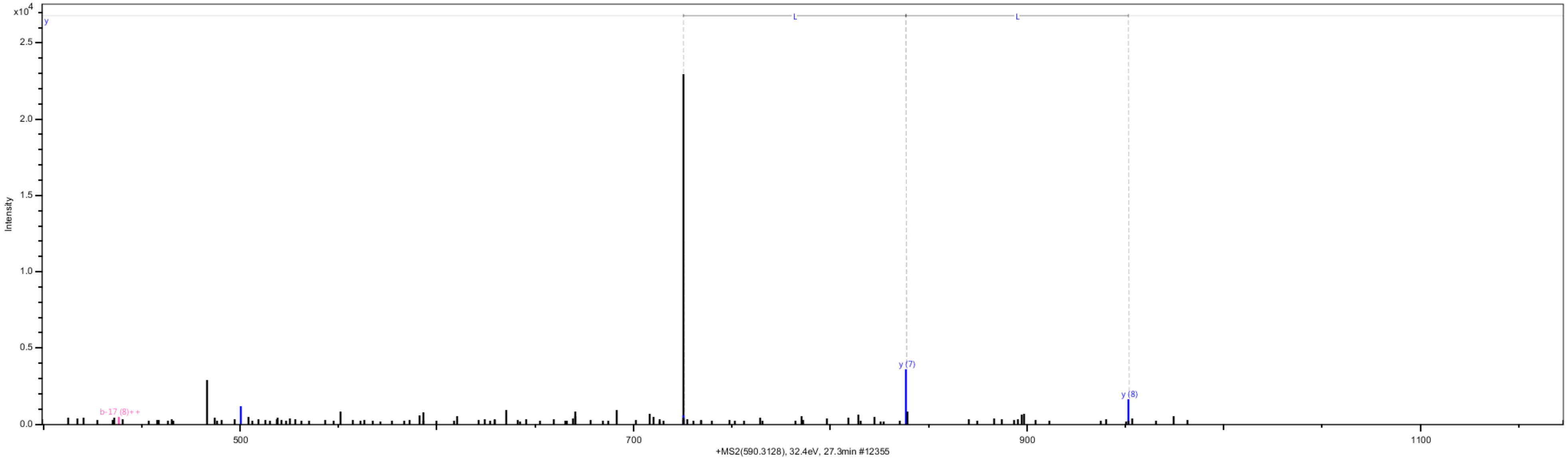

Peptide Match

**Sequence:**	R.VAAGAFQGLR.Q	**Score:**	48.6
**Modifications:**			

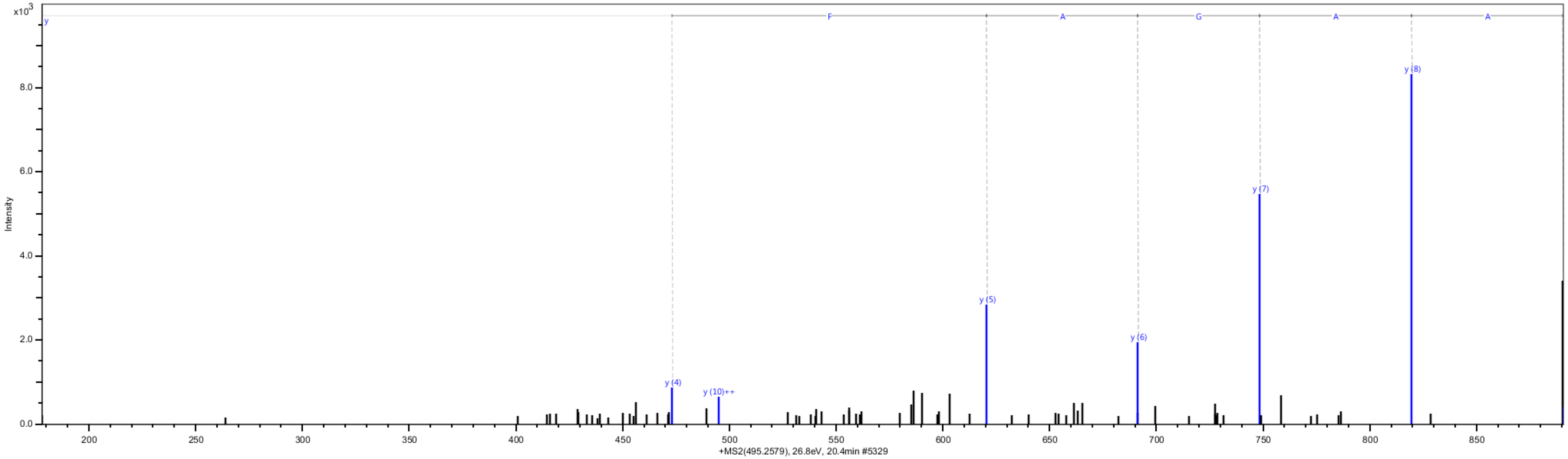

Peptide Match

**Sequence:**	R.DGFDISGNPWICDQNLSDLYR.W	**Score:**	27.6
**Modifications:**	Carbamidomethyl: 12		

Spectrum Report
Combine Search_2020-10-19 07:53:31

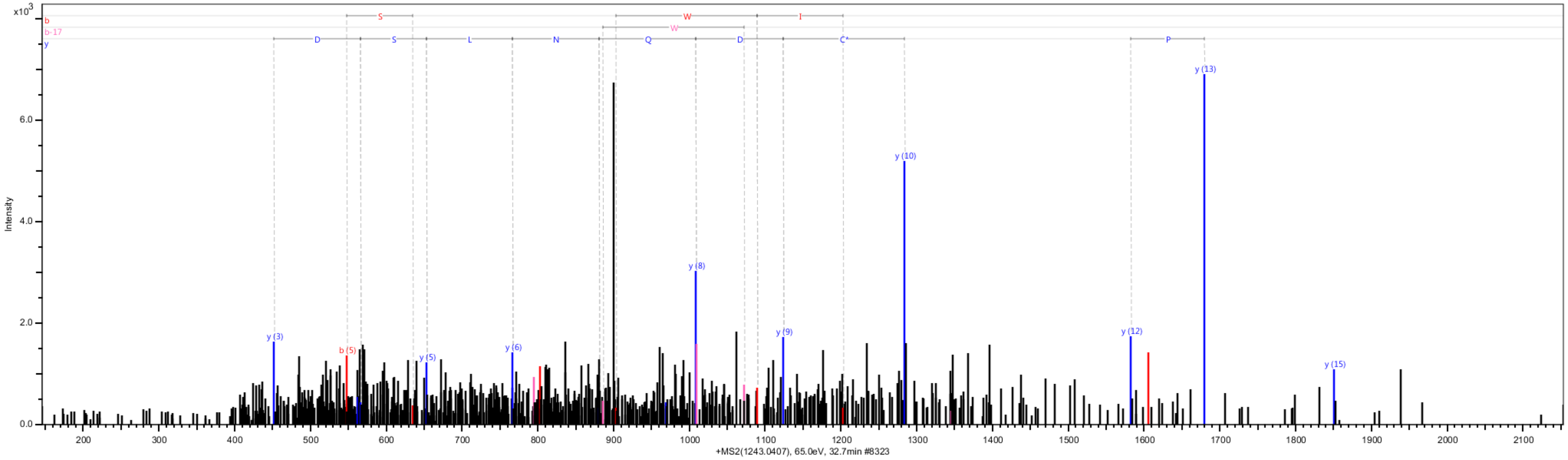

LUM_HUMAN

Accession: LUM_HUMAN
Database: SwissProt
MW [kDa] / pI: 38.4 / 6.2
Score: 128.0
Seq. Coverage [%]: 12.4
No. of Peptides: 4

Peptide Match

Sequence: R.LKEDAVSAAFK.G
Score: 38.7
Modifications:

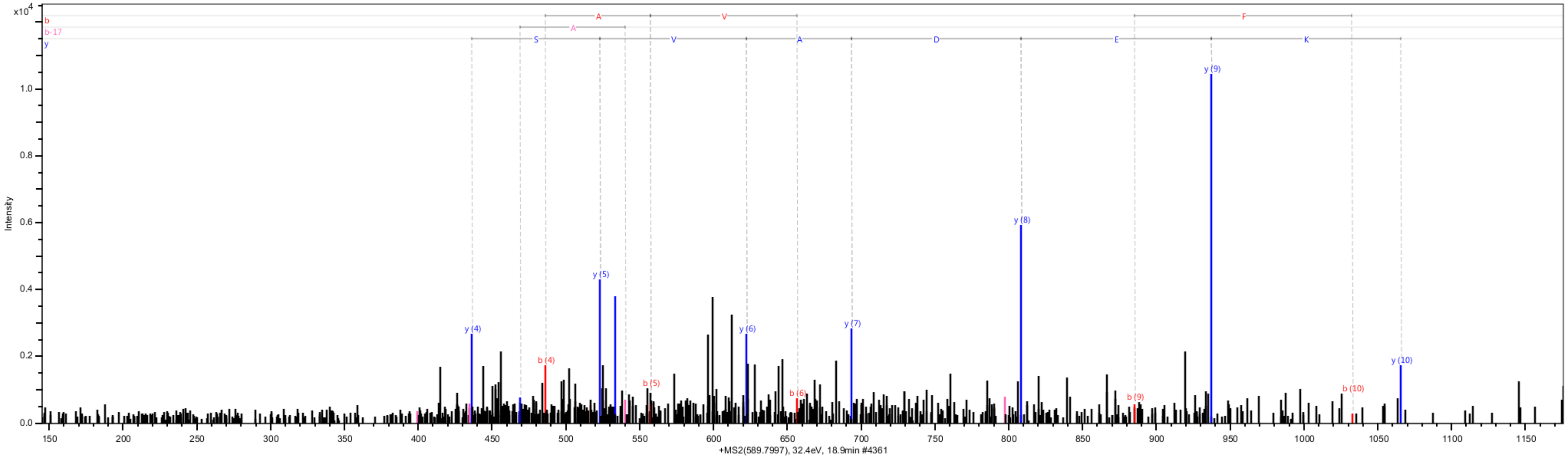

Peptide Match

Sequence: K.SLEYLDLSFNQIAR.L
Score: 26.2
Modifications:

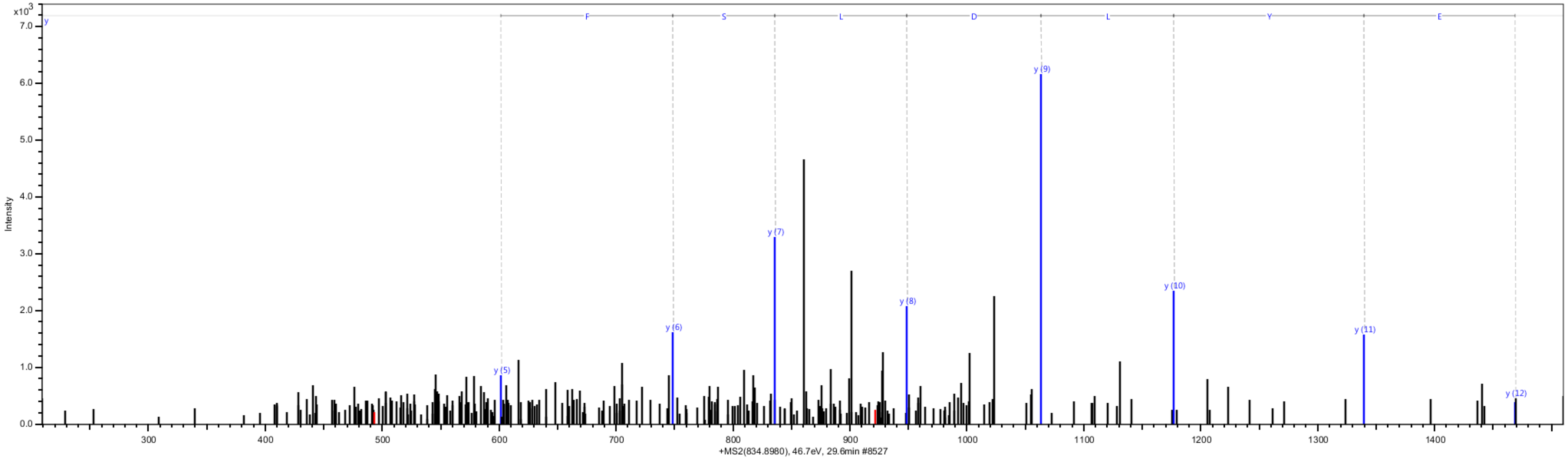

Peptide Match

Sequence: R.FNALQYLR.L **Score:** 22.3

Modifications:

Spectrum Report
Combine Search_2020-10-19 07:53:31

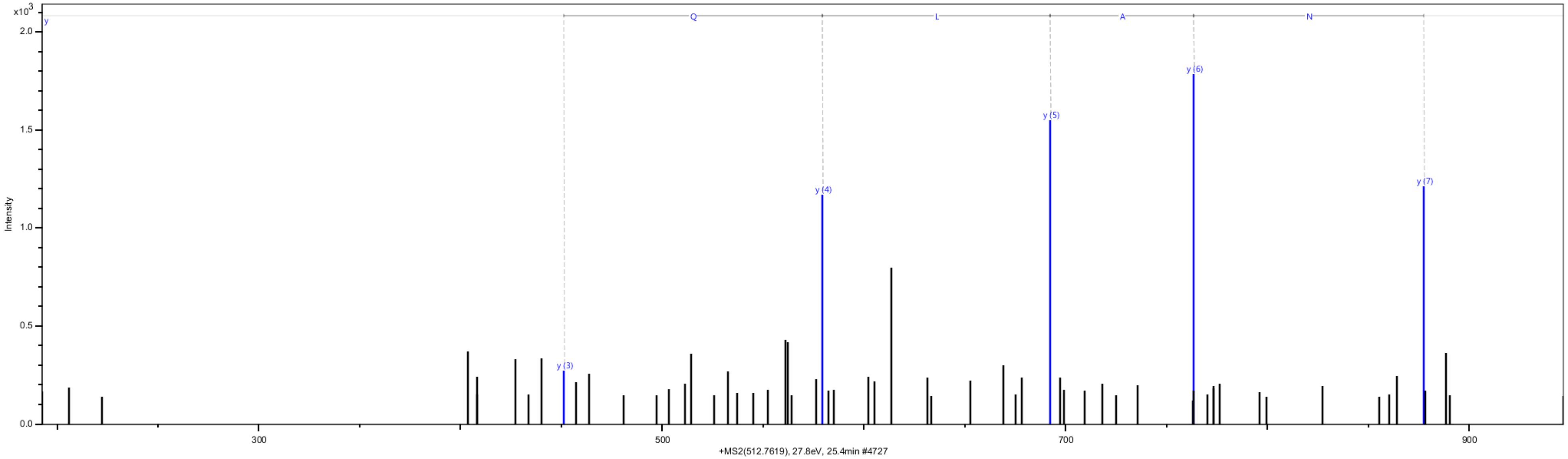

Peptide Match

**Sequence:**	K.ILGPLSYSK.I	**Score:**	40.7
**Modifications:**			

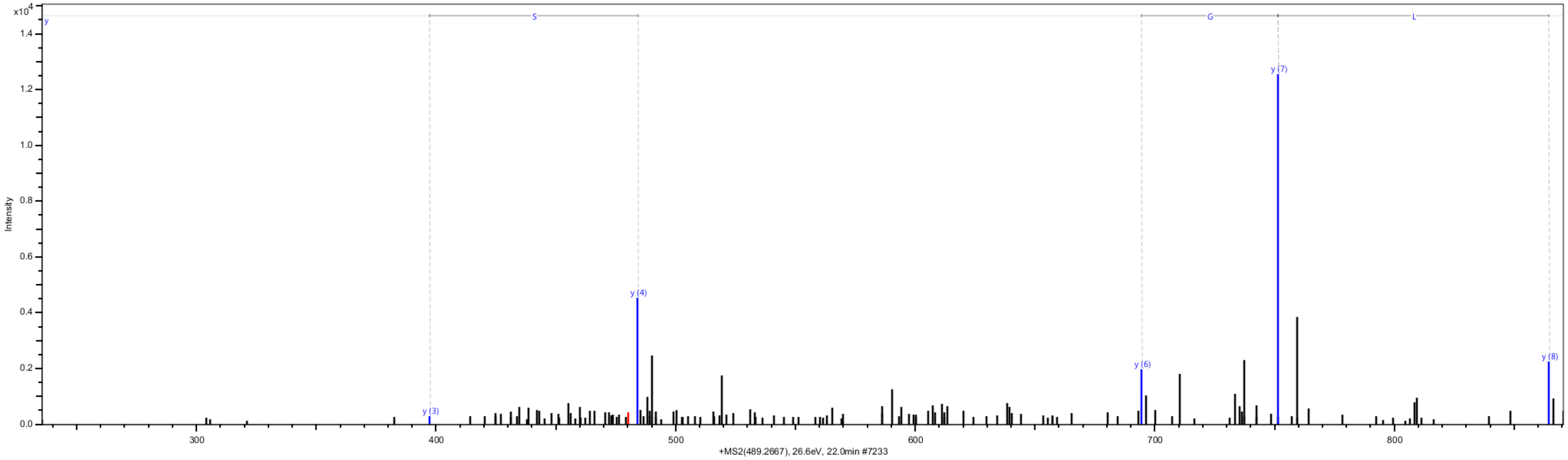

x10^4
Intensity
y
S
G
L
y (3)
y (4)
y (6)
y (7)
y (8)
0.0
0.2
0.4
0.6
0.8
1.0
1.2
1.4
300
400
500
600
700
800
+MS2(489.2667), 26.6eV, 22.0min #7233

APOA2_HUMAN

Accession: APOA2_HUMAN
Database: SwissProt
MW [kDa] / pI: 11.2 / 6.3
Score: 86.3
Seq. Coverage [%]: 21.0
No. of Peptides: 3

Peptide Match

Sequence: K.VKSPELQAEAK.S
Score: 21.6
Modifications:

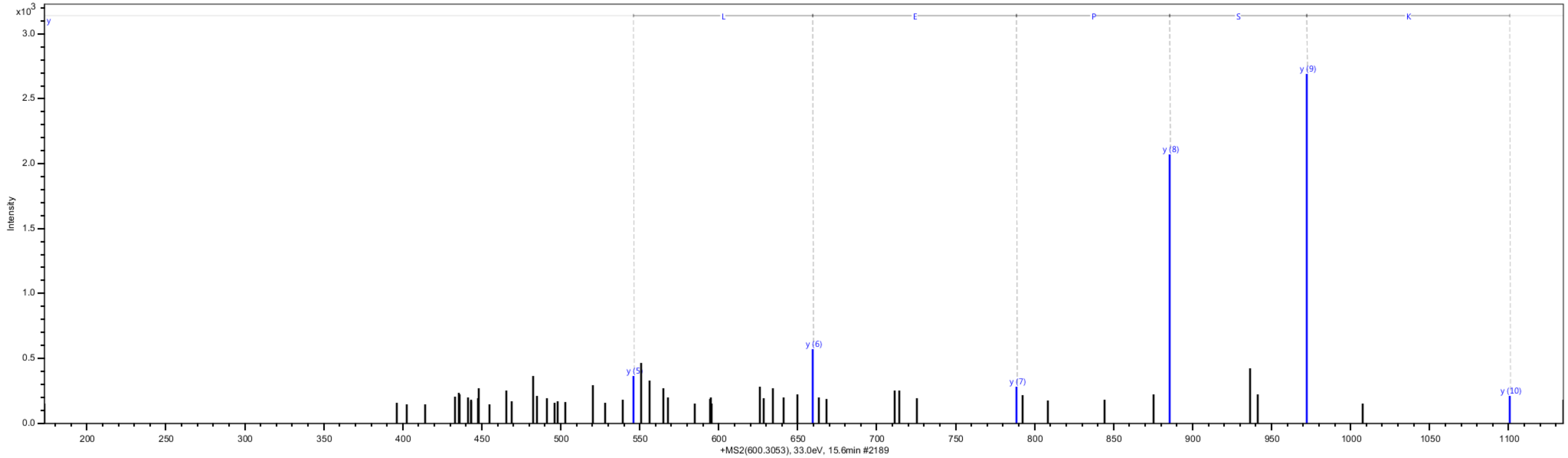

Peptide Match

Sequence: K.SKEQLTPLIK.K
Score: 43.6
Modifications:

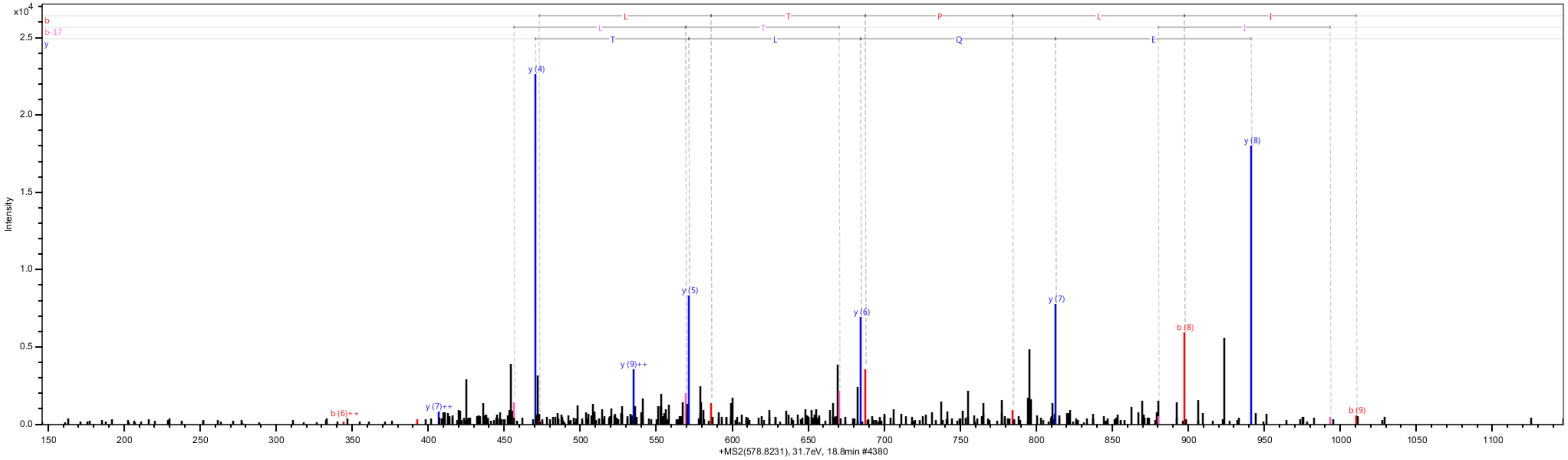

Peptide Match

Sequence: K.EQLTPLIK.K **Score:** 21.1

Modifications:

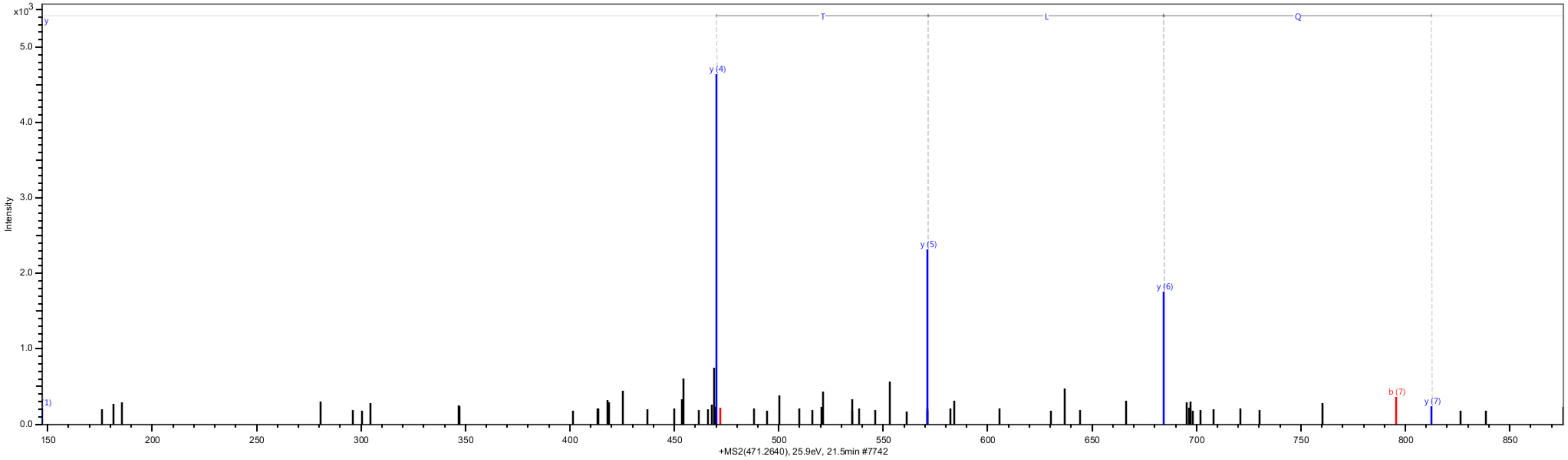
x10^3
5.0
4.0
3.0
2.0
1.0
0.0
Intensity
T
L
Q
y (4)
y (5)
y (6)
b (7)
y (7)
150
200
250
300
350
400
450
500
550
600
650
700
750
800
850
+MS2(471.2640), 25.9eV, 21.5min #7742

LV39_HUMAN

Accession: LV39_HUMAN
Database: SwissProt
MW [kDa] / pI: 12.3 / 6.8
Score: 82.7
Seq. Coverage [%]: 13.9
No. of Peptides: 1

Peptide Match

Sequence: R.FSGSNSGNTATLTISR.A
Score: 82.7
Modifications:

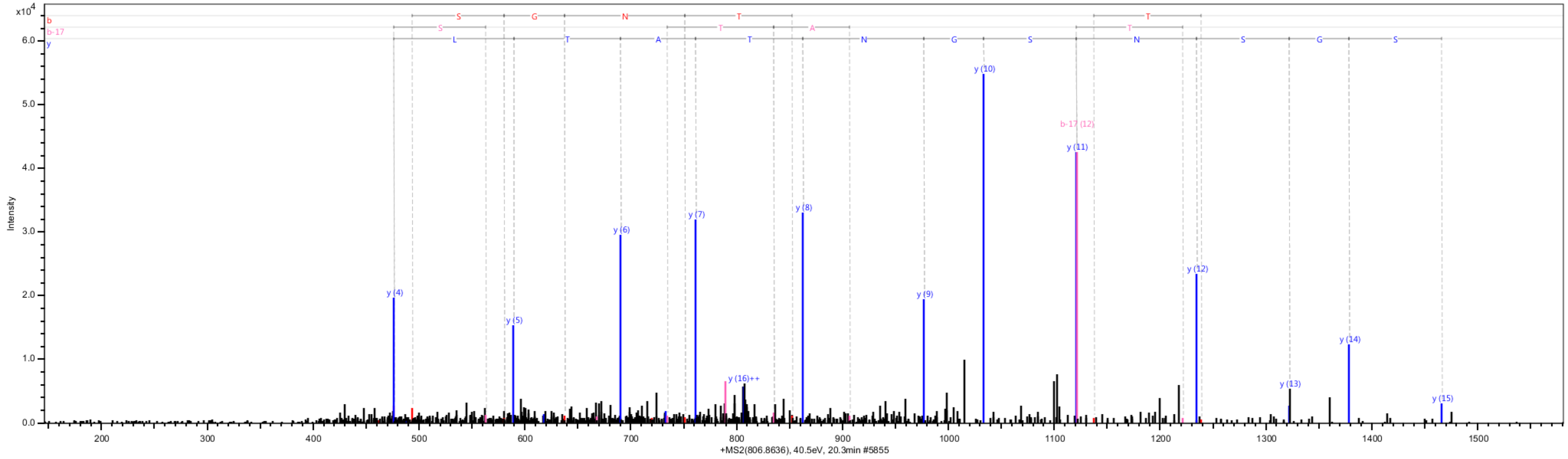

PLMN_HUMAN

**Accession:**	PLMN_HUMAN	**Score:**	107.3
**Database:**	SwissProt	**Seq. Coverage [%]:**	4.6
**MW [kDa] / pI:**	90.5 / 7.0	**No. of Peptides:**	4

Peptide Match

**Sequence:**	R.WELCDIPR.C	**Score:**	31.1
**Modifications:**	Carbamidomethyl: 4		

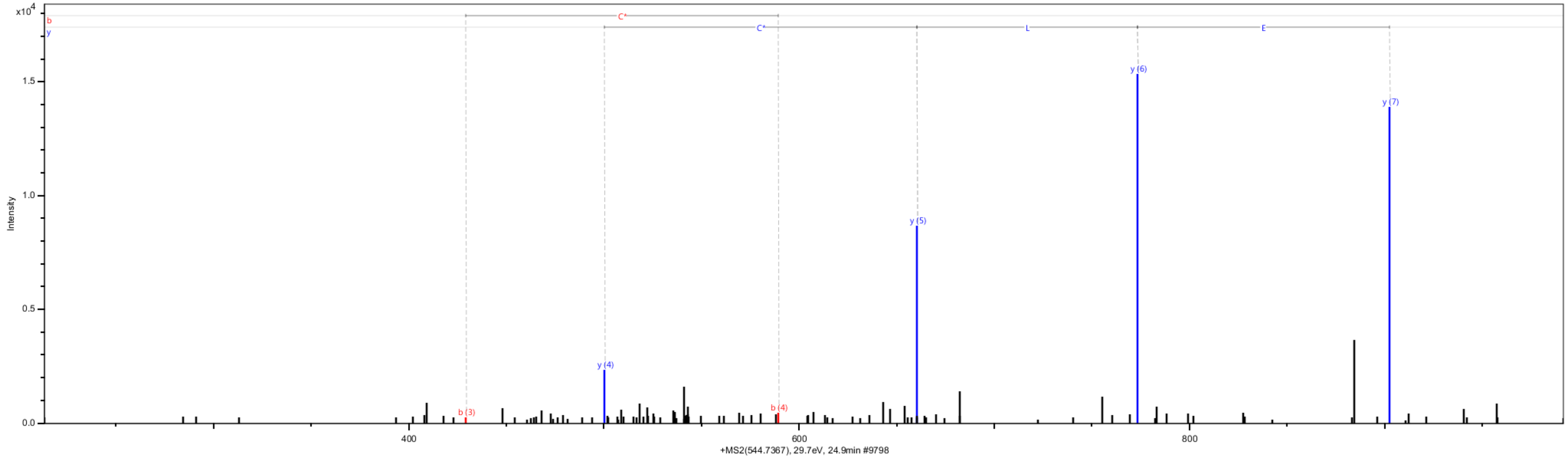

Peptide Match

**Sequence:**	R.HSIFTPETNPR.A	**Score:**	43.6
**Modifications:**			

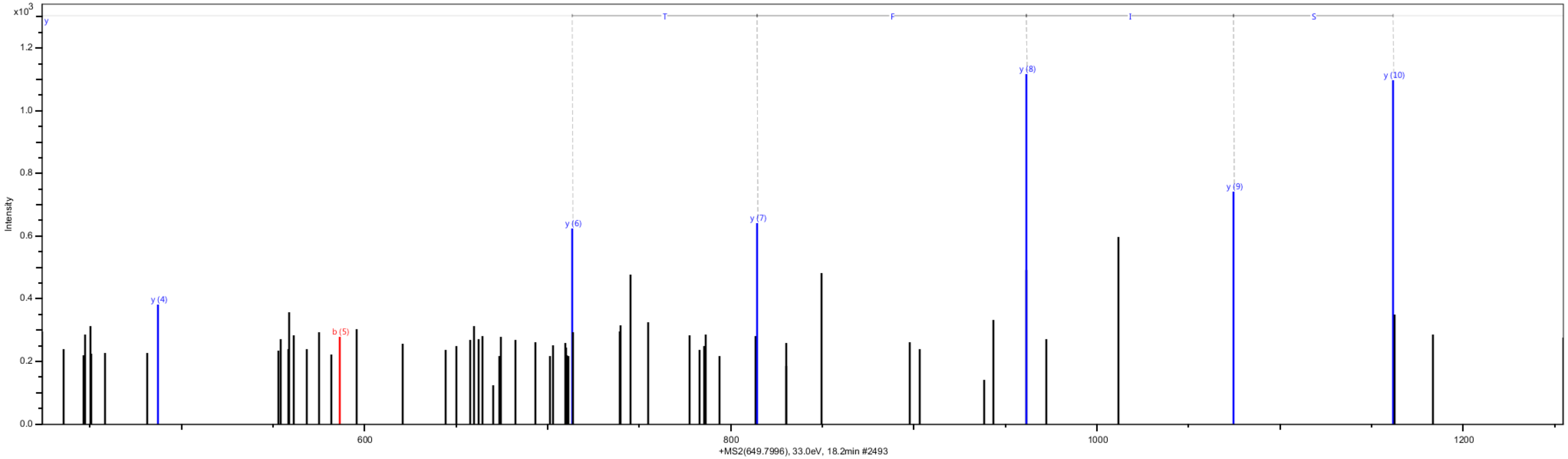

Peptide Match

**Sequence:**	R.LFLEPTRK.D	**Score:**	17.1
**Modifications:**			

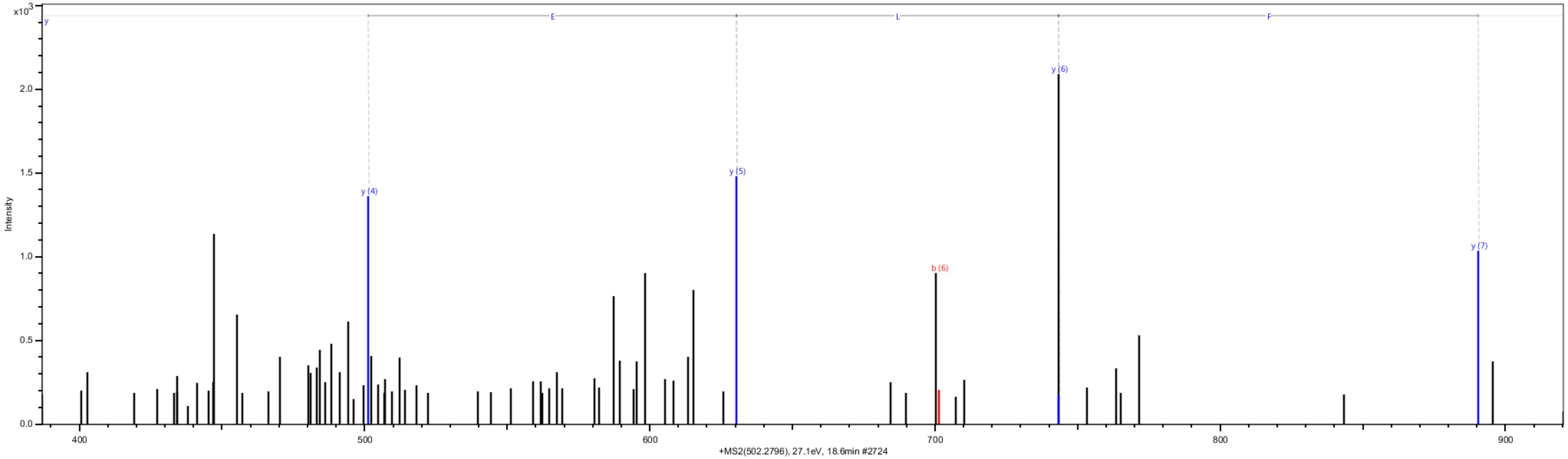

Peptide Match

**Sequence:**	K.LSSPAVITDK.V	**Score:**	15.4
**Modifications:**			

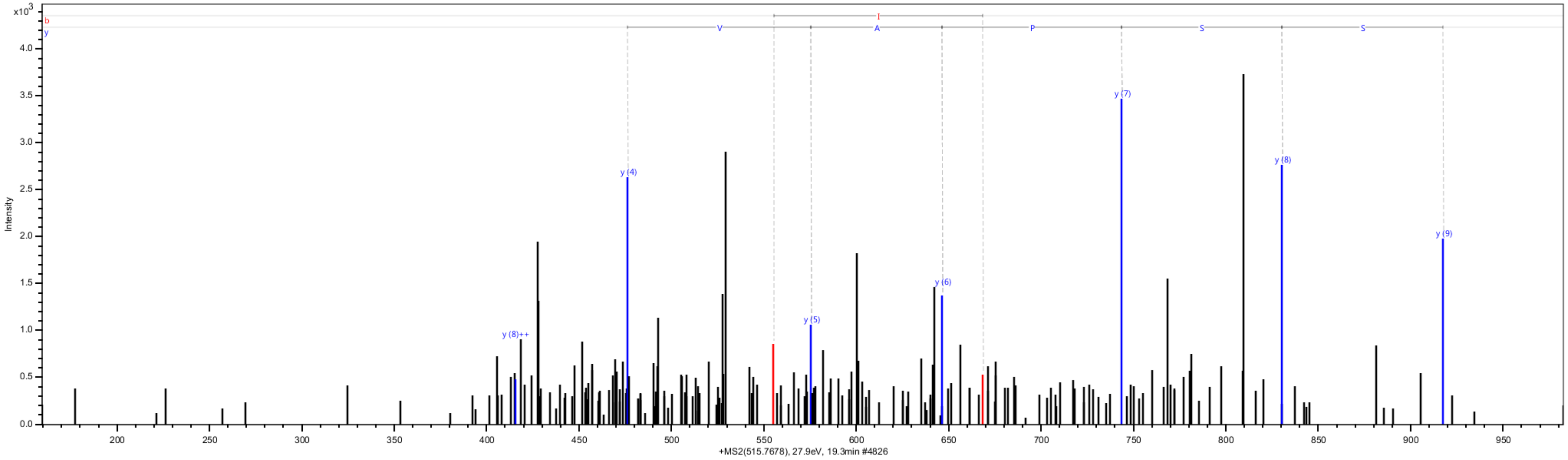
x10^3
b
y
Intensity
I
V
A
P
S
S
y (8)++
y (4)
y (5)
y (6)
y (7)
y (8)
y (9)
+MS2(515.7678), 27.9eV, 19.3min #4826

GELS_HUMAN

Accession: GELS_HUMAN | **Score:** 96.2
Database: SwissProt | **Seq. Coverage [%]:** 5.0
MW [kDa] / pI: 85.6 / 5.9 | **No. of Peptides:** 3

Peptide Match

Sequence: K.AGKEPGLQIWR.V **Score:** 28.6
Modifications:

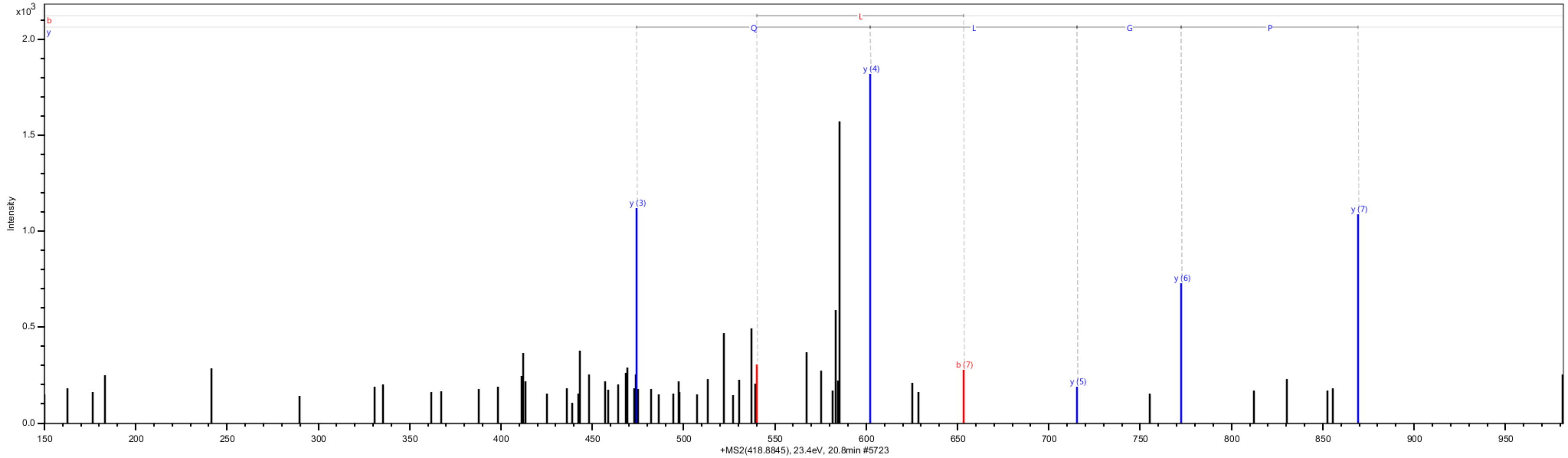

Peptide Match

Sequence: R.EVQGFESATFLGYFK.S **Score:** 15.4
Modifications:

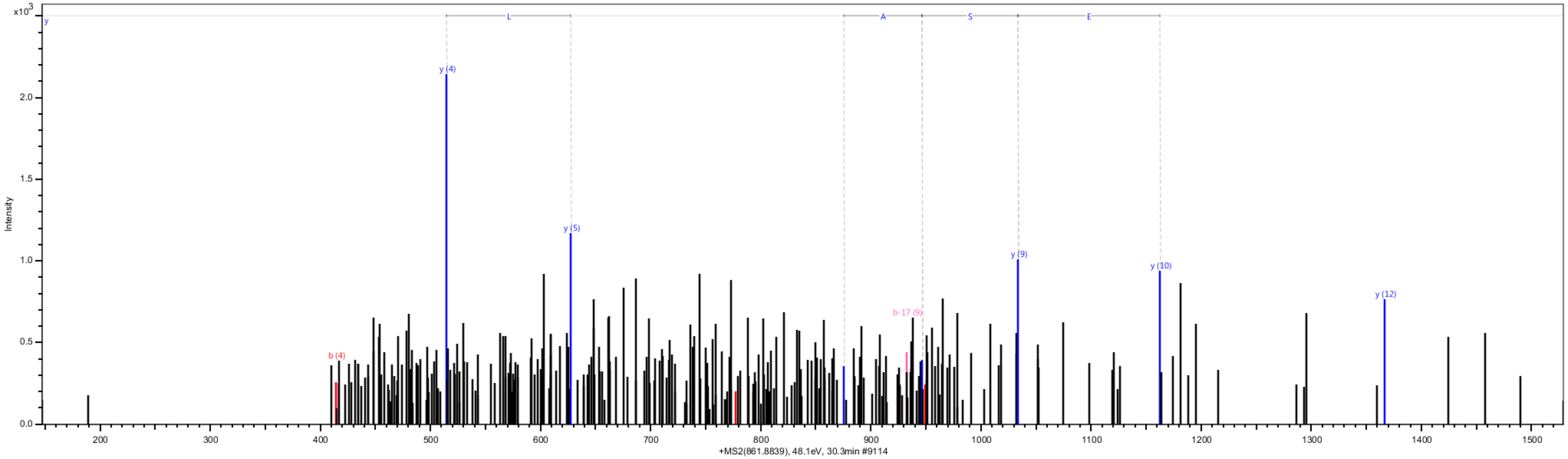

Peptide Match

Sequence: K.AGALNSNDAFVLK.T **Score:** 52.2

Modifications:

Spectrum Report
Combine Search_2020-10-19 07:53:31

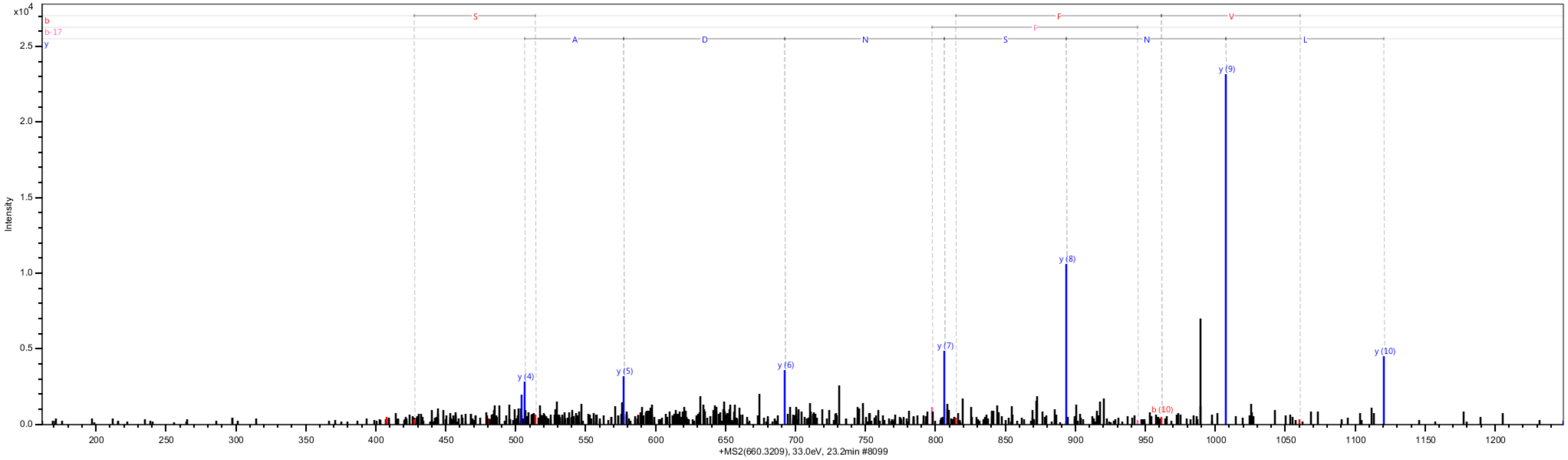

TRFE_HUMAN

**Accession:**	TRFE_HUMAN	**Score:**	2660.8
**Database:**	SwissProt	**Seq. Coverage [%]:**	50.9
**MW [kDa] / pI:**	77.0 / 6.8	**No. of Peptides:**	43

Peptide Match

**Sequence:**	R.WCAVSEHEATK.C	**Score:**	48.2
**Modifications:**	Carbamidomethyl: 2		

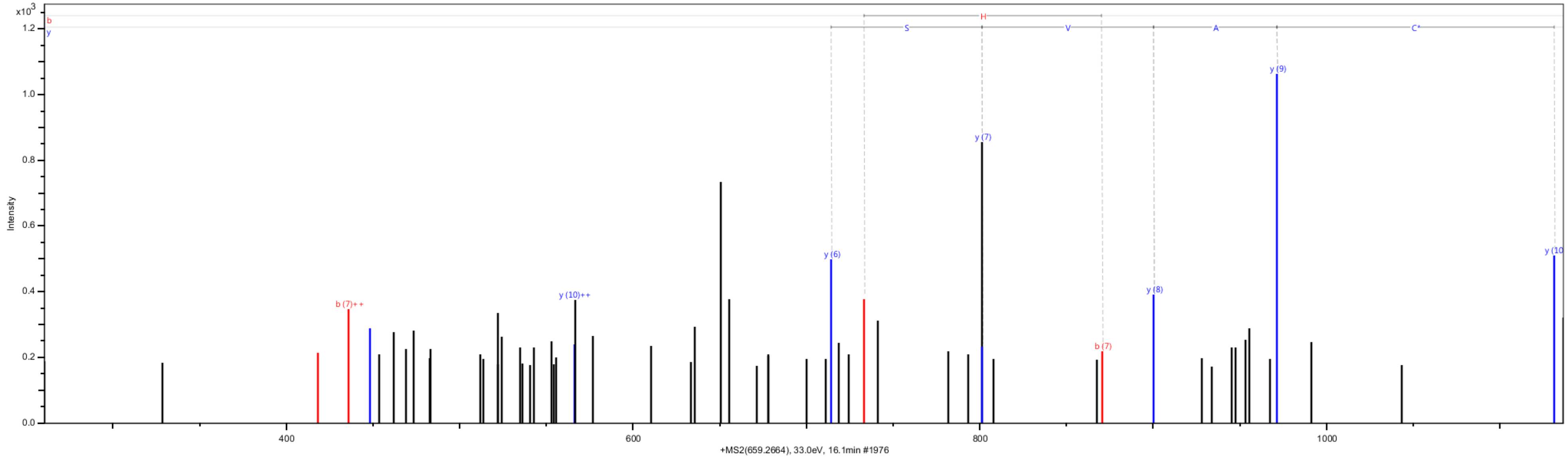

Peptide Match

**Sequence:**	K.SVIPSDGPSVACVK.K	**Score:**	84.1
**Modifications:**	Carbamidomethyl: 12		

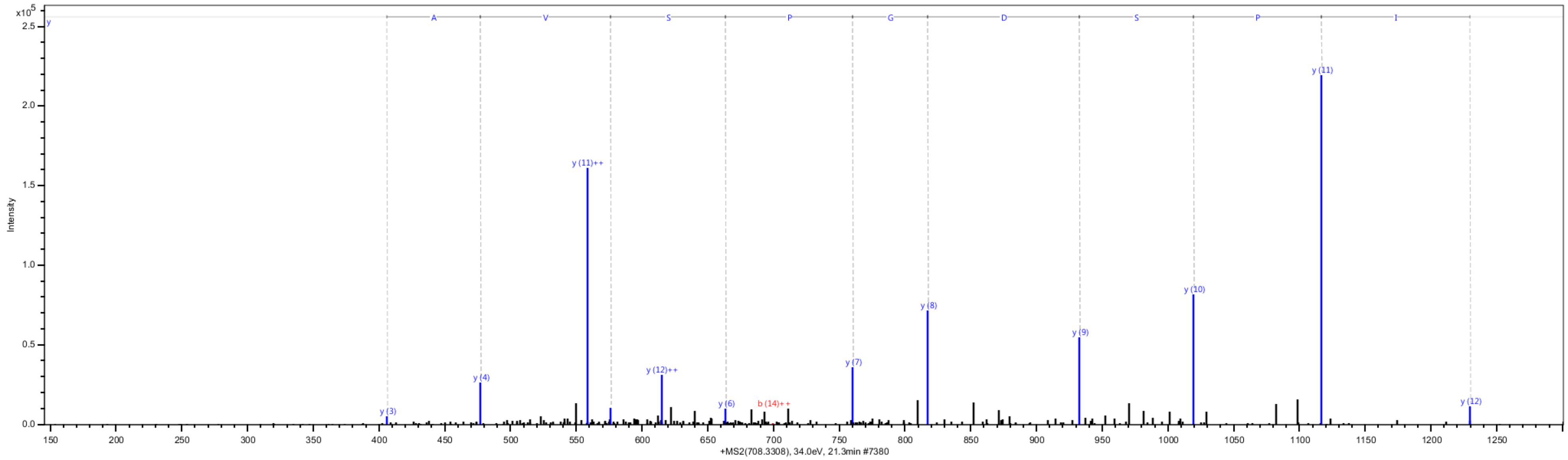

Peptide Match

Sequence: K.SVIPSDGPSVACVKK.A **Score:** 97.4
Modifications: Carbamidomethyl: 12

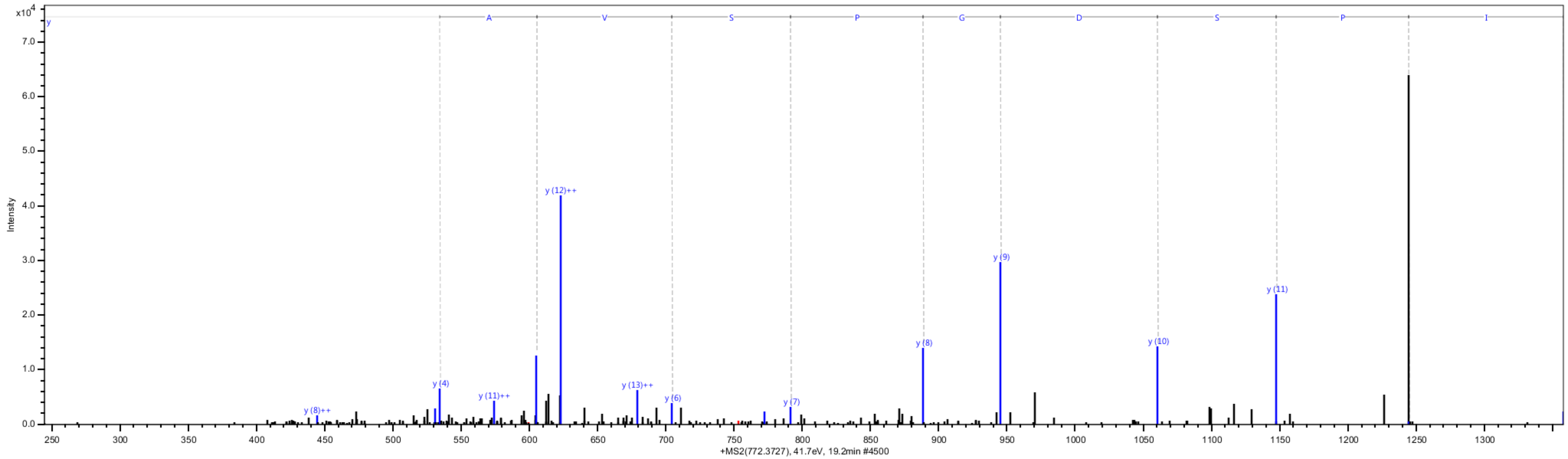

Peptide Match

**Sequence:**	K.KASYLDCIR.A	**Score:**	46.1
**Modifications:**	Carbamidomethyl: 7		

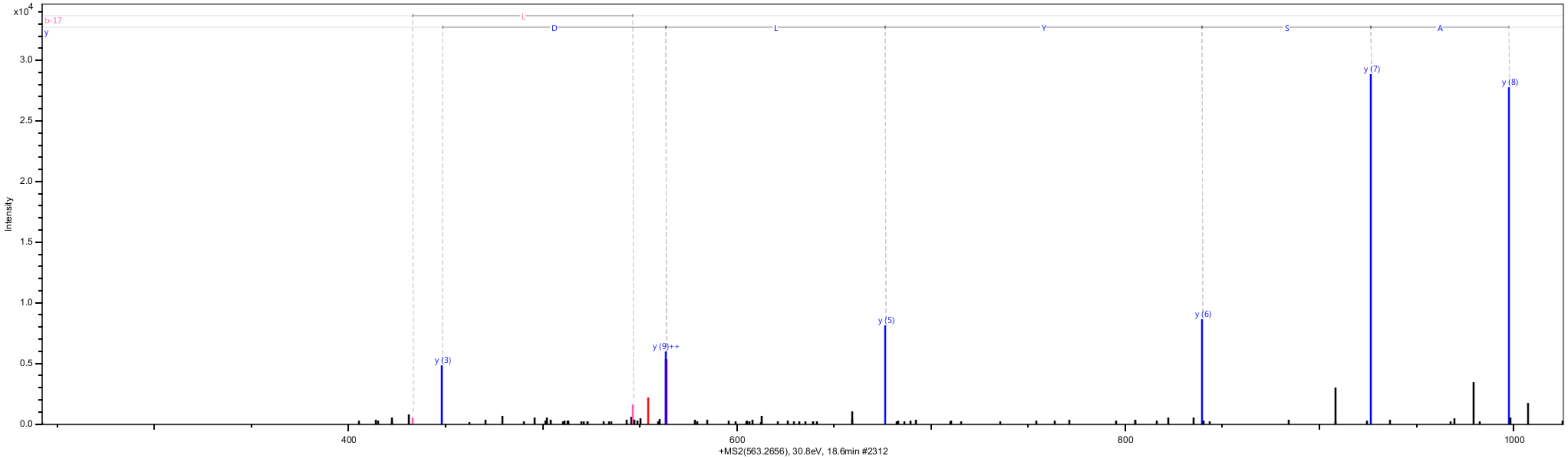

Peptide Match

**Sequence:**	K.ASYLDCIR.A	**Score:**	48.7
**Modifications:**	Carbamidomethyl: 6		

Spectrum Report
Combine Search_2020-10-19 07:53:31

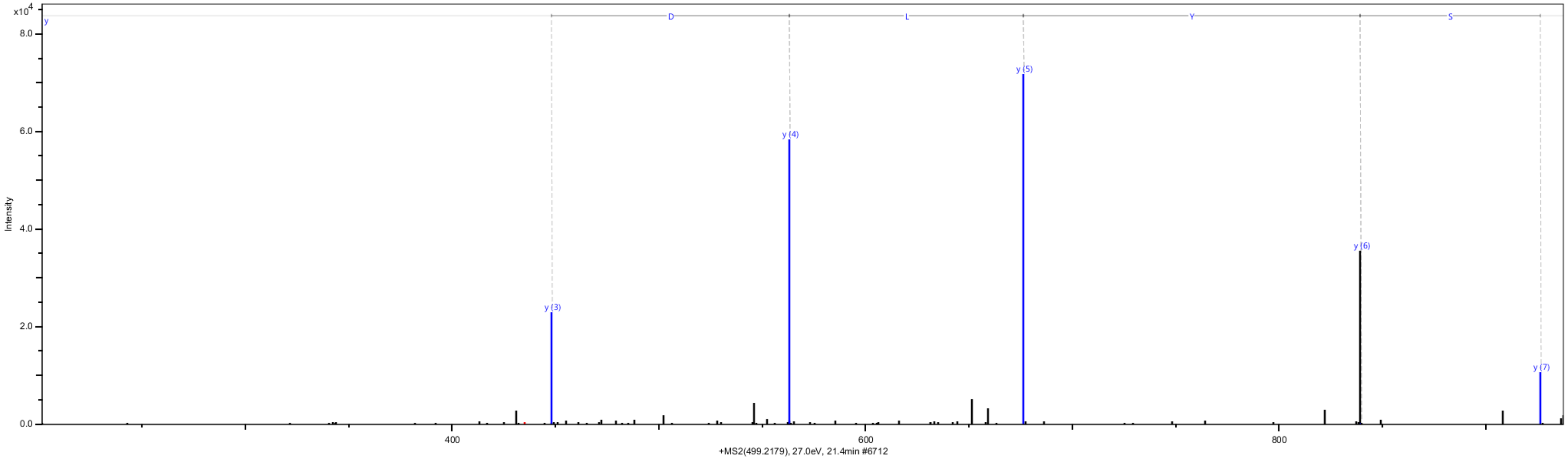

Peptide Match

**Sequence:**	K.EDPQTFYYAVAVVK.K	**Score:**	58.1
**Modifications:**			

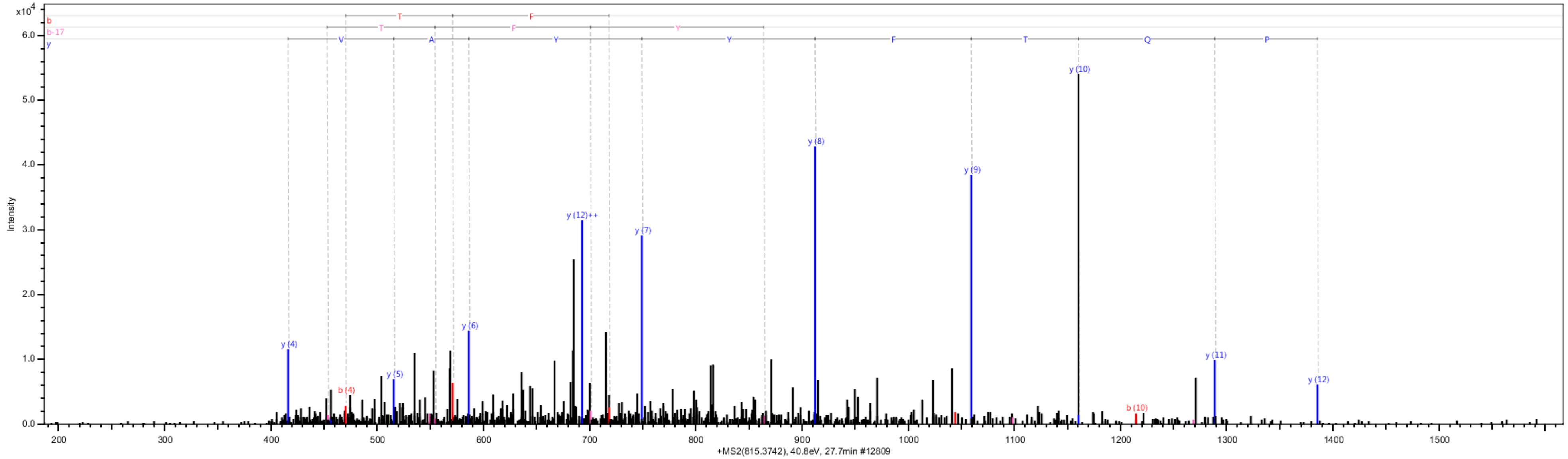

Peptide Match

Sequence: K.EDPQTFYYAVAVVKK.D **Score:** 26.2

Modifications:

Spectrum Report
Combine Search_2020-10-19 07:53:31

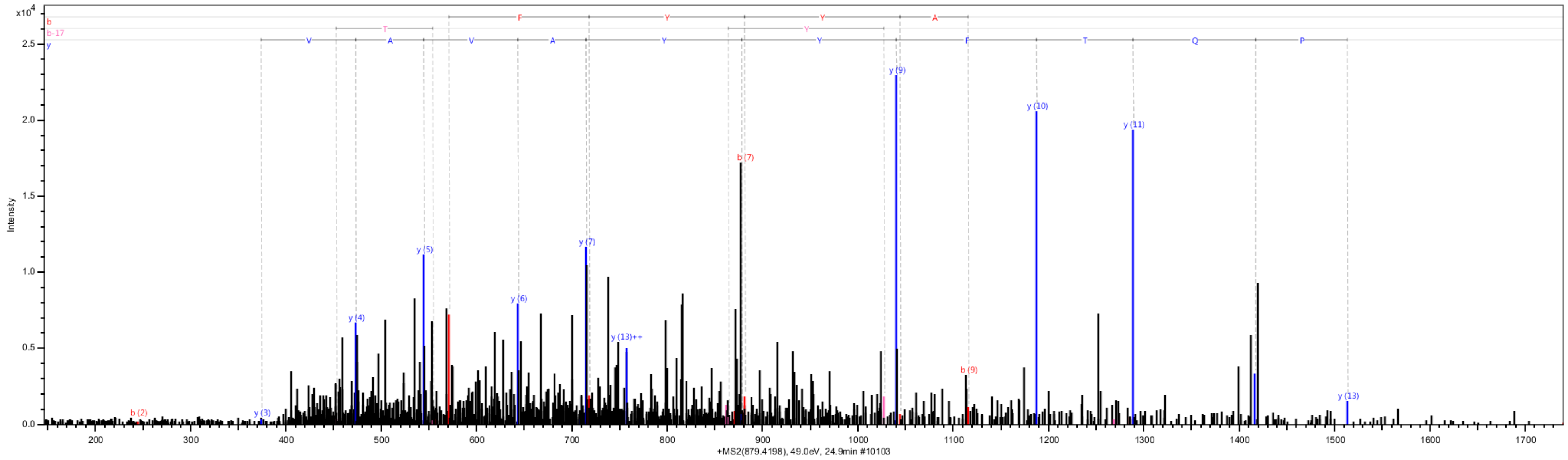

Peptide Match

**Sequence:**	K.KDSGFQMNQLR.G	**Score:**	16.3
**Modifications:**			

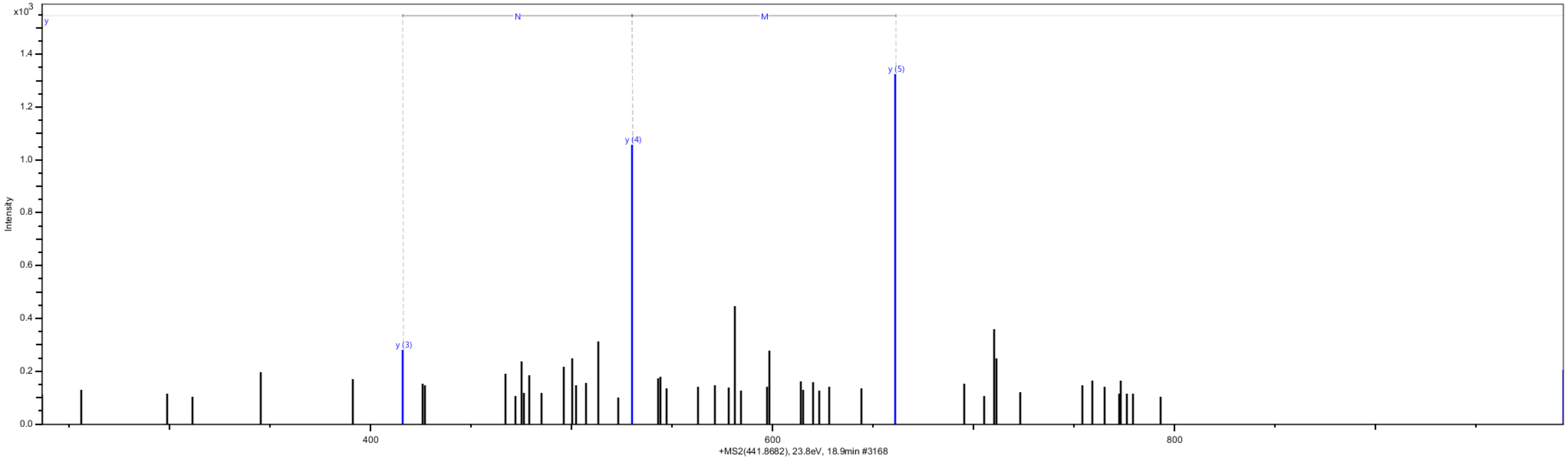

Peptide Match

Sequence: K.DSGFQMNQLR.G **Score:** 53.1

Modifications:

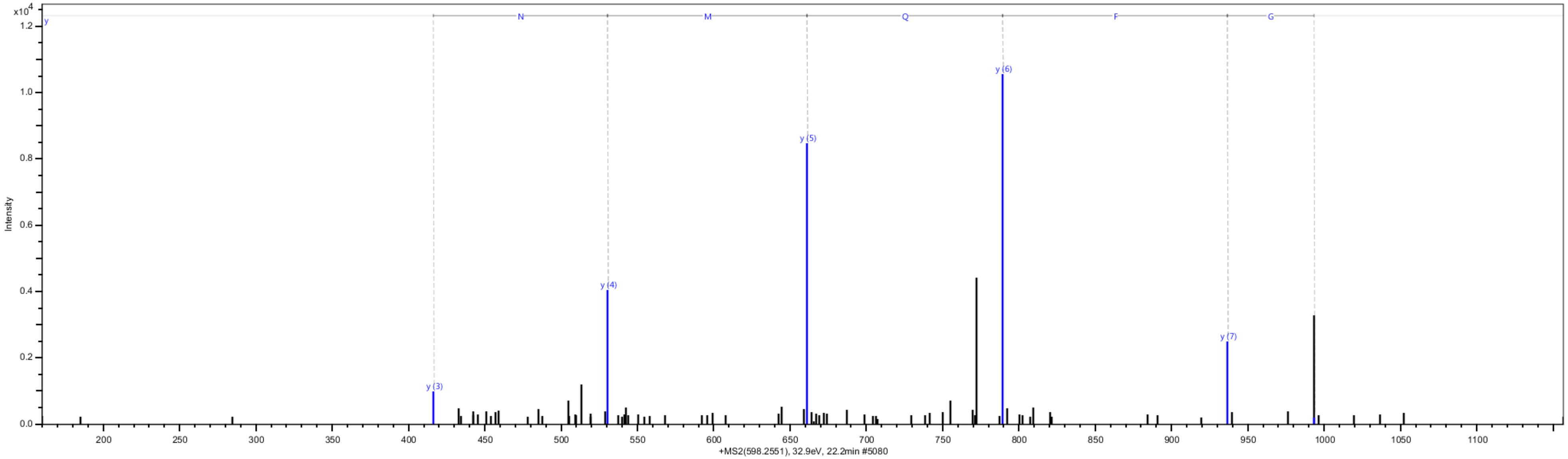

Peptide Match

Sequence: K.CLKDGAGDVAFVK.H **Score:** 74

Modifications: Carbamidomethyl: 1

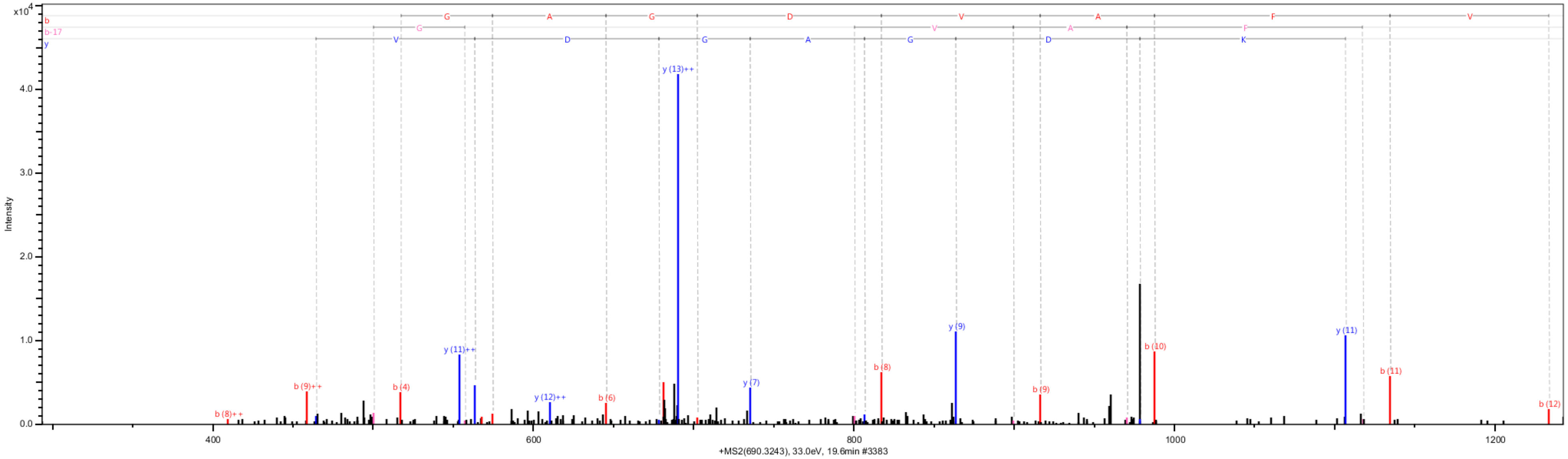

Peptide Match

Sequence: K.DGAGDVAFVK.H **Score:** 45.6

Modifications:

Spectrum Report
Combine Search_2020-10-19 07:53:31

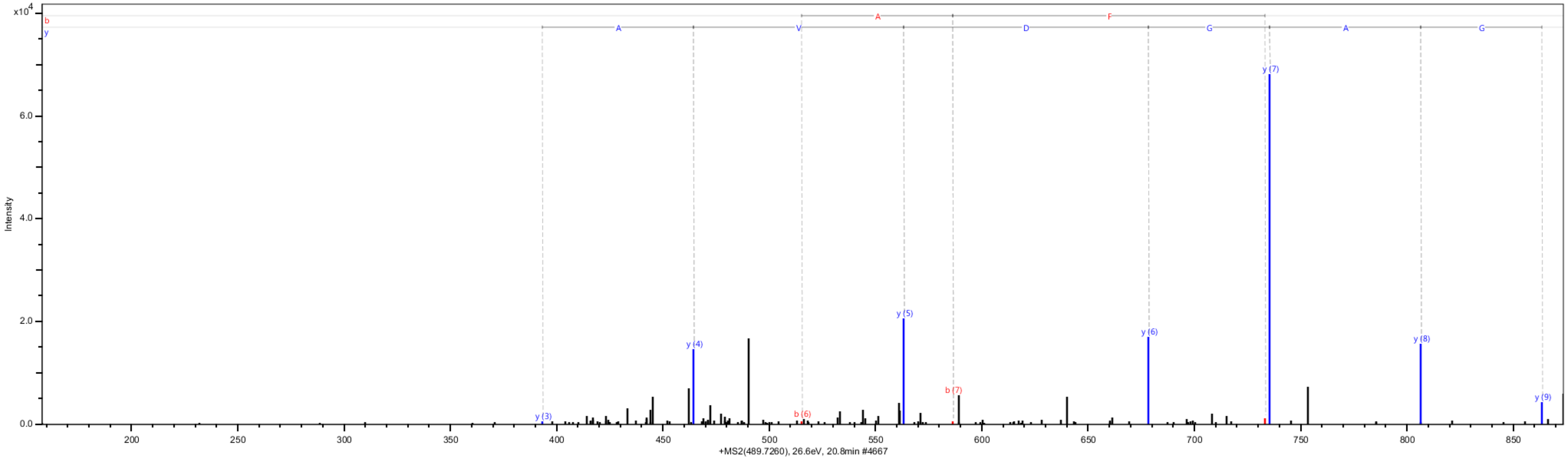

Peptide Match

Sequence: K.HSTIFENLANK.A **Score:** 57.9

Modifications:

Spectrum Report
Combine Search_2020-10-19 07:53:31

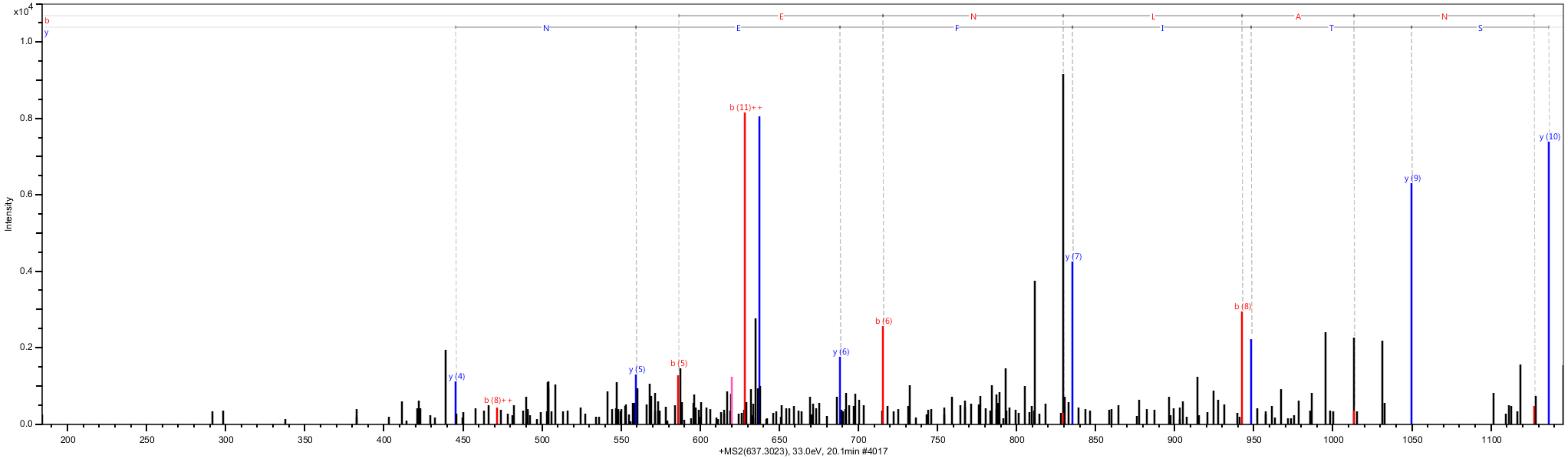

Peptide Match

Sequence: K.HSTIFENLANKADR.D **Score:** 21.5

Modifications:

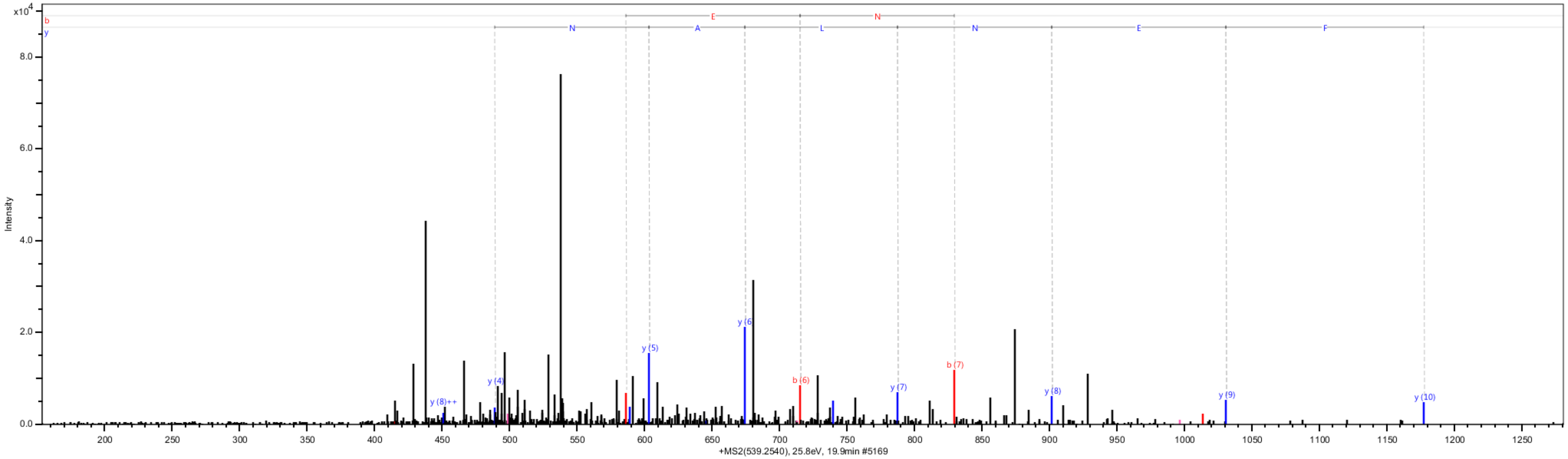

Peptide Match

Sequence: K.ADRDQYELLCLDNTR.K

Score: 42.9

Modifications: Carbamidomethyl: 10

Spectrum Report
Combine Search_2020-10-19 07:53:31

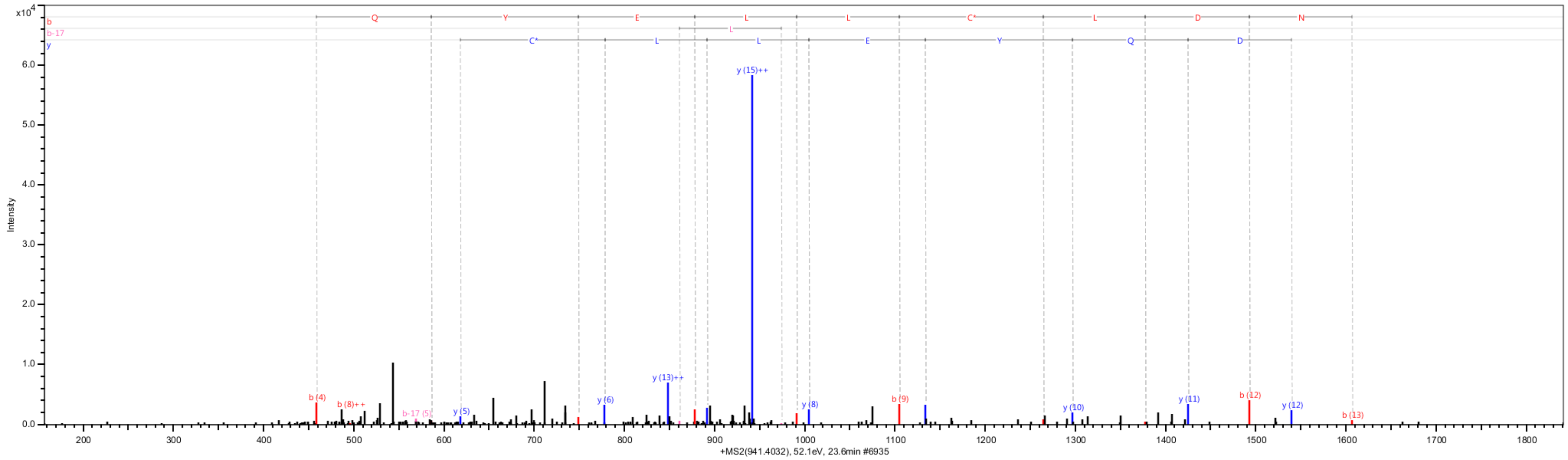

Peptide Match

**Sequence:**	R.DQYELLCLDNTR.K	**Score:**	47
**Modifications:**	Carbamidomethyl: 7		

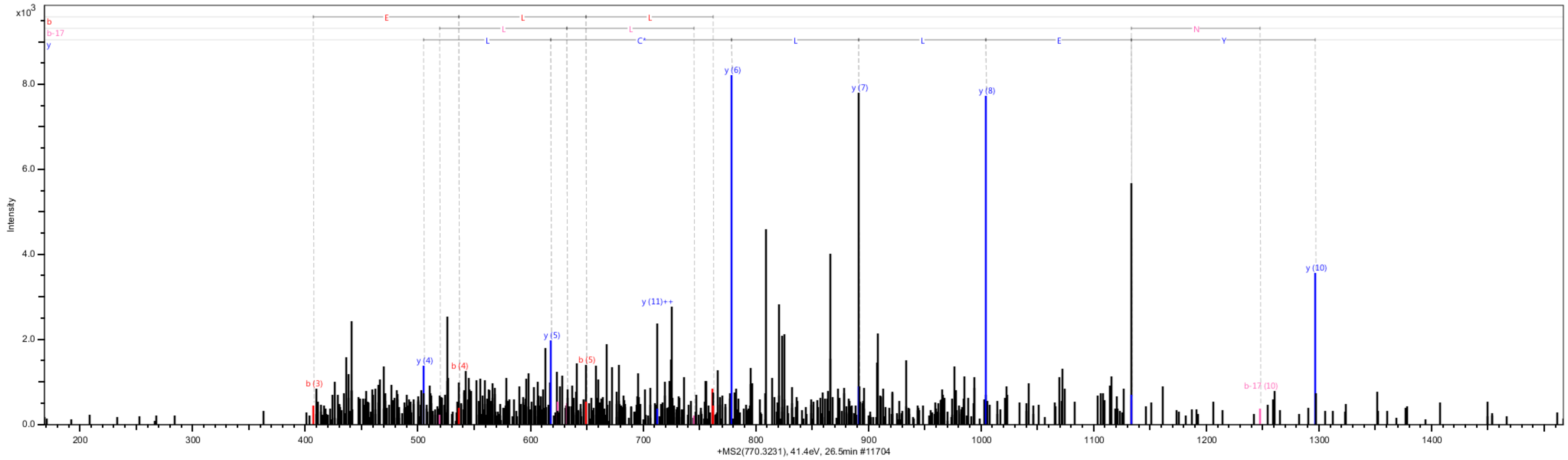

Peptide Match

**Sequence:**	K.DCHLAQVPSHTVVAR.S	**Score:**	87.9
**Modifications:**	Carbamidomethyl: 2		

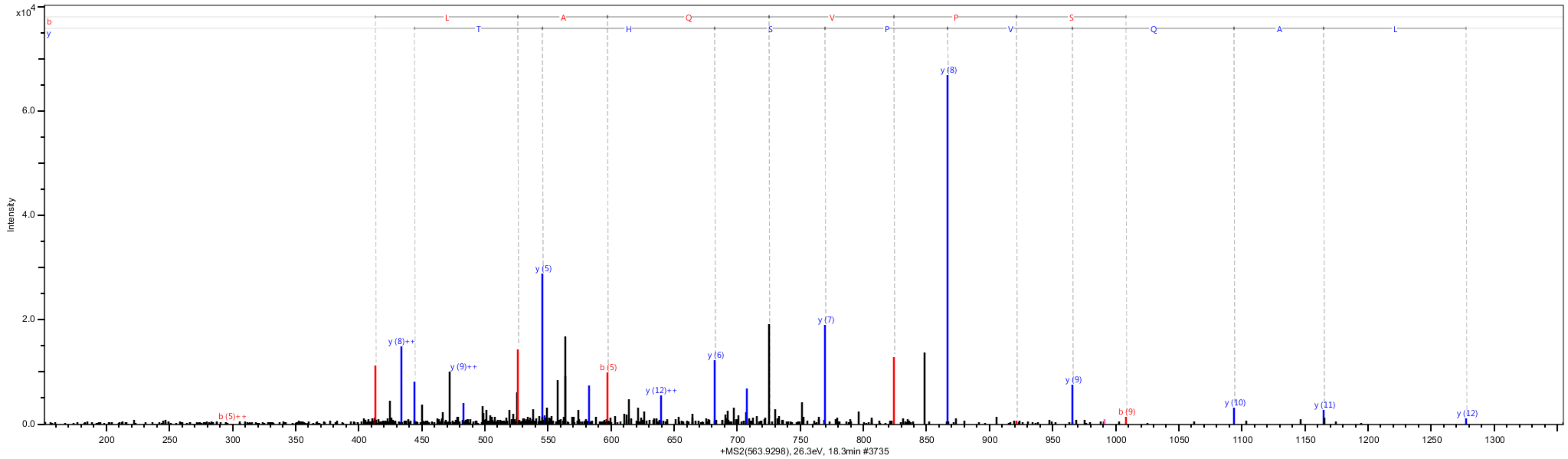

Peptide Match

Sequence: K.SKEFQLFSSPHGK.D **Score:** 49.4
Modifications:

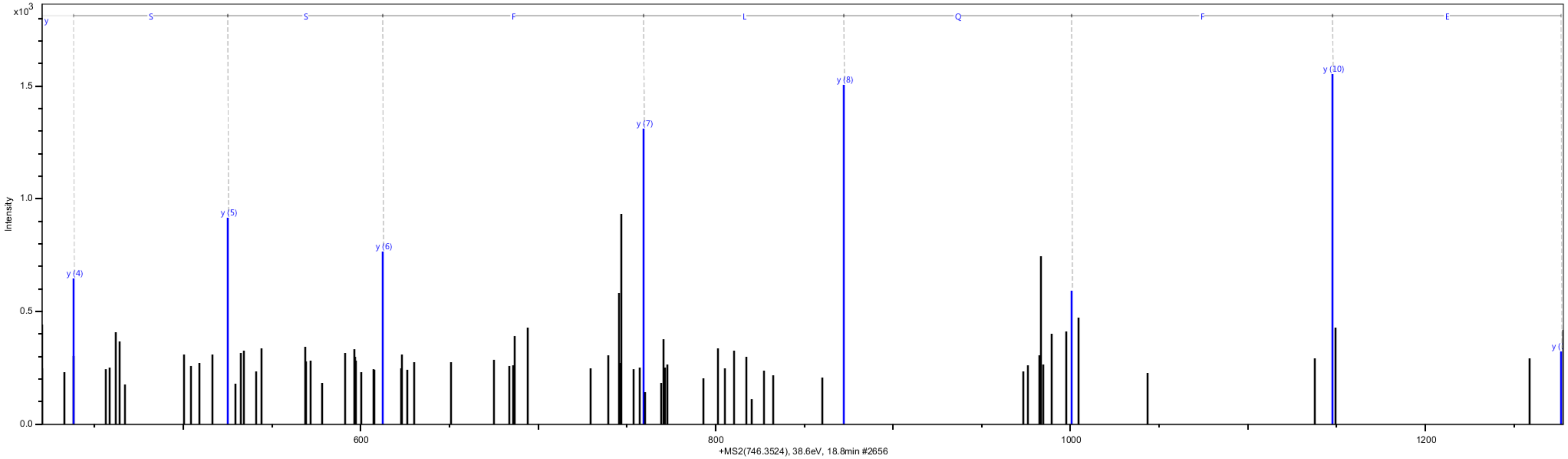

Peptide Match

Sequence: K.EFQLFSSPHGK.D **Score:** 50.8

Modifications:

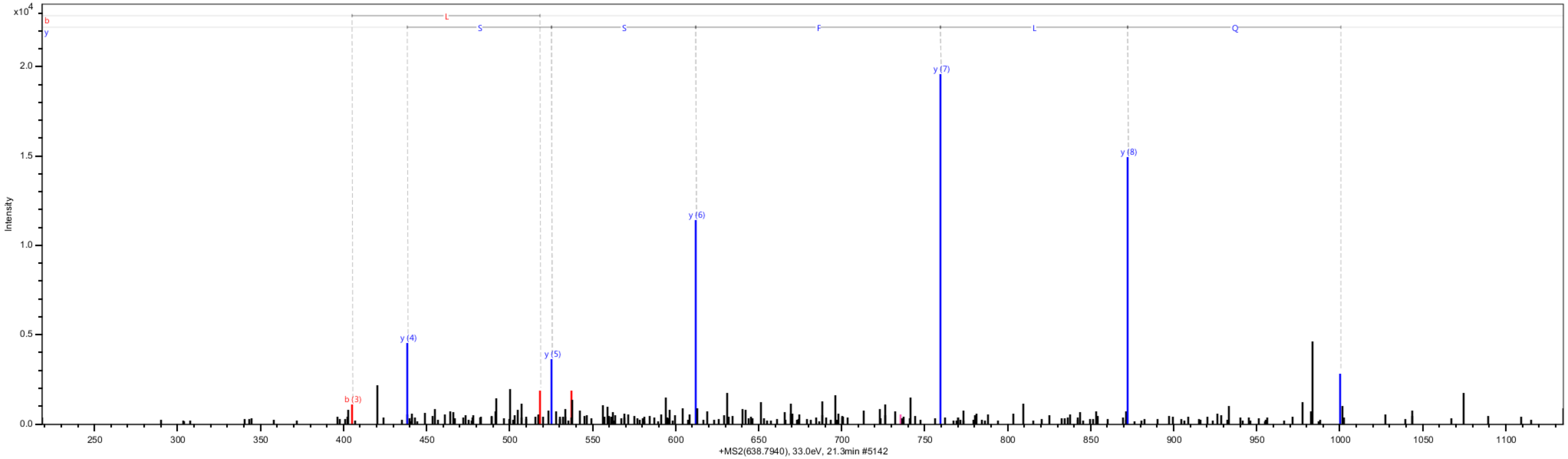

Peptide Match

Sequence: K.DLLFKDSAHGFLK.V **Score:** 58.9

Modifications:

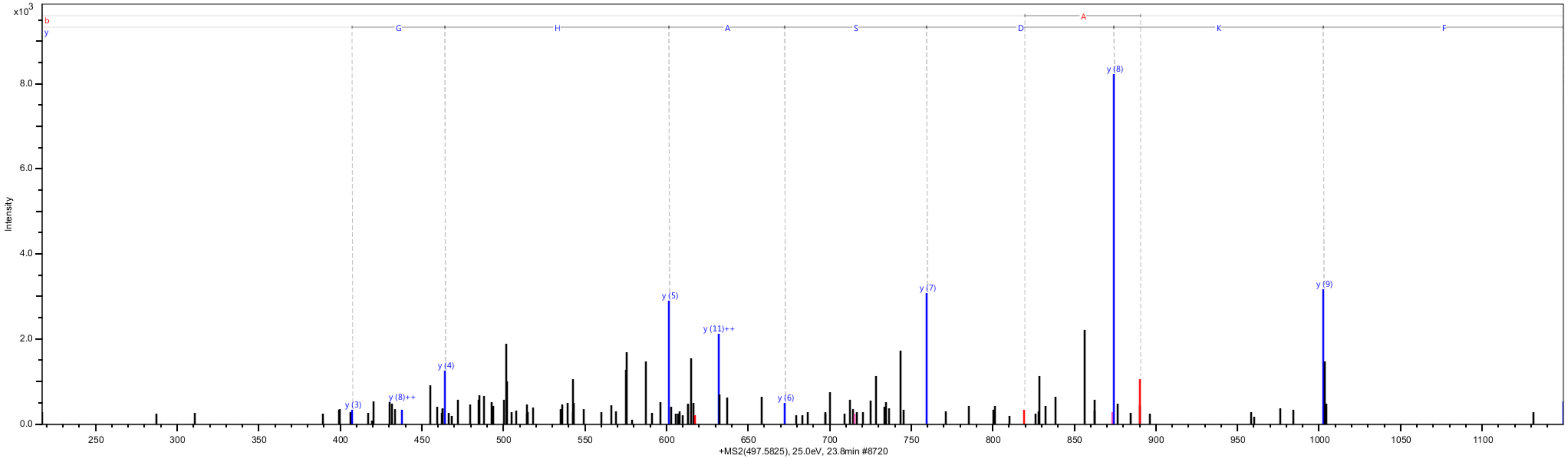

Peptide Match

**Sequence:**	K.DSAHGFLKVPPR.M	**Score:**	41.9
**Modifications:**			

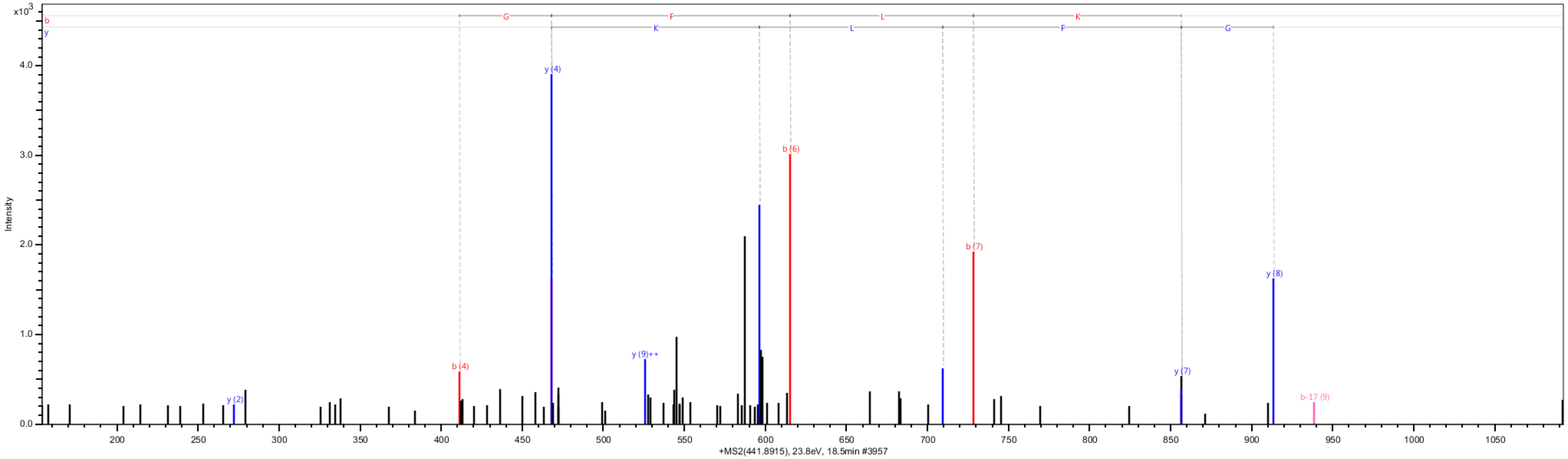

Peptide Match

Sequence: K.MYLGYEYVTAIR.N **Score:** 37.6

Modifications: Oxidation: 1

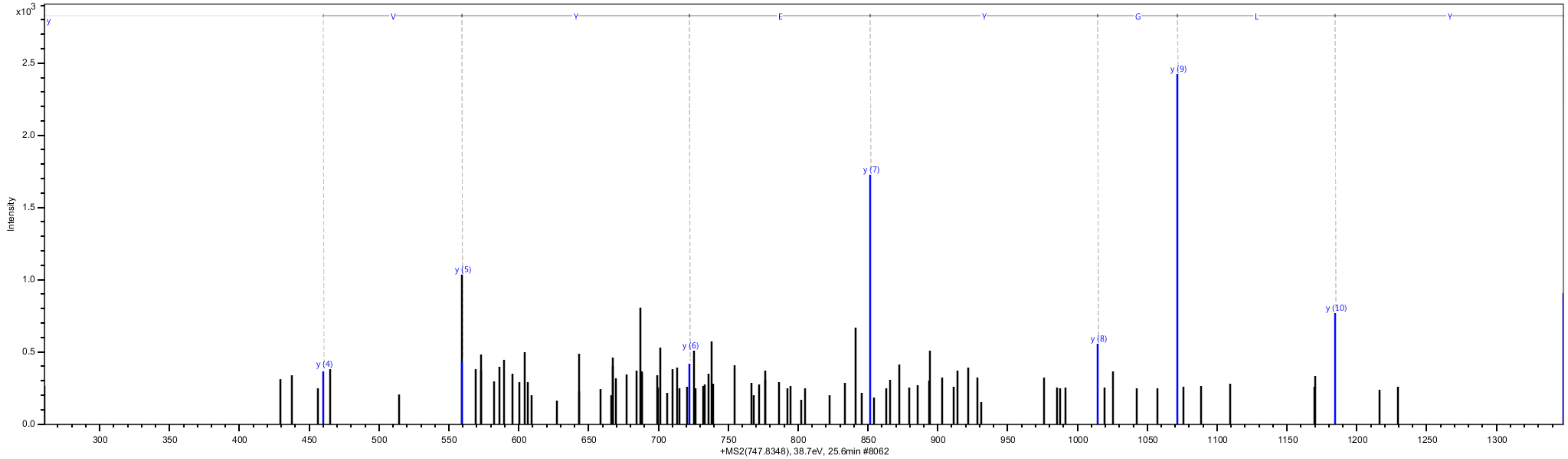

Peptide Match

Sequence: K.MYLGYEYVTAIR.N **Score:** 73.8
Modifications:

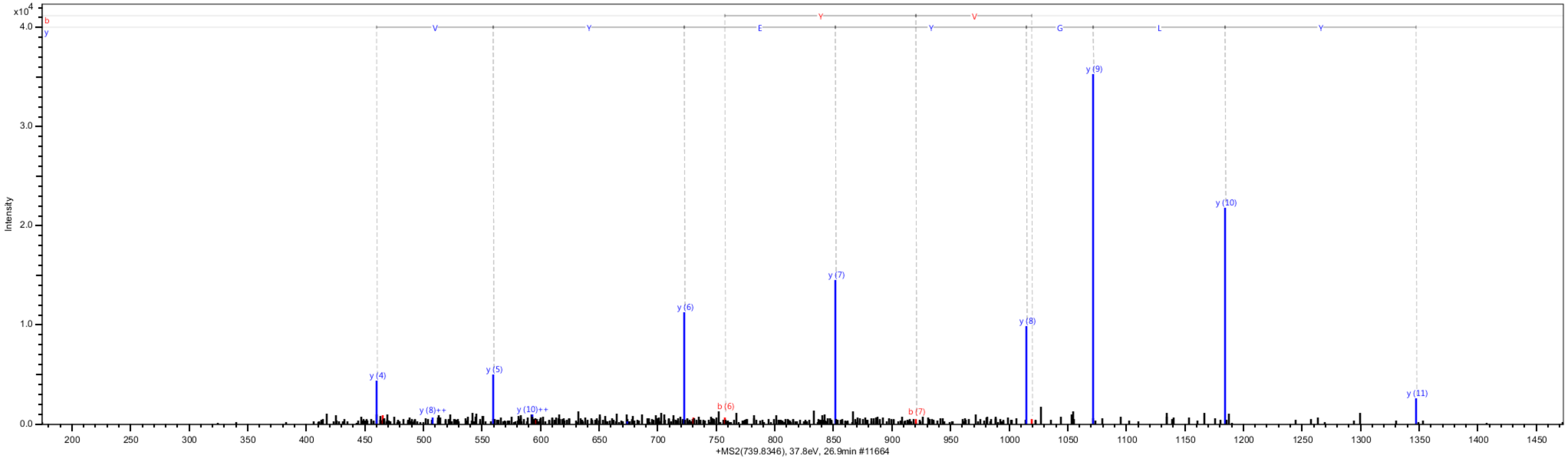

Peptide Match

**Sequence:**	R.EGTCPEAPTDECKPVK.W	**Score:**	53.7
**Modifications:**	Carbamidomethyl: 4, 12		

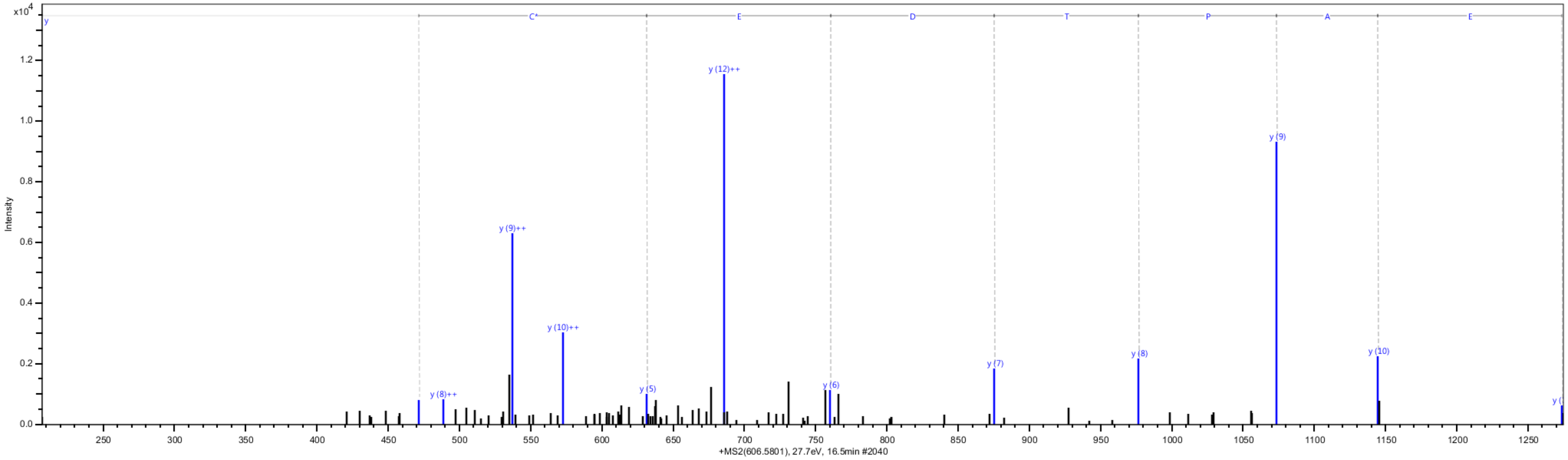

Peptide Match

**Sequence:**	R.LKCDEWSVNSVGK.I	**Score:**	68.3
**Modifications:**	Carbamidomethyl: 3		

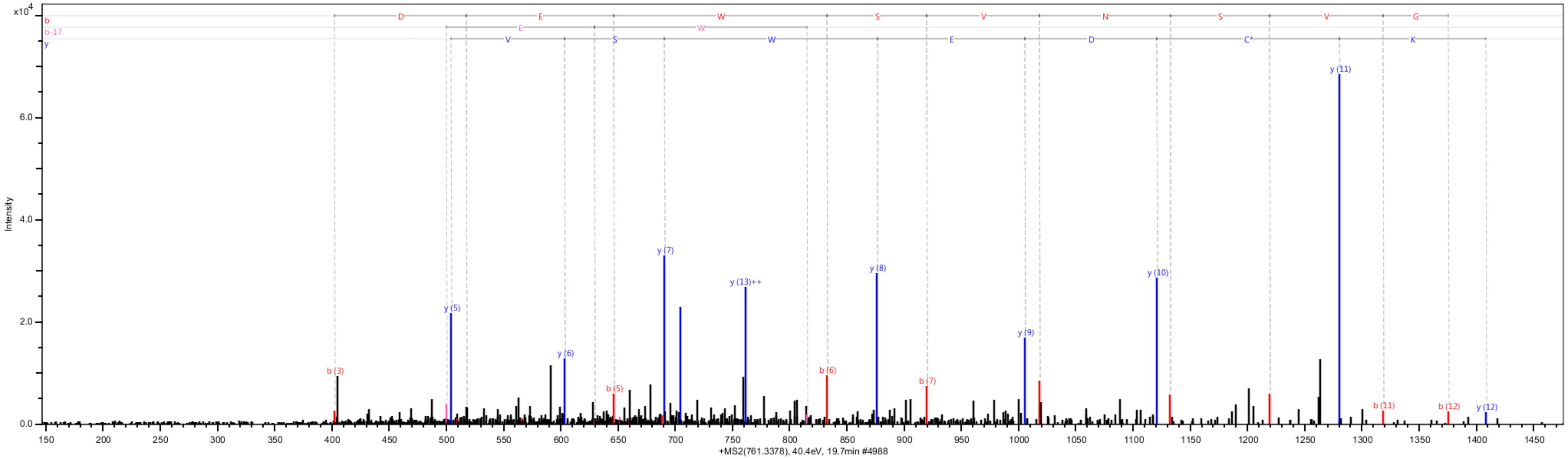

+MS2(761.3378), 40.4eV, 19.7min #4988

Peptide Match

**Sequence:**	K.IECVSAETTEDCIAK.I	**Score:**	109.6
**Modifications:**	Carbamidomethyl: 3, 12		

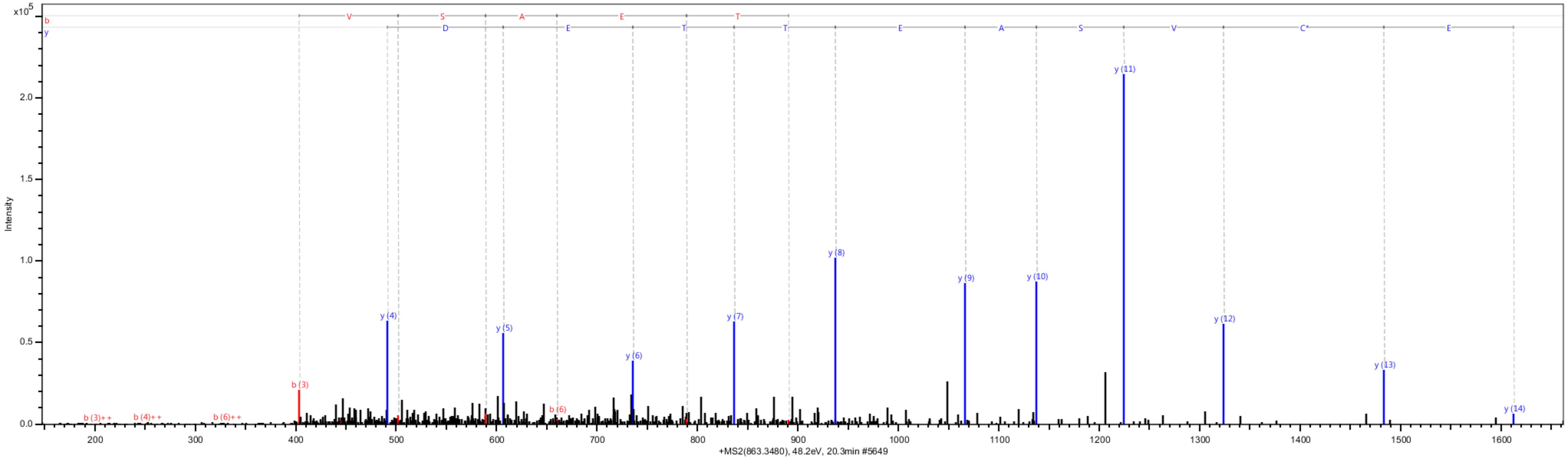

Peptide Match

Sequence: K.KSASDLTWDNLK.G **Score:** 88

Modifications:

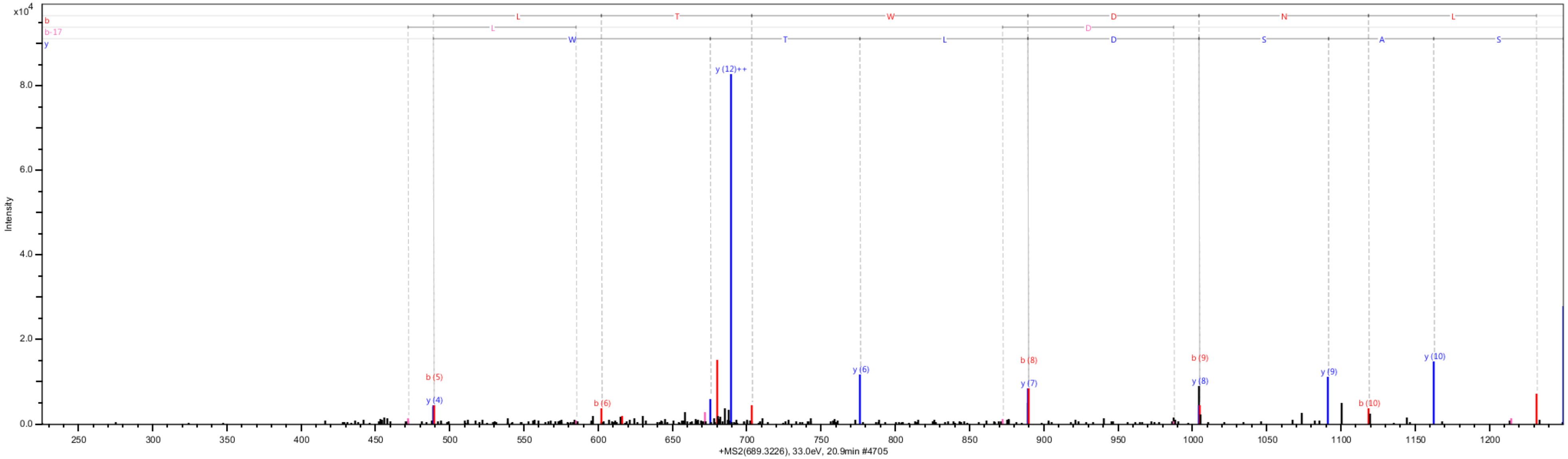

Peptide Match

Sequence: K.SASDLTWDNLK.G **Score:** 58.7

Modifications:

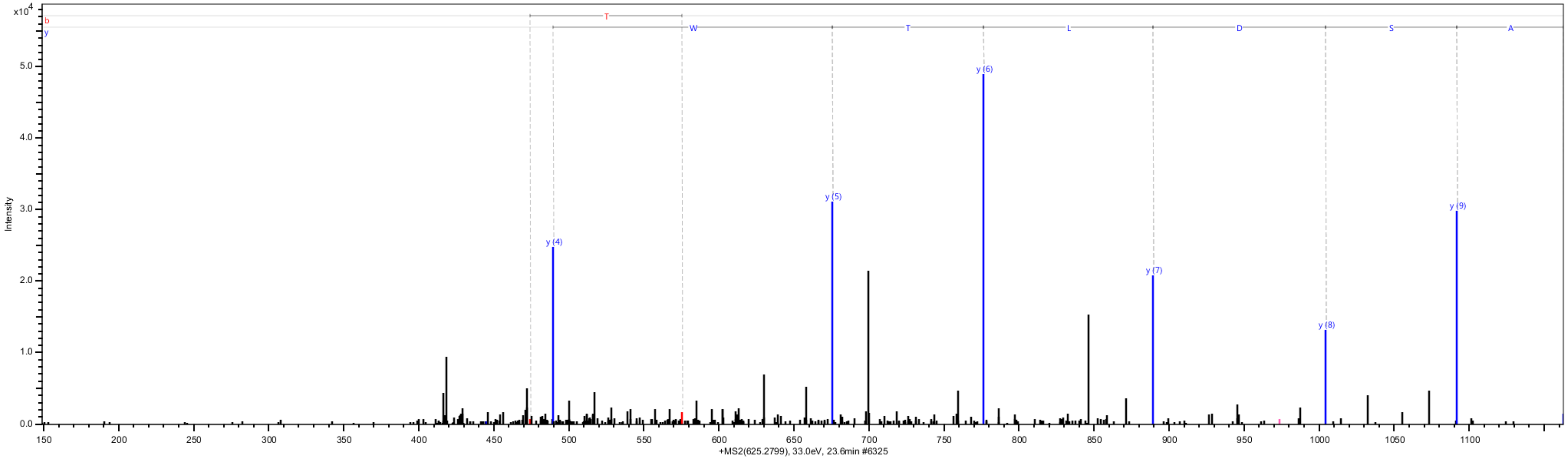

Peptide Match

Sequence: K.SASDLTWDNLKGK.K **Score:** 56.6

Modifications:

Spectrum Report
Combine Search_2020-10-19 07:53:31

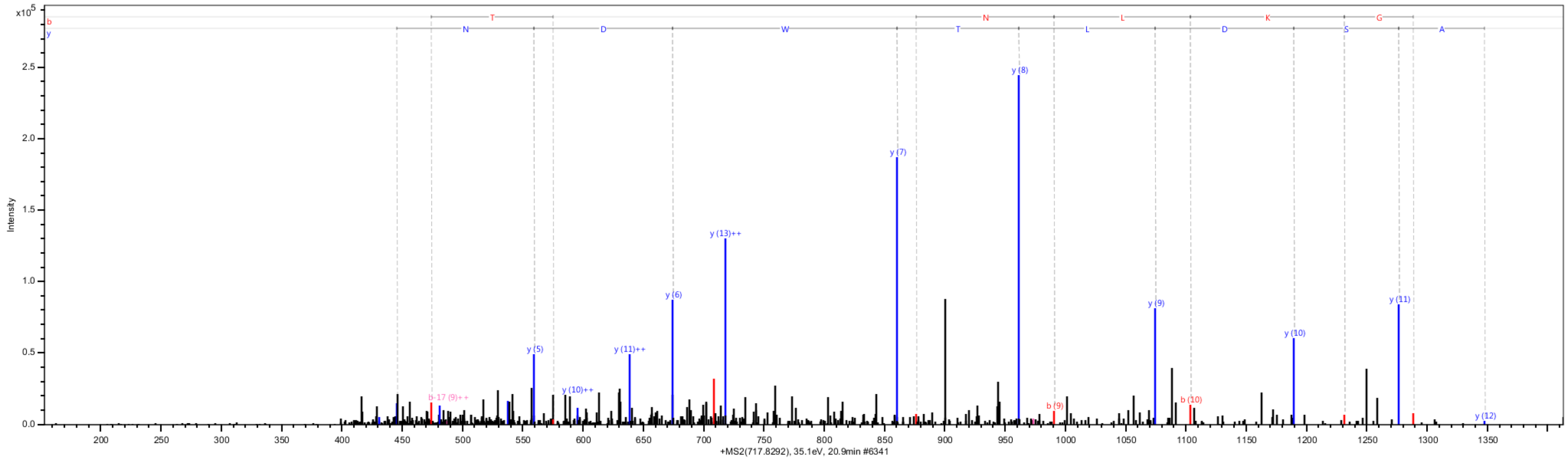

Peptide Match

Sequence: R.TAGWNIPMGLLYNK.I **Score:** 89.6

Modifications:

Spectrum Report
Combine Search_2020-10-19 07:53:31

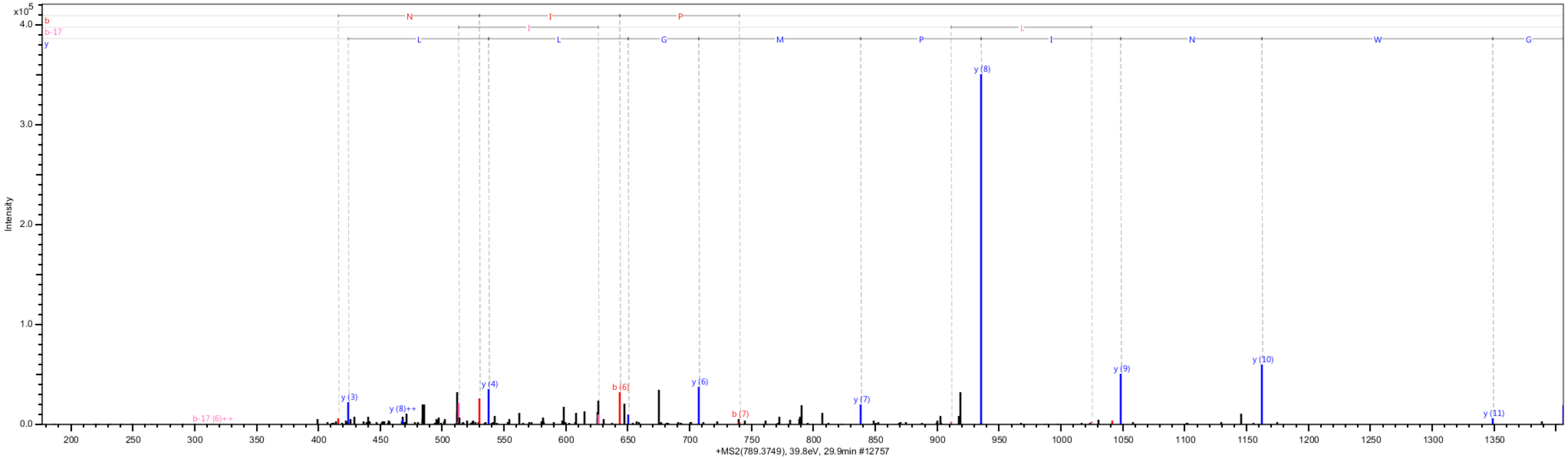

Peptide Match

**Sequence:**	R.FDEFFSEGCAPGSK.K	**Score:**	72.8
**Modifications:**	Carbamidomethyl: 9		

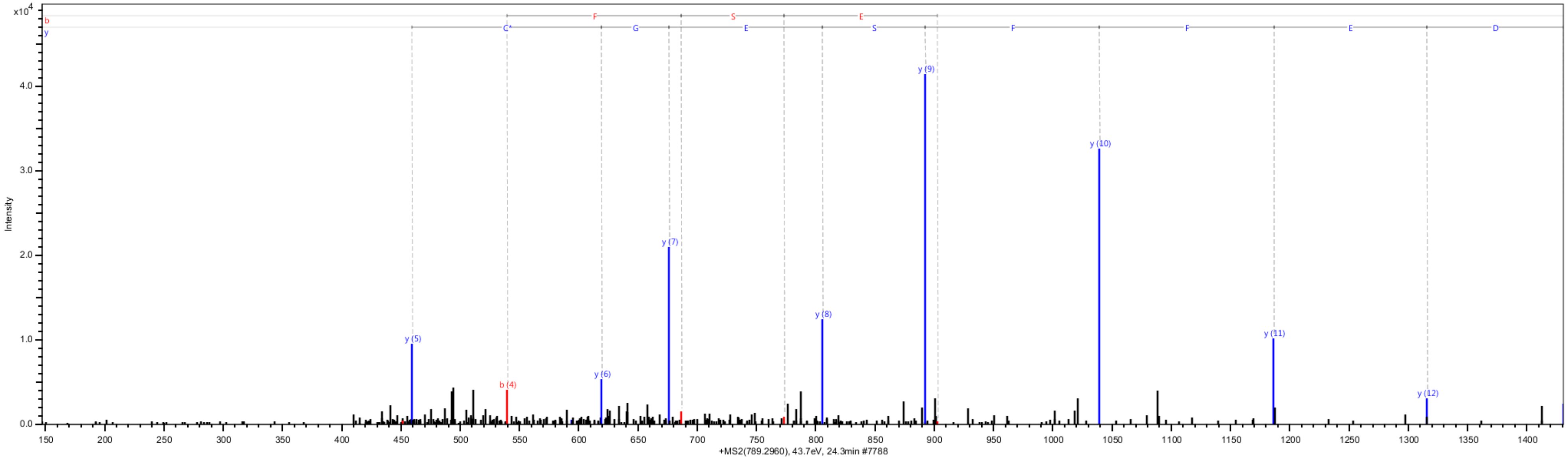

Peptide Match

Sequence: R.FDEFFSEGCAPGSKK.D **Score:** 55

Modifications: Carbamidomethyl: 9

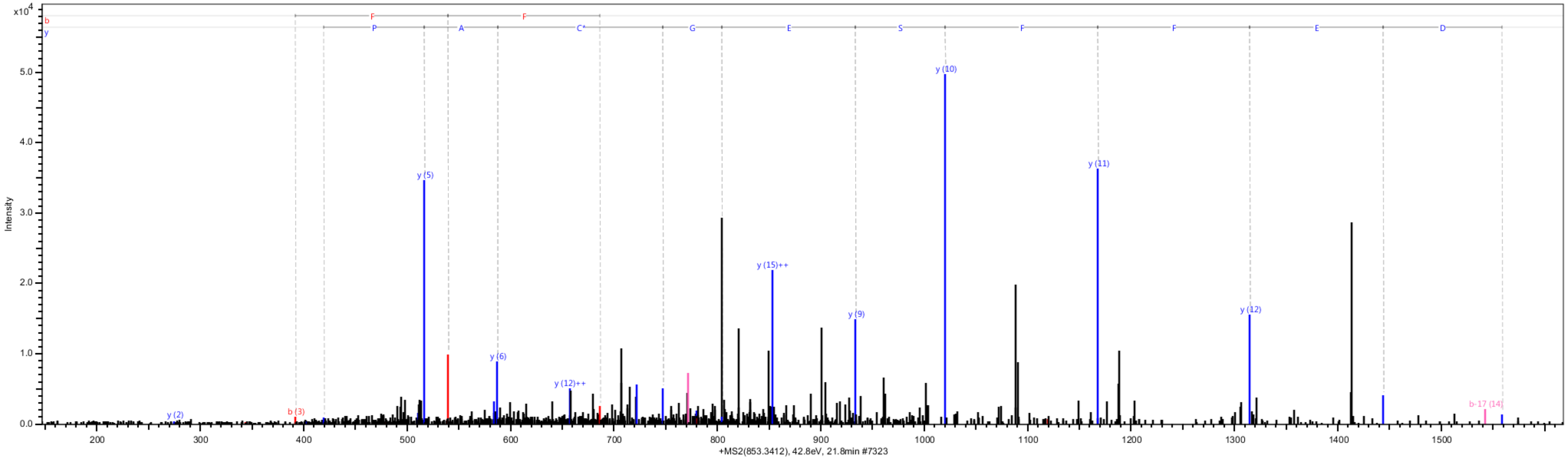

Peptide Match

Sequence: K.LCMGSGLNLCEPNNK.E **Score:** 56.4

Modifications: Carbamidomethyl: 2, 10

Spectrum Report
Combine Search_2020-10-19 07:53:31

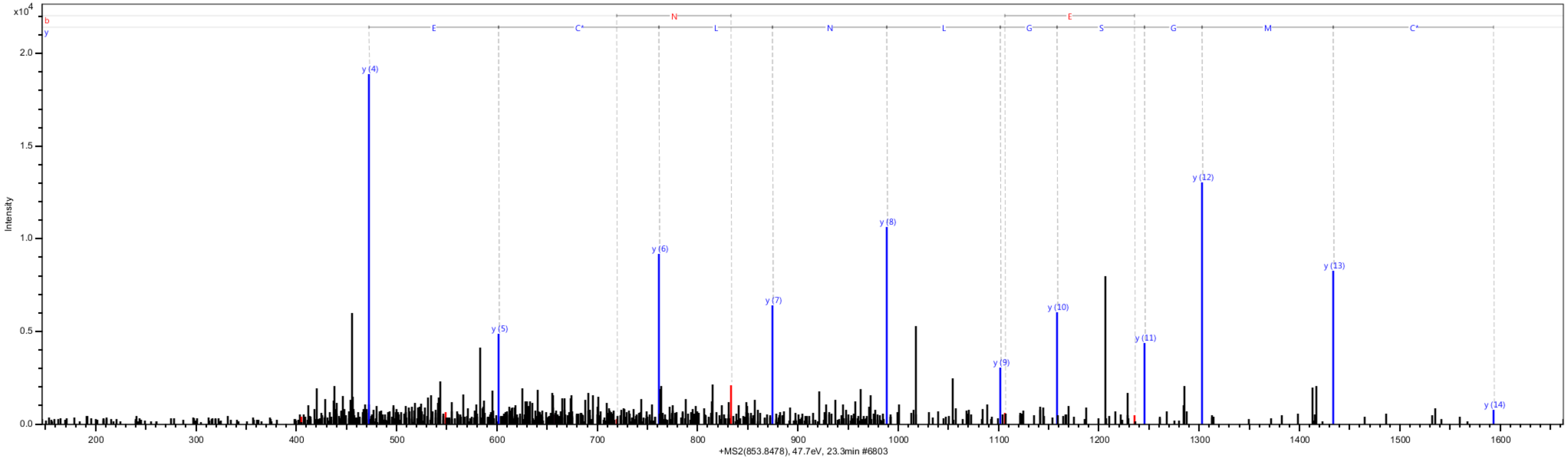

Peptide Match

Sequence: K.EGYYGYTGAFR.C **Score:** 69.6

Modifications:

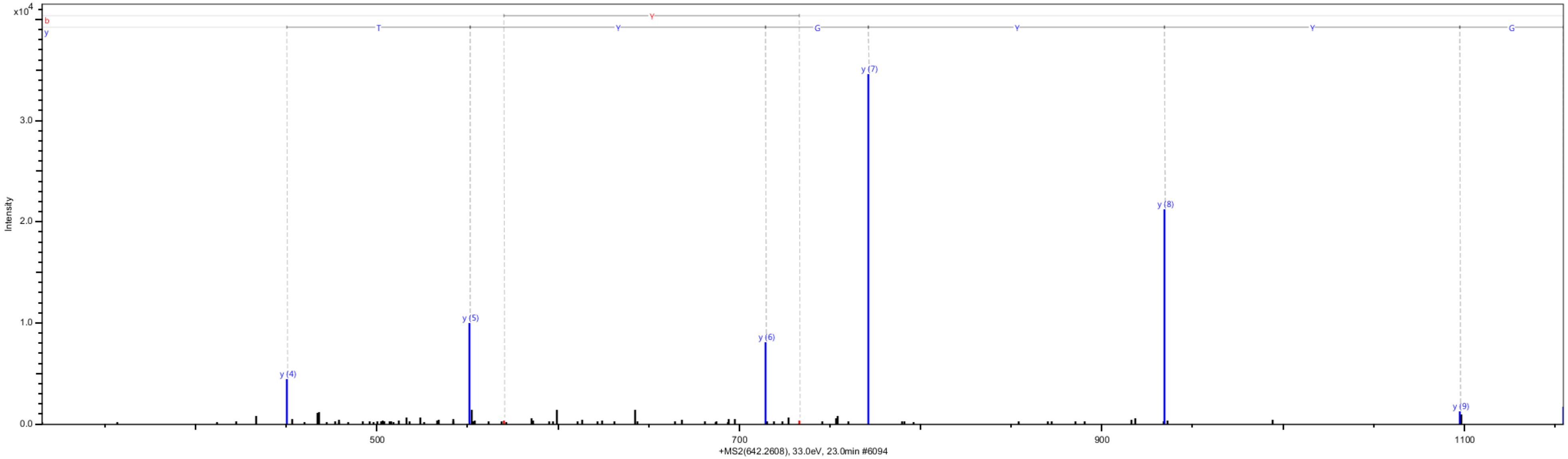

Peptide Match

**Sequence:**	R.CLVEKGDVAFVK.H	**Score:**	66.3
**Modifications:**	Carbamidomethyl: 1		

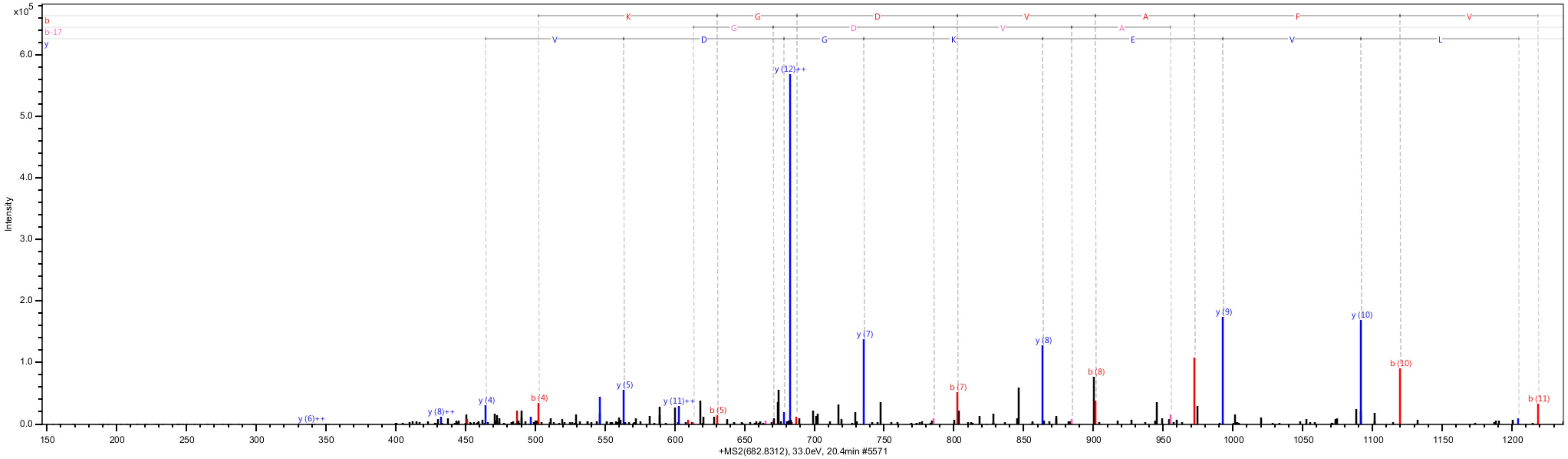

Peptide Match

Sequence: K.NLNEKDYELLCLDGTR.K **Score:** 96.7
Modifications: Carbamidomethyl: 11

Spectrum Report
Combine Search_2020-10-19 07:53:31

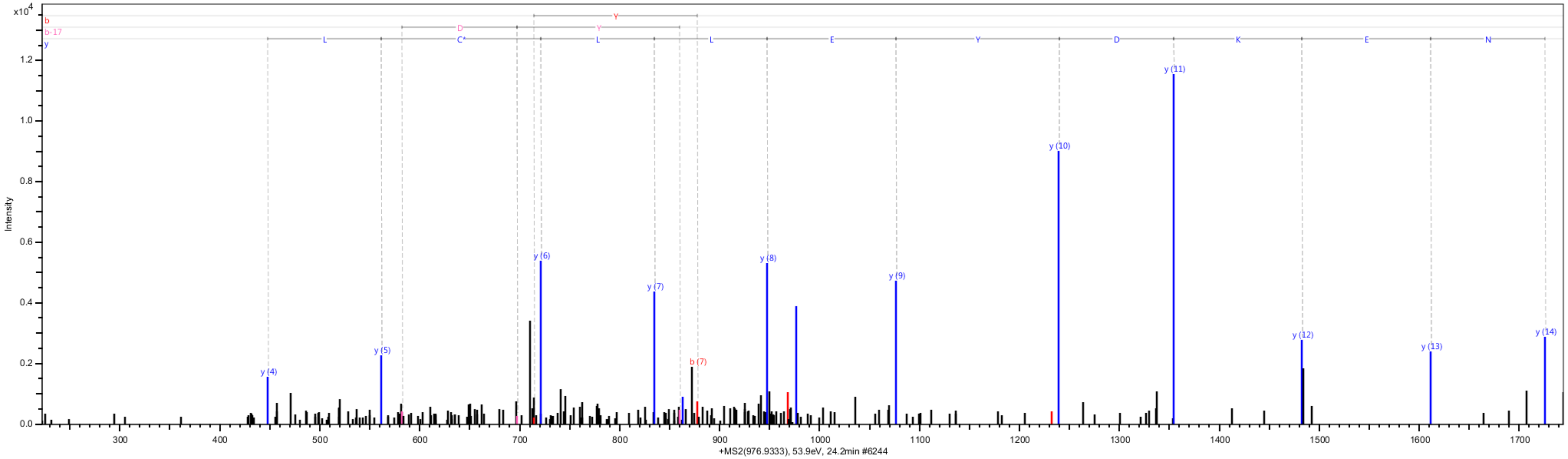

Peptide Match

Sequence: R.KPVEEYANCHLAR.A **Score:** 89.7
Modifications: Carbamidomethyl: 9

Spectrum Report
Combine Search_2020-10-19 07:53:31

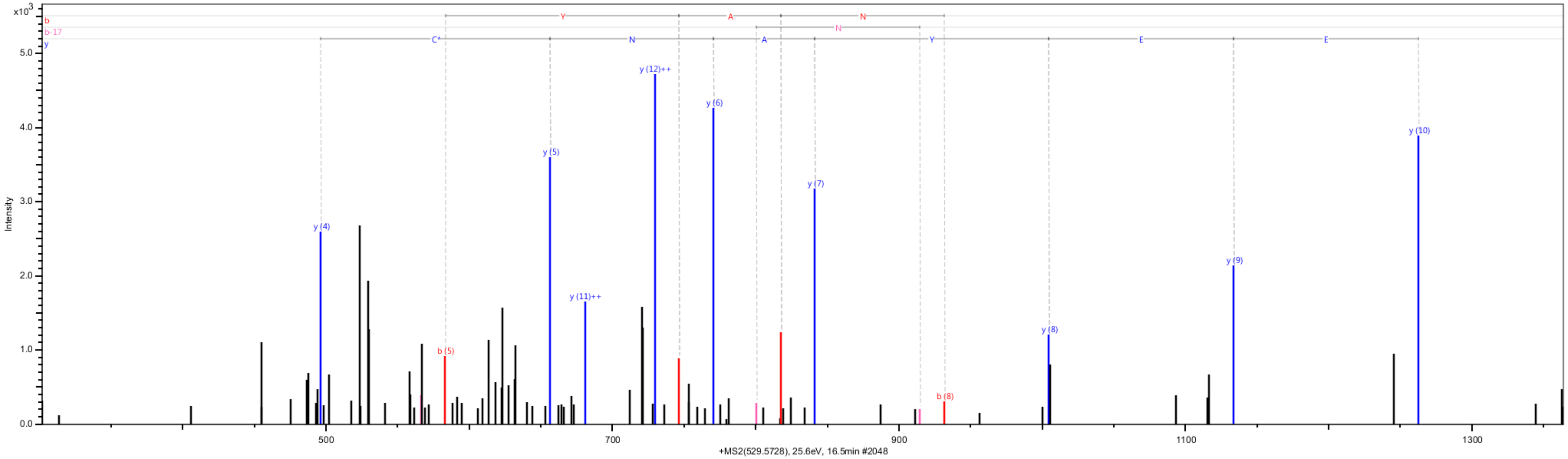

Peptide Match

Sequence: R.SETKDLLFR.D **Score:** 22.3

Modifications:

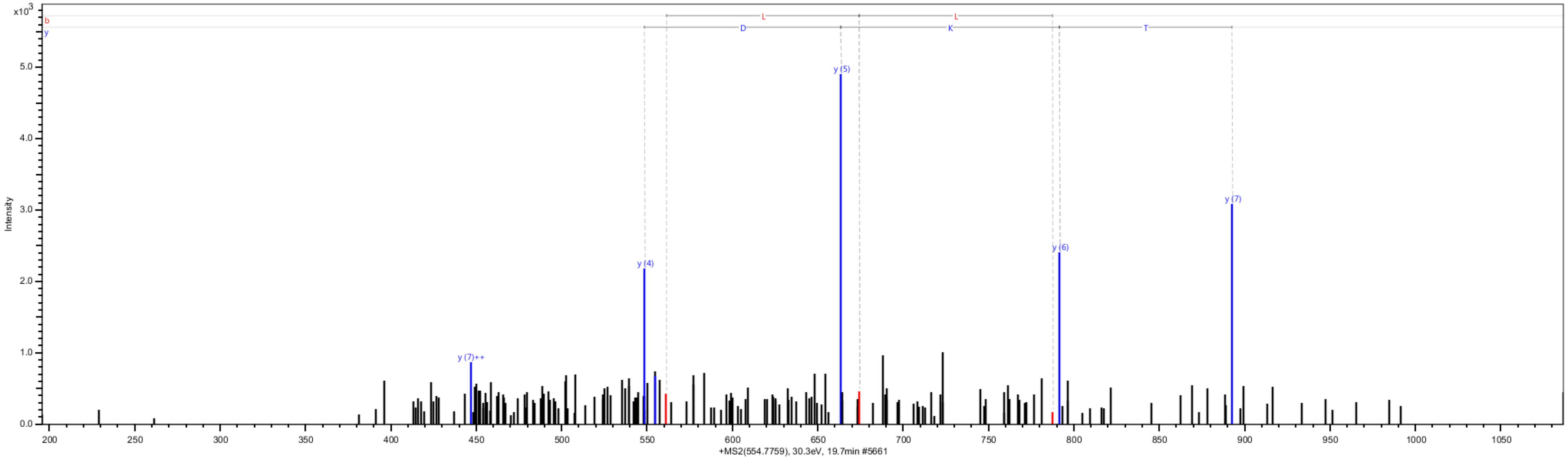

Peptide Match

Sequence: K.DLLFRDDTVCLAK.L **Score:** 67.6

Modifications: Carbamidomethyl: 10

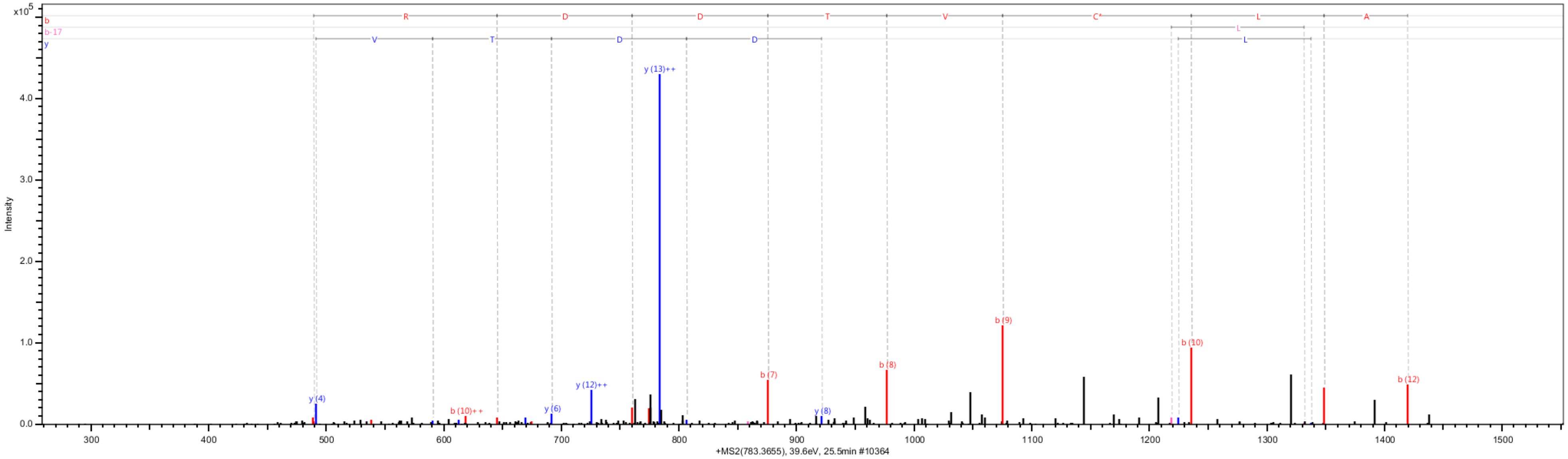

Peptide Match

Sequence: R.NTYEKYLGEEYVK.A **Score:** 70.6

Modifications:

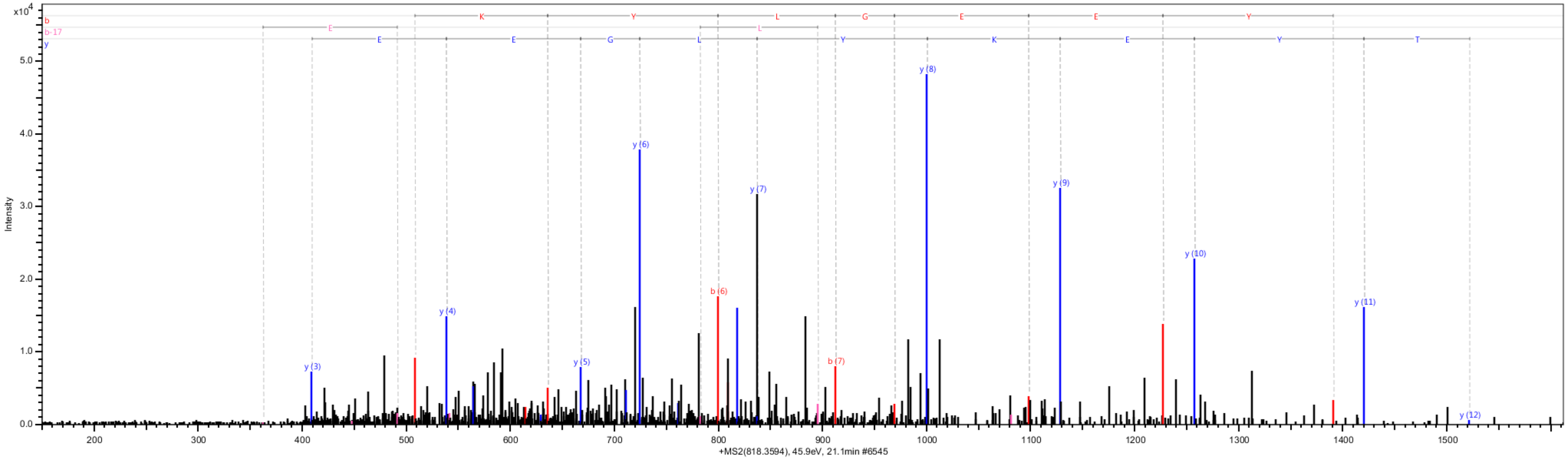

Peptide Match

Sequence: K.YLGEEYVK.A **Score:** 41.2

Modifications:

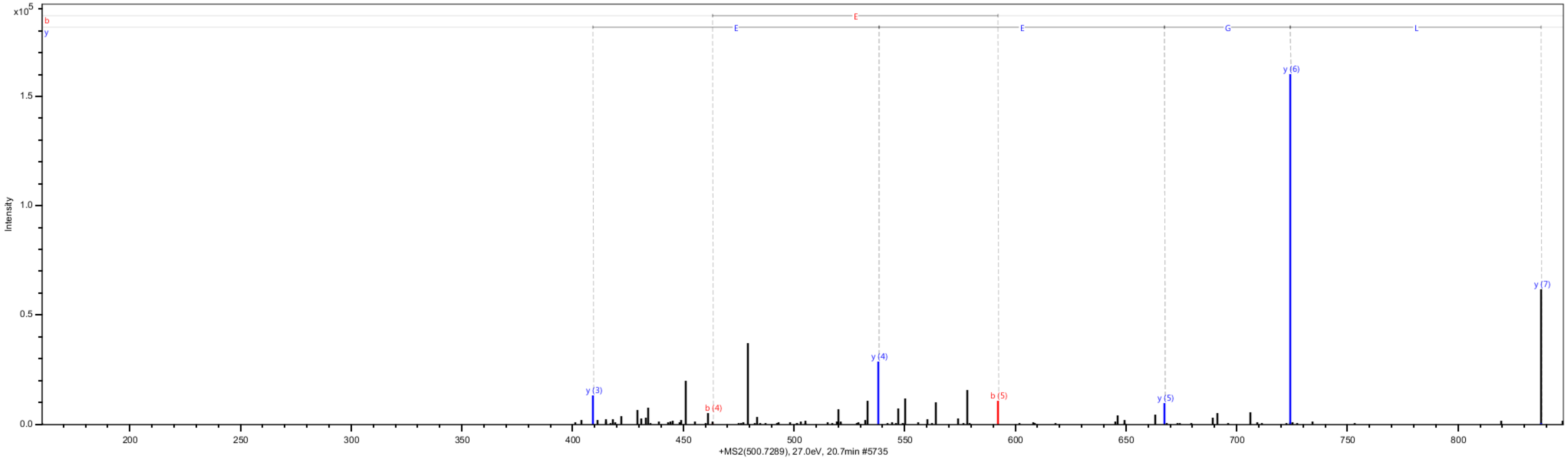

x10^5
b
y
E
E
E
G
L
y (6)
y (7)
y (4)
y (3)
b (4)
b (5)
y (5)
1.5
1.0
0.5
0.0
Intensity
200
250
300
350
400
450
500
550
600
650
700
750
800
+MS2(500.7289), 27.0eV, 20.7min #5735

Peptide Match

Sequence: R.KCSTSSLLEACTFR.R **Score:** 102.6
Modifications: Carbamidomethyl: 2, 11

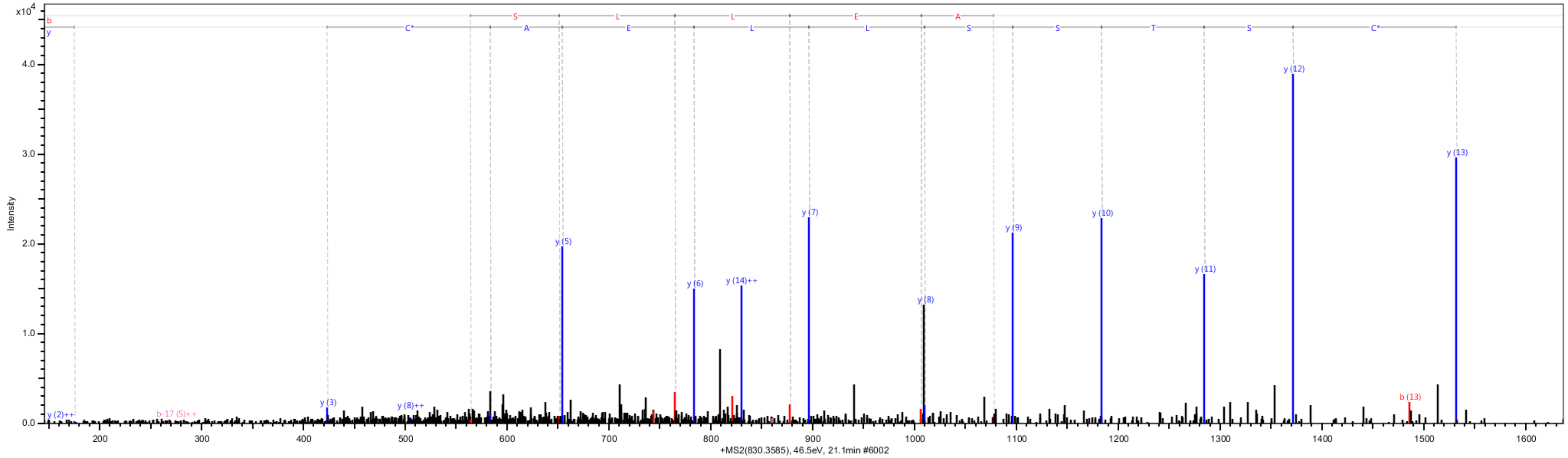

Peptide Match

Sequence: K.CSTSSLLEACTFR.R **Score:** 106.6
Modifications: Carbamidomethyl: 1, 10

Spectrum Report
Combine Search_2020-10-19 07:53:31

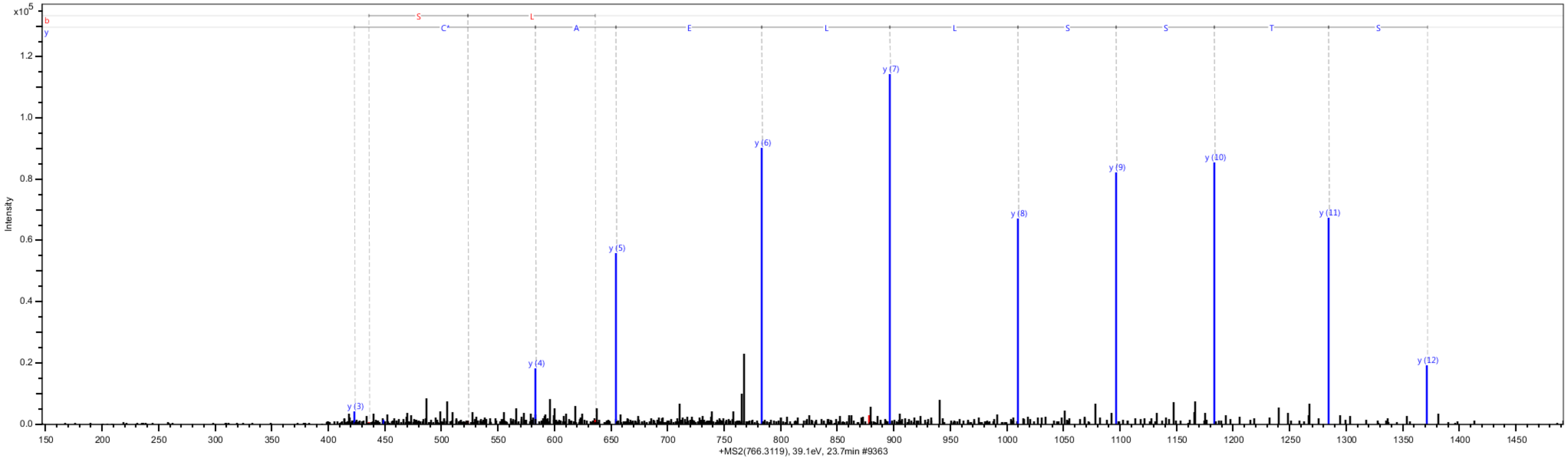

Peptide Match

**Sequence:**	K.CSTSSLLEACTFRRP.-	**Score:**	51.7
**Modifications:**	Carbamidomethyl: 1, 10		

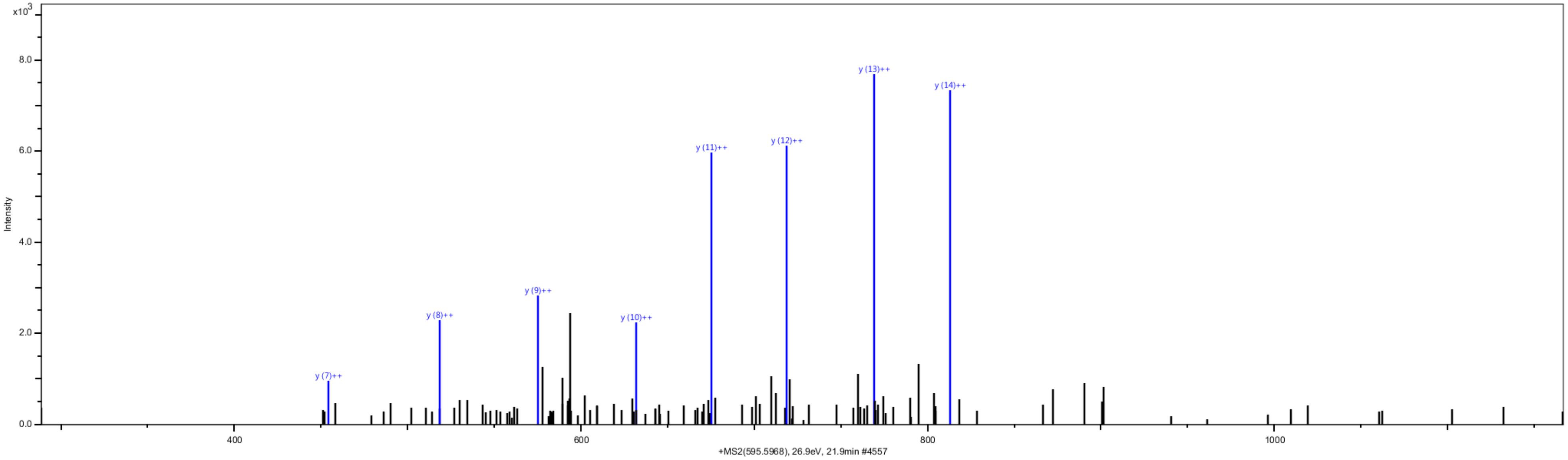
x10^3
8.0
6.0
4.0
2.0
0.0
Intensity
y (7)++
y (8)++
y (9)++
y (10)++
y (11)++
y (12)++
y (13)++
y (14)++
400
600
800
1000
+MS2(595.5968), 26.9eV, 21.9min #4557

CO3_HUMAN

Accession: CO3_HUMAN
Database: SwissProt
MW [kDa] / pI: 187.0 / 6.0
Score: 1298.8
Seq. Coverage [%]: 20.4
No. of Peptides: 37

Peptide Match

Sequence: K.TIYTPGSTVLYR.I
Score: 40
Modifications:

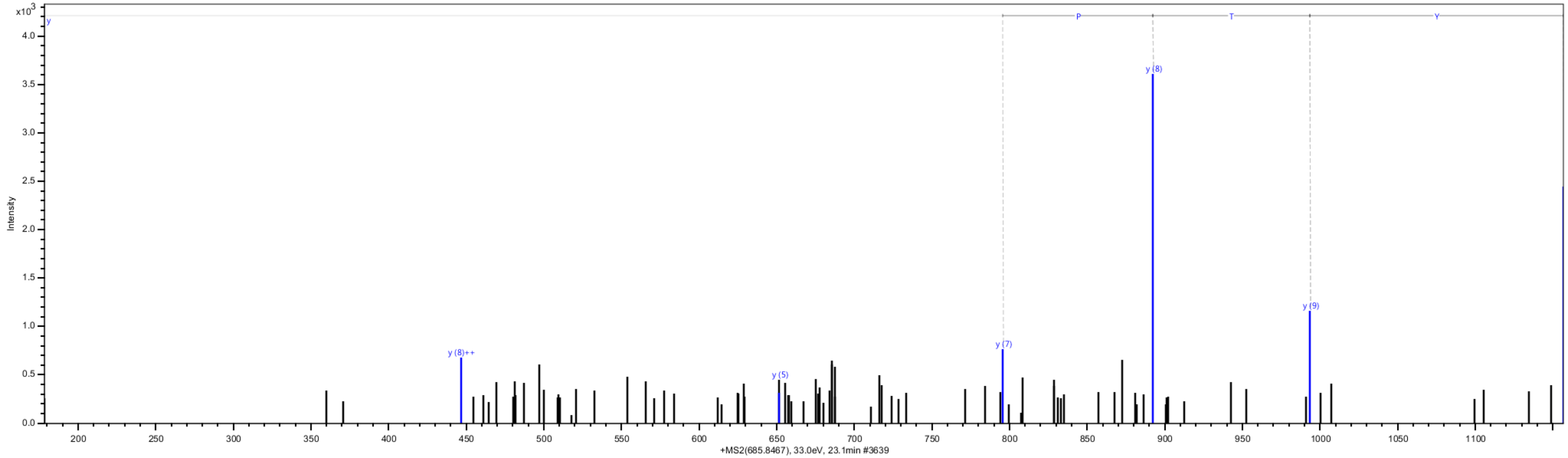

Peptide Match

Sequence: R.ISLPESLK.R
Score: 22.4
Modifications:

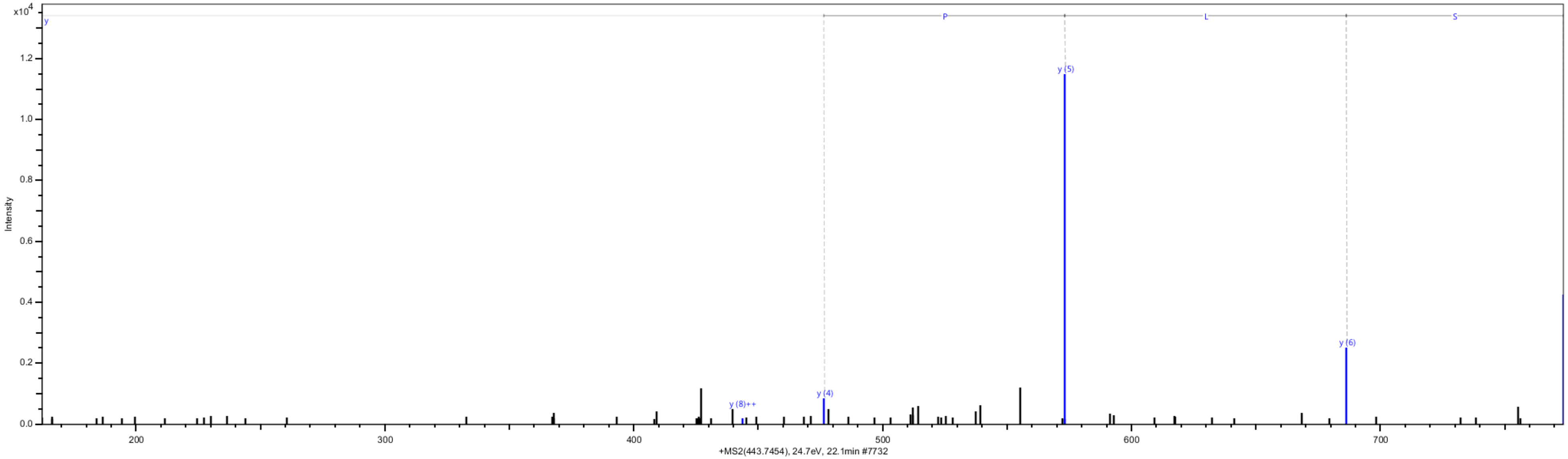

Peptide Match

**Sequence:**	R.ISLPESLKR.I	**Score:**	41.8
**Modifications:**			

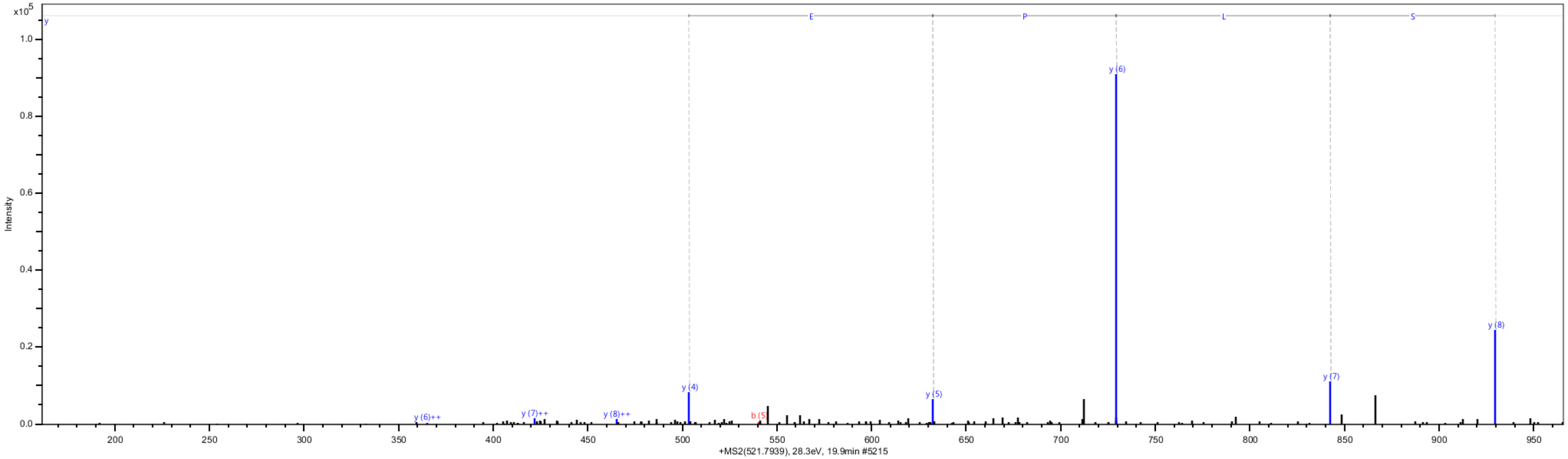

Peptide Match

Sequence: K.RIPIEDGSGEVVLSR.K **Score:** 40

Modifications:

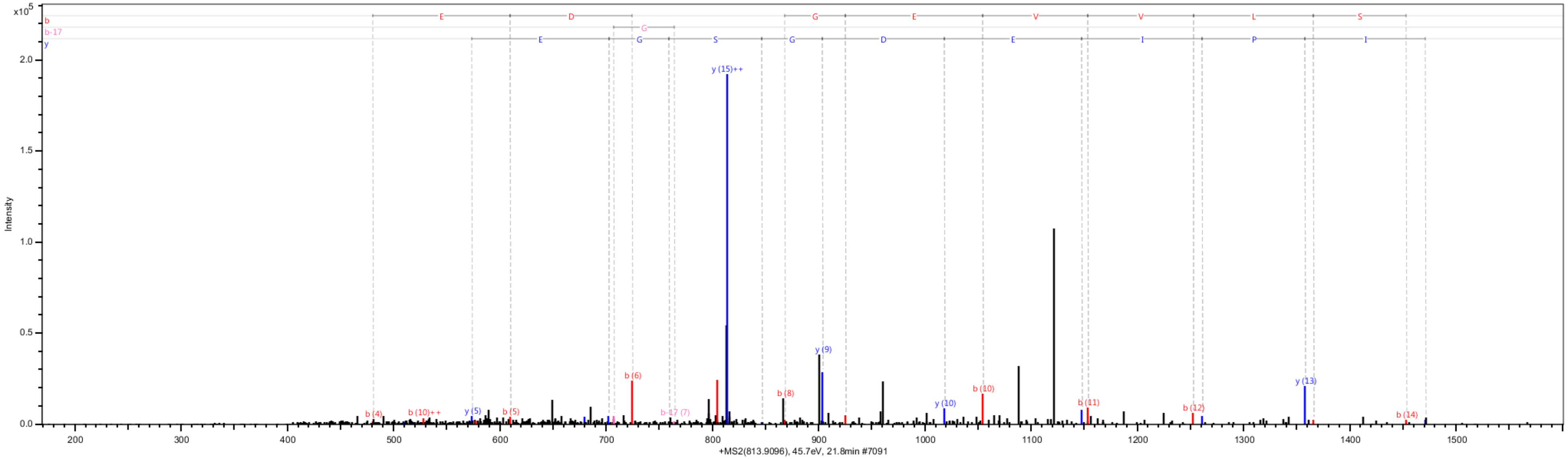

Peptide Match

Sequence: R.IPIEDGSGEVVLSR.K **Score:** 44.8

Modifications:

Spectrum Report
Combine Search_2020-10-19 07:53:31

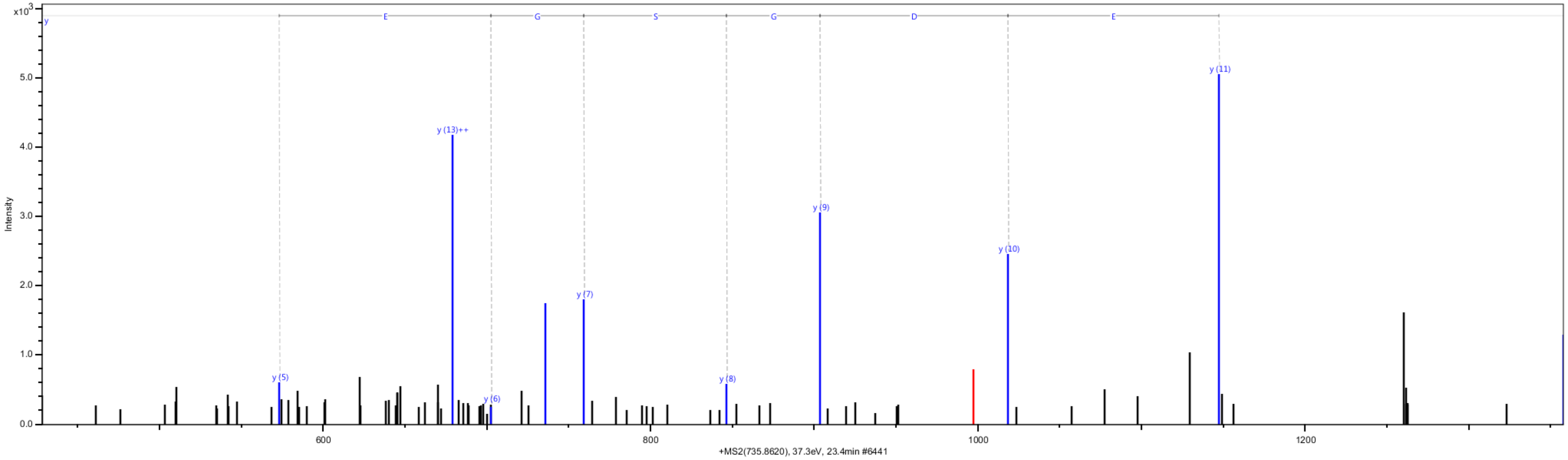

Peptide Match

**Sequence:**	R.IPIEDGSGEVVLSRK.V	**Score:**	29.1
**Modifications:**			

Spectrum Report
Combine Search_2020-10-19 07:53:31

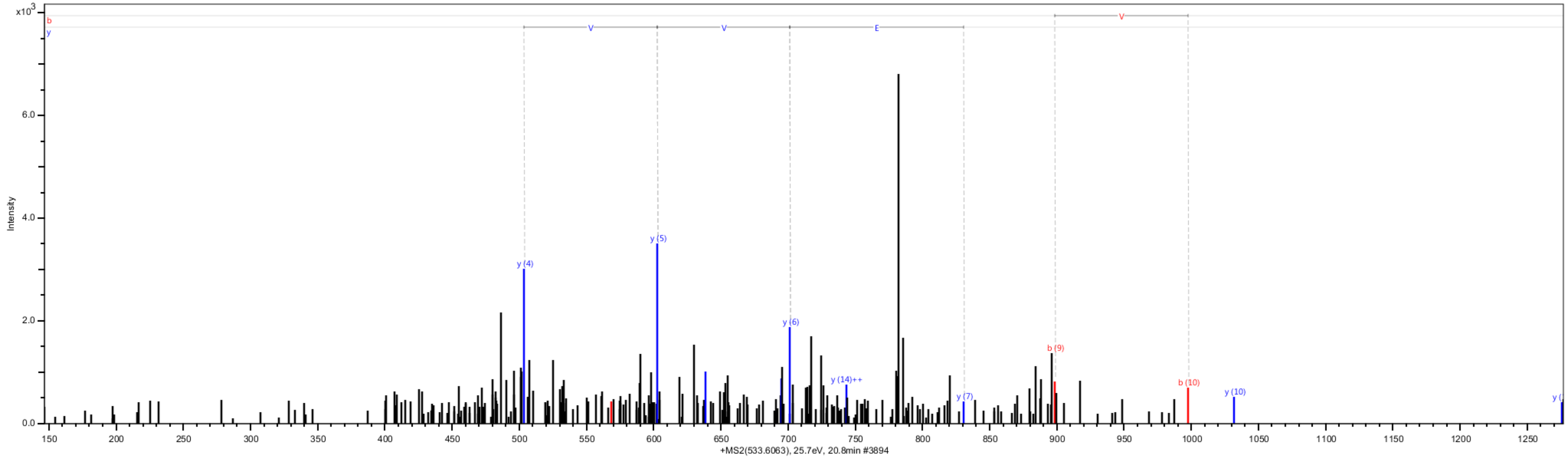

Peptide Match

**Sequence:**	R.KVLLDGVQNPR.A	**Score:**	46.4
**Modifications:**			

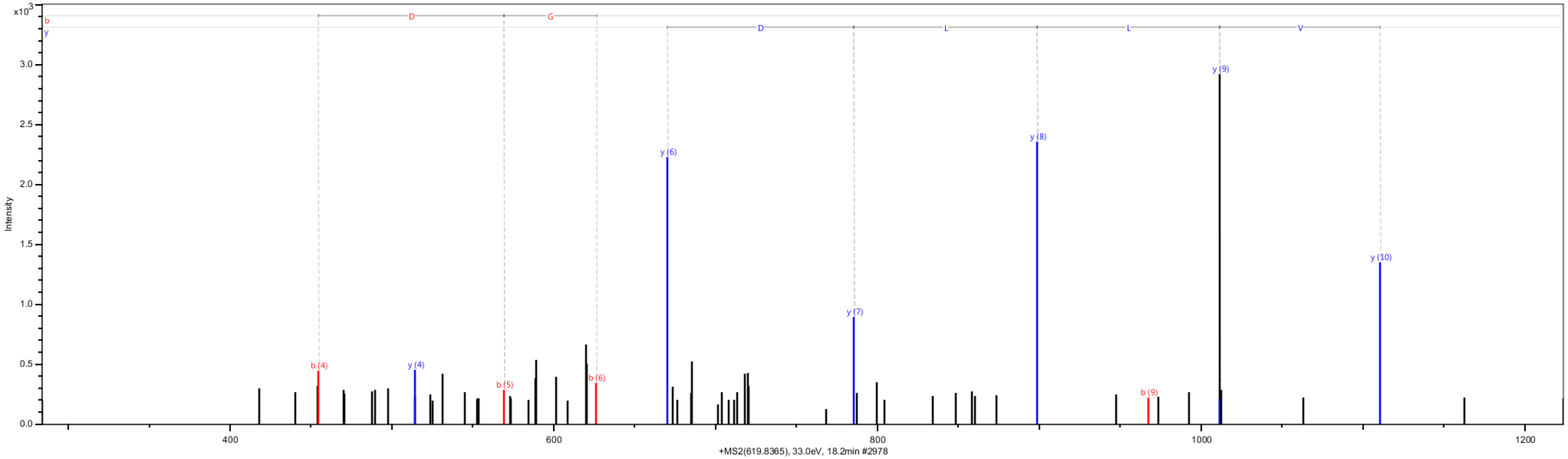

Peptide Match

**Sequence:**	K.VLLDGVQNPR.A	**Score:**	47.6
**Modifications:**			

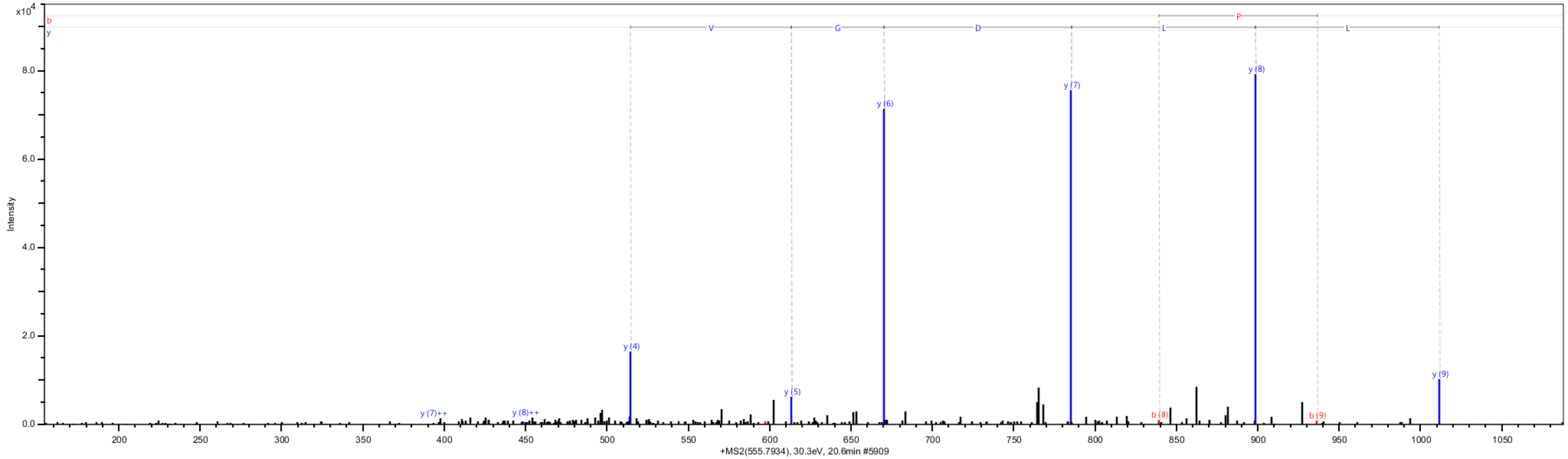

Peptide Match

Sequence: R.SGIPIVTSPYQIHFTK.T **Score:** 55.1

Modifications:

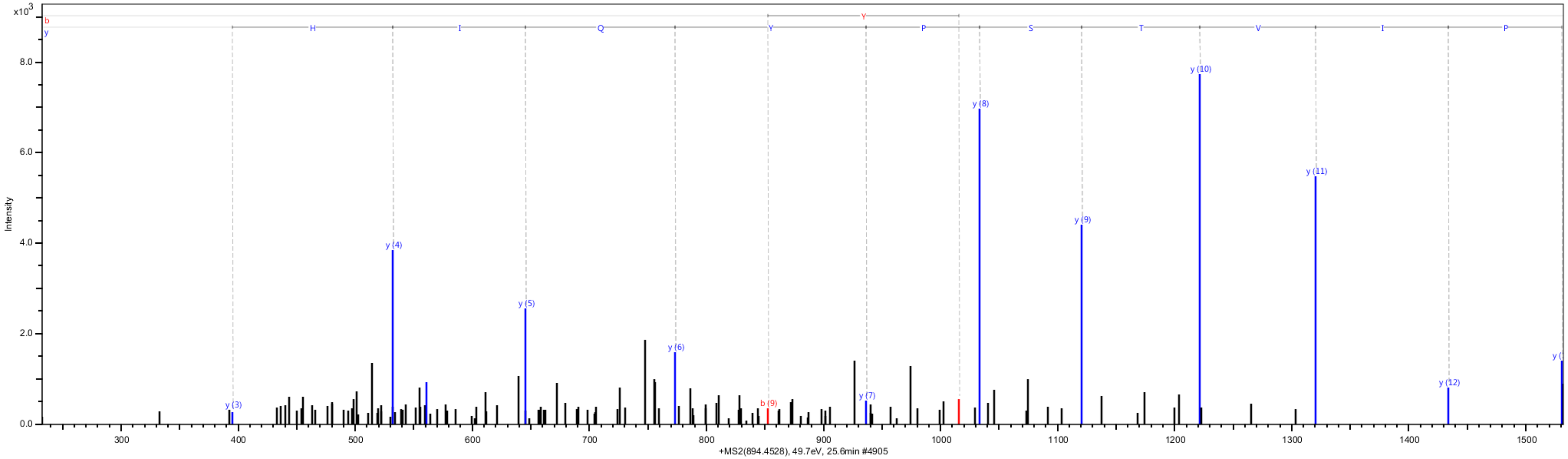

Peptide Match

Sequence: R.VPVAVQGEDTVQSLTQGDGVAK.L **Score:** 17.9

Modifications:

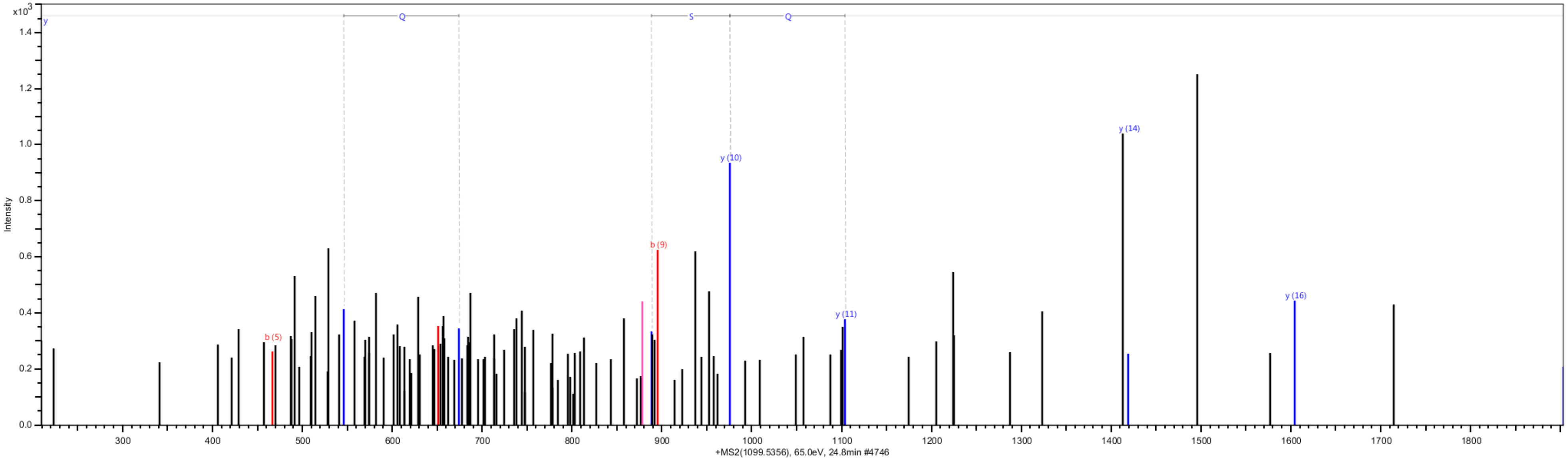

Peptide Match

Sequence: K.LSINTHPSQKPLSITVR.T **Score:** 81.2

Modifications:

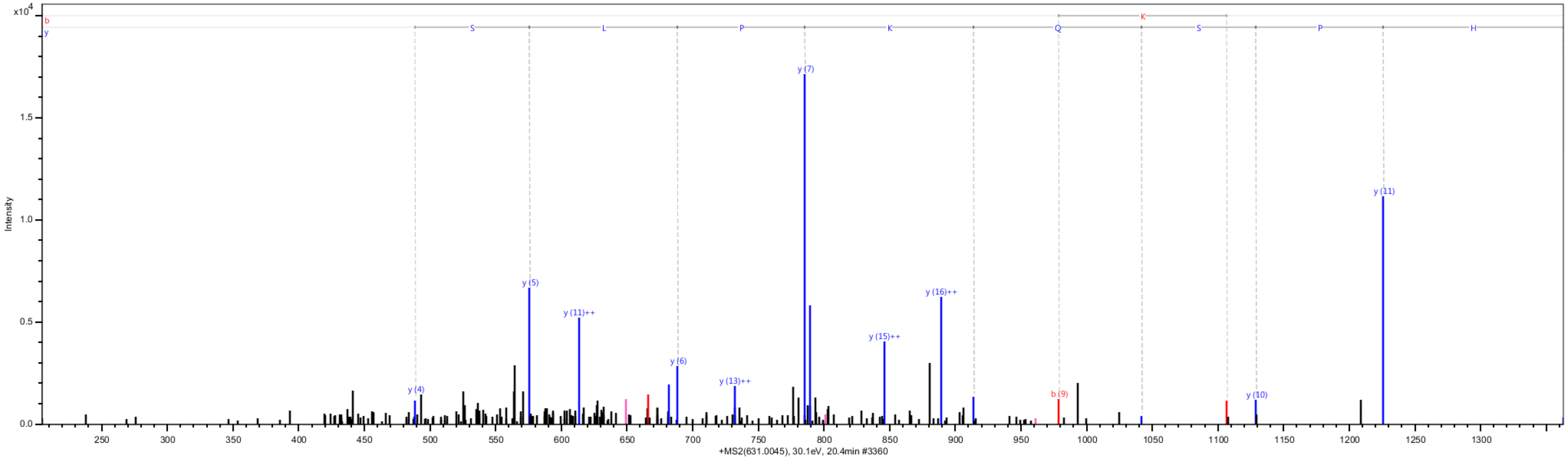

Peptide Match

Sequence: R.LVAYYTLIGASGQR.E **Score:** 77.8

Modifications:

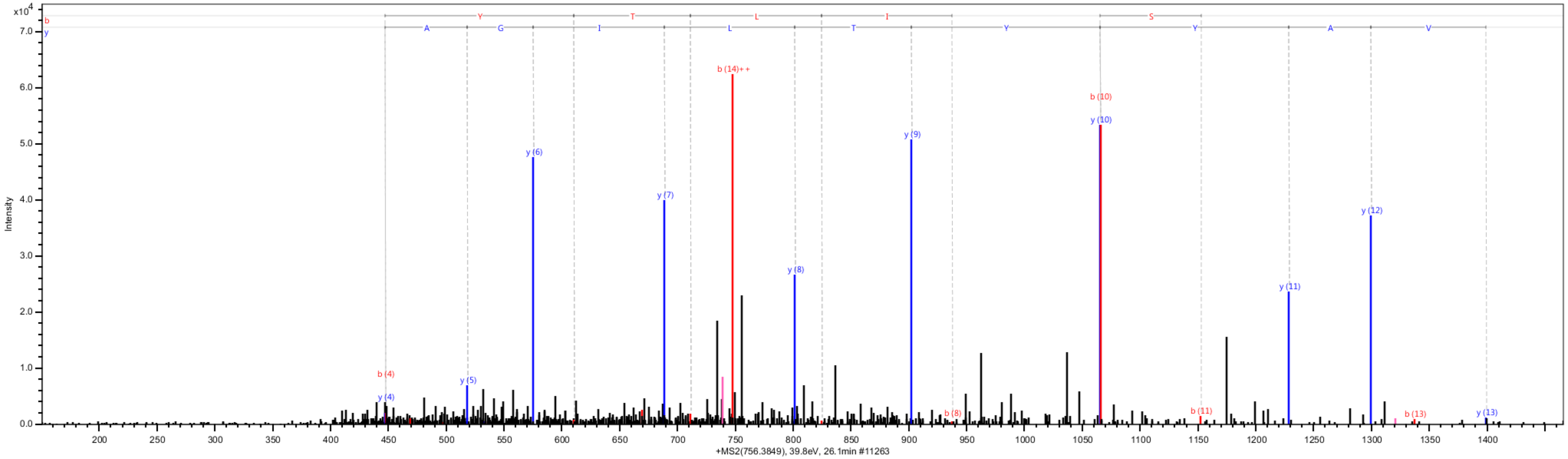

Peptide Match

**Sequence:**	R.EVVADSVWVDVK.D	**Score:**	36.9
**Modifications:**			

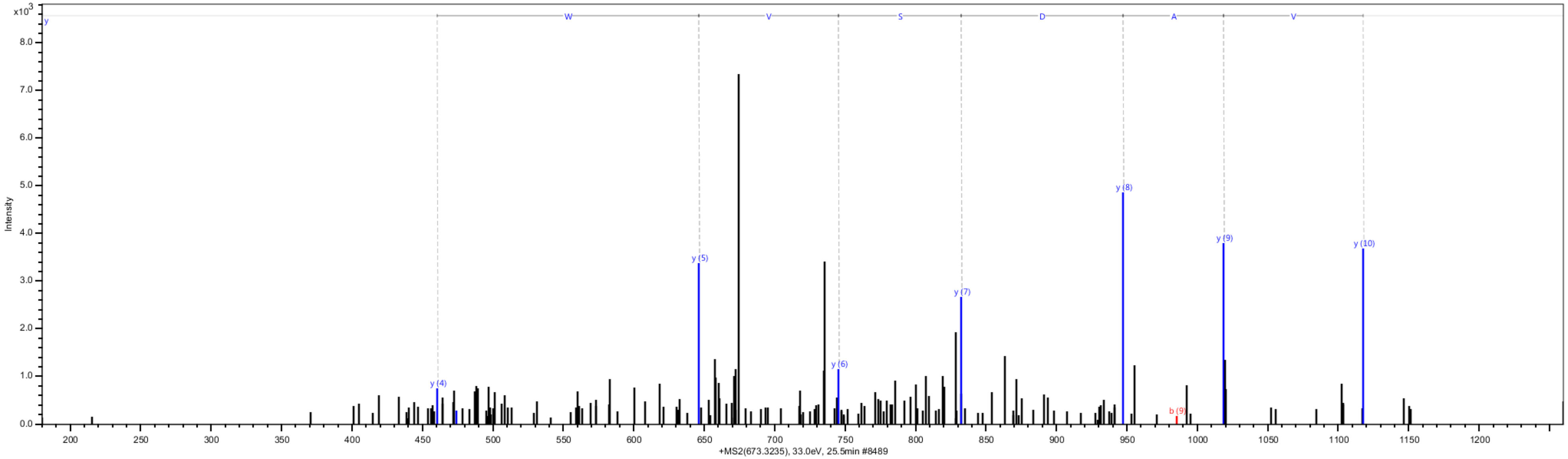

Peptide Match

Sequence: K.IWDVVEK.A **Score:** 18.5

Modifications:

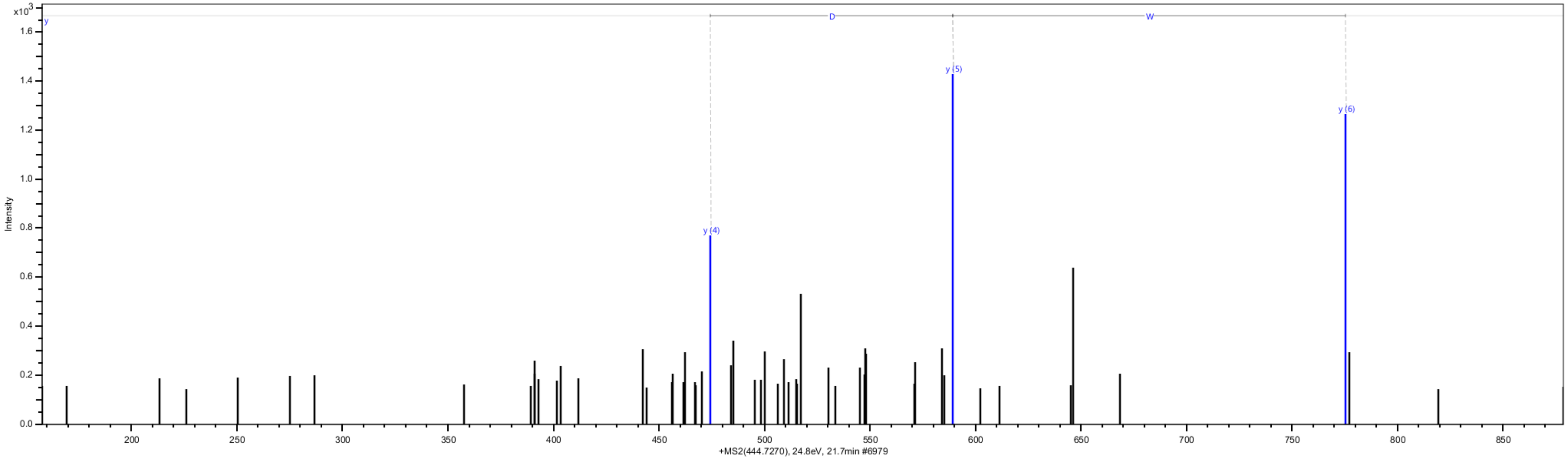

Peptide Match

Sequence: R.FISLGEACK.K **Score:** 15.8
Modifications: Carbamidomethyl: 8

Spectrum Report
Combine Search_2020-10-19 07:53:31

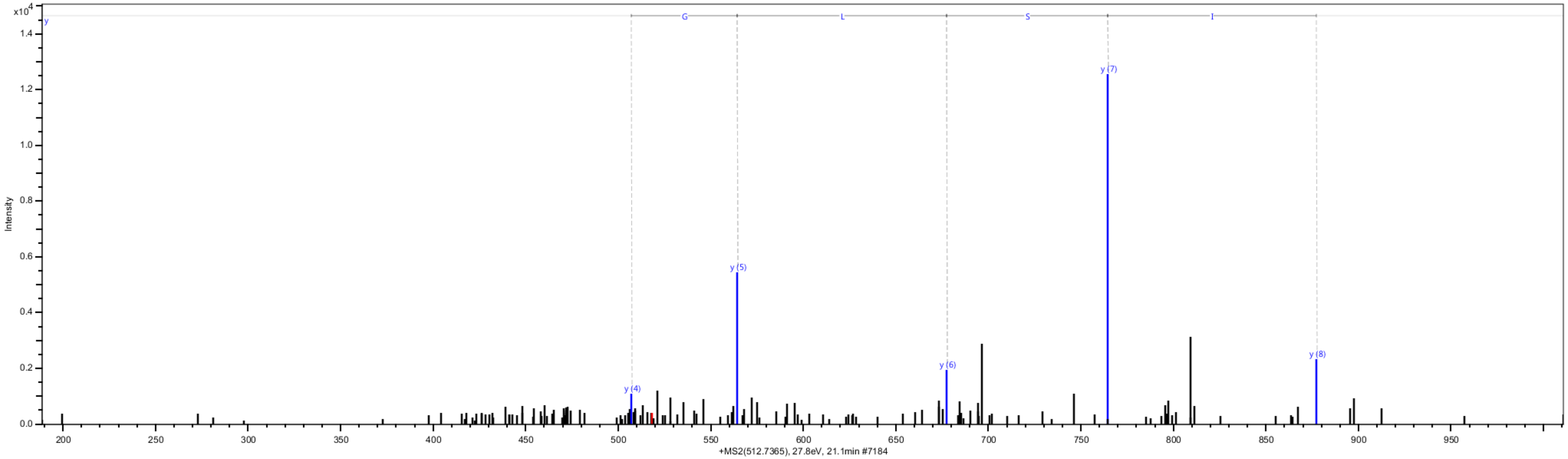

Peptide Match

**Sequence:**	R.FISLGEACKK.V	**Score:**	24
**Modifications:**	Carbamidomethyl: 8		

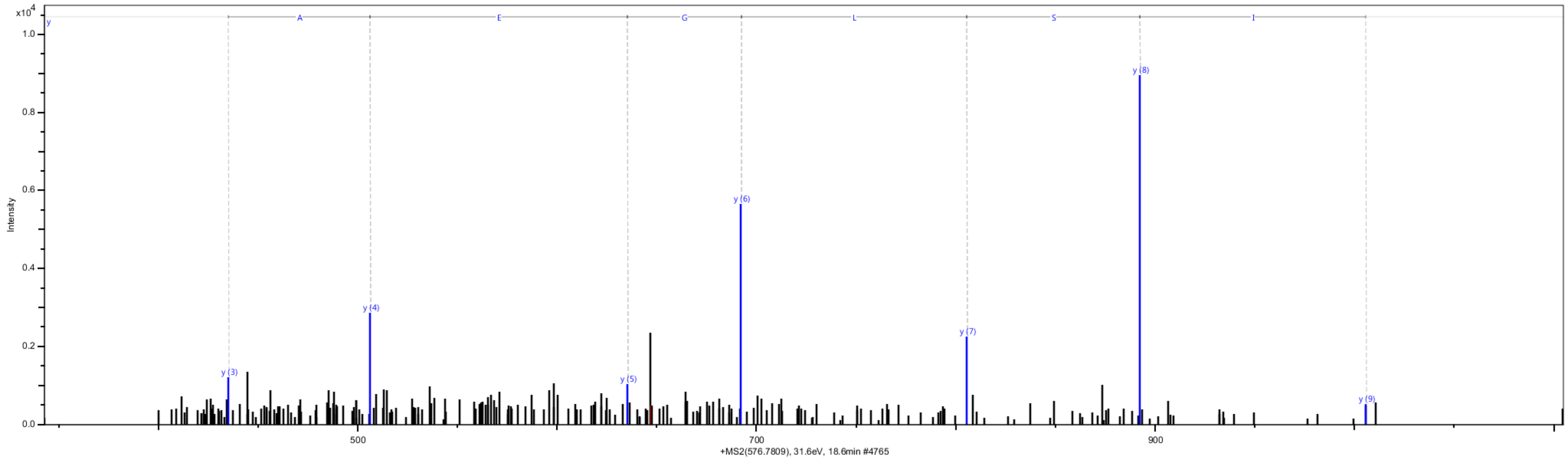

Peptide Match

**Sequence:**	K.KVFLDCCNYITELR.R	**Score:**	18.7
**Modifications:**	Carbamidomethyl: 6, 7		

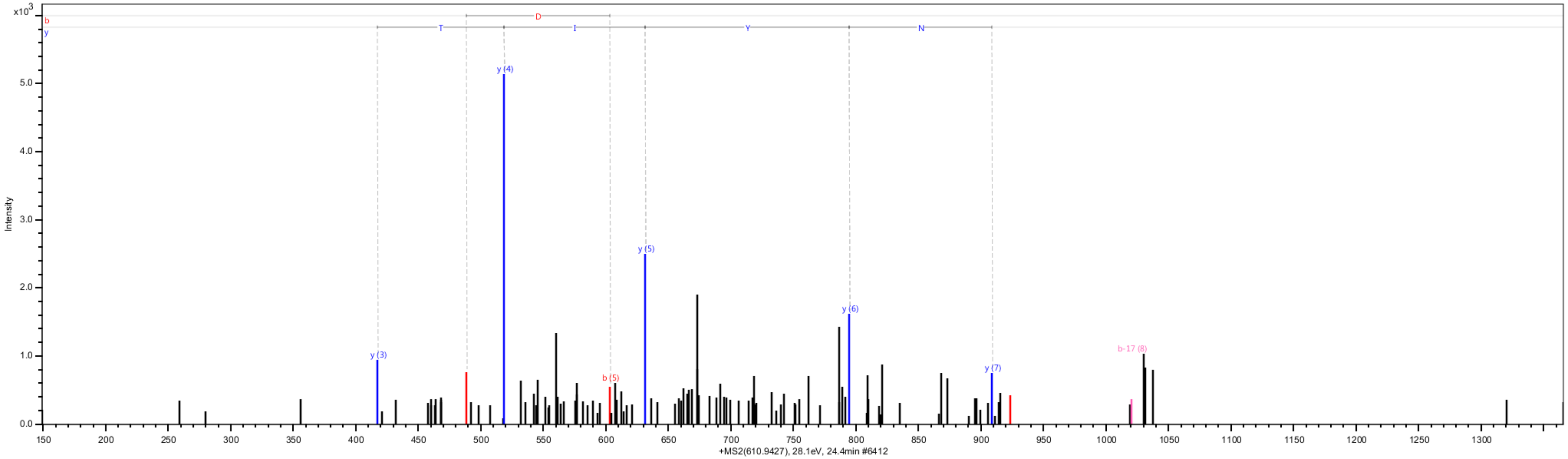

Peptide Match

**Sequence:**	K.VFLDCCNYITELR.R	**Score:**	51.2
**Modifications:**	Carbamidomethyl: 5, 6		

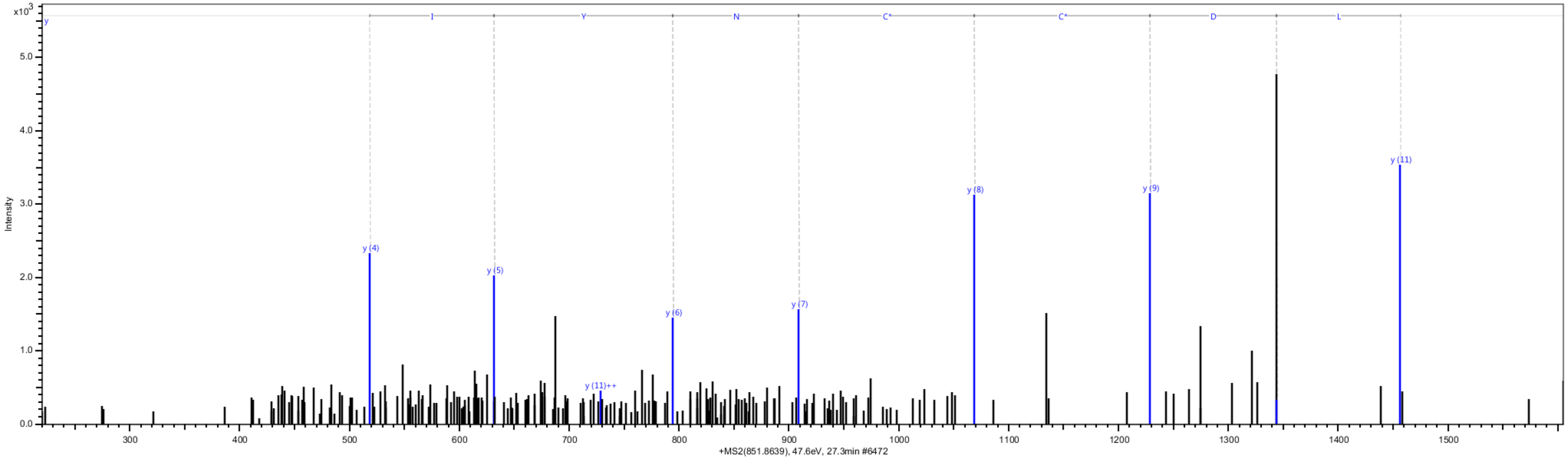

Peptide Match

**Sequence:**	K.VFLDCCNYITELRR.Q	**Score:**	48
**Modifications:**	Carbamidomethyl: 5, 6		

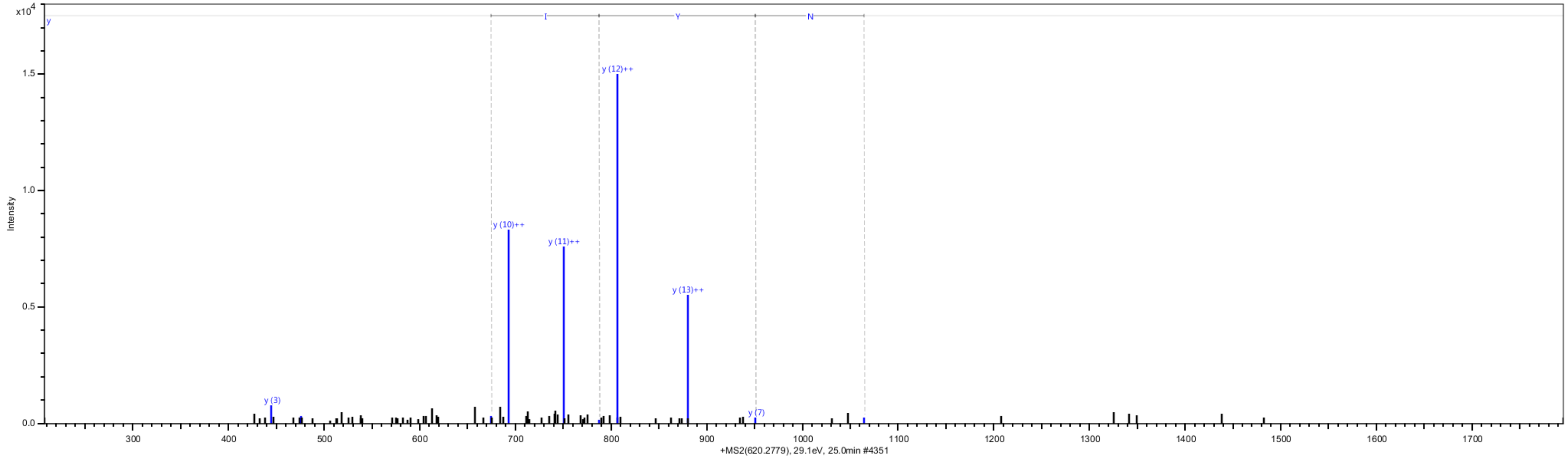

Peptide Match

**Sequence:**	R.SNLDEDIIAEENIVSR.S	**Score:**	50.7
**Modifications:**			

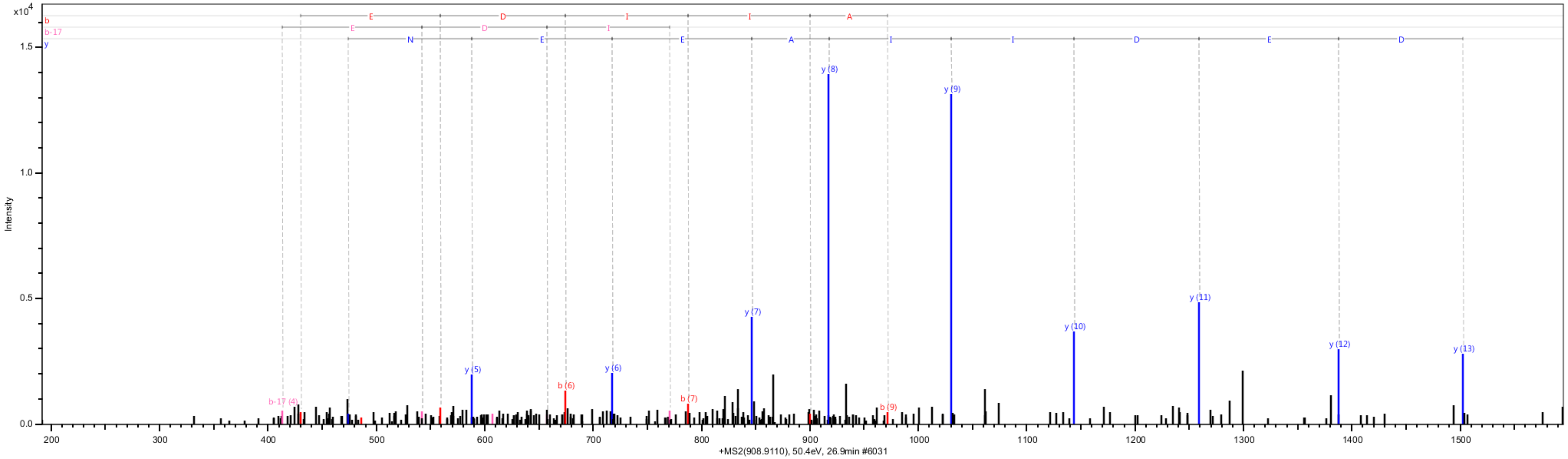

Peptide Match

**Sequence:**	R.LPYSVVR.N	**Score:**	21.7
**Modifications:**			

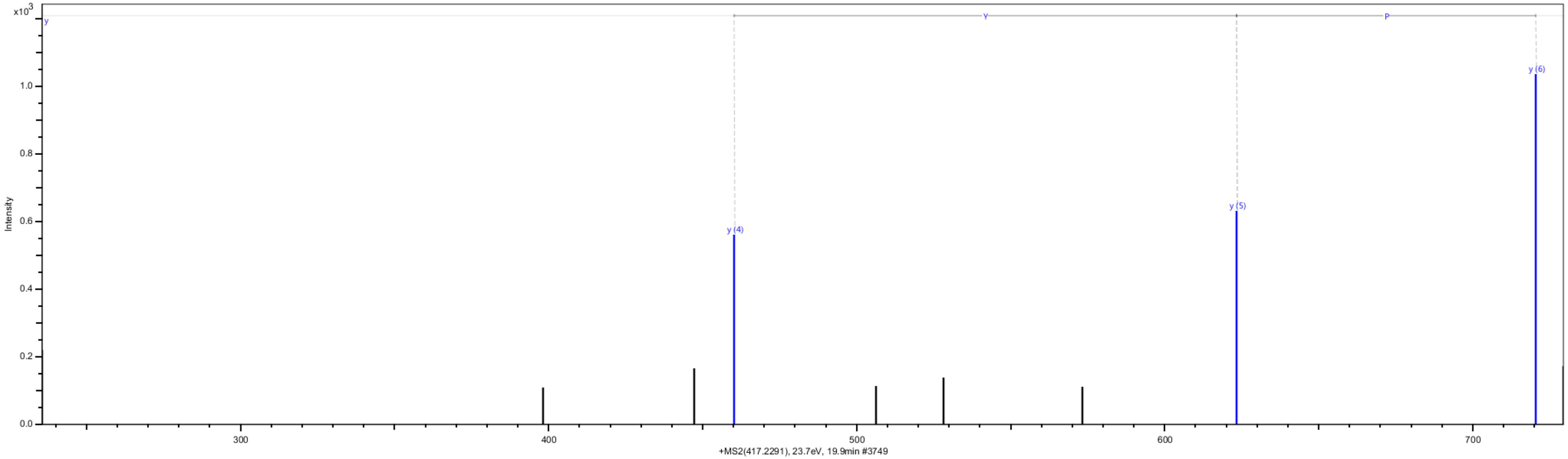

Peptide Match

Sequence: R.VELLHNPAFCSLATTK.R **Score:** 25.6

Modifications: Carbamidomethyl: 10

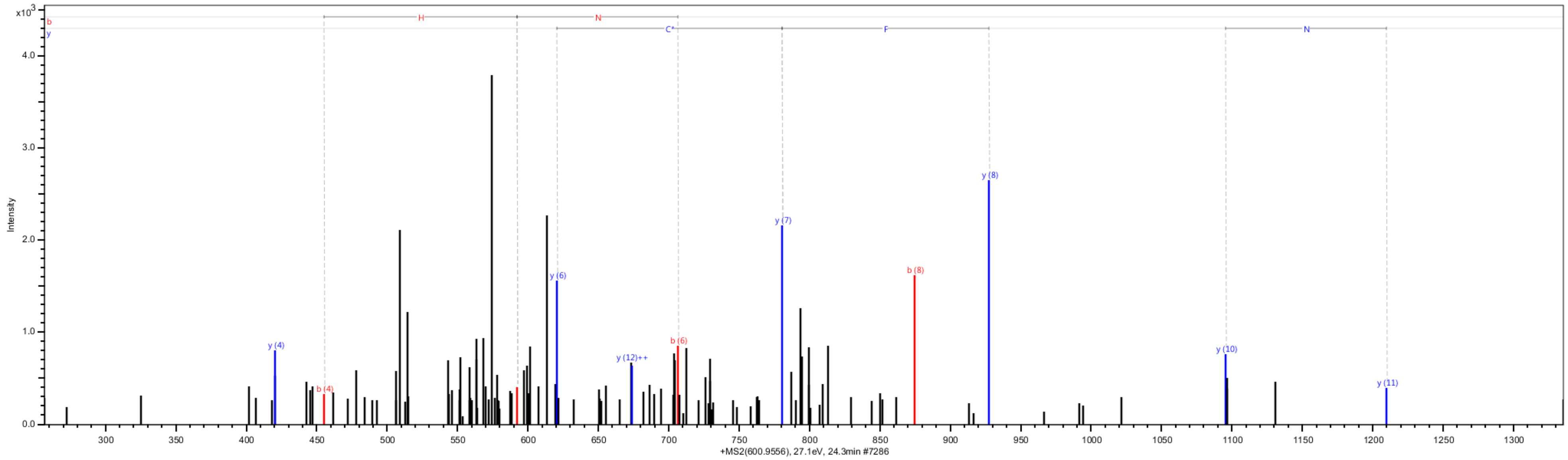

Peptide Match

Sequence: K.AAVYHHFISDGVR.K **Score:** 47.1

Modifications:

Spectrum Report
Combine Search_2020-10-19 07:53:31

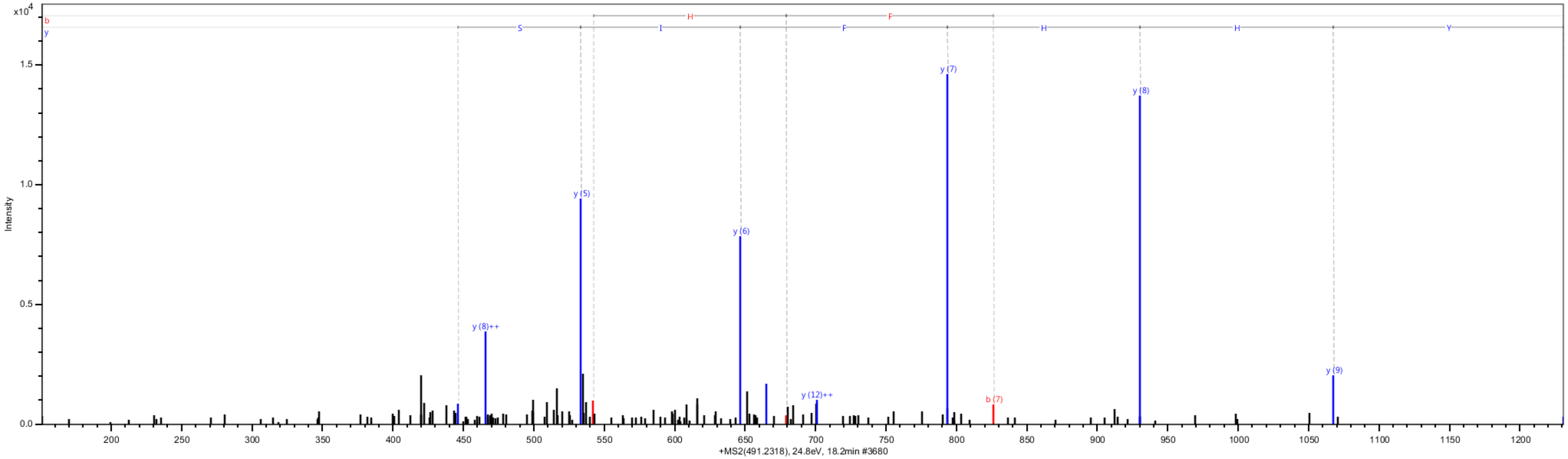

Peptide Match

Sequence: R.QGALELIK.K **Score:** 18

Modifications:

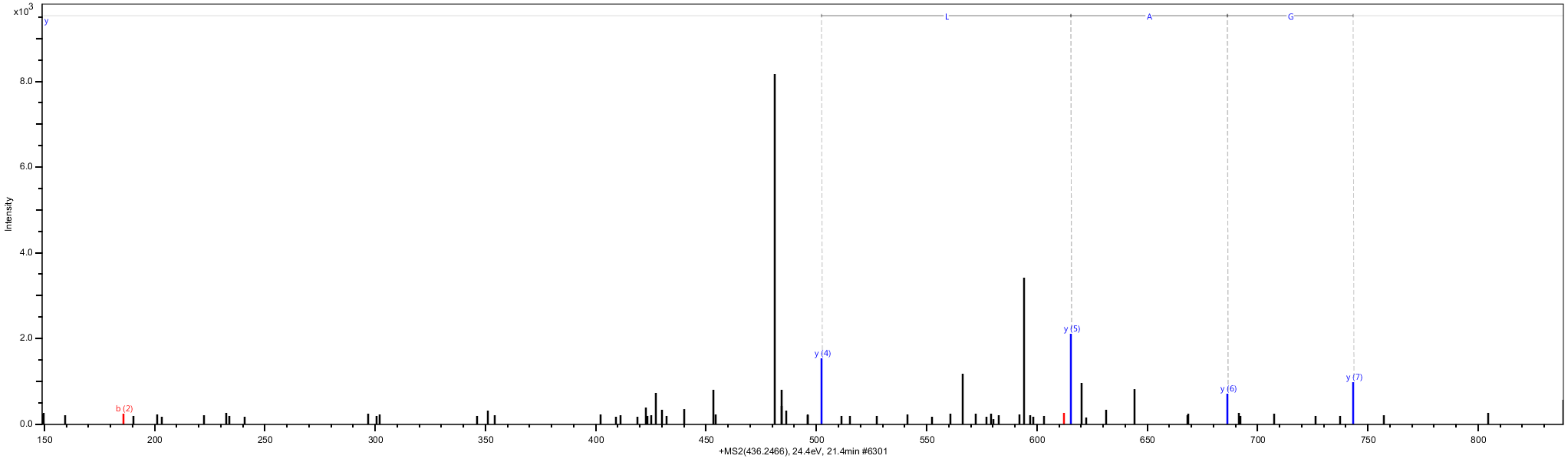

Peptide Match

**Sequence:**	K.KGYTQQLAFR.Q	**Score:**	31.4
**Modifications:**			

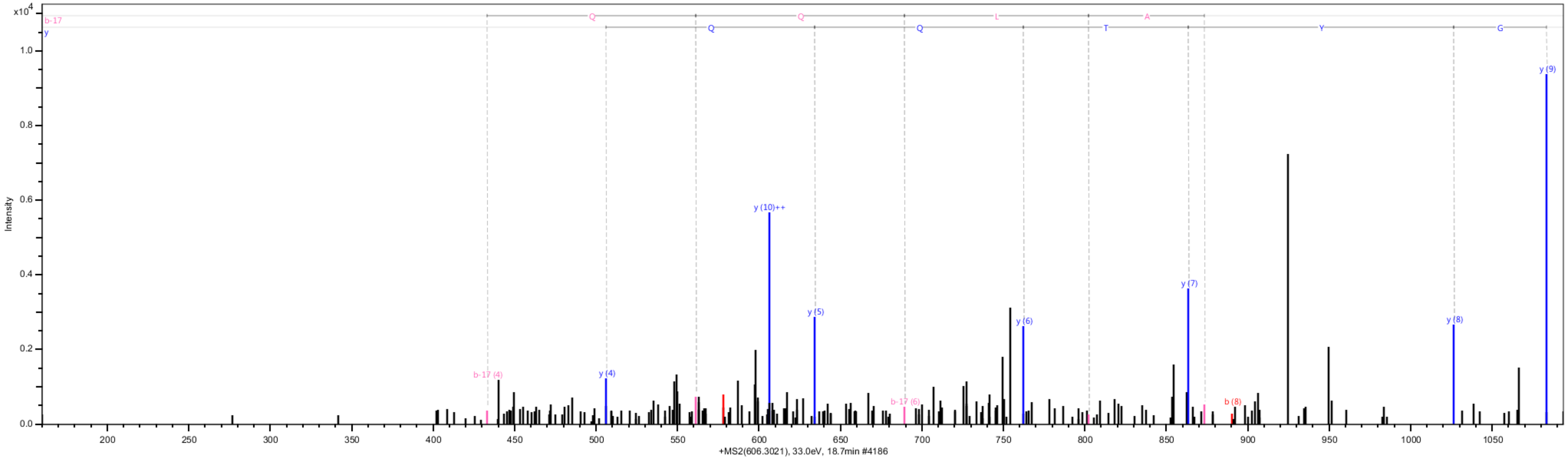

Peptide Match

Sequence: K.GYTQQLAFR.Q **Score:** 25.9

Modifications:

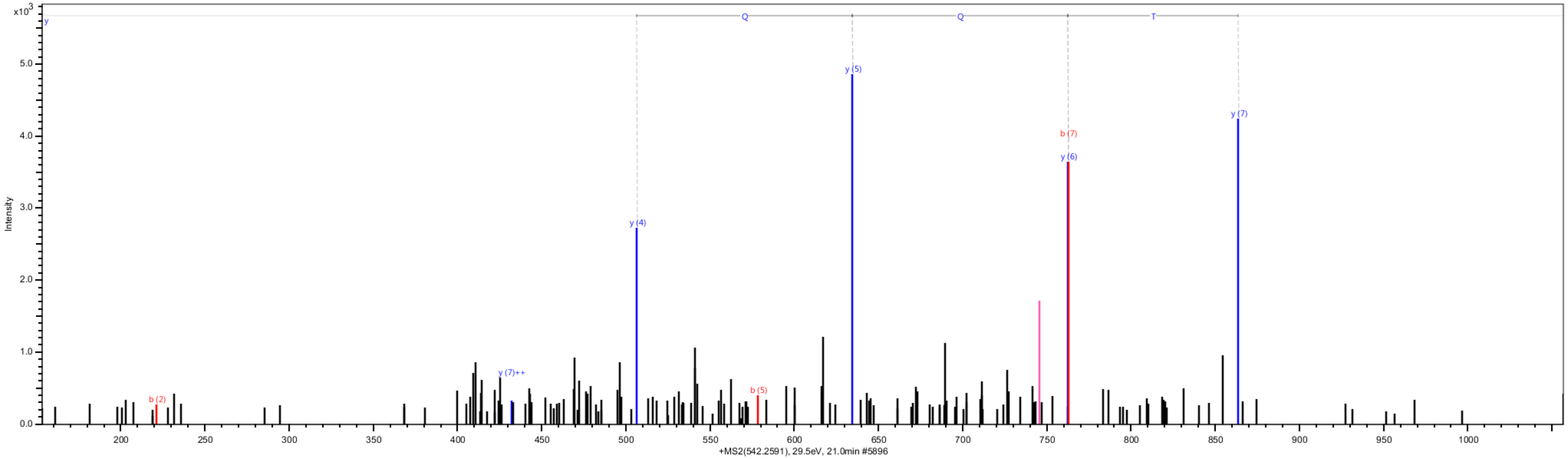

Peptide Match

**Sequence:**	R.QPSSAFAAFVKR.A	**Score:**	24.4
**Modifications:**			

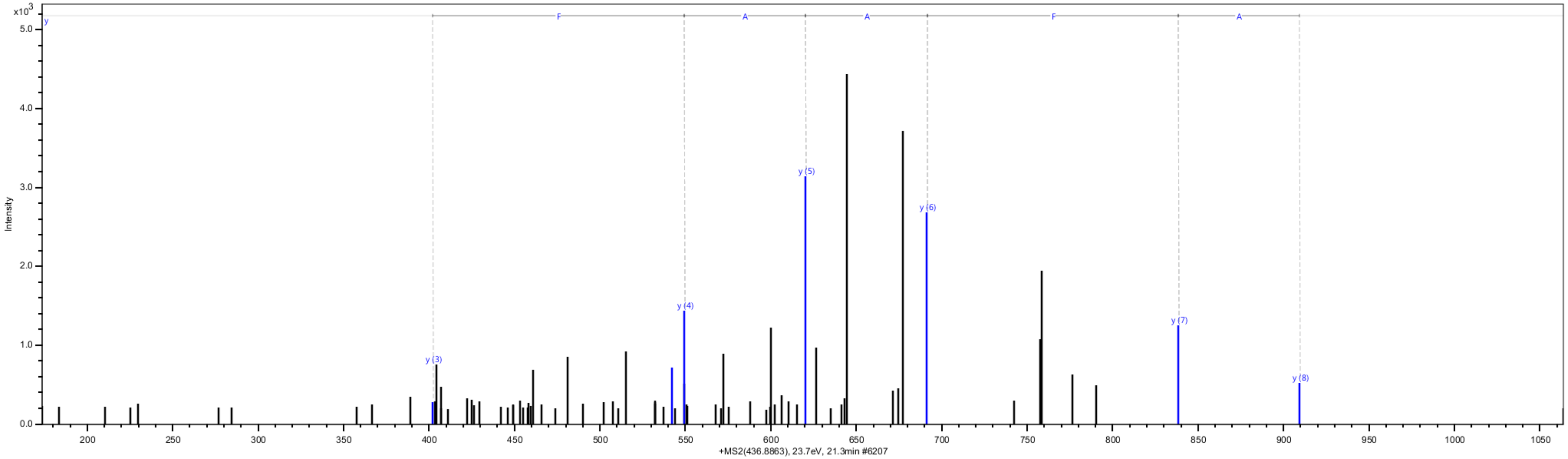

Peptide Match

Sequence: K.AGDFLEANYMNLQR.S **Score:** 19.8

Modifications:

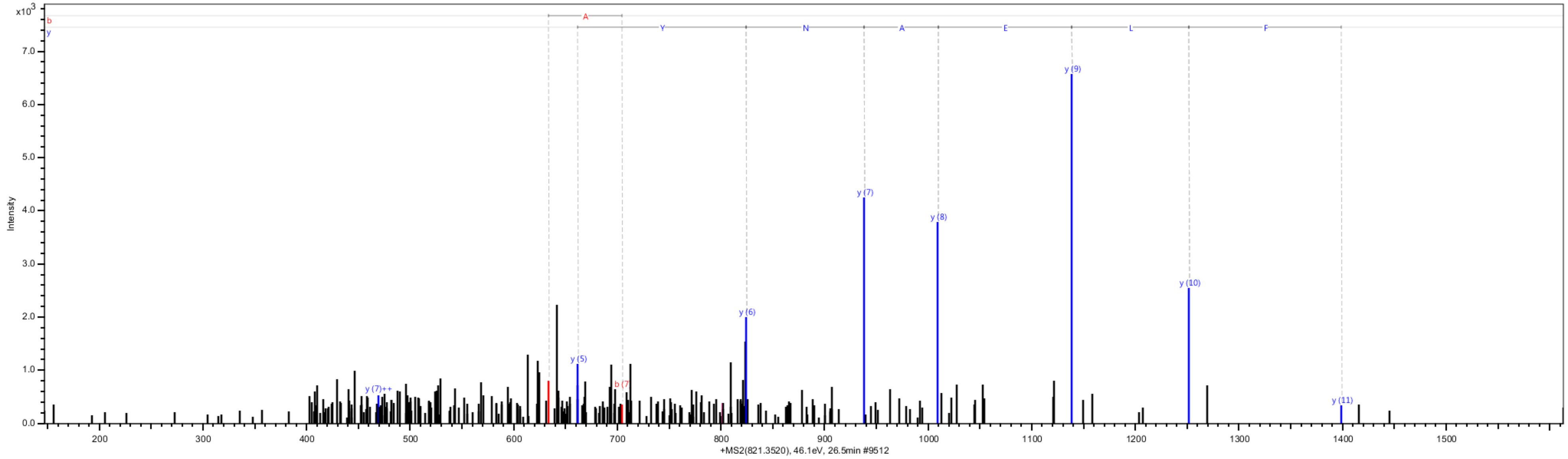

Peptide Match

Sequence: K.DFDFVPPVVR.W **Score:** 36.6

Modifications:

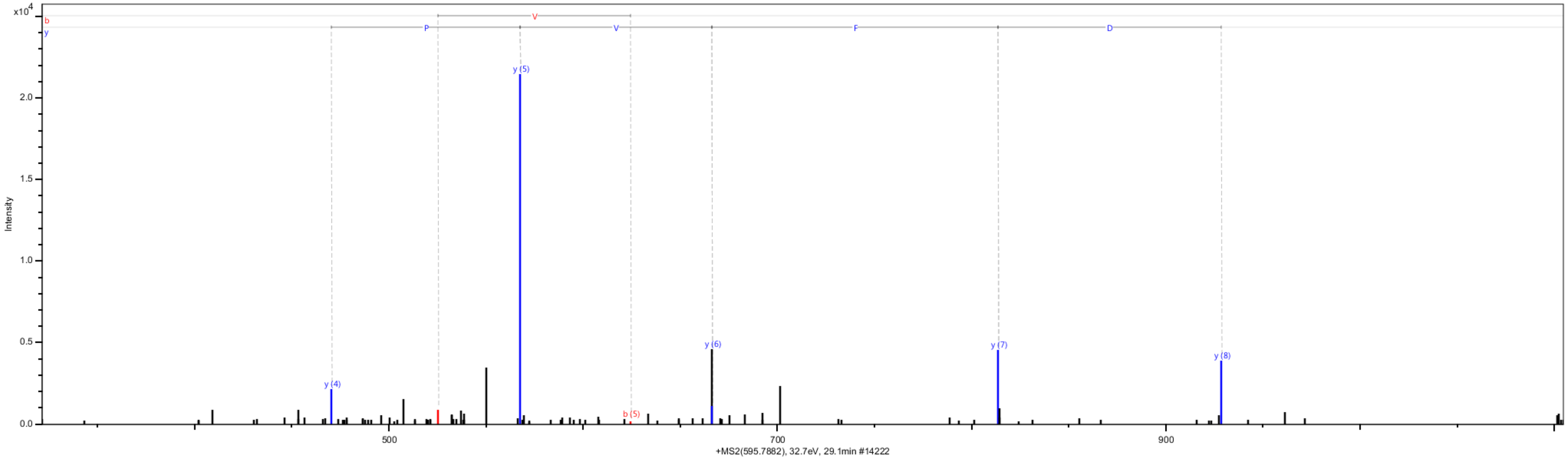

Peptide Match

**Sequence:**	R.IHWESASLLR.S	**Score:**	17.1
**Modifications:**			

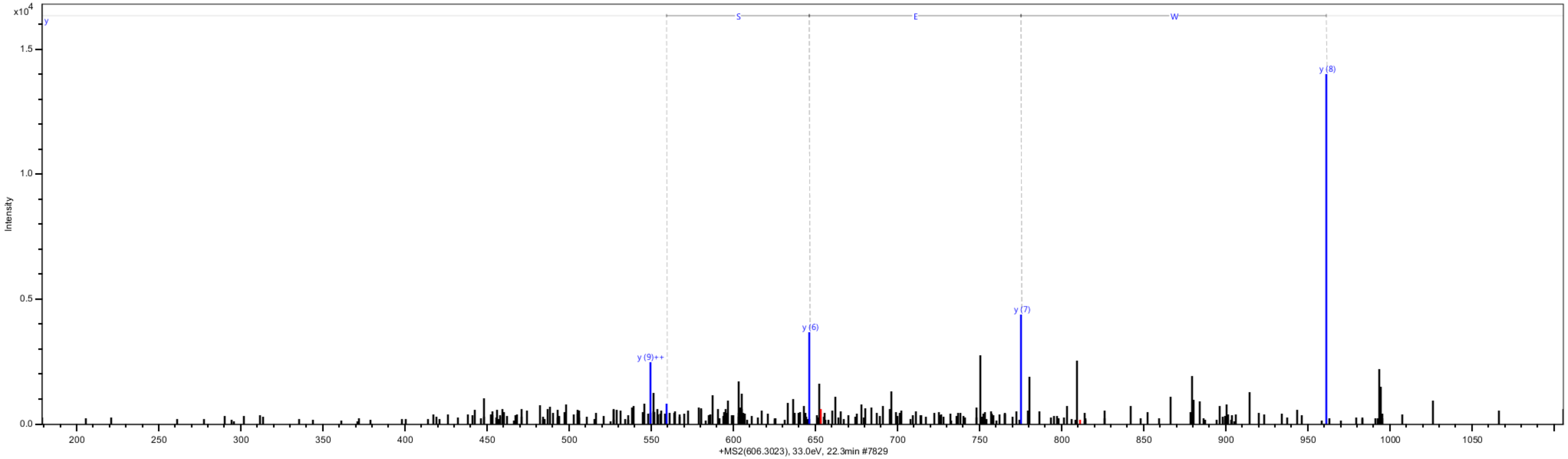

Peptide Match

Sequence: K.NTMILEICTR.Y **Score:** 35

Modifications: Carbamidomethyl: 8

Spectrum Report
Combine Search_2020-10-19 07:53:31

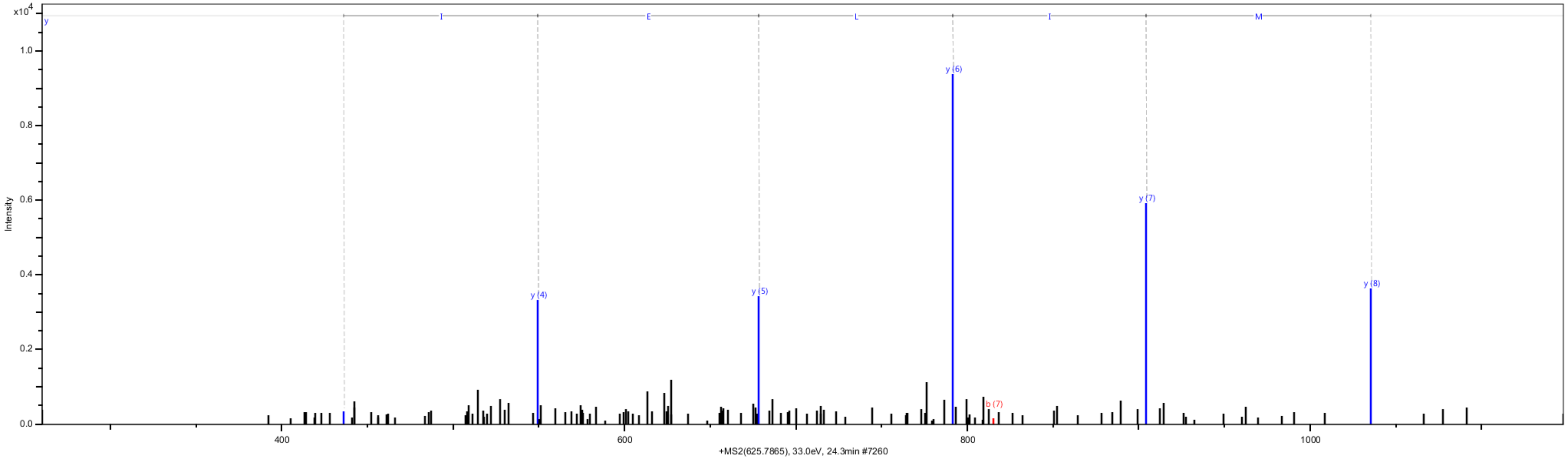

Peptide Match

Sequence: R.NTLIIYLDK.V **Score:** 40.7

Modifications:

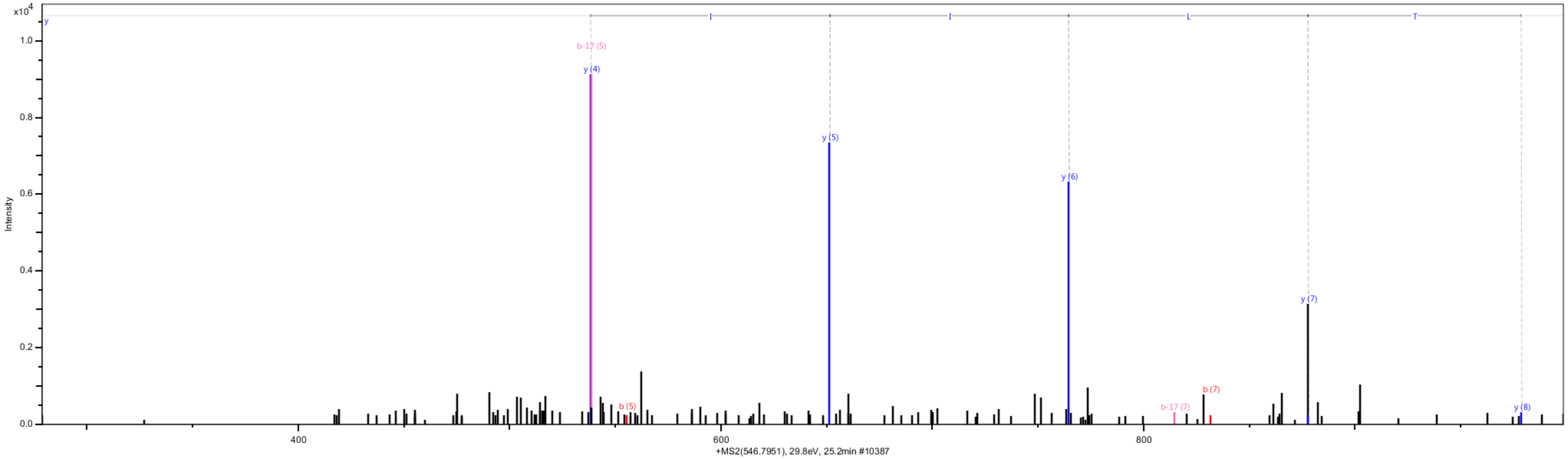

Peptide Match

**Sequence:**	K.VSHSEDDCLAFK.V	**Score:**	16.1
**Modifications:**	Carbamidomethyl: 8		

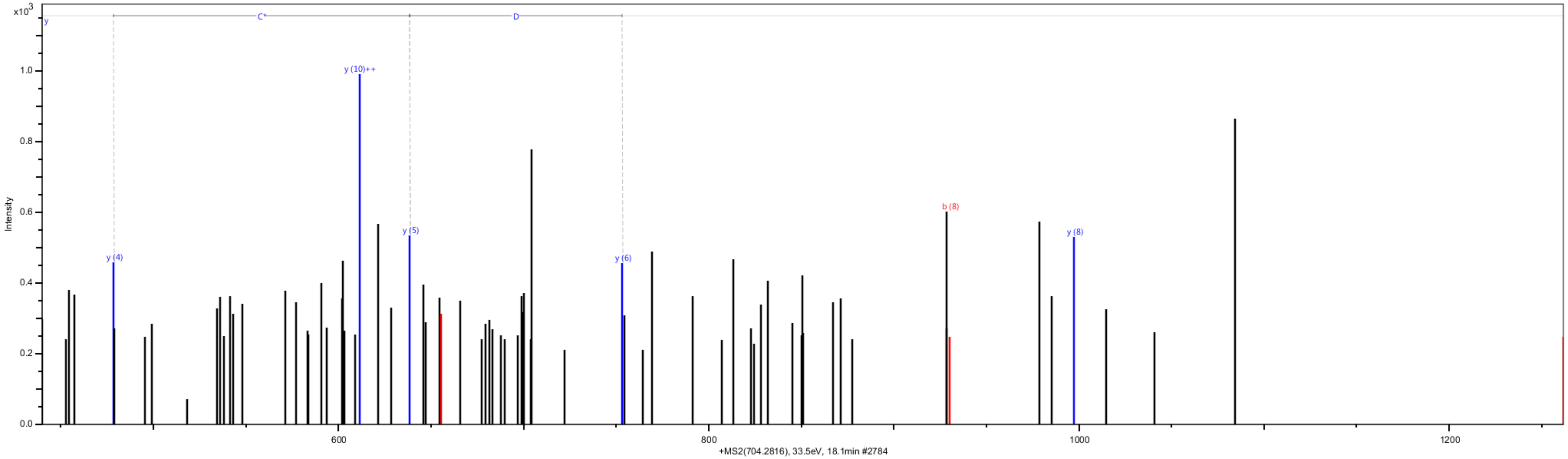

Peptide Match

Sequence: K.VYAYYNLEESCTR.F **Score:** 70.5

Modifications: Carbamidomethyl: 11

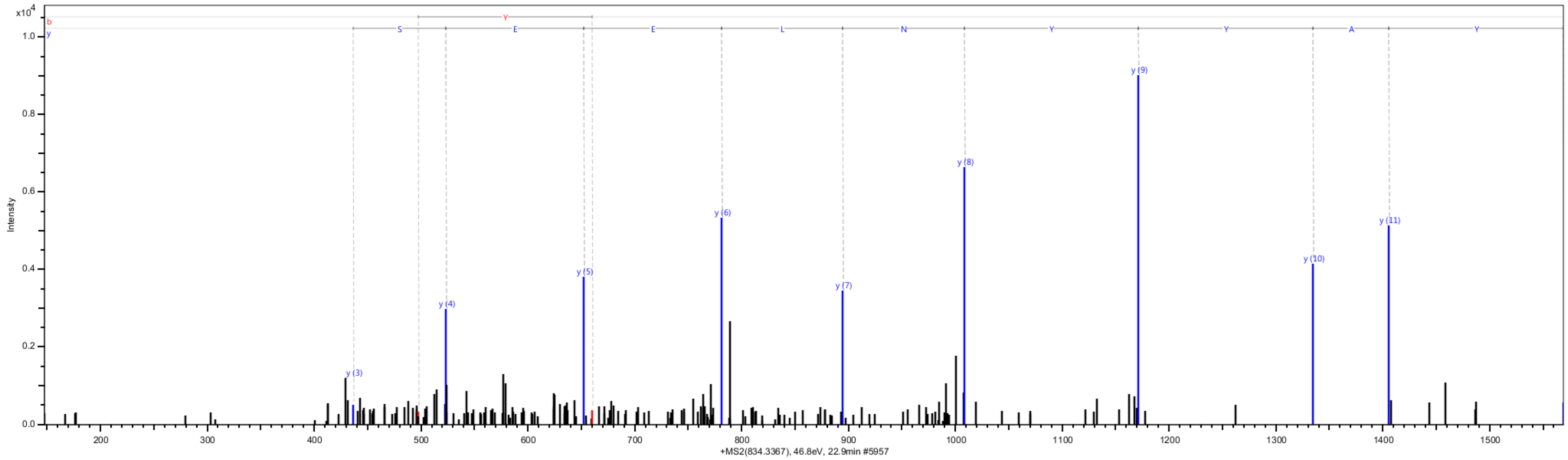

Peptide Match

**Sequence:**	R.LDKACEPGVDYVYK.T	**Score:**	19.1
**Modifications:**	Carbamidomethyl: 5		

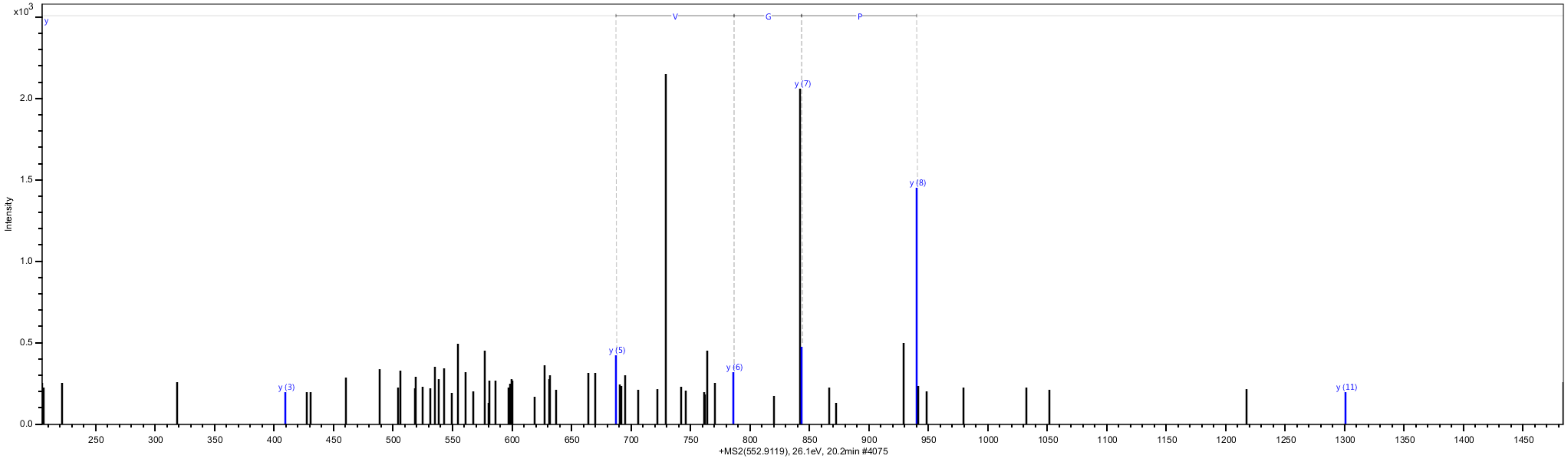

Peptide Match

Sequence: K.ACEPGVDYVYK.T **Score:** 17.2
Modifications: Carbamidomethyl: 2

Spectrum Report
Combine Search_2020-10-19 07:53:31

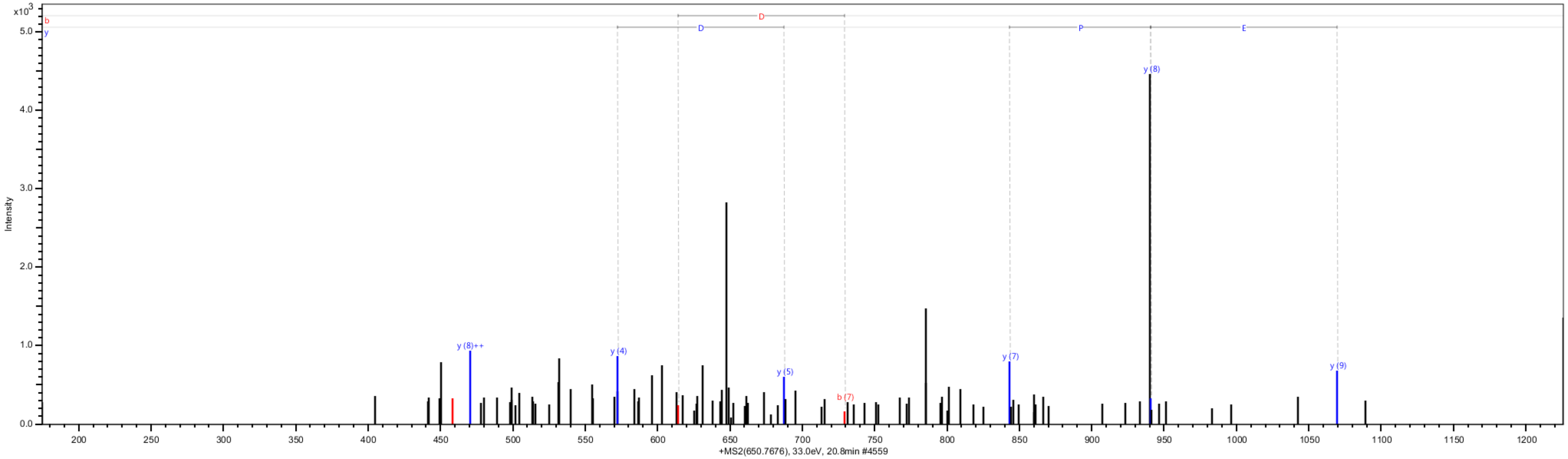

Peptide Match

**Sequence:**	R.TFISPIK.C	**Score:**	24.6
**Modifications:**			

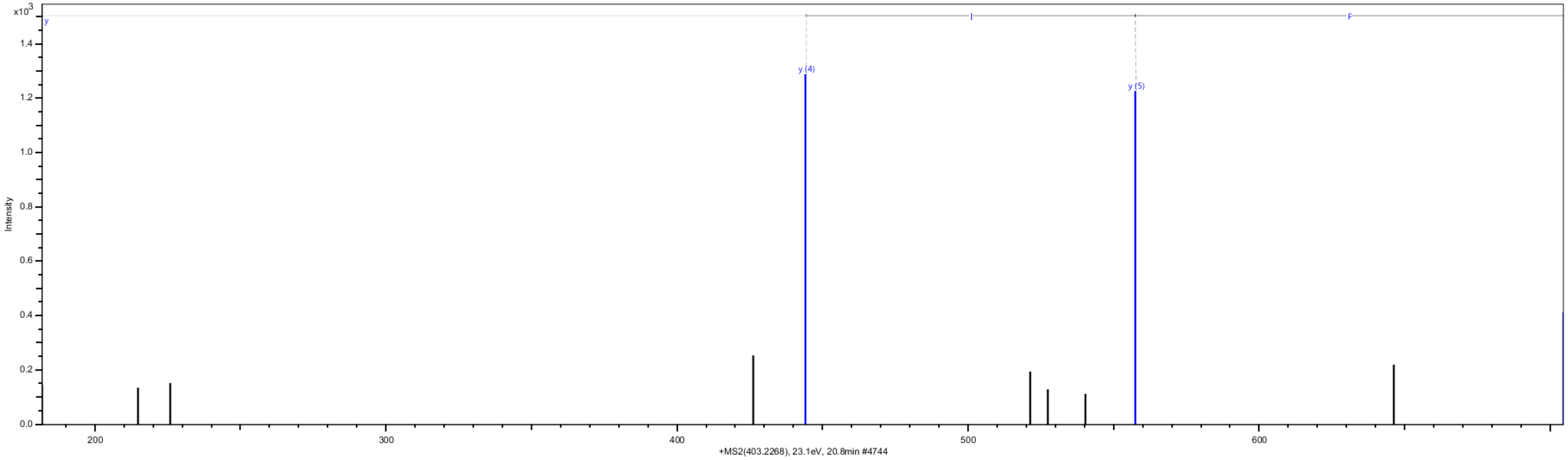
x10^3
y
I
F
y (4)
y (5)
1.4
1.2
1.0
0.8
0.6
0.4
0.2
0.0
Intensity
200
300
400
500
600
+MS2(403.2268), 23.1eV, 20.8min #4744

ALBU_HUMAN

Accession: ALBU_HUMAN **Score:** 3565.8
Database: SwissProt **Seq. Coverage [%]:** 73.9
MW [kDa] / pI: 69.3 / 5.9 **No. of Peptides:** 62

Peptide Match

Sequence: R.FKDLGEENFK.A **Score:** 74.7
Modifications:

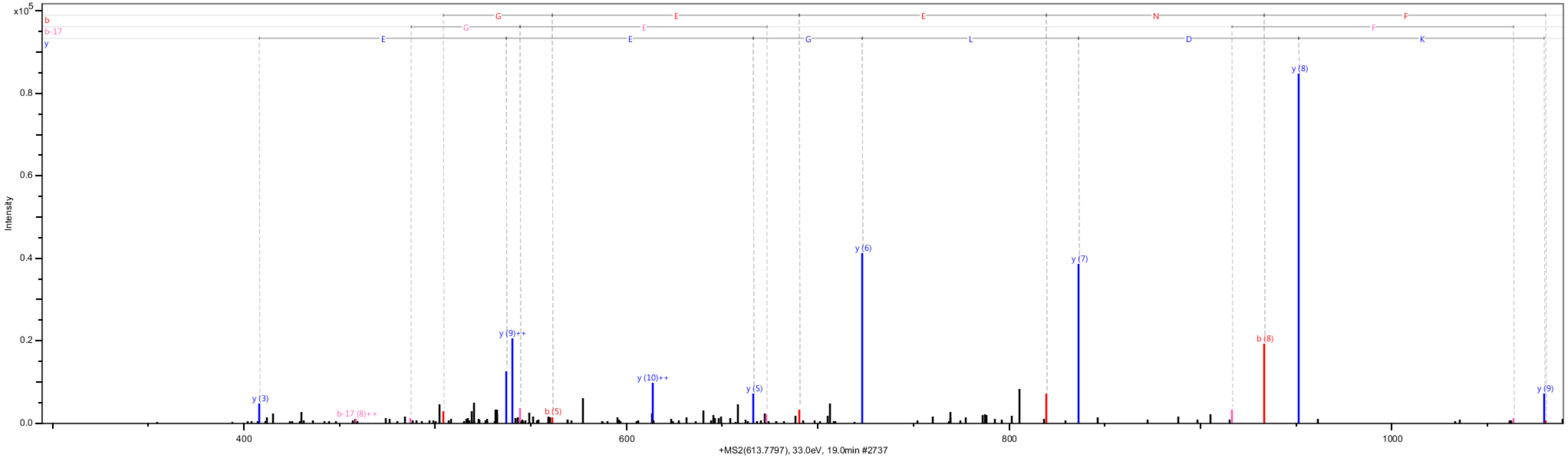

Peptide Match

Sequence: K.DLGEENFK.A **Score:** 21
Modifications:

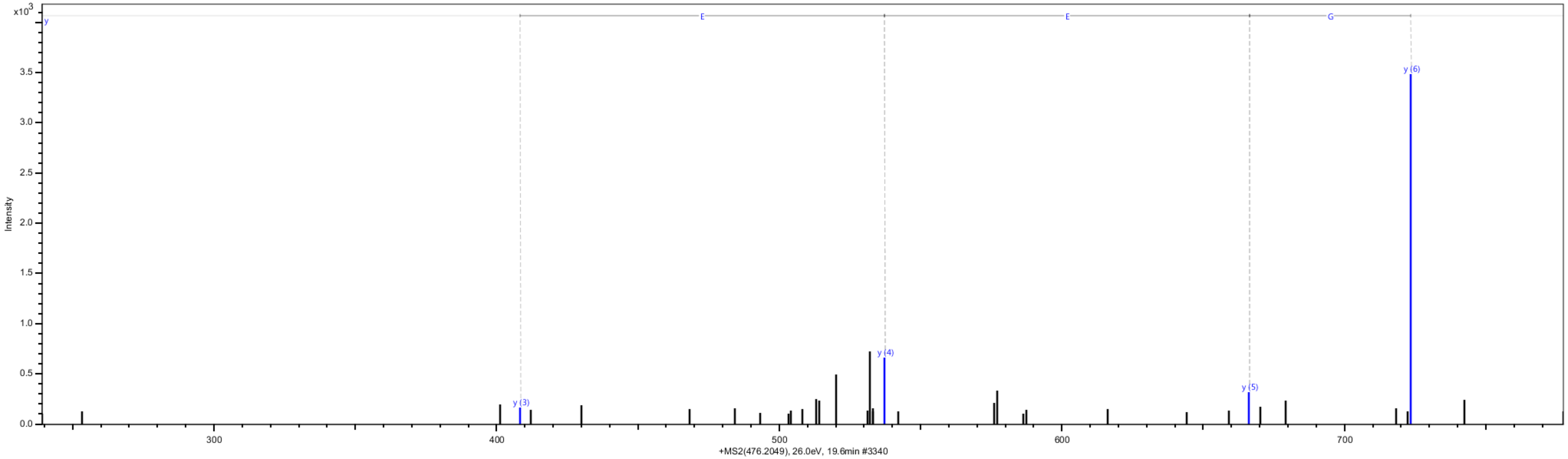

Peptide Match

**Sequence:**	K.LVNEVTEFAK.T	**Score:**	62.4
**Modifications:**			

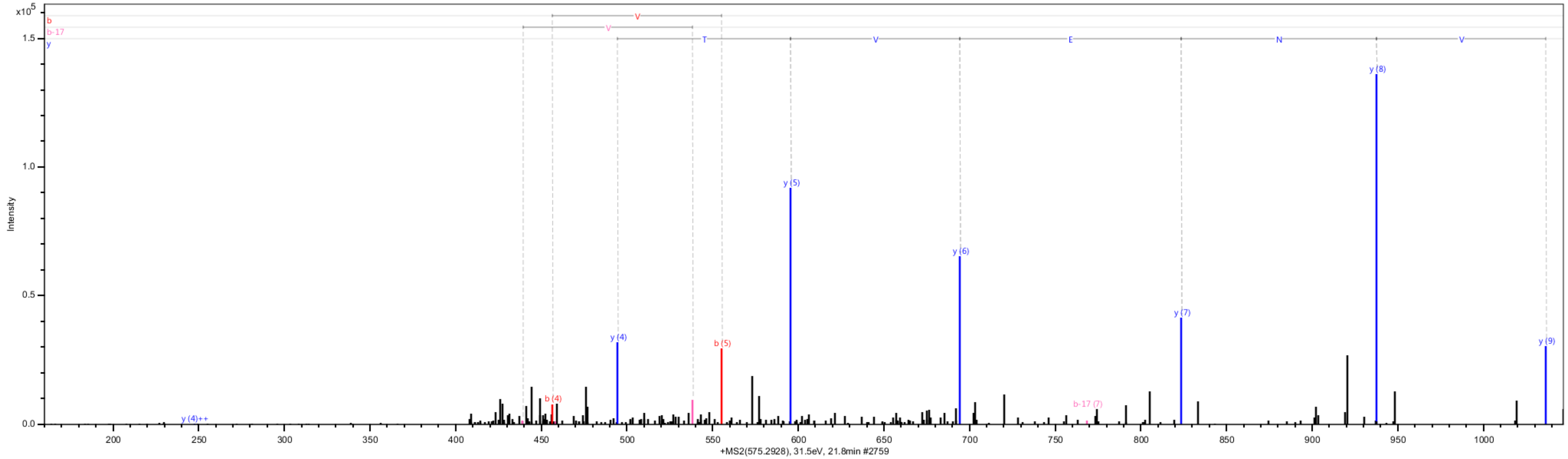

Peptide Match

**Sequence:**	K.TCVADESAENCDK.S	**Score:**	92.3
**Modifications:**	Carbamidomethyl: 2, 11		

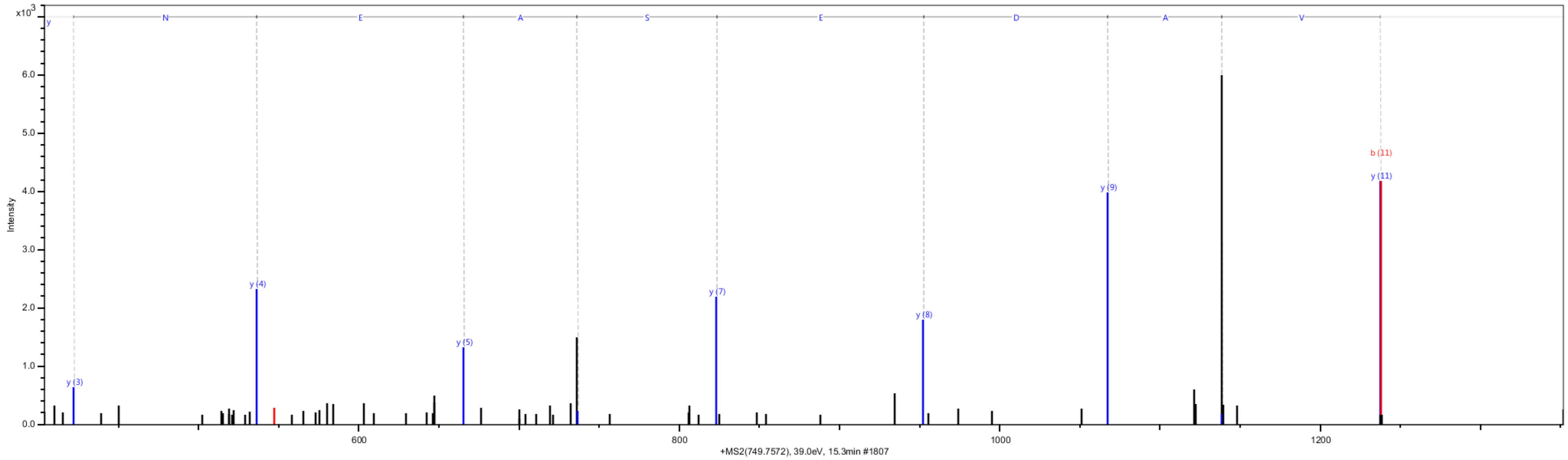

Peptide Match

Sequence: K.SLHTLFGDK.L **Score:** 40.2

Modifications:

Spectrum Report
Combine Search_2020-10-19 07:53:31

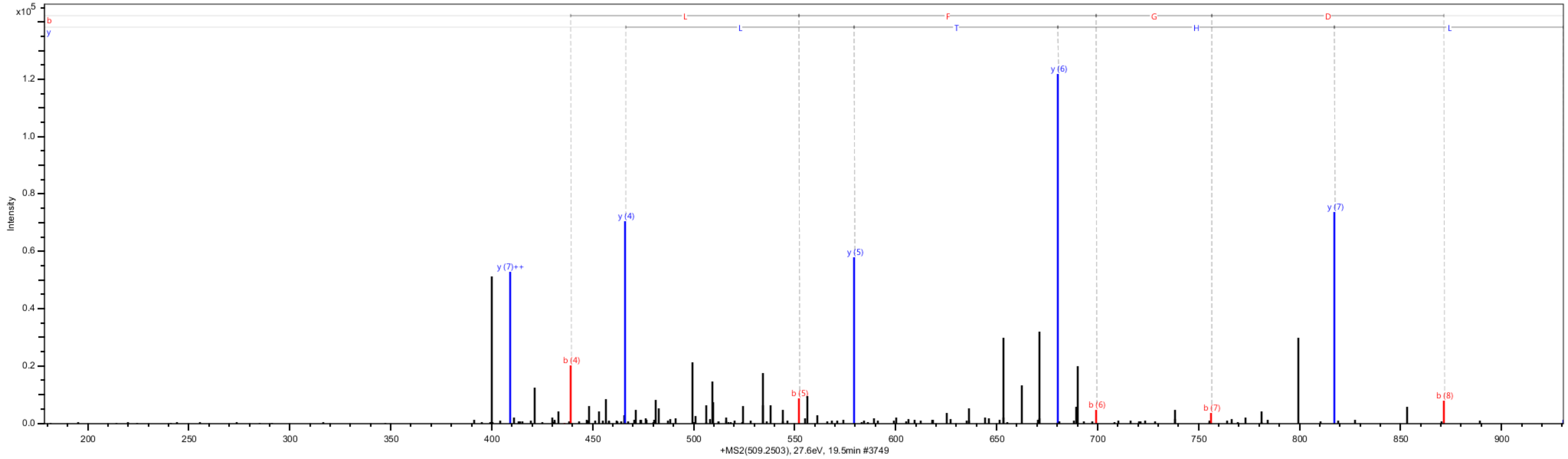

Peptide Match

Sequence: K.SLHTLFGDKLCTVATLR.E **Score:** 110
Modifications: Carbamidomethyl: 11

Spectrum Report
Combine Search_2020-10-19 07:53:31

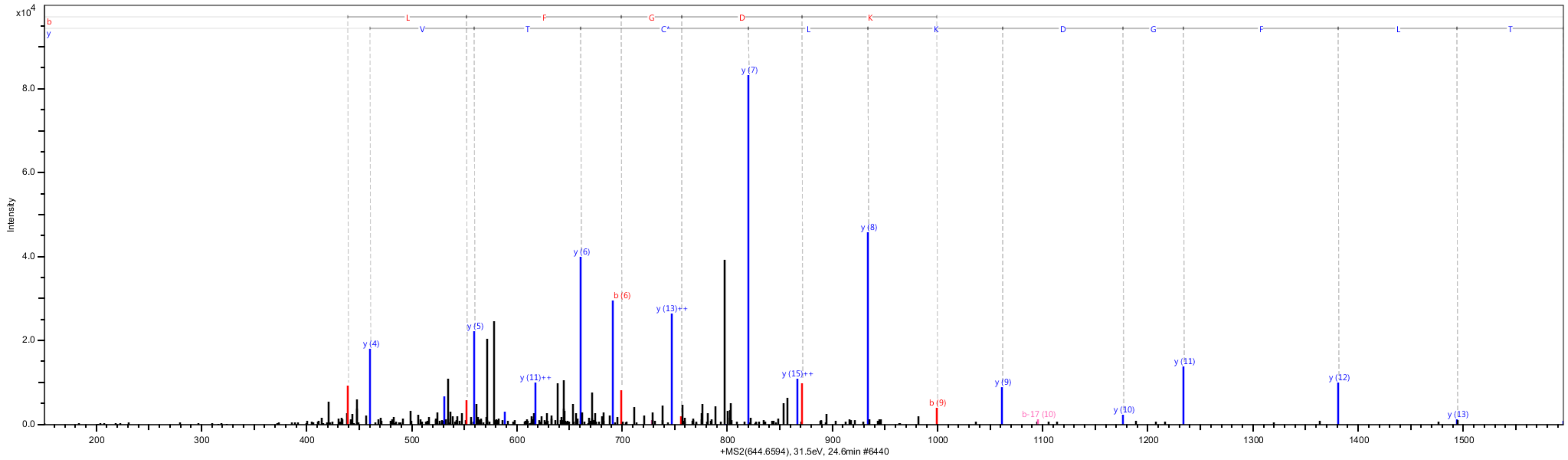

Peptide Match

**Sequence:**	K.LCTVATLR.E	**Score:**	40.9
**Modifications:**	Carbamidomethyl: 2		

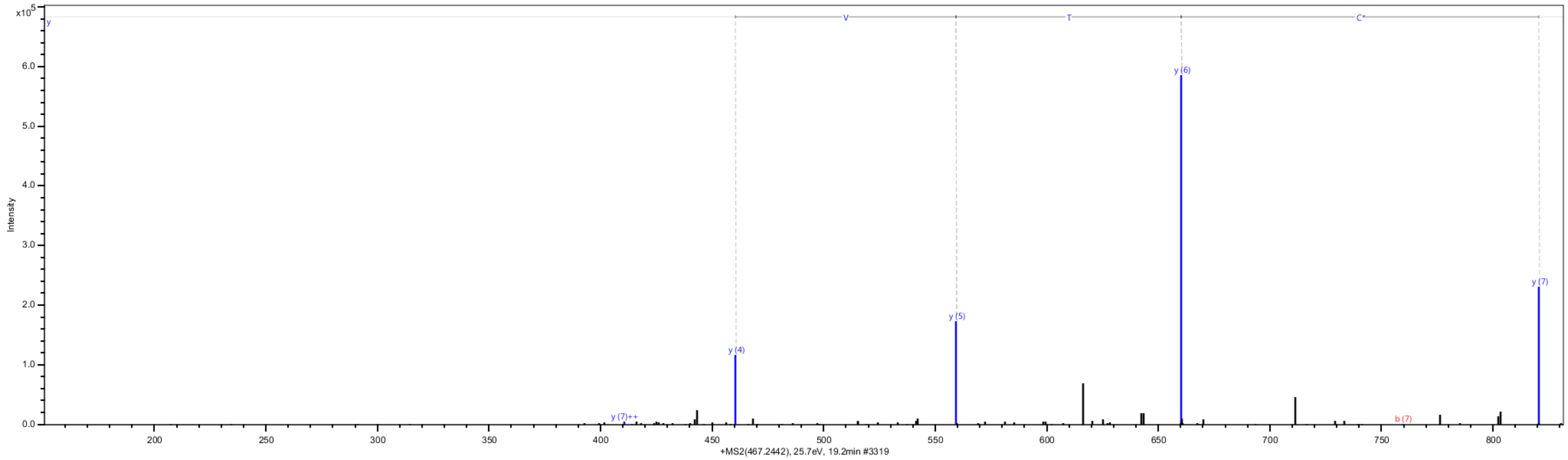

Peptide Match

Sequence: K.LCTVATLRETYGEMADCCAK.Q **Score:** 48

Modifications: Carbamidomethyl: 2, 17, 18

Spectrum Report
Combine Search_2020-10-19 07:53:31

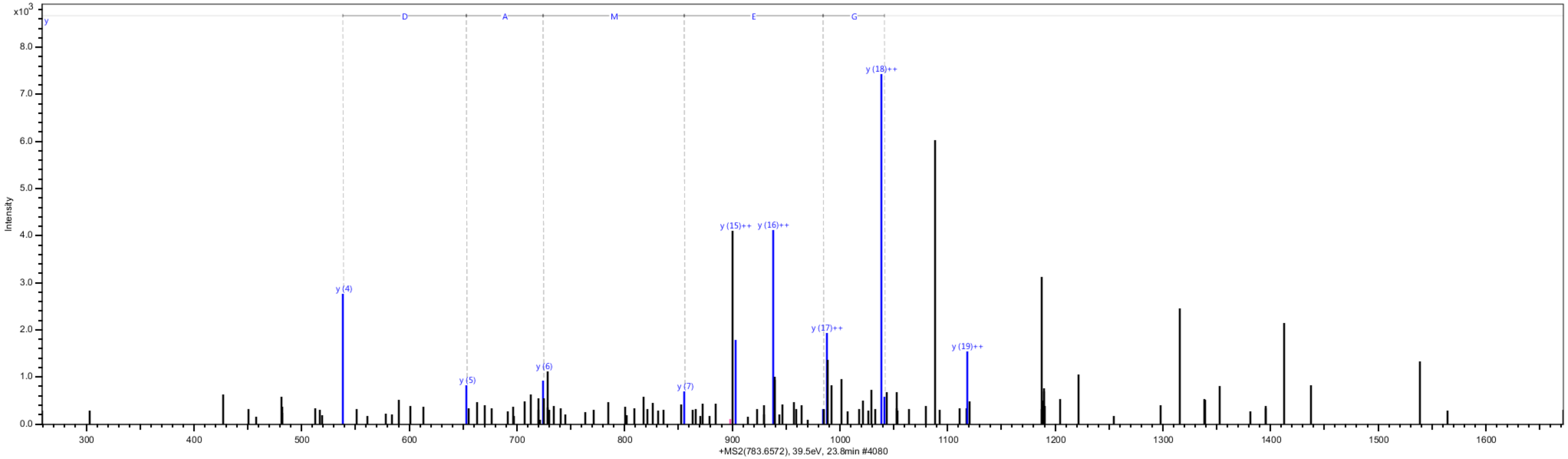

Peptide Match

Sequence: R.ETYGEMADCCAK.Q **Score:** 54.4

Modifications: Carbamidomethyl: 9, 10; Oxidation: 6

Spectrum Report
Combine Search_2020-10-19 07:53:31

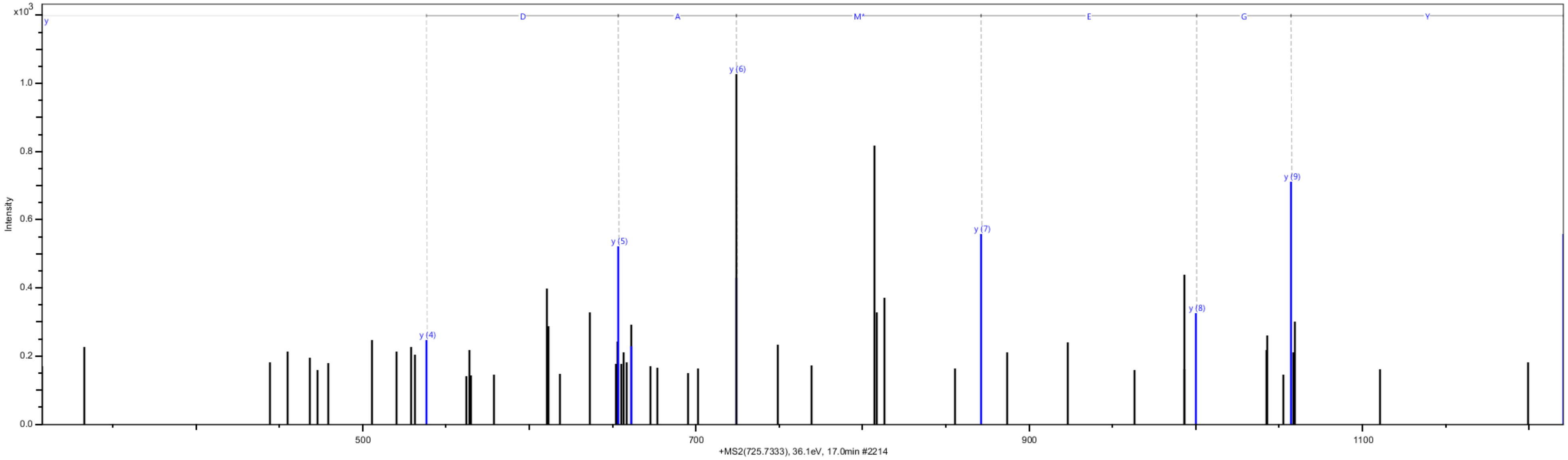

Peptide Match

**Sequence:**	R.ETYGEMADCCAK.Q	**Score:**	76.4
**Modifications:**	Carbamidomethyl: 9, 10		

Spectrum Report
Combine Search_2020-10-19 07:53:31

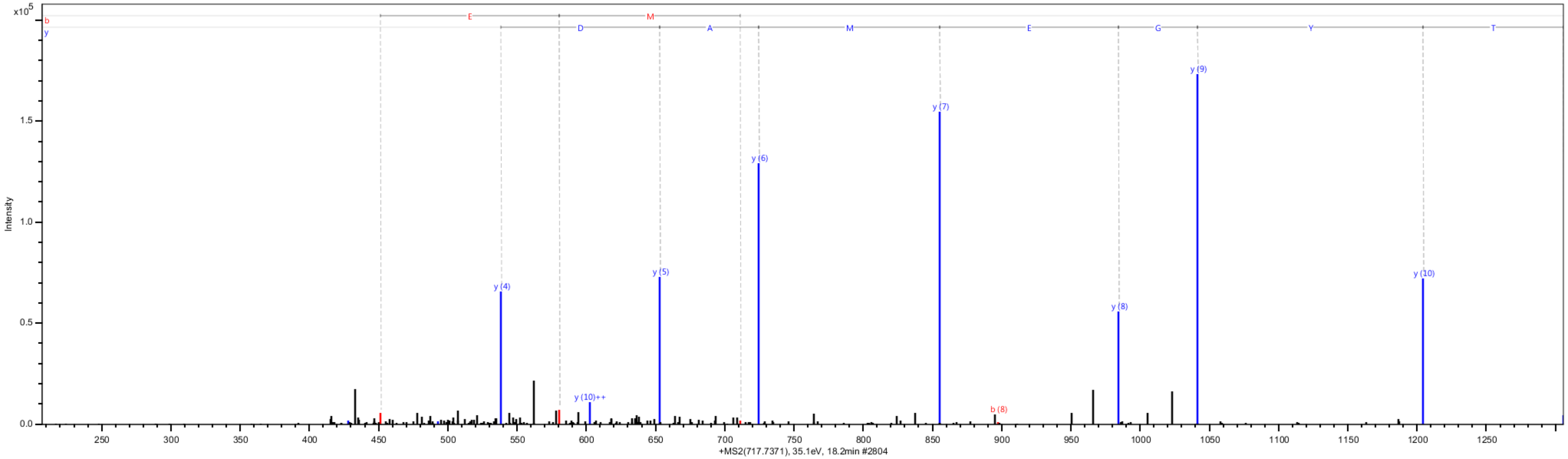

Peptide Match

**Sequence:**	R.NECFLQHKDDNPNLPR.L	**Score:**	22.8
**Modifications:**	Carbamidomethyl: 3		

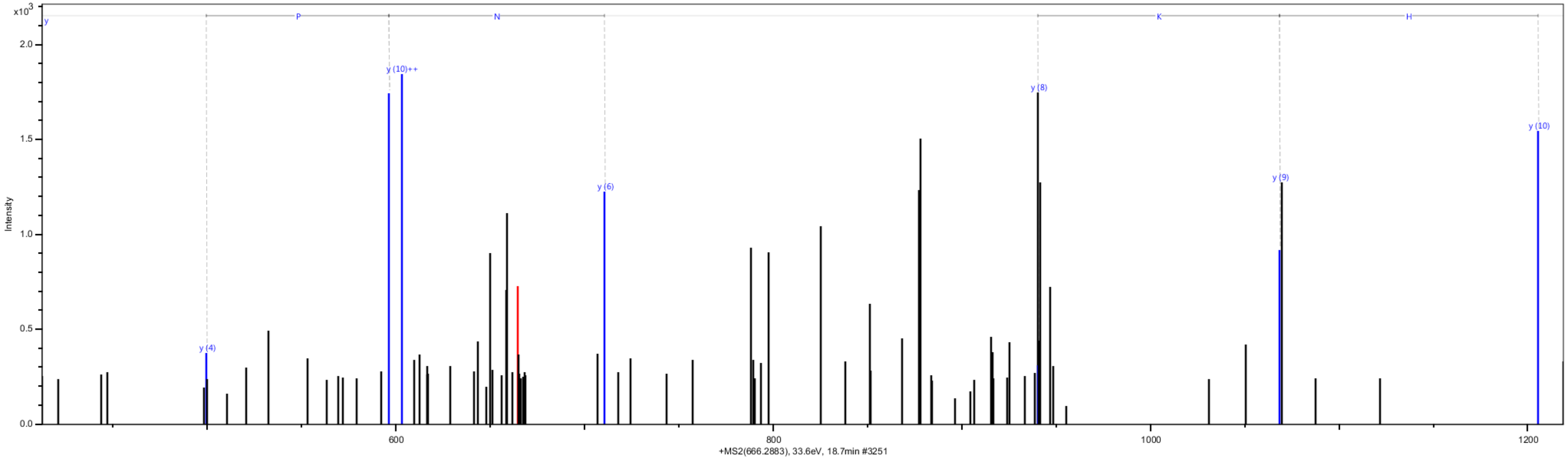

Peptide Match

**Sequence:**	R.LVRPEVDVMCTAFHDNEETFLK.K	**Score:**	30.1
**Modifications:**	Carbamidomethyl: 10; Oxidation: 9		

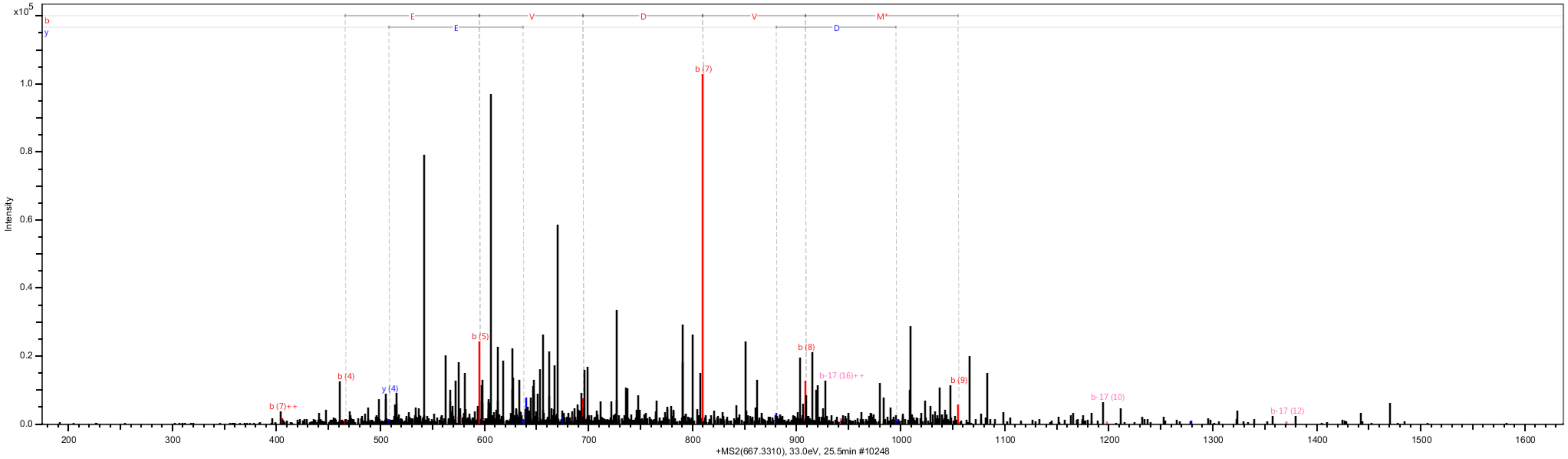

Peptide Match

Sequence: K.KYLYEIAR.R **Score:** 35

Modifications:

Spectrum Report
Combine Search_2020-10-19 07:53:31

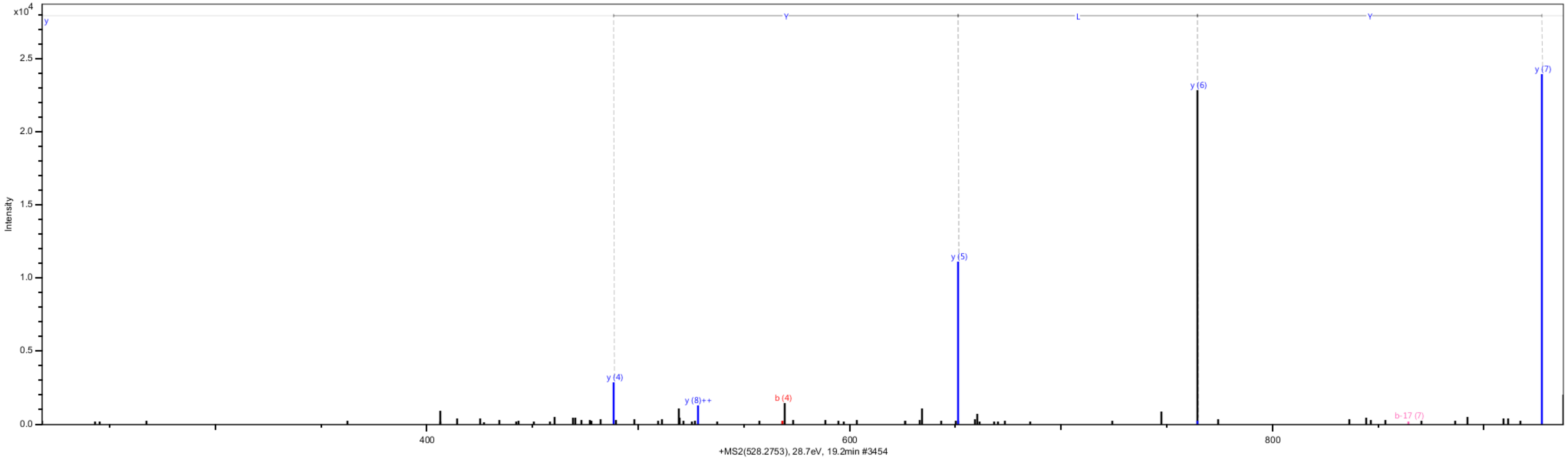

Peptide Match

**Sequence:**	K.YLYEIAR.R	**Score:**	32.6
**Modifications:**			

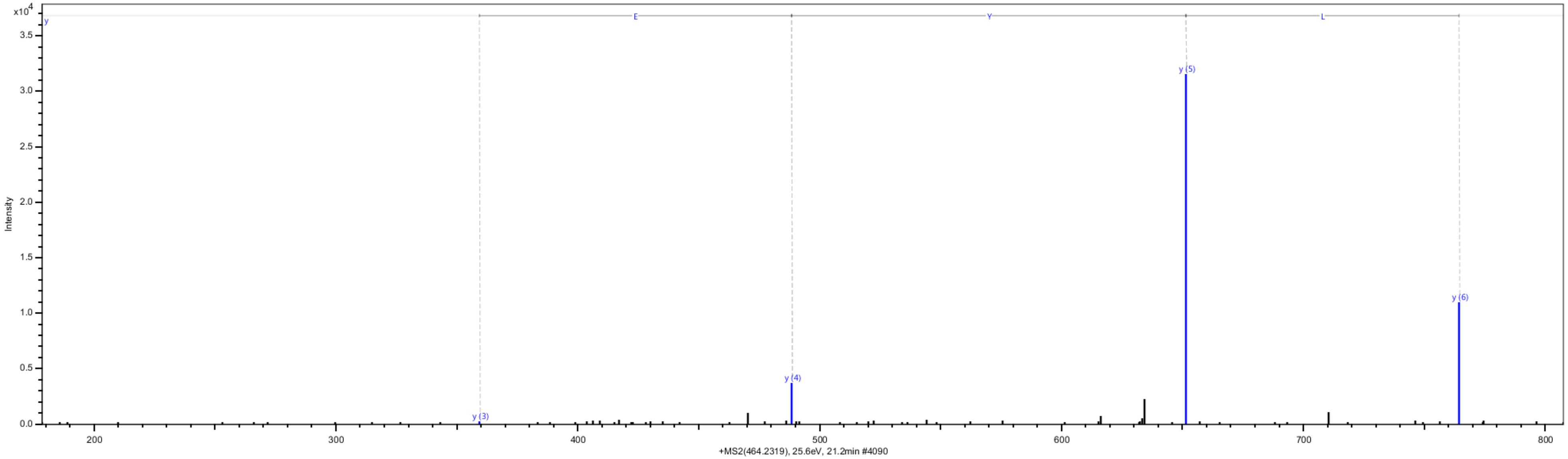

Peptide Match

Sequence: K.YLYEIARR.H **Score:** 33

Modifications:

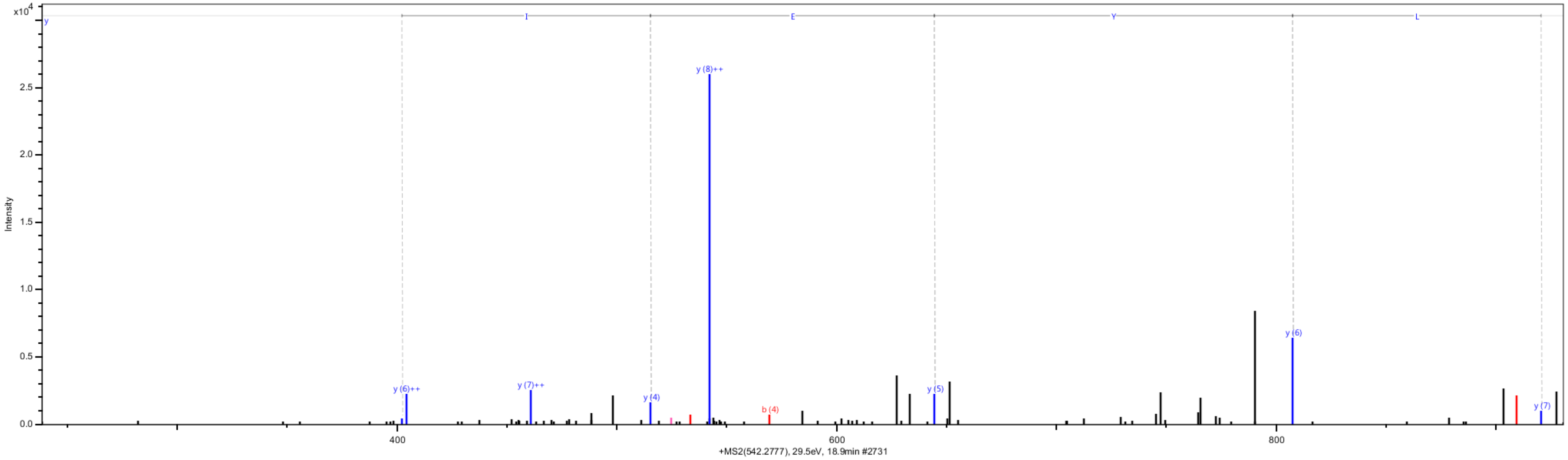

Peptide Match

Sequence: R.RHPYFYAPELLFFAK.R **Score:** 42.5

Modifications:

Spectrum Report
Combine Search_2020-10-19 07:53:31

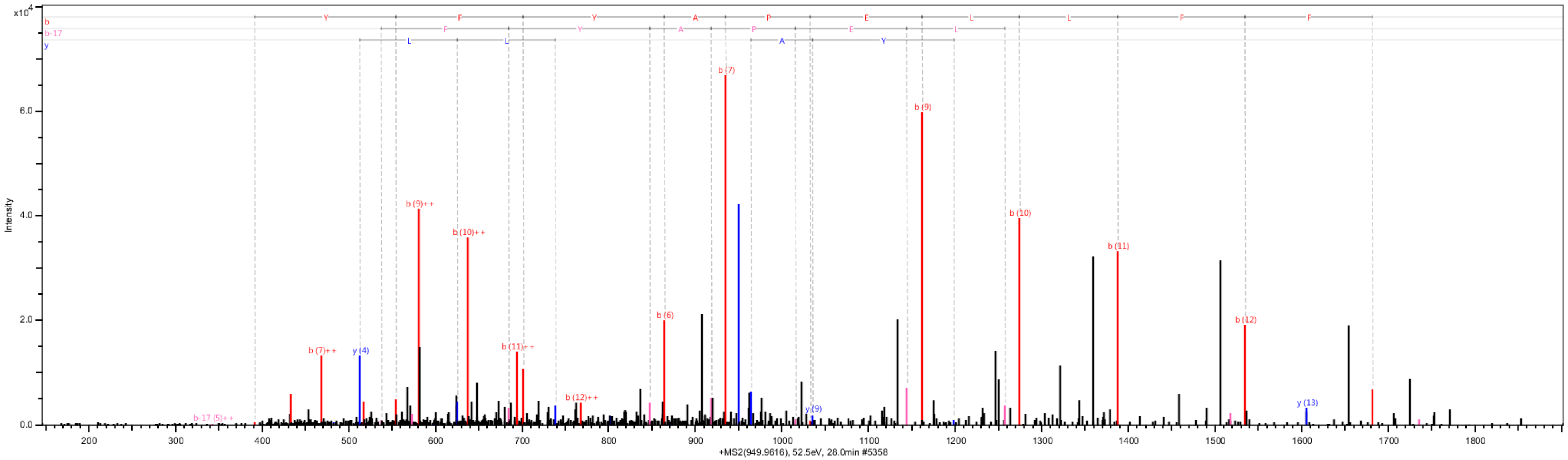

Peptide Match

**Sequence:**	R.HPYFYAPELLFFAK.R	**Score:**	60.7
**Modifications:**			

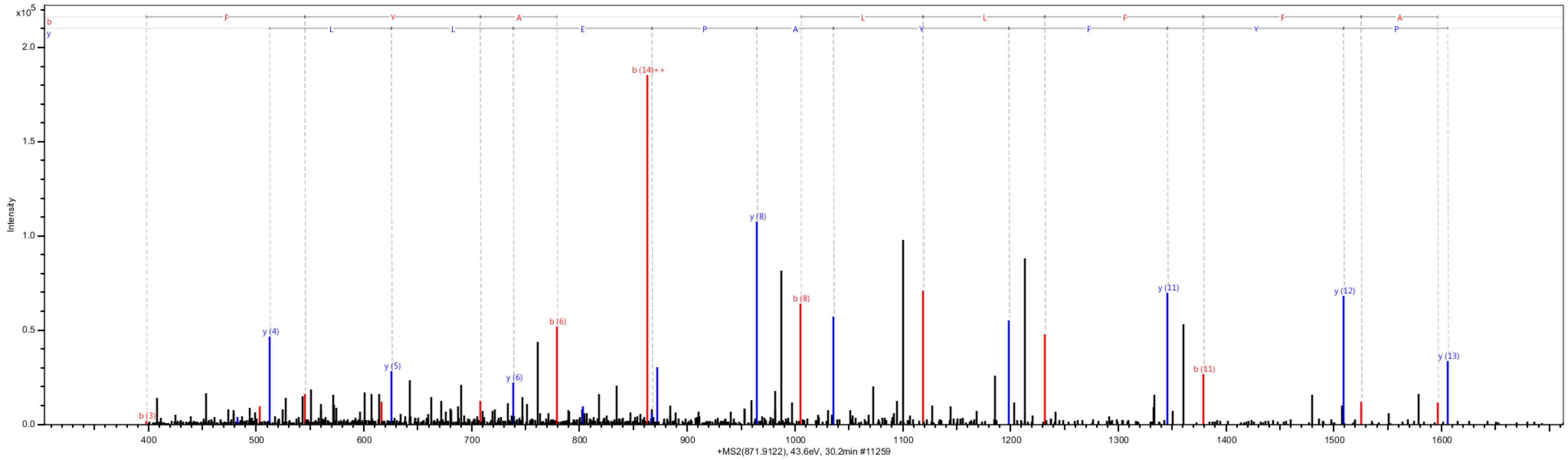

Peptide Match

**Sequence:**	R.YKAAFTECCQAADK.A	**Score:**	75.1
**Modifications:**	Carbamidomethyl: 8, 9		

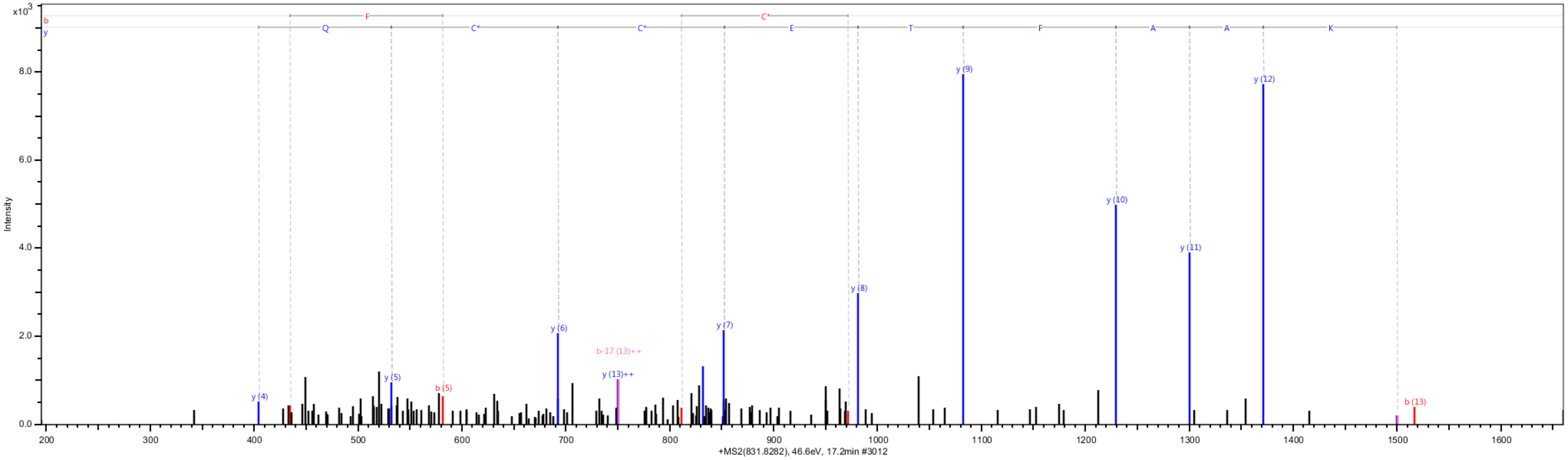

Peptide Match

**Sequence:**	K.AAFTECCQAADK.A	**Score:**	67.4
**Modifications:**	Carbamidomethyl: 6, 7		

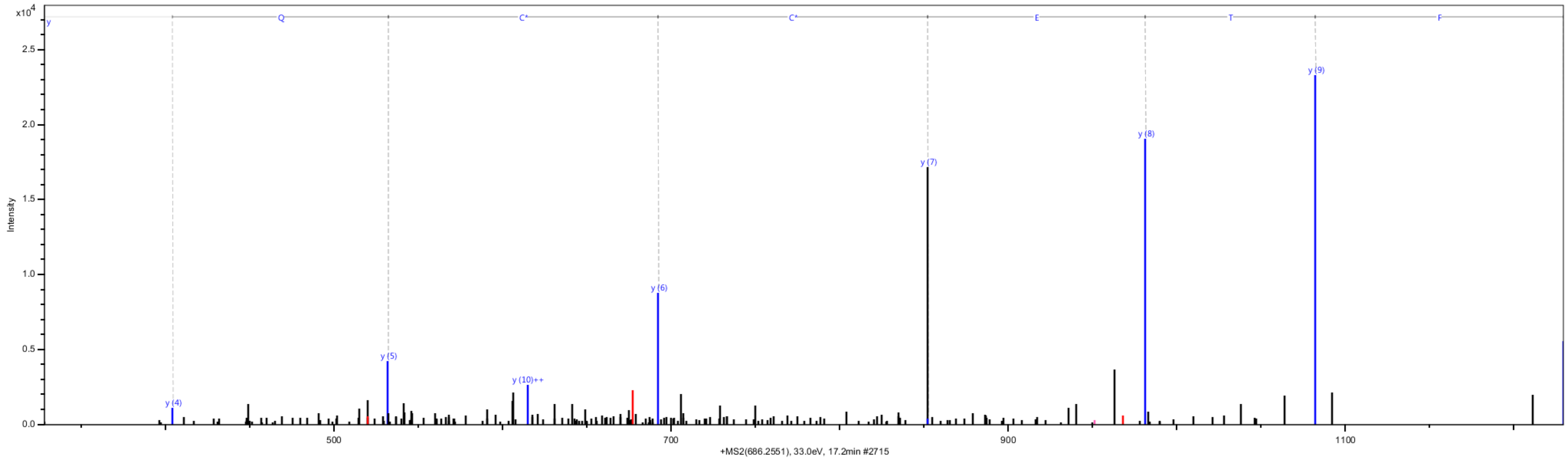

Peptide Match

**Sequence:**	K.AAFTECCQAADKAACLLPK.L	**Score:**	92.7
**Modifications:**	Carbamidomethyl: 6, 7, 15		

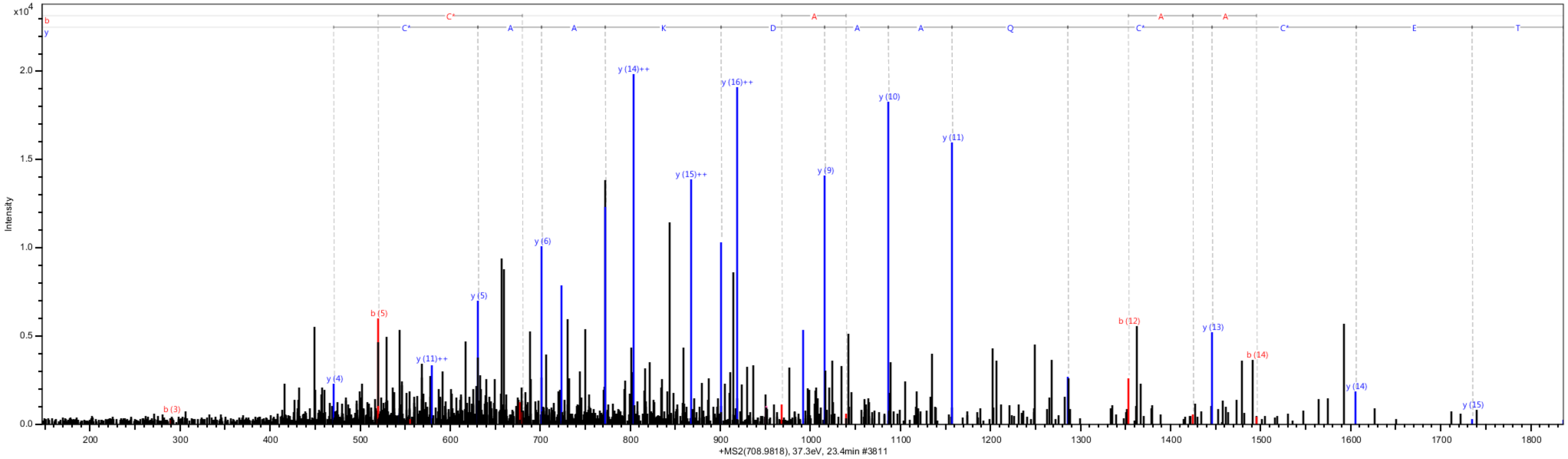

Peptide Match

**Sequence:**	K.AACLLPKLDELR.D	**Score:**	58.2
**Modifications:**	Carbamidomethyl: 3		

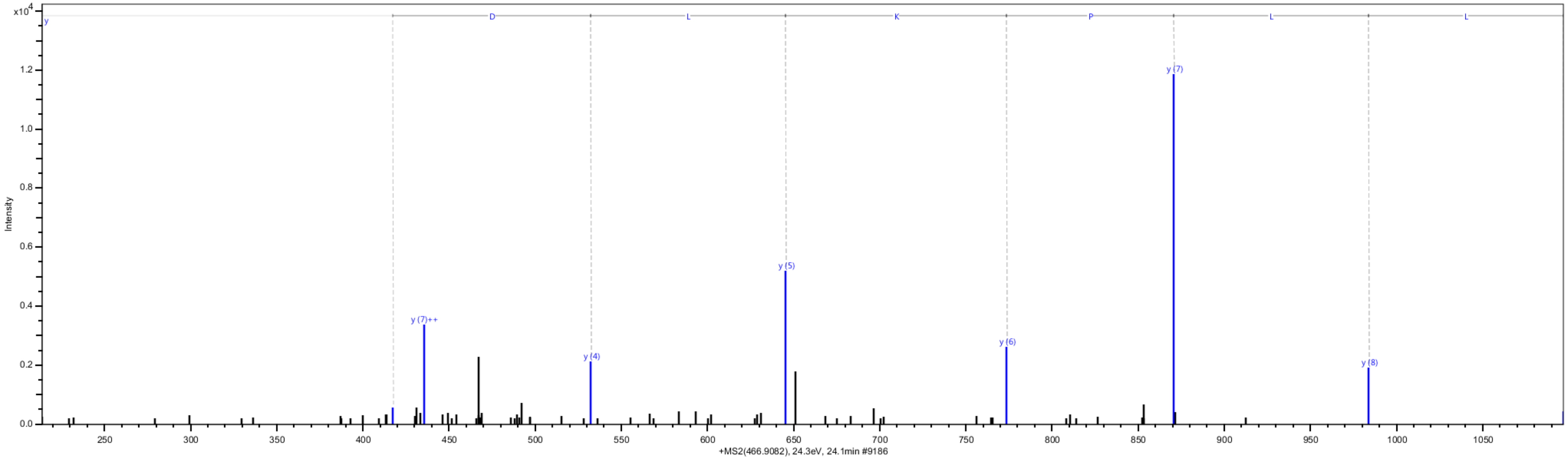

Peptide Match

Sequence: K.LDELRDEGK.A **Score:** 43.8

Modifications:

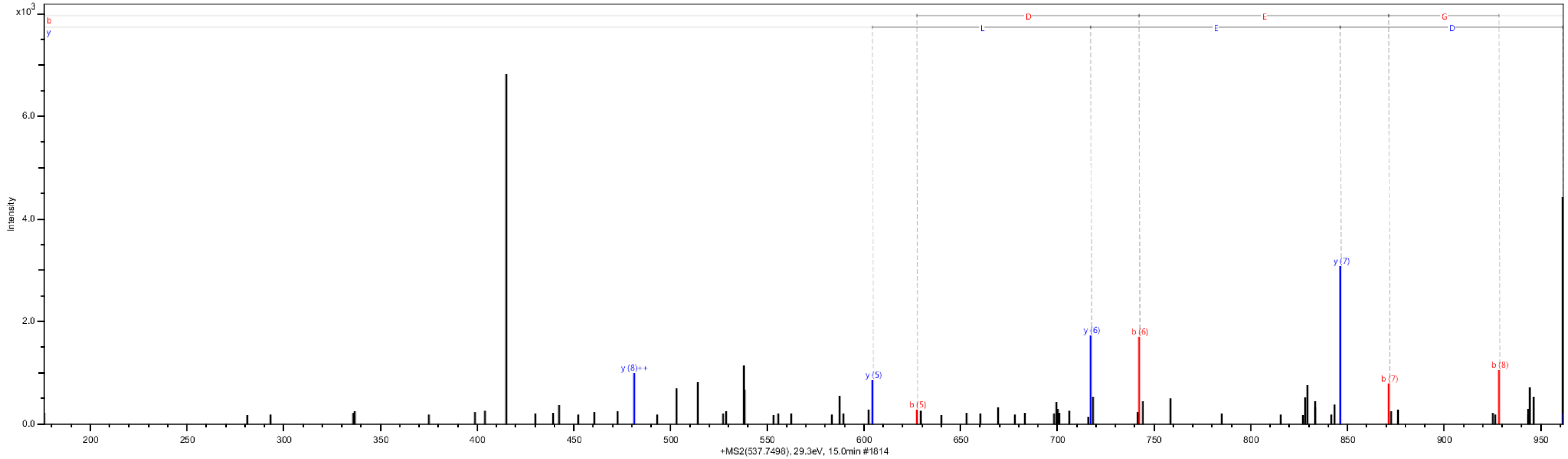

Peptide Match

**Sequence:**	R.LKCASLQK.F	**Score:**	21.3
**Modifications:**	Carbamidomethyl: 3		

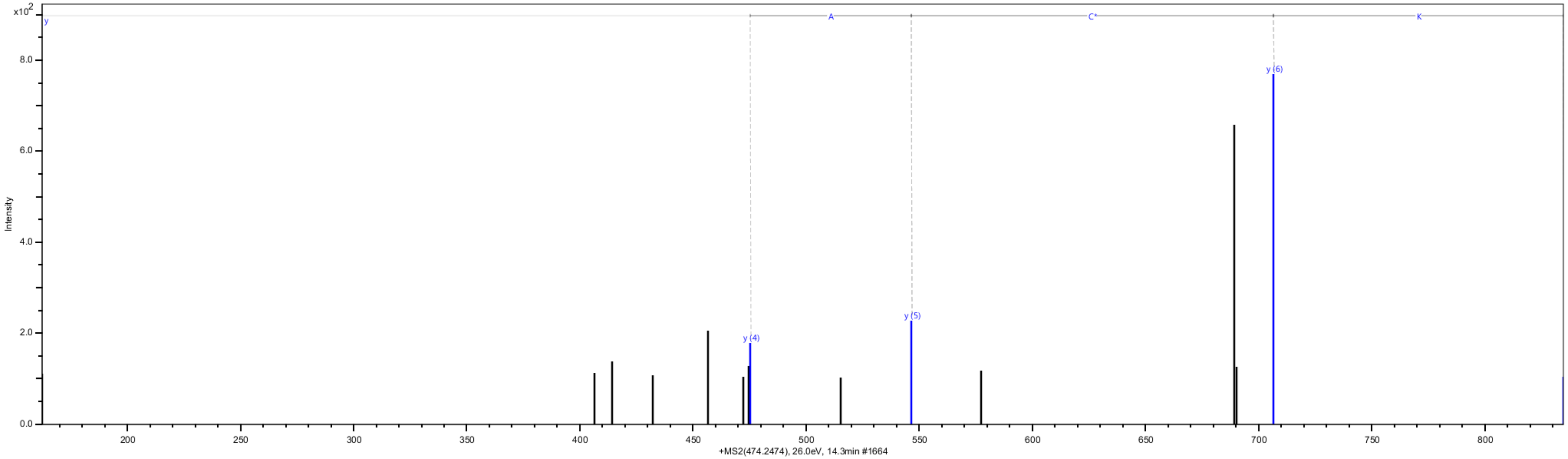

Peptide Match

**Sequence:**	K.CASLQKFGER.A	**Score:**	49
**Modifications:**	Carbamidomethyl: 1		

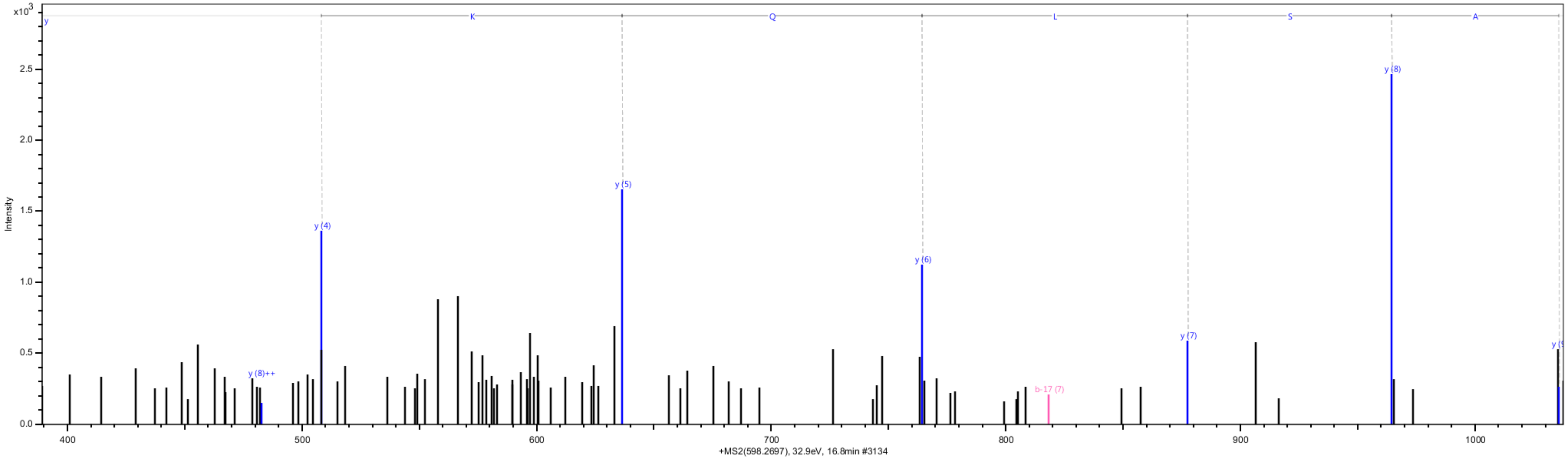

Peptide Match

Sequence: R.AFKAWAVAR.L **Score:** 48.4

Modifications:

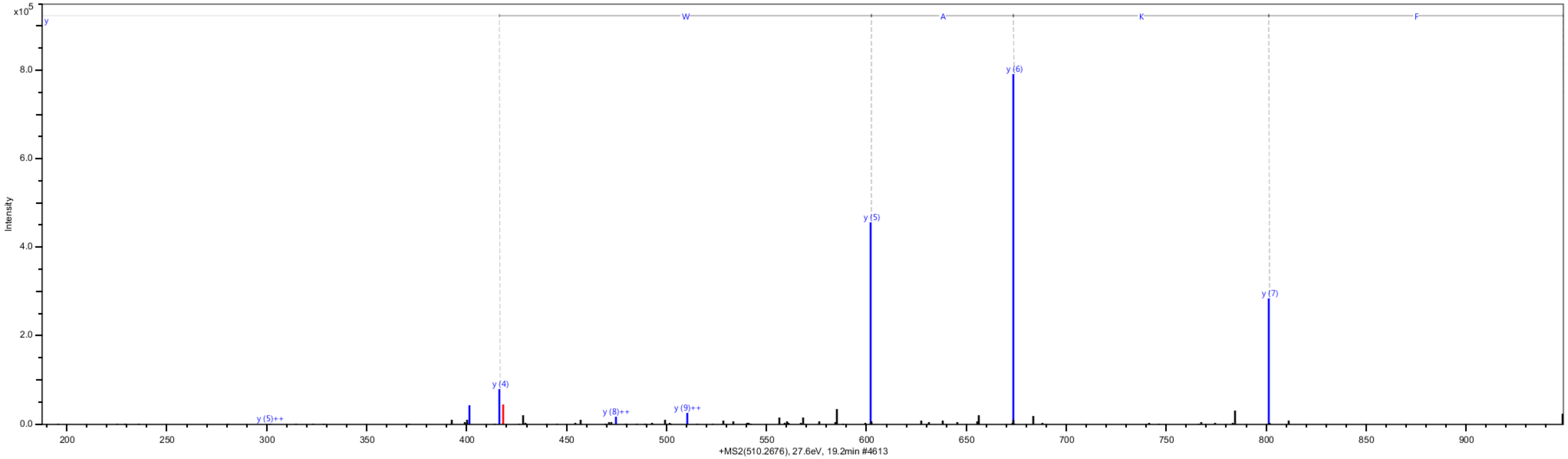

Peptide Match

Sequence: R.LSQRFPK.A **Score:** 28.4

Modifications:

Spectrum Report
Combine Search_2020-10-19 07:53:31

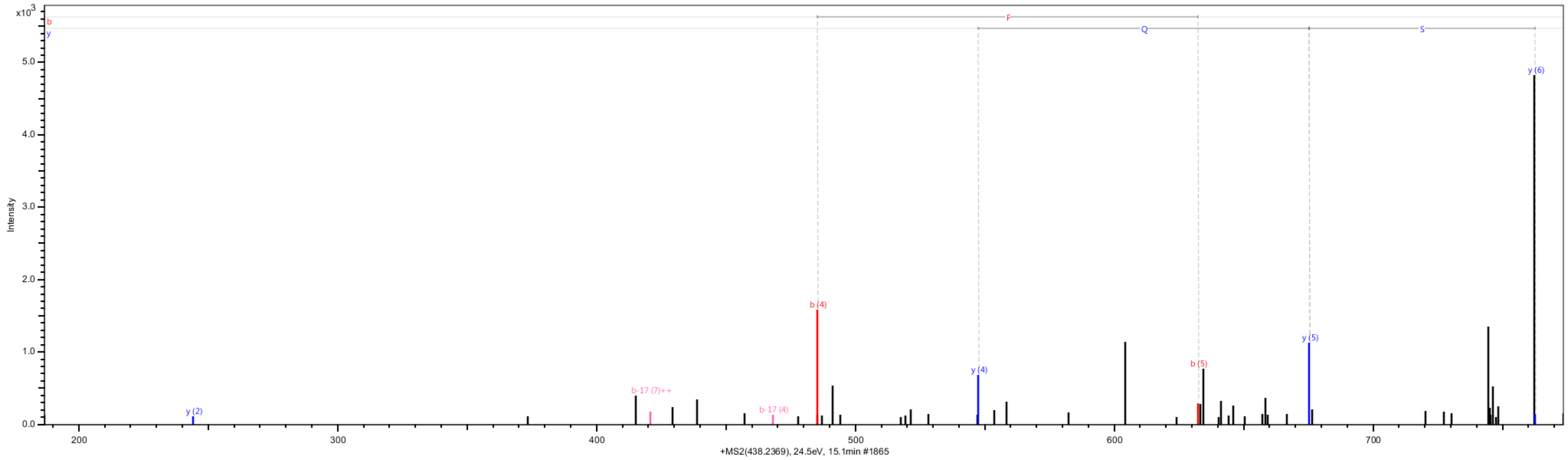

Peptide Match

**Sequence:**	R.FPKAEFAEVSK.L	**Score:**	62.5
**Modifications:**			

Spectrum Report
Combine Search_2020-10-19 07:53:31

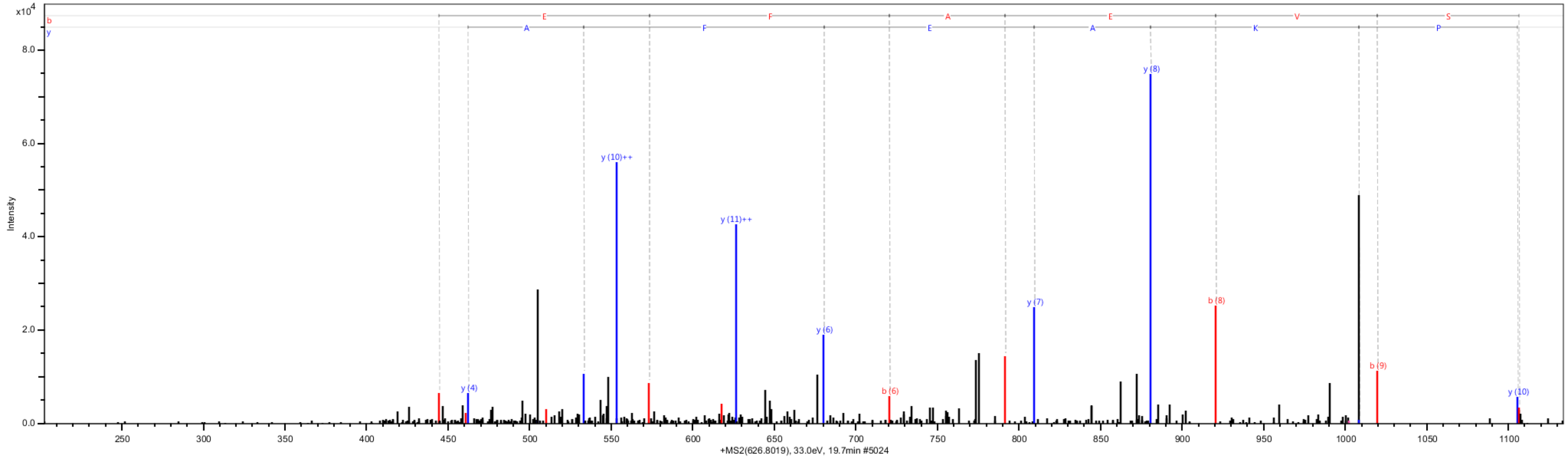

Peptide Match

**Sequence:**	K.AEFAEVSK.L	**Score:**	36
**Modifications:**			

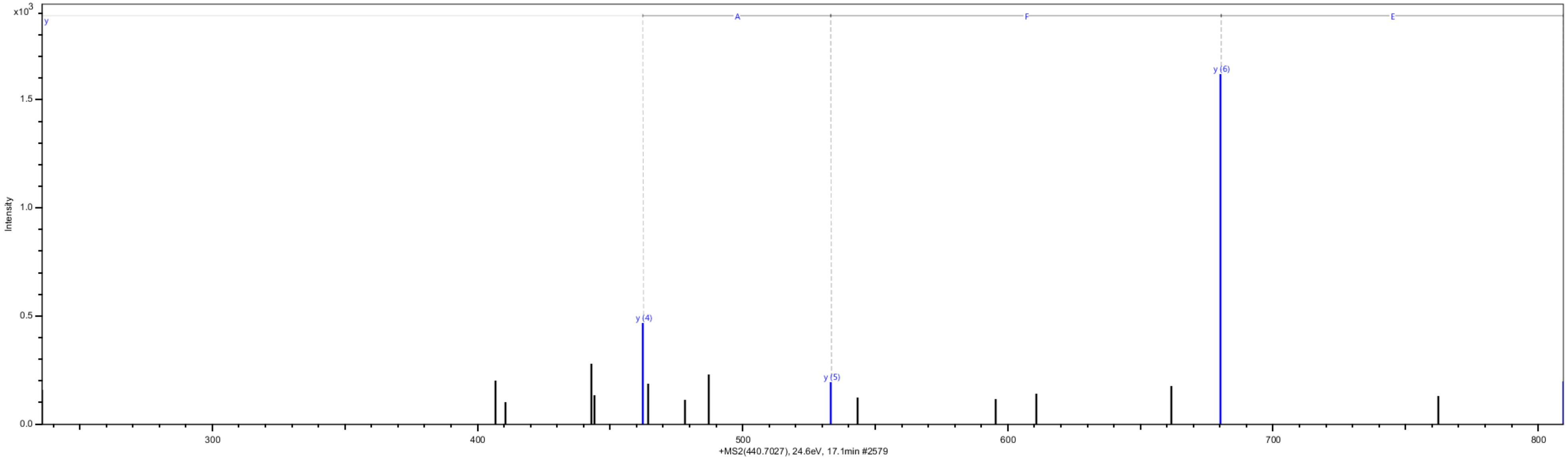

Peptide Match

Sequence: K.AEFAEVSKLVTDLTK.V **Score:** 124

Modifications:

Spectrum Report
Combine Search_2020-10-19 07:53:31

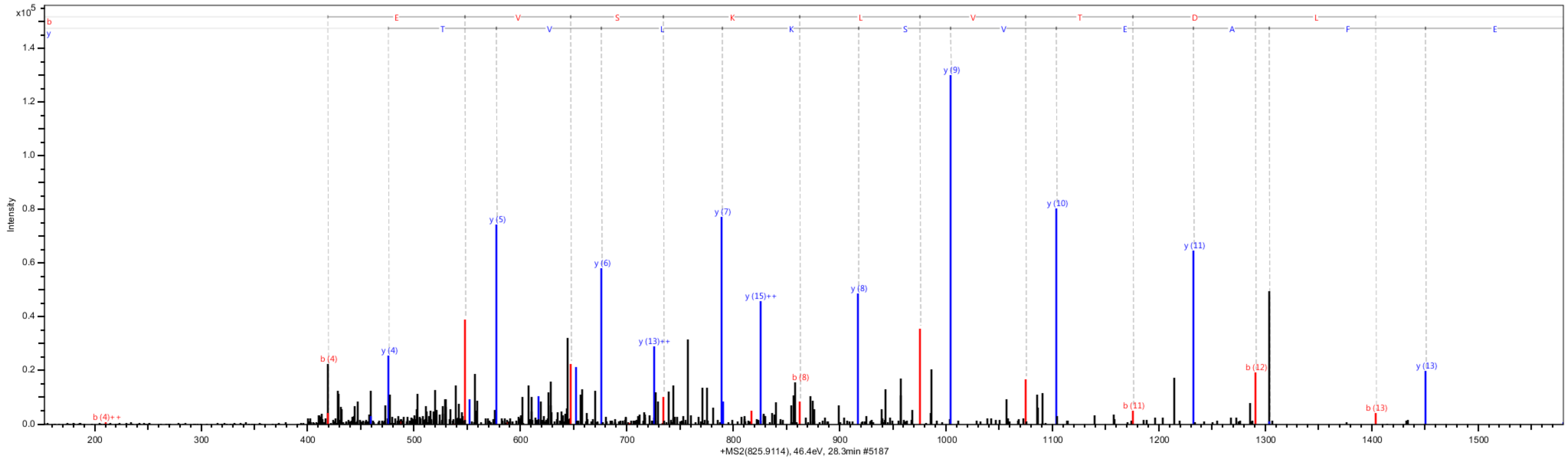

Peptide Match

**Sequence:**	K.LVTDLTK.V	**Score:**	32.3
**Modifications:**			

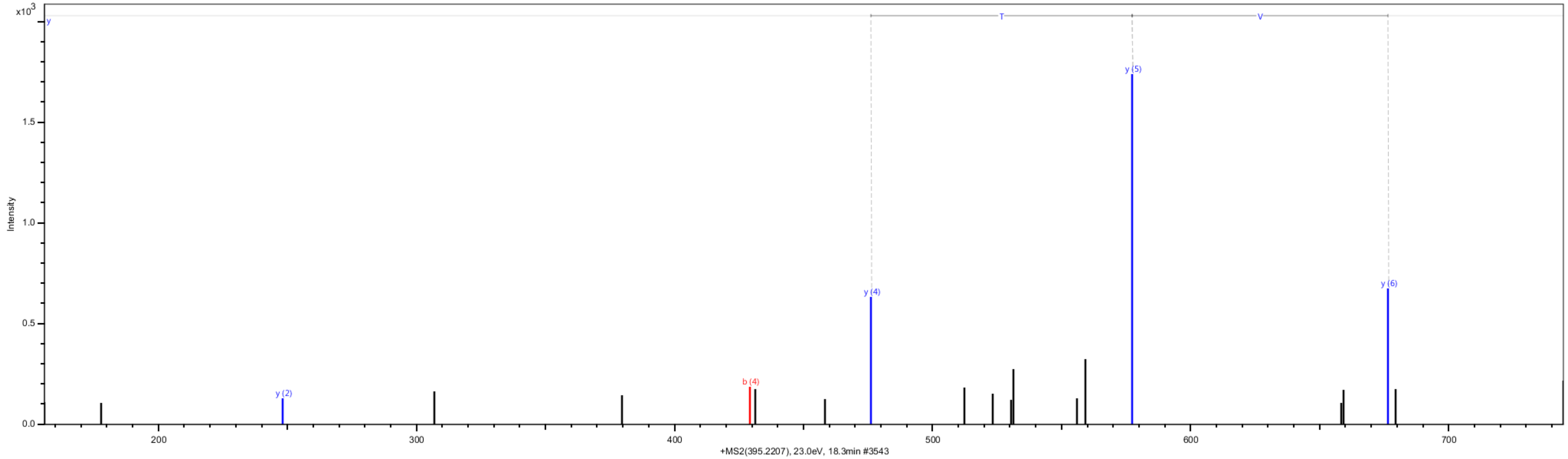

Peptide Match

**Sequence:**	K.YICENQDSISSK.L	**Score:**	86.4
**Modifications:**	Carbamidomethyl: 3		

Spectrum Report
Combine Search_2020-10-19 07:53:31

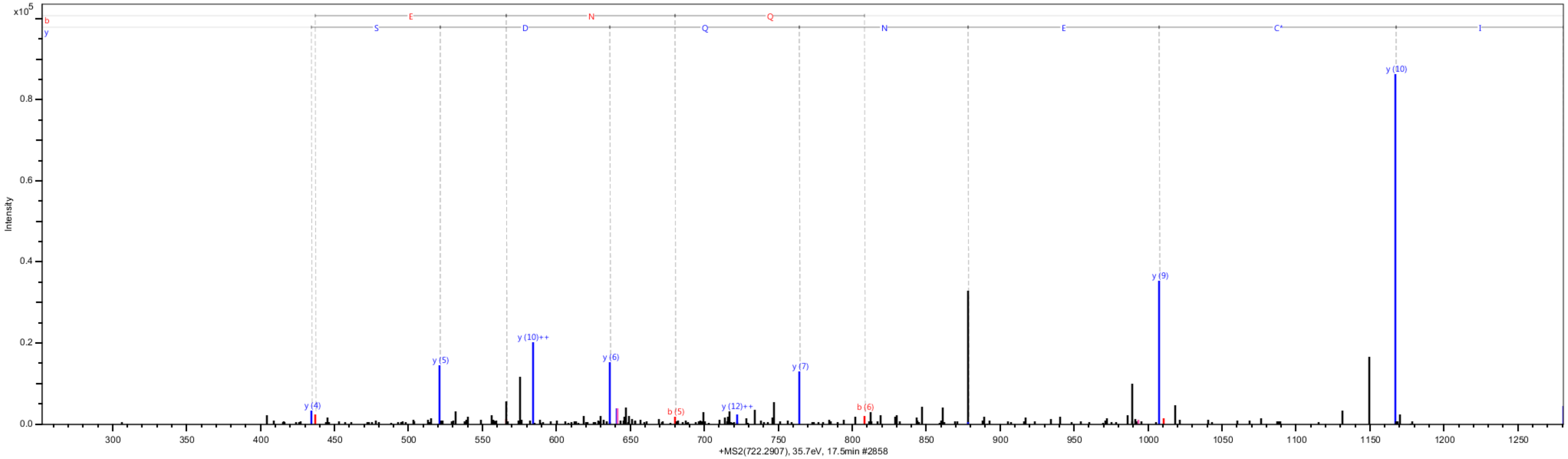

Peptide Match

**Sequence:**	K.YICENQDSISSKLK.E	**Score:**	62.5
**Modifications:**	Carbamidomethyl: 3		

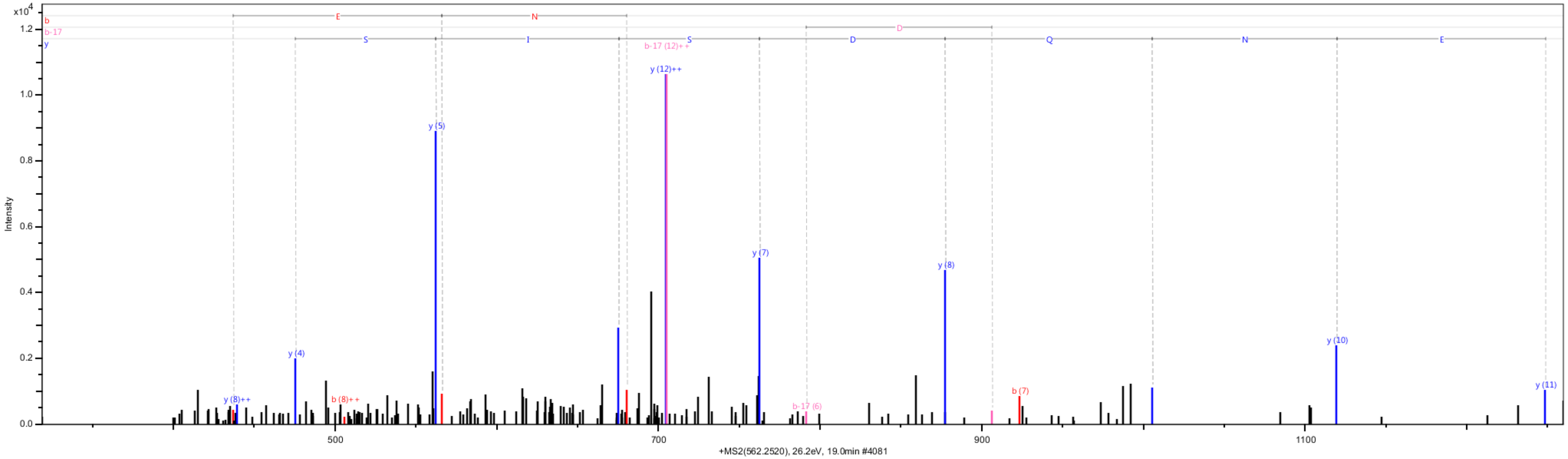

Peptide Match

**Sequence:**	K.LKECCEKPLLEK.S	**Score:**	65.8
**Modifications:**	Carbamidomethyl: 4, 5		

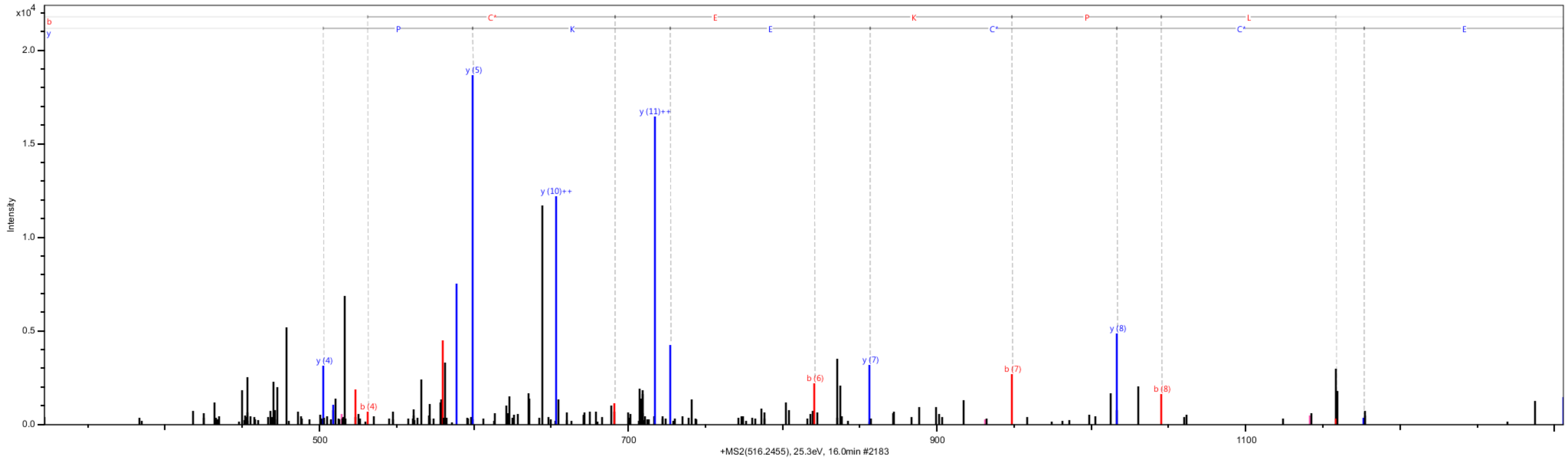

Peptide Match

**Sequence:**	K.ECCEKPLLEK.S	**Score:**	24.5
**Modifications:**	Carbamidomethyl: 2, 3		

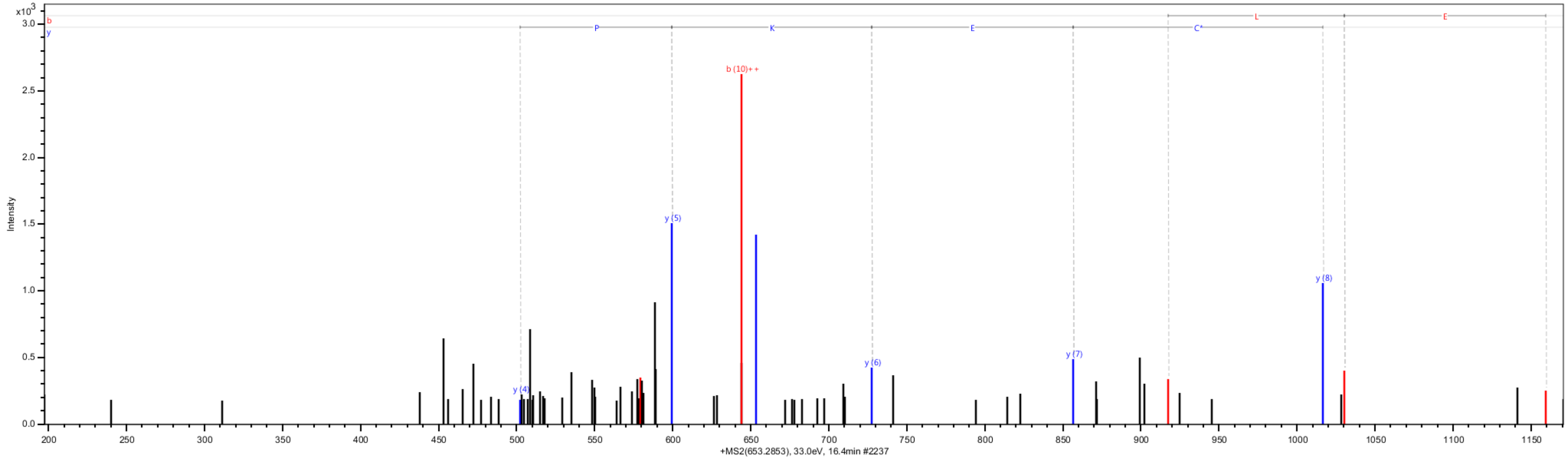

Peptide Match

Sequence: K.SHCIAEVENDEMPADLPSLAADFVESK DVCK.N **Score:** 35.8

Modifications: Carbamidomethyl: 3, 30

Spectrum Report
Combine Search_2020-10-19 07:53:31

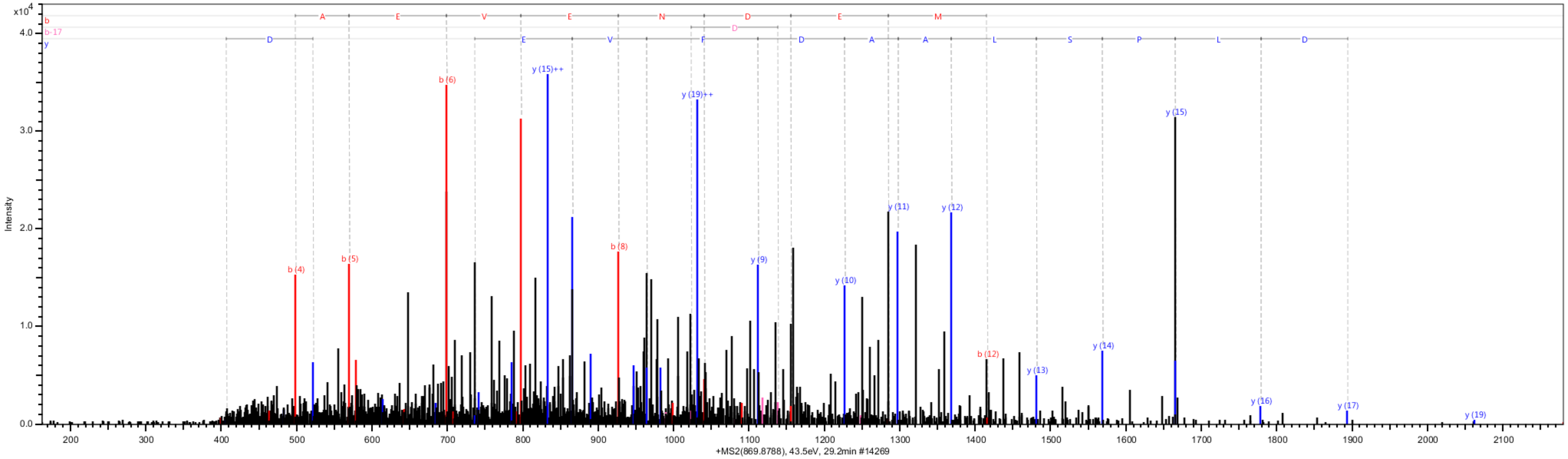

Peptide Match

Sequence: K.NYAEAKDVFLGMFLYEYAR.R **Score:** 16.4

Modifications:

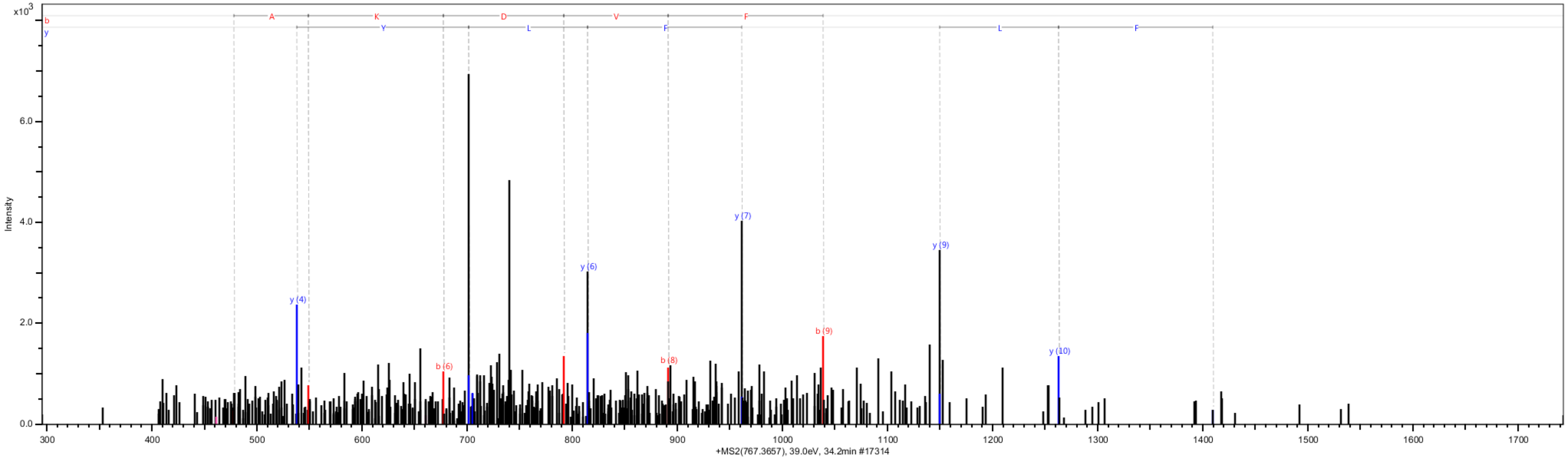

Peptide Match

**Sequence:**	K.DVFLGMFLYEYAR.R	**Score:**	90.6
**Modifications:**	Oxidation: 6		

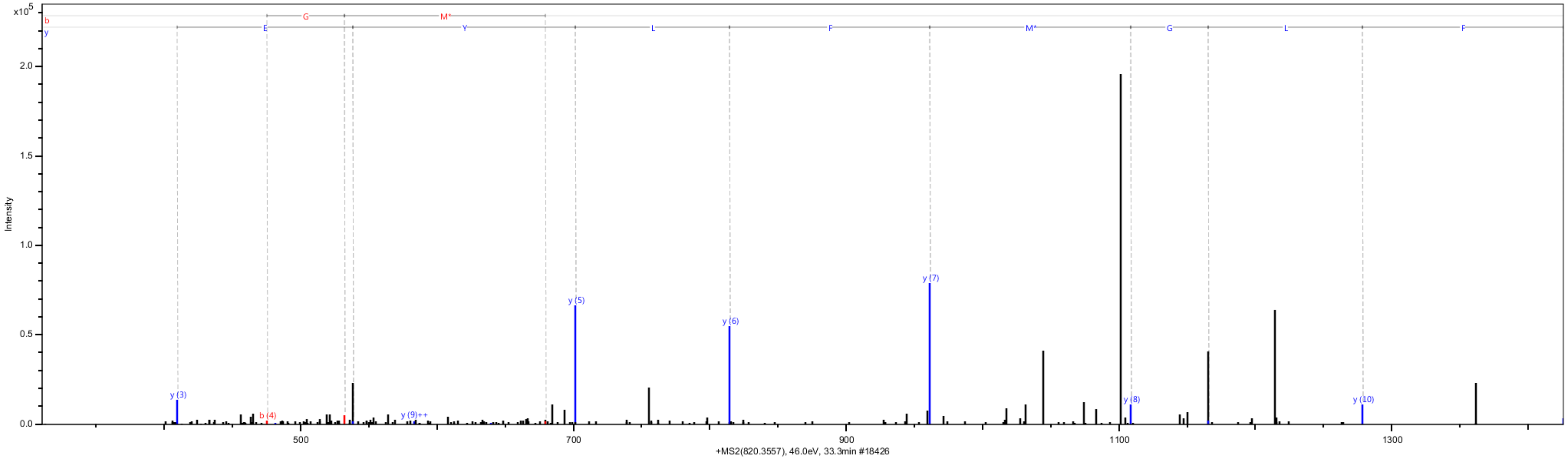

Peptide Match

Sequence: K.DVFLGMFLYEYAR.R **Score:** 105

Modifications:

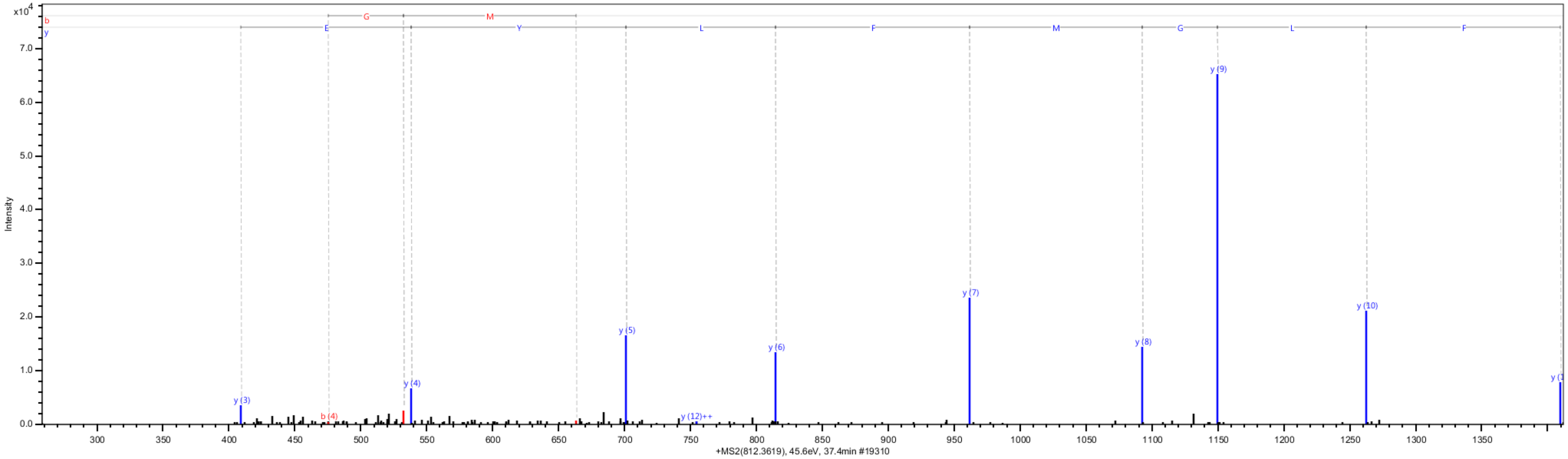

Peptide Match

Sequence: R.RHPDYSVVLLLR.L **Score:** 83

Modifications:

Spectrum Report
Combine Search_2020-10-19 07:53:31

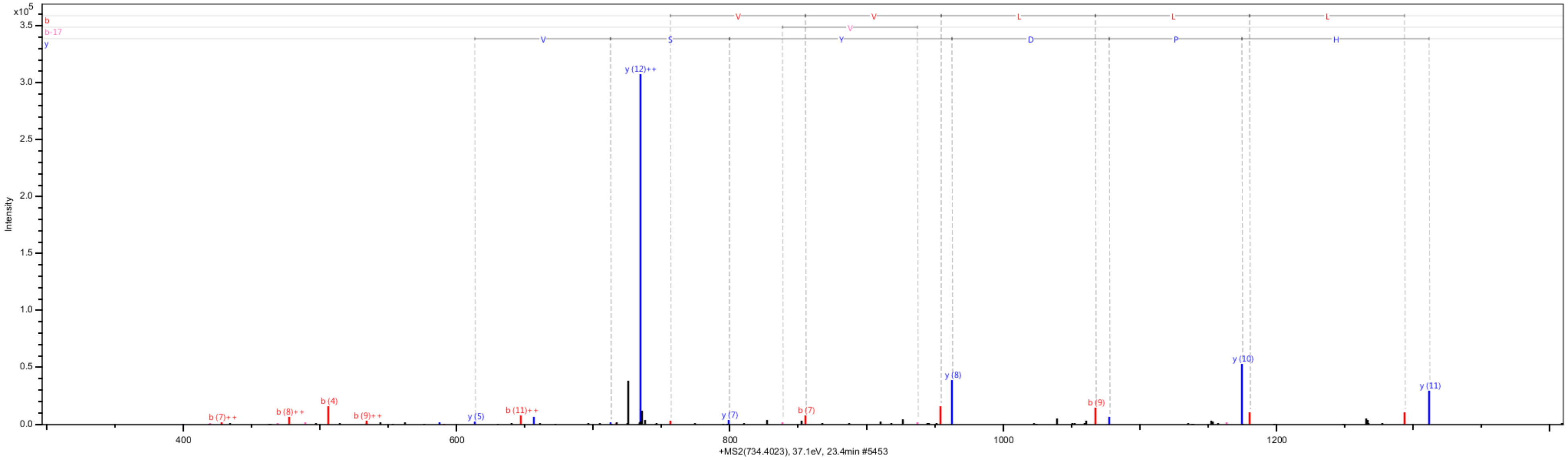

Peptide Match

Sequence: R.HPDYSVVLLLR.L **Score:** 83

Modifications:

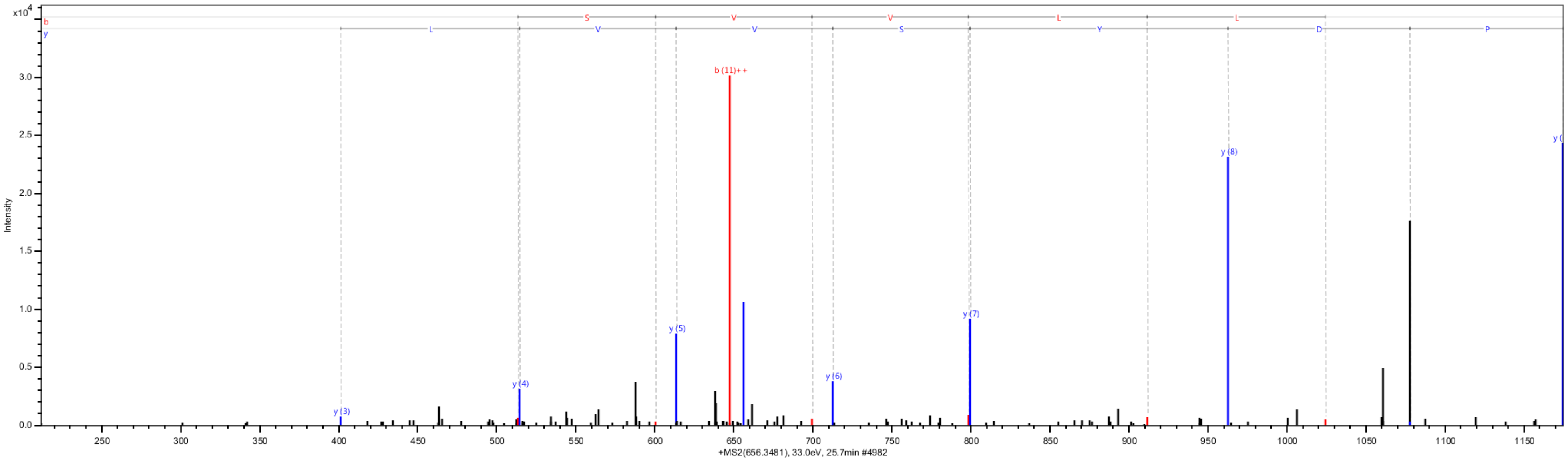

x10^4
b
y
S V V L L
L V V S Y D P
b (11)++
y (3)
y (4)
y (5)
y (6)
y (7)
y (8)
Intensity
+MS2(656.3481), 33.0eV, 25.7min #4982

Peptide Match

Sequence: R.LAKTYETTLEK.C **Score:** 55

Modifications:

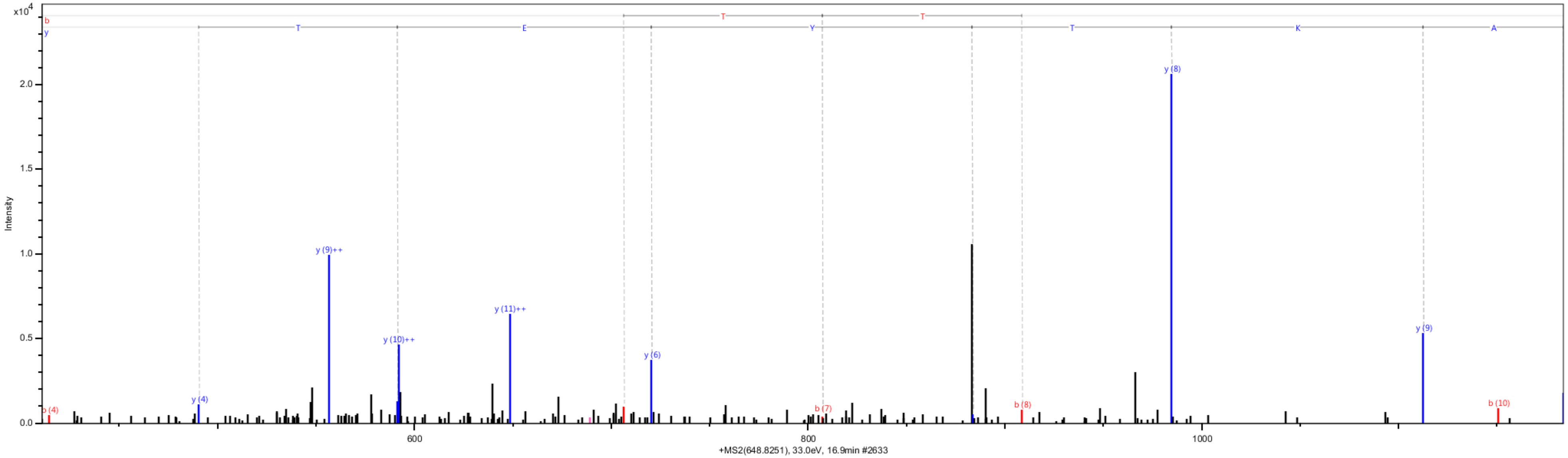

Peptide Match

Sequence: K.TYETTLEK.C **Score:** 26.1

Modifications:

Spectrum Report
Combine Search_2020-10-19 07:53:31

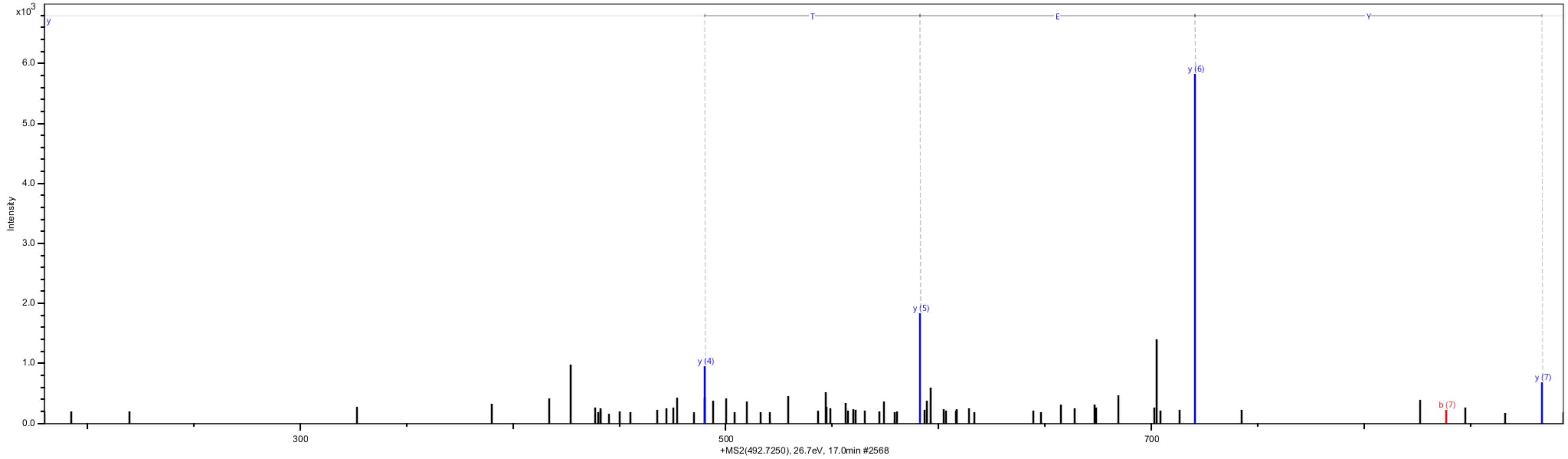

Peptide Match

Sequence: K.CCAAADPHECYAK.V **Score:** 54.6

Modifications: Carbamidomethyl: 1, 2, 10

Spectrum Report
Combine Search_2020-10-19 07:53:31

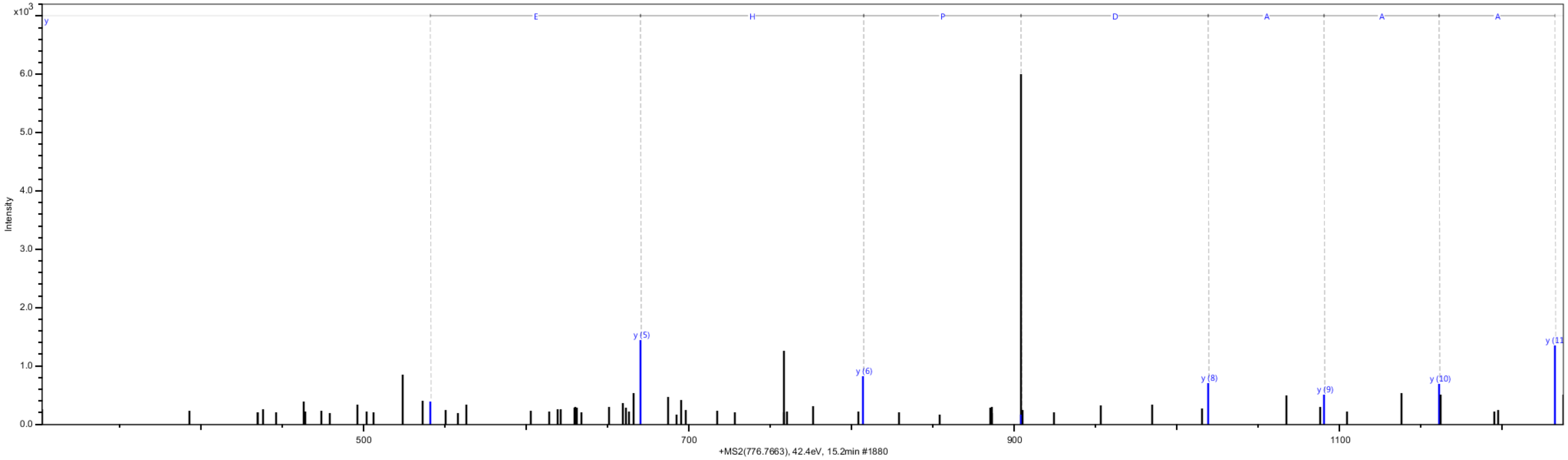

Peptide Match

**Sequence:**	K.QNCELFEQLGEYK.F	**Score:**	80.4
**Modifications:**	Carbamidomethyl: 3		

Spectrum Report
Combine Search_2020-10-19 07:53:31

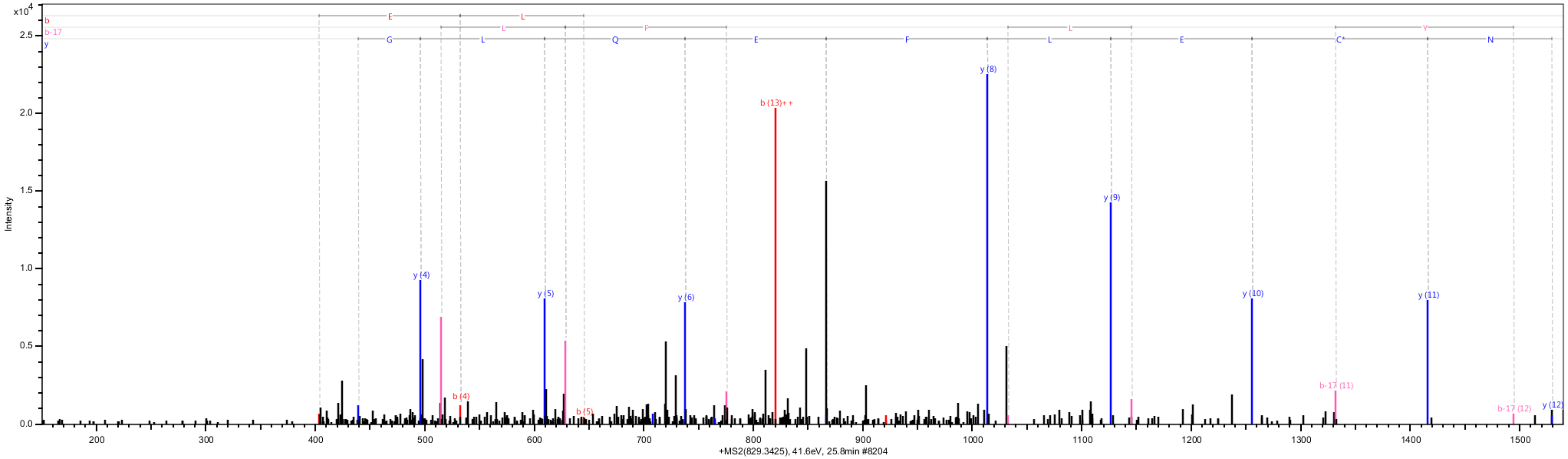

Peptide Match

**Sequence:**	K.FQNALLVR.Y	**Score:**	40.9
**Modifications:**			

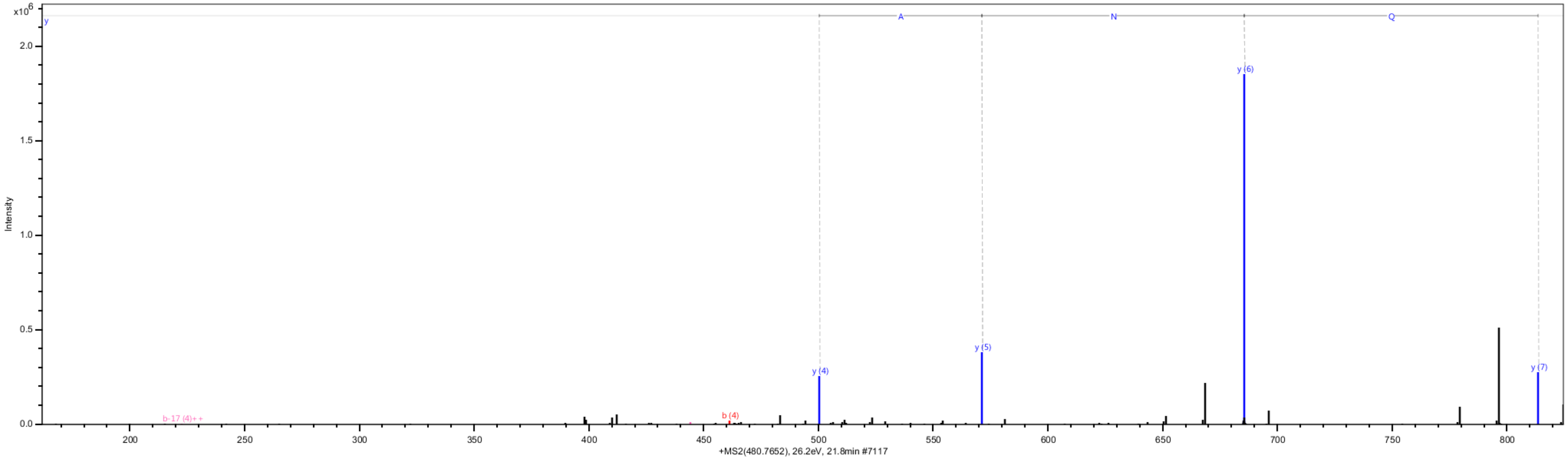

Peptide Match

**Sequence:**	K.FQNALLVRYTK.K	**Score:**	17.6
**Modifications:**			

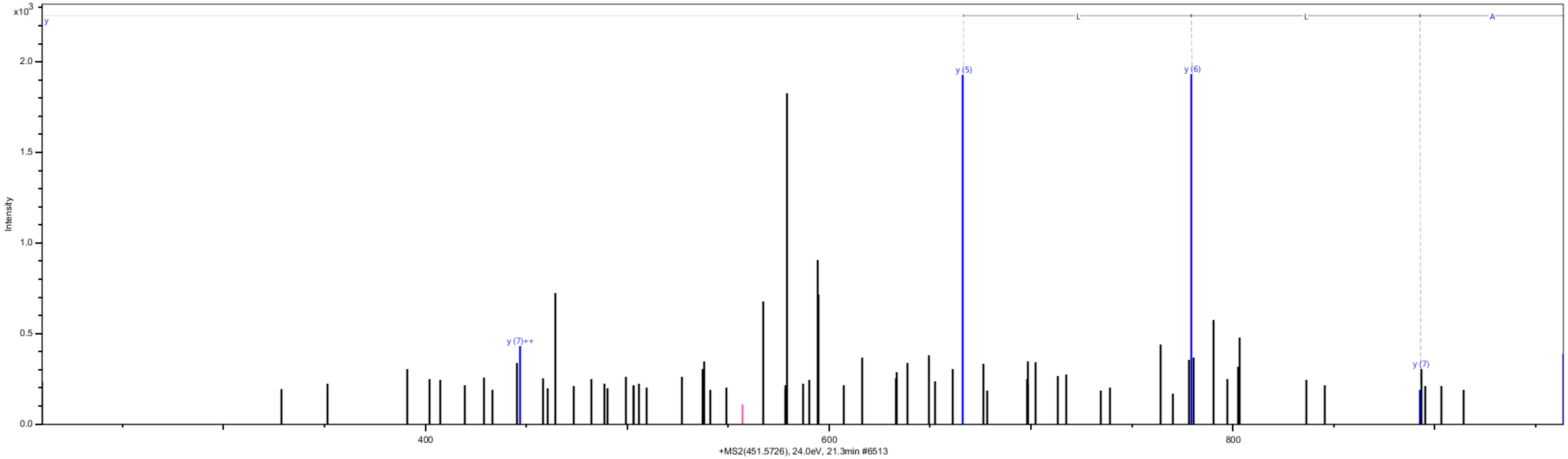

Peptide Match

Sequence: K.KVPQVSTPTLVEVSR.N **Score:** 126.2

Modifications:

Spectrum Report
Combine Search_2020-10-19 07:53:31

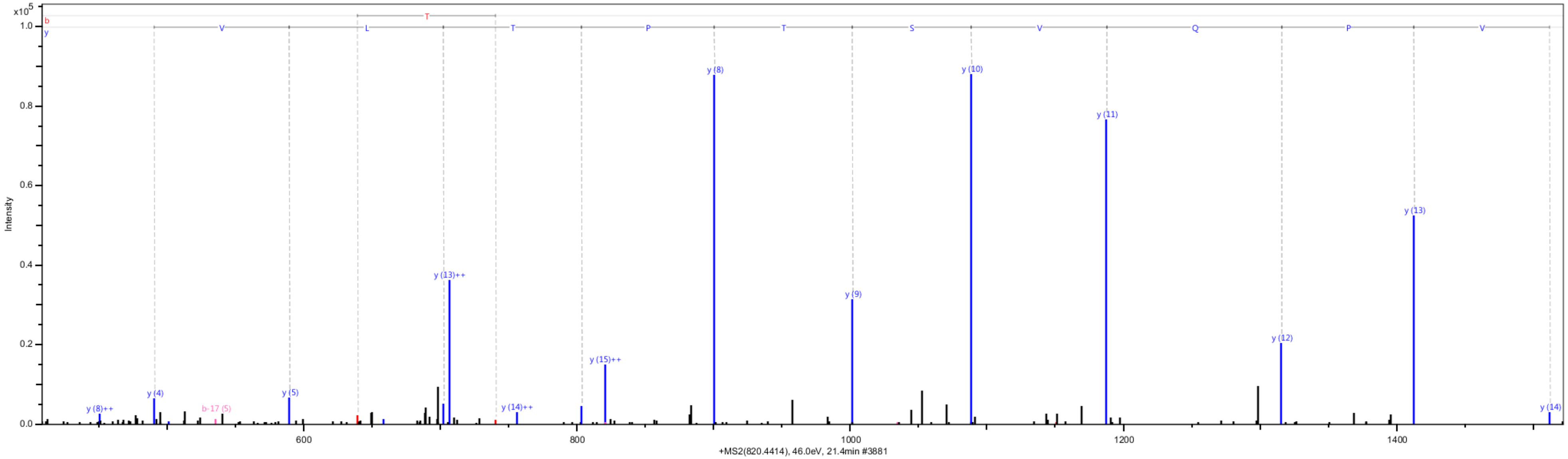

Peptide Match

**Sequence:**	K.VPQVSTPTLVEVSR.N	**Score:**	114
**Modifications:**			

Spectrum Report
Combine Search_2020-10-19 07:53:31

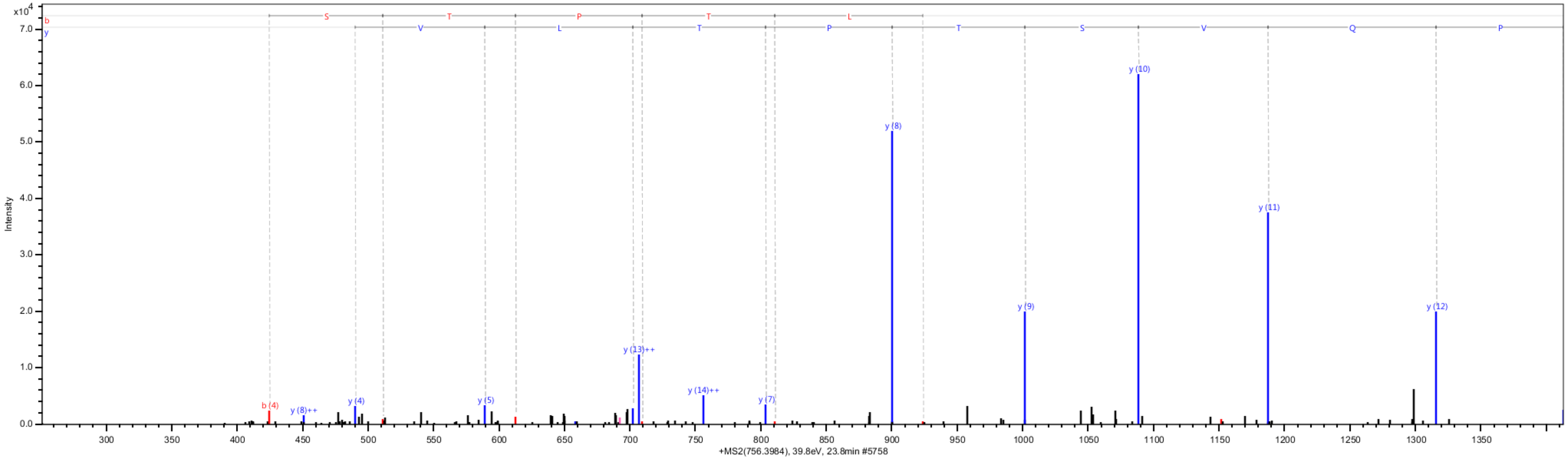

Peptide Match

**Sequence:**	K.VPQVSTPTLVEVSRNLGK.V	**Score:**	27.2
**Modifications:**			

Spectrum Report
Combine Search_2020-10-19 07:53:31

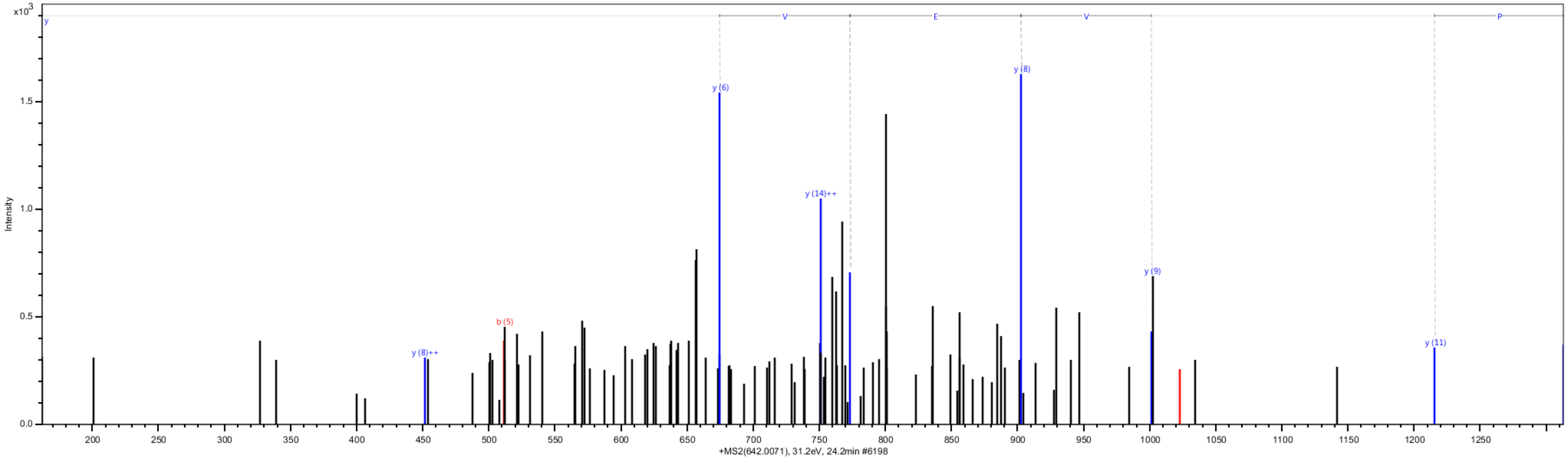

Peptide Match

**Sequence:**	K.RMPCAEDYLSVVLNQLCVLHEK.T	**Score:**	46.8
**Modifications:**	Carbamidomethyl: 4, 17; Oxidation: 2		

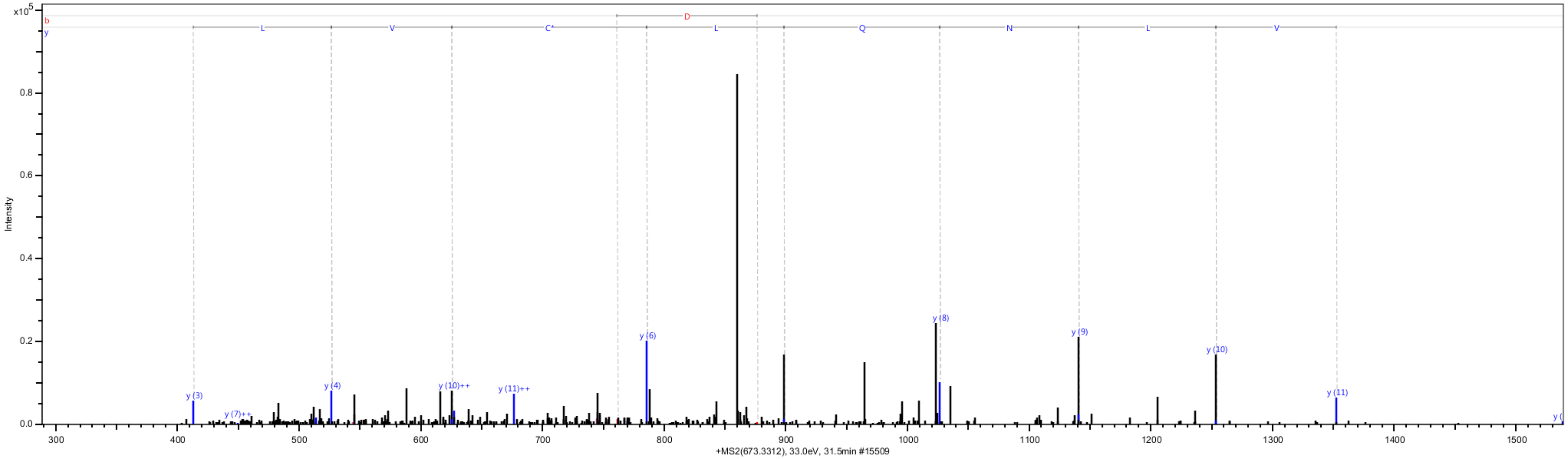

Peptide Match

**Sequence:**	K.TPVSDRVTK.C	**Score:**	33.8
**Modifications:**			

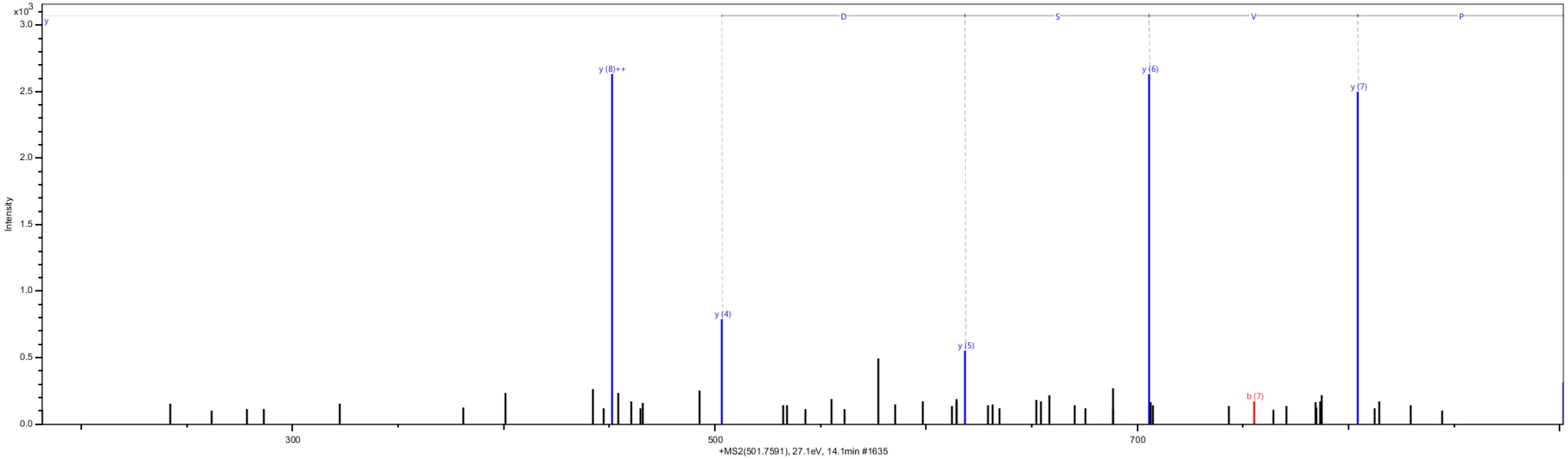

Peptide Match

**Sequence:**	K.CCTESLVNR.R	**Score:**	50.9
**Modifications:**	Carbamidomethyl: 1, 2		

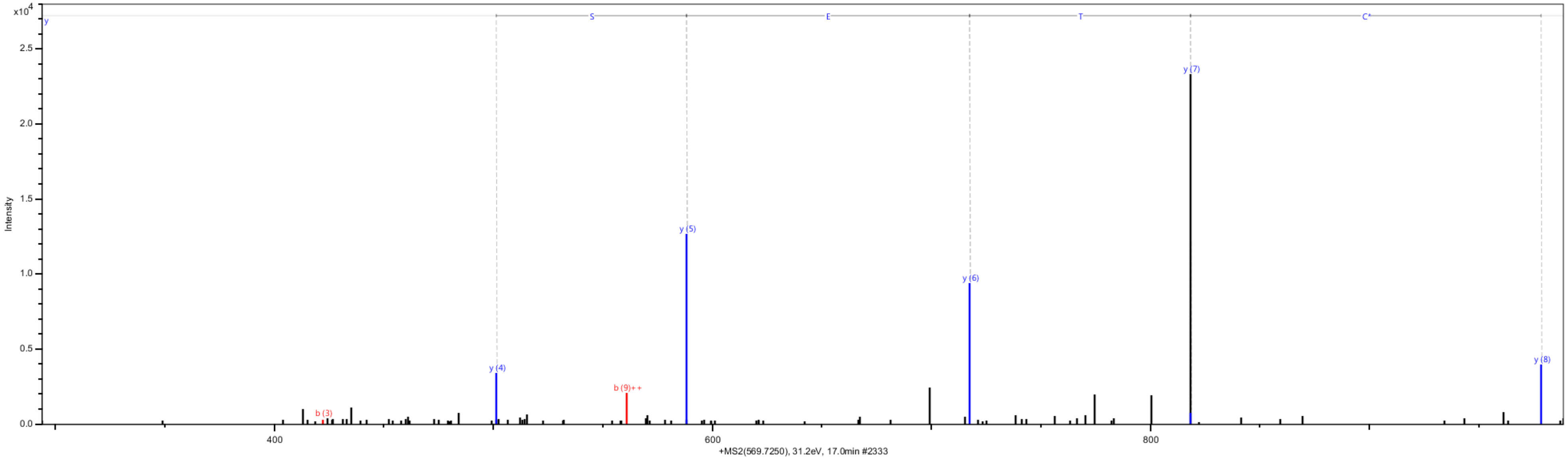

Peptide Match

**Sequence:**	R.RPCFSALEVDETYVPK.E	**Score:**	88.7
**Modifications:**	Carbamidomethyl: 3		

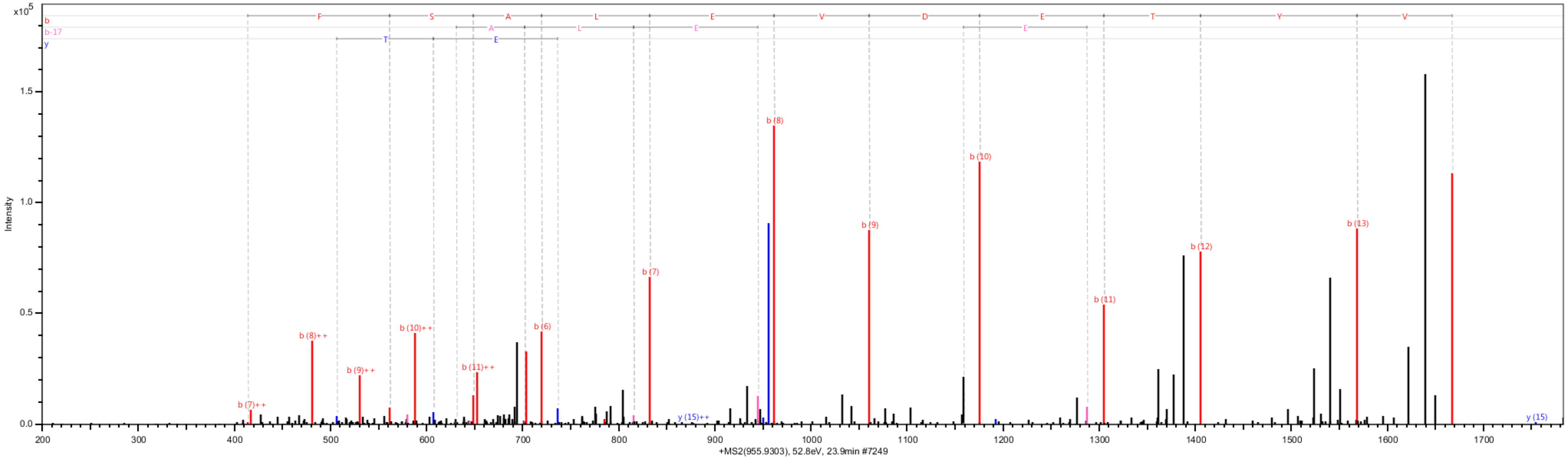

Peptide Match

Sequence: K.KQTALVELVK.H **Score:** 65.1

Modifications:

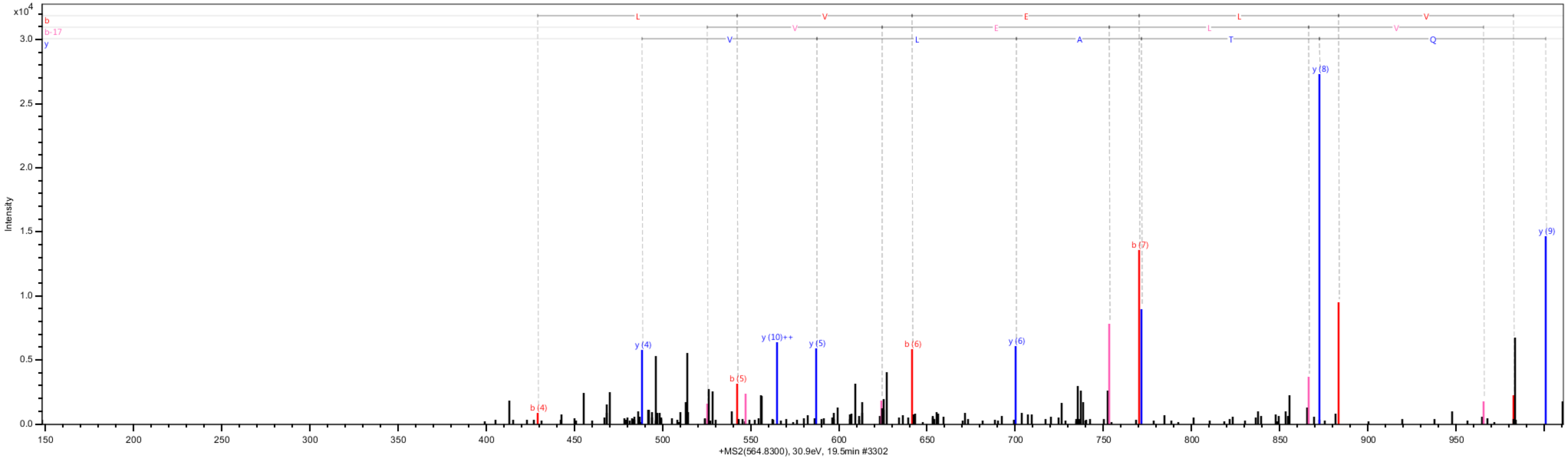

Peptide Match

**Sequence:**	K.QTALVELVK.H	**Score:**	51.3
**Modifications:**			

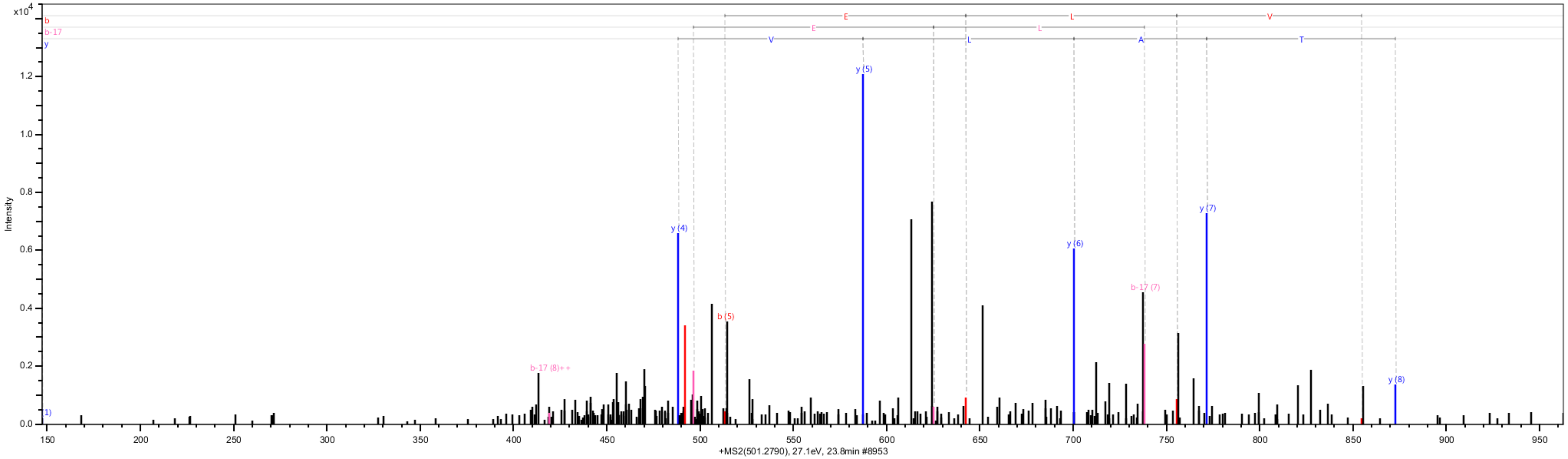

Peptide Match

Sequence: K.QTALVELVKHKPK.A **Score:** 59.4

Modifications:

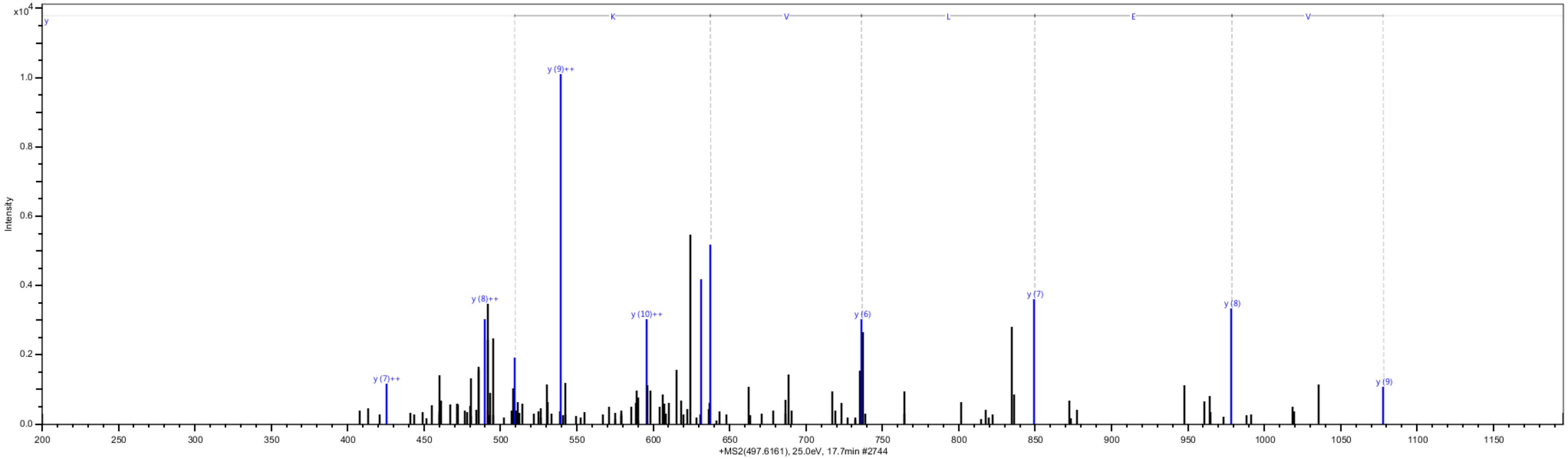

Peptide Match

Sequence: K.EQLKAVMDDFAAFVEK.C **Score:** 49.9

Modifications:

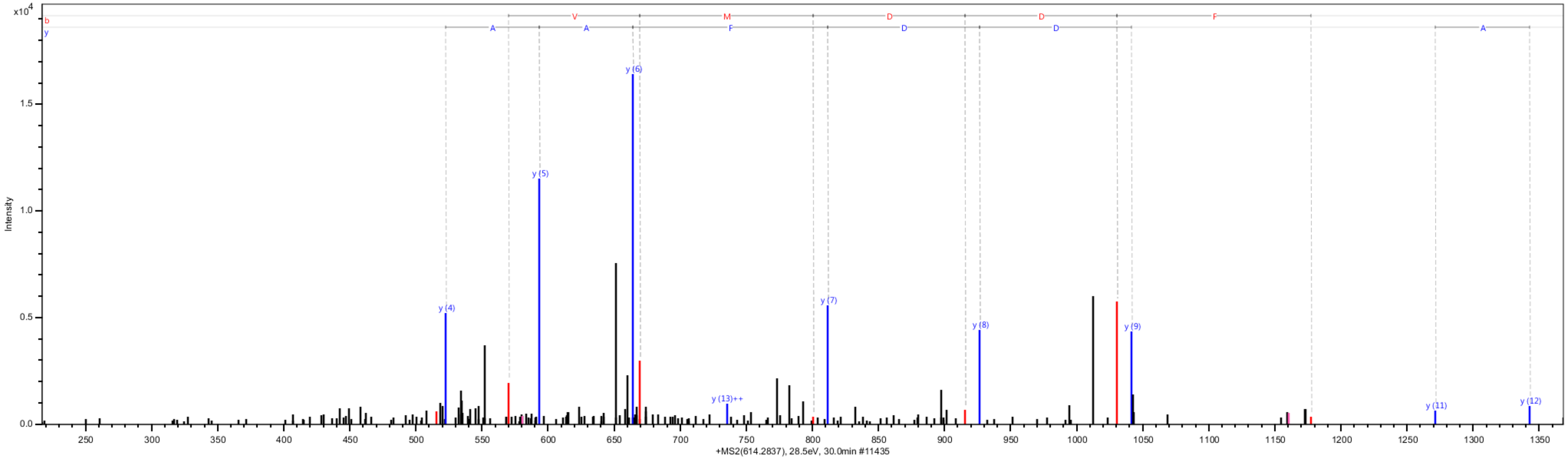

Peptide Match

**Sequence:**	K.AVMDDFAAFVEK.C	**Score:**	53.5
**Modifications:**	Oxidation: 3		

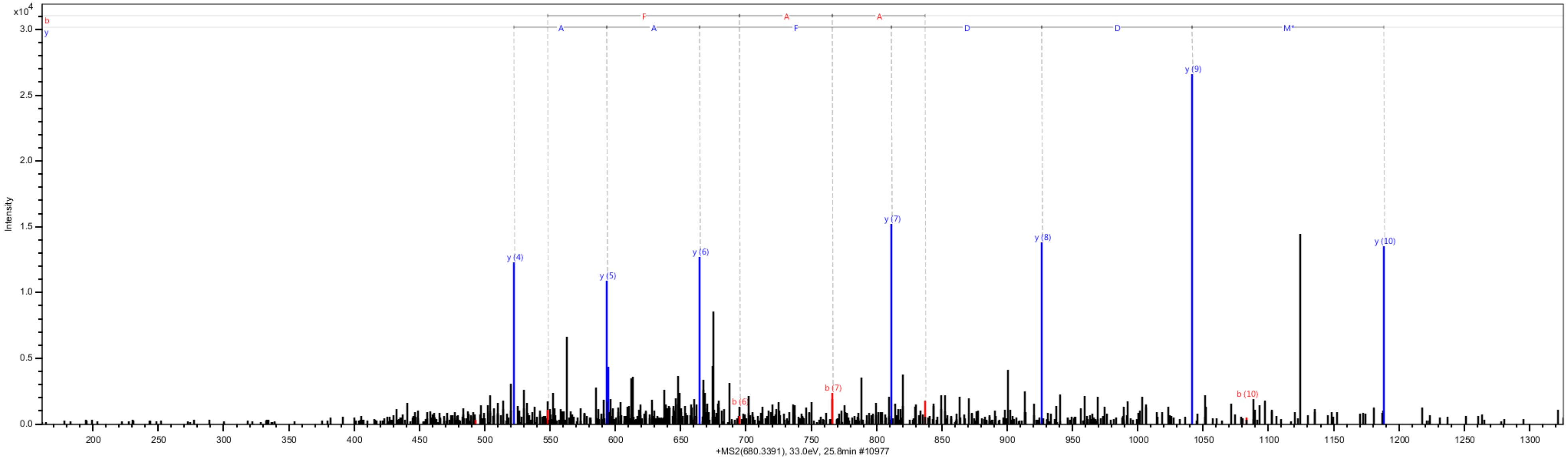

Peptide Match

Sequence: K.AVMDDFAAFVEK.C **Score:** 66.8

Modifications:

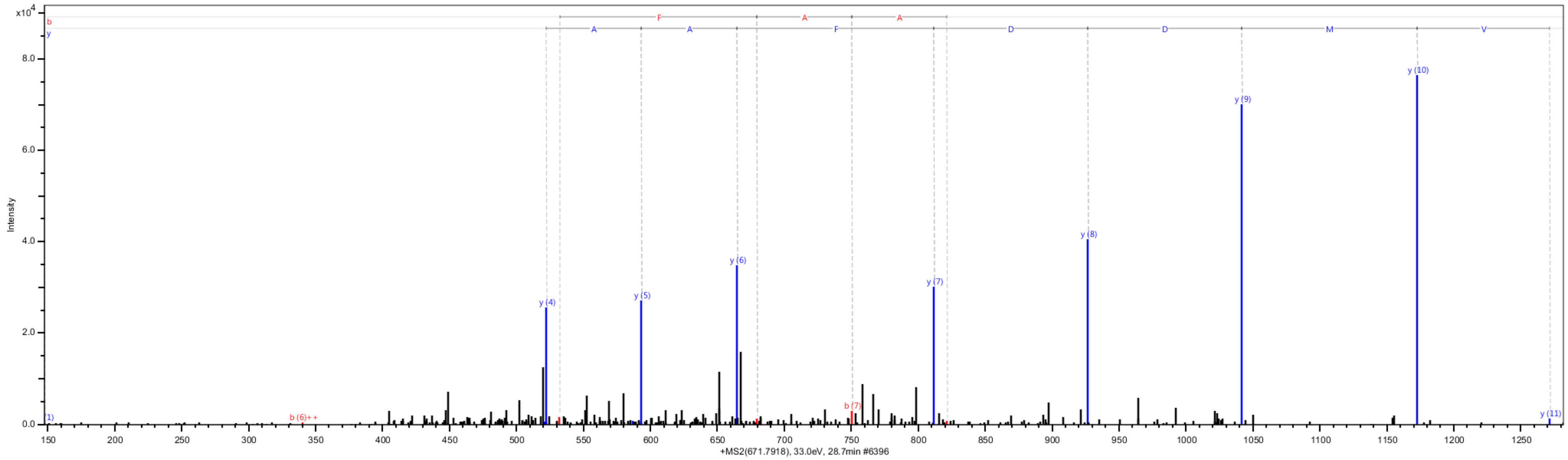

Peptide Match

**Sequence:**	K.AVMDDFAAFVEKCCK.A	**Score:**	73.2
**Modifications:**	Carbamidomethyl: 13, 14		

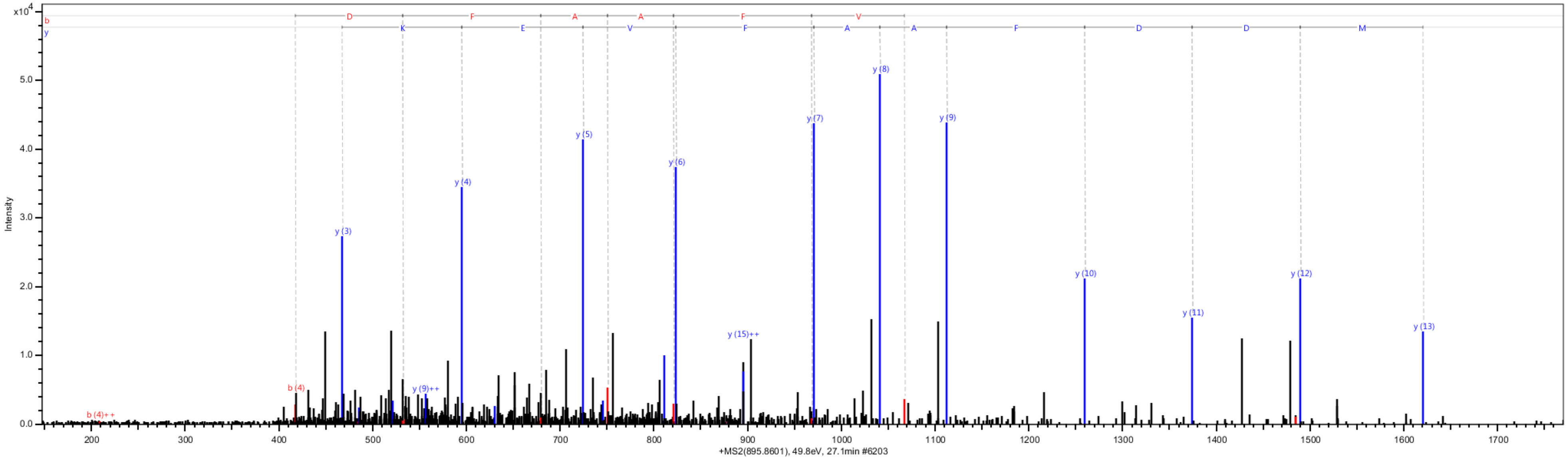

Peptide Match

Sequence: K.KLVAASQAALGL.- **Score:** 74

Modifications:

Spectrum Report
Combine Search_2020-10-19 07:53:31

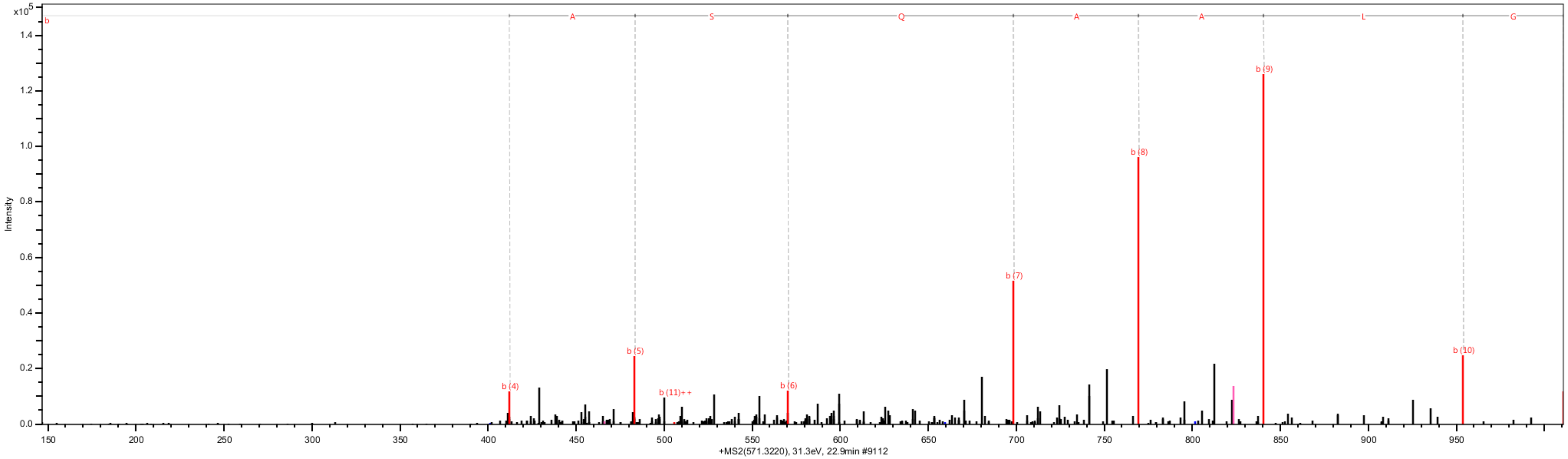

Peptide Match

**Sequence:**	K.LVAASQAALGL.-	**Score:**	41.3
**Modifications:**			

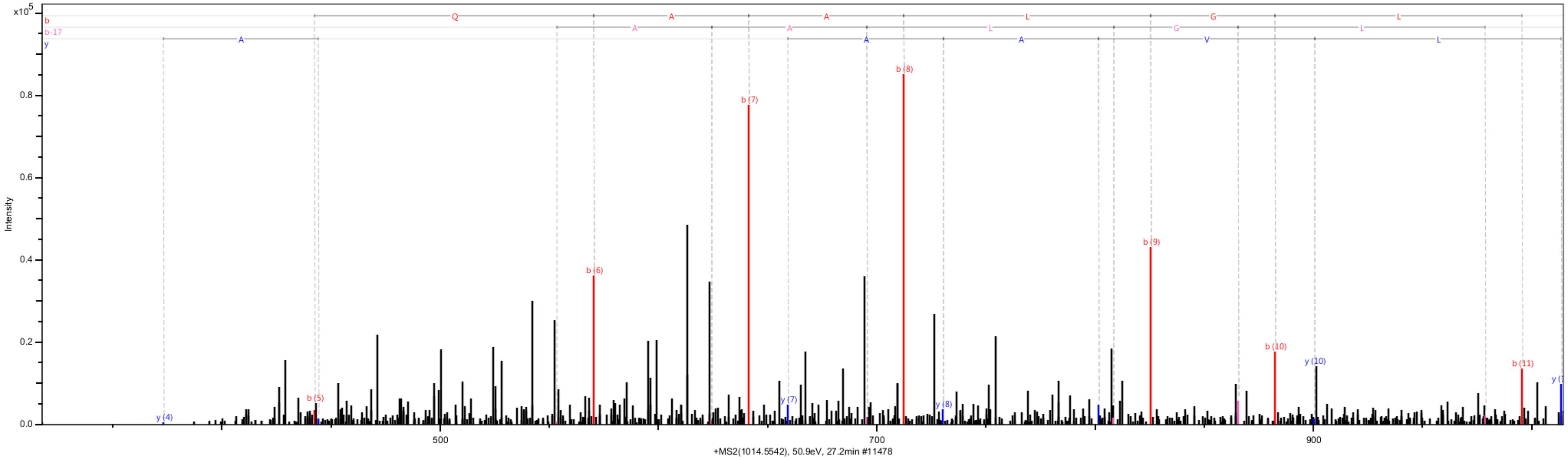
x10^5
Intensity
0.0
0.2
0.4
0.6
0.8
500
700
900
+MS2(1014.5542), 50.9eV, 27.2min #11478
b
b-17
y
b (5)
b (6)
b (7)
b (8)
b (9)
b (10)
b (11)
y (4)
y (7)
y (8)
y (10)

A1AT_HUMAN

Accession: A1AT_HUMAN
Database: SwissProt
MW [kDa] / pI: 46.7 / 5.4
Score: 621.5
Seq. Coverage [%]: 35.9
No. of Peptides: 14

Peptide Match

Sequence: K.ITPNLAEFAFSLYR.Q
Score: 126.4
Modifications:

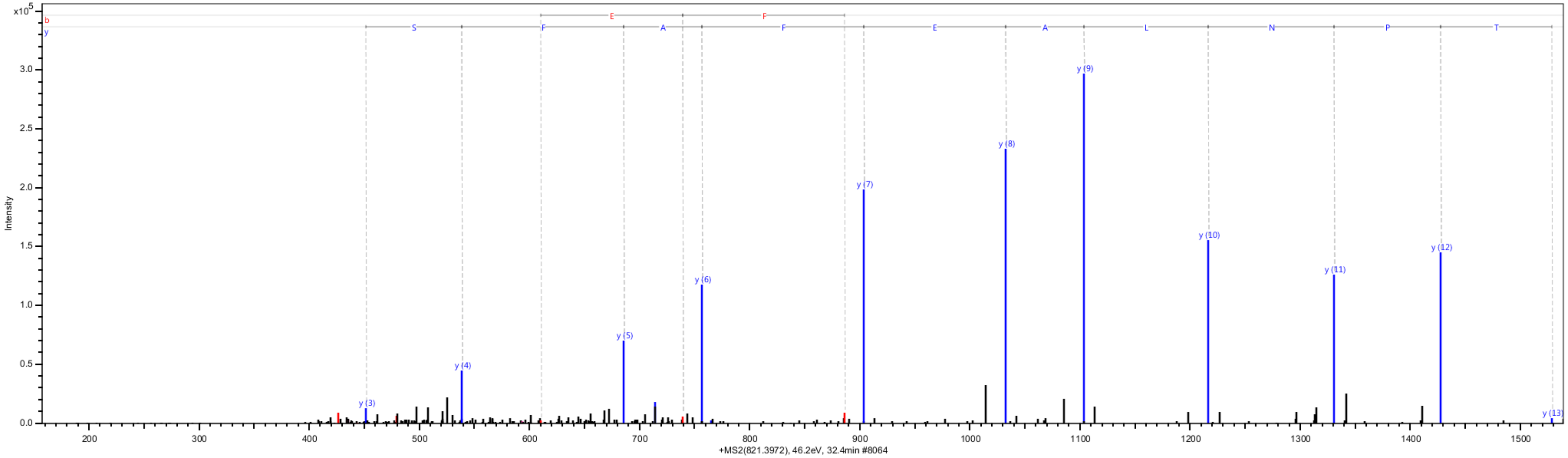

Peptide Match

Sequence: K.LVDKFLEDVK.K
Score: 26.1
Modifications:

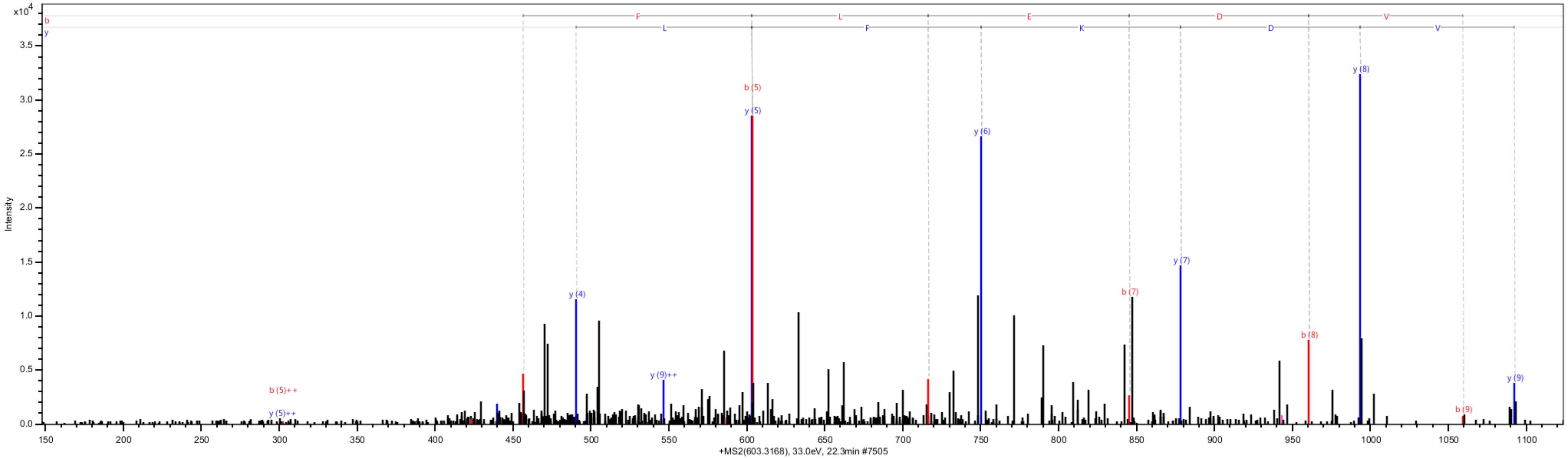

Peptide Match

Sequence: K.QINDYVEKGTQGK.I **Score:** 56.6

Modifications:

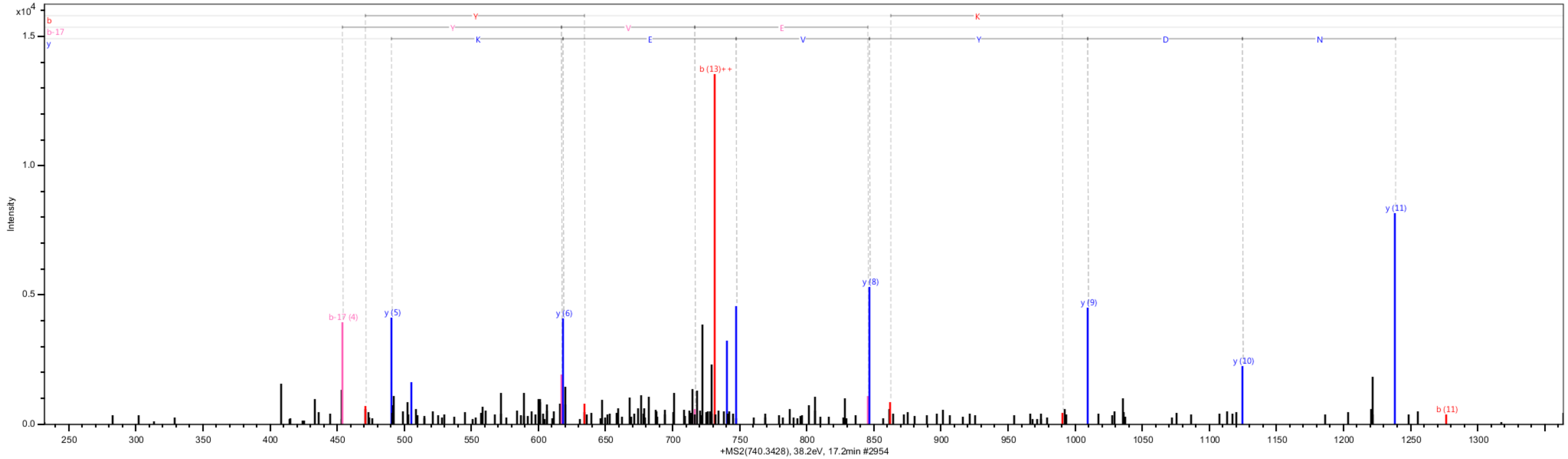

Peptide Match

Sequence: R.DTVFALVNYIFFK.G **Score:** 36.9

Modifications:

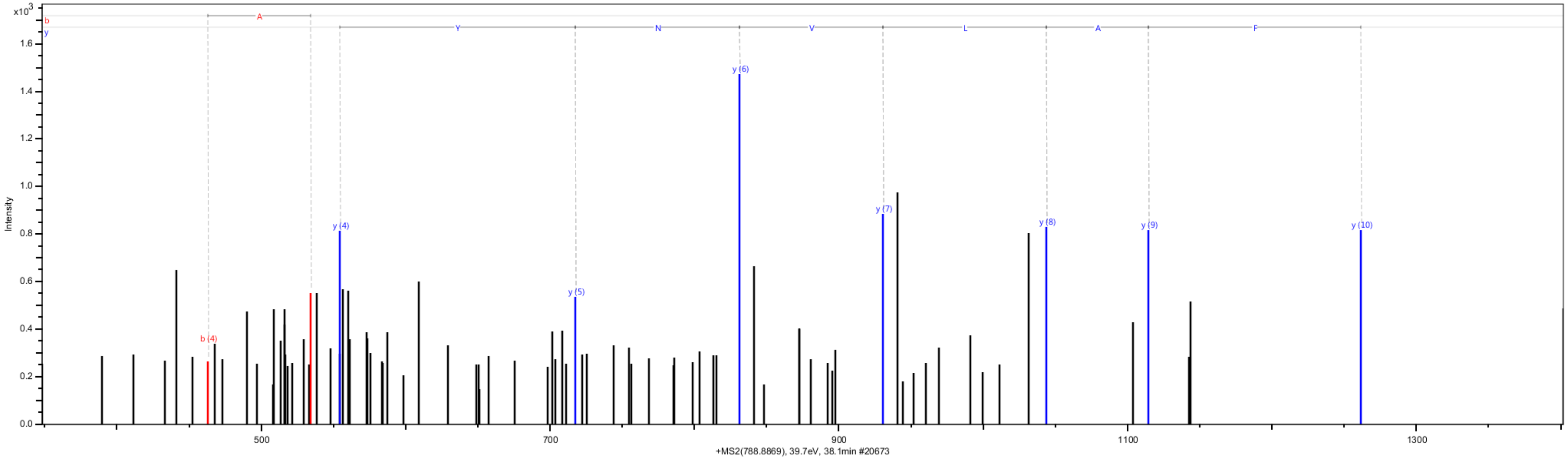

Peptide Match

**Sequence:**	K.RLGMFNIQHCK.K	**Score:**	29.1
**Modifications:**	Carbamidomethyl: 10		

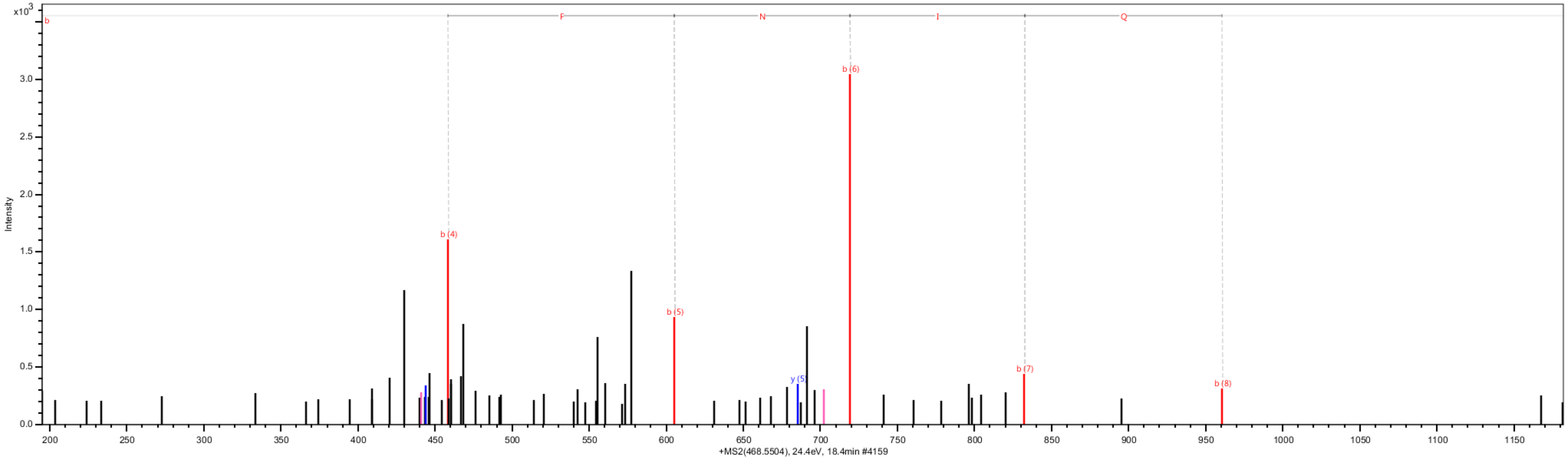

Peptide Match

**Sequence:**	R.LGMFNIQHCK.K	**Score:**	29.4
**Modifications:**	Carbamidomethyl: 9		

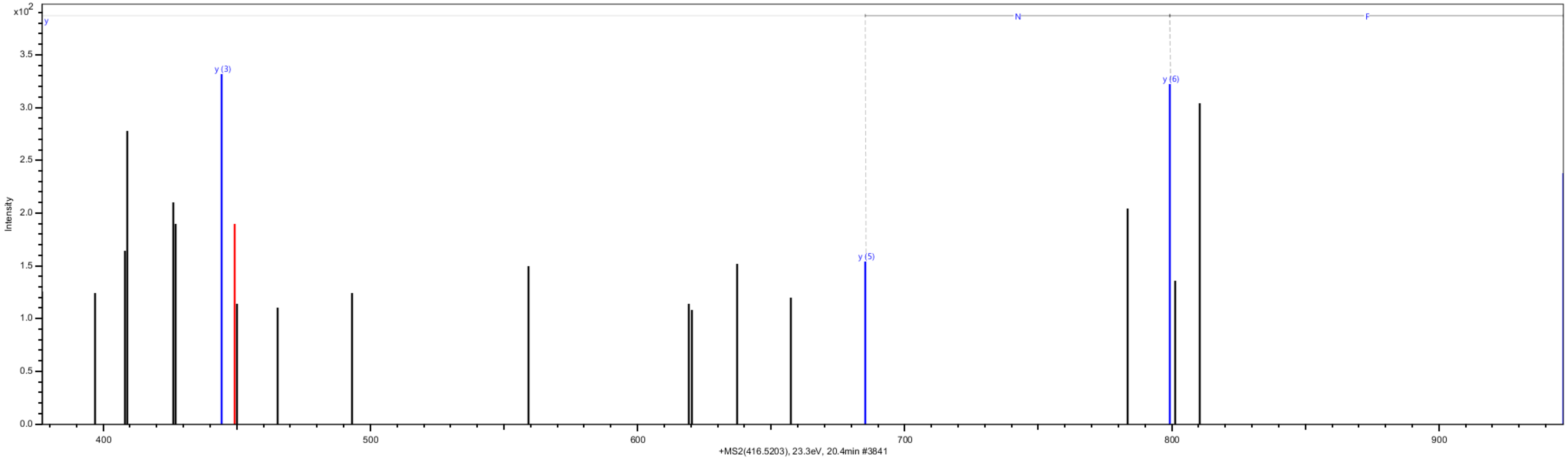

Peptide Match

Sequence: K.KLSSWVLLMK.Y **Score:** 37.8

Modifications:

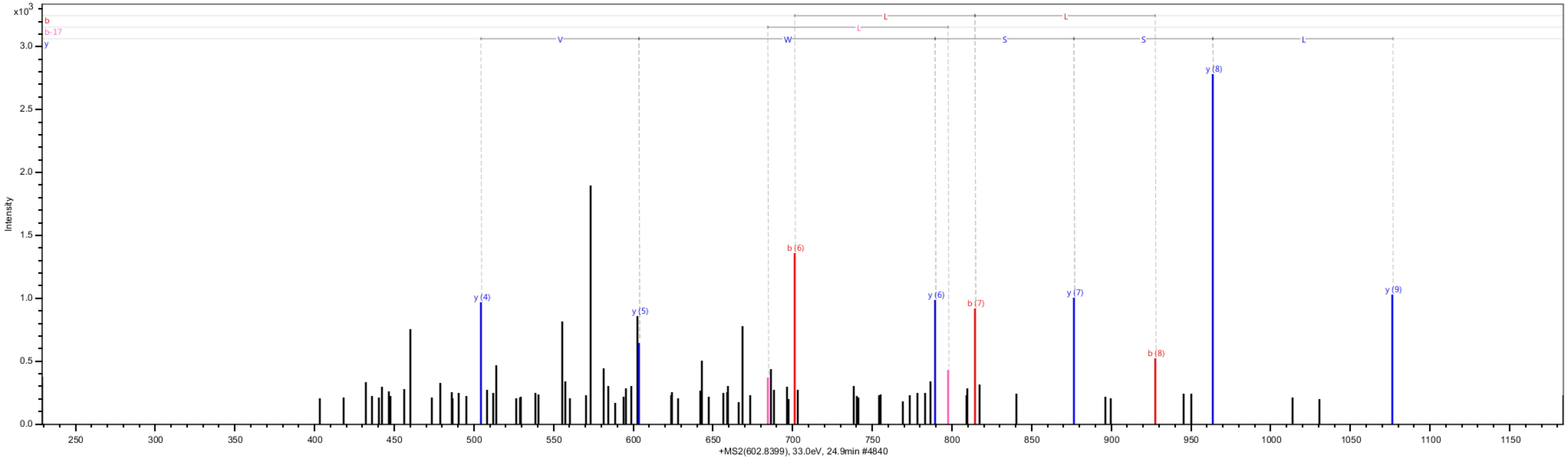

Peptide Match

Sequence: K.LSSWVLLMK.Y **Score:** 44.3

Modifications:

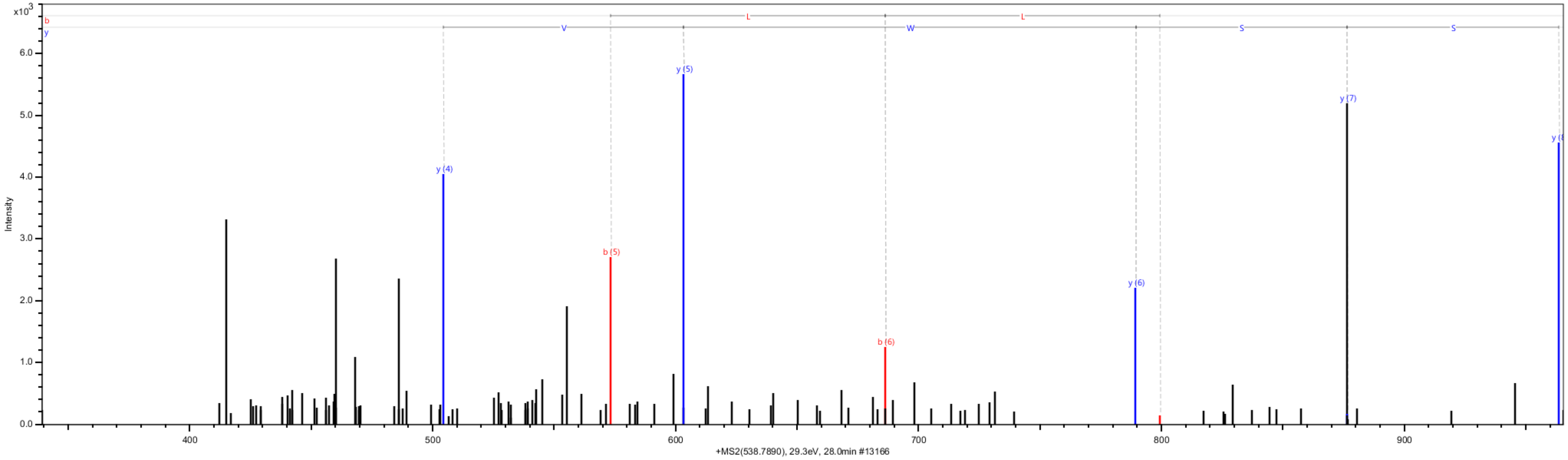

Peptide Match

**Sequence:**	K.LQHLENELTHDIITK.F	**Score:**	49
**Modifications:**			

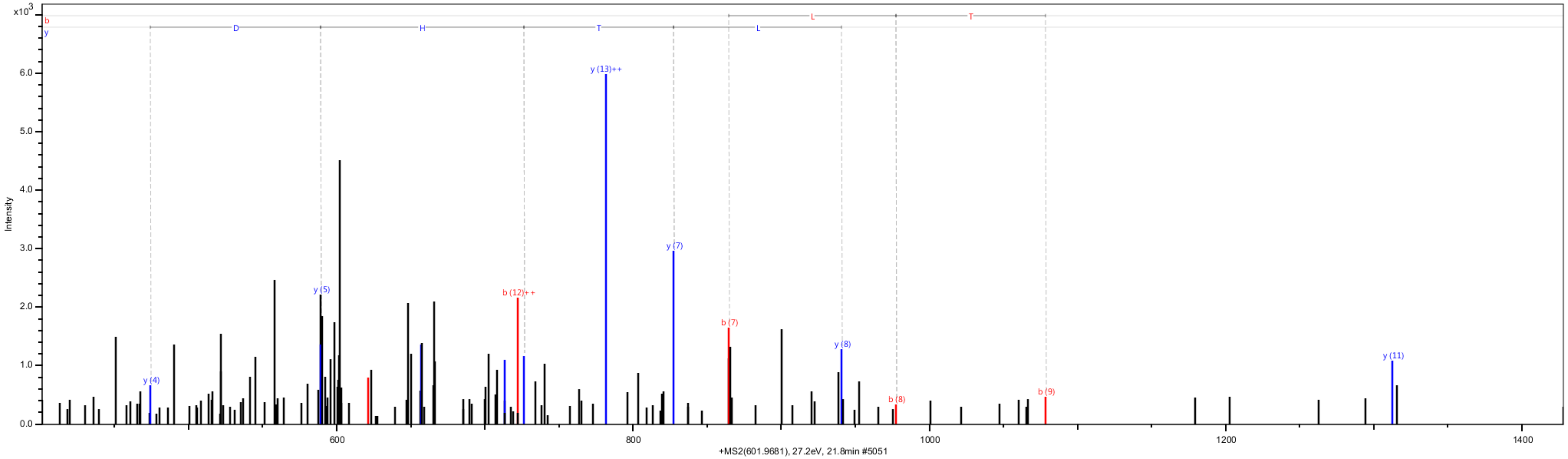

Peptide Match

Sequence: K.LSITGTYDLK.S **Score:** 53.1

Modifications:

Spectrum Report
Combine Search_2020-10-19 07:53:31

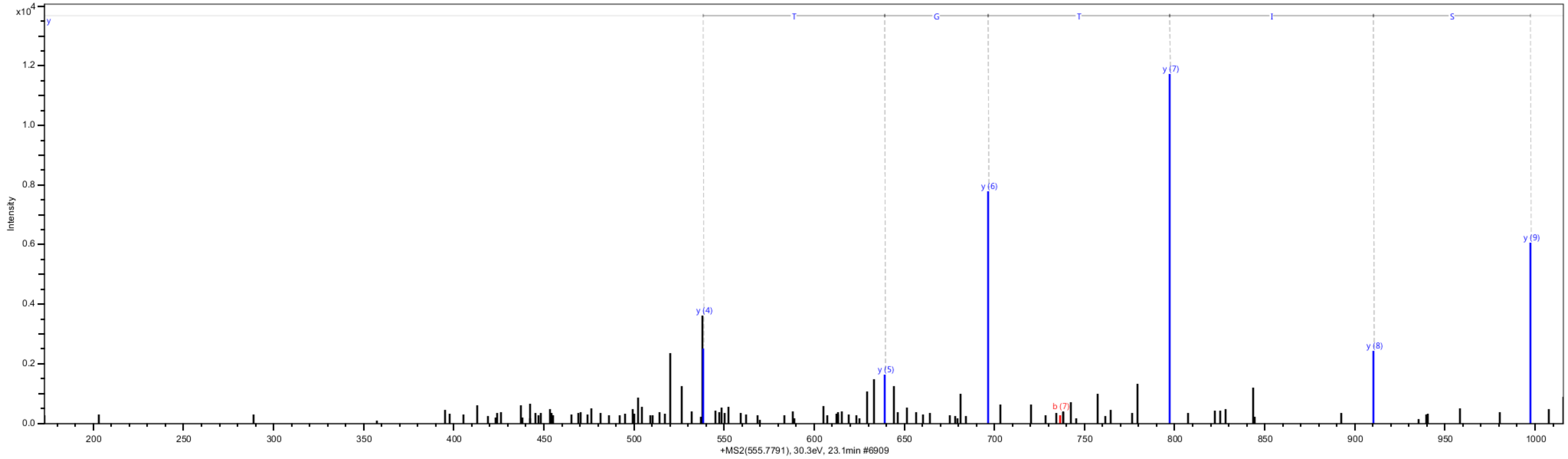

Peptide Match

Sequence: K.SVLGQLGITK.V **Score:** 30
Modifications:

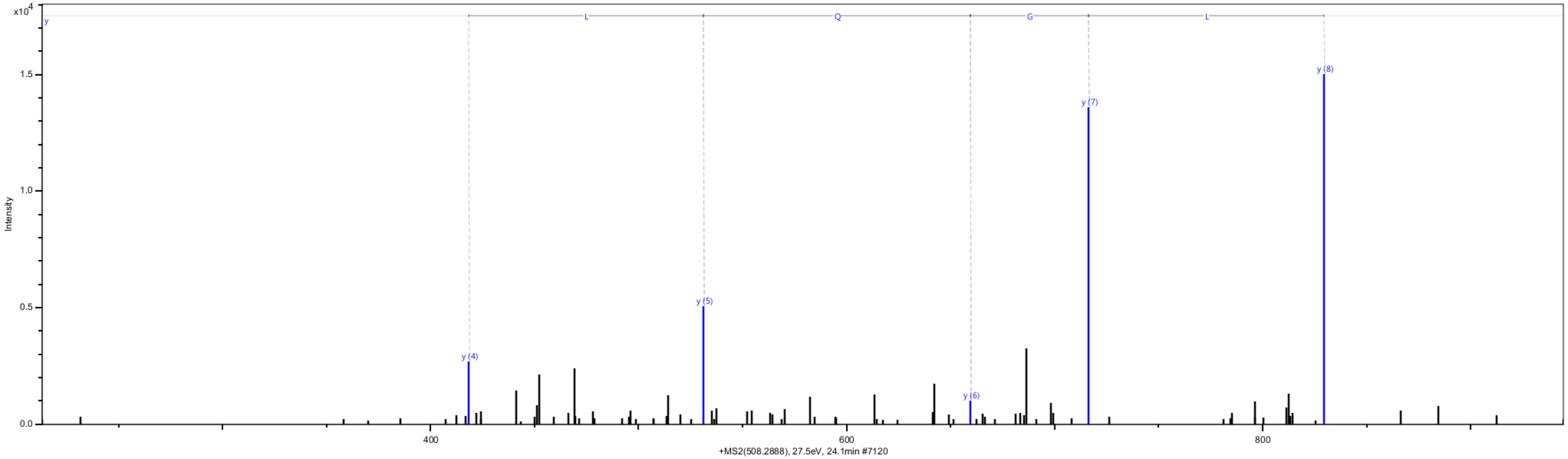

Peptide Match

Sequence: K.VFSNGADLSGVTEEAPLKLSK.A **Score:** 55

Modifications:

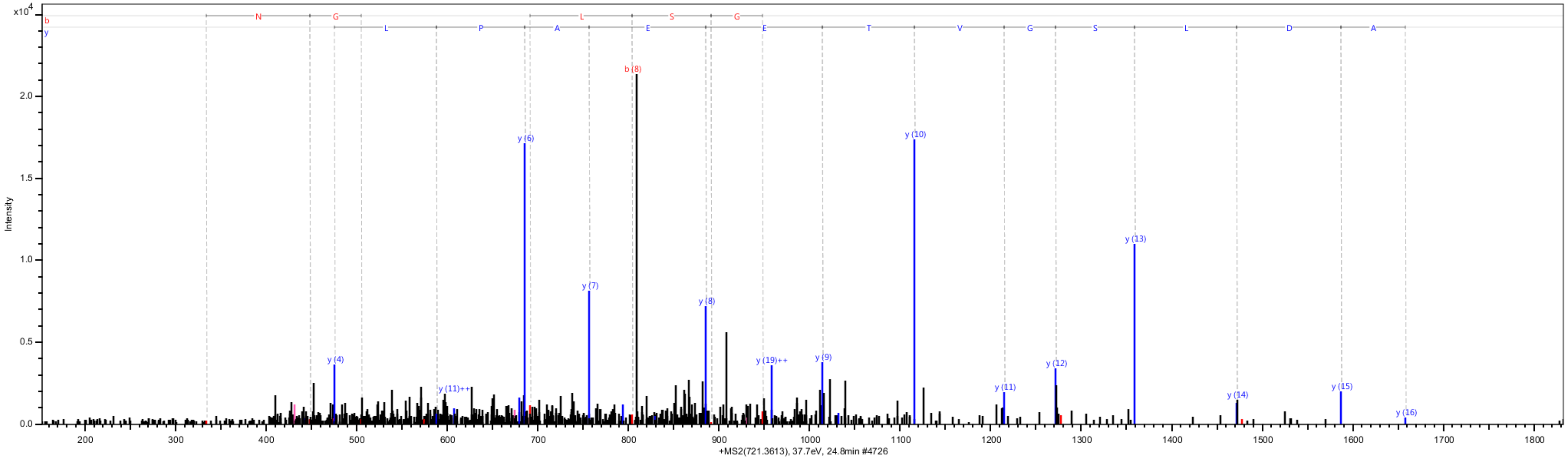

Peptide Match

Sequence: K.AVLTIDEK.G **Score:** 23.8

Modifications:

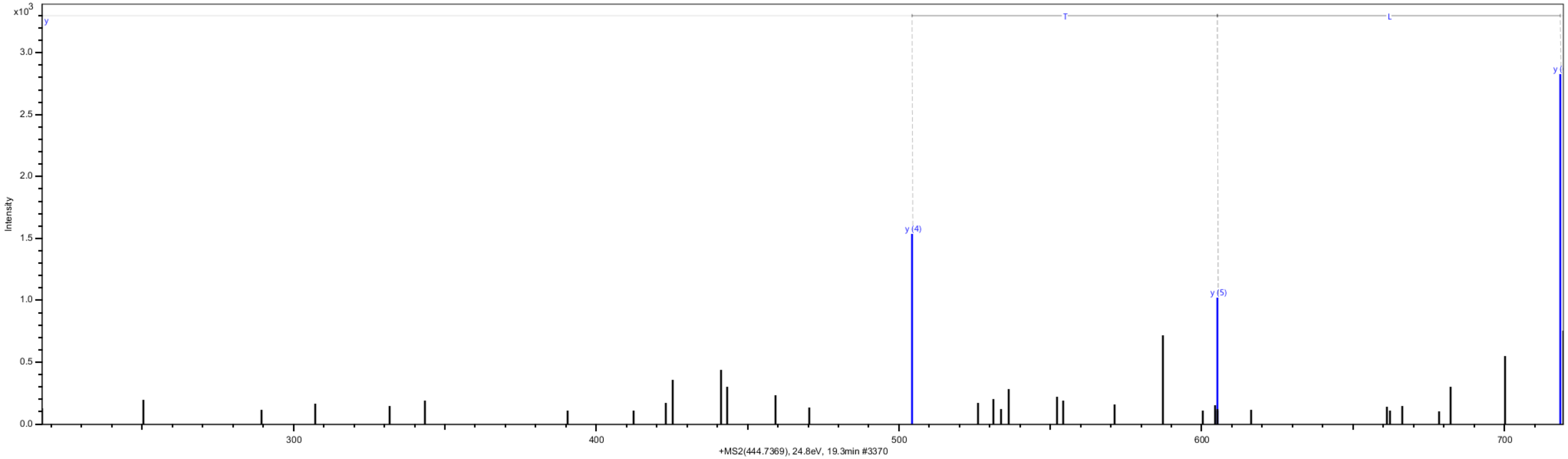

Peptide Match

**Sequence:**	K.FNKPFVFLMIEQNTK.S	**Score:**	23.9
**Modifications:**			

Spectrum Report
Combine Search_2020-10-19 07:53:31

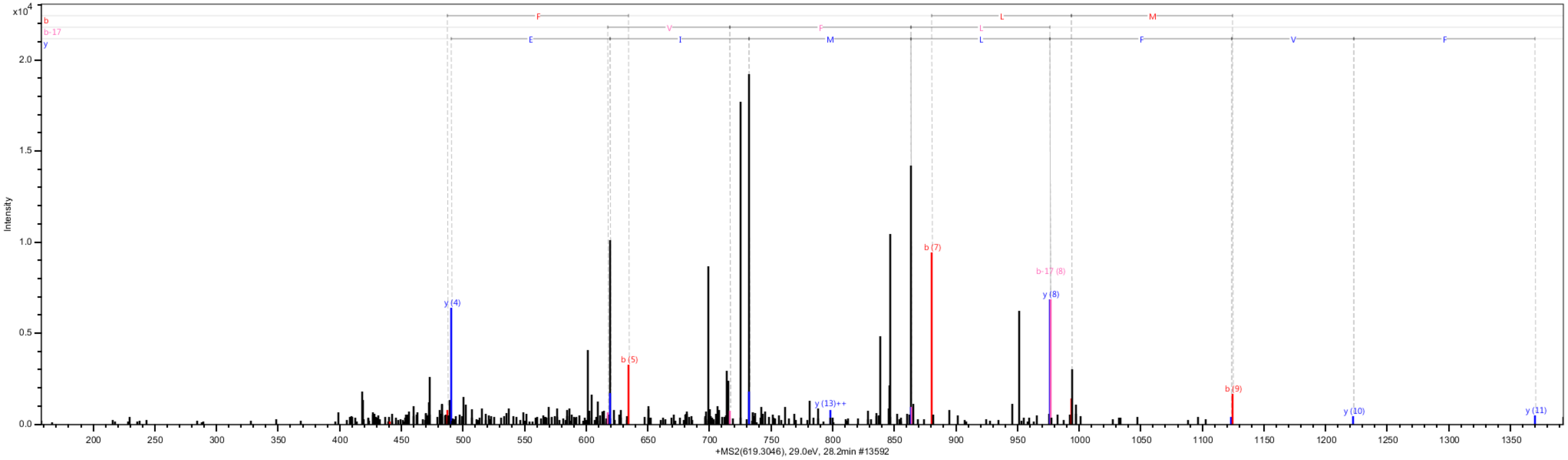

IGHA1_HUMAN

**Accession:**	IGHA1_HUMAN	**Score:**	578.8
**Database:**	SwissProt	**Seq. Coverage [%]:**	29.2
**MW [kDa] / pI:**	37.6 / 6.1	**No. of Peptides:**	10

Peptide Match

**Sequence:**	R.DASGVTFTWTPSSGK.S	**Score:**	72.2
**Modifications:**			

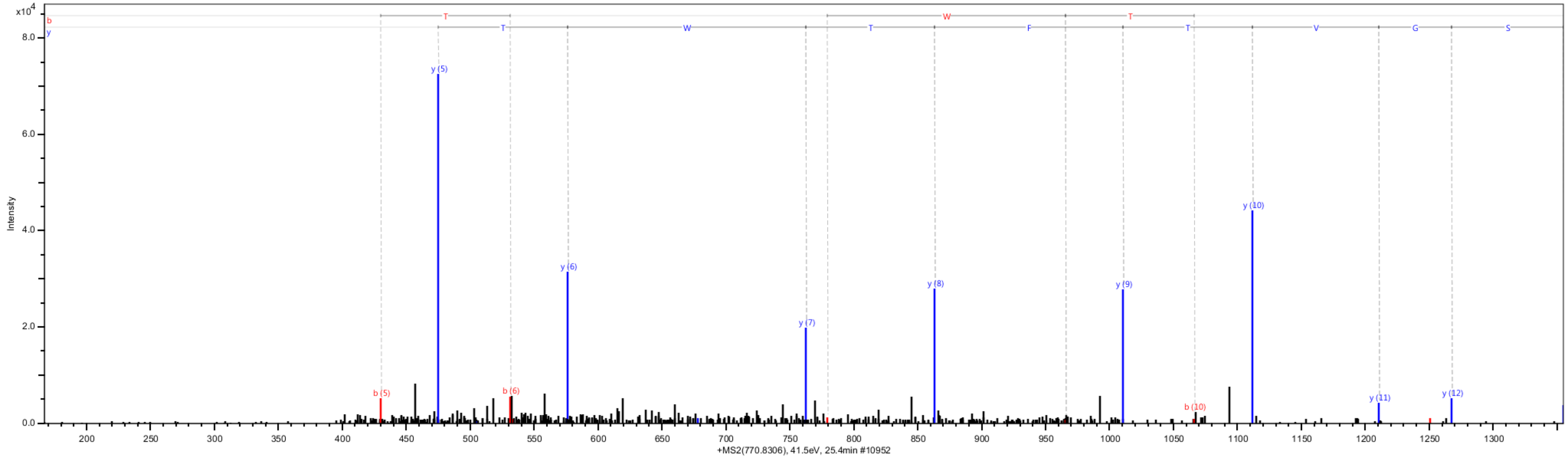

Peptide Match

**Sequence:**	K.TFTCTAAYPESK.T	**Score:**	61.4
**Modifications:**	Carbamidomethyl: 4		

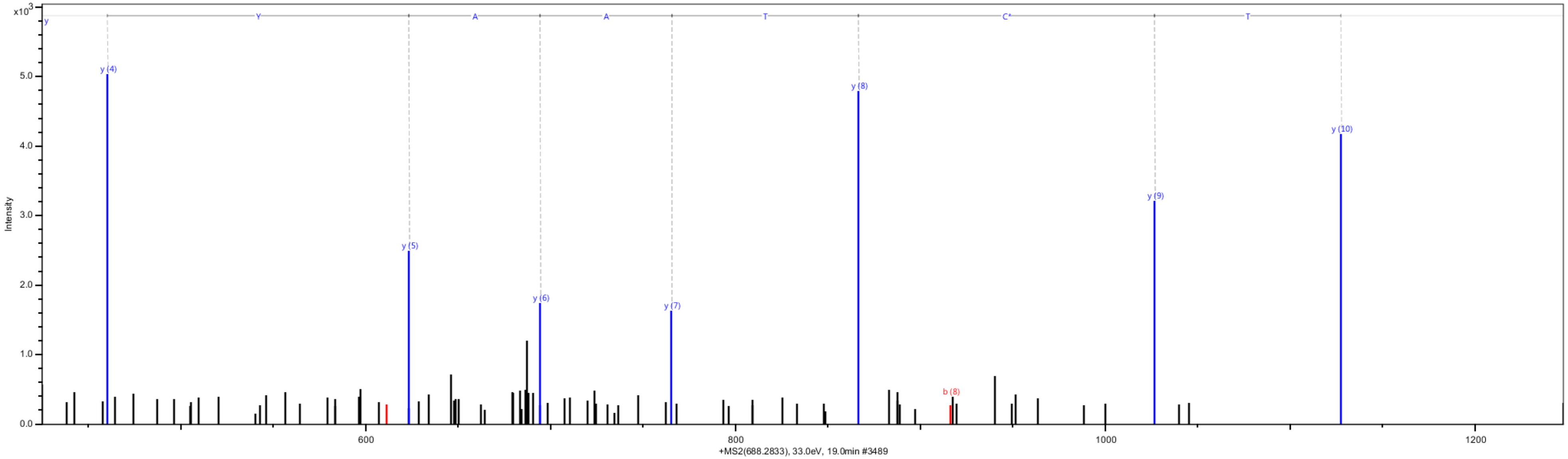

Peptide Match

Sequence: K.TPLTATLSK.S **Score:** 45.3

Modifications:

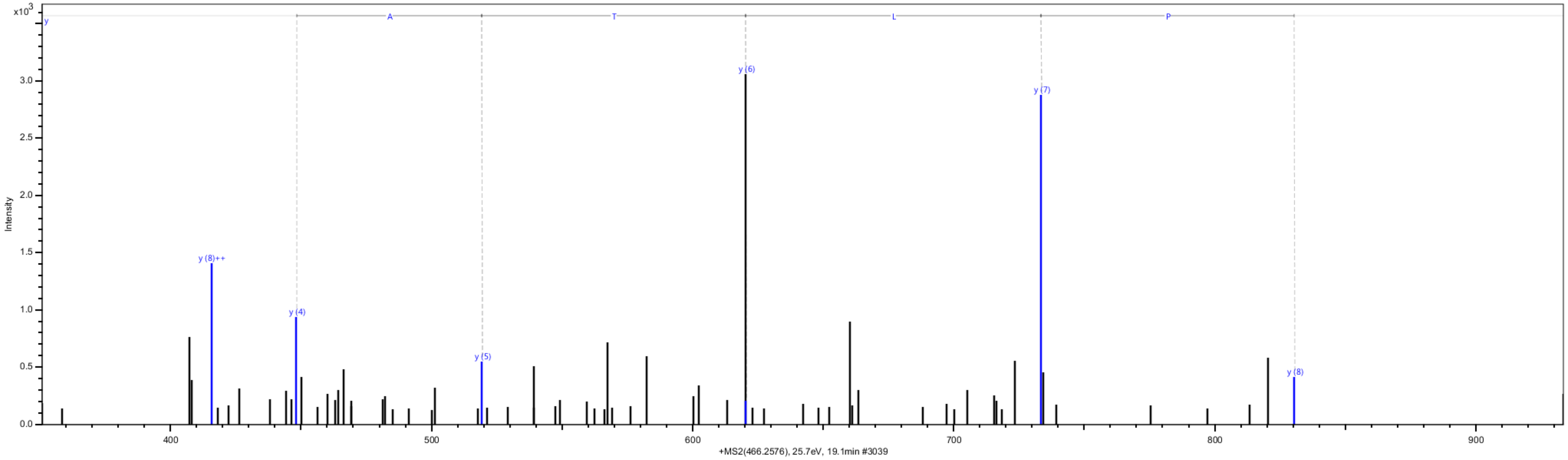

Peptide Match

**Sequence:**	R.GFSPKDVLVR.W	**Score:**	49.6
**Modifications:**			

Spectrum Report
Combine Search_2020-10-19 07:53:31

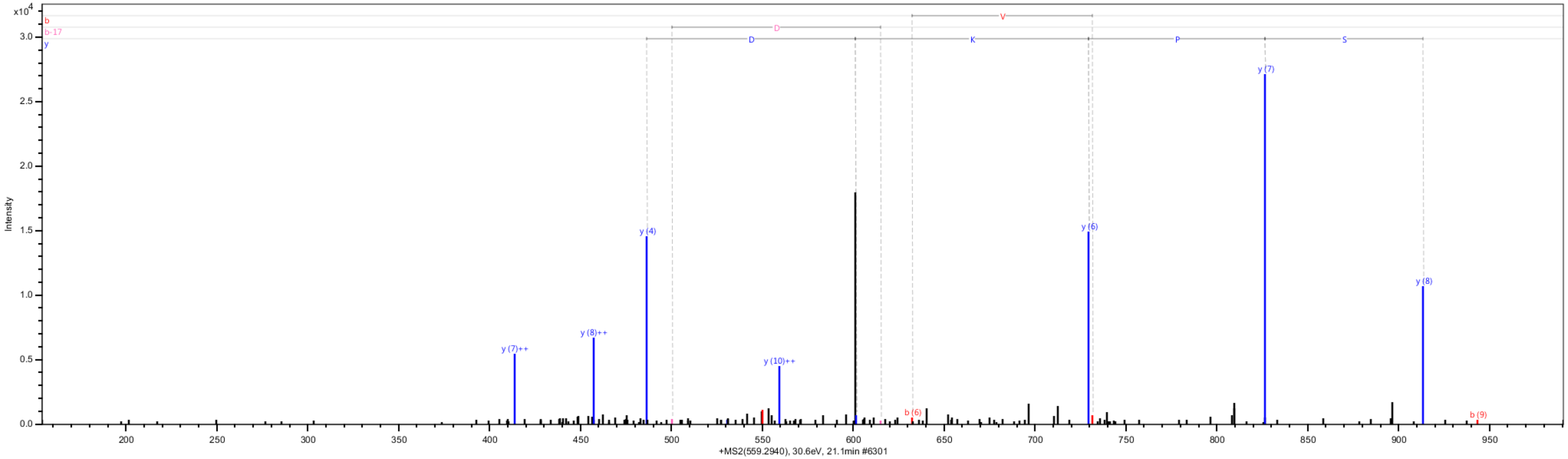

Peptide Match

Sequence: R.WLQGSQELPR.E **Score:** 45.5

Modifications:

Spectrum Report
Combine Search_2020-10-19 07:53:31

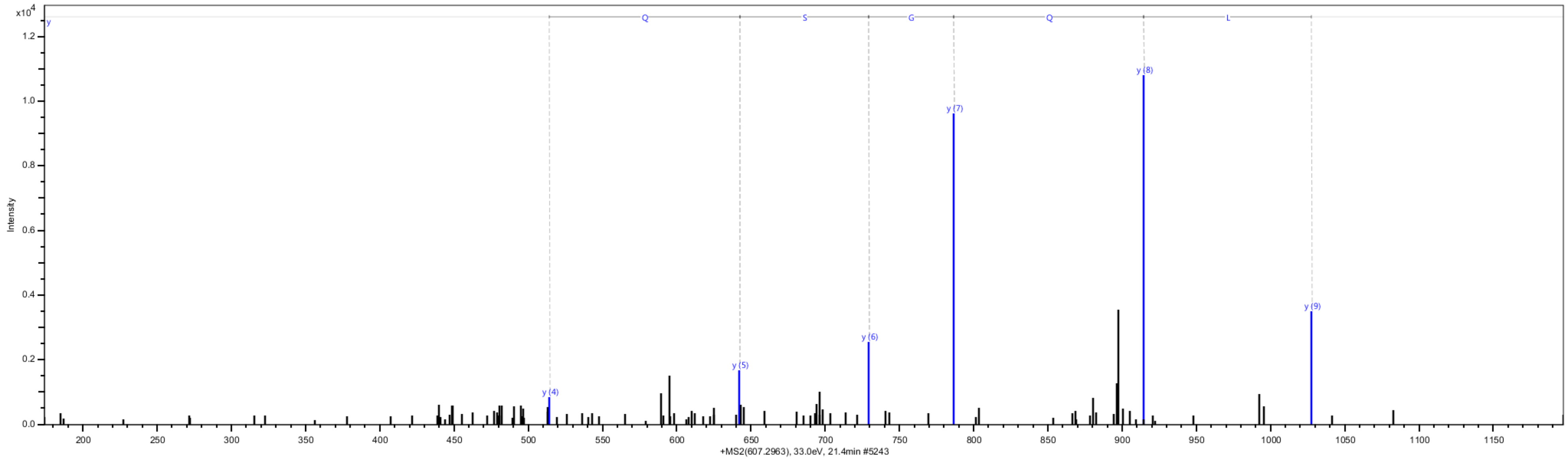

Peptide Match

Sequence: R.WLQGSQELPREK.Y **Score:** 84.2

Modifications:

Spectrum Report
Combine Search_2020-10-19 07:53:31

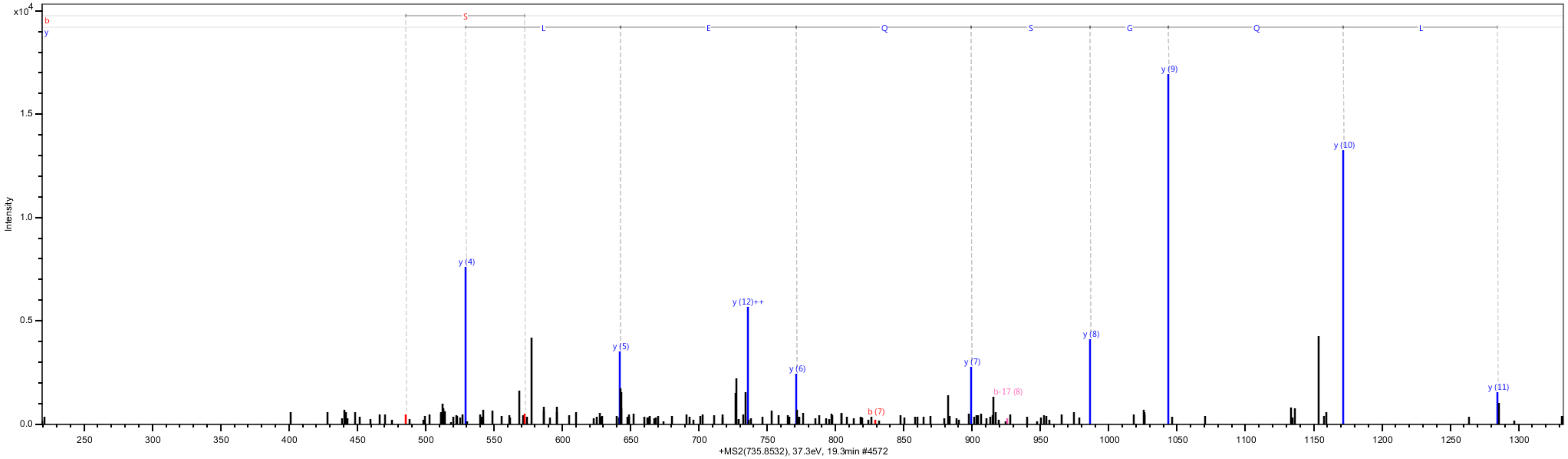

Peptide Match

**Sequence:**	R.EKYLTWASR.Q	**Score:**	37.7
**Modifications:**			

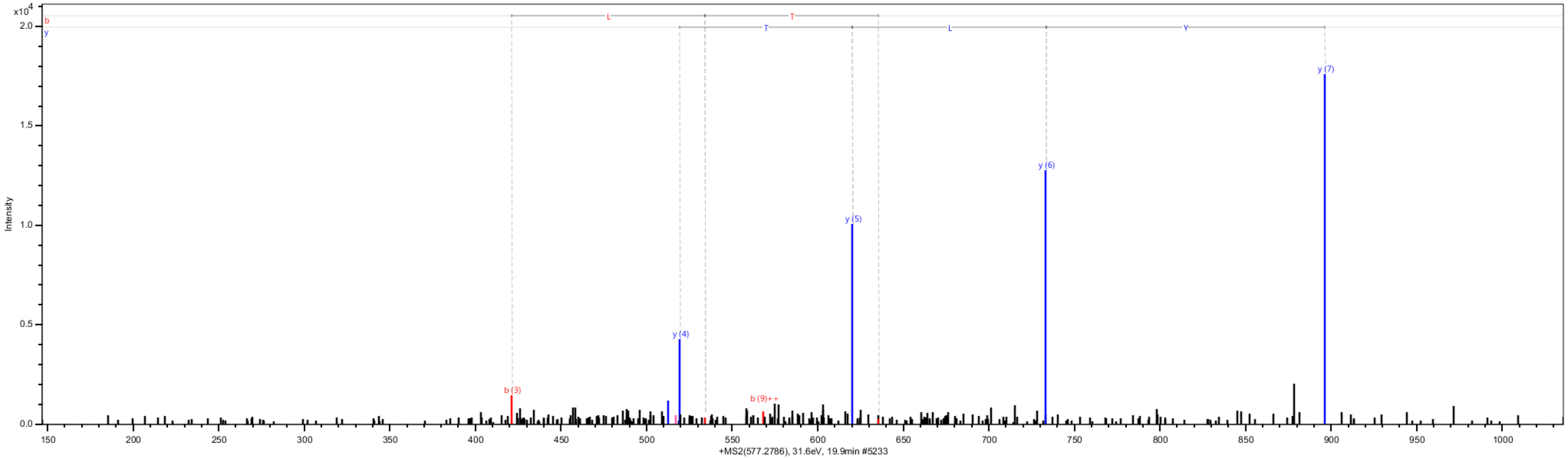

Peptide Match

**Sequence:**	K.YLTWASR.Q	**Score:**	28
**Modifications:**			

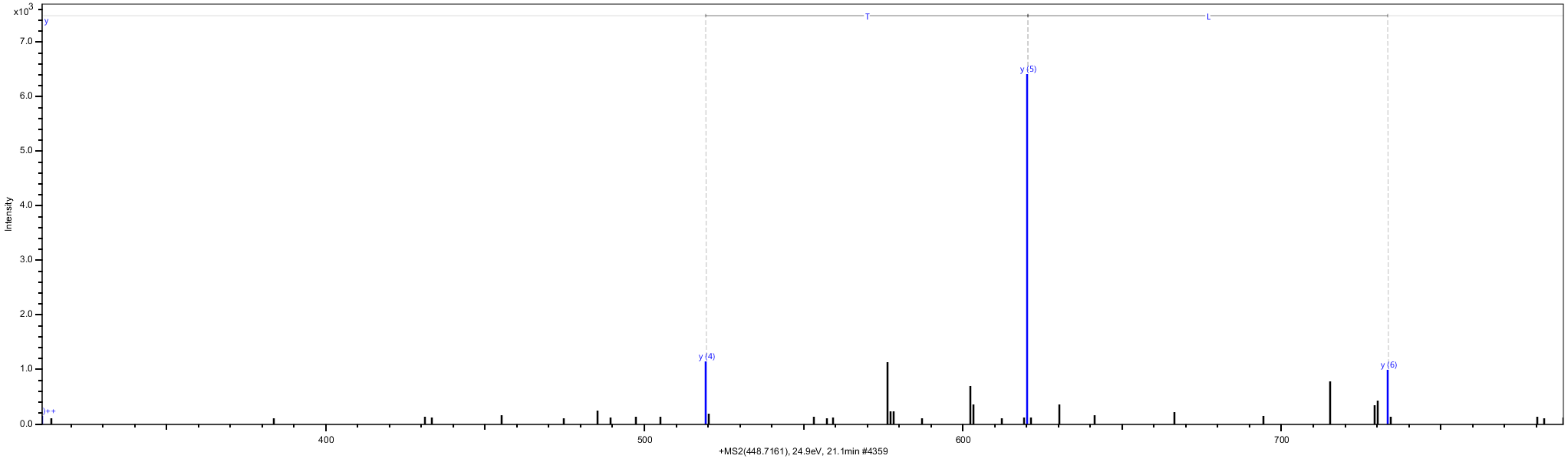

Peptide Match

Sequence: R.QEPSQGTTTFAVTSILR.V **Score:** 112.4

Modifications:

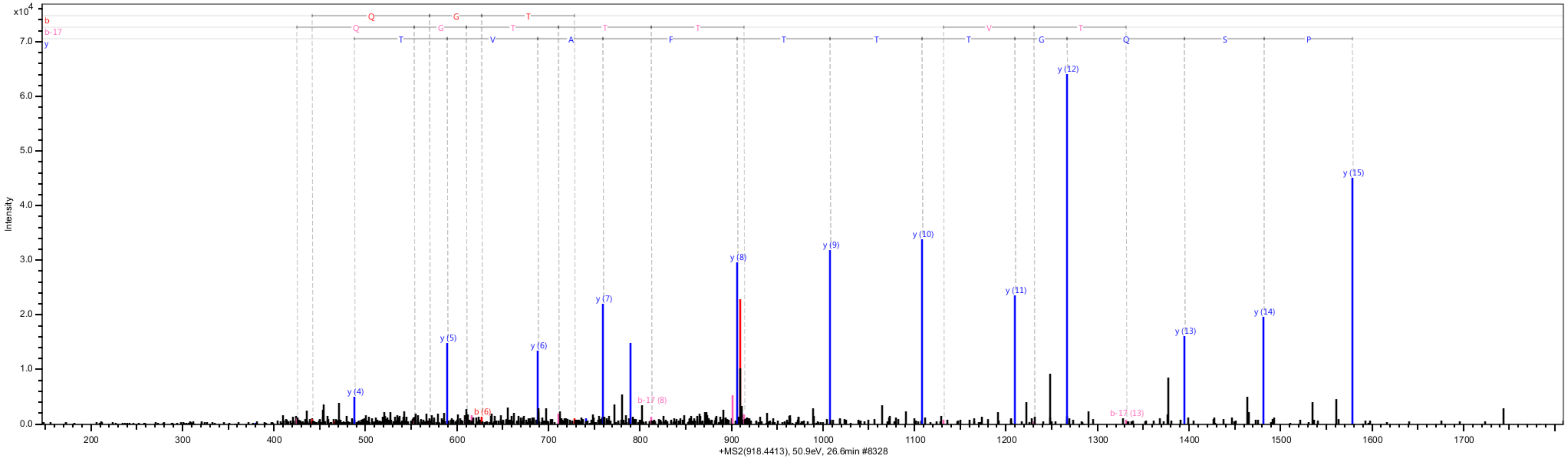

Peptide Match

**Sequence:**	K.KGDTFSCMVGHEALPLAFTQK.T	**Score:**	42.6
**Modifications:**	Carbamidomethyl: 7		

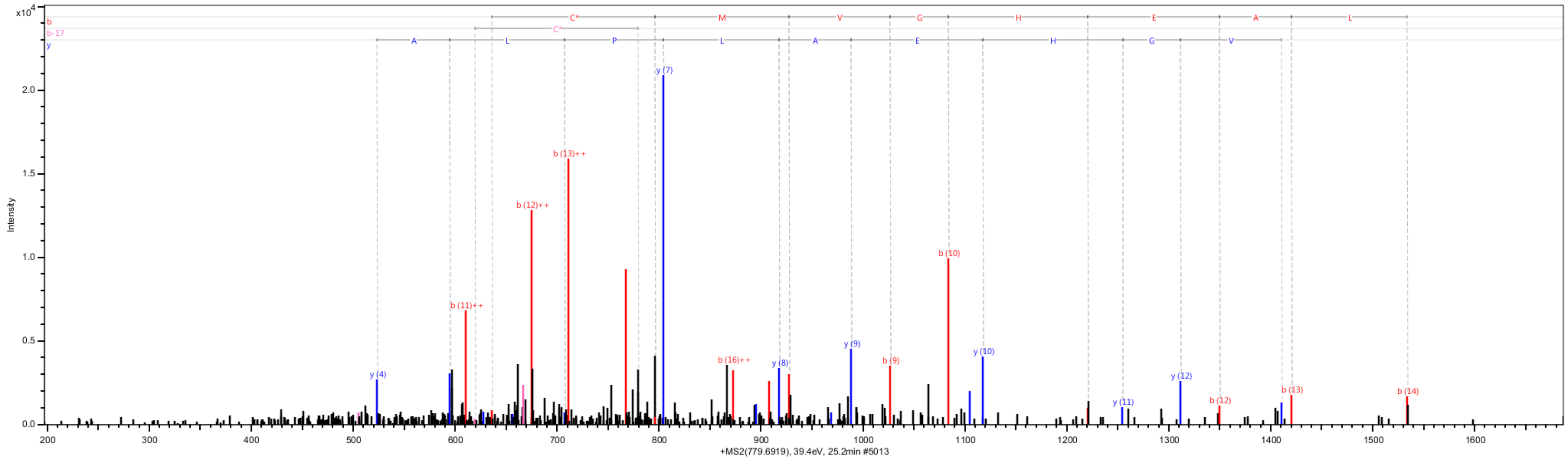
x10^4
Intensity
+MS2(779.6919), 39.4eV, 25.2min #5013
y (7)
b (13)++
b (12)++
b (11)++
b (10)
y (4)
b (16)++
y (8)
y (9)
b (9)
y (10)
y (11)
y (12)
b (12)
b (13)
b (14)

IGG1_HUMAN

Accession: IGG1_HUMAN
Database: SwissProt
MW [kDa] / pI: 49.3 / 8.9
Score: 654.7
Seq. Coverage [%]: 29.2
No. of Peptides: 12

Peptide Match

Sequence: R.LSCAASGFTFSR.Y
Score: 47.6
Modifications: Carbamidomethyl: 3

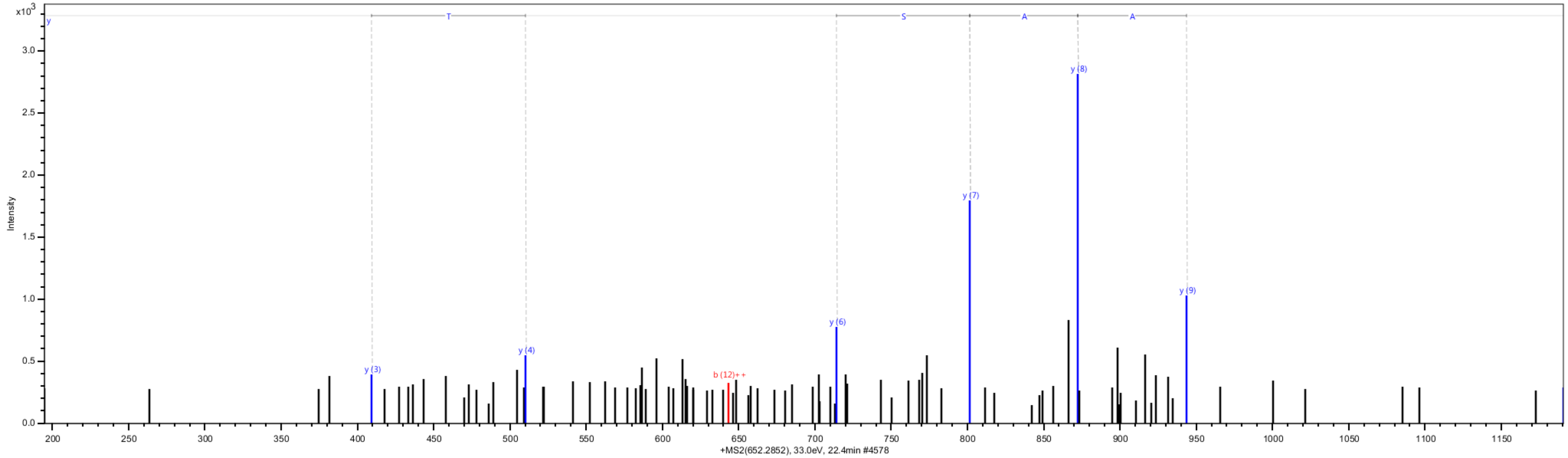

Peptide Match

Sequence: K.GPSVFPLAPSSK.S
Score: 64.6
Modifications:

Spectrum Report
Combine Search_2020-10-19 07:53:31

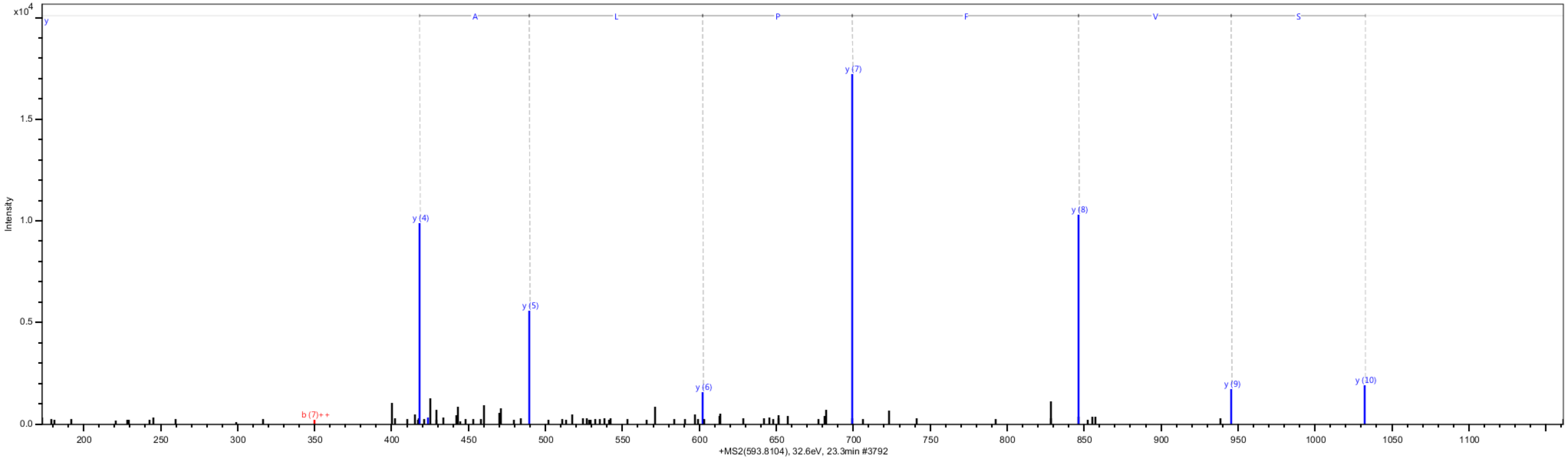

Peptide Match

**Sequence:**	K.STSGGTAALGCLVK.D	**Score:**	77.7
**Modifications:**	Carbamidomethyl: 11		

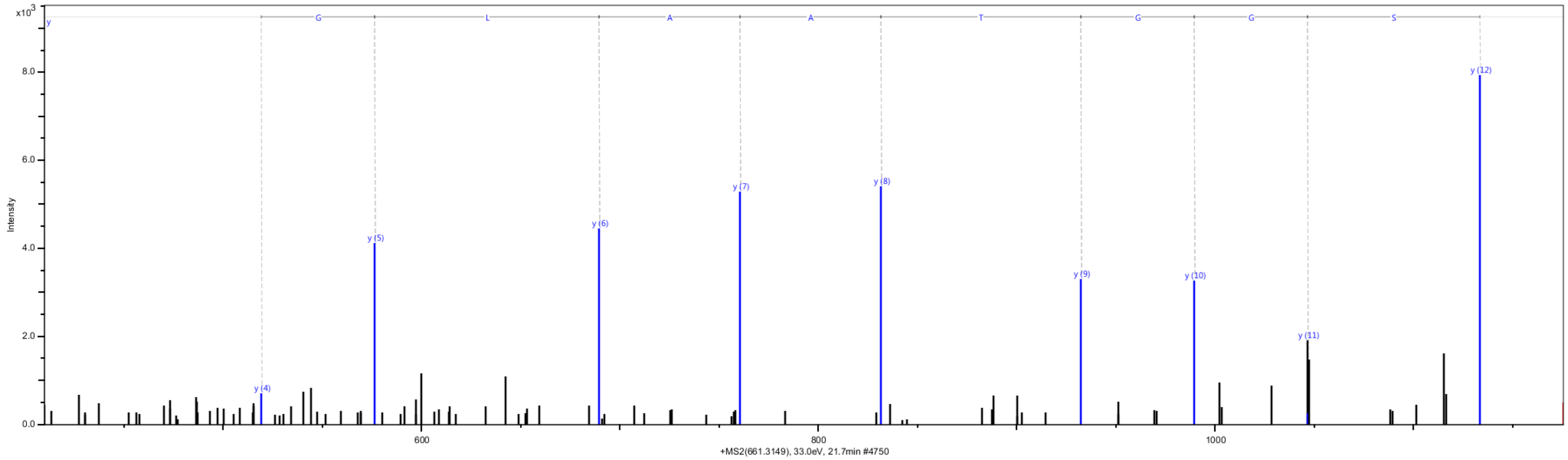

Peptide Match

**Sequence:**	K.DTLMISR.T	**Score:**	22.5
**Modifications:**			

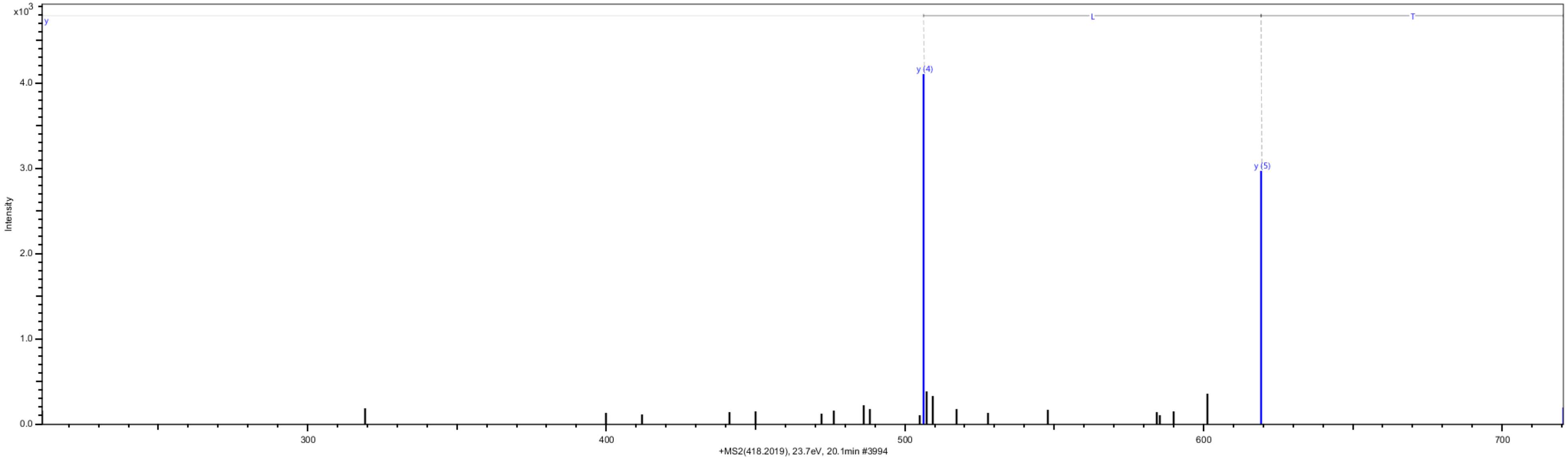

Peptide Match

Sequence: R.TPEVTCVVVDVSHEDPEVK.F **Score:** 123.9

Modifications: Carbamidomethyl: 6

Spectrum Report
Combine Search_2020-10-19 07:53:31

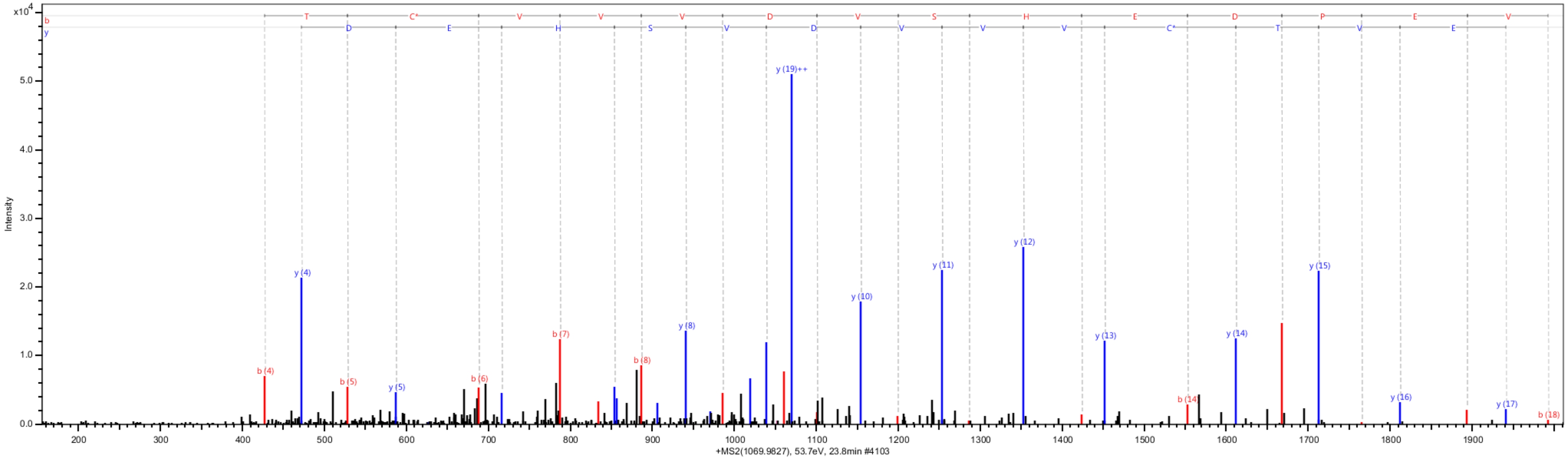

Peptide Match

Sequence: K.FNWYVDGVEVHNAK.T **Score:** 77

Modifications:

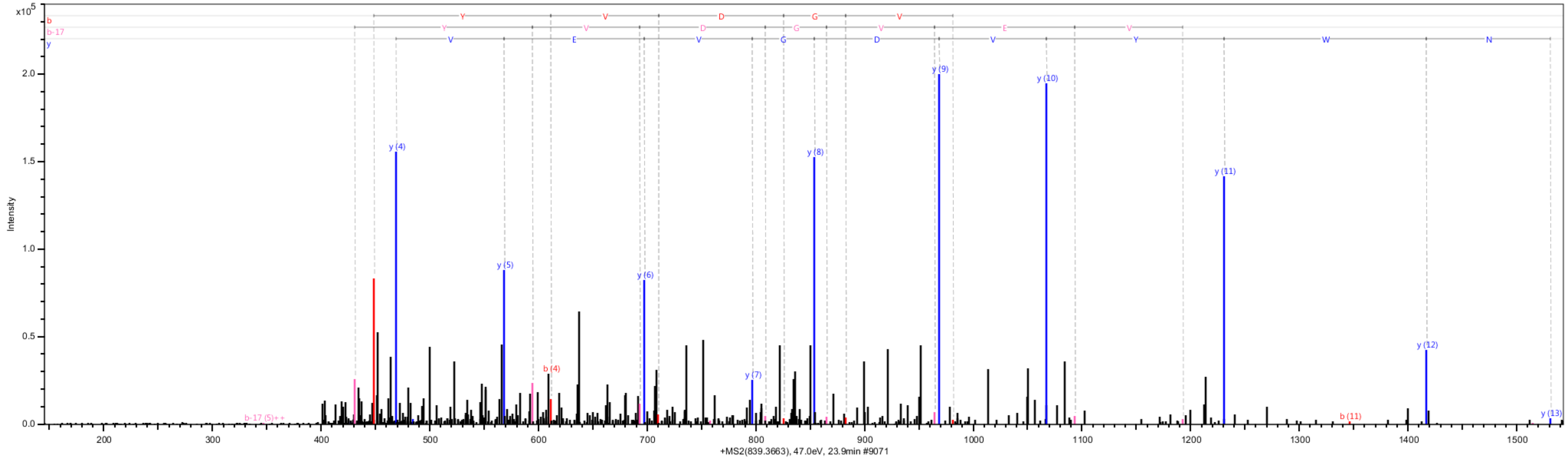

Peptide Match

Sequence: R.VVSVLTVLHQDWLNGK.E **Score:** 102.4

Modifications:

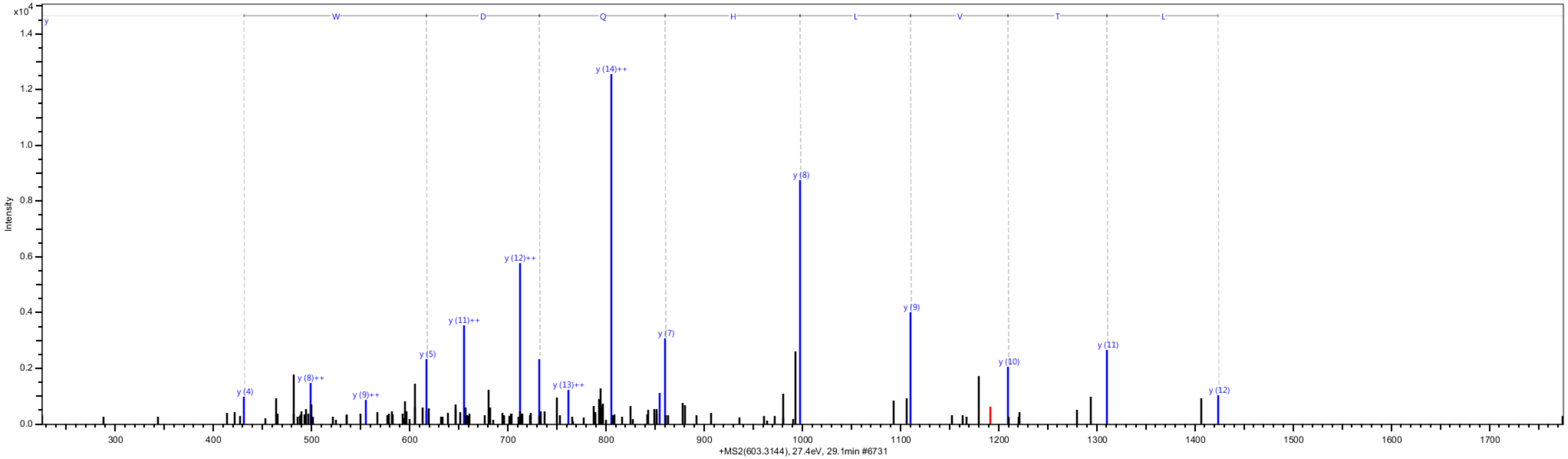

Peptide Match

**Sequence:**	K.VSNKALPAPIEK.T	**Score:**	17.5
**Modifications:**			

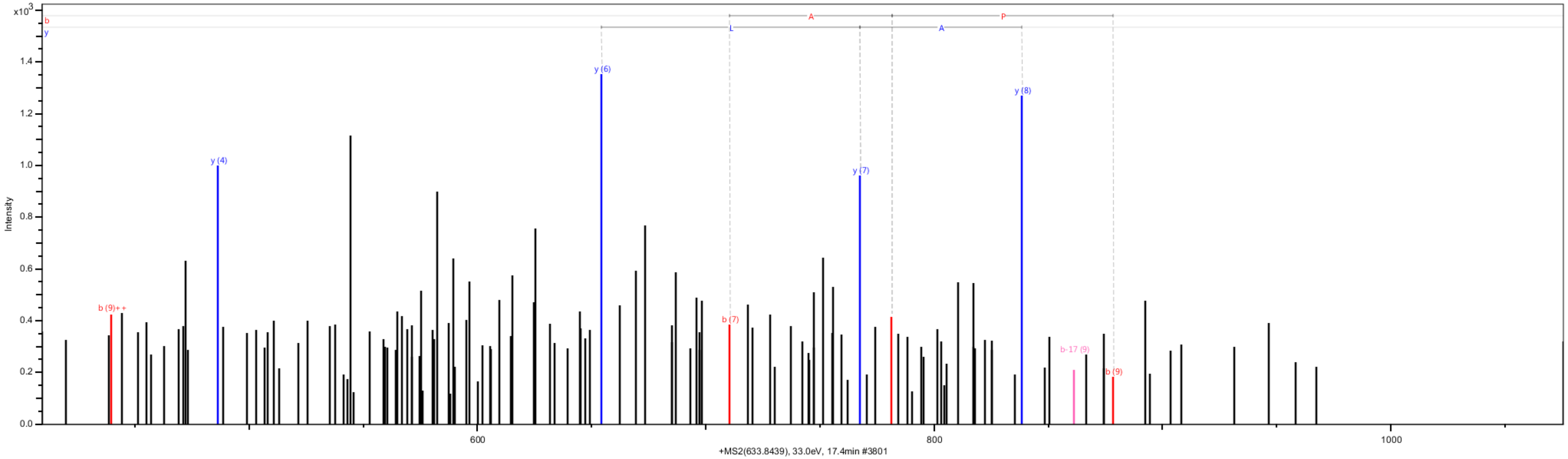

Peptide Match

Sequence: K.ALPAPIEK.T **Score:** 25.2

Modifications:

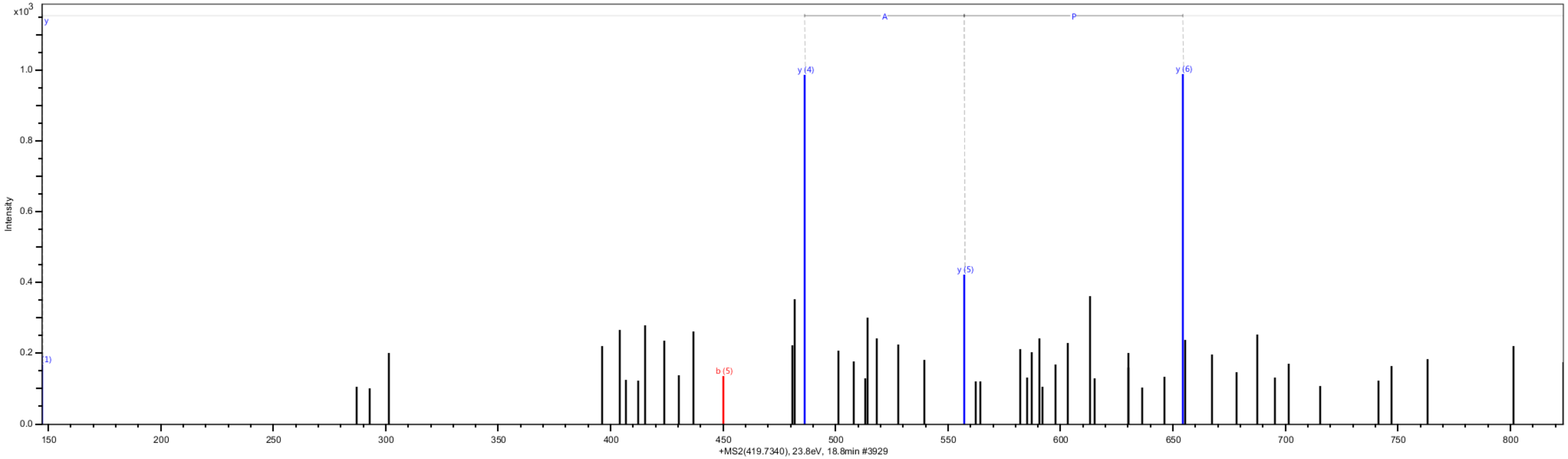

Peptide Match

Sequence: K.ALPAPIEKTISK.A **Score:** 22.3

Modifications:

Spectrum Report
Combine Search_2020-10-19 07:53:31

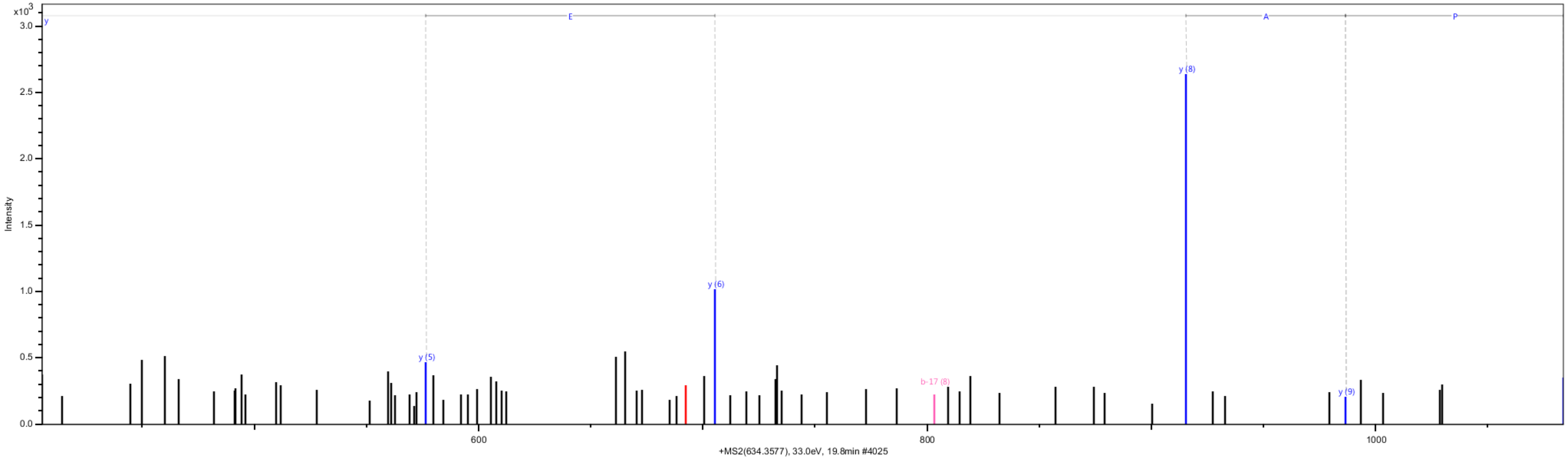

Peptide Match

Sequence: R.EPQVYTLPPSR.D **Score:** 27.7

Modifications:

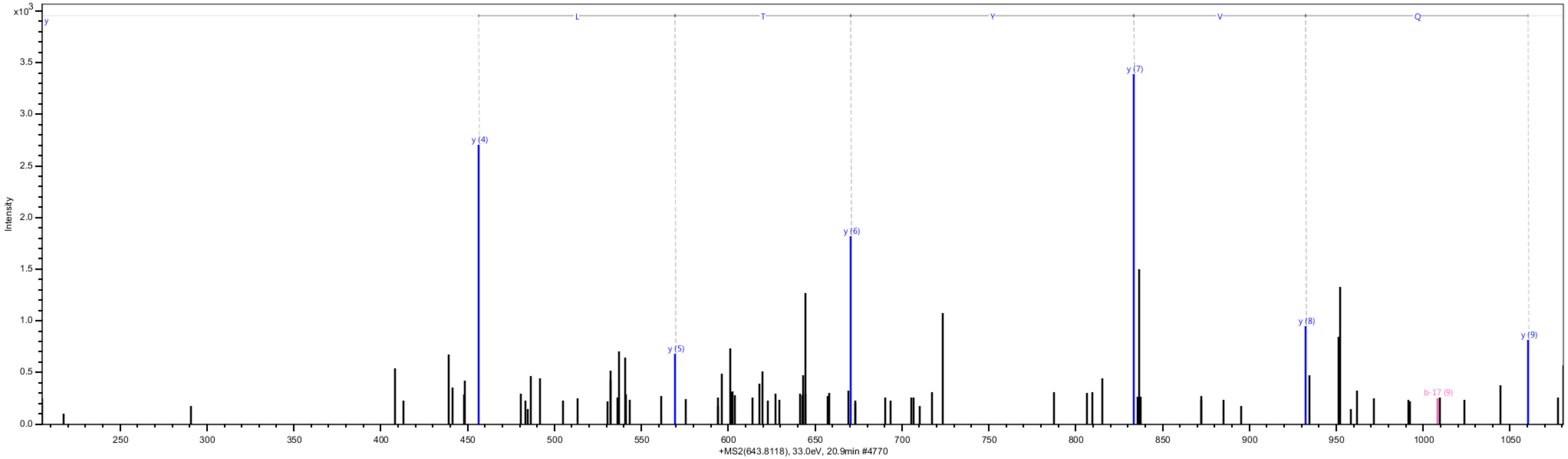

Peptide Match

**Sequence:**	K.NQVSLTCLVK.G	**Score:**	46.1
**Modifications:**	Carbamidomethyl: 7		

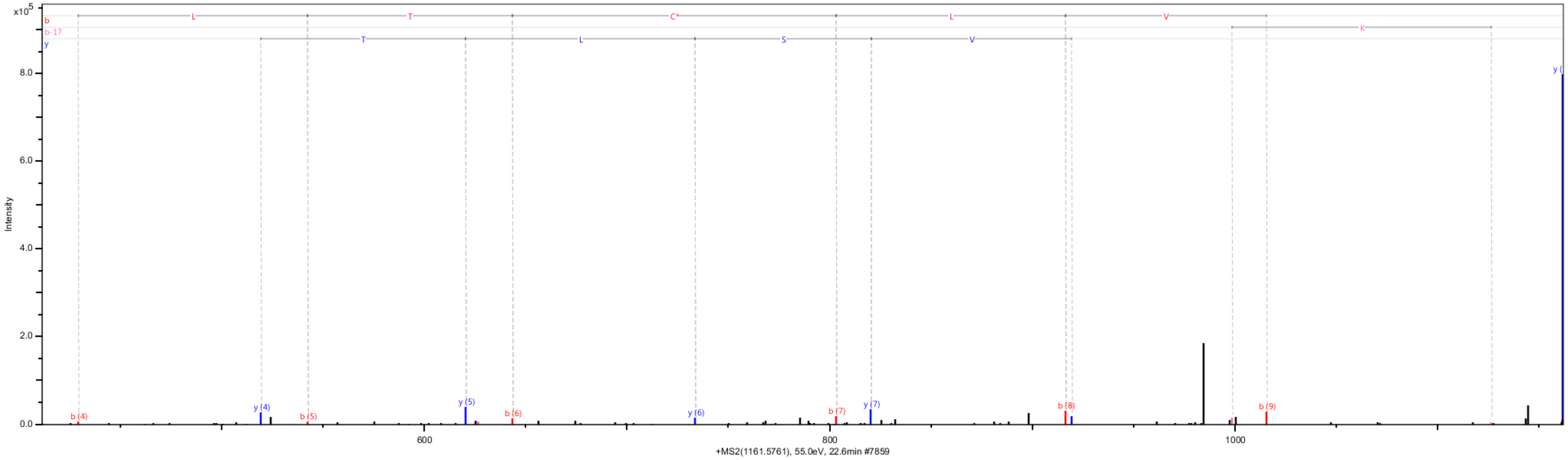
x10^5
b
b-17
y
L
T
C*
L
V
K
T
L
S
V
Intensity
8.0
6.0
4.0
2.0
0.0
b (4)
y (4)
b (5)
y (5)
b (6)
y (6)
b (7)
y (7)
b (8)
b (9)
600
800
1000
+MS2(1161.5761), 55.0eV, 22.6min #7859

HPT_HUMAN

**Accession:**	HPT_HUMAN	**Score:**	659.3
**Database:**	SwissProt	**Seq. Coverage [%]:**	33.5
**MW [kDa] / pI:**	45.2 / 6.1	**No. of Peptides:**	13

Peptide Match

Sequence: K.LRTEGDGVYTLNDK.K **Score:** 45.5

Modifications:

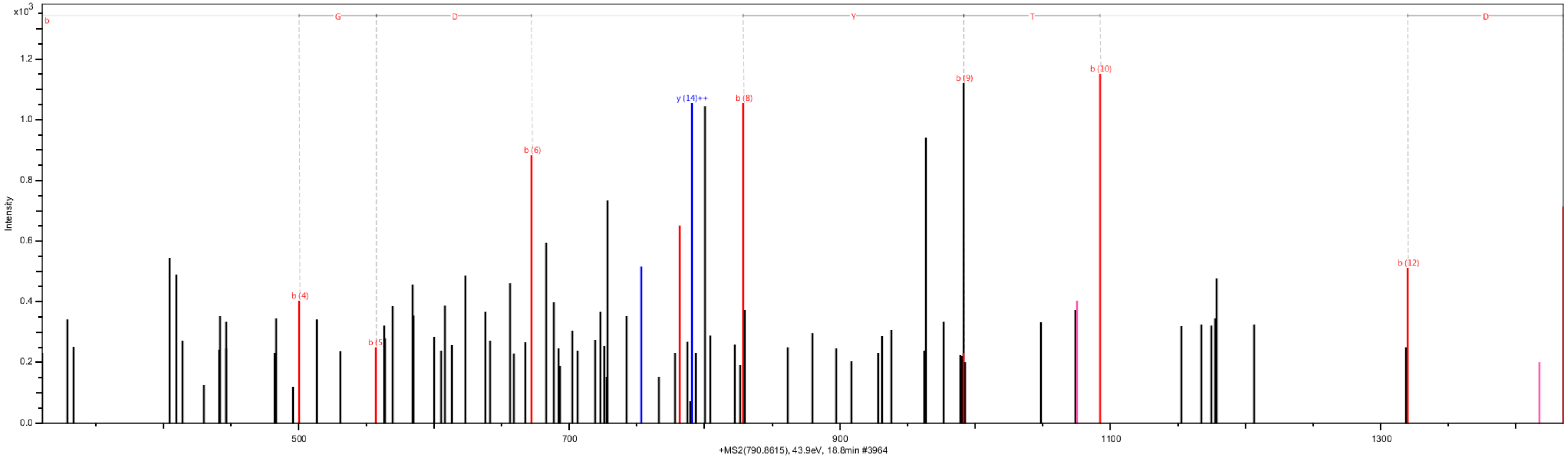

Peptide Match

Sequence: K.LRTEGDGVYTLNNEK.Q **Score:** 28.9

Modifications:

Spectrum Report
Combine Search_2020-10-19 07:53:31

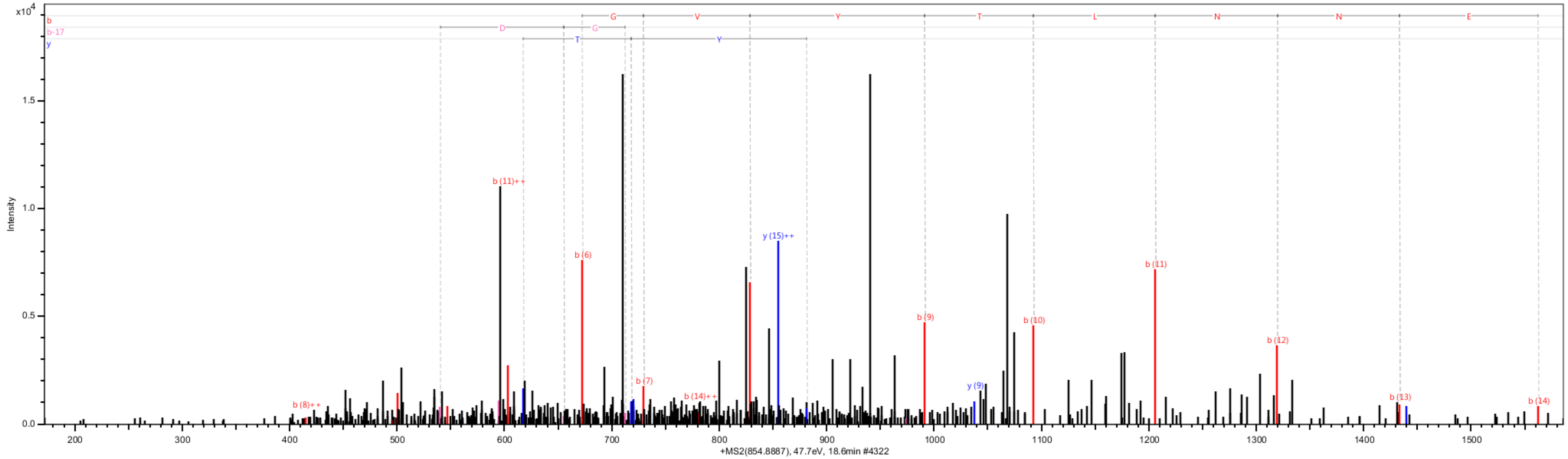

Peptide Match

Sequence: R.TEGDGVYTLNNEK.Q **Score:** 40.8

Modifications:

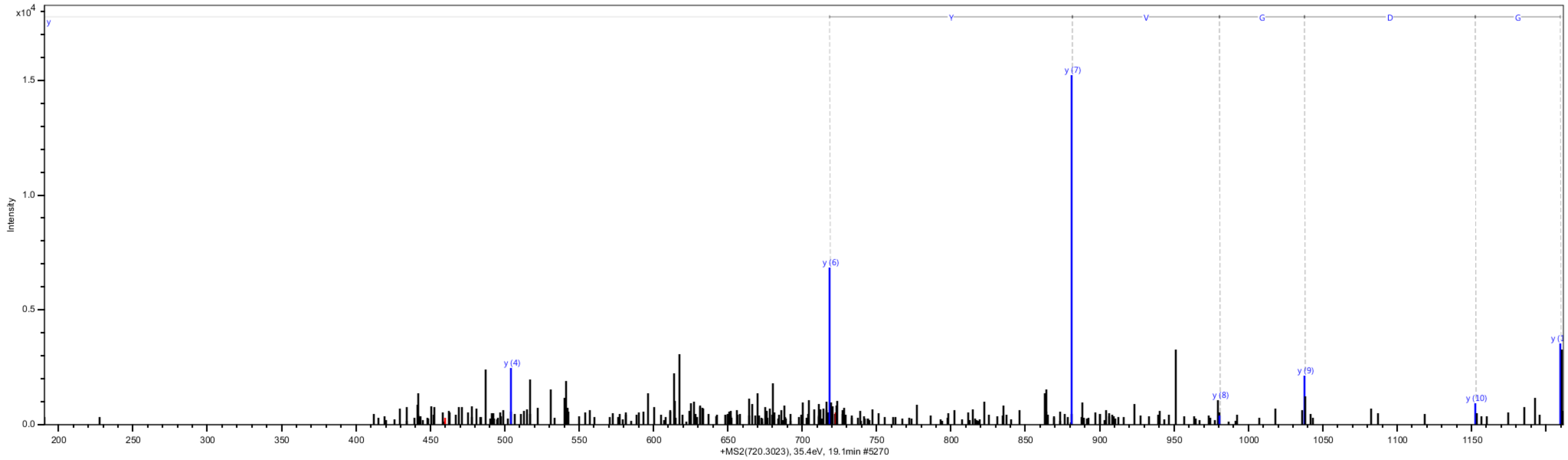

Peptide Match

Sequence: R.TEGDGVYTLNNEKQWINK.A **Score:** 82.2

Modifications:

Spectrum Report
Combine Search_2020-10-19 07:53:31

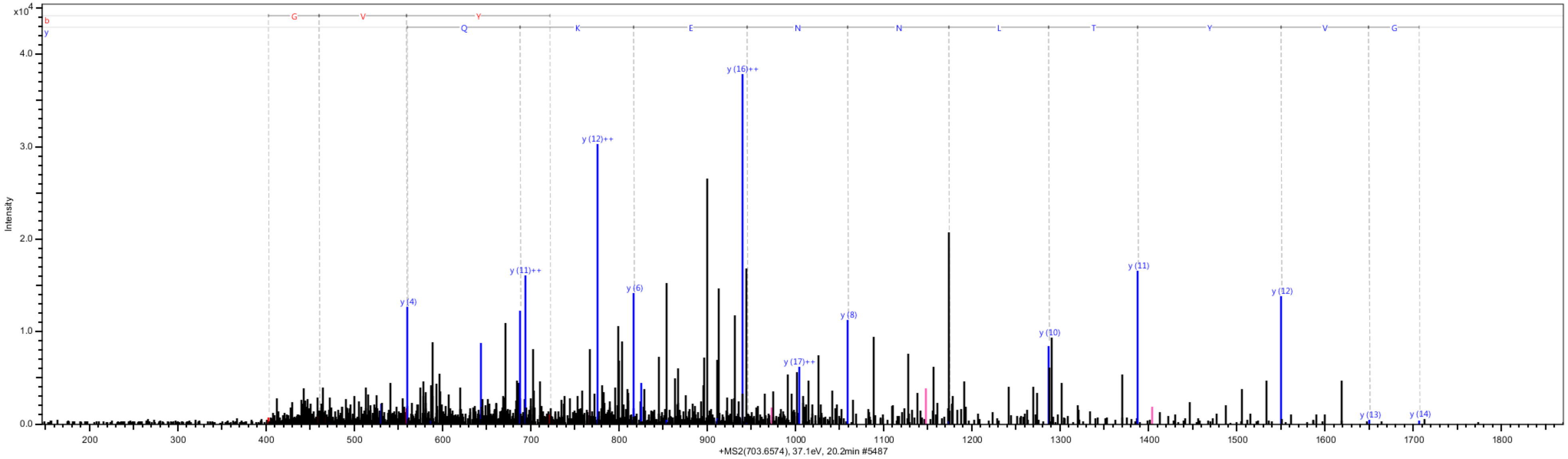

Peptide Match

**Sequence:**	K.GSFPWQAK.M	**Score:**	20.6
**Modifications:**			

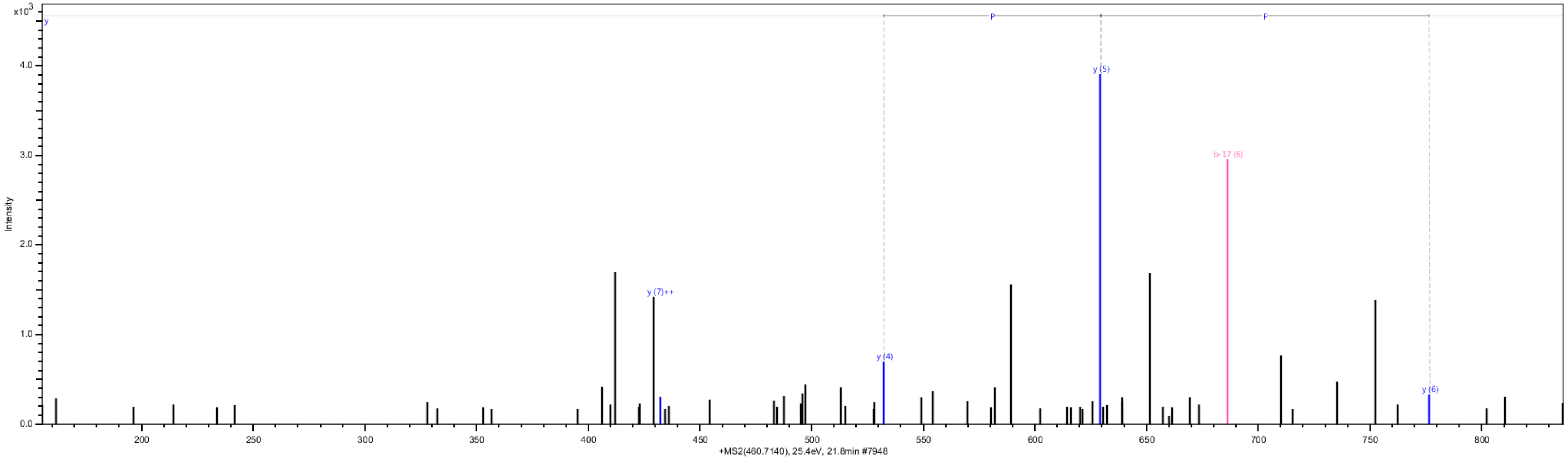

Peptide Match

Sequence: K.DIAPTLTLYVGK.K **Score:** 40.4

Modifications:

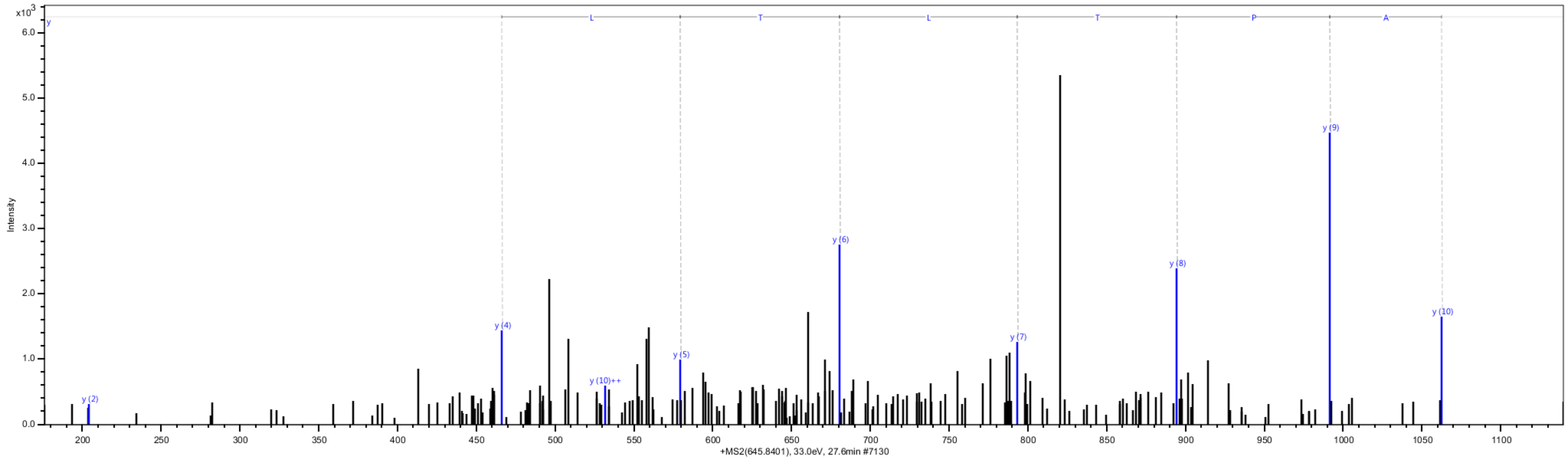

Peptide Match

Sequence: K.DIAPTLTLYVGKK.Q **Score:** 67.9

Modifications:

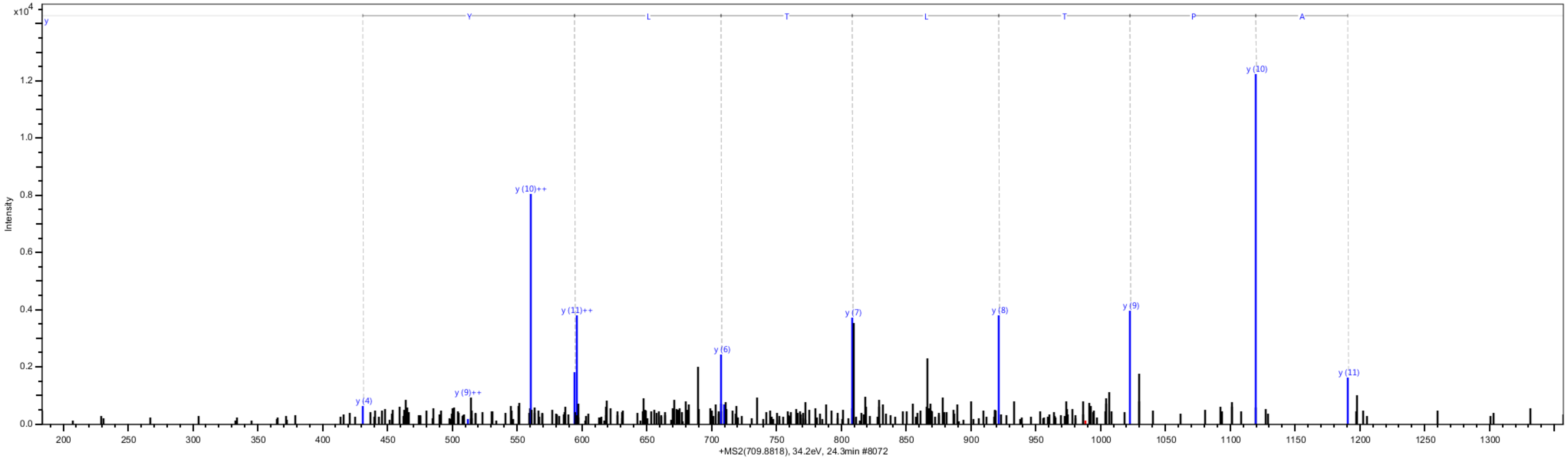

Peptide Match

Sequence: R.VMPICLPSKDYAEVGR.V **Score:** 52.2

Modifications: Carbamidomethyl: 5

Spectrum Report
Combine Search_2020-10-19 07:53:31

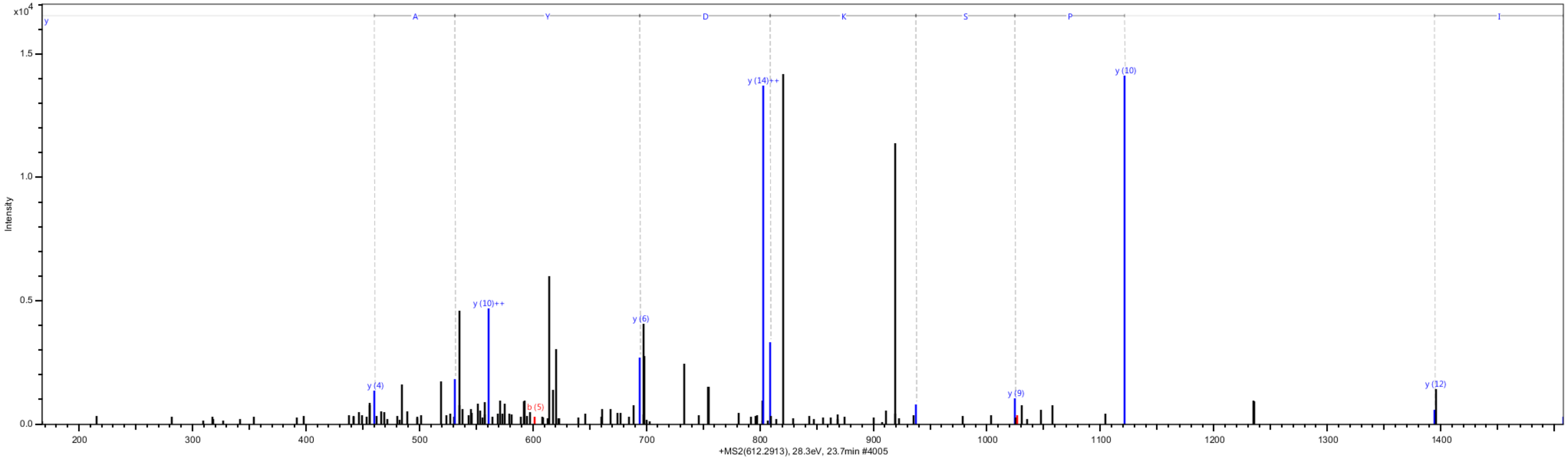

Peptide Match

**Sequence:**	R.VGYVSGWGR.N	**Score:**	60.7
**Modifications:**			

Spectrum Report
Combine Search_2020-10-19 07:53:31

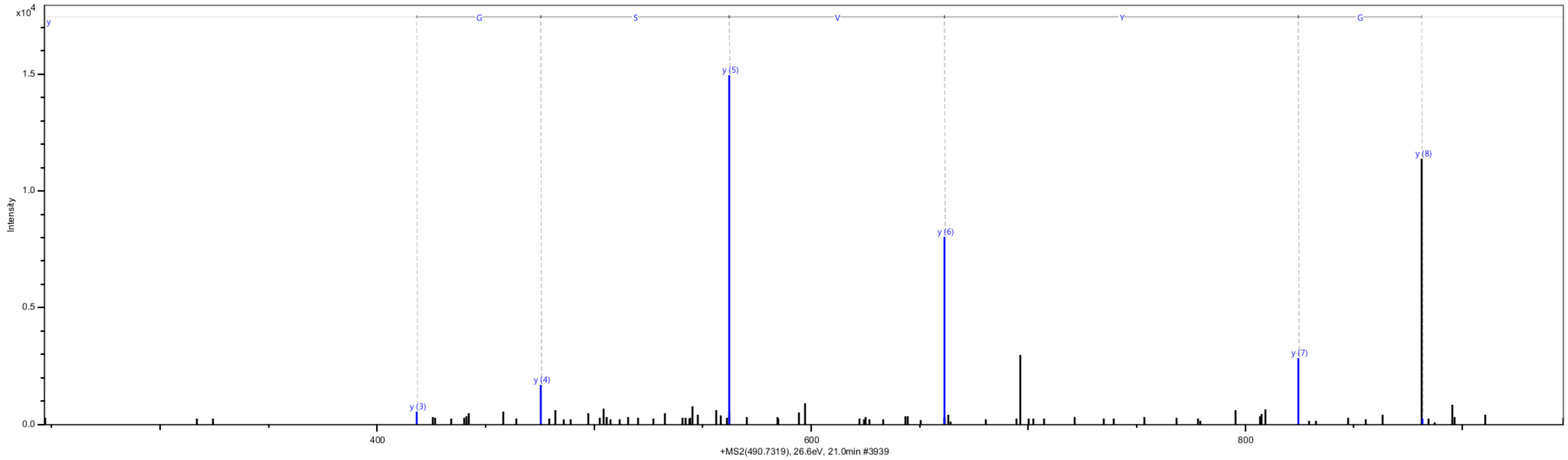

Peptide Match

**Sequence:**	K.YVMLPVADQDQCIR.H	**Score:**	30.2
**Modifications:**	Carbamidomethyl: 12		

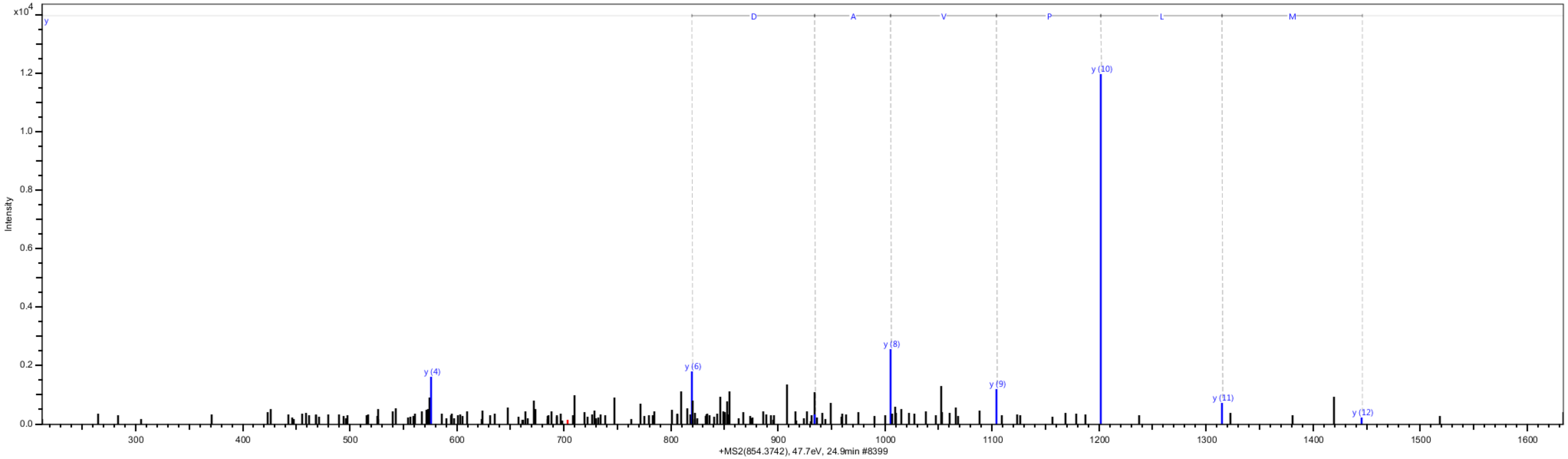

Peptide Match

**Sequence:**	K.SPVGVQPILNEHTFCAGMSK.Y	**Score:**	82.6
**Modifications:**	Carbamidomethyl: 15		

Spectrum Report
Combine Search_2020-10-19 07:53:31

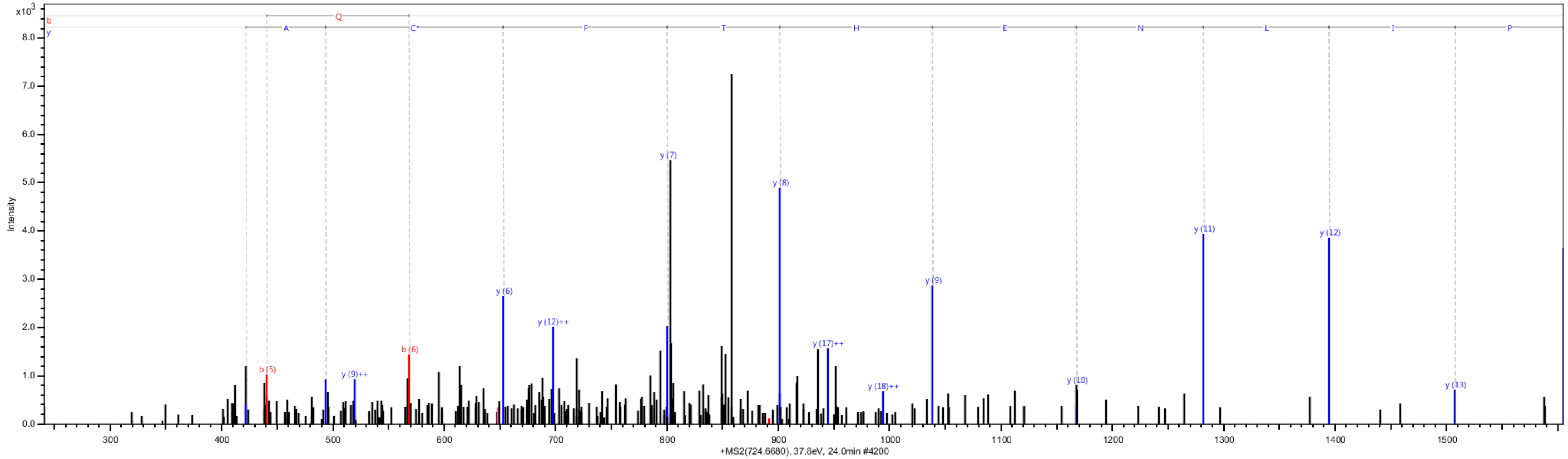

Peptide Match

**Sequence:**	K.SCAVAEYGVYVK.V	**Score:**	51.7
**Modifications:**	Carbamidomethyl: 2		

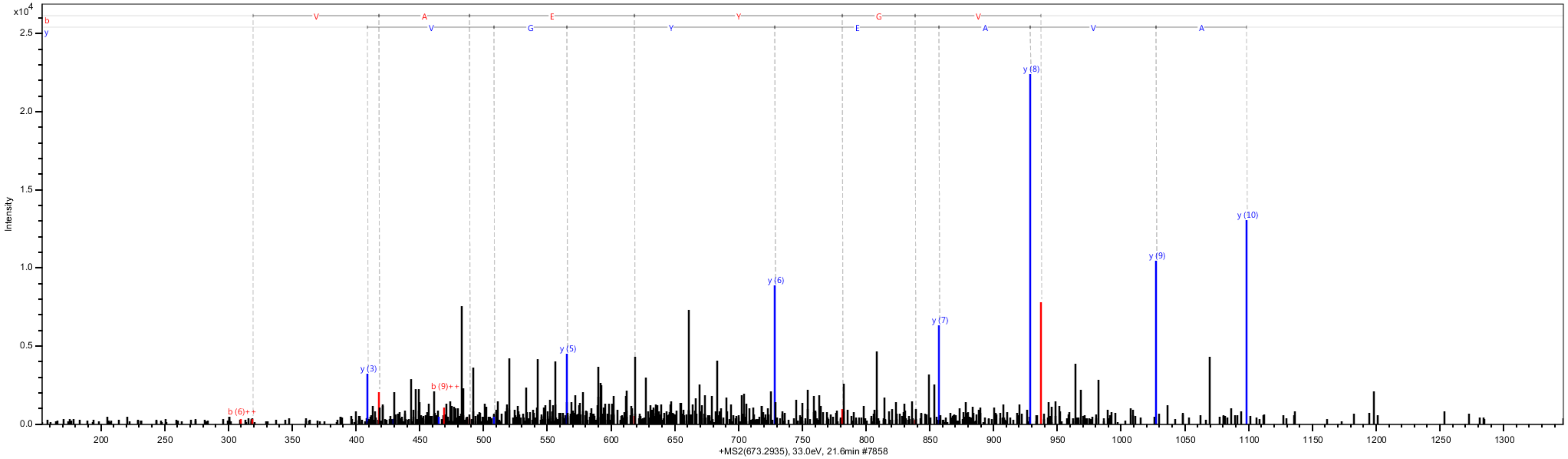

Peptide Match

Sequence: K.VTSIQDWVQK.T **Score:** 55.5

Modifications:

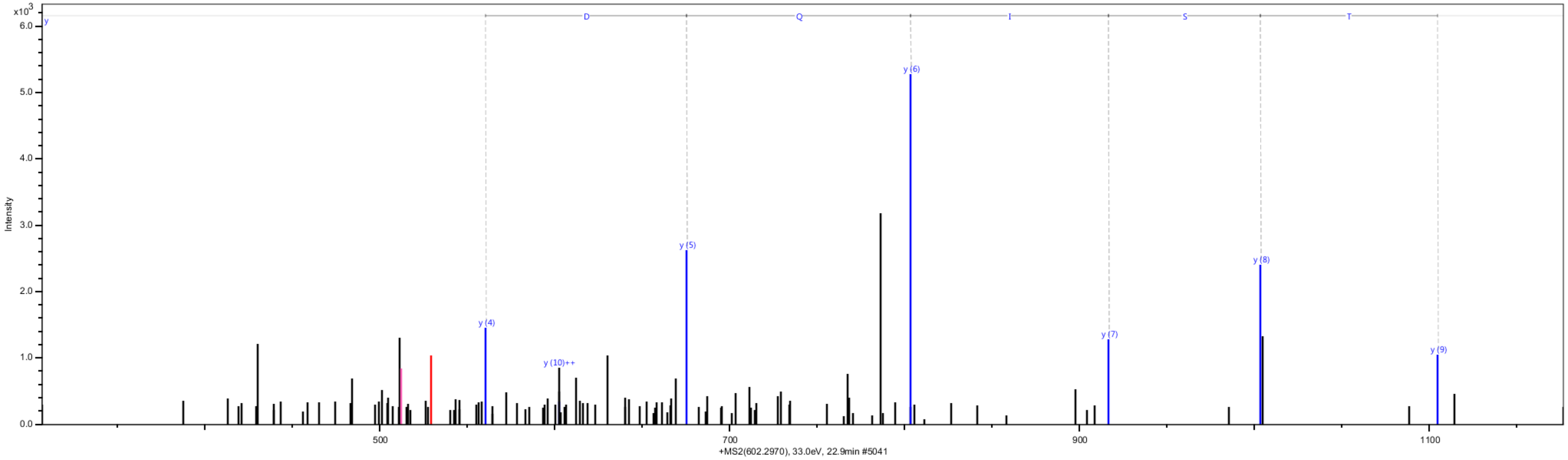
x10^3
6.0
5.0
4.0
3.0
2.0
1.0
0.0
Intensity
y
D
Q
I
S
T
y (6)
y (5)
y (8)
y (4)
y (7)
y (9)
y (10)++
500
700
900
1100
+MS2(602.2970), 33.0eV, 22.9min #5041

APOA1_HUMAN

**Accession:**	APOA1_HUMAN	**Score:**	1014.4
**Database:**	SwissProt	**Seq. Coverage [%]:**	49.4
**MW [kDa] / pI:**	30.8 / 5.6	**No. of Peptides:**	18

Peptide Match

Sequence: R.VKDLATVYVDVLK.D **Score:** 85.5
Modifications:

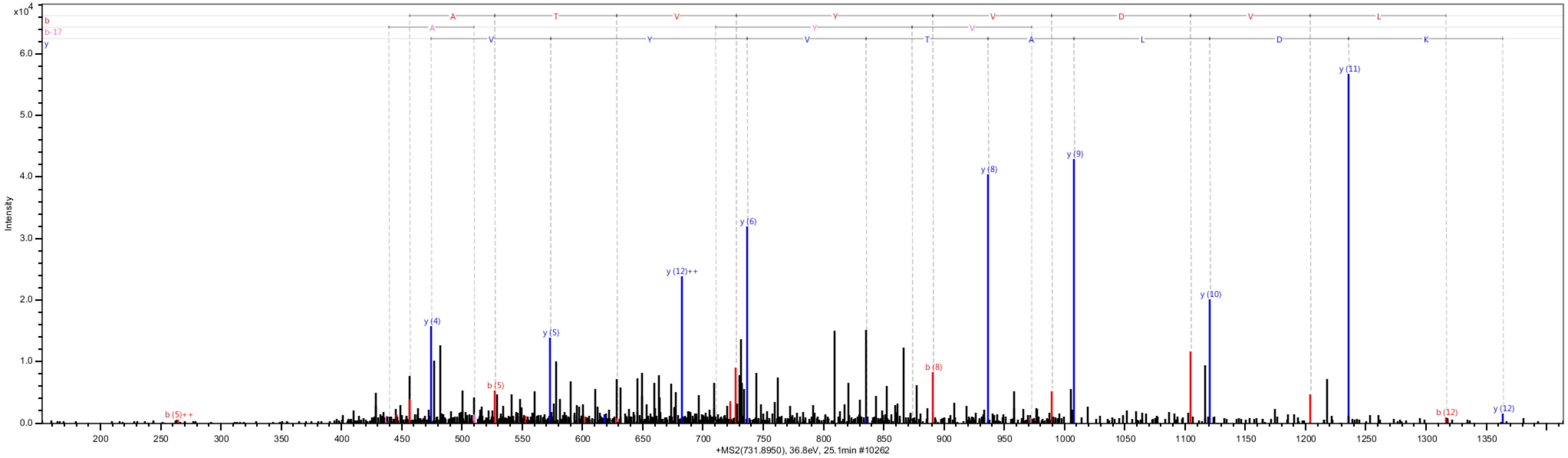

Peptide Match

Sequence: K.DLATVYVDVLK.D **Score:** 26.2
Modifications:

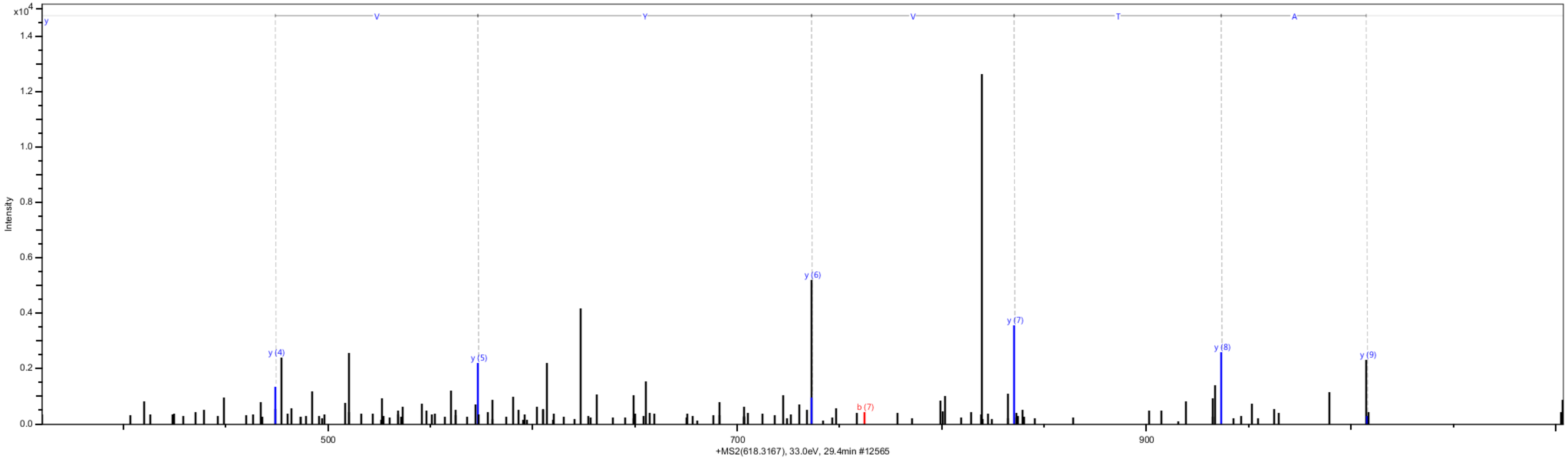

Peptide Match

Sequence: K.DLATVYYVDVLKDSGR.D **Score:** 68

Modifications:

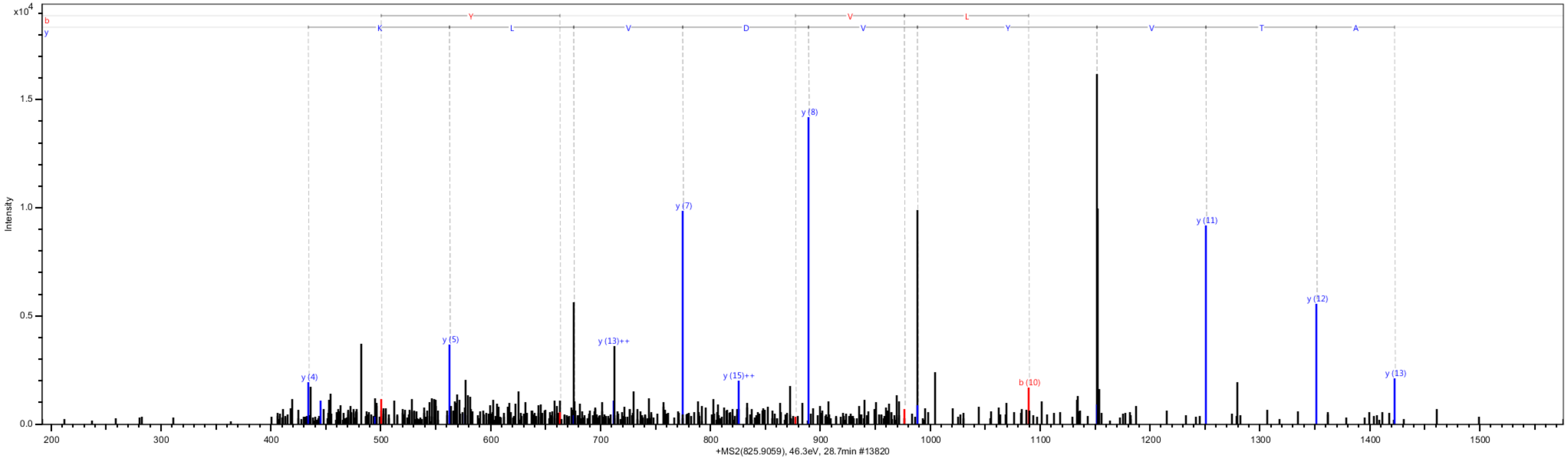

Peptide Match

Sequence: K.DSGRDYVSQFEGSALGK.Q **Score:** 67.9

Modifications:

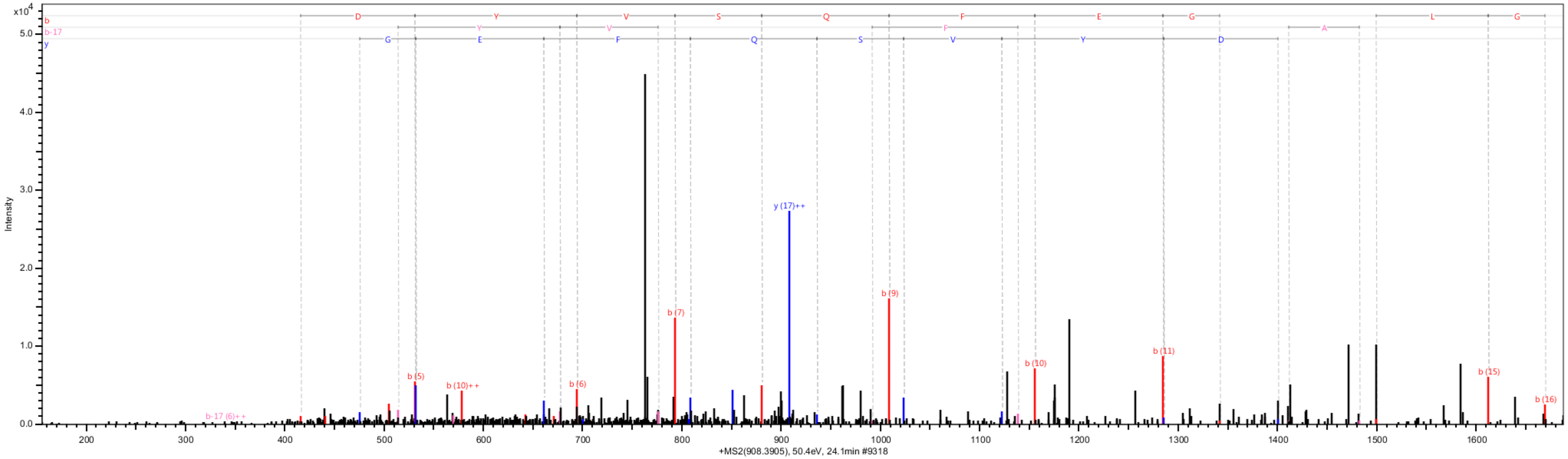

Peptide Match

Sequence: R.DYVSQFEGSALGK.Q **Score:** 39.7

Modifications:

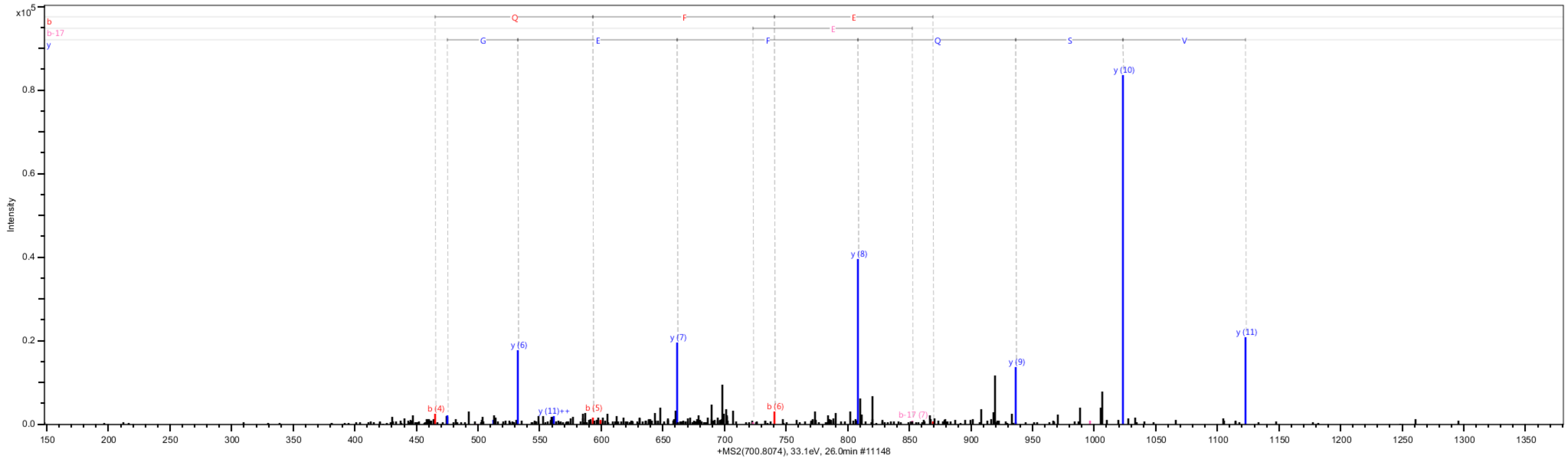

Peptide Match

Sequence: K.LLDNWDSVTSTFSK.L **Score:** 88.8

Modifications:

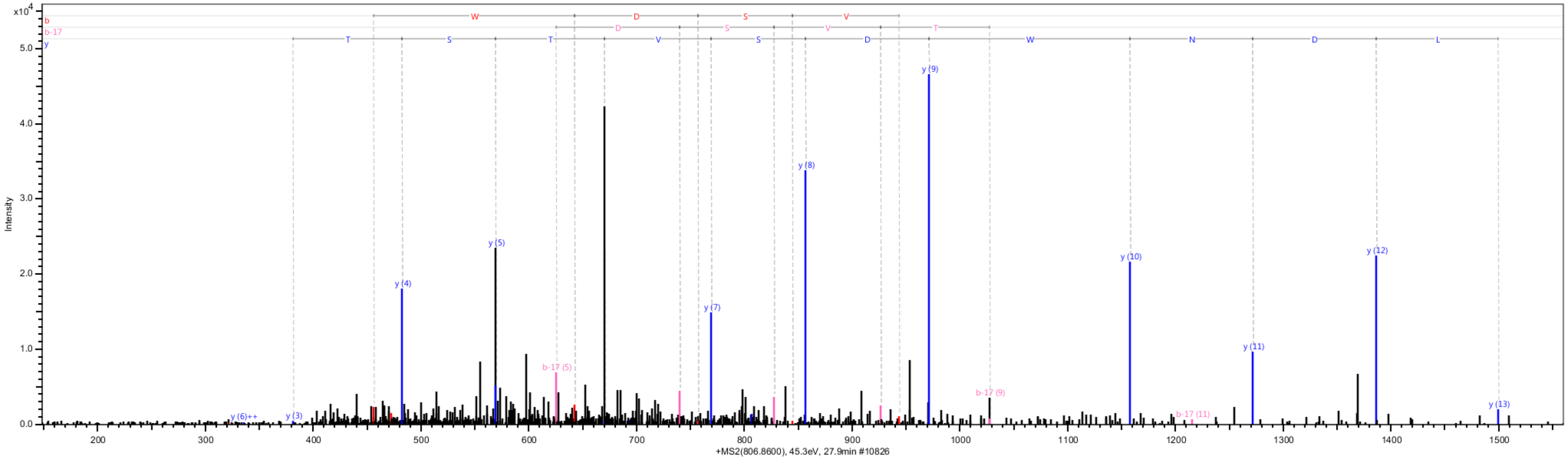

Peptide Match

Sequence: K.VQPYLDDFQK.K **Score:** 35.2

Modifications:

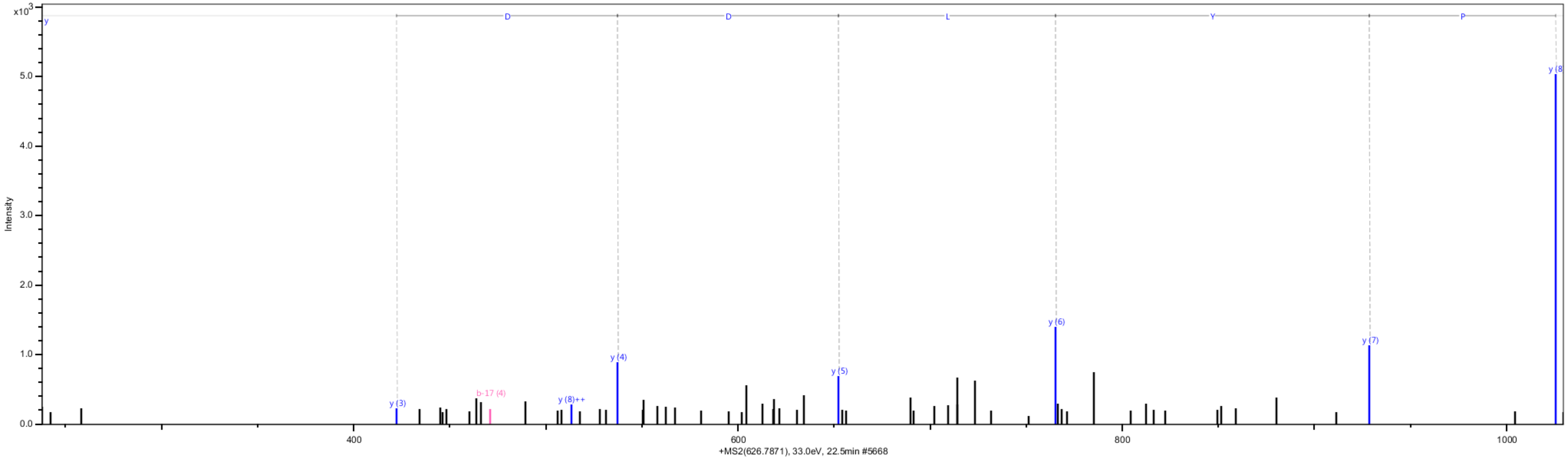

Peptide Match

Sequence: K.VQPYLDDFQKK.W **Score:** 45.1

Modifications:

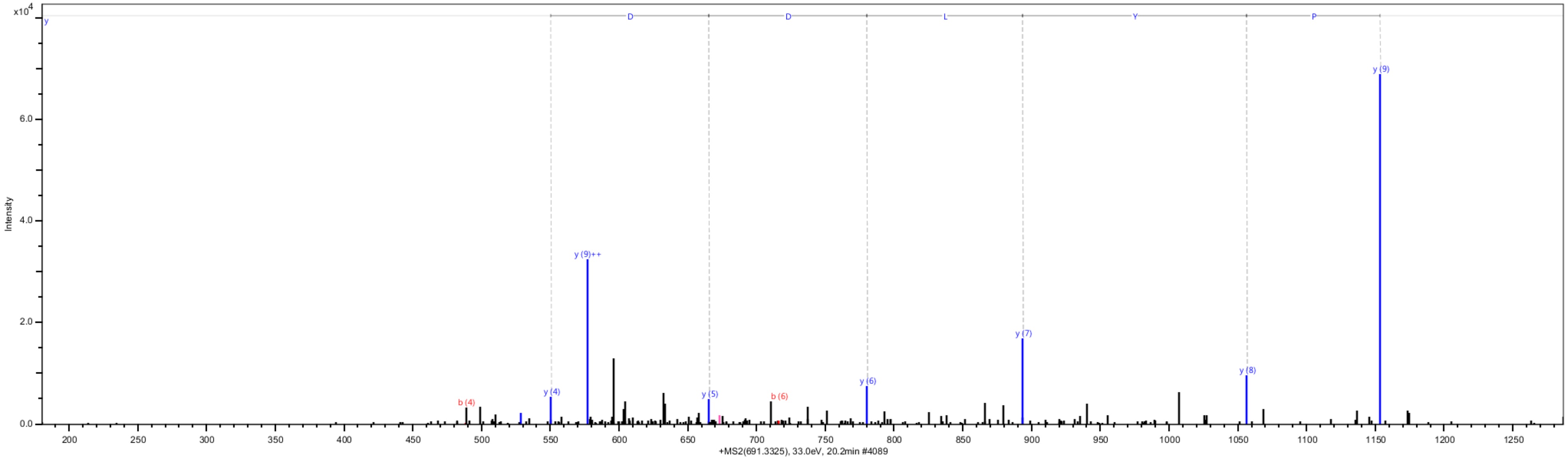

Peptide Match

**Sequence:**	K.WQEEMELYR.Q	**Score:**	25.9
**Modifications:**	Oxidation: 5		

Spectrum Report
Combine Search_2020-10-19 07:53:31

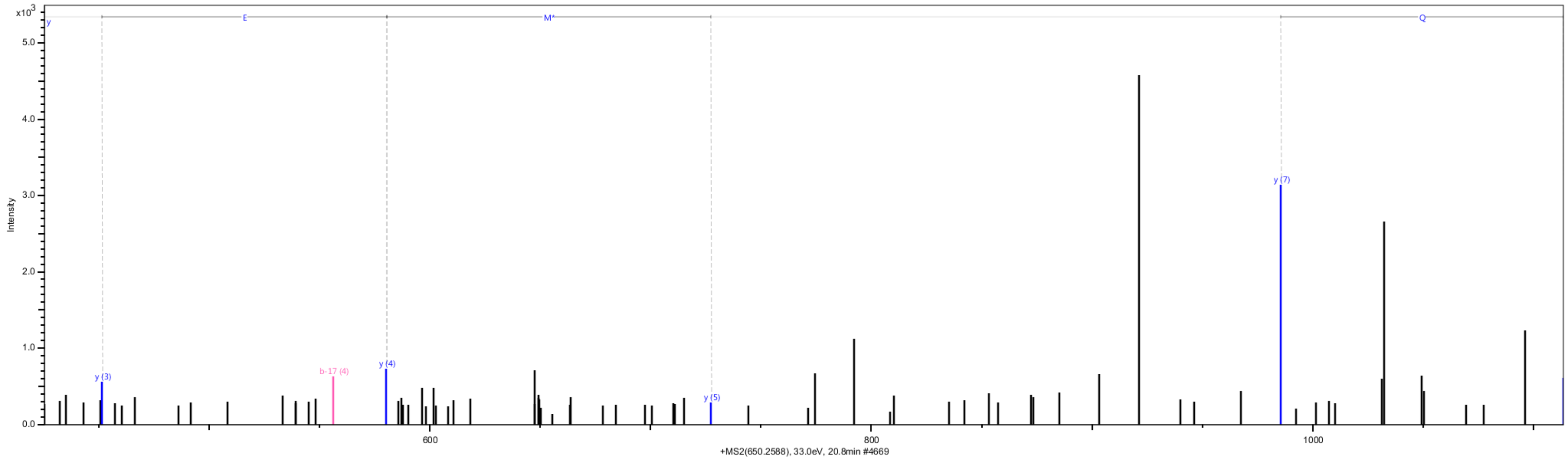

Peptide Match

**Sequence:**	K.WQEEMELYR.Q	**Score:**	36.2
**Modifications:**			

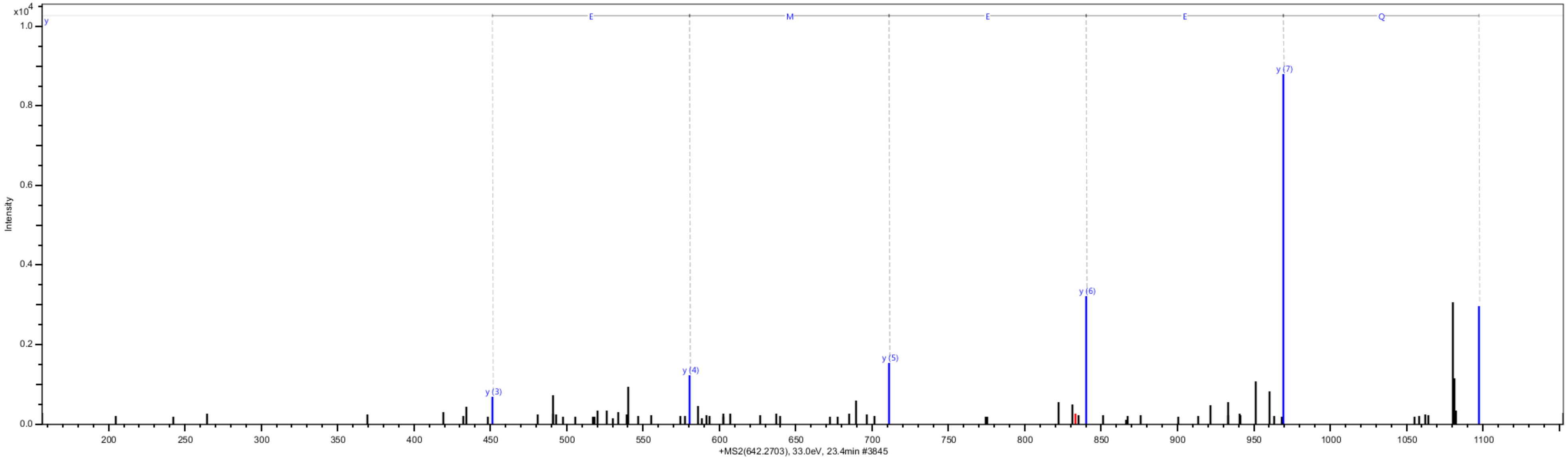

Peptide Match

Sequence: K.VEPLRAELQEGAR.Q **Score:** 57.5

Modifications:

Spectrum Report
Combine Search_2020-10-19 07:53:31

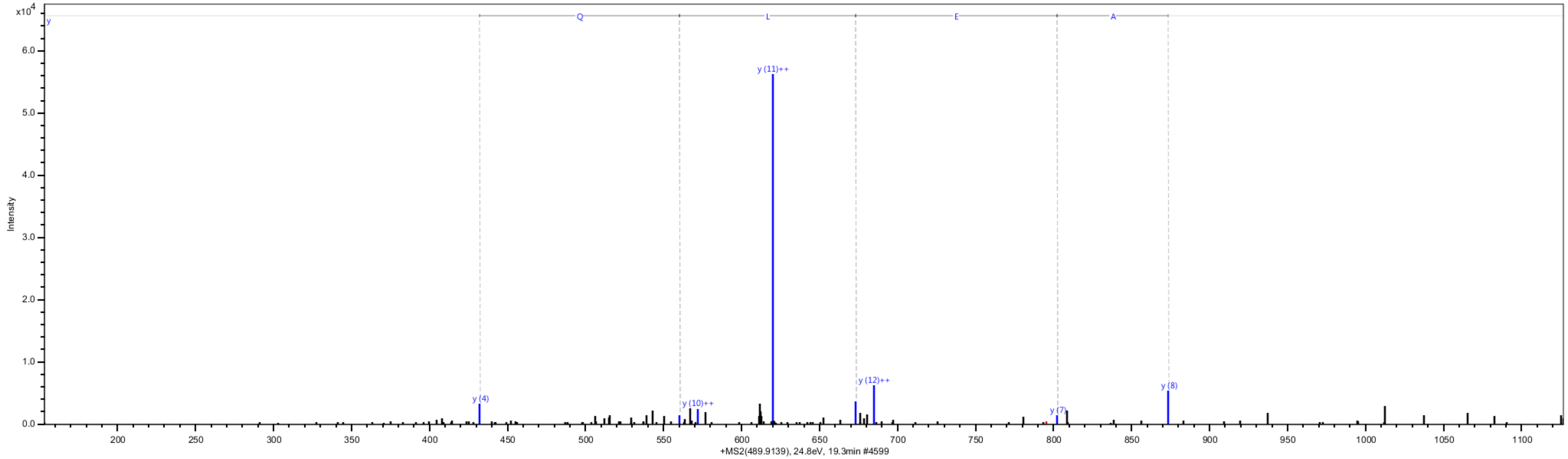

Peptide Match

Sequence: K.LSPLGEEMR.D **Score:** 50.2

Modifications:

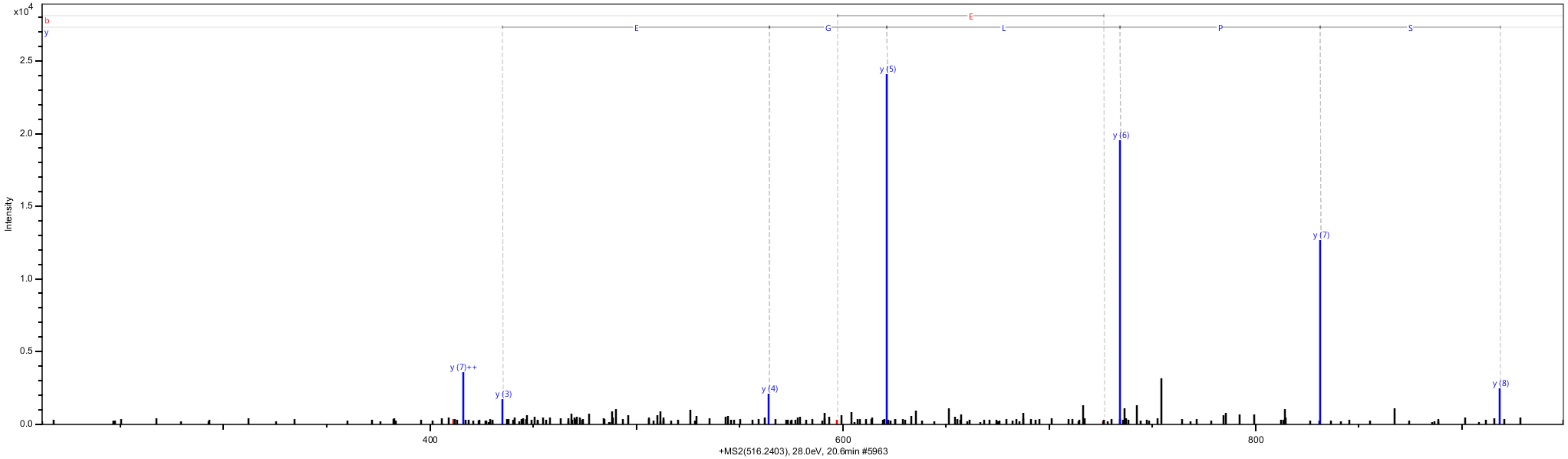

Peptide Match

Sequence: R.THLAPYSDELR.Q **Score:** 75.7

Modifications:

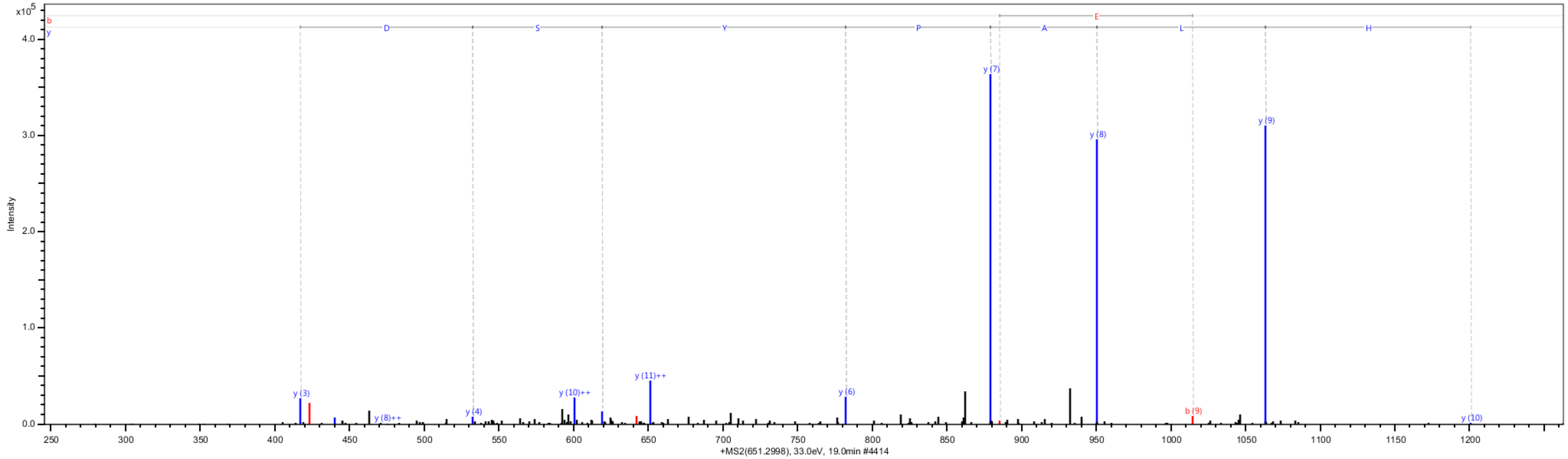

Peptide Match

Sequence: R.THLAPYSDELRQR.L **Score:** 65.8

Modifications:

Spectrum Report
Combine Search_2020-10-19 07:53:31

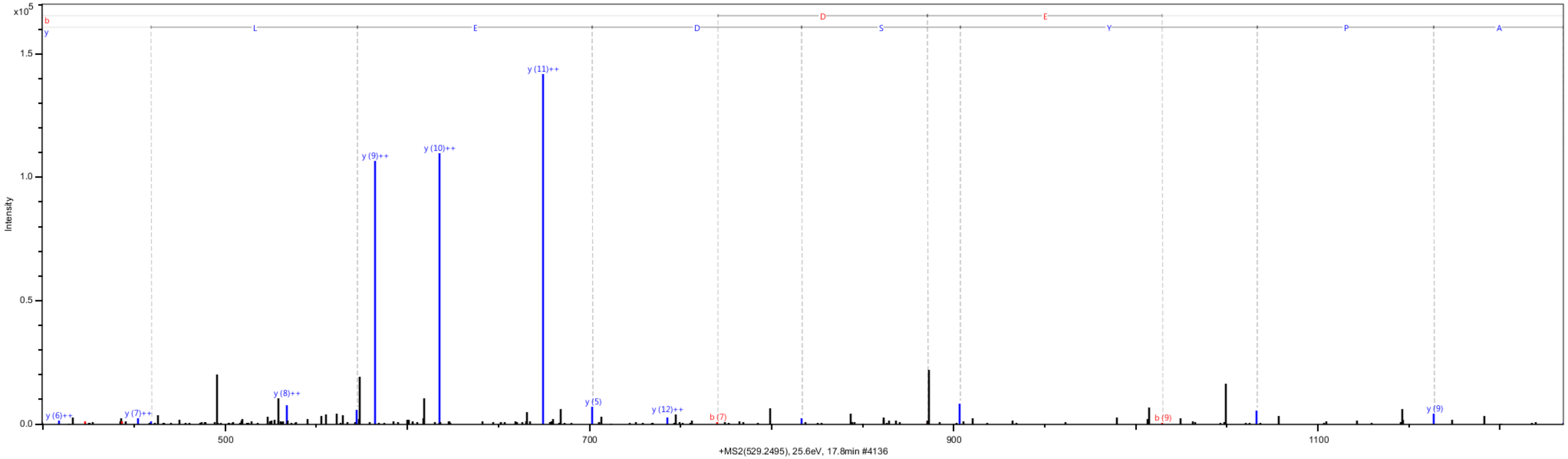

Peptide Match

**Sequence:**	K.AKPALEDLR.Q	**Score:**	17.8
**Modifications:**			

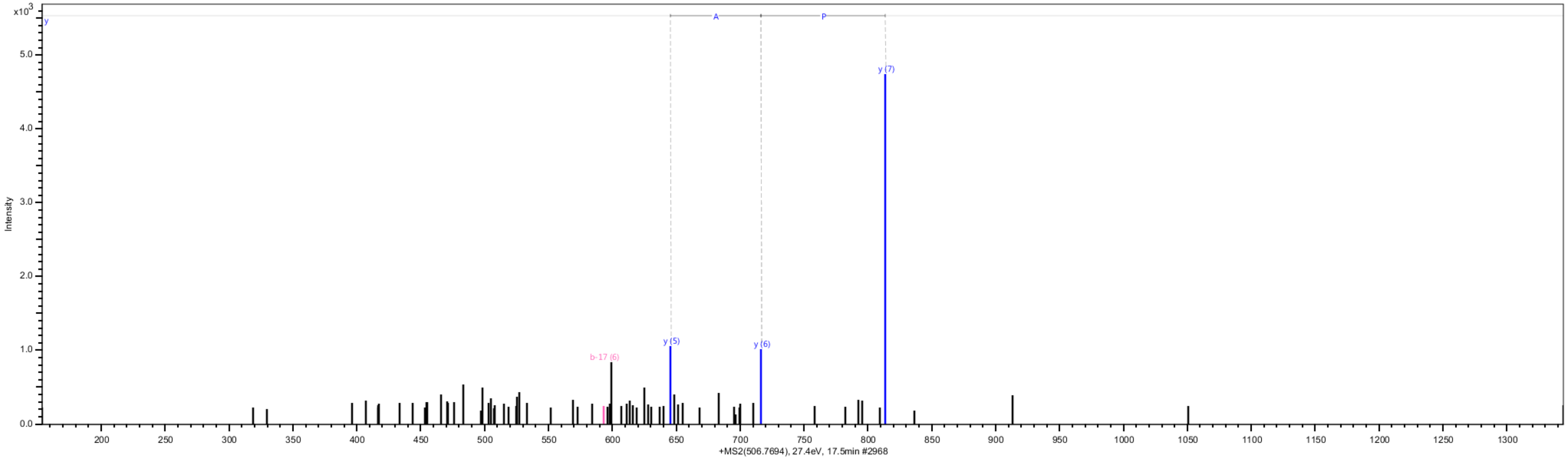

Peptide Match

**Sequence:**	R.QGLLPVLESFK.V	**Score:**	74.8
**Modifications:**			

Spectrum Report
Combine Search_2020-10-19 07:53:31

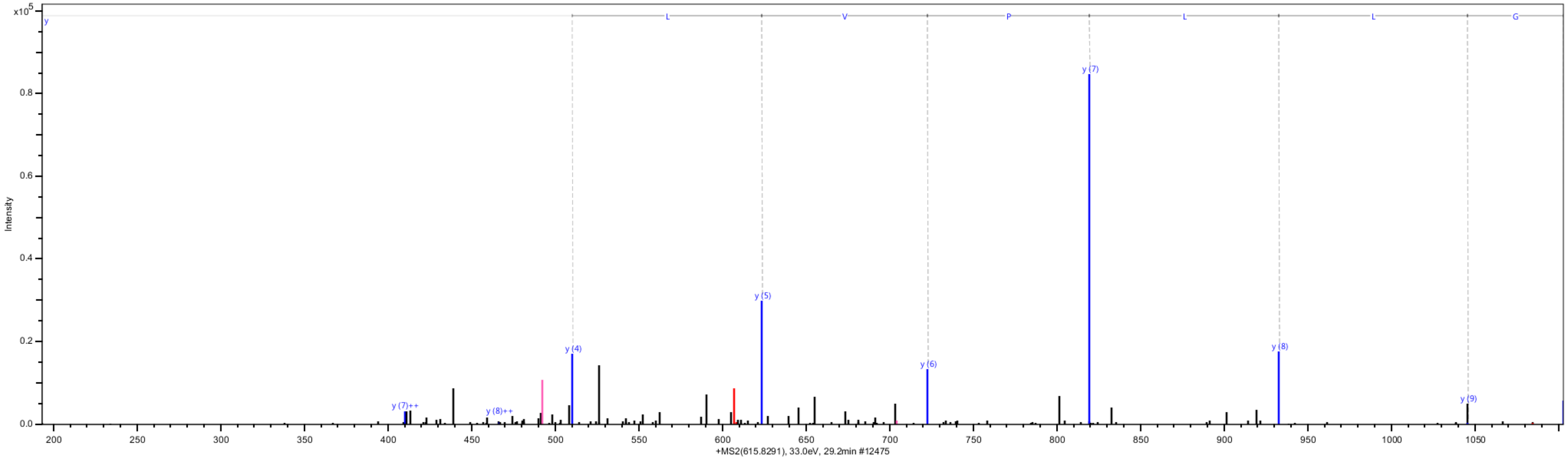

Peptide Match

**Sequence:**	K.VSFLSALEEYTK.K	**Score:**	94.5
**Modifications:**			

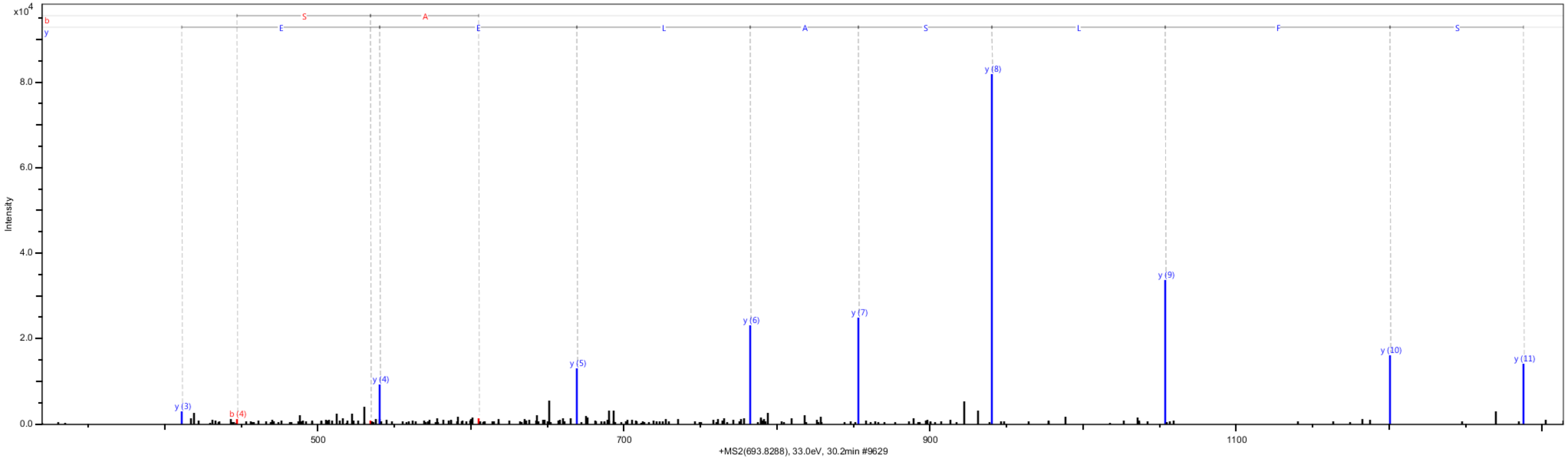

Peptide Match

**Sequence:**	K.VSFLSALEEYTKK.L	**Score:**	59.5
**Modifications:**			

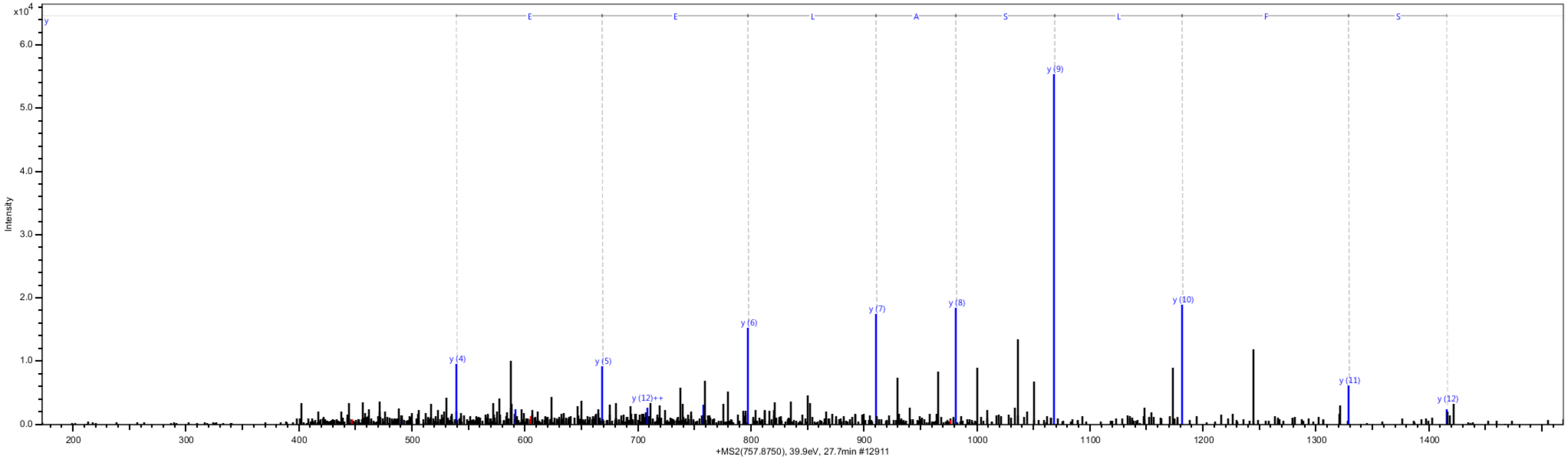

x10^4
Intensity
E E L A S L F S
y (4)
y (5)
y (12)++
y (6)
y (7)
y (8)
y (9)
y (10)
y (11)
y (12)
+MS2(757.8750), 39.9eV, 27.7min #12911

A2MG_HUMAN

**Accession:**	A2MG_HUMAN	**Score:**	679.9
**Database:**	SwissProt	**Seq. Coverage [%]:**	13.6
**MW [kDa] / pI:**	163.2 / 6.0	**No. of Peptides:**	17

Peptide Match

Sequence: K.NEDSLVFVQTDK.S **Score:** 33.5

Modifications:

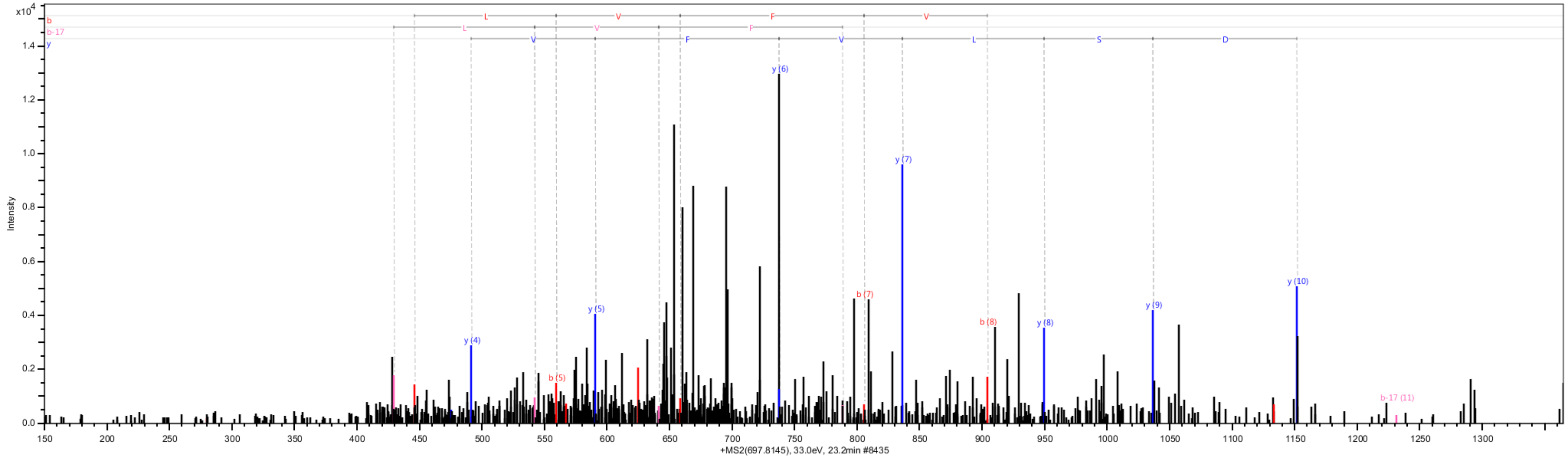

Peptide Match

Sequence: R.IAQWQSFQLEGGLK.Q **Score:** 51

Modifications:

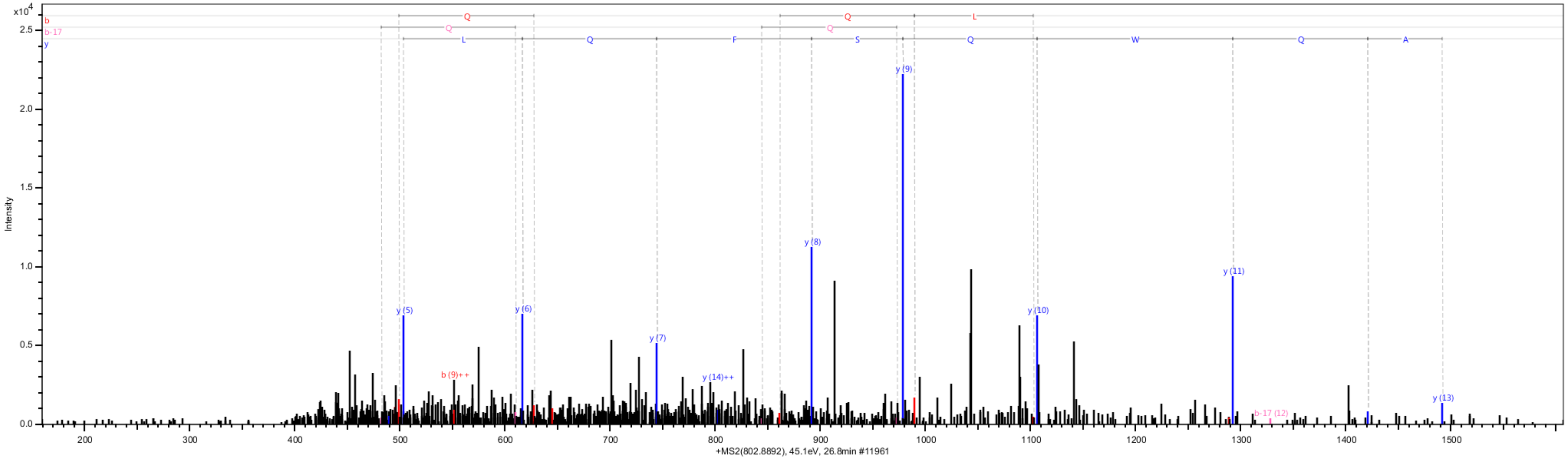

Peptide Match

Sequence: R.TEHPFTVEEFVLPK.F **Score:** 34.8

Modifications:

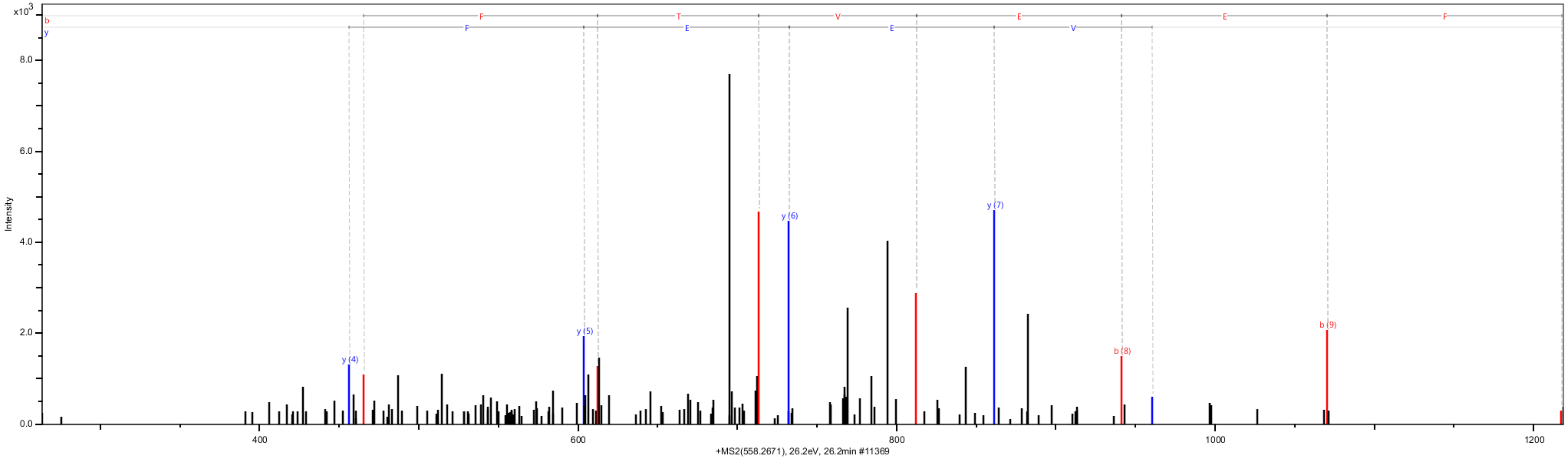

Peptide Match

Sequence: R.QGIPFFGQVR.L **Score:** 52.2

Modifications:

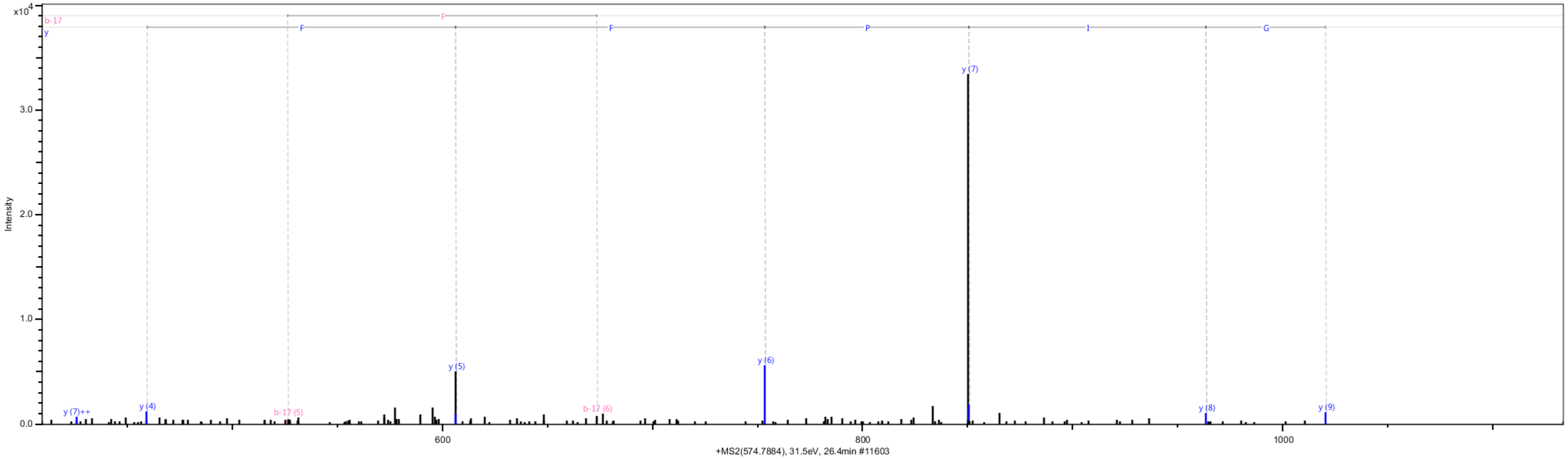

Peptide Match

**Sequence:**	R.VTAAPQSVCALR.A	**Score:**	89
**Modifications:**	Carbamidomethyl: 9		

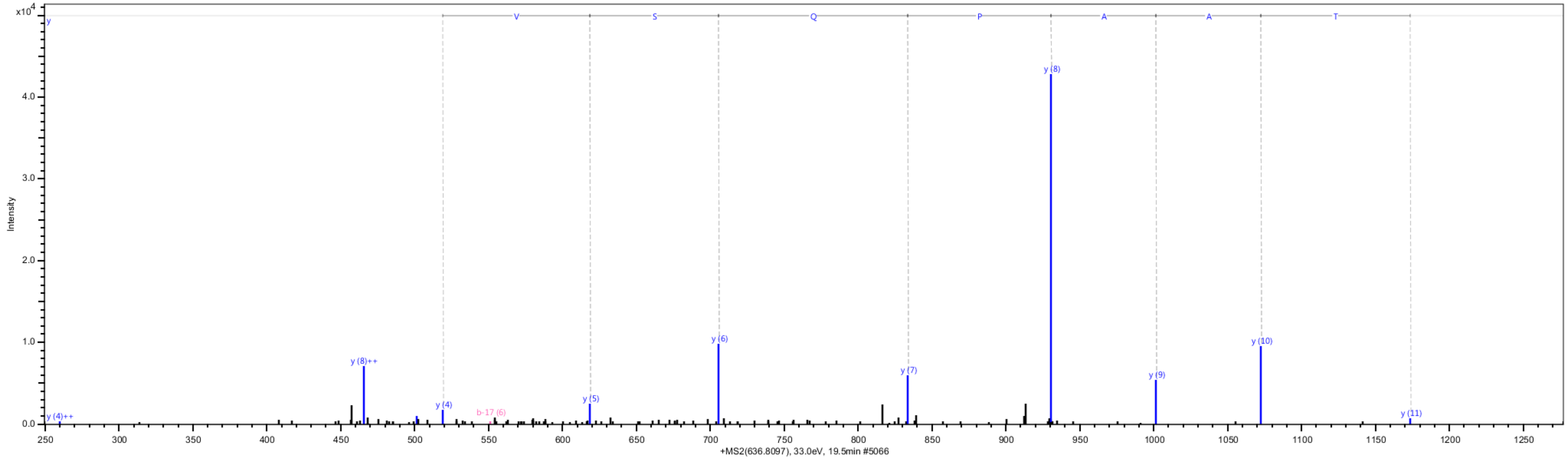

Peptide Match

**Sequence:**	R.VGFYESDVMGR.G	**Score:**	52.3
**Modifications:**			

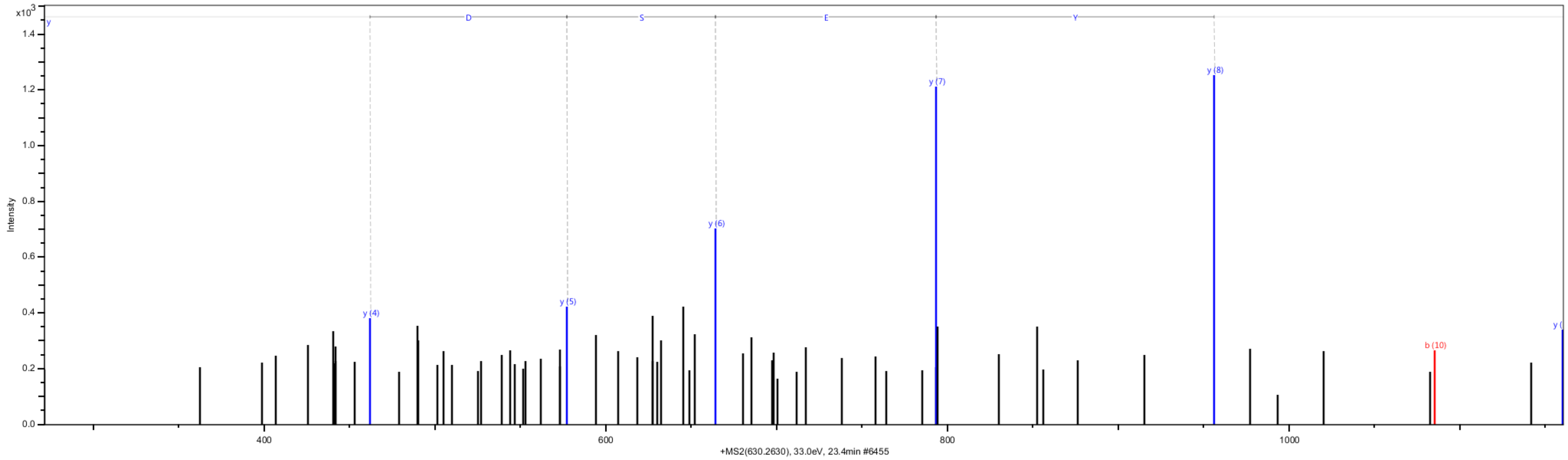

Peptide Match

Sequence: R.LVHVEEPHTETVR.K **Score:** 50.6

Modifications:

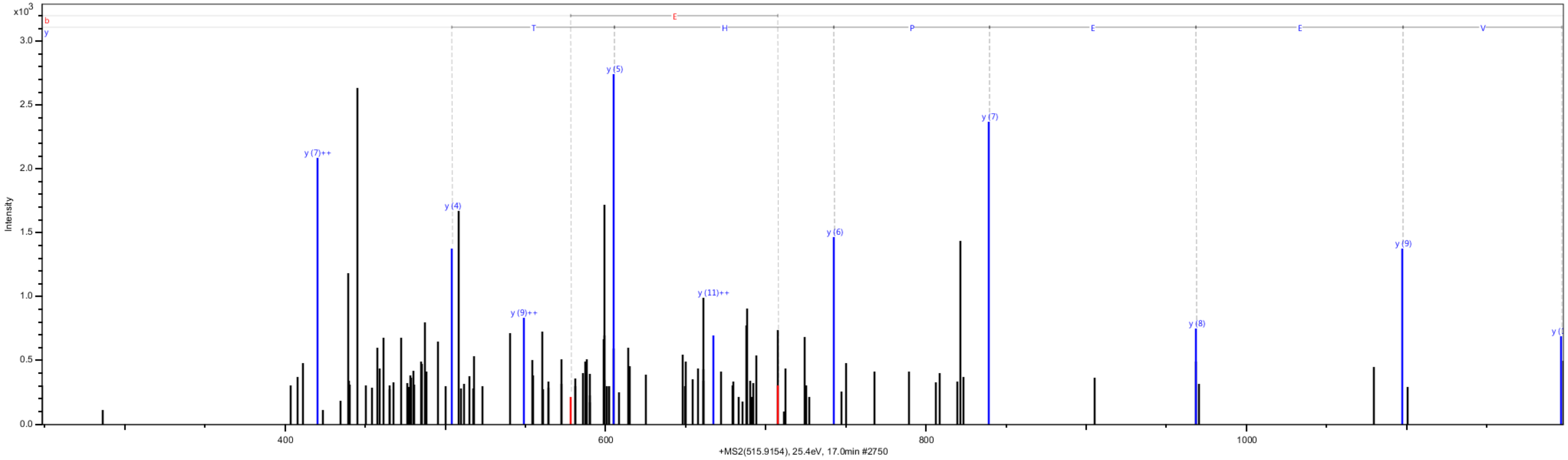

Peptide Match

Sequence: R.QTVSWAVTPK.S **Score:** 36.1

Modifications:

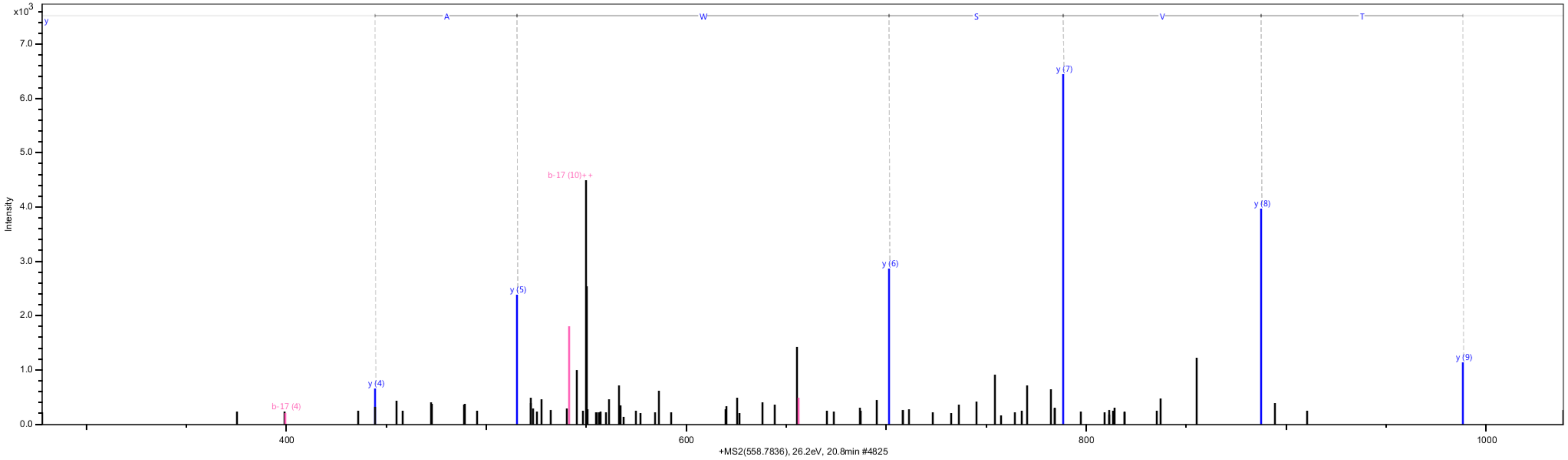

Peptide Match

**Sequence:**	K.DTVIKPLLVEPEGLEK.E	**Score:**	15.3
**Modifications:**			

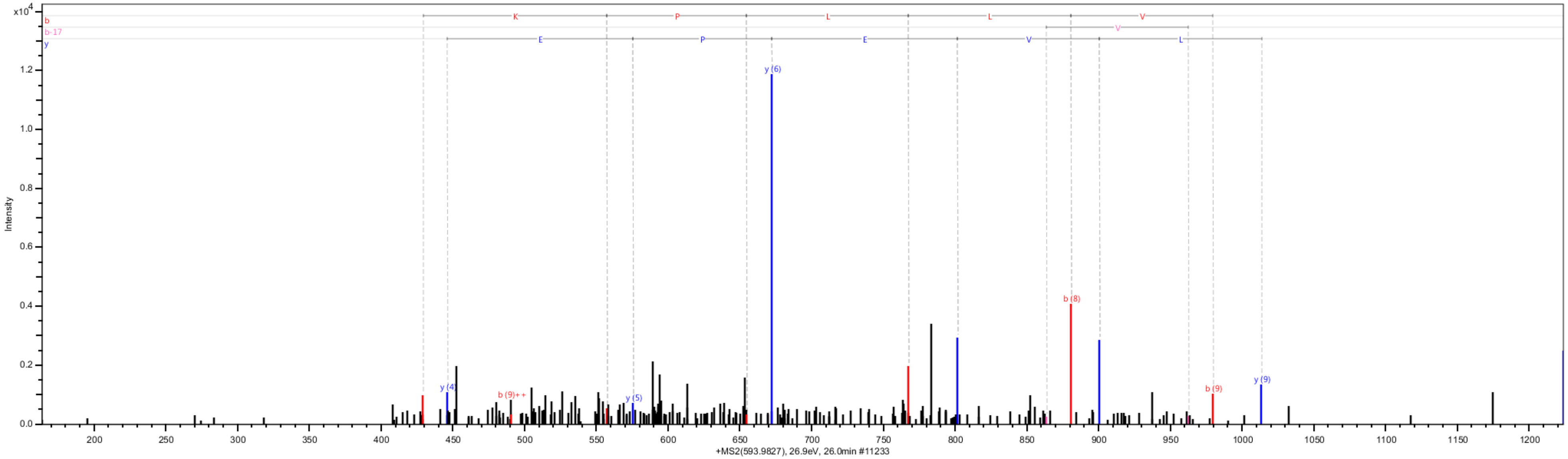

Peptide Match

Sequence: K.LPPNVVEESAR.A **Score:** 42.4

Modifications:

Spectrum Report
Combine Search_2020-10-19 07:53:31

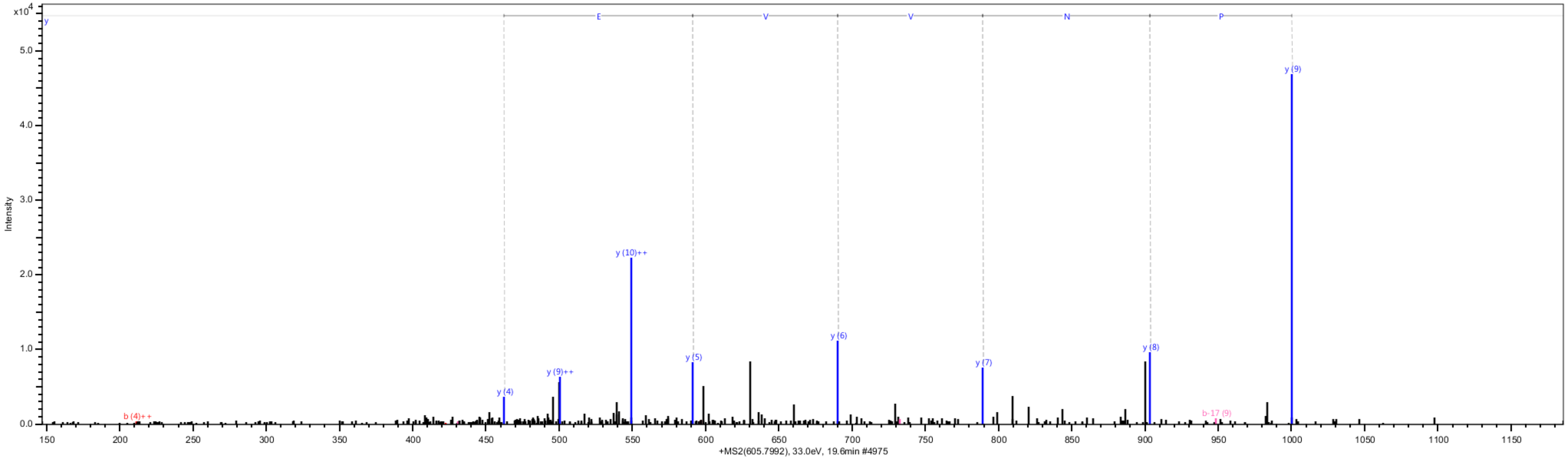

Peptide Match

Sequence: K.AIGYLNTGYQR.Q

Score: 21.9

Modifications:

Spectrum Report
Combine Search_2020-10-19 07:53:31

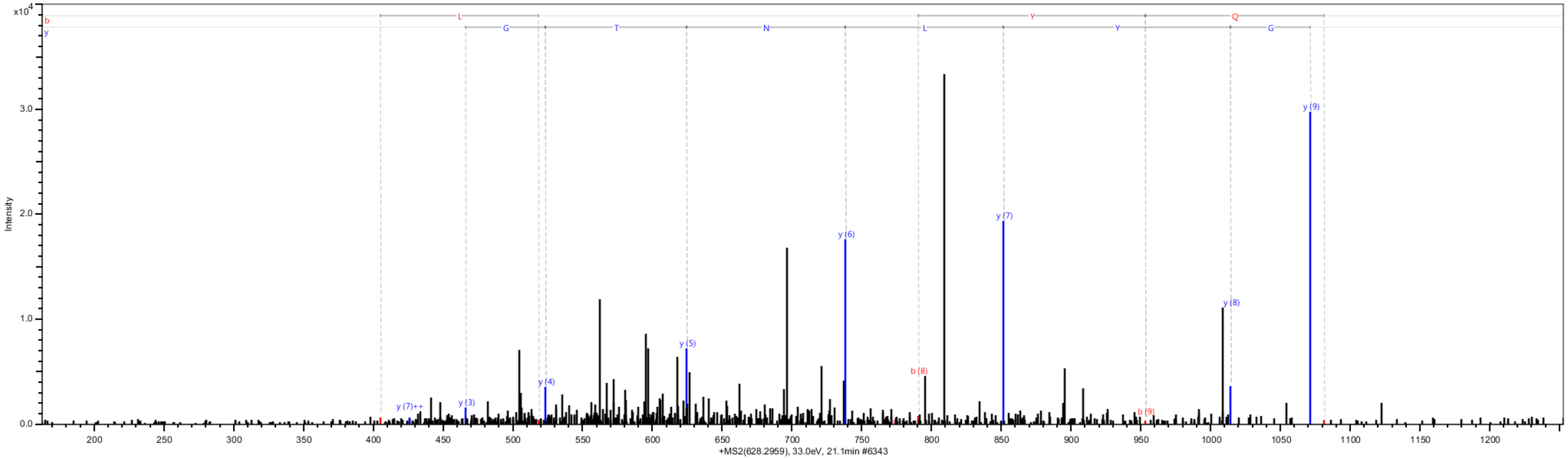

Peptide Match

**Sequence:**	R.NQGNTWLTAFVLK.T	**Score:**	28.4
**Modifications:**			

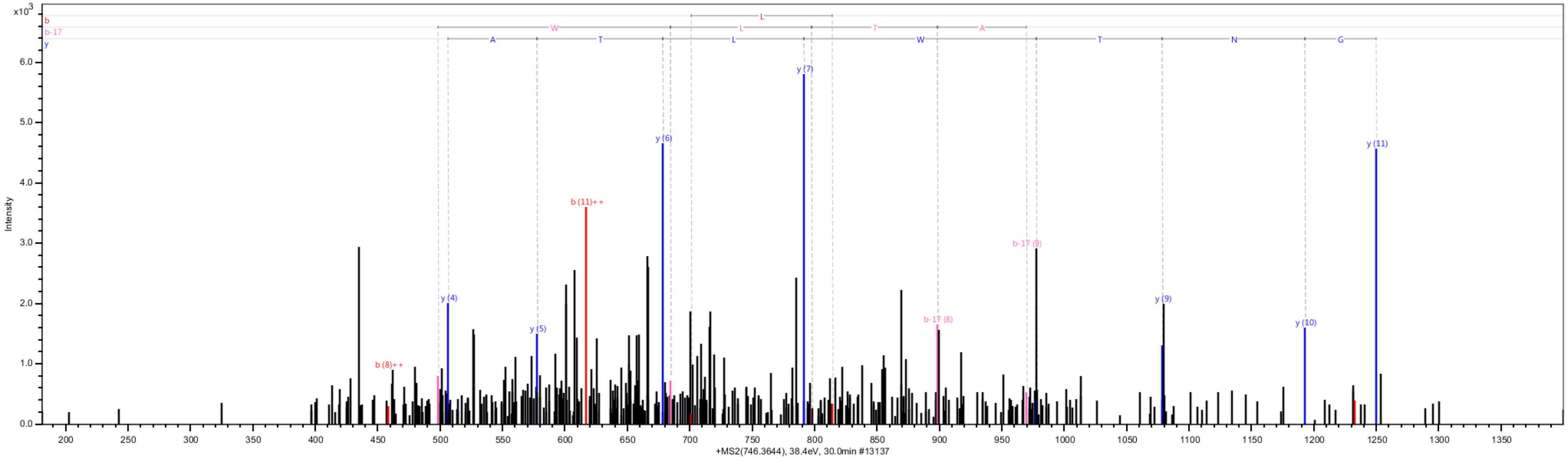

Peptide Match

Sequence: R.SSGSLLNNAIK.G **Score:** 35.8

Modifications:

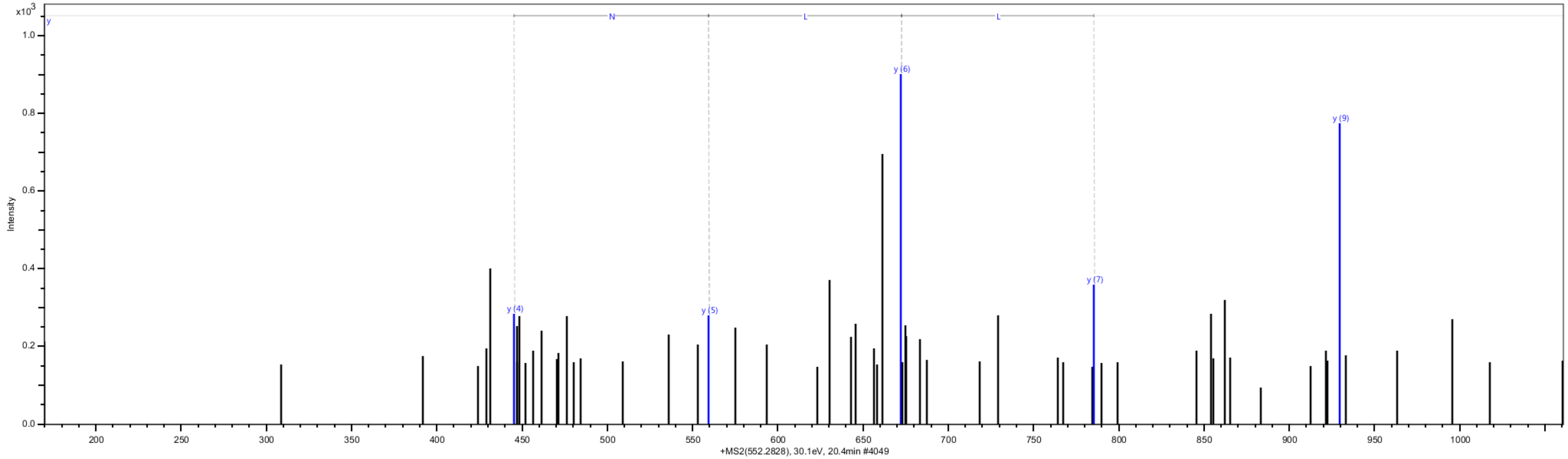

Peptide Match

Sequence: R.NALFCLESAWK.T **Score:** 45.5

Modifications: Carbamidomethyl: 5

Spectrum Report
Combine Search_2020-10-19 07:53:31

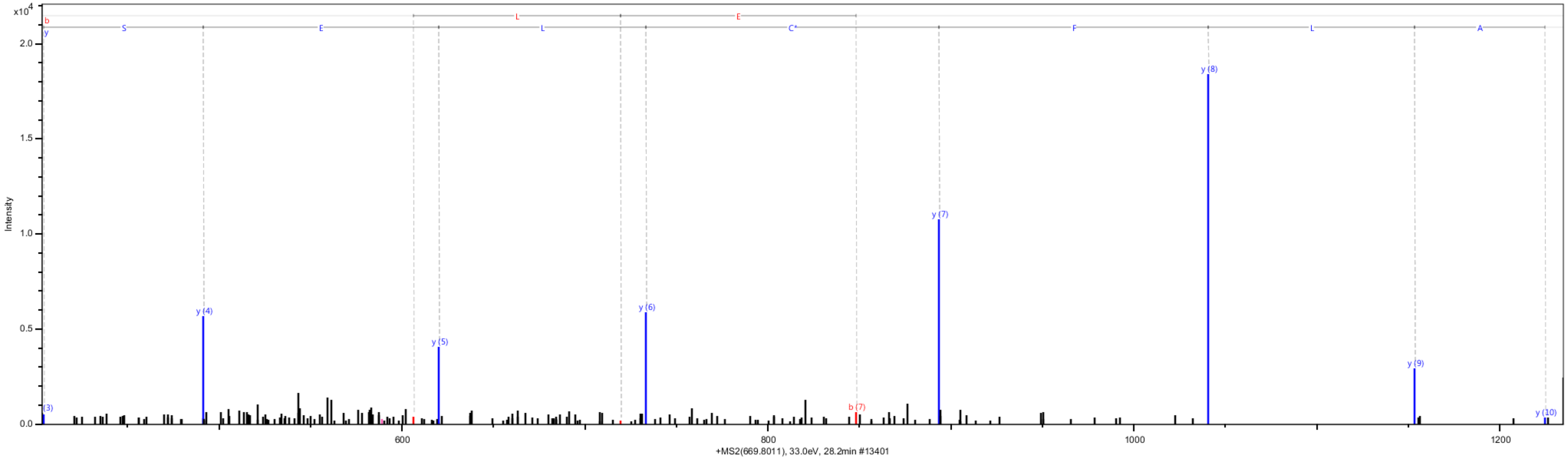

Peptide Match

**Sequence:**	K.ALLAYAFALAGNQDK.R	**Score:**	43.7
**Modifications:**			

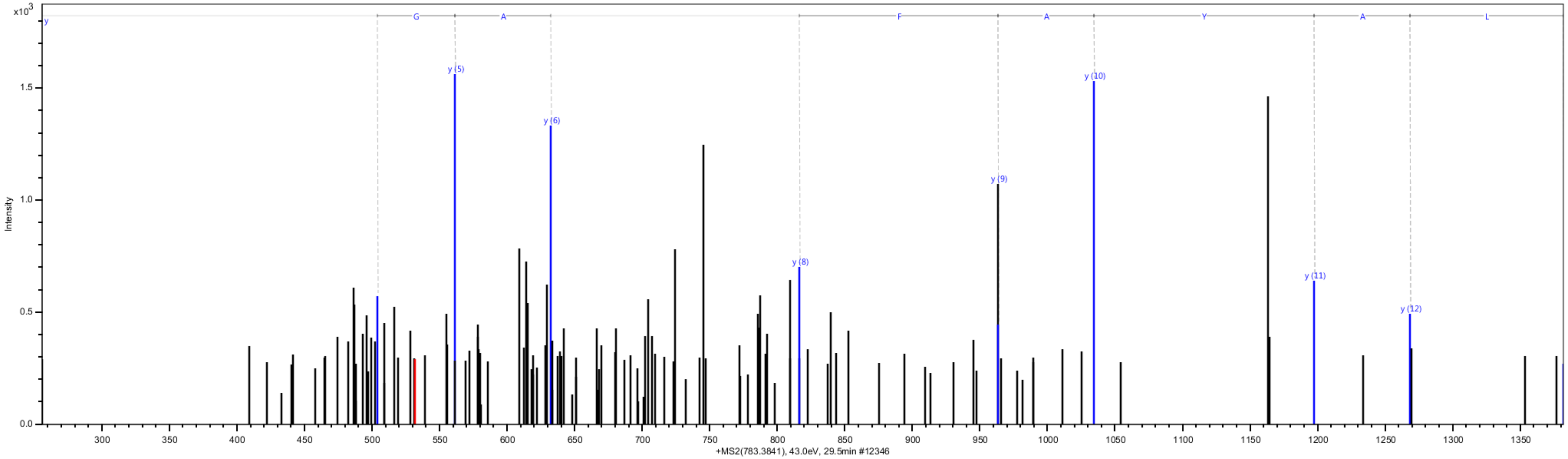

Peptide Match

**Sequence:**	K.ALLAYAFALAGNQDKR.K	**Score:**	16.5
**Modifications:**			

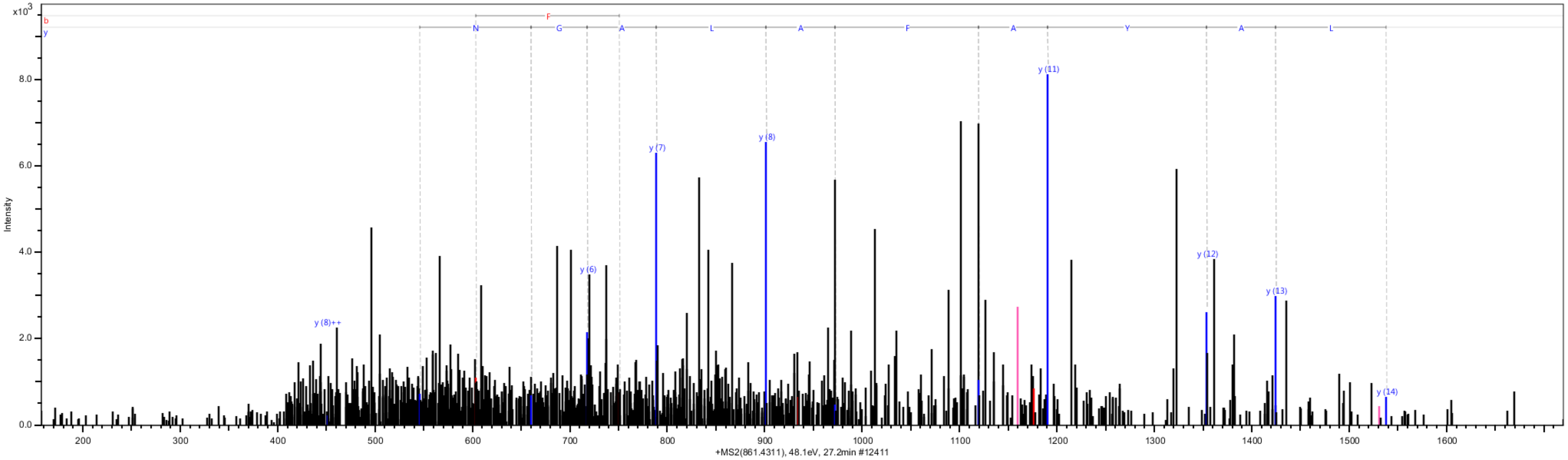

Peptide Match

Sequence: K.AAQVTIQSSGTFSSK.F **Score:** 30.8

Modifications:

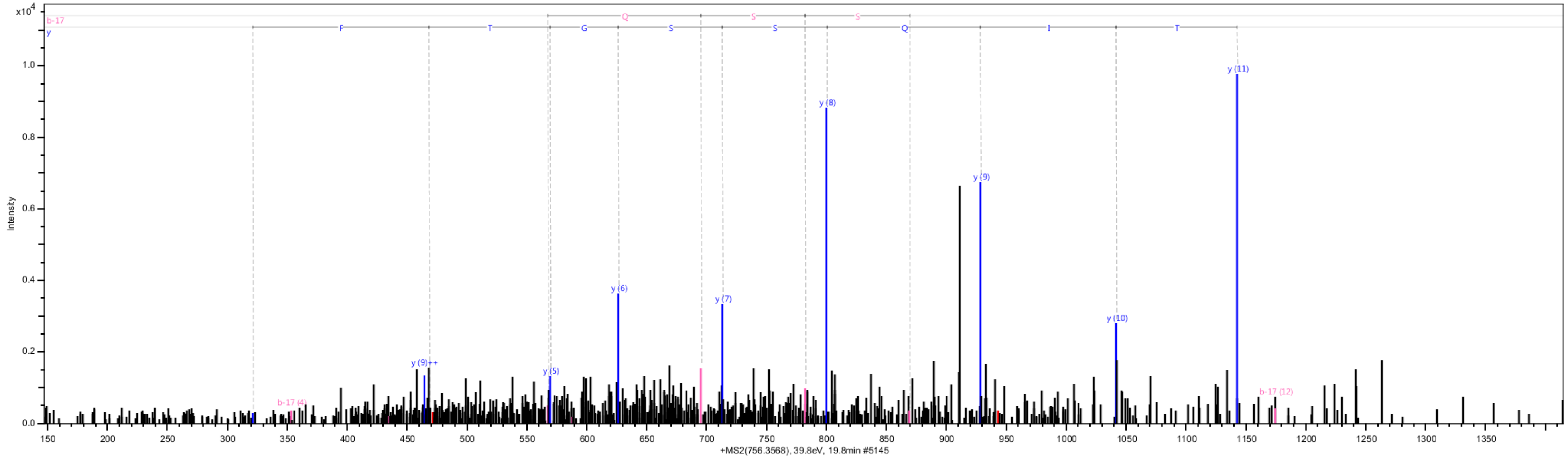
x10^4
b-17
y
F
T
G
S
S
Q
I
T
Q
S
S
Intensity
1.0
0.8
0.6
0.4
0.2
0.0
y (11)
y (8)
y (9)
y (6)
y (7)
y (10)
y (9)++
y (5)
b-17 (4)
b-17 (12)
150
200
250
300
350
400
450
500
550
600
650
700
750
800
850
900
950
1000
1050
1100
1150
1200
1250
1300
1350
+MS2(756.3568), 39.8eV, 19.8min #5145

IGHG2_HUMAN

Accession: IGHG2_HUMAN | **Score:** 515.0
Database: SwissProt | **Seq. Coverage [%]:** 13.8
MW [kDa] / pI: 35.9 / 7.7 | **No. of Peptides:** 5

Peptide Match

Sequence: R.STSESTAALGCLVK.D | **Score:** 78.9
Modifications: Carbamidomethyl: 11

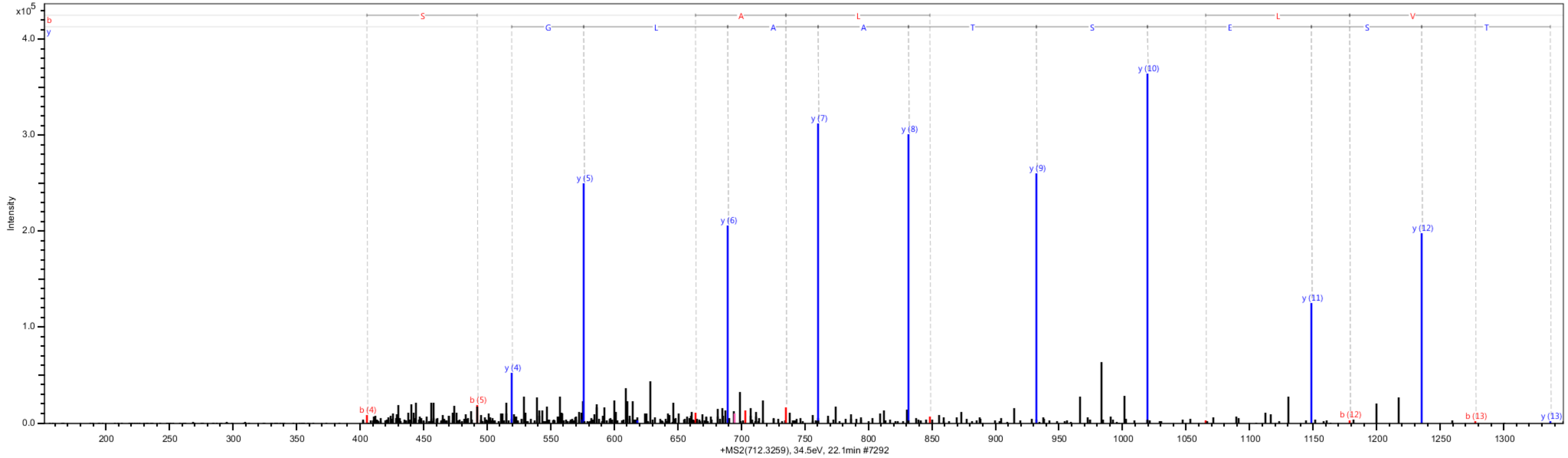

Peptide Match

Sequence: R.VVSVLTVVHQDWLNGK.E | **Score:** 101.9
Modifications:

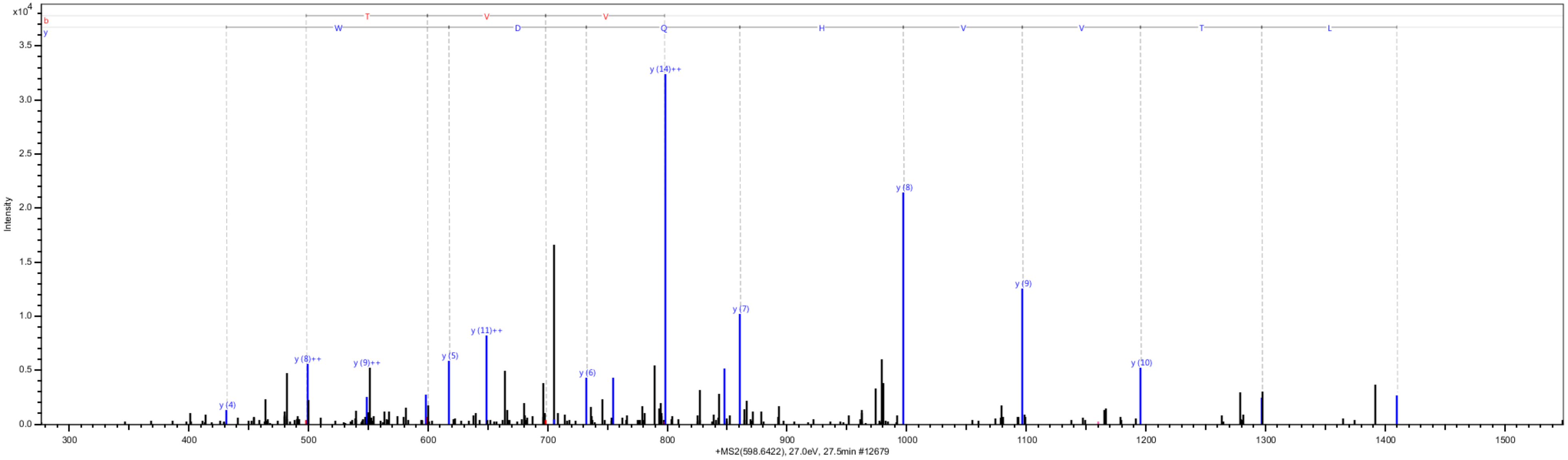

Peptide Match

Sequence: R.VVSVLTVVHQDWLNGKEYK.C **Score:** 93.7

Modifications:

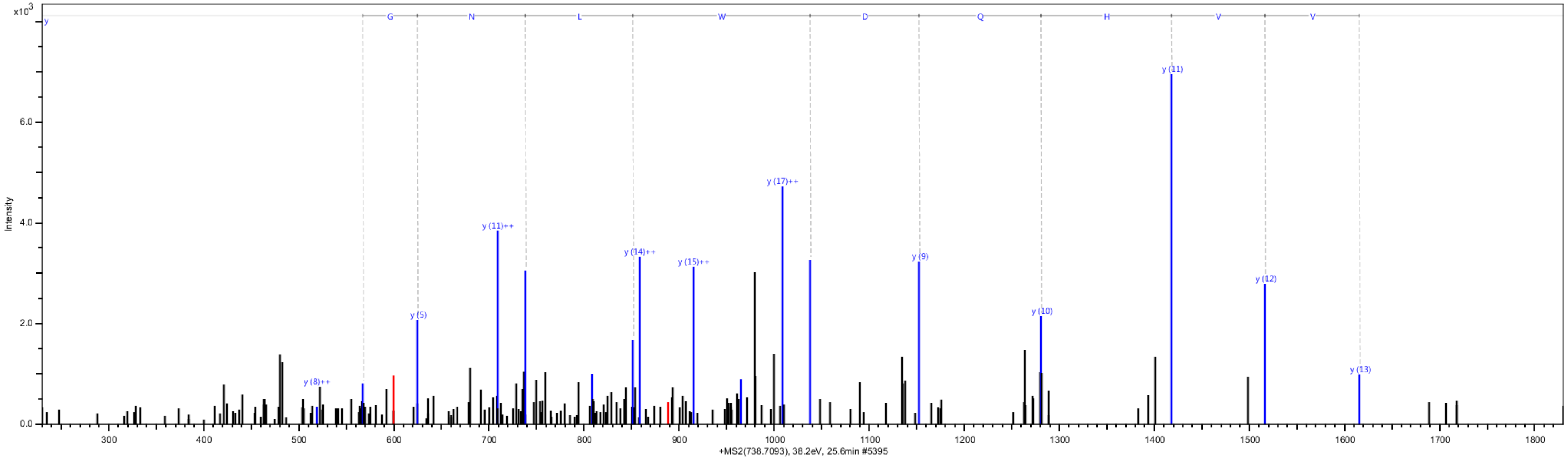

Peptide Match

Sequence: K.VSNKGLPAPIEK.T **Score:** 33.6

Modifications:

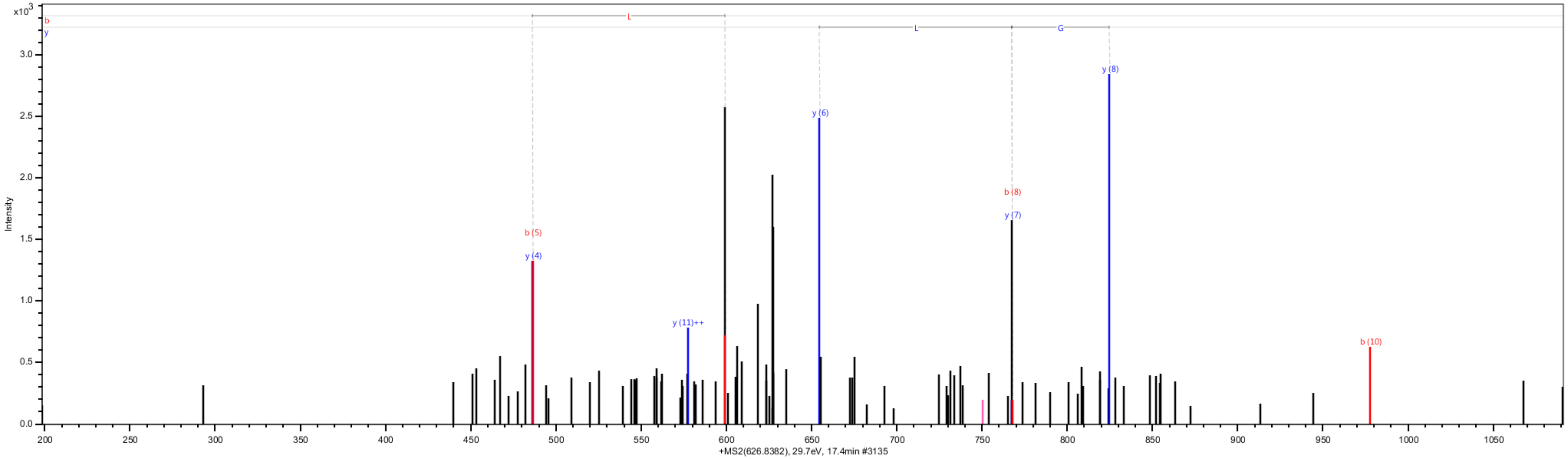

Peptide Match

Sequence: K.GLPAPIEK.T **Score:** 25

Modifications:

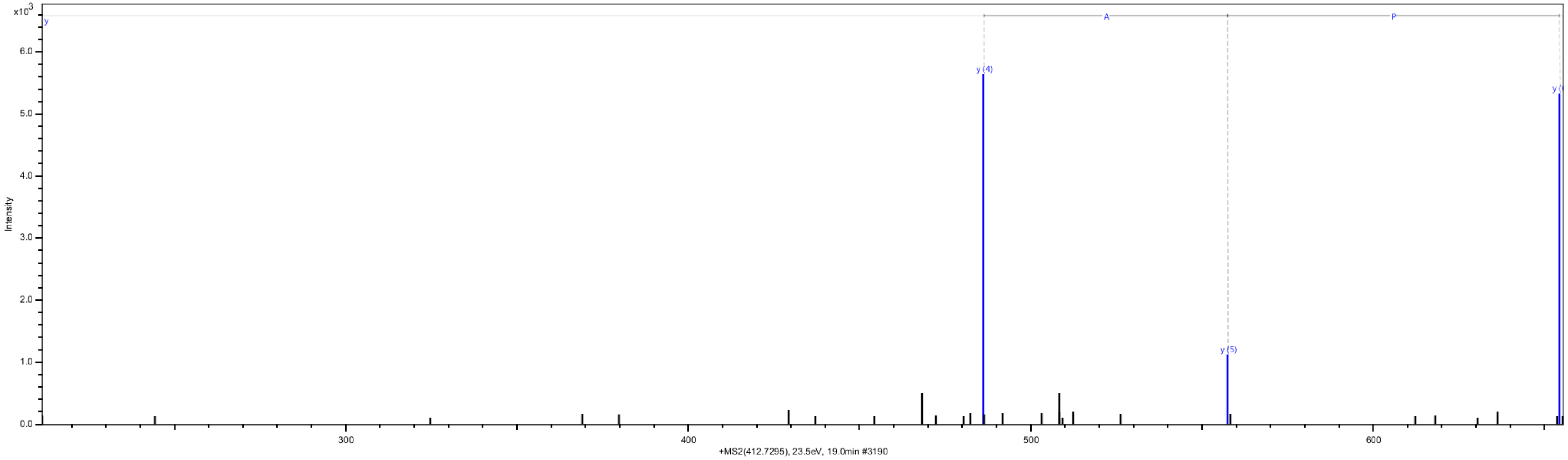
x10^3
y
A
P
y (4)
y (5)
6.0
5.0
4.0
3.0
2.0
1.0
0.0
Intensity
300
400
500
600
+MS2(412.7295), 23.5eV, 19.0min #3190

IGHG3_HUMAN

Accession: IGHG3_HUMAN
Database: SwissProt
MW [kDa] / pI: 41.3 / 8.2
Score: 561.9
Seq. Coverage [%]: 17.5
No. of Peptides: 5

Peptide Match

Sequence: K.GPSVFPLAPCSR.S
Score: 69.2
Modifications: Carbamidomethyl: 10

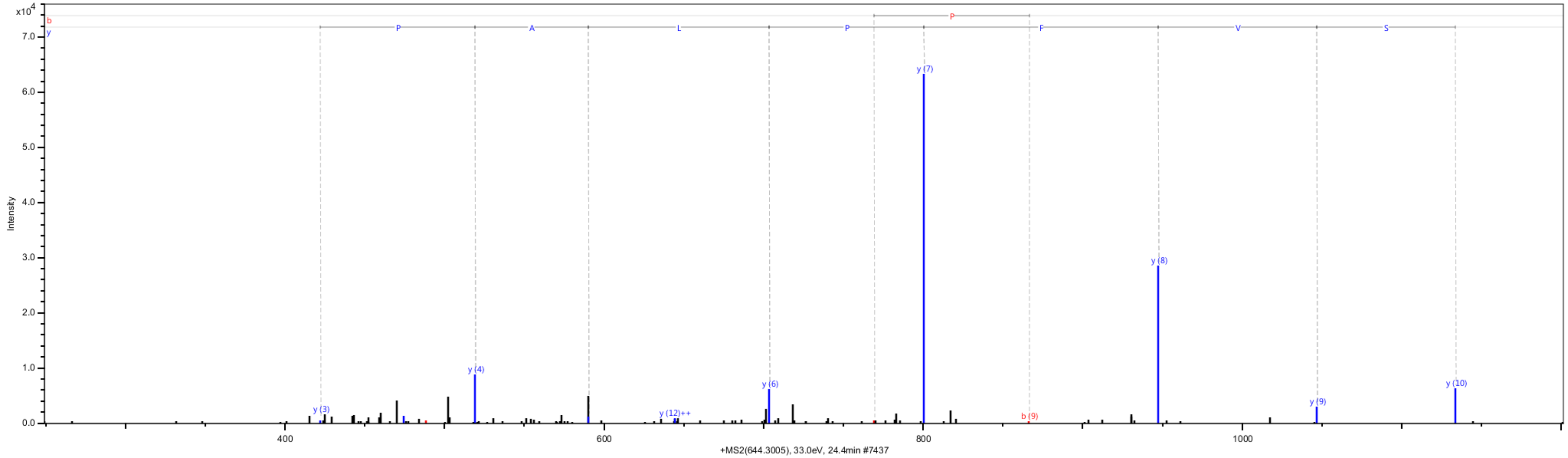

Peptide Match

Sequence: R.VELKTPLGDTTHTCPR.C
Score: 29.4
Modifications: Carbamidomethyl: 14

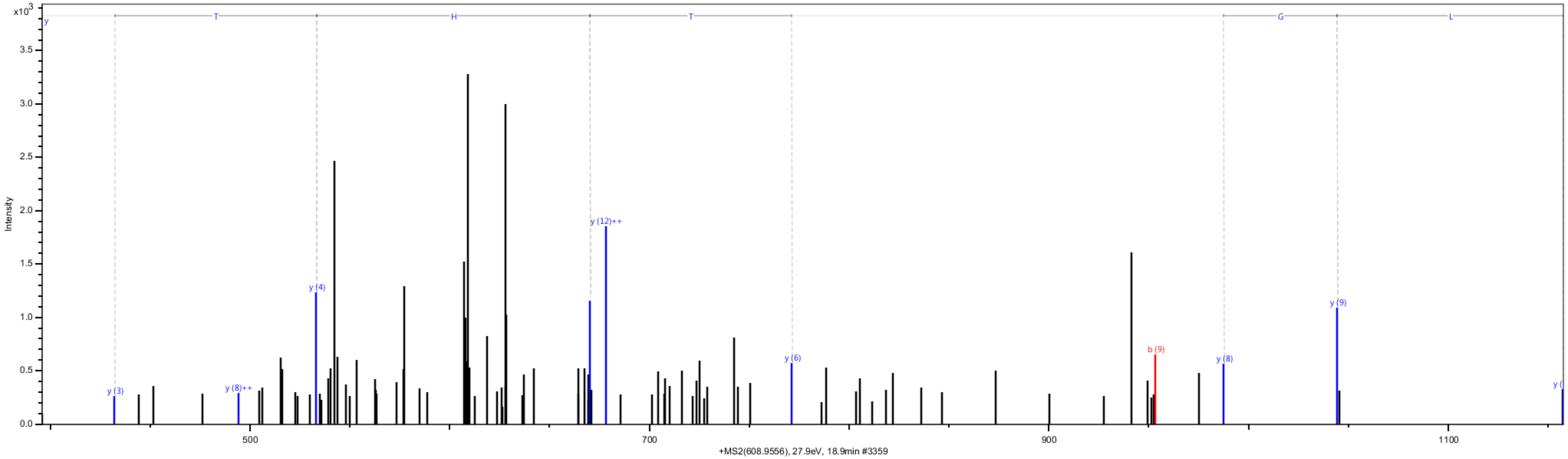

Peptide Match

**Sequence:**	K.SCDTPPPCPR.C	**Score:**	40.2
**Modifications:**	Carbamidomethyl: 2, 8		

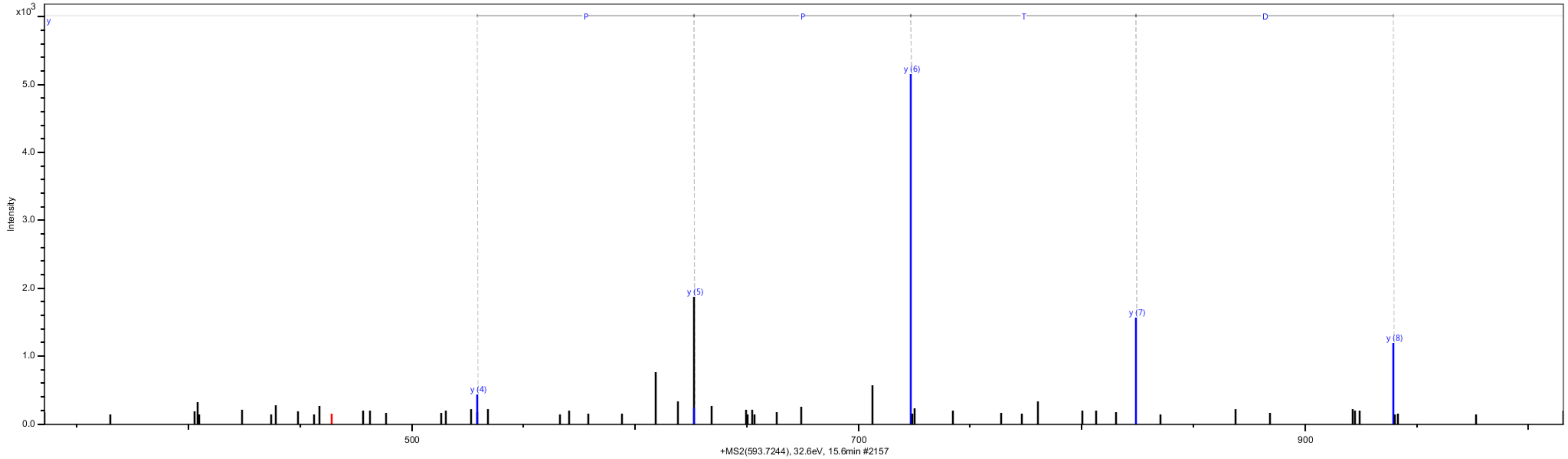

Peptide Match

Sequence: K.WYVDGVEVHNAK.T **Score:** 65.2

Modifications:

Spectrum Report
Combine Search_2020-10-19 07:53:31

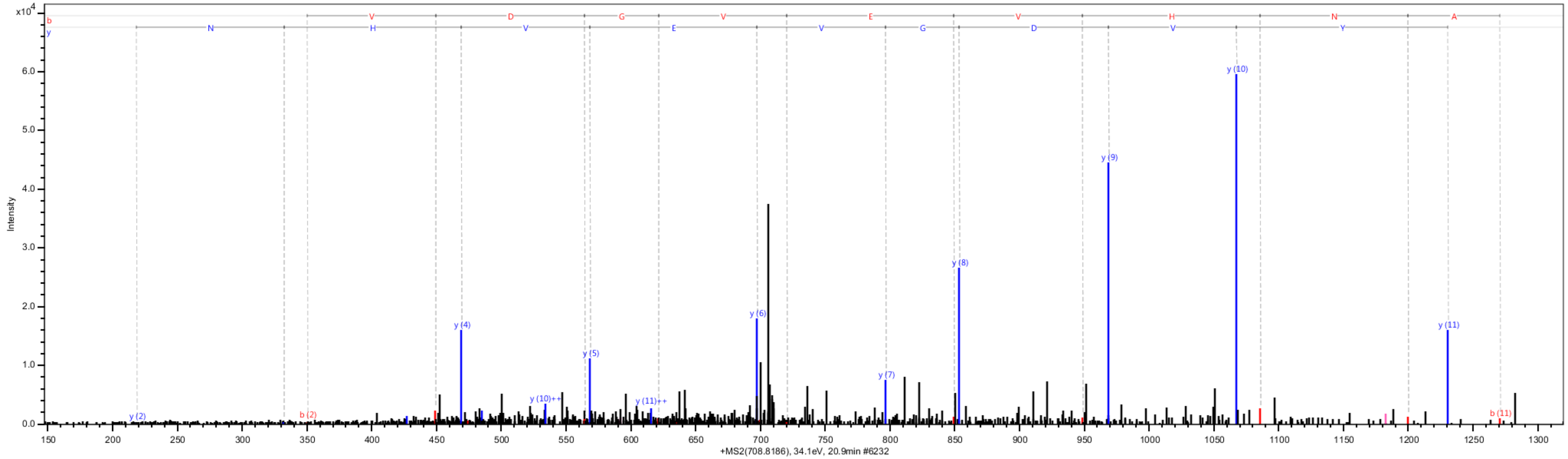

Peptide Match

Sequence: R.EPQVYTLPPSREEMTK.N **Score:** 16.3

Modifications:

Spectrum Report
Combine Search_2020-10-19 07:53:31

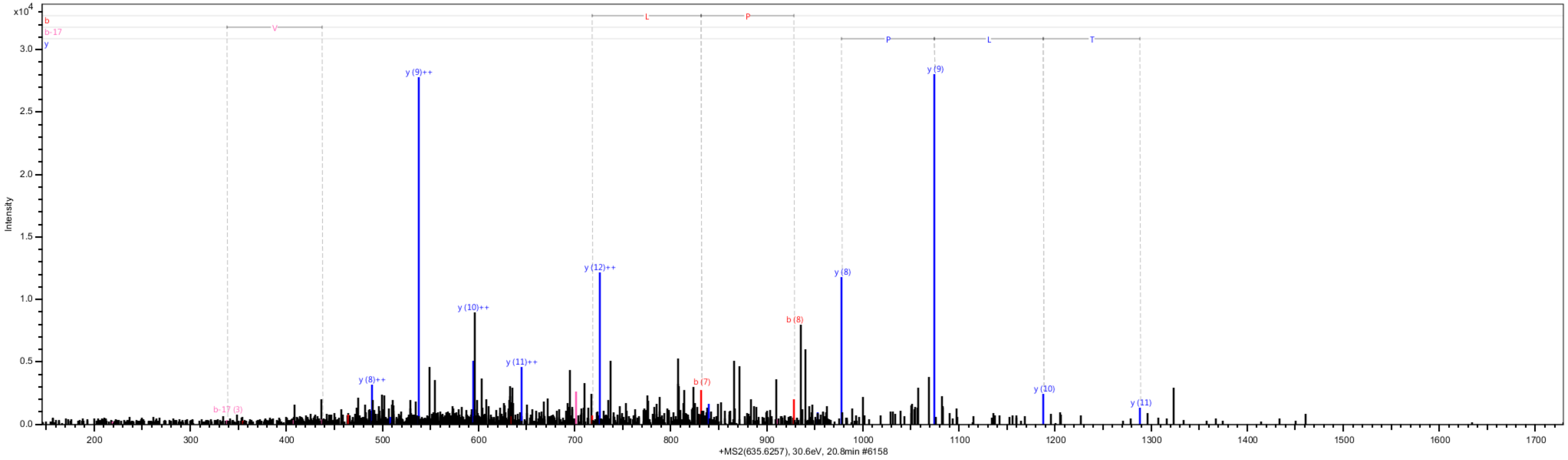

Supplement: RA-011-D0RA08389E-s001 [file RA-011-D0RA08389E-s001.pdf]
